# Zika virus targets the human thymic epithelium

**DOI:** 10.1038/s41598-020-58135-y

**Published:** 2020-01-28

**Authors:** Carolina V. Messias, Guilherme Loss-Morais, Joseane Biso de Carvalho, Mariela N. González, Daniela P. Cunha, Zilton Vasconcelos, Luis W. P. Arge, Désio A. Farias-de-Oliveira, Alexandra L. Gerber, Elyzabeth A. Portari, Nilma Ferreira, Lidiane M. S. Raphael, Myrna C. Bonaldo, Ingo Riederer, Maria E. Lopes Moreira, Vinicius Cotta-de-Almeida, Ana T. R. Vasconcelos, Daniella A. Mendes-da-Cruz, Wilson Savino

**Affiliations:** 1 Laboratory on Thymus Research, Oswaldo Cruz Institute, Oswaldo Cruz Foundation, Rio de Janeiro, Brazil; 2 National Institute of Science and Technology on Neuroimmunomodulation (INCT-NIM); Oswaldo Cruz Institute, Oswaldo Cruz Foundation, Rio de Janeiro, Brazil; 3 Bioinformatics Laboratory, National Laboratory for Scientific Computing, Petrópolis, Rio de Janeiro, Brazil; 4 Laboratory of Molecular Biology of Flavivirus, Oswaldo Cruz Institute, Oswaldo Cruz Foundation, Rio de Janeiro, Brazil; 5 Laboratory of High Complexity, Fernandes Figueira National Institute for the Health of Mother, Child, and Adolescent, Oswaldo Cruz Foundation, Rio de Janeiro, Brazil

**Keywords:** Viral infection, Lymphoid tissues

## Abstract

Previous work showed that the thymus can be infected by RNA viruses as HIV and HTLV-1. We thus hypothesized that the thymus might also be infected by the Zika virus (ZIKV). Herein we provide compelling evidence that ZIKV targets human thymic epithelial cells (TEC) *in vivo* and *in vitro*. ZIKV-infection enhances keratinization of TEC, with a decrease in proliferation and increase in cell death. Moreover, ZIKV modulates a high amount of coding RNAs with upregulation of genes related to cell adhesion and migration, as well as non-coding genes including miRNAs, circRNAs and lncRNAs. Moreover, we observed enhanced attachment of lymphoblastic T-cells to infected TEC, as well as virus transfer to those cells. Lastly, alterations in thymuses from babies congenitally infected were seen, with the presence of viral envelope protein in TEC. Taken together, our data reveals that the thymus, particularly the thymic epithelium, is a target for the ZIKV with changes in the expression of molecules that are relevant for interactions with developing thymocytes.

## Introduction

Zika virus (ZIKV) epidemics in 2015-2016 resulted in devastating effects, causing microcephaly, other related congenital defects at birth and neurodevelopmental delay after two years in children born from mothers infected by the virus during pregnancy^1–4^. Additionally, ZIKV infection in adults correlated with a rise in the frequency of cases of Guillain-Barré syndrome^5,6^.

Although the knowledge on the cellular and molecular alterations caused by the ZIKV in the nervous tissue largely increased in the last few years^2,7,8^, the effects of this virus upon hematopoietic tissues are much less defined.

In terms of secondary lymphoid organs, ZIKV RNA and protein have been described in lymph nodes of Rhesus monkeys in both paracortex and germinal centers^9,10^. The virus was also found in macrophages, dendritic cells, and B-cells, in both spleen and axillary lymph nodes of this non-human primate. However, in the same study, the presence of ZIKV RNA in T-cells was observed only in axillary lymph nodes from one animal^10^.

Much less is known on the putative infection of primary lymphoid organs, and more particularly in the thymus. This central lymphoid organ is responsible for the generation of T lymphocytes under the control of the thymic microenvironment, a three-dimensional cellular network mainly composed by thymic epithelial cells – TEC^11^. In this respect, it is interesting to note the thymus as a target organ for other RNA viruses, such as HIV^12^ and HTLV-1^13,14^. In fact, we showed that cultured human TEC can be infected by HTLV-1 and convey virus particles to lymphoblastic T cells^15,16^. We thus hypothesized that the thymic epithelium might also be infected by the Zika virus. Herein we provide compelling evidence that ZIKV targets human TEC both *in vivo* and *in vitro*. Data are provided showing that ZIKV-infection enhances keratinization of TEC, with a decrease in proliferation and increase in cell death. Moreover, *in vitro* data revealed that the virus could modulate a high amount of coding and non-coding genes (including miRNAs, circRNAs, and lncRNAs), with upregulation of genes encoding various cell adhesion and cell migration-related proteins. Accordingly, infected TEC enhanced adhesion of lymphoblastic lymphoma-derived T cells and conveyed the virus to this same cell type. Lastly, alterations in the thymic microenvironment from babies prenatally infected by ZIKV were observed, as well as the presence of the viral envelope protein in the thymus. Taken together, our data reveal that the thymus is a target for the ZIKV and may function as a reservoir of the virus during congenital infection.

## Results

### Cultured human TEC can be infected by the Zika virus

**Figure 1:**
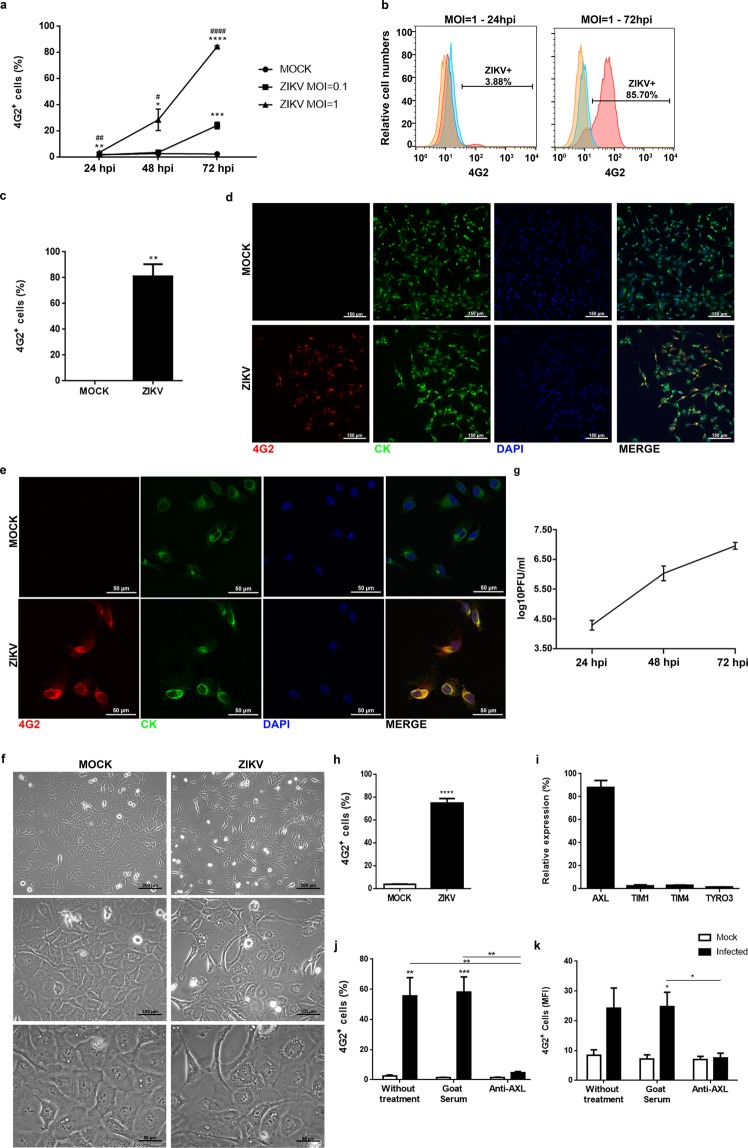
Human TEC can be infected *in vitro* by the Zika virus. (**a**) TEC were infected with ZIKV (MOI = 0.1 or 1) and the relative expression of 4G2^+^ cells was detected 24, 28 and 72 hours post-infection (hpi) by flow cytometry. Each time point represents the mean ± standard error. Asterisks represent statistical significance between MOCK and MOI = 1 (24, 48 and 72 h) and between MOCK and MOI = 0.1 (72 h). Hash marks represent statistical significance between MOI = 0.1 and MOI = 1. (**b**) Representative histograms of the relative expression of 4G2^+^ cells 24 and 72 hpi with MOI = 1. Orange curves represent isotype controls, blue curves represent MOCK and red curves represent ZIKV (n = 3). TEC were infected with ZIKV (MOI = 1) and (**c**) the percentage of 4G2^+^ cells were detected 72 hpi by immunofluorescence. Results are represented by the mean percentage of infection of the three replicates of each independent experiment. Representative images of immunofluorescence for 4G2 (viral protein, in red), cytokeratin (CK, in green) and DAPI (nuclei, in blue) (**d**) in lower and (**e**) higher magnifications (n = 3, in triplicates). (**f**) Representative images of TEC culture 72 hpi (n = 3). (**g**) ZIKV growth curve in TEC culture. Supernatant of ZIKV-infected TEC culture was harvested, and the presence of infective viral particles was verified in Vero cells. Results are shown as plaque-forming units and each time point represents the mean ± SD, of at least 5 wells (n ≥ 5). (**h**) Relative expression of 4G2 ^+^ cells detected by flow cytometry in TEC culture 72 hpi with 1 mL of supernatant from MOCK or ZIKV-infected TEC cultures (n = 3). (**i**) Relative expression of AXL, TIM1, TIM4 e TYRO3 receptors detected by flow cytometry in TEC (n = 3). (**j**) Relative expression and (**k**) Mean Fluorescence Intensity (MFI) of 4G2^+^ cells detected by flow cytometry 72 hpi (MOI = 1) after treatment with anti-AXL polyclonal antibody (10 µg/mL) (n = 3). *or p < 0.05, **or p < 0.01, ***p < 0.001, ****or p < 0.0001, (unpaired *t*-test or one-way ANOVA, followed by Tukey multiple comparison test).

We first investigated the infectivity and growth capacity in ZIKV-infected human TEC, evaluating the virus yield in cell monolayers. The human postnatal TEC line used was obtained by explant technique and limiting dilution cloning, being derived from fragments of a postnatal thymus from a child undergoing cardiac surgery^17^. Functionally these cells are able to produce cytokines, chemokines, and extracellular matrix proteins, and can adhere to freshly-isolated thymocytes, as well as acute lymphoblastic leukemia derived T cells^16,18^. In the first group of experiments, we applied infection doses of 0.1 and 1.0 MOI, with the cells being then harvested 24, 48 and 72 hours post-infection, and subjected to cytofluorometry or immunofluorescence with the 4G2 antibody for intracellular detection of the viral envelope protein. The mouse monoclonal 4G2 antibody recognizes an epitope on the envelope protein conserved in the flavivirus family, including Dengue virus, West Nile virus, Japanese Encephalitis virus and Zika virus^19^. As shown in Fig. 1a,b, we found a progressive increase in the relative numbers of infected cells, as ascertained by the two applied viral doses. Yet, the use of 1.0 MOI was much more efficient in promoting infection, so that around 90% of the cells were 4G2-positive after 72 hours. Accordingly, all further experiments were done using this infection dose at 72 hours post-infection and MOCK cells were used as control, as the 4G2 antibody present a slight background in MOCK cells when compared to the isotype control. The same percentage of infection was detected by immunofluorescence on adhered cells (Fig. 1c,d), and the presence of the ZIKV envelope protein could be detected in the cytoplasm - especially around the nucleus (Fig. 1e).

ZIKV infection induced cytopathic effects in TEC with the presence of higher amounts of floating cells in the culture and vacuole-like structures around the nucleus (Fig. 1f).

We also showed that infection of growing TEC resulted in the generation and release of viral infectious particles in the culture supernatants. Following 24, 48 and 72 hours post-infection, the obtained viral titers from TEC cultures were 4.29 ± 0.16, 6.03 ± 0.25, and 6.96 ± 0.11 log10 PFU/mL, as ascertained in Vero cell cultures (Fig. 1g). Furthermore, infected TEC-derived supernatants were able to convey ZIKV to uninfected TEC cultures (Fig. 1h).

Since human TEC could be infected by ZIKV, we searched for the membrane expression of well-known ZIKV receptors, namely TIM1, TIM4, TYRO3, and AXL. Actually, cultured human TEC constitutively expresses AXL, but not TIM1, TIM4, TYRO3 receptors (Fig. 1i). Blocking the AXL receptor with the corresponding neutralizing antibody completely prevented TEC infection by ZIKV (Fig. 1j,k), showing that ZIKV uses the AXL receptor to invade human TEC.

### Zika virus infection disturbs cell growth in cultured human TEC

**Figure 2:**
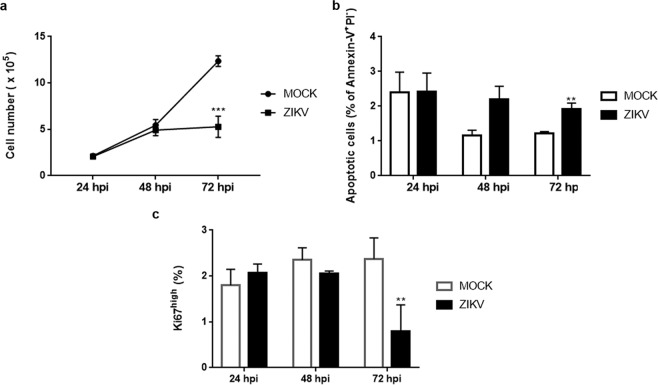
Zika virus infection down modulates the growth of cultured human thymic epithelial cells. TECs were infected with ZIKV (MOI = 1) and the (**a**) numbers of cells in culture were detected 24, 48 and 72 hours post-infection (hpi). Each time point represents the mean ± SEM (n = 3). (**b**) Percentage of apoptotic cells (annexin-V^+^PI^−^) detected by flow cytometry 24, 48 and 72 hpi. Each time point represents the mean ± SEM (n = 3) (**c**) Percentage of Ki67 high^+^ cells detected by flow cytometry 24, 48 and 72 hpi. Values represent the mean ± SEM (n = 3). **p < 0.01, ***p < 0.001 (unpaired *t*-test).

Considering that human TEC were sensitive to ZIKV infection, we wondered whether such an infection could also modulate the growth of these cells *in vitro*. The TEC counting in infected versus uninfected cultures showed that ZIKV infection induced a progressive reduction in the numbers of growing TEC (Fig. 2a). Experiments aiming at defining whether such a diminution was due to a decrease in cell proliferation or increase in cell death, revealed a diminution in the proportion of dividing cells (defined by the high expression of the Ki67 marker) and an increase in the relative numbers of apoptotic (but not necrotic) cells (Fig. 2b,c), as ascertained by PI and annexin-V double labeling. Differences in the proportion of apoptotic and dividing cells begin to appear 48 hours post-infection, although significant differences are only detected 72 hours post-infection. Of note, in addition to the small, but significant increase in the relative numbers of apoptotic cells, we observed increased amounts of cellular debris in ZIKV-infected TEC cultures when compared to controls (data not shown). It is interesting to note that such modulation of cell growth was not seen when we infected the same human TEC line by HTLV-1 neither in cell proliferation nor cell death^16^.

### Zika infection induces major changes in human TEC gene expression profile

The transcriptome analysis of ZIKV-infected TEC revealed 2,934 differentially expressed genes (DEGs) with p-adjusted < 0.01 and Log_2_ Fold Change > |1|, distributed in protein-coding genes, long non-coding RNAs, circRNAs and other RNAs classes (Table 1, Supplemental Table 1).

**Table 1:** Summary of the differentially Expressed Genes and miRNAs identified by RNAseq and small RNAseq in ZIKV-infected TEC.

Molecule	Expressed Genes	Upregulated*	Downregulated*	Total*
Protein Coding	15935	1471	106	**1577**
Long Non-Coding RNA	4113	629	26	**655**
circRNAs	13342	562	91	**653**
other RNA Classes**	321	3	46	**49**
Total DEGs	33711	2665	269	**2934**
miRNAs	591	42	25	**67**

*Statistical significance of DESEq. 2 with p-value adjusted < 0.01 and Log2 Fold Change > |1.0|; **miscRNA, scaRNA, scRNA, snoRNA and snRNA were included in this category.

**Figure 3:**
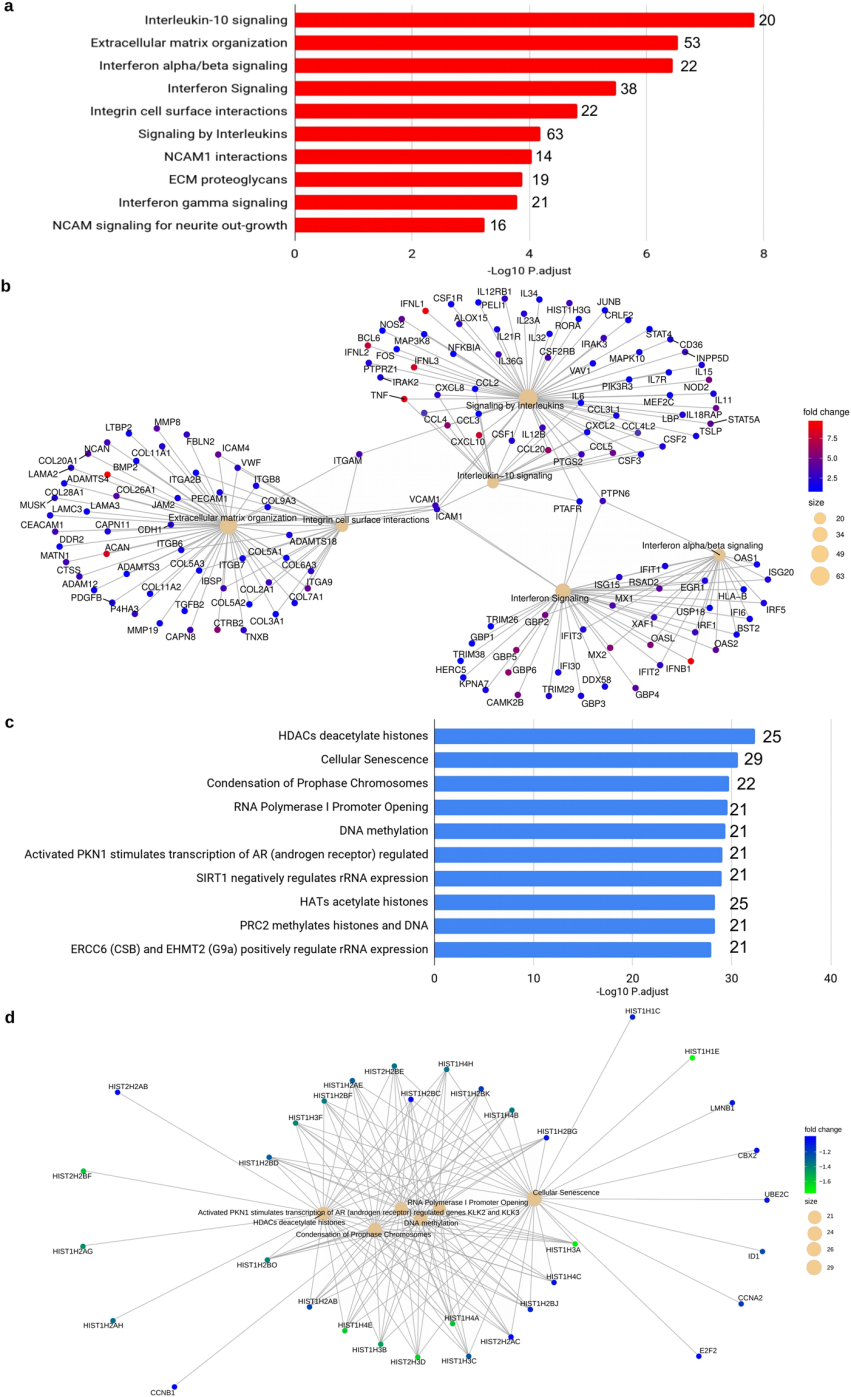
Enrichment Analyses of upregulated and downregulated genes from transcriptome of Zika virus-infected TEC. (**a**) Top 10 significant terms from the Reactome Enrichment Analysis of the upregulated genes. Horizontal red bars indicate the p-adjusted values (−Log_10_ scale) from the Reactome Enrichment Analysis. Numbers in the top of the red bars indicate the gene counting of each Reactome Term. (**b**) Cnetplot showing both upregulated genes and enriched Reactome terms. Pale yellow circles indicate the size of Reactome terms. The Log_2_ Fold Changes are indicated by the dot colors. Warmer colors indicate those most upregulated genes. (**c**) Top 10 significant terms from the Reactome Enrichment Analysis of the downregulated genes. Horizontal blue bars indicate the p-adjusted values (−Log_10_ scale) from the Reactome Enrichment Analysis. Numbers in the top of the blue bars indicates the gene counting of each Reactome Term. (**d**) Cnetplot showing both downregulated genes and enriched Reactome terms. Pale yellow circles indicate the size of the Reactome terms. The Log_2_ Fold Changes are indicated by the dot colors. Greener colors indicate the most downregulated genes.

The enrichment analysis of DEGs in ZIKV-infected TEC allowed to identify upregulated pathways involved with interleukin and interferon signaling, extracellular matrix organization, integrin cell surface interactions, NCAM1 interactions, NCAM signaling and ECM proteoglycans in Reactome enrichment (Fig. 3a,b, Supplemental Table 2). The GO enrichment analysis revealed categories also associated with extracellular matrix, response to virus and bacteria, cytokine production, defense response to virus (Supplemental Table 2). In addition, we found genes with enhanced expression by ZIKV-infected TEC such as CCL4, CCL5, CCL20, CXCL10, GBP2, GBP4, GBP5, GBP6, IFNB1, IFNL1, 2 and 3, IL12B, IL18RAP, MX1, MX2, NOS2, NOD2, OAS2, OASL, RSAD2, and TNF. Other upregulated antiviral genes were found, including IFIH1 and DDX58, which act as cytoplasmic sensors of viral nucleic acids, and DHX58, which mediates antiviral signaling and induction of type I interferons and proinflammatory cytokines (Supplemental Tables 1 and 2).

The Enrichment Analysis of Reactome also revealed upregulated genes included in extracellular matrix organization, and recognized as adhesion molecules, such as ADAMTS4, ACAM, ICAM1, ICAM4, VCAM1, ITGAM, PECAM1, CEACAM1, JAM2, MMP8, ITGA9, LAMA2 LAMA3, LAMA4, LAMC3, COL2A1, COL20A1, and COL26A1 (Fig. 3b). Interestingly, the GO enrichment revealed the category named as collagen fibril organization enriched, containing upregulated collagen fibril forming genes (COL2A1, COL3A1, COL5A1, COL5A2 COL5A3, COL11A1, and COL11A2), as shown Fig. 3 and Supplemental Table 2.

We also found upregulated genes that integrate extracellular matrix organization, interferon signaling, interleukin signaling, and integrin cell surface interaction, including ITGAM, VCAM1, ICAM1 in Reactome enrichment (Fig. 3). The transcriptome results were coherent with the cell growth assay that results in progressive reduction in the numbers of growing TEC, reduced cell proliferation and cell division, with a slight increase of apoptosis after ZIKV infection (Supplemental Table 2 and Fig. 2).

The Enrichment Analysis also revealed downregulated protein-coding genes related to histone 1 and 2 cluster, ubiquitin, cyclin, E2F Transcription Factor 2 (Fig. 3c, Supplemental Table 2). The downregulation of FBXO43, CKS2, AURKA, CCNB1, CDC20, UBE2C, CCNF, and CCNA2 genes could also contribute to the decreasing of cell growth (Fig. 2, Supplemental Tables 1 and 2) corroborating the reduction of cell proliferation *in vitro*. Moreover, we found downregulated genes (CCNB1, AURKA, PMAIP, and PLK3), which are also associated with the response to DNA damage.

### Long non-coding RNAs involved with an antiviral response in ZIKV-infected TEC

**Figure 4:**
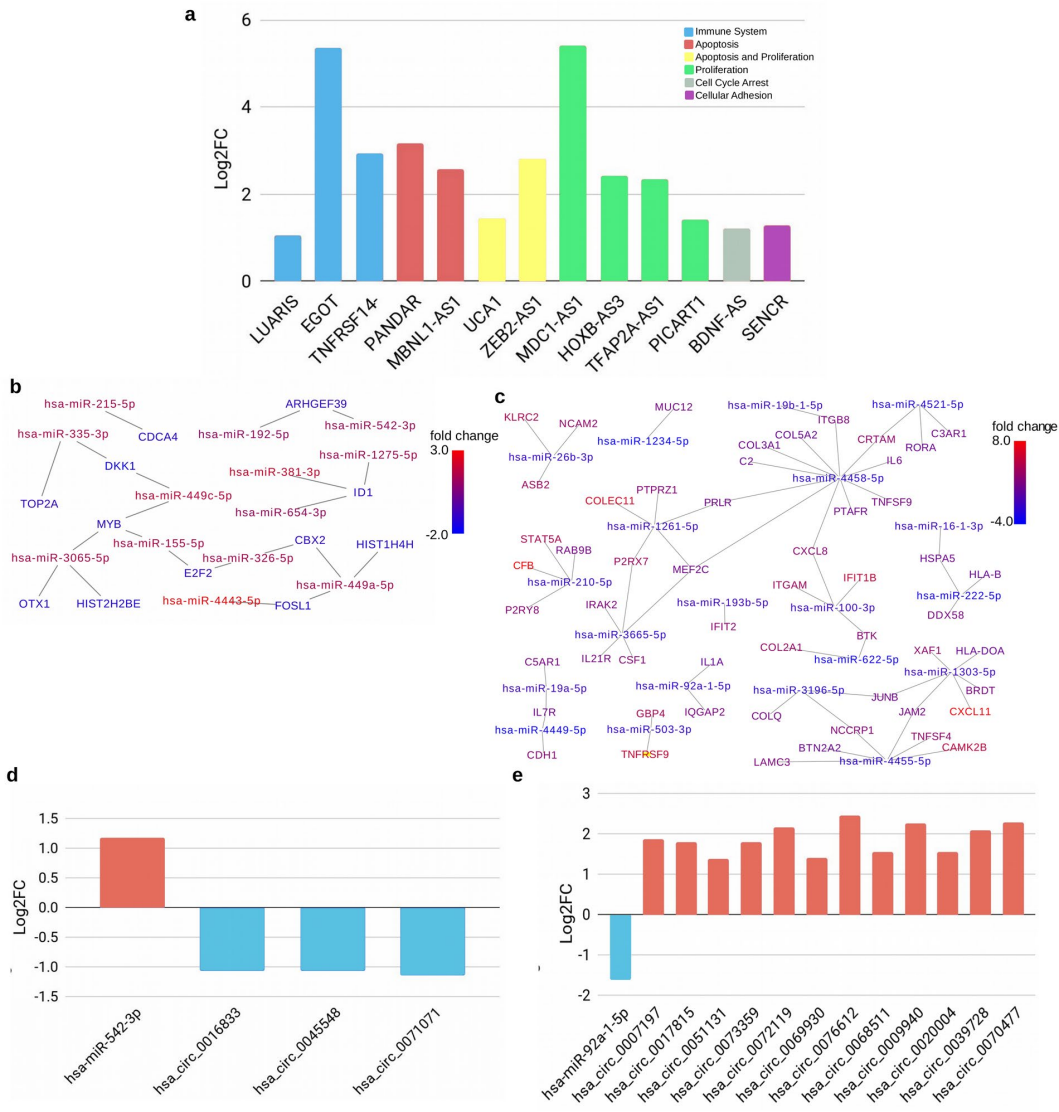
Non-coding genes response in human TEC infected with the Zika virus. (**a**) Expression of Long non-coding genes related to Immune System, Apoptosis, Apoptosis and Proliferation, Proliferation, Cell Cycle Arrest and Cellular Adhesion. (**b**) Network formed by MicroRNA and respective target transcripts, showing upregulated microRNAs and down-regulated targets. (**c**) Network formed by MicroRNA and respective target transcripts, showing down regulated microRNAs and upregulated targets. The Log_2_ Fold Changes were indicated by the continuous scaled colors. Warmer colors indicate upregulation, cooler colors indicate down regulation. (**d**) Expression analysis of Hsa-miR-542-3p and its putative circRNAs targets. (**e**) Expression analysis of Hsa-miR-92a-1-5p and its putative circRNAs targets. The data are expressed in Log2 Fold Change (Log2FC). Red and blue bars indicate up and downregulated genes, respectively.

The DGE analysis also identified 655 differentially expressed long non-coding RNAs (lncRNAs), with 629 upregulated and 26 downregulated in ZIKV-infected TEC (Table 1). Comprising among others, three upregulated lncRNAs, previously related with antiviral response (LUARIS, EGOT, TNFRSF14-AS1), two associated with regulation of apoptosis (PANDAR, MBNL1-AS1), two associated with both apoptosis and proliferation (UCA1, ZEB2-AS1), four with cell proliferation (MDC1-AS1, HOXB-AS3, TFAP2A-AS1, PICART1), one with cell cycle arrest (BDNF-AS) and one with cell adhesion (SENCR). Those lncRNAs showed log2 Fold Change ranging from 1.05 to 5.40 (Fig. 4a, Supplemental Table 1).

### miRNAs and circRNAs expression patterns reveal a fine control of transcription in ZIKV-infected TEC

Small RNA-seq analysis revealed 67 miRNAs differentially expressed in ZIKV-infected TEC, resulting in 42 upregulated and 25 downregulated microRNAs (Table 1 and Supplemental Table 1). The hsa-miR-7-3-3p was the most upregulated miRNA (log2 FC = 5.16) after ZIKV infection. The majority of miRNAs presented a slightly upregulation or downregulation with log2 FC > |1| including hsa-miR-155-5p (1.55) and hsa-miR-203a-3p (1.19).

The microRNA-target network (Fig. 4b,c) revealed 13 upregulated microRNAs that can modulate the expression of 12 downregulated target transcripts, including the hsa-miR-3065-5p regulating HIST2H2BE and MYB, hsa-miR-155-5p regulating MYB and E2F2, hsa-miR-449a-5p regulating HIST1H4H and hsa-miR-215-5p regulating CDCA4 (Fig. 4b). This analysis also showed 20 downregulated microRNAs, which target upregulated genes involved with immune system, viral response and extracellular matrix, as shown in hsa-miR-4458-5p that regulates the IL-6, CXCL8, C2, COL3A1, COL5A2, ITGB8, TNFSF9 genes, hsa-miR19b-5p targeting ITGB8 and CXCL8, hsa-miR-100-3p targeting CXCL8 and hsa-miR-222-5p regulating HLA-B and DDX58 genes (Fig. 4c).

The transcriptome analysis also revealed 562 upregulated and 91 downregulated circRNAs in ZIKV-infected TEC (Table 1). In order to identify the possible role of microRNA sponge by circRNAs, we conducted a microRNA target prediction with miRanda tool, using the circRNAs as targets. This strategy revealed the upregulated hsa-miR-542-3p targeting 3 circRNAs (Fig. 4d) and the downregulated hsa-miR-92a-1-5p, possibly regulated by 12 circRNAs (Fig. 4e).

Taken together, the transcriptome and small RNA-seq analyses revealed complex molecular responses of TEC in response to ZIKV infection, showing an intricate regulatory network, including different layers of transcription regulation. In this context, ICAM-1 and VCAM appeared as a main hub connecting distinct clusters of genes whose expression was altered secondary to ZIKV infection.

### Co-cultures of ZIKV-infected TEC with lymphoblastic T cells enhance heterocellular cell adhesion and virus transfer

**Figure 5:**
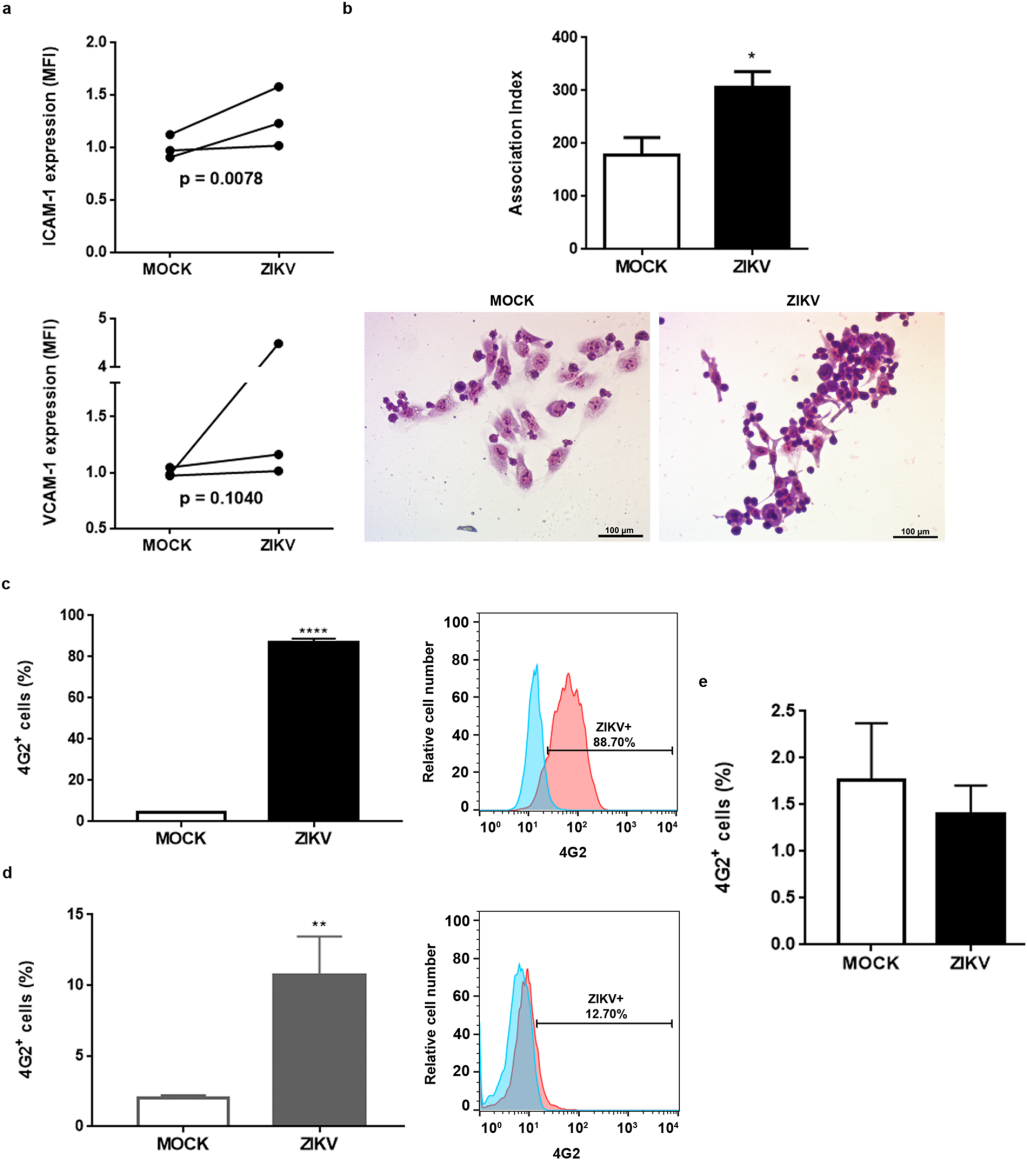
Zika virus infection in human cultured epithelial cells enhances cell adhesion and conveys the virus to lymphoblastic T cells. (**a**) Human TEC were infected with ZIKV (MOI = 1) and 72 hpi the expression of ICAM-1 (CD54) and VCAM-1 (CD106) were analyzed by flow cytometry. Graphs show the Mean Fluorescence Intensity (MFI) of these molecules. MFI values were normalized by the mean of the control group (n = 3). Human TEC were infected with ZIKV (MOI = 1) and 72 hpi uninfected CEM acute T-cell leukemia cell line was added to the culture. (**b**) Association index between CEM and TEC after 1 h of co-culture (adhesion). Values in the upper panel represent mean ± SEM. Representative microscopic fields after Giemsa staining are shown in the lower panel (n = 4). Relative expression of 4G2^+^ cells in (**c**) TEC and (**d**) CEM subpopulations were detected by flow cytometry after 24 h of co-culture. Values represent the mean ± SEM (n = 3, two experiments in duplicate). (**e**) CEM cells were not directly infected by ZIKV (MOI = 1). CEM cells were infected with ZIKV (MOI = 1) for 2 h and 72 hpi 4G2^+^ cells were analyzed by flow cytometry. Values represent the mean ± SEM (n = 3). *p < 0.05, **p < 0.01, ****p < 0.0001 (as determined by the unpaired *t*-test).

Considering that the expression of cell adhesion related-genes was enhanced in ZIKV-infected TEC, and the fact that ICAM-1 as well as VCAM are adhesion molecules known to play a role in TEC thymocyte interaction^20^, we investigated whether these proteins were upregulated in the membrane of ZIKV-infected TEC. We found enhanced numbers of TEC expressing ICAM-1 (CD54 – data not shown), as well as enhanced ICAM-1 in infected cells (Fig. 5a). This was more clear-cut when we analyzed the cells expressing high amounts of ICAM-1 (5.2% (±1.2) of ZIKV infected cells and 2.6% (±0.5) for controls (p = 0.05). As regards the expression of VCAM-1 (CD106), we did find higher membrane expression levels in infected cultures, although the differences were not statistically significant (p-value = 0.1).

We searched for a functional change on the ability of infected TEC to bind to lymphoblastic T cells. For that, the CEM acute T-cell leukemia cell line, previously known to adhere to human TEC^16^, was led to adhere to growing TEC 72 hours post-infection. Figure 5b shows that adhesion was significantly increased onto ZIKV-infected TEC cultures, as compared to controls. Importantly, we showed that 72 hours after TEC infection plus 24 hours of co-culture with CEM, a proportion (10.76% ± 1.98) of the T lymphoblasts were infected as revealed by immunolabeling with the 4G2 antibody (Fig. 5d). At this time point (96 hours post-infection), 86% of the TEC present in the co-culture were infected (Fig. 5c). Interestingly enough, we could not directly infect CEM cells with ZIKV (Fig. 5e) or with Zika virus-infected TEC supernatant (data not shown).

### The human thymic epithelium is altered after congenital *in vivo* ZIKV infection

**Figure 6:**
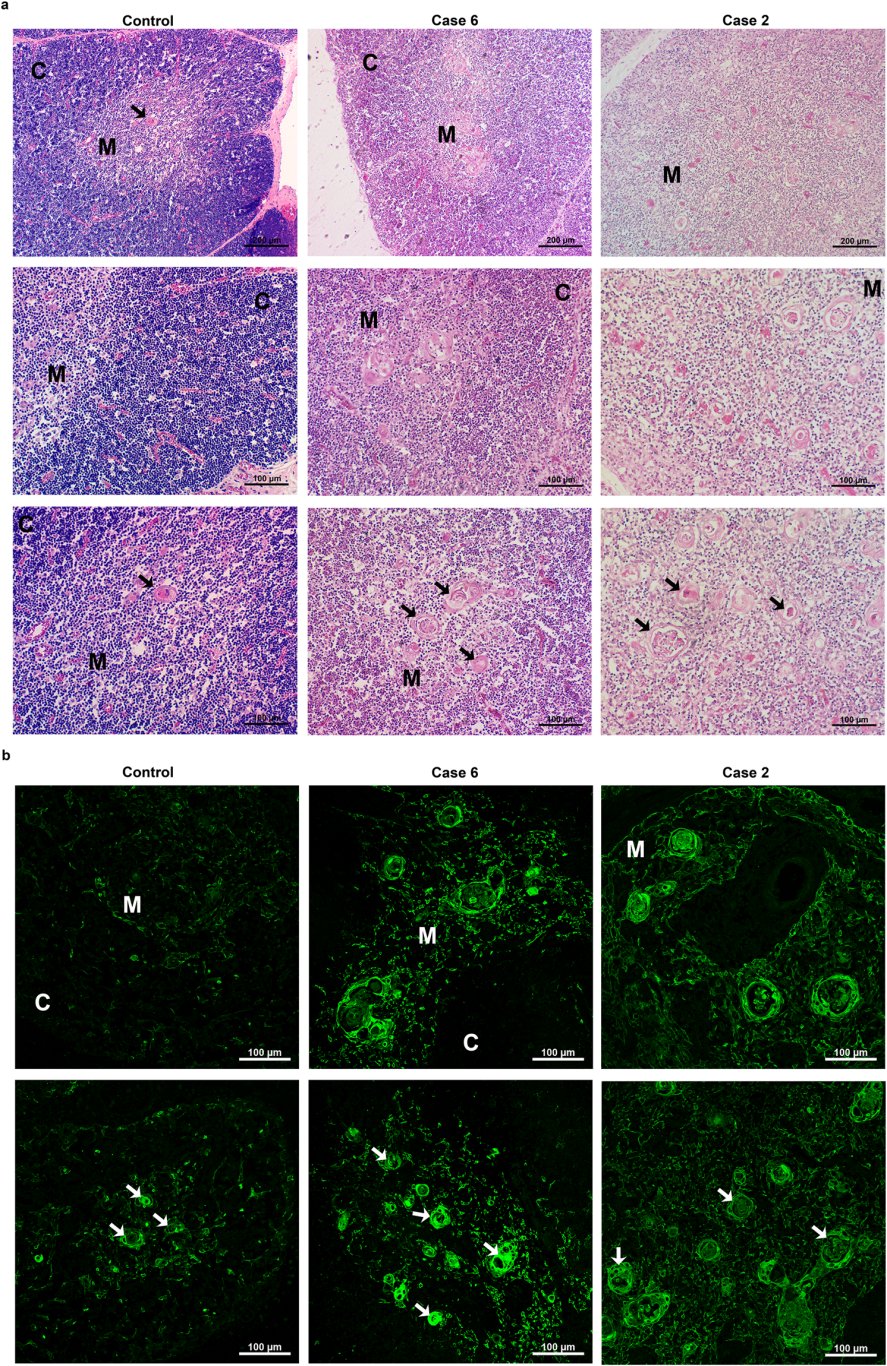
Congenital Zika virus infection induces major changes in the human thymic epithelial network. Representative images of (**a**) Hematoxylin-eosin and (**b**) cytokeratin staining of paraffin-embedded human thymus sections. A control case, case 6 with intermediate alterations and case 2 with severe alterations in the human thymic epithelial network are represented (n = 2 for controls and n = 6 for congenital ZIKV infection). Arrows show some of the Hassal’s corpuscles. C: cortical region; M: medullary region.

We also studied sections of six thymuses from children that died in postnatal life born from mothers infected by ZIKV during pregnancy. The histological profile of these specimens varied from virtually normal cortico-medullary pattern to a severe disruption of the cortex in one thymus, with clear-cut decrease in thymocytes (Fig. 6a). Yet, in all cases, we observed an increase in the numbers of the Hassall’s corpuscles, indicating an over differentiation of the TEC network. This feature was further confirmed by immunohistochemistry for cytokeratin detection (applied herein as a pan-marker of the whole TEC network) in TEC cultures, as seen in Supplemental Fig. 1. In fact, cytokeratin immunostaining revealed, in some thymus samples, a complete loss of the cortical/medullary distinction (Fig. 6b, case 2).

**Figure 7:**
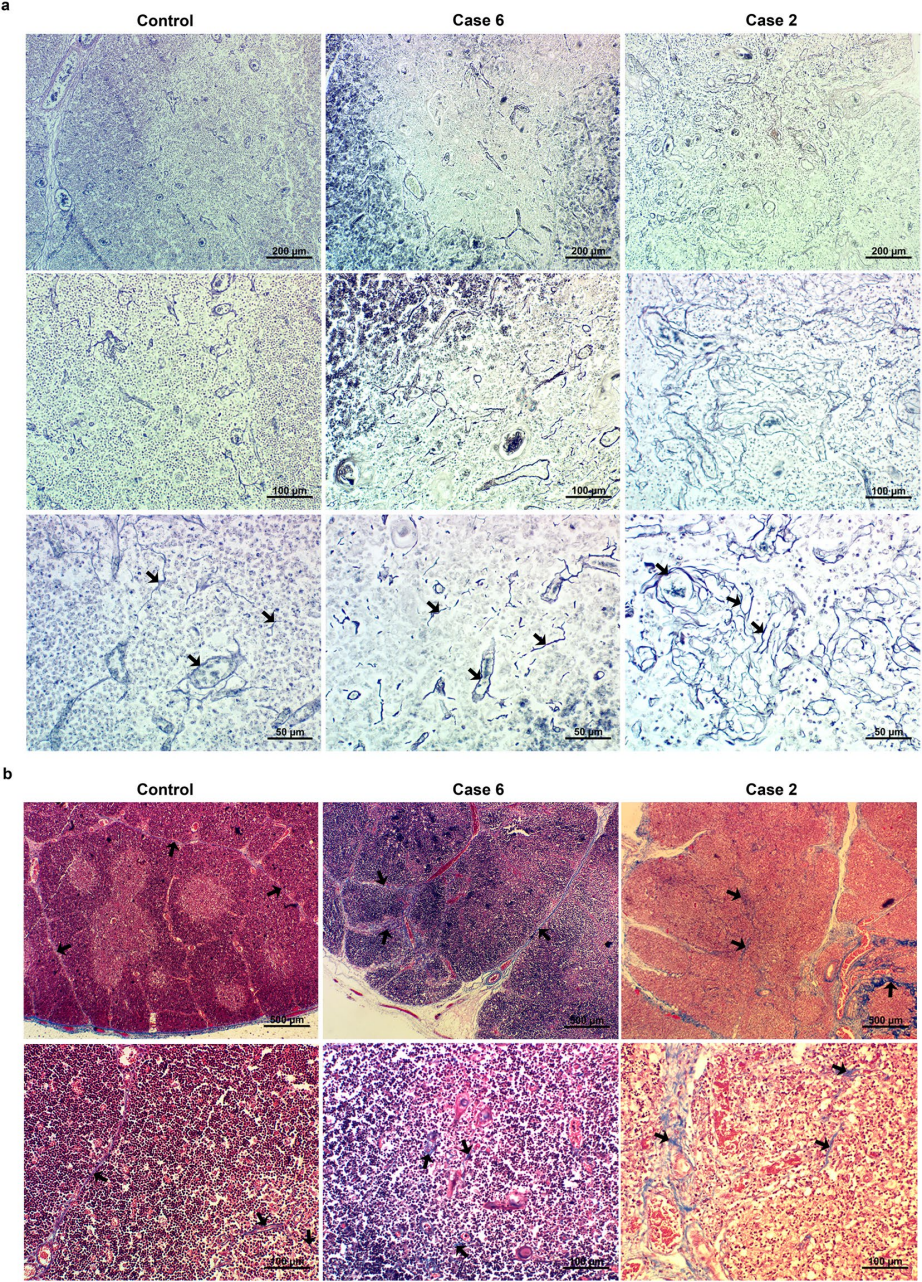
Congenital Zika virus infection induces changes in the human thymic extracellular matrix. Representative images of (**a**) Gomori’s reticulin (dark blue fibers) and (**b**) Masson’s trichrome staining (blue fibers) of paraffin-embedded human thymus sections. A control case, case 6 (with intermediate alterations) and case 2 (with severe alterations) in the human thymic epithelial network are represented. Arrows show some collagen fibers present in the images. (n = 2 for controls and n = 6 for congenital ZIKV infection).

Considering the transcriptome data, we also performed histological evaluations for ECM-containing fibers and fibrils. In the thymuses from ZIKV-infected newborns, there was an important increase in the reticulin network, indicating thickness of basement membranes in the thymic microenvironment. Additionally, Masson’s trichrome staining revealed a higher density of type I collagen-containing fibers in the thymic *septae*. These data are summarized in Fig. 7.

**Figure 8:**
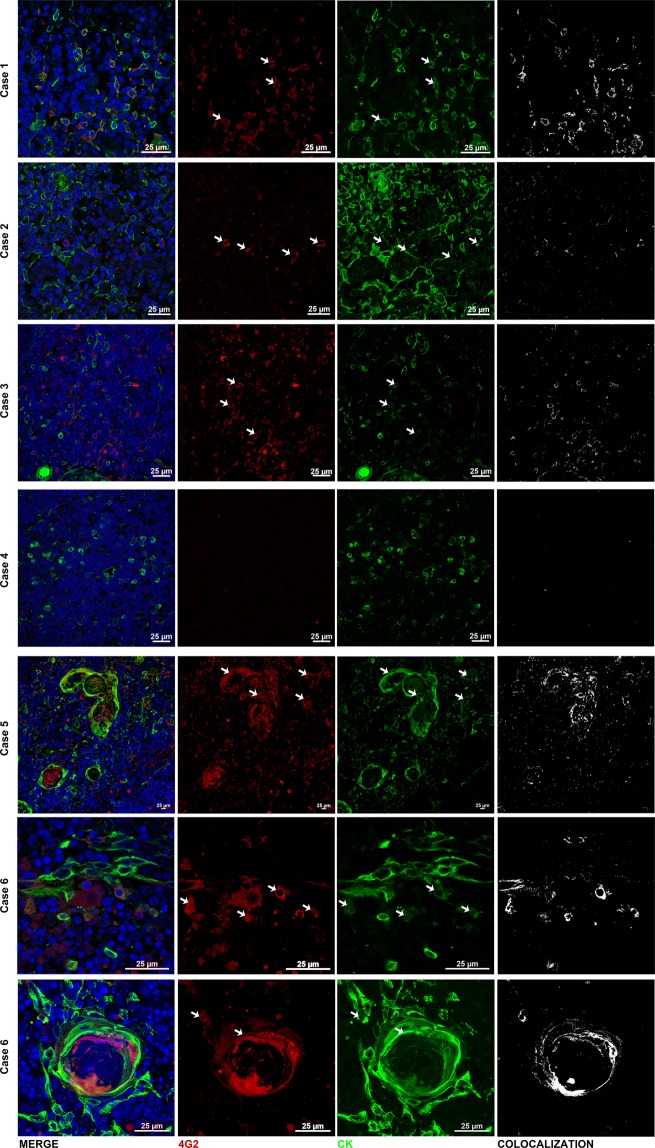
ZIKV envelope protein is found in human thymus of babies with congenital Zika virus infection. Representative images of immunofluorescence staining of 4G2 (viral protein, in red), cytokeratin (in green) and DAPI (nuclei, in blue) of paraffin-embedded human thymus sections (n = 2 for controls and n = 6 for congenital ZIKV infection). First column shows the merge of the three stainings. The last column shows the colocalization analysis for 4G2 and cytokeratin stainings; white points represent overlap between these two immunostainings. We could identify viral protein in five out of the six thymuses studied; most of cells labeled being epithelial. Arrows show some of the cells that were stained for both 4G2 and cytokeratin. Differences in bar sizes are due to the digital zoom used during image acquisition.

We also searched for the presence of the viral envelope protein in the same thymuses, using the 4G2 antibody, which recognizes any Flavivirus family envelope protein. As compared to normal thymuses (that were completely negative for 4G2 labeling), we did find specific immunostaining in thymus sections from 5 out of 6 thymuses evaluated. The presence of the envelope viral protein was seen in scattered TEC, both in the cortex and medulla of the thymic lobules, as well as in Hassall’s corpuscles (Fig. 8). It is important to point out that one thymus was positive for the presence of ZIKV ascertained by the RT-PCR (case 4 - Supplemental Table 4), although we were not able to detect the viral envelope protein in this case (case 4 – Fig. 8 and Supplemental Fig. 2a). None of the thymus preparations were positive for the presence of Dengue as well as Chikungunya viruses (Supplemental Table 4). Secondary antibodies were used as negative controls and did not generate any significant labeling (Supplemental Fig. 2b).

## Discussion

The behavior of the human immune system secondary to a congenital syndrome caused by the ZIKV remains largely unknown, although there is evidence that innate and acquired immune cells can be infected by the virus, as ascertained by studies on peripheral blood leukocytes^21,22^. Nevertheless, whether primary lymphoid organs are targeted by the ZIKV, as well as putative consequences upon interactions with B- and T-cell precursors and their corresponding microenvironments, remain to be demonstrated. Herein we tackled this issue by investigating whether or not the human thymic epithelium could be infected by ZIKV.

We first demonstrated that growing human TEC could indeed be infected, and that such infection occurred through the AXL receptor. Moreover, viral particles produced within TEC were infective. Interestingly, the human TEC line could also be infected with other RNA viruses, namely the retrovirus HTLV-1^15^. Moreover, pioneer work has shown that primary cultures of human TEC can also be infected with HIV^12^. This shows that human TEC is permissive to different types of RNA viruses.

Importantly, ZIKV did trigger an antiviral machinery response in human TEC, as ascertained by the upregulation in the expression of several genes related to antiviral response, including, among others, those related to the type 1 interferon signaling.

Major changes in TEC growth were also found after *in vitro* ZIKV infection, including a decrease in proliferation and increase in cell death, resulting in a reduction of total cell numbers after 3-day TEC cultures. RNA viruses such as ZIKV use strategies to modulate cell-cycle control and cell proliferation from the host and obtain cellular conditions favorable for their replication^23,24^. Accordingly, the data from RNA sequencing revealed few downregulated genes associated with the regulation of cell proliferation and apoptosis. These results are consistent with previous studies on ZIKV-infected human cell types, such as Sertoli cells in the seminiferous tubules, retinal pigment epithelium, neural progenitors, and astrocytes, which reported suppression of cell proliferation, as well as induction of antiviral defense pathways^24–27^.

It is known that adhesion of developing T-cells to the thymic epithelium is controlled by various cell-cell and cell-extracellular matrix interactions, including ICAM-1, VCAM-1, fibronectin, laminins, and their corresponding integrin receptors^28–32^. We found a large number of upregulated genes related to extracellular matrix, cell adhesion, and cell migration. Significant increase in the gene expression of various laminin alpha-chains as well as fibril forming collagen chains was seen in ZIKV-infected TEC, as well as higher levels of VCAM1 and ICAM-1 expression in TEC after ZIKV infection.

Enhanced expression of some matrix metalloproteinase genes, such as MMP8 and MMP19 (both having collagenolytic activity) was also observed, suggesting that the ECM-related metabolism of TEC is altered by ZIKV. Moreover, ZIKV augmented the gene expression of various chemokines; some of which (CCL4, CCL5, CCL20, and CXCL10, for example) being involved in intrathymic T-cell migration^33–36^.

In keeping with the transcriptome data related to extracellular matrix, cell adhesion, and cell migration, we demonstrated that infected TEC expressed higher levels of the ICAM-1 protein on their membranes. In keeping with the literature showing that ICAM-1 plays a major role in TEC adhesion to developing T-cells^37^, we found that lymphoblastic T cells adhered more to ZIKV-infected TEC than to uninfected TEC cultures. We can thus hypothesize that enhanced expression of adhesion molecules and chemokines would facilitate the encounter of developing T-cells with ZIKV-infected TEC, favoring in one hand the transfer of the virus to lymphocytes, and on the other hand, the establishment of an immunological synapse between the two cell types. This should also be placed in the context of the control of HLA-B gene expression, which was upregulated (Fig. 3b) in ZIKV-infected human TEC.

The control of gene expression in ZIKV-infected human TEC was also evaluated through the analysis of different non-coding RNAs. In fact, our robust transcriptome and microRNA expression analyses in ZIKV-infected TEC highlighted the molecular response of protein-coding genes and non-coding genes (lncRNAs, circRNAs and miRNAs). We observed miRNAs acting as probable regulators of genes associated with immune response, such as hsa-miR-222-5p (DDX58 and HLA-B), hsa-miR-1303-5p (HLA-DOA), hsa-miR-193b-5p (IFIT2), hsa-miR-100-3p (IFIT1B). Some miRNAs also have shown response to Cytokine Signaling in Immune system such as hsa-miR-503-3p (TNFRSF9) and hsa-miR-4458-5p (TNFSF9). The chemokines CXCL8 and CXCL11 could also be regulated by miRNAs in ZIKV-infected TEC. Taken together, these analyses indicate that miRNAs may play a relevant role in the intracellular control of antiviral immunity against ZIKV-infected TEC.

We also identified several cirRNAs which can serve as miRNA sponges and are crucial regulators of gene expression^38^. Some circRNAs present antiviral activity, interacting directly with the antiviral genes DDX58 (RIG-I), IFIH1 (MDA5) and TLR3^39^. The major class of non-coding lncRNAs also shows antiviral activity and involvement in cell proliferation and apoptosis. The LUARIS lncRNA expression level was decreased, indicating that it may be suppressed by IFNB1, which, in turn, was highly induced in ZIKV-infected human TEC. The expression of some interferon stimulated genes (ISGs) and chemokines showed altered levels of transcripts, indicating that LUARIS could act in antiviral immunity. The same regulation pattern of ISGs and chemokines was found in infection by other human viruses such as HBV, HCV, and EMCV^40^. Following ZIKV infection in TEC, we also detected an upregulation of the lncRNA EGOT, which is also induced after HCV infection, increasing viral replication^41^.

Studies involving lncRNA associated with cell proliferation and apoptosis and cell adhesion have been already described. We hypothesize that upregulation of PICART1 may also suppress proliferation and induce apoptosis in ZIKV-infected human TEC, as shown by other authors that this lncRNA impairing proliferation^42^ and inducing apoptosis^43^ in lung cancer cells. Another lncRNA, SENCR can stabilize the vascular endothelial cell adherent junctions^44^, which mediate cell adhesion and intracellular signals^45^. Accordingly, the upregulation of SENCR may be involved in regulating the enhancement of cell adhesion seen in ZIKV-infected TEC. The transcriptome and miRNA sequencing also revealed that genes associated with adhesion and ECM molecules were activated by infection by ZIKV, including coding and non-coding genes. Overall, our findings highlight a high complexity in the intracellular circuitry controlling ZIKV replication within human TEC.

In addition to extended *in vitro* studies showing that human TEC is infected and respond to ZIKV, we evaluated the presence of the virus as well as the general thymus microarchitecture in histological sections obtained from children who died early in life, whose mothers were infected with ZIKV during pregnancy. Although the number of specimens was low, thymuses were widely affected, varying from massive loss of cortical thymocytes, to preservation of the cortex in some cases. Within the thymic lobules and septae, we could see an increase in ECM deposition, as revealed by histological staining of collagen-containing fibrils. Most importantly, there was an alteration in the thymic epithelial network with a clear-cut increase in the formation of Hassall’s corpuscles, denoting a higher degree of keratinization (corresponding to terminal epithelial cell differentiation). In the same vein, we observed an increase in keratin contents in ZIKV-infected TEC cultures. It is important to point out that such enhancement of Hassall’s corpuscles is also found in cases of glucocorticoid treatment of patients with primary immunodeficiency^46^, as well as in mice treated with dexamethasone^47^.

In 5 out of the 6 thymuses analyzed, we detected the presence of viral protein colocalized with TEC, as ascertained by immunofluorescence using the 4G2 anti-flavivirus and anti-cytokeratin antibodies. Not only we detected the viral protein in Hassall’s corpuscles, as briefly reported by Valdespino-Vázquez and co-workers^48^, but also in TEC outside the corpuscles and that are located in close contact with developing thymocytes.

Rather surprisingly however, the virus genome was detected, by using qRT-PCR, only in one case. This can be explained by the fact the material applied was the bulk of thymic tissues whereas the immunohistochemical data revealed detectable amounts of the virus only in some microenvironmental cells (most of them epithelial). Moreover, in this particular case we did not see 4G2^+^ cells in the thymus sections we examined. One conceivable explanation for the fact that the only thymic extract positive for qRT-PCR was negative for immunolabeling with the 4G2 monoclonal antibody is that in this particular case, the presence of the virus was restricted to one part of the thymus that was not evaluated by immunohistochemistry.

Finally, from a conceptual point of view, it is conceivable that the human thymic epithelium is a target for ZIKV infection, with general but also specific consequences, as for example the modulation of adhesion molecules, extracellular matrix and cytokines and chemokines known to modulate intrathymic T-cell development.

## Methods and Patients

### Human TEC line and Zika virus infection

The human postnatal TEC line^17^ was maintained in RPMI-1640 medium - supplemented with 10% fetal calf serum, 2 g/L sodium bicarbonate, 2 g/L HEPES, 1X antimycotic solution (Sigma-Aldrich) and 5 µg/mL of plasmocin prophylactic (InvivoGen) at 37 °C, in an atmosphere containing 5% CO_2_.

The ZIKV strain used in this study was RIO-U1, which was isolated in 2016 from urine of a patient from Rio de Janeiro state, Brazil (GenBank Accession number: KU926309), as described elsewhere^49^. Viral stocks of ZIKV Rio-U1 were prepared by infecting Vero cell monolayers. The titer (PFU/ml) and the genome integrity were determined as described elsewhere^49^. Samples of the stock were used in the TEC infection assays. Vero cell cultures were also applied to test the infectivity of virus particles derived from infected TEC.

Unless described elsewhere, growing human TEC was plated in T-25 flasks (10^5^ cells) and infected 24 h later with 0.1 or 1.0 multiplicity of infection (MOI) or with 1 mL of infected TEC-derived supernatants for 1 h at 37**°**C in an atmosphere containing 5% CO_2_. After infection, culture medium was changed and cells were maintained in culture for 72 h. Controls (MOCK group) corresponded to cell grown under the same culture conditions but not infected by ZIKV.

### CEM cell line and ZIKV infection

CEM is an acute T-cell lymphoblastic leukemia cell line that was maintained in RPMI-1640 medium supplemented as described above. ZIKV Infection was performed with 1.0 MOI, as described previously for peripheral blood mononuclear cells^50^.

### Viral Multiplication in TEC line

An aliquot of supernatant was collected every 24 h after infection for three days and frozen at −80 °C. Briefly, Vero cells were seeded in a 24-well plate (5 × 10^4^ cells/cm^2^), 24 h before inoculation. Serial dilutions of the supernatant were used to infect cell monolayers. After 1 h incubation at 37 °C, the supernatant was replaced by 2.4% CMC (carboxymethyl cellulose) in Earle’s 199 complete medium supplemented with 5% FBS, followed by incubation for 7 days at 37 °C. Cells were fixed with 10% formaldehyde, washed, and dyed with 0.4% crystal violet. Viral titer was determined from the numbers of plaques visualized.

### Inhibition of infection

TEC were plated and 24 h later incubated for 30 min at 37 °C in an atmosphere containing 5% CO_2_ with culture media containing 10 µg/mL of goat anti-AXL polyclonal antibody (R&D Systems). Identical concentrations of IgG from normal goat serum were used as control. IgG amounts present in normal goat serum were quantified in Nanodrop™ 2000/2000c Spectrophotometers (Thermo Fisher Scientific). Cells were then infected as described previously in the presence of anti-AXL. Our experiments on the role of the AXL receptor in ZIKV entry in TECs were based on the paper of Hamel and co-workers, showing that exposure of the human skin fibroblast cell line HFF1 to ZIKV (and to Dengue virus, used as a positive control) resulted in comparable numbers of infected cells that were inhibited 70% and 50%, respectively, in the presence of the same neutralizing anti-AXL antibody^51^. Infection was quantified by flow cytometry as indicated below.

### Antibodies

See Supplementary Table 3.

### Flow cytometry

Intracellular staining of viral protein was performed with True-Nuclear™ transcriptional factor buffer set (Biolegend). Cells were incubated with 1x Fix Concentrate buffer for 40 min at 4 °C, followed by incubation with normal human serum for 20 min at 4 °C. Then, cells were stained with 4G2 antibody for 1 h at 4 °C and stained with secondary antibody for 30 min at 4 °C. After, cells were washed with 1x Perm buffer, as well as between each incubation. Subsequently, cells were fixed with formaldehyde 2% and evaluated by flow cytometry.

Cell proliferation status was performed by intracellular staining of Ki67 protein as indicated above for detection of viral protein, with the exception of that a single antibody incubation was realized for 30 min. We further evaluated those cells which are Ki67 high expressers, which correspond to those leaving cells in the mitosis phase of the cell cycle.

Cell death was analyzed by staining with Annexin-V-APC (Immunotools) and PI (Propidium iodide– Sigma-Aldrich). Cells were resuspended in 100 µL of 1x Annexin-V binding buffer (BD Biosciences) and incubated with Annexin-V-APC (1 µL) for 10 min at room temperature. PI (10 µg/mL) was added to the suspension prior acquisition on the flow cytometer.

To analyze ICAM-1 (CD54) and VCAM-1 (CD106) expression cells were incubated with normal human serum for 20 min at 4 °C and then incubated with CD54-PECY5 or CD106-APC antibodies for 30 min at 4 °C. After incubations cells were washed with PBS. Then, the cells were fixed with formaldehyde 2% and evaluated by flow cytometry.

The fluorescence intensity of labeled TEC was determined with FACSCanto II flow cytometer (BD Biosciences) Analyses were carried out using the BD FACSDiva 6.1.3 software (BD Biosciences).

### Immunofluorescence

TEC were seeded in 8 well (10^3^ cells/well) Lab-Tek^®^ Chamber Slide™ System and infected as described previously. After infection, the slides were washed twice with PBS and fixed in 4% paraformaldehyde for 15 min. Slides were washed again and treated with permeabilization buffer (0.1% saponin and 1% BSA – bovine albumin serum) for 15 min at 37 °C. Thereafter, samples were incubated for 40 min with blocking solution (0.2% normal goat serum, 1% FBS, 1% BSA, 0.3% X-100 triton) and submitted to staining with primary antibody for 1 h and with secondary antibody for 30 min. After staining, the slides were washed twice with permeabilization buffer, stained with DAPI for 10 min, washed and mounted with Dako Fluorescent Mounting Medium (DAKO).

Immunostained samples were analyzed by an AxioImager A2 device using the AxioVision Rel 4.8 software (Zeiss) or by Leica TSC SP8 Confocal device using LAS-X Software (Leica Microsystems). Negative controls, in which the secondary antibody was used alone, did not generate any significant labeling. The percentage of infection was achieved by the number of 4G2^+^ cells divided by the total number of cells per field. We analyzed 5 fields from 3 independent experiments and each experiment was performed in triplicate. The quantitative fluorescence analysis of cytokeratin expression was performed by transforming the specific staining into an eight-bit grey image for analysis of the corrected total cell fluorescence (CTCF) through the ImageJ software (NIH). At least 50 cells per condition were analyzed CTCF is calculated by the integrated density minus the area of a given cell multiplies by the mean fluorescence of background readings. When necessary, brightness and contrast adjustments were performed in the entire images.

### TEC adhesion to lymphoblastic T cells

Human TEC were seeded (10^4^ or 5 × 10^4^ cells) and 24 h later infected as described previously. After 72 h in culture, CEM cells (human T-cell acute lymphoblastic leukemia cell line) were added to the infected culture in a proportion of 50 CEM cells per TEC. We led these cells to adhere onto ZIKV-infected or non-infected-TEC during 1 h in serum-free RPMI medium at 37 °C in an atmosphere containing 5% CO_2_. Non-adherent CEM cells were gentle washed out with PBS at 37 °C. Culture flasks were fixed with methanol for 7 min and stained with Giemsa. The number of adherent CEM cells per at least 500 TEC were counted and the association index (AI) was calculated by the following formula, previously validated for this type of analysis^52,53^.

$${\rm{AI}}=\frac{{\rm{number}}\,{\rm{of}}\,{\rm{TEC}}\,{\rm{with}}\,{\rm{CEM}}\,{\rm{cells}}\,\times \,{\rm{number}}\,{\rm{of}}\,{\rm{CEM}}\,{\rm{cells}}\,{\rm{bound}}\,{\rm{to}}\,{\rm{TEC}}}{{\rm{total}}\,{\rm{TEC}}\,{\rm{number}}}\times 100$$

### Thymic epithelial and lymphoblastic T cell co-cultures

TEC were seeded in T-25 flasks (5 × 10^4^ cells) and 24 h later infected as described previously. Seventy-two hours post-infection, the medium was removed and CEM cells were added to the culture in a proportion of 25 CEM cells per TEC with fresh RPMI medium supplemented with 15% FBS. After 24 h of co-culture, non-adherent cells were removed by washing the flasks with PBS. Remaining adherent cells were recovered after addition of trypsin to the flasks. Both adherent and non-adherent cells were stained with 4G2 and anti-cytokeratin antibody by flow cytometry, as described previously. Anti-cytokeratin antibody was used for distinguishing CEM cells and TEC in adherent and non-adherent cells suspensions.

### RNA extraction

TEC were seeded in T-25 flasks (10^5^ cells) and 24 h later infected as described previously. Seventy-two hours post-infection, RNA extraction was performed with MOCK and ZIKV-infected TEC using RNeasy Plus Mini Kit (Qiagen, Hilden, Germany), following the manufacturer’s instructions for mRNA and small RNA extraction. Total RNA concentration and purity were determined by Nanodrop spectrophotometer. RNA integrity was verified through Agilent 2100 Bioanalyzer (Agilent Technologies).

### Transcriptome and small RNA library preparation and sequencing

The 12 transcriptome libraries were prepared from 500 ng of total RNA using the TruSeq Stranded Total RNA Sample Preparation Kit with Ribo-Zero Gold (Illumina). Additionally, the 12 small RNA libraries were prepared from 1 μg from the same total RNA of transcriptome analysis, using the TruSeq Small RNA Library Prep Kit (Illumina). All the steps of the protocols were carried out without any modifications, with the exception of the cDNA construction purification step on the small RNA protocol, which was performed using 3% Agarose Gel Cassette for targets between 100 bp − 250 bp in a BluePippin system (Sage Science). The quality control of each library was performed using the 2100 Bioanalyzer System with the Agilent High Sensitivity DNA Kit (Agilent). The libraries were quantified via qPCR using a KAPA Library Quantification Kits for Illumina platforms (KAPA Biosystems). The Libraries were sequenced on a NextSeq. 500 sequencing system (Illumina), for the RNA-seq paired-end reads (2 × 75 bp) were obtained using NextSeq. 500/550 High Output v2 kit (150 cycles) and for the small RNA, single-end reads (1 × 50 bp) were obtained using a NextSeq. 500/550 High Output v2 kit (75 cycles).

### Transcriptome analysis

The 12 transcriptome and 12 small RNA libraries were quality checked with FASTQC tool (https://www.bioinformatics.babraham.ac.uk/projects/fastqc/) and further trimmed with Trimmomatic tool^54^. For small RNAse libraries, only reads with length between 16-28 nucleotides were kept for posterior analysis. The high-quality transcriptome libraries were mapped separately on the Human genome (GRCH38.p12) from ENSEMBL databank (http://www.ensembl.org/info/data/ftp/index.html) using the STAR mapper (Version 2.6)^55^, with default parameters. The small RNA reads were mapped in the human microRNA precursors retrieved from miRBase (Release 22) (http://www.mirbase.org/) with Bowtie2 tool (version 2.2.1)^56^ running with default parameters.

The Differential Gene Expression (DGE) analysis was performed with DESeq. 2 package^57^ from R. The genes (protein-coding, lncRNAs, circRNAs, and other transcripts) and microRNAs with adjusted p-value < 0.01 and Log2 Fold Change > |1.0| were considered for further analyses. Both transcriptome and microRNA expression analysis was carried out in six biological replicates.

In order to identify microRNAs and their real targets, we retrieved the experimentally validated microRNA-target interactions from miRTarBase (http://mirtarbase.mbc.nctu.edu.tw/php/index.php) plus the predicted ones from SpidermiR package^58^, which includes DIANA^59^, miRanda^60^, PicTar^61^ and TargetScan^62^ microRNA target datasets. We compared with differentially expressed microRNAs and target genes from both transcriptome and small RNA seq experiments.

### Circular RNA (circRNAs) Analysis

Using the 12 transcriptome libraries, we performed a DGE analysis with DESEQ. 2 library^57^ of the circular RNAs (circRNAs) from human genome, based on circBase (http://www.circbase.org/). The circRNAs with adjusted p-value lower than 0.01 and Log_2_ Fold Change > |1.0| were used to characterize the interaction of microRNAs and circRNAs with miRanda tool^60^, using strict 5′ seed pairing, minimum score threshold, alignment length and identity of 140, 17 and 80%, respectively. The interactions were visualized with Cytoscape tool^63^.

### Enrichment analysis of DEGs

The Gene Ontology (GO)^64^ and ReactomePA^65^ were used to determine enrichment of biological processes and pathways, respectively on DEGs. We used the ClusterProfiler package from R^66^ for ReactomePA generation and GO enrichment by over-representation test. The enrichment was generated with Benjamini & Hochberg (BH) adjusted p-value method with a p-value cutoff of 0.01.

### Human thymus histology and immunostainings

We evaluated human thymus fragments from children who died up to 6 months after birth, and whose mothers were infected with ZIKV during pregnancy. The clinical features of these newborns are summarized in Supplemental Table 4. The use of organ histological sections for research has been approved by the Fernandes Figueira National Institute for the Health of Mother, Child, and Adolescent, Oswaldo Cruz Foundation review board under the number CAAE: 52675616.0.000.5269. All participants provided written informed consent. All methods were performed in accordance with the relevant guidelines and regulations. General histological profiles were evaluated through haematoxin-eosin staining. Gomori’s reticulin and Masson’s trichrome staining procedures were applied for revealing extracellular matrix containing compartments.

In brief, paraffin-embedded thymus sections (4 µm) were maintained at 65 °C overnight. The slides were deparaffinized in two passages in xylene and three passages in absolute alcohol for two min each. Samples were hydrated in distilled water and the peroxidase blockade was performed. The slides were washed with PBS and antigenic recovery was performed in water bath at 96 °C with 10% Trilogy solution for 40 min. After cooling for 20 min, the slides were washed with PBS and blocked with 2% BSA for 40 min. Thereafter, samples were incubated overnight at 4 °C with anti-cytokeratin and 4G2 antibody. The slides were washed twice with permeabilization buffer and incubated for 30 min with secondary antibodies for 30 min. After being washed twice with permeabilization buffer, samples were incubated for 10 min with DAPI and washed again. Immunostained samples were analyzed by the Leica TSC SP8 Confocal device using LAS-X Software.

Two thymuses derived from 2 infants were applied for controls. Although in both cases there was a pathological situation associated (pulmonary hypoplasia), their normal histological profile was confirmed by hematoxylin-eosin staining. Sections of these specimens were subjected to the same histological and immunofluorescence procedures.

### Statistical analysis

Unless described elsewhere, the statistical significance was approached using unpaired one- or two-tailed *t*-test or One-way ANOVA followed by Tukey’s multiple comparison test.

## Supplementary information

Supplemental material.

Supplementary Table 1.

Supplementary Table 2.

## Data Availability

The RNA-seq and small-RNA-seq data that support the findings of this study have been deposited in SRA database from NCBI with the accession codes PRJNA560342 and PRJNA561078. All other data are available in the article and its supplementary information files or from the corresponding authors upon request.

## Code availability

We use already published algorithms. See Methods section for full description of each analysis including input data, library, and algorithm version used, for which we provide corresponding web links or references.
