# Supplementary material for: Zika virus targets the human thymic epithelium
Source: Sci Rep. 2020 Jan 28;10:1378. doi: 10.1038/s41598-020-58135-y (PMC6987159; doi:10.1038/s41598-020-58135-y)
Supplement: Supplementary file 1 — Supplemental material. [file 41598_2020_58135_MOESM1_ESM.docx]

**Supplemental material**

**Zika virus targets the human thymic epithelium**

Carolina V. Messias, Guilherme Loss-Morais, Joseane Biso de Carvalho, Mariela N. González, Daniela P. Cunha, Zilton Vasconcelos, Luis W. P. Arge, Désio A. Farias-de-Oliveira, Alexandra L. Gerber, Elyzabeth A. Portari, Nilma Ferreira, Lidiane M. S. Raphael, Myrna C. Bonaldo, Ingo Riederer, Maria E. Lopes Moreira, Vinicius Cotta-de-Almeida, Ana T. R. Vasconcelos, Daniella A. Mendes-da-Cruz, Wilson Savino

**
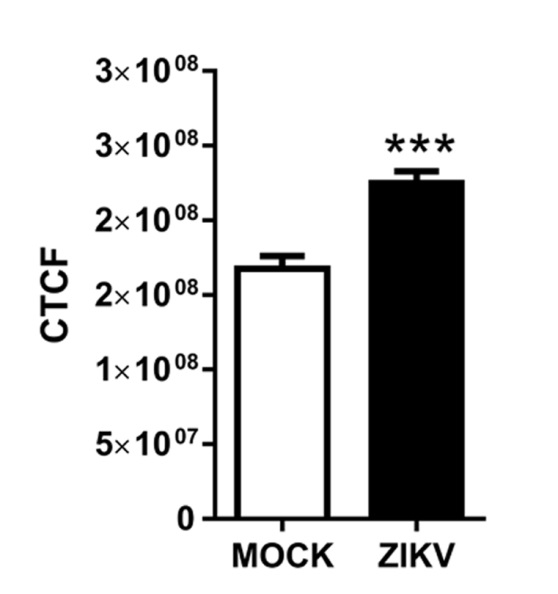
**

**Supplemental Figure 1. Zika virus-infected TEC has increased cytokeratin levels *in vitro*.** Corrected total cell fluorescence (CTCF) of immunofluorescence cytokeratin staining in TEC infected *in vitro* with ZIKV (MOI=1), 72 hours post-infection (hpi). Values represent mean ± SEM (n=3, in triplicate). **p < 0.01, ***p < 0.001 (unpaired *t*-test).


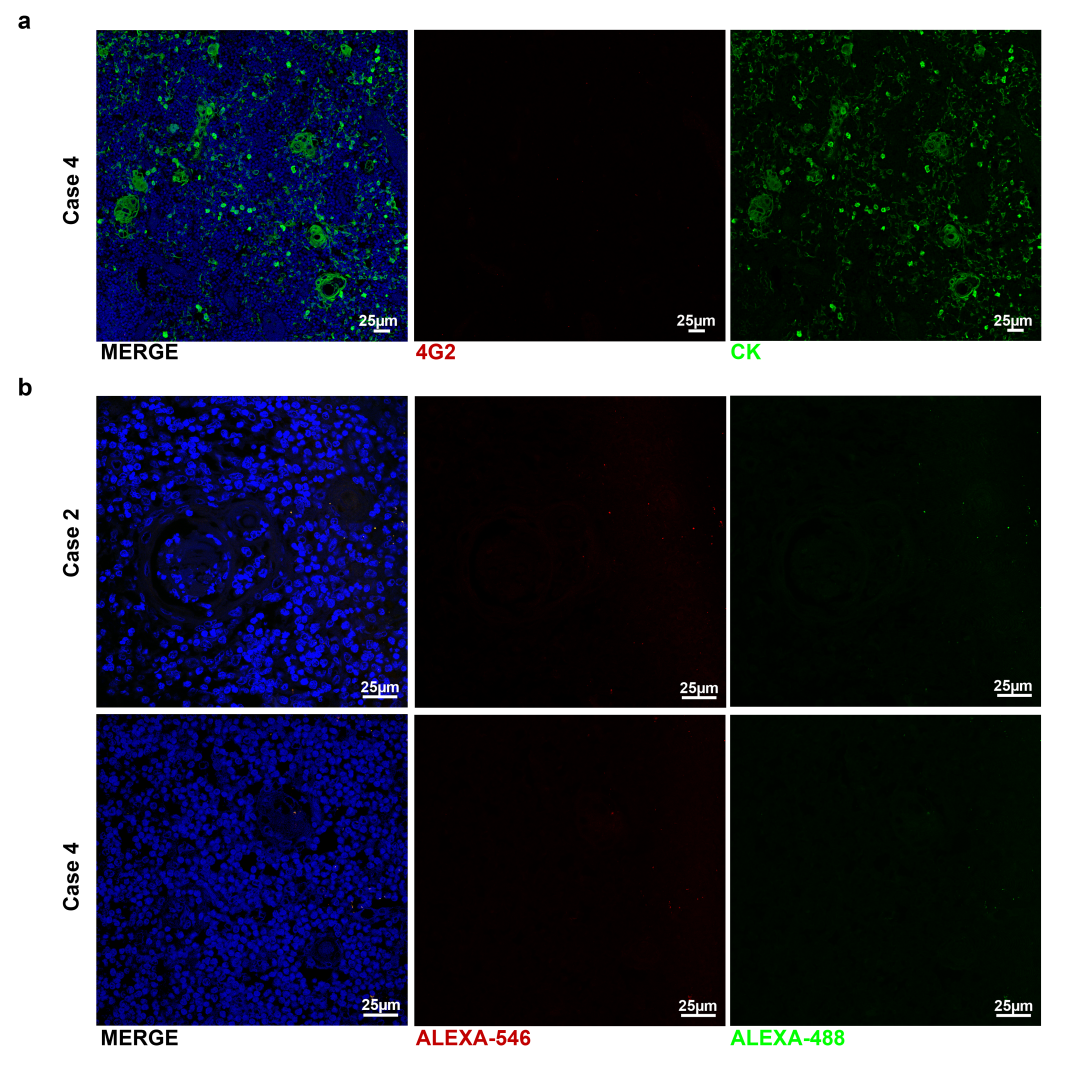


**Supplemental Figure 2:** (**a**) Representative image of immunofluorescence staining of 4G2 (viral protein, in red), cytokeratin (in green) and DAPI (nuclei, in blue) of paraffin-embedded human thymus sections from case 4. (**b**) Representative images of immunofluorescence staining of goat anti-mouse IgG Alexa Fluor^®^ 546 (used to detect viral protein, in red), goat anti-rabbit IgG Alexa Fluor^®^ 488 (used to detect cytokeratin, in green) and DAPI (nuclei, in blue) of paraffin-embedded human thymus sections from case 2 and case 4. Secondary antibodies were used as controls.

**Supplemental Table 1. List of differentially expressed genes, lncRNAs, miRNAs, other RNAs classes and circRNAs in cultured human TEC with or without ZIKV infection.**

**Supplemental Table 2. List of Reactome and GO enrichment analyses from up and downregulated genes sorted by significance.**

**Supplemental Table 3. Antibodies and isotype controls used in flow cytometry and immunofluorescence.**

| **Antibody or Isotype control** | **Company** | **Catalog number** | **Final Concentration** |
| --- | --- | --- | --- |
| Goat polyclonal anti-human TIM-1 | R&D systems | AF1750 | 4 µg/mL |
| Goat polyclonal anti-human TIM-4 | R&D systems | AF2929 | 20 µg/mL |
| Goat polyclonal anti-human AXL | R&D systems | AF154 | 2 µg/mL |
| Mouse monoclonal anti-human TYRO3 | R&D systems | MAB859 | 100 µg/mL |
| Mouse monoclonal 4G2 antibody (Anti-flavivirus - clone 4G2) | Bio-manguinhos (Rio de Janeiro, Brazil) | --- | 4 µg/mL |
| Rabbit polyclonal anti-cow Cytokeratin | Dako Co. | Z0622 | 214 µg/mL |
| Purified mouse IgG1 | eBioscience – Thermo Fisher Scientific | 14-4714-85 | 100 µg/mL |
| Purified goat IgG | Santa Cruz | SC2028 | 2, 4 or 20 µg/mL |
| Goat anti-rabbit IgG Alexa Fluor^®^ 488 | Invitrogen – Thermo Fisher Scientific | A11008 | 2.5 µg/mL |
| Goat anti-mouse IgG Alexa Fluor^®^ 546 | Invitrogen – Thermo Fisher Scientific | A11003 | 5 µg/mL |
| Donkey anti-goat IgG Alexa Fluor^®^ 488 | Invitrogen – Thermo Fisher Scientific | A11055 | 2.5 µg/mL |
| PECY5 mouse anti-human CD54 | BD Bioscience | 555512 | 1:5 (dilution) |
| APC mouse anti-human CD106 | BD Bioscience | 55147 | 1:5 (dilution) |
| PECY5 mouse IgG | eBioscience – Thermo Fisher Scientific | 15-4724-73 | 1:5 (dilution) |
| APC mouse IgG2b | BD Bioscience | 555745 | 1:5 (dilution) |

**Supplemental Table 4. General features of newborns who died in early postnatal life and whose mothers were infected by the Zika virus***

| **Case** | **Gender** | **Gestational age at birth** | **Gestational Trimester of infection** | **RT-PCR for ZIKV** | |  | **Clinical Manifestations** | | | |
| --- | --- | --- | --- | --- | --- | --- | --- | --- | --- | --- |
|  |  |  |  | CNS | Thymus |  | Microcephaly | Ventriculomegaly | Arthrogryposis | Pulmonary hypoplasia |
| 1 | Male | 41 weeks | Second | + | ND |  | - | + | + | + |
| 2 | Female | 38 weeks | First | + | - |  | + | + | + | - |
| 3 | Female | 40 weeks | Second | + | - |  | + | + | + | + |
| 4 | Female | 33 weeks | First | + | + |  | + | + | + | + |
| 5****** | Female | 38 weeks | First | + | - |  | - | + | - | + |
| 6*** | Female | 32 weeks | First | - | - |  | - | + | + | + |

* All cases were PCR negative for detection of dengue and chikungunya viruses; ** Congenital Syphilis; ***Positive for ZIKV in hypophysis; ND: not done.
