# Supplementary material for: Zika virus targets the human thymic epithelium
Source: Sci Rep. 2020 Jan 28;10:1378. doi: 10.1038/s41598-020-58135-y (PMC6987159; doi:10.1038/s41598-020-58135-y)
Supplement: Supplementary file 2 — Supplementary Table 1. [file 41598_2020_58135_MOESM2_ESM.pdf]

Zika virus targets the human thymic epithelium

Carolina V. Messias, Guilherme Loss-Morais, Joseane Biso de Carvalho, Mariela N. González,
Daniela P. Cunha, Zilton Vasconcelos, Luis W. P. Arge, Désio A. Farias-de-Oliveira, Alexandra L. Gerber,
Elyzabeth A. Portari, Nilma Ferreira, Lidiane M. S. Raphael, Myrna C. Bonaldo, Ingo Riederer,
Maria E. Lopes Moreira, Vinicius Cotta-de-Almeida, Ana T. R. Vasconcelos, Daniella A. Mendes-da-Cruz and
Wilson Savino

Supplemental Table 1: Raw Transcriptome expression data sorted by FDR significance

gene_symbol	class	ENSEMBL_ID	log2FoldChange	pvalue	padj
ATF3	protein_coding	ENSG00000162772	4,010440988	0	0
IFIT2	protein_coding	ENSG00000119922	2,758165659	0	0
IFIH1	protein_coding	ENSG00000115267	2,725554294	0	0
SLFN5	protein_coding	ENSG00000166750	2,606913477	0	0
TNFRSF10D	protein_coding	ENSG00000173530	1,498124589	0	0
CCN1	protein_coding	ENSG00000142871	-1,89685853	0	0
HBEGF	protein_coding	ENSG00000113070	2,380359431	2,54E-273	7,46E-270
IFIT3	protein_coding	ENSG00000119917	2,176770656	2,75E-258	7,05E-255
PLAT	protein_coding	ENSG00000104368	-1,288026748	8,17E-246	1,86E-242
PMAIP1	protein_coding	ENSG00000141682	2,723833772	6,94E-219	1,42E-215
HERPUD1	protein_coding	ENSG00000051108	1,65622856	7,87E-215	1,47E-211
CXCL8	protein_coding	ENSG00000169429	2,600747435	8,03E-214	1,37E-210
IFIT1	protein_coding	ENSG00000185745	1,872974901	7,19E-209	1,14E-205
CCL20	protein_coding	ENSG00000115009	5,960608054	6,82E-200	9,99E-197
CDCA4	protein_coding	ENSG00000170779	-1,261400098	3,47E-192	4,75E-189
OASL	protein_coding	ENSG00000135114	5,284170689	3,86E-192	4,95E-189
DNAJC3	protein_coding	ENSG00000102580	1,353611085	2,19E-176	2,64E-173
FILIP1L	protein_coding	ENSG00000168386	1,822805838	4,27E-176	4,87E-173
CSRNP1	protein_coding	ENSG00000144655	2,292498796	4,15E-172	4,48E-169
SNORD3B-2	snoRNA	ENSG00000262074	-2,639831236	7,92E-171	8,12E-168
SAMD9	protein_coding	ENSG00000205413	1,124039406	2,14E-168	2,09E-165
RF00012	snoRNA	ENSG00000212195	-3,022695604	6,5E-168	6,06E-165
PLAU	protein_coding	ENSG00000122861	-0,933859508	1,35E-163	1,21E-160
CCL5	protein_coding	ENSG00000271503	4,674060851	3,54E-162	3,02E-159
ERRFI1	protein_coding	ENSG00000116285	1,423362768	1,03E-160	8,47E-158
PPP1R15A	protein_coding	ENSG00000087074	1,659807292	8,52E-157	6,72E-154
HYOU1	protein_coding	ENSG00000149428	1,420280887	4,53E-155	3,44E-152
DDX58	protein_coding	ENSG00000107201	1,772570465	5,2E-155	3,81E-152
NOCT	protein_coding	ENSG00000151014	1,580460098	1,31E-149	9,24E-147

HIST1H1E	protein_coding	ENSG00000168298	-1,74275449	2,78E-146	1,9E-143
DNAJB11	protein_coding	ENSG00000090520	1,186676297	3,48E-146	2,3E-143
DNAJB9	protein_coding	ENSG00000128590	1,785416335	1,57E-145	1,01E-142
MXD1	protein_coding	ENSG00000059728	2,896058064	7,87E-144	4,89E-141
FOSB	protein_coding	ENSG00000125740	3,360863451	2,66E-142	1,6E-139
AC007952.4	lincRNA	ENSG00000262202	-2,677753635	3,64E-142	2,13E-139
AC109326.1	TEC	ENSG00000279602	-1,674108684	1,62E-141	9,2E-139
GPRC5A	protein_coding	ENSG00000013588	-1,46288514	5,14E-141	2,85E-138
ICAM1	protein_coding	ENSG00000090339	2,41919711	2,32E-139	1,25E-136
IRF1	protein_coding	ENSG00000125347	2,950260023	7,58E-139	3,98E-136
MANF	protein_coding	ENSG00000145050	1,143634104	1,49E-134	7,64E-132
RNVU1-19	snRNA	ENSG00000275538	-3,122857942	4,84E-134	2,42E-131
VXN	protein_coding	ENSG00000169085	2,768793234	1,69E-132	8,26E-130
IL7R	protein_coding	ENSG00000168685	1,431839297	2,01E-130	9,57E-128
SLC30A1	protein_coding	ENSG00000170385	1,064713851	1,17E-128	5,45E-126
SLC33A1	protein_coding	ENSG00000169359	1,180965644	1,04E-127	4,75E-125
IRAK2	protein_coding	ENSG00000134070	2,029259846	4,07E-127	1,81E-124
GEM	protein_coding	ENSG00000164949	1,575655172	1,25E-125	5,46E-123
XBP1	protein_coding	ENSG00000100219	1,059054043	3,05E-125	1,3E-122
ERN1	protein_coding	ENSG00000178607	2,615461805	1,12E-124	4,68E-122
FOSL1	protein_coding	ENSG00000175592	-1,37612245	8,14E-121	3,34E-118
PDIA4	protein_coding	ENSG00000155660	0,948981105	1,16E-120	4,65E-118
ZC3HAV1	protein_coding	ENSG00000105939	1,357258726	2,94E-120	1,16E-117
TGFB2	protein_coding	ENSG00000092969	1,331572785	1,36E-119	5,25E-117
RCAN1	protein_coding	ENSG00000159200	1,161727068	8,26E-119	3,14E-116
GBP5	protein_coding	ENSG00000154451	5,694231778	4,23E-118	1,58E-115
DDIT3	protein_coding	ENSG00000175197	2,329663544	8,88E-118	3,25E-115
CLDN1	protein_coding	ENSG00000163347	1,57652304	7,43E-117	2,68E-114
CAVIN2	protein_coding	ENSG00000168497	-1,147539484	1,53E-115	5,42E-113
DKK1	protein_coding	ENSG00000107984	-1,741902128	1,62E-115	5,64E-113
RF00003	snRNA	ENSG00000274210	-2,689991159	8,91E-115	3,05E-112
AKIRIN1	protein_coding	ENSG00000174574	0,747465644	6,29E-112	2,11E-109
RNF145	protein_coding	ENSG00000145860	-0,948534062	1,25E-110	4,12E-108
TRIM38	protein_coding	ENSG00000112343	1,058716013	2,69E-109	8,77E-107
CALR	protein_coding	ENSG00000179218	0,771539406	1,06E-104	3,4E-102
STAT1	protein_coding	ENSG00000115415	0,829550866	1,45E-104	4,58E-102
ZNF654	protein_coding	ENSG00000175105	1,336430368	3,68E-104	1,14E-101
BHLHE40	protein_coding	ENSG00000134107	1,431487541	3,11E-103	9,53E-101
DHX58	protein_coding	ENSG00000108771	2,507882391	1,35E-101	4,06E-99
HERC5	protein_coding	ENSG00000138646	1,31287876	1,63E-101	4,84E-99

PDIA3	protein_coding	ENSG00000167004	0,754988452	7,38E-101	2,16E-98
IFI44	protein_coding	ENSG00000137965	1,912090892	5,63E-100	1,63E-97
RNU1-1	snRNA	ENSG00000206652	-3,077379803	1,33E-99	3,78E-97
KITLG	protein_coding	ENSG00000049130	1,373673899	4,9E-99	1,38E-96
BTG1	protein_coding	ENSG00000133639	1,221029538	1,47E-98	4,06E-96
HECW2	protein_coding	ENSG00000138411	1,066454308	2,42E-98	6,62E-96
DUSP10	protein_coding	ENSG00000143507	1,019382704	3,05E-98	8,22E-96
PLEKHA4	protein_coding	ENSG00000105559	4,43879588	6,96E-97	1,85E-94
AURKB	protein_coding	ENSG00000178999	-0,988628897	2,6E-96	6,84E-94
FLRT2	protein_coding	ENSG00000185070	1,574392875	1,05E-95	2,72E-93
RNVU1-15	snRNA	ENSG00000207205	-3,002872577	1,3E-95	3,32E-93
SERPINE1	protein_coding	ENSG00000106366	-0,796113881	6,6E-95	1,67E-92
HRH1	protein_coding	ENSG00000196639	1,209688445	5,36E-93	1,34E-90
KLHL15	protein_coding	ENSG00000174010	1,182651522	1,93E-92	4,77E-90
SYT1	protein_coding	ENSG00000067715	2,22472915	1,99E-92	4,85E-90
TRANK1	protein_coding	ENSG00000168016	2,428953112	2,1E-92	5,06E-90
LACC1	protein_coding	ENSG00000179630	1,610637174	8,72E-92	2,08E-89
RNVU1-6	snRNA	ENSG00000201558	-3,586377985	1,13E-91	2,67E-89
CPEB4	protein_coding	ENSG00000113742	1,62998131	1,45E-91	3,38E-89
NEDD4L	protein_coding	ENSG00000049759	-0,723376485	2,22E-91	5,11E-89
TUFT1	protein_coding	ENSG00000143367	1,52635352	2,5E-91	5,7E-89
PPM1D	protein_coding	ENSG00000170836	1,01924627	5,87E-90	1,32E-87
TXNIP	protein_coding	ENSG00000265972	-1,076828514	2,78E-89	6,2E-87
TRAF1	protein_coding	ENSG00000056558	3,065848874	1,51E-88	3,34E-86
JAG1	protein_coding	ENSG00000101384	1,9145522	5,08E-88	1,11E-85
HIST1H3A	protein_coding	ENSG00000275714	-1,697538722	9,71E-87	2,1E-84
TM4SF1	protein_coding	ENSG00000169908	-1,109621614	3,08E-86	6,59E-84
CDC20	protein_coding	ENSG00000117399	-1,043248118	4,18E-86	8,83E-84
HIST1H4E	protein_coding	ENSG00000276966	-1,606835288	1,53E-85	3,2E-83
SLC41A2	protein_coding	ENSG00000136052	1,203826575	7,9E-85	1,64E-82
TBX3	protein_coding	ENSG00000135111	1,889216469	2,35E-84	4,82E-82
Z93241.1	lincRNA	ENSG00000270022	-3,702523551	2,47E-84	5,02E-82
HSPA5	protein_coding	ENSG00000044574	1,157689639	3,22E-84	6,47E-82
TEX14	protein_coding	ENSG00000121101	2,981315551	6,71E-84	1,34E-81
TIPARP	protein_coding	ENSG00000163659	0,762618088	1,02E-83	2E-81
PTGER4	protein_coding	ENSG00000171522	1,478989925	1,32E-83	2,58E-81
EFNB2	protein_coding	ENSG00000125266	1,720451287	2,01E-82	3,9E-80
RNF19B	protein_coding	ENSG00000116514	1,284947407	2,45E-82	4,7E-80
MBNL1-AS1	antisense	ENSG00000229619	2,564067277	3,76E-82	7,13E-80
NKX3-1	protein_coding	ENSG00000167034	1,544046289	4,14E-82	7,79E-80

CDC14A	protein_coding	ENSG00000079335	1,556927121	6,76E-82	1,26E-79
LNCOG	lincRNA	ENSG00000257219	2,119138938	3,44E-81	6,36E-79
HIST1H2BD	protein_coding	ENSG00000158373	-1,282573627	6,2E-81	1,14E-78
APOL6	protein_coding	ENSG00000221963	1,187256768	9,04E-81	1,64E-78
DYRK3	protein_coding	ENSG00000143479	1,010752376	1,73E-80	3,12E-78
OXTR	protein_coding	ENSG00000180914	-1,005651304	6,19E-80	1,1E-77
CPEB2	protein_coding	ENSG00000137449	1,658039707	6,98E-80	1,23E-77
KRCC1	protein_coding	ENSG00000172086	1,92570892	8,7E-80	1,52E-77
DNAH12	protein_coding	ENSG00000174844	4,901490733	2,72E-79	4,73E-77
KLF4	protein_coding	ENSG00000136826	1,793825304	9,59E-79	1,65E-76
MOK	protein_coding	ENSG00000080823	-0,613353105	1,74E-78	2,97E-76
TENT5A	protein_coding	ENSG00000112773	1,739200633	1,54E-77	2,62E-75
UBE2C	protein_coding	ENSG00000175063	-1,089869306	4,2E-77	7,07E-75
SPRY4	protein_coding	ENSG00000187678	1,252981648	4,95E-77	8,25E-75
DDX60	protein_coding	ENSG00000137628	1,377794715	6,32E-77	1,04E-74
ISG20	protein_coding	ENSG00000172183	2,076502331	3,26E-76	5,34E-74
FAM83D	protein_coding	ENSG00000101447	-0,829356089	1,12E-75	1,82E-73
ZEB2	protein_coding	ENSG00000169554	1,04947916	9,22E-75	1,49E-72
ATXN7L3B	protein_coding	ENSG00000253719	-0,737267874	1,11E-74	1,78E-72
RCSD1	protein_coding	ENSG00000198771	4,729195046	1,62E-74	2,57E-72
HIST1H4H	protein_coding	ENSG00000158406	-1,342673841	8,7E-74	1,37E-71
NFKBIA	protein_coding	ENSG00000100906	1,231000932	2,11E-73	3,3E-71
FNDC3B	protein_coding	ENSG00000075420	0,754214889	6,25E-73	9,71E-71
HIVEP1	protein_coding	ENSG00000095951	1,495193557	8,35E-73	1,29E-70
BACE1	protein_coding	ENSG00000186318	1,670911803	1,93E-72	2,96E-70
ZBTB10	protein_coding	ENSG00000205189	1,186281377	1,98E-72	3,01E-70
RNU5F-1	snRNA	ENSG00000199377	-1,712242515	2,73E-72	4,12E-70
ZSWIM6	protein_coding	ENSG00000130449	1,313200725	2,76E-72	4,13E-70
NFKBIE	protein_coding	ENSG00000146232	1,861954165	2,88E-72	4,27E-70
GADD45B	protein_coding	ENSG00000099860	1,680132264	3,51E-72	5,18E-70
HIST2H2BF	protein_coding	ENSG00000203814	-1,582008862	3,91E-72	5,72E-70
TNFRSF9	protein_coding	ENSG00000049249	5,44822266	1,61E-71	2,35E-69
HIST1H3B	protein_coding	ENSG00000274267	-1,481733961	2,75E-71	3,98E-69
LINC00702	lincRNA	ENSG00000233117	1,84017444	3,33E-71	4,74E-69
RF00003	snRNA	ENSG00000274428	-3,620741263	3,33E-71	4,74E-69
CASP10	protein_coding	ENSG00000003400	1,73043842	3,38E-71	4,78E-69
HIST1H4A	protein_coding	ENSG00000278637	-1,578987746	5,54E-71	7,79E-69
FOXD1	protein_coding	ENSG00000251493	-1,178530899	1,3E-70	1,82E-68
INHBA	protein_coding	ENSG00000122641	0,966163808	1,53E-70	2,12E-68
MAT2A	protein_coding	ENSG00000168906	-1,321852145	2,56E-70	3,52E-68

TDRD7	protein_coding	ENSG00000196116	1,529881348	3,94E-70	5,38E-68
HMGB2	protein_coding	ENSG00000164104	-1,100637486	7,23E-70	9,81E-68
TGFBI	protein_coding	ENSG00000120708	1,635201508	9,78E-70	1,32E-67
ZNF697	protein_coding	ENSG00000143067	1,974911104	1,61E-69	2,16E-67
IL6ST	protein_coding	ENSG00000134352	0,780123237	1,89E-69	2,52E-67
USP53	protein_coding	ENSG00000145390	1,235778308	3,16E-69	4,18E-67
CREBRF	protein_coding	ENSG00000164463	1,242413493	3,47E-69	4,56E-67
HIST1H4B	protein_coding	ENSG00000278705	-1,369694709	6,33E-69	8,27E-67
SGK1	protein_coding	ENSG00000118515	-0,697581572	1,12E-68	1,45E-66
EAF1	protein_coding	ENSG00000144597	0,969952289	2,03E-68	2,62E-66
RGL1	protein_coding	ENSG00000143344	1,742445834	2,91E-68	3,74E-66
FAM214A	protein_coding	ENSG00000047346	1,343855396	3,62E-68	4,61E-66
CDCA7	protein_coding	ENSG00000144354	-1,172418642	2,83E-67	3,59E-65
ISG15	protein_coding	ENSG00000187608	2,250568875	3,55E-67	4,46E-65
AC239868.1	transcribed_unprocesse	ENSG00000261716	2,644840191	3,88E-67	4,85E-65
CSF1	protein_coding	ENSG00000184371	2,407909186	7,14E-67	8,88E-65
SNRPA	protein_coding	ENSG00000077312	-1,012191746	8,85E-67	1,09E-64
SPRY2	protein_coding	ENSG00000136158	1,10181642	9,49E-67	1,17E-64
KCNQ3	protein_coding	ENSG00000184156	1,23046488	1,65E-66	2,01E-64
CDCA3	protein_coding	ENSG00000111665	-0,820724046	2,54E-66	3,08E-64
FLI1	protein_coding	ENSG00000151702	0,958507949	2,7E-66	3,25E-64
CRELD1	protein_coding	ENSG00000163703	1,492149873	7,4E-66	8,87E-64
UGCG	protein_coding	ENSG00000148154	-0,988663552	7,69E-66	9,17E-64
FRS2	protein_coding	ENSG00000166225	0,913060218	1,85E-65	2,19E-63
HIST1H2BO	protein_coding	ENSG00000274641	-1,39084365	2,33E-65	2,75E-63
ETV3	protein_coding	ENSG00000117036	1,195997322	4,42E-65	5,18E-63
PTGS2	protein_coding	ENSG00000073756	3,106746352	7,62E-65	8,87E-63
SFRP1	protein_coding	ENSG00000104332	-0,680140459	8,01E-65	9,28E-63
MYPN	protein_coding	ENSG00000138347	1,454294449	8,52E-65	9,81E-63
CD274	protein_coding	ENSG00000120217	2,915928672	1,47E-64	1,68E-62
ID1	protein_coding	ENSG00000125968	-1,239309629	1,9E-64	2,16E-62
BAMBI	protein_coding	ENSG00000095739	1,728533361	4,58E-64	5,19E-62
AL135905.1	antisense	ENSG00000266680	2,430847585	5,07E-64	5,72E-62
HIST2H3D	protein_coding	ENSG00000183598	-1,627452957	6,28E-64	7,04E-62
IL6	protein_coding	ENSG00000136244	1,312022183	6,96E-64	7,76E-62
BCL10	protein_coding	ENSG00000142867	0,770229204	1,59E-63	1,76E-61
CCRL2	protein_coding	ENSG00000121797	1,791022882	2,2E-63	2,42E-61
ENO3	protein_coding	ENSG00000108515	1,896120289	3,17E-63	3,48E-61
CD83	protein_coding	ENSG00000112149	1,502481964	3,99E-63	4,35E-61
MX1	protein_coding	ENSG00000157601	3,519998556	9,01E-63	9,78E-61

HIST2H2BE	protein_coding	ENSG00000184678	-1,334150844	2,36E-62	2,54E-60
RNU4ATAC	snRNA	ENSG00000264229	-2,891318717	2,46E-62	2,65E-60
PGF	protein_coding	ENSG00000119630	2,316010925	2,5E-62	2,67E-60
ABCC9	protein_coding	ENSG00000069431	4,752068735	3,93E-62	4,17E-60
TERF2IP	protein_coding	ENSG00000166848	0,608395748	6,52E-62	6,89E-60
MYEOV	protein_coding	ENSG00000172927	-0,895782138	3,87E-61	4,07E-59
RAB5A	protein_coding	ENSG00000144566	0,732166676	6,21E-61	6,5E-59
OPN3	protein_coding	ENSG00000054277	1,25347269	6,86E-61	7,15E-59
AMOTL2	protein_coding	ENSG00000114019	-0,855741965	1,33E-60	1,38E-58
CEP120	protein_coding	ENSG00000168944	0,850060081	2,38E-60	2,45E-58
RF00003	snRNA	ENSG00000206828	-3,090686743	2,65E-60	2,72E-58
SUV39H1	protein_coding	ENSG00000101945	-0,932038038	3,68E-60	3,76E-58
MYC	protein_coding	ENSG00000136997	-0,671925353	4,25E-60	4,32E-58
TIGAR	protein_coding	ENSG00000078237	0,802719356	9,63E-60	9,73E-58
CUBN	protein_coding	ENSG00000107611	2,753554752	1,28E-59	1,29E-57
SOD2	protein_coding	ENSG00000112096	0,976885718	1,33E-59	1,33E-57
RCCD1	protein_coding	ENSG00000166965	-0,972801903	2,05E-59	2,04E-57
TRIM55	protein_coding	ENSG00000147573	3,245360727	3,21E-59	3,18E-57
CCNF	protein_coding	ENSG00000162063	-1,155439748	4,07E-59	4,01E-57
EREG	protein_coding	ENSG00000124882	1,447368431	4,29E-59	4,21E-57
ENC1	protein_coding	ENSG00000171617	0,849772404	5,35E-59	5,22E-57
SEL1L	protein_coding	ENSG00000071537	0,68541029	5,85E-59	5,69E-57
TRIM26	protein_coding	ENSG00000234127	1,150377383	1,49E-58	1,44E-56
EFCAB5	protein_coding	ENSG00000176927	3,088567311	3,63E-58	3,49E-56
H1F0	protein_coding	ENSG00000189060	-0,679126524	3,76E-58	3,6E-56
WDR26	protein_coding	ENSG00000162923	0,786778029	4,72E-58	4,5E-56
LINC00944	lincRNA	ENSG00000256128	2,527900152	4,79E-58	4,55E-56
F3	protein_coding	ENSG00000117525	-0,67054241	5,77E-58	5,46E-56
ARID5B	protein_coding	ENSG00000150347	1,455006262	6,53E-58	6,14E-56
DHRS3	protein_coding	ENSG00000162496	1,520828996	7,66E-58	7,17E-56
HIST1H2BF	protein_coding	ENSG00000277224	-1,370525395	7,78E-58	7,25E-56
WDR47	protein_coding	ENSG00000085433	0,900829683	7,98E-58	7,41E-56
DDX60L	protein_coding	ENSG00000181381	1,259465814	9,33E-58	8,62E-56
DUSP16	protein_coding	ENSG00000111266	1,348114789	1,39E-57	1,28E-55
IFRD1	protein_coding	ENSG00000006652	1,119027362	1,95E-57	1,78E-55
GCNT2	protein_coding	ENSG00000111846	0,697332884	1,96E-57	1,78E-55
PSRC1	protein_coding	ENSG00000134222	-0,763181097	2,39E-57	2,17E-55
HIST1H2BK	protein_coding	ENSG00000197903	-1,186584132	3,71E-57	3,35E-55
CFAP57	protein_coding	ENSG00000243710	2,302896877	6,39E-57	5,75E-55
ARL5B	protein_coding	ENSG00000165997	1,089837842	1,24E-56	1,11E-54

UBL4A	protein_coding	ENSG00000102178	-0,940069064	1,4E-56	1,25E-54
FAM43A	protein_coding	ENSG00000185112	1,682201434	1,5E-56	1,33E-54
ITPRIPL1	protein_coding	ENSG00000198885	-1,162633149	1,84E-56	1,62E-54
AC245014.3	lincRNA	ENSG00000276216	-3,22648366	1,85E-56	1,63E-54
LINC00511	processed_transcript	ENSG00000227036	2,213829648	2,04E-56	1,79E-54
TNFSF9	protein_coding	ENSG00000125657	1,738049608	2,38E-56	2,08E-54
CEP85	protein_coding	ENSG00000130695	-0,712669205	3,12E-56	2,71E-54
CALD1	protein_coding	ENSG00000122786	0,760138491	3,34E-56	2,88E-54
ARMCX3	protein_coding	ENSG00000102401	0,687299973	3,34E-56	2,88E-54
AURKA	protein_coding	ENSG00000087586	-1,003123078	3,9E-56	3,35E-54
NEURL3	protein_coding	ENSG00000163121	4,746315872	4,68E-56	4E-54
PLCXD2	protein_coding	ENSG00000240891	0,82089713	4,93E-56	4,2E-54
RNU1-85P	snRNA	ENSG00000200997	-3,71560635	9,43E-56	7,99E-54
APOLD1	protein_coding	ENSG00000178878	1,408683293	1,52E-55	1,28E-53
LAMA3	protein_coding	ENSG00000053747	1,37173585	1,85E-55	1,56E-53
SESN2	protein_coding	ENSG00000130766	1,440975195	2,22E-55	1,86E-53
SLC30A7	protein_coding	ENSG00000162695	0,596702785	3,18E-55	2,65E-53
ACSL3	protein_coding	ENSG00000123983	0,719189392	3,27E-55	2,72E-53
TMEM217	protein_coding	ENSG00000172738	3,166110493	5,64E-55	4,66E-53
PARP14	protein_coding	ENSG00000173193	0,943652055	5,76E-55	4,75E-53
SDF2L1	protein_coding	ENSG00000128228	1,194477994	5,84E-55	4,79E-53
KDM6A	protein_coding	ENSG00000147050	0,782619328	6,17E-55	5,04E-53
MLLT11	protein_coding	ENSG00000213190	1,108978281	7,72E-55	6,28E-53
ZNF804A	protein_coding	ENSG00000170396	1,526275821	1,08E-54	8,74E-53
IL1A	protein_coding	ENSG00000115008	0,90751866	1,23E-54	9,91E-53
RND1	protein_coding	ENSG00000172602	3,689693451	1,32E-54	1,06E-52
HPS5	protein_coding	ENSG00000110756	0,603681272	1,77E-54	1,42E-52
NFKBIZ	protein_coding	ENSG00000144802	1,506532783	2,3E-54	1,83E-52
KNSTRN	protein_coding	ENSG00000128944	-1,005314776	2,38E-54	1,89E-52
PLK1	protein_coding	ENSG00000166851	-0,982703243	4,87E-54	3,86E-52
KIFC1	protein_coding	ENSG00000237649	-0,964222823	9,34E-54	7,37E-52
THAP2	protein_coding	ENSG00000173451	1,554206534	1,08E-53	8,49E-52
FAM167A	protein_coding	ENSG00000154319	-0,922343015	1,12E-53	8,78E-52
TRIB1	protein_coding	ENSG00000173334	1,009754447	1,65E-53	1,29E-51
SKP2	protein_coding	ENSG00000145604	-0,855998844	2,27E-53	1,76E-51
MICA	protein_coding	ENSG00000204520	1,262367459	3,29E-53	2,54E-51
OVGP1	protein_coding	ENSG00000085465	3,411833643	3,72E-53	2,86E-51
WARS	protein_coding	ENSG00000140105	0,888964699	4,68E-53	3,6E-51
SLC44A5	protein_coding	ENSG00000137968	1,390811401	4,99E-53	3,82E-51
LATS2	protein_coding	ENSG00000150457	-0,821708333	6,1E-53	4,65E-51

CRELD2	protein_coding	ENSG00000184164	0,892479081	9,17E-53	6,97E-51
NLRP3	protein_coding	ENSG00000162711	1,095168423	9,43E-53	7,13E-51
LAMA2	protein_coding	ENSG00000196569	2,135143043	1,02E-52	7,66E-51
RF00003	snRNA	ENSG00000273768	-2,631456821	1,19E-52	8,93E-51
AL354740.1	processed_transcript	ENSG00000225339	2,492987788	1,98E-52	1,48E-50
FKBP15	protein_coding	ENSG00000119321	0,590605487	2,12E-52	1,58E-50
C8orf58	protein_coding	ENSG00000241852	-0,872056311	2,27E-52	1,68E-50
CEACAM1	protein_coding	ENSG00000079385	3,283209902	2,95E-52	2,18E-50
STARD4	protein_coding	ENSG00000164211	0,802591135	3,85E-52	2,84E-50
ZFP62	protein_coding	ENSG00000196670	-1,027915703	5,2E-52	3,83E-50
WDR63	protein_coding	ENSG00000162643	1,887964308	5,4E-52	3,96E-50
FAM84B	protein_coding	ENSG00000168672	-0,815128567	5,92E-52	4,32E-50
KLF2	protein_coding	ENSG00000127528	-1,055842433	7,98E-52	5,8E-50
EPHB2	protein_coding	ENSG00000133216	1,03858939	8,27E-52	6E-50
DCLRE1B	protein_coding	ENSG00000118655	-0,83288322	1,01E-51	7,31E-50
TMEM45A	protein_coding	ENSG00000181458	1,017213098	1,4E-51	1,01E-49
CDCA8	protein_coding	ENSG00000134690	-0,886786162	1,48E-51	1,06E-49
SEMA3C	protein_coding	ENSG00000075223	0,628927074	1,87E-51	1,33E-49
ABTB2	protein_coding	ENSG00000166016	1,816723959	2,74E-51	1,94E-49
CENPA	protein_coding	ENSG00000115163	-1,039517818	2,74E-51	1,94E-49
KIAA1324	protein_coding	ENSG00000116299	0,959732806	2,95E-51	2,09E-49
TRAPPC6B	protein_coding	ENSG00000182400	0,839141711	3,3E-51	2,33E-49
RIOK3	protein_coding	ENSG00000101782	0,71147924	3,36E-51	2,36E-49
PLK3	protein_coding	ENSG00000173846	1,522673204	5,21E-51	3,65E-49
TSC22D2	protein_coding	ENSG00000196428	0,705729179	7,68E-51	5,36E-49
CLCN4	protein_coding	ENSG00000073464	1,028857991	9,17E-51	6,37E-49
KRTAP2-3	protein_coding	ENSG00000212724	-1,543675118	9,34E-51	6,47E-49
KIF20A	protein_coding	ENSG00000112984	-0,92617107	1,06E-50	7,29E-49
AC016831.1	lincRNA	ENSG00000226380	-1,287914312	1,24E-50	8,53E-49
HIST1H2AE	protein_coding	ENSG00000277075	-1,298022938	1,58E-50	1,08E-48
KRT17	protein_coding	ENSG00000128422	5,044785407	1,74E-50	1,19E-48
GEMIN4	protein_coding	ENSG00000179409	-0,982665595	1,88E-50	1,28E-48
TOB1	protein_coding	ENSG00000141232	1,035203408	1,92E-50	1,31E-48
HIST1H2BG	protein_coding	ENSG00000273802	-1,07761798	2,4E-50	1,63E-48
LINC01588	lincRNA	ENSG00000214900	1,594541186	6,02E-50	4,06E-48
SLU7	protein_coding	ENSG00000164609	0,677403241	7,52E-50	5,05E-48
HIST1H2AH	protein_coding	ENSG00000274997	-1,328736406	7,95E-50	5,33E-48
ZNF425	protein_coding	ENSG00000204947	3,309069604	8,33E-50	5,57E-48
SLC2A3	protein_coding	ENSG00000059804	0,948984123	9,65E-50	6,43E-48
POLR2A	protein_coding	ENSG00000181222	1,251887776	1,04E-49	6,89E-48

VEGFC	protein_coding	ENSG00000150630	0,937998221	1,07E-49	7,07E-48
KIF18B	protein_coding	ENSG00000186185	-1,099895526	1,11E-49	7,32E-48
SYNC	protein_coding	ENSG00000162520	0,761316227	1,39E-49	9,13E-48
ABLIM1	protein_coding	ENSG00000099204	1,510245237	1,59E-49	1,04E-47
ERCC6	protein_coding	ENSG00000225830	-0,735368318	1,61E-49	1,05E-47
ERCC6	protein_coding	ENSG00000225830	-0,735368318	1,61E-49	1,05E-47
MR1	protein_coding	ENSG00000153029	1,318139213	2,54E-49	1,65E-47
SMAD7	protein_coding	ENSG00000101665	1,13351513	6,72E-49	4,36E-47
P3H4	protein_coding	ENSG00000141696	-0,858948131	7,54E-49	4,88E-47
NUDCD3	protein_coding	ENSG00000015676	-0,535689806	1,66E-48	1,07E-46
TMEM177	protein_coding	ENSG00000144120	-1,032802153	1,73E-48	1,11E-46
PRKAB2	protein_coding	ENSG00000131791	0,881683394	5,25E-48	3,36E-46
RTL9	protein_coding	ENSG00000243978	3,981339566	8,2E-48	5,24E-46
PPP1R15B	protein_coding	ENSG00000158615	0,607805939	8,41E-48	5,36E-46
AC103740.1	antisense	ENSG00000259370	3,401544647	9,45E-48	6E-46
ATF6	protein_coding	ENSG00000118217	0,583245797	1,21E-47	7,65E-46
CCNB1	protein_coding	ENSG00000134057	-1,007364072	1,34E-47	8,44E-46
TRIM2	protein_coding	ENSG00000109654	0,922199534	1,4E-47	8,79E-46
KIF22	protein_coding	ENSG00000079616	-0,73450549	1,42E-47	8,92E-46
ITGA4	protein_coding	ENSG00000115232	-0,794314617	2,02E-47	1,26E-45
MAGEF1	protein_coding	ENSG00000177383	-0,770077374	2,36E-47	1,47E-45
ADTRP	protein_coding	ENSG00000111863	1,035943518	2,67E-47	1,66E-45
SCN3A	protein_coding	ENSG00000153253	5,533578492	3,03E-47	1,88E-45
HIST1H2BE	protein_coding	ENSG00000274290	2,819716471	3,15E-47	1,95E-45
IFI6	protein_coding	ENSG00000126709	1,61672595	3,19E-47	1,97E-45
SF3B3	protein_coding	ENSG00000189091	-0,605072872	4,37E-47	2,69E-45
MAP1LC3B	protein_coding	ENSG00000140941	1,060278315	4,68E-47	2,86E-45
RF00003	snRNA	ENSG00000273727	-3,850100441	6,86E-47	4,19E-45
CSGALNACT2	protein_coding	ENSG00000169826	0,7658956	7,15E-47	4,35E-45
TUBB6	protein_coding	ENSG00000176014	-0,678538158	8,01E-47	4,86E-45
HHAT	protein_coding	ENSG00000054392	1,230079796	9,81E-47	5,93E-45
SLC9A1	protein_coding	ENSG00000090020	1,491304269	1,37E-46	8,24E-45
PXDC1	protein_coding	ENSG00000168994	0,830152669	2,4E-46	1,44E-44
HIST1H2AG	protein_coding	ENSG00000196787	-1,428128055	2,47E-46	1,48E-44
KPNA2	protein_coding	ENSG00000182481	-0,847722051	4,37E-46	2,61E-44
C10orf55	protein_coding	ENSG00000222047	2,007214982	5,95E-46	3,55E-44
EFNA1	protein_coding	ENSG00000169242	2,642226451	8,62E-46	5,12E-44
ITPKC	protein_coding	ENSG00000086544	1,416720666	1,02E-45	6,06E-44
PRSS23	protein_coding	ENSG00000150687	0,577160259	1,13E-45	6,67E-44
PTPRK	protein_coding	ENSG00000152894	0,698262298	1,24E-45	7,31E-44

PHLDA2	protein_coding	ENSG00000181649	1,677329793	1,36E-45	7,97E-44
MAFF	protein_coding	ENSG00000185022	1,309925856	2,2E-45	1,29E-43
CANX	protein_coding	ENSG00000127022	0,506905641	2,23E-45	1,31E-43
HIST1H2BJ	protein_coding	ENSG00000124635	-1,123823928	3,88E-45	2,26E-43
GBP3	protein_coding	ENSG00000117226	1,913195819	6,54E-45	3,8E-43
TAPBPL	protein_coding	ENSG00000139192	2,155151199	7,25E-45	4,2E-43
GPR87	protein_coding	ENSG00000138271	2,630036819	7,4E-45	4,27E-43
TNIP3	protein_coding	ENSG00000050730	1,938501824	8,4E-45	4,84E-43
ZNF670	protein_coding	ENSG00000277462	1,273730632	1,02E-44	5,88E-43
LINC00346	lincRNA	ENSG00000255874	1,585309368	1,37E-44	7,84E-43
ID2	protein_coding	ENSG00000115738	1,432618258	1,49E-44	8,49E-43
DNAJC6	protein_coding	ENSG00000116675	0,621428498	1,73E-44	9,86E-43
ING1	protein_coding	ENSG00000153487	1,015804729	2,73E-44	1,55E-42
ATP6V0A1	protein_coding	ENSG00000033627	0,711583878	2,92E-44	1,65E-42
MEF2C	protein_coding	ENSG00000081189	1,395889338	3E-44	1,69E-42
MAML2	protein_coding	ENSG00000184384	1,035785957	3,41E-44	1,92E-42
LPXN	protein_coding	ENSG00000110031	1,871783479	4,02E-44	2,26E-42
CXCL2	protein_coding	ENSG00000081041	1,433694538	4,25E-44	2,38E-42
SLC16A12	protein_coding	ENSG00000152779	1,63750616	5,43E-44	3,04E-42
HJURP	protein_coding	ENSG00000123485	-0,689376787	6,28E-44	3,5E-42
EPAS1	protein_coding	ENSG00000116016	1,352803295	7E-44	3,89E-42
RBM24	protein_coding	ENSG00000112183	0,724367456	8,27E-44	4,58E-42
OSBP	protein_coding	ENSG00000110048	0,565558309	8,71E-44	4,81E-42
SRSF2	protein_coding	ENSG00000161547	-0,853155766	9,79E-44	5,4E-42
SMURF2	protein_coding	ENSG00000108854	-0,741395873	9,87E-44	5,42E-42
TRPV2	protein_coding	ENSG00000187688	1,22493076	1,17E-43	6,4E-42
ETV7	protein_coding	ENSG00000010030	4,229609326	1,84E-43	1E-41
HIST1H1C	protein_coding	ENSG00000187837	-1,112226833	2,43E-43	1,33E-41
PTX3	protein_coding	ENSG00000163661	1,000203494	4E-43	2,18E-41
LYSMD3	protein_coding	ENSG00000176018	0,803578531	4,2E-43	2,28E-41
ANGPTL4	protein_coding	ENSG00000167772	1,353287349	4,55E-43	2,46E-41
PELI1	protein_coding	ENSG00000197329	1,29020277	5,26E-43	2,84E-41
HIST1H3C	protein_coding	ENSG00000278272	-1,271144	6,51E-43	3,5E-41
CXCL3	protein_coding	ENSG00000163734	1,722741749	8,15E-43	4,38E-41
TDP1	protein_coding	ENSG00000042088	-0,83067572	8,62E-43	4,62E-41
SPRED3	protein_coding	ENSG00000188766	1,403563566	9,44E-43	5,04E-41
NATD1	protein_coding	ENSG00000274180	1,824899043	1,28E-42	6,84E-41
COL5A1	protein_coding	ENSG00000130635	1,305510131	1,44E-42	7,66E-41
PADI3	protein_coding	ENSG00000142619	3,051003303	1,45E-42	7,69E-41
DNAJB2	protein_coding	ENSG00000135924	0,984683349	2,82E-42	1,49E-40

ZNF140	protein_coding	ENSG00000196387	0,730205002	2,84E-42	1,5E-40
NOP56	protein_coding	ENSG00000101361	-0,691318559	3,41E-42	1,79E-40
JUNB	protein_coding	ENSG00000171223	1,391668337	7,29E-42	3,83E-40
ATP6V0A2	protein_coding	ENSG00000185344	0,839846424	8,12E-42	4,25E-40
KRT7	protein_coding	ENSG00000135480	1,575206988	8,74E-42	4,56E-40
MICB	protein_coding	ENSG00000204516	0,589384223	9,48E-42	4,94E-40
GINS1	protein_coding	ENSG00000101003	-1,010240687	9,57E-42	4,97E-40
ATP7A	protein_coding	ENSG00000165240	0,867547918	9,85E-42	5,1E-40
AC107959.2	antisense	ENSG00000246130	5,10018342	1,14E-41	5,88E-40
RRM2	protein_coding	ENSG00000171848	-1,065790303	1,18E-41	6,1E-40
RRM2	protein_coding	ENSG00000171848	-1,065790303	1,18E-41	6,1E-40
BMPER	protein_coding	ENSG00000164619	-0,680487893	1,65E-41	8,46E-40
BTN2A3P	transcribed_unprocesse	ENSG00000124549	1,407851425	1,97E-41	1,01E-39
WDR78	protein_coding	ENSG00000152763	2,25712957	2,18E-41	1,12E-39
CYTH4	protein_coding	ENSG00000100055	3,567796957	2,47E-41	1,26E-39
MT2A	protein_coding	ENSG00000125148	-0,811023004	2,61E-41	1,33E-39
GORAB	protein_coding	ENSG00000120370	1,109966482	3,22E-41	1,64E-39
ARHGEF39	protein_coding	ENSG00000137135	-1,048411627	3,25E-41	1,64E-39
KIF2C	protein_coding	ENSG00000142945	-0,599107703	3,34E-41	1,69E-39
MINDY3	protein_coding	ENSG00000148481	0,775714412	3,48E-41	1,75E-39
EDEM1	protein_coding	ENSG00000134109	0,574308243	5,06E-41	2,54E-39
LY6K	protein_coding	ENSG00000160886	-0,74400692	5,15E-41	2,58E-39
OSTM1	protein_coding	ENSG00000081087	0,81337969	5,39E-41	2,7E-39
SUSD1	protein_coding	ENSG00000106868	1,836382499	5,46E-41	2,73E-39
HNRNPF	protein_coding	ENSG00000169813	-0,686958166	9E-41	4,48E-39
TAP1	protein_coding	ENSG00000168394	1,067942028	1,08E-40	5,36E-39
PANX1	protein_coding	ENSG00000110218	0,562449302	1,16E-40	5,75E-39
DUSP6	protein_coding	ENSG00000139318	0,486374586	1,23E-40	6,05E-39
SAT1	protein_coding	ENSG00000130066	0,743063116	1,56E-40	7,68E-39
ZNF597	protein_coding	ENSG00000167981	1,166854484	1,64E-40	8,06E-39
FAM72B	protein_coding	ENSG00000188610	-0,867826763	1,71E-40	8,37E-39
CCNA2	protein_coding	ENSG00000145386	-1,1882988	1,75E-40	8,57E-39
APLF	protein_coding	ENSG00000169621	1,165970425	1,8E-40	8,81E-39
TGFA	protein_coding	ENSG00000163235	1,332070684	1,89E-40	9,18E-39
CENPO	protein_coding	ENSG00000138092	-0,719699191	1,89E-40	9,18E-39
MANEAL	protein_coding	ENSG00000185090	-0,804379419	2,13E-40	1,03E-38
HIST2H2AB	protein_coding	ENSG00000184270	-1,050550063	2,2E-40	1,06E-38
USP18	protein_coding	ENSG00000184979	1,049953781	3,13E-40	1,51E-38
SEC63	protein_coding	ENSG00000025796	0,846107137	3,27E-40	1,57E-38
SAV1	protein_coding	ENSG00000151748	0,631702149	3,65E-40	1,75E-38

FGFR3	protein_coding	ENSG00000068078	2,124336124	3,83E-40	1,84E-38
KRT81	protein_coding	ENSG00000205426	1,762843025	4,59E-40	2,19E-38
AC103591.3	antisense	ENSG00000273338	-3,111441242	4,76E-40	2,27E-38
GCH1	protein_coding	ENSG00000131979	1,444615052	5,18E-40	2,47E-38
YWHAG	protein_coding	ENSG00000170027	0,418539072	5,46E-40	2,59E-38
APOL2	protein_coding	ENSG00000128335	0,916581984	6,06E-40	2,87E-38
PMP22	protein_coding	ENSG00000109099	-1,074313404	8,29E-40	3,92E-38
ACTA2	protein_coding	ENSG00000107796	1,191419342	9,38E-40	4,42E-38
WWTR1	protein_coding	ENSG00000018408	0,543083599	1,45E-39	6,81E-38
DSC3	protein_coding	ENSG00000134762	0,598383087	1,51E-39	7,09E-38
KLF11	protein_coding	ENSG00000172059	-0,794092818	1,7E-39	7,95E-38
HYAL2	protein_coding	ENSG00000068001	-0,779302505	2,02E-39	9,42E-38
TUBA1C	protein_coding	ENSG00000167553	-0,708092875	2,19E-39	1,02E-37
TAB2	protein_coding	ENSG00000055208	0,612972883	2,5E-39	1,16E-37
HSP90B1	protein_coding	ENSG00000166598	0,566330718	2,64E-39	1,22E-37
NUAK1	protein_coding	ENSG00000074590	0,730714443	2,75E-39	1,27E-37
HIF1A-AS1	lincRNA	ENSG00000258777	2,723168682	2,84E-39	1,31E-37
HIVEP2	protein_coding	ENSG00000010818	0,953216844	2,89E-39	1,33E-37
BAZ2B	protein_coding	ENSG00000123636	0,982573054	3,08E-39	1,42E-37
SLC7A11	protein_coding	ENSG00000151012	0,808742662	3,2E-39	1,47E-37
PFKFB3	protein_coding	ENSG00000170525	0,907956567	4,2E-39	1,92E-37
NUP62	protein_coding	ENSG00000213024	-0,693083606	4,29E-39	1,96E-37
TOGARAM1	protein_coding	ENSG00000198718	0,795361149	5,41E-39	2,47E-37
LMNB1	protein_coding	ENSG00000113368	-1,084069209	5,68E-39	2,59E-37
MIR137HG	lincRNA	ENSG00000225206	-0,739074656	6,37E-39	2,89E-37
KDSR	protein_coding	ENSG00000119537	0,529801617	7,76E-39	3,51E-37
TMEM184C	protein_coding	ENSG00000164168	0,604689643	9,43E-39	4,26E-37
NCOA3	protein_coding	ENSG00000124151	0,822781237	1,07E-38	4,81E-37
MAP3K7CL	protein_coding	ENSG00000156265	0,90665077	1,08E-38	4,87E-37
ZC3HAV1L	protein_coding	ENSG00000146858	-1,134234592	1,23E-38	5,53E-37
PAG1	protein_coding	ENSG00000076641	0,953061985	1,37E-38	6,11E-37
ATP2B1	protein_coding	ENSG00000070961	0,719384339	1,36E-38	6,11E-37
TUBB	protein_coding	ENSG00000196230	-0,572132567	1,49E-38	6,62E-37
PIK3R3	protein_coding	ENSG00000117461	1,091124875	1,85E-38	8,21E-37
AC107959.3	antisense	ENSG00000253616	3,058847116	1,85E-38	8,22E-37
WDR82	protein_coding	ENSG00000164091	-0,665436665	2,32E-38	1,03E-36
ABCC2	protein_coding	ENSG00000023839	0,862132131	2,67E-38	1,18E-36
AHR	protein_coding	ENSG00000106546	0,704890708	3,08E-38	1,36E-36
BTN2A2	protein_coding	ENSG00000124508	1,128706359	3,17E-38	1,39E-36
FAM174B	protein_coding	ENSG00000185442	-0,793089114	3,61E-38	1,59E-36

DIPK2A	protein_coding	ENSG00000181744	0,730466231	4,41E-38	1,93E-36
CHST11	protein_coding	ENSG00000171310	0,834908392	4,54E-38	1,99E-36
SMIM14	protein_coding	ENSG00000163683	0,863614287	4,62E-38	2,02E-36
RELB	protein_coding	ENSG00000104856	1,278974924	4,67E-38	2,03E-36
IGFBP3	protein_coding	ENSG00000146674	-0,543817493	5,06E-38	2,2E-36
HIST1H2AB	protein_coding	ENSG00000278463	-1,216570334	5,06E-38	2,2E-36
NCAPD2	protein_coding	ENSG00000010292	-0,649103251	5,22E-38	2,26E-36
HSPH1	protein_coding	ENSG00000120694	0,450849123	6,18E-38	2,67E-36
KDM6B	protein_coding	ENSG00000132510	1,850467967	7,35E-38	3,17E-36
FERMT2	protein_coding	ENSG00000073712	0,619726462	8,35E-38	3,59E-36
TAF13	protein_coding	ENSG00000197780	0,701993076	1E-37	4,31E-36
SGTB	protein_coding	ENSG00000197860	0,669531573	1,17E-37	5E-36
ANKRA2	protein_coding	ENSG00000164331	0,898774419	1,26E-37	5,39E-36
CHTOP	protein_coding	ENSG00000160679	-0,563255369	1,34E-37	5,72E-36
PYGO2	protein_coding	ENSG00000163348	-0,96141516	1,4E-37	5,98E-36
ZNF773	protein_coding	ENSG00000152439	1,144811671	1,51E-37	6,4E-36
TOP2A	protein_coding	ENSG00000131747	-0,975600975	1,53E-37	6,49E-36
ABCA1	protein_coding	ENSG00000165029	0,996477656	1,68E-37	7,08E-36
CDAN1	protein_coding	ENSG00000140326	-0,930229779	1,69E-37	7,13E-36
KLHL9	protein_coding	ENSG00000198642	-0,673787651	1,86E-37	7,83E-36
HIST1H2BC	protein_coding	ENSG00000180596	-1,005815509	2,01E-37	8,43E-36
TUBA4A	protein_coding	ENSG00000127824	5,180400788	2,02E-37	8,48E-36
MYH15	protein_coding	ENSG00000144821	1,158399131	2,33E-37	9,75E-36
NR5A2	protein_coding	ENSG00000116833	2,990893716	2,56E-37	1,07E-35
CDC25B	protein_coding	ENSG00000101224	-0,833050313	3,13E-37	1,31E-35
EXOSC2	protein_coding	ENSG00000130713	-0,656117651	3,21E-37	1,33E-35
HIST3H2BB	protein_coding	ENSG00000196890	-0,934100974	3,21E-37	1,33E-35
EDN2	protein_coding	ENSG00000127129	3,534836482	3,5E-37	1,45E-35
CLK4	protein_coding	ENSG00000113240	1,322510171	3,56E-37	1,47E-35
ZNF222	protein_coding	ENSG00000159885	1,240884939	3,69E-37	1,52E-35
NFKB1	protein_coding	ENSG00000109320	0,681297639	3,87E-37	1,59E-35
CRISPLD2	protein_coding	ENSG00000103196	1,34010772	5,23E-37	2,15E-35
OPTN	protein_coding	ENSG00000123240	0,576971982	5,89E-37	2,41E-35
COL6A3	protein_coding	ENSG00000163359	2,745535605	6,56E-37	2,69E-35
CDCA7L	protein_coding	ENSG00000164649	-0,702704519	6,93E-37	2,83E-35
KDM5B	protein_coding	ENSG00000117139	0,621040459	6,96E-37	2,84E-35
GCNT1	protein_coding	ENSG00000187210	-0,930437319	8,65E-37	3,52E-35
ERBB3	protein_coding	ENSG00000065361	-1,001527641	9,9E-37	4,02E-35
AXL	protein_coding	ENSG00000167601	-0,624411891	1,04E-36	4,23E-35
ZMYND19	protein_coding	ENSG00000165724	-0,833667182	1,05E-36	4,25E-35

THBS1	protein_coding	ENSG00000137801	-0,890372894	1,09E-36	4,42E-35
P4HA3	protein_coding	ENSG00000149380	2,861274024	1,14E-36	4,61E-35
RNU5D-1	snRNA	ENSG00000200169	-2,00837085	1,2E-36	4,84E-35
PIK3R1	protein_coding	ENSG00000145675	0,81688672	1,25E-36	5,02E-35
AC243964.3	TEC	ENSG00000279095	1,208908905	1,54E-36	6,19E-35
BRD2	protein_coding	ENSG00000204256	0,8494753	1,85E-36	7,41E-35
KNOP1	protein_coding	ENSG00000103550	-0,728829164	2,03E-36	8,12E-35
ZNF79	protein_coding	ENSG00000196152	1,148940422	2,13E-36	8,48E-35
ARF6	protein_coding	ENSG00000165527	-0,59529038	2,36E-36	9,39E-35
SMOX	protein_coding	ENSG00000088826	1,259741151	2,43E-36	9,65E-35
RCC1	protein_coding	ENSG00000180198	-0,523155816	2,57E-36	1,02E-34
MKNK2	protein_coding	ENSG00000099875	1,064404769	3,01E-36	1,19E-34
PI4K2A	protein_coding	ENSG00000155252	0,912608439	4,95E-36	1,95E-34
PBLD	protein_coding	ENSG00000108187	1,700090158	4,98E-36	1,96E-34
HYLS1	protein_coding	ENSG00000198331	-0,850859803	5,09E-36	2E-34
ZNF441	protein_coding	ENSG00000197044	1,396800077	5,35E-36	2,1E-34
UNC13A	protein_coding	ENSG00000130477	2,88471945	5,51E-36	2,16E-34
GPR158	protein_coding	ENSG00000151025	1,557167079	5,75E-36	2,25E-34
PLK2	protein_coding	ENSG00000145632	-0,601095812	6,16E-36	2,4E-34
POMGNT2	protein_coding	ENSG00000144647	-0,678232331	6,44E-36	2,51E-34
HASPIN	protein_coding	ENSG00000177602	-0,680346946	6,54E-36	2,54E-34
KIF15	protein_coding	ENSG00000163808	-0,933043302	8,59E-36	3,33E-34
JRK	protein_coding	ENSG00000234616	-0,906038667	1,05E-35	4,08E-34
HAS3	protein_coding	ENSG00000103044	-1,116899945	1,19E-35	4,61E-34
INPP5D	protein_coding	ENSG00000168918	3,099134622	1,37E-35	5,27E-34
ZBTB20	protein_coding	ENSG00000181722	1,109715666	1,6E-35	6,16E-34
RRAD	protein_coding	ENSG00000166592	1,686338763	1,67E-35	6,4E-34
KRT18	protein_coding	ENSG00000111057	-0,587616654	1,78E-35	6,83E-34
MCM3	protein_coding	ENSG00000112118	-0,82625944	2,04E-35	7,8E-34
TADA2A	protein_coding	ENSG00000276234	-0,661366888	2,15E-35	8,22E-34
DCAF16	protein_coding	ENSG00000163257	-0,843538992	2,18E-35	8,31E-34
GTSE1	protein_coding	ENSG00000075218	-0,78986508	2,48E-35	9,45E-34
TMEM203	protein_coding	ENSG00000187713	-0,676549498	2,58E-35	9,78E-34
HDX	protein_coding	ENSG00000165259	1,007252847	2,6E-35	9,85E-34
C21orf91	protein_coding	ENSG00000154642	0,946843517	2,75E-35	1,04E-33
MLPH	protein_coding	ENSG00000115648	-0,536823444	3,08E-35	1,16E-33
WDR76	protein_coding	ENSG00000092470	-0,762119444	3,33E-35	1,25E-33
TRIM7	protein_coding	ENSG00000146054	-0,809940543	3,72E-35	1,4E-33
NOP16	protein_coding	ENSG00000048162	-0,84938723	3,72E-35	1,4E-33
PARP8	protein_coding	ENSG00000151883	0,777291508	4,17E-35	1,56E-33

HCG27	lincRNA	ENSG00000206344	2,391763141	4,38E-35	1,64E-33
VEZF1	protein_coding	ENSG00000136451	-0,743488631	4,54E-35	1,7E-33
NDC80	protein_coding	ENSG00000080986	-0,825363023	5,06E-35	1,89E-33
TFAP2A-AS1	antisense	ENSG00000229950	2,351395929	5,49E-35	2,04E-33
FBXO32	protein_coding	ENSG00000156804	1,712439054	5,96E-35	2,21E-33
POLR1B	protein_coding	ENSG00000125630	-0,460393321	6,66E-35	2,47E-33
RIPK1	protein_coding	ENSG00000137275	0,617451894	6,86E-35	2,54E-33
ARHGAP29	protein_coding	ENSG00000137962	-0,702873343	1,08E-34	4,01E-33
CLK1	protein_coding	ENSG00000013441	0,978937763	1,14E-34	4,2E-33
ERO1B	protein_coding	ENSG00000086619	0,957170453	1,15E-34	4,24E-33
CCNYL1	protein_coding	ENSG00000163249	0,709237777	1,19E-34	4,37E-33
ANO6	protein_coding	ENSG00000177119	0,55582402	1,25E-34	4,58E-33
SNHG3	processed_transcript	ENSG00000242125	-0,879884331	1,4E-34	5,12E-33
NUP85	protein_coding	ENSG00000125450	-0,684805282	1,78E-34	6,49E-33
JPH2	protein_coding	ENSG00000149596	1,819156592	2,37E-34	8,64E-33
ALAS1	protein_coding	ENSG00000023330	0,448716421	2,78E-34	1,01E-32
DDX23	protein_coding	ENSG00000174243	-0,604757729	2,98E-34	1,08E-32
NUAK2	protein_coding	ENSG00000163545	1,126398311	3,49E-34	1,27E-32
LTBP2	protein_coding	ENSG00000119681	1,351791888	3,54E-34	1,28E-32
RGS4	protein_coding	ENSG00000117152	-0,777839058	3,56E-34	1,29E-32
KLF7	protein_coding	ENSG00000118263	0,630865223	4,15E-34	1,5E-32
CSMD3	protein_coding	ENSG00000164796	1,001333515	5,27E-34	1,9E-32
CSRNP2	protein_coding	ENSG00000110925	0,904946367	5,29E-34	1,9E-32
AKAP8L	protein_coding	ENSG00000011243	0,702687307	5,4E-34	1,94E-32
PDIA6	protein_coding	ENSG00000143870	0,464917666	5,64E-34	2,02E-32
ZFAND2A	protein_coding	ENSG00000178381	0,814702393	6,53E-34	2,34E-32
CKS2	protein_coding	ENSG00000123975	-1,02813813	7,22E-34	2,58E-32
MEAK7	protein_coding	ENSG00000140950	-0,475100988	9,86E-34	3,52E-32
UNG	protein_coding	ENSG00000076248	-0,805781059	1,03E-33	3,65E-32
NEU1	protein_coding	ENSG00000204386	0,951120943	1,13E-33	4,02E-32
ERLIN2	protein_coding	ENSG00000147475	-0,524270929	1,19E-33	4,21E-32
INHBB	protein_coding	ENSG00000163083	-1,137141003	1,28E-33	4,53E-32
RAD23A	protein_coding	ENSG00000179262	-0,59392451	1,32E-33	4,68E-32
OAS2	protein_coding	ENSG00000111335	4,211914854	1,33E-33	4,7E-32
CABYR	protein_coding	ENSG00000154040	0,827389165	1,44E-33	5,09E-32
DNAJB5	protein_coding	ENSG00000137094	1,092907225	1,48E-33	5,21E-32
IMPDH2	protein_coding	ENSG00000178035	-0,485960267	1,5E-33	5,27E-32
TP53BP2	protein_coding	ENSG00000143514	0,564206218	1,63E-33	5,71E-32
TUBB4B	protein_coding	ENSG00000188229	-0,589225809	1,72E-33	6,04E-32
MCM7	protein_coding	ENSG00000166508	-0,568420635	2,2E-33	7,69E-32

NDRG1	protein_coding	ENSG00000104419	0,876373109	2,23E-33	7,77E-32
PPFIBP1	protein_coding	ENSG00000110841	0,501137655	2,26E-33	7,88E-32
ZNF239	protein_coding	ENSG00000196793	-0,769431433	2,45E-33	8,5E-32
HOMEZ	protein_coding	ENSG00000215271	1,482950831	3,69E-33	1,28E-31
BCL6	protein_coding	ENSG00000113916	1,135798733	4,09E-33	1,42E-31
NECTIN4	protein_coding	ENSG00000143217	3,071857811	4,44E-33	1,54E-31
NDC1	protein_coding	ENSG00000058804	-0,801414331	4,67E-33	1,61E-31
CSTF3	protein_coding	ENSG00000176102	0,532025134	4,81E-33	1,66E-31
FRMD7	protein_coding	ENSG00000165694	3,222918122	5,41E-33	1,86E-31
ACHE	protein_coding	ENSG00000087085	2,787587782	5,75E-33	1,98E-31
AKAP1	protein_coding	ENSG00000121057	-0,843328732	6,21E-33	2,13E-31
DZIP3	protein_coding	ENSG00000198919	0,605391374	6,34E-33	2,17E-31
SH3RF1	protein_coding	ENSG00000154447	0,579476885	6,64E-33	2,27E-31
TBCEL	protein_coding	ENSG00000154114	0,717247394	7,49E-33	2,56E-31
GLIPR1	protein_coding	ENSG00000139278	0,772311505	8,34E-33	2,84E-31
NEGR1	protein_coding	ENSG00000172260	0,693949265	8,75E-33	2,98E-31
SRSF7	protein_coding	ENSG00000115875	-0,614001948	9,95E-33	3,38E-31
WWP1	protein_coding	ENSG00000123124	0,626819488	1,22E-32	4,14E-31
SLC35F2	protein_coding	ENSG00000110660	0,467076254	1,29E-32	4,36E-31
MELK	protein_coding	ENSG00000165304	-0,699754398	1,31E-32	4,43E-31
FAM57A	protein_coding	ENSG00000167695	-0,674917762	1,39E-32	4,69E-31
MAP2	protein_coding	ENSG00000078018	1,781398809	1,48E-32	5E-31
GBP4	protein_coding	ENSG00000162654	4,005591482	1,83E-32	6,14E-31
CD22	protein_coding	ENSG00000012124	1,500888416	1,93E-32	6,49E-31
ZNF627	protein_coding	ENSG00000198551	0,976369052	1,98E-32	6,63E-31
TRNP1	protein_coding	ENSG00000253368	0,959000267	2,54E-32	8,49E-31
SHISA2	protein_coding	ENSG00000180730	-0,740978085	3,21E-32	1,07E-30
SERINC1	protein_coding	ENSG00000111897	0,489633374	3,24E-32	1,08E-30
STARD8	protein_coding	ENSG00000130052	-0,948530671	3,41E-32	1,14E-30
PPP2R5D	protein_coding	ENSG00000112640	-0,613847325	4,35E-32	1,45E-30
TNXB	protein_coding	ENSG00000168477	2,676300803	4,46E-32	1,48E-30
OSER1-DT	lincRNA	ENSG00000223891	1,54159198	4,65E-32	1,54E-30
FGF1	protein_coding	ENSG00000113578	0,613759779	4,84E-32	1,6E-30
USP43	protein_coding	ENSG00000154914	1,379966153	5,07E-32	1,67E-30
GEMIN5	protein_coding	ENSG00000082516	-0,575948998	5,07E-32	1,67E-30
SUOX	protein_coding	ENSG00000139531	-0,909081473	6,72E-32	2,21E-30
ITGAM	protein_coding	ENSG00000169896	3,538316434	8,02E-32	2,63E-30
HELQ	protein_coding	ENSG00000163312	0,84028643	8,06E-32	2,65E-30
EIF2AK3	protein_coding	ENSG00000172071	0,800969618	8,32E-32	2,72E-30
C1orf35	protein_coding	ENSG00000143793	-0,666699801	8,33E-32	2,72E-30

GRWD1	protein_coding	ENSG00000105447	-0,639716714	8,67E-32	2,83E-30
YWHAH	protein_coding	ENSG00000128245	-0,620797831	8,71E-32	2,84E-30
C1orf174	protein_coding	ENSG00000198912	-0,765225195	9,29E-32	3,03E-30
FKBP4	protein_coding	ENSG00000004478	-0,473862608	1,06E-31	3,45E-30
AF117829.1	lincRNA	ENSG00000251136	1,145048284	1,22E-31	3,96E-30
SYAP1	protein_coding	ENSG00000169895	0,430722522	1,28E-31	4,15E-30
HIST1H4C	protein_coding	ENSG00000197061	-1,05643012	1,32E-31	4,27E-30
ULBP2	protein_coding	ENSG00000131015	1,57171031	1,34E-31	4,34E-30
RAC3	protein_coding	ENSG00000169750	-0,928177793	1,43E-31	4,6E-30
ECT2	protein_coding	ENSG00000114346	-0,925372568	1,6E-31	5,15E-30
RIPK2	protein_coding	ENSG00000104312	0,737425349	1,64E-31	5,29E-30
TNFRSF10B	protein_coding	ENSG00000120889	0,5294375	1,75E-31	5,63E-30
YES1	protein_coding	ENSG00000176105	0,468494069	1,95E-31	6,25E-30
MID1IP1	protein_coding	ENSG00000165175	-0,674022541	2,28E-31	7,3E-30
RPP25	protein_coding	ENSG00000178718	-0,665326895	2,5E-31	8E-30
FAM214B	protein_coding	ENSG00000005238	0,959968581	2,56E-31	8,16E-30
NUDT4	protein_coding	ENSG00000173598	0,612125404	2,69E-31	8,56E-30
XDH	protein_coding	ENSG00000158125	0,769535959	2,86E-31	9,09E-30
SMURF1	protein_coding	ENSG00000198742	0,75524906	2,92E-31	9,27E-30
STC2	protein_coding	ENSG00000113739	0,831045801	2,93E-31	9,3E-30
PAPPA2	protein_coding	ENSG00000116183	2,336066648	3,36E-31	1,06E-29
KIF23	protein_coding	ENSG00000137807	-0,770797481	3,52E-31	1,11E-29
LOXL1-AS1	antisense	ENSG00000261801	-0,66828561	3,99E-31	1,26E-29
ZNF267	protein_coding	ENSG00000185947	0,849513803	4,57E-31	1,44E-29
CYLD	protein_coding	ENSG00000083799	0,790103572	4,75E-31	1,49E-29
GTF3C2	protein_coding	ENSG00000115207	-0,557995391	5,81E-31	1,83E-29
ATF7IP2	protein_coding	ENSG00000166669	0,974651279	5,87E-31	1,84E-29
PPTC7	protein_coding	ENSG00000196850	0,703083775	6,17E-31	1,93E-29
PHF13	protein_coding	ENSG00000116273	-0,849713522	6,34E-31	1,98E-29
CBX2	protein_coding	ENSG00000173894	-1,055129794	7,04E-31	2,2E-29
PRPF4	protein_coding	ENSG00000136875	-0,572960696	7,77E-31	2,42E-29
ERCC6L	protein_coding	ENSG00000186871	-1,00981668	7,79E-31	2,42E-29
EME1	protein_coding	ENSG00000154920	-0,795190911	7,87E-31	2,45E-29
SLC39A14	protein_coding	ENSG00000104635	0,422684027	8,55E-31	2,65E-29
SP140	protein_coding	ENSG00000079263	0,441872841	9,61E-31	2,98E-29
HNRNPDL	protein_coding	ENSG00000152795	-0,867896564	1,06E-30	3,27E-29
TSC22D3	protein_coding	ENSG00000157514	1,263922359	1,08E-30	3,32E-29
SUN3	protein_coding	ENSG00000164744	0,922688005	1,09E-30	3,36E-29
TSEN54	protein_coding	ENSG00000182173	-0,730511715	1,12E-30	3,46E-29
BTN3A2	protein_coding	ENSG00000186470	-0,585264262	1,14E-30	3,51E-29

STAMBPL1	protein_coding	ENSG00000138134	0,65593493	1,35E-30	4,14E-29
METTL3	protein_coding	ENSG00000165819	-0,52789912	1,48E-30	4,53E-29
G2E3	protein_coding	ENSG00000092140	-0,965398598	1,55E-30	4,76E-29
ITGB8	protein_coding	ENSG00000105855	2,034296157	1,64E-30	5,02E-29
MICAL2	protein_coding	ENSG00000133816	0,635956021	1,89E-30	5,78E-29
MICAL2	protein_coding	ENSG00000133816	0,635956021	1,89E-30	5,78E-29
TCF19	protein_coding	ENSG00000137310	-0,583166759	2,09E-30	6,36E-29
AMIGO2	protein_coding	ENSG00000139211	0,604508978	2,14E-30	6,52E-29
TRABD2A	protein_coding	ENSG00000186854	1,43920089	2,2E-30	6,69E-29
GLT8D1	protein_coding	ENSG00000016864	-0,554299424	2,72E-30	8,27E-29
EXOSC6	protein_coding	ENSG00000223496	-0,695127616	2,8E-30	8,48E-29
NFE4	protein_coding	ENSG00000230257	0,630984158	2,83E-30	8,56E-29
MCTP1	protein_coding	ENSG00000175471	0,510255339	2,88E-30	8,69E-29
THAP4	protein_coding	ENSG00000176946	-0,587527913	2,94E-30	8,86E-29
SELPLG	protein_coding	ENSG00000110876	1,67851851	3,4E-30	1,02E-28
SERPINB8	protein_coding	ENSG00000166401	0,76564749	3,66E-30	1,1E-28
AL078459.1	antisense	ENSG00000223653	3,930113707	3,73E-30	1,12E-28
YBX3	protein_coding	ENSG00000060138	0,373267092	3,74E-30	1,12E-28
KLHL21	protein_coding	ENSG00000162413	1,242049025	4,35E-30	1,3E-28
LINC00707	lincRNA	ENSG00000238266	-0,707183393	4,58E-30	1,37E-28
TMEM39A	protein_coding	ENSG00000176142	0,56641876	4,86E-30	1,45E-28
MED7	protein_coding	ENSG00000155868	-0,887137401	5,56E-30	1,66E-28
SLFN12	protein_coding	ENSG00000172123	0,553749725	6,84E-30	2,04E-28
PPP1R10	protein_coding	ENSG00000204569	0,718288644	9,25E-30	2,75E-28
ITCH	protein_coding	ENSG00000078747	0,40592101	9,38E-30	2,78E-28
MICALL2	protein_coding	ENSG00000164877	1,213559189	9,48E-30	2,81E-28
DNAJC1	protein_coding	ENSG00000136770	0,610814401	1,01E-29	2,98E-28
CUTC	protein_coding	ENSG00000119929	-0,695574084	1,07E-29	3,16E-28
ACSL5	protein_coding	ENSG00000197142	2,28279954	1,14E-29	3,35E-28
PRPS1	protein_coding	ENSG00000147224	-0,664700574	1,32E-29	3,9E-28
CLU	protein_coding	ENSG00000120885	0,969813292	1,37E-29	4,03E-28
LRP1B	protein_coding	ENSG00000168702	3,767470502	1,39E-29	4,09E-28
VASN	protein_coding	ENSG00000168140	1,755283733	1,39E-29	4,09E-28
RNF216	protein_coding	ENSG00000011275	-0,52348625	1,57E-29	4,6E-28
TUT7	protein_coding	ENSG00000083223	0,494899095	1,62E-29	4,75E-28
ZNF622	protein_coding	ENSG00000173545	0,573672027	1,69E-29	4,92E-28
PCK2	protein_coding	ENSG00000100889	1,168738171	1,7E-29	4,95E-28
SAMD8	protein_coding	ENSG00000156671	0,696848382	1,7E-29	4,95E-28
C2orf69	protein_coding	ENSG00000178074	-0,978646432	1,8E-29	5,23E-28
LINC01776	lincRNA	ENSG00000226053	1,562698346	1,92E-29	5,58E-28

APAF1	protein_coding	ENSG00000120868	0,535630104	2,03E-29	5,88E-28
CDKN1C	protein_coding	ENSG00000129757	1,65743597	2,04E-29	5,92E-28
TRIP13	protein_coding	ENSG00000071539	-0,592528972	2,13E-29	6,16E-28
GATA6	protein_coding	ENSG00000141448	1,973928621	2,3E-29	6,64E-28
GCA	protein_coding	ENSG00000115271	0,813386808	2,47E-29	7,13E-28
TCN2	protein_coding	ENSG00000185339	1,205463096	2,57E-29	7,42E-28
TEP1	protein_coding	ENSG00000129566	1,181259785	2,91E-29	8,36E-28
LPCAT1	protein_coding	ENSG00000153395	-0,93322385	2,96E-29	8,5E-28
SEPSECS	protein_coding	ENSG00000109618	0,831581955	3,08E-29	8,84E-28
TNFRSF10A	protein_coding	ENSG00000104689	0,632131714	3,1E-29	8,87E-28
ANKRD27	protein_coding	ENSG00000105186	-0,669527729	3,17E-29	9,06E-28
SLC12A4	protein_coding	ENSG00000124067	0,923357172	3,44E-29	9,82E-28
ECM2	protein_coding	ENSG00000106823	2,309771762	3,55E-29	1,01E-27
NCAPH	protein_coding	ENSG00000121152	-0,837977815	3,77E-29	1,07E-27
CAMK1G	protein_coding	ENSG00000008118	2,335054028	4,04E-29	1,15E-27
AL450992.1	antisense	ENSG00000229021	4,178878171	4,09E-29	1,16E-27
SPARC	protein_coding	ENSG00000113140	-0,41713261	4,11E-29	1,17E-27
RHOQ	protein_coding	ENSG00000119729	0,527753489	4,41E-29	1,25E-27
CSF2	protein_coding	ENSG00000164400	2,18511908	4,49E-29	1,27E-27
AC090409.1	lincRNA	ENSG00000267279	-0,905006393	4,66E-29	1,32E-27
ARHGAP24	protein_coding	ENSG00000138639	0,632918169	5,56E-29	1,57E-27
COQ10B	protein_coding	ENSG00000115520	0,633399027	5,63E-29	1,59E-27
UBTF	protein_coding	ENSG00000108312	-0,758810608	5,73E-29	1,61E-27
BORA	protein_coding	ENSG00000136122	-0,792975739	5,77E-29	1,62E-27
HIST2H2AC	protein_coding	ENSG00000184260	-1,026932868	5,76E-29	1,62E-27
PNMA1	protein_coding	ENSG00000176903	-0,57426658	5,85E-29	1,64E-27
ACVR1	protein_coding	ENSG00000115170	0,700487464	5,97E-29	1,67E-27
TGM4	protein_coding	ENSG00000163810	3,083117275	6,34E-29	1,77E-27
LIMK2	protein_coding	ENSG00000182541	0,793189691	6,85E-29	1,91E-27
CXXC1	protein_coding	ENSG00000154832	-0,60603878	6,85E-29	1,91E-27
UCK2	protein_coding	ENSG00000143179	-0,528499214	7,39E-29	2,06E-27
GRAP	protein_coding	ENSG00000154016	5,537825093	7,66E-29	2,13E-27
ARRDC3	protein_coding	ENSG00000113369	0,803271626	7,69E-29	2,14E-27
TLK2	protein_coding	ENSG00000146872	0,489707701	7,76E-29	2,15E-27
HERC6	protein_coding	ENSG00000138642	1,061977385	8,25E-29	2,28E-27
P2RX4	protein_coding	ENSG00000135124	0,982854365	9,13E-29	2,52E-27
AC100800.1	antisense	ENSG00000187229	2,807642278	9,79E-29	2,7E-27
BTG2	protein_coding	ENSG00000159388	1,078318137	1,01E-28	2,78E-27
CCBE1	protein_coding	ENSG00000183287	-0,519300709	1,1E-28	3,01E-27
RHOB	protein_coding	ENSG00000143878	0,812600296	1,13E-28	3,11E-27

OAS3	protein_coding	ENSG00000111331	0,981905236	1,37E-28	3,76E-27
SPC24	protein_coding	ENSG00000161888	-0,616340655	1,51E-28	4,13E-27
SDC4	protein_coding	ENSG00000124145	0,626440341	1,92E-28	5,26E-27
PRMT6	protein_coding	ENSG00000198890	-0,822487774	1,93E-28	5,27E-27
BRD8	protein_coding	ENSG00000112983	-0,503736498	2,13E-28	5,81E-27
FSCN1	protein_coding	ENSG00000075618	0,957201041	2,23E-28	6,08E-27
RHNO1	protein_coding	ENSG00000171792	-0,6328145	2,4E-28	6,54E-27
ST7L	protein_coding	ENSG00000007341	0,569320323	2,46E-28	6,68E-27
PLCB4	protein_coding	ENSG00000101333	0,626811751	2,71E-28	7,37E-27
TIGD1	protein_coding	ENSG00000221944	-1,026005995	2,74E-28	7,42E-27
MRPS26	protein_coding	ENSG00000125901	-0,742357588	2,79E-28	7,57E-27
CFAP54	protein_coding	ENSG00000188596	1,924286924	2,86E-28	7,75E-27
ERAP1	protein_coding	ENSG00000164307	0,658119937	3,12E-28	8,43E-27
DDR2	protein_coding	ENSG00000162733	1,358538898	3,55E-28	9,56E-27
RNF6	protein_coding	ENSG00000127870	0,529978008	4,18E-28	1,13E-26
C1orf216	protein_coding	ENSG00000142686	-0,655518356	4,33E-28	1,16E-26
THAP11	protein_coding	ENSG00000168286	-0,744154106	4,37E-28	1,17E-26
ARHGAP10	protein_coding	ENSG00000071205	0,450873401	5,09E-28	1,36E-26
NUP50-DT	lincRNA	ENSG00000226328	1,576021338	5,17E-28	1,38E-26
BCL7A	protein_coding	ENSG00000110987	-0,648768011	5,14E-28	1,38E-26
PMPCA	protein_coding	ENSG00000165688	-0,428483009	6,04E-28	1,61E-26
BAG3	protein_coding	ENSG00000151929	0,647653526	6,45E-28	1,72E-26
THEM6	protein_coding	ENSG00000130193	-0,858629644	6,71E-28	1,79E-26
ELOVL7	protein_coding	ENSG00000164181	0,918593052	7,07E-28	1,88E-26
STAT3	protein_coding	ENSG00000168610	0,543153141	7,41E-28	1,97E-26
CPOX	protein_coding	ENSG00000080819	-0,794087573	7,6E-28	2,02E-26
RANBP6	protein_coding	ENSG00000137040	-0,545334048	7,65E-28	2,03E-26
NAV3	protein_coding	ENSG00000067798	0,454309406	8,05E-28	2,13E-26
TMTC4	protein_coding	ENSG00000125247	-0,696252161	8,19E-28	2,16E-26
ARNTL	protein_coding	ENSG00000133794	0,703379131	8,4E-28	2,22E-26
ARHGAP31	protein_coding	ENSG00000031081	0,677221759	8,48E-28	2,23E-26
HNRNPR	protein_coding	ENSG00000125944	-0,777099028	9,88E-28	2,6E-26
CCL3	protein_coding	ENSG00000277632	1,101223988	1,02E-27	2,69E-26
TYW5	protein_coding	ENSG00000162971	0,95884762	1,07E-27	2,82E-26
RAD51	protein_coding	ENSG00000051180	-0,853311204	1,09E-27	2,86E-26
TNFAIP2	protein_coding	ENSG00000185215	1,241158652	1,1E-27	2,89E-26
PSMG4	protein_coding	ENSG00000180822	-0,673325527	1,13E-27	2,96E-26
CSTF1	protein_coding	ENSG00000101138	-0,553856116	1,14E-27	2,99E-26
RSAD2	protein_coding	ENSG00000134321	4,096765908	1,28E-27	3,34E-26
VPS26B	protein_coding	ENSG00000151502	-0,52454454	1,33E-27	3,46E-26

IGFBP1	protein_coding	ENSG00000146678	-0,654106379	1,38E-27	3,6E-26
HPS6	protein_coding	ENSG00000166189	-0,751949099	1,59E-27	4,13E-26
TFAP4	protein_coding	ENSG00000090447	-0,907671962	1,61E-27	4,17E-26
RBM23	protein_coding	ENSG00000100461	-0,533431923	1,66E-27	4,31E-26
MED20	protein_coding	ENSG00000124641	-0,59587339	1,9E-27	4,93E-26
MAP2K3	protein_coding	ENSG00000034152	0,846802174	1,91E-27	4,94E-26
ITGA5	protein_coding	ENSG00000161638	0,652718116	2,15E-27	5,56E-26
MCM4	protein_coding	ENSG00000104738	-0,699894329	2,43E-27	6,28E-26
CCNB2	protein_coding	ENSG00000157456	-0,705875066	2,46E-27	6,34E-26
PNMA2	protein_coding	ENSG00000240694	-0,588843918	2,49E-27	6,42E-26
SRRT	protein_coding	ENSG00000087087	-0,652652831	2,66E-27	6,83E-26
CCL4	protein_coding	ENSG00000275302	5,74379156	2,79E-27	7,16E-26
CCDC92	protein_coding	ENSG00000119242	1,076866765	2,92E-27	7,49E-26
TANK	protein_coding	ENSG00000136560	0,72609066	3,04E-27	7,8E-26
POMK	protein_coding	ENSG00000185900	-0,969357772	3,08E-27	7,88E-26
ZNF438	protein_coding	ENSG00000183621	0,818761779	3,1E-27	7,92E-26
HES1	protein_coding	ENSG00000114315	1,116139436	3,49E-27	8,9E-26
NUCB2	protein_coding	ENSG00000070081	0,63456779	3,6E-27	9,18E-26
VGLL3	protein_coding	ENSG00000206538	0,666610429	3,74E-27	9,52E-26
LDLRAP1	protein_coding	ENSG00000157978	-0,729500921	4,16E-27	1,06E-25
IL11	protein_coding	ENSG00000095752	1,890347319	4,32E-27	1,1E-25
RRM2B	protein_coding	ENSG00000048392	0,743808007	4,36E-27	1,11E-25
SPRED2	protein_coding	ENSG00000198369	0,779370812	5,3E-27	1,34E-25
RAP2B	protein_coding	ENSG00000181467	-0,346446108	5,84E-27	1,48E-25
CEBPG	protein_coding	ENSG00000153879	0,444775549	6,01E-27	1,52E-25
RACGAP1	protein_coding	ENSG00000161800	-0,677306578	6,15E-27	1,55E-25
FEN1	protein_coding	ENSG00000168496	-0,711486098	6,54E-27	1,65E-25
ARHGAP45	protein_coding	ENSG00000180448	1,608490937	6,61E-27	1,66E-25
AC105277.1	lincRNA	ENSG00000232453	1,105712782	6,67E-27	1,67E-25
NFIL3	protein_coding	ENSG00000165030	0,690215457	6,63E-27	1,67E-25
ENTPD6	protein_coding	ENSG00000197586	-0,547882107	6,94E-27	1,74E-25
INCENP	protein_coding	ENSG00000149503	-0,848947395	7,29E-27	1,83E-25
UVRAG	protein_coding	ENSG00000198382	0,519840438	7,37E-27	1,84E-25
TACC3	protein_coding	ENSG00000013810	-0,656092379	7,54E-27	1,88E-25
KLHDC3	protein_coding	ENSG00000124702	-0,635580587	7,63E-27	1,9E-25
C1GALT1C1	protein_coding	ENSG00000171155	-0,825629701	8,34E-27	2,08E-25
ZBTB43	protein_coding	ENSG00000169155	0,683205708	9,09E-27	2,26E-25
CTCF	protein_coding	ENSG00000102974	-0,678609868	9,5E-27	2,36E-25
DNAJC10	protein_coding	ENSG00000077232	0,443499449	9,69E-27	2,41E-25
HMGXB4	protein_coding	ENSG00000100281	-0,761766096	1,05E-26	2,59E-25

IFI16	protein_coding	ENSG00000163565	0,550207904	1,08E-26	2,69E-25
ZNFX1	protein_coding	ENSG00000124201	0,783049457	1,1E-26	2,73E-25
NCF2	protein_coding	ENSG00000116701	2,091579184	1,13E-26	2,79E-25
SDAD1P1	transcribed_processed_	ENSG00000228451	0,689897652	1,18E-26	2,91E-25
MXD3	protein_coding	ENSG00000213347	-0,876447346	1,26E-26	3,1E-25
SLC35B1	protein_coding	ENSG00000121073	0,526977201	1,32E-26	3,26E-25
LTA	protein_coding	ENSG00000226979	5,712979707	1,34E-26	3,3E-25
ZWINT	protein_coding	ENSG00000122952	-0,586973525	1,35E-26	3,31E-25
FAP	protein_coding	ENSG00000078098	4,729260929	1,43E-26	3,51E-25
SYTL2	protein_coding	ENSG00000137501	0,862391114	1,5E-26	3,67E-25
TSPYL4	protein_coding	ENSG00000187189	-0,816818442	1,51E-26	3,69E-25
ZNF790	protein_coding	ENSG00000197863	0,920689904	1,52E-26	3,72E-25
FICD	protein_coding	ENSG00000198855	0,882162841	1,72E-26	4,19E-25
MED13	protein_coding	ENSG00000108510	0,554595887	1,71E-26	4,19E-25
LINC00888	transcribed_unprocesse	ENSG00000240024	1,08889101	1,82E-26	4,42E-25
DNHD1	protein_coding	ENSG00000179532	1,739585744	1,83E-26	4,46E-25
PRPSAP2	protein_coding	ENSG00000141127	0,488717066	1,91E-26	4,64E-25
PDZD2	protein_coding	ENSG00000133401	2,349913294	2,04E-26	4,95E-25
FNIP2	protein_coding	ENSG00000052795	0,576570553	2,05E-26	4,96E-25
PHACTR4	protein_coding	ENSG00000204138	0,507626809	2,33E-26	5,65E-25
RAB30	protein_coding	ENSG00000137502	0,721100069	2,94E-26	7,12E-25
NR1D1	protein_coding	ENSG00000126368	1,591378175	3E-26	7,25E-25
PLCH2	protein_coding	ENSG00000149527	2,583240087	3,01E-26	7,26E-25
CAMKK1	protein_coding	ENSG00000004660	-1,209935765	3,06E-26	7,38E-25
KCTD5	protein_coding	ENSG00000167977	0,676269665	3,09E-26	7,45E-25
LAPTM5	protein_coding	ENSG00000162511	3,101446956	3,45E-26	8,29E-25
PRDM10	protein_coding	ENSG00000170325	-1,008745779	3,47E-26	8,34E-25
NXF1	protein_coding	ENSG00000162231	0,8164412	3,61E-26	8,66E-25
CDK1	protein_coding	ENSG00000170312	-0,904004508	3,79E-26	9,09E-25
POLD3	protein_coding	ENSG00000077514	-0,787994584	3,92E-26	9,38E-25
RAB3GAP2	protein_coding	ENSG00000118873	0,431698409	3,97E-26	9,49E-25
MAZ	protein_coding	ENSG00000103495	-0,623700194	4,03E-26	9,62E-25
FBXO5	protein_coding	ENSG00000112029	-0,987763297	4,06E-26	9,68E-25
CPEB3	protein_coding	ENSG00000107864	1,17849648	4,41E-26	1,05E-24
ZSCAN12	protein_coding	ENSG00000158691	0,762772997	4,4E-26	1,05E-24
ASB13	protein_coding	ENSG00000196372	-0,954517503	4,67E-26	1,11E-24
SERP1	protein_coding	ENSG00000120742	0,506219323	4,77E-26	1,13E-24
KLF13	protein_coding	ENSG00000169926	-0,792300409	4,82E-26	1,14E-24
FBN1	protein_coding	ENSG00000166147	0,83247056	5,06E-26	1,2E-24
PLEK2	protein_coding	ENSG00000100558	0,822881986	5,23E-26	1,24E-24

APOL1	protein_coding	ENSG00000100342	2,829254475	5,42E-26	1,28E-24
SCN2A	protein_coding	ENSG00000136531	1,301833784	5,76E-26	1,36E-24
AL357060.1	antisense	ENSG00000237499	1,948064883	5,95E-26	1,4E-24
HLA-B	protein_coding	ENSG00000234745	1,000633206	6,29E-26	1,48E-24
ANLN	protein_coding	ENSG00000011426	-0,649779866	6,52E-26	1,53E-24
FGD5-AS1	antisense	ENSG00000225733	-0,507704407	6,64E-26	1,56E-24
RYBP	protein_coding	ENSG00000163602	0,616214186	7,24E-26	1,7E-24
IL15	protein_coding	ENSG00000164136	2,038214564	7,44E-26	1,74E-24
SEC23B	protein_coding	ENSG00000101310	0,401464941	7,74E-26	1,81E-24
TOP1	protein_coding	ENSG00000198900	0,724030627	7,82E-26	1,83E-24
CLEC17A	protein_coding	ENSG00000187912	8,753514801	8,77E-26	2,05E-24
SPG7	protein_coding	ENSG00000197912	-0,631878309	9,7E-26	2,26E-24
FRMD5	protein_coding	ENSG00000171877	0,695256773	1,02E-25	2,37E-24
THAP10	protein_coding	ENSG00000129028	1,056846477	1,07E-25	2,48E-24
JMJD1C	protein_coding	ENSG00000171988	0,578784518	1,21E-25	2,82E-24
EPHA4	protein_coding	ENSG00000116106	0,747574462	1,23E-25	2,85E-24
ARC	protein_coding	ENSG00000198576	3,985932077	1,24E-25	2,87E-24
ERP44	protein_coding	ENSG00000023318	0,594945798	1,25E-25	2,9E-24
ALKBH2	protein_coding	ENSG00000189046	-0,728825828	1,3E-25	3,01E-24
CDC20P1	processed_pseudogene	ENSG00000231007	-0,986627841	1,48E-25	3,42E-24
WDR5	protein_coding	ENSG00000196363	-0,467802914	1,54E-25	3,56E-24
COLGALT1	protein_coding	ENSG00000130309	-0,557362945	1,55E-25	3,58E-24
P3H2	protein_coding	ENSG00000090530	0,520213347	1,57E-25	3,63E-24
SMYD5	protein_coding	ENSG00000135632	-0,529263378	1,59E-25	3,65E-24
RBM12	protein_coding	ENSG00000244462	-0,710044026	1,67E-25	3,83E-24
ARHGAP19	protein_coding	ENSG00000213390	-0,957914173	1,93E-25	4,44E-24
DUSP4	protein_coding	ENSG00000120875	0,673100669	2E-25	4,6E-24
HAPLN3	protein_coding	ENSG00000140511	1,964005317	2,01E-25	4,61E-24
AL024507.2	antisense	ENSG00000272476	1,767112298	2,21E-25	5,05E-24
ATP6V1A	protein_coding	ENSG00000114573	0,381855598	2,37E-25	5,43E-24
AC024940.1	antisense	ENSG00000177340	3,182490061	2,39E-25	5,45E-24
CYSRT1	protein_coding	ENSG00000197191	2,265599897	2,6E-25	5,93E-24
MCM10	protein_coding	ENSG00000065328	-0,726904197	2,7E-25	6,15E-24
DIO2	protein_coding	ENSG00000211448	3,569318482	2,75E-25	6,25E-24
SNORD13	snoRNA	ENSG00000239039	-1,520573999	2,8E-25	6,36E-24
TBC1D12	protein_coding	ENSG00000108239	0,549703349	2,89E-25	6,57E-24
FRMD6	protein_coding	ENSG00000139926	0,351676422	2,93E-25	6,64E-24
HSPA8	protein_coding	ENSG00000109971	-0,739539424	3,34E-25	7,58E-24
NFAT5	protein_coding	ENSG00000102908	0,74690615	3,42E-25	7,74E-24
DYNC1I1	protein_coding	ENSG00000158560	1,376095603	3,69E-25	8,35E-24

IFNB1	protein_coding	ENSG00000171855	9,009121805	3,86E-25	8,72E-24
HMGCS1	protein_coding	ENSG00000112972	0,52082246	4,24E-25	9,57E-24
EGR4	protein_coding	ENSG00000135625	6,93441413	4,77E-25	1,08E-23
FOS	protein_coding	ENSG00000170345	1,191985312	5,17E-25	1,16E-23
TLNRD1	protein_coding	ENSG00000140406	-0,729945074	5,31E-25	1,19E-23
BTG4	protein_coding	ENSG00000137707	1,982298388	5,89E-25	1,32E-23
KLHL28	protein_coding	ENSG00000179454	0,771811808	6,14E-25	1,38E-23
ADAM12	protein_coding	ENSG00000148848	1,563429755	6,51E-25	1,46E-23
SENCR	antisense	ENSG00000254703	1,298188391	6,67E-25	1,49E-23
RUVBL1	protein_coding	ENSG00000175792	-0,465816542	6,75E-25	1,51E-23
SYNPO	protein_coding	ENSG00000171992	1,115441835	6,83E-25	1,53E-23
SORCS2	protein_coding	ENSG00000184985	1,391348722	6,96E-25	1,55E-23
ING5	protein_coding	ENSG00000168395	-0,665953561	6,93E-25	1,55E-23
KCNN2	protein_coding	ENSG00000080709	1,219549964	7E-25	1,56E-23
ADM2	protein_coding	ENSG00000128165	1,430881121	7,33E-25	1,63E-23
HIST1H2AC	protein_coding	ENSG00000180573	-0,824768924	7,65E-25	1,7E-23
SYNE1	protein_coding	ENSG00000131018	0,884541572	7,91E-25	1,75E-23
NAT10	protein_coding	ENSG00000135372	-0,512175621	7,88E-25	1,75E-23
SUSD6	protein_coding	ENSG00000100647	1,064096244	8,09E-25	1,79E-23
INTS14	protein_coding	ENSG00000138614	-0,520951026	8,23E-25	1,82E-23
EGR1	protein_coding	ENSG00000120738	2,081891811	8,55E-25	1,89E-23
EVA1C	protein_coding	ENSG00000166979	-0,940099973	8,67E-25	1,91E-23
IP6K3	protein_coding	ENSG00000161896	5,420670601	9,19E-25	2,03E-23
PIM2	protein_coding	ENSG00000102096	-0,626745983	9,56E-25	2,11E-23
AC107959.1	antisense	ENSG00000245025	2,530450649	9,78E-25	2,15E-23
RNU1-2	snRNA	ENSG00000207005	-2,474493275	1,05E-24	2,3E-23
NDE1	protein_coding	ENSG00000072864	-0,589277394	1,08E-24	2,37E-23
KBTBD4	protein_coding	ENSG00000123444	-0,655911155	1,11E-24	2,44E-23
ADM	protein_coding	ENSG00000148926	0,654487184	1,15E-24	2,51E-23
FBXL13	protein_coding	ENSG00000161040	0,726836108	1,16E-24	2,55E-23
BCL2L13	protein_coding	ENSG00000099968	0,423779396	1,17E-24	2,55E-23
CENPI	protein_coding	ENSG00000102384	-0,645537571	1,21E-24	2,64E-23
OSMR	protein_coding	ENSG00000145623	0,450811751	1,24E-24	2,7E-23
IFNL1	protein_coding	ENSG00000182393	8,952448426	1,24E-24	2,71E-23
CHN1	protein_coding	ENSG00000128656	0,608172669	1,28E-24	2,78E-23
REL	protein_coding	ENSG00000162924	0,945596815	1,54E-24	3,35E-23
SLC12A2	protein_coding	ENSG00000064651	0,520059462	1,55E-24	3,36E-23
NOP14-AS1	antisense	ENSG00000249673	0,863041435	1,61E-24	3,49E-23
DSN1	protein_coding	ENSG00000149636	-0,69312684	1,65E-24	3,58E-23
PIK3C2A	protein_coding	ENSG00000011405	0,611989229	1,77E-24	3,83E-23

IRGQ	protein_coding	ENSG00000167378	0,922861678	1,78E-24	3,86E-23
STX6	protein_coding	ENSG00000135823	0,470798008	1,83E-24	3,94E-23
BUB1B	protein_coding	ENSG00000156970	-0,77601345	2,07E-24	4,46E-23
MAGT1	protein_coding	ENSG00000102158	0,589667248	2,1E-24	4,54E-23
RUNX1	protein_coding	ENSG00000159216	0,608346677	2,11E-24	4,55E-23
SLC25A12	protein_coding	ENSG00000115840	-0,592422348	2,16E-24	4,64E-23
CLEC2D	protein_coding	ENSG00000069493	1,986213552	2,33E-24	5,02E-23
ZBTB1	protein_coding	ENSG00000126804	0,571456315	2,57E-24	5,53E-23
GABARAPL1	protein_coding	ENSG00000139112	0,525092807	2,77E-24	5,94E-23
C6orf106	protein_coding	ENSG00000196821	0,519796454	2,8E-24	6E-23
CYGB	protein_coding	ENSG00000161544	2,768793712	2,81E-24	6,01E-23
NLE1	protein_coding	ENSG00000073536	-0,59952177	2,84E-24	6,07E-23
ELL2	protein_coding	ENSG00000118985	0,512244604	2,97E-24	6,35E-23
ZNF620	protein_coding	ENSG00000177842	0,909541264	3,05E-24	6,51E-23
FAM102B	protein_coding	ENSG00000162636	-0,749233697	3,16E-24	6,73E-23
PPP1R3B	protein_coding	ENSG00000173281	0,552394817	3,43E-24	7,3E-23
EPC1	protein_coding	ENSG00000120616	0,70282637	3,45E-24	7,33E-23
UGDH	protein_coding	ENSG00000109814	0,510415363	3,76E-24	8E-23
E2F2	protein_coding	ENSG00000007968	-1,038148095	3,77E-24	8,01E-23
DDX17	protein_coding	ENSG00000100201	-0,507236217	3,83E-24	8,12E-23
CD3EAP	protein_coding	ENSG00000117877	-0,476193915	3,86E-24	8,17E-23
LMAN1	protein_coding	ENSG00000074695	0,445195538	4,19E-24	8,87E-23
IL32	protein_coding	ENSG00000008517	1,56019472	4,35E-24	9,19E-23
ELAC2	protein_coding	ENSG00000006744	-0,475217332	4,47E-24	9,44E-23
ADAMTS18	protein_coding	ENSG00000140873	1,842070682	4,72E-24	9,96E-23
KIF4A	protein_coding	ENSG00000090889	-0,708322986	4,99E-24	1,05E-22
PIWIL2	protein_coding	ENSG00000197181	1,637437845	5,16E-24	1,09E-22
SPTLC2	protein_coding	ENSG00000100596	-0,49721495	5,42E-24	1,14E-22
FZR1	protein_coding	ENSG00000105325	-0,627748997	5,41E-24	1,14E-22
GEMIN6	protein_coding	ENSG00000152147	-0,881486079	5,86E-24	1,23E-22
HNRNPA1	protein_coding	ENSG00000135486	-0,600797193	6,08E-24	1,28E-22
MISP	protein_coding	ENSG00000099812	1,060034069	6,32E-24	1,32E-22
FHL2	protein_coding	ENSG00000115641	-0,461536636	6,5E-24	1,36E-22
BUB1	protein_coding	ENSG00000169679	-0,679123243	6,65E-24	1,39E-22
XYLT2	protein_coding	ENSG00000015532	-0,57877506	6,98E-24	1,46E-22
ZFP36L2	protein_coding	ENSG00000152518	-0,595545571	7,6E-24	1,59E-22
SCYL2	protein_coding	ENSG00000136021	0,440831791	7,92E-24	1,65E-22
GPRIN1	protein_coding	ENSG00000169258	-0,820584476	7,94E-24	1,65E-22
SWAP70	protein_coding	ENSG00000133789	0,434595497	8,19E-24	1,7E-22
FUBP1	protein_coding	ENSG00000162613	-0,511292749	8,42E-24	1,75E-22

TBC1D14	protein_coding	ENSG00000132405	-0,581538835	8,46E-24	1,76E-22
HCG20	lincRNA	ENSG00000228022	4,336810716	8,83E-24	1,83E-22
DNASE1	protein_coding	ENSG00000213918	1,1753128	8,85E-24	1,83E-22
HSPB8	protein_coding	ENSG00000152137	2,364454243	9,16E-24	1,9E-22
MTMR12	protein_coding	ENSG00000150712	-0,479840981	9,44E-24	1,95E-22
TMPO-AS1	antisense	ENSG00000257167	-0,673955652	1E-23	2,07E-22
BIRC5	protein_coding	ENSG00000089685	-0,678068499	1,06E-23	2,18E-22
ARSD	protein_coding	ENSG00000006756	0,966690763	1,08E-23	2,22E-22
SLC1A4	protein_coding	ENSG00000115902	0,625624433	1,08E-23	2,22E-22
UBR1	protein_coding	ENSG00000159459	0,45045989	1,08E-23	2,22E-22
CDK2	protein_coding	ENSG00000123374	-0,569240251	1,1E-23	2,27E-22
ZFPM2-AS1	processed_transcript	ENSG00000251003	0,819712645	1,14E-23	2,34E-22
ZFP36	protein_coding	ENSG00000128016	1,27280696	1,16E-23	2,37E-22
PLEKHO2	protein_coding	ENSG00000241839	0,838651324	1,17E-23	2,4E-22
RSAD1	protein_coding	ENSG00000136444	-0,52380634	1,17E-23	2,4E-22
MBOAT1	protein_coding	ENSG00000172197	-0,928917964	1,21E-23	2,47E-22
LMO7-AS1	antisense	ENSG00000261105	1,599338535	1,28E-23	2,61E-22
MATN2	protein_coding	ENSG00000132561	-0,428059581	1,33E-23	2,72E-22
PIP5K1A	protein_coding	ENSG00000143398	0,370351943	1,37E-23	2,79E-22
CRYBG2	protein_coding	ENSG00000176092	1,927705654	1,68E-23	3,42E-22
AP000695.2	antisense	ENSG00000233818	1,238549025	1,91E-23	3,89E-22
SRC	protein_coding	ENSG00000197122	0,802884247	1,91E-23	3,89E-22
NEURL1B	protein_coding	ENSG00000214357	-0,990194749	2,06E-23	4,17E-22
CSNK1G1	protein_coding	ENSG00000169118	0,615008996	2,07E-23	4,2E-22
TSPYL2	protein_coding	ENSG00000184205	0,842986221	2,1E-23	4,25E-22
CIDECP1	transcribed_unprocesse	ENSG00000186162	1,096550186	2,11E-23	4,27E-22
SUCO	protein_coding	ENSG00000094975	0,456096328	2,19E-23	4,43E-22
FAM13B	protein_coding	ENSG00000031003	0,596824602	2,31E-23	4,66E-22
MGAM	protein_coding	ENSG00000257335	1,140547312	2,32E-23	4,69E-22
SLC22A15	protein_coding	ENSG00000163393	0,833668362	2,35E-23	4,74E-22
POLE3	protein_coding	ENSG00000148229	-0,481709883	2,87E-23	5,79E-22
P4HB	protein_coding	ENSG00000185624	0,514075098	2,88E-23	5,8E-22
C18orf25	protein_coding	ENSG00000152242	0,412958856	2,93E-23	5,88E-22
APLP2	protein_coding	ENSG00000084234	0,395624209	2,94E-23	5,9E-22
AP1S1	protein_coding	ENSG00000106367	-0,544809582	3,02E-23	6,05E-22
MSH6	protein_coding	ENSG00000116062	-0,757265759	3,18E-23	6,38E-22
SEC24A	protein_coding	ENSG00000113615	0,57922702	3,3E-23	6,61E-22
AC104695.2	antisense	ENSG00000229951	5,301424653	3,38E-23	6,77E-22
FRRS1	protein_coding	ENSG00000156869	1,013550787	3,5E-23	6,99E-22
TIGD7	protein_coding	ENSG00000140993	-0,827242207	3,95E-23	7,89E-22

CCSER2	protein_coding	ENSG00000107771	0,485217608	4,06E-23	8,11E-22
SAMD12	protein_coding	ENSG00000177570	0,739744017	4,13E-23	8,24E-22
NCOA7	protein_coding	ENSG00000111912	0,682770507	4,39E-23	8,74E-22
PIPOX	protein_coding	ENSG00000179761	2,37600566	4,48E-23	8,9E-22
APPBP2	protein_coding	ENSG00000062725	0,329098102	4,59E-23	9,13E-22
CCAR2	protein_coding	ENSG00000158941	-0,499470152	4,82E-23	9,57E-22
UTP18	protein_coding	ENSG00000011260	-0,599683033	4,83E-23	9,57E-22
SMARCD2	protein_coding	ENSG00000108604	-0,531841151	4,87E-23	9,65E-22
F8	protein_coding	ENSG00000185010	0,876606421	4,98E-23	9,86E-22
HMGN4	protein_coding	ENSG00000182952	-0,534047343	5,04E-23	9,97E-22
DMD	protein_coding	ENSG00000198947	0,53814947	5,25E-23	1,04E-21
C4BPAP1	unprocessed_pseudoge	ENSG00000224462	1,990431274	5,42E-23	1,07E-21
ARID4B	protein_coding	ENSG00000054267	0,582191288	5,51E-23	1,08E-21
COPA	protein_coding	ENSG00000122218	0,319503093	5,49E-23	1,08E-21
PIF1	protein_coding	ENSG00000140451	-0,884304313	5,48E-23	1,08E-21
SPAG5	protein_coding	ENSG00000076382	-0,549146616	5,97E-23	1,17E-21
TTK	protein_coding	ENSG00000112742	-0,870169932	6,13E-23	1,2E-21
RAB21	protein_coding	ENSG00000080371	0,486310453	6,17E-23	1,21E-21
TTLL4	protein_coding	ENSG00000135912	-0,697368903	6,16E-23	1,21E-21
RSPH3	protein_coding	ENSG00000130363	0,916717591	6,24E-23	1,22E-21
TNFSF4	protein_coding	ENSG00000117586	2,920140655	6,38E-23	1,25E-21
PDK1	protein_coding	ENSG00000152256	-0,698105401	6,52E-23	1,28E-21
RIOX1	protein_coding	ENSG00000170468	-0,639804791	6,73E-23	1,31E-21
C17orf58	protein_coding	ENSG00000186665	-0,716095183	6,7E-23	1,31E-21
CBX4	protein_coding	ENSG00000141582	0,745206049	7,14E-23	1,39E-21
WDR66	protein_coding	ENSG00000158023	0,668519987	7,31E-23	1,42E-21
ATL3	protein_coding	ENSG00000184743	-0,58131085	7,4E-23	1,44E-21
RAB9A	protein_coding	ENSG00000123595	0,608862295	7,56E-23	1,47E-21
MTO1	protein_coding	ENSG00000135297	0,664538846	7,77E-23	1,51E-21
MMACHC	protein_coding	ENSG00000132763	-0,719978986	7,86E-23	1,52E-21
CXorf38	protein_coding	ENSG00000185753	-0,564549963	7,91E-23	1,53E-21
NUP50	protein_coding	ENSG00000093000	-0,628066593	8E-23	1,55E-21
IL4I1	protein_coding	ENSG00000104951	2,177146606	8,19E-23	1,59E-21
KBTBD6	protein_coding	ENSG00000165572	-0,676141835	8,71E-23	1,68E-21
CCNT2	protein_coding	ENSG00000082258	0,582308797	9,05E-23	1,74E-21
PPP4R3B	protein_coding	ENSG00000275052	0,386227394	8,99E-23	1,74E-21
P2RY1	protein_coding	ENSG00000169860	-0,938523729	9,01E-23	1,74E-21
ENPP2	protein_coding	ENSG00000136960	0,964204206	9,17E-23	1,76E-21
CDC7	protein_coding	ENSG00000097046	-0,763959192	9,16E-23	1,76E-21
PCDH9	protein_coding	ENSG00000184226	0,816097051	9,51E-23	1,83E-21

LINC00243	lincRNA	ENSG00000214894	2,274863891	9,9E-23	1,9E-21
ZNF557	protein_coding	ENSG00000130544	0,474358873	1,02E-22	1,96E-21
MCM2	protein_coding	ENSG00000073111	-0,622738736	1,19E-22	2,27E-21
PRMT7	protein_coding	ENSG00000132600	-0,602568909	1,2E-22	2,3E-21
B4GALT5	protein_coding	ENSG00000158470	-0,457426613	1,26E-22	2,41E-21
TARS2	protein_coding	ENSG00000143374	-0,512670941	1,29E-22	2,47E-21
GRM8	protein_coding	ENSG00000179603	3,804890613	1,3E-22	2,49E-21
CKAP2L	protein_coding	ENSG00000169607	-0,74600055	1,33E-22	2,54E-21
NFYA	protein_coding	ENSG00000001167	-0,554874275	1,34E-22	2,55E-21
NFE2L2	protein_coding	ENSG00000116044	0,504852995	1,35E-22	2,58E-21
DKC1	protein_coding	ENSG00000130826	-0,584284049	1,36E-22	2,59E-21
C5orf30	protein_coding	ENSG00000181751	-0,566689947	1,38E-22	2,62E-21
HMGB3	protein_coding	ENSG00000029993	-0,640072183	1,42E-22	2,69E-21
AC092435.1	lincRNA	ENSG00000248346	2,296842367	1,54E-22	2,92E-21
CAMKK2	protein_coding	ENSG00000110931	-0,542794699	1,64E-22	3,11E-21
NCAPG	protein_coding	ENSG00000109805	-0,652672565	1,69E-22	3,2E-21
PHLDA1	protein_coding	ENSG00000139289	-0,341423751	1,7E-22	3,21E-21
TLCD2	protein_coding	ENSG00000185561	1,487768366	1,81E-22	3,43E-21
TOX4	protein_coding	ENSG00000092203	0,445151447	1,81E-22	3,43E-21
GBP2	protein_coding	ENSG00000162645	5,003039607	1,91E-22	3,6E-21
CYP4F3	protein_coding	ENSG00000186529	3,420223031	1,93E-22	3,64E-21
ZMAT3	protein_coding	ENSG00000172667	0,55745965	1,96E-22	3,69E-21
PEAK1	protein_coding	ENSG00000173517	0,601172685	2,12E-22	3,99E-21
RMI2	protein_coding	ENSG00000175643	-1,004276761	2,25E-22	4,23E-21
PANK2	protein_coding	ENSG00000125779	0,52156285	2,37E-22	4,45E-21
TMEM140	protein_coding	ENSG00000146859	3,350922479	2,59E-22	4,87E-21
URB2	protein_coding	ENSG00000135763	-0,544185666	2,64E-22	4,94E-21
JUND	protein_coding	ENSG00000130522	0,984222381	2,84E-22	5,32E-21
ETS2	protein_coding	ENSG00000157557	0,723547261	2,91E-22	5,45E-21
GTF2B	protein_coding	ENSG00000137947	0,760007428	3,26E-22	6,09E-21
OSBPL10	protein_coding	ENSG00000144645	0,518222879	3,3E-22	6,17E-21
WDR3	protein_coding	ENSG00000065183	-0,583984822	3,58E-22	6,68E-21
AC124276.1	lincRNA	ENSG00000254991	1,645378329	3,78E-22	7,04E-21
PTCHD4	protein_coding	ENSG00000244694	0,737385023	3,86E-22	7,2E-21
CCDC12	protein_coding	ENSG00000160799	-0,663891509	3,88E-22	7,22E-21
TSPAN14	protein_coding	ENSG00000108219	-0,488964537	4E-22	7,44E-21
SLC20A1	protein_coding	ENSG00000144136	-0,293450709	4,1E-22	7,62E-21
MYH16	transcribed_unitary_pse	ENSG00000002079	1,242242884	4,14E-22	7,68E-21
RBM12B	protein_coding	ENSG00000183808	-0,659830553	4,16E-22	7,71E-21
TNF	protein_coding	ENSG00000232810	8,399873319	4,19E-22	7,76E-21

LINC01943	lincRNA	ENSG00000280721	2,5735659	4,2E-22	7,78E-21
SMO	protein_coding	ENSG00000128602	-0,596501086	4,59E-22	8,49E-21
LURAP1L	protein_coding	ENSG00000153714	1,244015397	4,66E-22	8,62E-21
CCDC77	protein_coding	ENSG00000120647	-0,672387408	4,67E-22	8,62E-21
ITGA6	protein_coding	ENSG00000091409	-0,60416759	4,69E-22	8,65E-21
ZBED8	protein_coding	ENSG00000221886	-1,07023153	4,75E-22	8,75E-21
FKBPL	protein_coding	ENSG00000204315	-0,717898114	4,98E-22	9,16E-21
DARS2	protein_coding	ENSG00000117593	-0,442713148	5,01E-22	9,21E-21
TUBB2A	protein_coding	ENSG00000137267	0,813063593	5,16E-22	9,48E-21
PBX3	protein_coding	ENSG00000167081	-0,476093301	5,47E-22	1E-20
DHRS13	protein_coding	ENSG00000167536	-0,995072653	6,31E-22	1,16E-20
NR2F1-AS1	antisense	ENSG00000237187	1,031206712	6,74E-22	1,24E-20
DAG1	protein_coding	ENSG00000173402	-0,584532787	7,2E-22	1,32E-20
BTBD3	protein_coding	ENSG00000132640	-0,521415765	7,36E-22	1,35E-20
NUP107	protein_coding	ENSG00000111581	-0,469250497	8,11E-22	1,48E-20
CGRRF1	protein_coding	ENSG00000100532	0,779200153	8,72E-22	1,59E-20
ZNF318	protein_coding	ENSG00000171467	-0,661395184	8,72E-22	1,59E-20
TSLP	protein_coding	ENSG00000145777	1,702222903	9,17E-22	1,67E-20
SH2D4A	protein_coding	ENSG00000104611	-0,588278049	9,33E-22	1,7E-20
AXIN1	protein_coding	ENSG00000103126	-0,610295202	9,51E-22	1,73E-20
BCAT2	protein_coding	ENSG00000105552	-0,5340983	9,86E-22	1,79E-20
PLA2G4C	protein_coding	ENSG00000105499	1,103149859	1,02E-21	1,86E-20
WDR6	protein_coding	ENSG00000178252	-0,681741031	1,03E-21	1,86E-20
PDCD6	protein_coding	ENSG00000249915	-0,454495161	1,04E-21	1,88E-20
SLMAP	protein_coding	ENSG00000163681	0,391264405	1,05E-21	1,91E-20
CCL3L1	protein_coding	ENSG00000276085	1,864105034	1,07E-21	1,94E-20
FDXACB1	protein_coding	ENSG00000255561	-1,161824151	1,09E-21	1,97E-20
CCT6P1	transcribed_unprocesse	ENSG00000228409	1,17897599	1,15E-21	2,07E-20
PGAM5	protein_coding	ENSG00000247077	-0,456867874	1,17E-21	2,1E-20
AC010168.2	sense_overlapping	ENSG00000261324	1,771201405	1,3E-21	2,35E-20
VPS37A	protein_coding	ENSG00000155975	0,503615964	1,35E-21	2,42E-20
CEACAM19	protein_coding	ENSG00000186567	2,028955951	1,41E-21	2,55E-20
SYNJ2	protein_coding	ENSG00000078269	0,628742835	1,43E-21	2,58E-20
LYST	protein_coding	ENSG00000143669	0,725592585	1,63E-21	2,92E-20
MYOSLID	lincRNA	ENSG00000229647	0,683132632	1,66E-21	2,98E-20
GPSM2	protein_coding	ENSG00000121957	-0,625654657	1,69E-21	3,04E-20
EIF4B	protein_coding	ENSG00000063046	-0,36657684	1,71E-21	3,07E-20
PLEKHA2	protein_coding	ENSG00000169499	-0,359345488	1,75E-21	3,14E-20
ZFP36L1	protein_coding	ENSG00000185650	-0,51969741	1,79E-21	3,2E-20
CSTF2T	protein_coding	ENSG00000177613	-0,50166735	2,12E-21	3,79E-20

ZC3H6	protein_coding	ENSG00000188177	0,863077004	2,12E-21	3,8E-20
TUBA1B	protein_coding	ENSG00000123416	-0,502306569	2,2E-21	3,93E-20
TXNRD1	protein_coding	ENSG00000198431	-0,518048198	2,21E-21	3,95E-20
DCAF4	protein_coding	ENSG00000119599	-0,529874144	2,28E-21	4,07E-20
CNOT4	protein_coding	ENSG00000080802	0,602442846	2,31E-21	4,11E-20
ELF1	protein_coding	ENSG00000120690	0,60794928	2,32E-21	4,12E-20
PMS2	protein_coding	ENSG00000122512	-0,547035992	2,33E-21	4,15E-20
EIF4A1	protein_coding	ENSG00000161960	-1,258144528	2,4E-21	4,27E-20
UBE2T	protein_coding	ENSG00000077152	-0,674474426	2,55E-21	4,52E-20
CLIC2	protein_coding	ENSG00000155962	3,367474839	2,68E-21	4,74E-20
C2CD2L	protein_coding	ENSG00000172375	0,818949348	2,67E-21	4,74E-20
AP1M1	protein_coding	ENSG00000072958	-0,497971763	2,68E-21	4,74E-20
BAG2	protein_coding	ENSG00000112208	-0,630125153	2,72E-21	4,8E-20
PIK3AP1	protein_coding	ENSG00000155629	2,366718557	2,84E-21	5,02E-20
CRABP2	protein_coding	ENSG00000143320	2,192627452	2,86E-21	5,05E-20
TRAPPC4	protein_coding	ENSG00000196655	-0,597706395	2,91E-21	5,14E-20
SDCCAG8	protein_coding	ENSG00000054282	0,441718242	2,99E-21	5,27E-20
BATF2	protein_coding	ENSG00000168062	1,36305503	3,17E-21	5,58E-20
FBXO11	protein_coding	ENSG00000138081	0,552478613	3,28E-21	5,77E-20
ARF4	protein_coding	ENSG00000168374	0,444876186	3,29E-21	5,78E-20
LINC01828	lincRNA	ENSG00000235885	3,225606691	3,3E-21	5,8E-20
WHAMM	protein_coding	ENSG00000156232	0,65754418	3,36E-21	5,9E-20
BZW2	protein_coding	ENSG00000136261	-0,456188324	3,41E-21	5,98E-20
IER5L	protein_coding	ENSG00000188483	1,101974935	3,6E-21	6,31E-20
PXMP4	protein_coding	ENSG00000101417	-0,658605384	3,67E-21	6,42E-20
FANCG	protein_coding	ENSG00000221829	-0,562969262	3,68E-21	6,44E-20
SDC1	protein_coding	ENSG00000115884	-0,519341032	3,71E-21	6,48E-20
MCM5	protein_coding	ENSG00000100297	-0,479650512	3,72E-21	6,5E-20
AC104461.1	processed_transcript	ENSG00000230623	0,727445248	4,15E-21	7,24E-20
CDPF1	protein_coding	ENSG00000205643	-0,874285472	4,21E-21	7,34E-20
CEP97	protein_coding	ENSG00000182504	-0,624514995	4,22E-21	7,35E-20
MATN1-AS1	antisense	ENSG00000186056	1,758949178	4,46E-21	7,77E-20
SNORD12C	snoRNA	ENSG00000209042	-1,872337401	4,72E-21	8,2E-20
H3F3B	protein_coding	ENSG00000132475	0,305670397	4,9E-21	8,5E-20
AC103740.2	lincRNA	ENSG00000259727	1,971052443	5,49E-21	9,53E-20
IGFBP4	protein_coding	ENSG00000141753	-0,532795111	5,6E-21	9,7E-20
C1QTNF1	protein_coding	ENSG00000173918	4,476076401	5,71E-21	9,89E-20
KAT5	protein_coding	ENSG00000172977	-0,497458594	5,82E-21	1,01E-19
SLC12A6	protein_coding	ENSG00000140199	0,583417834	6,04E-21	1,04E-19
NEB	protein_coding	ENSG00000183091	3,069959608	6,21E-21	1,07E-19

ARHGEF2	protein_coding	ENSG00000116584	0,535584199	6,21E-21	1,07E-19
ELP6	protein_coding	ENSG00000163832	-0,481247827	6,29E-21	1,09E-19
NSMCE4A	protein_coding	ENSG00000107672	-0,562810051	6,44E-21	1,11E-19
ATP6V1E1	protein_coding	ENSG00000131100	0,476502097	6,49E-21	1,12E-19
CTBP1-DT	antisense	ENSG00000196810	-0,618663159	6,62E-21	1,14E-19
DUBR	lincRNA	ENSG00000243701	-0,615682366	6,86E-21	1,18E-19
SLIT2	protein_coding	ENSG00000145147	1,165860433	7,15E-21	1,23E-19
TTC7A	protein_coding	ENSG00000068724	0,678027494	7,27E-21	1,25E-19
C4orf46	protein_coding	ENSG00000205208	-0,729094058	7,51E-21	1,29E-19
RRP1	protein_coding	ENSG00000160214	-0,493220716	7,71E-21	1,32E-19
PRR11	protein_coding	ENSG00000068489	-0,539768092	8,7E-21	1,49E-19
CHMP7	protein_coding	ENSG00000147457	-0,399944924	8,94E-21	1,53E-19
CXCL11	protein_coding	ENSG00000169248	7,60538358	9,14E-21	1,56E-19
TTI1	protein_coding	ENSG00000101407	-0,465313563	9,28E-21	1,58E-19
HOXC13-AS	antisense	ENSG00000249641	2,386498859	9,65E-21	1,64E-19
ZNF281	protein_coding	ENSG00000162702	0,473424159	9,61E-21	1,64E-19
CD82	protein_coding	ENSG00000085117	0,913630084	1,04E-20	1,77E-19
NRP1	protein_coding	ENSG00000099250	0,449644634	1,04E-20	1,77E-19
MANCR	lincRNA	ENSG00000231298	-0,862116906	1,06E-20	1,79E-19
SAMD9L	protein_coding	ENSG00000177409	1,107751867	1,08E-20	1,83E-19
RETSAT	protein_coding	ENSG00000042445	0,529641606	1,08E-20	1,83E-19
SLC25A45	protein_coding	ENSG00000162241	1,121144409	1,1E-20	1,87E-19
DAW1	protein_coding	ENSG00000123977	0,781289	1,11E-20	1,87E-19
C1orf112	protein_coding	ENSG00000000460	-0,533535197	1,1E-20	1,87E-19
ZNF14	protein_coding	ENSG00000105708	1,246267753	1,11E-20	1,88E-19
COA4	protein_coding	ENSG00000181924	-0,68264574	1,12E-20	1,9E-19
CTPS1	protein_coding	ENSG00000171793	-0,493580787	1,13E-20	1,91E-19
MOSPD1	protein_coding	ENSG00000101928	0,588109336	1,15E-20	1,93E-19
EXTL3	protein_coding	ENSG00000012232	-0,635970159	1,14E-20	1,93E-19
RCBTB2	protein_coding	ENSG00000136161	0,988007067	1,16E-20	1,96E-19
SLC2A13	protein_coding	ENSG00000151229	0,896131374	1,18E-20	1,98E-19
NUPR1	protein_coding	ENSG00000176046	3,768268741	1,2E-20	2,01E-19
MAK	protein_coding	ENSG00000111837	2,618298107	1,21E-20	2,03E-19
GPCPD1	protein_coding	ENSG00000125772	0,632183427	1,29E-20	2,17E-19
HMG20B	protein_coding	ENSG00000064961	-0,469769087	1,36E-20	2,27E-19
ARHGAP42	protein_coding	ENSG00000165895	0,697286971	1,55E-20	2,6E-19
MT1E	protein_coding	ENSG00000169715	-0,469380879	1,57E-20	2,63E-19
SLBP	protein_coding	ENSG00000163950	-0,596522172	1,62E-20	2,7E-19
FBXO34	protein_coding	ENSG00000178974	-0,429687432	1,65E-20	2,76E-19
XPO1	protein_coding	ENSG00000082898	-0,526723372	1,85E-20	3,08E-19

LEKR1	protein_coding	ENSG00000197980	1,384067968	1,86E-20	3,1E-19
METTL2B	protein_coding	ENSG00000165055	-0,519514913	1,91E-20	3,19E-19
EGOT	lincRNA	ENSG00000235947	5,368336128	1,93E-20	3,23E-19
LINC02310	lincRNA	ENSG00000258808	1,766034158	2,02E-20	3,36E-19
LINC01111	lincRNA	ENSG00000254300	3,686300866	2,03E-20	3,38E-19
CCDC50	protein_coding	ENSG00000152492	0,391277277	2,06E-20	3,43E-19
INTS2	protein_coding	ENSG00000108506	-0,474849578	2,08E-20	3,46E-19
NEK2	protein_coding	ENSG00000117650	-0,687504813	2,09E-20	3,47E-19
RFFL	protein_coding	ENSG00000092871	0,531032369	2,11E-20	3,51E-19
ENDOD1	protein_coding	ENSG00000149218	-0,628159333	2,21E-20	3,67E-19
NRBF2	protein_coding	ENSG00000148572	0,57548039	2,34E-20	3,87E-19
GNL3	protein_coding	ENSG00000163938	-0,607319708	2,34E-20	3,87E-19
SPATA18	protein_coding	ENSG00000163071	0,874941216	2,37E-20	3,92E-19
LINC00513	lincRNA	ENSG00000233559	2,563651162	2,39E-20	3,95E-19
CHCHD7	protein_coding	ENSG00000170791	0,537460433	2,42E-20	4E-19
TMEM41B	protein_coding	ENSG00000166471	0,560874761	2,48E-20	4,09E-19
EPB41L4B	protein_coding	ENSG00000095203	-0,587475803	2,52E-20	4,15E-19
FANCE	protein_coding	ENSG00000112039	-0,579559209	2,62E-20	4,32E-19
KIAA1217	protein_coding	ENSG00000120549	1,3104718	2,7E-20	4,43E-19
DENND4A	protein_coding	ENSG00000174485	0,482579289	2,7E-20	4,43E-19
CCND1	protein_coding	ENSG00000110092	-0,5010044	2,7E-20	4,43E-19
LGALS8	protein_coding	ENSG00000116977	0,46966408	2,76E-20	4,53E-19
OSGEPL1	protein_coding	ENSG00000128694	-0,727217892	2,78E-20	4,56E-19
ZNF146	protein_coding	ENSG00000167635	-0,592785961	2,83E-20	4,63E-19
ARL6IP1	protein_coding	ENSG00000170540	-0,522154809	2,95E-20	4,83E-19
VNN1	protein_coding	ENSG00000112299	1,062887689	3,24E-20	5,3E-19
SLC22A4	protein_coding	ENSG00000197208	1,027098626	3,3E-20	5,39E-19
BUB3	protein_coding	ENSG00000154473	-0,45472992	3,34E-20	5,46E-19
RICTOR	protein_coding	ENSG00000164327	0,586127319	3,45E-20	5,63E-19
MCM6	protein_coding	ENSG00000076003	-0,673373812	3,51E-20	5,72E-19
INKA2	protein_coding	ENSG00000197852	1,069788428	3,62E-20	5,89E-19
SEMA3A	protein_coding	ENSG00000075213	0,436977536	3,64E-20	5,92E-19
ABCB10	protein_coding	ENSG00000135776	-0,638642064	3,71E-20	6,03E-19
ANP32A	protein_coding	ENSG00000140350	-0,486998057	3,89E-20	6,32E-19
CALM2	protein_coding	ENSG00000143933	-0,859249492	4,32E-20	7,01E-19
IQCN	protein_coding	ENSG00000130518	4,222382551	4,42E-20	7,18E-19
RAPH1	protein_coding	ENSG00000173166	0,704030005	4,47E-20	7,25E-19
UBN2	protein_coding	ENSG00000157741	0,958923278	4,59E-20	7,43E-19
FAM78A	protein_coding	ENSG00000126882	-1,340592171	4,75E-20	7,68E-19
DBN1	protein_coding	ENSG00000113758	-0,436974176	4,93E-20	7,98E-19

AC016596.1	antisense	ENSG00000227908	2,145265748	5,14E-20	8,3E-19
BTN2A1	protein_coding	ENSG00000112763	0,655868921	5,47E-20	8,83E-19
ZNF317	protein_coding	ENSG00000130803	0,483348004	5,93E-20	9,56E-19
PRIM1	protein_coding	ENSG00000198056	-0,727546482	6,03E-20	9,72E-19
IMPDH1P10	processed_pseudogene	ENSG00000232133	2,631861633	6,28E-20	1,01E-18
AADAC	protein_coding	ENSG00000114771	1,740757385	6,25E-20	1,01E-18
AIMP2	protein_coding	ENSG00000106305	-0,516814423	6,33E-20	1,02E-18
ARHGAP11A	protein_coding	ENSG00000198826	-0,817574839	6,41E-20	1,03E-18
HYDIN	protein_coding	ENSG00000157423	1,43365789	6,64E-20	1,07E-18
SEC24D	protein_coding	ENSG00000150961	0,498940703	6,71E-20	1,08E-18
LRFN4	protein_coding	ENSG00000173621	-0,895005308	6,72E-20	1,08E-18
BMP2	protein_coding	ENSG00000125845	1,530964728	7,21E-20	1,15E-18
DERL2	protein_coding	ENSG00000072849	0,520392357	7,47E-20	1,2E-18
SLC2A6	protein_coding	ENSG00000160326	1,273349532	7,75E-20	1,24E-18
TEX2	protein_coding	ENSG00000136478	-0,387731516	8,13E-20	1,3E-18
SERPINE2	protein_coding	ENSG00000135919	0,361116008	8,4E-20	1,34E-18
NKRF	protein_coding	ENSG00000186416	-0,608283082	8,36E-20	1,34E-18
TADA1	protein_coding	ENSG00000152382	-0,751436853	8,9E-20	1,42E-18
VANGL1	protein_coding	ENSG00000173218	-0,660949564	9,09E-20	1,45E-18
VCPIP1	protein_coding	ENSG00000175073	0,561570196	9,22E-20	1,47E-18
NCEH1	protein_coding	ENSG00000144959	-0,400651954	9,49E-20	1,51E-18
AC023906.5	antisense	ENSG00000259712	2,098133237	9,74E-20	1,55E-18
SP1	protein_coding	ENSG00000185591	-0,4278044	9,8E-20	1,56E-18
SENP5	protein_coding	ENSG00000119231	0,294432691	1,01E-19	1,6E-18
LEMD1	protein_coding	ENSG00000186007	1,887591672	1,01E-19	1,61E-18
PVR	protein_coding	ENSG00000073008	0,434053323	1,04E-19	1,65E-18
RNF149	protein_coding	ENSG00000163162	0,439292761	1,06E-19	1,68E-18
TUBGCP3	protein_coding	ENSG00000126216	-0,421666943	1,07E-19	1,69E-18
PHF19	protein_coding	ENSG00000119403	-0,354251701	1,08E-19	1,7E-18
ADPGK	protein_coding	ENSG00000159322	-0,444363079	1,08E-19	1,71E-18
DHODH	protein_coding	ENSG00000102967	-0,599633939	1,1E-19	1,74E-18
PGRMC2	protein_coding	ENSG00000164040	-0,566575072	1,14E-19	1,8E-18
AFF3	protein_coding	ENSG00000144218	0,515947858	1,24E-19	1,96E-18
DGCR8	protein_coding	ENSG00000128191	-0,80494364	1,27E-19	1,99E-18
HLA-E	protein_coding	ENSG00000204592	0,49817634	1,29E-19	2,03E-18
WHRN	protein_coding	ENSG00000095397	1,241009538	1,31E-19	2,05E-18
ACOX1	protein_coding	ENSG00000161533	0,428001776	1,34E-19	2,11E-18
AC010442.1	3prime_overlapping_nc	ENSG00000188242	-0,923292553	1,35E-19	2,12E-18
GARS	protein_coding	ENSG00000106105	0,282247649	1,35E-19	2,12E-18
IL1RAP	protein_coding	ENSG00000196083	0,629641567	1,37E-19	2,15E-18

AC131011.1	antisense	ENSG00000233785	2,199362108	1,38E-19	2,16E-18
HSCB	protein_coding	ENSG00000100209	0,863754288	1,38E-19	2,16E-18
SPOP	protein_coding	ENSG00000121067	0,453000677	1,38E-19	2,16E-18
TMEM63B	protein_coding	ENSG00000137216	0,641011299	1,44E-19	2,25E-18
ZDBF2	protein_coding	ENSG00000204186	0,549337502	1,46E-19	2,28E-18
TSR1	protein_coding	ENSG00000167721	-0,443814865	1,46E-19	2,28E-18
SRSF1	protein_coding	ENSG00000136450	-0,614827144	1,51E-19	2,36E-18
SNRPD1	protein_coding	ENSG00000167088	-0,783692374	1,58E-19	2,46E-18
RFX3	protein_coding	ENSG00000080298	0,909723545	1,58E-19	2,47E-18
FUT11	protein_coding	ENSG00000196968	-0,588032124	1,59E-19	2,47E-18
FGF2	protein_coding	ENSG00000138685	0,551417594	1,71E-19	2,65E-18
H2AFZ	protein_coding	ENSG00000164032	-0,617357769	1,78E-19	2,76E-18
EXD2	protein_coding	ENSG00000081177	-0,590447014	1,83E-19	2,83E-18
CCNB3	protein_coding	ENSG00000147082	0,778622822	1,85E-19	2,87E-18
SCD5	protein_coding	ENSG00000145284	-0,379460558	1,87E-19	2,89E-18
TP53I3	protein_coding	ENSG00000115129	0,728126704	1,91E-19	2,96E-18
VPS8	protein_coding	ENSG00000156931	0,393380855	1,94E-19	3E-18
POPDC2	protein_coding	ENSG00000121577	3,002407906	2E-19	3,1E-18
PHEX	protein_coding	ENSG00000102174	1,128542565	2,04E-19	3,16E-18
CCDC174	protein_coding	ENSG00000154781	0,633505542	2,09E-19	3,22E-18
KCTD9	protein_coding	ENSG00000104756	0,442611863	2,15E-19	3,32E-18
TMEM250	protein_coding	ENSG00000238227	-0,692671113	2,16E-19	3,33E-18
CTDSPL	protein_coding	ENSG00000144677	-0,691148277	2,18E-19	3,36E-18
RIPK4	protein_coding	ENSG00000183421	1,262966206	2,2E-19	3,39E-18
NSD2	protein_coding	ENSG00000109685	-0,540779148	2,33E-19	3,58E-18
RBM15B	protein_coding	ENSG00000259956	-0,450050229	2,36E-19	3,63E-18
TAB1	protein_coding	ENSG00000100324	-0,567226238	2,41E-19	3,7E-18
H1FX	protein_coding	ENSG00000184897	-0,687951363	2,43E-19	3,73E-18
DEPDC7	protein_coding	ENSG00000121690	0,562194443	2,45E-19	3,76E-18
DEPDC1B	protein_coding	ENSG00000035499	-0,735169627	2,47E-19	3,79E-18
CALM3	protein_coding	ENSG00000160014	-0,334022729	2,65E-19	4,05E-18
RBMS3	protein_coding	ENSG00000144642	0,57545204	2,69E-19	4,12E-18
PSG4	protein_coding	ENSG00000243137	2,712318075	2,7E-19	4,13E-18
SAMD1	protein_coding	ENSG00000141858	-0,551166426	2,77E-19	4,24E-18
NUF2	protein_coding	ENSG00000143228	-0,778841164	2,8E-19	4,28E-18
CHAF1A	protein_coding	ENSG00000167670	-0,611908223	2,85E-19	4,36E-18
CEP95	protein_coding	ENSG00000258890	0,536877122	2,88E-19	4,39E-18
TINAGL1	protein_coding	ENSG00000142910	3,62108792	3,09E-19	4,71E-18
AL121987.2	antisense	ENSG00000227741	3,173593418	3,12E-19	4,75E-18
SUMO3	protein_coding	ENSG00000184900	-0,405907893	3,13E-19	4,76E-18

SNN	protein_coding	ENSG00000184602	-0,923526466	3,14E-19	4,78E-18
RNF187	protein_coding	ENSG00000168159	-0,520477973	3,17E-19	4,81E-18
ZNF691	protein_coding	ENSG00000164011	-0,936088826	3,31E-19	5,03E-18
APEX2	protein_coding	ENSG00000169188	-0,537993706	3,47E-19	5,27E-18
TEDC2	protein_coding	ENSG00000162062	-0,783999354	3,6E-19	5,46E-18
HIP1R	protein_coding	ENSG00000130787	0,882585994	3,62E-19	5,48E-18
BFSP1	protein_coding	ENSG00000125864	1,938936751	3,65E-19	5,52E-18
TMPO	protein_coding	ENSG00000120802	-0,622488143	3,7E-19	5,6E-18
TAOK3	protein_coding	ENSG00000135090	0,3851408	3,76E-19	5,68E-18
POGK	protein_coding	ENSG00000143157	-0,473081809	3,81E-19	5,76E-18
MFSD11	protein_coding	ENSG00000092931	0,62683001	3,85E-19	5,8E-18
COL7A1	protein_coding	ENSG00000114270	1,65920013	3,89E-19	5,86E-18
ERLIN1	protein_coding	ENSG00000107566	-0,481005895	3,88E-19	5,86E-18
FBXO45	protein_coding	ENSG00000174013	-0,533712882	3,89E-19	5,86E-18
SLC29A1	protein_coding	ENSG00000112759	-0,448089503	3,92E-19	5,9E-18
PM20D2	protein_coding	ENSG00000146281	-0,661376639	4,01E-19	6,03E-18
VIM	protein_coding	ENSG00000026025	-0,285288341	4,08E-19	6,14E-18
TPX2	protein_coding	ENSG00000088325	-0,475275182	4,14E-19	6,22E-18
PAPPA	protein_coding	ENSG00000182752	0,611736476	4,15E-19	6,23E-18
CCDC102B	protein_coding	ENSG00000150636	1,347158805	4,19E-19	6,28E-18
TRIB3	protein_coding	ENSG00000101255	0,695229448	4,27E-19	6,39E-18
LCMT2	protein_coding	ENSG00000168806	-0,597476731	4,26E-19	6,39E-18
PCNX2	protein_coding	ENSG00000135749	0,548290081	4,35E-19	6,51E-18
OTUD1	protein_coding	ENSG00000165312	0,912856424	4,36E-19	6,52E-18
ADAMTS4	protein_coding	ENSG00000158859	9,471475421	4,58E-19	6,85E-18
SEMA4A	protein_coding	ENSG00000196189	2,95041335	4,69E-19	7E-18
ALDH1A3	protein_coding	ENSG00000184254	-0,449027986	4,72E-19	7,03E-18
AOC2	protein_coding	ENSG00000131480	1,393261425	4,76E-19	7,1E-18
MCRS1	protein_coding	ENSG00000187778	-0,457622725	5,21E-19	7,76E-18
SPIN4	protein_coding	ENSG00000186767	-0,726225351	5,25E-19	7,81E-18
IMP3	protein_coding	ENSG00000177971	-0,632687486	5,54E-19	8,24E-18
KLLN	protein_coding	ENSG00000227268	1,353158942	5,57E-19	8,28E-18
NEK6	protein_coding	ENSG00000119408	-0,377474006	6,08E-19	9,02E-18
FBXO43	protein_coding	ENSG00000156509	-1,00297845	6,26E-19	9,29E-18
C8orf33	protein_coding	ENSG00000182307	-0,494802291	6,36E-19	9,44E-18
ZBTB22	protein_coding	ENSG00000236104	-0,630717281	6,41E-19	9,51E-18
SEMA5A	protein_coding	ENSG00000112902	1,437504451	6,81E-19	1,01E-17
LST1	protein_coding	ENSG00000204482	4,332810456	6,89E-19	1,02E-17
XPC	protein_coding	ENSG00000154767	-0,418571657	7E-19	1,03E-17
ZNF383	protein_coding	ENSG00000188283	0,715732546	7,04E-19	1,04E-17

SNHG14	processed_transcript	ENSG00000224078	0,975876056	7,18E-19	1,06E-17
LINC00592	lincRNA	ENSG00000258279	1,882585228	7,31E-19	1,08E-17
USP13	protein_coding	ENSG00000058056	-0,628073593	7,29E-19	1,08E-17
TIMELESS	protein_coding	ENSG00000111602	-0,587230628	7,62E-19	1,12E-17
SDCBP2	protein_coding	ENSG00000125775	2,441099306	7,87E-19	1,16E-17
PARP9	protein_coding	ENSG00000138496	0,620512079	8E-19	1,18E-17
INO80C	protein_coding	ENSG00000153391	0,728205147	8,11E-19	1,19E-17
AFF4	protein_coding	ENSG00000072364	0,594628472	8,4E-19	1,23E-17
RPL7L1	protein_coding	ENSG00000146223	-0,375970036	8,36E-19	1,23E-17
PVT1	lincRNA	ENSG00000249859	0,628294709	8,49E-19	1,25E-17
SHOC2	protein_coding	ENSG00000108061	0,435576091	8,49E-19	1,25E-17
WDR5B	protein_coding	ENSG00000196981	-0,72878058	8,51E-19	1,25E-17
ZNF561	protein_coding	ENSG00000171469	0,49199958	8,65E-19	1,27E-17
MAP4K4	protein_coding	ENSG00000071054	0,441539307	8,68E-19	1,27E-17
CANT1	protein_coding	ENSG00000171302	-0,406367378	8,94E-19	1,31E-17
HEPACAM	protein_coding	ENSG00000165478	4,354191352	9,09E-19	1,33E-17
ANKRD54	protein_coding	ENSG00000100124	-0,516837921	9,12E-19	1,33E-17
SYT11	protein_coding	ENSG00000132718	1,373534914	9,41E-19	1,37E-17
RABL3	protein_coding	ENSG00000144840	-0,651499585	9,64E-19	1,41E-17
LINC02605	lincRNA	ENSG00000261618	4,102558718	9,84E-19	1,43E-17
ZNF462	protein_coding	ENSG00000148143	0,796118991	9,87E-19	1,43E-17
POMT1	protein_coding	ENSG00000130714	-0,441738336	9,82E-19	1,43E-17
KIF11	protein_coding	ENSG00000138160	-0,64706903	9,79E-19	1,43E-17
DCBLD2	protein_coding	ENSG00000057019	0,330912465	1,01E-18	1,47E-17
LSM2	protein_coding	ENSG00000204392	-0,671142901	1,01E-18	1,47E-17
GINS3	protein_coding	ENSG00000181938	-0,736218704	1,05E-18	1,53E-17
AKAP17A	protein_coding	ENSG00000197976	0,734330988	1,06E-18	1,54E-17
BBC3	protein_coding	ENSG00000105327	1,033871883	1,09E-18	1,57E-17
DOCK2	protein_coding	ENSG00000134516	0,975346689	1,13E-18	1,63E-17
CCDC122	protein_coding	ENSG00000151773	0,763987252	1,13E-18	1,63E-17
RETREG2	protein_coding	ENSG00000144567	-0,420060955	1,14E-18	1,65E-17
UBE2G2	protein_coding	ENSG00000184787	-0,454862679	1,21E-18	1,74E-17
TICRR	protein_coding	ENSG00000140534	-0,708457352	1,21E-18	1,74E-17
ACER2	protein_coding	ENSG00000177076	0,862358849	1,24E-18	1,79E-17
MOSPD2	protein_coding	ENSG00000130150	0,508013838	1,26E-18	1,81E-17
FAT2	protein_coding	ENSG00000086570	3,730934329	1,32E-18	1,9E-17
C14orf28	protein_coding	ENSG00000179476	0,773224577	1,34E-18	1,92E-17
FJX1	protein_coding	ENSG00000179431	-0,53940853	1,37E-18	1,97E-17
YPEL2	protein_coding	ENSG00000175155	0,887993275	1,39E-18	2E-17
SLC27A4	protein_coding	ENSG00000167114	-0,648436429	1,4E-18	2,01E-17

DNAH3	protein_coding	ENSG00000158486	2,968836571	1,47E-18	2,11E-17
GALR2	protein_coding	ENSG00000182687	3,639774736	1,48E-18	2,12E-17
FRMD4B	protein_coding	ENSG00000114541	0,862076103	1,52E-18	2,17E-17
ABHD17C	protein_coding	ENSG00000136379	-0,821195963	1,52E-18	2,18E-17
TXNRD2	protein_coding	ENSG00000184470	-0,4886297	1,56E-18	2,23E-17
DPY19L2P1	transcribed_unprocesse	ENSG00000189212	0,57024848	1,67E-18	2,39E-17
CGAS	protein_coding	ENSG00000164430	0,552744357	1,74E-18	2,49E-17
LAPTM4A	protein_coding	ENSG00000068697	0,368722748	1,76E-18	2,51E-17
GPR156	protein_coding	ENSG00000175697	2,615604496	1,78E-18	2,54E-17
TRAP1	protein_coding	ENSG00000126602	-0,368027398	1,84E-18	2,62E-17
SCARF1	protein_coding	ENSG00000074660	2,69303135	1,84E-18	2,63E-17
PPL	protein_coding	ENSG00000118898	4,951464227	1,85E-18	2,64E-17
TM4SF18	protein_coding	ENSG00000163762	0,643330187	1,86E-18	2,65E-17
ST6GAL1	protein_coding	ENSG00000073849	-0,735751551	1,86E-18	2,65E-17
ALPK2	protein_coding	ENSG00000198796	0,435173426	1,92E-18	2,73E-17
KDM4B	protein_coding	ENSG00000127663	0,746745527	1,93E-18	2,74E-17
RBM28	protein_coding	ENSG00000106344	-0,46441409	2,01E-18	2,85E-17
FIGNL1	protein_coding	ENSG00000132436	-0,705633896	2,01E-18	2,85E-17
PLEKHG6	protein_coding	ENSG00000008323	7,755091329	2,13E-18	3,02E-17
SFXN2	protein_coding	ENSG00000156398	-0,55964336	2,29E-18	3,24E-17
SNHG5	processed_transcript	ENSG00000203875	0,557885488	2,31E-18	3,26E-17
PLCL2	protein_coding	ENSG00000154822	0,624562384	2,32E-18	3,29E-17
AL031777.1	processed_pseudogene	ENSG00000217275	3,675631354	2,35E-18	3,32E-17
PGM3	protein_coding	ENSG00000013375	0,35516674	2,37E-18	3,35E-17
CMTM3	protein_coding	ENSG00000140931	-0,512999681	2,41E-18	3,4E-17
GTF2A1	protein_coding	ENSG00000165417	0,523248318	2,48E-18	3,49E-17
NUP155	protein_coding	ENSG00000113569	-0,425772338	2,56E-18	3,61E-17
HLA-A	protein_coding	ENSG00000206503	0,580391254	2,57E-18	3,63E-17
TSPAN2	protein_coding	ENSG00000134198	1,923986751	2,6E-18	3,66E-17
SYVN1	protein_coding	ENSG00000162298	0,875153485	2,67E-18	3,76E-17
NR2C2AP	protein_coding	ENSG00000184162	-0,656065745	2,77E-18	3,89E-17
SND1	protein_coding	ENSG00000197157	0,400212727	2,83E-18	3,97E-17
PTER	protein_coding	ENSG00000165983	0,528899063	2,86E-18	4,01E-17
UMPS	protein_coding	ENSG00000114491	-0,406664127	3E-18	4,21E-17
CHMP4B	protein_coding	ENSG00000101421	0,40735812	3,02E-18	4,23E-17
CIAO1	protein_coding	ENSG00000144021	-0,378762609	3,2E-18	4,48E-17
KRT86	protein_coding	ENSG00000170442	2,463672647	3,28E-18	4,59E-17
FAM126A	protein_coding	ENSG00000122591	0,378543857	3,29E-18	4,6E-17
SLC35B3	protein_coding	ENSG00000124786	0,562420497	3,4E-18	4,75E-17
PRPSAP1	protein_coding	ENSG00000161542	-0,456274502	3,46E-18	4,83E-17

GCC2	protein_coding	ENSG00000135968	0,557700515	3,66E-18	5,11E-17
ARSK	protein_coding	ENSG00000164291	-0,578170505	3,67E-18	5,11E-17
LUZP1	protein_coding	ENSG00000169641	0,416880834	3,69E-18	5,15E-17
TIPARP-AS1	bidirectional_promoter_	ENSG00000243926	1,677441663	3,82E-18	5,32E-17
POLG2	protein_coding	ENSG00000256525	-0,624254169	3,86E-18	5,38E-17
DCAF5	protein_coding	ENSG00000139990	0,495682005	3,93E-18	5,47E-17
NANP	protein_coding	ENSG00000170191	-0,581085874	4,08E-18	5,67E-17
NFE2L3	protein_coding	ENSG00000050344	-0,404228477	4,13E-18	5,73E-17
PITX1	protein_coding	ENSG00000069011	-0,791426065	4,31E-18	5,98E-17
RPUSD4	protein_coding	ENSG00000165526	-0,473855604	4,32E-18	6E-17
AK4	protein_coding	ENSG00000162433	-0,641094251	4,46E-18	6,18E-17
PSG5	protein_coding	ENSG00000204941	0,938331613	4,85E-18	6,73E-17
RFC4	protein_coding	ENSG00000163918	-0,686137203	4,87E-18	6,75E-17
WASF3	protein_coding	ENSG00000132970	-0,54777835	5,22E-18	7,23E-17
ZNF827	protein_coding	ENSG00000151612	0,773370971	5,31E-18	7,35E-17
S100A3	protein_coding	ENSG00000188015	0,99564913	5,39E-18	7,45E-17
ATP6V1D	protein_coding	ENSG00000100554	0,5084561	5,4E-18	7,45E-17
PRKAB1	protein_coding	ENSG00000111725	0,542517409	5,42E-18	7,48E-17
TPP2	protein_coding	ENSG00000134900	0,403853232	5,94E-18	8,2E-17
SNUPN	protein_coding	ENSG00000169371	-0,617729307	6,1E-18	8,41E-17
LSM11	protein_coding	ENSG00000155858	-0,659995189	6,1E-18	8,41E-17
BCL2L11	protein_coding	ENSG00000153094	0,758049118	6,24E-18	8,59E-17
STK38L	protein_coding	ENSG00000211455	0,522876217	6,91E-18	9,5E-17
DAP	protein_coding	ENSG00000112977	0,434439951	7,14E-18	9,81E-17
FBXO28	protein_coding	ENSG00000143756	0,365001883	7,17E-18	9,86E-17
G3BP1	protein_coding	ENSG00000145907	-0,504991578	7,29E-18	1E-16
TG	protein_coding	ENSG00000042832	2,952112895	7,47E-18	1,02E-16
DPY19L2	protein_coding	ENSG00000177990	1,112307083	7,6E-18	1,04E-16
STARD7	protein_coding	ENSG00000084090	-0,4078579	7,63E-18	1,05E-16
HDHD5	protein_coding	ENSG00000069998	-0,594296836	7,68E-18	1,05E-16
TMEM19	protein_coding	ENSG00000139291	-0,510723971	7,74E-18	1,06E-16
CDT1	protein_coding	ENSG00000167513	-0,627742515	7,82E-18	1,07E-16
NUDT3	protein_coding	ENSG00000272325	-0,66393265	7,87E-18	1,08E-16
AP002956.1	protein_coding	ENSG00000076706	1,971096179	8,13E-18	1,11E-16
PPP2CB	protein_coding	ENSG00000104695	0,405041068	8,23E-18	1,12E-16
RNASEH2A	protein_coding	ENSG00000104889	-0,557726326	8,19E-18	1,12E-16
LRRC56	protein_coding	ENSG00000161328	2,417444359	8,33E-18	1,13E-16
IRS1	protein_coding	ENSG00000169047	-0,706609594	8,33E-18	1,13E-16
CTDSP1	protein_coding	ENSG00000144579	-0,606033712	8,39E-18	1,14E-16
SEC22C	protein_coding	ENSG00000093183	-0,343670525	8,87E-18	1,21E-16

GALNT14	protein_coding	ENSG00000158089	-0,514511217	9,35E-18	1,27E-16
ZNF280A	protein_coding	ENSG00000169548	1,325848547	9,6E-18	1,3E-16
ATP8B4	protein_coding	ENSG00000104043	1,867225485	9,84E-18	1,34E-16
NTHL1	protein_coding	ENSG00000065057	-0,810382189	9,96E-18	1,35E-16
DNA2	protein_coding	ENSG00000138346	-0,662065783	1,01E-17	1,37E-16
LPCAT2	protein_coding	ENSG00000087253	0,702426201	1,02E-17	1,38E-16
DCTPP1	protein_coding	ENSG00000179958	-0,684461073	1,02E-17	1,38E-16
FAM53C	protein_coding	ENSG00000120709	0,613635156	1,03E-17	1,4E-16
SFN	protein_coding	ENSG00000175793	-0,459428368	1,04E-17	1,41E-16
MRGBP	protein_coding	ENSG00000101189	-0,364685878	1,06E-17	1,44E-16
NELFCD	protein_coding	ENSG00000101158	-0,345912768	1,09E-17	1,47E-16
AP002851.1	bidirectional_promoter_	ENSG00000283959	1,963004738	1,13E-17	1,52E-16
FAM126B	protein_coding	ENSG00000155744	0,466037377	1,14E-17	1,53E-16
MAGEH1	protein_coding	ENSG00000187601	-0,633129879	1,14E-17	1,53E-16
ZNF585A	protein_coding	ENSG00000196967	1,03084238	1,14E-17	1,54E-16
MARCH5	protein_coding	ENSG00000198060	0,455763451	1,14E-17	1,54E-16
L3MBTL2	protein_coding	ENSG00000100395	-0,58796213	1,16E-17	1,56E-16
POLR1E	protein_coding	ENSG00000137054	-0,42285079	1,17E-17	1,57E-16
NCAPD3	protein_coding	ENSG00000151503	-0,463662009	1,18E-17	1,59E-16
TMEM121	protein_coding	ENSG00000184986	-0,974726108	1,19E-17	1,59E-16
PPME1	protein_coding	ENSG00000214517	0,417783016	1,2E-17	1,6E-16
SNRNP35	protein_coding	ENSG00000184209	-0,744666018	1,19E-17	1,6E-16
CIT	protein_coding	ENSG00000122966	-0,683625421	1,21E-17	1,62E-16
FBXO8	protein_coding	ENSG00000164117	0,606850429	1,22E-17	1,63E-16
SH3TC1	protein_coding	ENSG00000125089	0,886655456	1,29E-17	1,73E-16
ZNHIT6	protein_coding	ENSG00000117174	0,363336063	1,32E-17	1,77E-16
TTLL12	protein_coding	ENSG00000100304	-0,484784154	1,39E-17	1,86E-16
ZFAND3	protein_coding	ENSG00000156639	0,460748477	1,44E-17	1,93E-16
WDHD1	protein_coding	ENSG00000198554	-0,591603663	1,46E-17	1,95E-16
PATL2	protein_coding	ENSG00000229474	3,345640218	1,47E-17	1,96E-16
PIK3CA	protein_coding	ENSG00000121879	0,472350211	1,58E-17	2,1E-16
ROBO4	protein_coding	ENSG00000154133	0,793006444	1,59E-17	2,12E-16
AKAP9	protein_coding	ENSG00000127914	0,63370891	1,65E-17	2,19E-16
CHEK1	protein_coding	ENSG00000149554	-0,488730208	1,72E-17	2,29E-16
DHRS2	protein_coding	ENSG00000100867	1,360265903	1,74E-17	2,31E-16
USP38	protein_coding	ENSG00000170185	0,38860834	1,79E-17	2,37E-16
GALNT6	protein_coding	ENSG00000139629	0,495421679	1,83E-17	2,42E-16
PLEKHA6	protein_coding	ENSG00000143850	3,877465043	1,84E-17	2,44E-16
ERBIN	protein_coding	ENSG00000112851	-0,519564676	1,84E-17	2,44E-16
MAD2L1	protein_coding	ENSG00000164109	-0,764120213	1,86E-17	2,46E-16

TMEM52B	protein_coding	ENSG00000165685	3,19007147	1,91E-17	2,53E-16
MMP19	protein_coding	ENSG00000123342	1,520430844	1,95E-17	2,58E-16
ITPR2	protein_coding	ENSG00000123104	0,56104847	1,98E-17	2,62E-16
GNPTAB	protein_coding	ENSG00000111670	-0,400541963	2E-17	2,65E-16
SPTB	protein_coding	ENSG00000070182	1,099280117	2,02E-17	2,67E-16
DNAJC14	protein_coding	ENSG00000135392	-0,430576266	2,03E-17	2,68E-16
CIITA	protein_coding	ENSG00000179583	-0,88811584	2,1E-17	2,77E-16
PDE4C	protein_coding	ENSG00000105650	2,664033094	2,11E-17	2,78E-16
ZNF530	protein_coding	ENSG00000183647	-0,543031716	2,12E-17	2,79E-16
NECAB1	protein_coding	ENSG00000123119	2,738654631	2,2E-17	2,9E-16
GOLGB1	protein_coding	ENSG00000173230	0,61590984	2,21E-17	2,91E-16
SDE2	protein_coding	ENSG00000143751	0,411577002	2,27E-17	2,98E-16
ZYX	protein_coding	ENSG00000159840	0,659167374	2,29E-17	3,01E-16
AGPAT5	protein_coding	ENSG00000155189	-0,462375041	2,32E-17	3,04E-16
NUP93	protein_coding	ENSG00000102900	-0,331149897	2,33E-17	3,06E-16
PHF21A	protein_coding	ENSG00000135365	0,65127033	2,38E-17	3,12E-16
CASC11	lincRNA	ENSG00000249375	3,972602342	2,41E-17	3,15E-16
ZFYVE1	protein_coding	ENSG00000165861	0,691281306	2,48E-17	3,25E-16
ZNF165	protein_coding	ENSG00000197279	1,246562803	2,54E-17	3,32E-16
TTBK2	protein_coding	ENSG00000128881	0,631762862	2,6E-17	3,41E-16
AZIN1-AS1	antisense	ENSG00000253320	1,712761339	2,65E-17	3,46E-16
ZNF720	protein_coding	ENSG00000197302	0,682920801	2,69E-17	3,51E-16
RNF220	protein_coding	ENSG00000187147	-0,446484012	2,72E-17	3,55E-16
KCTD4	protein_coding	ENSG00000180332	-0,718010811	2,72E-17	3,55E-16
CTSS	protein_coding	ENSG00000163131	2,99137606	2,74E-17	3,57E-16
CPNE5	protein_coding	ENSG00000124772	2,892803424	2,8E-17	3,65E-16
EFCAB2	protein_coding	ENSG00000203666	0,845286159	2,85E-17	3,71E-16
RAD54L	protein_coding	ENSG00000085999	-0,548855273	2,87E-17	3,73E-16
S100PBP	protein_coding	ENSG00000116497	-0,410600547	2,88E-17	3,75E-16
WDR62	protein_coding	ENSG00000075702	-0,569955037	2,91E-17	3,79E-16
CISD3	protein_coding	ENSG00000277972	-0,492377112	2,94E-17	3,82E-16
OS9	protein_coding	ENSG00000135506	0,402317358	2,95E-17	3,83E-16
NSF	protein_coding	ENSG00000073969	0,295794809	2,96E-17	3,84E-16
PUDP	protein_coding	ENSG00000130021	-0,471400891	3,09E-17	4,01E-16
TAF2	protein_coding	ENSG00000064313	0,374005156	3,15E-17	4,08E-16
LBR	protein_coding	ENSG00000143815	-0,587093889	3,17E-17	4,11E-16
MCFD2	protein_coding	ENSG00000180398	0,315791061	3,2E-17	4,14E-16
SHBG	protein_coding	ENSG00000129214	3,327266057	3,3E-17	4,26E-16
PPP5C	protein_coding	ENSG00000011485	-0,417812935	3,34E-17	4,32E-16
TGFBR3	protein_coding	ENSG00000069702	-0,530132213	3,4E-17	4,4E-16

C11orf68	protein_coding	ENSG00000175573	-0,665812654	3,41E-17	4,4E-16
STMN1	protein_coding	ENSG00000117632	-0,563231378	3,44E-17	4,44E-16
RFWD3	protein_coding	ENSG00000168411	-0,415614655	3,52E-17	4,54E-16
MLH3	protein_coding	ENSG00000119684	-0,460597245	3,53E-17	4,54E-16
MTRF1L	protein_coding	ENSG00000112031	0,570907842	3,57E-17	4,6E-16
MAP3K14	protein_coding	ENSG00000006062	0,705345444	3,6E-17	4,64E-16
RNU6ATAC	snRNA	ENSG00000221676	-0,994494181	3,64E-17	4,68E-16
VEGFA	protein_coding	ENSG00000112715	0,659953465	3,91E-17	5,03E-16
AC005865.2	transcribed_unprocesse	ENSG00000250770	1,8961722	3,99E-17	5,12E-16
AUNIP	protein_coding	ENSG00000127423	-0,565206873	4,12E-17	5,29E-16
DDX11	protein_coding	ENSG00000013573	-0,644398545	4,12E-17	5,29E-16
TCEANC2	protein_coding	ENSG00000116205	-0,546671699	4,19E-17	5,37E-16
CAND1	protein_coding	ENSG00000111530	-0,47799495	4,25E-17	5,45E-16
KLF5	protein_coding	ENSG00000102554	0,711666882	4,29E-17	5,49E-16
DDX39A	protein_coding	ENSG00000123136	-0,546092151	4,51E-17	5,77E-16
HIST1H3F	protein_coding	ENSG00000277775	-1,402948939	4,79E-17	6,12E-16
MBNL2	protein_coding	ENSG00000139793	0,424262882	4,82E-17	6,16E-16
SLC8B1	protein_coding	ENSG00000089060	0,664302799	4,96E-17	6,33E-16
TMCC3	protein_coding	ENSG00000057704	0,421587341	5,04E-17	6,43E-16
AC016999.1	antisense	ENSG00000229915	3,659529773	5,12E-17	6,53E-16
FARP2	protein_coding	ENSG00000006607	-0,530716696	5,23E-17	6,66E-16
COPG1	protein_coding	ENSG00000181789	0,323813833	5,4E-17	6,87E-16
TNIP1	protein_coding	ENSG00000145901	0,438014453	5,42E-17	6,89E-16
ARHGEF37	protein_coding	ENSG00000183111	2,235001026	5,52E-17	7,02E-16
POC1A	protein_coding	ENSG00000164087	-0,501018826	5,71E-17	7,26E-16
OGFOD3	protein_coding	ENSG00000181396	-0,613430262	5,86E-17	7,45E-16
SRGAP3	protein_coding	ENSG00000196220	2,723937071	6,07E-17	7,71E-16
RGS2	protein_coding	ENSG00000116741	0,728664767	6,11E-17	7,76E-16
MAPK12	protein_coding	ENSG00000188130	-0,536376095	6,18E-17	7,84E-16
DNAJC17	protein_coding	ENSG00000104129	-0,584562352	6,22E-17	7,89E-16
CHAF1B	protein_coding	ENSG00000159259	-0,519740121	6,55E-17	8,3E-16
NADK2	protein_coding	ENSG00000152620	-0,505858163	6,63E-17	8,39E-16
KLF10	protein_coding	ENSG00000155090	0,429288037	6,64E-17	8,4E-16
HILPDA	protein_coding	ENSG00000135245	-0,915122857	6,74E-17	8,52E-16
AC097359.2	antisense	ENSG00000270194	1,393948581	6,95E-17	8,78E-16
MAN1B1	protein_coding	ENSG00000177239	-0,389227843	7,18E-17	9,07E-16
ACTN2	protein_coding	ENSG00000077522	4,500333561	7,44E-17	9,38E-16
SKIL	protein_coding	ENSG00000136603	0,622892079	7,53E-17	9,5E-16
MYO6	protein_coding	ENSG00000196586	0,415125531	7,53E-17	9,5E-16
ASXL1	protein_coding	ENSG00000171456	-0,491001455	7,76E-17	9,78E-16

BAZ1A	protein_coding	ENSG00000198604	0,425685074	7,77E-17	9,79E-16
SELENOK	protein_coding	ENSG00000113811	0,614460701	7,87E-17	9,9E-16
LSM14B	protein_coding	ENSG00000149657	-0,410763083	7,94E-17	9,99E-16
SORBS1	protein_coding	ENSG00000095637	2,64621238	7,95E-17	1E-15
BIRC3	protein_coding	ENSG00000023445	0,473212493	8,12E-17	1,02E-15
FKBP1A	protein_coding	ENSG00000088832	0,315498863	8,2E-17	1,03E-15
PIK3R4	protein_coding	ENSG00000196455	-0,376977955	8,21E-17	1,03E-15
GFPT1	protein_coding	ENSG00000198380	0,383210908	8,33E-17	1,04E-15
WIPI1	protein_coding	ENSG00000070540	0,496769428	8,48E-17	1,06E-15
AC012377.1	lincRNA	ENSG00000246863	2,06903585	8,57E-17	1,07E-15
PAFAH2	protein_coding	ENSG00000158006	-0,444918833	8,65E-17	1,08E-15
NUP133	protein_coding	ENSG00000069248	-0,311206832	8,86E-17	1,11E-15
RP2	protein_coding	ENSG00000102218	0,576582255	9,05E-17	1,13E-15
HMGXB3	protein_coding	ENSG00000113716	0,51991624	9,02E-17	1,13E-15
NR2C2	protein_coding	ENSG00000177463	-0,525362023	9,04E-17	1,13E-15
PDCD11	protein_coding	ENSG00000148843	-0,540952707	9,29E-17	1,16E-15
RAB2B	protein_coding	ENSG00000129472	0,641676786	9,38E-17	1,17E-15
IARS2	protein_coding	ENSG00000067704	-0,40412061	9,39E-17	1,17E-15
PPP2R5A	protein_coding	ENSG00000066027	-0,374791839	9,47E-17	1,18E-15
DUS2	protein_coding	ENSG00000167264	-0,544270411	9,65E-17	1,2E-15
ACTN1-AS1	antisense	ENSG00000259062	2,75434216	9,7E-17	1,21E-15
ZNF350	protein_coding	ENSG00000256683	0,747976633	1,01E-16	1,25E-15
C9orf40	protein_coding	ENSG00000135045	-0,671003282	1,03E-16	1,28E-15
AGAP2-AS1	antisense	ENSG00000255737	-0,58732084	1,04E-16	1,29E-15
ZBTB33	protein_coding	ENSG00000177485	-0,56289645	1,04E-16	1,29E-15
CBLB	protein_coding	ENSG00000114423	0,634241399	1,06E-16	1,32E-15
WTAP	protein_coding	ENSG00000146457	0,419546248	1,07E-16	1,32E-15
AP000692.2	antisense	ENSG00000273199	-1,712774756	1,08E-16	1,34E-15
NEIL2	protein_coding	ENSG00000154328	-0,480351098	1,08E-16	1,34E-15
LONRF1	protein_coding	ENSG00000154359	-0,829217015	1,09E-16	1,35E-15
GNAL	protein_coding	ENSG00000141404	2,98270324	1,1E-16	1,36E-15
HSPBAP1	protein_coding	ENSG00000169087	0,569977696	1,15E-16	1,42E-15
CTU2	protein_coding	ENSG00000174177	-0,522145957	1,17E-16	1,45E-15
CEACAMP10	transcribed_processed_	ENSG00000241104	1,583743954	1,26E-16	1,56E-15
RAD21	protein_coding	ENSG00000164754	-0,512455707	1,41E-16	1,73E-15
FANCF	protein_coding	ENSG00000183161	-0,738383472	1,41E-16	1,74E-15
NUSAP1	protein_coding	ENSG00000137804	-0,63980587	1,48E-16	1,82E-15
KLHL23	protein_coding	ENSG00000213160	-0,712472048	1,49E-16	1,83E-15
KLHL23	protein_coding	ENSG00000213160	-0,712472048	1,49E-16	1,83E-15
C6orf222	protein_coding	ENSG00000189325	9,103489562	1,56E-16	1,91E-15

DPYSL3	protein_coding	ENSG00000113657	-0,591447857	1,62E-16	1,99E-15
CDKN2AIP	protein_coding	ENSG00000168564	0,569716575	1,64E-16	2,02E-15
ETS1	protein_coding	ENSG00000134954	0,253731065	1,69E-16	2,07E-15
SETDB2	protein_coding	ENSG00000136169	0,639589268	1,73E-16	2,12E-15
TAF7	protein_coding	ENSG00000178913	-0,467078367	1,76E-16	2,15E-15
UHRF1	protein_coding	ENSG00000276043	-0,606911549	1,82E-16	2,23E-15
PRLR	protein_coding	ENSG00000113494	1,437722077	1,83E-16	2,25E-15
SNORD3B-1	snoRNA	ENSG00000265185	-2,134991112	1,84E-16	2,25E-15
CUL4A	protein_coding	ENSG00000139842	-0,33560821	1,88E-16	2,3E-15
TMEM187	protein_coding	ENSG00000177854	-0,967204105	1,93E-16	2,36E-15
CYP4F2	protein_coding	ENSG00000186115	4,709469785	1,95E-16	2,38E-15
CPSF7	protein_coding	ENSG00000149532	-0,39136105	1,97E-16	2,4E-15
FNDC3A	protein_coding	ENSG00000102531	0,340832173	2,01E-16	2,45E-15
EXOSC3	protein_coding	ENSG00000107371	-0,516754022	2,02E-16	2,46E-15
C1orf52	protein_coding	ENSG00000162642	0,501286828	2,03E-16	2,48E-15
RGS20	protein_coding	ENSG00000147509	0,597417754	2,17E-16	2,64E-15
ACTR5	protein_coding	ENSG00000101442	-0,685466437	2,25E-16	2,74E-15
CSPG4	protein_coding	ENSG00000173546	1,729001656	2,27E-16	2,76E-15
RB1	protein_coding	ENSG00000139687	0,431584755	2,34E-16	2,84E-15
FOXF2	protein_coding	ENSG00000137273	-0,906842081	2,46E-16	2,99E-15
ZNF33A	protein_coding	ENSG00000189180	-0,613083362	2,48E-16	3,01E-15
TMEM47	protein_coding	ENSG00000147027	0,659272793	2,54E-16	3,08E-15
CHST10	protein_coding	ENSG00000115526	-0,692808363	2,54E-16	3,08E-15
CITED2	protein_coding	ENSG00000164442	-0,297037063	2,61E-16	3,17E-15
NCBP2	protein_coding	ENSG00000114503	-0,436164988	2,65E-16	3,21E-15
AC007319.1	antisense	ENSG00000224063	1,518691754	2,66E-16	3,22E-15
RPS6KA3	protein_coding	ENSG00000177189	0,386011823	2,67E-16	3,23E-15
C2orf92	protein_coding	ENSG00000228486	2,245212958	2,76E-16	3,33E-15
RNF152	protein_coding	ENSG00000176641	1,014927738	2,75E-16	3,33E-15
PIGC	protein_coding	ENSG00000135845	-0,547606631	2,8E-16	3,39E-15
TOR3A	protein_coding	ENSG00000186283	-0,538606873	2,81E-16	3,4E-15
NUP35	protein_coding	ENSG00000163002	-0,718481705	2,86E-16	3,45E-15
FOXO3	protein_coding	ENSG00000118689	0,581604896	3,04E-16	3,66E-15
RPL3L	protein_coding	ENSG00000140986	4,99836674	3,23E-16	3,89E-15
SLC50A1	protein_coding	ENSG00000169241	0,5589826	3,24E-16	3,9E-15
SNRNP40	protein_coding	ENSG00000060688	-0,424788182	3,26E-16	3,92E-15
SLITRK6	protein_coding	ENSG00000184564	1,593135721	3,31E-16	3,98E-15
GPRASP2	protein_coding	ENSG00000158301	-0,485514564	3,35E-16	4,02E-15
GPRASP2	protein_coding	ENSG00000158301	-0,485514564	3,35E-16	4,02E-15
CDCA2	protein_coding	ENSG00000184661	-0,434944463	3,38E-16	4,06E-15

MSL1	protein_coding	ENSG00000188895	-0,443720937	3,46E-16	4,15E-15
TIGD6	protein_coding	ENSG00000164296	0,689501329	3,46E-16	4,16E-15
EXOSC10	protein_coding	ENSG00000171824	-0,388638488	3,47E-16	4,16E-15
PLB1	protein_coding	ENSG00000163803	1,912396469	3,51E-16	4,21E-15
C1S	protein_coding	ENSG00000182326	1,996396656	3,55E-16	4,26E-15
ZMYM1	protein_coding	ENSG00000197056	-0,755820006	3,6E-16	4,3E-15
ZNF248	protein_coding	ENSG00000198105	-0,709795763	3,66E-16	4,37E-15
LINC01554	lincRNA	ENSG00000236882	2,966569544	3,81E-16	4,56E-15
PDGFRL	protein_coding	ENSG00000104213	-0,738728326	3,89E-16	4,64E-15
C6orf58	protein_coding	ENSG00000184530	4,019806865	3,94E-16	4,7E-15
GGT1	protein_coding	ENSG00000100031	2,616767356	4,05E-16	4,84E-15
UBR2	protein_coding	ENSG00000024048	0,403168813	4,08E-16	4,87E-15
JADE1	protein_coding	ENSG00000077684	-0,463572961	4,21E-16	5,02E-15
AL109918.1	transcribed_unprocesse	ENSG00000216775	0,49993093	4,28E-16	5,1E-15
BEST3	protein_coding	ENSG00000127325	0,76148851	4,34E-16	5,17E-15
ROR1	protein_coding	ENSG00000185483	0,498497623	4,35E-16	5,18E-15
NUP205	protein_coding	ENSG00000155561	-0,335065804	4,36E-16	5,18E-15
ZNF561-AS1	processed_transcript	ENSG00000267106	0,946455981	4,38E-16	5,21E-15
SMIM7	protein_coding	ENSG00000214046	-0,522534691	4,39E-16	5,22E-15
ATP8B1	protein_coding	ENSG00000081923	-0,342374973	4,43E-16	5,26E-15
KLHDC1	protein_coding	ENSG00000197776	1,601700024	4,48E-16	5,31E-15
CDH10	protein_coding	ENSG00000040731	1,304237936	4,64E-16	5,51E-15
KCNJ2	protein_coding	ENSG00000123700	0,665918119	4,66E-16	5,52E-15
HDGF	protein_coding	ENSG00000143321	-0,285578256	4,66E-16	5,52E-15
SSUH2	protein_coding	ENSG00000125046	3,189322074	4,67E-16	5,53E-15
C1orf54	protein_coding	ENSG00000118292	3,043294832	4,8E-16	5,68E-15
MYCBP	protein_coding	ENSG00000214114	-0,560000454	4,85E-16	5,74E-15
CHTF8	protein_coding	ENSG00000168802	-0,423161772	4,97E-16	5,87E-15
CHTF8	protein_coding	ENSG00000168802	-0,423161772	4,97E-16	5,87E-15
ZNF395	protein_coding	ENSG00000186918	-0,570932631	5E-16	5,91E-15
BMPR2	protein_coding	ENSG00000204217	0,480497897	5,08E-16	6E-15
CACHD1	protein_coding	ENSG00000158966	0,612369266	5,17E-16	6,1E-15
GYPC	protein_coding	ENSG00000136732	0,463695477	5,19E-16	6,12E-15
TEX10	protein_coding	ENSG00000136891	0,385328838	5,2E-16	6,13E-15
ZBED4	protein_coding	ENSG00000100426	-0,630865971	5,25E-16	6,19E-15
CLK3	protein_coding	ENSG00000179335	0,402493565	5,31E-16	6,25E-15
ARMC6	protein_coding	ENSG00000105676	-0,527573723	5,32E-16	6,25E-15
PINLYP	protein_coding	ENSG00000234465	4,632734037	5,37E-16	6,32E-15
HARS2	protein_coding	ENSG00000112855	-0,463263683	5,46E-16	6,42E-15
RNU5A-1	snRNA	ENSG00000199568	-1,016243962	5,74E-16	6,74E-15

PTPRH	protein_coding	ENSG00000080031	0,590777677	5,86E-16	6,88E-15
CLK2	protein_coding	ENSG00000176444	-0,583633411	5,92E-16	6,95E-15
ANKRD1	protein_coding	ENSG00000148677	-0,526959257	5,94E-16	6,96E-15
NIF3L1	protein_coding	ENSG00000196290	-0,728626793	5,94E-16	6,96E-15
NEU3	protein_coding	ENSG00000162139	-0,470212865	6,04E-16	7,07E-15
RAB9B	protein_coding	ENSG00000123570	1,635057385	6,04E-16	7,08E-15
ARID4A	protein_coding	ENSG00000032219	0,523220584	6,15E-16	7,19E-15
WDCP	protein_coding	ENSG00000163026	-0,452883672	6,26E-16	7,32E-15
DNAJC9	protein_coding	ENSG00000213551	-0,572004193	6,29E-16	7,35E-15
SPATA9	protein_coding	ENSG00000145757	3,224430227	6,43E-16	7,5E-15
WDR77	protein_coding	ENSG00000116455	-0,43946865	6,43E-16	7,5E-15
FARSB	protein_coding	ENSG00000116120	-0,456387704	6,55E-16	7,64E-15
PTP4A1	protein_coding	ENSG00000112245	0,479616004	6,56E-16	7,65E-15
BRAF	protein_coding	ENSG00000157764	0,502520166	6,58E-16	7,67E-15
ARIH1	protein_coding	ENSG00000166233	0,338539486	6,59E-16	7,68E-15
CHCHD4	protein_coding	ENSG00000163528	-0,470178546	7,16E-16	8,33E-15
ZNF449	protein_coding	ENSG00000173275	-0,697999483	7,2E-16	8,38E-15
AC003092.1	lincRNA	ENSG00000236453	0,958352732	7,23E-16	8,41E-15
PGRMC1	protein_coding	ENSG00000101856	0,316733968	7,23E-16	8,41E-15
EFHD2	protein_coding	ENSG00000142634	-0,526939543	7,4E-16	8,6E-15
REV3L	protein_coding	ENSG00000009413	0,61001526	7,65E-16	8,87E-15
POU2F2	protein_coding	ENSG00000028277	2,080852447	7,78E-16	9,03E-15
CCDC127	protein_coding	ENSG00000164366	-0,484107131	7,8E-16	9,04E-15
MEF2A	protein_coding	ENSG00000068305	0,410844597	8,03E-16	9,3E-15
TRMU	protein_coding	ENSG00000100416	-0,426357364	8,36E-16	9,68E-15
SMC4	protein_coding	ENSG00000113810	-0,654510215	8,7E-16	1,01E-14
KDM1A	protein_coding	ENSG00000004487	-0,36595661	8,9E-16	1,03E-14
AP4E1	protein_coding	ENSG00000081014	0,386508767	9,02E-16	1,04E-14
CARS	protein_coding	ENSG00000110619	0,329769621	9,08E-16	1,05E-14
TUBG1	protein_coding	ENSG00000131462	-0,306443622	9,17E-16	1,06E-14
FBXO9	protein_coding	ENSG00000112146	-0,344314946	9,16E-16	1,06E-14
ARL15	protein_coding	ENSG00000185305	0,745525374	9,31E-16	1,07E-14
PDE1C	protein_coding	ENSG00000154678	-0,348733669	9,27E-16	1,07E-14
HNRNPA0	protein_coding	ENSG00000177733	-0,473822935	9,29E-16	1,07E-14
ZFPM1	protein_coding	ENSG00000179588	1,352759392	9,49E-16	1,09E-14
ILF3-DT	lincRNA	ENSG00000267100	-0,836387529	9,55E-16	1,1E-14
FMN1	protein_coding	ENSG00000248905	0,561898608	9,61E-16	1,11E-14
SRSF3	protein_coding	ENSG00000112081	-0,409161634	9,63E-16	1,11E-14
AKR1B10	protein_coding	ENSG00000198074	3,716351342	9,74E-16	1,12E-14
AL445487.1	processed_pseudogene	ENSG00000226535	3,458262842	9,93E-16	1,14E-14

ADAMTS12	protein_coding	ENSG00000151388	2,792918527	9,94E-16	1,14E-14
LRP3	protein_coding	ENSG00000130881	-0,617699061	1E-15	1,15E-14
ADGRF5	protein_coding	ENSG00000069122	1,045362581	1,01E-15	1,16E-14
MCC	protein_coding	ENSG00000171444	0,69241409	1,02E-15	1,16E-14
RPA1	protein_coding	ENSG00000132383	-0,44796322	1,01E-15	1,16E-14
SLC19A1	protein_coding	ENSG00000173638	-0,729216914	1,01E-15	1,16E-14
ADCK2	protein_coding	ENSG00000133597	-0,465096462	1,05E-15	1,2E-14
PPIB	protein_coding	ENSG00000166794	0,474235505	1,06E-15	1,21E-14
TRMT10C	protein_coding	ENSG00000174173	-0,458577313	1,1E-15	1,26E-14
COA3	protein_coding	ENSG00000183978	-0,597682695	1,1E-15	1,26E-14
BRI3BP	protein_coding	ENSG00000184992	-0,473456529	1,13E-15	1,29E-14
PANK4	protein_coding	ENSG00000157881	-0,512653112	1,13E-15	1,29E-14
ANKEF1	protein_coding	ENSG00000132623	0,577823855	1,15E-15	1,31E-14
KCTD2	protein_coding	ENSG00000180901	-0,512624572	1,17E-15	1,33E-14
SUPT16H	protein_coding	ENSG00000092201	-0,551434436	1,17E-15	1,33E-14
PARP2	protein_coding	ENSG00000129484	-0,45783729	1,18E-15	1,35E-14
JAZF1	protein_coding	ENSG00000153814	0,885941004	1,24E-15	1,41E-14
PSG7	protein_coding	ENSG00000221878	2,140910382	1,25E-15	1,42E-14
RBKS	protein_coding	ENSG00000171174	1,138101207	1,28E-15	1,46E-14
RBAK	protein_coding	ENSG00000146587	0,600217803	1,29E-15	1,46E-14
KPNA4	protein_coding	ENSG00000186432	0,291922495	1,32E-15	1,5E-14
RMC1	protein_coding	ENSG00000141452	0,446972266	1,34E-15	1,51E-14
PCNPP3	processed_pseudogene	ENSG00000213344	2,576730447	1,38E-15	1,57E-14
LPIN1	protein_coding	ENSG00000134324	0,483828951	1,41E-15	1,59E-14
RPSAP52	transcribed_processed_	ENSG00000241749	1,772785431	1,47E-15	1,66E-14
IL23A	protein_coding	ENSG00000110944	2,237508538	1,55E-15	1,75E-14
NOLC1	protein_coding	ENSG00000166197	-0,484006244	1,55E-15	1,75E-14
ARHGAP27	protein_coding	ENSG00000159314	0,928920393	1,56E-15	1,77E-14
ARID5A	protein_coding	ENSG00000196843	0,802666819	1,59E-15	1,79E-14
RRS1	protein_coding	ENSG00000179041	-0,476644923	1,59E-15	1,8E-14
HAUS8	protein_coding	ENSG00000131351	-0,571712113	1,62E-15	1,83E-14
GLOD4	protein_coding	ENSG00000167699	-0,449514845	1,67E-15	1,89E-14
THAP9	protein_coding	ENSG00000168152	0,962713324	1,72E-15	1,93E-14
CCDC148	protein_coding	ENSG00000153237	0,668441402	1,76E-15	1,98E-14
LAMTOR1	protein_coding	ENSG00000149357	-0,586440165	1,76E-15	1,98E-14
HGH1	protein_coding	ENSG00000235173	-0,672593108	1,79E-15	2,01E-14
FAM155B	protein_coding	ENSG00000130054	-0,786142691	1,8E-15	2,02E-14
SLC35G2	protein_coding	ENSG00000168917	0,573194267	1,85E-15	2,08E-14
PUM1	protein_coding	ENSG00000134644	-0,457346692	2,13E-15	2,39E-14
NAGA	protein_coding	ENSG00000198951	-0,507608569	2,14E-15	2,41E-14

SEPHS1	protein_coding	ENSG00000086475	-0,498830827	2,16E-15	2,43E-14
ZNF678	protein_coding	ENSG00000181450	-0,755231428	2,21E-15	2,48E-14
PSCA	protein_coding	ENSG00000167653	1,445627265	2,22E-15	2,49E-14
MAST4	protein_coding	ENSG00000069020	0,764941229	2,22E-15	2,49E-14
MRE11	protein_coding	ENSG00000020922	-0,528977287	2,26E-15	2,53E-14
COL5A2	protein_coding	ENSG00000204262	1,007547366	2,3E-15	2,57E-14
SH3PXD2B	protein_coding	ENSG00000174705	-0,686384027	2,31E-15	2,58E-14
MYH2	protein_coding	ENSG00000125414	2,329795604	2,33E-15	2,6E-14
B3GNT5	protein_coding	ENSG00000176597	2,266322992	2,36E-15	2,63E-14
DELE1	protein_coding	ENSG00000081791	-0,393701144	2,35E-15	2,63E-14
PTPN22	protein_coding	ENSG00000134242	0,674917246	2,64E-15	2,95E-14
BRAP	protein_coding	ENSG00000089234	0,382521206	2,7E-15	3,02E-14
CACNB3	protein_coding	ENSG00000167535	-0,678782015	2,74E-15	3,06E-14
ACTG1	protein_coding	ENSG00000184009	-0,354630456	2,76E-15	3,07E-14
SLC39A10	protein_coding	ENSG00000196950	-0,501738224	2,75E-15	3,07E-14
KIAA1147	protein_coding	ENSG00000257093	-0,728148967	2,76E-15	3,07E-14
TRAF5	protein_coding	ENSG00000082512	-0,715152816	2,85E-15	3,17E-14
THOC6	protein_coding	ENSG00000131652	-0,485874175	2,88E-15	3,2E-14
DYRK1A	protein_coding	ENSG00000157540	0,400097172	2,91E-15	3,23E-14
SLCO1A2	protein_coding	ENSG00000084453	0,515112449	2,92E-15	3,25E-14
RIOX2	protein_coding	ENSG00000170854	-0,404195499	2,97E-15	3,3E-14
LINC01623	lincRNA	ENSG00000225595	2,402269317	3,01E-15	3,34E-14
ADAP1	protein_coding	ENSG00000105963	2,704660687	3,05E-15	3,38E-14
ALDH18A1	protein_coding	ENSG00000059573	-0,330867598	3,06E-15	3,39E-14
REEP4	protein_coding	ENSG00000168476	-0,475103269	3,06E-15	3,39E-14
CGREF1	protein_coding	ENSG00000138028	4,651386373	3,16E-15	3,49E-14
MATN1	protein_coding	ENSG00000162510	3,268447739	3,16E-15	3,49E-14
MIB2	protein_coding	ENSG00000197530	0,858457585	3,16E-15	3,49E-14
QRICH1	protein_coding	ENSG00000198218	-0,375877744	3,21E-15	3,54E-14
PARD3B	protein_coding	ENSG00000116117	0,595949791	3,22E-15	3,56E-14
MND1	protein_coding	ENSG00000121211	-0,708636567	3,3E-15	3,65E-14
KLHL30	protein_coding	ENSG00000168427	6,625836787	3,34E-15	3,69E-14
ZNF32	protein_coding	ENSG00000169740	-0,624131365	3,35E-15	3,69E-14
MZF1-AS1	antisense	ENSG00000267858	1,857621573	3,39E-15	3,73E-14
ATP10D	protein_coding	ENSG00000145246	0,400670654	3,61E-15	3,98E-14
BTBD19	protein_coding	ENSG00000222009	1,724014166	3,65E-15	4,02E-14
BTN3A1	protein_coding	ENSG00000026950	-0,507926626	3,66E-15	4,02E-14
CENPF	protein_coding	ENSG00000117724	-0,781378303	3,68E-15	4,05E-14
KDM5A	protein_coding	ENSG00000073614	0,332522544	3,71E-15	4,08E-14
INPP5A	protein_coding	ENSG00000068383	0,389868087	3,81E-15	4,19E-14

ORC1	protein_coding	ENSG00000085840	-0,551536112	3,84E-15	4,22E-14
GART	protein_coding	ENSG00000159131	-0,407791747	3,85E-15	4,23E-14
CRLF3	protein_coding	ENSG00000176390	-0,576624998	3,88E-15	4,25E-14
USP15	protein_coding	ENSG00000135655	0,520150329	3,9E-15	4,27E-14
KIF26A	protein_coding	ENSG00000066735	-0,817416487	3,93E-15	4,3E-14
ZNF891	protein_coding	ENSG00000214029	-0,854156764	3,96E-15	4,33E-14
SNAPC5	protein_coding	ENSG00000174446	-0,541749579	3,99E-15	4,37E-14
ZNF749	protein_coding	ENSG00000186230	-0,768520015	4,1E-15	4,48E-14
POLD1	protein_coding	ENSG00000062822	-0,518361304	4,16E-15	4,55E-14
ATF5	protein_coding	ENSG00000169136	0,603435594	4,27E-15	4,67E-14
ZDHHC18	protein_coding	ENSG00000204160	-0,557568213	4,29E-15	4,69E-14
RRAGC	protein_coding	ENSG00000116954	0,500520868	4,44E-15	4,85E-14
RPL36A	protein_coding	ENSG00000241343	-1,08395006	4,47E-15	4,88E-14
CHD7	protein_coding	ENSG00000171316	-0,818128611	4,51E-15	4,91E-14
RBM5	protein_coding	ENSG00000003756	0,443123089	4,57E-15	4,98E-14
C7orf31	protein_coding	ENSG00000153790	1,356813545	4,68E-15	5,1E-14
DNAH17	protein_coding	ENSG00000187775	6,422318431	4,72E-15	5,14E-14
LINC00355	lincRNA	ENSG00000227674	0,792543749	4,8E-15	5,22E-14
UBAP2L	protein_coding	ENSG00000143569	-0,466015441	4,87E-15	5,29E-14
HMCN2	protein_coding	ENSG00000148357	2,61673134	4,97E-15	5,4E-14
ZNF830	protein_coding	ENSG00000198783	-0,526990514	5,1E-15	5,54E-14
BIK	protein_coding	ENSG00000100290	1,454951589	5,25E-15	5,7E-14
NONO	protein_coding	ENSG00000147140	-0,312065979	5,28E-15	5,73E-14
C12orf56	protein_coding	ENSG00000185306	-0,435904109	5,36E-15	5,82E-14
TNFAIP8L1	protein_coding	ENSG00000185361	-0,527985633	5,39E-15	5,84E-14
C1orf198	protein_coding	ENSG00000119280	-0,469601511	5,44E-15	5,89E-14
ABCA5	protein_coding	ENSG00000154265	0,63730972	5,48E-15	5,93E-14
HOXB-AS3	antisense	ENSG00000233101	2,409972056	5,49E-15	5,94E-14
BHLHE40-AS1	antisense	ENSG00000235831	3,516655953	5,5E-15	5,95E-14
OSGIN1	protein_coding	ENSG00000140961	0,726213121	5,54E-15	5,98E-14
CLPTM1L	protein_coding	ENSG00000049656	0,304133397	5,53E-15	5,98E-14
ACYP1	protein_coding	ENSG00000119640	-0,715545436	5,55E-15	6E-14
GRIA1	protein_coding	ENSG00000155511	2,772960657	5,57E-15	6,01E-14
HECW2-AS1	antisense	ENSG00000229056	1,092455239	5,61E-15	6,06E-14
UBAP1	protein_coding	ENSG00000165006	0,43713631	5,62E-15	6,06E-14
WWC1	protein_coding	ENSG00000113645	-0,542235807	5,69E-15	6,13E-14
NFS1	protein_coding	ENSG00000244005	-0,530709677	5,7E-15	6,14E-14
TNFAIP6	protein_coding	ENSG00000123610	6,865425794	5,85E-15	6,29E-14
ZNF791	protein_coding	ENSG00000173875	0,382921638	5,93E-15	6,38E-14
LARP4	protein_coding	ENSG00000161813	0,371390739	6,01E-15	6,47E-14

GNS	protein_coding	ENSG00000135677	0,375431995	6,18E-15	6,64E-14
CLCN3	protein_coding	ENSG00000109572	0,315353877	6,37E-15	6,84E-14
SH3RF2	protein_coding	ENSG00000156463	-0,417275857	6,51E-15	6,99E-14
PSTPIP2	protein_coding	ENSG00000152229	1,126047563	6,67E-15	7,16E-14
ZBTB14	protein_coding	ENSG00000198081	-0,862720511	6,78E-15	7,28E-14
IP6K2	protein_coding	ENSG00000068745	0,359478064	6,95E-15	7,45E-14
DPYSL4	protein_coding	ENSG00000151640	1,264611452	7,18E-15	7,7E-14
AAAS	protein_coding	ENSG00000094914	-0,455336963	7,2E-15	7,71E-14
N4BP3	protein_coding	ENSG00000145911	1,680239428	7,25E-15	7,76E-14
CENPX	protein_coding	ENSG00000169689	-0,534608364	7,63E-15	8,16E-14
TENT4A	protein_coding	ENSG00000112941	-0,493644935	7,86E-15	8,4E-14
NEIL3	protein_coding	ENSG00000109674	-0,70117656	7,87E-15	8,42E-14
HEATR1	protein_coding	ENSG00000119285	-0,378326635	8,17E-15	8,73E-14
SETD6	protein_coding	ENSG00000103037	-0,541682125	8,21E-15	8,77E-14
ZBTB3	protein_coding	ENSG00000185670	-0,879433047	8,56E-15	9,13E-14
IMP4	protein_coding	ENSG00000136718	-0,390524072	8,72E-15	9,3E-14
BCL3	protein_coding	ENSG00000069399	1,208764739	8,79E-15	9,37E-14
H2AFV	protein_coding	ENSG00000105968	-0,356692059	8,91E-15	9,49E-14
DTL	protein_coding	ENSG00000143476	-0,507263635	9,12E-15	9,7E-14
AL158206.1	sense_overlapping	ENSG00000260912	0,901381592	9,12E-15	9,7E-14
STARD10	protein_coding	ENSG00000214530	0,858714827	9,33E-15	9,92E-14
HEATR9	protein_coding	ENSG00000270379	6,874639497	9,35E-15	9,93E-14
ZNF674	protein_coding	ENSG00000251192	0,527001837	9,34E-15	9,93E-14
SLC39A11	protein_coding	ENSG00000133195	-0,40953262	9,36E-15	9,94E-14
CEP63	protein_coding	ENSG00000182923	0,420195011	9,44E-15	1E-13
KLF6	protein_coding	ENSG00000067082	-0,305037629	9,45E-15	1E-13
PPP4R1L	transcribed_unprocesse	ENSG00000124224	0,807635134	9,46E-15	1E-13
AKT2	protein_coding	ENSG00000105221	-0,399367216	9,52E-15	1,01E-13
SLF1	protein_coding	ENSG00000133302	-0,574346219	9,5E-15	1,01E-13
SAPCD2	protein_coding	ENSG00000186193	-0,617362714	9,93E-15	1,05E-13
MALINC1	antisense	ENSG00000245146	1,3633229	1,01E-14	1,07E-13
C2orf68	protein_coding	ENSG00000168887	-0,488490459	1,01E-14	1,07E-13
MINPP1	protein_coding	ENSG00000107789	-0,451120187	1,02E-14	1,08E-13
TBC1D4	protein_coding	ENSG00000136111	-0,40442253	1,12E-14	1,18E-13
BBS7	protein_coding	ENSG00000138686	0,47765615	1,16E-14	1,23E-13
ZNF669	protein_coding	ENSG00000188295	0,473839677	1,17E-14	1,23E-13
WNT10B	protein_coding	ENSG00000169884	-0,856407717	1,21E-14	1,28E-13
PSG2	protein_coding	ENSG00000242221	2,456528923	1,22E-14	1,29E-13
RWDD2B	protein_coding	ENSG00000156253	0,45289652	1,24E-14	1,31E-13
WNT5B	protein_coding	ENSG00000111186	-0,39694941	1,25E-14	1,32E-13

CD99L2	protein_coding	ENSG00000102181	-0,377140043	1,26E-14	1,33E-13
USP32	protein_coding	ENSG00000170832	0,286910859	1,29E-14	1,36E-13
UBE2H	protein_coding	ENSG00000186591	0,337394316	1,3E-14	1,37E-13
PDGFB	protein_coding	ENSG00000100311	1,930931724	1,32E-14	1,38E-13
PARD6B	protein_coding	ENSG00000124171	0,626521698	1,32E-14	1,38E-13
SURF4	protein_coding	ENSG00000148248	0,288129959	1,31E-14	1,38E-13
ZC3HC1	protein_coding	ENSG00000091732	-0,420087804	1,32E-14	1,38E-13
ZNF461	protein_coding	ENSG00000197808	0,734743602	1,34E-14	1,4E-13
DHX34	protein_coding	ENSG00000134815	-0,520668346	1,34E-14	1,41E-13
ZNF134	protein_coding	ENSG00000213762	0,336319851	1,36E-14	1,42E-13
ZNF81	protein_coding	ENSG00000197779	0,525147446	1,38E-14	1,45E-13
GATM	protein_coding	ENSG00000171766	3,215973092	1,45E-14	1,51E-13
HSPA1B	protein_coding	ENSG00000204388	0,626113891	1,44E-14	1,51E-13
HMCES	protein_coding	ENSG00000183624	-0,417362013	1,48E-14	1,55E-13
WRNIP1	protein_coding	ENSG00000124535	-0,33009726	1,5E-14	1,56E-13
CTSO	protein_coding	ENSG00000256043	0,821301323	1,54E-14	1,61E-13
GNA15	protein_coding	ENSG00000060558	1,22698953	1,6E-14	1,67E-13
GFOD2	protein_coding	ENSG00000141098	-0,559974137	1,6E-14	1,67E-13
SELENOS	protein_coding	ENSG00000131871	0,424253085	1,62E-14	1,69E-13
BCS1L	protein_coding	ENSG00000074582	-0,412696855	1,64E-14	1,71E-13
MRPS18B	protein_coding	ENSG00000204568	-0,433787318	1,65E-14	1,72E-13
POGLUT1	protein_coding	ENSG00000163389	0,443055589	1,67E-14	1,74E-13
AC021092.1	antisense	ENSG00000186019	1,497648449	1,69E-14	1,76E-13
IVNS1ABP	protein_coding	ENSG00000116679	-0,337730072	1,73E-14	1,8E-13
ANKRD42	protein_coding	ENSG00000137494	0,623415097	1,76E-14	1,83E-13
ZSCAN26	protein_coding	ENSG00000197062	0,461984109	1,79E-14	1,86E-13
CTTNBP2NL	protein_coding	ENSG00000143079	0,307811968	1,79E-14	1,86E-13
DHRS7B	protein_coding	ENSG00000109016	0,616120527	1,8E-14	1,87E-13
ZNF772	protein_coding	ENSG00000197128	-0,419742817	1,82E-14	1,88E-13
EHMT2	protein_coding	ENSG00000204371	-0,497969634	1,82E-14	1,88E-13
ANTXR2	protein_coding	ENSG00000163297	0,332459444	1,84E-14	1,91E-13
LIX1L	protein_coding	ENSG00000271601	-0,464927042	1,94E-14	2,01E-13
PDLIM7	protein_coding	ENSG00000196923	0,568771381	1,98E-14	2,05E-13
NOD1	protein_coding	ENSG00000106100	-0,826770903	2,02E-14	2,09E-13
SLC25A19	protein_coding	ENSG00000125454	-0,639119341	2,03E-14	2,1E-13
APOL3	protein_coding	ENSG00000128284	2,581230963	2,04E-14	2,11E-13
KYAT3	protein_coding	ENSG00000137944	0,469764971	2,05E-14	2,11E-13
TMEM245	protein_coding	ENSG00000106771	0,248556919	2,07E-14	2,14E-13
RPL22L1	protein_coding	ENSG00000163584	-0,704958207	2,08E-14	2,15E-13
GINS2	protein_coding	ENSG00000131153	-0,704370846	2,12E-14	2,19E-13

TP53INP2	protein_coding	ENSG00000078804	0,838422073	2,13E-14	2,2E-13
LMBRD2	protein_coding	ENSG00000164187	0,515669696	2,2E-14	2,27E-13
AC016747.1	processed_transcript	ENSG00000212978	-0,468793803	2,22E-14	2,29E-13
FAHD2CP	transcribed_unprocesse	ENSG00000231584	1,117993025	2,22E-14	2,29E-13
MRTO4	protein_coding	ENSG00000053372	-0,534414606	2,26E-14	2,33E-13
ATG4D	protein_coding	ENSG00000130734	0,758610515	2,28E-14	2,34E-13
POLE2	protein_coding	ENSG00000100479	-0,780230591	2,31E-14	2,38E-13
ZNF586	protein_coding	ENSG00000083828	-0,579945772	2,36E-14	2,42E-13
CAMSAP1	protein_coding	ENSG00000130559	-0,48543527	2,38E-14	2,44E-13
AC147651.4	antisense	ENSG00000237181	1,943909109	2,4E-14	2,46E-13
SRD5A3-AS1	processed_transcript	ENSG00000249700	4,639529748	2,4E-14	2,46E-13
HLA-C	protein_coding	ENSG00000204525	0,529714521	2,39E-14	2,46E-13
UBR3	protein_coding	ENSG00000144357	0,429438047	2,44E-14	2,5E-13
LIMA1	protein_coding	ENSG00000050405	-0,322836647	2,44E-14	2,5E-13
AC018647.2	antisense	ENSG00000271122	-0,607044272	2,48E-14	2,54E-13
TAF5L	protein_coding	ENSG00000135801	-0,457026998	2,52E-14	2,58E-13
ITPRIPL2	protein_coding	ENSG00000205730	0,508919385	2,58E-14	2,64E-13
FADS2	protein_coding	ENSG00000134824	0,731532474	2,64E-14	2,7E-13
BRPF3	protein_coding	ENSG00000096070	0,578617757	2,64E-14	2,7E-13
TAF6L	protein_coding	ENSG00000162227	-0,602913062	2,64E-14	2,7E-13
CHIC2	protein_coding	ENSG00000109220	0,465574285	2,65E-14	2,71E-13
SLC35E4	protein_coding	ENSG00000100036	0,75917729	2,68E-14	2,74E-13
YDJC	protein_coding	ENSG00000161179	-0,514535805	2,68E-14	2,74E-13
FAM217B	protein_coding	ENSG00000196227	-0,564039749	2,75E-14	2,81E-13
TRIM65	protein_coding	ENSG00000141569	-0,450730467	2,87E-14	2,92E-13
ARF5	protein_coding	ENSG00000004059	-0,398978058	2,95E-14	3,01E-13
PTTG1	protein_coding	ENSG00000164611	-0,476168443	2,98E-14	3,03E-13
PRUNE1	protein_coding	ENSG00000143363	-0,612142712	3,06E-14	3,11E-13
KAT8	protein_coding	ENSG00000103510	-0,544658907	3,07E-14	3,12E-13
CKAP2	protein_coding	ENSG00000136108	-0,62221032	3,1E-14	3,15E-13
PAQR4	protein_coding	ENSG00000162073	-0,668723594	3,11E-14	3,16E-13
RFLNA	protein_coding	ENSG00000178882	-0,438182476	3,13E-14	3,18E-13
RFLNA	protein_coding	ENSG00000178882	-0,438182476	3,13E-14	3,18E-13
TMEM136	protein_coding	ENSG00000181264	-0,456380612	3,21E-14	3,25E-13
GNB1L	protein_coding	ENSG00000185838	-0,715074391	3,28E-14	3,33E-13
ZNF699	protein_coding	ENSG00000196110	0,603601817	3,33E-14	3,37E-13
FOXRED2	protein_coding	ENSG00000100350	-0,603681457	3,4E-14	3,45E-13
LINC02086	lincRNA	ENSG00000244649	1,687420863	3,52E-14	3,57E-13
KIAA1755	protein_coding	ENSG00000149633	0,863903834	3,53E-14	3,57E-13
CCDC186	protein_coding	ENSG00000165813	0,592547236	3,55E-14	3,59E-13

ST20-AS1	antisense	ENSG00000259642	1,13968202	3,57E-14	3,61E-13
PYCR3	protein_coding	ENSG00000104524	-0,711198777	3,72E-14	3,76E-13
FAM117B	protein_coding	ENSG00000138439	-0,732586658	3,74E-14	3,78E-13
C3	protein_coding	ENSG00000125730	1,753273026	3,87E-14	3,91E-13
ZNF841	protein_coding	ENSG00000197608	0,751007777	3,92E-14	3,95E-13
SDCBP	protein_coding	ENSG00000137575	0,324746269	3,93E-14	3,96E-13
PLEKHG3	protein_coding	ENSG00000126822	-0,829102901	3,95E-14	3,99E-13
MRNIP	protein_coding	ENSG00000161010	-0,746258737	4,01E-14	4,04E-13
SHMT2	protein_coding	ENSG00000182199	0,453528825	4,02E-14	4,05E-13
TRAM2-AS1	lincRNA	ENSG00000225791	0,54289304	4,07E-14	4,1E-13
INO80D	protein_coding	ENSG00000114933	0,563755142	4,1E-14	4,12E-13
DEPDC1	protein_coding	ENSG00000024526	-0,612117381	4,18E-14	4,2E-13
EVA1A	protein_coding	ENSG00000115363	-0,425459042	4,2E-14	4,22E-13
PDZPH1P	transcribed_unitary_pse	ENSG00000226926	2,50330653	4,21E-14	4,23E-13
STYK1	protein_coding	ENSG00000060140	1,232198414	4,22E-14	4,24E-13
SDK1	protein_coding	ENSG00000146555	1,059423776	4,24E-14	4,25E-13
MCMBP	protein_coding	ENSG00000197771	-0,522211906	4,27E-14	4,29E-13
DBF4	protein_coding	ENSG00000006634	-0,625173851	4,41E-14	4,42E-13
FZD2	protein_coding	ENSG00000180340	-0,506787912	4,49E-14	4,5E-13
SLC38A2	protein_coding	ENSG00000134294	-0,363356937	4,57E-14	4,58E-13
CCNE2	protein_coding	ENSG00000175305	-0,632756048	4,57E-14	4,58E-13
GBP1	protein_coding	ENSG00000117228	1,348685603	4,61E-14	4,62E-13
YJU2	protein_coding	ENSG00000105248	-0,525373979	4,62E-14	4,62E-13
GAS5	processed_transcript	ENSG00000234741	-0,398245109	4,63E-14	4,63E-13
ZNF143	protein_coding	ENSG00000166478	-0,506737446	4,7E-14	4,69E-13
WDR12	protein_coding	ENSG00000138442	-0,40476296	4,84E-14	4,83E-13
ZNRF3	protein_coding	ENSG00000183579	-0,659913253	4,86E-14	4,85E-13
ZBTB45	protein_coding	ENSG00000119574	-0,784558951	4,99E-14	4,98E-13
ZNF408	protein_coding	ENSG00000175213	0,719744499	5,02E-14	5,01E-13
PDCL	protein_coding	ENSG00000136940	-0,444525376	5,02E-14	5,01E-13
STX12	protein_coding	ENSG00000117758	0,413634456	5,12E-14	5,1E-13
GOLGA4	protein_coding	ENSG00000144674	0,537470997	5,14E-14	5,12E-13
CPB2-AS1	antisense	ENSG00000235903	1,678744295	5,15E-14	5,13E-13
DDX18	protein_coding	ENSG00000088205	-0,393314845	5,2E-14	5,17E-13
EPM2AIP1	protein_coding	ENSG00000178567	-0,451860259	5,21E-14	5,18E-13
GALNT11	protein_coding	ENSG00000178234	-0,574660615	5,2E-14	5,18E-13
SRFBP1	protein_coding	ENSG00000151304	0,427394191	5,26E-14	5,22E-13
EEF2K	protein_coding	ENSG00000103319	-0,519712843	5,43E-14	5,39E-13
SERPINH1	protein_coding	ENSG00000149257	0,534795005	5,46E-14	5,42E-13
TCOF1	protein_coding	ENSG00000070814	-0,55752427	5,5E-14	5,46E-13

ZBED1	protein_coding	ENSG00000214717	-0,447495888	5,72E-14	5,67E-13
B4GAT1	protein_coding	ENSG00000174684	-0,487584335	5,78E-14	5,73E-13
BEST1	protein_coding	ENSG00000167995	3,668018918	5,8E-14	5,75E-13
B4GALT2	protein_coding	ENSG00000117411	-0,438938485	5,84E-14	5,79E-13
CBX3	protein_coding	ENSG00000122565	-0,504566605	5,93E-14	5,87E-13
RC3H1	protein_coding	ENSG00000135870	0,394409625	6,08E-14	6,02E-13
RNF26	protein_coding	ENSG00000173456	-0,554732073	6,14E-14	6,07E-13
POLD2	protein_coding	ENSG00000106628	-0,299824212	6,27E-14	6,2E-13
CRYBB2P1	transcribed_unprocesse	ENSG00000100058	0,62056763	6,34E-14	6,26E-13
STAT2	protein_coding	ENSG00000170581	0,458893943	6,39E-14	6,31E-13
KYNU	protein_coding	ENSG00000115919	0,487007446	6,4E-14	6,32E-13
ATF4	protein_coding	ENSG00000128272	-0,303934941	6,74E-14	6,65E-13
INSYN2B	protein_coding	ENSG00000204767	0,366097521	6,85E-14	6,76E-13
PSMG3	protein_coding	ENSG00000157778	-0,478927173	6,88E-14	6,78E-13
PRDM15	protein_coding	ENSG00000141956	-0,659740791	6,95E-14	6,85E-13
MIS18A	protein_coding	ENSG00000159055	-0,538886996	7,05E-14	6,94E-13
TTC41P	transcribed_unitary_pse	ENSG00000214198	1,5886232	7,05E-14	6,94E-13
SPAG9	protein_coding	ENSG00000008294	0,357208719	7,1E-14	6,98E-13
CD177	protein_coding	ENSG00000204936	2,754598791	7,12E-14	7E-13
BRD9	protein_coding	ENSG00000028310	-0,473569736	7,15E-14	7,03E-13
AC024909.1	antisense	ENSG00000274021	1,859573116	7,18E-14	7,05E-13
NUDT2	protein_coding	ENSG00000164978	-0,605814058	7,21E-14	7,07E-13
NT5C	protein_coding	ENSG00000125458	-0,497656454	7,39E-14	7,25E-13
RNF111	protein_coding	ENSG00000157450	0,456364238	7,43E-14	7,28E-13
DPYSL2	protein_coding	ENSG00000092964	-0,411263678	7,45E-14	7,3E-13
PRXL2B	protein_coding	ENSG00000157870	-0,710441573	7,49E-14	7,34E-13
SLC11A2	protein_coding	ENSG00000110911	-0,379457878	7,59E-14	7,43E-13
KIAA0930	protein_coding	ENSG00000100364	-0,452704092	7,68E-14	7,52E-13
FP565260.1	protein_coding	ENSG00000275464	-0,642617716	7,74E-14	7,58E-13
ARL8A	protein_coding	ENSG00000143862	0,453184888	7,84E-14	7,67E-13
CYP1A1	protein_coding	ENSG00000140465	2,283508539	7,88E-14	7,7E-13
LIG3	protein_coding	ENSG00000005156	-0,427380873	7,91E-14	7,72E-13
ARL14	protein_coding	ENSG00000179674	1,243662309	7,92E-14	7,74E-13
SNAI2	protein_coding	ENSG00000019549	0,496305692	8,03E-14	7,84E-13
PEA15	protein_coding	ENSG00000162734	0,312721794	8,06E-14	7,87E-13
PPP1R18	protein_coding	ENSG00000146112	0,404248082	8,08E-14	7,88E-13
PPP1R18	protein_coding	ENSG00000146112	0,404248082	8,08E-14	7,88E-13
CEP152	protein_coding	ENSG00000103995	-0,482863639	8,13E-14	7,92E-13
ZFHX2	protein_coding	ENSG00000136367	1,650441721	8,15E-14	7,94E-13
TAX1BP1	protein_coding	ENSG00000106052	0,348249684	8,24E-14	8,02E-13

ADAMTS3	protein_coding	ENSG00000156140	1,118831186	8,26E-14	8,04E-13
PKIA	protein_coding	ENSG00000171033	0,567679241	8,31E-14	8,09E-13
NMT1	protein_coding	ENSG00000136448	-0,289501895	8,37E-14	8,14E-13
POLR3E	protein_coding	ENSG00000058600	-0,37959871	8,57E-14	8,33E-13
COX19	protein_coding	ENSG00000240230	0,565310982	8,61E-14	8,37E-13
ZNF587B	protein_coding	ENSG00000269343	-0,60363339	8,68E-14	8,43E-13
GMNN	protein_coding	ENSG00000112312	-0,569512084	8,91E-14	8,65E-13
PIP4K2B	protein_coding	ENSG00000276293	-0,352217368	8,93E-14	8,66E-13
PPP1R3F	protein_coding	ENSG00000049769	1,31773328	8,95E-14	8,68E-13
MTERF3	protein_coding	ENSG00000156469	-0,525948829	8,98E-14	8,7E-13
MRPL17	protein_coding	ENSG00000158042	-0,561272667	9E-14	8,72E-13
STX2	protein_coding	ENSG00000111450	-0,425013439	9,09E-14	8,81E-13
ZC3H12C	protein_coding	ENSG00000149289	0,513256421	9,35E-14	9,05E-13
TROAP	protein_coding	ENSG00000135451	-0,387974209	9,39E-14	9,09E-13
SOS2	protein_coding	ENSG00000100485	0,419068138	9,51E-14	9,2E-13
ZNF496	protein_coding	ENSG00000162714	-0,374845889	9,68E-14	9,36E-13
SLC35B2	protein_coding	ENSG00000157593	-0,435315826	9,78E-14	9,44E-13
PES1	protein_coding	ENSG00000100029	-0,316351512	9,86E-14	9,52E-13
BLZF1	protein_coding	ENSG00000117475	0,470598461	9,96E-14	9,61E-13
POFUT1	protein_coding	ENSG00000101346	-0,38078576	9,97E-14	9,62E-13
XRCC2	protein_coding	ENSG00000196584	-0,555149081	1,02E-13	9,88E-13
INPP1	protein_coding	ENSG00000151689	0,415757107	1,03E-13	9,96E-13
MROH8	protein_coding	ENSG00000101353	1,2317923	1,05E-13	1,01E-12
SCAF4	protein_coding	ENSG00000156304	0,522001425	1,07E-13	1,03E-12
VKORC1L1	protein_coding	ENSG00000196715	-0,456827335	1,07E-13	1,03E-12
RPA2	protein_coding	ENSG00000117748	-0,469626191	1,07E-13	1,03E-12
B2M	protein_coding	ENSG00000166710	0,446034128	1,09E-13	1,04E-12
IMPDH1	protein_coding	ENSG00000106348	-0,375941251	1,1E-13	1,06E-12
MPP2	protein_coding	ENSG00000108852	-0,616690742	1,1E-13	1,06E-12
PLCB1	protein_coding	ENSG00000182621	0,496791787	1,11E-13	1,07E-12
AL354920.1	antisense	ENSG00000254473	1,118361134	1,12E-13	1,08E-12
RSF1	protein_coding	ENSG00000048649	0,397108798	1,12E-13	1,08E-12
AC010343.3	lincRNA	ENSG00000250697	0,581246602	1,14E-13	1,1E-12
FAM200A	protein_coding	ENSG00000221909	0,674223761	1,18E-13	1,13E-12
ZNF324B	protein_coding	ENSG00000249471	-0,767872811	1,18E-13	1,13E-12
DTX3L	protein_coding	ENSG00000163840	0,302962636	1,19E-13	1,14E-12
RTL8B	protein_coding	ENSG00000212747	-0,445214168	1,19E-13	1,14E-12
ZC3H12A	protein_coding	ENSG00000163874	1,080981941	1,31E-13	1,25E-12
CHAC1	protein_coding	ENSG00000128965	0,917761483	1,32E-13	1,26E-12
ARRDC3-AS1	lincRNA	ENSG00000281357	1,81365805	1,33E-13	1,27E-12

AC005821.1	antisense	ENSG00000265702	2,225266756	1,34E-13	1,28E-12
RSRC2	protein_coding	ENSG00000111011	0,409907905	1,34E-13	1,28E-12
DAZAP2	protein_coding	ENSG00000183283	-0,337959101	1,34E-13	1,28E-12
CCNT1	protein_coding	ENSG00000129315	0,238749032	1,36E-13	1,29E-12
CALCRL	protein_coding	ENSG00000064989	2,079414468	1,38E-13	1,31E-12
NMRAL1	protein_coding	ENSG00000153406	-0,422193651	1,37E-13	1,31E-12
DVL2	protein_coding	ENSG00000004975	-0,431824573	1,37E-13	1,31E-12
C22orf39	protein_coding	ENSG00000242259	-0,434780661	1,37E-13	1,31E-12
GUCD1	protein_coding	ENSG00000138867	-0,397503736	1,39E-13	1,32E-12
TMBIM1	protein_coding	ENSG00000135926	0,355971481	1,4E-13	1,33E-12
MEGF8	protein_coding	ENSG00000105429	0,803561982	1,44E-13	1,37E-12
SMG9	protein_coding	ENSG00000105771	-0,422572201	1,44E-13	1,37E-12
TIPIN	protein_coding	ENSG00000075131	-0,606974267	1,44E-13	1,37E-12
LUARIS	antisense	ENSG00000231638	1,052731865	1,46E-13	1,39E-12
LINC01358	lincRNA	ENSG00000237352	1,853852339	1,49E-13	1,41E-12
EIF3D	protein_coding	ENSG00000100353	-0,337298385	1,5E-13	1,43E-12
LRP8	protein_coding	ENSG00000157193	-0,601901235	1,57E-13	1,48E-12
ABHD3	protein_coding	ENSG00000158201	0,687327967	1,59E-13	1,51E-12
PTPN14	protein_coding	ENSG00000152104	0,553495725	1,6E-13	1,51E-12
CCNE1	protein_coding	ENSG00000105173	-0,605817122	1,59E-13	1,51E-12
TRIM35	protein_coding	ENSG00000104228	0,560722298	1,61E-13	1,52E-12
PADI1	protein_coding	ENSG00000142623	1,463187936	1,65E-13	1,56E-12
ATAD5	protein_coding	ENSG00000176208	-0,561271255	1,65E-13	1,56E-12
CFAP70	protein_coding	ENSG00000156042	1,544700152	1,66E-13	1,57E-12
NAB1	protein_coding	ENSG00000138386	0,374059014	1,73E-13	1,63E-12
MTFR1L	protein_coding	ENSG00000117640	-0,342517252	1,73E-13	1,64E-12
GRIPAP1	protein_coding	ENSG00000068400	0,575165964	1,76E-13	1,66E-12
PRDM1	protein_coding	ENSG00000057657	0,466831722	1,81E-13	1,7E-12
FANCC	protein_coding	ENSG00000158169	-0,514000723	1,81E-13	1,71E-12
PHF20	protein_coding	ENSG00000025293	-0,314204274	1,85E-13	1,75E-12
CD59	protein_coding	ENSG00000085063	-0,41170011	1,86E-13	1,75E-12
MEIS3	protein_coding	ENSG00000105419	0,679712921	1,88E-13	1,77E-12
PRC1	protein_coding	ENSG00000198901	-0,317824941	1,88E-13	1,77E-12
METTL17	protein_coding	ENSG00000165792	-0,336069976	1,93E-13	1,81E-12
KCTD20	protein_coding	ENSG00000112078	-0,28478682	1,93E-13	1,82E-12
NADK	protein_coding	ENSG00000008130	-0,469975672	1,96E-13	1,84E-12
ACSF2	protein_coding	ENSG00000167107	0,502866147	1,97E-13	1,85E-12
MAOA	protein_coding	ENSG00000189221	0,454930627	1,97E-13	1,85E-12
CKAP4	protein_coding	ENSG00000136026	-0,331748264	1,97E-13	1,85E-12
SFXN4	protein_coding	ENSG00000183605	-0,481820165	2,07E-13	1,94E-12

NIPAL2	protein_coding	ENSG00000104361	1,071842471	2,08E-13	1,95E-12
KBTBD8	protein_coding	ENSG00000163376	0,79500645	2,1E-13	1,97E-12
IVL	protein_coding	ENSG00000163207	-0,68328138	2,1E-13	1,97E-12
AC073957.3	antisense	ENSG00000273151	6,220133313	2,13E-13	2E-12
SENP6	protein_coding	ENSG00000112701	0,317881621	2,15E-13	2,01E-12
SUPV3L1	protein_coding	ENSG00000156502	0,333024554	2,16E-13	2,02E-12
RFC2	protein_coding	ENSG00000049541	-0,434388204	2,16E-13	2,02E-12
LARS2	protein_coding	ENSG00000011376	-0,357460464	2,17E-13	2,03E-12
ZC3H10	protein_coding	ENSG00000135482	-0,573531897	2,21E-13	2,07E-12
PINK1-AS	antisense	ENSG00000117242	0,814021327	2,24E-13	2,09E-12
AC009804.1	processed_pseudogene	ENSG00000274879	4,069873894	2,29E-13	2,14E-12
BNC1	protein_coding	ENSG00000169594	-0,30707331	2,29E-13	2,14E-12
ENTPD7	protein_coding	ENSG00000198018	0,487581121	2,31E-13	2,15E-12
NR2F1	protein_coding	ENSG00000175745	0,884948868	2,36E-13	2,2E-12
MFN1	protein_coding	ENSG00000171109	-0,426098356	2,43E-13	2,26E-12
BEND3	protein_coding	ENSG00000178409	-0,724100043	2,46E-13	2,29E-12
DLK2	protein_coding	ENSG00000171462	-0,791974857	2,49E-13	2,32E-12
NTN4	protein_coding	ENSG00000074527	-0,29423637	2,51E-13	2,34E-12
KRT8P12	transcribed_processed_	ENSG00000229320	0,728793604	2,52E-13	2,34E-12
ZNF22	protein_coding	ENSG00000165512	-0,49811317	2,55E-13	2,37E-12
PLK4	protein_coding	ENSG00000142731	-0,526562874	2,61E-13	2,43E-12
PIP4P2	protein_coding	ENSG00000155099	0,484701571	2,65E-13	2,46E-12
SRSF10	protein_coding	ENSG00000188529	-0,469132213	2,66E-13	2,47E-12
CASP8AP2	protein_coding	ENSG00000118412	-0,57727233	2,66E-13	2,47E-12
LDLRAD3	protein_coding	ENSG00000179241	-0,464956023	2,75E-13	2,55E-12
BCL2A1	protein_coding	ENSG00000140379	0,697898239	2,79E-13	2,59E-12
CAPS2	protein_coding	ENSG00000180881	0,632375743	2,9E-13	2,69E-12
CREBL2	protein_coding	ENSG00000111269	0,386759545	2,94E-13	2,73E-12
LAMP1	protein_coding	ENSG00000185896	0,321532778	2,95E-13	2,73E-12
RNF216P1	transcribed_unprocesse	ENSG00000196204	-0,469893714	3,03E-13	2,81E-12
LCP1	protein_coding	ENSG00000136167	0,855018769	3,11E-13	2,88E-12
COPS7B	protein_coding	ENSG00000144524	-0,429615531	3,14E-13	2,9E-12
PTPN3	protein_coding	ENSG00000070159	-0,503147615	3,15E-13	2,91E-12
CRY1	protein_coding	ENSG00000008405	0,358836774	3,22E-13	2,97E-12
ZNRF1	protein_coding	ENSG00000186187	-0,478560778	3,25E-13	3E-12
TDO2	protein_coding	ENSG00000151790	1,078841814	3,34E-13	3,08E-12
MPHOSPH9	protein_coding	ENSG00000051825	-0,465092676	3,35E-13	3,09E-12
SYT16	protein_coding	ENSG00000139973	0,496675861	3,36E-13	3,1E-12
TP53BP1	protein_coding	ENSG00000067369	-0,348639787	3,37E-13	3,1E-12
HP1BP3	protein_coding	ENSG00000127483	-0,316519014	3,37E-13	3,11E-12

AGO3	protein_coding	ENSG00000126070	0,421312833	3,39E-13	3,12E-12
KRT80	protein_coding	ENSG00000167767	0,483455867	3,51E-13	3,23E-12
CDC37L1	protein_coding	ENSG00000106993	0,560123445	3,53E-13	3,25E-12
KDELC2	protein_coding	ENSG00000178202	-0,485057565	3,54E-13	3,26E-12
SLC7A5	protein_coding	ENSG00000103257	-0,593186496	3,57E-13	3,28E-12
ZMYM3	protein_coding	ENSG00000147130	-0,472060705	3,64E-13	3,34E-12
HIST1H4D	protein_coding	ENSG00000277157	3,535706378	3,65E-13	3,35E-12
TAF3	protein_coding	ENSG00000165632	0,588522826	3,65E-13	3,35E-12
CALHM4	protein_coding	ENSG00000164451	1,237839396	3,68E-13	3,37E-12
C1orf162	protein_coding	ENSG00000143110	2,842870108	3,72E-13	3,41E-12
GTF2H3	protein_coding	ENSG00000111358	-0,329460699	3,73E-13	3,42E-12
FUT1	protein_coding	ENSG00000174951	5,290364636	3,76E-13	3,45E-12
PPIL2	protein_coding	ENSG00000100023	-0,479062268	3,77E-13	3,45E-12
GPR1	protein_coding	ENSG00000183671	-0,540451264	3,76E-13	3,45E-12
CSNK1D	protein_coding	ENSG00000141551	-0,356176141	3,86E-13	3,53E-12
ZNF596	protein_coding	ENSG00000172748	1,172840011	3,87E-13	3,54E-12
ACAN	protein_coding	ENSG00000157766	8,223169771	3,9E-13	3,56E-12
CNOT9	protein_coding	ENSG00000144580	-0,385745446	3,97E-13	3,63E-12
OR2B6	protein_coding	ENSG00000124657	1,927657882	4E-13	3,66E-12
AARS	protein_coding	ENSG00000090861	-0,27104052	4,05E-13	3,69E-12
IL31RA	protein_coding	ENSG00000164509	0,393462265	4,07E-13	3,71E-12
LMNB2	protein_coding	ENSG00000176619	-0,572909204	4,08E-13	3,72E-12
GASAL1	lincRNA	ENSG00000253669	0,875758632	4,12E-13	3,75E-12
HOXB9	protein_coding	ENSG00000170689	0,480966464	4,29E-13	3,91E-12
FZD4	protein_coding	ENSG00000174804	0,988528185	4,38E-13	4E-12
PLIN4	protein_coding	ENSG00000167676	3,025284574	4,5E-13	4,1E-12
MCU	protein_coding	ENSG00000156026	-0,37321016	4,53E-13	4,13E-12
DLGAP5	protein_coding	ENSG00000126787	-0,533663936	4,55E-13	4,14E-12
ATP13A3	protein_coding	ENSG00000133657	0,39421812	4,59E-13	4,18E-12
HMGN2	protein_coding	ENSG00000198830	-0,422328643	4,62E-13	4,2E-12
GNG12-AS1	antisense	ENSG00000232284	2,186281217	4,69E-13	4,26E-12
CEP192	protein_coding	ENSG00000101639	-0,37760174	4,81E-13	4,37E-12
CARM1	protein_coding	ENSG00000142453	-0,395327481	4,88E-13	4,43E-12
AC011446.2	antisense	ENSG00000267598	2,639037505	5,01E-13	4,55E-12
AC051619.8	sense_intronic	ENSG00000260035	-3,209251338	5,01E-13	4,55E-12
MB21D2	protein_coding	ENSG00000180611	0,328536529	5,05E-13	4,58E-12
SMC1A	protein_coding	ENSG00000072501	-0,485295913	5,1E-13	4,62E-12
SHMT1	protein_coding	ENSG00000176974	-0,385150708	5,14E-13	4,66E-12
HTRA1	protein_coding	ENSG00000166033	0,901664211	5,3E-13	4,8E-12
THAP9-AS1	antisense	ENSG00000251022	0,44698445	5,36E-13	4,85E-12

PBK	protein_coding	ENSG00000168078	-0,595933238	5,48E-13	4,96E-12
CENPBD1P1	transcribed_processed_	ENSG00000213753	-0,534600716	5,59E-13	5,05E-12
DNAH6	protein_coding	ENSG00000115423	2,651660453	5,67E-13	5,13E-12
ANAPC5	protein_coding	ENSG00000089053	-0,242751248	5,68E-13	5,13E-12
FOSL2	protein_coding	ENSG00000075426	-0,510278898	5,68E-13	5,13E-12
CEBPB-AS1	antisense	ENSG00000277449	3,632552088	5,71E-13	5,16E-12
KRT15	protein_coding	ENSG00000171346	-0,308750339	5,83E-13	5,26E-12
ETNK1	protein_coding	ENSG00000139163	-0,532052792	5,96E-13	5,38E-12
ADAMTS6	protein_coding	ENSG00000049192	-0,35140418	6,18E-13	5,58E-12
ANGPT2	protein_coding	ENSG00000091879	6,093860528	6,26E-13	5,63E-12
ZBTB2	protein_coding	ENSG00000181472	0,389809912	6,26E-13	5,63E-12
RNU2-63P	snRNA	ENSG00000222724	-3,69968203	6,25E-13	5,63E-12
ZNF419	protein_coding	ENSG00000105136	0,661444867	6,36E-13	5,73E-12
SEC11C	protein_coding	ENSG00000166562	0,553670885	6,44E-13	5,79E-12
SPOCD1	protein_coding	ENSG00000134668	0,666191124	6,52E-13	5,86E-12
AC061992.1	lincRNA	ENSG00000266970	2,917407687	6,79E-13	6,1E-12
PMEPA1	protein_coding	ENSG00000124225	0,826166359	6,89E-13	6,19E-12
AP3M2	protein_coding	ENSG00000070718	-0,387899216	6,92E-13	6,22E-12
LIMD1	protein_coding	ENSG00000144791	-0,425902377	6,95E-13	6,24E-12
CD27-AS1	processed_transcript	ENSG00000215039	1,244705196	6,97E-13	6,26E-12
RFC5	protein_coding	ENSG00000111445	-0,345964306	6,99E-13	6,27E-12
EHD1	protein_coding	ENSG00000110047	0,519575401	7,04E-13	6,31E-12
IFNL3	protein_coding	ENSG00000197110	8,149856213	7,05E-13	6,32E-12
LYPD1	protein_coding	ENSG00000150551	1,245115293	7,06E-13	6,33E-12
CLMP	protein_coding	ENSG00000166250	-0,421050283	7,11E-13	6,36E-12
SPHK1	protein_coding	ENSG00000176170	0,675774278	7,16E-13	6,41E-12
CLGN	protein_coding	ENSG00000153132	0,595651523	7,28E-13	6,51E-12
TRIM25	protein_coding	ENSG00000121060	0,32892322	7,28E-13	6,51E-12
TM2D3	protein_coding	ENSG00000184277	-0,440652884	7,28E-13	6,51E-12
LOX	protein_coding	ENSG00000113083	0,312390187	7,37E-13	6,58E-12
KLRD1	protein_coding	ENSG00000134539	6,135241579	7,64E-13	6,82E-12
COL11A2	protein_coding	ENSG00000204248	1,397072755	7,81E-13	6,97E-12
FOXD2	protein_coding	ENSG00000186564	-1,376720003	8,05E-13	7,18E-12
FGFR1	protein_coding	ENSG00000077782	0,562380814	8,3E-13	7,4E-12
ZNF814	protein_coding	ENSG00000204514	-0,512146514	8,72E-13	7,77E-12
NUDT21	protein_coding	ENSG00000167005	-0,389670417	8,85E-13	7,88E-12
LINC00963	processed_transcript	ENSG00000204054	0,492385472	9,05E-13	8,05E-12
NAMPT	protein_coding	ENSG00000105835	0,482700015	9,15E-13	8,14E-12
MIR7-3HG	lincRNA	ENSG00000176840	8,026137699	9,32E-13	8,29E-12
MIEF2	protein_coding	ENSG00000177427	-0,573662985	9,33E-13	8,3E-12

SPTY2D1	protein_coding	ENSG00000179119	0,396605973	9,55E-13	8,49E-12
MGA	protein_coding	ENSG00000174197	-0,453211626	9,75E-13	8,65E-12
TSHZ1	protein_coding	ENSG00000179981	-0,753521448	9,73E-13	8,65E-12
TP53INP1	protein_coding	ENSG00000164938	0,371066258	1,03E-12	9,11E-12
AC025181.2	lincRNA	ENSG00000272086	1,813218863	1,03E-12	9,15E-12
ATP4A	protein_coding	ENSG00000105675	6,345711324	1,03E-12	9,18E-12
NIP7	protein_coding	ENSG00000132603	-0,464861989	1,04E-12	9,19E-12
AL356414.1	lincRNA	ENSG00000205300	7,900241724	1,07E-12	9,51E-12
CACNA1G	protein_coding	ENSG00000006283	3,747261471	1,12E-12	9,89E-12
SLC5A3	protein_coding	ENSG00000198743	-0,509686955	1,12E-12	9,95E-12
RAB40B	protein_coding	ENSG00000141542	-0,824055891	1,13E-12	9,98E-12
PPP2R1B	protein_coding	ENSG00000137713	-0,41789982	1,15E-12	1,01E-11
LIF	protein_coding	ENSG00000128342	0,702159247	1,17E-12	1,03E-11
TRIM21	protein_coding	ENSG00000132109	0,607022834	1,17E-12	1,03E-11
DNTTIP1	protein_coding	ENSG00000101457	0,390191388	1,17E-12	1,03E-11
PAFAH1B2	protein_coding	ENSG00000168092	-0,291172055	1,17E-12	1,03E-11
RUNX2	protein_coding	ENSG00000124813	0,637457495	1,21E-12	1,07E-11
KIDINS220	protein_coding	ENSG00000134313	0,401788113	1,23E-12	1,08E-11
MMP15	protein_coding	ENSG00000102996	-0,827716578	1,22E-12	1,08E-11
PEF1	protein_coding	ENSG00000162517	-0,439582009	1,25E-12	1,1E-11
NELFB	protein_coding	ENSG00000188986	-0,46698255	1,25E-12	1,1E-11
ESPL1	protein_coding	ENSG00000135476	-0,635095115	1,24E-12	1,1E-11
SLC12A7	protein_coding	ENSG00000113504	0,747400886	1,26E-12	1,11E-11
TRAFD1	protein_coding	ENSG00000135148	0,485147496	1,28E-12	1,13E-11
IL13RA1	protein_coding	ENSG00000131724	0,344106292	1,32E-12	1,16E-11
SERTAD1	protein_coding	ENSG00000197019	0,553932567	1,35E-12	1,18E-11
MRPL20-AS1	processed_transcript	ENSG00000224870	-0,658295472	1,35E-12	1,19E-11
LGMN	protein_coding	ENSG00000100600	0,419394237	1,37E-12	1,2E-11
LIPH	protein_coding	ENSG00000163898	1,953096384	1,37E-12	1,21E-11
SNRNP48	protein_coding	ENSG00000168566	-0,415103981	1,39E-12	1,22E-11
SLC49A4	protein_coding	ENSG00000138463	0,315850377	1,43E-12	1,25E-11
YPEL5	protein_coding	ENSG00000119801	0,426622087	1,45E-12	1,27E-11
ANXA5	protein_coding	ENSG00000164111	0,344043057	1,45E-12	1,27E-11
ERC1	protein_coding	ENSG00000082805	0,419279849	1,48E-12	1,29E-11
AC009549.1	lincRNA	ENSG00000270607	1,175018086	1,5E-12	1,31E-11
SYT9	protein_coding	ENSG00000170743	1,040856259	1,52E-12	1,33E-11
ITGB2	protein_coding	ENSG00000160255	0,656412395	1,52E-12	1,33E-11
HOXC13	protein_coding	ENSG00000123364	0,771075151	1,53E-12	1,34E-11
FTH1	protein_coding	ENSG00000167996	0,328540035	1,53E-12	1,34E-11
CDCP1	protein_coding	ENSG00000163814	-0,400795928	1,54E-12	1,34E-11

PWWP3A	protein_coding	ENSG00000160953	-0,514532508	1,54E-12	1,34E-11
LINC00370	processed_transcript	ENSG00000223617	3,707624744	1,57E-12	1,37E-11
GMCL1	protein_coding	ENSG00000087338	-0,472287755	1,57E-12	1,37E-11
PPRC1	protein_coding	ENSG00000148840	-0,475430492	1,57E-12	1,37E-11
TMEM101	protein_coding	ENSG00000091947	-0,520822228	1,57E-12	1,37E-11
RRP1B	protein_coding	ENSG00000160208	-0,433857627	1,59E-12	1,39E-11
EML2-AS1	processed_transcript	ENSG00000267757	1,345466963	1,63E-12	1,42E-11
FAM20B	protein_coding	ENSG00000116199	-0,375549746	1,63E-12	1,42E-11
GOLGA5	protein_coding	ENSG00000066455	0,390111699	1,66E-12	1,44E-11
PSD3	protein_coding	ENSG00000156011	-0,420448526	1,65E-12	1,44E-11
CDK7	protein_coding	ENSG00000134058	0,383697873	1,69E-12	1,47E-11
TENT2	protein_coding	ENSG00000164329	0,379058457	1,7E-12	1,48E-11
WDR46	protein_coding	ENSG00000227057	-0,351318783	1,72E-12	1,5E-11
SFT2D2	protein_coding	ENSG00000213064	-0,478088131	1,73E-12	1,5E-11
GADD45A	protein_coding	ENSG00000116717	0,283900964	1,75E-12	1,52E-11
AC009133.1	antisense	ENSG00000238045	2,332495315	1,77E-12	1,54E-11
SRP68	protein_coding	ENSG00000167881	0,330159666	1,86E-12	1,62E-11
PFAS	protein_coding	ENSG00000178921	-0,576063047	1,88E-12	1,63E-11
STMN3	protein_coding	ENSG00000197457	-0,553658906	1,89E-12	1,64E-11
C6orf120	protein_coding	ENSG00000185127	-0,4868492	1,91E-12	1,66E-11
ALG2	protein_coding	ENSG00000119523	0,415514783	1,96E-12	1,69E-11
HIST1H2BB	protein_coding	ENSG00000276410	-0,613823903	1,99E-12	1,72E-11
FBXW8	protein_coding	ENSG00000174989	0,571316114	2E-12	1,73E-11
WDFY2	protein_coding	ENSG00000139668	-0,303801526	2,01E-12	1,74E-11
RIC1	protein_coding	ENSG00000107036	0,4252692	2,03E-12	1,75E-11
C1orf21	protein_coding	ENSG00000116667	-0,408395033	2,06E-12	1,78E-11
HLA-L	transcribed_unprocesse	ENSG00000243753	1,246377076	2,06E-12	1,78E-11
HIST2H4A	protein_coding	ENSG00000270882	2,245576078	2,07E-12	1,79E-11
TTLL6	protein_coding	ENSG00000170703	1,299042052	2,08E-12	1,8E-11
FADS1	protein_coding	ENSG00000149485	0,303639055	2,09E-12	1,81E-11
THAP7	protein_coding	ENSG00000184436	-0,526061714	2,12E-12	1,83E-11
RTL10	protein_coding	ENSG00000215012	-0,524282197	2,13E-12	1,84E-11
GRHL3	protein_coding	ENSG00000158055	5,114580682	2,18E-12	1,88E-11
DIPK1B	protein_coding	ENSG00000165716	-0,607432197	2,24E-12	1,93E-11
RBL2	protein_coding	ENSG00000103479	-0,472505101	2,25E-12	1,94E-11
SMIM10	protein_coding	ENSG00000184785	-0,54087773	2,27E-12	1,96E-11
CCDC82	protein_coding	ENSG00000149231	0,544557886	2,31E-12	1,99E-11
PUM2	protein_coding	ENSG00000055917	-0,40351249	2,31E-12	1,99E-11
PUS7	protein_coding	ENSG00000091127	-0,390100884	2,39E-12	2,06E-11
FGF5	protein_coding	ENSG00000138675	-0,435572301	2,48E-12	2,13E-11

USP29	protein_coding	ENSG00000131864	5,924734813	2,49E-12	2,14E-11
DENND6A	protein_coding	ENSG00000174839	-0,512607878	2,49E-12	2,14E-11
MBD5	protein_coding	ENSG00000204406	0,578721458	2,52E-12	2,16E-11
FBXL12	protein_coding	ENSG00000127452	0,54765027	2,54E-12	2,18E-11
ERBB2	protein_coding	ENSG00000141736	-0,525703513	2,54E-12	2,18E-11
TRIML2	protein_coding	ENSG00000179046	1,19901705	2,56E-12	2,19E-11
BUD13	protein_coding	ENSG00000137656	-0,471187443	2,59E-12	2,22E-11
FANCB	protein_coding	ENSG00000181544	-0,480566259	2,59E-12	2,22E-11
AC129492.1	protein_coding	ENSG00000179094	0,892182767	2,64E-12	2,26E-11
DGKH	protein_coding	ENSG00000102780	0,463572011	2,68E-12	2,3E-11
CXCL10	protein_coding	ENSG00000169245	7,812059544	2,71E-12	2,31E-11
CHSY1	protein_coding	ENSG00000131873	-0,413031107	2,71E-12	2,31E-11
MED22	protein_coding	ENSG00000148297	-0,570180051	2,7E-12	2,31E-11
VWF	protein_coding	ENSG00000110799	2,439884464	2,75E-12	2,35E-11
PLSCR4	protein_coding	ENSG00000114698	0,477304338	2,75E-12	2,35E-11
CCSAP	protein_coding	ENSG00000154429	-0,496840729	2,76E-12	2,35E-11
COQ3	protein_coding	ENSG00000132423	-0,664453967	2,79E-12	2,38E-11
UAP1	protein_coding	ENSG00000117143	-0,388338794	2,83E-12	2,41E-11
BRCA1	protein_coding	ENSG00000012048	-0,422536149	2,85E-12	2,43E-11
SEC61A1	protein_coding	ENSG00000058262	0,355465281	2,86E-12	2,44E-11
TSGA10	protein_coding	ENSG00000135951	0,991775832	2,89E-12	2,46E-11
MDM2	protein_coding	ENSG00000135679	-0,376306365	2,9E-12	2,47E-11
FBXW7	protein_coding	ENSG00000109670	0,44762722	3,04E-12	2,59E-11
MON2	protein_coding	ENSG00000061987	0,322167943	3,06E-12	2,6E-11
TUBD1	protein_coding	ENSG00000108423	-0,617339106	3,1E-12	2,64E-11
ARHGEF1	protein_coding	ENSG00000076928	-0,526052728	3,14E-12	2,67E-11
RAD51AP1	protein_coding	ENSG00000111247	-0,579491279	3,18E-12	2,7E-11
NTM	protein_coding	ENSG00000182667	0,50510816	3,22E-12	2,74E-11
ARVCF	protein_coding	ENSG00000099889	0,858785117	3,25E-12	2,76E-11
FBXL2	protein_coding	ENSG00000153558	-0,562762295	3,27E-12	2,77E-11
ENO1	protein_coding	ENSG00000074800	-0,340975229	3,31E-12	2,81E-11
MMRN2	protein_coding	ENSG00000173269	4,371559536	3,39E-12	2,88E-11
GIN1	protein_coding	ENSG00000145723	-0,701020044	3,39E-12	2,88E-11
NELFA	protein_coding	ENSG00000185049	-0,374843416	3,47E-12	2,94E-11
PNPLA8	protein_coding	ENSG00000135241	0,486962746	3,5E-12	2,96E-11
ABCA12	protein_coding	ENSG00000144452	2,428620217	3,51E-12	2,97E-11
DUSP8	protein_coding	ENSG00000184545	2,026492442	3,54E-12	2,99E-11
LIG4	protein_coding	ENSG00000174405	0,552702195	3,64E-12	3,08E-11
OAS1	protein_coding	ENSG00000089127	1,714941185	3,66E-12	3,1E-11
BOK	protein_coding	ENSG00000176720	-0,576833034	3,87E-12	3,27E-11

LINC02101	lincRNA	ENSG00000248132	6,156099907	3,88E-12	3,28E-11
C19orf48	protein_coding	ENSG00000167747	-0,52272607	3,89E-12	3,28E-11
GNG5	protein_coding	ENSG00000174021	0,369261922	3,91E-12	3,3E-11
AL365181.3	antisense	ENSG00000272405	1,022557516	3,92E-12	3,31E-11
SLC2A12	protein_coding	ENSG00000146411	0,639118513	3,94E-12	3,32E-11
FANCD2	protein_coding	ENSG00000144554	-0,337128757	3,98E-12	3,35E-11
KRT34	protein_coding	ENSG00000131737	0,476670435	3,99E-12	3,36E-11
SLC1A3	protein_coding	ENSG00000079215	4,674748831	4E-12	3,37E-11
SGO2	protein_coding	ENSG00000163535	-0,628184385	4,01E-12	3,38E-11
SERBP1	protein_coding	ENSG00000142864	-0,363664067	4,04E-12	3,4E-11
MORF4L2	protein_coding	ENSG00000123562	-0,405997208	4,05E-12	3,41E-11
PHF5A	protein_coding	ENSG00000100410	-0,485757811	4,09E-12	3,44E-11
URGCP	protein_coding	ENSG00000106608	-0,385741449	4,1E-12	3,45E-11
NR4A2	protein_coding	ENSG00000153234	1,332460379	4,12E-12	3,46E-11
ZBTB38	protein_coding	ENSG00000177311	-0,428569984	4,15E-12	3,48E-11
PNPLA3	protein_coding	ENSG00000100344	0,857534642	4,27E-12	3,58E-11
MED11	protein_coding	ENSG00000161920	-0,612908049	4,34E-12	3,64E-11
FILIP1	protein_coding	ENSG00000118407	2,030893087	4,41E-12	3,7E-11
VPS28	protein_coding	ENSG00000160948	-0,375647517	4,42E-12	3,7E-11
MAPRE3	protein_coding	ENSG00000084764	0,858085436	4,46E-12	3,73E-11
ANKMY2	protein_coding	ENSG00000106524	-0,47025492	4,46E-12	3,74E-11
CMBL	protein_coding	ENSG00000164237	0,409070418	4,48E-12	3,75E-11
DLG1	protein_coding	ENSG00000075711	-0,32257759	4,51E-12	3,77E-11
AL161421.1	antisense	ENSG00000275202	0,757435185	4,54E-12	3,8E-11
PPM1K	protein_coding	ENSG00000163644	0,676005919	4,58E-12	3,83E-11
TCERG1	protein_coding	ENSG00000113649	-0,477008177	4,58E-12	3,83E-11
FAM160B1	protein_coding	ENSG00000151553	-0,466436001	4,64E-12	3,88E-11
EGR2	protein_coding	ENSG00000122877	4,60347572	4,73E-12	3,95E-11
FAM72A	protein_coding	ENSG00000196550	-0,623609567	4,88E-12	4,07E-11
TMOD3	protein_coding	ENSG00000138594	-0,301747804	4,91E-12	4,1E-11
NCK1	protein_coding	ENSG00000158092	-0,472250739	4,93E-12	4,12E-11
ADAT2	protein_coding	ENSG00000189007	-0,714011026	4,98E-12	4,16E-11
ACAD8	protein_coding	ENSG00000151498	-0,440787685	5,01E-12	4,17E-11
AC007879.3	lincRNA	ENSG00000234902	1,989524804	5,04E-12	4,2E-11
PIGM	protein_coding	ENSG00000143315	-0,548328051	5,1E-12	4,25E-11
RPL32P3	transcribed_unprocesse	ENSG00000251474	0,685842049	5,22E-12	4,35E-11
KDM2A	protein_coding	ENSG00000173120	0,52928642	5,3E-12	4,41E-11
MRPS34	protein_coding	ENSG00000074071	-0,485330359	5,3E-12	4,41E-11
TRIM36	protein_coding	ENSG00000152503	0,726101498	5,33E-12	4,43E-11
TRPS1	protein_coding	ENSG00000104447	0,70182018	5,34E-12	4,44E-11

SFPQ	protein_coding	ENSG00000116560	-0,355745813	5,35E-12	4,45E-11
AL391832.2	lincRNA	ENSG00000238005	2,678534703	5,37E-12	4,46E-11
PDCD7	protein_coding	ENSG00000090470	-0,328298192	5,4E-12	4,48E-11
TDRD9	protein_coding	ENSG00000156414	0,953651514	5,41E-12	4,49E-11
PPP1R35	protein_coding	ENSG00000160813	-0,546972976	5,46E-12	4,53E-11
DNAAF5	protein_coding	ENSG00000164818	-0,42638988	5,48E-12	4,54E-11
MIIP	protein_coding	ENSG00000116691	-0,55032484	5,47E-12	4,54E-11
XPO7	protein_coding	ENSG00000130227	-0,346565542	5,51E-12	4,57E-11
LYPD6	protein_coding	ENSG00000187123	-0,663059043	5,59E-12	4,63E-11
PHF7	protein_coding	ENSG00000010318	-0,536698686	5,62E-12	4,65E-11
ZNF714	protein_coding	ENSG00000160352	-0,578961093	5,65E-12	4,68E-11
RNU5A-8P	snRNA	ENSG00000200972	-1,213772019	5,69E-12	4,7E-11
MTFR2	protein_coding	ENSG00000146410	-0,613060269	5,7E-12	4,71E-11
PAK1IP1	protein_coding	ENSG00000111845	-0,484597608	5,71E-12	4,72E-11
NINJ1	protein_coding	ENSG00000131669	0,676505433	5,73E-12	4,74E-11
AP002833.2	antisense	ENSG00000255317	1,566964663	5,75E-12	4,75E-11
ZSCAN31	protein_coding	ENSG00000235109	1,054255097	5,88E-12	4,86E-11
GRIN2C	protein_coding	ENSG00000161509	1,550338124	5,97E-12	4,93E-11
TOR1A	protein_coding	ENSG00000136827	0,340443997	5,98E-12	4,94E-11
PYY	protein_coding	ENSG00000131096	1,882057779	6E-12	4,95E-11
NOP14	protein_coding	ENSG00000087269	-0,378595685	6,03E-12	4,97E-11
CREB3	protein_coding	ENSG00000107175	0,516790132	6,18E-12	5,09E-11
MFAP5	protein_coding	ENSG00000197614	-0,621745196	6,27E-12	5,17E-11
GID4	protein_coding	ENSG00000141034	-0,418574608	6,36E-12	5,24E-11
AKAP3	protein_coding	ENSG00000111254	2,303646506	6,38E-12	5,25E-11
ANKRD36B	protein_coding	ENSG00000196912	-0,74656236	6,39E-12	5,26E-11
RPLP0P2	transcribed_processed_	ENSG00000243742	1,178128678	6,39E-12	5,26E-11
RNU4-1	snRNA	ENSG00000200795	-0,662819839	6,48E-12	5,32E-11
ACVR2A	protein_coding	ENSG00000121989	0,473178132	6,53E-12	5,36E-11
S1PR5	protein_coding	ENSG00000180739	-0,861942446	6,55E-12	5,38E-11
EMC3	protein_coding	ENSG00000125037	0,381956799	6,65E-12	5,46E-11
ELOA	protein_coding	ENSG00000011007	-0,310184849	6,65E-12	5,46E-11
RFC3	protein_coding	ENSG00000133119	-0,508304161	6,68E-12	5,48E-11
AC023157.1	processed_pseudogene	ENSG00000223722	2,131252714	6,74E-12	5,53E-11
YPEL1	protein_coding	ENSG00000100027	-0,85807726	6,76E-12	5,54E-11
HS6ST1	protein_coding	ENSG00000136720	-0,505960053	6,83E-12	5,6E-11
LRRC58	protein_coding	ENSG00000163428	-0,614725322	7,02E-12	5,75E-11
LINC02432	lincRNA	ENSG00000248810	0,492970668	7,05E-12	5,77E-11
CCDC30	protein_coding	ENSG00000186409	1,015960034	7,09E-12	5,8E-11
FHL1	protein_coding	ENSG00000022267	0,265791971	7,19E-12	5,88E-11

ZNF641	protein_coding	ENSG00000167528	0,712127735	7,25E-12	5,93E-11
UBQLN4	protein_coding	ENSG00000160803	-0,41086186	7,27E-12	5,94E-11
COG7	protein_coding	ENSG00000168434	-0,553728188	7,38E-12	6,03E-11
ODR4	protein_coding	ENSG00000157181	0,413172105	7,46E-12	6,09E-11
AMD1	protein_coding	ENSG00000123505	-0,458528159	7,45E-12	6,09E-11
CNIH3	protein_coding	ENSG00000143786	0,748620163	7,54E-12	6,15E-11
PRKD2	protein_coding	ENSG00000105287	0,540202522	7,62E-12	6,22E-11
RBM39	protein_coding	ENSG00000131051	-0,343193014	7,69E-12	6,27E-11
PLXNA3	protein_coding	ENSG00000130827	1,011771355	7,72E-12	6,29E-11
LSM7	protein_coding	ENSG00000130332	-0,514972432	7,91E-12	6,44E-11
BARD1	protein_coding	ENSG00000138376	-0,515623768	7,9E-12	6,44E-11
AC073130.3	TEC	ENSG00000279086	2,156005487	7,96E-12	6,48E-11
FAM20C	protein_coding	ENSG00000177706	-0,890425283	8,01E-12	6,51E-11
RBMX	protein_coding	ENSG00000147274	-0,412561007	8,06E-12	6,55E-11
MTHFR	protein_coding	ENSG00000177000	0,717161419	8,08E-12	6,57E-11
GAS2L1	protein_coding	ENSG00000185340	-0,611364888	8,11E-12	6,59E-11
SLCO4A1	protein_coding	ENSG00000101187	-0,51147033	8,13E-12	6,61E-11
CTR9	protein_coding	ENSG00000198730	0,32167758	8,17E-12	6,63E-11
BRD3	protein_coding	ENSG00000169925	-0,574269401	8,16E-12	6,63E-11
CFB	protein_coding	ENSG00000243649	7,643164838	8,19E-12	6,64E-11
AL050341.2	antisense	ENSG00000259943	-1,00126808	8,3E-12	6,73E-11
CDKN3	protein_coding	ENSG00000100526	-0,540118418	8,35E-12	6,77E-11
ROBO1	protein_coding	ENSG00000169855	0,453769996	8,4E-12	6,81E-11
UGGT1	protein_coding	ENSG00000136731	0,446755575	8,44E-12	6,83E-11
MRAS	protein_coding	ENSG00000158186	0,468054665	8,48E-12	6,87E-11
TOMM20	protein_coding	ENSG00000173726	-0,343532255	8,61E-12	6,97E-11
ANP32B	protein_coding	ENSG00000136938	-0,475812014	8,64E-12	6,99E-11
KCTD11	protein_coding	ENSG00000213859	0,545296387	8,99E-12	7,27E-11
AC116366.1	antisense	ENSG00000234290	3,583575953	9,24E-12	7,47E-11
MAPK8IP2	protein_coding	ENSG00000008735	0,737420452	9,25E-12	7,47E-11
S100A2	protein_coding	ENSG00000196754	0,935592829	9,3E-12	7,51E-11
FAM219A	protein_coding	ENSG00000164970	0,590574634	9,38E-12	7,57E-11
CDIP1	protein_coding	ENSG00000089486	0,646334691	9,52E-12	7,68E-11
PLPP2	protein_coding	ENSG00000141934	-0,368596584	9,63E-12	7,77E-11
PAXIP1	protein_coding	ENSG00000157212	-0,488185508	9,66E-12	7,79E-11
HM13	protein_coding	ENSG00000101294	0,343461481	9,84E-12	7,94E-11
AC006329.1	antisense	ENSG00000232445	-0,812855038	9,91E-12	7,98E-11
ABHD5	protein_coding	ENSG00000011198	0,381747393	9,95E-12	8,01E-11
CCNL1	protein_coding	ENSG00000163660	0,561019686	1,01E-11	8,12E-11
CAMSAP2	protein_coding	ENSG00000118200	0,387517198	1,02E-11	8,2E-11

MME	protein_coding	ENSG00000196549	-0,300532435	1,02E-11	8,25E-11
DGAT2	protein_coding	ENSG00000062282	-0,995617849	1,03E-11	8,25E-11
IFNL2	protein_coding	ENSG00000183709	7,589418281	1,03E-11	8,26E-11
TCTN3	protein_coding	ENSG00000119977	-0,343628029	1,04E-11	8,33E-11
SAYSD1	protein_coding	ENSG00000112167	-0,483982346	1,07E-11	8,61E-11
AL450384.2	antisense	ENSG00000240291	1,691326859	1,07E-11	8,62E-11
SLC6A7	protein_coding	ENSG00000011083	6,147448921	1,08E-11	8,65E-11
AC007114.1	lincRNA	ENSG00000263004	1,497040392	1,08E-11	8,69E-11
TCP11L2	protein_coding	ENSG00000166046	0,783987437	1,09E-11	8,7E-11
KRT18P55	transcribed_unprocesse	ENSG00000265480	1,030676589	1,1E-11	8,84E-11
IRF2BP1	protein_coding	ENSG00000170604	-0,60891648	1,1E-11	8,85E-11
GOSR2	protein_coding	ENSG00000108433	0,298789063	1,11E-11	8,88E-11
MIR210HG	lincRNA	ENSG00000247095	-1,549173624	1,12E-11	8,97E-11
ELP5	protein_coding	ENSG00000170291	-0,416307204	1,14E-11	9,09E-11
CPT2	protein_coding	ENSG00000157184	0,469681402	1,15E-11	9,23E-11
SNRPE	protein_coding	ENSG00000182004	-0,43843174	1,17E-11	9,33E-11
NADSYN1	protein_coding	ENSG00000172890	0,43733732	1,17E-11	9,37E-11
HSD17B12	protein_coding	ENSG00000149084	0,450337783	1,18E-11	9,46E-11
COG3	protein_coding	ENSG00000136152	0,423657159	1,18E-11	9,46E-11
ANKRD33B	protein_coding	ENSG00000164236	0,665779098	1,19E-11	9,54E-11
SUN1	protein_coding	ENSG00000164828	-0,367282898	1,2E-11	9,55E-11
NIPAL4	protein_coding	ENSG00000172548	1,287439097	1,21E-11	9,65E-11
TSEN2	protein_coding	ENSG00000154743	-0,559247706	1,21E-11	9,66E-11
USP54	protein_coding	ENSG00000166348	0,488365995	1,23E-11	9,84E-11
SASH1	protein_coding	ENSG00000111961	0,402462695	1,24E-11	9,86E-11
NBEAP1	transcribed_unprocesse	ENSG00000258590	0,927926945	1,24E-11	9,86E-11
INTS11	protein_coding	ENSG00000127054	-0,325447665	1,24E-11	9,89E-11
ABCA7	protein_coding	ENSG00000064687	0,977203124	1,26E-11	1E-10
AC060766.1	transcribed_processed_	ENSG00000267102	0,700755443	1,27E-11	1,01E-10
RNU6-2	snRNA	ENSG00000207357	-1,701112377	1,28E-11	1,02E-10
SMARCA5	protein_coding	ENSG00000153147	0,224938106	1,29E-11	1,03E-10
DAZAP1	protein_coding	ENSG00000071626	-0,255611745	1,31E-11	1,04E-10
NUP160	protein_coding	ENSG00000030066	-0,368928398	1,31E-11	1,04E-10
SUN2	protein_coding	ENSG00000100242	-0,554524975	1,33E-11	1,05E-10
AL662795.2	TEC	ENSG00000280128	1,253522005	1,32E-11	1,05E-10
NLRX1	protein_coding	ENSG00000160703	0,594777521	1,33E-11	1,06E-10
POLR2D	protein_coding	ENSG00000144231	-0,260017183	1,33E-11	1,06E-10
CREM	protein_coding	ENSG00000095794	0,524907154	1,37E-11	1,08E-10
SPEN	protein_coding	ENSG00000065526	0,47410218	1,36E-11	1,08E-10
ABL1	protein_coding	ENSG00000097007	-0,455632919	1,38E-11	1,1E-10

AL138724.1	antisense	ENSG00000272269	0,963916364	1,4E-11	1,11E-10
CES2	protein_coding	ENSG00000172831	0,519210263	1,4E-11	1,11E-10
CAMK2G	protein_coding	ENSG00000148660	-0,287681912	1,42E-11	1,12E-10
NCBP2-AS2	protein_coding	ENSG00000270170	-0,610115846	1,42E-11	1,12E-10
GLS2	protein_coding	ENSG00000135423	7,687997901	1,44E-11	1,14E-10
LUCAT1	antisense	ENSG00000248323	1,085768393	1,47E-11	1,17E-10
FAM136A	protein_coding	ENSG00000035141	-0,3313991	1,48E-11	1,17E-10
FAM131A	protein_coding	ENSG00000175182	-0,571825171	1,5E-11	1,18E-10
TBX19	protein_coding	ENSG00000143178	1,240733526	1,51E-11	1,19E-10
LPAR3	protein_coding	ENSG00000171517	-0,308781245	1,51E-11	1,19E-10
MOGS	protein_coding	ENSG00000115275	-0,450657357	1,5E-11	1,19E-10
ASPM	protein_coding	ENSG00000066279	-0,598593962	1,56E-11	1,23E-10
CREB5	protein_coding	ENSG00000146592	3,30514952	1,57E-11	1,24E-10
BATF3	protein_coding	ENSG00000123685	-0,4516331	1,59E-11	1,26E-10
PODXL	protein_coding	ENSG00000128567	0,366635487	1,63E-11	1,28E-10
HDAC3	protein_coding	ENSG00000171720	-0,287689861	1,66E-11	1,31E-10
EGR3	protein_coding	ENSG00000179388	3,775047453	1,68E-11	1,32E-10
HHEX	protein_coding	ENSG00000152804	-0,6746844	1,67E-11	1,32E-10
ABCA10	protein_coding	ENSG00000154263	2,010491003	1,7E-11	1,34E-10
DNAJB12	protein_coding	ENSG00000148719	-0,352942556	1,71E-11	1,34E-10
ANXA2	protein_coding	ENSG00000182718	-0,393931953	1,7E-11	1,34E-10
RBM4B	protein_coding	ENSG00000173914	-0,478956697	1,7E-11	1,34E-10
CKM	protein_coding	ENSG00000104879	7,590786748	1,72E-11	1,35E-10
N6AMT1	protein_coding	ENSG00000156239	-0,471657651	1,74E-11	1,36E-10
FBLN2	protein_coding	ENSG00000163520	2,888879272	1,8E-11	1,42E-10
METAP1	protein_coding	ENSG00000164024	-0,365088459	1,81E-11	1,42E-10
RP9	protein_coding	ENSG00000164610	0,535330744	1,82E-11	1,43E-10
LINC01426	lincRNA	ENSG00000234380	0,951835654	1,84E-11	1,44E-10
HIST1H4I	protein_coding	ENSG00000276180	2,281611496	1,84E-11	1,44E-10
SAP30L	protein_coding	ENSG00000164576	-0,396039566	1,84E-11	1,44E-10
HOXA13	protein_coding	ENSG00000106031	-1,257732541	1,84E-11	1,44E-10
ACVR1B	protein_coding	ENSG00000135503	-0,450402794	1,85E-11	1,45E-10
FUT4	protein_coding	ENSG00000196371	-0,674264172	1,88E-11	1,47E-10
OCRL	protein_coding	ENSG00000122126	0,299612916	1,89E-11	1,48E-10
RRAGA	protein_coding	ENSG00000155876	-0,297767578	1,91E-11	1,49E-10
RIC8A	protein_coding	ENSG00000177963	-0,310641938	1,93E-11	1,51E-10
AC021218.1	lincRNA	ENSG00000204876	3,699329152	1,96E-11	1,53E-10
DHX15	protein_coding	ENSG00000109606	-0,373309877	1,97E-11	1,54E-10
SPATS2	protein_coding	ENSG00000123352	0,328446197	1,99E-11	1,55E-10
CDCA5	protein_coding	ENSG00000146670	-0,357197877	1,99E-11	1,55E-10

AP002008.1	antisense	ENSG00000254980	1,968659652	1,99E-11	1,56E-10
SLC7A7	protein_coding	ENSG00000155465	2,160542613	2,01E-11	1,56E-10
EPOR	protein_coding	ENSG00000187266	-0,510938896	2E-11	1,56E-10
USP19	protein_coding	ENSG00000172046	-0,401735047	2,02E-11	1,58E-10
DNAJC25	protein_coding	ENSG00000059769	0,542682426	2,05E-11	1,6E-10
DTYMK	protein_coding	ENSG00000168393	-0,451964584	2,06E-11	1,6E-10
RGS5	protein_coding	ENSG00000143248	0,605543631	2,08E-11	1,61E-10
NAE1	protein_coding	ENSG00000159593	-0,479934982	2,11E-11	1,64E-10
FAM8A1	protein_coding	ENSG00000137414	0,318023694	2,15E-11	1,67E-10
AC108676.1	sense_overlapping	ENSG00000244675	1,459884901	2,15E-11	1,67E-10
LINC01119	lincRNA	ENSG00000239332	1,411291802	2,16E-11	1,68E-10
TONSL	protein_coding	ENSG00000160949	-0,60813774	2,19E-11	1,7E-10
FXR2	protein_coding	ENSG00000129245	0,336667842	2,2E-11	1,71E-10
IRF7	protein_coding	ENSG00000185507	0,864500531	2,24E-11	1,74E-10
DCUN1D2	protein_coding	ENSG00000150401	0,561272316	2,25E-11	1,74E-10
ADPRHL2	protein_coding	ENSG00000116863	-0,399459651	2,26E-11	1,75E-10
DEK	protein_coding	ENSG00000124795	-0,51190679	2,27E-11	1,76E-10
PNPLA2	protein_coding	ENSG00000177666	0,73262227	2,29E-11	1,77E-10
ING3	protein_coding	ENSG00000071243	0,509756341	2,3E-11	1,78E-10
GNB4	protein_coding	ENSG00000114450	-0,447840976	2,3E-11	1,78E-10
MESD	protein_coding	ENSG00000117899	-0,346700976	2,31E-11	1,79E-10
PQLC3	protein_coding	ENSG00000162976	-0,46917065	2,32E-11	1,8E-10
SEMA3B	protein_coding	ENSG00000012171	1,824433413	2,35E-11	1,81E-10
MPST	protein_coding	ENSG00000128309	-0,435200506	2,35E-11	1,81E-10
TARDBP	protein_coding	ENSG00000120948	-0,470608197	2,35E-11	1,82E-10
RNASE7	protein_coding	ENSG00000165799	7,473665347	2,37E-11	1,83E-10
SPATA12	protein_coding	ENSG00000186451	1,887693167	2,4E-11	1,85E-10
PLS1	protein_coding	ENSG00000120756	0,484427544	2,39E-11	1,85E-10
IKBKB	protein_coding	ENSG00000104365	-0,369582853	2,41E-11	1,86E-10
NAA40	protein_coding	ENSG00000110583	-0,423235333	2,43E-11	1,87E-10
ATG4A	protein_coding	ENSG00000101844	0,42445351	2,47E-11	1,9E-10
SNAPC3	protein_coding	ENSG00000164975	0,383607297	2,46E-11	1,9E-10
SENP2	protein_coding	ENSG00000163904	0,258013218	2,47E-11	1,91E-10
CYB5R1	protein_coding	ENSG00000159348	0,429590979	2,5E-11	1,92E-10
AL132780.2	antisense	ENSG00000258457	1,536777649	2,55E-11	1,97E-10
MALSU1	protein_coding	ENSG00000156928	-0,420536463	2,57E-11	1,98E-10
RNU1-13P	snRNA	ENSG00000238825	-3,637397449	2,61E-11	2,01E-10
PLPBP	protein_coding	ENSG00000147471	0,340190119	2,63E-11	2,02E-10
SKA1	protein_coding	ENSG00000154839	-0,405257852	2,63E-11	2,02E-10
TRAM1	protein_coding	ENSG00000067167	0,452824242	2,67E-11	2,05E-10

REELD1	protein_coding	ENSG00000250673	3,882715598	2,71E-11	2,08E-10
GFM2	protein_coding	ENSG00000164347	0,34047196	2,71E-11	2,08E-10
TUSC1	protein_coding	ENSG00000198680	-0,491052017	2,7E-11	2,08E-10
HLA-K	unprocessed_pseudoge	ENSG00000230795	2,206251127	2,71E-11	2,08E-10
CEP350	protein_coding	ENSG00000135837	0,39104124	2,72E-11	2,09E-10
ROM1	protein_coding	ENSG00000149489	1,566375983	2,75E-11	2,1E-10
MTHFD1	protein_coding	ENSG00000100714	-0,341985593	2,74E-11	2,1E-10
PRCP	protein_coding	ENSG00000137509	0,356233052	2,77E-11	2,12E-10
SUPT7L	protein_coding	ENSG00000119760	0,320374487	2,78E-11	2,13E-10
GREB1	protein_coding	ENSG00000196208	0,816668349	2,8E-11	2,14E-10
CACNG6	protein_coding	ENSG00000130433	0,758065645	2,8E-11	2,14E-10
KIF9-AS1	antisense	ENSG00000227398	1,019406418	2,83E-11	2,16E-10
NXT1	protein_coding	ENSG00000132661	-0,566150152	2,84E-11	2,17E-10
RTN4R	protein_coding	ENSG00000040608	-0,844629936	2,86E-11	2,19E-10
MET	protein_coding	ENSG00000105976	-0,350643825	2,89E-11	2,21E-10
NCOA5	protein_coding	ENSG00000124160	-0,488021217	2,9E-11	2,22E-10
EIF4E2	protein_coding	ENSG00000135930	-0,427032267	2,99E-11	2,28E-10
ZNF141	protein_coding	ENSG00000131127	-0,832466435	2,99E-11	2,28E-10
PAQR8	protein_coding	ENSG00000170915	0,650291885	3E-11	2,29E-10
ZRSR2	protein_coding	ENSG00000169249	0,615824382	3E-11	2,29E-10
RGMB-AS1	antisense	ENSG00000246763	1,831554093	3,02E-11	2,31E-10
LETMD1	protein_coding	ENSG00000050426	-0,311339865	3,02E-11	2,31E-10
PAPSS1	protein_coding	ENSG00000138801	-0,366099728	3,04E-11	2,32E-10
SLC25A15	protein_coding	ENSG00000102743	-0,449328961	3,04E-11	2,32E-10
ITGA3	protein_coding	ENSG00000005884	-0,388788858	3,07E-11	2,34E-10
LRIG1	protein_coding	ENSG00000144749	-0,476475497	3,07E-11	2,34E-10
TMEM182	protein_coding	ENSG00000170417	0,66957425	3,1E-11	2,36E-10
RAET1K	transcribed_unprocesse	ENSG00000218358	2,660661611	3,13E-11	2,38E-10
LRRC45	protein_coding	ENSG00000169683	-0,556363284	3,15E-11	2,39E-10
UBQLN1	protein_coding	ENSG00000135018	0,271432373	3,16E-11	2,4E-10
DUSP18	protein_coding	ENSG00000167065	-0,584593689	3,18E-11	2,42E-10
EXOC3	protein_coding	ENSG00000180104	-0,298669037	3,2E-11	2,43E-10
CD99P1	transcribed_unprocesse	ENSG00000223773	0,820787522	3,34E-11	2,54E-10
DLGAP1-AS2	antisense	ENSG00000262001	0,659791273	3,39E-11	2,57E-10
SHC4	protein_coding	ENSG00000185634	1,94794537	3,41E-11	2,58E-10
SYNJ1	protein_coding	ENSG00000159082	0,457797488	3,4E-11	2,58E-10
AL139124.1	antisense	ENSG00000273450	2,53163294	3,48E-11	2,64E-10
NUP153	protein_coding	ENSG00000124789	-0,344073277	3,49E-11	2,64E-10
LPCAT4	protein_coding	ENSG00000176454	-0,691569153	3,48E-11	2,64E-10
TMEM97	protein_coding	ENSG00000109084	-0,432808046	3,49E-11	2,65E-10

ENTR1	protein_coding	ENSG00000165689	-0,270298385	3,53E-11	2,67E-10
CNBP	protein_coding	ENSG00000169714	-0,434239791	3,52E-11	2,67E-10
ZSCAN30	protein_coding	ENSG00000186814	0,434547684	3,54E-11	2,68E-10
RRM1	protein_coding	ENSG00000167325	-0,360826408	3,61E-11	2,73E-10
LTB	protein_coding	ENSG00000227507	5,658420904	3,64E-11	2,75E-10
FAM111A-DT	lincRNA	ENSG00000245571	0,562242172	3,69E-11	2,79E-10
PLEKHJ1	protein_coding	ENSG00000104886	-0,463363171	3,74E-11	2,82E-10
LINC02535	lincRNA	ENSG00000234155	0,59061298	3,74E-11	2,83E-10
MEN1	protein_coding	ENSG00000133895	-0,469249718	3,78E-11	2,85E-10
ZNF624	protein_coding	ENSG00000197566	-0,560981461	3,78E-11	2,85E-10
FAAHP1	transcribed_unprocesse	ENSG00000232022	0,972511158	3,87E-11	2,92E-10
SPOUT1	protein_coding	ENSG00000198917	-0,436745528	3,89E-11	2,93E-10
RGS19	protein_coding	ENSG00000171700	-0,514032168	3,88E-11	2,93E-10
AC073508.3	antisense	ENSG00000278834	-0,72206285	3,97E-11	2,99E-10
PHLDB3	protein_coding	ENSG00000176531	0,563739212	3,98E-11	3E-10
TARBP1	protein_coding	ENSG00000059588	-0,426386054	3,99E-11	3E-10
KNL1	protein_coding	ENSG00000137812	-0,45757112	4E-11	3,01E-10
EXO1	protein_coding	ENSG00000174371	-0,427770268	4,04E-11	3,04E-10
AL139260.1	antisense	ENSG00000228436	1,610324192	4,05E-11	3,05E-10
AC125807.2	lincRNA	ENSG00000250899	-0,731834913	4,06E-11	3,05E-10
RIN2	protein_coding	ENSG00000132669	-0,323154554	4,16E-11	3,12E-10
A4GALT	protein_coding	ENSG00000128274	-0,842515731	4,16E-11	3,12E-10
CPED1	protein_coding	ENSG00000106034	1,068042146	4,19E-11	3,15E-10
DPH2	protein_coding	ENSG00000132768	-0,427275649	4,21E-11	3,16E-10
SLC25A24	protein_coding	ENSG00000085491	-0,432145678	4,23E-11	3,18E-10
ASB5	protein_coding	ENSG00000164122	2,829149878	4,25E-11	3,19E-10
PARVA	protein_coding	ENSG00000197702	0,216793359	4,28E-11	3,21E-10
HNRNPH2	protein_coding	ENSG00000126945	-0,449457856	4,29E-11	3,21E-10
HNRNPA3	protein_coding	ENSG00000170144	-0,491344006	4,29E-11	3,21E-10
ZNF33B	protein_coding	ENSG00000196693	-0,490244735	4,3E-11	3,22E-10
MRPL16	protein_coding	ENSG00000166902	-0,508280538	4,32E-11	3,23E-10
GLMP	protein_coding	ENSG00000198715	-0,385040509	4,39E-11	3,28E-10
SPAST	protein_coding	ENSG00000021574	-0,471720046	4,39E-11	3,29E-10
AC136604.2	antisense	ENSG00000244945	2,546883249	4,48E-11	3,35E-10
GNAS	protein_coding	ENSG00000087460	0,210631041	4,5E-11	3,36E-10
SIN3A	protein_coding	ENSG00000169375	-0,359549151	4,49E-11	3,36E-10
SLC37A2	protein_coding	ENSG00000134955	0,934193328	4,51E-11	3,37E-10
RNPS1	protein_coding	ENSG00000205937	-0,280862551	4,62E-11	3,45E-10
CLNS1A	protein_coding	ENSG00000074201	-0,40128746	4,63E-11	3,46E-10
QPCT	protein_coding	ENSG00000115828	0,886458192	4,68E-11	3,49E-10

ZFAND5	protein_coding	ENSG00000107372	0,309501465	4,68E-11	3,49E-10
LINC01694	lincRNA	ENSG00000233922	-0,562006524	4,73E-11	3,53E-10
ATP6V0A4	protein_coding	ENSG00000105929	0,66755333	4,75E-11	3,54E-10
MRPL27	protein_coding	ENSG00000108826	-0,525849721	4,76E-11	3,54E-10
MMS22L	protein_coding	ENSG00000146263	-0,511202204	4,88E-11	3,63E-10
OSBPL2	protein_coding	ENSG00000130703	0,383993217	4,93E-11	3,67E-10
IFRD2	protein_coding	ENSG00000214706	-0,356859285	4,93E-11	3,67E-10
ATF6B	protein_coding	ENSG00000213676	-0,35350238	4,95E-11	3,68E-10
SBK3	protein_coding	ENSG00000231274	2,570813152	4,98E-11	3,71E-10
ZNF211	protein_coding	ENSG00000121417	0,461241364	5,02E-11	3,73E-10
CWF19L1	protein_coding	ENSG00000095485	-0,411358916	5,23E-11	3,89E-10
PTGES3	protein_coding	ENSG00000110958	-0,403250995	5,26E-11	3,9E-10
DPY19L3	protein_coding	ENSG00000178904	0,428459061	5,27E-11	3,92E-10
HMCN1	protein_coding	ENSG00000143341	1,086390545	5,3E-11	3,94E-10
HFE	protein_coding	ENSG00000010704	-0,362047135	5,38E-11	3,99E-10
SHROOM1	protein_coding	ENSG00000164403	-0,618261203	5,46E-11	4,05E-10
NEK10	protein_coding	ENSG00000163491	0,535375004	5,57E-11	4,13E-10
LINC01435	lincRNA	ENSG00000229981	1,620377204	5,79E-11	4,29E-10
JHY	protein_coding	ENSG00000109944	0,691936278	5,92E-11	4,39E-10
DSG3	protein_coding	ENSG00000134757	3,605731236	6,18E-11	4,58E-10
PFN2	protein_coding	ENSG00000070087	-0,384211237	6,19E-11	4,58E-10
AL139385.1	antisense	ENSG00000275880	2,517526973	6,26E-11	4,63E-10
YY1	protein_coding	ENSG00000100811	-0,315710306	6,26E-11	4,64E-10
TEDC1	protein_coding	ENSG00000185347	-0,613061172	6,34E-11	4,69E-10
BAIAP2L1	protein_coding	ENSG00000006453	0,373354385	6,38E-11	4,71E-10
ERI3	protein_coding	ENSG00000117419	-0,346844502	6,37E-11	4,71E-10
CASP2	protein_coding	ENSG00000106144	-0,504185116	6,41E-11	4,74E-10
MAP4K3-DT	antisense	ENSG00000231312	0,793451464	6,49E-11	4,79E-10
CNNM1	protein_coding	ENSG00000119946	-0,569508736	6,49E-11	4,79E-10
BTBD7	protein_coding	ENSG00000011114	-0,425747601	6,51E-11	4,81E-10
LINC01006	lincRNA	ENSG00000182648	-0,576299402	6,6E-11	4,87E-10
IFT122	protein_coding	ENSG00000163913	-0,377491718	6,73E-11	4,97E-10
HNRNPK	protein_coding	ENSG00000165119	-0,246032463	6,98E-11	5,15E-10
HELZ	protein_coding	ENSG00000198265	0,438413974	7,01E-11	5,17E-10
MAP3K8	protein_coding	ENSG00000107968	1,366279672	7,08E-11	5,21E-10
ARL6IP6	protein_coding	ENSG00000177917	-0,491912531	7,1E-11	5,23E-10
PRPS2	protein_coding	ENSG00000101911	-0,457757803	7,14E-11	5,25E-10
PODNL1	protein_coding	ENSG00000132000	2,860153772	7,18E-11	5,29E-10
LINC02541	lincRNA	ENSG00000230943	4,313480696	7,25E-11	5,33E-10
IAH1	protein_coding	ENSG00000134330	-0,460675787	7,26E-11	5,34E-10

ALYREF	protein_coding	ENSG00000183684	-0,318589518	7,33E-11	5,39E-10
ANKRD28	protein_coding	ENSG00000206560	-0,331485889	7,34E-11	5,39E-10
CTPS2	protein_coding	ENSG00000047230	-0,414821667	7,42E-11	5,45E-10
AL450322.1	antisense	ENSG00000228353	7,51843711	7,45E-11	5,47E-10
AC018816.1	antisense	ENSG00000235978	1,929617126	7,44E-11	5,47E-10
TFEC	protein_coding	ENSG00000105967	1,256442301	7,56E-11	5,54E-10
ERCC1	protein_coding	ENSG00000012061	0,392558114	7,55E-11	5,54E-10
STPG1	protein_coding	ENSG00000001460	0,538135189	7,62E-11	5,59E-10
AL121603.2	antisense	ENSG00000258738	0,958978985	7,67E-11	5,62E-10
SLC22A5	protein_coding	ENSG00000197375	0,564578246	7,79E-11	5,71E-10
AC243772.2	antisense	ENSG00000233030	5,457287417	7,87E-11	5,77E-10
MYLIP	protein_coding	ENSG00000007944	0,761283469	7,93E-11	5,8E-10
WRAP73	protein_coding	ENSG00000116213	-0,467810197	8,06E-11	5,9E-10
NGF	protein_coding	ENSG00000134259	0,655804288	8,1E-11	5,92E-10
PGPEP1	protein_coding	ENSG00000130517	0,535097187	8,1E-11	5,92E-10
AC004585.1	lincRNA	ENSG00000266088	-0,551840026	8,14E-11	5,95E-10
MALT1	protein_coding	ENSG00000172175	0,30102249	8,15E-11	5,95E-10
FANCI	protein_coding	ENSG00000140525	-0,348395207	8,14E-11	5,95E-10
NEDD4	protein_coding	ENSG00000069869	0,305819632	8,22E-11	6E-10
FAM133B	protein_coding	ENSG00000234545	0,429924511	8,23E-11	6,01E-10
FAM83G	protein_coding	ENSG00000188522	0,499752433	8,26E-11	6,03E-10
JUP	protein_coding	ENSG00000173801	0,686679829	8,27E-11	6,04E-10
UBR5-AS1	antisense	ENSG00000246263	1,094286396	8,31E-11	6,06E-10
CNST	protein_coding	ENSG00000162852	0,315304889	8,37E-11	6,1E-10
SFXN3	protein_coding	ENSG00000107819	-0,287884269	8,56E-11	6,24E-10
ORC2	protein_coding	ENSG00000115942	-0,351962377	8,56E-11	6,24E-10
SLC44A3-AS1	transcribed_processed_	ENSG00000224081	1,117451342	8,6E-11	6,26E-10
NBPF3	protein_coding	ENSG00000142794	-0,428497358	8,64E-11	6,29E-10
HOXB6	protein_coding	ENSG00000108511	-0,720125036	8,72E-11	6,35E-10
GOPC	protein_coding	ENSG00000047932	0,387656688	8,77E-11	6,38E-10
UBFD1	protein_coding	ENSG00000103353	0,31993141	8,93E-11	6,5E-10
RAD51D	protein_coding	ENSG00000185379	-0,418895743	8,97E-11	6,52E-10
CTBP1	protein_coding	ENSG00000159692	-0,307339023	9,02E-11	6,56E-10
LINC01303	lincRNA	ENSG00000250548	1,160944128	9,05E-11	6,57E-10
BTBD6	protein_coding	ENSG00000184887	-0,323205129	9,07E-11	6,58E-10
RANGAP1	protein_coding	ENSG00000100401	-0,388756979	9,15E-11	6,65E-10
RAB8A	protein_coding	ENSG00000167461	-0,268082998	9,32E-11	6,76E-10
SNHG16	processed_transcript	ENSG00000163597	-0,221973603	9,46E-11	6,86E-10
AC087190.2	lincRNA	ENSG00000261392	1,951289139	9,5E-11	6,89E-10
PACSIN3	protein_coding	ENSG00000165912	-0,478205692	9,51E-11	6,89E-10

EEF1AKNMT	protein_coding	ENSG00000010165	-0,36114651	9,63E-11	6,98E-10
PARP10	protein_coding	ENSG00000178685	0,797683114	9,75E-11	7,06E-10
TPST2	protein_coding	ENSG00000128294	-0,450357601	9,9E-11	7,17E-10
H2AFX	protein_coding	ENSG00000188486	-0,495787379	9,93E-11	7,19E-10
MRI1	protein_coding	ENSG00000037757	-0,366126741	1E-10	7,23E-10
RAB11A	protein_coding	ENSG00000103769	-0,385070424	1,01E-10	7,32E-10
EBI3	protein_coding	ENSG00000105246	0,618451031	1,02E-10	7,36E-10
ASB7	protein_coding	ENSG00000183475	0,384461238	1,02E-10	7,37E-10
C2CD5	protein_coding	ENSG00000111731	-0,336083454	1,02E-10	7,39E-10
GSG1	protein_coding	ENSG00000111305	1,894521439	1,03E-10	7,41E-10
LINC02475	lincRNA	ENSG00000251350	-1,054358663	1,03E-10	7,45E-10
TMEM69	protein_coding	ENSG00000159596	-0,366622704	1,03E-10	7,45E-10
MIS18BP1	protein_coding	ENSG00000129534	-0,518728286	1,04E-10	7,48E-10
UNC13D	protein_coding	ENSG00000092929	1,432401119	1,04E-10	7,53E-10
KPNB1	protein_coding	ENSG00000108424	-0,38184494	1,05E-10	7,57E-10
COPS7A	protein_coding	ENSG00000111652	-0,328129041	1,05E-10	7,58E-10
TIMM13	protein_coding	ENSG00000099800	-0,401629412	1,1E-10	7,9E-10
WDR34	protein_coding	ENSG00000119333	-0,393296424	1,1E-10	7,93E-10
EWSR1	protein_coding	ENSG00000182944	-0,275119622	1,11E-10	8E-10
RAB11B-AS1	antisense	ENSG00000269386	1,159029317	1,12E-10	8,06E-10
RALB	protein_coding	ENSG00000144118	0,268331925	1,13E-10	8,13E-10
TMEM156	protein_coding	ENSG00000121895	0,958610397	1,14E-10	8,23E-10
PIM1	protein_coding	ENSG00000137193	0,619219899	1,15E-10	8,29E-10
AL049610.1	processed_pseudogene	ENSG00000180284	3,269661967	1,16E-10	8,31E-10
SECISBP2L	protein_coding	ENSG00000138593	0,311634421	1,17E-10	8,38E-10
SNHG12	antisense	ENSG00000197989	0,585196514	1,17E-10	8,4E-10
MFSD2A	protein_coding	ENSG00000168389	0,877649766	1,18E-10	8,47E-10
CLDN16	protein_coding	ENSG00000113946	3,752016903	1,18E-10	8,49E-10
CRLF2	protein_coding	ENSG00000205755	1,56295201	1,22E-10	8,75E-10
CSRNP3	protein_coding	ENSG00000178662	2,013535682	1,24E-10	8,9E-10
RSPRY1	protein_coding	ENSG00000159579	0,27899237	1,25E-10	8,93E-10
SLC25A25	protein_coding	ENSG00000148339	0,482513051	1,25E-10	8,98E-10
AGO4	protein_coding	ENSG00000134698	-0,477964864	1,28E-10	9,2E-10
MERTK	protein_coding	ENSG00000153208	0,487851566	1,33E-10	9,55E-10
C1orf159	protein_coding	ENSG00000131591	-0,638595276	1,37E-10	9,83E-10
ZER1	protein_coding	ENSG00000160445	0,45451215	1,38E-10	9,91E-10
CENPH	protein_coding	ENSG00000153044	-0,448598437	1,39E-10	9,93E-10
LRRC15	protein_coding	ENSG00000172061	4,066832648	1,39E-10	9,95E-10
MCAT	protein_coding	ENSG00000100294	-0,425860842	1,4E-10	0,000000001
SCMH1	protein_coding	ENSG00000010803	-0,463308569	1,4E-10	0,000000001

AC026401.3	lincRNA	ENSG00000280206	-0,666741439	1,42E-10	1,02E-09
SCG5	protein_coding	ENSG00000166922	0,867028878	1,43E-10	1,02E-09
GAB2	protein_coding	ENSG00000033327	-0,446241073	1,42E-10	1,02E-09
AL139039.3	antisense	ENSG00000237685	1,731300165	1,46E-10	1,04E-09
CRYZ	protein_coding	ENSG00000116791	-0,409153491	1,45E-10	1,04E-09
ARHGDIA	protein_coding	ENSG00000141522	-0,451936743	1,47E-10	1,05E-09
CNTRL	protein_coding	ENSG00000119397	-0,458990935	1,48E-10	1,05E-09
ZNRD1ASP	transcribed_unitary_pse	ENSG00000204623	0,740257945	1,47E-10	1,05E-09
AF241725.1	lincRNA	ENSG00000237527	0,599709738	1,5E-10	1,07E-09
SLAIN1	protein_coding	ENSG00000139737	-0,420611683	1,51E-10	1,07E-09
EXOSC9	protein_coding	ENSG00000123737	-0,480554317	1,53E-10	1,09E-09
LARP6	protein_coding	ENSG00000166173	0,376793674	1,55E-10	1,1E-09
ENTPD4	protein_coding	ENSG00000197217	-0,423965013	1,55E-10	1,1E-09
AC067852.2	antisense	ENSG00000266962	-0,538363661	1,57E-10	1,11E-09
ZFP14	protein_coding	ENSG00000142065	-0,824103406	1,56E-10	1,11E-09
SIX1	protein_coding	ENSG00000126778	-0,826410943	1,57E-10	1,12E-09
AC010327.3	antisense	ENSG00000267577	-0,684014476	1,6E-10	1,13E-09
AP004247.2	lincRNA	ENSG00000255146	2,653660139	1,6E-10	1,13E-09
LINC01215	lincRNA	ENSG00000271856	7,191097249	1,6E-10	1,14E-09
RANBP3L	protein_coding	ENSG00000164188	7,245060464	1,6E-10	1,14E-09
ECI1	protein_coding	ENSG00000167969	-0,392363981	1,61E-10	1,14E-09
SLC9B2	protein_coding	ENSG00000164038	-0,442462626	1,61E-10	1,14E-09
MRPS16	protein_coding	ENSG00000182180	-0,295611485	1,62E-10	1,15E-09
CCNQ	protein_coding	ENSG00000262919	-0,45387442	1,62E-10	1,15E-09
LINC00662	lincRNA	ENSG00000261824	0,485481839	1,64E-10	1,16E-09
RSBN1L	protein_coding	ENSG00000187257	-0,451958981	1,63E-10	1,16E-09
LINC01275	lincRNA	ENSG00000237595	1,781196456	1,66E-10	1,18E-09
CDC45	protein_coding	ENSG00000093009	-0,374071318	1,66E-10	1,18E-09
ID3	protein_coding	ENSG00000117318	-0,417216933	1,66E-10	1,18E-09
AC009005.1	antisense	ENSG00000267751	-0,674568082	1,72E-10	1,21E-09
PSG1	protein_coding	ENSG00000231924	2,443800152	1,71E-10	1,21E-09
RXRB	protein_coding	ENSG00000204231	-0,44860148	1,71E-10	1,21E-09
AC092053.3	antisense	ENSG00000284669	2,881892954	1,72E-10	1,22E-09
TMED2	protein_coding	ENSG00000086598	0,331547662	1,72E-10	1,22E-09
DCLRE1A	protein_coding	ENSG00000198924	-0,417068351	1,74E-10	1,23E-09
PTPRJ	protein_coding	ENSG00000149177	-0,415530431	1,76E-10	1,24E-09
KIAA1143	protein_coding	ENSG00000163807	-0,402323972	1,77E-10	1,25E-09
SERPINI1	protein_coding	ENSG00000163536	1,537350166	1,8E-10	1,27E-09
CPNE2	protein_coding	ENSG00000140848	-0,424381327	1,8E-10	1,27E-09
GRAMD2B	protein_coding	ENSG00000155324	0,303082241	1,83E-10	1,29E-09

NPM3	protein_coding	ENSG00000107833	-0,535471816	1,83E-10	1,29E-09
SFMBT1	protein_coding	ENSG00000163935	-0,357240505	1,85E-10	1,3E-09
PRAG1	protein_coding	ENSG00000275342	0,658763765	1,86E-10	1,31E-09
POP7	protein_coding	ENSG00000172336	-0,418724101	1,86E-10	1,31E-09
LITAF	protein_coding	ENSG00000189067	0,360740551	1,91E-10	1,34E-09
SMIM12	protein_coding	ENSG00000163866	-0,39843325	1,92E-10	1,35E-09
ADAM17	protein_coding	ENSG00000151694	0,245209208	1,95E-10	1,37E-09
PEX11A	protein_coding	ENSG00000166821	-0,644140145	1,96E-10	1,38E-09
MEIS1	protein_coding	ENSG00000143995	0,747532115	1,98E-10	1,39E-09
CNN2	protein_coding	ENSG00000064666	0,309835428	1,98E-10	1,39E-09
TMEM200B	protein_coding	ENSG00000253304	-0,544802627	1,98E-10	1,39E-09
MEIG1	protein_coding	ENSG00000197889	2,032446089	1,99E-10	1,4E-09
CSRP2	protein_coding	ENSG00000175183	0,529217198	1,99E-10	1,4E-09
PA2G4	protein_coding	ENSG00000170515	-0,271612998	1,99E-10	1,4E-09
AC098613.1	antisense	ENSG00000223552	3,034597328	2,01E-10	1,41E-09
DYRK4	protein_coding	ENSG00000010219	0,48365846	2,03E-10	1,42E-09
CEP250	protein_coding	ENSG00000126001	-0,647023953	2,03E-10	1,42E-09
RIMS3	protein_coding	ENSG00000117016	-0,611922683	2,04E-10	1,43E-09
CEP85L	protein_coding	ENSG00000111860	0,518918738	2,08E-10	1,46E-09
HACD3	protein_coding	ENSG00000074696	-0,39193627	2,08E-10	1,46E-09
LINC00973	lincRNA	ENSG00000240476	0,502360002	2,1E-10	1,47E-09
SLIT3	protein_coding	ENSG00000184347	0,684192822	2,1E-10	1,47E-09
SKA3	protein_coding	ENSG00000165480	-0,487169831	2,09E-10	1,47E-09
EYA3	protein_coding	ENSG00000158161	-0,308912353	2,12E-10	1,49E-09
S1PR3	protein_coding	ENSG00000213694	0,881558104	2,17E-10	1,52E-09
S1PR3	protein_coding	ENSG00000213694	0,881558104	2,17E-10	1,52E-09
ITGA1	protein_coding	ENSG00000213949	0,344939537	2,18E-10	1,52E-09
ANKRD2	protein_coding	ENSG00000165887	0,912686688	2,2E-10	1,53E-09
NF2	protein_coding	ENSG00000186575	-0,375636799	2,2E-10	1,53E-09
PRSS12	protein_coding	ENSG00000164099	-0,563641227	2,19E-10	1,53E-09
PPP4R1	protein_coding	ENSG00000154845	-0,300599145	2,24E-10	1,56E-09
BRIX1	protein_coding	ENSG00000113460	-0,451480345	2,26E-10	1,57E-09
NFKB2	protein_coding	ENSG00000077150	0,43935205	2,3E-10	1,6E-09
ATP6V1H	protein_coding	ENSG00000047249	0,339122814	2,3E-10	1,6E-09
CYP7A1	protein_coding	ENSG00000167910	7,316894004	2,31E-10	1,61E-09
ZNF74	protein_coding	ENSG00000185252	-0,540301854	2,31E-10	1,61E-09
HIST1H1T	protein_coding	ENSG00000187475	1,593088279	2,33E-10	1,62E-09
PAICS	protein_coding	ENSG00000128050	-0,382950117	2,32E-10	1,62E-09
PTCD3	protein_coding	ENSG00000132300	-0,28552333	2,34E-10	1,63E-09
QTRT2	protein_coding	ENSG00000151576	-0,297514569	2,36E-10	1,64E-09

RTL6	protein_coding	ENSG00000188636	-0,405842452	2,36E-10	1,64E-09
FEM1C	protein_coding	ENSG00000145780	0,428837346	2,38E-10	1,65E-09
NIT2	protein_coding	ENSG00000114021	-0,339285357	2,37E-10	1,65E-09
ANKRD16	protein_coding	ENSG00000134461	-0,596772609	2,37E-10	1,65E-09
ATG13	protein_coding	ENSG00000175224	0,337671146	2,44E-10	1,69E-09
FCGBP	protein_coding	ENSG00000275395	1,331312936	2,47E-10	1,71E-09
CBL	protein_coding	ENSG00000110395	0,44768999	2,48E-10	1,72E-09
JOSD1	protein_coding	ENSG00000100221	0,29139767	2,48E-10	1,72E-09
PFDN6	protein_coding	ENSG00000204220	-0,387190197	2,52E-10	1,75E-09
TAB3	protein_coding	ENSG00000157625	0,386481495	2,58E-10	1,79E-09
RAET1L	protein_coding	ENSG00000155918	7,193809698	2,61E-10	1,81E-09
POLR1C	protein_coding	ENSG00000171453	-0,356048267	2,61E-10	1,81E-09
NSUN6	protein_coding	ENSG00000241058	0,422980381	2,64E-10	1,83E-09
PRNP	protein_coding	ENSG00000171867	-0,290889259	2,66E-10	1,84E-09
SRPK2	protein_coding	ENSG00000135250	0,351993894	2,68E-10	1,85E-09
CYFIP2	protein_coding	ENSG00000055163	0,320641003	2,67E-10	1,85E-09
APC2	protein_coding	ENSG00000115266	-0,757945926	2,68E-10	1,85E-09
RNU5B-1	snRNA	ENSG00000200156	-0,977597571	2,68E-10	1,85E-09
JAK1	protein_coding	ENSG00000162434	0,27721864	2,7E-10	1,86E-09
SH2B1	protein_coding	ENSG00000178188	-0,490615985	2,7E-10	1,87E-09
SCAI	protein_coding	ENSG00000173611	-0,473176161	2,77E-10	1,91E-09
CCDC157	protein_coding	ENSG00000187860	1,471558009	2,85E-10	1,96E-09
BAX	protein_coding	ENSG00000087088	-0,363937808	2,83E-10	1,96E-09
PPP2R2C	protein_coding	ENSG00000074211	-0,471718331	2,84E-10	1,96E-09
HSD3BP5	transcribed_unprocesse	ENSG00000198857	5,736889182	2,85E-10	1,96E-09
RCC2	protein_coding	ENSG00000179051	-0,358194708	2,9E-10	0,000000002
DDB2	protein_coding	ENSG00000134574	-0,255698397	2,93E-10	2,02E-09
GLIS3	protein_coding	ENSG00000107249	0,505009474	2,96E-10	2,04E-09
NORAD	lincRNA	ENSG00000260032	-0,589176984	3,02E-10	2,08E-09
MUSK	protein_coding	ENSG00000030304	2,135174315	3,02E-10	2,08E-09
RAET1E	protein_coding	ENSG00000164520	0,724651571	3,06E-10	2,11E-09
DCAF1	protein_coding	ENSG00000145041	-0,384650742	3,07E-10	2,11E-09
PIP4K2C	protein_coding	ENSG00000166908	0,329699047	3,09E-10	2,12E-09
FAM3C	protein_coding	ENSG00000196937	0,421579709	3,1E-10	2,13E-09
PKD1L2	polymorphic_pseudoge	ENSG00000166473	0,620759994	3,25E-10	2,24E-09
YIPF6	protein_coding	ENSG00000181704	0,286925242	3,25E-10	2,24E-09
MRPL24	protein_coding	ENSG00000143314	-0,420663939	3,29E-10	2,26E-09
TMEM171	protein_coding	ENSG00000157111	-0,430362253	3,29E-10	2,26E-09
SPSB2	protein_coding	ENSG00000111671	-0,745101006	3,32E-10	2,28E-09
USP47	protein_coding	ENSG00000170242	0,334802098	3,34E-10	2,3E-09

PPARGC1B	protein_coding	ENSG00000155846	-0,668904073	3,38E-10	2,32E-09
ALOX5AP	protein_coding	ENSG00000132965	2,206858686	3,43E-10	2,35E-09
GTPBP4	protein_coding	ENSG00000107937	-0,309671378	3,45E-10	2,36E-09
SETDB1	protein_coding	ENSG00000143379	-0,334836109	3,44E-10	2,36E-09
AC026310.2	antisense	ENSG00000255921	3,317201189	3,48E-10	2,39E-09
ESAM	protein_coding	ENSG00000149564	2,295352529	3,49E-10	2,39E-09
VPS52	protein_coding	ENSG00000223501	-0,25326024	3,51E-10	2,4E-09
MAP7D3	protein_coding	ENSG00000129680	-0,375637004	3,5E-10	2,4E-09
DENND5A	protein_coding	ENSG00000184014	0,255485916	3,54E-10	2,42E-09
IPO5	protein_coding	ENSG00000065150	-0,312746702	3,53E-10	2,42E-09
LINC01819	lincRNA	ENSG00000231826	2,387461478	3,64E-10	2,49E-09
PLCD4	protein_coding	ENSG00000115556	1,406435589	3,66E-10	2,5E-09
EHBP1	protein_coding	ENSG00000115504	0,325676364	3,71E-10	2,53E-09
FOXM1	protein_coding	ENSG00000111206	-0,356551119	3,71E-10	2,54E-09
MEX3A	protein_coding	ENSG00000254726	-0,703351502	3,74E-10	2,56E-09
ME1	protein_coding	ENSG00000065833	0,558286741	3,8E-10	2,59E-09
NUDCD2	protein_coding	ENSG00000170584	-0,346611879	3,8E-10	2,59E-09
RELCH	protein_coding	ENSG00000134444	0,374034695	3,81E-10	2,6E-09
AC117394.2	antisense	ENSG00000244268	2,697623243	3,82E-10	2,61E-09
CENPB	protein_coding	ENSG00000125817	-0,47587611	3,83E-10	2,61E-09
EMG1	protein_coding	ENSG00000126749	-0,421337113	3,85E-10	2,62E-09
PUSL1	protein_coding	ENSG00000169972	-0,444231987	3,85E-10	2,62E-09
DDX51	protein_coding	ENSG00000185163	-0,463227469	3,88E-10	2,64E-09
KBTBD7	protein_coding	ENSG00000120696	-0,509805712	3,9E-10	2,66E-09
ADSS	protein_coding	ENSG00000035687	-0,326705427	3,92E-10	2,67E-09
PPOX	protein_coding	ENSG00000143224	-0,589813456	3,93E-10	2,67E-09
RAD1	protein_coding	ENSG00000113456	-0,472771394	4,03E-10	2,74E-09
LINC02577	lincRNA	ENSG00000228742	0,810616267	4,06E-10	2,76E-09
AC027307.2	antisense	ENSG00000267317	0,629750386	4,13E-10	2,81E-09
PICART1	lincRNA	ENSG00000246640	1,40538738	4,17E-10	2,83E-09
IGFBP7	protein_coding	ENSG00000163453	0,434114093	4,17E-10	2,83E-09
AFAP1L2	protein_coding	ENSG00000169129	-0,762681674	4,16E-10	2,83E-09
ASB2	protein_coding	ENSG00000100628	3,437975395	4,28E-10	2,9E-09
ENTPD1-AS1	antisense	ENSG00000226688	-0,544049634	4,32E-10	2,93E-09
FOXN2	protein_coding	ENSG00000170802	0,446084166	4,35E-10	2,95E-09
LSM6	protein_coding	ENSG00000164167	-0,556090941	4,38E-10	2,97E-09
DLL4	protein_coding	ENSG00000128917	4,195936686	4,42E-10	2,99E-09
RHEBL1	protein_coding	ENSG00000167550	0,805844932	4,41E-10	2,99E-09
LIG1	protein_coding	ENSG00000105486	-0,301766018	4,43E-10	0,000000003
PRKAG1	protein_coding	ENSG00000181929	-0,364007921	4,45E-10	3,01E-09

TVP23B	protein_coding	ENSG00000171928	0,339070453	4,48E-10	3,03E-09
ATAD2B	protein_coding	ENSG00000119778	0,472549308	4,55E-10	3,08E-09
AC068594.1	lincRNA	ENSG00000263718	3,40920713	4,59E-10	3,1E-09
PIDD1	protein_coding	ENSG00000177595	0,790099739	4,59E-10	3,1E-09
TFB1M	protein_coding	ENSG00000029639	-0,486776434	4,59E-10	3,1E-09
RNU1-4	snRNA	ENSG00000207389	-3,287638826	4,6E-10	3,11E-09
HIST4H4	protein_coding	ENSG00000197837	-0,614676133	4,67E-10	3,15E-09
AC135050.6	sense_intronic	ENSG00000278133	-0,770321424	4,66E-10	3,15E-09
UAP1L1	protein_coding	ENSG00000197355	0,529180263	4,69E-10	3,17E-09
ANKRD9	protein_coding	ENSG00000156381	-0,607487541	4,7E-10	3,17E-09
FAM129B	protein_coding	ENSG00000136830	-0,378010663	4,71E-10	3,18E-09
NRG1	protein_coding	ENSG00000157168	-0,339482839	4,76E-10	3,21E-09
SLC6A12	protein_coding	ENSG00000111181	5,663009096	4,8E-10	3,23E-09
MTR	protein_coding	ENSG00000116984	-0,379611474	4,79E-10	3,23E-09
TIA1	protein_coding	ENSG00000116001	-0,347048734	4,8E-10	3,24E-09
AC022092.1	antisense	ENSG00000249639	5,661398651	4,85E-10	3,26E-09
PUF60	protein_coding	ENSG00000179950	-0,336136979	4,99E-10	3,36E-09
IMPACT	protein_coding	ENSG00000154059	-0,378648323	5E-10	3,36E-09
INTS10	protein_coding	ENSG00000104613	-0,321565497	5,02E-10	3,38E-09
RSRP1	protein_coding	ENSG00000117616	-0,629878522	5,04E-10	3,39E-09
MEIOB	protein_coding	ENSG00000162039	-0,435022116	5,05E-10	3,4E-09
WFDC21P	transcribed_unitary_pse	ENSG00000261040	1,770625061	5,08E-10	3,42E-09
MSH2	protein_coding	ENSG00000095002	-0,432171695	5,14E-10	3,45E-09
TMEM50B	protein_coding	ENSG00000142188	0,50481362	5,15E-10	3,46E-09
GGPS1	protein_coding	ENSG00000152904	0,37098438	5,17E-10	3,47E-09
MPP7	protein_coding	ENSG00000150054	0,675379363	5,22E-10	3,5E-09
GPR37L1	protein_coding	ENSG00000170075	7,216855932	5,33E-10	3,58E-09
PDCD6IP	protein_coding	ENSG00000170248	-0,294037042	5,37E-10	3,6E-09
SMARCA5-AS1	antisense	ENSG00000245112	3,573360529	5,4E-10	3,62E-09
F2R	protein_coding	ENSG00000181104	-0,360305064	5,4E-10	3,62E-09
CXorf58	protein_coding	ENSG00000165182	3,297429171	5,41E-10	3,63E-09
TMEM232	protein_coding	ENSG00000186952	1,735863119	5,46E-10	3,66E-09
APEX1	protein_coding	ENSG00000100823	-0,414304736	5,49E-10	3,68E-09
OMA1	protein_coding	ENSG00000162600	-0,428023127	5,49E-10	3,68E-09
SLF2	protein_coding	ENSG00000119906	-0,339305064	5,57E-10	3,73E-09
WRAP53	protein_coding	ENSG00000141499	-0,329512129	5,6E-10	3,75E-09
EIF4E	protein_coding	ENSG00000151247	-0,466754958	5,61E-10	3,75E-09
ZNF850	protein_coding	ENSG00000267041	-0,406316046	5,64E-10	3,77E-09
Z99943.1	antisense	ENSG00000232194	2,314748426	5,66E-10	3,78E-09
TNS3	protein_coding	ENSG00000136205	-0,460797818	5,7E-10	3,81E-09

RNF180	protein_coding	ENSG00000164197	4,276707753	5,71E-10	3,82E-09
NEK1	protein_coding	ENSG00000137601	0,394198236	5,72E-10	3,82E-09
DIP2A	protein_coding	ENSG00000160305	-0,432659018	5,76E-10	3,85E-09
STX16	protein_coding	ENSG00000124222	0,516508431	5,79E-10	3,86E-09
SH2B3	protein_coding	ENSG00000111252	0,420499154	5,78E-10	3,86E-09
RAD9B	protein_coding	ENSG00000151164	1,238721491	5,8E-10	3,87E-09
ADCY3	protein_coding	ENSG00000138031	-0,479049119	5,8E-10	3,87E-09
NUP37	protein_coding	ENSG00000075188	-0,491548811	5,85E-10	3,9E-09
ZNF692	protein_coding	ENSG00000171163	-0,642058242	5,9E-10	3,93E-09
ACVR2B	protein_coding	ENSG00000114739	-0,637062746	5,93E-10	3,95E-09
ZNF175	protein_coding	ENSG00000105497	0,43520118	5,98E-10	3,98E-09
MGAT1	protein_coding	ENSG00000131446	-0,368271539	5,98E-10	3,98E-09
GREM1	protein_coding	ENSG00000166923	4,516397064	6,03E-10	4,01E-09
NALCN	protein_coding	ENSG00000102452	0,605243329	6,09E-10	4,05E-09
INO80E	protein_coding	ENSG00000169592	-0,334208396	6,09E-10	4,05E-09
KATNAL2	protein_coding	ENSG00000167216	1,053188769	6,2E-10	4,12E-09
SMARCC1	protein_coding	ENSG00000173473	-0,301587502	6,29E-10	4,18E-09
TOB2P1	processed_pseudogene	ENSG00000176933	1,038182148	6,34E-10	4,21E-09
NPFFR1	protein_coding	ENSG00000148734	5,559280101	6,35E-10	4,22E-09
NANS	protein_coding	ENSG00000095380	0,369503048	6,42E-10	4,27E-09
RORA	protein_coding	ENSG00000069667	1,113556548	6,45E-10	4,28E-09
AC034223.2	lincRNA	ENSG00000251281	0,686380148	6,51E-10	4,32E-09
AGBL3	protein_coding	ENSG00000146856	0,958010422	6,58E-10	4,36E-09
MCM3AP	protein_coding	ENSG00000160294	-0,395279422	6,58E-10	4,36E-09
KCNS3	protein_coding	ENSG00000170745	-0,715015805	6,58E-10	4,36E-09
VAC14	protein_coding	ENSG00000103043	0,342460876	6,68E-10	4,43E-09
SARAF	protein_coding	ENSG00000133872	0,308516007	6,71E-10	4,45E-09
AC093157.1	antisense	ENSG00000233184	0,818826693	6,77E-10	4,49E-09
CD163L1	protein_coding	ENSG00000177675	0,453809768	6,84E-10	4,53E-09
NRF1	protein_coding	ENSG00000106459	-0,432259006	6,86E-10	4,54E-09
BMF	protein_coding	ENSG00000104081	2,239976812	7,01E-10	4,64E-09
CEBPB	protein_coding	ENSG00000172216	0,363921461	7,13E-10	4,71E-09
JPT2	protein_coding	ENSG00000206053	-0,345343591	7,2E-10	4,76E-09
ANXA1	protein_coding	ENSG00000135046	-0,397860015	7,2E-10	4,76E-09
DLEU2	antisense	ENSG00000231607	-0,727567071	7,22E-10	4,77E-09
SVIL	protein_coding	ENSG00000197321	0,386695588	7,26E-10	4,8E-09
NLN	protein_coding	ENSG00000123213	-0,358769754	7,28E-10	4,81E-09
FZD1	protein_coding	ENSG00000157240	-0,644933098	7,4E-10	4,88E-09
GGCT	protein_coding	ENSG00000006625	-0,5929019	7,43E-10	4,91E-09
PPP1R12A-AS1	bidirectional_promoter_	ENSG00000257557	1,144545535	7,47E-10	4,93E-09

ZNF639	protein_coding	ENSG00000121864	-0,420074905	7,47E-10	4,93E-09
ADORA2B	protein_coding	ENSG00000170425	-0,340354071	7,53E-10	4,96E-09
LINC00862	lincRNA	ENSG00000203721	1,073813058	7,59E-10	0,000000005
LINC01537	lincRNA	ENSG00000227467	1,440037257	7,69E-10	5,06E-09
SCO1	protein_coding	ENSG00000133028	-0,339357742	7,72E-10	5,08E-09
ARL13B	protein_coding	ENSG00000169379	0,400348888	7,78E-10	5,12E-09
PKMYT1	protein_coding	ENSG00000127564	-0,503914898	7,77E-10	5,12E-09
NUBP1	protein_coding	ENSG00000103274	-0,424908879	7,81E-10	5,14E-09
TJAP1	protein_coding	ENSG00000137221	0,463976892	7,83E-10	5,15E-09
FAM171A1	protein_coding	ENSG00000148468	-0,340990001	7,84E-10	5,15E-09
RAN	protein_coding	ENSG00000132341	-0,396136426	8,13E-10	5,34E-09
RGS3	protein_coding	ENSG00000138835	0,487282544	8,17E-10	5,37E-09
ADNP2	protein_coding	ENSG00000101544	-0,345843623	8,22E-10	5,4E-09
HIST1H3E	protein_coding	ENSG00000274750	0,369225611	8,38E-10	5,5E-09
ZGRF1	protein_coding	ENSG00000138658	-0,554280366	8,4E-10	5,52E-09
VASH1	protein_coding	ENSG00000071246	-0,729420838	8,56E-10	5,62E-09
BHLHE41	protein_coding	ENSG00000123095	2,500641685	8,59E-10	5,63E-09
ARAF	protein_coding	ENSG00000078061	-0,373720506	8,58E-10	5,63E-09
LINC02547	lincRNA	ENSG00000254486	1,88437265	8,67E-10	5,69E-09
HTATSF1	protein_coding	ENSG00000102241	-0,398671567	8,78E-10	5,75E-09
UNK	protein_coding	ENSG00000132478	-0,310116629	8,79E-10	5,76E-09
GRK5	protein_coding	ENSG00000198873	0,659439239	8,98E-10	5,88E-09
KIF20B	protein_coding	ENSG00000138182	-0,539062917	9,07E-10	5,94E-09
PDE6C	protein_coding	ENSG00000095464	3,622798567	9,19E-10	6,02E-09
HEMK1	protein_coding	ENSG00000114735	-0,362458383	9,21E-10	6,03E-09
LINC02057	lincRNA	ENSG00000249279	0,599579849	9,28E-10	6,07E-09
CCDC93	protein_coding	ENSG00000125633	0,27852264	9,3E-10	6,08E-09
PEX11B	protein_coding	ENSG00000131779	-0,515013702	9,36E-10	6,12E-09
MRPL43	protein_coding	ENSG00000055950	-0,452497511	9,38E-10	6,13E-09
SEPT4	protein_coding	ENSG00000108387	3,360817518	9,53E-10	6,23E-09
ZNF436	protein_coding	ENSG00000125945	0,443713833	9,54E-10	6,23E-09
ANGEL1	protein_coding	ENSG00000013523	-0,509283354	9,59E-10	6,26E-09
SMC3	protein_coding	ENSG00000108055	-0,505311755	9,69E-10	6,33E-09
FAM124A	protein_coding	ENSG00000150510	-0,867956663	9,72E-10	6,34E-09
VWDE	protein_coding	ENSG00000146530	2,11064355	9,77E-10	6,38E-09
VDAC3	protein_coding	ENSG00000078668	-0,44310231	9,84E-10	6,42E-09
AL365181.2	lincRNA	ENSG00000272068	1,959719187	0,000000001	6,55E-09
RALGPS2	protein_coding	ENSG00000116191	-0,376989416	1,01E-09	6,58E-09
ISCA2	protein_coding	ENSG00000165898	-0,392087909	1,01E-09	6,58E-09
DYNLL2	protein_coding	ENSG00000264364	0,390721629	1,02E-09	6,61E-09

DIXDC1	protein_coding	ENSG00000150764	-0,597573494	1,02E-09	6,64E-09
PNP	protein_coding	ENSG00000198805	0,361711996	1,02E-09	6,65E-09
PPAT	protein_coding	ENSG00000128059	-0,369832501	1,03E-09	6,68E-09
SOX12	protein_coding	ENSG00000177732	-0,627301894	1,03E-09	6,71E-09
MIR17HG	processed_transcript	ENSG00000215417	-1,613228089	1,05E-09	6,8E-09
ART4	protein_coding	ENSG00000111339	4,071012145	1,05E-09	6,81E-09
ARPP19	protein_coding	ENSG00000128989	-0,381503114	1,05E-09	6,83E-09
GBX2	protein_coding	ENSG00000168505	0,492930215	1,05E-09	6,84E-09
AL162595.1	bidirectional_promoter_	ENSG00000177788	-1,080889743	1,08E-09	0,000000007
CDC25A	protein_coding	ENSG00000164045	-0,421265374	1,09E-09	7,05E-09
UTP25	protein_coding	ENSG00000117597	0,283745412	1,09E-09	7,06E-09
AC048341.1	lincRNA	ENSG00000257354	1,043279175	1,1E-09	7,12E-09
OLFM3	protein_coding	ENSG00000118733	7,020144125	1,1E-09	7,12E-09
TMIE	protein_coding	ENSG00000181585	1,628631349	1,11E-09	7,19E-09
TLK1	protein_coding	ENSG00000198586	0,334612715	1,11E-09	7,2E-09
XRCC3	protein_coding	ENSG00000126215	-0,447317068	1,11E-09	7,2E-09
AC073283.2	lincRNA	ENSG00000226087	4,444895514	1,14E-09	7,41E-09
AKR1D1	protein_coding	ENSG00000122787	2,763888303	1,15E-09	7,47E-09
ZNF417	protein_coding	ENSG00000173480	-0,476474258	1,15E-09	7,47E-09
HMMR	protein_coding	ENSG00000072571	-0,515052247	1,18E-09	7,65E-09
AC061975.1	lincRNA	ENSG00000260777	1,190133216	1,19E-09	7,7E-09
CBX1	protein_coding	ENSG00000108468	-0,307611642	1,2E-09	7,74E-09
PASK	protein_coding	ENSG00000115687	-0,523066855	1,2E-09	7,74E-09
TMCO3	protein_coding	ENSG00000150403	-0,244429136	1,2E-09	7,75E-09
AC022217.3	antisense	ENSG00000253736	2,586321012	1,2E-09	7,76E-09
SCNN1A	protein_coding	ENSG00000111319	1,604479836	1,21E-09	7,79E-09
TLE1	protein_coding	ENSG00000196781	-0,320198206	1,21E-09	7,81E-09
AC009226.1	antisense	ENSG00000223725	1,729710592	1,22E-09	7,87E-09
NFATC2	protein_coding	ENSG00000101096	4,966368824	1,23E-09	7,93E-09
SMG7	protein_coding	ENSG00000116698	0,381804455	1,24E-09	8,02E-09
IPO9	protein_coding	ENSG00000198700	-0,302512727	1,27E-09	8,18E-09
DDX54	protein_coding	ENSG00000123064	-0,375663345	1,29E-09	8,34E-09
AL353759.1	antisense	ENSG00000283064	5,486730783	1,3E-09	8,4E-09
ZADH2	protein_coding	ENSG00000180011	-0,371720013	1,31E-09	8,44E-09
C2CD4A	protein_coding	ENSG00000198535	4,039242847	1,31E-09	8,45E-09
SMARCA1	protein_coding	ENSG00000102038	-0,311796029	1,31E-09	8,45E-09
TIMM8A	protein_coding	ENSG00000126953	-0,433169322	1,32E-09	8,47E-09
SLC1A5	protein_coding	ENSG00000105281	0,446911473	1,33E-09	8,53E-09
AC007364.1	antisense	ENSG00000231969	4,461480862	1,34E-09	8,64E-09
IL12A	protein_coding	ENSG00000168811	0,824994989	1,34E-09	8,64E-09

ZNF559	protein_coding	ENSG00000188321	-0,463016287	1,38E-09	8,9E-09
NUDT15	protein_coding	ENSG00000136159	-0,36866703	1,39E-09	8,92E-09
MAPK10	protein_coding	ENSG00000109339	1,612358699	1,4E-09	8,99E-09
MED15	protein_coding	ENSG00000099917	0,411497712	1,4E-09	9,01E-09
DPH6	protein_coding	ENSG00000134146	-0,550182162	1,4E-09	9,01E-09
EGLN1	protein_coding	ENSG00000135766	-0,243216999	1,41E-09	9,04E-09
AC132008.2	transcribed_unprocesse	ENSG00000214135	-0,46819196	1,41E-09	9,06E-09
HMGCL	protein_coding	ENSG00000117305	0,521972363	1,42E-09	9,11E-09
AAK1	protein_coding	ENSG00000115977	0,4210644	1,43E-09	9,16E-09
LINC00911	lincRNA	ENSG00000259107	2,191348232	1,43E-09	9,18E-09
PCYT1A	protein_coding	ENSG00000161217	0,208304085	1,44E-09	9,22E-09
THUMPD2	protein_coding	ENSG00000138050	0,442679722	1,44E-09	9,25E-09
RPL29P19	processed_pseudogene	ENSG00000224594	1,093674091	1,45E-09	9,31E-09
SPRY1	protein_coding	ENSG00000164056	0,87384177	1,45E-09	9,31E-09
UBTD2	protein_coding	ENSG00000168246	-0,414533383	1,46E-09	9,31E-09
PATZ1	protein_coding	ENSG00000100105	-0,527148131	1,48E-09	9,44E-09
B4GALNT2	protein_coding	ENSG00000167080	3,238933084	1,48E-09	9,48E-09
CNTN5	protein_coding	ENSG00000149972	0,956788202	1,49E-09	9,52E-09
DFFA	protein_coding	ENSG00000160049	-0,242184879	1,5E-09	9,57E-09
ZNF302	protein_coding	ENSG00000089335	-0,475244659	1,51E-09	9,62E-09
NR2F2-AS1	antisense	ENSG00000247809	0,779661633	1,51E-09	9,63E-09
ANKRD31	protein_coding	ENSG00000145700	1,197894619	1,53E-09	9,79E-09
STX11	protein_coding	ENSG00000135604	1,358200452	1,55E-09	9,9E-09
ANKRD36	protein_coding	ENSG00000135976	-0,532069612	1,56E-09	9,94E-09
MCM3AP-AS1	antisense	ENSG00000215424	-0,753403649	1,57E-09	0,00000001
ZFP90	protein_coding	ENSG00000184939	0,319930455	1,58E-09	1,01E-08
DNAJA1	protein_coding	ENSG00000086061	0,244555053	1,58E-09	1,01E-08
PRPF3	protein_coding	ENSG00000117360	-0,30114263	1,59E-09	1,01E-08
SCIN	protein_coding	ENSG00000006747	3,112704662	1,63E-09	1,04E-08
CCDC85B	protein_coding	ENSG00000175602	-0,46820504	1,65E-09	1,05E-08
MKLN1-AS	processed_transcript	ENSG00000236753	0,720793957	1,67E-09	1,06E-08
USP5	protein_coding	ENSG00000111667	-0,296603703	1,67E-09	1,06E-08
UBAC1	protein_coding	ENSG00000130560	-0,331807175	1,67E-09	1,06E-08
AFAP1L1	protein_coding	ENSG00000157510	-0,355456666	1,66E-09	1,06E-08
PKN3	protein_coding	ENSG00000160447	-0,403552924	1,66E-09	1,06E-08
RNF227	protein_coding	ENSG00000179859	-1,097960901	1,71E-09	1,09E-08
TAGLN	protein_coding	ENSG00000149591	0,77491527	1,73E-09	0,000000011
ILVBL	protein_coding	ENSG00000105135	-0,431838774	1,73E-09	0,000000011
STARD7-AS1	processed_transcript	ENSG00000204685	0,783023658	1,75E-09	1,11E-08
HELB	protein_coding	ENSG00000127311	0,492452428	1,77E-09	1,12E-08

CALCOCO2	protein_coding	ENSG00000136436	-0,279986511	1,77E-09	1,12E-08
RPIA	protein_coding	ENSG00000153574	-0,377694475	1,77E-09	1,12E-08
HMBS	protein_coding	ENSG00000256269	-0,502089415	1,76E-09	1,12E-08
DEDD2	protein_coding	ENSG00000160570	0,520209887	1,78E-09	1,13E-08
ITPRIP	protein_coding	ENSG00000148841	0,514795861	1,78E-09	1,13E-08
ITGB6	protein_coding	ENSG00000115221	1,752660617	1,8E-09	1,14E-08
ODF2	protein_coding	ENSG00000136811	-0,310185325	1,8E-09	1,14E-08
ZSWIM3	protein_coding	ENSG00000132801	0,700357218	1,82E-09	1,15E-08
PLPP5	protein_coding	ENSG00000147535	0,367413409	1,81E-09	1,15E-08
TRMT2A	protein_coding	ENSG00000099899	-0,368805641	1,82E-09	1,15E-08
ZNF618	protein_coding	ENSG00000157657	-0,557508396	1,81E-09	1,15E-08
PTPRB	protein_coding	ENSG00000127329	0,546329175	1,83E-09	1,16E-08
PFKFB2	protein_coding	ENSG00000123836	0,349717714	1,83E-09	1,16E-08
ZNF607	protein_coding	ENSG00000198182	-0,585861888	1,83E-09	1,16E-08
LINC01647	lincRNA	ENSG00000235643	4,949496691	1,86E-09	1,17E-08
AC006449.6	lincRNA	ENSG00000277969	0,67167073	1,85E-09	1,17E-08
VEPH1	protein_coding	ENSG00000197415	0,295912773	1,85E-09	1,17E-08
PHF23	protein_coding	ENSG00000040633	-0,320071389	1,87E-09	1,18E-08
SUCLG2-AS1	lincRNA	ENSG00000241316	0,68049428	1,89E-09	1,19E-08
PTGER3	protein_coding	ENSG00000050628	3,751548982	1,88E-09	1,19E-08
SSBP4	protein_coding	ENSG00000130511	-0,446305258	1,89E-09	1,19E-08
AL357033.4	antisense	ENSG00000277496	1,813992818	1,9E-09	0,000000012
PWP1	protein_coding	ENSG00000136045	-0,341323883	1,91E-09	0,000000012
FAM186B	protein_coding	ENSG00000135436	2,526916102	1,92E-09	1,21E-08
AGPAT4	protein_coding	ENSG00000026652	0,710736204	1,94E-09	1,22E-08
TANGO6	protein_coding	ENSG00000103047	-0,33078251	1,94E-09	1,22E-08
CKS1B	protein_coding	ENSG00000173207	-0,452228652	0,000000002	1,26E-08
HSD17B7P2	transcribed_unprocesse	ENSG00000099251	2,071757326	2,02E-09	1,27E-08
PPP1R26	protein_coding	ENSG00000196422	-0,553826033	2,03E-09	1,28E-08
FAM161A	protein_coding	ENSG00000170264	-0,599644984	2,05E-09	1,29E-08
GSDMD	protein_coding	ENSG00000104518	-0,362629059	2,06E-09	0,000000013
DHX35	protein_coding	ENSG00000101452	-0,387213359	2,1E-09	1,32E-08
LRPPRC	protein_coding	ENSG00000138095	-0,280896511	2,11E-09	1,33E-08
COIL	protein_coding	ENSG00000121058	-0,408866484	2,13E-09	1,34E-08
ALDH1B1	protein_coding	ENSG00000137124	-0,37168897	2,15E-09	1,35E-08
TMEM135	protein_coding	ENSG00000166575	0,373555635	2,17E-09	1,36E-08
SDC3	protein_coding	ENSG00000162512	-0,459905451	2,16E-09	1,36E-08
CDKN1A	protein_coding	ENSG00000124762	0,446227312	2,2E-09	1,38E-08
KIAA1614-AS1	antisense	ENSG00000232586	0,858529034	2,22E-09	1,39E-08
AC015936.1	lincRNA	ENSG00000267334	-1,409829389	2,22E-09	1,39E-08

MRM3	protein_coding	ENSG00000171861	-0,343553128	2,22E-09	1,39E-08
CPXM1	protein_coding	ENSG00000088882	5,439529137	2,25E-09	1,41E-08
SAP18	protein_coding	ENSG00000150459	0,389880442	2,24E-09	1,41E-08
RAD18	protein_coding	ENSG00000070950	-0,344165575	2,24E-09	1,41E-08
UCK1	protein_coding	ENSG00000130717	-0,346863036	2,25E-09	1,41E-08
LINC00863	bidirectional_promoter_	ENSG00000224914	0,776191651	2,27E-09	1,42E-08
USP28	protein_coding	ENSG00000048028	-0,278920341	2,27E-09	1,42E-08
MTERF2	protein_coding	ENSG00000120832	-0,606822064	2,27E-09	1,42E-08
ZNF256	protein_coding	ENSG00000152454	0,409152778	2,3E-09	1,44E-08
PPP3CC	protein_coding	ENSG00000120910	0,356177052	2,3E-09	1,44E-08
AC012158.1	transcribed_processed_	ENSG00000243024	1,135722537	2,32E-09	1,45E-08
MED12L	protein_coding	ENSG00000144893	1,075304799	2,33E-09	1,46E-08
ATP8B3	protein_coding	ENSG00000130270	-0,36828778	2,33E-09	1,46E-08
LOXL2	protein_coding	ENSG00000134013	-0,304255981	2,36E-09	1,47E-08
EIF2B4	protein_coding	ENSG00000115211	-0,361823463	2,36E-09	1,47E-08
BLOC1S4	protein_coding	ENSG00000186222	-0,543860129	2,35E-09	1,47E-08
NUP188	protein_coding	ENSG00000095319	-0,358626092	2,36E-09	1,48E-08
MEAF6	protein_coding	ENSG00000163875	-0,390997655	2,39E-09	1,49E-08
USO1	protein_coding	ENSG00000138768	0,338234881	2,4E-09	0,000000015
RNF144B	protein_coding	ENSG00000137393	0,368044973	2,42E-09	1,51E-08
FGFR1OP2	protein_coding	ENSG00000111790	0,411410774	2,44E-09	1,52E-08
NBPF26	protein_coding	ENSG00000273136	0,550538424	2,46E-09	1,53E-08
BRIP1	protein_coding	ENSG00000136492	-0,385862916	2,5E-09	1,56E-08
OLFM1	protein_coding	ENSG00000130558	-0,531383405	2,53E-09	1,57E-08
RPRD2	protein_coding	ENSG00000163125	0,391180256	2,54E-09	1,58E-08
SOGA1	protein_coding	ENSG00000149639	-0,48877705	2,53E-09	1,58E-08
L1CAM	protein_coding	ENSG00000198910	-0,458623167	2,56E-09	1,59E-08
ZBTB11	protein_coding	ENSG00000066422	-0,217950563	2,58E-09	0,000000016
MAN1A2	protein_coding	ENSG00000198162	0,318318051	2,6E-09	1,62E-08
CTNNB1	protein_coding	ENSG00000168036	-0,211836937	2,63E-09	1,64E-08
KRT17P4	transcribed_unprocesse	ENSG00000205312	5,356810716	2,64E-09	1,64E-08
SMS	protein_coding	ENSG00000102172	-0,383027081	2,65E-09	1,65E-08
TRAPPC12	protein_coding	ENSG00000171853	-0,331102024	2,69E-09	1,67E-08
ZNF217	protein_coding	ENSG00000171940	-0,290972922	2,7E-09	1,68E-08
PCNA	protein_coding	ENSG00000132646	-0,373226858	2,71E-09	1,68E-08
SDCBP2-AS1	antisense	ENSG00000234684	0,960959854	2,75E-09	1,71E-08
LY96	protein_coding	ENSG00000154589	0,897465684	2,76E-09	1,71E-08
GLIS2	protein_coding	ENSG00000126603	0,868079772	2,77E-09	1,71E-08
PFDN1	protein_coding	ENSG00000113068	0,380373852	2,76E-09	1,71E-08
PDCD2L	protein_coding	ENSG00000126249	-0,534536495	2,75E-09	1,71E-08

PEX2	protein_coding	ENSG00000164751	-0,553373608	2,77E-09	1,71E-08
LCOR	protein_coding	ENSG00000196233	0,379084901	2,77E-09	1,72E-08
ACSS2	protein_coding	ENSG00000131069	0,381507523	2,81E-09	1,74E-08
LINC01234	lincRNA	ENSG00000249550	-0,34160063	2,82E-09	1,75E-08
CLIP4	protein_coding	ENSG00000115295	0,279245325	2,82E-09	1,75E-08
ZEB2-AS1	antisense	ENSG00000238057	2,811733452	2,85E-09	1,76E-08
GPAM	protein_coding	ENSG00000119927	-0,393781758	2,87E-09	1,78E-08
LINC00431	transcribed_unprocesse	ENSG00000225760	6,864138003	2,92E-09	0,000000018
EVI5L	protein_coding	ENSG00000142459	0,439425418	2,94E-09	1,82E-08
MAPKAPK3	protein_coding	ENSG00000114738	-0,322561335	2,95E-09	1,82E-08
FPGT	protein_coding	ENSG00000254685	-0,542406658	2,98E-09	1,84E-08
MAPKAPK2	protein_coding	ENSG00000162889	0,301713196	3,02E-09	1,86E-08
FGD6	protein_coding	ENSG00000180263	0,274903115	3,02E-09	1,86E-08
LSMEM1	protein_coding	ENSG00000181016	2,348018148	3,05E-09	1,88E-08
NPHP4	protein_coding	ENSG00000131697	-0,517509231	3,08E-09	0,000000019
DGCR2	protein_coding	ENSG00000070413	-0,357603453	3,11E-09	1,92E-08
C12orf49	protein_coding	ENSG00000111412	0,269562988	3,17E-09	1,95E-08
SFXN1	protein_coding	ENSG00000164466	-0,339072679	3,17E-09	1,95E-08
NKPD1	protein_coding	ENSG00000179846	5,402571325	3,2E-09	1,97E-08
TBC1D22B	protein_coding	ENSG00000065491	0,348164298	3,22E-09	1,98E-08
SLC9A7	protein_coding	ENSG00000065923	0,303734927	3,23E-09	1,99E-08
ERMARD	protein_coding	ENSG00000130023	-0,44227249	3,23E-09	1,99E-08
RXRA	protein_coding	ENSG00000186350	-0,44413095	3,22E-09	1,99E-08
Z99774.1	lincRNA	ENSG00000206028	2,041744711	3,28E-09	2,02E-08
SCML2	protein_coding	ENSG00000102098	-0,392921745	3,32E-09	2,04E-08
FAM86DP	transcribed_unprocesse	ENSG00000244026	0,525946067	3,32E-09	2,04E-08
FIBP	protein_coding	ENSG00000172500	-0,320124877	3,34E-09	2,05E-08
TRIM28	protein_coding	ENSG00000130726	-0,334888037	3,33E-09	2,05E-08
FANCA	protein_coding	ENSG00000187741	-0,526904003	3,34E-09	2,05E-08
XKR8	protein_coding	ENSG00000158156	-0,570480444	3,33E-09	2,05E-08
FFAR2	protein_coding	ENSG00000126262	5,306758992	3,36E-09	2,06E-08
PRSS36	protein_coding	ENSG00000178226	1,545661489	3,36E-09	2,07E-08
MARCKS	protein_coding	ENSG00000277443	-0,415493915	3,37E-09	2,07E-08
ZACN	protein_coding	ENSG00000186919	4,825331032	3,42E-09	0,000000021
RNF114	protein_coding	ENSG00000124226	0,281488798	3,43E-09	0,000000021
RPL13AP20	processed_pseudogene	ENSG00000234498	-0,637670661	3,44E-09	2,11E-08
PIGU	protein_coding	ENSG00000101464	-0,396894037	3,45E-09	2,11E-08
ARHGAP31-AS1	antisense	ENSG00000241155	1,707255985	3,46E-09	2,12E-08
DHCR24	protein_coding	ENSG00000116133	-0,303922813	3,47E-09	2,13E-08
IWS1	protein_coding	ENSG00000163166	-0,362557975	3,48E-09	2,13E-08

C8orf86	protein_coding	ENSG00000196166	6,848218661	3,49E-09	2,14E-08
DUT	protein_coding	ENSG00000128951	-0,409652052	3,53E-09	2,16E-08
AL078604.2	lincRNA	ENSG00000237927	6,777097113	3,65E-09	2,23E-08
CLCA2	protein_coding	ENSG00000137975	1,273423664	3,65E-09	2,24E-08
PCBP1	protein_coding	ENSG00000169564	-0,271841723	3,67E-09	2,24E-08
CD2BP2	protein_coding	ENSG00000169217	-0,310799049	3,65E-09	2,24E-08
RRAS2	protein_coding	ENSG00000133818	0,302647631	3,68E-09	2,25E-08
SNORD12B	snoRNA	ENSG00000222365	-0,944451132	3,68E-09	2,25E-08
SEPT4-AS1	antisense	ENSG00000264672	2,239374481	3,71E-09	2,27E-08
AP002478.1	antisense	ENSG00000266401	2,162907518	3,72E-09	2,27E-08
TRABD	protein_coding	ENSG00000170638	-0,46821164	3,73E-09	2,28E-08
TLR6	protein_coding	ENSG00000174130	0,733326297	3,81E-09	2,32E-08
NEXN-AS1	antisense	ENSG00000235927	3,932675529	3,82E-09	2,33E-08
CEP170B	protein_coding	ENSG00000099814	0,611616961	3,83E-09	2,34E-08
SNX12	protein_coding	ENSG00000147164	-0,280323993	3,86E-09	2,35E-08
KRI1	protein_coding	ENSG00000129347	-0,382447593	3,87E-09	2,36E-08
NEDD9	protein_coding	ENSG00000111859	4,313766759	3,91E-09	2,38E-08
ADGRG1	protein_coding	ENSG00000205336	0,542358519	3,9E-09	2,38E-08
SNHG8	lincRNA	ENSG00000269893	-0,333328836	3,96E-09	2,41E-08
GOT1	protein_coding	ENSG00000120053	0,30894162	3,96E-09	2,41E-08
NRAS	protein_coding	ENSG00000213281	-0,370646635	3,96E-09	2,41E-08
ZNF432	protein_coding	ENSG00000256087	0,406378148	0,000000004	2,43E-08
CKAP5	protein_coding	ENSG00000175216	-0,346548301	3,99E-09	2,43E-08
DHX33	protein_coding	ENSG00000005100	-0,363906597	4,03E-09	2,45E-08
AP5M1	protein_coding	ENSG00000053770	0,328827375	4,05E-09	2,46E-08
ADIPOR1	protein_coding	ENSG00000159346	0,256296281	4,04E-09	2,46E-08
NAV2	protein_coding	ENSG00000166833	0,479709727	4,12E-09	0,000000025
TMEM200A	protein_coding	ENSG00000164484	-0,218304592	4,12E-09	0,000000025
RIMKLB	protein_coding	ENSG00000166532	-0,36706278	4,12E-09	0,000000025
BLCAP	protein_coding	ENSG00000166619	0,418670766	4,14E-09	2,51E-08
SMARCD1	protein_coding	ENSG00000066117	-0,347880725	4,14E-09	2,51E-08
CCDC117	protein_coding	ENSG00000159873	0,362023211	4,15E-09	2,52E-08
NR1H4	protein_coding	ENSG00000012504	4,512981845	4,2E-09	2,55E-08
DNER	protein_coding	ENSG00000187957	-0,197294076	4,26E-09	2,58E-08
TMEM150B	protein_coding	ENSG00000180061	6,803256937	4,27E-09	2,59E-08
LAMC2	protein_coding	ENSG00000058085	0,289791701	4,28E-09	2,59E-08
SGMS2	protein_coding	ENSG00000164023	0,349108062	4,29E-09	0,000000026
LEF1	protein_coding	ENSG00000138795	-0,630460914	4,29E-09	0,000000026
TMEM38A	protein_coding	ENSG00000072954	0,81796294	4,34E-09	2,63E-08
ZNF621	protein_coding	ENSG00000172888	0,339902047	4,37E-09	2,65E-08

ECSIT	protein_coding	ENSG00000130159	-0,421058858	4,42E-09	2,67E-08
PTRH1	protein_coding	ENSG00000187024	-0,443914743	4,41E-09	2,67E-08
TMEM44-AS1	antisense	ENSG00000231770	0,735348085	4,46E-09	2,69E-08
LINC02551	lincRNA	ENSG00000254842	2,587294262	4,46E-09	0,000000027
CDK20	protein_coding	ENSG00000156345	0,800352841	4,47E-09	0,000000027
SPIN1	protein_coding	ENSG00000106723	-0,267437348	4,47E-09	0,000000027
MED18	protein_coding	ENSG00000130772	-0,477447592	4,49E-09	2,71E-08
HAL	protein_coding	ENSG00000084110	2,186024581	4,51E-09	2,72E-08
MED26	protein_coding	ENSG00000105085	0,582517761	4,62E-09	2,79E-08
POLRMT	protein_coding	ENSG00000099821	-0,453705473	4,62E-09	2,79E-08
MED8	protein_coding	ENSG00000159479	-0,317659817	4,68E-09	2,82E-08
MRPS27	protein_coding	ENSG00000113048	-0,275360635	4,7E-09	2,83E-08
PNRC1	protein_coding	ENSG00000146278	0,405619509	4,74E-09	2,86E-08
WDR1	protein_coding	ENSG00000071127	0,169778548	4,76E-09	2,87E-08
AC027290.1	lincRNA	ENSG00000256152	4,977683595	4,8E-09	2,89E-08
KIF18A	protein_coding	ENSG00000121621	-0,441556948	4,8E-09	2,89E-08
CRYBG1	protein_coding	ENSG00000112297	1,89012158	4,84E-09	2,91E-08
FBL	protein_coding	ENSG00000105202	-0,292026786	4,9E-09	2,95E-08
C6orf47	protein_coding	ENSG00000204439	-0,438285882	4,91E-09	2,95E-08
AL590763.1	processed_pseudogene	ENSG00000215120	5,350838089	4,93E-09	2,97E-08
CLIP1	protein_coding	ENSG00000130779	0,345323063	4,94E-09	2,97E-08
EPPK1	protein_coding	ENSG00000261150	3,786853702	4,97E-09	2,99E-08
BAZ1B	protein_coding	ENSG00000009954	-0,39532643	5,06E-09	3,04E-08
CNN1	protein_coding	ENSG00000130176	2,115544534	5,14E-09	3,09E-08
NGDN	protein_coding	ENSG00000129460	-0,421290583	5,15E-09	3,09E-08
KIAA0825	protein_coding	ENSG00000185261	1,13202146	5,24E-09	3,15E-08
ACBD3	protein_coding	ENSG00000182827	0,293943227	5,38E-09	3,23E-08
LINC00518	lincRNA	ENSG00000183674	0,999621042	5,41E-09	3,25E-08
RHBDD2	protein_coding	ENSG00000005486	0,509544281	5,42E-09	3,25E-08
KHK	protein_coding	ENSG00000138030	-0,727291162	5,43E-09	3,26E-08
BAK1	protein_coding	ENSG00000030110	0,547687253	5,45E-09	3,27E-08
CCHCR1	protein_coding	ENSG00000204536	-0,39183204	5,48E-09	3,28E-08
LGR6	protein_coding	ENSG00000133067	3,527034797	5,5E-09	0,000000033
TES	protein_coding	ENSG00000135269	0,260032393	5,5E-09	0,000000033
CEBPA	protein_coding	ENSG00000245848	-1,057362588	5,5E-09	0,000000033
AC090236.2	sense_intronic	ENSG00000267504	-1,591268297	5,57E-09	3,34E-08
POLR3C	protein_coding	ENSG00000186141	0,298304319	5,6E-09	3,35E-08
AC005622.1	transcribed_unprocesse	ENSG00000283525	3,583678746	5,61E-09	3,36E-08
VSIG1	protein_coding	ENSG00000101842	5,257776356	5,64E-09	3,37E-08
IRF2BP2	protein_coding	ENSG00000168264	-0,274929526	5,73E-09	3,42E-08

NR2F2	protein_coding	ENSG00000185551	-0,294745921	5,75E-09	3,44E-08
COL16A1	protein_coding	ENSG00000084636	0,769695354	5,78E-09	3,46E-08
ZNF324	protein_coding	ENSG00000083812	0,608927289	5,79E-09	3,46E-08
PLPP1	protein_coding	ENSG00000067113	0,548690037	5,8E-09	3,46E-08
NAA20	protein_coding	ENSG00000173418	-0,427295786	5,83E-09	3,48E-08
CLCF1	protein_coding	ENSG00000175505	0,705877203	5,84E-09	3,49E-08
SERPINA10	protein_coding	ENSG00000140093	5,360793901	5,88E-09	3,51E-08
BRAT1	protein_coding	ENSG00000106009	-0,425594578	5,93E-09	3,54E-08
GLE1	protein_coding	ENSG00000119392	-0,232649756	5,95E-09	3,55E-08
EMP2	protein_coding	ENSG00000213853	-0,376314019	5,94E-09	3,55E-08
CFAP58	protein_coding	ENSG00000120051	1,236301222	6,16E-09	3,67E-08
DNAJC18	protein_coding	ENSG00000170464	0,379782831	6,16E-09	3,67E-08
NBPF1	protein_coding	ENSG00000219481	0,307255472	6,18E-09	3,68E-08
CASC3	protein_coding	ENSG00000108349	-0,243996591	6,21E-09	0,000000037
ADGRA3	protein_coding	ENSG00000152990	-0,438511908	6,22E-09	0,000000037
TFRC	protein_coding	ENSG00000072274	-0,373734861	6,23E-09	3,71E-08
TBC1D8B	protein_coding	ENSG00000133138	0,432890602	6,25E-09	3,72E-08
AC015813.6	TEC	ENSG00000279207	0,784856798	6,27E-09	3,73E-08
VWCE	protein_coding	ENSG00000167992	1,287296848	6,39E-09	0,000000038
LRFN3	protein_coding	ENSG00000126243	-0,583139975	6,4E-09	3,81E-08
SLC4A11	protein_coding	ENSG00000088836	0,912936886	6,44E-09	3,83E-08
PPP1R8	protein_coding	ENSG00000117751	-0,305825643	6,48E-09	3,85E-08
ALOX12B	protein_coding	ENSG00000179477	3,091452775	6,63E-09	3,93E-08
GATAD2B	protein_coding	ENSG00000143614	0,470355038	6,62E-09	3,93E-08
PABPC1	protein_coding	ENSG00000070756	0,23151949	6,62E-09	3,93E-08
DSCC1	protein_coding	ENSG00000136982	-0,519886287	6,69E-09	3,97E-08
CAD	protein_coding	ENSG00000084774	-0,449001332	6,7E-09	3,98E-08
ZNF341-AS1	antisense	ENSG00000230753	6,762407975	6,77E-09	4,02E-08
YIPF1	protein_coding	ENSG00000058799	0,357012721	6,77E-09	4,02E-08
BLOC1S2	protein_coding	ENSG00000196072	0,331402771	6,82E-09	4,04E-08
DPP8	protein_coding	ENSG00000074603	0,268378209	6,81E-09	4,04E-08
CHERP	protein_coding	ENSG00000085872	-0,492970362	6,82E-09	4,04E-08
CRACR2A	protein_coding	ENSG00000130038	-0,519824914	6,82E-09	4,04E-08
TRIM45	protein_coding	ENSG00000134253	-0,634336857	6,84E-09	4,05E-08
THOC3	protein_coding	ENSG00000051596	-0,435917889	6,91E-09	4,09E-08
POLN	protein_coding	ENSG00000130997	0,974835901	6,94E-09	4,11E-08
TFDP1	protein_coding	ENSG00000198176	-0,270948209	6,95E-09	4,11E-08
TIAM2	protein_coding	ENSG00000146426	0,788557652	6,99E-09	4,14E-08
C12orf10	protein_coding	ENSG00000139637	-0,370026458	7,02E-09	4,15E-08
SPC25	protein_coding	ENSG00000152253	-0,505749658	7,09E-09	4,19E-08

HEG1	protein_coding	ENSG00000173706	-0,445516164	7,15E-09	4,22E-08
WDR89	protein_coding	ENSG00000140006	-0,38616263	7,18E-09	4,24E-08
SEMA4F	protein_coding	ENSG00000135622	-0,466753354	7,25E-09	4,28E-08
UBC	protein_coding	ENSG00000150991	0,231813335	7,3E-09	4,31E-08
NME2	protein_coding	ENSG00000243678	-1,088164244	7,32E-09	4,32E-08
ALDH8A1	protein_coding	ENSG00000118514	2,57334863	7,5E-09	4,42E-08
F11R	protein_coding	ENSG00000158769	0,424145577	7,49E-09	4,42E-08
FYCO1	protein_coding	ENSG00000163820	-0,447834512	7,5E-09	4,42E-08
ARHGAP5	protein_coding	ENSG00000100852	0,329954264	7,56E-09	4,46E-08
C5orf51	protein_coding	ENSG00000205765	0,321488331	7,64E-09	4,51E-08
AC139718.1	lincRNA	ENSG00000248432	1,026757549	7,71E-09	4,54E-08
NME6	protein_coding	ENSG00000172113	-0,395566434	7,71E-09	4,54E-08
OARD1	protein_coding	ENSG00000124596	-0,406852295	7,83E-09	4,61E-08
TICAM1	protein_coding	ENSG00000127666	0,565870826	7,87E-09	4,63E-08
HNRNPL	protein_coding	ENSG00000104824	-0,209251987	7,86E-09	4,63E-08
CASP4	protein_coding	ENSG00000196954	0,3091981	7,93E-09	4,67E-08
CNPPD1	protein_coding	ENSG00000115649	-0,361686929	7,96E-09	4,69E-08
TBRG4	protein_coding	ENSG00000136270	-0,327309202	8,05E-09	4,73E-08
GLS	protein_coding	ENSG00000115419	0,305013406	8,11E-09	4,77E-08
RNASEL	protein_coding	ENSG00000135828	0,751083823	8,14E-09	4,78E-08
IL24	protein_coding	ENSG00000162892	-0,615805062	8,18E-09	4,81E-08
AL049840.3	sense_intronic	ENSG00000269940	-0,588589649	8,21E-09	4,82E-08
ARHGEF26	protein_coding	ENSG00000114790	-0,561014019	8,22E-09	4,83E-08
OCIAD2	protein_coding	ENSG00000145247	0,400340647	8,32E-09	4,89E-08
URI1	protein_coding	ENSG00000105176	-0,369461388	8,43E-09	4,95E-08
C17orf75	protein_coding	ENSG00000108666	-0,40018669	8,52E-09	0,00000005
PITRM1	protein_coding	ENSG00000107959	0,221474263	8,53E-09	5,01E-08
ZNF603P	unprocessed_pseudoge	ENSG00000216901	3,364101264	8,56E-09	5,02E-08
SUGP2	protein_coding	ENSG00000064607	-0,398925023	8,61E-09	5,05E-08
BMP1	protein_coding	ENSG00000168487	0,53206556	8,65E-09	5,07E-08
ARHGAP28	protein_coding	ENSG00000088756	0,786509137	8,73E-09	5,12E-08
AC096636.1	TEC	ENSG00000279333	1,979332318	8,78E-09	5,14E-08
TRPM6	protein_coding	ENSG00000119121	1,673577202	8,81E-09	5,16E-08
PARP6	protein_coding	ENSG00000137817	0,351594428	8,82E-09	5,16E-08
ACTL6A	protein_coding	ENSG00000136518	-0,374216826	8,8E-09	5,16E-08
IDS	protein_coding	ENSG00000010404	0,319440632	8,83E-09	5,17E-08
ENKD1	protein_coding	ENSG00000124074	-0,46242674	8,9E-09	5,21E-08
POLR3G	protein_coding	ENSG00000113356	-0,523932114	8,96E-09	5,24E-08
MTHFD1L	protein_coding	ENSG00000120254	-0,264876028	9,03E-09	5,28E-08
ICAM4	protein_coding	ENSG00000105371	4,474956958	9,09E-09	5,31E-08

PHC2	protein_coding	ENSG00000134686	-0,308501172	9,13E-09	5,33E-08
MRPS21	protein_coding	ENSG00000266472	-0,336198585	9,14E-09	5,34E-08
AL138749.1	lincRNA	ENSG00000226798	2,189801445	9,19E-09	5,36E-08
SMIM13	protein_coding	ENSG00000224531	-0,388962833	9,19E-09	5,36E-08
RBM14	protein_coding	ENSG00000239306	-0,41656461	9,18E-09	5,36E-08
SARS	protein_coding	ENSG00000031698	0,333089974	9,28E-09	5,42E-08
LINC00882	lincRNA	ENSG00000242759	1,202338441	9,31E-09	5,43E-08
AQP3	protein_coding	ENSG00000165272	2,520046987	9,47E-09	5,52E-08
SRPX2	protein_coding	ENSG00000102359	0,541580674	9,69E-09	5,65E-08
URM1	protein_coding	ENSG00000167118	-0,377957317	9,69E-09	5,65E-08
STIL	protein_coding	ENSG00000123473	-0,347898032	9,76E-09	5,69E-08
SLX4	protein_coding	ENSG00000188827	-0,587882615	9,83E-09	5,72E-08
TBC1D9B	protein_coding	ENSG00000197226	-0,370080558	9,94E-09	5,79E-08
ABHD10	protein_coding	ENSG00000144827	-0,426200016	9,99E-09	5,81E-08
NFKBIB	protein_coding	ENSG00000104825	0,421672812	0,00000001	5,83E-08
SGCB	protein_coding	ENSG00000163069	0,358614014	1,01E-08	5,86E-08
PDHX	protein_coding	ENSG00000110435	0,28322202	1,01E-08	5,88E-08
P4HA1	protein_coding	ENSG00000122884	0,339896343	1,02E-08	5,91E-08
RCAN3	protein_coding	ENSG00000117602	-0,358934888	1,02E-08	5,91E-08
CADPS2	protein_coding	ENSG00000081803	0,3927376	1,03E-08	0,00000006
PRDM2	protein_coding	ENSG00000116731	0,463891226	1,05E-08	6,07E-08
TGM5	protein_coding	ENSG00000104055	2,114384875	1,05E-08	6,09E-08
C5orf34	protein_coding	ENSG00000172244	0,463490701	1,06E-08	6,14E-08
VASP	protein_coding	ENSG00000125753	0,40493921	1,06E-08	6,18E-08
ARHGAP30	protein_coding	ENSG00000186517	3,05555609	1,08E-08	6,25E-08
STK17A	protein_coding	ENSG00000164543	0,355218807	1,09E-08	6,31E-08
LINC02328	lincRNA	ENSG00000258733	1,745560497	1,09E-08	6,32E-08
PTK2B	protein_coding	ENSG00000120899	0,898397597	0,000000011	0,000000064
KIAA0753	protein_coding	ENSG00000198920	0,255310651	1,11E-08	6,41E-08
MNX1	protein_coding	ENSG00000130675	-0,662722144	1,11E-08	6,43E-08
SAXO2	protein_coding	ENSG00000188659	2,014475868	1,11E-08	6,44E-08
CCNK	protein_coding	ENSG00000090061	0,53025195	1,11E-08	6,44E-08
PNRC2	protein_coding	ENSG00000189266	-0,371502539	1,11E-08	6,44E-08
AC018464.1	lincRNA	ENSG00000234520	0,922733147	1,12E-08	6,49E-08
PTK7	protein_coding	ENSG00000112655	-0,489238184	1,12E-08	0,000000065
KPNA1	protein_coding	ENSG00000114030	0,188118152	1,12E-08	6,51E-08
AC084824.5	antisense	ENSG00000276148	2,668876109	1,13E-08	6,52E-08
STEAP1B	protein_coding	ENSG00000105889	-0,293912549	1,14E-08	0,000000066
RAI1	protein_coding	ENSG00000108557	-0,508882048	1,15E-08	6,62E-08
ELP1	protein_coding	ENSG00000070061	-0,30769167	1,17E-08	6,77E-08

TNNC1	protein_coding	ENSG00000114854	1,639102589	1,18E-08	6,79E-08
FRK	protein_coding	ENSG00000111816	1,074207853	0,000000012	6,92E-08
CCDC28B	protein_coding	ENSG00000160050	-0,923144692	1,21E-08	0,00000007
SNORD14E	snoRNA	ENSG00000200879	-1,532999457	1,22E-08	7,04E-08
KDM3B	protein_coding	ENSG00000120733	-0,260967191	1,22E-08	7,05E-08
CCDC8	protein_coding	ENSG00000169515	-0,628768086	1,22E-08	7,05E-08
COL12A1	protein_coding	ENSG00000111799	0,379417209	1,24E-08	7,17E-08
TEAD4	protein_coding	ENSG00000197905	-0,379562681	1,24E-08	7,17E-08
LINC02340	lincRNA	ENSG00000275294	2,393035578	1,25E-08	7,22E-08
AP005264.1	lincRNA	ENSG00000267069	1,028301238	1,25E-08	7,22E-08
VIPAS39	protein_coding	ENSG00000151445	-0,38472264	1,26E-08	7,25E-08
SNORD20	snoRNA	ENSG00000207280	-1,551601789	1,26E-08	7,28E-08
PIMREG	protein_coding	ENSG00000129195	-0,319156031	1,29E-08	7,42E-08
KCTD18	protein_coding	ENSG00000155729	0,344897725	0,000000013	7,46E-08
RGS12	protein_coding	ENSG00000159788	-0,450957543	0,000000013	7,46E-08
AF131215.5	sense_intronic	ENSG00000255310	0,721053482	0,000000013	7,46E-08
AC104534.1	antisense	ENSG00000268756	4,693705317	0,000000013	7,49E-08
AP000904.1	lincRNA	ENSG00000273415	2,882877835	1,31E-08	7,51E-08
EVI2A	protein_coding	ENSG00000126860	-0,594981793	1,31E-08	7,52E-08
HIRIP3	protein_coding	ENSG00000149929	-0,399276992	1,34E-08	7,69E-08
ASCC3	protein_coding	ENSG00000112249	0,284403146	1,34E-08	7,71E-08
LNX2	protein_coding	ENSG00000139517	0,453034163	1,34E-08	7,72E-08
ARCN1	protein_coding	ENSG00000095139	0,202806775	1,35E-08	7,73E-08
ZNF485	protein_coding	ENSG00000198298	-0,619264202	1,35E-08	7,76E-08
TCTN2	protein_coding	ENSG00000168778	-0,42799176	1,36E-08	7,78E-08
CDC42SE1	protein_coding	ENSG00000197622	0,201105984	1,36E-08	0,000000078
NUP43	protein_coding	ENSG00000120253	-0,280974702	1,37E-08	7,86E-08
BOP1	protein_coding	ENSG00000261236	-0,349488472	1,38E-08	7,93E-08
FEZ1	protein_coding	ENSG00000149557	4,704744152	0,000000014	8,03E-08
CYP27B1	protein_coding	ENSG00000111012	0,542555244	1,41E-08	8,07E-08
SRSF5	protein_coding	ENSG00000100650	-0,312167075	1,42E-08	8,11E-08
DDHD2	protein_coding	ENSG00000085788	-0,269163663	1,42E-08	8,13E-08
METTL1	protein_coding	ENSG00000037897	-0,426888032	1,42E-08	8,13E-08
ZNF589	protein_coding	ENSG00000164048	-0,550540534	1,42E-08	8,14E-08
AMDHD1	protein_coding	ENSG00000139344	1,020625168	1,42E-08	8,15E-08
MRPL30	protein_coding	ENSG00000185414	-0,314419838	1,43E-08	8,16E-08
PHKA2	protein_coding	ENSG00000044446	-0,346645701	1,46E-08	8,33E-08
SNORD15B	snoRNA	ENSG00000207445	-0,676272384	1,49E-08	0,000000085
FSD1L	protein_coding	ENSG00000106701	0,640614344	1,49E-08	8,53E-08
PIR	protein_coding	ENSG00000087842	0,40192337	1,49E-08	8,53E-08

WASHC4	protein_coding	ENSG00000136051	0,277971413	0,000000015	8,59E-08
RBL1	protein_coding	ENSG00000080839	-0,423334087	0,000000015	8,59E-08
C1QL4	protein_coding	ENSG00000186897	-0,722965986	0,000000015	8,59E-08
IQCC	protein_coding	ENSG00000160051	-0,476605798	1,55E-08	8,85E-08
AC068647.2	transcribed_unprocesse	ENSG00000250271	0,908278048	1,55E-08	8,85E-08
LARS	protein_coding	ENSG00000133706	-0,308007969	1,55E-08	8,86E-08
VANGL2	protein_coding	ENSG00000162738	6,761717725	1,56E-08	8,92E-08
MLLT3	protein_coding	ENSG00000171843	0,278679268	1,57E-08	8,95E-08
COL11A1	protein_coding	ENSG00000060718	2,429290527	1,58E-08	9,02E-08
MAP3K4	protein_coding	ENSG00000085511	-0,324009943	1,58E-08	9,03E-08
PACRGL	protein_coding	ENSG00000163138	-0,393624063	1,61E-08	9,16E-08
APBA3	protein_coding	ENSG00000011132	0,53189927	1,61E-08	9,17E-08
SRI	protein_coding	ENSG00000075142	-0,366808216	1,61E-08	9,17E-08
HSD11B1-AS1	antisense	ENSG00000227591	1,291275023	1,62E-08	9,24E-08
PRADC1	protein_coding	ENSG00000135617	-0,479591016	1,64E-08	9,34E-08
RPSAP12	processed_pseudogene	ENSG00000240087	1,429808656	1,67E-08	9,53E-08
HNRNPA1P48	protein_coding	ENSG00000224578	-0,639417385	1,68E-08	9,58E-08
FAM131B	protein_coding	ENSG00000159784	4,841080021	1,69E-08	9,59E-08
CCT6A	protein_coding	ENSG00000146731	-0,303299006	1,69E-08	9,63E-08
PCID2	protein_coding	ENSG00000126226	-0,336904332	0,000000017	9,64E-08
RTN4IP1	protein_coding	ENSG00000130347	-0,421578984	0,000000017	9,64E-08
RRP7A	protein_coding	ENSG00000189306	-0,303798694	0,000000017	9,67E-08
TMA16	protein_coding	ENSG00000198498	-0,372509327	1,71E-08	9,71E-08
VLDLR-AS1	antisense	ENSG00000236404	0,924040516	1,71E-08	9,73E-08
TRIM8	protein_coding	ENSG00000171206	0,42195919	1,72E-08	9,73E-08
PPCDC	protein_coding	ENSG00000138621	-0,41745699	1,74E-08	9,86E-08
CHD6	protein_coding	ENSG00000124177	0,379306039	1,76E-08	9,96E-08
ST8SIA4	protein_coding	ENSG00000113532	0,842006573	1,78E-08	0,000000101
DPH7	protein_coding	ENSG00000148399	-0,396674527	1,78E-08	0,000000101
PDK2	protein_coding	ENSG00000005882	-0,346183431	1,81E-08	0,000000103
LINC00880	lincRNA	ENSG00000243629	4,74925776	1,85E-08	0,000000105
DISP1	protein_coding	ENSG00000154309	0,814303328	1,85E-08	0,000000105
RBBP7	protein_coding	ENSG00000102054	-0,245830462	1,87E-08	0,000000106
UGT2B7	protein_coding	ENSG00000171234	6,532364365	1,89E-08	0,000000107
COL8A1	protein_coding	ENSG00000144810	0,85846993	1,89E-08	0,000000107
ZFYVE21	protein_coding	ENSG00000100711	-0,441829501	0,000000019	0,000000107
IL17RD	protein_coding	ENSG00000144730	-0,499589382	1,89E-08	0,000000107
PTPN6	protein_coding	ENSG00000111679	3,132203562	1,92E-08	0,000000108
UACA	protein_coding	ENSG00000137831	-0,283239896	1,91E-08	0,000000108
POLQ	protein_coding	ENSG00000051341	-0,364881037	1,94E-08	0,00000011

QSOX2	protein_coding	ENSG00000165661	-0,369721099	1,94E-08	0,00000011
RPS6KL1	protein_coding	ENSG00000198208	4,743954553	1,97E-08	0,000000111
USP8	protein_coding	ENSG00000138592	0,269284731	1,97E-08	0,000000111
OGG1	protein_coding	ENSG00000114026	-0,405516098	1,98E-08	0,000000112
AL109955.1	antisense	ENSG00000218018	6,751267165	2,01E-08	0,000000113
CHMP2B	protein_coding	ENSG00000083937	0,398686306	2,02E-08	0,000000114
DHTKD1	protein_coding	ENSG00000181192	-0,249420073	2,01E-08	0,000000114
AC104653.1	lincRNA	ENSG00000228857	2,887892119	2,07E-08	0,000000117
CEBPZOS	protein_coding	ENSG00000218739	-0,39322175	2,11E-08	0,000000119
AP5Z1	protein_coding	ENSG00000242802	0,564040172	2,14E-08	0,000000121
ZNF155	protein_coding	ENSG00000204920	0,374746505	2,14E-08	0,000000121
PCYT2	protein_coding	ENSG00000185813	-0,355096352	2,15E-08	0,000000121
OR7E122P	transcribed_unprocesse	ENSG00000215160	6,704151977	2,14E-08	0,000000121
WARS2	protein_coding	ENSG00000116874	-0,373491221	2,19E-08	0,000000123
DCK	protein_coding	ENSG00000156136	-0,588994837	2,18E-08	0,000000123
SERPINB1	protein_coding	ENSG00000021355	0,323959303	2,21E-08	0,000000124
C2CD2	protein_coding	ENSG00000157617	-0,330343608	0,000000022	0,000000124
AC004980.1	transcribed_unprocesse	ENSG00000205485	0,962333316	2,21E-08	0,000000124
STAT5A	protein_coding	ENSG00000126561	4,25338988	2,22E-08	0,000000125
UBR7	protein_coding	ENSG00000012963	-0,360620709	2,22E-08	0,000000125
HLA-DOB	protein_coding	ENSG00000241106	2,469826458	2,23E-08	0,000000126
CLEC2B	protein_coding	ENSG00000110852	0,913858664	2,27E-08	0,000000127
CLN8	protein_coding	ENSG00000182372	0,354500273	2,26E-08	0,000000127
YME1L1	protein_coding	ENSG00000136758	0,207288543	2,29E-08	0,000000128
SMUG1	protein_coding	ENSG00000123415	-0,355825472	2,28E-08	0,000000128
HOXC6	protein_coding	ENSG00000197757	0,754756187	2,31E-08	0,000000129
CENPE	protein_coding	ENSG00000138778	-0,496961814	2,31E-08	0,000000129
SRP14-AS1	bidirectional_promoter_	ENSG00000248508	0,90854015	2,31E-08	0,00000013
AL356234.3	lincRNA	ENSG00000283265	0,8805671	2,31E-08	0,00000013
VCL	protein_coding	ENSG00000035403	0,234269608	2,32E-08	0,00000013
HEATR3	protein_coding	ENSG00000155393	-0,431869351	2,34E-08	0,000000131
PRRC2A	protein_coding	ENSG00000204469	-0,489696146	2,35E-08	0,000000131
MYO16	protein_coding	ENSG00000041515	3,314750157	2,39E-08	0,000000134
IVD	protein_coding	ENSG00000128928	-0,35256274	2,42E-08	0,000000135
NEK7	protein_coding	ENSG00000151414	-0,366407277	2,45E-08	0,000000137
AC024451.2	processed_pseudogene	ENSG00000253330	3,54493225	2,46E-08	0,000000138
SNIP1	protein_coding	ENSG00000163877	0,315238165	2,48E-08	0,000000139
CBX1P3	processed_pseudogene	ENSG00000237379	5,215452631	2,52E-08	0,000000141
NIFK-AS1	antisense	ENSG00000236859	-0,529702455	2,53E-08	0,000000142
VSIR	protein_coding	ENSG00000107738	0,512095713	2,58E-08	0,000000144

BYSL	protein_coding	ENSG00000112578	-0,256859643	0,000000026	0,000000145
ATXN1	protein_coding	ENSG00000124788	0,364686851	2,62E-08	0,000000146
ME3	protein_coding	ENSG00000151376	0,392858193	2,63E-08	0,000000147
ZNF440	protein_coding	ENSG00000171295	0,702257743	2,65E-08	0,000000148
MKLN1	protein_coding	ENSG00000128585	0,231942284	2,65E-08	0,000000148
LSM8	protein_coding	ENSG00000128534	-0,371659489	2,66E-08	0,000000148
TRMT12	protein_coding	ENSG00000183665	-0,427697792	2,65E-08	0,000000148
ZNF341	protein_coding	ENSG00000131061	-0,632225399	2,64E-08	0,000000148
ALKBH4	protein_coding	ENSG00000160993	-0,52065731	2,67E-08	0,000000149
RNVU1-3	snRNA	ENSG00000201183	-2,811854346	2,67E-08	0,000000149
TMEM186	protein_coding	ENSG00000184857	-0,543408543	2,69E-08	0,00000015
MUC20	protein_coding	ENSG00000176945	3,735280011	2,71E-08	0,000000151
GDPD1	protein_coding	ENSG00000153982	0,680283918	2,72E-08	0,000000152
VPS36	protein_coding	ENSG00000136100	-0,332564457	2,73E-08	0,000000152
OGDH	protein_coding	ENSG00000105953	0,29228357	2,75E-08	0,000000153
SUV39H2	protein_coding	ENSG00000152455	-0,362952405	2,76E-08	0,000000154
AL021807.1	lincRNA	ENSG00000272468	1,392910935	2,79E-08	0,000000155
AC084824.1	processed_pseudogene	ENSG00000257511	1,1889833	2,78E-08	0,000000155
TCEA3	protein_coding	ENSG00000204219	0,943043472	2,78E-08	0,000000155
ICMT	protein_coding	ENSG00000116237	-0,261895693	2,78E-08	0,000000155
TMEM168	protein_coding	ENSG00000146802	-0,378288257	2,78E-08	0,000000155
LINC01226	processed_transcript	ENSG00000284543	4,591616443	2,82E-08	0,000000157
CEACAM20	protein_coding	ENSG00000273777	6,682692066	2,85E-08	0,000000158
IPO8	protein_coding	ENSG00000133704	-0,324521648	2,85E-08	0,000000158
EXOC8	protein_coding	ENSG00000116903	0,28195857	2,87E-08	0,000000159
PARP1	protein_coding	ENSG00000143799	-0,294294841	2,86E-08	0,000000159
BLM	protein_coding	ENSG00000197299	-0,360753683	2,86E-08	0,000000159
WDR7	protein_coding	ENSG00000091157	0,320002005	2,89E-08	0,00000016
RASSF3	protein_coding	ENSG00000153179	0,258509296	2,88E-08	0,00000016
THRAP3	protein_coding	ENSG00000054118	-0,298332447	2,89E-08	0,00000016
PPIL4	protein_coding	ENSG00000131013	0,398780497	2,91E-08	0,000000161
HMGA2	protein_coding	ENSG00000149948	-0,306090479	0,000000029	0,000000161
ARRDC4	protein_coding	ENSG00000140450	-0,369858086	2,93E-08	0,000000162
SNHG4	lincRNA	ENSG00000281398	-0,622846794	2,94E-08	0,000000163
ZNF233	protein_coding	ENSG00000159915	0,603555794	2,96E-08	0,000000164
GCNT3	protein_coding	ENSG00000140297	0,564095984	2,96E-08	0,000000164
AREG	protein_coding	ENSG00000109321	1,130830756	2,98E-08	0,000000165
PTRH2	protein_coding	ENSG00000141378	-0,355716027	2,99E-08	0,000000165
LINC02098	lincRNA	ENSG00000272575	2,506169708	3,03E-08	0,000000167
GRK6	protein_coding	ENSG00000198055	-0,278591504	3,02E-08	0,000000167

ZFP64	protein_coding	ENSG00000020256	-0,352695491	3,02E-08	0,000000167
CLCC1	protein_coding	ENSG00000121940	-0,278062314	3,04E-08	0,000000168
POLR3K	protein_coding	ENSG00000161980	-0,514779128	3,06E-08	0,000000169
SFT2D3	protein_coding	ENSG00000173349	-0,515904313	3,06E-08	0,000000169
ZNF724	protein_coding	ENSG00000196081	-0,798169261	3,05E-08	0,000000169
PSMD10	protein_coding	ENSG00000101843	-0,359925774	3,08E-08	0,00000017
ADAMTS16	protein_coding	ENSG00000145536	0,374485335	3,14E-08	0,000000173
AK3	protein_coding	ENSG00000147853	0,358355499	3,14E-08	0,000000173
MARK3	protein_coding	ENSG00000075413	0,226211314	3,15E-08	0,000000174
GTF2E1	protein_coding	ENSG00000153767	-0,340023159	3,18E-08	0,000000175
BRSK2	protein_coding	ENSG00000174672	-0,553044019	0,000000032	0,000000176
SNORA75	snoRNA	ENSG00000206885	-2,102562865	3,21E-08	0,000000177
ISG20L2	protein_coding	ENSG00000143319	-0,3238485	3,24E-08	0,000000179
AC002401.4	lincRNA	ENSG00000276851	1,433845895	3,28E-08	0,000000181
FAM98A	protein_coding	ENSG00000119812	-0,26981627	3,33E-08	0,000000183
GSDME	protein_coding	ENSG00000105928	-0,319424458	3,32E-08	0,000000183
TIMM50	protein_coding	ENSG00000105197	-0,30590663	3,34E-08	0,000000184
ALOXE3	protein_coding	ENSG00000179148	1,030546389	3,37E-08	0,000000186
PRKACB	protein_coding	ENSG00000142875	0,398398268	3,39E-08	0,000000186
SHPK	protein_coding	ENSG00000197417	-0,656807575	3,41E-08	0,000000187
HNRNPA2B1	protein_coding	ENSG00000122566	-0,434994223	3,44E-08	0,000000189
TRA2A	protein_coding	ENSG00000164548	0,279798748	3,48E-08	0,000000191
NMNAT1	protein_coding	ENSG00000173614	0,536232069	3,49E-08	0,000000192
POLDIP3	protein_coding	ENSG00000100227	-0,307044373	3,49E-08	0,000000192
CHCHD5	protein_coding	ENSG00000125611	-0,388802004	3,52E-08	0,000000193
LRP1	protein_coding	ENSG00000123384	0,578606475	3,53E-08	0,000000194
SPOCK1	protein_coding	ENSG00000152377	0,267981837	3,53E-08	0,000000194
ATP2A2	protein_coding	ENSG00000174437	0,309154459	3,55E-08	0,000000195
DLGAP1	protein_coding	ENSG00000170579	4,504699567	0,000000036	0,000000197
UBE2G1	protein_coding	ENSG00000132388	-0,404538314	0,000000036	0,000000197
LINC02599	lincRNA	ENSG00000233858	2,066085873	3,62E-08	0,000000198
IL13RA2	protein_coding	ENSG00000123496	0,846739979	3,62E-08	0,000000198
ZBTB49	protein_coding	ENSG00000168826	0,624997427	0,000000036	0,000000198
EID2B	protein_coding	ENSG00000176401	-0,816024682	3,62E-08	0,000000198
DMTF1	protein_coding	ENSG00000135164	-0,329388762	3,62E-08	0,000000199
PRKG1-AS1	antisense	ENSG00000236671	0,920310619	3,66E-08	0,0000002
LINC00240	lincRNA	ENSG00000224843	1,183558772	3,67E-08	0,000000201
E2F7	protein_coding	ENSG00000165891	0,318106194	3,69E-08	0,000000202
PLEKHM1	protein_coding	ENSG00000225190	0,441399801	3,72E-08	0,000000204
EFTUD2	protein_coding	ENSG00000108883	-0,234705759	3,73E-08	0,000000204

MLH1	protein_coding	ENSG00000076242	-0,213255079	3,74E-08	0,000000205
HMSD	protein_coding	ENSG00000221887	0,552590758	3,77E-08	0,000000206
LAS1L	protein_coding	ENSG00000001497	-0,274130351	3,78E-08	0,000000206
RPL36AL	protein_coding	ENSG00000165502	-0,340312312	3,78E-08	0,000000206
KMO	protein_coding	ENSG00000117009	1,935923511	3,78E-08	0,000000207
CEP44	protein_coding	ENSG00000164118	-0,424451801	0,000000038	0,000000207
ABCE1	protein_coding	ENSG00000164163	-0,388611647	3,84E-08	0,000000209
PAPLN	protein_coding	ENSG00000100767	-0,588510705	3,83E-08	0,000000209
EXT1	protein_coding	ENSG00000182197	-0,179215919	3,84E-08	0,00000021
RELT	protein_coding	ENSG00000054967	0,455690721	3,87E-08	0,000000211
LGI4	protein_coding	ENSG00000153902	4,071920783	0,000000039	0,000000212
FYB1	protein_coding	ENSG00000082074	2,725575105	3,89E-08	0,000000212
SPR	protein_coding	ENSG00000116096	-0,30545002	0,000000039	0,000000212
RNU1-122P	snRNA	ENSG00000202408	-2,996329411	0,000000039	0,000000212
PML	protein_coding	ENSG00000140464	0,515900203	3,95E-08	0,000000215
PHF2	protein_coding	ENSG00000197724	-0,470234566	3,97E-08	0,000000216
RNU4-2	snRNA	ENSG00000202538	-0,540051022	3,98E-08	0,000000217
TMCO1	protein_coding	ENSG00000143183	0,311172939	4,01E-08	0,000000218
SMG8	protein_coding	ENSG00000167447	0,259630142	4,01E-08	0,000000218
LINC01578	processed_transcript	ENSG00000272888	0,540266911	4,02E-08	0,000000219
LCE1C	protein_coding	ENSG00000197084	1,945882776	4,02E-08	0,000000219
TTL	protein_coding	ENSG00000114999	-0,277928943	4,03E-08	0,000000219
CCT3	protein_coding	ENSG00000163468	-0,300904062	4,05E-08	0,00000022
KNTC1	protein_coding	ENSG00000184445	-0,31847106	4,04E-08	0,00000022
LINC01933	lincRNA	ENSG00000254226	2,749022601	4,06E-08	0,000000221
SERINC2	protein_coding	ENSG00000168528	0,596777661	4,08E-08	0,000000221
HADH	protein_coding	ENSG00000138796	-0,376730578	4,09E-08	0,000000222
BSDC1	protein_coding	ENSG00000160058	0,367328114	4,12E-08	0,000000224
MAP3K10	protein_coding	ENSG00000130758	0,527242199	0,000000042	0,000000228
NGFR	protein_coding	ENSG00000064300	2,577590489	4,22E-08	0,000000229
B4GALT1-AS1	antisense	ENSG00000233554	2,248407063	4,24E-08	0,00000023
SRRM1P2	processed_pseudogene	ENSG00000242195	3,078192376	4,27E-08	0,000000231
DPH1	protein_coding	ENSG00000108963	-0,312700613	4,28E-08	0,000000232
CENPJ	protein_coding	ENSG00000151849	-0,403304377	0,000000043	0,000000233
CD24	protein_coding	ENSG00000272398	-0,725897653	4,31E-08	0,000000233
SLCO2B1	protein_coding	ENSG00000137491	2,543890352	4,33E-08	0,000000234
CTDSP2	protein_coding	ENSG00000175215	-0,371324564	4,34E-08	0,000000235
TTC30B	protein_coding	ENSG00000196659	-0,526155924	4,36E-08	0,000000236
NASP	protein_coding	ENSG00000132780	-0,384793537	4,44E-08	0,00000024
NCKIPSD	protein_coding	ENSG00000213672	-0,352916517	4,47E-08	0,000000242

ARHGEF4	protein_coding	ENSG00000136002	-0,545256835	4,48E-08	0,000000242
MYB	protein_coding	ENSG00000118513	-1,156928111	4,47E-08	0,000000242
ZNF23	protein_coding	ENSG00000167377	0,844123339	4,52E-08	0,000000245
TAF5	protein_coding	ENSG00000148835	-0,319570108	4,56E-08	0,000000246
USP42	protein_coding	ENSG00000106346	-0,402121056	4,57E-08	0,000000247
AC008840.1	antisense	ENSG00000250240	2,094479236	4,61E-08	0,000000249
EDA2R	protein_coding	ENSG00000131080	0,249011803	4,65E-08	0,000000251
PHLPP2	protein_coding	ENSG00000040199	-0,379773098	4,64E-08	0,000000251
KIAA1841	protein_coding	ENSG00000162929	-0,511451565	4,65E-08	0,000000251
HELZ2	protein_coding	ENSG00000130589	0,996015857	4,69E-08	0,000000253
SSR3	protein_coding	ENSG00000114850	-0,31399208	4,68E-08	0,000000253
RNU5E-1	snRNA	ENSG00000199347	-1,17691505	4,69E-08	0,000000253
ARHGAP9	protein_coding	ENSG00000123329	4,494089574	4,81E-08	0,000000259
ZSCAN12P1	transcribed_unprocesse	ENSG00000219891	2,066005923	4,86E-08	0,000000262
CPA4	protein_coding	ENSG00000128510	0,300684101	4,93E-08	0,000000266
ORC6	protein_coding	ENSG00000091651	-0,434285989	4,93E-08	0,000000266
SLC22A18	protein_coding	ENSG00000110628	0,716202049	4,96E-08	0,000000267
AC018781.1	lincRNA	ENSG00000248599	4,068164367	4,97E-08	0,000000268
ABHD4	protein_coding	ENSG00000100439	0,359253289	4,99E-08	0,000000268
RBBP9	protein_coding	ENSG00000089050	-0,343390747	0,00000005	0,000000269
CWC25	protein_coding	ENSG00000273559	-0,274589026	5,02E-08	0,00000027
EID1	protein_coding	ENSG00000255302	-0,387871449	5,06E-08	0,000000272
MAP3K3	protein_coding	ENSG00000198909	-0,353817078	5,07E-08	0,000000273
AC027281.1	lincRNA	ENSG00000262890	2,850730794	5,14E-08	0,000000276
STX1A	protein_coding	ENSG00000106089	0,492808271	5,14E-08	0,000000277
DDX55	protein_coding	ENSG00000111364	-0,27825383	5,16E-08	0,000000277
RNU5E-4P	snRNA	ENSG00000201801	-1,939376032	5,15E-08	0,000000277
GPR108	protein_coding	ENSG00000125734	0,346971436	5,17E-08	0,000000278
SHC2	protein_coding	ENSG00000129946	0,920619179	0,000000052	0,000000279
NBPF12	protein_coding	ENSG00000268043	0,625344714	0,000000052	0,000000279
LRRC49	protein_coding	ENSG00000137821	0,416203387	5,21E-08	0,00000028
WDR4	protein_coding	ENSG00000160193	-0,427558424	5,23E-08	0,00000028
CSE1L	protein_coding	ENSG00000124207	-0,333627079	5,25E-08	0,000000281
PSEN1	protein_coding	ENSG00000080815	0,214108972	5,26E-08	0,000000282
RGS1	protein_coding	ENSG00000090104	6,478626845	5,37E-08	0,000000288
AC021066.1	antisense	ENSG00000257830	2,731955226	5,39E-08	0,000000289
OIP5	protein_coding	ENSG00000104147	-0,390453276	5,39E-08	0,000000289
TMEM41A	protein_coding	ENSG00000163900	-0,29640595	5,41E-08	0,00000029
ASAP2	protein_coding	ENSG00000151693	-0,291109947	5,47E-08	0,000000293
XPO5	protein_coding	ENSG00000124571	-0,243604745	5,49E-08	0,000000294

AC007728.2	antisense	ENSG00000261644	3,090628779	0,000000055	0,000000295
PCMTD2	protein_coding	ENSG00000203880	-0,352318598	0,000000055	0,000000295
HTATSF1P2	processed_pseudogene	ENSG00000271361	0,352614039	5,54E-08	0,000000296
MFSD14B	protein_coding	ENSG00000148110	-0,278679378	5,54E-08	0,000000296
MYO1A	protein_coding	ENSG00000166866	2,018583322	5,56E-08	0,000000297
FAM111A	protein_coding	ENSG00000166801	-0,2996955	5,55E-08	0,000000297
FAM86HP	transcribed_unprocesse	ENSG00000253540	1,073620436	5,55E-08	0,000000297
DUXAP8	processed_transcript	ENSG00000206195	0,311862122	5,58E-08	0,000000298
SPPL3	protein_coding	ENSG00000157837	0,335295152	5,59E-08	0,000000299
SNORD14A	snoRNA	ENSG00000272034	-1,516151529	0,000000056	0,000000299
NEO1	protein_coding	ENSG00000067141	0,968639998	5,65E-08	0,000000301
GPR155	protein_coding	ENSG00000163328	0,727067026	5,63E-08	0,000000301
AC004835.1	lincRNA	ENSG00000230392	4,477799678	5,65E-08	0,000000302
CYREN	protein_coding	ENSG00000122783	-0,280530512	5,69E-08	0,000000304
ZDHHC12	protein_coding	ENSG00000160446	-0,429005691	0,000000057	0,000000304
SLC3A2	protein_coding	ENSG00000168003	-0,269442377	5,72E-08	0,000000305
AL360270.2	sense_overlapping	ENSG00000261654	1,523897744	5,72E-08	0,000000305
AL024508.1	antisense	ENSG00000234263	4,040934661	5,77E-08	0,000000307
VRK1	protein_coding	ENSG00000100749	-0,401451628	5,79E-08	0,000000309
SBDSP1	transcribed_unprocesse	ENSG00000225648	0,352048959	5,81E-08	0,00000031
RFPL1	protein_coding	ENSG00000128250	6,431522867	5,87E-08	0,000000312
ATL2	protein_coding	ENSG00000119787	-0,360851558	5,95E-08	0,000000317
ATG10	protein_coding	ENSG00000152348	-0,416481241	5,98E-08	0,000000318
DGKA	protein_coding	ENSG00000065357	0,315661432	5,99E-08	0,000000319
CDC42BPA	protein_coding	ENSG00000143776	0,280719035	6,01E-08	0,00000032
MED31	protein_coding	ENSG00000108590	-0,498802719	6,04E-08	0,000000321
CAMK2B	protein_coding	ENSG00000058404	4,945036371	6,08E-08	0,000000323
GCLC	protein_coding	ENSG00000001084	0,321639297	6,07E-08	0,000000323
DLL3	protein_coding	ENSG00000090932	-0,679714156	6,07E-08	0,000000323
C12orf57	protein_coding	ENSG00000111678	0,514868386	6,11E-08	0,000000324
CGB7	protein_coding	ENSG00000196337	4,965016581	6,12E-08	0,000000325
TNFRSF8	protein_coding	ENSG00000120949	2,83342527	6,12E-08	0,000000325
PCDHB5	protein_coding	ENSG00000113209	-0,595864578	6,15E-08	0,000000327
TOX	protein_coding	ENSG00000198846	3,641083463	0,000000062	0,000000329
MAP3K2	protein_coding	ENSG00000169967	0,285907944	6,26E-08	0,000000332
AL157896.1	antisense	ENSG00000235410	1,034261294	6,34E-08	0,000000336
PTGES2	protein_coding	ENSG00000148334	-0,31160232	6,35E-08	0,000000337
HLTF	protein_coding	ENSG00000071794	-0,320381359	6,36E-08	0,000000337
AC008736.2	lincRNA	ENSG00000267475	1,12550892	6,39E-08	0,000000339
MYL5	protein_coding	ENSG00000215375	0,630291262	6,39E-08	0,000000339

PAFAH1B1	protein_coding	ENSG00000007168	-0,312588432	6,39E-08	0,000000339
GSEC	antisense	ENSG00000280832	0,762728168	6,42E-08	0,00000034
SF3A2	protein_coding	ENSG00000104897	-0,356451804	6,42E-08	0,00000034
IL20RB	protein_coding	ENSG00000174564	0,983107843	6,44E-08	0,000000341
FGF21	protein_coding	ENSG00000105550	6,409103453	6,49E-08	0,000000343
CCDC43	protein_coding	ENSG00000180329	0,238396901	6,48E-08	0,000000343
CTSC	protein_coding	ENSG00000109861	-0,305624785	6,49E-08	0,000000343
EMC7	protein_coding	ENSG00000134153	0,319873615	0,000000065	0,000000344
AC036222.1	lincRNA	ENSG00000264451	2,688948644	6,56E-08	0,000000347
CFH	protein_coding	ENSG00000000971	0,708311518	6,57E-08	0,000000347
PKD1L1	protein_coding	ENSG00000158683	1,27795578	6,58E-08	0,000000348
LRP5	protein_coding	ENSG00000162337	-0,515552111	6,58E-08	0,000000348
MUC22	protein_coding	ENSG00000261272	4,562719252	6,64E-08	0,00000035
MOB1A	protein_coding	ENSG00000114978	0,245430408	6,65E-08	0,000000351
HMGB1	protein_coding	ENSG00000189403	-0,331689269	6,65E-08	0,000000351
LRP5L	protein_coding	ENSG00000100068	0,911144345	0,000000067	0,000000353
ZFP1	protein_coding	ENSG00000184517	-0,373288254	6,68E-08	0,000000353
AC007406.3	lincRNA	ENSG00000256540	6,412299691	6,72E-08	0,000000355
UNC119	protein_coding	ENSG00000109103	-0,359086504	6,74E-08	0,000000355
AL392023.2	antisense	ENSG00000259048	1,175323313	6,76E-08	0,000000356
BTF3L4	protein_coding	ENSG00000134717	-0,392791638	6,78E-08	0,000000357
HELLS	protein_coding	ENSG00000119969	-0,355425638	6,79E-08	0,000000358
SCARB1	protein_coding	ENSG00000073060	0,391647623	6,86E-08	0,000000361
AC036176.1	antisense	ENSG00000267390	2,763855208	6,96E-08	0,000000366
HCG18	antisense	ENSG00000231074	0,225177659	6,96E-08	0,000000366
AC006262.1	processed_transcript	ENSG00000268460	4,903008632	0,00000007	0,000000368
DBT	protein_coding	ENSG00000137992	0,292317844	0,00000007	0,000000368
IGF2BP1	protein_coding	ENSG00000159217	-0,272737223	6,99E-08	0,000000368
KIAA0586	protein_coding	ENSG00000100578	-0,294364791	6,99E-08	0,000000368
CEACAM22P	transcribed_unprocesse	ENSG00000230666	4,035043893	6,98E-08	0,000000368
SCARNA22	scaRNA	ENSG00000249784	-0,854842713	7,04E-08	0,00000037
MTMR7	protein_coding	ENSG00000003987	1,955010462	7,07E-08	0,000000371
SOCS7	protein_coding	ENSG00000274211	-0,491214994	7,12E-08	0,000000374
E2F1	protein_coding	ENSG00000101412	-0,335152882	7,14E-08	0,000000375
MARS2	protein_coding	ENSG00000247626	-0,421781221	7,13E-08	0,000000375
PRKCA	protein_coding	ENSG00000154229	0,394160608	7,19E-08	0,000000377
MYL12A	protein_coding	ENSG00000101608	0,355824907	7,22E-08	0,000000379
ANKUB1	protein_coding	ENSG00000206199	3,726961134	7,31E-08	0,000000384
TRIP4	protein_coding	ENSG00000103671	0,357366155	7,31E-08	0,000000384
NUDT6	protein_coding	ENSG00000170917	-0,619246397	7,31E-08	0,000000384

TIFA	protein_coding	ENSG00000145365	0,419307844	7,38E-08	0,000000387
ZWILCH	protein_coding	ENSG00000174442	-0,321108787	7,39E-08	0,000000387
KCNC4	protein_coding	ENSG00000116396	-0,568067531	0,000000074	0,000000388
AC012435.1	transcribed_processed_	ENSG00000260103	2,836962596	7,47E-08	0,000000391
PSME4	protein_coding	ENSG00000068878	0,342354291	7,64E-08	0,0000004
AL139393.2	antisense	ENSG00000272841	1,529807501	7,66E-08	0,000000401
XIRP1	protein_coding	ENSG00000168334	1,911971056	0,000000077	0,000000403
ELOVL6	protein_coding	ENSG00000170522	-0,50174863	7,77E-08	0,000000407
AC079313.2	antisense	ENSG00000258137	4,529442509	7,82E-08	0,000000409
AC011477.6	processed_pseudogene	ENSG00000271499	6,424378834	7,86E-08	0,000000411
ASTN2	protein_coding	ENSG00000148219	0,412313131	7,97E-08	0,000000417
TAPT1	protein_coding	ENSG00000169762	0,412536594	0,00000008	0,000000418
MRPS7	protein_coding	ENSG00000125445	-0,318807194	8,06E-08	0,000000422
BMP8B	protein_coding	ENSG00000116985	-1,060738018	0,000000081	0,000000423
PPP1R12C	protein_coding	ENSG00000125503	0,492189636	8,11E-08	0,000000424
CDC25C	protein_coding	ENSG00000158402	-0,34803709	8,25E-08	0,000000431
CDC37	protein_coding	ENSG00000105401	0,223875814	8,27E-08	0,000000432
SNORD83A	snoRNA	ENSG00000209482	-1,238727079	8,28E-08	0,000000432
ALDH1A2	protein_coding	ENSG00000128918	2,221275258	0,000000083	0,000000433
GTPBP6	protein_coding	ENSG00000178605	-0,374373969	8,37E-08	0,000000436
CCDC71	protein_coding	ENSG00000177352	-0,381604548	8,36E-08	0,000000436
SPTLC1	protein_coding	ENSG00000090054	0,27461763	8,38E-08	0,000000437
ZNF132	protein_coding	ENSG00000131849	0,596434253	8,42E-08	0,000000439
MRM2	protein_coding	ENSG00000122687	-0,301540403	8,42E-08	0,000000439
MIP	protein_coding	ENSG00000135517	4,525895123	8,52E-08	0,000000444
B4GALT4	protein_coding	ENSG00000121578	-0,197519424	8,55E-08	0,000000445
ELL	protein_coding	ENSG00000105656	0,482815505	8,59E-08	0,000000447
ARL8B	protein_coding	ENSG00000134108	0,278486069	8,63E-08	0,000000449
RGS9BP	protein_coding	ENSG00000186326	-0,879444288	8,62E-08	0,000000449
EMC9	protein_coding	ENSG00000100908	-0,354712262	8,66E-08	0,000000451
ZDHHC20	protein_coding	ENSG00000180776	0,272981664	8,68E-08	0,000000452
AL391422.4	antisense	ENSG00000270504	0,460345028	8,72E-08	0,000000453
FBXO31	protein_coding	ENSG00000103264	-0,357883131	8,74E-08	0,000000455
ZNF512	protein_coding	ENSG00000243943	-0,355962397	8,76E-08	0,000000456
IKZF3	protein_coding	ENSG00000161405	6,419482378	0,000000088	0,000000458
UPP1	protein_coding	ENSG00000183696	0,306280639	8,81E-08	0,000000458
TMEM131	protein_coding	ENSG00000075568	0,377240324	8,91E-08	0,000000463
ATP5MC1	protein_coding	ENSG00000159199	-0,369896861	8,96E-08	0,000000465
GPR161	protein_coding	ENSG00000143147	-0,399718525	8,95E-08	0,000000465
COX20	protein_coding	ENSG00000203667	-0,410784245	0,00000009	0,000000467

DSTN	protein_coding	ENSG00000125868	0,292793177	9,02E-08	0,000000468
VMP1	protein_coding	ENSG00000062716	-0,311855974	9,11E-08	0,000000473
SLC25A10	protein_coding	ENSG00000183048	-0,592965705	9,12E-08	0,000000473
RARRES3	protein_coding	ENSG00000133321	1,750310224	9,19E-08	0,000000477
PIK3C3	protein_coding	ENSG00000078142	0,281526106	9,21E-08	0,000000477
MIR34AHG	lincRNA	ENSG00000228526	0,821166739	9,23E-08	0,000000478
TTLL11	protein_coding	ENSG00000175764	0,647184	9,23E-08	0,000000478
TBL1X	protein_coding	ENSG00000101849	-0,355447307	9,23E-08	0,000000478
ATPSCKMT	protein_coding	ENSG00000150756	-0,417250262	9,23E-08	0,000000478
SBDS	protein_coding	ENSG00000126524	0,282818204	9,27E-08	0,00000048
KANSL1	protein_coding	ENSG00000120071	0,28027739	9,27E-08	0,00000048
TCF3	protein_coding	ENSG00000071564	-0,330863826	9,33E-08	0,000000483
COQ2	protein_coding	ENSG00000173085	-0,434917285	9,34E-08	0,000000483
MUC4	protein_coding	ENSG00000145113	0,986293196	9,44E-08	0,000000488
PPP4R2	protein_coding	ENSG00000163605	-0,37459917	9,47E-08	0,00000049
TIMM10	protein_coding	ENSG00000134809	-0,392170725	9,53E-08	0,000000493
TMEM267	protein_coding	ENSG00000151881	-0,468475268	9,57E-08	0,000000495
DNMBP	protein_coding	ENSG00000107554	0,315147735	0,000000096	0,000000496
TRAF2	protein_coding	ENSG00000127191	-0,357832129	9,63E-08	0,000000497
AC099062.1	lincRNA	ENSG00000284240	2,736038002	9,64E-08	0,000000498
RARA	protein_coding	ENSG00000131759	0,581572609	0,000000097	0,000000501
ADCY10	protein_coding	ENSG00000143199	2,295541329	0,000000099	0,000000511
MRPS25	protein_coding	ENSG00000131368	-0,293334793	9,89E-08	0,000000511
TMPRSS9	protein_coding	ENSG00000178297	4,897917938	9,94E-08	0,000000513
ZFAS1	antisense	ENSG00000177410	0,351438069	9,96E-08	0,000000514
CYTOR	lincRNA	ENSG00000222041	-0,345162169	9,96E-08	0,000000514
PRAME	protein_coding	ENSG00000185686	-0,20616106	0,0000001	0,000000518
ABCF2	protein_coding	ENSG00000033050	-0,232571387	0,000000101	0,000000518
DHX36	protein_coding	ENSG00000174953	0,239891002	0,000000101	0,000000519
AC104170.1	antisense	ENSG00000227070	1,394594854	0,000000102	0,000000523
SOCS3	protein_coding	ENSG00000184557	0,401644615	0,000000102	0,000000524
PAFAH1B3	protein_coding	ENSG00000079462	-0,361128076	0,000000102	0,000000524
SH2D2A	protein_coding	ENSG00000027869	2,018517425	0,000000102	0,000000525
TTC30A	protein_coding	ENSG00000197557	-0,425817738	0,000000102	0,000000528
PTK6	protein_coding	ENSG00000101213	0,712289352	0,000000103	0,000000531
RELL2	protein_coding	ENSG00000164620	0,70812606	0,000000104	0,000000534
TRA2B	protein_coding	ENSG00000136527	-0,256729138	0,000000104	0,000000536
CDA	protein_coding	ENSG00000158825	0,622107954	0,000000104	0,000000537
LNCTAM34A	lincRNA	ENSG00000234546	1,118833038	0,000000105	0,000000542
PGAP1	protein_coding	ENSG00000197121	0,464621769	0,000000105	0,000000542

MIR100HG	processed_transcript	ENSG00000255248	0,400174267	0,000000106	0,000000545
ARHGEF28	protein_coding	ENSG00000214944	-0,194440463	0,000000106	0,000000545
ZCCHC9	protein_coding	ENSG00000131732	-0,373547461	0,000000106	0,000000545
POLR2J4	processed_transcript	ENSG00000214783	1,574391161	0,000000106	0,000000546
MED21	protein_coding	ENSG00000152944	-0,389961537	0,000000106	0,000000546
AC010319.3	antisense	ENSG00000269053	3,401025369	0,000000108	0,000000553
AL138781.1	lincRNA	ENSG00000260193	1,42788007	0,000000109	0,000000559
REM2	protein_coding	ENSG00000139890	3,165071319	0,000000109	0,00000056
CARS2	protein_coding	ENSG00000134905	-0,212160789	0,000000109	0,000000561
PSMD7	protein_coding	ENSG00000103035	-0,275042884	0,00000011	0,000000562
TMEM107	protein_coding	ENSG00000179029	-0,348900763	0,00000011	0,000000562
GALNT18	protein_coding	ENSG00000110328	-0,438907495	0,00000011	0,000000562
C16orf70	protein_coding	ENSG00000125149	-0,381313522	0,00000011	0,000000564
ANKRD55	protein_coding	ENSG00000164512	0,390530946	0,000000111	0,000000568
COMMD4	protein_coding	ENSG00000140365	-0,347277258	0,000000111	0,000000568
FAN1	protein_coding	ENSG00000198690	-0,260119697	0,000000111	0,00000057
KCTD14	protein_coding	ENSG00000151364	-0,482830976	0,000000112	0,000000572
AC095055.1	antisense	ENSG00000270681	1,66263755	0,000000113	0,000000578
AC112777.1	processed_pseudogene	ENSG00000256663	-0,627373638	0,000000113	0,000000579
TBC1D23	protein_coding	ENSG00000036054	0,350707681	0,000000113	0,000000579
VWA1	protein_coding	ENSG00000179403	-1,453036148	0,000000113	0,00000058
MOB1B	protein_coding	ENSG00000173542	0,339621643	0,000000114	0,000000582
HOXB5	protein_coding	ENSG00000120075	-0,899473539	0,000000114	0,000000584
KCNQ1OT1	antisense	ENSG00000269821	0,574467439	0,000000115	0,000000586
RANP4	processed_pseudogene	ENSG00000225125	3,601203626	0,000000115	0,000000587
SNORD3A	snoRNA	ENSG00000263934	-1,058704538	0,000000115	0,000000587
ARMC5	protein_coding	ENSG00000140691	0,644326203	0,000000115	0,000000588
TEC	protein_coding	ENSG00000135605	0,539399094	0,000000115	0,000000588
ARL4D	protein_coding	ENSG00000175906	-0,474569538	0,000000115	0,000000588
AL109741.1	antisense	ENSG00000225938	0,994444804	0,000000116	0,000000589
SNORA13	snoRNA	ENSG00000238363	-1,429555038	0,000000115	0,000000589
RPP14	protein_coding	ENSG00000163684	0,333927237	0,000000118	0,000000604
SLC22A1	protein_coding	ENSG00000175003	2,663632377	0,000000119	0,000000606
TINCR	protein_coding	ENSG00000223573	1,38213516	0,000000119	0,000000606
MMP16	protein_coding	ENSG00000156103	0,35539368	0,00000012	0,00000061
CCDC137	protein_coding	ENSG00000185298	-0,291711663	0,00000012	0,00000061
HEXIM1	protein_coding	ENSG00000186834	0,41581617	0,00000012	0,000000611
SLC9A9	protein_coding	ENSG00000181804	1,081829752	0,00000012	0,000000613
LRRN3	protein_coding	ENSG00000173114	6,217281695	0,000000121	0,000000618
APTR	lincRNA	ENSG00000214293	0,784900712	0,000000122	0,000000622

UTP20	protein_coding	ENSG00000120800	-0,323079702	0,000000123	0,000000626
ADGRE1	protein_coding	ENSG00000174837	0,351896873	0,000000123	0,000000627
ADRB1	protein_coding	ENSG00000043591	-0,698038458	0,000000124	0,000000629
ALDH5A1	protein_coding	ENSG00000112294	-0,446002969	0,000000124	0,00000063
KCNQ1	protein_coding	ENSG00000053918	6,282057842	0,000000124	0,000000631
CPS1	protein_coding	ENSG00000021826	0,363030048	0,000000124	0,000000632
BMT2	protein_coding	ENSG00000164603	0,424404513	0,000000125	0,000000633
HSD3B7	protein_coding	ENSG00000099377	-0,700813412	0,000000125	0,000000636
MCIDAS	protein_coding	ENSG00000234602	-0,78183815	0,000000126	0,000000639
KLHL11	protein_coding	ENSG00000178502	-0,312586118	0,000000126	0,000000641
AC026202.2	antisense	ENSG00000233912	3,592089644	0,000000127	0,000000643
UQCR11	protein_coding	ENSG00000127540	-0,438650028	0,000000127	0,000000643
SEC14L1	protein_coding	ENSG00000129657	0,281766699	0,000000128	0,000000647
CC2D1B	protein_coding	ENSG00000154222	-0,264418901	0,000000128	0,000000647
ELK4	protein_coding	ENSG00000158711	-0,378493354	0,000000128	0,000000649
GNL2	protein_coding	ENSG00000134697	-0,247012018	0,000000128	0,00000065
CLPB	protein_coding	ENSG00000162129	-0,292886302	0,000000128	0,00000065
ADGRE5	protein_coding	ENSG00000123146	0,364176893	0,000000128	0,000000651
NUDT12	protein_coding	ENSG00000112874	-0,652086389	0,000000128	0,000000651
DPF3	protein_coding	ENSG00000205683	0,492567662	0,000000129	0,000000653
COBLL1	protein_coding	ENSG00000082438	0,343620566	0,00000013	0,000000658
INPP5J	protein_coding	ENSG00000185133	1,976429718	0,00000013	0,00000066
KRTAP5-AS1	antisense	ENSG00000233930	6,347192486	0,000000131	0,000000661
ZNF484	protein_coding	ENSG00000127081	0,475604622	0,000000131	0,000000662
DRP2	protein_coding	ENSG00000102385	0,98702375	0,000000131	0,000000663
ESCO1	protein_coding	ENSG00000141446	0,287768531	0,000000131	0,000000665
INHBE	protein_coding	ENSG00000139269	3,947662514	0,000000132	0,000000667
TLCD1	protein_coding	ENSG00000160606	-0,443330691	0,000000132	0,000000669
SMARCAD1	protein_coding	ENSG00000163104	-0,334064575	0,000000133	0,00000067
LDHA	protein_coding	ENSG00000134333	-0,366141255	0,000000133	0,000000672
TACC1	protein_coding	ENSG00000147526	0,228651396	0,000000134	0,000000677
AC091212.1	antisense	ENSG00000239462	2,495325818	0,000000135	0,00000068
CNOT6L	protein_coding	ENSG00000138767	-0,383275976	0,000000135	0,00000068
AL356599.1	antisense	ENSG00000235652	1,635321224	0,000000137	0,00000069
PRIM2	protein_coding	ENSG00000146143	-0,387909849	0,000000137	0,000000693
MYOM1	protein_coding	ENSG00000101605	2,741612941	0,000000138	0,000000697
CCL2	protein_coding	ENSG00000108691	1,359353409	0,000000138	0,000000697
APPL1	protein_coding	ENSG00000157500	-0,303181731	0,000000138	0,000000697
MRPL15	protein_coding	ENSG00000137547	-0,370134829	0,000000139	0,000000699
SLC7A2	protein_coding	ENSG00000003989	0,340234873	0,000000139	0,000000702

MPI	protein_coding	ENSG00000178802	-0,295437411	0,000000141	0,000000708
MTMR9	protein_coding	ENSG00000104643	0,284046513	0,000000141	0,000000712
FASTKD1	protein_coding	ENSG00000138399	-0,388050954	0,000000143	0,000000723
HPS3	protein_coding	ENSG00000163755	0,253060942	0,000000144	0,000000727
ALG10	protein_coding	ENSG00000139133	-0,459968576	0,000000145	0,000000728
MIB1	protein_coding	ENSG00000101752	-0,3404479	0,000000145	0,000000731
ZNF551	protein_coding	ENSG00000204519	-0,461227381	0,000000146	0,000000736
MAF1	protein_coding	ENSG00000179632	-0,270062083	0,000000148	0,000000743
HPCAL1	protein_coding	ENSG00000115756	-0,264887737	0,000000148	0,000000744
MANBAL	protein_coding	ENSG00000101363	-0,314163343	0,000000149	0,000000749
SNORD116-19	snoRNA	ENSG00000207460	2,205322847	0,000000149	0,00000075
TRIM23	protein_coding	ENSG00000113595	0,332631617	0,00000015	0,000000754
SOX13	protein_coding	ENSG00000143842	0,435442234	0,00000015	0,000000755
SPATS2L	protein_coding	ENSG00000196141	0,206072833	0,00000015	0,000000755
NMRK1	protein_coding	ENSG00000106733	0,714572946	0,000000151	0,000000757
AC099489.1	protein_coding	ENSG00000188897	1,855819677	0,000000152	0,000000763
ANAPC11	protein_coding	ENSG00000141552	-0,363412817	0,000000152	0,000000765
CSGALNACT1	protein_coding	ENSG00000147408	4,784816664	0,000000153	0,000000766
CTXN1	protein_coding	ENSG00000178531	-0,641945528	0,000000153	0,000000766
PRPF4B	protein_coding	ENSG00000112739	-0,41982071	0,000000153	0,000000767
TMEM269	protein_coding	ENSG00000274386	1,890357426	0,000000153	0,000000768
C2orf78	protein_coding	ENSG00000187833	6,381400221	0,000000154	0,00000077
AC092327.2	lincRNA	ENSG00000269826	4,46179794	0,000000154	0,000000771
KDM2B	protein_coding	ENSG00000089094	-0,357776845	0,000000154	0,000000772
VDR	protein_coding	ENSG00000111424	0,38992478	0,000000154	0,000000773
MAPK13	protein_coding	ENSG00000156711	0,359929212	0,000000155	0,000000777
SMARCE1	protein_coding	ENSG00000073584	-0,322473225	0,000000155	0,000000777
AC005840.4	antisense	ENSG00000276718	4,800427188	0,000000157	0,000000783
FAM71A	protein_coding	ENSG00000162771	4,471206159	0,000000157	0,000000784
TMBIM4	protein_coding	ENSG00000155957	-0,721424569	0,000000157	0,000000786
SMG1P2	unprocessed_pseudoge	ENSG00000205534	1,072250702	0,000000158	0,000000791
AP000662.1	sense_overlapping	ENSG00000254602	0,910081998	0,000000158	0,000000792
CARD8	protein_coding	ENSG00000105483	-0,365967891	0,000000159	0,000000794
NCS1	protein_coding	ENSG00000107130	-0,276451245	0,00000016	0,000000802
GSTO2	protein_coding	ENSG00000065621	1,080926993	0,000000162	0,000000811
AL021707.6	antisense	ENSG00000272669	2,110035924	0,000000163	0,000000813
ZSCAN4	protein_coding	ENSG00000180532	4,837894598	0,000000163	0,000000816
IBTK	protein_coding	ENSG00000005700	0,248807135	0,000000164	0,000000817
PSMG2	protein_coding	ENSG00000128789	-0,397993284	0,000000164	0,000000818
AL049629.1	antisense	ENSG00000255202	2,413202721	0,000000165	0,000000825

ARID3B	protein_coding	ENSG00000179361	0,587530066	0,000000165	0,000000825
PRODH	protein_coding	ENSG00000100033	1,420540318	0,000000166	0,000000828
ROCK1	protein_coding	ENSG00000067900	0,308661508	0,000000169	0,000000842
CTU1	protein_coding	ENSG00000142544	-0,671285535	0,00000017	0,000000848
FBLIM1	protein_coding	ENSG00000162458	0,494499575	0,000000171	0,000000852
SLC35A4	protein_coding	ENSG00000176087	-0,336035948	0,000000171	0,000000852
PALLD	protein_coding	ENSG00000129116	0,234950834	0,000000173	0,000000859
DSP	protein_coding	ENSG00000096696	-0,475063376	0,000000172	0,000000859
POU2F3	protein_coding	ENSG00000137709	-1,57085853	0,000000173	0,000000862
ZNF117	protein_coding	ENSG00000152926	0,895797794	0,000000173	0,000000864
ZNF117	protein_coding	ENSG00000152926	0,895797794	0,000000173	0,000000864
RAPGEF2	protein_coding	ENSG00000109756	0,352370339	0,000000174	0,000000865
LINC00303	lincRNA	ENSG00000176754	6,206733548	0,000000174	0,000000868
POLR2H	protein_coding	ENSG00000163882	-0,298375577	0,000000174	0,000000868
TTC33	protein_coding	ENSG00000113638	-0,535963412	0,000000175	0,000000869
CLDN6	protein_coding	ENSG00000184697	2,311493111	0,000000178	0,000000883
CXCR4	protein_coding	ENSG00000121966	-0,82457665	0,000000178	0,000000883
ZNF718	protein_coding	ENSG00000250312	-0,537654339	0,000000178	0,000000886
COLEC11	protein_coding	ENSG00000118004	6,22508829	0,000000179	0,00000089
IL34	protein_coding	ENSG00000157368	1,443652499	0,000000179	0,000000891
ZHX3	protein_coding	ENSG00000174306	-0,398741826	0,000000179	0,000000891
PARPBP	protein_coding	ENSG00000185480	-0,426333098	0,00000018	0,000000893
SNX16	protein_coding	ENSG00000104497	0,438479421	0,000000182	0,000000903
PGP	protein_coding	ENSG00000184207	-0,383804116	0,000000182	0,000000903
ATXN7L1	protein_coding	ENSG00000146776	0,65758589	0,000000182	0,000000904
PROSER3	protein_coding	ENSG00000167595	-0,650654193	0,000000183	0,000000907
TRIM59	protein_coding	ENSG00000213186	-0,409452471	0,000000184	0,00000091
C12orf66	protein_coding	ENSG00000174206	0,40634977	0,000000186	0,000000923
DCUN1D3	protein_coding	ENSG00000188215	0,28811943	0,000000186	0,000000923
GPX8	protein_coding	ENSG00000164294	-0,333565914	0,000000186	0,000000923
RHBDF1	protein_coding	ENSG00000007384	0,610252207	0,000000188	0,00000093
AC015849.1	antisense	ENSG00000270240	6,247762105	0,000000188	0,000000932
BRF1	protein_coding	ENSG00000185024	-0,411027136	0,000000191	0,000000945
PNMA8A	protein_coding	ENSG00000182013	-0,274975054	0,000000191	0,000000947
AC118755.1	antisense	ENSG00000262296	3,862235305	0,000000192	0,000000948
TOR2A	protein_coding	ENSG00000160404	-0,503664155	0,000000192	0,000000949
SMPD1	protein_coding	ENSG00000166311	0,432095772	0,000000193	0,000000954
CFAP53	protein_coding	ENSG00000172361	0,882257892	0,000000193	0,000000956
CRB2	protein_coding	ENSG00000148204	6,135529199	0,000000195	0,000000965
SIRT1	protein_coding	ENSG00000096717	0,295356775	0,000000196	0,000000968

NOP2	protein_coding	ENSG00000111641	-0,285078254	0,000000196	0,00000097
MRTFB	protein_coding	ENSG00000186260	-0,497819624	0,000000197	0,000000975
DEPDC1-AS1	antisense	ENSG00000234264	2,187306241	0,000000198	0,00000098
AC008124.1	lincRNA	ENSG00000273015	-0,552152657	0,000000199	0,000000984
CHST14	protein_coding	ENSG00000169105	-0,308035781	0,000000199	0,000000984
AL049796.1	bidirectional_promoter_	ENSG00000260464	-1,381213933	0,000000201	0,000000991
KCTD16	protein_coding	ENSG00000183775	1,903976284	0,000000201	0,000000993
ZNF385C	protein_coding	ENSG00000187595	4,765886181	0,000000203	0,000001
DIRAS1	protein_coding	ENSG00000176490	-0,772919288	0,000000203	0,000001
THUMPD3	protein_coding	ENSG00000134077	-0,347570593	0,000000205	0,00000101
DDIT4	protein_coding	ENSG00000168209	0,512529801	0,000000207	0,00000102
OSER1	protein_coding	ENSG00000132823	0,382181078	0,000000207	0,00000102
KIF1BP	protein_coding	ENSG00000198954	-0,241696049	0,000000206	0,00000102
WDR18	protein_coding	ENSG00000065268	-0,378860926	0,000000206	0,00000102
IPP	protein_coding	ENSG00000197429	-0,286379545	0,000000208	0,00000103
NUDT1	protein_coding	ENSG00000106268	-0,416067657	0,00000021	0,00000103
UBR4	protein_coding	ENSG00000127481	0,414121622	0,000000211	0,00000104
AC073857.1	TEC	ENSG00000280120	-0,785763423	0,000000211	0,00000104
RAB33A	protein_coding	ENSG00000134594	3,499914668	0,000000214	0,00000105
AHCYL2	protein_coding	ENSG00000158467	0,457543664	0,000000213	0,00000105
USP49	protein_coding	ENSG00000164663	0,451039138	0,000000213	0,00000105
MANBA	protein_coding	ENSG00000109323	0,42296355	0,000000212	0,00000105
TTC26	protein_coding	ENSG00000105948	0,394494294	0,000000212	0,00000105
TBC1D9	protein_coding	ENSG00000109436	0,391954914	0,000000213	0,00000105
TPM1	protein_coding	ENSG00000140416	-0,260530074	0,000000215	0,00000106
BRWD1	protein_coding	ENSG00000185658	-0,33297945	0,000000216	0,00000106
UBE2O	protein_coding	ENSG00000175931	-0,429023244	0,000000216	0,00000106
CEP55	protein_coding	ENSG00000138180	-0,299788044	0,000000217	0,00000107
MCRIP2	protein_coding	ENSG00000172366	-0,601944665	0,000000223	0,00000109
AC097059.2	lincRNA	ENSG00000236098	0,529779167	0,000000224	0,0000011
COA7	protein_coding	ENSG00000162377	-0,279872317	0,000000225	0,0000011
BRD3OS	protein_coding	ENSG00000235106	-0,36680911	0,000000224	0,0000011
SEC14L2	protein_coding	ENSG00000100003	0,464146655	0,000000227	0,00000111
CENPV	protein_coding	ENSG00000166582	-0,33767236	0,000000229	0,00000112
PHLPP1	protein_coding	ENSG00000081913	-0,409688998	0,000000232	0,00000114
NEURL1	protein_coding	ENSG00000107954	1,845619935	0,000000234	0,00000115
CDK2AP2	protein_coding	ENSG00000167797	0,342928612	0,000000234	0,00000115
ATP5MC2	protein_coding	ENSG00000135390	-0,340001705	0,000000235	0,00000115
VLDLR	protein_coding	ENSG00000147852	-0,47718727	0,000000236	0,00000116
RANGRF	protein_coding	ENSG00000108961	-0,383260243	0,000000239	0,00000117

PCLO	protein_coding	ENSG00000186472	0,481704402	0,00000024	0,00000118
PPP2R5B	protein_coding	ENSG00000068971	0,439748425	0,000000241	0,00000118
PSMD2	protein_coding	ENSG00000175166	0,191991205	0,000000241	0,00000118
MYBL2	protein_coding	ENSG00000101057	-0,295450759	0,00000024	0,00000118
ZNF232	protein_coding	ENSG00000167840	-0,366341807	0,000000244	0,00000119
PGLYRP2	protein_coding	ENSG00000161031	6,193766154	0,000000248	0,00000121
ADGRV1	protein_coding	ENSG00000164199	1,768990378	0,00000025	0,00000122
CD70	protein_coding	ENSG00000125726	0,464258348	0,00000025	0,00000122
AC004590.1	antisense	ENSG00000251239	1,716972848	0,000000251	0,00000123
AC010173.1	antisense	ENSG00000258101	1,269400998	0,000000251	0,00000123
AACS	protein_coding	ENSG00000081760	0,320777636	0,000000251	0,00000123
SSNA1	protein_coding	ENSG00000176101	-0,405644339	0,000000252	0,00000123
ZCCHC3	protein_coding	ENSG00000247315	-0,425848842	0,000000251	0,00000123
SLC39A3	protein_coding	ENSG00000141873	-0,398714703	0,000000253	0,00000124
AC009237.3	transcribed_unprocesse	ENSG00000229689	0,430297843	0,000000255	0,00000124
VTI1A	protein_coding	ENSG00000151532	0,281312693	0,000000256	0,00000125
CHMP1A	protein_coding	ENSG00000131165	-0,324593494	0,000000261	0,00000127
CDC123	protein_coding	ENSG00000151465	-0,342116409	0,000000266	0,0000013
SLC35F6	protein_coding	ENSG00000213699	0,3509058	0,000000268	0,00000131
SESTD1	protein_coding	ENSG00000187231	-0,407868413	0,00000027	0,00000131
SCNN1D	protein_coding	ENSG00000162572	0,939197104	0,000000271	0,00000132
SHROOM3	protein_coding	ENSG00000138771	0,374472218	0,000000271	0,00000132
AC011611.3	antisense	ENSG00000257453	2,242092225	0,000000273	0,00000133
AC011611.4	antisense	ENSG00000257839	1,414298107	0,000000274	0,00000133
AP001831.1	antisense	ENSG00000254733	6,106822122	0,000000275	0,00000134
AC004825.2	lincRNA	ENSG00000274818	1,342049402	0,000000275	0,00000134
C20orf27	protein_coding	ENSG00000101220	-0,371624772	0,000000278	0,00000135
DENND1B	protein_coding	ENSG00000213047	0,286679614	0,00000028	0,00000136
AL138756.1	sense_overlapping	ENSG00000259953	1,824840888	0,00000028	0,00000136
ZNF883	transcribed_unprocesse	ENSG00000228623	-0,499218689	0,00000028	0,00000136
DCUN1D4	protein_coding	ENSG00000109184	-0,424066098	0,000000284	0,00000138
DNAJB13	protein_coding	ENSG00000187726	1,532081232	0,000000287	0,00000139
AC087783.2	processed_pseudogene	ENSG00000271369	6,132241169	0,000000288	0,0000014
WEE1	protein_coding	ENSG00000166483	-0,329958613	0,000000287	0,0000014
KLHL36	protein_coding	ENSG00000135686	-0,416643047	0,000000289	0,0000014
AL022313.2	lincRNA	ENSG00000228719	4,715906594	0,000000292	0,00000142
UBQLN2	protein_coding	ENSG00000188021	-0,286875846	0,000000292	0,00000142
CAPN11	protein_coding	ENSG00000137225	2,207114287	0,000000295	0,00000143
MAL2	protein_coding	ENSG00000147676	2,128581961	0,000000295	0,00000143
OSGIN2	protein_coding	ENSG00000164823	0,350573546	0,000000296	0,00000144

DAPK3	protein_coding	ENSG00000167657	0,326922631	0,000000297	0,00000144
SGO1	protein_coding	ENSG00000129810	-0,378624244	0,000000296	0,00000144
SAMD4A	protein_coding	ENSG00000020577	0,349761752	0,000000299	0,00000145
PCED1B	protein_coding	ENSG00000179715	1,139250986	0,000000302	0,00000146
CEP78	protein_coding	ENSG00000148019	-0,299668139	0,000000301	0,00000146
SPATA1	protein_coding	ENSG00000122432	1,568575871	0,000000305	0,00000148
AC008074.2	lincRNA	ENSG00000260101	1,640642861	0,000000308	0,00000149
TNFSF15	protein_coding	ENSG00000181634	2,891033114	0,000000307	0,00000149
SHC1	protein_coding	ENSG00000160691	0,303356105	0,000000308	0,00000149
STK24	protein_coding	ENSG00000102572	0,189703927	0,000000308	0,00000149
CASS4	protein_coding	ENSG00000087589	1,815748002	0,000000313	0,00000151
CDADC1	protein_coding	ENSG00000102543	0,43224763	0,000000313	0,00000151
TUBE1	protein_coding	ENSG00000074935	0,378960444	0,000000312	0,00000151
SLC5A6	protein_coding	ENSG00000138074	-0,268502037	0,000000314	0,00000152
PCAT1	antisense	ENSG00000253438	2,222805301	0,000000319	0,00000154
GLRB	protein_coding	ENSG00000109738	0,403678409	0,00000032	0,00000155
CSK	protein_coding	ENSG00000103653	-0,324451997	0,00000032	0,00000155
FCHO2	protein_coding	ENSG00000157107	0,345628555	0,000000324	0,00000156
NIPBL	protein_coding	ENSG00000164190	0,287628144	0,000000324	0,00000156
ZEB1	protein_coding	ENSG00000148516	0,285284099	0,000000324	0,00000156
RNF144A	protein_coding	ENSG00000151692	-0,505340849	0,000000324	0,00000156
SLC6A17	protein_coding	ENSG00000197106	-0,539886483	0,000000323	0,00000156
IKZF5	protein_coding	ENSG00000095574	0,423812985	0,000000325	0,00000157
NSD1	protein_coding	ENSG00000165671	-0,325567564	0,000000329	0,00000159
U62317.1	lincRNA	ENSG00000272666	6,180560079	0,000000332	0,0000016
IL1RAPL1	protein_coding	ENSG00000169306	0,63335928	0,000000335	0,00000161
TRIM37	protein_coding	ENSG00000108395	0,204411055	0,000000334	0,00000161
IFT81	protein_coding	ENSG00000122970	-0,402551825	0,000000335	0,00000161
GTPBP1	protein_coding	ENSG00000100226	0,410471115	0,000000336	0,00000162
KIF21A	protein_coding	ENSG00000139116	0,362153479	0,000000336	0,00000162
CENPW	protein_coding	ENSG00000203760	-0,416200131	0,000000336	0,00000162
ZKSCAN2	protein_coding	ENSG00000155592	0,408743675	0,00000034	0,00000163
EDN1	protein_coding	ENSG00000078401	0,320807596	0,000000338	0,00000163
BANF1	protein_coding	ENSG00000175334	-0,350321425	0,000000339	0,00000163
MGAT4C	protein_coding	ENSG00000182050	4,724325476	0,000000342	0,00000164
TCF20	protein_coding	ENSG00000100207	-0,399334145	0,000000342	0,00000165
HAUS6	protein_coding	ENSG00000147874	-0,361606627	0,000000348	0,00000167
ZNF512B	protein_coding	ENSG00000196700	-0,459864373	0,000000347	0,00000167
EGFL6	protein_coding	ENSG00000198759	4,304471798	0,000000349	0,00000168
PPIF	protein_coding	ENSG00000108179	-0,220480377	0,000000356	0,00000171

NUDC	protein_coding	ENSG00000090273	-0,23111402	0,000000357	0,00000171
SLC5A10	protein_coding	ENSG00000154025	4,218120745	0,00000036	0,00000173
XYLB	protein_coding	ENSG00000093217	-0,501264578	0,00000036	0,00000173
JADE2	protein_coding	ENSG00000043143	-0,368309389	0,000000362	0,00000174
NCKAP5L	protein_coding	ENSG00000167566	0,552479802	0,000000365	0,00000175
FAM91A1	protein_coding	ENSG00000176853	0,244383136	0,000000365	0,00000175
RBFA	protein_coding	ENSG00000101546	-0,44700094	0,000000365	0,00000175
PTAFR	protein_coding	ENSG00000169403	1,333296651	0,000000369	0,00000177
MOAP1	protein_coding	ENSG00000165943	0,386023851	0,000000369	0,00000177
FAM107B	protein_coding	ENSG00000065809	-0,281937099	0,000000369	0,00000177
AL158840.1	antisense	ENSG00000237919	6,04949067	0,000000374	0,00000179
PEX6	protein_coding	ENSG00000124587	0,422130637	0,000000374	0,00000179
AL390195.2	sense_overlapping	ENSG00000260948	1,072631342	0,000000373	0,00000179
FER	protein_coding	ENSG00000151422	0,282603616	0,000000377	0,0000018
UTP14C	protein_coding	ENSG00000253797	0,278359555	0,000000376	0,0000018
NIPSNAP3A	protein_coding	ENSG00000136783	-0,446449439	0,00000038	0,00000182
AC011477.1	transcribed_unprocesse	ENSG00000267419	0,978662283	0,000000381	0,00000182
PIGX	protein_coding	ENSG00000163964	-0,304332482	0,000000382	0,00000183
RABIF	protein_coding	ENSG00000183155	-0,314430802	0,000000382	0,00000183
AC107068.1	lincRNA	ENSG00000259959	-0,760027106	0,000000384	0,00000184
CNP	protein_coding	ENSG00000173786	-0,302970654	0,000000385	0,00000184
HAUS4	protein_coding	ENSG00000092036	-0,354173484	0,000000385	0,00000184
TMEM9	protein_coding	ENSG00000116857	-0,340144319	0,000000389	0,00000186
AC092803.1	lincRNA	ENSG00000224535	4,441305488	0,000000393	0,00000188
PRMT5	protein_coding	ENSG00000100462	-0,246121087	0,000000396	0,00000189
SMYD4	protein_coding	ENSG00000186532	-0,308536351	0,000000396	0,00000189
AL645608.1	antisense	ENSG00000224969	6,113144919	0,000000399	0,0000019
GCNT4	protein_coding	ENSG00000176928	1,328622447	0,000000398	0,0000019
TMEM18	protein_coding	ENSG00000151353	-0,382420823	0,000000398	0,0000019
ZNF566	protein_coding	ENSG00000186017	-0,412397284	0,000000399	0,0000019
SPA17	protein_coding	ENSG00000064199	-0,467380957	0,000000401	0,00000191
CENPT	protein_coding	ENSG00000102901	-0,353434122	0,000000402	0,00000192
PLXNC1	protein_coding	ENSG00000136040	2,461798828	0,000000408	0,00000194
ABHD18	protein_coding	ENSG00000164074	0,410489526	0,000000408	0,00000194
TMED5	protein_coding	ENSG00000117500	0,312769894	0,000000407	0,00000194
UBE2Q1	protein_coding	ENSG00000160714	0,249620915	0,000000407	0,00000194
TMEM106C	protein_coding	ENSG00000134291	-0,305745419	0,000000408	0,00000194
BBS12	protein_coding	ENSG00000181004	0,66282794	0,00000041	0,00000195
RIN3	protein_coding	ENSG00000100599	0,505181449	0,000000409	0,00000195
MRPS2	protein_coding	ENSG00000122140	-0,282342687	0,000000411	0,00000196

RLF	protein_coding	ENSG00000117000	0,250035242	0,000000415	0,00000197
UNC45B	protein_coding	ENSG00000141161	4,288126004	0,000000415	0,00000198
HPX	protein_coding	ENSG00000110169	2,025372823	0,000000417	0,00000198
PLCE1	protein_coding	ENSG00000138193	-0,374394865	0,00000042	0,000002
PHKG1	protein_coding	ENSG00000164776	1,665372157	0,000000423	0,00000201
ETV6	protein_coding	ENSG00000139083	0,349444025	0,000000424	0,00000201
BCAS3	protein_coding	ENSG00000141376	0,305181241	0,000000425	0,00000202
AC011481.1	protein_coding	ENSG00000130204	-0,324602168	0,000000427	0,00000203
NME3	protein_coding	ENSG00000103024	-0,427015472	0,000000429	0,00000204
AL355304.1	lincRNA	ENSG00000232618	3,220315899	0,000000433	0,00000206
TMEM173	protein_coding	ENSG00000184584	0,45261179	0,000000435	0,00000206
RAB8B	protein_coding	ENSG00000166128	0,302261119	0,000000433	0,00000206
IPPK	protein_coding	ENSG00000127080	0,319482109	0,000000436	0,00000207
NRDC	protein_coding	ENSG00000078618	0,166936699	0,000000436	0,00000207
USP1	protein_coding	ENSG00000162607	-0,393352236	0,000000436	0,00000207
TRIM27	protein_coding	ENSG00000204713	-0,223415388	0,000000442	0,00000209
ARGLU1	protein_coding	ENSG00000134884	-0,26934233	0,00000044	0,00000209
MSTO2P	unprocessed_pseudoge	ENSG00000203761	1,054639921	0,000000442	0,00000209
ADHFE1	protein_coding	ENSG00000147576	1,432464994	0,000000443	0,0000021
CACNB1	protein_coding	ENSG00000067191	0,64016265	0,000000447	0,00000212
DNAJC11	protein_coding	ENSG00000007923	-0,293151828	0,000000448	0,00000212
FERMT1	protein_coding	ENSG00000101311	-0,306103463	0,000000452	0,00000214
DSTNP2	transcribed_unprocesse	ENSG00000248593	-0,70105539	0,000000464	0,00000219
PMS2P1	unprocessed_pseudoge	ENSG00000078319	-0,413439813	0,000000462	0,00000219
AL139099.1	antisense	ENSG00000258377	1,846381803	0,000000466	0,00000221
MTM1	protein_coding	ENSG00000171100	0,343891648	0,000000468	0,00000221
AMMECR1L	protein_coding	ENSG00000144233	-0,238131034	0,000000468	0,00000221
SLC35E1	protein_coding	ENSG00000127526	-0,296959709	0,000000467	0,00000221
CPSF3	protein_coding	ENSG00000119203	-0,302570117	0,000000468	0,00000221
FAR1	protein_coding	ENSG00000197601	-0,390955761	0,000000467	0,00000221
GPATCH2	protein_coding	ENSG00000092978	0,259990849	0,000000472	0,00000223
TAF1B	protein_coding	ENSG00000115750	-0,365945717	0,000000471	0,00000223
ZNF322	protein_coding	ENSG00000181315	-0,400918905	0,000000473	0,00000223
CCDC88C	protein_coding	ENSG00000015133	-0,683592111	0,000000474	0,00000224
BRF2	protein_coding	ENSG00000104221	0,371064692	0,000000478	0,00000226
EIF3B	protein_coding	ENSG00000106263	-0,242651317	0,000000481	0,00000227
TIMM22	protein_coding	ENSG00000177370	-0,281337452	0,000000482	0,00000227
LTBP1	protein_coding	ENSG00000049323	-0,48438334	0,000000481	0,00000227
ACOX3	protein_coding	ENSG00000087008	-0,372271745	0,000000483	0,00000228
CDK10	protein_coding	ENSG00000185324	-0,38663401	0,000000483	0,00000228

ZNF385D	protein_coding	ENSG00000151789	4,654886057	0,000000488	0,0000023
LAMC3	protein_coding	ENSG00000050555	1,991632933	0,000000487	0,0000023
CATSPERD	protein_coding	ENSG00000174898	2,460541015	0,000000491	0,00000231
SAMD3	protein_coding	ENSG00000164483	0,410709208	0,00000049	0,00000231
UFM1	protein_coding	ENSG00000120686	0,311462339	0,000000491	0,00000231
MESP1	protein_coding	ENSG00000166823	-0,761254964	0,000000491	0,00000231
AC090192.2	lincRNA	ENSG00000253227	6,02745879	0,000000492	0,00000232
WLS	protein_coding	ENSG00000116729	0,240072074	0,000000493	0,00000232
PDCD2	protein_coding	ENSG00000071994	-0,271856024	0,000000494	0,00000232
PTGIR	protein_coding	ENSG00000160013	0,516713661	0,000000494	0,00000233
ARHGAP18	protein_coding	ENSG00000146376	-0,30022506	0,0000005	0,00000235
DLL1	protein_coding	ENSG00000198719	-0,791308928	0,000000499	0,00000235
IFFO2	protein_coding	ENSG00000169991	0,480273759	0,000000503	0,00000236
ATP6V0D1	protein_coding	ENSG00000159720	0,291535454	0,000000505	0,00000237
AL928654.1	antisense	ENSG00000251602	-0,552208746	0,000000507	0,00000238
GUCY1B1	protein_coding	ENSG00000061918	-0,557738857	0,000000507	0,00000238
PRR5	protein_coding	ENSG00000186654	-0,7663006	0,000000506	0,00000238
RF00019	misc_RNA	ENSG00000222881	-1,803471228	0,000000508	0,00000239
HSPA14	protein_coding	ENSG00000284024	0,573093013	0,000000513	0,00000241
PHYHIP	protein_coding	ENSG00000168490	1,159428373	0,000000519	0,00000243
BRD7	protein_coding	ENSG00000166164	-0,330802431	0,000000517	0,00000243
CCR10	protein_coding	ENSG00000184451	1,20973128	0,000000522	0,00000245
NT5DC2	protein_coding	ENSG00000168268	-0,261342782	0,000000523	0,00000245
AC097662.1	antisense	ENSG00000236432	1,278152461	0,000000526	0,00000246
SRCIN1	protein_coding	ENSG00000277363	0,806431918	0,000000524	0,00000246
GOLPH3L	protein_coding	ENSG00000143457	0,270372584	0,000000525	0,00000246
ZNF3	protein_coding	ENSG00000166526	0,316855051	0,000000527	0,00000247
SCD	protein_coding	ENSG00000099194	0,358672708	0,000000533	0,0000025
POLA2	protein_coding	ENSG00000014138	-0,249319723	0,000000534	0,0000025
BLOC1S5	protein_coding	ENSG00000188428	-0,327789555	0,000000536	0,00000251
MRPL36	protein_coding	ENSG00000171421	-0,349471378	0,000000541	0,00000253
GASK1B	protein_coding	ENSG00000164125	1,532610766	0,000000542	0,00000254
PDS5A	protein_coding	ENSG00000121892	-0,290399931	0,000000544	0,00000255
WFDC3	protein_coding	ENSG00000124116	1,536283268	0,000000548	0,00000256
AMHR2	protein_coding	ENSG00000135409	1,968620571	0,000000549	0,00000257
PXT1	protein_coding	ENSG00000179165	1,960978733	0,00000055	0,00000257
CP	protein_coding	ENSG00000047457	1,232366974	0,000000551	0,00000258
TRIP12	protein_coding	ENSG00000153827	0,229044093	0,000000558	0,00000261
ABI2	protein_coding	ENSG00000138443	-0,279351784	0,000000558	0,00000261
PCGF6	protein_coding	ENSG00000156374	-0,346588932	0,000000562	0,00000263

B4GALNT1	protein_coding	ENSG00000135454	-0,382375166	0,000000565	0,00000264
GLP2R	protein_coding	ENSG00000065325	2,032161504	0,000000567	0,00000265
PPP2R2A	protein_coding	ENSG00000221914	0,260966695	0,00000057	0,00000266
MCHR1	protein_coding	ENSG00000128285	3,572639236	0,000000577	0,00000269
TSC1	protein_coding	ENSG00000165699	0,247263809	0,000000576	0,00000269
GIPC2	protein_coding	ENSG00000137960	1,047760156	0,000000582	0,00000271
ZNF195	protein_coding	ENSG00000005801	0,384758226	0,000000581	0,00000271
VOPP1	protein_coding	ENSG00000154978	-0,268427887	0,000000581	0,00000271
PRR7	protein_coding	ENSG00000131188	-0,422319151	0,000000585	0,00000273
KLHL29	protein_coding	ENSG00000119771	0,532075145	0,000000588	0,00000274
PSPH	protein_coding	ENSG00000146733	0,336400253	0,00000059	0,00000275
LPIN2	protein_coding	ENSG00000101577	0,377472485	0,000000593	0,00000276
NUS1	protein_coding	ENSG00000153989	0,318032584	0,000000593	0,00000276
NUMA1	protein_coding	ENSG00000137497	-0,515596329	0,000000597	0,00000278
CRB1	protein_coding	ENSG00000134376	4,366532356	0,000000602	0,0000028
YEATS4	protein_coding	ENSG00000127337	-0,505594757	0,000000601	0,0000028
EMC1-AS1	antisense	ENSG00000230424	2,297325692	0,000000603	0,00000281
DEGS1	protein_coding	ENSG00000143753	-0,246155794	0,000000604	0,00000281
SZRD1	protein_coding	ENSG00000055070	-0,271555202	0,000000604	0,00000281
SATB1	protein_coding	ENSG00000182568	0,517259945	0,000000607	0,00000282
WTAPP1	transcribed_processed_	ENSG00000255282	2,153165963	0,000000606	0,00000282
AKAP8	protein_coding	ENSG00000105127	-0,263919272	0,000000616	0,00000286
COQ9	protein_coding	ENSG00000088682	-0,332416661	0,000000618	0,00000287
DLC1	protein_coding	ENSG00000164741	-0,233778548	0,000000621	0,00000288
ZNF473	protein_coding	ENSG00000142528	-0,253429253	0,00000062	0,00000288
CPAMD8	protein_coding	ENSG00000160111	1,65370296	0,000000621	0,00000289
MRPL14	protein_coding	ENSG00000180992	-0,359790956	0,000000628	0,00000292
IRAK3	protein_coding	ENSG00000090376	3,145805297	0,000000631	0,00000293
GRHPR	protein_coding	ENSG00000137106	-0,28030403	0,000000633	0,00000294
USP9X	protein_coding	ENSG00000124486	0,297112781	0,000000638	0,00000296
WEE2-AS1	antisense	ENSG00000228775	1,935519085	0,000000641	0,00000297
ITGAV	protein_coding	ENSG00000138448	0,248558729	0,000000641	0,00000297
LETM1	protein_coding	ENSG00000168924	-0,300179345	0,000000643	0,00000298
ZEB1-AS1	antisense	ENSG00000237036	-0,587111172	0,000000645	0,00000299
VIM-AS1	antisense	ENSG00000229124	0,552247885	0,000000648	0,000003
PPAN	protein_coding	ENSG00000130810	-0,627374917	0,000000649	0,00000301
OIP5-AS1	processed_transcript	ENSG00000247556	-0,276254033	0,000000651	0,00000302
TBC1D16	protein_coding	ENSG00000167291	-0,419619702	0,000000653	0,00000302
ADAMTS7	protein_coding	ENSG00000136378	1,119897756	0,000000665	0,00000308
SUMF1	protein_coding	ENSG00000144455	-0,262820616	0,000000668	0,00000309

LINC02362	lincRNA	ENSG00000249096	2,331939698	0,000000672	0,00000311
BPGM	protein_coding	ENSG00000172331	0,311293327	0,000000673	0,00000311
CHST6	protein_coding	ENSG00000183196	5,943724521	0,000000675	0,00000312
KIF14	protein_coding	ENSG00000118193	-0,385994538	0,000000675	0,00000312
THOC1	protein_coding	ENSG00000079134	-0,345836253	0,000000678	0,00000313
FNBP1L	protein_coding	ENSG00000137942	-0,26468752	0,000000681	0,00000315
KCNN4	protein_coding	ENSG00000104783	-0,336024506	0,000000681	0,00000315
TNIP2	protein_coding	ENSG00000168884	0,344118057	0,000000689	0,00000319
FAM241A	protein_coding	ENSG00000174749	0,485783869	0,000000692	0,0000032
KLF12	protein_coding	ENSG00000118922	0,374084081	0,000000693	0,0000032
ATP11C	protein_coding	ENSG00000101974	-0,346742279	0,000000696	0,00000321
KCTD15	protein_coding	ENSG00000153885	-0,395881634	0,0000007	0,00000323
TYRP1	protein_coding	ENSG00000107165	0,646218198	0,000000702	0,00000324
E2F4	protein_coding	ENSG00000205250	-0,27057725	0,000000701	0,00000324
ANAPC15	protein_coding	ENSG00000110200	-0,373011242	0,000000701	0,00000324
TASOR	protein_coding	ENSG00000163946	-0,285535012	0,000000703	0,00000325
CELSR1	protein_coding	ENSG00000075275	-0,465534998	0,000000705	0,00000325
LINC01802	lincRNA	ENSG00000225064	4,24711484	0,00000071	0,00000327
UQCC1	protein_coding	ENSG00000101019	0,273805498	0,000000711	0,00000328
TGS1	protein_coding	ENSG00000137574	0,239062865	0,000000713	0,00000329
CDNF	protein_coding	ENSG00000185267	1,176639342	0,000000717	0,0000033
ZNF470	protein_coding	ENSG00000197016	0,469823591	0,000000718	0,00000331
AC010834.2	antisense	ENSG00000253848	2,389248687	0,000000721	0,00000332
NOTCH1	protein_coding	ENSG00000148400	0,594876265	0,000000721	0,00000332
CBX6	protein_coding	ENSG00000183741	-0,424869345	0,000000722	0,00000332
AC145098.1	antisense	ENSG00000248996	3,39078091	0,000000723	0,00000333
LINC02158	antisense	ENSG00000225611	4,103236718	0,000000726	0,00000334
EED	protein_coding	ENSG00000074266	-0,279262199	0,000000726	0,00000334
RTCA	protein_coding	ENSG00000137996	0,219065269	0,000000728	0,00000335
FAM72D	protein_coding	ENSG00000215784	-0,616506076	0,000000729	0,00000335
CLEC4M	protein_coding	ENSG00000104938	3,712329642	0,000000731	0,00000336
CSF3	protein_coding	ENSG00000108342	1,805993831	0,000000736	0,00000338
STX5	protein_coding	ENSG00000162236	0,266965059	0,000000738	0,00000339
MTSS1L	protein_coding	ENSG00000132613	-0,366061189	0,000000737	0,00000339
HGS	protein_coding	ENSG00000185359	-0,281438866	0,000000743	0,00000341
CRYAB	protein_coding	ENSG00000109846	2,87969403	0,000000747	0,00000343
TRIP6	protein_coding	ENSG00000087077	-0,339684473	0,000000748	0,00000343
TMEM62	protein_coding	ENSG00000137842	0,364685916	0,000000748	0,00000344
AL645608.8	lincRNA	ENSG00000273443	2,410068841	0,000000759	0,00000348
DOCK5	protein_coding	ENSG00000147459	-0,318620655	0,00000076	0,00000349

PPIH	protein_coding	ENSG00000171960	-0,363667594	0,000000761	0,00000349
ATP5F1D	protein_coding	ENSG00000099624	-0,370171606	0,000000763	0,0000035
ZNF655	protein_coding	ENSG00000197343	0,225153165	0,000000765	0,00000351
AC093425.1	processed_transcript	ENSG00000224609	1,769643527	0,000000768	0,00000352
RF00003	snRNA	ENSG00000270722	-0,824058839	0,000000771	0,00000353
MRPL3	protein_coding	ENSG00000114686	-0,350093075	0,000000773	0,00000354
PPP4R4	protein_coding	ENSG00000119698	0,440523056	0,000000775	0,00000355
PSIP1	protein_coding	ENSG00000164985	-0,347725274	0,000000777	0,00000356
ZNF76	protein_coding	ENSG00000065029	-0,311371509	0,000000784	0,00000359
ARHGEF3	protein_coding	ENSG00000163947	0,385244203	0,000000788	0,00000361
SEMA6D	protein_coding	ENSG00000137872	2,396335339	0,000000796	0,00000364
PARP4	protein_coding	ENSG00000102699	-0,234753722	0,000000795	0,00000364
FSTL3	protein_coding	ENSG00000070404	0,363929419	0,000000807	0,00000369
NIPAL3	protein_coding	ENSG00000001461	0,237263881	0,000000806	0,00000369
GNPAT	protein_coding	ENSG00000116906	-0,279198717	0,000000806	0,00000369
MYBBP1A	protein_coding	ENSG00000132382	-0,359113212	0,000000823	0,00000376
FSD1	protein_coding	ENSG00000105255	-0,318945906	0,000000827	0,00000378
RHOBTB2	protein_coding	ENSG00000008853	-0,444234219	0,00000083	0,00000379
GINS4	protein_coding	ENSG00000147536	-0,25821947	0,000000832	0,0000038
AC005865.1	lincRNA	ENSG00000236908	2,176433248	0,000000836	0,00000382
ZNF71	protein_coding	ENSG00000197951	-0,307087962	0,000000836	0,00000382
TRAM1L1	protein_coding	ENSG00000174599	-0,69990399	0,000000841	0,00000384
DROSHA	protein_coding	ENSG00000113360	-0,321809794	0,000000848	0,00000387
MAP3K20	protein_coding	ENSG00000091436	-0,228939498	0,000000852	0,00000389
TMEM131L	protein_coding	ENSG00000121210	-0,381465418	0,000000853	0,00000389
LANCL2	protein_coding	ENSG00000132434	-0,32334303	0,000000856	0,00000391
ZNF792	protein_coding	ENSG00000180884	-0,428123847	0,000000859	0,00000392
FBXO42	protein_coding	ENSG00000037637	0,276244536	0,000000871	0,00000397
ELF4	protein_coding	ENSG00000102034	0,296595062	0,000000872	0,00000398
DENND6A-AS1	antisense	ENSG00000239801	4,532524096	0,000000876	0,00000399
EEF2KMT	protein_coding	ENSG00000118894	-0,387639083	0,000000876	0,00000399
CPNE8	protein_coding	ENSG00000139117	0,302738586	0,000000879	0,000004
DNAAF2	protein_coding	ENSG00000165506	-0,336297241	0,000000879	0,000004
ECE2	protein_coding	ENSG00000145194	-0,494002842	0,000000881	0,00000401
C1orf68	protein_coding	ENSG00000198854	3,626591718	0,000000883	0,00000402
KDM7A	protein_coding	ENSG00000006459	0,566798686	0,000000891	0,00000405
EIF2B2	protein_coding	ENSG00000119718	0,288983766	0,000000892	0,00000406
FSCN2	protein_coding	ENSG00000186765	3,636376299	0,000000895	0,00000407
NAA15	protein_coding	ENSG00000164134	-0,243257604	0,000000911	0,00000414
TTF2	protein_coding	ENSG00000116830	-0,256676434	0,00000091	0,00000414

UCA1	lincRNA	ENSG00000214049	1,442747595	0,000000913	0,00000415
CAT	protein_coding	ENSG00000121691	-0,295483073	0,000000915	0,00000416
ODC1	protein_coding	ENSG00000115758	0,21069644	0,000000918	0,00000417
ZNF704	protein_coding	ENSG00000164684	-0,521335418	0,000000919	0,00000417
LINC00887	lincRNA	ENSG00000214145	4,637243368	0,000000924	0,0000042
INTS3	protein_coding	ENSG00000143624	-0,310725186	0,000000927	0,00000421
SNORD3C	snoRNA	ENSG00000264940	-0,920944352	0,000000928	0,00000421
UBIAD1	protein_coding	ENSG00000120942	-0,304855196	0,00000093	0,00000422
GP1BA	protein_coding	ENSG00000185245	4,59592325	0,000000936	0,00000425
DSEL	protein_coding	ENSG00000171451	0,23334075	0,000000942	0,00000427
C6orf136	protein_coding	ENSG00000204564	-0,498851489	0,000000946	0,00000429
NIPSNAP2	protein_coding	ENSG00000146729	-0,348471849	0,000000948	0,0000043
DHRS7	protein_coding	ENSG00000100612	0,29678739	0,000000951	0,00000431
DHFR2	protein_coding	ENSG00000178700	-0,386975044	0,000000954	0,00000432
RASA1	protein_coding	ENSG00000145715	-0,303269566	0,000000956	0,00000433
PLA2G6	protein_coding	ENSG00000184381	0,77044191	0,000000959	0,00000434
VPS13C	protein_coding	ENSG00000129003	0,300955571	0,000000959	0,00000434
ACTR1A	protein_coding	ENSG00000138107	-0,194061549	0,000000958	0,00000434
GJC1	protein_coding	ENSG00000182963	-0,302057357	0,000000957	0,00000434
EXOSC7	protein_coding	ENSG00000075914	-0,272746794	0,000000961	0,00000435
BRMS1L	protein_coding	ENSG00000100916	0,422606061	0,000000966	0,00000437
CCDC26	lincRNA	ENSG00000229140	2,278384614	0,000000971	0,00000439
CINP	protein_coding	ENSG00000100865	-0,348099244	0,000000971	0,00000439
WDR49	protein_coding	ENSG00000174776	1,529717098	0,000000974	0,00000441
MAGEE1	protein_coding	ENSG00000198934	-0,486574444	0,000000974	0,00000441
CNOT11	protein_coding	ENSG00000158435	-0,190063223	0,000000977	0,00000442
KCND3	protein_coding	ENSG00000171385	2,018863689	0,000000993	0,00000449
H2AFY2	protein_coding	ENSG00000099284	0,593118094	0,000001	0,00000453
SLC27A5	protein_coding	ENSG00000083807	-0,439941166	0,000001	0,00000454
AP1S2	protein_coding	ENSG00000182287	0,385382389	0,00000101	0,00000456
LINC00565	lincRNA	ENSG00000260910	1,759763542	0,00000102	0,00000459
DONSON	protein_coding	ENSG00000159147	-0,352727885	0,00000102	0,0000046
HSPA2	protein_coding	ENSG00000126803	-0,253536996	0,00000102	0,00000461
LYAR	protein_coding	ENSG00000145220	-0,291236203	0,00000102	0,00000461
ZDHHC7	protein_coding	ENSG00000153786	-0,22189568	0,00000103	0,00000465
THRB	protein_coding	ENSG00000151090	0,404857516	0,00000104	0,00000469
AHSA1	protein_coding	ENSG00000100591	-0,191423291	0,00000104	0,00000469
PLEKHG4	protein_coding	ENSG00000196155	-0,393221416	0,00000104	0,0000047
ADD1	protein_coding	ENSG00000087274	-0,176467558	0,00000105	0,00000472
AC083973.1	antisense	ENSG00000253408	1,383184796	0,00000105	0,00000473

KIRREL3	protein_coding	ENSG00000149571	-0,299194271	0,00000105	0,00000473
LINC00339	processed_transcript	ENSG00000218510	-0,361497559	0,00000105	0,00000474
RASAL2	protein_coding	ENSG00000075391	0,239324541	0,00000105	0,00000474
MYL4	protein_coding	ENSG00000198336	2,675737901	0,00000106	0,00000475
TRIM39	protein_coding	ENSG00000204599	0,351136295	0,00000105	0,00000475
METTL2A	protein_coding	ENSG00000087995	-0,345451392	0,00000106	0,00000475
AC105411.1	antisense	ENSG00000259867	2,317131063	0,00000106	0,00000476
AL591846.2	antisense	ENSG00000237605	2,265283336	0,00000106	0,00000476
RERGL	protein_coding	ENSG00000111404	2,410550744	0,00000106	0,00000476
MPP4	protein_coding	ENSG00000082126	0,292342799	0,00000106	0,00000476
TP53I11	protein_coding	ENSG00000175274	1,702967362	0,00000106	0,00000477
DOCK9	protein_coding	ENSG00000088387	0,332274196	0,00000106	0,00000477
CERS5	protein_coding	ENSG00000139624	0,286289074	0,00000106	0,00000477
AC008972.2	lincRNA	ENSG00000272081	1,231313292	0,00000106	0,00000478
IST1	protein_coding	ENSG00000182149	-0,174345105	0,00000107	0,00000483
RFPL4AP6	unprocessed_pseudoge	ENSG00000278292	5,819786181	0,00000108	0,00000485
AC019080.1	sense_overlapping	ENSG00000213963	0,982591844	0,00000108	0,00000486
TSFM	protein_coding	ENSG00000123297	-0,329516277	0,00000109	0,00000488
AL121772.1	lincRNA	ENSG00000274414	2,809361643	0,00000109	0,00000489
RAD21L1	protein_coding	ENSG00000244588	0,580120003	0,0000011	0,00000492
C11orf71	protein_coding	ENSG00000180425	-0,48784392	0,0000011	0,00000492
LINC01136	lincRNA	ENSG00000233791	2,7754533	0,0000011	0,00000494
IFNGR2	protein_coding	ENSG00000159128	0,252373304	0,00000111	0,00000497
AZIN2	protein_coding	ENSG00000142920	0,589242161	0,00000112	0,00000502
KCTD12	protein_coding	ENSG00000178695	-0,363121604	0,00000112	0,00000503
AKNAD1	protein_coding	ENSG00000162641	0,724285283	0,00000113	0,00000505
UBE2D2	protein_coding	ENSG00000131508	-0,178351327	0,00000114	0,00000509
NOL4L	protein_coding	ENSG00000197183	0,758022453	0,00000114	0,00000511
ZSWIM4	protein_coding	ENSG00000132003	0,435334179	0,00000114	0,00000511
CTBS	protein_coding	ENSG00000117151	0,391364486	0,00000114	0,00000512
PSD4	protein_coding	ENSG00000125637	-0,886557448	0,00000115	0,00000515
ARL4A	protein_coding	ENSG00000122644	0,230503918	0,00000116	0,00000518
TUBB2B	protein_coding	ENSG00000137285	0,695291639	0,00000116	0,00000519
VKORC1	protein_coding	ENSG00000167397	-0,446135891	0,00000116	0,00000519
GTF2H2	protein_coding	ENSG00000145736	-0,457996812	0,00000116	0,00000519
GTF2H2	protein_coding	ENSG00000145736	-0,457996812	0,00000116	0,00000519
KLHDC7A	protein_coding	ENSG00000179023	4,109008848	0,00000117	0,00000522
AC022167.2	antisense	ENSG00000260276	1,346309564	0,00000117	0,00000524
NREP	protein_coding	ENSG00000134986	-0,33677908	0,00000117	0,00000524
RBBP4	protein_coding	ENSG00000162521	-0,264265836	0,00000117	0,00000525

SHLD2	protein_coding	ENSG00000122376	-0,330231361	0,00000118	0,00000526
IFNL3P1	unprocessed_pseudoge	ENSG00000268510	4,484205517	0,00000118	0,00000526
ANO9	protein_coding	ENSG00000185101	3,125481914	0,00000118	0,00000528
RBMX2	protein_coding	ENSG00000134597	-0,29825872	0,00000119	0,00000529
EXOSC4	protein_coding	ENSG00000178896	-0,378931606	0,00000119	0,00000531
FAM222A	protein_coding	ENSG00000139438	0,742811756	0,0000012	0,00000534
KLRC2	protein_coding	ENSG00000205809	3,991689016	0,0000012	0,00000535
ACP7	protein_coding	ENSG00000183760	4,531009943	0,00000121	0,00000538
PYCARD	protein_coding	ENSG00000103490	-0,739830169	0,00000121	0,00000538
LEMD1-AS1	antisense	ENSG00000226235	5,809203	0,00000121	0,00000542
AC133785.1	antisense	ENSG00000233221	1,071021761	0,00000122	0,00000543
TRIM41	protein_coding	ENSG00000146063	-0,444844653	0,00000122	0,00000545
OTX1	protein_coding	ENSG00000115507	-1,296166094	0,00000123	0,00000546
RASSF7	protein_coding	ENSG00000099849	-0,468366778	0,00000123	0,00000549
KCNH1	protein_coding	ENSG00000143473	0,615648808	0,00000124	0,00000553
MSX1	protein_coding	ENSG00000163132	0,718352137	0,00000124	0,00000554
ARPC5	protein_coding	ENSG00000162704	-0,295527196	0,00000124	0,00000554
STXBP5-AS1	antisense	ENSG00000233452	1,085442084	0,00000125	0,00000555
TPCN1	protein_coding	ENSG00000186815	0,351391278	0,00000125	0,00000555
SP110	protein_coding	ENSG00000135899	0,28814434	0,00000125	0,00000555
TNFRSF11A	protein_coding	ENSG00000141655	-0,345588895	0,00000125	0,00000555
E4F1	protein_coding	ENSG00000167967	-0,366183223	0,00000125	0,00000555
CREB1	protein_coding	ENSG00000118260	-0,246861366	0,00000126	0,00000559
HECA	protein_coding	ENSG00000112406	0,3122016	0,00000127	0,00000563
SMIM2-AS1	antisense	ENSG00000227258	0,964969413	0,00000127	0,00000564
ZCCHC17	protein_coding	ENSG00000121766	-0,299862966	0,00000127	0,00000564
FNDC7	protein_coding	ENSG00000143107	3,334453805	0,00000127	0,00000566
C2CD3	protein_coding	ENSG00000168014	0,300907158	0,00000127	0,00000566
MARVELD2	protein_coding	ENSG00000152939	1,967140892	0,00000128	0,00000568
DCP1A	protein_coding	ENSG00000272886	0,28537497	0,00000128	0,00000569
OOSP1P1	processed_pseudogene	ENSG00000238037	6,132874067	0,00000128	0,0000057
FGR	protein_coding	ENSG00000000938	4,480625637	0,00000128	0,0000057
GMPS	protein_coding	ENSG00000163655	-0,254785525	0,00000128	0,0000057
ATP1B1	protein_coding	ENSG00000143153	0,217366127	0,00000129	0,00000572
TMEM109	protein_coding	ENSG00000110108	-0,267421743	0,00000129	0,00000572
AC119751.3	unprocessed_pseudoge	ENSG00000248583	-0,511007071	0,00000129	0,00000573
USP22	protein_coding	ENSG00000124422	-0,273252537	0,0000013	0,00000575
IGF2R	protein_coding	ENSG00000197081	0,400738598	0,0000013	0,00000578
AC019197.1	antisense	ENSG00000236283	3,944227671	0,00000131	0,00000581
CLIP2	protein_coding	ENSG00000106665	0,47713316	0,00000132	0,00000585

ANK2	protein_coding	ENSG00000145362	0,280589252	0,00000133	0,00000589
AC060766.7	processed_transcript	ENSG00000267745	0,676692628	0,00000133	0,0000059
RNF10	protein_coding	ENSG00000022840	0,24495436	0,00000133	0,0000059
MAN1C1	protein_coding	ENSG00000117643	2,189113685	0,00000133	0,00000591
KISS1	protein_coding	ENSG00000170498	1,513650982	0,00000134	0,00000593
PCOLCE2	protein_coding	ENSG00000163710	-0,370552868	0,00000134	0,00000593
MFNG	protein_coding	ENSG00000100060	0,938516767	0,00000135	0,00000595
ISL2	protein_coding	ENSG00000159556	0,780248624	0,00000134	0,00000595
FAM92B	protein_coding	ENSG00000153789	4,435026883	0,00000136	0,000006
KTI12	protein_coding	ENSG00000198841	-0,413607914	0,00000136	0,000006
VTI1B	protein_coding	ENSG00000100568	-0,211449115	0,00000136	0,00000601
RASGRF1	protein_coding	ENSG00000058335	0,860160922	0,00000136	0,00000603
SLC31A2	protein_coding	ENSG00000136867	0,374134394	0,00000138	0,00000607
AC068473.5	lincRNA	ENSG00000274828	-0,902660811	0,00000138	0,00000609
AMPD3	protein_coding	ENSG00000133805	0,442174159	0,00000138	0,0000061
FRMD6-AS1	antisense	ENSG00000273888	0,829777207	0,00000139	0,00000612
SCHIP1	protein_coding	ENSG00000151967	0,803140214	0,00000139	0,00000613
CSNK2A2	protein_coding	ENSG00000070770	0,25326129	0,00000139	0,00000613
ALG3	protein_coding	ENSG00000214160	-0,291392635	0,0000014	0,00000617
AASDHPPT	protein_coding	ENSG00000149313	-0,289457534	0,00000141	0,00000623
NETO2	protein_coding	ENSG00000171208	-0,33393395	0,00000142	0,00000624
DDAH2	protein_coding	ENSG00000213722	-0,356058686	0,00000142	0,00000625
CBR4	protein_coding	ENSG00000145439	-0,322721798	0,00000143	0,00000631
GBP6	protein_coding	ENSG00000183347	5,903654721	0,00000143	0,00000632
RB1CC1	protein_coding	ENSG00000023287	0,214932955	0,00000144	0,00000635
MIER1	protein_coding	ENSG00000198160	-0,368606116	0,00000145	0,00000637
ATP6V1FNB	protein_coding	ENSG00000272899	1,687006667	0,00000145	0,00000638
ALDH3A2	protein_coding	ENSG00000072210	-0,238045566	0,00000146	0,00000642
HSPB9	protein_coding	ENSG00000260325	5,928592485	0,00000147	0,00000646
TRAC	TR_C_gene	ENSG00000277734	5,935812646	0,00000147	0,00000646
UBA2	protein_coding	ENSG00000126261	-0,331730714	0,00000147	0,00000648
LINC00941	lincRNA	ENSG00000235884	0,345335618	0,00000148	0,0000065
FBXO6	protein_coding	ENSG00000116663	-0,706143989	0,00000148	0,0000065
TLE3	protein_coding	ENSG00000140332	-0,441321164	0,00000148	0,00000652
VMA21	protein_coding	ENSG00000160131	-0,320003879	0,00000149	0,00000656
IFT80	protein_coding	ENSG00000068885	0,441881532	0,0000015	0,00000658
SQSTM1	protein_coding	ENSG00000161011	0,287785624	0,00000151	0,00000662
EARS2	protein_coding	ENSG00000103356	-0,288762788	0,00000151	0,00000664
METRNL	protein_coding	ENSG00000176845	0,492567531	0,00000153	0,0000067
NODAL	protein_coding	ENSG00000156574	4,413989286	0,00000154	0,00000675

RCN2	protein_coding	ENSG00000117906	-0,354177909	0,00000154	0,00000675
ARMC4	protein_coding	ENSG00000169126	-0,301108143	0,00000155	0,00000679
ARHGAP33	protein_coding	ENSG00000004777	-0,700035374	0,00000156	0,00000683
PRR20G	protein_coding	ENSG00000239620	4,524270226	0,00000156	0,00000684
NR4A3	protein_coding	ENSG00000119508	1,440513897	0,00000158	0,00000692
INTS6L	protein_coding	ENSG00000165359	0,484137109	0,00000158	0,00000693
MEX3D	protein_coding	ENSG00000181588	-0,403684198	0,00000158	0,00000694
KXD1	protein_coding	ENSG00000105700	-0,217932421	0,00000159	0,00000698
TMEM192	protein_coding	ENSG00000170088	0,267301198	0,0000016	0,000007
NAP1L4	protein_coding	ENSG00000205531	-0,159578922	0,0000016	0,00000702
TMEM147-AS1	antisense	ENSG00000236144	-0,551474093	0,00000162	0,00000708
NUDT19	protein_coding	ENSG00000213965	-0,313651096	0,00000162	0,0000071
AC005520.2	antisense	ENSG00000259065	1,724572279	0,00000163	0,00000715
KMT2C	protein_coding	ENSG00000055609	0,334570483	0,00000163	0,00000715
DDX50	protein_coding	ENSG00000107625	-0,271086385	0,00000164	0,00000716
ENAH	protein_coding	ENSG00000154380	0,238964091	0,00000164	0,00000717
MTMR10	protein_coding	ENSG00000166912	0,255456932	0,00000165	0,00000722
BACH1	protein_coding	ENSG00000156273	0,211953549	0,00000165	0,00000722
GIGYF1	protein_coding	ENSG00000146830	-0,625320328	0,00000166	0,00000726
HSP90AA1	protein_coding	ENSG00000080824	-0,352803216	0,00000166	0,00000727
TAF4	protein_coding	ENSG00000130699	-0,471618662	0,00000168	0,00000736
LINC02288	lincRNA	ENSG00000246548	4,385754505	0,00000169	0,00000738
MTCL1	protein_coding	ENSG00000168502	-0,432644259	0,00000169	0,00000739
PIK3IP1	protein_coding	ENSG00000100100	1,292801851	0,00000169	0,0000074
CTNNBIP1	protein_coding	ENSG00000178585	0,331612891	0,00000171	0,00000745
TMEM51	protein_coding	ENSG00000171729	-0,367043355	0,00000171	0,00000745
KLHL12	protein_coding	ENSG00000117153	-0,255735709	0,00000171	0,00000746
AAMP	protein_coding	ENSG00000127837	-0,240829318	0,00000171	0,00000747
HECW1	protein_coding	ENSG00000002746	0,857062919	0,00000171	0,00000748
LYRM7	protein_coding	ENSG00000186687	-0,404586426	0,00000172	0,00000748
AC011611.2	lincRNA	ENSG00000257329	4,086353454	0,00000172	0,00000752
PRR5L	protein_coding	ENSG00000135362	-0,282648941	0,00000173	0,00000752
RAB1B	protein_coding	ENSG00000174903	-0,257277775	0,00000173	0,00000754
LANCL1	protein_coding	ENSG00000115365	-0,246250604	0,00000173	0,00000755
ZYG11A	protein_coding	ENSG00000203995	-0,411725089	0,00000173	0,00000755
ADAMTS1	protein_coding	ENSG00000154734	-0,312608769	0,00000174	0,00000757
RECQL4	protein_coding	ENSG00000160957	-0,409723307	0,00000174	0,00000757
ISCU	protein_coding	ENSG00000136003	0,23436444	0,00000175	0,00000763
MXI1	protein_coding	ENSG00000119950	-0,346834857	0,00000175	0,00000764
WDR36	protein_coding	ENSG00000134987	-0,2784427	0,00000176	0,00000767

TRIM6	protein_coding	ENSG00000121236	-0,40528909	0,00000176	0,00000767
RORB	protein_coding	ENSG00000198963	2,66302594	0,00000177	0,00000769
NAPRT	protein_coding	ENSG00000147813	-0,826134265	0,00000177	0,00000769
BACE2	protein_coding	ENSG00000182240	-0,259356069	0,00000179	0,0000078
IER3-AS1	antisense	ENSG00000272273	2,185576779	0,0000018	0,00000784
MARK4	protein_coding	ENSG00000007047	0,418152297	0,00000182	0,0000079
MRPL4	protein_coding	ENSG00000105364	-0,294158819	0,00000182	0,0000079
PIAS3	protein_coding	ENSG00000131788	-0,297694501	0,00000182	0,00000791
AL356215.1	antisense	ENSG00000255521	1,719147736	0,00000183	0,00000793
PIWIL4	protein_coding	ENSG00000134627	1,676556835	0,00000183	0,00000794
POLR3GL	protein_coding	ENSG00000121851	0,357984086	0,00000183	0,00000796
ZFYVE9	protein_coding	ENSG00000157077	-0,255572521	0,00000183	0,00000796
RCE1	protein_coding	ENSG00000173653	-0,366709762	0,00000183	0,00000796
LINC01629	lincRNA	ENSG00000258602	2,239796743	0,00000184	0,00000797
CCL4L2	protein_coding	ENSG00000276070	3,981469021	0,00000184	0,00000797
RPAP2	protein_coding	ENSG00000122484	0,236756945	0,00000184	0,00000798
TLR4	protein_coding	ENSG00000136869	1,507473583	0,00000186	0,00000808
ZMYM4	protein_coding	ENSG00000146463	0,191282669	0,00000186	0,00000808
RAB40C	protein_coding	ENSG00000197562	-0,469742037	0,00000186	0,00000808
MYO5B	protein_coding	ENSG00000167306	0,588846303	0,00000187	0,0000081
AC092747.2	unprocessed_pseudoge	ENSG00000256625	1,893406909	0,00000187	0,00000811
AC107308.1	sense_intronic	ENSG00000277945	-1,151295427	0,00000188	0,00000812
LAMB1	protein_coding	ENSG00000091136	-0,307084672	0,00000188	0,00000815
MRPS17	protein_coding	ENSG00000239789	-0,436943322	0,00000189	0,00000819
EIF4G2	protein_coding	ENSG00000110321	0,148633411	0,0000019	0,00000821
DNAH5	protein_coding	ENSG00000039139	0,921165878	0,0000019	0,00000822
SNW1	protein_coding	ENSG00000100603	-0,253683261	0,00000191	0,00000824
KIAA0513	protein_coding	ENSG00000135709	0,465835124	0,00000191	0,00000826
RAB39A	protein_coding	ENSG00000179331	-0,624636718	0,00000192	0,0000083
PLEKHM3	protein_coding	ENSG00000178385	0,341176765	0,00000192	0,00000831
GPANK1	protein_coding	ENSG00000204438	-0,292349267	0,00000193	0,00000836
ZNF767P	transcribed_unprocesse	ENSG00000133624	0,671337464	0,00000194	0,00000838
GTF2H5	protein_coding	ENSG00000272047	-0,364213608	0,00000195	0,00000843
LRRC39	protein_coding	ENSG00000122477	1,391065851	0,00000196	0,00000844
WASL	protein_coding	ENSG00000106299	0,249206592	0,00000196	0,00000844
SPOCK2	protein_coding	ENSG00000107742	2,33438984	0,00000196	0,00000846
TXK	protein_coding	ENSG00000074966	-0,348483893	0,00000196	0,00000846
C4orf3	protein_coding	ENSG00000164096	-0,323719156	0,00000198	0,00000853
AC027682.6	antisense	ENSG00000276075	1,220027173	0,00000199	0,00000857
CH25H	protein_coding	ENSG00000138135	5,729957533	0,00000199	0,00000857

KIF13A	protein_coding	ENSG00000137177	-0,310232075	0,00000199	0,00000858
PHF3	protein_coding	ENSG00000118482	-0,292146086	0,00000199	0,0000086
CCT5	protein_coding	ENSG00000150753	-0,292714313	0,000002	0,00000861
AC019069.1	lincRNA	ENSG00000272711	-0,531226687	0,00000201	0,00000867
ALKBH7	protein_coding	ENSG00000125652	-0,523172813	0,00000202	0,0000087
RAVER2	protein_coding	ENSG00000162437	-0,365426379	0,00000205	0,00000883
PACSIN2	protein_coding	ENSG00000100266	-0,184185514	0,00000206	0,00000885
HNRNPLL	protein_coding	ENSG00000143889	-0,227452948	0,00000206	0,00000886
AVPI1	protein_coding	ENSG00000119986	-0,331643334	0,00000208	0,00000896
SCYL1	protein_coding	ENSG00000142186	0,304190334	0,00000211	0,00000908
PAK4	protein_coding	ENSG00000130669	-0,335469	0,00000211	0,00000908
RIPOR2	protein_coding	ENSG00000111913	4,551125412	0,00000211	0,00000909
TOMM70	protein_coding	ENSG00000154174	-0,245469512	0,00000211	0,00000909
LAMC1	protein_coding	ENSG00000135862	-0,290316172	0,00000212	0,00000911
DENND2D	protein_coding	ENSG00000162777	1,388375495	0,00000212	0,00000912
KLF18	protein_coding	ENSG00000283039	5,788712442	0,00000213	0,00000914
SEC61G	protein_coding	ENSG00000132432	0,282900204	0,00000213	0,00000916
CCDC68	protein_coding	ENSG00000166510	0,490490915	0,00000214	0,00000918
P3H1	protein_coding	ENSG00000117385	-0,261411198	0,00000214	0,00000919
AL355574.1	bidirectional_promoter_	ENSG00000238058	-0,88740814	0,00000214	0,0000092
NEK11	protein_coding	ENSG00000114670	0,364817952	0,00000215	0,00000921
CYFIP1	protein_coding	ENSG00000273749	-0,145215258	0,00000215	0,00000924
SF3B2	protein_coding	ENSG00000087365	-0,185046949	0,00000217	0,00000932
ZNF474	protein_coding	ENSG00000164185	2,388032621	0,00000218	0,00000935
AL049775.1	lincRNA	ENSG00000205562	0,886983305	0,00000219	0,00000941
CCDC106	protein_coding	ENSG00000173581	-0,33873474	0,00000219	0,00000941
B9D2	protein_coding	ENSG00000123810	-0,478775053	0,00000222	0,00000954
ALLC	protein_coding	ENSG00000151360	4,397337335	0,00000223	0,00000955
YIPF5	protein_coding	ENSG00000145817	0,244727634	0,00000223	0,00000957
PABPN1	protein_coding	ENSG00000100836	-0,378248184	0,00000223	0,00000957
CDH4	protein_coding	ENSG00000179242	0,398155625	0,00000224	0,0000096
SERPINA6	protein_coding	ENSG00000170099	5,755289638	0,00000226	0,00000968
SUCLG2	protein_coding	ENSG00000172340	-0,314304673	0,00000226	0,00000968
SMAD1	protein_coding	ENSG00000170365	-0,343400646	0,00000227	0,00000971
HSD17B1	protein_coding	ENSG00000108786	1,596985482	0,00000227	0,00000973
CYYR1-AS1	antisense	ENSG00000197934	1,486893111	0,00000228	0,00000974
HAT1	protein_coding	ENSG00000128708	-0,37484942	0,00000228	0,00000977
CACNA2D2	protein_coding	ENSG00000007402	4,011274143	0,0000023	0,00000983
PGS1	protein_coding	ENSG00000087157	-0,319493168	0,0000023	0,00000983
ITPRID2	protein_coding	ENSG00000138434	-0,209872065	0,00000233	0,00000996

MMP24	protein_coding	ENSG00000125966	-0,488489072	0,00000234	0,00000998
TNFRSF1A	protein_coding	ENSG00000067182	-0,234546128	0,00000234	0,00000999
C15orf61	protein_coding	ENSG00000189227	-0,44605712	0,00000234	0,00000999
HLA-H	unprocessed_pseudoge	ENSG00000206341	0,745570237	0,00000234	0,00001
TUBA4B	protein_coding	ENSG00000243910	5,737730064	0,00000236	0,0000101
PSME3	protein_coding	ENSG00000131467	-0,215092003	0,00000236	0,0000101
PRDX3	protein_coding	ENSG00000165672	-0,296992664	0,00000238	0,0000101
PPP1R14B	protein_coding	ENSG00000173457	-0,281533731	0,00000238	0,0000102
ADNP	protein_coding	ENSG00000101126	-0,298721096	0,0000024	0,0000102
HMGB1P5	transcribed_processed_	ENSG00000132967	-0,459602603	0,0000024	0,0000102
AC135279.3	antisense	ENSG00000277895	1,739444129	0,00000242	0,0000103
CIC	protein_coding	ENSG00000079432	0,488279767	0,00000243	0,0000103
POMGNT1	protein_coding	ENSG00000085998	-0,207131212	0,00000242	0,0000103
HAUS5	protein_coding	ENSG00000249115	-0,310619653	0,0000024	0,0000103
GPATCH1	protein_coding	ENSG00000076650	-0,318386064	0,00000242	0,0000103
ABCG4	protein_coding	ENSG00000172350	1,861532877	0,00000244	0,0000104
CDK4	protein_coding	ENSG00000135446	-0,273878683	0,00000243	0,0000104
AC005264.1	antisense	ENSG00000267551	1,423100852	0,00000246	0,0000105
RNLS	protein_coding	ENSG00000184719	0,471099735	0,00000247	0,0000105
SRRD	protein_coding	ENSG00000100104	-0,24443179	0,00000247	0,0000105
BBS2	protein_coding	ENSG00000125124	-0,262869497	0,00000247	0,0000105
MPHOSPH10	protein_coding	ENSG00000124383	-0,351198654	0,00000246	0,0000105
MLLT1	protein_coding	ENSG00000130382	-0,356690308	0,00000246	0,0000105
ARHGEF18	protein_coding	ENSG00000104880	0,446439236	0,00000249	0,0000106
C3orf14	protein_coding	ENSG00000114405	-0,381525798	0,0000025	0,0000106
BMS1P1	transcribed_unprocesse	ENSG00000204177	0,954230746	0,00000248	0,0000106
CCDC103	protein_coding	ENSG00000167131	3,883060159	0,00000252	0,0000107
OTULIN	protein_coding	ENSG00000154124	-0,237409975	0,00000252	0,0000107
NUDT8	protein_coding	ENSG00000167799	-0,510413514	0,00000252	0,0000107
FCRLA	protein_coding	ENSG00000132185	2,979717782	0,00000255	0,0000108
WDR48	protein_coding	ENSG00000114742	-0,210529822	0,00000254	0,0000108
FUT10	protein_coding	ENSG00000172728	-0,379906311	0,00000254	0,0000108
DLX2	protein_coding	ENSG00000115844	1,041237886	0,00000256	0,0000109
MYSM1	protein_coding	ENSG00000162601	0,330544519	0,00000256	0,0000109
TXNRD3	protein_coding	ENSG00000197763	-0,303705161	0,00000258	0,0000109
TXNRD3	protein_coding	ENSG00000197763	-0,303705161	0,00000258	0,0000109
NDRG4	protein_coding	ENSG00000103034	3,977088895	0,00000258	0,000011
PPP1R3C	protein_coding	ENSG00000119938	0,332458433	0,0000026	0,000011
CHCHD6	protein_coding	ENSG00000159685	-0,262151045	0,0000026	0,000011
NARS2	protein_coding	ENSG00000137513	-0,280309806	0,0000026	0,000011

MORF4L1	protein_coding	ENSG00000185787	-0,305644099	0,0000026	0,000011
EEF1AKMT2	protein_coding	ENSG00000203791	-0,535976471	0,00000259	0,000011
SLC18B1	protein_coding	ENSG00000146409	-0,544586881	0,00000259	0,000011
MGRN1	protein_coding	ENSG00000102858	0,375486278	0,00000261	0,0000111
PRMT2	protein_coding	ENSG00000160310	-0,188446163	0,00000263	0,0000111
IGF2BP2	protein_coding	ENSG00000073792	-0,210235892	0,00000263	0,0000111
TK1	protein_coding	ENSG00000167900	-0,29959268	0,00000263	0,0000111
AC092910.3	lincRNA	ENSG00000242622	0,699214717	0,00000266	0,0000112
TBC1D10A	protein_coding	ENSG00000099992	0,499476703	0,00000265	0,0000112
MOB3C	protein_coding	ENSG00000142961	0,383459588	0,00000265	0,0000112
LINC01173	lincRNA	ENSG00000280744	1,833269059	0,00000268	0,0000113
WDR25	protein_coding	ENSG00000176473	0,566962652	0,00000268	0,0000113
NABP2	protein_coding	ENSG00000139579	-0,272034597	0,00000268	0,0000113
SRD5A3	protein_coding	ENSG00000128039	-0,281582121	0,00000267	0,0000113
GLRX2	protein_coding	ENSG00000023572	0,350979395	0,00000272	0,0000115
KLHDC7B	protein_coding	ENSG00000130487	4,297576649	0,00000275	0,0000116
SGPL1	protein_coding	ENSG00000166224	-0,209642014	0,00000274	0,0000116
PEMT	protein_coding	ENSG00000133027	-0,288012086	0,00000274	0,0000116
USP46	protein_coding	ENSG00000109189	-0,296891728	0,00000275	0,0000116
RBM26-AS1	antisense	ENSG00000227354	0,814264233	0,00000277	0,0000117
C19orf18	protein_coding	ENSG00000177025	1,059547698	0,00000278	0,0000117
EMP1	protein_coding	ENSG00000134531	-0,17012212	0,00000277	0,0000117
CARHSP1	protein_coding	ENSG00000153048	-0,320253104	0,00000277	0,0000117
LGALS1	protein_coding	ENSG00000100097	-0,329195333	0,00000278	0,0000117
EPB41	protein_coding	ENSG00000159023	0,256672542	0,0000028	0,0000118
EFCAB14	protein_coding	ENSG00000159658	-0,288269468	0,00000281	0,0000118
MCM8	protein_coding	ENSG00000125885	-0,330818013	0,00000281	0,0000118
NFIC	protein_coding	ENSG00000141905	-0,463527035	0,00000279	0,0000118
LINC01687	lincRNA	ENSG00000233215	1,639319271	0,00000283	0,0000119
ACYP2	protein_coding	ENSG00000170634	0,577732371	0,00000283	0,0000119
TMEM44	protein_coding	ENSG00000145014	-0,277172883	0,00000283	0,0000119
DNMT1	protein_coding	ENSG00000130816	-0,33509246	0,00000283	0,0000119
MAP2K7	protein_coding	ENSG00000076984	-0,357515517	0,00000283	0,0000119
NHP2	protein_coding	ENSG00000145912	-0,333340296	0,00000286	0,000012
SDK2	protein_coding	ENSG00000069188	2,666609538	0,0000029	0,0000122
SLC26A4	protein_coding	ENSG00000091137	0,731823218	0,00000289	0,0000122
MAP3K1	protein_coding	ENSG00000095015	0,263137176	0,00000291	0,0000122
SMCO2	protein_coding	ENSG00000165935	1,415753936	0,00000292	0,0000123
SYT14	protein_coding	ENSG00000143469	1,070345787	0,00000292	0,0000123
LRCH1	protein_coding	ENSG00000136141	0,27540446	0,00000292	0,0000123

USP39	protein_coding	ENSG00000168883	-0,226532603	0,00000293	0,0000123
AC243965.2	antisense	ENSG00000275552	3,000334039	0,00000296	0,0000124
CCDC84	protein_coding	ENSG00000186166	0,445944579	0,00000294	0,0000124
UQCRFS1	protein_coding	ENSG00000169021	-0,299336966	0,00000295	0,0000124
FAM227B	protein_coding	ENSG00000166262	0,684189222	0,00000297	0,0000125
CNNM3	protein_coding	ENSG00000168763	-0,43826324	0,00000299	0,0000126
KMT5C	protein_coding	ENSG00000133247	-0,592396392	0,000003	0,0000126
GALNT15	protein_coding	ENSG00000131386	3,208591629	0,00000304	0,0000127
RALGAPA1	protein_coding	ENSG00000174373	0,314700196	0,00000303	0,0000127
RPS10	protein_coding	ENSG00000124614	-0,626258921	0,00000303	0,0000127
AC092687.3	lincRNA	ENSG00000272275	1,475339719	0,00000305	0,0000128
C10orf67	protein_coding	ENSG00000179133	1,075960356	0,00000304	0,0000128
DNMT3B	protein_coding	ENSG00000088305	-0,367297151	0,00000304	0,0000128
ALG6	protein_coding	ENSG00000088035	-0,469053452	0,00000305	0,0000128
LINC01605	lincRNA	ENSG00000253161	-0,31236283	0,0000031	0,000013
ERICD	lincRNA	ENSG00000280303	1,674102151	0,00000314	0,0000131
DCAF13	protein_coding	ENSG00000164934	-0,317065217	0,00000313	0,0000131
RARS	protein_coding	ENSG00000113643	-0,255246828	0,00000315	0,0000132
TMEM237	protein_coding	ENSG00000155755	-0,331571426	0,00000318	0,0000133
CLCA3P	transcribed_unprocesse	ENSG00000153923	1,077317826	0,00000319	0,0000133
MKX	protein_coding	ENSG00000150051	0,540605315	0,00000321	0,0000134
ANKRD52	protein_coding	ENSG00000139645	-0,397159532	0,00000319	0,0000134
MSRA	protein_coding	ENSG00000175806	0,382153143	0,00000322	0,0000135
NDUFS2	protein_coding	ENSG00000158864	-0,204397291	0,00000324	0,0000135
PPIL1	protein_coding	ENSG00000137168	-0,359538591	0,00000323	0,0000135
NAA25	protein_coding	ENSG00000111300	-0,209523071	0,00000329	0,0000138
SETMAR	protein_coding	ENSG00000170364	-0,403653703	0,0000033	0,0000138
ARPC2	protein_coding	ENSG00000163466	0,204676898	0,00000331	0,0000139
SMARCB1	protein_coding	ENSG00000099956	-0,192183903	0,00000332	0,0000139
ROR2	protein_coding	ENSG00000169071	-0,512285412	0,00000333	0,0000139
DACT3	protein_coding	ENSG00000197380	0,988419716	0,00000336	0,000014
C9orf85	protein_coding	ENSG00000155621	0,424938353	0,00000335	0,000014
C4orf33	protein_coding	ENSG00000151470	0,390184739	0,00000334	0,000014
ACAT1	protein_coding	ENSG00000075239	-0,197414224	0,00000336	0,000014
SLC7A11-AS1	processed_transcript	ENSG00000250033	2,662630334	0,00000338	0,0000141
GDF15	protein_coding	ENSG00000130513	0,333402501	0,00000337	0,0000141
VAPA	protein_coding	ENSG00000101558	-0,286470243	0,00000337	0,0000141
PLEKHM1P1	transcribed_unprocesse	ENSG00000214176	0,56368925	0,00000338	0,0000141
APOD	protein_coding	ENSG00000189058	2,540078924	0,00000341	0,0000142
IRF5	protein_coding	ENSG00000128604	1,922625774	0,0000034	0,0000142

CLPTM1	protein_coding	ENSG00000104853	0,237809941	0,00000339	0,0000142
ARHGAP26	protein_coding	ENSG00000145819	0,58750401	0,00000344	0,0000143
MKI67	protein_coding	ENSG00000148773	-0,368867545	0,00000342	0,0000143
SLC38A4	protein_coding	ENSG00000139209	1,997026746	0,00000346	0,0000144
CDKL5	protein_coding	ENSG00000008086	0,422514796	0,00000344	0,0000144
PICALM	protein_coding	ENSG00000073921	0,18349862	0,00000347	0,0000145
NRARP	protein_coding	ENSG00000198435	-1,318791313	0,00000349	0,0000145
GPR85	protein_coding	ENSG00000164604	0,60025202	0,00000353	0,0000147
SPDL1	protein_coding	ENSG00000040275	-0,284434728	0,00000353	0,0000147
CDK15	protein_coding	ENSG00000138395	-0,406364161	0,00000356	0,0000148
NPEPPS	protein_coding	ENSG00000141279	0,192590506	0,00000357	0,0000149
ENDOG	protein_coding	ENSG00000167136	-0,486299651	0,00000357	0,0000149
AC010776.2	antisense	ENSG00000267774	1,75255202	0,00000362	0,000015
LINC01844	lincRNA	ENSG00000236714	5,622223789	0,00000362	0,000015
METTL16	protein_coding	ENSG00000127804	-0,20018829	0,00000362	0,000015
MEGF9	protein_coding	ENSG00000106780	-0,431988061	0,00000361	0,000015
GTF2IP20	transcribed_unprocesse	ENSG00000272645	0,734257198	0,0000036	0,000015
AC002378.1	antisense	ENSG00000225676	2,984587741	0,00000365	0,0000151
TRPM1	protein_coding	ENSG00000134160	3,794084422	0,00000364	0,0000151
SOWAHD	protein_coding	ENSG00000187808	-2,3180643	0,00000364	0,0000151
AC092943.1	processed_pseudogene	ENSG00000243116	2,620278753	0,00000366	0,0000152
FLRT1	protein_coding	ENSG00000126500	5,707372059	0,00000367	0,0000152
SRRM3	protein_coding	ENSG00000177679	-0,653161547	0,00000365	0,0000152
AC116535.1	antisense	ENSG00000246308	1,578949252	0,00000372	0,0000154
FBXL20	protein_coding	ENSG00000108306	0,28282227	0,00000371	0,0000154
METTL4	protein_coding	ENSG00000101574	-0,35500354	0,00000372	0,0000154
GREB1L	protein_coding	ENSG00000141449	-0,364725536	0,00000373	0,0000155
ITSN2	protein_coding	ENSG00000198399	0,224114063	0,00000376	0,0000156
PHF14	protein_coding	ENSG00000106443	-0,291643764	0,00000375	0,0000156
L34079.3	antisense	ENSG00000269583	4,360524542	0,00000379	0,0000157
WDR74	protein_coding	ENSG00000133316	0,242718596	0,00000378	0,0000157
PPFIA1	protein_coding	ENSG00000131626	-0,325799876	0,00000379	0,0000157
SEMA4D	protein_coding	ENSG00000187764	-0,54972313	0,00000379	0,0000157
CERS6	protein_coding	ENSG00000172292	0,21880647	0,00000381	0,0000158
AHCTF1	protein_coding	ENSG00000153207	-0,225552862	0,00000381	0,0000158
NME4	protein_coding	ENSG00000103202	-0,316715725	0,00000382	0,0000158
COL3A1	protein_coding	ENSG00000168542	1,237825041	0,00000384	0,0000159
TENM3	protein_coding	ENSG00000218336	0,327278068	0,00000385	0,0000159
TIMM21	protein_coding	ENSG00000075336	-0,398503222	0,00000383	0,0000159
NBPF11	protein_coding	ENSG00000263956	0,519249102	0,00000386	0,000016

LRRC37A2	protein_coding	ENSG00000238083	0,48298767	0,00000387	0,000016
MRPS22	protein_coding	ENSG00000175110	-0,322797263	0,00000386	0,000016
GEMIN2	protein_coding	ENSG00000092208	-0,389792959	0,00000387	0,000016
AL390719.1	transcribed_unprocesse	ENSG00000217801	0,855541681	0,0000039	0,0000161
ZNF823	protein_coding	ENSG00000197933	-0,515685022	0,00000402	0,0000166
STARD4-AS1	antisense	ENSG00000246859	1,419366409	0,00000405	0,0000167
HAP1	protein_coding	ENSG00000173805	1,636130914	0,00000406	0,0000168
LRRC8D	protein_coding	ENSG00000171492	-0,226891619	0,00000407	0,0000168
ENAM	protein_coding	ENSG00000132464	3,414017634	0,00000409	0,0000169
SLC35D1	protein_coding	ENSG00000116704	0,218800384	0,00000409	0,0000169
PNISR	protein_coding	ENSG00000132424	-0,326939564	0,0000041	0,0000169
VAV2	protein_coding	ENSG00000160293	-0,357463946	0,0000041	0,0000169
PECR	protein_coding	ENSG00000115425	-0,421000125	0,00000412	0,000017
ZBTB6	protein_coding	ENSG00000186130	0,322262301	0,00000414	0,0000171
HIVEP3	protein_coding	ENSG00000127124	0,570854749	0,00000417	0,0000172
UBE2S	protein_coding	ENSG00000108106	-0,348770779	0,00000417	0,0000172
AC090673.1	antisense	ENSG00000256083	1,811995092	0,00000419	0,0000173
ABCA9	protein_coding	ENSG00000154258	5,612871692	0,00000421	0,0000173
NOL11	protein_coding	ENSG00000130935	-0,247766901	0,00000418	0,0000173
WDR19	protein_coding	ENSG00000157796	-0,373304255	0,00000419	0,0000173
PAMR1	protein_coding	ENSG00000149090	5,598395422	0,00000423	0,0000174
MYBPHL	protein_coding	ENSG00000221986	2,161581149	0,00000422	0,0000174
TSPAN5	protein_coding	ENSG00000168785	-0,198486535	0,00000423	0,0000174
MYLK	protein_coding	ENSG00000065534	-0,267493552	0,00000423	0,0000174
URB1	protein_coding	ENSG00000142207	-0,343115777	0,00000424	0,0000174
TWIST1	protein_coding	ENSG00000122691	-0,58218676	0,00000422	0,0000174
AC245041.2	lincRNA	ENSG00000276850	0,813209945	0,00000424	0,0000175
NCOA4	protein_coding	ENSG00000266412	0,260008344	0,00000425	0,0000175
CNPY4	protein_coding	ENSG00000166997	-0,507523031	0,0000043	0,0000177
LTF	protein_coding	ENSG00000012223	2,038914695	0,00000434	0,0000178
CMTM7	protein_coding	ENSG00000153551	-0,315565029	0,00000434	0,0000178
ABCC5	protein_coding	ENSG00000114770	-0,34244445	0,00000432	0,0000178
ATE1-AS1	transcribed_unitary_pse	ENSG00000226864	2,203300209	0,00000433	0,0000178
DHRS9	protein_coding	ENSG00000073737	3,146115155	0,00000437	0,0000179
TNFRSF14	protein_coding	ENSG00000157873	0,550887893	0,00000435	0,0000179
PRMT3	protein_coding	ENSG00000185238	-0,321222521	0,00000435	0,0000179
C18orf54	protein_coding	ENSG00000166845	-0,350183404	0,00000437	0,0000179
ADORA1	protein_coding	ENSG00000163485	-0,523029115	0,00000434	0,0000179
AL137003.2	lincRNA	ENSG00000272341	0,755512846	0,00000439	0,000018
RAD23B	protein_coding	ENSG00000119318	-0,184119501	0,00000439	0,000018

ISL1	protein_coding	ENSG00000016082	0,50219146	0,00000445	0,0000182
SIL1	protein_coding	ENSG00000120725	0,294140527	0,00000445	0,0000182
DAB2	protein_coding	ENSG00000153071	0,205108723	0,00000444	0,0000182
AGAP1	protein_coding	ENSG00000157985	-0,267301175	0,00000443	0,0000182
EPHA10	protein_coding	ENSG00000183317	-0,753170323	0,00000443	0,0000182
AL590327.1	protein_coding	ENSG00000179627	-0,923690632	0,00000443	0,0000182
PGM2L1	protein_coding	ENSG00000165434	0,504939089	0,00000448	0,0000183
GANC	protein_coding	ENSG00000214013	0,288625888	0,00000447	0,0000183
EXOSC8	protein_coding	ENSG00000120699	-0,32791414	0,00000445	0,0000183
ZNF668	protein_coding	ENSG00000167394	-0,401776278	0,00000447	0,0000183
CFAP45	protein_coding	ENSG00000213085	0,858529319	0,0000045	0,0000184
THADA	protein_coding	ENSG00000115970	-0,195873622	0,0000045	0,0000184
NHSL1	protein_coding	ENSG00000135540	0,508905828	0,00000453	0,0000185
AC139795.2	antisense	ENSG00000247679	1,535420555	0,00000456	0,0000186
AC016924.1	lincRNA	ENSG00000250934	4,288368967	0,00000456	0,0000186
BNIP3	protein_coding	ENSG00000176171	-0,255034944	0,00000455	0,0000186
KRIT1	protein_coding	ENSG00000001631	-0,287471682	0,00000456	0,0000186
CAB39	protein_coding	ENSG00000135932	0,209453214	0,0000046	0,0000188
UCN2	protein_coding	ENSG00000145040	3,020146091	0,00000461	0,0000189
IGSF11	protein_coding	ENSG00000144847	2,531104583	0,00000461	0,0000189
BLK	protein_coding	ENSG00000136573	1,578471043	0,00000463	0,0000189
MED29	protein_coding	ENSG00000063322	0,276216992	0,00000461	0,0000189
HCFC1	protein_coding	ENSG00000172534	-0,434271523	0,00000461	0,0000189
DUSP1	protein_coding	ENSG00000120129	0,25342636	0,00000465	0,000019
COPZ1	protein_coding	ENSG00000111481	-0,218675173	0,00000464	0,000019
HNRNPUL1	protein_coding	ENSG00000105323	-0,223351817	0,00000466	0,000019
RAI14	protein_coding	ENSG00000039560	-0,237096089	0,00000465	0,000019
NUTF2	protein_coding	ENSG00000102898	-0,22521644	0,00000469	0,0000191
SRSF8	protein_coding	ENSG00000263465	-0,249914082	0,00000471	0,0000192
KIF24	protein_coding	ENSG00000186638	-0,407037997	0,0000047	0,0000192
LINC01524	lincRNA	ENSG00000234948	2,311553699	0,00000473	0,0000193
COL26A1	protein_coding	ENSG00000160963	3,92385657	0,00000473	0,0000193
SNCG	protein_coding	ENSG00000173267	1,299543212	0,00000473	0,0000193
GDI1	protein_coding	ENSG00000203879	0,195211643	0,00000474	0,0000193
TMEM185B	protein_coding	ENSG00000226479	-0,228265676	0,00000477	0,0000194
FKBP2	protein_coding	ENSG00000173486	0,338778436	0,00000478	0,0000195
ZC3H14	protein_coding	ENSG00000100722	-0,247687282	0,00000479	0,0000195
PYCR1	protein_coding	ENSG00000183010	-0,289191722	0,00000479	0,0000195
DMAC1	protein_coding	ENSG00000137038	-0,331743239	0,00000478	0,0000195
MAML1	protein_coding	ENSG00000161021	-0,358394974	0,00000477	0,0000195

KRT8	protein_coding	ENSG00000170421	-0,256363151	0,00000481	0,0000196
PDF	protein_coding	ENSG00000258429	-0,771688543	0,00000482	0,0000196
CBX7	protein_coding	ENSG00000100307	-0,448643775	0,00000485	0,0000197
MIR99AHG	lincRNA	ENSG00000215386	0,900231659	0,00000492	0,00002
FOXP1	protein_coding	ENSG00000114861	0,22150833	0,00000495	0,0000201
AC010735.2	lincRNA	ENSG00000272622	1,9359394	0,00000496	0,0000202
HHIPL1	protein_coding	ENSG00000182218	4,261418791	0,00000496	0,0000202
RFPL4A	protein_coding	ENSG00000223638	3,391602622	0,00000497	0,0000202
HERC4	protein_coding	ENSG00000148634	-0,177346052	0,00000496	0,0000202
DRG1	protein_coding	ENSG00000185721	-0,291688869	0,00000496	0,0000202
RTL8A	protein_coding	ENSG00000203950	-0,323299378	0,00000496	0,0000202
PLXNA2	protein_coding	ENSG00000076356	0,441587065	0,000005	0,0000203
SERTAD2	protein_coding	ENSG00000179833	0,272799372	0,000005	0,0000203
C16orf72	protein_coding	ENSG00000182831	0,228558025	0,00000499	0,0000203
AL078612.2	processed_transcript	ENSG00000255375	1,842994209	0,00000505	0,0000205
SERINC4	protein_coding	ENSG00000184716	2,276667491	0,00000505	0,0000205
STEAP3	protein_coding	ENSG00000115107	-0,239873115	0,00000504	0,0000205
MFSD5	protein_coding	ENSG00000182544	-0,305007257	0,00000504	0,0000205
LINC02298	lincRNA	ENSG00000257556	0,748945079	0,00000508	0,0000206
TET2	protein_coding	ENSG00000168769	0,297084524	0,00000507	0,0000206
ACAD9	protein_coding	ENSG00000177646	-0,171066388	0,00000508	0,0000206
RNF4	protein_coding	ENSG00000063978	-0,233762961	0,00000507	0,0000206
SPAG5-AS1	processed_transcript	ENSG00000227543	1,486165852	0,0000051	0,0000207
MTTP	protein_coding	ENSG00000138823	2,730144493	0,00000511	0,0000207
MYO7B	protein_coding	ENSG00000169994	2,520617797	0,00000511	0,0000207
SERPINF2	protein_coding	ENSG00000167711	2,372073167	0,00000511	0,0000207
ESYT2	protein_coding	ENSG00000117868	0,167701848	0,00000515	0,0000208
CLN6	protein_coding	ENSG00000128973	-0,406396282	0,00000519	0,000021
LINC01633	lincRNA	ENSG00000260976	5,716947676	0,00000522	0,0000211
PPP1R2	protein_coding	ENSG00000184203	0,284140068	0,00000522	0,0000211
TTC39A	protein_coding	ENSG00000085831	2,718808372	0,00000523	0,0000212
CLEC12B	protein_coding	ENSG00000256660	1,117463984	0,00000525	0,0000212
SPATA33	protein_coding	ENSG00000167523	-0,473205035	0,00000523	0,0000212
SDSL	protein_coding	ENSG00000139410	0,560586512	0,00000526	0,0000213
VPS9D1	protein_coding	ENSG00000075399	0,555984104	0,00000527	0,0000213
RNF25	protein_coding	ENSG00000163481	0,3082509	0,0000053	0,0000214
PTCH2	protein_coding	ENSG00000117425	-1,46675559	0,0000053	0,0000214
ABT1	protein_coding	ENSG00000146109	-0,297387393	0,00000532	0,0000215
UBE2Q2	protein_coding	ENSG00000140367	-0,300708091	0,00000531	0,0000215
RNF122	protein_coding	ENSG00000133874	-0,516919271	0,00000531	0,0000215

IDH2	protein_coding	ENSG00000182054	-0,227014702	0,00000535	0,0000216
GGA2	protein_coding	ENSG00000103365	-0,283242683	0,00000535	0,0000216
TTC12	protein_coding	ENSG00000149292	-0,364558669	0,00000536	0,0000216
WWTR1-AS1	antisense	ENSG00000241313	0,958594843	0,00000537	0,0000217
FOXA1	protein_coding	ENSG00000129514	-0,68519584	0,00000537	0,0000217
LURAP1L-AS1	antisense	ENSG00000235448	0,750865022	0,00000541	0,0000218
TRIM44	protein_coding	ENSG00000166326	0,227870394	0,0000054	0,0000218
DDX31	protein_coding	ENSG00000125485	-0,232241348	0,0000054	0,0000218
PLCG1	protein_coding	ENSG00000124181	-0,292404829	0,00000541	0,0000218
ZNF644	protein_coding	ENSG00000122482	0,266226193	0,00000544	0,0000219
SUGT1	protein_coding	ENSG00000165416	-0,260062989	0,00000543	0,0000219
TARBP2	protein_coding	ENSG00000139546	-0,344292147	0,00000542	0,0000219
GPR132	protein_coding	ENSG00000183484	2,737961951	0,00000546	0,000022
MAPK14	protein_coding	ENSG00000112062	-0,172767622	0,00000547	0,000022
RPUSD3	protein_coding	ENSG00000156990	-0,27957191	0,00000548	0,0000221
RITA1	protein_coding	ENSG00000139405	-0,309936298	0,0000055	0,0000221
FBXL14	protein_coding	ENSG00000171823	-0,450897064	0,0000055	0,0000221
PDGFRB	protein_coding	ENSG00000113721	0,888252644	0,0000055	0,0000222
IL17RC	protein_coding	ENSG00000163702	-0,30868	0,00000553	0,0000222
SPINK6	protein_coding	ENSG00000178172	-0,53879878	0,00000552	0,0000222
NRCAM	protein_coding	ENSG00000091129	2,235440954	0,00000554	0,0000223
IDNK	protein_coding	ENSG00000148057	0,830462208	0,00000553	0,0000223
SLC25A44	protein_coding	ENSG00000160785	0,258117637	0,00000554	0,0000223
TBC1D10B	protein_coding	ENSG00000169221	-0,329546019	0,00000554	0,0000223
C1QTNF1-AS1	antisense	ENSG00000265096	5,55978348	0,00000557	0,0000224
KLHL26	protein_coding	ENSG00000167487	0,541345716	0,00000561	0,0000225
CWC15	protein_coding	ENSG00000150316	-0,332678997	0,0000056	0,0000225
TRIM63	protein_coding	ENSG00000158022	0,665223107	0,00000563	0,0000226
WRB	protein_coding	ENSG00000182093	-0,295118325	0,00000565	0,0000227
ULBP1	protein_coding	ENSG00000111981	0,647906126	0,00000567	0,0000228
RYR1	protein_coding	ENSG00000196218	4,222545696	0,00000574	0,000023
BEST4	protein_coding	ENSG00000142959	3,290616901	0,00000576	0,0000231
TMEM138	protein_coding	ENSG00000149483	-0,283472387	0,00000576	0,0000231
CLSPN	protein_coding	ENSG00000092853	-0,378945668	0,00000577	0,0000231
AR	protein_coding	ENSG00000169083	-0,577299529	0,00000575	0,0000231
SPAG7	protein_coding	ENSG00000091640	-0,222136707	0,00000578	0,0000232
SNRNP25	protein_coding	ENSG00000161981	-0,272754473	0,00000579	0,0000232
DDX28	protein_coding	ENSG00000182810	-0,337934571	0,00000578	0,0000232
GTF2H2C	protein_coding	ENSG00000183474	-0,364062789	0,00000578	0,0000232
NIN	protein_coding	ENSG00000100503	-0,379801063	0,00000579	0,0000232

ZNF84	protein_coding	ENSG00000198040	-0,297793533	0,00000582	0,0000233
LINC00672	protein_coding	ENSG00000263874	1,000161924	0,00000584	0,0000234
DAGLA	protein_coding	ENSG00000134780	-0,73144168	0,00000583	0,0000234
PTPN13	protein_coding	ENSG00000163629	-0,253636966	0,00000586	0,0000235
AL590226.2	lincRNA	ENSG00000279141	5,574503585	0,0000059	0,0000236
SEPT2	protein_coding	ENSG00000168385	-0,233416874	0,00000591	0,0000236
WNT4	protein_coding	ENSG00000162552	2,373948661	0,00000592	0,0000237
BFAR	protein_coding	ENSG00000103429	-0,222905243	0,00000591	0,0000237
APP	protein_coding	ENSG00000142192	-0,168747115	0,00000597	0,0000239
TEFM	protein_coding	ENSG00000172171	-0,369666817	0,00000596	0,0000239
AL133163.2	lincRNA	ENSG00000258860	3,863707743	0,00000599	0,000024
MEP1A	protein_coding	ENSG00000112818	3,825732984	0,000006	0,000024
PLEKHM2	protein_coding	ENSG00000116786	0,297673239	0,000006	0,000024
LARP1B	protein_coding	ENSG00000138709	0,297650168	0,00000599	0,000024
SAMHD1	protein_coding	ENSG00000101347	0,1993367	0,00000603	0,0000241
CPA2	protein_coding	ENSG00000158516	5,701958324	0,00000605	0,0000242
RRNAD1	protein_coding	ENSG00000143303	0,516219473	0,0000061	0,0000244
TEAD3	protein_coding	ENSG00000007866	0,442690535	0,00000618	0,0000247
CD160	protein_coding	ENSG00000117281	2,834284845	0,0000062	0,0000248
RIF1	protein_coding	ENSG00000080345	-0,323110947	0,00000622	0,0000248
VPS11	protein_coding	ENSG00000160695	0,219731069	0,00000623	0,0000249
SLC6A13	protein_coding	ENSG00000010379	2,843801059	0,00000627	0,000025
METTL14	protein_coding	ENSG00000145388	-0,280660287	0,00000627	0,000025
RAPGEF5	protein_coding	ENSG00000136237	-0,49879914	0,00000626	0,000025
POLR3H	protein_coding	ENSG00000100413	-0,24024954	0,00000631	0,0000252
MYO5C	protein_coding	ENSG00000128833	1,222954759	0,00000634	0,0000253
MAGI3	protein_coding	ENSG00000081026	-0,253879381	0,00000636	0,0000253
MRFAP1L1	protein_coding	ENSG00000178988	-0,248400351	0,00000638	0,0000254
MFHAS1	protein_coding	ENSG00000147324	-0,365777059	0,00000638	0,0000254
AC090023.2	lincRNA	ENSG00000256915	3,819626118	0,00000647	0,0000257
AC004475.1	protein_coding	ENSG00000105705	0,240819889	0,00000644	0,0000257
TGOLN2	protein_coding	ENSG00000152291	-0,253377667	0,00000645	0,0000257
PLEKHA8	protein_coding	ENSG00000106086	-0,280869768	0,00000646	0,0000257
RPA3	protein_coding	ENSG00000106399	-0,359195697	0,00000648	0,0000258
SFI1	protein_coding	ENSG00000198089	-0,343351484	0,00000659	0,0000262
AP000619.1	transcribed_unprocesse	ENSG00000225678	4,128305632	0,00000658	0,0000262
FAM104B	protein_coding	ENSG00000182518	-0,40968566	0,00000661	0,0000263
PGAM1	protein_coding	ENSG00000171314	-0,240229271	0,00000666	0,0000265
PSG9	protein_coding	ENSG00000183668	1,132884004	0,00000669	0,0000266
LIN54	protein_coding	ENSG00000189308	0,246767591	0,00000669	0,0000266

GKN1	protein_coding	ENSG00000169605	1,895124953	0,0000067	0,0000267
CARNMT1	protein_coding	ENSG00000156017	-0,360498163	0,00000672	0,0000267
AC037198.1	sense_intronic	ENSG00000276107	-0,764474573	0,00000672	0,0000267
AC079949.2	lincRNA	ENSG00000278266	-0,812636314	0,00000675	0,0000268
GRIA4	protein_coding	ENSG00000152578	5,558193147	0,00000676	0,0000268
RELA	protein_coding	ENSG00000173039	0,308158502	0,00000676	0,0000268
CALR4P	transcribed_unitary_pse	ENSG00000227742	1,986000961	0,00000674	0,0000268
ZNF24	protein_coding	ENSG00000172466	0,252361062	0,00000683	0,0000271
RABEPK	protein_coding	ENSG00000136933	-0,298549313	0,00000684	0,0000271
IDE	protein_coding	ENSG00000119912	0,221748367	0,00000684	0,0000272
ZNF552	protein_coding	ENSG00000178935	-0,606156407	0,00000685	0,0000272
LINC01137	antisense	ENSG00000233621	0,688680855	0,00000689	0,0000273
STK17B	protein_coding	ENSG00000081320	0,29304848	0,00000691	0,0000274
FBXO36	protein_coding	ENSG00000153832	-0,593929112	0,0000069	0,0000274
AL589935.1	processed_transcript	ENSG00000232295	3,678228582	0,00000695	0,0000275
HMGN1	protein_coding	ENSG00000205581	-0,245857548	0,00000694	0,0000275
ATAD2	protein_coding	ENSG00000156802	-0,30265369	0,00000701	0,0000278
RUSC1	protein_coding	ENSG00000160753	-0,358967456	0,00000702	0,0000278
PABPC4	protein_coding	ENSG00000090621	0,186867189	0,00000705	0,0000279
C5orf24	protein_coding	ENSG00000181904	-0,347088224	0,00000706	0,0000279
SNORD4A	snoRNA	ENSG00000238578	-0,776389815	0,00000704	0,0000279
TPI1P2	transcribed_processed_	ENSG00000230359	0,865287256	0,00000704	0,0000279
AC145207.5	lincRNA	ENSG00000263731	-0,471088485	0,0000071	0,0000281
METTL21A	protein_coding	ENSG00000144401	-0,232349884	0,00000709	0,0000281
PSMB5	protein_coding	ENSG00000100804	-0,253569779	0,0000071	0,0000281
ZNF846	protein_coding	ENSG00000196605	0,622033909	0,00000714	0,0000283
TMEM229B	protein_coding	ENSG00000198133	0,975104036	0,00000717	0,0000284
APOBEC3B	protein_coding	ENSG00000179750	-0,330875957	0,00000718	0,0000284
PARN	protein_coding	ENSG00000140694	-0,234855893	0,00000721	0,0000285
IQGAP2	protein_coding	ENSG00000145703	1,889528553	0,00000723	0,0000286
CERS2	protein_coding	ENSG00000143418	0,155184377	0,00000723	0,0000286
CLIC1	protein_coding	ENSG00000213719	-0,26271909	0,00000726	0,0000287
RAB44	protein_coding	ENSG00000255587	3,816695723	0,00000728	0,0000288
AL133163.1	processed_pseudogene	ENSG00000240023	5,61399495	0,00000731	0,0000289
HCAR3	protein_coding	ENSG00000255398	5,557281275	0,00000738	0,0000291
PDCD1LG2	protein_coding	ENSG00000197646	1,179913755	0,00000737	0,0000291
TCF7L2	protein_coding	ENSG00000148737	0,415218816	0,00000736	0,0000291
FAM111B	protein_coding	ENSG00000189057	-0,426499761	0,00000737	0,0000291
CCT6B	protein_coding	ENSG00000132141	0,910016288	0,00000746	0,0000294
MYO18A	protein_coding	ENSG00000196535	-0,367061396	0,00000745	0,0000294

RIPPLY2	protein_coding	ENSG00000203877	-0,9578174	0,00000747	0,0000295
CTRB2	protein_coding	ENSG00000168928	5,630554102	0,00000752	0,0000296
C1orf143	lincRNA	ENSG00000228208	2,458041451	0,00000752	0,0000297
SPTBN5	protein_coding	ENSG00000137877	1,530611228	0,00000752	0,0000297
SNRPB	protein_coding	ENSG00000125835	-0,364871563	0,00000752	0,0000297
AC027237.3	antisense	ENSG00000259426	1,011827674	0,00000757	0,0000298
PIK3CB	protein_coding	ENSG00000051382	0,31229918	0,00000757	0,0000298
SP6	protein_coding	ENSG00000189120	1,775216275	0,00000761	0,00003
MPLKIP	protein_coding	ENSG00000168303	-0,290746787	0,00000761	0,00003
AC099329.2	processed_transcript	ENSG00000273328	5,609617915	0,00000765	0,0000301
LAPTM4B	protein_coding	ENSG00000104341	-0,139456277	0,00000764	0,0000301
SOCS4	protein_coding	ENSG00000180008	0,314104649	0,0000077	0,0000303
MAPRE2	protein_coding	ENSG00000166974	0,378728261	0,00000773	0,0000304
CPT1A	protein_coding	ENSG00000110090	-0,211793413	0,00000772	0,0000304
AC098487.1	lincRNA	ENSG00000248161	0,963356925	0,00000775	0,0000305
ATAD3A	protein_coding	ENSG00000197785	-0,34434229	0,00000776	0,0000305
GATA2	protein_coding	ENSG00000179348	-0,348761529	0,00000782	0,0000307
ARL2	protein_coding	ENSG00000213465	-0,357638934	0,0000078	0,0000307
MIR503HG	lincRNA	ENSG00000223749	-0,903524224	0,00000789	0,000031
MMP8	protein_coding	ENSG00000118113	4,081383691	0,00000789	0,000031
NPAS1	protein_coding	ENSG00000130751	1,213967441	0,00000789	0,000031
DUSP22	protein_coding	ENSG00000112679	-0,342121867	0,00000789	0,000031
SIK3	protein_coding	ENSG00000160584	0,342885568	0,00000791	0,0000311
AP5S1	protein_coding	ENSG00000125843	-0,437790107	0,00000791	0,0000311
SMIM8	protein_coding	ENSG00000111850	-0,448595589	0,00000791	0,0000311
INTS5	protein_coding	ENSG00000185085	-0,377362273	0,00000794	0,0000312
KCNN3	protein_coding	ENSG00000143603	4,090790601	0,00000798	0,0000313
CETN3	protein_coding	ENSG00000153140	-0,321575033	0,00000799	0,0000313
CHRNA6	protein_coding	ENSG00000147434	4,216384907	0,00000807	0,0000316
DKK3	protein_coding	ENSG00000050165	0,299770141	0,00000805	0,0000316
SNORD104	snoRNA	ENSG00000199753	-1,002138054	0,00000807	0,0000316
RRP9	protein_coding	ENSG00000114767	-0,270852224	0,0000081	0,0000318
AC022816.1	lincRNA	ENSG00000230647	4,249763845	0,00000815	0,000032
SRPRA	protein_coding	ENSG00000182934	0,24273414	0,00000815	0,000032
PIGO	protein_coding	ENSG00000165282	-0,3464224	0,00000815	0,000032
NAA30	protein_coding	ENSG00000139977	0,204967296	0,00000823	0,0000322
XRN2	protein_coding	ENSG00000088930	-0,213495588	0,00000825	0,0000323
KIF2A	protein_coding	ENSG00000068796	-0,223258873	0,00000826	0,0000324
STAG1	protein_coding	ENSG00000118007	-0,260634398	0,00000828	0,0000324
ANKS6	protein_coding	ENSG00000165138	-0,351774184	0,00000844	0,000033

CEP112	protein_coding	ENSG00000154240	0,343693301	0,00000849	0,0000332
CEP89	protein_coding	ENSG00000121289	-0,227121308	0,00000847	0,0000332
TOP3A	protein_coding	ENSG00000177302	-0,228108641	0,00000847	0,0000332
SPRYD3	protein_coding	ENSG00000167778	-0,330218617	0,0000085	0,0000332
MAPKAPK5	protein_coding	ENSG00000089022	-0,220241389	0,00000854	0,0000334
MRPL11	protein_coding	ENSG00000174547	-0,307425415	0,00000853	0,0000334
BAG4	protein_coding	ENSG00000156735	-0,235411239	0,00000857	0,0000335
TSC22D4	protein_coding	ENSG00000166925	0,427994361	0,00000864	0,0000338
TMEM218	protein_coding	ENSG00000150433	-0,300224126	0,00000865	0,0000338
TRIO	protein_coding	ENSG00000038382	-0,340585055	0,00000865	0,0000338
RNF103	protein_coding	ENSG00000239305	0,320855742	0,00000868	0,0000339
LENG8	protein_coding	ENSG00000167615	-0,431491564	0,00000869	0,0000339
ITPKB	protein_coding	ENSG00000143772	-0,415888308	0,00000872	0,000034
RPL23AP77	processed_pseudogene	ENSG00000267301	3,854935295	0,00000875	0,0000341
IKBKE	protein_coding	ENSG00000263528	0,38725303	0,00000875	0,0000341
SESN1	protein_coding	ENSG00000080546	0,301214412	0,00000874	0,0000341
AL136309.4	antisense	ENSG00000254821	4,109958587	0,00000882	0,0000344
NYAP1	protein_coding	ENSG00000166924	0,912386477	0,00000881	0,0000344
LSM4	protein_coding	ENSG00000130520	-0,272763528	0,00000888	0,0000346
ZKSCAN5	protein_coding	ENSG00000196652	0,251594719	0,0000089	0,0000347
ESM1	protein_coding	ENSG00000164283	0,226149395	0,00000896	0,0000349
LRRC42	protein_coding	ENSG00000116212	-0,23226322	0,00000897	0,000035
AC089983.1	antisense	ENSG00000257732	0,35723052	0,00000902	0,0000351
SEMA3G	protein_coding	ENSG00000010319	1,907587574	0,00000901	0,0000351
TERF2	protein_coding	ENSG00000132604	0,317815184	0,00000901	0,0000351
TRIP11	protein_coding	ENSG00000100815	0,301933684	0,00000901	0,0000351
KYAT1	protein_coding	ENSG00000171097	-0,468333854	0,00000901	0,0000351
GPATCH8	protein_coding	ENSG00000186566	0,27134801	0,00000903	0,0000352
BEND6	protein_coding	ENSG00000151917	0,713086837	0,00000906	0,0000353
TCFL5	protein_coding	ENSG00000101190	-0,282390831	0,00000908	0,0000353
TMEM51-AS1	antisense	ENSG00000175147	1,785670493	0,0000091	0,0000354
EEF1A1	protein_coding	ENSG00000156508	-0,306994719	0,00000911	0,0000354
APOE	protein_coding	ENSG00000130203	1,245873991	0,00000912	0,0000355
CABIN1	protein_coding	ENSG00000099991	-0,320565103	0,00000913	0,0000355
AC009506.1	antisense	ENSG00000224152	1,09064089	0,00000917	0,0000356
RPE	protein_coding	ENSG00000197713	-0,295005367	0,00000916	0,0000356
KCNG1	protein_coding	ENSG00000026559	0,44183219	0,00000926	0,000036
PXYLP1	protein_coding	ENSG00000155893	-0,360419545	0,00000925	0,000036
DEAF1	protein_coding	ENSG00000177030	-0,264643937	0,00000933	0,0000363
TMED7	protein_coding	ENSG00000134970	0,409180724	0,00000947	0,0000368

KCNQ5	protein_coding	ENSG00000185760	0,333252393	0,00000946	0,0000368
PSMD12	protein_coding	ENSG00000197170	0,223758496	0,00000948	0,0000368
FNTB	protein_coding	ENSG00000257365	-0,394016258	0,00000951	0,0000369
FNTB	protein_coding	ENSG00000257365	-0,394016258	0,00000951	0,0000369
GPKOW	protein_coding	ENSG00000068394	-0,225356365	0,00000954	0,000037
AARS2	protein_coding	ENSG00000124608	-0,340106999	0,00000956	0,0000371
KLC1	protein_coding	ENSG00000126214	-0,299763305	0,00000959	0,0000372
ABRAXAS1	protein_coding	ENSG00000163322	-0,496108811	0,00000958	0,0000372
LSR	protein_coding	ENSG00000105699	1,508678164	0,00000961	0,0000373
PUS1	protein_coding	ENSG00000177192	-0,318433164	0,00000965	0,0000374
AC209007.1	processed_pseudogene	ENSG00000215899	5,6347367	0,00000966	0,0000375
SNRPA1	protein_coding	ENSG00000131876	0,242650088	0,00000968	0,0000375
TMEM263	protein_coding	ENSG00000151135	0,279671639	0,0000097	0,0000376
SUMO2	protein_coding	ENSG00000188612	-0,357213186	0,00000971	0,0000376
AC243829.4	antisense	ENSG00000277089	4,162772237	0,00000973	0,0000377
ANK3	protein_coding	ENSG00000151150	0,782971381	0,00000972	0,0000377
UHRF1BP1L	protein_coding	ENSG00000111647	0,231921787	0,0000098	0,0000379
TRIM22	protein_coding	ENSG00000132274	0,537383532	0,00000981	0,000038
EIF3L	protein_coding	ENSG00000100129	-0,173356043	0,00000982	0,000038
ZNF385A	protein_coding	ENSG00000161642	0,975486496	0,00000985	0,0000381
DHPS	protein_coding	ENSG00000095059	-0,250352902	0,00000984	0,0000381
DIMT1	protein_coding	ENSG00000086189	-0,39302523	0,00000985	0,0000381
SNRPF	protein_coding	ENSG00000139343	-0,308661554	0,00000988	0,0000382
ZNF785	protein_coding	ENSG00000197162	-0,474269677	0,00000987	0,0000382
LINC02029	lincRNA	ENSG00000241544	2,616739251	0,00000997	0,0000385
HTR3E	protein_coding	ENSG00000186038	5,400263337	0,00000999	0,0000386
LRRFIP2	protein_coding	ENSG00000093167	0,199661093	0,00001	0,0000387
MARCH10	protein_coding	ENSG00000173838	1,50749717	0,0000101	0,000039
SARS2	protein_coding	ENSG00000104835	-0,750860504	0,0000101	0,000039
MAGOHB	protein_coding	ENSG00000111196	-0,374945772	0,0000101	0,0000391
SLC23A2	protein_coding	ENSG00000089057	-0,333828828	0,0000101	0,0000392
HIST1H1A	protein_coding	ENSG00000124610	3,629325392	0,0000102	0,0000393
STT3B	protein_coding	ENSG00000163527	0,165574949	0,0000102	0,0000394
L3MBTL3	protein_coding	ENSG00000198945	-0,449682892	0,0000102	0,0000395
AL049775.2	lincRNA	ENSG00000258902	1,021796703	0,0000103	0,0000397
UNC5B	protein_coding	ENSG00000107731	2,634429863	0,0000103	0,0000397
SNU13	protein_coding	ENSG00000100138	-0,23327235	0,0000103	0,0000398
TWNK	protein_coding	ENSG00000107815	-0,361646648	0,0000104	0,00004
FAT1	protein_coding	ENSG00000083857	0,33218756	0,0000105	0,0000403
PTPRM	protein_coding	ENSG00000173482	0,354409228	0,0000105	0,0000404

TNPO3	protein_coding	ENSG00000064419	-0,217321815	0,0000105	0,0000405
NCAPG2	protein_coding	ENSG00000146918	-0,264707777	0,0000105	0,0000406
RBX1	protein_coding	ENSG00000100387	-0,307057832	0,0000105	0,0000406
EDARADD	protein_coding	ENSG00000186197	-0,442736431	0,0000105	0,0000406
IQCE	protein_coding	ENSG00000106012	-0,327507077	0,0000106	0,0000409
TMEM223	protein_coding	ENSG00000168569	-0,399454631	0,0000107	0,0000411
ZNF107	protein_coding	ENSG00000196247	-0,457236078	0,0000107	0,0000413
NR4A1	protein_coding	ENSG00000123358	0,474479976	0,0000108	0,0000415
BAIAP2-DT	lincRNA	ENSG00000226137	-0,427623043	0,0000109	0,0000418
CCN2	protein_coding	ENSG00000118523	-0,236825049	0,0000109	0,0000419
GTF2IRD2P1	transcribed_unprocesse	ENSG00000214544	5,467669289	0,000011	0,0000422
OXLD1	protein_coding	ENSG00000204237	-0,354910094	0,000011	0,0000423
DAAM1	protein_coding	ENSG00000100592	0,28764478	0,000011	0,0000425
WDFY3	protein_coding	ENSG00000163625	0,255556238	0,0000112	0,000043
CSTF2	protein_coding	ENSG00000101811	-0,231512719	0,0000112	0,000043
DPM2	protein_coding	ENSG00000136908	-0,36703409	0,0000112	0,000043
IL21-AS1	antisense	ENSG00000227145	3,998789468	0,0000112	0,0000431
DUSP13	protein_coding	ENSG00000079393	3,999895172	0,0000112	0,0000431
TNRC6C	protein_coding	ENSG00000078687	0,389277016	0,0000112	0,0000431
PLBD1	protein_coding	ENSG00000121316	-0,389982445	0,0000112	0,0000431
PRRX2	protein_coding	ENSG00000167157	-0,436954478	0,0000113	0,0000433
LAMP2	protein_coding	ENSG00000005893	0,198699788	0,0000113	0,0000435
TMEM175	protein_coding	ENSG00000127419	0,414631419	0,0000114	0,0000436
CORO1C	protein_coding	ENSG00000110880	-0,193440587	0,0000114	0,0000437
CCDC34	protein_coding	ENSG00000109881	-0,320916623	0,0000114	0,0000439
DNALI1	protein_coding	ENSG00000163879	5,419898703	0,0000115	0,0000441
CFL1P1	transcribed_unprocesse	ENSG00000223820	0,987473565	0,0000115	0,0000442
MEST	protein_coding	ENSG00000106484	2,037728503	0,0000115	0,0000443
TJP2	protein_coding	ENSG00000119139	-0,354042424	0,0000116	0,0000443
NOC3L	protein_coding	ENSG00000173145	0,193966736	0,0000116	0,0000444
AL138976.2	antisense	ENSG00000259775	2,00390706	0,0000116	0,0000445
C4orf54	protein_coding	ENSG00000248713	3,238528156	0,0000116	0,0000445
RUNDC1	protein_coding	ENSG00000198863	-0,258592092	0,0000117	0,0000446
ERGIC1	protein_coding	ENSG00000113719	-0,144011241	0,0000117	0,0000447
PCLAF	protein_coding	ENSG00000166803	-0,460196249	0,0000117	0,0000449
INPP5E	protein_coding	ENSG00000148384	-0,406644991	0,0000117	0,000045
RANBP3	protein_coding	ENSG00000031823	-0,210521461	0,0000118	0,0000453
HDAC9	protein_coding	ENSG00000048052	0,288735056	0,000012	0,0000459
AL451042.2	antisense	ENSG00000227959	1,363572732	0,000012	0,000046
NPAT	protein_coding	ENSG00000149308	-0,255284756	0,000012	0,000046

GLDC	protein_coding	ENSG00000178445	-0,335593798	0,0000121	0,0000461
C4B	protein_coding	ENSG00000224389	2,589411191	0,0000121	0,0000464
UBTD1	protein_coding	ENSG00000165886	0,500936577	0,0000123	0,000047
RPL17	protein_coding	ENSG00000265681	-0,430571035	0,0000123	0,000047
CXCR2	protein_coding	ENSG00000180871	3,73539093	0,0000123	0,0000471
CCDC130	protein_coding	ENSG00000104957	0,334690267	0,0000123	0,0000471
MCM9	protein_coding	ENSG00000111877	-0,34525022	0,0000124	0,0000473
RNF14	protein_coding	ENSG00000013561	-0,270196857	0,0000124	0,0000474
FBXL16	protein_coding	ENSG00000127585	-1,083664637	0,0000125	0,0000477
NAT1	protein_coding	ENSG00000171428	0,430082757	0,0000127	0,0000483
MUC20P1	unprocessed_pseudoge	ENSG00000224769	3,589856864	0,0000127	0,0000484
EXOSC10-AS1	antisense	ENSG00000230337	1,190544255	0,0000127	0,0000486
IRGM	protein_coding	ENSG00000237693	2,638757516	0,0000127	0,0000486
MARCH1	protein_coding	ENSG00000145416	2,62700586	0,0000127	0,0000486
RPP40	protein_coding	ENSG00000124787	-0,360949097	0,0000128	0,0000487
GPR37	protein_coding	ENSG00000170775	-0,627087766	0,0000129	0,0000491
C16orf87	protein_coding	ENSG00000155330	0,573924539	0,0000129	0,0000492
ABCA6	protein_coding	ENSG00000154262	2,800244032	0,0000129	0,0000493
SPCS2	protein_coding	ENSG00000118363	0,281310518	0,000013	0,0000494
PUM3	protein_coding	ENSG00000080608	-0,289195398	0,000013	0,0000494
BSG	protein_coding	ENSG00000172270	0,216221638	0,000013	0,0000497
NMNAT2	protein_coding	ENSG00000157064	0,340599813	0,0000131	0,00005
KCTD6	protein_coding	ENSG00000168301	-0,42598922	0,0000133	0,0000506
SLITRK5	protein_coding	ENSG00000165300	-0,494586115	0,0000133	0,0000506
AC023906.2	lincRNA	ENSG00000259178	2,598520968	0,0000133	0,0000507
AHRR	protein_coding	ENSG00000063438	-0,39598237	0,0000133	0,0000507
RAB3IL1	protein_coding	ENSG00000167994	-0,485957011	0,0000133	0,0000507
IFT22	protein_coding	ENSG00000128581	-0,325044279	0,0000134	0,000051
VPS50	protein_coding	ENSG00000004766	-0,279756052	0,0000134	0,0000511
NUMBL	protein_coding	ENSG00000105245	-0,402345647	0,0000135	0,0000513
TAF12	protein_coding	ENSG00000120656	0,2759157	0,0000135	0,0000514
HSPA12A	protein_coding	ENSG00000165868	-0,335741112	0,0000135	0,0000514
XRN1	protein_coding	ENSG00000114127	0,225213746	0,0000136	0,0000517
GRK4	protein_coding	ENSG00000125388	-0,56262259	0,0000136	0,0000517
GAS7	protein_coding	ENSG00000007237	5,39487292	0,0000137	0,0000522
AL160408.1	lincRNA	ENSG00000228044	1,257676835	0,0000138	0,0000523
BECN1	protein_coding	ENSG00000126581	0,150759361	0,0000138	0,0000523
ERI1	protein_coding	ENSG00000104626	-0,286424741	0,0000138	0,0000525
ACBD7	protein_coding	ENSG00000176244	-0,62436666	0,0000139	0,0000527
NOP58	protein_coding	ENSG00000055044	-0,261297079	0,0000139	0,0000529

AC016027.1	antisense	ENSG00000225335	1,832852911	0,000014	0,0000531
AGPAT3	protein_coding	ENSG00000160216	-0,290411764	0,000014	0,0000531
YY1AP1	protein_coding	ENSG00000163374	0,214975647	0,000014	0,0000532
ZNF671	protein_coding	ENSG00000083814	0,447792448	0,000014	0,0000533
TEX37	protein_coding	ENSG00000172073	1,731179869	0,0000141	0,0000534
MSH4	protein_coding	ENSG00000057468	1,297745654	0,0000141	0,0000534
RNU1-120P	snRNA	ENSG00000199879	-3,478629501	0,0000141	0,0000535
MYMX	protein_coding	ENSG00000262179	3,679772057	0,0000141	0,0000536
AL357033.3	antisense	ENSG00000276317	2,250820438	0,0000142	0,0000537
ZNF275	protein_coding	ENSG00000063587	0,372832541	0,0000142	0,0000537
GID8	protein_coding	ENSG00000101193	-0,185895718	0,0000142	0,0000537
PGAP2	protein_coding	ENSG00000148985	-0,337842135	0,0000142	0,0000538
CDK9	protein_coding	ENSG00000136807	0,252531709	0,0000142	0,0000539
ZNF280D	protein_coding	ENSG00000137871	-0,260535574	0,0000143	0,000054
AP001000.1	processed_pseudogene	ENSG00000254612	-0,452565274	0,0000143	0,0000541
C5AR1	protein_coding	ENSG00000197405	1,768139417	0,0000143	0,0000541
AFDN	protein_coding	ENSG00000130396	-0,369017878	0,0000143	0,0000541
BLVRA	protein_coding	ENSG00000106605	-0,283824843	0,0000143	0,0000542
AMDHD2	protein_coding	ENSG00000162066	-0,417553964	0,0000144	0,0000544
EXOC3L2	protein_coding	ENSG00000283632	3,969577659	0,0000144	0,0000546
PLIN3	protein_coding	ENSG00000105355	-0,243966836	0,0000146	0,0000551
PARP3	protein_coding	ENSG00000041880	0,282511427	0,0000146	0,0000552
MRPL54	protein_coding	ENSG00000183617	-0,377979809	0,0000148	0,0000559
TYW1B	protein_coding	ENSG00000277149	0,659356437	0,000015	0,0000566
MDC1-AS1	antisense	ENSG00000224328	5,403508403	0,000015	0,0000568
ICOS	protein_coding	ENSG00000163600	2,980519199	0,0000151	0,0000571
PLCD1	protein_coding	ENSG00000187091	0,53576223	0,0000151	0,0000572
SIGIRR	protein_coding	ENSG00000185187	-0,437650368	0,0000151	0,0000572
RHBDD3	protein_coding	ENSG00000100263	0,474973913	0,0000152	0,0000574
ZNF426	protein_coding	ENSG00000130818	0,219476511	0,0000152	0,0000575
GPR180	protein_coding	ENSG00000152749	-0,314429804	0,0000152	0,0000575
PITPNM2	protein_coding	ENSG00000090975	-0,53447387	0,0000152	0,0000575
TRPM2	protein_coding	ENSG00000142185	0,524262815	0,0000153	0,0000578
RPL7A	protein_coding	ENSG00000148303	-0,264900169	0,0000153	0,0000579
LRPAP1	protein_coding	ENSG00000163956	0,204845704	0,0000154	0,0000581
FARP1	protein_coding	ENSG00000152767	-0,197474332	0,0000154	0,0000581
TMEM150C	protein_coding	ENSG00000249242	-0,41970452	0,0000154	0,0000581
SOX30	protein_coding	ENSG00000039600	2,980036769	0,0000155	0,0000585
LPP-AS2	antisense	ENSG00000270959	0,479020462	0,0000155	0,0000586
DDX46	protein_coding	ENSG00000145833	-0,337857869	0,0000156	0,0000586

MATR3	protein_coding	ENSG00000280987	-0,548336618	0,0000156	0,0000586
ITFG1	protein_coding	ENSG00000129636	0,270765328	0,0000156	0,0000587
MRGPRF	protein_coding	ENSG00000172935	-0,331619456	0,0000156	0,0000588
FBXO4	protein_coding	ENSG00000151876	-0,364056805	0,0000156	0,0000588
MLLT10	protein_coding	ENSG00000078403	-0,299471064	0,0000157	0,0000592
AOX3P	transcribed_unprocesse	ENSG00000244301	4,024118985	0,0000157	0,0000592
AC141586.1	transcribed_unprocesse	ENSG00000215154	0,819567288	0,0000157	0,0000592
STYXL1	protein_coding	ENSG00000127952	0,361698708	0,000016	0,0000601
STX10	protein_coding	ENSG00000104915	-0,227399927	0,000016	0,0000601
AL353708.1	lincRNA	ENSG00000260360	1,581305621	0,000016	0,0000602
MX2	protein_coding	ENSG00000183486	5,425556154	0,000016	0,0000602
SLC43A2	protein_coding	ENSG00000167703	0,759269641	0,000016	0,0000602
INAFM2	protein_coding	ENSG00000259330	-0,232489241	0,000016	0,0000602
TXN2	protein_coding	ENSG00000100348	-0,290388071	0,000016	0,0000602
ZNF394	protein_coding	ENSG00000160908	0,279685359	0,000016	0,0000603
HSPE1	protein_coding	ENSG00000115541	-0,307222628	0,000016	0,0000603
FBXO21	protein_coding	ENSG00000135108	-0,212427713	0,0000161	0,0000604
GALK1	protein_coding	ENSG00000108479	-0,4010476	0,0000161	0,0000605
COQ7	protein_coding	ENSG00000167186	-0,317164025	0,0000161	0,0000606
SSPO	protein_coding	ENSG00000197558	2,829509566	0,0000162	0,0000607
SUSD4	protein_coding	ENSG00000143502	0,486619624	0,0000162	0,0000607
CLUAP1	protein_coding	ENSG00000103351	-0,328350953	0,0000162	0,0000607
MBD3	protein_coding	ENSG00000071655	-0,356838956	0,0000162	0,0000607
SUMO2P17	transcribed_processed_	ENSG00000248278	1,500135869	0,0000163	0,0000612
GYS1	protein_coding	ENSG00000104812	0,322665048	0,0000164	0,0000614
SUPT6H	protein_coding	ENSG00000109111	0,340884518	0,0000164	0,0000615
ZNF584	protein_coding	ENSG00000171574	-0,307300486	0,0000164	0,0000616
TRIM67	protein_coding	ENSG00000119283	3,930311379	0,0000165	0,0000617
ZNF304	protein_coding	ENSG00000131845	0,262316275	0,0000165	0,000062
AC093001.1	antisense	ENSG00000244468	2,027520259	0,0000166	0,0000622
GSE1	protein_coding	ENSG00000131149	0,355334419	0,0000167	0,0000624
FKBP9	protein_coding	ENSG00000122642	-0,18420257	0,0000166	0,0000624
CEP57L1	protein_coding	ENSG00000183137	-0,30175303	0,0000167	0,0000625
TAZ	protein_coding	ENSG00000102125	-0,331289609	0,0000167	0,0000625
IFT172	protein_coding	ENSG00000138002	-0,365113005	0,0000167	0,0000625
PPM1N	protein_coding	ENSG00000213889	0,860284359	0,0000168	0,0000628
HAUS1	protein_coding	ENSG00000152240	-0,328683248	0,0000168	0,000063
PPP1CC	protein_coding	ENSG00000186298	-0,222536485	0,0000168	0,0000631
FZD3	protein_coding	ENSG00000104290	-0,466954894	0,0000169	0,0000631
RDH14	protein_coding	ENSG00000240857	-0,319674399	0,000017	0,0000635

FBN2	protein_coding	ENSG00000138829	0,328106888	0,0000171	0,0000638
CDH2	protein_coding	ENSG00000170558	0,183915484	0,0000171	0,0000639
LINC02542	lincRNA	ENSG00000226453	3,526457515	0,0000171	0,000064
FADD	protein_coding	ENSG00000168040	-0,20311013	0,0000171	0,000064
CNTNAP1	protein_coding	ENSG00000108797	0,466846497	0,0000172	0,0000642
AC125257.1	sense_overlapping	ENSG00000259623	-0,336217995	0,0000172	0,0000643
ZNF19	protein_coding	ENSG00000157429	0,734139357	0,0000173	0,0000646
LBX2	protein_coding	ENSG00000179528	0,481612917	0,0000173	0,0000646
CYP4A22-AS1	lincRNA	ENSG00000225506	1,316312632	0,0000173	0,0000647
CDK18	protein_coding	ENSG00000117266	2,060526971	0,0000174	0,0000648
TMEM60	protein_coding	ENSG00000135211	-0,395402777	0,0000175	0,0000653
B3GLCT	protein_coding	ENSG00000187676	-0,272757479	0,0000176	0,0000656
PSEN2	protein_coding	ENSG00000143801	-0,222874652	0,0000176	0,0000658
EVC2	protein_coding	ENSG00000173040	0,373045528	0,0000177	0,0000659
SAMMSON	lincRNA	ENSG00000240405	1,532756441	0,0000177	0,0000661
AC009812.4	lincRNA	ENSG00000260317	1,474007801	0,0000177	0,0000661
PAX8-AS1	processed_transcript	ENSG00000189223	1,732405105	0,0000177	0,0000662
RTF1	protein_coding	ENSG00000137815	-0,217344334	0,0000178	0,0000663
SH3BP5-AS1	antisense	ENSG00000224660	0,791007657	0,0000178	0,0000664
FCAMR	protein_coding	ENSG00000162897	5,370314015	0,0000178	0,0000665
HYKK	protein_coding	ENSG00000188266	-0,504264128	0,0000178	0,0000665
CISD2	protein_coding	ENSG00000145354	0,338742556	0,0000179	0,0000667
FAM189B	protein_coding	ENSG00000160767	-0,408193232	0,0000179	0,0000668
TRMT5	protein_coding	ENSG00000126814	-0,265881409	0,000018	0,0000671
AL031599.1	lincRNA	ENSG00000224228	2,405945065	0,0000181	0,0000676
XRRA1	protein_coding	ENSG00000166435	0,427948931	0,0000184	0,0000685
SUGT1P1	unprocessed_pseudoge	ENSG00000226823	0,670601889	0,0000184	0,0000687
TCTE1	protein_coding	ENSG00000146221	1,540379024	0,0000186	0,0000692
GBA2	protein_coding	ENSG00000070610	0,29386764	0,0000186	0,0000694
E2F6	protein_coding	ENSG00000169016	-0,262768514	0,0000187	0,0000695
EBLN3P	lincRNA	ENSG00000281649	-0,229931603	0,0000188	0,0000698
SSR2	protein_coding	ENSG00000163479	-0,274565063	0,0000193	0,0000717
KSR1	protein_coding	ENSG00000141068	0,467361659	0,0000196	0,0000731
HENMT1	protein_coding	ENSG00000162639	-0,262992919	0,0000198	0,0000736
PPP2R5C	protein_coding	ENSG00000078304	-0,220651664	0,00002	0,0000742
NRXN2	protein_coding	ENSG00000110076	3,589469594	0,0000201	0,0000747
FAM83H	protein_coding	ENSG00000180921	2,260176971	0,0000201	0,0000748
FST	protein_coding	ENSG00000134363	0,457262192	0,0000201	0,0000748
SERPINB10	protein_coding	ENSG00000242550	3,499575281	0,0000202	0,000075
GTF2IP14	unprocessed_pseudoge	ENSG00000226002	1,37109282	0,0000202	0,0000752

NAGPA	protein_coding	ENSG00000103174	-0,324084836	0,0000203	0,0000753
TSEN34	protein_coding	ENSG00000170892	-0,306484308	0,0000203	0,0000754
NFATC2IP	protein_coding	ENSG00000176953	-0,249484254	0,0000206	0,0000764
CYP11A1	protein_coding	ENSG00000140459	0,919762879	0,0000207	0,0000768
ABCC3	protein_coding	ENSG00000108846	1,653911613	0,0000207	0,0000769
MRPL34	protein_coding	ENSG00000130312	-0,363226493	0,0000208	0,0000772
USP2-AS1	antisense	ENSG00000245248	-0,771009751	0,0000209	0,0000774
UNC5CL	protein_coding	ENSG00000124602	1,96652989	0,0000209	0,0000774
FAF2	protein_coding	ENSG00000113194	0,161285037	0,0000209	0,0000774
RPL7P19	processed_pseudogene	ENSG00000241458	5,297902184	0,0000209	0,0000775
TEAD2	protein_coding	ENSG00000074219	-0,319377	0,0000209	0,0000775
ARID2	protein_coding	ENSG00000189079	-0,258946851	0,000021	0,0000778
ACTB	protein_coding	ENSG00000075624	-0,138337706	0,0000211	0,0000782
AC051619.7	sense_intronic	ENSG00000259932	-1,683724777	0,0000211	0,0000782
MTREX	protein_coding	ENSG00000039123	-0,232356225	0,0000211	0,0000783
FSIP2	protein_coding	ENSG00000188738	1,1053989	0,0000213	0,0000788
RFPL1S	antisense	ENSG00000225465	2,800590962	0,0000213	0,0000789
ZSCAN16	protein_coding	ENSG00000196812	0,585049124	0,0000213	0,0000789
AFG3L1P	transcribed_unitary_pse	ENSG00000223959	-0,472751569	0,0000213	0,000079
AC092919.2	TEC	ENSG00000279673	-0,675325506	0,0000215	0,0000794
AC254633.1	lincRNA	ENSG00000272482	1,696573675	0,0000215	0,0000795
TBX4	protein_coding	ENSG00000121075	3,178595461	0,0000215	0,0000795
C1orf115	protein_coding	ENSG00000162817	-0,375901092	0,0000215	0,0000795
MEGF6	protein_coding	ENSG00000162591	0,886386422	0,0000216	0,0000797
KLHL1	protein_coding	ENSG00000150361	2,417909031	0,0000217	0,0000804
NARF	protein_coding	ENSG00000141562	-0,216012984	0,0000218	0,0000805
TDRD3	protein_coding	ENSG00000083544	-0,365674209	0,0000219	0,0000809
ATG2B	protein_coding	ENSG00000066739	0,235336083	0,000022	0,0000811
BMPR1A	protein_coding	ENSG00000107779	-0,247621418	0,000022	0,0000812
TEX30	protein_coding	ENSG00000151287	-0,390811869	0,000022	0,0000812
LINC02051	lincRNA	ENSG00000226859	3,166023443	0,0000221	0,0000815
ELFN1	protein_coding	ENSG00000225968	0,483415861	0,0000221	0,0000815
NRM	protein_coding	ENSG00000137404	-0,349465756	0,0000221	0,0000816
POC1B-AS1	antisense	ENSG00000270344	0,877305539	0,0000221	0,0000817
NUP98	protein_coding	ENSG00000110713	-0,17488966	0,0000222	0,0000818
ZNF775	protein_coding	ENSG00000196456	-0,682588709	0,0000223	0,0000822
ATP2C1	protein_coding	ENSG00000017260	0,160790498	0,0000223	0,0000824
LARS2-AS1	antisense	ENSG00000232455	5,195754578	0,0000224	0,0000826
ADAMTS13	protein_coding	ENSG00000160323	0,729763471	0,0000224	0,0000828
STX3	protein_coding	ENSG00000166900	0,183947578	0,0000225	0,000083

SRBD1	protein_coding	ENSG00000068784	-0,240973591	0,0000225	0,000083
NCOA4P4	processed_pseudogene	ENSG00000249215	3,950925116	0,0000227	0,0000835
FLAD1	protein_coding	ENSG00000160688	-0,268036296	0,0000227	0,0000835
AC025164.1	antisense	ENSG00000245904	2,621703902	0,0000227	0,0000837
GALNT3	protein_coding	ENSG00000115339	2,707249723	0,0000227	0,0000837
AC083805.2	antisense	ENSG00000257953	1,553202433	0,0000231	0,000085
FXYD3	protein_coding	ENSG00000089356	5,242610907	0,0000231	0,0000851
MYH10	protein_coding	ENSG00000133026	-0,275770447	0,0000231	0,0000851
SLC7A10	protein_coding	ENSG00000130876	5,319305925	0,0000231	0,0000852
HEATR4	protein_coding	ENSG00000187105	2,383058301	0,0000232	0,0000853
PITPNM1	protein_coding	ENSG00000110697	0,373694463	0,0000232	0,0000855
TRIM13	protein_coding	ENSG00000204977	-0,244523101	0,0000233	0,0000856
CEBPZ	protein_coding	ENSG00000115816	-0,288567127	0,0000234	0,000086
ZNF529-AS1	antisense	ENSG00000233527	0,555232811	0,0000234	0,0000862
CYP4V2	protein_coding	ENSG00000145476	0,650194746	0,0000235	0,0000866
FBXO16	protein_coding	ENSG00000214050	0,490011308	0,0000236	0,0000866
ANKRD65	protein_coding	ENSG00000235098	3,565236114	0,0000237	0,000087
PRKCZ	protein_coding	ENSG00000067606	0,27857162	0,0000239	0,0000878
FBXO17	protein_coding	ENSG00000269190	-0,248946538	0,000024	0,0000881
UBE2D4	protein_coding	ENSG00000078967	-0,30104707	0,000024	0,0000884
BST1	protein_coding	ENSG00000109743	1,02608167	0,0000241	0,0000885
DLD	protein_coding	ENSG00000091140	-0,235030233	0,0000243	0,0000891
ERH	protein_coding	ENSG00000100632	-0,280725405	0,0000243	0,0000891
LMNA	protein_coding	ENSG00000160789	-0,21008941	0,0000244	0,0000895
AC010595.1	lincRNA	ENSG00000250874	0,740425826	0,0000244	0,0000896
KCTD13	protein_coding	ENSG00000174943	0,488400987	0,0000244	0,0000896
HIP1	protein_coding	ENSG00000127946	-0,366666817	0,0000246	0,0000903
COLQ	protein_coding	ENSG00000206561	1,655176077	0,0000247	0,0000905
AC118755.2	antisense	ENSG00000265349	2,417120346	0,0000247	0,0000907
ADCY9	protein_coding	ENSG00000162104	-0,328391736	0,0000248	0,0000911
AC022211.2	antisense	ENSG00000263843	-0,851793859	0,0000249	0,0000914
MOSMO	protein_coding	ENSG00000185716	0,261644991	0,000025	0,0000915
VAMP1	protein_coding	ENSG00000139190	0,724014581	0,000025	0,0000916
CCDC107	protein_coding	ENSG00000159884	-0,378172647	0,000025	0,0000918
RASIP1	protein_coding	ENSG00000105538	0,96676337	0,0000253	0,0000927
USP36	protein_coding	ENSG00000055483	0,300775137	0,0000253	0,0000928
CDC27	protein_coding	ENSG00000004897	-0,255765322	0,0000253	0,0000928
SERPINB5	protein_coding	ENSG00000206075	5,298345566	0,0000255	0,0000936
DKK2	protein_coding	ENSG00000155011	3,450607331	0,0000255	0,0000936
RBM8A	protein_coding	ENSG00000265241	-0,248257904	0,0000256	0,0000937

B3GALT6	protein_coding	ENSG00000176022	-0,3065618	0,0000258	0,0000943
NBEA	protein_coding	ENSG00000172915	0,358103619	0,0000259	0,0000947
UGP2	protein_coding	ENSG00000169764	0,259747479	0,000026	0,0000951
ZNF674-AS1	lincRNA	ENSG00000230844	-0,357110595	0,000026	0,0000952
TMEM128	protein_coding	ENSG00000132406	0,327202603	0,000026	0,0000952
VPS72	protein_coding	ENSG00000163159	-0,244675745	0,000026	0,0000953
LDHB	protein_coding	ENSG00000111716	-0,202817464	0,0000262	0,0000956
RTKN	protein_coding	ENSG00000114993	-0,264039658	0,0000263	0,000096
KCNJ1	protein_coding	ENSG00000151704	2,915725562	0,0000264	0,0000964
AL133517.1	transcribed_processed_	ENSG00000232450	1,238579908	0,0000264	0,0000966
ORAI3	protein_coding	ENSG00000175938	0,622896071	0,0000265	0,0000967
PRR14L	protein_coding	ENSG00000183530	-0,284027873	0,0000265	0,0000967
ADGRG6	protein_coding	ENSG00000112414	-0,183294074	0,0000265	0,0000969
PRPF31	protein_coding	ENSG00000105618	-0,173709006	0,0000267	0,0000975
AL590004.3	lincRNA	ENSG00000260604	0,87623489	0,0000268	0,0000978
SSRP1	protein_coding	ENSG00000149136	-0,156035642	0,0000268	0,0000979
AC097059.1	lincRNA	ENSG00000226835	0,782116146	0,0000269	0,0000981
CCR4	protein_coding	ENSG00000183813	3,951133801	0,0000269	0,0000981
ANKRD13D	protein_coding	ENSG00000172932	-0,315711658	0,0000271	0,0000989
CD55	protein_coding	ENSG00000196352	0,250038743	0,0000271	0,000099
LSM5	protein_coding	ENSG00000106355	-0,332184986	0,0000272	0,000099
EMILIN2	protein_coding	ENSG00000132205	-0,400834822	0,0000272	0,000099
ZBTB44	protein_coding	ENSG00000196323	-0,271131796	0,0000272	0,0000993
DCDC2C	protein_coding	ENSG00000214866	3,879383635	0,0000273	0,0000994
ZNF594	protein_coding	ENSG00000180626	-0,307735577	0,0000273	0,0000994
MRPS28	protein_coding	ENSG00000147586	-0,415143077	0,0000273	0,0000994
SF3B4	protein_coding	ENSG00000143368	-0,246420511	0,0000274	0,0000999
AC007786.1	lincRNA	ENSG00000267498	2,540314377	0,0000275	0,0001
CCDC73	protein_coding	ENSG00000186714	2,877104351	0,0000276	0,0001
WDR45B	protein_coding	ENSG00000141580	0,125491658	0,0000275	0,0001
TAC3	protein_coding	ENSG00000166863	3,174667231	0,0000277	0,000101
VDAC1	protein_coding	ENSG00000213585	-0,185438684	0,0000277	0,000101
ZW10	protein_coding	ENSG00000086827	-0,249223843	0,0000279	0,000101
RADIL	protein_coding	ENSG00000157927	-0,471055749	0,0000278	0,000101
SCFD1	protein_coding	ENSG00000092108	0,230020434	0,0000281	0,000102
ZNF271P	transcribed_unitary_pse	ENSG00000257267	-0,231666528	0,0000279	0,000102
CCDC69	protein_coding	ENSG00000198624	0,866451216	0,0000283	0,000103
INPPL1	protein_coding	ENSG00000165458	0,326679855	0,0000284	0,000103
POLDIP2	protein_coding	ENSG00000004142	-0,183210087	0,0000284	0,000103
IFITM2	protein_coding	ENSG00000185201	-0,247307355	0,0000283	0,000103

LRRK1	protein_coding	ENSG00000154237	-0,332957712	0,0000282	0,000103
PRDM6	protein_coding	ENSG00000061455	2,388879013	0,0000287	0,000104
HYAL1	protein_coding	ENSG00000114378	1,267079357	0,0000286	0,000104
SLC10A7	protein_coding	ENSG00000120519	0,34989389	0,0000285	0,000104
DENR	protein_coding	ENSG00000139726	-0,224578427	0,0000285	0,000104
PHF12	protein_coding	ENSG00000109118	-0,267239339	0,0000285	0,000104
TRIM14	protein_coding	ENSG00000106785	-0,27055062	0,0000285	0,000104
PPP2R3B	protein_coding	ENSG00000167393	-0,355718057	0,0000286	0,000104
ELF3-AS1	antisense	ENSG00000234678	5,290210994	0,000029	0,000105
AC021054.1	antisense	ENSG00000177406	0,558019926	0,0000288	0,000105
AC036214.1	lincRNA	ENSG00000249328	3,539952696	0,0000291	0,000105
RGS7	protein_coding	ENSG00000182901	0,393634414	0,0000289	0,000105
BCL2	protein_coding	ENSG00000171791	-0,396241683	0,0000288	0,000105
GSTA9P	transcribed_unprocesse	ENSG00000243236	5,214281082	0,000029	0,000105
AC078923.1	lincRNA	ENSG00000258077	1,653595078	0,0000291	0,000106
AL355596.1	lincRNA	ENSG00000259828	-0,615334557	0,0000292	0,000106
OSBPL9	protein_coding	ENSG00000117859	0,215519122	0,0000292	0,000106
NPTXR	protein_coding	ENSG00000221890	0,710885444	0,0000296	0,000107
NCAPH2	protein_coding	ENSG00000025770	-0,256224215	0,0000296	0,000107
WDR91	protein_coding	ENSG00000105875	-0,449610124	0,0000294	0,000107
BSN	protein_coding	ENSG00000164061	-0,733745969	0,0000294	0,000107
SKAP2	protein_coding	ENSG00000005020	0,231942981	0,0000298	0,000108
ANAPC13	protein_coding	ENSG00000129055	-0,199943592	0,0000299	0,000108
SPIDR	protein_coding	ENSG00000164808	-0,222217107	0,0000297	0,000108
GLI2	protein_coding	ENSG00000074047	-0,609342088	0,0000298	0,000108
AC243571.2	antisense	ENSG00000277501	3,549677358	0,0000302	0,000109
DCTD	protein_coding	ENSG00000129187	-0,150195712	0,00003	0,000109
GAS2L3	protein_coding	ENSG00000139354	-0,25445305	0,0000302	0,000109
FIS1	protein_coding	ENSG00000214253	-0,332433142	0,0000301	0,000109
LYSMD2	protein_coding	ENSG00000140280	-0,351458464	0,0000302	0,000109
LINCMD1	lincRNA	ENSG00000225613	3,820877679	0,0000306	0,000111
AC083843.3	lincRNA	ENSG00000259820	0,984897941	0,0000307	0,000111
PPY	protein_coding	ENSG00000108849	2,817100087	0,0000308	0,000111
AC010618.1	protein_coding	ENSG00000130313	-0,280113741	0,0000306	0,000111
FBXL4	protein_coding	ENSG00000112234	-0,281843833	0,0000307	0,000111
TYSND1	protein_coding	ENSG00000156521	-0,357714163	0,0000307	0,000111
PPM1B	protein_coding	ENSG00000138032	0,21269022	0,000031	0,000112
SCRN1	protein_coding	ENSG00000136193	0,18987606	0,0000311	0,000112
CD320	protein_coding	ENSG00000167775	-0,318763583	0,0000311	0,000112
SLC16A6	protein_coding	ENSG00000108932	0,888675201	0,0000312	0,000113

TTLL3	protein_coding	ENSG00000214021	0,639210476	0,0000312	0,000113
GGNBP2	protein_coding	ENSG00000278311	0,208568021	0,0000313	0,000113
TNKS1BP1	protein_coding	ENSG00000149115	0,365926954	0,0000317	0,000114
PSME2	protein_coding	ENSG00000100911	0,294148418	0,0000315	0,000114
ARFGEF2	protein_coding	ENSG00000124198	0,212602422	0,0000315	0,000114
HNF4G	protein_coding	ENSG00000164749	0,797214503	0,000032	0,000115
PAPOLG	protein_coding	ENSG00000115421	0,244806418	0,000032	0,000115
CD81	protein_coding	ENSG00000110651	-0,243782527	0,0000318	0,000115
LINC01085	transcribed_processed_	ENSG00000248698	-0,325037961	0,000032	0,000115
ERICH2	protein_coding	ENSG00000204334	1,172721631	0,0000323	0,000116
PRICKLE3	protein_coding	ENSG00000012211	0,627453488	0,0000322	0,000116
DOCK1	protein_coding	ENSG00000150760	-0,198977577	0,0000324	0,000116
THAP12	protein_coding	ENSG00000137492	-0,217837514	0,0000323	0,000116
MTCH2	protein_coding	ENSG00000109919	-0,241182785	0,0000321	0,000116
MSX2	protein_coding	ENSG00000120149	-0,415814785	0,0000322	0,000116
KLHL4	protein_coding	ENSG00000102271	0,861338661	0,0000326	0,000117
FBXL19	protein_coding	ENSG00000099364	-0,454092431	0,0000325	0,000117
AL355512.1	lincRNA	ENSG00000273143	1,317617941	0,0000327	0,000118
ACTN1	protein_coding	ENSG00000072110	0,213273887	0,0000329	0,000118
ILF3	protein_coding	ENSG00000129351	-0,217027408	0,0000328	0,000118
PARK7	protein_coding	ENSG00000116288	-0,22634427	0,0000328	0,000118
SNRPD2	protein_coding	ENSG00000125743	-0,285805205	0,0000329	0,000118
ZNF680	protein_coding	ENSG00000173041	-0,559727727	0,0000328	0,000118
AC011601.1	lincRNA	ENSG00000257515	2,126669095	0,0000332	0,000119
GRAMD2A	protein_coding	ENSG00000175318	3,092066041	0,0000332	0,000119
ENOX2	protein_coding	ENSG00000165675	0,327643554	0,0000332	0,000119
METAP2	protein_coding	ENSG00000111142	0,187222341	0,0000331	0,000119
TSN	protein_coding	ENSG00000211460	-0,228236169	0,0000331	0,000119
TOPBP1	protein_coding	ENSG00000163781	-0,237462094	0,0000331	0,000119
LINC01204	lincRNA	ENSG00000229563	1,062166985	0,0000336	0,00012
TLX2	protein_coding	ENSG00000115297	3,772170815	0,0000335	0,00012
AC008250.2	sense_intronic	ENSG00000275567	5,218955063	0,0000335	0,00012
EIF4BP6	processed_pseudogene	ENSG00000197258	-0,423592705	0,0000338	0,000121
TP53	protein_coding	ENSG00000141510	0,265810019	0,0000338	0,000121
SLC25A30	protein_coding	ENSG00000174032	0,242728795	0,0000339	0,000121
FGD2	protein_coding	ENSG00000146192	1,227715133	0,0000339	0,000122
RAB10	protein_coding	ENSG00000084733	-0,189987206	0,0000342	0,000122
ACACA	protein_coding	ENSG00000278540	-0,245463876	0,000034	0,000122
LRRC8A	protein_coding	ENSG00000136802	-0,258522006	0,0000339	0,000122
ZSCAN32	protein_coding	ENSG00000140987	-0,304393295	0,0000339	0,000122

TM4SF1-AS1	antisense	ENSG00000240541	0,655998198	0,0000345	0,000123
BAP1	protein_coding	ENSG00000163930	-0,247801744	0,0000343	0,000123
PCIF1	protein_coding	ENSG00000100982	-0,297990903	0,0000344	0,000123
FXN	protein_coding	ENSG00000165060	-0,35725906	0,0000344	0,000123
PSMA2	protein_coding	ENSG00000106588	-0,658076573	0,0000344	0,000123
SIRT4	protein_coding	ENSG00000089163	0,878999657	0,0000346	0,000124
TSPYL5	protein_coding	ENSG00000180543	-0,393468238	0,0000347	0,000124
ITFG2	protein_coding	ENSG00000111203	-0,369865023	0,0000349	0,000125
VRK3	protein_coding	ENSG00000105053	-0,225624192	0,0000353	0,000126
ESD	protein_coding	ENSG00000139684	-0,276187184	0,0000352	0,000126
PTGFR	protein_coding	ENSG00000122420	0,320719202	0,0000355	0,000127
XRCC6	protein_coding	ENSG00000196419	-0,232548632	0,0000354	0,000127
USP4	protein_coding	ENSG00000114316	-0,235957919	0,0000356	0,000127
USP4	protein_coding	ENSG00000114316	-0,235957919	0,0000356	0,000127
TMEM167A	protein_coding	ENSG00000174695	-0,270429424	0,0000356	0,000127
SLC29A2	protein_coding	ENSG00000174669	-0,58274598	0,0000354	0,000127
FAM120B	protein_coding	ENSG00000112584	0,211299803	0,0000357	0,000128
FAHD1	protein_coding	ENSG00000180185	-0,268580732	0,0000358	0,000128
EPN3	protein_coding	ENSG00000049283	3,43276113	0,0000362	0,000129
RNF19A	protein_coding	ENSG00000034677	0,208944507	0,0000363	0,000129
COPB2	protein_coding	ENSG00000184432	0,138114361	0,0000362	0,000129
INTS8	protein_coding	ENSG00000164941	-0,224300078	0,0000362	0,000129
HCP5	sense_overlapping	ENSG00000206337	1,599314482	0,0000361	0,000129
AF127577.4	lincRNA	ENSG00000235609	1,926911506	0,0000363	0,00013
C11orf45	protein_coding	ENSG00000174370	0,380202121	0,0000365	0,00013
STRIP1	protein_coding	ENSG00000143093	-0,244171039	0,0000365	0,00013
MUC17	protein_coding	ENSG00000169876	5,075798894	0,0000367	0,000131
MAFG	protein_coding	ENSG00000197063	0,375755189	0,0000368	0,000131
WFDC10B	protein_coding	ENSG00000182931	3,477377434	0,0000371	0,000132
CA8	protein_coding	ENSG00000178538	2,753641616	0,0000371	0,000132
DIAPH2-AS1	antisense	ENSG00000236256	3,458383842	0,0000373	0,000133
TNKS2-AS1	antisense	ENSG00000228701	1,750958057	0,0000375	0,000133
SLC26A2	protein_coding	ENSG00000155850	0,227211045	0,0000372	0,000133
NBL1	protein_coding	ENSG00000158747	-0,428529613	0,0000373	0,000133
NBL1	protein_coding	ENSG00000158747	-0,428529613	0,0000373	0,000133
CCDC61	protein_coding	ENSG00000104983	-0,519684433	0,0000373	0,000133
LDHAP4	transcribed_processed_	ENSG00000214110	-0,504601143	0,0000372	0,000133
TRAK2	protein_coding	ENSG00000115993	0,163260169	0,0000377	0,000134
DDX41	protein_coding	ENSG00000183258	-0,207027991	0,0000376	0,000134
CIAPIN1	protein_coding	ENSG00000005194	-0,24808481	0,0000376	0,000134

PTHLH	protein_coding	ENSG00000087494	2,308140402	0,0000378	0,000135
DPP9	protein_coding	ENSG00000142002	-0,260968597	0,000038	0,000135
CHCHD1	protein_coding	ENSG00000172586	-0,290696839	0,0000386	0,000137
DUXB	protein_coding	ENSG00000282757	5,128265793	0,0000387	0,000138
TMEM92	protein_coding	ENSG00000167105	0,813752509	0,0000387	0,000138
SNX30	protein_coding	ENSG00000148158	-0,253778977	0,0000388	0,000138
PDHB	protein_coding	ENSG00000168291	-0,26971744	0,0000388	0,000138
SALL4	protein_coding	ENSG00000101115	0,645539366	0,0000391	0,000139
ANAPC7	protein_coding	ENSG00000196510	-0,185024981	0,0000392	0,000139
ITGAE	protein_coding	ENSG00000083457	-0,347121642	0,000039	0,000139
XXYLT1-AS2	antisense	ENSG00000230266	1,70524578	0,0000394	0,00014
CFLAR-AS1	antisense	ENSG00000226312	0,594741628	0,0000394	0,00014
MASTL	protein_coding	ENSG00000120539	-0,222913538	0,0000394	0,00014
FBXW4	protein_coding	ENSG00000107829	0,365230958	0,0000397	0,000141
GOLM1	protein_coding	ENSG00000135052	-0,212601653	0,0000396	0,000141
RFLNB	protein_coding	ENSG00000183688	-0,231508987	0,0000396	0,000141
NAT14	protein_coding	ENSG00000090971	-0,310268109	0,0000397	0,000141
TENM2	protein_coding	ENSG00000145934	2,142109368	0,00004	0,000142
TTN	protein_coding	ENSG00000155657	1,076414055	0,0000401	0,000142
ASNS	protein_coding	ENSG00000070669	0,752704298	0,00004	0,000142
ANAPC1	protein_coding	ENSG00000153107	-0,24359156	0,00004	0,000142
S100A6	protein_coding	ENSG00000197956	-0,324625023	0,00004	0,000142
NAF1	protein_coding	ENSG00000145414	-0,359106622	0,0000402	0,000142
KIF28P	transcribed_unitary_pse	ENSG00000223519	2,827268063	0,0000401	0,000142
ZNF41	protein_coding	ENSG00000147124	0,309682597	0,0000404	0,000143
AC118549.1	protein_coding	ENSG00000036549	-0,211838043	0,0000405	0,000143
NID1	protein_coding	ENSG00000116962	-0,295119408	0,0000403	0,000143
AL049840.4	sense_intronic	ENSG00000269958	-0,488613877	0,0000402	0,000143
AC004784.1	antisense	ENSG00000282943	0,79388195	0,0000406	0,000144
MYRIP	protein_coding	ENSG00000170011	1,459675972	0,0000405	0,000144
IBA57	protein_coding	ENSG00000181873	0,397699616	0,0000406	0,000144
PPT1	protein_coding	ENSG00000131238	-0,252769862	0,0000407	0,000144
POLG	protein_coding	ENSG00000140521	-0,283651609	0,0000406	0,000144
AL606469.1	antisense	ENSG00000224215	1,687083277	0,0000411	0,000145
TBCD	protein_coding	ENSG00000141556	-0,219718801	0,000041	0,000145
CYC1	protein_coding	ENSG00000179091	-0,284502377	0,0000411	0,000145
TTC5	protein_coding	ENSG00000136319	-0,291712692	0,0000411	0,000145
AC026250.1	lincRNA	ENSG00000245522	0,487122873	0,0000411	0,000146
ANTXR1	protein_coding	ENSG00000169604	-0,172389245	0,0000414	0,000146
COMTD1	protein_coding	ENSG00000165644	-0,412469868	0,0000412	0,000146

BSN-DT	lincRNA	ENSG00000226913	1,432261145	0,0000416	0,000147
USHBP1	protein_coding	ENSG00000130307	3,449964073	0,0000415	0,000147
NUCKS1	protein_coding	ENSG00000069275	-0,231998232	0,0000415	0,000147
INSYN2	protein_coding	ENSG00000188916	3,450105829	0,0000417	0,000148
SHQ1	protein_coding	ENSG00000144736	-0,252511391	0,000042	0,000148
PKNOX1	protein_coding	ENSG00000160199	-0,2966497	0,0000418	0,000148
ZBTB40	protein_coding	ENSG00000184677	-0,30506818	0,000042	0,000148
AL136164.3	TEC	ENSG00000279289	1,150978189	0,0000419	0,000148
NAALADL1	protein_coding	ENSG00000168060	5,241617997	0,0000423	0,000149
OR1F1	protein_coding	ENSG00000168124	5,079406766	0,0000421	0,000149
CHRAC1	protein_coding	ENSG00000104472	-0,258148626	0,0000422	0,000149
AC007389.5	lincRNA	ENSG00000281920	5,071811748	0,0000426	0,00015
SOX9-AS1	processed_transcript	ENSG00000234899	1,1913125	0,0000428	0,000151
RPARP-AS1	processed_transcript	ENSG00000269609	-0,485000368	0,000043	0,000151
PKNOX2	protein_coding	ENSG00000165495	5,129970005	0,0000428	0,000151
DCHS1	protein_coding	ENSG00000166341	3,470540331	0,0000429	0,000151
DCP1B	protein_coding	ENSG00000151065	0,269278028	0,0000427	0,000151
ETF1	protein_coding	ENSG00000120705	0,15664998	0,0000429	0,000151
AP000769.1	transcribed_unprocesse	ENSG00000173727	0,483774929	0,0000427	0,000151
MYDGF	protein_coding	ENSG00000074842	0,234121586	0,0000431	0,000152
STK38	protein_coding	ENSG00000112079	0,154345355	0,000043	0,000152
HNRNPH3	protein_coding	ENSG00000096746	-0,298261673	0,0000432	0,000152
AP001453.1	antisense	ENSG00000256116	1,948090064	0,0000433	0,000153
BOD1	protein_coding	ENSG00000145919	-0,286012311	0,0000434	0,000153
EVI2B	protein_coding	ENSG00000185862	-0,514924396	0,0000434	0,000153
HSPD1	protein_coding	ENSG00000144381	-0,225283131	0,0000438	0,000154
POP5	protein_coding	ENSG00000167272	-0,230116029	0,0000438	0,000154
SLC6A8	protein_coding	ENSG00000130821	0,37572957	0,000044	0,000155
HDAC5	protein_coding	ENSG00000108840	0,333961585	0,000044	0,000155
AC026333.3	lincRNA	ENSG00000256249	3,772787576	0,0000445	0,000156
RAC2	protein_coding	ENSG00000128340	0,228830852	0,0000443	0,000156
SP140L	protein_coding	ENSG00000185404	0,227439687	0,0000443	0,000156
MDM4	protein_coding	ENSG00000198625	-0,241699664	0,0000443	0,000156
CT62	protein_coding	ENSG00000225362	1,171701835	0,0000448	0,000157
ZC3H7A	protein_coding	ENSG00000122299	0,21915628	0,0000446	0,000157
RMI1	protein_coding	ENSG00000178966	-0,403277744	0,0000446	0,000157
DLG1-AS1	antisense	ENSG00000227375	0,89494791	0,0000449	0,000158
ADGRG4	protein_coding	ENSG00000156920	3,705579137	0,0000449	0,000158
TNFAIP3	protein_coding	ENSG00000118503	0,250077606	0,000045	0,000158
LINC01094	lincRNA	ENSG00000251442	1,639815125	0,0000453	0,000159

MBTPS2	protein_coding	ENSG00000012174	0,304756756	0,0000452	0,000159
PAIP2	protein_coding	ENSG00000120727	0,227949807	0,0000454	0,000159
NOB1	protein_coding	ENSG00000141101	-0,235600306	0,0000453	0,000159
THEMIS2	protein_coding	ENSG00000130775	0,94649711	0,0000455	0,00016
UBE2K	protein_coding	ENSG00000078140	-0,260368264	0,0000458	0,000161
TM7SF2	protein_coding	ENSG00000149809	0,562809532	0,0000461	0,000162
HDAC8	protein_coding	ENSG00000147099	-0,258259423	0,0000462	0,000162
HLA-DRB5	protein_coding	ENSG00000198502	-0,265082023	0,0000462	0,000162
ZNF821	protein_coding	ENSG00000102984	0,507273952	0,0000467	0,000164
TGFBR2	protein_coding	ENSG00000163513	-0,176329292	0,0000467	0,000164
FAM234B	protein_coding	ENSG00000084444	-0,326340862	0,0000469	0,000164
SULF2	protein_coding	ENSG00000196562	1,688088412	0,0000472	0,000165
CYSTM1	protein_coding	ENSG00000120306	0,322705005	0,0000471	0,000165
AL645568.1	antisense	ENSG00000203739	0,93471146	0,0000474	0,000166
LINC02446	lincRNA	ENSG00000256039	5,096490678	0,0000473	0,000166
RHOU	protein_coding	ENSG00000116574	0,660282009	0,0000474	0,000166
KATNBL1	protein_coding	ENSG00000134152	-0,271365747	0,0000474	0,000166
CLASP2	protein_coding	ENSG00000163539	-0,222497074	0,0000478	0,000167
RPL10A	protein_coding	ENSG00000198755	-0,240683482	0,0000478	0,000167
DNPH1	protein_coding	ENSG00000112667	-0,306682995	0,0000477	0,000167
SERPINA2	polymorphic_pseudoge	ENSG00000258597	5,085142835	0,0000482	0,000168
SP3	protein_coding	ENSG00000172845	-0,233537042	0,0000482	0,000168
USP37	protein_coding	ENSG00000135913	-0,23613139	0,0000479	0,000168
MRPS12	protein_coding	ENSG00000128626	-0,301152693	0,0000482	0,000168
ZFAT	protein_coding	ENSG00000066827	-0,343434796	0,0000482	0,000168
AC106795.1	transcribed_unprocesse	ENSG00000170089	-0,407933336	0,0000482	0,000168
AC121761.1	antisense	ENSG00000257497	0,831513637	0,0000484	0,000169
AL390760.1	lincRNA	ENSG00000203364	2,289747792	0,0000483	0,000169
KCNK6	protein_coding	ENSG00000099337	1,304525456	0,0000482	0,000169
ZC3H8	protein_coding	ENSG00000144161	0,265247598	0,0000484	0,000169
SIPA1	protein_coding	ENSG00000213445	-0,281480902	0,0000487	0,00017
TOMM5	protein_coding	ENSG00000175768	-0,669008594	0,0000487	0,00017
AC007686.3	antisense	ENSG00000273729	1,604528933	0,0000488	0,000171
LRRC66	protein_coding	ENSG00000188993	1,879932474	0,000049	0,000171
COLGALT2	protein_coding	ENSG00000198756	0,593606026	0,000049	0,000171
SF1	protein_coding	ENSG00000168066	-0,218531292	0,000049	0,000171
ZNF273	protein_coding	ENSG00000198039	-0,544164929	0,000049	0,000171
MYLK4	protein_coding	ENSG00000145949	1,394936916	0,0000492	0,000172
CUZD1	protein_coding	ENSG00000138161	0,625117603	0,0000494	0,000172
AC022239.1	antisense	ENSG00000269954	-0,875496623	0,0000498	0,000174

SEPT14P12	processed_pseudogene	ENSG00000235748	-1,298972683	0,0000499	0,000174
TPM2	protein_coding	ENSG00000198467	0,228175371	0,0000498	0,000174
PPM1G	protein_coding	ENSG00000115241	-0,189231589	0,0000499	0,000174
RAB14	protein_coding	ENSG00000119396	-0,200699254	0,0000497	0,000174
DCPS	protein_coding	ENSG00000110063	-0,248703135	0,00005	0,000174
ZNF689	protein_coding	ENSG00000156853	-0,295011933	0,00005	0,000174
ASF1B	protein_coding	ENSG00000105011	-0,217251597	0,0000503	0,000175
AC007448.3	antisense	ENSG00000266473	2,790829126	0,0000504	0,000176
AC010883.1	antisense	ENSG00000234936	2,052129908	0,0000504	0,000176
PPWD1	protein_coding	ENSG00000113593	-0,21715556	0,0000506	0,000176
OPA3	protein_coding	ENSG00000125741	-0,235319126	0,0000504	0,000176
LCN15	protein_coding	ENSG00000177984	3,439089438	0,0000507	0,000177
SPG21	protein_coding	ENSG00000090487	-0,1660944	0,0000507	0,000177
ASCC2	protein_coding	ENSG00000100325	0,211203007	0,0000511	0,000178
MCRIP1	protein_coding	ENSG00000225663	-0,379224738	0,0000511	0,000178
KRT8P9	processed_pseudogene	ENSG00000259470	1,912870403	0,0000516	0,000179
AHCYL1	protein_coding	ENSG00000168710	0,13788797	0,0000516	0,000179
TYRO3	protein_coding	ENSG00000092445	-0,259282228	0,0000514	0,000179
SORD	protein_coding	ENSG00000140263	-0,316080199	0,0000516	0,000179
SCAND2P	transcribed_unprocesse	ENSG00000176700	0,429433145	0,0000516	0,000179
AL358332.1	lincRNA	ENSG00000258942	5,188890969	0,0000516	0,00018
HOOK1	protein_coding	ENSG00000134709	3,449292195	0,0000523	0,000181
NLRP1	protein_coding	ENSG00000091592	0,293294372	0,000052	0,000181
CPNE3	protein_coding	ENSG00000085719	-0,261688821	0,000052	0,000181
RNF44	protein_coding	ENSG00000146083	-0,426066187	0,000052	0,000181
TANGO2	protein_coding	ENSG00000183597	0,253486496	0,0000523	0,000182
YTHDF1	protein_coding	ENSG00000149658	0,185679551	0,0000525	0,000182
POLA1	protein_coding	ENSG00000101868	-0,260081728	0,0000524	0,000182
RPUSD2	protein_coding	ENSG00000166133	-0,328846557	0,0000526	0,000183
SNORD6	snoRNA	ENSG00000202314	-1,410960309	0,0000527	0,000183
TBILA	antisense	ENSG00000261488	0,844005432	0,000053	0,000184
SCN8A	protein_coding	ENSG00000196876	0,295893482	0,0000532	0,000184
CSNK1A1P1	transcribed_processed_	ENSG00000223518	1,464158223	0,0000532	0,000184
TBPL1	protein_coding	ENSG00000028839	0,250818295	0,0000534	0,000185
PRKAR2A	protein_coding	ENSG00000114302	-0,18051885	0,0000532	0,000185
LIMCH1	protein_coding	ENSG00000064042	-0,209797534	0,0000536	0,000186
AC008914.1	antisense	ENSG00000262211	3,424255062	0,000054	0,000187
TTC25	protein_coding	ENSG00000204815	1,160376645	0,0000547	0,000189
RNF185	protein_coding	ENSG00000138942	0,205346726	0,0000546	0,000189
TRAIP	protein_coding	ENSG00000183763	-0,28910873	0,0000546	0,000189

CREB3L1	protein_coding	ENSG00000157613	0,83391978	0,0000547	0,00019
FZD8	protein_coding	ENSG00000177283	0,551529861	0,0000548	0,00019
PPP2R3C	protein_coding	ENSG00000092020	0,244688814	0,0000549	0,00019
ITGB1	protein_coding	ENSG00000150093	-0,21112888	0,0000548	0,00019
TTC31	protein_coding	ENSG00000115282	-0,302761699	0,0000549	0,00019
ELMOD3	protein_coding	ENSG00000115459	0,288563224	0,0000552	0,000191
PPM1F	protein_coding	ENSG00000100034	-0,27630187	0,0000552	0,000191
ANKRD63	protein_coding	ENSG00000230778	-1,414743324	0,0000559	0,000193
AC083862.2	antisense	ENSG00000272941	4,982297756	0,0000561	0,000194
PTPRF	protein_coding	ENSG00000142949	-0,258798795	0,000056	0,000194
A1BG-AS1	antisense	ENSG00000268895	0,404872008	0,0000564	0,000195
CA11	protein_coding	ENSG00000063180	0,586658701	0,0000567	0,000196
CDC42	protein_coding	ENSG00000070831	-0,257621324	0,0000567	0,000196
ANKRD10	protein_coding	ENSG00000088448	0,237543282	0,000057	0,000197
SKA2	protein_coding	ENSG00000182628	-0,286066569	0,000057	0,000197
CENPC	protein_coding	ENSG00000145241	-0,241993085	0,0000572	0,000198
S100A5	protein_coding	ENSG00000196420	1,521928949	0,0000575	0,000199
ICK	protein_coding	ENSG00000112144	0,276931617	0,0000577	0,000199
TMEM74	protein_coding	ENSG00000164841	-0,766950814	0,0000575	0,000199
CRIP2	protein_coding	ENSG00000182809	0,440472589	0,0000578	0,0002
TJP1	protein_coding	ENSG00000104067	0,217555228	0,0000579	0,0002
ZDHHC8	protein_coding	ENSG00000099904	-0,350336903	0,0000581	0,000201
GPM6A	protein_coding	ENSG00000150625	1,632180784	0,0000584	0,000202
ZUP1	protein_coding	ENSG00000153975	0,344096905	0,0000584	0,000202
MGAT5	protein_coding	ENSG00000152127	-0,268261493	0,0000585	0,000202
MICOS13	protein_coding	ENSG00000174917	-0,376075652	0,0000585	0,000202
ELMSAN1	protein_coding	ENSG00000156030	0,36719584	0,0000589	0,000203
PLEKHG1	protein_coding	ENSG00000120278	3,356199574	0,0000593	0,000204
WBP2	protein_coding	ENSG00000132471	0,294271061	0,0000592	0,000204
TPRN	protein_coding	ENSG00000176058	0,392076353	0,0000595	0,000205
DNAAF3	protein_coding	ENSG00000167646	-0,418937744	0,0000596	0,000205
P2RX5	protein_coding	ENSG00000083454	-0,509474853	0,0000595	0,000205
C8orf34	protein_coding	ENSG00000165084	1,961272421	0,0000598	0,000206
ABLIM2	protein_coding	ENSG00000163995	1,673640414	0,0000599	0,000206
BST2	protein_coding	ENSG00000130303	1,658796852	0,00006	0,000206
PCGF5	protein_coding	ENSG00000180628	0,270755759	0,0000597	0,000206
DLAT	protein_coding	ENSG00000150768	-0,211903395	0,00006	0,000206
TBCCD1	protein_coding	ENSG00000113838	-0,288778328	0,0000598	0,000206
AHDC1	protein_coding	ENSG00000126705	-0,354365195	0,0000599	0,000206
AC046143.1	antisense	ENSG00000229334	1,045543597	0,0000601	0,000207

TMEM159	protein_coding	ENSG00000011638	0,441170942	0,0000602	0,000207
STAG2	protein_coding	ENSG00000101972	-0,22324608	0,0000603	0,000207
ARID3A	protein_coding	ENSG00000116017	-0,335623757	0,0000602	0,000207
AC087257.2	unprocessed_pseudoge	ENSG00000256056	3,772114087	0,0000602	0,000207
LINC-PINT	processed_transcript	ENSG00000231721	0,498980208	0,0000606	0,000208
AZI2	protein_coding	ENSG00000163512	0,238806852	0,0000605	0,000208
RIN1	protein_coding	ENSG00000174791	-0,315664584	0,0000606	0,000208
FKBP14	protein_coding	ENSG00000106080	0,262738843	0,000061	0,000209
DCAF8	protein_coding	ENSG00000132716	-0,249407159	0,0000609	0,000209
MED25	protein_coding	ENSG00000104973	-0,31115009	0,0000609	0,000209
PLEKHA8P1	transcribed_processed_	ENSG00000134297	0,403105503	0,0000607	0,000209
GLYATL1	protein_coding	ENSG00000166840	0,795878991	0,0000612	0,00021
KLHL7	protein_coding	ENSG00000122550	0,270558889	0,000061	0,00021
MIGA1	protein_coding	ENSG00000180488	-0,262477	0,0000613	0,00021
MAPK7	protein_coding	ENSG00000166484	-0,341060418	0,0000611	0,00021
TMEM242	protein_coding	ENSG00000215712	0,270334121	0,0000622	0,000214
DUSP14	protein_coding	ENSG00000276023	0,199180427	0,0000624	0,000214
AC092168.1	lincRNA	ENSG00000228488	3,68206762	0,0000626	0,000215
ZNF510	protein_coding	ENSG00000081386	0,245046483	0,0000628	0,000215
DIAPH1	protein_coding	ENSG00000131504	-0,217474042	0,0000629	0,000216
BX470102.1	antisense	ENSG00000238279	2,369644459	0,0000632	0,000217
AC012603.1	lincRNA	ENSG00000271849	2,668659205	0,0000634	0,000217
NPTN	protein_coding	ENSG00000156642	-0,202779849	0,0000634	0,000217
LINC01160	lincRNA	ENSG00000231346	2,606724033	0,0000639	0,000219
AC080038.1	protein_coding	ENSG00000011028	0,333462661	0,0000639	0,000219
GATB	protein_coding	ENSG00000059691	-0,271121398	0,000064	0,000219
LINC01225	transcribed_unitary_pse	ENSG00000260386	5,056104806	0,000064	0,000219
AC079922.2	lincRNA	ENSG00000237753	0,48567185	0,0000642	0,00022
RASGRP3	protein_coding	ENSG00000152689	1,952355996	0,0000644	0,00022
ADRA2C	protein_coding	ENSG00000184160	-0,495167415	0,0000643	0,00022
AC007389.1	processed_transcript	ENSG00000204929	2,477517548	0,0000645	0,000221
MIR4435-2HG	lincRNA	ENSG00000172965	-0,181332162	0,000065	0,000222
LARP1P1	processed_pseudogene	ENSG00000217159	3,274108992	0,0000649	0,000222
COL20A1	protein_coding	ENSG00000101203	3,756079749	0,000065	0,000222
FHDC1	protein_coding	ENSG00000137460	1,312248472	0,0000647	0,000222
KDM4D	protein_coding	ENSG00000186280	0,676602872	0,0000652	0,000223
IFFO1	protein_coding	ENSG00000010295	0,641588423	0,0000653	0,000223
NECTIN3	protein_coding	ENSG00000177707	0,245123356	0,0000653	0,000223
TOMM22	protein_coding	ENSG00000100216	-0,280672454	0,0000652	0,000223
TP53I13	protein_coding	ENSG00000167543	-0,383914531	0,0000651	0,000223

LINC01139	lincRNA	ENSG00000215808	-0,28834891	0,0000655	0,000224
ZBTB25	protein_coding	ENSG00000089775	-0,287067237	0,0000655	0,000224
ISCA1	protein_coding	ENSG00000135070	-0,336981588	0,0000657	0,000224
AC005261.1	lincRNA	ENSG00000268205	-0,466538967	0,0000658	0,000225
CBLN2	protein_coding	ENSG00000141668	-0,484120181	0,0000659	0,000225
LINC00706	lincRNA	ENSG00000281186	0,899983331	0,0000662	0,000226
AK1	protein_coding	ENSG00000106992	0,630579955	0,0000661	0,000226
FAM86B3P	transcribed_unprocesse	ENSG00000173295	0,742009665	0,0000663	0,000226
LINGO1	protein_coding	ENSG00000169783	3,857040541	0,0000666	0,000227
MCTP2	protein_coding	ENSG00000140563	-0,861253761	0,0000664	0,000227
RPL23AP7	transcribed_processed_	ENSG00000240356	-0,339556332	0,0000666	0,000227
ADPRM	protein_coding	ENSG00000170222	0,292647087	0,0000668	0,000228
DNMBP-AS1	antisense	ENSG00000227695	3,415741624	0,0000672	0,000229
TEX264	protein_coding	ENSG00000164081	-0,298276214	0,000067	0,000229
AC104411.1	antisense	ENSG00000241770	5,011774428	0,0000676	0,00023
KCNB1	protein_coding	ENSG00000158445	0,529891826	0,0000676	0,00023
PUS10	protein_coding	ENSG00000162927	0,358907331	0,0000674	0,00023
PLD1	protein_coding	ENSG00000075651	0,254928391	0,0000676	0,00023
REXO4	protein_coding	ENSG00000148300	-0,227944059	0,0000676	0,00023
ILF2	protein_coding	ENSG00000143621	-0,253986547	0,0000674	0,00023
ZNF687-AS1	antisense	ENSG00000232671	0,919433751	0,000068	0,000231
OSMR-AS1	lincRNA	ENSG00000249740	1,10542438	0,000068	0,000231
STYX	protein_coding	ENSG00000198252	-0,302796676	0,0000677	0,000231
G3BP2	protein_coding	ENSG00000138757	0,164637869	0,000068	0,000232
AC067931.2	TEC	ENSG00000280326	5,001795983	0,000068	0,000232
NCMAP	protein_coding	ENSG00000184454	2,666542906	0,0000685	0,000233
GRINA	protein_coding	ENSG00000178719	0,286585048	0,0000688	0,000234
OGFR-AS1	antisense	ENSG00000229873	4,991566948	0,000069	0,000235
XKR6	protein_coding	ENSG00000171044	0,580042137	0,000069	0,000235
PAF1	protein_coding	ENSG00000006712	-0,14945533	0,0000692	0,000235
C10orf88	protein_coding	ENSG00000119965	0,215108538	0,0000695	0,000236
AP001458.1	antisense	ENSG00000254964	3,359745336	0,0000698	0,000237
AL358472.5	antisense	ENSG00000284738	1,648765924	0,0000698	0,000237
AL157400.2	antisense	ENSG00000232936	2,531528527	0,0000702	0,000238
POLR1A	protein_coding	ENSG00000068654	-0,289103838	0,0000699	0,000238
SSH3	protein_coding	ENSG00000172830	-0,43110842	0,0000701	0,000238
OTOF	protein_coding	ENSG00000115155	5,039868003	0,0000705	0,00024
ABCB9	protein_coding	ENSG00000150967	0,309909192	0,0000708	0,00024
LYRM2	protein_coding	ENSG00000083099	-0,2375142	0,0000708	0,00024
ADRA1B	protein_coding	ENSG00000170214	1,015145612	0,0000709	0,000241

PHB2	protein_coding	ENSG00000215021	-0,259792439	0,0000711	0,000241
USP21	protein_coding	ENSG00000143258	-0,343555817	0,000071	0,000241
AKAP5	protein_coding	ENSG00000179841	-0,506177071	0,0000709	0,000241
MOV10L1	protein_coding	ENSG00000073146	3,063750859	0,0000712	0,000242
MRPS23	protein_coding	ENSG00000181610	-0,284878383	0,0000714	0,000242
HOXB13	protein_coding	ENSG00000159184	-0,310578927	0,0000713	0,000242
CRIM1-DT	lincRNA	ENSG00000260025	0,430487467	0,0000716	0,000243
TTC28-AS1	processed_transcript	ENSG00000235954	-0,362811018	0,0000719	0,000243
BCL2L14	protein_coding	ENSG00000121380	3,315744975	0,0000716	0,000243
TMEM253	protein_coding	ENSG00000232070	1,986474263	0,0000716	0,000243
HHLA3	protein_coding	ENSG00000197568	0,687576753	0,0000715	0,000243
ZNF202	protein_coding	ENSG00000166261	-0,291541978	0,0000717	0,000243
ASTE1	protein_coding	ENSG00000034533	-0,30632803	0,0000715	0,000243
HPF1	protein_coding	ENSG00000056050	-0,325390222	0,0000717	0,000243
AC017002.3	lincRNA	ENSG00000240350	2,658625663	0,000072	0,000244
AL162727.1	lincRNA	ENSG00000233817	1,169130279	0,0000722	0,000244
SNHG15	lincRNA	ENSG00000232956	-0,256461682	0,0000722	0,000244
PLCD3	protein_coding	ENSG00000161714	0,287115306	0,0000732	0,000248
LY6E	protein_coding	ENSG00000160932	-0,299995615	0,0000732	0,000248
GSAP	protein_coding	ENSG00000186088	0,466941146	0,0000735	0,000249
KIAA1191	protein_coding	ENSG00000122203	0,171495703	0,0000736	0,000249
CDK12	protein_coding	ENSG00000167258	-0,242664129	0,0000736	0,000249
RNF5	protein_coding	ENSG00000204308	-0,258173168	0,0000735	0,000249
BCKDK	protein_coding	ENSG00000103507	-0,25982155	0,0000737	0,000249
SULT1C2P1	transcribed_unprocesse	ENSG00000237223	4,98283443	0,0000735	0,000249
HPS1	protein_coding	ENSG00000107521	0,316145887	0,0000739	0,00025
CDH3	protein_coding	ENSG00000062038	2,175579316	0,0000744	0,000251
ENSA	protein_coding	ENSG00000143420	-0,1698439	0,0000742	0,000251
RUVBL2	protein_coding	ENSG00000183207	-0,213755412	0,0000742	0,000251
BAALC-AS1	antisense	ENSG00000247081	0,976069159	0,0000745	0,000252
R3HCC1	protein_coding	ENSG00000104679	-0,235098675	0,0000746	0,000252
ENG	protein_coding	ENSG00000106991	-0,248000416	0,0000747	0,000252
AL031587.5	TEC	ENSG00000278948	0,429330548	0,0000745	0,000252
AC009812.1	antisense	ENSG00000251867	1,555274546	0,0000749	0,000253
AC139768.1	protein_coding	ENSG00000184271	0,597951817	0,0000748	0,000253
RPF2	protein_coding	ENSG00000197498	-0,286489923	0,0000749	0,000253
AASS	protein_coding	ENSG00000008311	0,250842473	0,0000752	0,000254
AP000866.1	antisense	ENSG00000245498	0,995820554	0,0000755	0,000255
MIR222HG	lincRNA	ENSG00000270069	-0,499171094	0,0000755	0,000255
METTL18	protein_coding	ENSG00000171806	-0,414488928	0,0000757	0,000255

PRR9	protein_coding	ENSG00000203783	-0,763155155	0,0000758	0,000256
CATSPER2P1	transcribed_unprocesse	ENSG00000205771	0,78040578	0,000076	0,000256
AL354718.1	processed_pseudogene	ENSG00000275160	1,691047733	0,0000763	0,000257
ADCY6	protein_coding	ENSG00000174233	0,346212871	0,0000763	0,000257
DENND1A	protein_coding	ENSG00000119522	0,217002466	0,0000762	0,000257
MPDU1	protein_coding	ENSG00000129255	-0,270219674	0,0000761	0,000257
PMS1	protein_coding	ENSG00000064933	-0,278986893	0,0000761	0,000257
NKIRAS1	protein_coding	ENSG00000197885	0,294917792	0,0000766	0,000258
RBM33	protein_coding	ENSG00000184863	0,227108729	0,000077	0,000259
ATG2A	protein_coding	ENSG00000110046	0,394615811	0,0000775	0,000261
WDR45	protein_coding	ENSG00000196998	0,284024623	0,0000776	0,000261
ASB8	protein_coding	ENSG00000177981	-0,197693932	0,0000776	0,000261
SART3	protein_coding	ENSG00000075856	-0,206024701	0,0000775	0,000261
AC011921.1	antisense	ENSG00000259349	2,114861481	0,0000779	0,000262
CPNE8-AS1	antisense	ENSG00000257718	0,759319615	0,0000782	0,000263
PRRG4	protein_coding	ENSG00000135378	3,003085612	0,0000782	0,000263
MAPK8IP1	protein_coding	ENSG00000121653	-0,415769948	0,0000785	0,000264
Z93930.2	antisense	ENSG00000226471	0,90324561	0,0000787	0,000265
AC116312.1	antisense	ENSG00000272411	3,33364266	0,0000794	0,000267
DPYD	protein_coding	ENSG00000188641	0,170497096	0,0000795	0,000267
AL355388.2	antisense	ENSG00000273002	1,298778944	0,0000799	0,000269
ACD	protein_coding	ENSG00000102977	-0,31761682	0,0000801	0,000269
TNFSF13B	protein_coding	ENSG00000102524	2,018122063	0,0000805	0,000271
ANAPC10	protein_coding	ENSG00000164162	0,279453717	0,0000808	0,000271
RUFY1	protein_coding	ENSG00000176783	-0,203950852	0,0000805	0,000271
CHD2	protein_coding	ENSG00000173575	-0,2991393	0,0000806	0,000271
SPATA2	protein_coding	ENSG00000158480	0,393883964	0,0000811	0,000272
MAFG-DT	bidirectional_promoter_	ENSG00000265688	-0,478089564	0,0000812	0,000273
AC025419.1	lincRNA	ENSG00000250748	1,220917902	0,0000814	0,000273
ZNF296	protein_coding	ENSG00000170684	0,718644821	0,0000814	0,000273
TRAPPC3L	protein_coding	ENSG00000173626	5,230310108	0,0000817	0,000274
ACIN1	protein_coding	ENSG00000100813	-0,258013201	0,0000816	0,000274
SURF2	protein_coding	ENSG00000148291	-0,263481938	0,0000821	0,000275
CMTM4	protein_coding	ENSG00000183723	-0,325839536	0,0000819	0,000275
ACTA2-AS1	antisense	ENSG00000180139	1,58627128	0,0000824	0,000276
PSMC3	protein_coding	ENSG00000165916	-0,171995814	0,0000824	0,000276
EMC8	protein_coding	ENSG00000131148	-0,240987322	0,0000821	0,000276
BICD2	protein_coding	ENSG00000185963	-0,277157223	0,0000822	0,000276
ANKMY1	protein_coding	ENSG00000144504	-0,402860242	0,0000823	0,000276
UTP6	protein_coding	ENSG00000108651	-0,264686843	0,0000826	0,000277

MYLK-AS1	antisense	ENSG00000239523	0,58160918	0,0000828	0,000278
NOD2	protein_coding	ENSG00000167207	5,029595449	0,0000831	0,000278
RGP1	protein_coding	ENSG00000107185	0,305521759	0,0000828	0,000278
RIBC2	protein_coding	ENSG00000128408	-0,565857909	0,0000831	0,000278
OTOA	protein_coding	ENSG00000155719	3,627553903	0,0000832	0,000279
SSH2	protein_coding	ENSG00000141298	-0,215937967	0,0000838	0,00028
SMPD4	protein_coding	ENSG00000136699	-0,246520796	0,0000835	0,00028
DIPK1A	protein_coding	ENSG00000154511	-0,327707209	0,0000839	0,000281
AL133215.2	lincRNA	ENSG00000273162	-1,419515395	0,0000841	0,000282
AC020916.1	antisense	ENSG00000267519	-0,652799377	0,0000846	0,000283
CYP2U1	protein_coding	ENSG00000155016	0,411465665	0,000085	0,000284
UFC1	protein_coding	ENSG00000143222	-0,248018115	0,0000853	0,000285
HSD17B8	protein_coding	ENSG00000204228	-0,390331755	0,0000852	0,000285
IL12B	protein_coding	ENSG00000113302	3,267594804	0,0000856	0,000286
RFPL3S	lincRNA	ENSG00000205853	1,768386712	0,0000859	0,000287
ARMC1	protein_coding	ENSG00000104442	-0,304010812	0,000086	0,000287
MAP3K6	protein_coding	ENSG00000142733	-0,310003467	0,0000858	0,000287
AC004917.1	sense_overlapping	ENSG00000243797	1,729512494	0,0000858	0,000287
MAN2B1	protein_coding	ENSG00000104774	0,287612283	0,0000863	0,000288
LYG1	protein_coding	ENSG00000144214	1,678659049	0,0000868	0,00029
LRRC2	protein_coding	ENSG00000163827	1,614217794	0,0000867	0,00029
LINC00158	lincRNA	ENSG00000185433	3,280027493	0,0000871	0,000291
SLC28A3	protein_coding	ENSG00000197506	0,508182514	0,0000872	0,000291
NIFK	protein_coding	ENSG00000155438	-0,252402176	0,0000873	0,000291
ZNF142	protein_coding	ENSG00000115568	-0,312282675	0,0000872	0,000291
ZNF812P	transcribed_unprocesse	ENSG00000224689	3,184429035	0,0000871	0,000291
SGTA	protein_coding	ENSG00000104969	-0,182784698	0,0000878	0,000293
CTSF	protein_coding	ENSG00000174080	-0,288313793	0,0000877	0,000293
TMOD2	protein_coding	ENSG00000128872	-0,300029068	0,000088	0,000293
SERPINB6	protein_coding	ENSG00000124570	-0,200593846	0,0000882	0,000294
UBN1	protein_coding	ENSG00000118900	-0,300968883	0,0000881	0,000294
AP005329.2	antisense	ENSG00000265399	2,135654743	0,0000886	0,000295
POLH	protein_coding	ENSG00000170734	0,140800706	0,0000885	0,000295
CFAP97	protein_coding	ENSG00000164323	-0,265902702	0,0000886	0,000295
CD302	protein_coding	ENSG00000241399	-0,344689716	0,0000892	0,000297
TTYH2	protein_coding	ENSG00000141540	-0,449221718	0,0000891	0,000297
AC243964.2	antisense	ENSG00000266903	1,31540952	0,0000894	0,000298
PTDSS2	protein_coding	ENSG00000174915	-0,227808195	0,0000894	0,000298
PRR3	protein_coding	ENSG00000204576	-0,23784395	0,0000893	0,000298
DNAH8	protein_coding	ENSG00000124721	3,314490992	0,0000898	0,000299

PSG8	protein_coding	ENSG00000124467	2,117808995	0,0000899	0,000299
KTN1	protein_coding	ENSG00000126777	0,215886879	0,0000898	0,000299
C19orf24	protein_coding	ENSG00000228300	-0,309730636	0,0000899	0,000299
CLCN6	protein_coding	ENSG00000011021	0,374696064	0,0000901	0,0003
QARS	protein_coding	ENSG00000172053	-0,144040151	0,0000901	0,0003
SCAF8	protein_coding	ENSG00000213079	-0,195512748	0,0000905	0,000301
MED24	protein_coding	ENSG00000008838	-0,224452439	0,0000906	0,000301
MBLAC2	protein_coding	ENSG00000176055	0,311333432	0,000091	0,000303
FUCA1	protein_coding	ENSG00000179163	0,308084392	0,0000913	0,000303
BEX4	protein_coding	ENSG00000102409	0,206779494	0,0000913	0,000303
EPHB4	protein_coding	ENSG00000196411	-0,250354844	0,000091	0,000303
SLC5A8	protein_coding	ENSG00000256870	4,886000004	0,0000916	0,000304
AC007879.4	lincRNA	ENSG00000240440	1,929269244	0,0000917	0,000305
RNF219	protein_coding	ENSG00000152193	-0,233433953	0,0000918	0,000305
ANKRD46	protein_coding	ENSG00000186106	0,375448501	0,0000927	0,000308
STMP1	protein_coding	ENSG00000243317	-0,264535906	0,0000926	0,000308
BICC1	protein_coding	ENSG00000122870	-0,19805578	0,000093	0,000309
HK2	protein_coding	ENSG00000159399	-0,243437608	0,0000932	0,000309
OAF	protein_coding	ENSG00000184232	-0,515137304	0,0000937	0,000311
IFIT1B	protein_coding	ENSG00000204010	5,193338559	0,000094	0,000312
ZNF433-AS1	processed_transcript	ENSG00000219665	0,381681298	0,0000945	0,000313
MED17	protein_coding	ENSG00000042429	-0,513122273	0,0000944	0,000313
VPS33A	protein_coding	ENSG00000139719	-0,269729112	0,0000953	0,000316
TPM1-AS	antisense	ENSG00000259498	2,088220902	0,0000958	0,000318
LTN1	protein_coding	ENSG00000198862	-0,204300505	0,0000962	0,000319
NAPEPLD	protein_coding	ENSG00000161048	-0,467721623	0,0000961	0,000319
SLC16A2	protein_coding	ENSG00000147100	0,206667179	0,0000964	0,00032
LINC02004	lincRNA	ENSG00000240006	1,189850104	0,0000973	0,000323
TAF4B	protein_coding	ENSG00000141384	0,217822011	0,0000974	0,000323
VEGFD	protein_coding	ENSG00000165197	3,619933505	0,0000977	0,000324
IGSF23	protein_coding	ENSG00000216588	2,141369325	0,0000983	0,000326
FBXO3	protein_coding	ENSG00000110429	-0,275217122	0,0000983	0,000326
CLUH	protein_coding	ENSG00000132361	-0,307229859	0,0000985	0,000326
LRR1	protein_coding	ENSG00000165501	-0,297482278	0,0000989	0,000327
EFNA4	protein_coding	ENSG00000243364	-0,811271043	0,0000986	0,000327
AC137936.2	transcribed_unitary_pse	ENSG00000283511	1,948994533	0,0000989	0,000327
MOV10	protein_coding	ENSG00000155363	-0,276331299	0,000099	0,000328
TNRC6A	protein_coding	ENSG00000090905	-0,29552741	0,0000991	0,000328
CACNA1A	protein_coding	ENSG00000141837	1,496189968	0,0000995	0,000329
AL031777.3	protein_coding	ENSG00000282988	0,615653527	0,0000995	0,000329

PTPN12	protein_coding	ENSG00000127947	0,243718636	0,0000994	0,000329
ZNF514	protein_coding	ENSG00000144026	-0,331849383	0,0000997	0,00033
CA3	protein_coding	ENSG00000164879	3,711818504	0,000100319	0,000331648
NGF-AS1	antisense	ENSG00000228035	4,819837546	0,000100677	0,000332778
NISCH	protein_coding	ENSG00000010322	0,348130166	0,000100858	0,000333321
OSGEP	protein_coding	ENSG00000092094	-0,212560951	0,000100987	0,000333696
DUSP23	protein_coding	ENSG00000158716	-0,362642063	0,000101441	0,000335141
F12	protein_coding	ENSG00000131187	-0,429900867	0,00010153	0,000335382
AC002467.1	antisense	ENSG00000241764	0,726195104	0,000101691	0,00033586
AP000695.1	antisense	ENSG00000230479	0,640665614	0,000101941	0,000336574
FBXL5	protein_coding	ENSG00000118564	-0,190067194	0,000102037	0,00033684
FN3KRP	protein_coding	ENSG00000141560	-0,211324968	0,000102578	0,000338571
OAZ1	protein_coding	ENSG00000104904	0,248661362	0,00010279	0,000339216
PSG10P	transcribed_unprocesse	ENSG00000248257	3,227085123	0,000102985	0,000339805
RDH13	protein_coding	ENSG00000160439	-0,355013071	0,000103223	0,000340534
LINC01827	lincRNA	ENSG00000234919	2,59103082	0,000103783	0,000342326
B3GNT9	protein_coding	ENSG00000237172	-0,2860316	0,000103856	0,000342513
MZT1	protein_coding	ENSG00000204899	-0,41361451	0,00010389	0,000342568
KBTBD11	protein_coding	ENSG00000176595	-1,006468416	0,000104063	0,000343084
NDRG3	protein_coding	ENSG00000101079	-0,202663414	0,000104496	0,000344458
BCO2	protein_coding	ENSG00000197580	1,360292485	0,000104819	0,000345467
PBX2	protein_coding	ENSG00000204304	-0,227563406	0,000104946	0,000345828
SHPRH	protein_coding	ENSG00000146414	-0,268980154	0,000105257	0,0003468
POU5F1B	protein_coding	ENSG00000212993	2,895426445	0,000105854	0,000348708
CRYM	protein_coding	ENSG00000103316	1,453041041	0,000105895	0,000348787
NDST1	protein_coding	ENSG00000070614	-0,293475751	0,000105948	0,000348908
LINC01510	lincRNA	ENSG00000231210	2,202482482	0,000105975	0,000348939
DMAC2L	protein_coding	ENSG00000125375	-0,266667133	0,000106181	0,000349524
NAGS	protein_coding	ENSG00000161653	-0,304256419	0,000106187	0,000349524
LRRIQ3	protein_coding	ENSG00000162620	0,49845945	0,000106723	0,000351233
LINC01010	lincRNA	ENSG00000236700	2,440874695	0,000106848	0,000351586
MRPL50	protein_coding	ENSG00000136897	-0,305756484	0,000106864	0,000351586
PKHD1	protein_coding	ENSG00000170927	1,122036834	0,000106995	0,000351959
TIMP1	protein_coding	ENSG00000102265	0,20788894	0,000107127	0,000352338
DNAJC15	protein_coding	ENSG00000120675	2,827860944	0,000107211	0,000352557
SMYD2	protein_coding	ENSG00000143499	-0,196093484	0,000107522	0,000353522
TFB2M	protein_coding	ENSG00000162851	-0,29472171	0,000107545	0,000353543
NPAS2	protein_coding	ENSG00000170485	-0,334212889	0,000107598	0,00035366
HLA-F	protein_coding	ENSG00000204642	0,551086405	0,000107858	0,000354458
SLC6A4	protein_coding	ENSG00000108576	2,969084459	0,000107889	0,000354504

KCNJ15	protein_coding	ENSG00000157551	5,169306306	0,000108304	0,000355809
SNX7	protein_coding	ENSG00000162627	-0,227657851	0,000108452	0,000356236
CDC20B	protein_coding	ENSG00000164287	1,96017808	0,000108525	0,000356421
LTBP3	protein_coding	ENSG00000168056	0,462451758	0,000108853	0,000357442
AGPS	protein_coding	ENSG00000018510	-0,19521125	0,000108954	0,000357715
RANBP1	protein_coding	ENSG00000099901	-0,227584816	0,000109096	0,000358124
AP3D1	protein_coding	ENSG00000065000	0,291960176	0,000109135	0,000358194
DYRK2	protein_coding	ENSG00000127334	-0,30471169	0,000109416	0,000359058
GLT8D2	protein_coding	ENSG00000120820	1,939429677	0,000109784	0,000360211
MRPS9	protein_coding	ENSG00000135972	0,205665741	0,000109891	0,000360503
RBM48	protein_coding	ENSG00000127993	0,318046679	0,000109982	0,000360743
CIAO2A	protein_coding	ENSG00000166797	-0,359922749	0,000110005	0,000360763
PELP1	protein_coding	ENSG00000141456	-0,255485797	0,000110071	0,000360919
AC099811.3	lincRNA	ENSG00000267658	1,964442806	0,000110267	0,000361506
EFCAB11	protein_coding	ENSG00000140025	-0,39545901	0,000110921	0,00036359
NUP88	protein_coding	ENSG00000108559	-0,22488177	0,000111007	0,000363816
WARS2-AS1	antisense	ENSG00000231365	-0,225595003	0,000111137	0,000364181
AC022217.1	processed_pseudogene	ENSG00000213386	2,883483304	0,000111284	0,000364606
FAM72C	protein_coding	ENSG00000263513	-0,954636831	0,000111369	0,000364828
PCSK9	protein_coding	ENSG00000169174	4,902658965	0,000111483	0,000365143
PAGR1	protein_coding	ENSG00000280789	-0,725460543	0,000111827	0,000366209
IL36G	protein_coding	ENSG00000136688	3,146204845	0,000112023	0,000366792
RAD17P2	processed_pseudogene	ENSG00000223724	4,938927369	0,000112232	0,00036742
AC096564.1	antisense	ENSG00000245293	2,416159914	0,000112255	0,000367436
OFCC1	transcribed_unitary_pse	ENSG00000181355	1,374983579	0,000112419	0,000367913
CASP6	protein_coding	ENSG00000138794	-0,378386771	0,000112452	0,000367962
PANDAR	lincRNA	ENSG00000281450	3,161167408	0,000112654	0,000368508
RIOK1	protein_coding	ENSG00000124784	-0,219965566	0,000112638	0,000368508
AC073130.1	processed_transcript	ENSG00000237870	-0,484895202	0,000112715	0,000368588
ERBB4	protein_coding	ENSG00000178568	1,933273352	0,0001127	0,000368588
ISOC1	protein_coding	ENSG00000066583	-0,232174525	0,000112786	0,000368763
IER5	protein_coding	ENSG00000162783	0,203441154	0,00011347	0,000370939
MAPT	protein_coding	ENSG00000186868	-0,486731232	0,000113811	0,000371995
AL683813.1	lincRNA	ENSG00000232611	0,835726986	0,00011434	0,000373664
AL355916.1	lincRNA	ENSG00000232774	1,751334699	0,000114444	0,000373944
HMOX2	protein_coding	ENSG00000103415	-0,200309126	0,000114884	0,000375322
LYRM4	protein_coding	ENSG00000214113	-0,286361712	0,000115421	0,000377017
ATG9A	protein_coding	ENSG00000198925	0,284635863	0,000115478	0,000377143
ATP7B	protein_coding	ENSG00000123191	-0,25507071	0,000115814	0,000378119
AC009113.1	antisense	ENSG00000259877	-0,718525896	0,000116043	0,000378805

TP63	protein_coding	ENSG00000073282	3,232277578	0,000116238	0,000379383
AC138207.2	antisense	ENSG00000264107	1,102907015	0,000116448	0,000380009
AC010255.3	protein_coding	ENSG00000250803	1,609336619	0,000116861	0,000381294
ATP5MC3	protein_coding	ENSG00000154518	-0,221602108	0,000117172	0,000382248
ZNF547	protein_coding	ENSG00000152433	0,638395577	0,000117548	0,000383414
PPP1R32	protein_coding	ENSG00000162148	1,456275179	0,000117807	0,000384197
DUOX2	protein_coding	ENSG00000140279	3,163619517	0,000117827	0,000384203
DDX19A	protein_coding	ENSG00000168872	-0,272352257	0,000118471	0,00038624
LMO7	protein_coding	ENSG00000136153	-0,181008192	0,000119503	0,000389544
ARMC8	protein_coding	ENSG00000114098	-0,213448585	0,000119988	0,000391061
SLC38A3	protein_coding	ENSG00000188338	4,838506415	0,000120016	0,000391092
AC005726.1	protein_coding	ENSG00000258472	-0,840214332	0,000120487	0,000392564
AC008622.2	antisense	ENSG00000275719	1,425933197	0,000120838	0,000393646
COX10-AS1	processed_transcript	ENSG00000236088	-0,431684198	0,000120898	0,000393777
GALNT10	protein_coding	ENSG00000164574	0,179599859	0,00012097	0,000393949
F2RL1	protein_coding	ENSG00000164251	0,593205477	0,000121354	0,000395139
FSTL4	protein_coding	ENSG00000053108	3,268168913	0,000121461	0,000395424
TXNDC15	protein_coding	ENSG00000113621	-0,218318395	0,000121542	0,000395623
ATP2B2	protein_coding	ENSG00000157087	3,576160047	0,000121699	0,000396071
NCAN	protein_coding	ENSG00000130287	3,18247709	0,000122266	0,000397853
RARB	protein_coding	ENSG00000077092	0,470197375	0,000123188	0,000400727
AL096701.3	antisense	ENSG00000240591	2,369236658	0,000123302	0,000401036
NUMB	protein_coding	ENSG00000133961	0,204376799	0,000123619	0,000402
MYCL	protein_coding	ENSG00000116990	-0,865852365	0,000123848	0,000402682
SETX	protein_coding	ENSG00000107290	0,222637762	0,000123937	0,000402909
SLC30A10	protein_coding	ENSG00000196660	4,852362896	0,000124215	0,000403749
ZNF443	protein_coding	ENSG00000180855	-0,56268754	0,000124742	0,000405396
ZNF30	protein_coding	ENSG00000168661	-0,340379601	0,000124814	0,000405567
AC084117.1	sense_intronic	ENSG00000256006	-1,484136296	0,000124912	0,00040582
KAT14	protein_coding	ENSG00000149474	-0,312377819	0,000125043	0,000406182
TMEM87B	protein_coding	ENSG00000153214	0,199478448	0,00012527	0,000406854
GPAT4	protein_coding	ENSG00000158669	-0,192107153	0,000125575	0,000407781
TMEM256	protein_coding	ENSG00000205544	-0,390493468	0,000126344	0,000410211
AL031055.1	lincRNA	ENSG00000271784	3,527313168	0,000127135	0,000412714
RTCB	protein_coding	ENSG00000100220	-0,165857242	0,000127164	0,000412744
SIRT2	protein_coding	ENSG00000068903	0,257428923	0,000127286	0,000413075
MRPL42	protein_coding	ENSG00000198015	-0,241836496	0,000128181	0,000415914
AC009779.2	antisense	ENSG00000258056	0,471016969	0,000128837	0,00041791
ANK1	protein_coding	ENSG00000029534	1,839477677	0,000128835	0,00041791
AP001972.5	TEC	ENSG00000279117	0,475263096	0,000128877	0,000417973

CBWD6	protein_coding	ENSG00000215126	0,630241014	0,000129352	0,000419383
ZNF192P1	transcribed_unprocesse	ENSG00000226314	0,986018447	0,000129353	0,000419383
TMUB2	protein_coding	ENSG00000168591	-0,225171246	0,000129478	0,000419725
NLRP12	protein_coding	ENSG00000142405	3,246765065	0,000129795	0,000420684
ZSCAN25	protein_coding	ENSG00000197037	0,281376921	0,00012983	0,000420732
PDE5A	protein_coding	ENSG00000138735	0,309363591	0,000130041	0,000421349
CRYZL2P	transcribed_unitary_pse	ENSG00000242193	-0,267192752	0,000130096	0,00042146
VAMP7	protein_coding	ENSG00000124333	0,220197893	0,000130327	0,000422142
CCDC86	protein_coding	ENSG00000110104	-0,207446735	0,000130487	0,000422595
DNPEP	protein_coding	ENSG00000123992	-0,189345518	0,000130899	0,000423861
GPR63	protein_coding	ENSG00000112218	-0,619253559	0,000131466	0,000425632
DCAKD	protein_coding	ENSG00000172992	-0,217946428	0,000131637	0,000426116
AC013451.2	lincRNA	ENSG00000258976	0,825278098	0,00013193	0,000426929
AL355338.1	lincRNA	ENSG00000274605	0,314996222	0,000131923	0,000426929
EHMT1	protein_coding	ENSG00000181090	-0,234040565	0,00013215	0,000427574
CTSW	protein_coding	ENSG00000172543	5,007791286	0,000133162	0,000430783
SLC25A17	protein_coding	ENSG00000100372	-0,284193339	0,000133457	0,000431667
AC022217.2	antisense	ENSG00000253295	3,137393285	0,000134123	0,000433753
MSRB2	protein_coding	ENSG00000148450	0,354072812	0,000134147	0,000433762
MIA3	protein_coding	ENSG00000154305	0,224512969	0,000134444	0,000434654
SHOC1	protein_coding	ENSG00000165181	1,803650591	0,000134675	0,000435334
MTBP	protein_coding	ENSG00000172167	-0,25765897	0,000134735	0,000435459
CNR2	protein_coding	ENSG00000188822	4,799210458	0,000134867	0,000435816
C7	protein_coding	ENSG00000112936	2,205319192	0,00013497	0,000436081
PDCL3	protein_coding	ENSG00000115539	-0,282510789	0,000135004	0,000436122
BAIAP2	protein_coding	ENSG00000175866	0,316998492	0,000135257	0,000436859
TEX21P	transcribed_unitary_pse	ENSG00000234911	-1,03637974	0,000135275	0,000436859
BCLAF3	protein_coding	ENSG00000173681	0,264826577	0,00013533	0,000436968
ARHGAP35	protein_coding	ENSG00000160007	-0,276960907	0,000135906	0,000438759
CIPC	protein_coding	ENSG00000198894	-0,231550099	0,000136148	0,00043947
LINC01376	lincRNA	ENSG00000236204	1,092467034	0,000136745	0,000441329
GAST	protein_coding	ENSG00000184502	4,91183866	0,000137877	0,000444912
RTP4	protein_coding	ENSG00000136514	4,934134648	0,000137929	0,000444928
C1orf61	protein_coding	ENSG00000125462	3,75228907	0,000137908	0,000444928
EXOC7	protein_coding	ENSG00000182473	0,20738347	0,000137947	0,000444928
LINC01546	lincRNA	ENSG00000228459	1,361078281	0,000138143	0,000445488
DENND3	protein_coding	ENSG00000105339	0,264553799	0,000138477	0,000446497
AC022001.3	antisense	ENSG00000272483	2,771764489	0,000138954	0,000447964
FOXP3	protein_coding	ENSG00000049768	2,25481147	0,000139075	0,000448143
ARPIN	protein_coding	ENSG00000242498	-0,34026384	0,000139055	0,000448143

OTUB2	protein_coding	ENSG00000089723	-0,437948154	0,000139033	0,000448143
HES2	protein_coding	ENSG00000069812	0,533117397	0,000139568	0,00044966
CCDC74A	protein_coding	ENSG00000163040	-0,304926791	0,000140289	0,000451913
AC022107.1	TEC	ENSG00000280187	-0,598530011	0,000140659	0,000453035
REV1	protein_coding	ENSG00000135945	-0,253081465	0,000140896	0,000453726
THEM4	protein_coding	ENSG00000159445	-0,205330431	0,000141098	0,000454304
ZNF707	protein_coding	ENSG00000181135	0,484403411	0,000141221	0,00045463
C4orf36	protein_coding	ENSG00000163633	0,395941572	0,000141907	0,000456766
BTLA	protein_coding	ENSG00000186265	2,796915228	0,000142273	0,000457802
TRAF3IP1	protein_coding	ENSG00000204104	-0,331082441	0,000142274	0,000457802
SEMA3D	protein_coding	ENSG00000153993	0,334988793	0,000142401	0,000458139
AL360175.1	lincRNA	ENSG00000233470	1,16667121	0,000142593	0,000458686
HMGN2P5	processed_pseudogene	ENSG00000234664	-0,308707729	0,000142678	0,000458887
AL590666.2	antisense	ENSG00000229953	0,791982971	0,000143038	0,000459975
LINC00184	lincRNA	ENSG00000224939	1,518669615	0,00014369	0,000461997
UOX	transcribed_unitary_pse	ENSG00000240520	2,093610924	0,000144352	0,000464054
ZKSCAN3	protein_coding	ENSG00000189298	0,309569162	0,000144577	0,000464703
SLC51A	protein_coding	ENSG00000163959	3,45115379	0,000145442	0,000467411
HNRNPU	protein_coding	ENSG00000153187	-0,268756319	0,000145627	0,000467932
IGSF3	protein_coding	ENSG00000143061	-0,443901992	0,000145668	0,000467989
SLC41A1	protein_coding	ENSG00000133065	0,268545319	0,000147755	0,000474622
APOBEC3G	protein_coding	ENSG00000239713	0,284680048	0,00014783	0,000474788
SYNPO2	protein_coding	ENSG00000172403	1,309753989	0,000148425	0,000476624
PLA2G15	protein_coding	ENSG00000103066	-0,362463146	0,00014872	0,000477498
ITGBL1	protein_coding	ENSG00000198542	0,493749754	0,000149225	0,000479043
FGFR4	protein_coding	ENSG00000160867	-0,319827537	0,000149448	0,000479685
LDB1	protein_coding	ENSG00000198728	0,275146833	0,000149803	0,000480748
GTF2A2	protein_coding	ENSG00000140307	0,260256193	0,000149839	0,000480787
SLC7A8	protein_coding	ENSG00000092068	3,534000265	0,000149871	0,000480814
UTP14A	protein_coding	ENSG00000156697	-0,234343138	0,000150216	0,000481847
CENPK	protein_coding	ENSG00000123219	-0,287957808	0,000150373	0,000482274
NPM1P9	processed_pseudogene	ENSG00000231066	-0,862891085	0,000150804	0,000483581
RNU11	snRNA	ENSG00000274978	-3,172891187	0,000150962	0,000484012
NRIP1	protein_coding	ENSG00000180530	0,206796787	0,000151608	0,000486009
LINC01191	lincRNA	ENSG00000234199	2,857655497	0,000151878	0,000486797
FTSJ3	protein_coding	ENSG00000108592	-0,198860531	0,000152144	0,000487575
GMNC	protein_coding	ENSG00000205835	1,474146455	0,000152448	0,000488473
PMEL	protein_coding	ENSG00000185664	0,877204951	0,000152653	0,000489051
RPL3	protein_coding	ENSG00000100316	-0,179055831	0,000152719	0,000489189
HRASLS2	protein_coding	ENSG00000133328	2,285898828	0,000153189	0,000490616

WDR11	protein_coding	ENSG00000120008	0,149177876	0,000153381	0,000491155
SLC26A7	protein_coding	ENSG00000147606	2,156386848	0,000153514	0,000491503
LRRC37A6P	transcribed_processed_	ENSG00000230445	-0,524484557	0,00015397	0,000492886
EIF6	protein_coding	ENSG00000242372	-0,254221032	0,000154912	0,000495824
DMPK	protein_coding	ENSG00000104936	0,323263421	0,00015527	0,000496892
LINC01252	lincRNA	ENSG00000247157	4,849190698	0,000155402	0,000497238
CDH24	protein_coding	ENSG00000139880	-0,326644847	0,000155987	0,000499031
LINC01128	processed_transcript	ENSG00000228794	0,455926175	0,000156604	0,000500928
HLA-DMA	protein_coding	ENSG00000204257	-0,29676848	0,000156731	0,000501254
MGAT4B	protein_coding	ENSG00000161013	-0,239091135	0,000157055	0,000502213
SNAI3	protein_coding	ENSG00000185669	3,169543318	0,000157202	0,000502605
JRKL	protein_coding	ENSG00000183340	-0,312295201	0,0001575	0,000503481
XRCC5	protein_coding	ENSG00000079246	-0,195332558	0,000157679	0,000503975
GOLGA1	protein_coding	ENSG00000136935	-0,185708816	0,000157706	0,000503981
ZBTB26	protein_coding	ENSG00000171448	-0,301315188	0,000157956	0,000504703
PCDH7	protein_coding	ENSG00000169851	0,76811724	0,000158032	0,000504864
NR5A1	protein_coding	ENSG00000136931	3,41178665	0,000158147	0,000505153
SNX6	protein_coding	ENSG00000129515	-0,287391418	0,000159499	0,000509393
AC091152.4	TEC	ENSG00000279879	1,354331629	0,000160298	0,000511866
TCEAL8	protein_coding	ENSG00000180964	-0,27268262	0,00016042	0,000512176
PDE3A	protein_coding	ENSG00000172572	1,539660349	0,000161001	0,00051395
SPX	protein_coding	ENSG00000134548	1,848694436	0,000161756	0,000516279
DDOST	protein_coding	ENSG00000244038	0,215777057	0,000162009	0,000517007
HTRA2	protein_coding	ENSG00000115317	-0,249610982	0,000162525	0,000518572
INSR	protein_coding	ENSG00000171105	-0,241071972	0,000162565	0,000518621
DPY30	protein_coding	ENSG00000162961	-0,272344564	0,000163365	0,000521092
ESR1	protein_coding	ENSG00000091831	1,327583762	0,000163407	0,000521143
SIAH2	protein_coding	ENSG00000181788	-0,211514167	0,000163658	0,000521862
C14orf119	protein_coding	ENSG00000179933	-0,253095173	0,000163697	0,000521907
SNORD46	snoRNA	ENSG00000200913	-0,621463231	0,000163887	0,000522433
EFCAB12	protein_coding	ENSG00000172771	1,994879361	0,000163982	0,000522653
RNF13	protein_coding	ENSG00000082996	0,187776128	0,000164426	0,000523987
PRELID2	protein_coding	ENSG00000186314	-0,368279803	0,000164547	0,000524292
LARP7	protein_coding	ENSG00000174720	-0,272954993	0,000164853	0,000525185
AL136309.2	lincRNA	ENSG00000234817	1,023857017	0,000165028	0,000525661
AC074117.1	antisense	ENSG00000234072	-0,740935618	0,000165077	0,000525734
TPRKB	protein_coding	ENSG00000144034	-0,322600888	0,000165265	0,000526251
SP2-AS1	antisense	ENSG00000234494	0,463467779	0,000165354	0,000526419
CCDC125	protein_coding	ENSG00000183323	-0,304329669	0,000165369	0,000526419
NOA1	protein_coding	ENSG00000084092	-0,216382222	0,000165899	0,000528024

RAMP2-AS1	lincRNA	ENSG00000197291	3,116211594	0,000166246	0,000529046
MED6	protein_coding	ENSG00000133997	-0,292371714	0,000167189	0,000531966
PRELID1	protein_coding	ENSG00000169230	-0,207918186	0,000167245	0,000532061
SPTBN1	protein_coding	ENSG00000115306	-0,308275514	0,000167536	0,000532902
C12orf65	protein_coding	ENSG00000130921	-0,225020224	0,000168531	0,000535985
KLHDC8B	protein_coding	ENSG00000185909	-0,36901986	0,000169422	0,000538735
MORF4L1P1	processed_pseudogene	ENSG00000218283	-0,338785184	0,000169468	0,0005388
ZNF460	protein_coding	ENSG00000197714	-0,310047287	0,000169591	0,000539106
NBAS	protein_coding	ENSG00000151779	0,127272161	0,000169719	0,00053943
LINC01473	lincRNA	ENSG00000237877	1,68727126	0,000169821	0,000539671
AC010457.1	antisense	ENSG00000251257	0,277823307	0,000170177	0,000540718
BOLA3-AS1	antisense	ENSG00000225439	-0,871170802	0,000170306	0,000541043
U2AF2	protein_coding	ENSG00000063244	-0,176320368	0,000170773	0,000542442
HIST1H2BN	protein_coding	ENSG00000233822	1,93657165	0,000171128	0,000543486
C5orf56	processed_transcript	ENSG00000197536	0,909258783	0,000171423	0,000544338
INHCAP	transcribed_unprocesse	ENSG00000242337	2,197773346	0,000171766	0,000545345
KCNH4	protein_coding	ENSG00000089558	3,160757467	0,000172317	0,000547008
AC092053.2	antisense	ENSG00000283849	2,306540051	0,000172692	0,000547944
SLC35B4	protein_coding	ENSG00000205060	-0,201079478	0,00017265	0,000547944
MFF	protein_coding	ENSG00000168958	-0,207200634	0,000172679	0,000547944
EBP	protein_coding	ENSG00000147155	-0,200587695	0,000172727	0,000547971
FOXS1	protein_coding	ENSG00000179772	3,153493698	0,000172926	0,000548519
GTPBP3	protein_coding	ENSG00000130299	-0,279889429	0,000173012	0,000548705
SLC1A2	protein_coding	ENSG00000110436	3,091313902	0,000173288	0,000549327
INCA1	protein_coding	ENSG00000196388	0,933007938	0,000173282	0,000549327
AP001528.2	antisense	ENSG00000255471	1,552195102	0,00017351	0,000549946
TMEM178A	protein_coding	ENSG00000152154	3,386678003	0,00017382	0,000550843
OXSM	protein_coding	ENSG00000151093	-0,304120354	0,000174017	0,000551379
AC008966.1	antisense	ENSG00000247796	-0,37297189	0,000174485	0,000552778
ZNF77	protein_coding	ENSG00000175691	-0,392440979	0,000174728	0,000553464
DGKQ	protein_coding	ENSG00000145214	0,359557803	0,000174833	0,000553709
C7orf50	protein_coding	ENSG00000146540	-0,268884239	0,000175517	0,00055579
ARMCX5	protein_coding	ENSG00000125962	0,294436121	0,000175905	0,000556933
VCAM1	protein_coding	ENSG00000162692	3,0974627	0,00017635	0,000558255
MRPL22	protein_coding	ENSG00000082515	-0,248133577	0,00017676	0,000559468
AC103770.1	antisense	ENSG00000254251	3,094804546	0,000177104	0,000560471
RSPO2	protein_coding	ENSG00000147655	3,445549099	0,000177621	0,000562019
LMAN2	protein_coding	ENSG00000169223	0,216497966	0,000177757	0,000562362
AL731556.2	unprocessed_pseudoge	ENSG00000277981	1,738454402	0,000177832	0,000562512
LINC01539	lincRNA	ENSG00000267712	4,755103594	0,000177937	0,000562717

NCOR1	protein_coding	ENSG00000141027	0,218866949	0,000177951	0,000562717
MAB21L3	protein_coding	ENSG00000173212	1,870462498	0,000178275	0,000563655
ZNF550	protein_coding	ENSG00000251369	0,226473168	0,000178671	0,000564818
WDR61	protein_coding	ENSG00000140395	-0,280146213	0,0001787	0,000564822
SCGB1A1	protein_coding	ENSG00000149021	4,733037717	0,00017983	0,000568306
ANO3	protein_coding	ENSG00000134343	1,438055592	0,000180213	0,000569431
P2RX7	protein_coding	ENSG00000089041	3,438011821	0,000180708	0,000570905
IFT140	protein_coding	ENSG00000187535	-0,341144614	0,000181095	0,00057204
MMP14	protein_coding	ENSG00000157227	-0,254529721	0,000181279	0,000572532
PLXNB3	protein_coding	ENSG00000198753	0,52269549	0,000181725	0,000573852
PHKA1	protein_coding	ENSG00000067177	-0,198579131	0,000181785	0,000573955
SETD1A	protein_coding	ENSG00000099381	-0,384949885	0,000182347	0,000575641
CDC23	protein_coding	ENSG00000094880	-0,18244534	0,000182864	0,000577184
COL17A1	protein_coding	ENSG00000065618	0,399719642	0,000182951	0,000577369
TSPAN19	protein_coding	ENSG00000231738	1,921651645	0,00018363	0,000579381
RPS27L	protein_coding	ENSG00000185088	-0,258384094	0,000183645	0,000579381
AP3M1	protein_coding	ENSG00000185009	-0,17164182	0,000184971	0,000583476
GLCCI1	protein_coding	ENSG00000106415	0,338709463	0,000185833	0,000586105
C1QL1	protein_coding	ENSG00000131094	-0,503115789	0,000185885	0,000586178
RHPN2	protein_coding	ENSG00000131941	-0,195623331	0,000186276	0,000587236
SGSM2	protein_coding	ENSG00000141258	-0,342741753	0,000186278	0,000587236
DDX4	protein_coding	ENSG00000152670	4,774264046	0,000187966	0,000592413
SGIP1	protein_coding	ENSG00000118473	2,243662735	0,000187978	0,000592413
SPANXA2-OT1	lincRNA	ENSG00000277215	1,330627027	0,00018849	0,000593845
TRIM5	protein_coding	ENSG00000132256	0,188070832	0,000188477	0,000593845
LRRC7	protein_coding	ENSG00000033122	1,022632949	0,000188593	0,000594077
NDUFB10	protein_coding	ENSG00000140990	-0,241434872	0,000190153	0,000598899
NEBL	protein_coding	ENSG00000078114	2,511241056	0,00019054	0,000600028
HSPA4L	protein_coding	ENSG00000164070	0,187402619	0,000191094	0,00060168
CHMP4C	protein_coding	ENSG00000164695	0,274813274	0,00019126	0,000602109
SAAL1	protein_coding	ENSG00000166788	-0,230899573	0,000192201	0,00060498
SMAP2	protein_coding	ENSG00000084070	0,199469419	0,000192303	0,000605206
IFIT5	protein_coding	ENSG00000152778	0,230436384	0,000194041	0,000610583
COLEC10	protein_coding	ENSG00000184374	0,376023937	0,000194172	0,000610901
TMEM54	protein_coding	ENSG00000121900	-0,247968889	0,000194341	0,000611339
HYDIN2	transcribed_unprocesse	ENSG00000276975	1,041577728	0,000194435	0,000611543
PRNCR1	lincRNA	ENSG00000282961	3,003324153	0,000194858	0,000612779
TTLL7	protein_coding	ENSG00000137941	0,304548493	0,000195275	0,000613941
PYY2	transcribed_unprocesse	ENSG00000237575	2,548316353	0,000195288	0,000613941
AGAP6	protein_coding	ENSG00000204149	0,434531316	0,000195861	0,00061565

MOXD1	protein_coding	ENSG00000079931	-0,129010309	0,000196589	0,000617842
KLHL20	protein_coding	ENSG00000076321	0,203248934	0,000196755	0,000618175
RPN1	protein_coding	ENSG00000163902	0,155945272	0,000196749	0,000618175
TGDS	protein_coding	ENSG00000088451	0,341541357	0,000197444	0,000620246
SP5	protein_coding	ENSG00000204335	-1,772942231	0,000198023	0,000621969
STAB1	protein_coding	ENSG00000010327	3,449938474	0,00019873	0,000624093
C1D	protein_coding	ENSG00000197223	-0,41690853	0,000198917	0,000624586
LETM2	protein_coding	ENSG00000165046	0,458123316	0,000199854	0,000627431
KAZALD1	protein_coding	ENSG00000107821	0,618043965	0,000200545	0,000629503
AL354696.2	antisense	ENSG00000278390	0,947132057	0,000201013	0,000630876
ANKRD40	protein_coding	ENSG00000154945	-0,200179517	0,000201261	0,000631559
PQLC1	protein_coding	ENSG00000122490	-0,308207069	0,000201414	0,000631943
TRAF3IP2	protein_coding	ENSG00000056972	0,346054501	0,000201734	0,000632848
CHRNA10	protein_coding	ENSG00000129749	1,085810873	0,000202625	0,000635546
CRTAP	protein_coding	ENSG00000170275	-0,172102565	0,000202695	0,000635667
GMFG	protein_coding	ENSG00000130755	1,9164415	0,00020356	0,000638282
PEX11G	protein_coding	ENSG00000104883	0,712368851	0,000203727	0,000638709
SYNCRIP	protein_coding	ENSG00000135316	-0,241168091	0,000204011	0,000639501
TUBAL3	protein_coding	ENSG00000178462	1,995962361	0,000204425	0,000640701
AC104794.2	lincRNA	ENSG00000260077	-0,82247067	0,000204608	0,000641115
SLC8A2	protein_coding	ENSG00000118160	3,015196774	0,000204619	0,000641115
DPY19L1	protein_coding	ENSG00000173852	-0,216315008	0,000205095	0,000642436
PITX2	protein_coding	ENSG00000164093	-0,496870977	0,000205103	0,000642436
MIF4GD	protein_coding	ENSG00000125457	-0,291817341	0,000205449	0,000643421
ZNF48	protein_coding	ENSG00000180035	-0,284754094	0,000206398	0,000646294
PRDM8	protein_coding	ENSG00000152784	0,52559734	0,000206461	0,000646336
MMADHC	protein_coding	ENSG00000168288	-0,247046547	0,000206475	0,000646336
PADI2	protein_coding	ENSG00000117115	0,7125643	0,000207546	0,00064959
RPS6KC1	protein_coding	ENSG00000136643	0,164666007	0,00020758	0,000649597
AC090409.2	lincRNA	ENSG00000267316	0,694090154	0,000207815	0,000650233
EBPL	protein_coding	ENSG00000123179	-0,296662147	0,000207866	0,000650295
ORC4	protein_coding	ENSG00000115947	0,237591103	0,000208454	0,000652034
AC090114.2	lincRNA	ENSG00000273270	-0,421598481	0,00020864	0,000652516
C14orf93	protein_coding	ENSG00000100802	-0,33070164	0,000209948	0,000656508
THYN1	protein_coding	ENSG00000151500	-0,305091225	0,000210397	0,000657811
AOC3	protein_coding	ENSG00000131471	0,891772495	0,000210579	0,000658186
COL9A2	protein_coding	ENSG00000049089	0,753266859	0,000210581	0,000658186
PANK1	protein_coding	ENSG00000152782	0,407205405	0,000212009	0,000662548
ALG14	protein_coding	ENSG00000172339	-0,274403141	0,000212237	0,000663161
PCDH12	protein_coding	ENSG00000113555	2,485491489	0,000212663	0,000664391

NAP1L1P3	processed_pseudogene	ENSG00000213371	1,176489693	0,000212827	0,0006648
ZNRD1	protein_coding	ENSG00000066379	-0,290108187	0,000213054	0,00066541
HOOK2	protein_coding	ENSG00000095066	0,381003736	0,000213097	0,00066544
VWA2	protein_coding	ENSG00000165816	5,050177776	0,000213539	0,000666719
IQSEC1	protein_coding	ENSG00000144711	-0,333210654	0,000213962	0,00066794
CENPM	protein_coding	ENSG00000100162	-0,309864898	0,000214153	0,000668434
SOAT1	protein_coding	ENSG00000057252	0,157648398	0,000215145	0,000671428
GAR1	protein_coding	ENSG00000109534	-0,388350743	0,000215674	0,000672975
AL831711.1	lincRNA	ENSG00000283317	-1,123683411	0,000215789	0,000673234
RNF165	protein_coding	ENSG00000141622	4,897454254	0,000215831	0,000673261
TXLNG	protein_coding	ENSG00000086712	-0,205236625	0,000216638	0,000675677
SPART	protein_coding	ENSG00000133104	-0,212568487	0,00021672	0,00067583
RAD54B	protein_coding	ENSG00000197275	-0,411369091	0,000218182	0,000680284
ASPRV1	protein_coding	ENSG00000244617	1,688730822	0,000218472	0,000681086
NUBPL	protein_coding	ENSG00000151413	-0,22484054	0,000218565	0,000681273
AP001189.5	lincRNA	ENSG00000255363	2,178511328	0,000218721	0,000681584
SEC61B	protein_coding	ENSG00000106803	0,237683627	0,000218732	0,000681584
MCUB	protein_coding	ENSG00000005059	-0,282020585	0,000219417	0,000683617
ZBTB34	protein_coding	ENSG00000177125	0,3620638	0,000219495	0,000683756
PKN2-AS1	antisense	ENSG00000237505	1,914897288	0,000219577	0,000683906
SYTL4	protein_coding	ENSG00000102362	-0,34022313	0,000219841	0,000684624
NRG3	protein_coding	ENSG00000185737	2,334058894	0,0002199	0,000684704
LINC00571	lincRNA	ENSG00000223685	1,771600971	0,00022014	0,000685347
TELO2	protein_coding	ENSG00000100726	-0,328778613	0,000220216	0,00068548
OVOL1	protein_coding	ENSG00000172818	3,083108229	0,000220602	0,000686578
KCNIP3	protein_coding	ENSG00000115041	-0,319129036	0,000220872	0,000687314
AXIN2	protein_coding	ENSG00000168646	-0,829587633	0,00022126	0,000688418
DNAJC19	protein_coding	ENSG00000205981	-0,266749591	0,000221707	0,000689702
MYBPC1	protein_coding	ENSG00000196091	1,843842497	0,000222053	0,000690673
TSKU	protein_coding	ENSG00000182704	-0,309247138	0,000222359	0,000691522
AL158801.2	lincRNA	ENSG00000258413	1,78237852	0,000222627	0,000692251
HCAR2	protein_coding	ENSG00000182782	3,066476616	0,000223649	0,000695321
SPATA13	protein_coding	ENSG00000182957	-0,521008875	0,00022379	0,000695656
FBXL17	protein_coding	ENSG00000145743	0,221860022	0,000224954	0,000699166
DPH5	protein_coding	ENSG00000117543	-0,28754225	0,000225206	0,000699845
KRT8P3	processed_pseudogene	ENSG00000254285	-0,294929124	0,000225313	0,00070007
ATP1A1	protein_coding	ENSG00000163399	0,166802173	0,000226363	0,000703228
AC010186.2	transcribed_unprocesse	ENSG00000256594	0,728375327	0,000226863	0,000704674
ZNF442	protein_coding	ENSG00000198342	4,745648563	0,000227357	0,000706048
GOSR1	protein_coding	ENSG00000108587	0,174699576	0,000227374	0,000706048

PLEK	protein_coding	ENSG00000115956	4,71338699	0,00022758	0,000706472
RGS6	protein_coding	ENSG00000182732	2,462335375	0,000227577	0,000706472
C12orf45	protein_coding	ENSG00000151131	0,218788932	0,000228069	0,000707883
IGF2BP3	protein_coding	ENSG00000136231	-0,211304762	0,000228133	0,000707974
IL18RAP	protein_coding	ENSG00000115607	4,654038311	0,000228255	0,000708245
OMG	protein_coding	ENSG00000126861	3,614271456	0,000228903	0,000710151
SLFNL1	protein_coding	ENSG00000171790	3,057403309	0,000229032	0,000710336
PDLIM4	protein_coding	ENSG00000131435	0,335810887	0,000229028	0,000710336
NT5C3B	protein_coding	ENSG00000141698	-0,194257038	0,000229129	0,000710529
STRBP	protein_coding	ENSG00000165209	-0,221284234	0,00022918	0,000710578
PDGFD	protein_coding	ENSG00000170962	0,527790701	0,000229461	0,000711343
XPA	protein_coding	ENSG00000136936	0,261454799	0,000230134	0,000713321
NELFE	protein_coding	ENSG00000204356	-0,229727187	0,000230322	0,000713796
TAAR1	protein_coding	ENSG00000146399	4,761500954	0,000230373	0,000713847
PLAA	protein_coding	ENSG00000137055	0,137687454	0,000230591	0,000714415
FURIN	protein_coding	ENSG00000140564	0,244166617	0,000231362	0,000716695
CHPF2	protein_coding	ENSG00000033100	-0,296892874	0,000231483	0,000716962
ESPN	protein_coding	ENSG00000187017	1,83492451	0,00023215	0,000718919
PPP1R1B	protein_coding	ENSG00000131771	4,583497424	0,000232437	0,000719699
AC011500.3	antisense	ENSG00000269792	4,692444228	0,000232841	0,000720839
GLI1	protein_coding	ENSG00000111087	1,947257945	0,000233663	0,000723275
CASC18	lincRNA	ENSG00000257859	1,896348488	0,000234755	0,000726547
HEIH	lincRNA	ENSG00000278970	-0,273225165	0,000235379	0,000728367
TGFB2-AS1	antisense	ENSG00000232480	1,362890964	0,00023565	0,000729096
FAM86FP	transcribed_unprocesse	ENSG00000164845	0,502001524	0,000235862	0,000729643
TMEM161B-AS1	antisense	ENSG00000247828	0,502118909	0,000236092	0,000730245
UPF3B	protein_coding	ENSG00000125351	-0,256892611	0,000237156	0,000733424
SMAGP	protein_coding	ENSG00000170545	-0,231857454	0,000237217	0,000733502
NWD1	protein_coding	ENSG00000188039	1,441649366	0,000237901	0,000735508
SBF2	protein_coding	ENSG00000133812	-0,193549499	0,000238539	0,000737367
MYL9	protein_coding	ENSG00000101335	0,236555941	0,000239689	0,000740812
AC130343.2	antisense	ENSG00000277597	1,826342984	0,000240059	0,000741781
PPM1A	protein_coding	ENSG00000100614	-0,219757015	0,000240075	0,000741781
ARSJ	protein_coding	ENSG00000180801	0,332022372	0,000241162	0,000745025
PMM2	protein_coding	ENSG00000140650	-0,277437039	0,000241243	0,000745165
CD276	protein_coding	ENSG00000103855	-0,202123472	0,000241388	0,0007455
LINC01939	lincRNA	ENSG00000228799	1,259287637	0,000244037	0,000753455
TPGS2	protein_coding	ENSG00000134779	-0,167422972	0,000245057	0,000756491
KANSL1L	protein_coding	ENSG00000144445	0,314780498	0,000246223	0,000759975
ZNF57	protein_coding	ENSG00000171970	-0,344523214	0,000246655	0,000761195

WSB1	protein_coding	ENSG00000109046	-0,217764517	0,000246821	0,000761478
C21orf58	protein_coding	ENSG00000160298	-0,351582356	0,000246803	0,000761478
AADAT	protein_coding	ENSG00000109576	-0,355690895	0,000248293	0,000765904
AHCY	protein_coding	ENSG00000101444	-0,235616081	0,000249324	0,000768967
EP300	protein_coding	ENSG00000100393	0,258173043	0,000249716	0,000770061
ELMO2	protein_coding	ENSG00000062598	-0,215989889	0,000252625	0,000778914
GUCY1B2	transcribed_unitary_pse	ENSG00000123201	1,640980485	0,00025404	0,00078316
SPATA7	protein_coding	ENSG00000042317	0,380222906	0,000254326	0,000783922
METTL9	protein_coding	ENSG00000197006	-0,212622443	0,000254743	0,000785092
AL445985.1	lincRNA	ENSG00000228741	2,678870073	0,000254987	0,000785723
FEM1A	protein_coding	ENSG00000141965	-0,240131534	0,00025505	0,000785801
CACNA1C	protein_coding	ENSG00000151067	2,337740254	0,000255122	0,000785903
KBTBD12	protein_coding	ENSG00000187715	4,807985152	0,00025585	0,00078803
TRNAU1AP	protein_coding	ENSG00000180098	-0,241497428	0,000256388	0,000789567
MUC5AC	protein_coding	ENSG00000215182	4,634607711	0,000256686	0,000790368
LINC00886	lincRNA	ENSG00000240875	-0,633186805	0,000256789	0,000790564
SH3D19	protein_coding	ENSG00000109686	0,229790893	0,000256911	0,000790821
CDKN2AIPNL	protein_coding	ENSG00000237190	-0,219159584	0,000258191	0,000794644
PTPA	protein_coding	ENSG00000119383	-0,238085527	0,000258368	0,000795069
EEPD1	protein_coding	ENSG00000122547	-0,37993509	0,000259407	0,000798145
C8orf37	protein_coding	ENSG00000156172	0,286229572	0,000259847	0,000799294
ALG10B	protein_coding	ENSG00000175548	-0,308150459	0,000259858	0,000799294
SERPINF1	protein_coding	ENSG00000132386	2,101238489	0,000261272	0,000803521
TVP23C	protein_coding	ENSG00000175106	0,592003468	0,000261487	0,000804064
SIMC1	protein_coding	ENSG00000170085	-0,456703028	0,000261793	0,000804883
BANP	protein_coding	ENSG00000172530	-0,256624155	0,00026271	0,000807581
LINC02363	lincRNA	ENSG00000180712	4,545971451	0,00026476	0,000813762
NSMAF	protein_coding	ENSG00000035681	-0,203269584	0,000264928	0,000814155
KHDC4	protein_coding	ENSG00000132680	-0,274459851	0,000265573	0,000816016
ABCA3	protein_coding	ENSG00000167972	-0,294324304	0,000266707	0,000819376
AC016708.1	antisense	ENSG00000229267	0,878364116	0,000267225	0,000820846
MTHFD2	protein_coding	ENSG00000065911	0,152280868	0,000267644	0,000822011
CFAP97D1	protein_coding	ENSG00000231256	1,48958719	0,000268369	0,000824113
SRSF6	protein_coding	ENSG00000124193	-0,202787533	0,000269001	0,00082593
CES1P2	unprocessed_pseudoge	ENSG00000260765	3,409510828	0,000269323	0,000826795
INKA2-AS1	bidirectional_promoter_	ENSG00000227811	0,772290217	0,000269492	0,000827189
SNHG11	processed_transcript	ENSG00000174365	0,327548414	0,000269678	0,0008276
APOO	protein_coding	ENSG00000184831	-0,216639552	0,000269706	0,0008276
NAA80	protein_coding	ENSG00000243477	-0,409912882	0,000270714	0,000830567
AP1B1	protein_coding	ENSG00000100280	0,236501024	0,000270851	0,000830863

SMC5	protein_coding	ENSG00000198887	-0,24140309	0,000271459	0,000832604
PRKX	protein_coding	ENSG00000183943	-0,20000405	0,000271655	0,000833081
CCNB1IP1	protein_coding	ENSG00000100814	0,281377642	0,000271912	0,000833745
TMEM160	protein_coding	ENSG00000130748	-0,420150495	0,00027257	0,000835638
HNRNPUL2	protein_coding	ENSG00000214753	-0,416201449	0,000272613	0,000835644
AL049543.1	antisense	ENSG00000246350	-0,954572939	0,00027276	0,000835899
POP1	protein_coding	ENSG00000104356	-0,227756054	0,000272778	0,000835899
LINC01165	antisense	ENSG00000229081	2,990189321	0,000272944	0,000836283
ZMYND12	protein_coding	ENSG00000066185	2,281776598	0,000273448	0,000837703
RPGRIP1L	protein_coding	ENSG00000103494	-0,335106655	0,000274226	0,000839962
LRRC25	protein_coding	ENSG00000175489	2,995999231	0,000275152	0,000842568
ACTN4	protein_coding	ENSG00000130402	-0,202503767	0,000275159	0,000842568
CLDN12	protein_coding	ENSG00000157224	-0,247355604	0,000275836	0,000844512
LIPC	protein_coding	ENSG00000166035	2,014218205	0,000275943	0,000844589
PPP1R9A	protein_coding	ENSG00000158528	-0,331008219	0,000275907	0,000844589
AL390755.1	TEC	ENSG00000279965	2,945909235	0,000276459	0,000846043
C11orf24	protein_coding	ENSG00000171067	-0,228105081	0,000276664	0,000846543
EFNB1	protein_coding	ENSG00000090776	0,438885775	0,000276993	0,000847424
BDH1	protein_coding	ENSG00000161267	1,745553667	0,000278075	0,000850607
AC078820.1	lincRNA	ENSG00000258088	1,815564801	0,000279321	0,000854292
ASH1L	protein_coding	ENSG00000116539	0,216976884	0,000279937	0,000856048
SLC2A4RG	protein_coding	ENSG00000125520	-0,280835765	0,000280832	0,000858657
AL450322.2	antisense	ENSG00000229672	2,43962392	0,000281021	0,000859105
SAMD14	protein_coding	ENSG00000167100	1,156209679	0,00028113	0,000859311
BRSK1	protein_coding	ENSG00000160469	0,454444506	0,000283335	0,000865921
ARMH1	protein_coding	ENSG00000198520	2,465595508	0,000284048	0,000867972
AC092809.4	antisense	ENSG00000237101	4,5738548	0,000284429	0,000869006
ZNF569	protein_coding	ENSG00000196437	-0,288412432	0,000284832	0,000870108
AL359551.1	lincRNA	ENSG00000231877	2,199555198	0,000286087	0,000873812
HIF1A	protein_coding	ENSG00000100644	0,170119594	0,000286308	0,000874356
C12orf60	protein_coding	ENSG00000182993	0,515656395	0,000286688	0,000875385
SRP54	protein_coding	ENSG00000100883	0,201629108	0,000286975	0,000876133
AC242376.2	transcribed_unprocesse	ENSG00000274471	-0,865620181	0,000288089	0,000879403
C3AR1	protein_coding	ENSG00000171860	1,47182587	0,000289316	0,000883017
CYP2S1	protein_coding	ENSG00000167600	3,054704423	0,000289646	0,000883893
AC010307.3	antisense	ENSG00000249478	4,605548875	0,000290592	0,000886648
THAP3	protein_coding	ENSG00000041988	-0,291859901	0,000291386	0,000888938
APOL5	protein_coding	ENSG00000128313	4,553564738	0,000292501	0,000892208
TRAK1	protein_coding	ENSG00000182606	-0,220931368	0,000292706	0,0008927
CETN4P	transcribed_unitary_pse	ENSG00000224786	0,936420909	0,000293466	0,000894882

AP4M1	protein_coding	ENSG00000221838	-0,26506307	0,00029428	0,000897101
IBSP	protein_coding	ENSG00000029559	3,314025186	0,000294976	0,000899088
CATSPERG	protein_coding	ENSG00000099338	1,156063231	0,000295406	0,000900265
AGAP3	protein_coding	ENSG00000133612	-0,321583652	0,000295479	0,000900352
THRB-AS1	processed_transcript	ENSG00000228791	1,908732259	0,000295595	0,000900466
COA1	protein_coding	ENSG00000106603	-0,195740452	0,000295608	0,000900466
NPRL2	protein_coding	ENSG00000114388	-0,252989863	0,000295648	0,000900466
TMEM14B	protein_coding	ENSG00000137210	-0,24938974	0,000295763	0,000900683
MAPKAPK5-AS1	lincRNA	ENSG00000234608	-0,219609463	0,000295823	0,000900732
PLXNA4	protein_coding	ENSG00000221866	2,988615323	0,000296536	0,000902769
EI24	protein_coding	ENSG00000149547	-0,130655412	0,000296845	0,000903577
CRTAC1	protein_coding	ENSG00000095713	2,985395308	0,000297241	0,000904645
GXYLT1	protein_coding	ENSG00000151233	-0,297934341	0,000297946	0,000906657
AC105942.1	antisense	ENSG00000235501	0,423666431	0,000298479	0,000908146
PNLIPRP3	protein_coding	ENSG00000203837	1,968882457	0,000298693	0,000908663
C1QBP	protein_coding	ENSG00000108561	-0,278577874	0,000298935	0,000909262
CPSF2	protein_coding	ENSG00000165934	-0,190315195	0,000299413	0,000910581
AC022784.1	lincRNA	ENSG00000248538	1,016058839	0,000299568	0,000910917
STARD9	protein_coding	ENSG00000159433	-0,373345217	0,000299831	0,000911582
SH3BP4	protein_coding	ENSG00000130147	0,171493664	0,000301059	0,00091518
JMJD6	protein_coding	ENSG00000070495	-0,176284263	0,000301667	0,000916892
SPTLC3	protein_coding	ENSG00000172296	-0,266503074	0,000302341	0,000918806
TMEM86A	protein_coding	ENSG00000151117	1,071043774	0,000302487	0,000919113
RBM3	protein_coding	ENSG00000102317	-0,262635968	0,000303433	0,000921851
FRMPD2	protein_coding	ENSG00000170324	3,410507676	0,000303533	0,000922017
UCKL1-AS1	antisense	ENSG00000280213	1,183270725	0,000303727	0,000922471
AC007842.1	processed_pseudogene	ENSG00000269069	1,350784567	0,000304112	0,000923503
PSAT1	protein_coding	ENSG00000135069	3,247723755	0,000304516	0,000924592
FAAH	protein_coding	ENSG00000117480	1,129979999	0,000304628	0,000924795
CIP2A	protein_coding	ENSG00000163507	-0,224280172	0,000304711	0,00092491
SOCS2	protein_coding	ENSG00000120833	-0,261445388	0,000304995	0,000925635
ATRAID	protein_coding	ENSG00000138085	-0,2775936	0,000305084	0,000925769
SMG7-AS1	processed_transcript	ENSG00000232860	1,10861533	0,000305355	0,000926334
ASIC1	protein_coding	ENSG00000110881	-0,467455436	0,000305361	0,000926334
C11orf80	protein_coding	ENSG00000173715	-0,263713159	0,000305456	0,000926486
MINK1	protein_coding	ENSG00000141503	-0,270947735	0,000306064	0,000928194
TMEM81	protein_coding	ENSG00000174529	0,601561483	0,000307558	0,000932588
ZNF581	protein_coding	ENSG00000171425	-0,294149885	0,000307737	0,00093299
IGF2-AS	antisense	ENSG00000099869	3,083684339	0,000309732	0,000938902
GFM1	protein_coding	ENSG00000168827	-0,212765413	0,000310702	0,000941703

TST	protein_coding	ENSG00000128311	0,470575904	0,000311512	0,000944018
BICD1	protein_coding	ENSG00000151746	-0,253126861	0,000311858	0,000944926
TAMM41	protein_coding	ENSG00000144559	-0,243325995	0,000312299	0,000946124
AC022613.2	antisense	ENSG00000259523	0,721221679	0,000312646	0,000947034
LINC00589	lincRNA	ENSG00000251191	3,043176935	0,000313108	0,000948293
AKIP1	protein_coding	ENSG00000166452	-0,205595067	0,000314299	0,000951759
PIK3R5	protein_coding	ENSG00000141506	2,272206731	0,000314819	0,000953196
TNNC2	protein_coding	ENSG00000101470	1,80569562	0,000315941	0,00095645
ESYT1	protein_coding	ENSG00000139641	-0,147809911	0,000317495	0,000960871
RPL41	protein_coding	ENSG00000229117	-0,263699822	0,000317488	0,000960871
DRAM1	protein_coding	ENSG00000136048	0,164107356	0,000318449	0,000963616
IQGAP3	protein_coding	ENSG00000183856	-0,244105967	0,000319367	0,00096625
LBP	protein_coding	ENSG00000129988	2,404456749	0,000319458	0,000966385
GLYCTK	protein_coding	ENSG00000168237	-0,325663019	0,000320082	0,000968128
FAM19A2	protein_coding	ENSG00000198673	0,936143012	0,000320142	0,000968163
PRRC1	protein_coding	ENSG00000164244	-0,20890225	0,000320188	0,000968163
SIVA1	protein_coding	ENSG00000184990	-0,334237121	0,000320546	0,000969083
OASL2P	unitary_pseudogene	ENSG00000283542	4,541707716	0,000320586	0,000969083
EGFLAM-AS1	antisense	ENSG00000249491	2,599239626	0,000321493	0,00097168
AJ239322.1	lincRNA	ENSG00000235615	2,373495986	0,00032164	0,000971982
ZFYVE27	protein_coding	ENSG00000155256	0,302607282	0,000321788	0,000972286
ATXN7	protein_coding	ENSG00000163635	0,297993445	0,000322892	0,000975478
SELENOP	protein_coding	ENSG00000250722	2,086917089	0,000323428	0,000976952
ANXA11	protein_coding	ENSG00000122359	-0,175343689	0,00032374	0,000977752
SPINT2	protein_coding	ENSG00000167642	-0,562842041	0,000324178	0,00097893
SDHAF1	protein_coding	ENSG00000205138	-0,377234791	0,00032433	0,000979243
ZMYND8	protein_coding	ENSG00000101040	-0,221238103	0,00032491	0,000980851
GOLT1A	protein_coding	ENSG00000174567	1,398205385	0,000325177	0,000981513
CA13	protein_coding	ENSG00000185015	0,538147722	0,00032611	0,000984184
HMGB2P1	transcribed_processed_	ENSG00000267736	2,922638503	0,000326746	0,000985959
CEPT1	protein_coding	ENSG00000134255	-0,215918515	0,000327871	0,000989209
N4BP2L1	protein_coding	ENSG00000139597	2,124384351	0,000328178	0,000989988
CLCA4	protein_coding	ENSG00000016602	4,577682736	0,000328425	0,000990588
ZNF253	protein_coding	ENSG00000256771	0,517610203	0,000328961	0,000992059
DENND2A	protein_coding	ENSG00000146966	0,34926703	0,000330751	0,00099731
PRSS57	protein_coding	ENSG00000185198	2,88787562	0,000330809	0,000997338
FNBP1	protein_coding	ENSG00000187239	-0,190034891	0,000331068	0,000997971
ABCD1	protein_coding	ENSG00000101986	0,388803529	0,000331609	0,000999457
STXBP6	protein_coding	ENSG00000168952	0,201802782	0,000331825	0,000999961
AC006504.1	lincRNA	ENSG00000261770	0,94911003	0,000332277	0,001001174

CFAP65	protein_coding	ENSG00000181378	4,704376133	0,000333095	0,001003494
C12orf75	protein_coding	ENSG00000235162	-0,176757057	0,000334165	0,001006567
ATF7IP	protein_coding	ENSG00000171681	0,195527469	0,000334401	0,001007131
TF	protein_coding	ENSG00000091513	3,007038844	0,000334489	0,001007247
TTPAL	protein_coding	ENSG00000124120	0,141923836	0,000335679	0,001010682
ZNF365	protein_coding	ENSG00000138311	0,226119645	0,000337276	0,001015342
CSAG3	protein_coding	ENSG00000268916	3,298284331	0,000339233	0,001021083
RPSAP39	processed_pseudogene	ENSG00000244002	3,297663077	0,000339383	0,001021387
AC116424.1	antisense	ENSG00000250129	0,759618421	0,000339512	0,001021626
MINCR	antisense	ENSG00000253716	-0,453623616	0,000339663	0,001021928
UNC45A	protein_coding	ENSG00000140553	-0,172345191	0,000340204	0,001023407
LINC01348	lincRNA	ENSG00000280587	0,914907466	0,0003406	0,001024446
ATN1	protein_coding	ENSG00000111676	0,480770423	0,00034076	0,00102478
AEBP2	protein_coding	ENSG00000139154	0,20241627	0,000341136	0,001025758
MAFK	protein_coding	ENSG00000198517	0,40495752	0,000341939	0,001028022
MIR600HG	sense_intronic	ENSG00000236901	-0,506553788	0,000343013	0,0010311
C16orf91	protein_coding	ENSG00000174109	-0,334216652	0,0003434	0,001032113
TAF11	protein_coding	ENSG00000064995	-0,220696805	0,000344734	0,001035969
ISY1	protein_coding	ENSG00000240682	-0,216892016	0,000345378	0,0010376
CAPG	protein_coding	ENSG00000042493	-0,222527834	0,000345338	0,0010376
PKP1	protein_coding	ENSG00000081277	3,253747508	0,000345548	0,00103796
VPS37B	protein_coding	ENSG00000139722	0,226079682	0,000345672	0,00103818
PLRG1	protein_coding	ENSG00000171566	0,167095501	0,000345865	0,001038609
CHRNA5	protein_coding	ENSG00000169684	-0,312761803	0,000346054	0,001039023
RBCK1	protein_coding	ENSG00000125826	0,255435707	0,000346706	0,001040828
CAPN2	protein_coding	ENSG00000162909	-0,116164289	0,000347699	0,001043658
TMED8	protein_coding	ENSG00000100580	-0,204752656	0,000348602	0,001046213
TM9SF3	protein_coding	ENSG00000077147	0,184462686	0,000349331	0,001048249
HPRT1	protein_coding	ENSG00000165704	-0,267779058	0,000349532	0,001048697
LINC00467	lincRNA	ENSG00000153363	0,260019897	0,000350249	0,001050628
FBXO48	protein_coding	ENSG00000204923	0,421064217	0,000350278	0,001050628
TGM2	protein_coding	ENSG00000198959	-0,208053289	0,000350362	0,001050728
AC005256.1	lincRNA	ENSG00000267073	2,942520308	0,000350703	0,001051597
RIPOR3	protein_coding	ENSG00000042062	3,252479538	0,000351268	0,001053138
AC005532.1	processed_transcript	ENSG00000230825	1,51330903	0,000352199	0,001055775
GABRA3	protein_coding	ENSG00000011677	0,394201088	0,000352599	0,001056819
RSU1	protein_coding	ENSG00000148484	0,183690281	0,000353275	0,001058536
TRAPPC3	protein_coding	ENSG00000054116	-0,198058369	0,000353228	0,001058536
ZMIZ1	protein_coding	ENSG00000108175	0,389923534	0,000353575	0,00105928
EZH1	protein_coding	ENSG00000108799	-0,22387685	0,000354414	0,001061638

ZNF345	protein_coding	ENSG00000251247	-0,480629973	0,000354487	0,001061702
USP51	protein_coding	ENSG00000247746	-0,468308656	0,000354625	0,001061958
SLC15A4	protein_coding	ENSG00000139370	-0,223981035	0,000354957	0,001062798
BDNF-AS	processed_transcript	ENSG00000245573	1,210208385	0,000355195	0,001063355
DIAPH3	protein_coding	ENSG00000139734	-0,208830706	0,000356	0,001065609
C12orf4	protein_coding	ENSG00000047621	0,214610293	0,000356388	0,001066616
COL4A1	protein_coding	ENSG00000187498	0,274259552	0,00035655	0,001066944
SLC31A1	protein_coding	ENSG00000136868	0,175909447	0,000357572	0,001069846
AC104777.2	lincRNA	ENSG00000237220	2,914313358	0,000357803	0,001070252
SLC44A3	protein_coding	ENSG00000143036	0,45585976	0,000357812	0,001070252
NSMF	protein_coding	ENSG00000165802	-0,214045591	0,000358484	0,001072105
DES	protein_coding	ENSG00000175084	2,499784141	0,000361254	0,001080234
AC119677.1	antisense	ENSG00000229528	1,732757968	0,000361968	0,001082211
LINC01560	lincRNA	ENSG00000196741	-0,858353663	0,000362862	0,001084724
CTNND1	protein_coding	ENSG00000198561	-0,201914216	0,000363647	0,001086755
CLPP	protein_coding	ENSG00000125656	-0,207747764	0,000363637	0,001086755
NCAM2	protein_coding	ENSG00000154654	2,888737885	0,000365629	0,001092518
ZKSCAN4	protein_coding	ENSG00000187626	0,319053788	0,000365792	0,001092848
UBR5	protein_coding	ENSG00000104517	0,207305228	0,000366345	0,001094339
CFAP44	protein_coding	ENSG00000206530	0,415349754	0,000366731	0,001095332
CCT7	protein_coding	ENSG00000135624	-0,166903554	0,000366964	0,00109587
AC126182.2	processed_pseudogene	ENSG00000244441	2,225980662	0,000367402	0,001097019
GIPR	protein_coding	ENSG00000010310	1,436752905	0,000367685	0,001097703
APIP	protein_coding	ENSG00000149089	-0,23885294	0,000367745	0,001097721
PDE11A	protein_coding	ENSG00000128655	0,744213652	0,000368191	0,001098894
KCNV1	protein_coding	ENSG00000164794	1,0332013	0,000368389	0,001099166
PDS5B	protein_coding	ENSG00000083642	-0,235104407	0,000368361	0,001099166
CORO1B	protein_coding	ENSG00000172725	0,207331825	0,000370819	0,001106255
PTBP2	protein_coding	ENSG00000117569	0,200636162	0,000371397	0,001107817
SNX21	protein_coding	ENSG00000124104	-0,285665704	0,000371662	0,001108447
EIF4EBP1	protein_coding	ENSG00000187840	-0,247677705	0,00037303	0,001112202
MAP3K9	protein_coding	ENSG00000006432	-0,337578151	0,000372994	0,001112202
RBM25	protein_coding	ENSG00000119707	-0,266015038	0,000373756	0,001114207
AC016876.1	antisense	ENSG00000233223	-0,51846168	0,000375681	0,001119619
INAVA	protein_coding	ENSG00000163362	1,290444248	0,00037568	0,001119619
AC026412.1	transcribed_unprocesse	ENSG00000188002	0,495533362	0,000375881	0,001120052
C3orf33	protein_coding	ENSG00000174928	0,347736755	0,000377321	0,00112418
TRAPPC8	protein_coding	ENSG00000153339	0,150169491	0,00037786	0,001125623
TINAG	protein_coding	ENSG00000137251	2,973983651	0,000379257	0,001129618
GFPT2	protein_coding	ENSG00000131459	0,723298247	0,00037946	0,001130061

AP002748.3	antisense	ENSG00000255517	0,614435988	0,00037963	0,001130403
SPRY3	protein_coding	ENSG00000168939	1,69860186	0,000380575	0,001133052
TAF9	protein_coding	ENSG00000273841	-0,223660927	0,000380644	0,001133093
CYP1B1	protein_coding	ENSG00000138061	0,641043809	0,000380701	0,001133099
ZC3H7B	protein_coding	ENSG00000100403	0,319376826	0,000380835	0,001133331
KLHL10	protein_coding	ENSG00000161594	1,749492581	0,000381073	0,001133875
AC104506.1	antisense	ENSG00000273204	0,987619503	0,000381644	0,00113541
ALKBH1	protein_coding	ENSG00000100601	0,243077725	0,000383512	0,001140803
AGAP4	protein_coding	ENSG00000188234	0,545116799	0,000384219	0,001142738
ZDHHC16	protein_coding	ENSG00000171307	-0,168307864	0,000384305	0,001142828
FOXK1	protein_coding	ENSG00000164916	-0,354173383	0,000384397	0,001142937
GSPT2	protein_coding	ENSG00000189369	-0,214039244	0,000384828	0,001143889
RPL6	protein_coding	ENSG00000089009	-0,240045861	0,000384808	0,001143889
MUC12	protein_coding	ENSG00000205277	1,591494539	0,000385101	0,001144534
NEURL2	protein_coding	ENSG00000124257	0,837788085	0,000385747	0,001146286
CSF2RB	protein_coding	ENSG00000100368	3,345464473	0,000386039	0,00114699
FAM3A	protein_coding	ENSG00000071889	-0,235299715	0,000386508	0,001148215
WIPI2	protein_coding	ENSG00000157954	-0,138491029	0,000386922	0,001149279
COX15	protein_coding	ENSG00000014919	-0,195907366	0,000387493	0,001150807
AC005837.1	antisense	ENSG00000261335	-0,960602765	0,000387672	0,001151174
WBP4	protein_coding	ENSG00000120688	0,250419838	0,000389155	0,001155409
GAS6-DT	lincRNA	ENSG00000272695	0,354998292	0,00038997	0,001157495
ZNF416	protein_coding	ENSG00000083817	0,302945951	0,000389922	0,001157495
SLC26A11	protein_coding	ENSG00000181045	0,380638282	0,00039037	0,001158515
RERE	protein_coding	ENSG00000142599	0,288511493	0,000390784	0,001159574
CNTROB	protein_coding	ENSG00000170037	-0,289391227	0,000392314	0,001163947
USP31	protein_coding	ENSG00000103404	-0,278953163	0,000392641	0,001164748
LINC01091	lincRNA	ENSG00000249464	4,720112054	0,000393305	0,001166548
CDC73	protein_coding	ENSG00000134371	0,210011529	0,000393881	0,001168088
ANXA8	protein_coding	ENSG00000265190	0,822369139	0,000394012	0,001168307
ZSCAN29	protein_coding	ENSG00000140265	-0,2158784	0,000394376	0,001169218
AC010624.1	sense_overlapping	ENSG00000204666	1,524983405	0,000394967	0,001170801
EXOC6	protein_coding	ENSG00000138190	-0,269119484	0,000395584	0,001172461
TSR2	protein_coding	ENSG00000158526	-0,17813863	0,000396008	0,001173547
SYTL3	protein_coding	ENSG00000164674	0,390524787	0,000396548	0,001174978
TTC1	protein_coding	ENSG00000113312	0,20263683	0,000396965	0,001176045
FABP5P3	transcribed_processed_	ENSG00000241735	4,555634682	0,000397078	0,001176209
BTBD2	protein_coding	ENSG00000133243	-0,259674322	0,000397453	0,00117715
PLPP6	protein_coding	ENSG00000205808	-0,39682353	0,000398467	0,001179982
ANPEP	protein_coding	ENSG00000166825	-0,231948102	0,000400101	0,00118465

ZNF180	protein_coding	ENSG00000167384	-0,232096524	0,000400198	0,001184765
CBFA2T2	protein_coding	ENSG00000078699	0,299668337	0,000400388	0,001185158
AC022509.4	antisense	ENSG00000278095	2,404662111	0,00040098	0,00118674
MRPL52	protein_coding	ENSG00000172590	-0,264915718	0,000401122	0,001186988
STAU2	protein_coding	ENSG00000040341	0,193913046	0,000402253	0,001189991
ITGB1BP1	protein_coding	ENSG00000119185	-0,237008692	0,000402248	0,001189991
SYNJ2BP	protein_coding	ENSG00000213463	-0,195371876	0,000402641	0,001190966
TTC3P1	processed_pseudogene	ENSG00000215105	-0,263024855	0,00040307	0,001192064
FBXO44	protein_coding	ENSG00000132879	-0,292297628	0,00040338	0,00119281
GPAT3	protein_coding	ENSG00000138678	-0,278413195	0,000404076	0,001194694
FAM149B1	protein_coding	ENSG00000138286	0,245685977	0,000404433	0,001195578
SYTL1	protein_coding	ENSG00000142765	1,273132359	0,000404603	0,001195736
PBXIP1	protein_coding	ENSG00000163346	-0,265539512	0,000404581	0,001195736
AC109347.1	antisense	ENSG00000260526	1,196983386	0,000406066	0,001199887
CMTR2	protein_coding	ENSG00000180917	-0,194831117	0,000406637	0,0012014
HNRNPC	protein_coding	ENSG00000092199	-0,219573359	0,000406834	0,00120181
DGKE	protein_coding	ENSG00000153933	0,269895114	0,000406958	0,001202004
AL050343.1	antisense	ENSG00000266993	1,404566072	0,0004073	0,00120284
MON1A	protein_coding	ENSG00000164077	-0,256923319	0,000408247	0,001205462
PPP1R14D	protein_coding	ENSG00000166143	4,580025193	0,000408697	0,001206617
GTF2F1	protein_coding	ENSG00000125651	0,183101595	0,000409627	0,001209191
PCGF1	protein_coding	ENSG00000115289	-0,274835092	0,000409858	0,001209699
RANBP2	protein_coding	ENSG00000153201	0,232508822	0,000410125	0,001210311
CHCHD3	protein_coding	ENSG00000106554	0,169907836	0,000410657	0,001211706
AZIN1	protein_coding	ENSG00000155096	0,133226674	0,000410773	0,001211875
FOXP2	protein_coding	ENSG00000128573	1,530482551	0,000410978	0,001212304
ZNF37A	protein_coding	ENSG00000075407	0,310678565	0,000411585	0,001213922
SPTLC1P4	processed_pseudogene	ENSG00000232678	3,274065111	0,000412247	0,001215699
AC008014.1	lincRNA	ENSG00000257261	0,557983032	0,000412837	0,001217263
POLE	protein_coding	ENSG00000177084	-0,331882623	0,0004134	0,001218747
FLNC	protein_coding	ENSG00000128591	0,324800601	0,000413585	0,001219118
OTOP2	protein_coding	ENSG00000183034	4,631806839	0,000414662	0,001222118
ZIC2	protein_coding	ENSG00000043355	-0,322265511	0,000414956	0,001222809
ZNF467	protein_coding	ENSG00000181444	1,627591991	0,000415929	0,001225499
PCDHGA2	protein_coding	ENSG00000081853	-0,523941169	0,000417974	0,001231349
DENND5B	protein_coding	ENSG00000170456	-0,208014893	0,000418283	0,001232082
C5	protein_coding	ENSG00000106804	0,456841326	0,000418405	0,001232263
SELENOF	protein_coding	ENSG00000183291	0,248892629	0,000418623	0,001232728
AL139011.1	antisense	ENSG00000228606	2,016708017	0,000418772	0,001232991
TBC1D10C	protein_coding	ENSG00000175463	2,932751087	0,000419784	0,001235793

ZNF517	protein_coding	ENSG00000197363	0,506500666	0,000420105	0,001236559
TRMT61A	protein_coding	ENSG00000166166	-0,298868398	0,00042043	0,001237339
CD27	protein_coding	ENSG00000139193	4,516704572	0,000422053	0,001241756
SLA	protein_coding	ENSG00000155926	1,669049888	0,000422047	0,001241756
GTF3C5	protein_coding	ENSG00000148308	-0,199965191	0,000422112	0,001241756
AC092123.1	antisense	ENSG00000261118	1,221379921	0,000423109	0,001244508
GPX7	protein_coding	ENSG00000116157	4,691140622	0,000423734	0,001246169
MAPKAP1	protein_coding	ENSG00000119487	-0,130106798	0,000424163	0,001247252
SPATC1L	protein_coding	ENSG00000160284	-0,319342202	0,00042469	0,001248624
BX284668.5	lincRNA	ENSG00000238142	-0,341966092	0,000425313	0,001250274
TLN2	protein_coding	ENSG00000171914	-0,239756577	0,000426323	0,001253065
LINC02137	lincRNA	ENSG00000260186	2,883437874	0,000426734	0,001254093
SIGMAR1	protein_coding	ENSG00000147955	-0,192768398	0,000427446	0,001256004
LEO1	protein_coding	ENSG00000166477	-0,207639412	0,000427584	0,00125623
UCKL1	protein_coding	ENSG00000198276	-0,198388832	0,000428658	0,001259207
YEATS2	protein_coding	ENSG00000163872	-0,19666526	0,000429068	0,00126023
NRSN2-AS1	antisense	ENSG00000225377	0,55097597	0,000429672	0,001261824
UPF3A	protein_coding	ENSG00000169062	-0,209809292	0,000429872	0,001262229
SIRPB2	protein_coding	ENSG00000196209	4,405820832	0,000430324	0,001263375
DPEP2NB	protein_coding	ENSG00000263201	3,290560051	0,000431995	0,001268101
RNASEH2B	protein_coding	ENSG00000136104	-0,26237731	0,000434307	0,001274705
CATSPERE	protein_coding	ENSG00000179397	1,131367473	0,000434851	0,00127612
BAHCC1	protein_coding	ENSG00000266074	0,463985872	0,000435309	0,001276916
STIP1	protein_coding	ENSG00000168439	-0,146899411	0,000435261	0,001276916
SF3B5	protein_coding	ENSG00000169976	-0,235941071	0,00043521	0,001276916
DUSP19	protein_coding	ENSG00000162999	0,35064642	0,000435495	0,001277277
SNORA66	snoRNA	ENSG00000207523	-0,912002702	0,000437209	0,00128212
AC112721.2	lincRNA	ENSG00000222032	2,841481073	0,000437508	0,001282816
CACYBP	protein_coding	ENSG00000116161	-0,209768892	0,000437834	0,001283587
CCDC38	protein_coding	ENSG00000165972	1,728194674	0,000438042	0,001284012
AC022400.6	lincRNA	ENSG00000272140	1,315653999	0,000438939	0,001286459
AC078880.3	lincRNA	ENSG00000271579	2,84867301	0,000440124	0,001289748
C3orf67	protein_coding	ENSG00000163689	0,337141068	0,000440505	0,001290679
UTS2B	protein_coding	ENSG00000188958	1,305706008	0,000440742	0,001291191
MBTPS1	protein_coding	ENSG00000140943	-0,136057006	0,000440897	0,00129146
SS18	protein_coding	ENSG00000141380	-0,163507054	0,000441198	0,001292155
CCDC167	protein_coding	ENSG00000198937	-0,336422843	0,000441508	0,00129288
VPS4A	protein_coding	ENSG00000132612	-0,190600022	0,000443236	0,001297755
COX20P1	processed_pseudogene	ENSG00000213025	-0,3363604	0,000445888	0,001305334
SLC26A8	protein_coding	ENSG00000112053	3,190177292	0,000449003	0,001314263

GADD45GIP1	protein_coding	ENSG00000179271	-0,287162291	0,000449257	0,001314822
SAMD12-AS1	lincRNA	ENSG00000281641	1,084324593	0,000450366	0,001317877
JMJD4	protein_coding	ENSG00000081692	-0,23342597	0,000450466	0,001317984
CFAP298	protein_coding	ENSG00000159079	-0,292783817	0,000451337	0,001320344
GMEB1	protein_coding	ENSG00000162419	-0,231110975	0,000453458	0,001326168
CD36	protein_coding	ENSG00000135218	1,797103655	0,000453891	0,001327246
UBE2D3-AS1	antisense	ENSG00000246560	0,801267271	0,000454252	0,001328112
AC016727.1	antisense	ENSG00000270820	0,717958201	0,000454435	0,001328458
CENPN	protein_coding	ENSG00000166451	-0,213223505	0,000459812	0,001343985
CCDC136	protein_coding	ENSG00000128596	0,587831322	0,000459966	0,001344245
HCST	protein_coding	ENSG00000126264	0,754233078	0,000460987	0,001347035
CUX2	protein_coding	ENSG00000111249	4,429017813	0,000461852	0,001349371
TFAM	protein_coding	ENSG00000108064	-0,23275873	0,000463486	0,001353953
FN1	protein_coding	ENSG00000115414	0,246975311	0,000464367	0,001356302
ETFDH	protein_coding	ENSG00000171503	-0,273194717	0,000464423	0,001356302
CIAO3	protein_coding	ENSG00000103245	-0,30670276	0,000468251	0,001367286
TMTC3	protein_coding	ENSG00000139324	0,219460446	0,000468459	0,001367699
LINC01322	lincRNA	ENSG00000244128	-0,274871923	0,000468803	0,001368511
ATP2A1	protein_coding	ENSG00000196296	1,00380278	0,000469176	0,001369404
ZBTB7B	protein_coding	ENSG00000160685	0,369805205	0,000469776	0,001370959
PMF1	protein_coding	ENSG00000160783	-0,300099719	0,000469997	0,00137141
GPR179	protein_coding	ENSG00000277399	2,624534292	0,000470151	0,001371663
WDR70	protein_coding	ENSG00000082068	-0,217876914	0,000470862	0,001373543
PPM1M	protein_coding	ENSG00000164088	-0,222556897	0,000471087	0,001374004
SEC24C	protein_coding	ENSG00000176986	-0,185697182	0,000472178	0,001376598
CLSTN1	protein_coding	ENSG00000171603	-0,222147264	0,000472048	0,001376598
MDM1	protein_coding	ENSG00000111554	-0,275265592	0,000472132	0,001376598
TMEM181	protein_coding	ENSG00000146433	-0,234941271	0,000472891	0,001378482
CYBRD1	protein_coding	ENSG00000071967	0,14707914	0,000473092	0,001378872
TLR3	protein_coding	ENSG00000164342	0,609083587	0,000476344	0,001388153
ARMC7	protein_coding	ENSG00000125449	-0,358099151	0,000477048	0,001390007
CLTA	protein_coding	ENSG00000122705	0,196884233	0,000479413	0,001396699
AC007318.2	transcribed_processed_	ENSG00000273763	4,401483584	0,000482767	0,001406271
POT1	protein_coding	ENSG00000128513	-0,247628019	0,000486348	0,001416099
RAVER1	protein_coding	ENSG00000161847	-0,339863589	0,000486284	0,001416099
GALNT9	protein_coding	ENSG00000182870	4,471212702	0,000487209	0,001418405
AC022916.1	antisense	ENSG00000266947	0,732173749	0,00048818	0,001421029
SLC9A8	protein_coding	ENSG00000197818	0,289644394	0,000488273	0,001421098
TLR1	protein_coding	ENSG00000174125	1,052278382	0,00049032	0,001426852
SRSF4	protein_coding	ENSG00000116350	-0,160937726	0,000491403	0,001429803

TMEM170B	protein_coding	ENSG00000205269	-0,32262936	0,000493112	0,00143457
SH3GL1	protein_coding	ENSG00000141985	-0,221880278	0,000494205	0,001437548
IPMK	protein_coding	ENSG00000151151	0,267841553	0,000494316	0,001437665
CPSF4	protein_coding	ENSG00000160917	0,25598643	0,000494432	0,001437799
WDR92	protein_coding	ENSG00000243667	-0,38831842	0,000495119	0,001439593
XAF1	protein_coding	ENSG00000132530	2,816805938	0,00049549	0,001440468
DDX19B	protein_coding	ENSG00000157349	0,198486347	0,000497053	0,001444806
SPP1	protein_coding	ENSG00000118785	0,244600515	0,000497657	0,001446359
PARP11	protein_coding	ENSG00000111224	0,518297796	0,000499209	0,001450662
LVRN	protein_coding	ENSG00000172901	1,961514021	0,000500166	0,00145296
PLP2	protein_coding	ENSG00000102007	-0,158149713	0,000500212	0,00145296
AC024075.2	sense_intronic	ENSG00000269044	-0,57265003	0,000500197	0,00145296
PTAR1	protein_coding	ENSG00000188647	-0,234111155	0,000500729	0,001454255
MED30	protein_coding	ENSG00000164758	-0,261794279	0,00050136	0,001455883
TUG1	bidirectional_promoter_	ENSG00000253352	-0,2395352	0,000502102	0,001457831
TPPP3	protein_coding	ENSG00000159713	1,441474821	0,000502961	0,001460118
STK11IP	protein_coding	ENSG00000144589	-0,264181334	0,000503898	0,00146263
KDM3A	protein_coding	ENSG00000115548	0,206773429	0,000504774	0,001464965
NAPA	protein_coding	ENSG00000105402	0,253030349	0,000505648	0,001467294
AC007996.1	sense_intronic	ENSG00000277534	-0,471875755	0,000506617	0,0014699
ELMO3	protein_coding	ENSG00000102890	-0,768678513	0,000507132	0,001471185
MED9	protein_coding	ENSG00000141026	-0,266718454	0,00050889	0,001476075
CRTAM	protein_coding	ENSG00000109943	2,84934561	0,000510005	0,001479102
CHID1	protein_coding	ENSG00000177830	-0,24580801	0,000510536	0,001480433
AC005972.3	lincRNA	ENSG00000283538	4,367880713	0,000510834	0,001481086
HIST1H3G	protein_coding	ENSG00000273983	3,180132678	0,000512478	0,001485644
TMSB10	protein_coding	ENSG00000034510	-0,253095806	0,000513406	0,001488121
TPP1	protein_coding	ENSG00000166340	0,357393339	0,000515342	0,001493522
KPRP	protein_coding	ENSG00000203786	-1,200893308	0,000518072	0,001501223
SNX4	protein_coding	ENSG00000114520	-0,216669982	0,000518616	0,001502586
GJC2	protein_coding	ENSG00000198835	1,029557189	0,000519623	0,001505292
PIGW	protein_coding	ENSG00000277161	-0,23087453	0,000520649	0,001508051
NFYC	protein_coding	ENSG00000066136	-0,168712852	0,000521693	0,001510861
DCLK1	protein_coding	ENSG00000133083	0,35567492	0,000522615	0,001513319
LINC01732	lincRNA	ENSG00000237292	4,352164018	0,000524038	0,001517224
TDRP	protein_coding	ENSG00000180190	-0,29788104	0,000524484	0,001518302
RAB17	protein_coding	ENSG00000124839	4,413185642	0,000525484	0,001520779
ENTPD5	protein_coding	ENSG00000187097	-0,262558157	0,000525488	0,001520779
TDGF1P7	processed_pseudogene	ENSG00000269584	4,444001497	0,000526982	0,001524888
PRSS27	protein_coding	ENSG00000172382	1,462348843	0,000527523	0,001526238

AL591846.1	processed_pseudogene	ENSG00000224114	-0,250135977	0,000527768	0,001526732
TMPRSS7	protein_coding	ENSG00000176040	0,774278063	0,000530744	0,001535123
LLGL1	protein_coding	ENSG00000131899	-0,238326059	0,000531253	0,001536378
COL2A1	protein_coding	ENSG00000139219	3,092745121	0,00053425	0,001544829
EMX1	protein_coding	ENSG00000135638	3,137140943	0,000535052	0,001546929
TESMIN	protein_coding	ENSG00000132749	-0,254450158	0,000535907	0,001549184
BABAM2	protein_coding	ENSG00000158019	0,177021054	0,000538042	0,001555137
CAVIN3	protein_coding	ENSG00000170955	-0,278954267	0,000538291	0,001555636
EPDR1	protein_coding	ENSG00000086289	-0,203436998	0,000539993	0,001560334
AC137630.1	antisense	ENSG00000223343	1,44016788	0,000540834	0,001562546
RASA4CP	transcribed_unprocessed	ENSG00000228903	2,112463996	0,000542224	0,001566242
HOXA6	protein_coding	ENSG00000106006	-0,990463593	0,000542529	0,00156678
CD34	protein_coding	ENSG00000174059	1,026044151	0,00054359	0,001569401
TSEN15	protein_coding	ENSG00000198860	-0,257071179	0,000543569	0,001569401
AL034374.1	lincRNA	ENSG00000271367	1,833794007	0,000543939	0,001569746
CXCR5	protein_coding	ENSG00000160683	2,785671837	0,000543818	0,001569746
SEH1L	protein_coding	ENSG00000085415	-0,239735908	0,00054388	0,001569746
AL035681.1	antisense	ENSG00000235513	2,316659648	0,000544657	0,001571599
RTKN2	protein_coding	ENSG00000182010	-0,280502814	0,000545715	0,001574429
RBMS3-AS3	antisense	ENSG00000235904	1,396375963	0,000546322	0,001575957
RNF41	protein_coding	ENSG00000181852	0,232288516	0,000548253	0,001581306
PNN	protein_coding	ENSG00000100941	-0,2411682	0,000548509	0,001581822
NUDT11	protein_coding	ENSG00000196368	-0,441254031	0,00054941	0,001584198
GEMIN8	protein_coding	ENSG00000046647	-0,311395911	0,000549622	0,001584586
BX284668.2	lincRNA	ENSG00000228549	-0,712307635	0,000549876	0,001585096
TXNDC11	protein_coding	ENSG00000153066	0,254459103	0,000553699	0,001595893
EXOC2	protein_coding	ENSG00000112685	-0,165143709	0,000554263	0,001597294
JAM2	protein_coding	ENSG00000154721	1,70113001	0,000556148	0,0016025
AC007036.1	lincRNA	ENSG00000227017	4,605647897	0,0005572	0,001605305
ERCC4	protein_coding	ENSG00000175595	0,228878786	0,00055732	0,001605425
IL12RB1	protein_coding	ENSG00000096996	2,83414756	0,000557456	0,001605591
SCN11A	protein_coding	ENSG00000168356	2,824090159	0,000561276	0,00161623
DEC1	protein_coding	ENSG00000173077	1,352888294	0,000561307	0,00161623
WDFY3-AS2	antisense	ENSG00000180769	0,728139119	0,00056142	0,001616329
TOR4A	protein_coding	ENSG00000198113	-0,275320674	0,000562845	0,001620204
AC037459.3	antisense	ENSG00000253200	0,590282615	0,000563304	0,001621298
PXN-AS1	antisense	ENSG00000255857	-0,382331164	0,000564376	0,001624153
SCUBE2	protein_coding	ENSG00000175356	0,989494859	0,00056605	0,001628743
NBPF14	protein_coding	ENSG00000270629	0,29116909	0,000567664	0,001633158
NKIRAS2	protein_coding	ENSG00000168256	0,250011614	0,000567967	0,0016338

LMOD1	protein_coding	ENSG00000163431	2,857476796	0,000569621	0,00163833
LINC02210	transcribed_unitary_pse	ENSG00000204650	-0,216489737	0,000570529	0,001640712
GNG2	protein_coding	ENSG00000186469	2,806738427	0,00057169	0,001643819
CDK13	protein_coding	ENSG00000065883	0,171968552	0,000572546	0,001646049
DBF4B	protein_coding	ENSG00000161692	-0,275761217	0,000572873	0,001646759
OTUD7B	protein_coding	ENSG00000264522	0,250567466	0,000575068	0,001652837
SOWAHC	protein_coding	ENSG00000198142	0,198150009	0,000575946	0,001655127
CRIM1	protein_coding	ENSG00000150938	-0,229827145	0,000576694	0,001657047
Z68871.1	lincRNA	ENSG00000239407	0,497079987	0,000581247	0,001669896
AL604028.1	sense_intronic	ENSG00000230896	-0,587949445	0,000582074	0,001672036
AC107956.1	processed_pseudogene	ENSG00000197149	-0,27737056	0,00058282	0,001673943
TBC1D3L	protein_coding	ENSG00000274512	0,861418283	0,000582953	0,001674091
TBC1D3L	protein_coding	ENSG00000274512	0,861418283	0,000582953	0,001674091
CRHBP	protein_coding	ENSG00000145708	1,086773249	0,000583459	0,001675311
DMAC2	protein_coding	ENSG00000105341	-0,197336455	0,000584742	0,001678761
AL663074.1	antisense	ENSG00000233069	1,07857273	0,000586083	0,001682375
OXA1L	protein_coding	ENSG00000155463	-0,170536306	0,000586286	0,001682721
LRRFIP1P1	processed_pseudogene	ENSG00000240429	0,422097381	0,000586393	0,001682794
CCDC13	protein_coding	ENSG00000244607	1,673854119	0,000586499	0,001682861
CNTN2	protein_coding	ENSG00000184144	2,805768422	0,000586827	0,001683566
SMNDC1	protein_coding	ENSG00000119953	0,22130668	0,000587365	0,001684874
SH3GLB2	protein_coding	ENSG00000148341	-0,217690624	0,000588552	0,001688044
AC108058.1	antisense	ENSG00000238273	4,342216392	0,000590477	0,001693328
AP000873.3	antisense	ENSG00000254551	4,492529258	0,00059196	0,001697344
AL135925.1	lincRNA	ENSG00000272447	0,901787157	0,000594589	0,001704645
MT1X	protein_coding	ENSG00000187193	0,410638467	0,000594738	0,001704834
MACO1	protein_coding	ENSG00000204178	0,244543313	0,000596925	0,001710863
HIST1H2AA	protein_coding	ENSG00000164508	4,40247046	0,000597233	0,001711377
NAV1	protein_coding	ENSG00000134369	-0,22013034	0,000597271	0,001711377
ZNF138	protein_coding	ENSG00000197008	-0,355851717	0,00059884	0,001715393
LBX1-AS1	processed_transcript	ENSG00000227128	3,227226921	0,000599621	0,001717391
COL28A1	protein_coding	ENSG00000215018	1,753684386	0,000600097	0,001718514
TMED1	protein_coding	ENSG00000099203	-0,245771437	0,00060062	0,001719772
EPCAM-DT	lincRNA	ENSG00000234690	0,984680279	0,000600715	0,001719804
PPIL3	protein_coding	ENSG00000240344	-0,250887672	0,00060214	0,001723642
TNKS2	protein_coding	ENSG00000107854	0,1339275	0,000602314	0,0017239
HOXB2	protein_coding	ENSG00000173917	-0,481718251	0,000603265	0,001726381
CCT4	protein_coding	ENSG00000115484	-0,18810766	0,000603648	0,001727235
MUC2	protein_coding	ENSG00000198788	4,430357775	0,000605381	0,001731953
LMTK2	protein_coding	ENSG00000164715	0,273628424	0,000605627	0,001732415

SPRYD4	protein_coding	ENSG00000176422	-0,2410033	0,000605791	0,001732642
MLST8	protein_coding	ENSG00000167965	-0,220017986	0,00060599	0,001732968
PCBD1	protein_coding	ENSG00000166228	-0,188230345	0,000606451	0,001734044
DBR1	protein_coding	ENSG00000138231	-0,172018484	0,000606818	0,001734852
KPNA7	protein_coding	ENSG00000185467	1,815405526	0,00060785	0,001737076
CCDC91	protein_coding	ENSG00000123106	0,208748092	0,000607751	0,001737076
FBXO22	protein_coding	ENSG00000167196	0,154812096	0,000607791	0,001737076
SLC2A9	protein_coding	ENSG00000109667	2,301283975	0,000609444	0,001741146
PKD2	protein_coding	ENSG00000118762	0,213746697	0,00060939	0,001741146
NDUFA6-DT	processed_transcript	ENSG00000237037	-0,618515927	0,000610059	0,001742662
CDK5R1	protein_coding	ENSG00000176749	-0,404496058	0,000610955	0,001744978
BCAM	protein_coding	ENSG00000187244	-0,458464094	0,0006122	0,00174829
PJA2	protein_coding	ENSG00000198961	0,15200985	0,000613555	0,001751916
SSX2IP	protein_coding	ENSG00000117155	-0,20862897	0,000613645	0,00175193
PDZD7	protein_coding	ENSG00000186862	0,762923963	0,000614638	0,001754519
DGKI	protein_coding	ENSG00000157680	0,883099068	0,000617874	0,001763267
FAM186A	protein_coding	ENSG00000185958	2,26284126	0,000619671	0,00176815
VPS39	protein_coding	ENSG00000166887	0,208058119	0,000620983	0,0017714
FBXO38	protein_coding	ENSG00000145868	0,170713351	0,000620975	0,0017714
NUP210	protein_coding	ENSG00000132182	-0,258770528	0,000621085	0,001771443
TRIM72	protein_coding	ENSG00000177238	2,864105934	0,000621616	0,001772711
PRDX4	protein_coding	ENSG00000123131	-0,275089769	0,000626111	0,001785283
GIPC1	protein_coding	ENSG00000123159	-0,220475869	0,000626434	0,001785956
DNAJC5B	protein_coding	ENSG00000147570	1,830035105	0,00062696	0,001787205
AC027288.3	lincRNA	ENSG00000257894	2,002855383	0,000628348	0,001790914
AL355974.2	lincRNA	ENSG00000275830	2,25259447	0,000629538	0,001793808
TSPAN17	protein_coding	ENSG00000048140	-0,172058511	0,000629518	0,001793808
SCLY	protein_coding	ENSG00000132330	-0,515759492	0,000630191	0,00179542
MCL1	protein_coding	ENSG00000143384	-0,200633846	0,000631632	0,001799273
BPHL	protein_coding	ENSG00000137274	-0,256899643	0,000632631	0,001801869
AC093495.1	antisense	ENSG00000228242	1,238188307	0,000632841	0,001802218
SNRPG	protein_coding	ENSG00000143977	-0,30431554	0,000633149	0,001802845
IL21R	protein_coding	ENSG00000103522	1,755110112	0,000633429	0,001803391
C1orf100	protein_coding	ENSG00000173728	2,89345825	0,000634481	0,001806136
P4HTM	protein_coding	ENSG00000178467	0,327460124	0,000636382	0,001811296
EPS8L2	protein_coding	ENSG00000177106	0,227839818	0,000636618	0,001811717
ZNF414	protein_coding	ENSG00000133250	-0,414050099	0,000637666	0,001814445
LINC02660	lincRNA	ENSG00000226005	3,16487743	0,000638134	0,001815525
LINC02100	lincRNA	ENSG00000248693	1,483160353	0,000638791	0,001817142
DGKD	protein_coding	ENSG00000077044	0,244062532	0,000640937	0,001822996

HCG11	lincRNA	ENSG00000228223	-0,337535615	0,000641621	0,001824688
GJB3	protein_coding	ENSG00000188910	-0,306829787	0,000642232	0,001826173
SNORA73B	snoRNA	ENSG00000200087	-0,480437113	0,000642701	0,001827251
CALM1	protein_coding	ENSG00000198668	0,134672889	0,000643317	0,001828751
DRAXIN	protein_coding	ENSG00000162490	2,564665237	0,000645069	0,001833475
KLHL6	protein_coding	ENSG00000172578	2,844147559	0,000647373	0,001839771
LRRC3	protein_coding	ENSG00000160233	-0,744476614	0,000650591	0,001848659
GABRG2	protein_coding	ENSG00000113327	0,420410752	0,000652004	0,001852418
LNCOC1	antisense	ENSG00000253741	0,923244797	0,00065247	0,001853486
PLIN2	protein_coding	ENSG00000147872	0,167971442	0,000655754	0,001862557
MAPKBP1	protein_coding	ENSG00000137802	0,304016303	0,000656734	0,001865082
MEPCE	protein_coding	ENSG00000146834	-0,253919206	0,000656917	0,001865341
KCNJ5	protein_coding	ENSG00000120457	1,718838823	0,000658001	0,001868163
C2CD6	protein_coding	ENSG00000155754	1,614885357	0,000658273	0,001868676
POU2AF1	protein_coding	ENSG00000110777	2,761196447	0,00065886	0,001870083
AP001596.1	antisense	ENSG00000232692	0,681107872	0,000659004	0,001870226
PSG3	protein_coding	ENSG00000221826	2,118656976	0,000659093	0,001870226
AL356356.1	antisense	ENSG00000237781	1,564609606	0,000659713	0,001871725
AVIL	protein_coding	ENSG00000135407	1,16775023	0,000660054	0,001872177
AOX1	protein_coding	ENSG00000138356	0,148985426	0,000660017	0,001872177
SRRM5	protein_coding	ENSG00000226763	1,800690179	0,000661484	0,001875974
MIDN	protein_coding	ENSG00000167470	0,31969711	0,000662834	0,001879543
SMAD2	protein_coding	ENSG00000175387	-0,169089541	0,000663577	0,00188139
AL157392.3	processed_transcript	ENSG00000239665	0,413299495	0,000664725	0,001884382
AP002784.1	lincRNA	ENSG00000250519	3,121696911	0,00066677	0,00188992
HSD17B11	protein_coding	ENSG00000198189	-0,236821379	0,000668249	0,00189385
FBRS	protein_coding	ENSG00000156860	0,353042025	0,000669371	0,001896767
TAF1A-AS1	antisense	ENSG00000225265	0,646886122	0,000669691	0,00189741
PPP1R36	protein_coding	ENSG00000165807	1,680846188	0,000672327	0,001904617
AL353795.3	TEC	ENSG00000279608	1,183948094	0,000674066	0,001909281
RASAL1	protein_coding	ENSG00000111344	4,402162448	0,000674672	0,001910731
PRELP	protein_coding	ENSG00000188783	1,176784417	0,00067486	0,001911001
MTG2	protein_coding	ENSG00000101181	-0,197239196	0,000675929	0,001913763
DGCR11	sense_intronic	ENSG00000273311	-0,427318203	0,000676417	0,001914881
IQCK	protein_coding	ENSG00000174628	0,485421083	0,000676524	0,001914918
KLF16	protein_coding	ENSG00000129911	-0,315928277	0,000679308	0,001922534
AC009093.6	lincRNA	ENSG00000277999	1,141325079	0,000679655	0,00192325
EXOSC5	protein_coding	ENSG00000077348	-0,303698083	0,00067986	0,001923566
CTSV	protein_coding	ENSG00000136943	-0,254787978	0,000680259	0,00192443
AL353740.1	lincRNA	ENSG00000203434	0,589249669	0,000681673	0,001928163

AL805961.2	lincRNA	ENSG00000284703	3,061634779	0,000682058	0,001928987
ZNF407	protein_coding	ENSG00000215421	-0,228731929	0,000683383	0,001932466
ATXN2L	protein_coding	ENSG00000168488	0,236624926	0,000685029	0,001936856
MRPL57	protein_coding	ENSG00000173141	-0,230781779	0,000685362	0,001937529
AL356124.1	lincRNA	ENSG00000226149	0,880411814	0,000685582	0,00193779
NES	protein_coding	ENSG00000132688	1,965271718	0,000685738	0,00193779
ATRX	protein_coding	ENSG00000085224	0,262225218	0,000685669	0,00193779
TMUB1	protein_coding	ENSG00000164897	-0,313551989	0,000686205	0,001938845
TUT1	protein_coding	ENSG00000149016	-0,330669	0,000687227	0,001941464
PPP1R13L	protein_coding	ENSG00000104881	0,253380136	0,000690293	0,001949857
LRRC14	protein_coding	ENSG00000160959	-0,36280225	0,000690471	0,001950091
AK6	protein_coding	ENSG00000085231	-0,252649982	0,000692107	0,001954173
CD58	protein_coding	ENSG00000116815	-0,302609394	0,000692016	0,001954173
GINM1	protein_coding	ENSG00000055211	0,207603736	0,000693377	0,001957489
ACAD10	protein_coding	ENSG00000111271	-0,243295039	0,000693605	0,001957863
AL662844.4	antisense	ENSG00000272501	0,63584339	0,000694592	0,001960111
TPRG1	protein_coding	ENSG00000188001	1,259922007	0,000694513	0,001960111
CBFB	protein_coding	ENSG00000067955	-0,2418543	0,000697407	0,001967785
LINC00649	antisense	ENSG00000237945	0,350488604	0,000697633	0,001968149
KLHL32	protein_coding	ENSG00000186231	1,825262092	0,000698568	0,001970516
LINC01291	lincRNA	ENSG00000204792	-0,209839685	0,000699394	0,001972576
SS18L1	protein_coding	ENSG00000184402	-0,269307903	0,00069979	0,001973421
AC112484.1	antisense	ENSG00000231305	1,251056638	0,000703707	0,001984194
TRMT44	protein_coding	ENSG00000155275	0,249619984	0,00070409	0,001984728
RNVU1-7	snRNA	ENSG00000206585	-1,218268389	0,000704022	0,001984728
LRRC8B	protein_coding	ENSG00000197147	-0,261840326	0,00070536	0,001988034
AL138812.1	lincRNA	ENSG00000254686	2,121490073	0,000705678	0,001988659
RAG1	protein_coding	ENSG00000166349	0,484718604	0,000710979	0,002003321
LINC02574	lincRNA	ENSG00000233975	4,451743752	0,000711308	0,002003973
AC137767.1	lincRNA	ENSG00000256092	0,768379062	0,000712666	0,002007522
AC079447.1	protein_coding	ENSG00000241962	1,534569415	0,000715211	0,002014415
TRAF6	protein_coding	ENSG00000175104	0,193913878	0,000716299	0,002017204
GRM4	protein_coding	ENSG00000124493	4,497710247	0,000720463	0,002028652
AL445363.1	lincRNA	ENSG00000259002	4,438517117	0,000722733	0,002034765
AC090517.2	sense_intronic	ENSG00000274667	-1,030556587	0,000723361	0,002036252
PEBP1	protein_coding	ENSG00000089220	-0,142455006	0,000723551	0,002036509
SLC16A3	protein_coding	ENSG00000141526	-0,275409108	0,000723695	0,002036633
APH1A	protein_coding	ENSG00000117362	-0,192712279	0,000726402	0,002043954
WDR54	protein_coding	ENSG00000005448	-0,230514818	0,000726496	0,002043954
MYOM3	protein_coding	ENSG00000142661	2,067014238	0,000726646	0,002044097

AC138150.2	antisense	ENSG00000267288	0,764639016	0,000727873	0,002047266
ESRP1	protein_coding	ENSG00000104413	2,067922113	0,00072916	0,002050608
KDM1B	protein_coding	ENSG00000165097	0,182515181	0,000730389	0,002053498
ARHGAP39	protein_coding	ENSG00000147799	-0,416226698	0,000730355	0,002053498
PDIA3P1	transcribed_processed_	ENSG00000180867	0,369984054	0,00073164	0,002056735
SNX18	protein_coding	ENSG00000178996	-0,192161513	0,000734528	0,002064571
TBC1D30	protein_coding	ENSG00000111490	1,636451358	0,000740348	0,002080643
CELF3	protein_coding	ENSG00000159409	3,066469119	0,000741296	0,002083021
CDH5	protein_coding	ENSG00000179776	2,153452341	0,000742237	0,00208538
XIRP2-AS1	antisense	ENSG00000254552	2,992379609	0,000743026	0,002087311
MORC1	protein_coding	ENSG00000114487	2,740389143	0,000743542	0,002088476
LHX4	protein_coding	ENSG00000121454	-0,512106939	0,000743947	0,002089328
PAQR7	protein_coding	ENSG00000182749	-0,173473149	0,000744086	0,002089432
MFAP3	protein_coding	ENSG00000037749	0,171034341	0,000744393	0,002090007
LINC00351	lincRNA	ENSG00000226317	0,663628375	0,000744598	0,002090296
C22orf24	processed_transcript	ENSG00000128254	2,198956487	0,00074665	0,002095771
ABCB8	protein_coding	ENSG00000197150	-0,256704171	0,000746901	0,002096189
KLF3	protein_coding	ENSG00000109787	-0,190845516	0,000747855	0,002098578
SLC5A1	protein_coding	ENSG00000100170	3,07835278	0,000748613	0,002100416
AC010207.1	lincRNA	ENSG00000260633	0,838135326	0,000749418	0,002102389
PIK3CD	protein_coding	ENSG00000171608	-0,248391447	0,000750093	0,002103995
TAGLN2	protein_coding	ENSG00000158710	-0,184297133	0,000750875	0,002105899
ANKRD34A	protein_coding	ENSG00000272031	0,668789405	0,000752745	0,002110856
TFAP2A	protein_coding	ENSG00000137203	-0,185602154	0,000753805	0,00211325
ARMCX6	protein_coding	ENSG00000198960	-0,234020194	0,000753763	0,00211325
ECI2	protein_coding	ENSG00000198721	-0,199691456	0,000754228	0,002114149
RING1	protein_coding	ENSG00000204227	-0,162678025	0,000756921	0,002121115
DOCK7	protein_coding	ENSG00000116641	0,173912036	0,000757292	0,002121774
ADAM10	protein_coding	ENSG00000137845	0,141839026	0,000757363	0,002121774
C1orf195	lincRNA	ENSG00000204464	4,450346539	0,000758409	0,002124126
LYRM9	protein_coding	ENSG00000232859	0,617541215	0,000759574	0,002127097
CIRBP-AS1	antisense	ENSG00000267493	0,872220079	0,000760845	0,002130365
CMIP	protein_coding	ENSG00000153815	-0,222222431	0,000762684	0,002135223
LRMP	protein_coding	ENSG00000118308	2,670739553	0,000763895	0,002138321
AC006460.2	bidirectional_promoter_	ENSG00000284052	0,877046214	0,000764051	0,002138468
RCC1L	protein_coding	ENSG00000274523	-0,21541949	0,000766804	0,00214588
HEXB	protein_coding	ENSG00000049860	0,129750356	0,000767899	0,002148651
FOXD2-AS1	lincRNA	ENSG00000237424	-0,794829809	0,000768516	0,002150085
AC092818.1	lincRNA	ENSG00000254038	2,565421834	0,000769919	0,002153715
INPP4B	protein_coding	ENSG00000109452	0,723622639	0,000771156	0,002156882

GABPB1	protein_coding	ENSG00000104064	-0,197747456	0,000772299	0,002159784
PCNX4	protein_coding	ENSG00000126773	-0,18727362	0,000772982	0,002161398
SPATA17	protein_coding	ENSG00000162814	0,691587518	0,000776204	0,002170113
AC016831.3	processed_pseudogene	ENSG00000232716	1,806625416	0,00077714	0,002172433
SH3YL1	protein_coding	ENSG00000035115	0,270296219	0,000777784	0,002173937
CXADR	protein_coding	ENSG00000154639	0,242871865	0,00077832	0,00217514
NECTIN2	protein_coding	ENSG00000130202	0,230330695	0,000781144	0,002182734
AC012073.1	antisense	ENSG00000271936	-0,474379239	0,000781297	0,002182863
DVL3	protein_coding	ENSG00000161202	0,222375143	0,000782881	0,002186991
IGBP1P4	processed_pseudogene	ENSG00000250325	2,971871293	0,000785978	0,002195305
SRSF11	protein_coding	ENSG00000116754	-0,187328067	0,000786071	0,002195305
VPS13B	protein_coding	ENSG00000132549	0,191679859	0,000786992	0,002197576
WNT3	protein_coding	ENSG00000108379	-0,271022674	0,000789606	0,002204577
NCL	protein_coding	ENSG00000115053	-0,245136898	0,00079031	0,002206241
C19orf81	protein_coding	ENSG00000235034	-0,734660552	0,000790667	0,002206937
SNHG29	processed_transcript	ENSG00000175061	-0,240474377	0,000792144	0,00221046
AC091825.1	TEC	ENSG00000280047	1,387735424	0,000792087	0,00221046
AL160408.4	lincRNA	ENSG00000241475	1,104809729	0,000792878	0,002212205
CNDP2	protein_coding	ENSG00000133313	-0,198656493	0,000795107	0,002218124
RHOBTB1	protein_coding	ENSG00000072422	-0,308073555	0,000795993	0,002220295
CCNG1	protein_coding	ENSG00000113328	0,188457004	0,000797335	0,002223734
PHTF1	protein_coding	ENSG00000116793	-0,163701818	0,000797563	0,002224068
FAM50B	protein_coding	ENSG00000145945	-0,196703616	0,0007981	0,002225262
RNF138	protein_coding	ENSG00000134758	-0,287664462	0,000799071	0,002227667
DARS-AS1	antisense	ENSG00000231890	0,734579843	0,000800315	0,002230832
ATP6V0E1	protein_coding	ENSG00000113732	0,207581256	0,000800665	0,002231505
CHFR	protein_coding	ENSG00000072609	0,24408707	0,000801396	0,002233239
BROX	protein_coding	ENSG00000162819	0,165767542	0,000802903	0,002237134
NT5DC3	protein_coding	ENSG00000111696	-0,22635447	0,000803025	0,002237169
ZBTB12	protein_coding	ENSG00000204366	-0,536756375	0,000803232	0,002237442
MEI1	protein_coding	ENSG00000167077	1,492504242	0,000805741	0,002243894
VPS41	protein_coding	ENSG00000006715	0,150951567	0,000805767	0,002243894
SNX17	protein_coding	ENSG00000115234	-0,214786927	0,000807109	0,002247326
ALG1L	protein_coding	ENSG00000189366	2,695693508	0,000809488	0,00225335
EFCAB6	protein_coding	ENSG00000186976	1,382130156	0,000809492	0,00225335
IER2	protein_coding	ENSG00000160888	0,278524196	0,00081	0,002254459
DAGLB	protein_coding	ENSG00000164535	-0,146489752	0,000810343	0,002255108
ABI3	protein_coding	ENSG00000108798	2,789565062	0,000811455	0,002257894
MALAT1	lincRNA	ENSG00000251562	-0,463058972	0,000811867	0,002258735
CORO6	protein_coding	ENSG00000167549	-0,368634863	0,000813989	0,002264331

AC087477.2	lincRNA	ENSG00000259275	1,23304564	0,000814737	0,002266106
GJA5	protein_coding	ENSG00000265107	-0,443856791	0,000816074	0,002269516
APOBEC2	protein_coding	ENSG00000124701	2,840549993	0,000816352	0,00226998
METTL5	protein_coding	ENSG00000138382	-0,207818999	0,000816913	0,002271233
C12orf80	lincRNA	ENSG00000257137	2,658882695	0,000817093	0,002271427
PDGFA	protein_coding	ENSG00000197461	0,208790236	0,000818499	0,002275026
AC138819.1	antisense	ENSG00000249849	2,665006571	0,000818856	0,00227571
NDUFS3	protein_coding	ENSG00000213619	-0,2432637	0,000822077	0,002284175
AC009268.2	sense_intronic	ENSG00000278472	1,141813506	0,000822125	0,002284175
REEP2	protein_coding	ENSG00000132563	-0,457951092	0,000822367	0,002284539
CAMK4	protein_coding	ENSG00000152495	-0,251885383	0,000823952	0,002288632
KHDRBS3	protein_coding	ENSG00000131773	-0,244431199	0,000827086	0,002297026
SEC1P	transcribed_unitary_pse	ENSG00000232871	2,717462719	0,000828891	0,002301726
AC044802.2	antisense	ENSG00000258122	1,086115069	0,000829065	0,002301899
AC138356.1	antisense	ENSG00000183154	2,124653336	0,00083151	0,002308375
KLF9	protein_coding	ENSG00000119138	0,494131529	0,000833044	0,002312319
SLC27A1	protein_coding	ENSG00000130304	-0,344758573	0,000833202	0,002312446
AC124067.2	sense_intronic	ENSG00000253414	0,449254629	0,000834544	0,002315856
TRIM32	protein_coding	ENSG00000119401	-0,177713527	0,000836349	0,002320552
GDF9	protein_coding	ENSG00000164404	1,207669105	0,000838066	0,002325002
BTK	protein_coding	ENSG00000010671	2,369581087	0,000840572	0,002331639
RPL13A	protein_coding	ENSG00000142541	-0,235738282	0,000840709	0,002331703
ADGRL2	protein_coding	ENSG00000117114	0,129128041	0,000842344	0,002335606
MZF1	protein_coding	ENSG00000099326	-0,315053539	0,000842335	0,002335606
PEX5	protein_coding	ENSG00000139197	-0,188877398	0,000842516	0,002335768
LINC01166	lincRNA	ENSG00000232903	2,611579539	0,000844379	0,002340615
CCDC81	protein_coding	ENSG00000149201	1,017303669	0,000845574	0,002343611
XPR1	protein_coding	ENSG00000143324	0,165405336	0,00084652	0,002345916
DLG5	protein_coding	ENSG00000151208	-0,269396255	0,000849244	0,002353148
BAAT	protein_coding	ENSG00000136881	1,645826191	0,00084979	0,002354343
TNKS	protein_coding	ENSG00000173273	0,194812537	0,000850668	0,002356457
AC010273.1	antisense	ENSG00000248664	1,023757318	0,000851331	0,002357973
AC244021.1	lincRNA	ENSG00000227082	1,514568314	0,000852178	0,002360002
IQCJ-SCHIP1	protein_coding	ENSG00000283154	0,320833207	0,000853365	0,002362968
IQCJ-SCHIP1	protein_coding	ENSG00000283154	0,320833207	0,000853365	0,002362968
AL358472.3	antisense	ENSG00000273026	1,371414697	0,00085422	0,002365019
ZBTB47	protein_coding	ENSG00000114853	-0,389714795	0,00085531	0,002367717
AC012313.5	lincRNA	ENSG00000268912	0,571618254	0,000856374	0,002370343
ZNRF2	protein_coding	ENSG00000180233	-0,358884637	0,000856574	0,002370576
HCN4	protein_coding	ENSG00000138622	2,117659228	0,000856705	0,002370617

CATSPER1	protein_coding	ENSG00000175294	0,36086486	0,000857708	0,002373073
RHOBTB3	protein_coding	ENSG00000164292	0,163625593	0,000857948	0,002373417
AL132711.1	lincRNA	ENSG00000258717	2,939227118	0,000858204	0,002373805
LRRC75A	protein_coding	ENSG00000181350	0,590777743	0,000858754	0,002375007
TCP11	protein_coding	ENSG00000124678	0,953068445	0,000859174	0,002375847
NCOA1	protein_coding	ENSG00000084676	0,203824816	0,000865276	0,002392398
BBS10	protein_coding	ENSG00000179941	-0,256533683	0,000867041	0,002396955
PDCL3P4	processed_pseudogene	ENSG00000244119	1,335363438	0,000867794	0,002398713
CORIN	protein_coding	ENSG00000145244	0,596801513	0,000868432	0,002400153
TPT1-AS1	processed_transcript	ENSG00000170919	0,349636886	0,000869157	0,002401834
HMX2	protein_coding	ENSG00000188816	-0,862218065	0,000870361	0,002404838
BCL9	protein_coding	ENSG00000116128	-0,431757646	0,000871074	0,002406482
AL109615.3	antisense	ENSG00000237686	3,049779676	0,000872447	0,002409953
CLHC1	protein_coding	ENSG00000162994	0,439344645	0,000876033	0,002419457
THOC5	protein_coding	ENSG00000100296	-0,120852125	0,000876124	0,002419457
AC098847.1	transcribed_processed_	ENSG00000267669	2,681780167	0,00087667	0,002420638
DNAJC3-DT	lincRNA	ENSG00000247400	0,557443144	0,000878047	0,002424114
OLR1	protein_coding	ENSG00000173391	0,577855402	0,000878894	0,002426126
SLC38A5	protein_coding	ENSG00000017483	1,264188719	0,000880582	0,002430457
CAPN8	protein_coding	ENSG00000203697	3,159502552	0,00088102	0,00243134
SNORD101	snoRNA	ENSG00000206754	-0,739318099	0,000882097	0,002433985
BIVM	protein_coding	ENSG00000134897	-0,295302627	0,000884838	0,002441219
PARVB	protein_coding	ENSG00000188677	-0,211101499	0,00088806	0,00244978
POLR3B	protein_coding	ENSG00000013503	-0,211060677	0,000892122	0,002460653
EVC	protein_coding	ENSG00000072840	-0,245255874	0,000892413	0,002461127
AL159166.1	lincRNA	ENSG00000231811	2,92044545	0,000893501	0,002463795
KDELR1	protein_coding	ENSG00000105438	0,157967984	0,000893692	0,00246399
EFHB	protein_coding	ENSG00000163576	1,280442928	0,000898114	0,002475848
AC100847.1	lincRNA	ENSG00000278703	1,17250479	0,000901528	0,002484926
DNAJB6	protein_coding	ENSG00000105993	-0,119516743	0,000901841	0,002485455
THSD4	protein_coding	ENSG00000187720	0,223559985	0,000903182	0,002488606
CCDC51	protein_coding	ENSG00000164051	-0,21477473	0,000903227	0,002488606
MIEF1	protein_coding	ENSG00000100335	-0,155560341	0,000904507	0,002491799
NSL1	protein_coding	ENSG00000117697	-0,263403545	0,000907585	0,002499941
USH2A	protein_coding	ENSG00000042781	0,497601799	0,000907968	0,002500662
DOLPP1	protein_coding	ENSG00000167130	-0,218433984	0,000908373	0,00250144
NOS2	protein_coding	ENSG00000007171	4,333547776	0,000909058	0,00250299
LCAT	protein_coding	ENSG00000213398	0,599562342	0,000909257	0,002503204
RF00494	snoRNA	ENSG00000201592	-0,450830096	0,000912701	0,002512348
TNNT1	protein_coding	ENSG00000105048	-0,139865233	0,000917416	0,002524986

GPATCH2L	protein_coding	ENSG00000089916	0,162871283	0,000917734	0,002525523
WDR55	protein_coding	ENSG00000120314	-0,167271012	0,000918911	0,002528424
SH3PXD2A-AS1	antisense	ENSG00000280693	2,611284782	0,000920412	0,002532214
SERPINB2	protein_coding	ENSG00000197632	-0,393724626	0,00092177	0,002535609
MRPL20	protein_coding	ENSG00000242485	-0,265020056	0,000923657	0,002540458
ATXN7L3	protein_coding	ENSG00000087152	-0,253516683	0,000925878	0,002546226
SLC45A4	protein_coding	ENSG00000022567	0,273332519	0,000926813	0,002548456
HOXA10	protein_coding	ENSG00000253293	-0,383648758	0,000928541	0,002552865
DNAH2	protein_coding	ENSG00000183914	1,681304897	0,000928886	0,00255347
RBFOX3	protein_coding	ENSG00000167281	1,9595963	0,000929896	0,002555906
ARIH2OS	protein_coding	ENSG00000221883	0,731210623	0,000934012	0,002566876
CAPS	protein_coding	ENSG00000105519	1,087214719	0,000934432	0,002567684
DBNDD1	protein_coding	ENSG00000003249	0,293635511	0,000935143	0,002568951
RNF135	protein_coding	ENSG00000181481	-0,200989963	0,00093507	0,002568951
AP000654.1	lincRNA	ENSG00000269895	2,696076564	0,000938402	0,002577557
AP001020.2	sense_intronic	ENSG00000264635	1,469354998	0,000940845	0,002583923
RFT1	protein_coding	ENSG00000163933	-0,181554678	0,000944346	0,002593189
AC092807.2	lincRNA	ENSG00000272691	1,350812252	0,000947378	0,002601167
ZDHHC23	protein_coding	ENSG00000184307	-0,305519763	0,000948906	0,002605015
TBC1D2B	protein_coding	ENSG00000167202	-0,236749949	0,000949548	0,002606427
AC073370.1	lincRNA	ENSG00000230526	4,278438153	0,000950278	0,002608081
AC246785.2	processed_pseudogene	ENSG00000233586	2,641313799	0,00096128	0,002637924
WWC2	protein_coding	ENSG00000151718	0,143029484	0,000962326	0,002640441
ARRB1	protein_coding	ENSG00000137486	-0,242209282	0,000965866	0,002649802
RPS26	protein_coding	ENSG00000197728	-0,219144273	0,00096651	0,002651214
AP001330.5	lincRNA	ENSG00000271882	1,516020332	0,000967278	0,002652933
LINC00471	lincRNA	ENSG00000181798	0,632938703	0,000967396	0,002652933
HEATR5B	protein_coding	ENSG00000008869	-0,173375998	0,000970505	0,002661104
ATP2B1-AS1	lincRNA	ENSG00000271614	0,850119328	0,000971587	0,002663715
AL807761.4	antisense	ENSG00000230782	4,305481713	0,000973512	0,002668637
HES6	protein_coding	ENSG00000144485	0,450675467	0,000981401	0,002689901
AC022296.3	lincRNA	ENSG00000272832	2,976180032	0,000981705	0,002690017
PCBP1-AS1	processed_transcript	ENSG00000179818	0,207950175	0,000981678	0,002690017
CSTA	protein_coding	ENSG00000121552	1,308510707	0,000983165	0,002693363
NKTR	protein_coding	ENSG00000114857	0,251822856	0,000983189	0,002693363
KRTAP2-2	protein_coding	ENSG00000214518	3,144269316	0,000984878	0,00269763
AATF	protein_coding	ENSG00000275700	-0,14546401	0,000985707	0,00269954
ZFYVE26	protein_coding	ENSG00000072121	-0,218127341	0,000987062	0,002702891
DUSP5P1	processed_pseudogene	ENSG00000183929	-0,663680055	0,000988484	0,002706422
MRPL23	protein_coding	ENSG00000214026	-0,264375203	0,000990032	0,002710298

MRPL23	protein_coding	ENSG00000214026	-0,264375203	0,000990032	0,002710298
NPC2	protein_coding	ENSG00000119655	0,193623396	0,000991096	0,00271285
HDGFL2	protein_coding	ENSG00000167674	-0,18097299	0,000994402	0,002721172
COL18A1	protein_coding	ENSG00000182871	-0,317022979	0,000994386	0,002721172
AC087667.1	lincRNA	ENSG00000244791	2,959305026	0,000996623	0,002726886
ITGA9	protein_coding	ENSG00000144668	4,327781198	0,000997005	0,002727204
RNU6-781P	snRNA	ENSG00000252186	3,041126111	0,000996951	0,002727204
LIPE-AS1	antisense	ENSG00000213904	0,484214177	0,000998866	0,002731565
AC137932.2	processed_transcript	ENSG00000261253	0,523047859	0,000998746	0,002731565
ADAMTSL2	protein_coding	ENSG00000197859	4,306436593	0,00099954	0,002733044
LINC01572	lincRNA	ENSG00000261008	0,399132151	0,000999697	0,002733109
UBA6-AS1	antisense	ENSG00000248049	0,223603747	0,001001337	0,002737229
LINC01562	lincRNA	ENSG00000203356	1,870548534	0,001002678	0,002740527
POLR2G	protein_coding	ENSG00000168002	-0,219260039	0,001003438	0,00274224
MRPL18	protein_coding	ENSG00000112110	-0,257717899	0,001006572	0,002750439
AL080276.2	antisense	ENSG00000227627	-0,793589892	0,001006922	0,002751028
TRIR	protein_coding	ENSG00000123144	-0,182179841	0,00100969	0,002758222
LINC01619	processed_transcript	ENSG00000257242	1,528060244	0,001012319	0,002765038
SMIM38	protein_coding	ENSG00000284713	2,023970998	0,001012873	0,002766181
EFEMP1	protein_coding	ENSG00000115380	-0,234526559	0,001014458	0,002770141
OPN5	protein_coding	ENSG00000124818	2,151049374	0,001014841	0,002770818
ELP2	protein_coding	ENSG00000134759	-0,151148903	0,001020456	0,002785779
NUPL2	protein_coding	ENSG00000136243	0,231497912	0,001020666	0,002785981
GDAP1L1	protein_coding	ENSG00000124194	0,919909644	0,00102175	0,002788569
SLC45A3	protein_coding	ENSG00000158715	-0,314781013	0,001022617	0,002790564
SNORD88A	snoRNA	ENSG00000221241	-1,287060111	0,001026596	0,002801048
FANCL	protein_coding	ENSG00000115392	-0,222695786	0,001027294	0,002802582
CRYM-AS1	transcribed_unitary_pse	ENSG00000189149	0,740606635	0,001027706	0,002803331
MBD6	protein_coding	ENSG00000166987	0,430071469	0,001031082	0,002812165
ELOVL2	protein_coding	ENSG00000197977	-0,184482768	0,001034108	0,002820045
COL13A1	protein_coding	ENSG00000197467	-0,248829668	0,001034425	0,002820535
SARNP	protein_coding	ENSG00000205323	0,418688941	0,001035295	0,002822531
UCHL3	protein_coding	ENSG00000118939	0,334752858	0,001035661	0,002823154
RNF24	protein_coding	ENSG00000101236	0,302703111	0,001039829	0,002834137
UGT2B26P	unprocessed_pseudoge	ENSG00000250919	2,090374017	0,001040096	0,00283449
PAXIP1-AS1	lincRNA	ENSG00000273344	-0,45554695	0,001041196	0,002837109
AL359233.1	antisense	ENSG00000258696	1,116627101	0,001044922	0,002846884
ALPK3	protein_coding	ENSG00000136383	0,963405581	0,001051512	0,002864078
UTP23	protein_coding	ENSG00000147679	0,223929055	0,001051875	0,002864351
ZNF8	protein_coding	ENSG00000278129	-0,320556605	0,001051891	0,002864351

AC009831.1	antisense	ENSG00000263823	1,152046091	0,001053543	0,002868468
MIR5689HG	lincRNA	ENSG00000229401	0,6321473	0,001055231	0,002872681
VAV1	protein_coding	ENSG00000141968	1,545345657	0,001057951	0,002879705
ATP6V1B2	protein_coding	ENSG00000147416	0,138541825	0,001058408	0,002880567
NFYB	protein_coding	ENSG00000120837	-0,208157111	0,001058826	0,002881322
UBL7-AS1	antisense	ENSG00000247240	-0,337259422	0,001065726	0,002899713
ISPD	protein_coding	ENSG00000214960	0,54991731	0,001066072	0,002899886
MPP3	protein_coding	ENSG00000161647	-0,403153872	0,001065949	0,002899886
AVEN	protein_coding	ENSG00000169857	0,215798441	0,001066619	0,00290099
AC004704.1	lincRNA	ENSG00000249815	0,250899638	0,001068107	0,002904651
OCLN	protein_coding	ENSG00000197822	0,227826024	0,001073045	0,002917305
MSH5	protein_coding	ENSG00000204410	-0,68104958	0,001072965	0,002917305
FAM66C	antisense	ENSG00000226711	0,855726696	0,001074916	0,002922006
ABTB1	protein_coding	ENSG00000114626	-0,344061083	0,001075265	0,002922565
STX4	protein_coding	ENSG00000103496	0,245035977	0,001075602	0,002922728
RWDD3	protein_coding	ENSG00000122481	-0,520430276	0,001075609	0,002922728
PARGP1	transcribed_unprocesse	ENSG00000239883	0,35308214	0,001075953	0,002923274
PDCD4-AS1	antisense	ENSG00000203497	0,489410923	0,001077993	0,002928198
SNHG9	lincRNA	ENSG00000255198	-0,437818317	0,001078051	0,002928198
RPAIN	protein_coding	ENSG00000129197	0,193612278	0,001078206	0,002928232
PHF24	protein_coding	ENSG00000122733	4,252545339	0,001079435	0,00293118
NFE2L1	protein_coding	ENSG00000082641	0,17146058	0,001083069	0,00294066
AC083799.1	sense_intronic	ENSG00000203644	-0,333474525	0,001088016	0,002953701
FAM86EP	transcribed_unprocesse	ENSG00000251669	0,339680755	0,001091046	0,002961534
SAMD5	protein_coding	ENSG00000203727	-0,344571707	0,001093858	0,00296838
ZNF497	protein_coding	ENSG00000174586	-1,004258274	0,001093747	0,00296838
SNORA71D	snoRNA	ENSG00000200354	-0,492592984	0,00109456	0,002969893
AC009318.1	antisense	ENSG00000257176	1,684102645	0,001096004	0,002973419
NACC2	protein_coding	ENSG00000148411	-0,247695092	0,00109707	0,002975918
ZNF696	protein_coding	ENSG00000185730	-0,363062898	0,001099253	0,002981444
WDR53	protein_coding	ENSG00000185798	-0,233341496	0,001099703	0,002982269
CHP1	protein_coding	ENSG00000187446	0,133857199	0,001101346	0,002986331
PHF6	protein_coding	ENSG00000156531	-0,177688273	0,001102117	0,002988027
DEPDC5	protein_coding	ENSG00000100150	-0,297406156	0,001111258	0,003012014
AC016888.1	antisense	ENSG00000266036	2,958210349	0,001112928	0,003016141
MICAL3	protein_coding	ENSG00000243156	-0,234823267	0,00111473	0,003020624
AC103702.2	lincRNA	ENSG00000272763	-0,330200739	0,001115037	0,003021057
RTN2	protein_coding	ENSG00000125744	-0,233166374	0,001115331	0,003021456
FOXN3	protein_coding	ENSG00000053254	-0,221317147	0,001117409	0,003026684
MKKS	protein_coding	ENSG00000125863	-0,205375211	0,001117795	0,003027331

BCAS2	protein_coding	ENSG00000116752	0,22283793	0,00111807	0,003027674
KIAA1958	protein_coding	ENSG00000165185	-0,314276873	0,001120954	0,003035085
LIPA	protein_coding	ENSG00000107798	0,189291783	0,001122077	0,003037724
ADAP2	protein_coding	ENSG00000184060	1,355355054	0,001122531	0,003038551
TNFRSF14-AS1	antisense	ENSG00000238164	2,929389552	0,001123359	0,00303977
CRYBA1	protein_coding	ENSG00000108255	2,057773286	0,001123283	0,00303977
MCF2L2	protein_coding	ENSG00000053524	0,702765123	0,001123425	0,00303977
TSPAN33	protein_coding	ENSG00000158457	-0,284382394	0,001123884	0,003040609
FBP2	protein_coding	ENSG00000130957	2,926927377	0,001124469	0,003041792
RHOT1	protein_coding	ENSG00000126858	-0,208079884	0,00112529	0,00304361
SMG1P3	transcribed_unprocesse	ENSG00000180747	0,440444725	0,001130283	0,003056712
SLC5A5	protein_coding	ENSG00000105641	2,569056882	0,001131962	0,003060849
PRMT1	protein_coding	ENSG00000126457	-0,197257626	0,001132347	0,003061486
Z95115.1	antisense	ENSG00000261188	-0,49200082	0,001136014	0,003070997
RILPL1	protein_coding	ENSG00000188026	-0,215546475	0,001137296	0,003074058
AC090204.1	lincRNA	ENSG00000247134	0,262403026	0,001138693	0,003077428
PSG6	protein_coding	ENSG00000170848	1,547781336	0,001139075	0,003078053
SPATA6L	protein_coding	ENSG00000106686	1,635508493	0,001141696	0,00308473
ANKDD1B	protein_coding	ENSG00000189045	1,456759765	0,001142613	0,003086801
GAPDHP14	processed_pseudogene	ENSG00000236056	2,233103116	0,00114321	0,003088008
XKR7	protein_coding	ENSG00000260903	2,108741141	0,001143892	0,003089442
KHSRP	protein_coding	ENSG00000088247	-0,179619156	0,001145334	0,003092929
ZNHIT2	protein_coding	ENSG00000174276	-0,466126341	0,001148277	0,003100469
ANGPT4	protein_coding	ENSG00000101280	2,819459285	0,00115055	0,003106198
TMEM119	protein_coding	ENSG00000183160	2,579480295	0,001151534	0,003108445
CKMT2-AS1	antisense	ENSG00000247572	-0,284841426	0,001154353	0,003115643
FAAP100	protein_coding	ENSG00000185504	-0,292539794	0,001155421	0,003118116
SH3BGR	protein_coding	ENSG00000185437	0,496820011	0,001156436	0,003120444
REXO5	protein_coding	ENSG00000005189	-0,242103394	0,001157632	0,003123262
BCAS1	protein_coding	ENSG00000064787	1,533429718	0,001161247	0,003132603
HIGD2A	protein_coding	ENSG00000146066	-0,205183521	0,001161884	0,003133909
ALDH1L1	protein_coding	ENSG00000144908	2,529201815	0,001166503	0,003145955
KAT2B	protein_coding	ENSG00000114166	0,19005841	0,00116895	0,003152138
TAF9B	protein_coding	ENSG00000187325	-0,207993289	0,001172489	0,003161266
N4BP2L2	protein_coding	ENSG00000244754	-0,177459979	0,001175075	0,003167823
NANOS1	protein_coding	ENSG00000188613	0,568887148	0,001176429	0,003171055
PSG11	protein_coding	ENSG00000243130	1,487660009	0,001176797	0,003171631
LCE1B	protein_coding	ENSG00000196734	1,920929788	0,001177661	0,003173541
AC131235.1	processed_pseudogene	ENSG00000228205	-0,563244596	0,001178042	0,003174152
MTRF1	protein_coding	ENSG00000120662	-0,274499266	0,00117856	0,003175131

ME2	protein_coding	ENSG00000082212	0,164779305	0,001178931	0,003175713
WASHC2C	protein_coding	ENSG00000172661	-0,139279846	0,001179461	0,003176723
GDNF	protein_coding	ENSG00000168621	1,064155955	0,001184441	0,003189718
ITGA2B	protein_coding	ENSG00000005961	1,27706278	0,001185089	0,003191042
C2	protein_coding	ENSG00000166278	1,172239536	0,001186037	0,003193175
AC010618.4	lincRNA	ENSG00000269439	0,96183338	0,001186249	0,003193328
PTPN4	protein_coding	ENSG00000088179	0,171850225	0,001187884	0,00319731
WTIP	protein_coding	ENSG00000142279	-0,252680154	0,001188663	0,003198986
OTUD4	protein_coding	ENSG00000164164	-0,228518106	0,001191044	0,003204974
RNF32	protein_coding	ENSG00000105982	0,435631181	0,001192709	0,003209034
RPL10P7	processed_pseudogene	ENSG00000242052	2,626607362	0,00119669	0,003219323
AF111167.2	antisense	ENSG00000259319	0,879132008	0,001198684	0,003224265
C16orf71	protein_coding	ENSG00000166246	0,924115347	0,001208253	0,003249576
AC068152.1	processed_transcript	ENSG00000262879	0,288707808	0,001209924	0,003253644
C16orf74	protein_coding	ENSG00000154102	-0,4448858	0,001210433	0,003254585
CYP27C1	protein_coding	ENSG00000186684	-0,308199043	0,001211137	0,003256053
ADAM19	protein_coding	ENSG00000135074	-0,213144095	0,001211541	0,00325671
PKP3	protein_coding	ENSG00000184363	-0,26809618	0,001212335	0,00325842
CSDC2	protein_coding	ENSG00000172346	2,041076021	0,001214121	0,003262791
ZBBX	protein_coding	ENSG00000169064	0,715721651	0,001218511	0,00327416
MCTS2P	protein_coding	ENSG00000101898	-0,585310485	0,001219008	0,003275067
AC009962.1	antisense	ENSG00000260742	1,194292266	0,001220257	0,003277846
PTGES3P1	processed_pseudogene	ENSG00000234518	-0,460738465	0,001220363	0,003277846
MIA2	protein_coding	ENSG00000150527	0,16713977	0,001229595	0,003302212
EGFLAM	protein_coding	ENSG00000164318	2,211330472	0,00123034	0,00330378
C17orf78	protein_coding	ENSG00000278505	2,497848667	0,001230973	0,003305048
LIN28A	protein_coding	ENSG00000131914	1,449645204	0,001231179	0,003305169
AFTPH	protein_coding	ENSG00000119844	0,142714088	0,001236994	0,003320344
EHF	protein_coding	ENSG00000135373	1,247471703	0,001238465	0,003323859
TUBGCP5	protein_coding	ENSG00000275835	-0,239132305	0,001245521	0,003341919
PET117	protein_coding	ENSG00000232838	-0,779708599	0,001253168	0,003361558
KLKP1	transcribed_unprocesse	ENSG00000197588	2,307801698	0,001253026	0,003361558
SACS	protein_coding	ENSG00000151835	-0,307088556	0,001254403	0,003364432
AL080250.1	lincRNA	ENSG00000225793	0,87152949	0,00125707	0,003370995
DPF1	protein_coding	ENSG00000011332	-0,29242873	0,001257179	0,003370995
RAB1A	protein_coding	ENSG00000138069	0,160797873	0,001258739	0,003374737
LINC02321	lincRNA	ENSG00000258884	1,144294725	0,001264628	0,003390082
RNF224	protein_coding	ENSG00000233198	2,59757301	0,001266643	0,003395041
LIFR	protein_coding	ENSG00000113594	0,189312404	0,001273329	0,003412514
LINC01465	lincRNA	ENSG00000221949	0,641240682	0,00127496	0,003416441

FAM90A1	protein_coding	ENSG00000171847	2,527669394	0,001275651	0,003417844
AC117394.1	processed_pseudogene	ENSG00000241671	2,786539049	0,001279767	0,003428424
TSSK3	protein_coding	ENSG00000162526	0,882802658	0,001280162	0,003428588
STK35	protein_coding	ENSG00000125834	-0,270723633	0,001280094	0,003428588
AC092368.3	lincRNA	ENSG00000261512	0,77196918	0,001283404	0,003436821
MAN2A1	protein_coding	ENSG00000112893	0,145313675	0,001285546	0,003442109
LINC01985	lincRNA	ENSG00000227260	2,248717093	0,001289764	0,003452951
VPS26A	protein_coding	ENSG00000122958	0,20890226	0,001294993	0,003466497
PIP5KL1	protein_coding	ENSG00000167103	0,726186073	0,001295594	0,003467655
SPIN3	protein_coding	ENSG00000204271	-0,284261423	0,001296564	0,003469797
EXOC3L1	protein_coding	ENSG00000179044	1,801883733	0,001299399	0,00347693
TTC21A	protein_coding	ENSG00000168026	0,525721262	0,001301446	0,003481953
MTIF2	protein_coding	ENSG00000085760	-0,165526867	0,001306215	0,003494257
APOBEC3F	protein_coding	ENSG00000128394	0,41851063	0,001308854	0,00350086
XXYLT1	protein_coding	ENSG00000173950	-0,258758418	0,001309405	0,003501878
AKAP11	protein_coding	ENSG00000023516	0,175728699	0,001317066	0,003521905
LIPJ	protein_coding	ENSG00000204022	1,351852808	0,001319	0,003526618
TNRC6B	protein_coding	ENSG00000100354	0,307883565	0,001319421	0,003526825
TUSC2	protein_coding	ENSG00000114383	-0,219984639	0,001319314	0,003526825
SLC25A34	protein_coding	ENSG00000162461	1,248499933	0,00131974	0,003527218
AC004233.2	protein_coding	ENSG00000270168	2,494591398	0,001321408	0,003531216
IFI30	protein_coding	ENSG00000216490	1,871536786	0,001323088	0,003534994
OR7E102P	unprocessed_pseudoge	ENSG00000168992	2,066972216	0,001323167	0,003534994
ARHGAP4	protein_coding	ENSG00000089820	-0,253184102	0,001324113	0,003537063
NRTN	protein_coding	ENSG00000171119	-0,546676257	0,001326633	0,003543332
UNC50	protein_coding	ENSG00000115446	-0,180842359	0,001327324	0,003544716
KLHL30-AS1	TEC	ENSG00000279484	2,573360371	0,001328023	0,00354612
NOL8	protein_coding	ENSG00000198000	-0,168793899	0,001329853	0,003550544
DDX20	protein_coding	ENSG00000064703	-0,15399928	0,001332243	0,003556463
MPND	protein_coding	ENSG00000008382	-0,315521155	0,001335151	0,003563763
ALDH16A1	protein_coding	ENSG00000161618	-0,303297316	0,001335911	0,003565327
HIST1H3PS1	processed_pseudogene	ENSG00000220875	0,975183672	0,001338062	0,003570601
SFSWAP	protein_coding	ENSG00000061936	-0,186217436	0,001338312	0,003570806
AC105760.2	antisense	ENSG00000227252	-1,25322663	0,001339014	0,003572214
USP6	protein_coding	ENSG00000129204	2,282343565	0,001340869	0,003576698
CD52	protein_coding	ENSG00000169442	2,246202814	0,001341322	0,003577439
AC005840.3	processed_pseudogene	ENSG00000256913	2,77395831	0,001341995	0,003578326
ELF3	protein_coding	ENSG00000163435	1,303465414	0,001342003	0,003578326
AC084757.3	lincRNA	ENSG00000259705	2,532777765	0,001343378	0,003581527
AC020928.2	lincRNA	ENSG00000267260	-0,476561247	0,001344607	0,003584336

ZNF638	protein_coding	ENSG00000075292	-0,170255644	0,001346313	0,003588419
PTPN18	protein_coding	ENSG00000072135	-0,206412049	0,001350086	0,003598008
ACSM3	protein_coding	ENSG00000005187	-0,421870214	0,00135073	0,003599257
CASTOR3	protein_coding	ENSG00000239521	0,43893293	0,001351431	0,003600655
AC132872.4	TEC	ENSG00000280407	1,248919354	0,00135454	0,003608472
SCN7A	protein_coding	ENSG00000136546	2,786120878	0,001358068	0,0036174
POLR3D	protein_coding	ENSG00000168495	0,177209491	0,001358371	0,003617735
PREX1	protein_coding	ENSG00000124126	0,238153325	0,001360409	0,003622694
TMEM161A	protein_coding	ENSG00000064545	-0,233720841	0,001360647	0,003622858
ITGB4	protein_coding	ENSG00000132470	-0,262069638	0,001360831	0,003622878
AC078880.5	lincRNA	ENSG00000276972	2,222922734	0,001361853	0,003625128
LINC00052	lincRNA	ENSG00000259527	-1,728892464	0,001362203	0,003625588
C3orf20	protein_coding	ENSG00000131379	2,231701633	0,001367171	0,003638339
AC092171.3	lincRNA	ENSG00000234432	0,948049171	0,001368709	0,003641959
IL4R	protein_coding	ENSG00000077238	-0,222164602	0,001372059	0,003650401
LINC01176	lincRNA	ENSG00000281404	1,713289411	0,001378541	0,00366717
CNGA1	protein_coding	ENSG00000198515	0,752834071	0,001378952	0,003667788
STK40	protein_coding	ENSG00000196182	0,23306078	0,001381232	0,003673375
GRB7	protein_coding	ENSG00000141738	0,999163314	0,001382109	0,003675231
ANO10	protein_coding	ENSG00000160746	0,133718893	0,00138273	0,003676406
MIR4500HG	lincRNA	ENSG00000228824	0,698335777	0,001384602	0,003680904
COQ8A	protein_coding	ENSG00000163050	-0,2002399	0,001385292	0,003682263
PIP5K1C	protein_coding	ENSG00000186111	-0,256954589	0,001386344	0,003684582
TMF1	protein_coding	ENSG00000144747	0,16109667	0,001393517	0,003703165
SPNS3	protein_coding	ENSG00000182557	1,036550115	0,001394043	0,003704084
WAC	protein_coding	ENSG00000095787	-0,17580728	0,001398397	0,003715172
LARP1	protein_coding	ENSG00000155506	-0,24476624	0,001403369	0,003727898
ITLN2	protein_coding	ENSG00000158764	2,926286554	0,00140723	0,00373767
CLIC4	protein_coding	ENSG00000169504	0,164134729	0,001409898	0,00374427
LHFPL2	protein_coding	ENSG00000145685	-0,205942254	0,001410609	0,003745674
AC099518.1	lincRNA	ENSG00000260430	1,325166294	0,001410851	0,003745832
MVP	protein_coding	ENSG00000013364	-0,199738633	0,001412161	0,003748826
GPR107	protein_coding	ENSG00000148358	0,131309121	0,001413208	0,003751118
SORBS2	protein_coding	ENSG00000154556	-0,214388794	0,00141762	0,003762342
SASS6	protein_coding	ENSG00000156876	-0,265157262	0,001421214	0,003771394
SGCA	protein_coding	ENSG00000108823	0,940976762	0,001424103	0,003778572
PTPRU	protein_coding	ENSG00000060656	0,999328338	0,001424971	0,003780386
OAZ3	protein_coding	ENSG00000143450	0,694769738	0,001426068	0,003782807
UPK2	protein_coding	ENSG00000110375	2,546992469	0,001431357	0,003796312
HCLS1	protein_coding	ENSG00000180353	1,020677655	0,00143153	0,003796312

RFPL4B	protein_coding	ENSG00000251258	2,459653228	0,001434689	0,003804197
PEX3	protein_coding	ENSG00000034693	-0,259778209	0,001435996	0,003807172
ZNF549	protein_coding	ENSG00000121406	-0,234396437	0,001436356	0,003807633
FTX	lincRNA	ENSG00000230590	0,343681311	0,001436872	0,003808508
PKP4	protein_coding	ENSG00000144283	-0,164134113	0,001437374	0,003809347
TYW3	protein_coding	ENSG00000162623	-0,179453577	0,001438942	0,003813009
BTC	protein_coding	ENSG00000174808	1,159687339	0,001440126	0,003815161
RAB12	protein_coding	ENSG00000206418	0,215577099	0,001440006	0,003815161
CFD	protein_coding	ENSG00000197766	-0,279098429	0,001443724	0,003824199
SLC25A1	protein_coding	ENSG00000100075	-0,230846813	0,001447393	0,003833424
FOXRED1	protein_coding	ENSG00000110074	-0,212891656	0,001448747	0,003836514
AC008440.1	antisense	ENSG00000228323	1,726455485	0,001452446	0,003845812
AC027607.1	antisense	ENSG00000248936	2,478212032	0,001452756	0,003846137
HOOK3	protein_coding	ENSG00000168172	0,208908852	0,001454253	0,003849603
AC011365.1	antisense	ENSG00000254042	2,243311035	0,001454763	0,003850456
DDX6	protein_coding	ENSG00000110367	-0,165762574	0,001455491	0,003851887
FRMPD4	protein_coding	ENSG00000169933	1,571296588	0,001457673	0,003857164
GAS6	protein_coding	ENSG00000183087	-0,249011047	0,001459391	0,003861211
MIF	protein_coding	ENSG00000240972	-0,225952537	0,001461973	0,003867543
DEDD	protein_coding	ENSG00000158796	0,212398289	0,001463648	0,003871476
EEF1A2	protein_coding	ENSG00000101210	0,201701315	0,00146548	0,003875821
LPAR6	protein_coding	ENSG00000139679	2,053309491	0,001466874	0,003879008
PRTG	protein_coding	ENSG00000166450	-0,319147995	0,001469309	0,003884947
ASF1A	protein_coding	ENSG00000111875	0,233383885	0,001470468	0,003887509
AL021707.1	antisense	ENSG00000225450	1,804000164	0,001474144	0,003896726
DDX10	protein_coding	ENSG00000178105	-0,177193645	0,001476909	0,003903532
RAP1B	protein_coding	ENSG00000127314	0,222678641	0,001477714	0,003905155
BLVRB	protein_coding	ENSG00000090013	0,267040029	0,001481122	0,003913659
AC090193.1	antisense	ENSG00000253286	2,808990969	0,00148143	0,003913966
GACAT2	antisense	ENSG00000265962	1,154174677	0,001484291	0,00392102
RPL13P5	transcribed_processed_	ENSG00000240370	-0,609764686	0,001484582	0,003921284
FES	protein_coding	ENSG00000182511	1,539426585	0,001485622	0,003923527
AC099850.1	antisense	ENSG00000224738	0,562093704	0,001488983	0,003931896
AC017104.5	unprocessed_pseudoge	ENSG00000283491	0,810691469	0,001496294	0,003950694
SNX9	protein_coding	ENSG00000130340	-0,155982341	0,001497537	0,003953466
PORCN	protein_coding	ENSG00000102312	0,230830312	0,001499736	0,003958762
ATP1A1-AS1	processed_transcript	ENSG00000203865	1,02183238	0,00150021	0,003959505
SMIM24	protein_coding	ENSG00000095932	2,463731199	0,001500784	0,00396051
PBRM1	protein_coding	ENSG00000163939	-0,214966313	0,001503452	0,003967041
SSC4D	protein_coding	ENSG00000146700	0,868142498	0,001504591	0,003969536

STAT4	protein_coding	ENSG00000138378	1,11095554	0,001506811	0,003974881
AC009487.2	processed_pseudogene	ENSG00000226121	-0,43900367	0,001508449	0,003978691
CDK6	protein_coding	ENSG00000105810	0,219757157	0,001511075	0,003985103
ZNF511	protein_coding	ENSG00000198546	-0,308373132	0,001512344	0,003987937
RGS9	protein_coding	ENSG00000108370	0,473944551	0,001513467	0,003990208
SLC25A13	protein_coding	ENSG00000004864	-0,183354327	0,001513594	0,003990208
ZNF507	protein_coding	ENSG00000168813	0,184873258	0,001517292	0,003999441
COL9A3	protein_coding	ENSG00000092758	1,657157414	0,001520713	0,004007945
NTPCR	protein_coding	ENSG00000135778	-0,226023325	0,001524248	0,004016744
COTL1	protein_coding	ENSG00000103187	0,126442507	0,001525297	0,004018994
KCNJ11	protein_coding	ENSG00000187486	-1,017111225	0,001534527	0,004042794
VPS18	protein_coding	ENSG00000104142	0,248443167	0,001537896	0,004051148
TTC38	protein_coding	ENSG00000075234	-0,230685065	0,001538235	0,004051521
LINC00909	lincRNA	ENSG00000264247	0,319598855	0,001539129	0,004053355
CAPZB	protein_coding	ENSG00000077549	0,122046108	0,001540411	0,004055169
ARL16	protein_coding	ENSG00000214087	-0,192992011	0,001540324	0,004055169
PPIA	protein_coding	ENSG00000196262	-0,209855873	0,00154016	0,004055169
CASP1	protein_coding	ENSG00000137752	0,465057	0,001544624	0,004065739
ZBED3-AS1	antisense	ENSG00000250802	0,613494716	0,001547379	0,004072313
FGF14-AS2	lincRNA	ENSG00000272143	-0,910829425	0,001547519	0,004072313
PLXNB2	protein_coding	ENSG00000196576	0,260887135	0,00154785	0,004072663
AC120053.1	antisense	ENSG00000271971	1,568904753	0,001549197	0,004075683
KLHL31	protein_coding	ENSG00000124743	2,758717247	0,001550254	0,004077943
CHADL	protein_coding	ENSG00000100399	1,410739198	0,00155326	0,004085324
GCM1	protein_coding	ENSG00000137270	2,479432211	0,001559418	0,004100996
ANXA2P2	processed_pseudogene	ENSG00000231991	-0,345279772	0,001566457	0,004118979
AC002480.1	antisense	ENSG00000232759	0,355902806	0,001567093	0,004119595
ACVR1C	protein_coding	ENSG00000123612	-0,554716066	0,001567071	0,004119595
STAG3L4	transcribed_unprocesse	ENSG00000106610	-0,308497544	0,001569977	0,004126648
SMDT1	protein_coding	ENSG00000183172	0,329390718	0,001578105	0,004147009
PSAP	protein_coding	ENSG00000197746	0,148260341	0,00157833	0,004147009
ITPA	protein_coding	ENSG00000125877	-0,23722243	0,00157825	0,004147009
C2orf27A	transcribed_unprocesse	ENSG00000197927	0,332299338	0,001580999	0,00415349
AC084082.1	lincRNA	ENSG00000253190	-0,553166869	0,001587656	0,004170445
LPAR1	protein_coding	ENSG00000198121	-0,1697888	0,001588166	0,00417125
LINC02150	lincRNA	ENSG00000248150	1,789262017	0,001592169	0,004181228
TIMM23	protein_coding	ENSG00000265354	-0,193909036	0,001597117	0,004193686
LRBA	protein_coding	ENSG00000198589	0,16599837	0,001599112	0,004198384
LIMD2	protein_coding	ENSG00000136490	-0,472028217	0,001603449	0,004209233
LNCAROD	lincRNA	ENSG00000231131	-0,321653181	0,00160652	0,004216755

NACA	protein_coding	ENSG00000196531	-0,171060495	0,001607049	0,004217604
SNAPC2	protein_coding	ENSG00000104976	-0,23647847	0,001608336	0,004220441
C15orf54	lincRNA	ENSG00000175746	0,989555408	0,001611314	0,004227716
CACNA2D1	protein_coding	ENSG00000153956	0,211553022	0,001613735	0,004233527
HEXIM2	protein_coding	ENSG00000168517	0,333136737	0,001621052	0,004252148
SLC20A2	protein_coding	ENSG00000168575	0,167984358	0,001621248	0,004252148
MSL2	protein_coding	ENSG00000174579	0,180971545	0,001623352	0,004257122
RBM4	protein_coding	ENSG00000173933	-0,337837573	0,001624528	0,00425966
AC004130.1	antisense	ENSG00000271133	2,021334606	0,001625294	0,004261125
CLTRN	protein_coding	ENSG00000147003	2,432766015	0,001625596	0,004261373
SNORD72	snoRNA	ENSG00000212296	-1,188688215	0,001628089	0,004267361
AC008079.1	antisense	ENSG00000280007	2,70217836	0,001630182	0,004271824
LZIC	protein_coding	ENSG00000162441	-0,219117099	0,001630208	0,004271824
AC069499.1	processed_pseudogene	ENSG00000241634	-0,437027594	0,001632872	0,004278259
ATP5PO	protein_coding	ENSG00000241837	-0,258308439	0,001634034	0,004280755
CCDC190	protein_coding	ENSG00000185860	0,742686666	0,001636354	0,004286287
MTRR	protein_coding	ENSG00000124275	-0,161822662	0,001638499	0,004291357
NAA35	protein_coding	ENSG00000135040	0,156623721	0,001640737	0,004296668
AL590399.1	lincRNA	ENSG00000204802	1,244184187	0,001641756	0,004298788
MYO9A	protein_coding	ENSG00000066933	-0,21600302	0,001644897	0,004306464
PLEKHH1	protein_coding	ENSG00000054690	-0,471093447	0,001659266	0,004343529
UTP11	protein_coding	ENSG00000183520	-0,256258526	0,001660232	0,004345503
TNPO2	protein_coding	ENSG00000105576	0,254875015	0,00166098	0,004346906
ATRN	protein_coding	ENSG00000088812	-0,213093773	0,001665531	0,004357704
FRG2FP	transcribed_unprocesse	ENSG00000232783	1,139283726	0,001665479	0,004357704
PMS2CL	transcribed_unprocesse	ENSG00000187953	0,324474982	0,001667644	0,004362676
OSTF1	protein_coding	ENSG00000134996	-0,222225177	0,001669524	0,004367038
HCG15	antisense	ENSG00000227214	1,001636518	0,001670299	0,004368507
B4GALNT4	protein_coding	ENSG00000182272	-0,261797212	0,001671834	0,004371963
SMCR8	protein_coding	ENSG00000176994	0,178026641	0,001676801	0,004384394
KLHL24	protein_coding	ENSG00000114796	0,176058071	0,001679499	0,004390888
AL365436.2	sense_intronic	ENSG00000232536	1,285513222	0,001680739	0,00439357
RNF139	protein_coding	ENSG00000170881	-0,194178993	0,001681521	0,004395054
MAGEA1	protein_coding	ENSG00000198681	-0,580678489	0,001682466	0,004396837
ZNF300P1	transcribed_unprocesse	ENSG00000197083	2,897804383	0,001682631	0,004396837
SCRN2	protein_coding	ENSG00000141295	-0,229640088	0,001686611	0,004406113
ZBTB24	protein_coding	ENSG00000112365	-0,204443216	0,001687215	0,004407129
FP565260.3	protein_coding	ENSG00000277117	1,065418841	0,001688039	0,004408719
FLYWCH1	protein_coding	ENSG00000059122	0,30071905	0,001691935	0,004418333
LINC01952	antisense	ENSG00000234183	-1,422720134	0,00169497	0,004425694

AC092807.3	lincRNA	ENSG00000282057	-0,200990697	0,001695489	0,004426486
ATIC	protein_coding	ENSG00000138363	-0,229082917	0,001699393	0,004436115
FAXC	protein_coding	ENSG00000146267	-0,494768666	0,001704874	0,004449855
ATXN3	protein_coding	ENSG00000066427	-0,220005914	0,001708464	0,004458658
C11orf91	protein_coding	ENSG00000205177	1,483410239	0,001710137	0,004462456
CDH1	protein_coding	ENSG00000039068	2,452462891	0,001713349	0,00447027
AL161937.1	lincRNA	ENSG00000234698	1,469143442	0,001715155	0,004474412
FITM2	protein_coding	ENSG00000197296	-0,186605732	0,001720102	0,004486746
AL355974.1	lincRNA	ENSG00000274331	1,610419775	0,001721355	0,004489443
AC104447.1	TEC	ENSG00000280173	-0,606731506	0,001722508	0,004491881
FAM117A	protein_coding	ENSG00000121104	0,319839334	0,001725449	0,004498977
ZNF451	protein_coding	ENSG00000112200	0,188046751	0,001729074	0,004507855
MED13L	protein_coding	ENSG00000123066	0,239427617	0,001732504	0,004516226
AC117402.1	lincRNA	ENSG00000206532	0,957946601	0,001733259	0,004517619
RRAGB	protein_coding	ENSG00000083750	-0,2190184	0,001737981	0,004529352
AC009139.1	antisense	ENSG00000260922	0,75224379	0,001739048	0,004531556
SATB2	protein_coding	ENSG00000119042	0,214079852	0,001739956	0,004533345
ZNF780B	protein_coding	ENSG00000128000	-0,175168993	0,001740654	0,004534587
C6orf15	protein_coding	ENSG00000204542	2,451480897	0,001741527	0,004536288
LINC01704	lincRNA	ENSG00000231666	1,544561557	0,001741865	0,004536592
DCAF4L1	protein_coding	ENSG00000182308	1,142332518	0,001742141	0,004536732
DLGAP4	protein_coding	ENSG00000080845	0,209529188	0,001743813	0,004540511
APMAP	protein_coding	ENSG00000101474	-0,151489607	0,001745857	0,004545256
GUCA1B	protein_coding	ENSG00000112599	0,792128336	0,001753633	0,004564921
ALKBH5	protein_coding	ENSG00000091542	-0,153664862	0,001753874	0,004564968
THG1L	protein_coding	ENSG00000113272	-0,218252319	0,00175628	0,004570651
LIMS1	protein_coding	ENSG00000169756	0,196691075	0,001759239	0,004577771
HIST1H3J	protein_coding	ENSG00000197153	0,992437726	0,001760019	0,004579194
EHD4	protein_coding	ENSG00000103966	0,150144187	0,001760232	0,004579194
RN7SL809P	misc_RNA	ENSG00000241217	-1,502504915	0,00176513	0,004591354
BCAT1	protein_coding	ENSG00000060982	0,161508117	0,001768975	0,004600771
SNX14	protein_coding	ENSG00000135317	-0,191128536	0,001776234	0,004619065
LRRC37A11P	transcribed_unprocesse	ENSG00000214553	1,871612397	0,00178103	0,00463095
SLC34A3	protein_coding	ENSG00000198569	2,672212378	0,001786478	0,004643937
GAA	protein_coding	ENSG00000171298	0,324273683	0,001787307	0,004645503
GRIK5	protein_coding	ENSG00000105737	1,389287133	0,001788606	0,004648289
AL133353.1	bidirectional_promoter_	ENSG00000229278	1,183731314	0,001796159	0,004667329
EFL1	protein_coding	ENSG00000140598	0,123822559	0,001796681	0,004668093
RPS14	protein_coding	ENSG00000164587	-0,260128847	0,001798045	0,004670454
SNORA38	snoRNA	ENSG00000200816	-0,409427455	0,00179788	0,004670454

TIGD5	protein_coding	ENSG00000179886	-0,354322371	0,00180231	0,004680941
SYNE4	protein_coding	ENSG00000181392	1,542451001	0,001808172	0,004695569
ZSWIM1	protein_coding	ENSG00000168612	-0,268126665	0,001809462	0,004698325
AIM2	protein_coding	ENSG00000163568	4,396532321	0,001818879	0,004721582
AC083899.2	processed_pseudogene	ENSG00000233671	1,255003459	0,001820222	0,00472447
YBEY	protein_coding	ENSG00000182362	-0,247484704	0,001820591	0,004724828
FUT8-AS1	antisense	ENSG00000276116	0,566902484	0,001821489	0,004725963
IZUMO1	protein_coding	ENSG00000182264	2,195515476	0,001821349	0,004725963
SPAG16	protein_coding	ENSG00000144451	-0,275630314	0,001833321	0,004756059
GLDN	protein_coding	ENSG00000186417	0,787489835	0,001837099	0,004765258
ISCA1P4	processed_pseudogene	ENSG00000259405	2,172329893	0,001844879	0,004784834
CDKL3	protein_coding	ENSG00000006837	0,819529537	0,001846355	0,004787451
CLCN7	protein_coding	ENSG00000103249	0,246495372	0,001846126	0,004787451
LINC00485	lincRNA	ENSG00000258169	1,390679852	0,001849067	0,004793272
ITGB3BP	protein_coding	ENSG00000142856	-0,289492864	0,001849001	0,004793272
NAXE	protein_coding	ENSG00000163382	-0,242652754	0,001849828	0,004794639
AC016650.1	antisense	ENSG00000250981	2,389800767	0,001857987	0,004815177
SAMM50	protein_coding	ENSG00000100347	-0,14879286	0,001863998	0,004830144
COPS2	protein_coding	ENSG00000166200	0,210725779	0,001870285	0,004845824
CPSF6	protein_coding	ENSG00000111605	-0,1705538	0,001870818	0,004846592
LINC01684	lincRNA	ENSG00000237484	0,93831804	0,001871552	0,004847882
NOP9	protein_coding	ENSG00000196943	-0,210331597	0,001873765	0,004853002
MVB12B	protein_coding	ENSG00000196814	-0,266281101	0,00187731	0,004861569
SUGCT	protein_coding	ENSG00000175600	0,260632018	0,001878929	0,004864533
CHMP6	protein_coding	ENSG00000176108	-0,197512644	0,001878908	0,004864533
AP001505.1	lincRNA	ENSG00000276529	-0,664017747	0,001879451	0,004865271
AC027031.2	lincRNA	ENSG00000254615	-0,323161026	0,001891113	0,004894841
LINC01772	lincRNA	ENSG00000226029	0,753335387	0,001895633	0,004904685
SH3GLB1	protein_coding	ENSG00000097033	0,147107407	0,001895562	0,004904685
MRPL48	protein_coding	ENSG00000175581	-0,268444513	0,001895492	0,004904685
DALRD3	protein_coding	ENSG00000178149	-0,268177767	0,001902998	0,004923119
SRL	protein_coding	ENSG00000185739	2,665081077	0,001908559	0,004936262
TRAPPC10	protein_coding	ENSG00000160218	0,220323599	0,001915364	0,004952612
EXO5	protein_coding	ENSG00000164002	-0,195983926	0,0019152	0,004952612
CIR1	protein_coding	ENSG00000138433	0,194371236	0,001920386	0,00496497
TTC13	protein_coding	ENSG00000143643	0,163970322	0,001924516	0,004975023
COMMD9	protein_coding	ENSG00000110442	-0,187663561	0,001936589	0,0050056
GRIN2D	protein_coding	ENSG00000105464	0,562447931	0,001938041	0,00500839
SLAIN2	protein_coding	ENSG00000109171	0,178893576	0,001938156	0,00500839
ARMC9	protein_coding	ENSG00000135931	-0,20723143	0,001942812	0,005019786

PDHA1	protein_coding	ENSG00000131828	-0,128401022	0,00194367	0,005021372
PDE12	protein_coding	ENSG00000174840	0,132045633	0,001946598	0,005028304
AC005899.6	lincRNA	ENSG00000274341	2,175307351	0,001947373	0,00502967
RSRC1	protein_coding	ENSG00000174891	-0,228253434	0,001950932	0,005038228
ITGB1-DT	lincRNA	ENSG00000229656	0,290605086	0,001958176	0,0050563
PTEN	protein_coding	ENSG00000171862	0,128394724	0,001960622	0,005061977
UQCC2	protein_coding	ENSG00000137288	-0,266779233	0,001961275	0,005063027
NPIPB2	protein_coding	ENSG00000234719	2,383575038	0,001963877	0,005068409
PXN	protein_coding	ENSG00000089159	-0,192795663	0,00196369	0,005068409
AFMID	protein_coding	ENSG00000183077	-0,231836926	0,001964101	0,005068409
AC025754.2	lincRNA	ENSG00000271874	1,578572682	0,001965985	0,005072632
ABHD6	protein_coding	ENSG00000163686	0,250482713	0,001971797	0,005086989
LINC02085	lincRNA	ENSG00000214407	0,617765412	0,001977445	0,005100917
LINC01607	lincRNA	ENSG00000272138	2,382716813	0,001979661	0,005105991
MOGAT1	protein_coding	ENSG00000124003	1,85781257	0,001980777	0,005108228
DUSP3	protein_coding	ENSG00000108861	-0,094266601	0,001990953	0,005133825
EGF	protein_coding	ENSG00000138798	0,82192272	0,001996294	0,005146951
PILRB	protein_coding	ENSG00000121716	-0,45251755	0,001997504	0,005149424
PGAP3	protein_coding	ENSG00000161395	-0,335159048	0,001999813	0,005154728
TXNDC5	protein_coding	ENSG00000239264	-0,466614906	0,002002664	0,005161428
COL5A3	protein_coding	ENSG00000080573	1,331870592	0,00200417	0,005164659
YWHAQ	protein_coding	ENSG00000134308	-0,158644321	0,002009782	0,005178471
AC010280.2	lincRNA	ENSG00000248884	2,203370696	0,002011557	0,005182393
ZNF189	protein_coding	ENSG00000136870	0,221490466	0,002015557	0,005192045
AL356056.2	antisense	ENSG00000231187	0,791070849	0,002015946	0,005192396
SBF1	protein_coding	ENSG00000100241	0,260427473	0,002018606	0,005198553
MRPL45	protein_coding	ENSG00000278845	-0,234132907	0,002018843	0,005198553
MAP3K2-DT	lincRNA	ENSG00000236682	0,532572638	0,002022981	0,005208555
DLEU1	processed_transcript	ENSG00000176124	-0,36878559	0,002024322	0,005211353
AC012313.2	antisense	ENSG00000268049	0,771087735	0,002025372	0,005213402
EML2	protein_coding	ENSG00000125746	0,217795459	0,002028352	0,005220418
DHCR7	protein_coding	ENSG00000172893	0,202514161	0,002030059	0,005224153
DZIP1L	protein_coding	ENSG00000158163	-0,233039466	0,002031963	0,005228398
MRPL10	protein_coding	ENSG00000159111	-0,190794198	0,002038888	0,005245559
FOPNL	protein_coding	ENSG00000133393	-0,235514983	0,002044399	0,005259074
SIAH1	protein_coding	ENSG00000196470	-0,320575292	0,002044654	0,005259074
LINC02522	lincRNA	ENSG00000231056	1,779254055	0,002045488	0,005260506
FIZ1	protein_coding	ENSG00000179943	-0,362338238	0,002045724	0,005260506
NT5M	protein_coding	ENSG00000205309	-0,332217483	0,002046038	0,005260654
BIRC6	protein_coding	ENSG00000115760	0,209193898	0,002050921	0,005272549

SH2B2	protein_coding	ENSG00000160999	-0,571807886	0,002055823	0,005284487
AC097103.2	antisense	ENSG00000248932	0,417085938	0,00206077	0,00529654
CCDC62	protein_coding	ENSG00000130783	0,515459849	0,002062249	0,005299677
AGMO	protein_coding	ENSG00000187546	1,18521666	0,002071139	0,005321857
TAGLN3	protein_coding	ENSG00000144834	1,808234944	0,002073379	0,005326946
P2RY8	protein_coding	ENSG00000182162	2,501112101	0,002077009	0,005335603
ATP6V1G1	protein_coding	ENSG00000136888	0,200326866	0,002079499	0,00534133
GFRA1	protein_coding	ENSG00000151892	1,060279595	0,002083072	0,005349838
AC005083.1	processed_transcript	ENSG00000233834	2,722753405	0,002083529	0,005350341
POLR2B	protein_coding	ENSG00000047315	-0,149180278	0,002083967	0,005350797
SAE1	protein_coding	ENSG00000142230	-0,120282508	0,00208464	0,005351587
SSB	protein_coding	ENSG00000138385	-0,217436407	0,002084797	0,005351587
LOXL3	protein_coding	ENSG00000115318	0,737043404	0,002085708	0,005353257
TRIM29	protein_coding	ENSG00000137699	2,394311039	0,002087415	0,005356966
AP003469.4	lincRNA	ENSG00000261087	1,451124826	0,002088185	0,005358274
IGLV1-51	IG_V_gene	ENSG00000211644	1,434848237	0,002088525	0,005358475
SRRM1	protein_coding	ENSG00000133226	-0,224787288	0,002088793	0,005358492
AC007405.3	lincRNA	ENSG00000239467	0,548445921	0,002098568	0,005382895
TRIM54	protein_coding	ENSG00000138100	2,421245251	0,002110355	0,005411776
C19orf53	protein_coding	ENSG00000104979	-0,213745543	0,002111344	0,005413635
UBALD2	protein_coding	ENSG00000185262	0,25378999	0,002112205	0,005415165
HLA-DOA	protein_coding	ENSG00000204252	1,103451059	0,002114987	0,00542162
RPS2P32	processed_pseudogene	ENSG00000232818	-0,439743469	0,002122333	0,005439771
CCDC88B	protein_coding	ENSG00000168071	0,583259434	0,002124166	0,00544336
TRMT61B	protein_coding	ENSG00000171103	-0,203348863	0,002124264	0,00544336
AC020661.3	antisense	ENSG00000259617	2,724368355	0,002130816	0,005459468
KIF5B	protein_coding	ENSG00000170759	0,157093577	0,002132861	0,005464025
TMEM165	protein_coding	ENSG00000134851	-0,206702491	0,002139992	0,005481607
ENPP4	protein_coding	ENSG00000001561	0,229923234	0,002141225	0,005484082
RABL2B	protein_coding	ENSG00000079974	-0,351234884	0,002146267	0,005496308
AMBRA1	protein_coding	ENSG00000110497	0,194613788	0,002147862	0,005499707
KDM4C	protein_coding	ENSG00000107077	0,187292564	0,002151233	0,005507652
SNORA33	snoRNA	ENSG00000200534	-0,635692643	0,002162233	0,005535121
LINC01841	lincRNA	ENSG00000266913	1,513191044	0,002162717	0,00553567
POGZ	protein_coding	ENSG00000143442	-0,20324479	0,002164449	0,005539411
OSBPL8	protein_coding	ENSG00000091039	0,193335518	0,002172328	0,005558883
SERAC1	protein_coding	ENSG00000122335	-0,263910543	0,002179482	0,005576493
EIF3A	protein_coding	ENSG00000107581	-0,219693296	0,002184411	0,005588409
SPRED1	protein_coding	ENSG00000166068	-0,186061516	0,002184708	0,005588472
SHANK3	protein_coding	ENSG00000251322	-0,349082828	0,002192319	0,00560724

MEF2D	protein_coding	ENSG00000116604	0,211662777	0,002193634	0,005609904
LINC01907	lincRNA	ENSG00000226125	2,655774032	0,002196733	0,005616409
ATP8A2	protein_coding	ENSG00000132932	0,378162781	0,002196999	0,005616409
SLC7A6	protein_coding	ENSG00000103064	-0,185367504	0,00219677	0,005616409
CENPBD1	protein_coding	ENSG00000177946	-0,216049644	0,002198946	0,005620687
AC078883.2	antisense	ENSG00000226963	1,522834548	0,002200812	0,005623353
AP001412.1	antisense	ENSG00000272948	-1,078750423	0,00220075	0,005623353
NBPF25P	transcribed_unprocesse	ENSG00000272150	0,526253836	0,002200431	0,005623353
AFG1L	protein_coding	ENSG00000135537	0,291624219	0,002202313	0,005626488
ITGB7	protein_coding	ENSG00000139626	1,697094149	0,002203603	0,005629081
HNRNPA1P7	processed_pseudogene	ENSG00000215492	-0,618351393	0,002206186	0,005634277
ARHGEF19	protein_coding	ENSG00000142632	-0,246800589	0,002205923	0,005634277
AP001970.1	antisense	ENSG00000254710	2,355198748	0,002207343	0,00563653
MTPAP	protein_coding	ENSG00000107951	-0,160255271	0,00220819	0,005637992
BASP1	protein_coding	ENSG00000176788	0,116063267	0,002210464	0,005643095
FAM71F1	protein_coding	ENSG00000135248	1,520038418	0,002213494	0,005649424
SREK1IP1	protein_coding	ENSG00000153006	-0,255908431	0,002213304	0,005649424
OBSCN	protein_coding	ENSG00000154358	0,535343475	0,002214744	0,00565191
PPP1R26-AS1	antisense	ENSG00000225361	0,50159881	0,002217059	0,005657115
SNORD100	snoRNA	ENSG00000221500	-0,614032999	0,002221345	0,005667345
RIMS4	protein_coding	ENSG00000101098	1,063219115	0,002225516	0,00567728
SLC25A22	protein_coding	ENSG00000177542	-0,259721437	0,002239164	0,005711387
NCCRP1	protein_coding	ENSG00000188505	2,321312543	0,002239953	0,005712687
LYRM1	protein_coding	ENSG00000102897	0,251891449	0,002240626	0,005713694
ALOX15	protein_coding	ENSG00000161905	2,586955349	0,002245927	0,005726499
GBF1	protein_coding	ENSG00000107862	0,214710082	0,002248593	0,005732585
VSIG10	protein_coding	ENSG00000176834	-0,148042806	0,002252976	0,005743044
SLC9A3-AS1	processed_transcript	ENSG00000225138	-0,387266999	0,002254595	0,005746458
CLCN5	protein_coding	ENSG00000171365	-0,162698885	0,002258599	0,005755948
CDK16	protein_coding	ENSG00000102225	-0,148178806	0,002264304	0,00576977
ZNF266	protein_coding	ENSG00000174652	-0,216625721	0,00226734	0,005776789
ACBD6	protein_coding	ENSG00000230124	-0,122596259	0,002268025	0,005777816
ADCY8	protein_coding	ENSG00000155897	1,922906404	0,002269801	0,005781622
AC011468.5	antisense	ENSG00000275055	-0,747613771	0,00227024	0,005782021
FAAP24	protein_coding	ENSG00000131944	-0,273010862	0,00227186	0,00578543
HPS4	protein_coding	ENSG00000100099	-0,181832017	0,002276726	0,005797102
PDE4DIP	protein_coding	ENSG00000178104	0,167525408	0,002285536	0,005818811
YTHDF3	protein_coding	ENSG00000185728	0,115187203	0,002289344	0,005827782
S100A13	protein_coding	ENSG00000189171	0,218576206	0,002292412	0,005834867
XIAPP3	processed_pseudogene	ENSG00000180152	1,441961734	0,002295936	0,005842189

MYL10	protein_coding	ENSG00000106436	2,328989243	0,002296143	0,005842189
PLCB2	protein_coding	ENSG00000137841	0,903881842	0,002295677	0,005842189
AL021154.1	lincRNA	ENSG00000235052	1,331527166	0,002298438	0,005847303
SDHA	protein_coding	ENSG00000073578	-0,112014623	0,002301812	0,005855162
AF131215.6	sense_intronic	ENSG00000269918	0,542310863	0,002315582	0,005889457
MAGOH	protein_coding	ENSG00000162385	-0,206398929	0,002320134	0,005900304
PGBD2	protein_coding	ENSG00000185220	-0,290518603	0,002328134	0,005919914
ANKRD36C	protein_coding	ENSG00000174501	-0,241182773	0,002328912	0,005921159
ZNF285	protein_coding	ENSG00000267508	-0,381556939	0,002330997	0,005925724
PRKCH	protein_coding	ENSG00000027075	0,256144129	0,002332093	0,005927776
AC005540.1	antisense	ENSG00000235852	0,737737344	0,00233411	0,005932169
AC116562.4	transcribed_unprocesse	ENSG00000284727	0,760940378	0,002339317	0,005944666
MYO18B	protein_coding	ENSG00000133454	2,355141367	0,002342134	0,005951087
KCTD21	protein_coding	ENSG00000188997	-0,289214763	0,002348131	0,005965584
TMEM178B	protein_coding	ENSG00000261115	-0,268794811	0,002357439	0,005988491
PDK3	protein_coding	ENSG00000067992	-0,638245965	0,002359541	0,005993088
RPL7L1P12	processed_pseudogene	ENSG00000231981	2,587863666	0,002362609	0,006000137
TMEM35A	protein_coding	ENSG00000126950	2,295395198	0,002365211	0,006006002
AC245052.4	antisense	ENSG00000237017	2,345876075	0,002369167	0,006015303
LAMTOR3	protein_coding	ENSG00000109270	0,204388559	0,002370328	0,006017118
CLN5	protein_coding	ENSG00000102805	0,188871398	0,002370469	0,006017118
CCDC138	protein_coding	ENSG00000163006	-0,292600408	0,002371324	0,006018545
ROS1	protein_coding	ENSG00000047936	2,299278936	0,002373172	0,00602249
TCAF2	protein_coding	ENSG00000170379	0,696148365	0,002378868	0,006036196
COMMD8	protein_coding	ENSG00000169019	-0,302396644	0,002381092	0,006041092
MSTO1	protein_coding	ENSG00000125459	0,248813161	0,002393185	0,006071024
SNORD116-18	snoRNA	ENSG00000206688	1,343140817	0,002395782	0,00607686
C4A	protein_coding	ENSG00000244731	0,915100153	0,002396584	0,006078143
C4A	protein_coding	ENSG00000244731	0,915100153	0,002396584	0,006078143
CDK19	protein_coding	ENSG00000155111	0,230015263	0,002411176	0,006114393
TMCO6	protein_coding	ENSG00000113119	-0,286976368	0,002411715	0,006115004
PGK1	protein_coding	ENSG00000102144	-0,163580302	0,002414136	0,006120387
AP000790.1	processed_transcript	ENSG00000214788	2,695126635	0,00241452	0,006120605
AC114489.1	antisense	ENSG00000224409	2,366961549	0,002418402	0,006129686
JAZF1-AS1	antisense	ENSG00000234336	2,600732172	0,002419948	0,006132848
ARID1B	protein_coding	ENSG00000049618	-0,213153074	0,002423646	0,006141461
SNAI1	protein_coding	ENSG00000124216	1,239935564	0,002425156	0,006144528
SLC4A2	protein_coding	ENSG00000164889	0,247795458	0,002426962	0,006148345
TRMT112	protein_coding	ENSG00000173113	-0,188063784	0,0024303	0,006156039
WDR24	protein_coding	ENSG00000127580	-0,27587953	0,002433756	0,006164033

TIAM1	protein_coding	ENSG00000156299	0,206015269	0,002438105	0,006174284
CYP26B1	protein_coding	ENSG00000003137	2,432511237	0,002439712	0,006177593
SEPT7P1	processed_pseudogene	ENSG00000259090	2,634272185	0,002443266	0,006185828
NME7	protein_coding	ENSG00000143156	0,146289462	0,002451886	0,006206884
PCDH1	protein_coding	ENSG00000156453	0,333475968	0,002454397	0,006212474
SLC44A1	protein_coding	ENSG00000070214	-0,173261834	0,002466039	0,006241173
AC010359.1	lincRNA	ENSG00000269961	0,968670025	0,002467055	0,00624273
OR6E1P	unprocessed_pseudoge	ENSG00000235213	1,342844396	0,002467263	0,00624273
ZC3H12D	protein_coding	ENSG00000178199	2,277272481	0,002467937	0,006243663
GOLGA2P10	transcribed_unprocesse	ENSG00000255769	0,415529541	0,002468948	0,006245451
FAM120A	protein_coding	ENSG00000048828	-0,164563767	0,002469877	0,006247031
AL358472.4	antisense	ENSG00000282386	0,679330807	0,002470764	0,006248074
MRTFA	protein_coding	ENSG00000196588	0,262297071	0,002470898	0,006248074
ARSE	protein_coding	ENSG00000157399	0,338005086	0,002473556	0,006254024
APC	protein_coding	ENSG00000134982	0,199718801	0,002476317	0,006260231
TPCN2	protein_coding	ENSG00000162341	0,239478824	0,00247894	0,00626609
RPL10	protein_coding	ENSG00000147403	-0,208037573	0,002482901	0,00627533
NDUFA2	protein_coding	ENSG00000131495	-0,238508546	0,002491683	0,006296749
KIAA0232	protein_coding	ENSG00000170871	-0,180275771	0,002492576	0,006298229
AC005899.8	antisense	ENSG00000279762	0,961192212	0,002497384	0,006309602
AL021392.1	antisense	ENSG00000234869	2,144901628	0,002498219	0,006310934
AC015712.2	antisense	ENSG00000259583	-0,488160919	0,002501107	0,006317451
CCAR1	protein_coding	ENSG00000060339	0,167400826	0,002502496	0,006320181
WDR72	protein_coding	ENSG00000166415	0,775750877	0,002502973	0,006320609
MSANTD2	protein_coding	ENSG00000120458	0,283988445	0,002514815	0,006349729
NUFIP1	protein_coding	ENSG00000083635	-0,175796794	0,002521178	0,006365013
TM2D2	protein_coding	ENSG00000169490	0,195773352	0,002532085	0,006390975
RPLP1P6	transcribed_processed_	ENSG00000213433	0,661217405	0,002539028	0,00640771
RAPGEF3	protein_coding	ENSG00000079337	0,371871875	0,002542751	0,006415527
FASTKD2	protein_coding	ENSG00000118246	-0,191073386	0,002542607	0,006415527
BLOC1S1	protein_coding	ENSG00000135441	-0,289949506	0,002543807	0,006417402
RPL29	protein_coding	ENSG00000162244	-0,195153962	0,002546152	0,006422529
ADCK5	protein_coding	ENSG00000173137	0,363991988	0,002549953	0,006431324
TMTC1	protein_coding	ENSG00000133687	1,254800625	0,002554301	0,006441499
IFITM10	protein_coding	ENSG00000244242	2,331224221	0,002556312	0,006445779
NOX5	protein_coding	ENSG00000255346	0,78000547	0,00255854	0,006450602
XIAP	protein_coding	ENSG00000101966	-0,221821383	0,002568476	0,006474858
C17orf53	protein_coding	ENSG00000125319	0,234811441	0,002569273	0,006476072
MTFP1	protein_coding	ENSG00000242114	-0,50738992	0,002574887	0,006489425
LINC02569	antisense	ENSG00000235781	0,815899887	0,002577392	0,006494939

BMP6	protein_coding	ENSG00000153162	-0,267292384	0,002578917	0,006497984
HNRNPA1P16	processed_pseudogene	ENSG00000262333	-0,926747884	0,002580572	0,006501354
SUDS3	protein_coding	ENSG00000111707	-0,153076307	0,002582531	0,006505491
AC026471.1	antisense	ENSG00000260267	-0,435302066	0,002583049	0,006505997
TMEM65	protein_coding	ENSG00000164983	-0,258490971	0,002583773	0,006507022
NUP214	protein_coding	ENSG00000126883	-0,193663812	0,002585687	0,006511043
AC090971.3	unprocessed_pseudoge	ENSG00000259556	1,792132563	0,002588352	0,006516952
GNA12	protein_coding	ENSG00000146535	-0,184748024	0,002590958	0,006522713
MYO1E	protein_coding	ENSG00000157483	0,192465844	0,002595561	0,0065335
AP002495.1	processed_transcript	ENSG00000248671	1,523872515	0,00259912	0,00654013
EID2	protein_coding	ENSG00000176396	-0,22207178	0,002599077	0,00654013
SIM2	protein_coding	ENSG00000159263	-0,394882335	0,002599152	0,00654013
CSNK2B	protein_coding	ENSG00000204435	0,187418035	0,002601723	0,006545798
AC069360.1	lincRNA	ENSG00000250041	1,367625512	0,002610639	0,006567424
CYB5D1	protein_coding	ENSG00000182224	-0,345744606	0,002612872	0,006572234
AC124066.1	antisense	ENSG00000265263	0,710323274	0,002616981	0,006581764
USP25	protein_coding	ENSG00000155313	0,126336838	0,002619143	0,006586392
QTRT1	protein_coding	ENSG00000213339	-0,190134385	0,00261992	0,00658754
DCUN1D5	protein_coding	ENSG00000137692	-0,1806693	0,002625863	0,006601672
G0S2	protein_coding	ENSG00000123689	-0,149545829	0,002629602	0,006610263
ALG11	protein_coding	ENSG00000253710	0,243401574	0,002631173	0,006613402
SF3A3	protein_coding	ENSG00000183431	-0,142530293	0,002635257	0,006622854
MSRB3	protein_coding	ENSG00000174099	0,115994376	0,002641455	0,006637618
ZFYVE19	protein_coding	ENSG00000166140	-0,211253276	0,002645817	0,006647766
ALB	protein_coding	ENSG00000163631	2,500546194	0,002648292	0,006653168
TMEM234	protein_coding	ENSG00000160055	-0,315750873	0,002650334	0,006657484
SH2D3C	protein_coding	ENSG00000095370	1,162604377	0,002653318	0,006664163
PRRC2C	protein_coding	ENSG00000117523	0,232423108	0,002657003	0,006671783
DCXR	protein_coding	ENSG00000169738	-0,205168774	0,002656763	0,006671783
BTG3	protein_coding	ENSG00000154640	-0,207680649	0,002657819	0,006673015
AC009498.1	lincRNA	ENSG00000237271	1,92615694	0,00265853	0,006673984
CARD9	protein_coding	ENSG00000187796	1,071365698	0,002662823	0,006683944
ARL5A	protein_coding	ENSG00000162980	-0,291753586	0,002663376	0,006684514
LINC00629	lincRNA	ENSG00000227060	1,929675311	0,002673656	0,006709493
NAXD	protein_coding	ENSG00000213995	0,164967218	0,002674485	0,006710751
LUC7L	protein_coding	ENSG00000007392	-0,157131786	0,002677365	0,006717157
AC025754.1	antisense	ENSG00000250234	2,481704067	0,002677946	0,006717792
COPRS	protein_coding	ENSG00000172301	-0,224257411	0,002678834	0,006719197
EGFL7	protein_coding	ENSG00000172889	-0,227796879	0,002682261	0,006726971
PSMD10P2	transcribed_processed_	ENSG00000226652	1,925345993	0,002684483	0,00673172

CLTC	protein_coding	ENSG00000141367	0,116161452	0,002690413	0,006745767
UBXN7	protein_coding	ENSG00000163960	0,169733908	0,002693653	0,006753065
DHRS12	protein_coding	ENSG00000102796	0,489423278	0,002696058	0,006758268
NFATC4	protein_coding	ENSG00000100968	0,60649902	0,002698243	0,00676292
TOP3B	protein_coding	ENSG00000100038	0,233195737	0,002700305	0,006767259
RNF212	protein_coding	ENSG00000178222	-0,138523205	0,002700764	0,006767583
AC060766.4	lincRNA	ENSG00000267547	0,484596554	0,002703203	0,006771285
ZG16B	protein_coding	ENSG00000162078	1,209019949	0,002703232	0,006771285
ZDHHC13	protein_coding	ENSG00000177054	0,209494238	0,002702627	0,006771285
VTRNA1-3	misc_RNA	ENSG00000202515	-0,51017922	0,002713254	0,00679556
ST7	protein_coding	ENSG00000004866	0,214724284	0,002716027	0,006801674
LAG3	protein_coding	ENSG00000089692	1,416441597	0,002718126	0,006806101
MLF1	protein_coding	ENSG00000178053	-0,26159766	0,002721799	0,006814465
TAP2	protein_coding	ENSG00000204267	0,180445924	0,002725126	0,006821664
MYO19	protein_coding	ENSG00000278259	-0,159637363	0,002725339	0,006821664
AC087623.2	lincRNA	ENSG00000272092	1,013377614	0,002727545	0,006826351
CCDC191	protein_coding	ENSG00000163617	-0,428318895	0,002728845	0,006828771
MAP3K21	protein_coding	ENSG00000143674	-0,254433132	0,002730151	0,006831205
OR2W6P	unprocessed_pseudoge	ENSG00000168126	1,132548034	0,002732984	0,006837462
TRPC4	protein_coding	ENSG00000133107	1,757546621	0,002734999	0,006841667
IRS2	protein_coding	ENSG00000185950	0,226207267	0,002735876	0,006843028
PIGB	protein_coding	ENSG00000069943	0,199233867	0,002740272	0,006851516
DNAJC7	protein_coding	ENSG00000168259	-0,156007967	0,002740007	0,006851516
NFATC1	protein_coding	ENSG00000131196	-0,331636558	0,002740022	0,006851516
POLR2I	protein_coding	ENSG00000105258	-0,189980444	0,002744183	0,006860457
SIX5	protein_coding	ENSG00000177045	-0,341260603	0,002744535	0,006860502
SLC30A4	protein_coding	ENSG00000104154	0,198191376	0,002747032	0,006865906
AC109992.1	processed_pseudogene	ENSG00000242479	2,615257978	0,002747971	0,006867417
CTNNA1	protein_coding	ENSG00000044115	0,104984326	0,002749828	0,006871219
TMEM183A	protein_coding	ENSG00000163444	-0,212911253	0,002753013	0,006878341
CLDND2	protein_coding	ENSG00000160318	-1,583472878	0,00275511	0,006882741
CACNA1E	protein_coding	ENSG00000198216	2,440916936	0,002756315	0,006884913
RBSN	protein_coding	ENSG00000131381	-0,142816117	0,002756843	0,006885392
CREBZF	protein_coding	ENSG00000137504	-0,279295444	0,002763984	0,006902388
DYNC1I2	protein_coding	ENSG00000077380	-0,149384946	0,002771984	0,006921522
AC087752.3	sense_intronic	ENSG00000253878	0,50074062	0,002773795	0,006925202
CEACAM16	protein_coding	ENSG00000213892	2,471829253	0,002777528	0,006933678
PIAS4	protein_coding	ENSG00000105229	-0,211641575	0,002782627	0,00694556
ARL9	protein_coding	ENSG00000196503	2,493744111	0,002785165	0,00695105
LINC00622	sense_overlapping	ENSG00000260941	1,855630697	0,002791884	0,006966132

SNORD82	snoRNA	ENSG00000202400	-1,463188882	0,002791887	0,006966132
SIN3B	protein_coding	ENSG00000127511	0,194024915	0,002797283	0,006978747
CEP72	protein_coding	ENSG00000112877	-0,19070116	0,002797769	0,006979111
RWDD2A	protein_coding	ENSG00000013392	0,225513813	0,002799853	0,00698346
RAB3IP	protein_coding	ENSG00000127328	0,176335892	0,002806131	0,006998267
C1orf116	protein_coding	ENSG00000182795	1,229367612	0,002810809	0,007009082
HAGHL	protein_coding	ENSG00000103253	-0,332545457	0,002811575	0,007010138
MRPS5	protein_coding	ENSG00000144029	-0,130600667	0,002815138	0,007018169
TBL3	protein_coding	ENSG00000183751	-0,236758148	0,002826644	0,007045998
BAHD1	protein_coding	ENSG00000140320	-0,279408085	0,00283169	0,007057718
VPS53	protein_coding	ENSG00000141252	0,189925453	0,00283296	0,0070588
SLC35G1	protein_coding	ENSG00000176273	-0,222637615	0,002832757	0,0070588
BOC	protein_coding	ENSG00000144857	-0,665595064	0,002833156	0,0070588
KCNU1	protein_coding	ENSG00000215262	1,323586302	0,002834894	0,00706227
COL27A1	protein_coding	ENSG00000196739	0,365754822	0,002837558	0,007068048
LINC01054	antisense	ENSG00000229723	2,319399964	0,002842378	0,007079195
AC078785.1	antisense	ENSG00000240057	1,324702709	0,002851387	0,007100769
SDF2	protein_coding	ENSG00000132581	-0,220549106	0,002852263	0,007102089
NBEAL2	protein_coding	ENSG00000160796	-0,320566738	0,002863744	0,007129813
AL445231.1	antisense	ENSG00000236137	1,400590289	0,002865159	0,007132468
AC009533.1	unprocessed_pseudoge	ENSG00000111788	-0,384998386	0,002878789	0,007165529
PRKAR1B	protein_coding	ENSG00000188191	-0,225741277	0,00288121	0,007170685
PTPRZ1	protein_coding	ENSG00000106278	2,331839393	0,002882818	0,007173816
MRPS31	protein_coding	ENSG00000102738	-0,206663036	0,002883213	0,007173928
MRPL35	protein_coding	ENSG00000132313	-0,164144206	0,002885692	0,007179227
GGT7	protein_coding	ENSG00000131067	0,349706681	0,002886581	0,007180567
AC090589.1	processed_pseudogene	ENSG00000243802	1,407472101	0,002888712	0,007184997
ASPSCR1	protein_coding	ENSG00000169696	-0,189698448	0,002895943	0,007201434
FAM171A2	protein_coding	ENSG00000161682	-0,343848733	0,002896125	0,007201434
PKDCC	protein_coding	ENSG00000162878	-0,721366059	0,002896374	0,007201434
PEX7	protein_coding	ENSG00000112357	-0,267346146	0,002896952	0,007201999
AC087521.2	antisense	ENSG00000246250	1,698845475	0,002899938	0,007208547
PDIA5	protein_coding	ENSG00000065485	-0,175737409	0,002902588	0,007214261
KIAA0100	protein_coding	ENSG00000007202	-0,165749484	0,002907476	0,007225535
TMEM151B	protein_coding	ENSG00000178233	1,74857717	0,002907872	0,007225644
PDE1A	protein_coding	ENSG00000115252	1,821981669	0,002926928	0,007272114
ST3GAL3	protein_coding	ENSG00000126091	0,232712325	0,002930658	0,007280498
ZNF675	protein_coding	ENSG00000197372	-0,257123578	0,002940091	0,007303049
SYNM	protein_coding	ENSG00000182253	-0,273727928	0,002946234	0,00731742
TMEM102	protein_coding	ENSG00000181284	-0,404737567	0,002948009	0,007320943

MON1B	protein_coding	ENSG00000103111	0,170880996	0,002953885	0,007334647
B3GALT5	protein_coding	ENSG00000183778	0,331138826	0,002957435	0,007342572
EP300-AS1	antisense	ENSG00000231993	-0,821838057	0,002961393	0,007351509
AL512306.2	lincRNA	ENSG00000240219	2,229411494	0,002971639	0,007376052
SLC4A1AP	protein_coding	ENSG00000163798	0,150402521	0,002973078	0,007378729
MYO10	protein_coding	ENSG00000145555	0,236348812	0,002974048	0,007380245
FAM201A	antisense	ENSG00000204860	-0,590590468	0,002974409	0,007380247
FAM83A	protein_coding	ENSG00000147689	-0,246184653	0,002976135	0,007383637
ALS2CR12	protein_coding	ENSG00000155749	0,697389798	0,002976659	0,007384044
AP000569.1	lincRNA	ENSG00000273102	0,823633008	0,002982568	0,007397808
ICE1	protein_coding	ENSG00000164151	-0,173542672	0,002983308	0,007398749
LINC02649	lincRNA	ENSG00000215244	1,343742437	0,002983819	0,00739912
MARCH6	protein_coding	ENSG00000145495	0,148420066	0,002991049	0,007416153
SVBP	protein_coding	ENSG00000177868	-0,290777951	0,002994446	0,007423677
TDP2	protein_coding	ENSG00000111802	0,155880133	0,00300895	0,007458733
PARP15	protein_coding	ENSG00000173200	2,245387961	0,003028041	0,00750515
C20orf197	lincRNA	ENSG00000176659	-0,314681804	0,003031305	0,007512332
RNF38	protein_coding	ENSG00000137075	-0,215475442	0,003032674	0,007514819
RORA-AS1	antisense	ENSG00000245534	0,86850966	0,00303828	0,00752689
RBM17	protein_coding	ENSG00000134453	-0,128490539	0,003038196	0,00752689
AC098934.1	transcribed_unprocesse	ENSG00000214796	-0,687079895	0,003040792	0,007532202
AC114488.1	antisense	ENSG00000229167	2,228817583	0,003043916	0,00753903
VASH1-AS1	lincRNA	ENSG00000258301	-0,57092482	0,003053012	0,007560646
SEC31A	protein_coding	ENSG00000138674	0,101477588	0,003053895	0,007561921
PRPF40A	protein_coding	ENSG00000196504	0,160709157	0,003055409	0,007564756
SIX4	protein_coding	ENSG00000100625	0,171189417	0,00305753	0,007569095
AL355581.1	antisense	ENSG00000227678	1,157505101	0,003058415	0,007570371
AP3B2	protein_coding	ENSG00000103723	0,720585112	0,003066973	0,007590639
RGPD8	protein_coding	ENSG00000169629	-0,39231668	0,003073272	0,007605309
ZNF546	protein_coding	ENSG00000187187	0,314468777	0,003075684	0,007610359
AC127024.5	lincRNA	ENSG00000266490	0,829973742	0,003083665	0,007629188
TMEM209	protein_coding	ENSG00000146842	-0,193826828	0,0030865	0,007635282
CCND2	protein_coding	ENSG00000118971	0,691788987	0,003090175	0,007641607
NBEAL1	protein_coding	ENSG00000144426	0,22764762	0,003089506	0,007641607
PBDC1	protein_coding	ENSG00000102390	-0,249632854	0,003089981	0,007641607
PNPLA1	protein_coding	ENSG00000180316	2,205350671	0,003090765	0,007642146
AC106028.3	sense_intronic	ENSG00000260361	1,564733971	0,003092724	0,007646066
KANSL1-AS1	antisense	ENSG00000214401	0,483637578	0,003100898	0,007665351
FDFT1	protein_coding	ENSG00000079459	0,146207851	0,00310683	0,00767909
FSD2	protein_coding	ENSG00000186628	2,529561623	0,00311195	0,007690819

LINC00971	lincRNA	ENSG00000242641	1,714024658	0,003116809	0,007701897
CCDC115	protein_coding	ENSG00000136710	-0,189490904	0,003117439	0,007702527
KCP	protein_coding	ENSG00000135253	0,935289835	0,003117945	0,00770285
IMPG2	protein_coding	ENSG00000081148	0,738341777	0,003119677	0,007706199
FOXJ1	protein_coding	ENSG00000129654	-0,32424915	0,003123515	0,00771475
AL008628.1	sense_overlapping	ENSG00000261003	2,306674587	0,00312676	0,007721837
BRDT	protein_coding	ENSG00000137948	1,853648449	0,003127228	0,007722063
USP8P1	processed_pseudogene	ENSG00000214892	2,544863537	0,003128556	0,007724412
OTUD6B	protein_coding	ENSG00000155100	-0,225429724	0,003132495	0,007733206
GOLGA2P7	transcribed_unprocesse	ENSG00000225151	-0,551596028	0,003134677	0,007737662
POLR3F	protein_coding	ENSG00000132664	0,235582904	0,003146179	0,007765119
MRPL28	protein_coding	ENSG00000086504	-0,203272917	0,003146635	0,007765309
MYRFL	protein_coding	ENSG00000166268	1,689009035	0,003150874	0,007774834
SLC44A4	protein_coding	ENSG00000204385	1,204422812	0,003154102	0,007781862
TRERF1	protein_coding	ENSG00000124496	-0,241048068	0,003160042	0,00779443
TIMM17B	protein_coding	ENSG00000126768	-0,253397812	0,003160335	0,00779443
KAT2A	protein_coding	ENSG00000108773	-0,199361867	0,003161123	0,007795436
AC139749.1	lincRNA	ENSG00000254872	2,176570017	0,003163694	0,007800838
TTC27	protein_coding	ENSG00000018699	-0,145668566	0,003164081	0,007800854
IQANK1	protein_coding	ENSG00000203499	2,418722872	0,003164478	0,007800896
VPS54	protein_coding	ENSG00000143952	-0,159691725	0,00316549	0,007802452
COG2	protein_coding	ENSG00000135775	-0,197726015	0,003170512	0,007813891
LCA5	protein_coding	ENSG00000135338	0,41771202	0,003172039	0,007816714
ALMS1	protein_coding	ENSG00000116127	-0,205786093	0,003172927	0,007817964
AC026271.1	processed_pseudogene	ENSG00000174977	-0,376599547	0,003175749	0,007823977
AC008267.5	lincRNA	ENSG00000237310	0,507648987	0,003176427	0,007824708
TP53RK	protein_coding	ENSG00000172315	0,193250532	0,003190006	0,007857215
EIF1AX	protein_coding	ENSG00000173674	-0,209339155	0,003200969	0,00788327
HKR1	protein_coding	ENSG00000181666	0,258112835	0,003206711	0,007895818
LRIF1	protein_coding	ENSG00000121931	0,170606977	0,003207018	0,007895818
BCKDHB	protein_coding	ENSG00000083123	-0,220837674	0,003207219	0,007895818
LINC00336	lincRNA	ENSG00000197251	2,223105696	0,003213688	0,007910794
C9orf64	protein_coding	ENSG00000165118	-0,251955555	0,003216777	0,007917449
AGBL2	protein_coding	ENSG00000165923	-0,50685736	0,003217491	0,007918256
AL513314.2	lincRNA	ENSG00000276997	1,575393074	0,003219873	0,007923167
PRKD3	protein_coding	ENSG00000115825	-0,168292939	0,003223248	0,00793052
MINDY1	protein_coding	ENSG00000143409	0,314678551	0,003224747	0,007933258
ABHD15	protein_coding	ENSG00000168792	-0,233219073	0,003227905	0,007940073
MRPL45P2	transcribed_unprocesse	ENSG00000228782	0,294769366	0,003229861	0,007943932
WDR64	protein_coding	ENSG00000162843	2,001635056	0,003236787	0,007960011

ZNF740	protein_coding	ENSG00000139651	-0,192776538	0,003248284	0,007987328
TLE4	protein_coding	ENSG00000106829	-0,148497898	0,003251051	0,007993175
STAR	protein_coding	ENSG00000147465	2,256858655	0,003254769	0,008001357
CERS6-AS1	processed_transcript	ENSG00000227617	2,331134563	0,003257469	0,008007033
CDKN1B	protein_coding	ENSG00000111276	-0,149398909	0,003260883	0,008014465
TRIP10	protein_coding	ENSG00000125733	-0,121952511	0,003261372	0,008014707
DNAH10	protein_coding	ENSG00000197653	0,708580087	0,003264739	0,008021058
SYDE2	protein_coding	ENSG00000097096	0,200582619	0,003264566	0,008021058
CALML3-AS1	antisense	ENSG00000205488	2,193855816	0,003268584	0,008029543
PGGHG	protein_coding	ENSG00000142102	-0,347850085	0,003270036	0,008032148
LINC02014	lincRNA	ENSG00000248243	2,211495413	0,003278751	0,008052591
AC110597.1	lincRNA	ENSG00000260578	0,934126732	0,003284368	0,008065419
CRNDE	lincRNA	ENSG00000245694	-0,289350077	0,003287762	0,008072787
PLXNA1	protein_coding	ENSG00000114554	-0,237152805	0,00329292	0,008084486
LTBR	protein_coding	ENSG00000111321	-0,141194872	0,003303075	0,008108447
AC011498.7	TEC	ENSG00000280239	0,749317797	0,003304466	0,00811089
HARBI1	protein_coding	ENSG00000180423	0,314968859	0,003317881	0,008142843
HMGB1P31	processed_pseudogene	ENSG00000233266	1,17978146	0,003324032	0,008156964
ZNF488	protein_coding	ENSG00000265763	-0,412573717	0,003325097	0,008158601
FZD6	protein_coding	ENSG00000164930	-0,169480752	0,00332785	0,00816438
Z99129.4	TEC	ENSG00000279453	-0,420910588	0,003329747	0,008168055
GPR35	protein_coding	ENSG00000178623	1,16576761	0,003335655	0,00818157
NHLRC2	protein_coding	ENSG00000196865	-0,191252764	0,00333781	0,008185876
HACL1	protein_coding	ENSG00000131373	-0,172139014	0,003338783	0,008187283
TSPAN1	protein_coding	ENSG00000117472	0,284539305	0,003340561	0,008190665
LINC01301	lincRNA	ENSG00000251396	0,996393628	0,003345887	0,008201761
GPNMB	protein_coding	ENSG00000136235	0,976911704	0,00334562	0,008201761
FTLP3	processed_pseudogene	ENSG00000226608	0,536306368	0,003348692	0,008207658
AC020604.1	TEC	ENSG00000279254	2,487993385	0,003353474	0,008218396
MARK1	protein_coding	ENSG00000116141	-0,243868184	0,003358502	0,008229734
BCCIP	protein_coding	ENSG00000107949	-0,195673713	0,00335913	0,00823029
CALML4	protein_coding	ENSG00000129007	-1,785656852	0,003360284	0,008232135
EPHX1	protein_coding	ENSG00000143819	0,249033437	0,003369013	0,008252534
GNAT2	protein_coding	ENSG00000134183	1,156389943	0,003377881	0,008273267
AHNAK	protein_coding	ENSG00000124942	-0,376507335	0,003378502	0,0082738
CBX5	protein_coding	ENSG00000094916	0,195531032	0,003381438	0,008280001
DDX12P	unprocessed_pseudoge	ENSG00000214826	-0,428987633	0,00338235	0,008281246
SDHD	protein_coding	ENSG00000204370	-0,231576069	0,003383595	0,008283305
AL353807.3	unprocessed_pseudoge	ENSG00000246203	0,453844459	0,003386426	0,008289247
LINC00235	lincRNA	ENSG00000277142	-0,899931849	0,003393905	0,008306562

AC016949.1	sense_intronic	ENSG00000230732	1,0289368	0,003395134	0,008308578
PKMP3	processed_pseudogene	ENSG00000220563	1,631351214	0,003401951	0,008324268
HAUS3	protein_coding	ENSG00000214367	-0,245859131	0,003403553	0,008327195
ZNF337	protein_coding	ENSG00000130684	-0,202784633	0,003413313	0,008350077
BIRC2	protein_coding	ENSG00000110330	0,174650167	0,003415339	0,008354036
SLC35D2	protein_coding	ENSG00000130958	-0,24035988	0,003423977	0,008374168
CFAP299	protein_coding	ENSG00000197826	0,903529373	0,003426365	0,008379008
SRP54-AS1	transcribed_unitary_pse	ENSG00000258704	0,467282639	0,003427484	0,008380745
AP000873.2	processed_transcript	ENSG00000247137	0,483473506	0,003431341	0,008389176
PTPRC	protein_coding	ENSG00000081237	1,612333756	0,003433392	0,008392229
PRIMPOL	protein_coding	ENSG00000164306	-0,201564687	0,003433408	0,008392229
WASH2P	transcribed_unprocesse	ENSG00000146556	0,54339151	0,003450387	0,008432726
PECAM1	protein_coding	ENSG00000261371	1,975373846	0,003460329	0,008455719
SNX19	protein_coding	ENSG00000120451	0,123890467	0,003460619	0,008455719
ALDH1A1	protein_coding	ENSG00000165092	-0,381212362	0,003461533	0,008456943
FIRRE	processed_transcript	ENSG00000213468	0,271556853	0,003462319	0,008457856
MELTF	protein_coding	ENSG00000163975	-0,216999968	0,003465347	0,008464245
RHOT2	protein_coding	ENSG00000140983	-0,244222555	0,003465915	0,008464625
CSF1R	protein_coding	ENSG00000182578	1,332313735	0,003477103	0,008488731
ZNF770	protein_coding	ENSG00000198146	0,159702294	0,003476961	0,008488731
PSMA6	protein_coding	ENSG00000100902	-0,286598337	0,003477441	0,008488731
CPNE7	protein_coding	ENSG00000178773	-0,299441905	0,003476769	0,008488731
AC116049.2	lincRNA	ENSG00000249413	1,366626218	0,003480763	0,008495829
KRT19	protein_coding	ENSG00000171345	-0,194358946	0,003494072	0,008527299
PYM1	protein_coding	ENSG00000170473	-0,195196661	0,003497793	0,008535364
LINC02261	lincRNA	ENSG00000249699	2,15822236	0,003498665	0,008536097
AL137060.1	sense_intronic	ENSG00000274270	1,141016687	0,003498926	0,008536097
ATG4B	protein_coding	ENSG00000168397	-0,172893561	0,003506277	0,008553014
NPIPP1	transcribed_unprocesse	ENSG00000188599	0,8181477	0,003512644	0,008567527
TMEM43	protein_coding	ENSG00000170876	-0,102888712	0,003514037	0,008569905
ARNT	protein_coding	ENSG00000143437	-0,154392636	0,003535112	0,008620276
ADAM9	protein_coding	ENSG00000168615	-0,156800256	0,003540733	0,008631974
STOML2	protein_coding	ENSG00000165283	-0,209529701	0,003540751	0,008631974
AL513497.1	TEC	ENSG00000279443	0,556559083	0,003544282	0,008639555
ARAP2	protein_coding	ENSG00000047365	0,239967876	0,003550015	0,008652503
TOP2B	protein_coding	ENSG00000077097	-0,18619477	0,003553012	0,008658778
STRA6	protein_coding	ENSG00000137868	2,250230961	0,003555394	0,008663554
AL442128.2	lincRNA	ENSG00000277767	1,092963075	0,003556405	0,008664988
AL355377.2	TEC	ENSG00000279489	1,958788411	0,003561542	0,008676473
CFAP43	protein_coding	ENSG00000197748	0,673024051	0,003566311	0,008687058

SNORA14B	snoRNA	ENSG00000207181	0,647263259	0,003574631	0,008706292
GLIDR	lincRNA	ENSG00000278175	0,508138616	0,003601501	0,008770694
AC073195.1	lincRNA	ENSG00000271855	1,029000698	0,003604638	0,008777292
AC097634.1	lincRNA	ENSG00000270562	2,161147365	0,003610273	0,008789968
KIAA2026	protein_coding	ENSG00000183354	0,196728898	0,003614913	0,008800222
GPR146	protein_coding	ENSG00000164849	0,762202675	0,003619056	0,008809262
TEX19	protein_coding	ENSG00000182459	0,71537167	0,003620354	0,008811375
C16orf58	protein_coding	ENSG00000140688	0,222742399	0,003623525	0,008818046
AC007191.1	TEC	ENSG00000279407	-0,702943956	0,003628944	0,008830187
LIN9	protein_coding	ENSG00000183814	-0,238720694	0,003632267	0,008837223
GRHL1	protein_coding	ENSG00000134317	-0,466318228	0,003633954	0,008840281
ATP6AP1	protein_coding	ENSG00000071553	0,180553749	0,003635517	0,008843033
WDR73	protein_coding	ENSG00000177082	-0,189627195	0,003641867	0,008857427
AP003721.4	antisense	ENSG00000257052	1,571629994	0,003646747	0,008868244
AFF1	protein_coding	ENSG00000172493	0,198531297	0,003652822	0,008881965
GPR176	protein_coding	ENSG00000166073	0,131542037	0,003663254	0,008906275
CCDC66	protein_coding	ENSG00000180376	0,181654758	0,003669154	0,008919563
UBASH3B	protein_coding	ENSG00000154127	-0,120885681	0,003670456	0,00892167
NGEF	protein_coding	ENSG00000066248	0,798398806	0,003678375	0,008939859
GOLPH3	protein_coding	ENSG00000113384	-0,147884995	0,003690705	0,008968763
AC080013.1	antisense	ENSG00000240207	1,014559696	0,003695685	0,008979801
ACTG2	protein_coding	ENSG00000163017	1,402712187	0,003699623	0,008988305
HMGN3	protein_coding	ENSG00000118418	-0,243541483	0,00370012	0,008988448
SPINT1	protein_coding	ENSG00000166145	1,829651055	0,0037096	0,009010408
RBM22	protein_coding	ENSG00000086589	-0,148587443	0,003715071	0,009022629
NUBP2	protein_coding	ENSG00000095906	-0,278469618	0,003720347	0,009034373
KIAA1109	protein_coding	ENSG00000138688	0,249140577	0,003722855	0,009039393
BHLHB9	protein_coding	ENSG00000198908	-0,210236408	0,003724906	0,009043302
CYP2E1	protein_coding	ENSG00000130649	0,750888961	0,003727589	0,009047675
NRXN3	protein_coding	ENSG00000021645	0,182287499	0,00372752	0,009047675
FBXO33	protein_coding	ENSG00000165355	0,167709536	0,003729087	0,00905024
PSMB9	protein_coding	ENSG00000240065	0,256918418	0,00373291	0,009058445
LINC02204	lincRNA	ENSG00000280639	2,175833002	0,003741111	0,009077273
CAVIN1	protein_coding	ENSG00000177469	-0,176986311	0,003743593	0,009082221
THOP1	protein_coding	ENSG00000172009	-0,182150817	0,003745152	0,009084929
SPIN2B	protein_coding	ENSG00000186787	-0,418869734	0,003747811	0,009090304
IFT46	protein_coding	ENSG00000118096	0,229571964	0,003748933	0,009091149
OGA	protein_coding	ENSG00000198408	0,15026264	0,003749046	0,009091149
AC079601.1	antisense	ENSG00000257225	2,133303878	0,003753674	0,009101295
MMS19	protein_coding	ENSG00000155229	-0,134021345	0,003755023	0,009103449

FAM173A	protein_coding	ENSG00000103254	-0,302978227	0,00375545	0,009103449
ZSWIM7	protein_coding	ENSG00000214941	-0,224704779	0,003757978	0,009108501
C2orf42	protein_coding	ENSG00000115998	0,251529082	0,003761504	0,00911597
NBR2	bidirectional_promoter_	ENSG00000198496	0,369928033	0,003763722	0,009120266
DMTN	protein_coding	ENSG00000158856	1,719341567	0,003764855	0,009121936
AC009118.2	antisense	ENSG00000276131	-0,927308728	0,003768538	0,009129781
PRPF19	protein_coding	ENSG00000110107	-0,176601438	0,003772535	0,009138384
CFLAR	protein_coding	ENSG00000003402	-0,121632589	0,003774904	0,009143041
LINC01920	lincRNA	ENSG00000236049	2,118537216	0,003795272	0,009191289
SOCS1	protein_coding	ENSG00000185338	0,677237215	0,003800015	0,009201688
RABGAP1	protein_coding	ENSG00000011454	0,123551977	0,003800897	0,009202738
RABGAP1	protein_coding	ENSG00000011454	0,123551977	0,003800897	0,009202738
ZNF207	protein_coding	ENSG00000010244	-0,128032728	0,003801998	0,009204318
CCDC58	protein_coding	ENSG00000160124	-0,269367436	0,003804545	0,009209396
AC006058.1	lincRNA	ENSG00000261786	0,542460656	0,003811711	0,009225653
CNOT6	protein_coding	ENSG00000113300	-0,182150854	0,003814249	0,009229619
RRP7BP	transcribed_unprocesse	ENSG00000182841	-0,28541979	0,003814163	0,009229619
AL590617.2	antisense	ENSG00000225177	0,809991913	0,003815897	0,009232518
GTF2IRD2B	protein_coding	ENSG00000174428	0,623073696	0,003826941	0,009258146
HSDL1	protein_coding	ENSG00000103160	-0,130550786	0,003833024	0,009271767
NINL	protein_coding	ENSG00000101004	-0,277627924	0,003839778	0,009287009
NDUFB7	protein_coding	ENSG00000099795	-0,249551946	0,003851852	0,009315115
WBP11	protein_coding	ENSG00000084463	-0,132544519	0,003854053	0,009319338
DFFB	protein_coding	ENSG00000169598	-0,28753324	0,003854635	0,009319648
GPER1	protein_coding	ENSG00000164850	1,220313986	0,003858988	0,009329072
AC100810.1	antisense	ENSG00000253982	-0,320431437	0,003862015	0,009333508
SRM	protein_coding	ENSG00000116649	-0,181245654	0,003861764	0,009333508
ING2	protein_coding	ENSG00000168556	-0,205671328	0,003862188	0,009333508
FLYWCH2	protein_coding	ENSG00000162076	-0,234525233	0,003863759	0,009336205
HNF4A	protein_coding	ENSG00000101076	2,130467908	0,003867239	0,009342411
OSCAR	protein_coding	ENSG00000170909	0,709241184	0,003866838	0,009342411
APOBEC3H	protein_coding	ENSG00000100298	1,040683378	0,003870788	0,009349883
HMGN2P3	processed_pseudogene	ENSG00000230330	-0,411180247	0,003882154	0,009376234
ZNF213-AS1	antisense	ENSG00000263072	0,37525739	0,003884386	0,009380519
RBM12B-AS1	TEC	ENSG00000279331	1,668390499	0,003888994	0,009390542
AC026470.1	protein_coding	ENSG00000102921	0,197883377	0,00389152	0,009395536
ARHGEF6	protein_coding	ENSG00000129675	0,688481866	0,003892591	0,009397014
PCBD2	protein_coding	ENSG00000132570	-0,228199446	0,00389734	0,009407373
ZNF571	protein_coding	ENSG00000180479	0,37938987	0,003898175	0,009408281
LPP	protein_coding	ENSG00000145012	0,219958726	0,003902044	0,009416511

DCAF11	protein_coding	ENSG00000100897	-0,148260853	0,003910709	0,009436312
MYO1D	protein_coding	ENSG00000176658	0,094793465	0,00391764	0,009451923
CYP2R1	protein_coding	ENSG00000186104	-0,237730788	0,00391886	0,009453755
CACNA1I	protein_coding	ENSG00000100346	2,122398223	0,00391952	0,009454235
LINC02392	lincRNA	ENSG00000258183	1,179404748	0,003925968	0,009468675
HMGB1P6	processed_pseudogene	ENSG00000259781	-0,296092407	0,003928101	0,009472705
GPC6	protein_coding	ENSG00000183098	2,116464747	0,003929789	0,009475662
SLC22A18AS	protein_coding	ENSG00000254827	1,803349705	0,003945082	0,009511418
GNG11	protein_coding	ENSG00000127920	0,259525246	0,003946548	0,009513834
RFC1	protein_coding	ENSG00000035928	-0,168030019	0,00394911	0,009517772
ZNF526	protein_coding	ENSG00000167625	-0,225597253	0,003948889	0,009517772
UTRN	protein_coding	ENSG00000152818	0,215924923	0,003956437	0,00953431
UBE2Q2L	protein_coding	ENSG00000259511	-1,08197881	0,003958795	0,009538872
KCTD1	protein_coding	ENSG00000134504	0,187699611	0,003960496	0,00954185
BCOR	protein_coding	ENSG00000183337	0,236587449	0,003977003	0,009580495
SLC39A8	protein_coding	ENSG00000138821	-0,165475382	0,003993678	0,009619534
WDFY1	protein_coding	ENSG00000085449	-0,137049306	0,003995449	0,00962267
HINT1	protein_coding	ENSG00000169567	-0,18025945	0,003995924	0,009622684
AC002066.1	antisense	ENSG00000237813	1,040807279	0,003999006	0,009628251
IRF6	protein_coding	ENSG00000117595	0,75886639	0,003999175	0,009628251
UBE2J1	protein_coding	ENSG00000198833	-0,163367754	0,004000748	0,00963091
TTC8	protein_coding	ENSG00000165533	0,227556164	0,004004278	0,009638274
KIAA1549L	protein_coding	ENSG00000110427	-0,198332582	0,004005575	0,009640267
HBP1	protein_coding	ENSG00000105856	0,186347563	0,004007539	0,009643861
CAV2	protein_coding	ENSG00000105971	-0,222027128	0,004018031	0,009667975
OXCT1	protein_coding	ENSG00000083720	-0,179230498	0,004031886	0,009700175
ANKRD34B	protein_coding	ENSG00000189127	2,151491727	0,004049636	0,009741735
P3H2-AS1	antisense	ENSG00000225764	1,3427075	0,004052819	0,009748249
PLBD1-AS1	antisense	ENSG00000256751	0,848769892	0,004055116	0,00975263
AC079880.1	processed_pseudogene	ENSG00000270228	1,387500499	0,004056566	0,009754973
DNAJA3	protein_coding	ENSG00000103423	-0,1611346	0,004060718	0,009763813
C19orf44	protein_coding	ENSG00000105072	-0,234989697	0,004061243	0,009763932
MZT2A	protein_coding	ENSG00000173272	-0,223258575	0,004062927	0,009766836
PPP1R1C	protein_coding	ENSG00000150722	0,34162315	0,004070817	0,009784654
GGACT	protein_coding	ENSG00000134864	0,36840968	0,004072889	0,009788488
DRAM2	protein_coding	ENSG00000156171	0,205934433	0,004073872	0,009789703
CRACR2B	protein_coding	ENSG00000177685	0,872879785	0,004088362	0,009823371
CKB	protein_coding	ENSG00000166165	-0,221456148	0,004091958	0,00983086
ZNF319	protein_coding	ENSG00000166188	0,439265799	0,004099741	0,009848406
RAB7B	protein_coding	ENSG00000276600	1,078638061	0,004105338	0,009860695

AC073316.1	lincRNA	ENSG00000217455	2,107975344	0,004106513	0,009861249
RPL4	protein_coding	ENSG00000174444	-0,125567136	0,00410653	0,009861249
LINC02223	lincRNA	ENSG00000249937	2,168259687	0,004113734	0,009877391
ACOT9	protein_coding	ENSG00000123130	-0,150015749	0,004116249	0,009882274
YARS2	protein_coding	ENSG00000139131	-0,145288493	0,004118319	0,009886087
AC007285.1	antisense	ENSG00000227014	1,064778843	0,004127581	0,00990716
C11orf95	protein_coding	ENSG00000188070	-0,311565465	0,004129064	0,00990956
DECR2	protein_coding	ENSG00000242612	-0,289291453	0,004130649	0,009912205
FOXE1	protein_coding	ENSG00000178919	-0,202277883	0,004131375	0,009912786
COL4A5	protein_coding	ENSG00000188153	0,221210443	0,00413217	0,009913534
MBOAT2	protein_coding	ENSG00000143797	0,129529832	0,00413411	0,009915868
RPL24	protein_coding	ENSG00000114391	-0,239421068	0,004133885	0,009915868
SNORA74A	snoRNA	ENSG00000200959	-0,368906118	0,004140814	0,009930788
PDE6A	protein_coding	ENSG00000132915	0,995780255	0,00414146	0,009931175
ADAM22	protein_coding	ENSG00000008277	-0,26224125	0,00415373	0,009958271
SLC12A9	protein_coding	ENSG00000146828	-0,264511151	0,004153566	0,009958271
AC087645.2	lincRNA	ENSG00000267737	0,917014501	0,004155558	0,009961488
AC110048.2	lincRNA	ENSG00000277152	0,93435282	0,004156151	0,009961745
CBR1	protein_coding	ENSG00000159228	-0,210856345	0,004162169	0,009975005
KDELR2	protein_coding	ENSG00000136240	0,124427586	0,004167904	0,009987582
RHBDL2	protein_coding	ENSG00000158315	0,343055604	0,004182692	0,010021847
AC109927.1	antisense	ENSG00000250195	2,330928001	0,004186396	0,010029551
AL671277.1	unprocessed_pseudoge	ENSG00000227766	1,114099794	0,004187263	0,010030456
CARD8-AS1	antisense	ENSG00000268001	0,437025699	0,00419931	0,01005814
GPR3	protein_coding	ENSG00000181773	-0,393830889	0,004204991	0,010070571
RRS1-AS1	lincRNA	ENSG00000246145	1,662202919	0,004206656	0,01007268
P2RY2	protein_coding	ENSG00000175591	1,540629131	0,004206854	0,01007268
AL049597.2	antisense	ENSG00000261737	0,869216471	0,004222558	0,010109101
CALB2	protein_coding	ENSG00000172137	0,951173343	0,004244866	0,01016132
SENP3	protein_coding	ENSG00000161956	-0,223977133	0,004249961	0,010172329
CBX8	protein_coding	ENSG00000141570	-0,257487596	0,004259179	0,010193205
BCAR1	protein_coding	ENSG00000050820	0,200630086	0,004265813	0,010207889
LNPEP	protein_coding	ENSG00000113441	-0,189071928	0,004268066	0,010212089
LRFN1	protein_coding	ENSG00000128011	-0,484947872	0,004303226	0,010295015
ZNF136	protein_coding	ENSG00000196646	0,194508185	0,004304396	0,010296614
SMIM20	protein_coding	ENSG00000250317	-0,23371689	0,004310898	0,010310964
BPNT1	protein_coding	ENSG00000162813	0,137639549	0,004312947	0,010314661
VSTM1	protein_coding	ENSG00000189068	0,190574041	0,004320552	0,010331646
TNFRSF25	protein_coding	ENSG00000215788	-0,528139487	0,004331576	0,010356799
CHRM3	protein_coding	ENSG00000133019	2,375651191	0,00433845	0,010372025

ANKRD44	protein_coding	ENSG00000065413	-0,238728512	0,004349387	0,01039696
MAPRE1	protein_coding	ENSG00000101367	-0,149930237	0,004353212	0,010404892
AC079336.5	antisense	ENSG00000266718	2,105125346	0,004357878	0,010414832
MYO15A	protein_coding	ENSG00000091536	1,513179779	0,004365091	0,010430853
TMED3	protein_coding	ENSG00000166557	-0,145556223	0,004372852	0,010448181
LINC00942	lincRNA	ENSG00000249628	2,07134745	0,004377475	0,010458011
GFRA3	protein_coding	ENSG00000146013	1,530812849	0,004389113	0,010484592
GOLGA2P5	transcribed_unprocesse	ENSG00000238105	1,499832655	0,004414023	0,010542869
AC090197.1	processed_transcript	ENSG00000253837	2,328065638	0,004414859	0,010543268
PPFIA2	protein_coding	ENSG00000139220	2,062974959	0,004415218	0,010543268
BCL11B	protein_coding	ENSG00000127152	1,155300605	0,004417126	0,010546595
HSD11B1	protein_coding	ENSG00000117594	0,923324168	0,00441951	0,010550318
MYBL1	protein_coding	ENSG00000185697	0,171574518	0,004419714	0,010550318
AL359258.3	TEC	ENSG00000280186	1,932216743	0,00443031	0,010574382
AC138904.1	lincRNA	ENSG00000246465	-0,58460627	0,00443915	0,010594248
LINC00920	lincRNA	ENSG00000246898	0,406202399	0,004445026	0,010607037
GRIN2A	protein_coding	ENSG00000183454	1,95637247	0,004445581	0,010607127
NHLRC3	protein_coding	ENSG00000188811	-0,235501548	0,00444754	0,010610566
TENM4	protein_coding	ENSG00000149256	1,509755345	0,004449544	0,010614114
VPS37D	protein_coding	ENSG00000176428	0,400935562	0,004463988	0,010647331
MEFV	protein_coding	ENSG00000103313	1,918200276	0,004479214	0,010682405
NPHS1	protein_coding	ENSG00000161270	2,182902896	0,004481543	0,010686717
CERK	protein_coding	ENSG00000100422	-0,121234607	0,004483464	0,010690055
C3orf38	protein_coding	ENSG00000179021	0,16270025	0,004489087	0,010700973
SPATA5	protein_coding	ENSG00000145375	-0,235992582	0,004489043	0,010700973
CBR3	protein_coding	ENSG00000159231	-0,206054626	0,004494189	0,01071189
AC061975.3	unprocessed_pseudoge	ENSG00000264029	0,769793399	0,004497217	0,010717862
CFAP74	protein_coding	ENSG00000142609	2,08904938	0,004502728	0,010729749
B4GALT1	protein_coding	ENSG00000086062	0,21459222	0,004503412	0,010730131
AL109809.1	transcribed_unitary_pse	ENSG00000232528	2,06266138	0,004512734	0,010751096
AL358472.2	lincRNA	ENSG00000272654	-1,014141485	0,004516952	0,010759168
NAA38	protein_coding	ENSG00000183011	-0,240229401	0,004517172	0,010759168
NT5C3A	protein_coding	ENSG00000122643	-0,209832412	0,004517981	0,010759847
SLTM	protein_coding	ENSG00000137776	-0,187322106	0,004530072	0,010787388
FAM162A	protein_coding	ENSG00000114023	-0,207798703	0,004538165	0,010805405
CHD9	protein_coding	ENSG00000177200	0,177567505	0,004538868	0,010805826
RAB36	protein_coding	ENSG00000100228	-0,494838422	0,004547183	0,010824364
FAF1	protein_coding	ENSG00000185104	-0,155040862	0,004558549	0,010850162
AC104046.1	transcribed_processed_	ENSG00000259630	0,841781611	0,004560473	0,010853482
AC026740.1	antisense	ENSG00000271781	0,676976794	0,004563163	0,010858622

LSM10	protein_coding	ENSG00000181817	-0,242594642	0,004573949	0,010883028
ABCA13	protein_coding	ENSG00000179869	0,97585775	0,004577741	0,010890786
GZF1	protein_coding	ENSG00000125812	0,186577952	0,004578948	0,010892395
CTH	protein_coding	ENSG00000116761	0,414731051	0,004584421	0,010903755
MPZL1	protein_coding	ENSG00000197965	-0,105669565	0,004584787	0,010903755
FRMD6-AS2	lincRNA	ENSG00000258537	1,187869515	0,004586265	0,010906005
SNRPGP15	processed_pseudogene	ENSG00000224543	-0,58750405	0,004605631	0,010950788
KMT5B	protein_coding	ENSG00000110066	-0,155895482	0,004610643	0,010961433
AC093673.1	antisense	ENSG00000232533	0,331384896	0,004620865	0,010984462
SLC4A8	protein_coding	ENSG00000050438	-0,210696806	0,004630944	0,011007146
CAMLG	protein_coding	ENSG00000164615	-0,193343659	0,004639994	0,011027378
ZFAND1	protein_coding	ENSG00000104231	0,202563671	0,004644313	0,011036363
NPHP3-AS1	antisense	ENSG00000248724	1,777989915	0,004645148	0,01103707
DMWD	protein_coding	ENSG00000185800	0,234701083	0,004652002	0,011052074
FAM216A	protein_coding	ENSG00000204856	-0,167313624	0,004657479	0,011063806
AL442663.3	antisense	ENSG00000258813	0,78644313	0,004664551	0,011078319
TBK1	protein_coding	ENSG00000183735	-0,170983585	0,004664669	0,011078319
HES4	protein_coding	ENSG00000188290	0,340799503	0,004667695	0,011084223
RORC	protein_coding	ENSG00000143365	2,052447974	0,004678069	0,01110757
LINC01594	antisense	ENSG00000225328	1,932834081	0,004680014	0,011110904
FAM192A	protein_coding	ENSG00000172775	-0,139668729	0,004683111	0,011116969
SUPT3H	protein_coding	ENSG00000196284	0,215599705	0,004693712	0,011140844
OTOGL	protein_coding	ENSG00000165899	0,885509781	0,004706781	0,011170571
PAK1	protein_coding	ENSG00000149269	0,420985252	0,004713484	0,011185184
SPATA2L	protein_coding	ENSG00000158792	-0,262961142	0,004733157	0,011230571
RABGGTB	protein_coding	ENSG00000137955	-0,187271971	0,004752322	0,011274739
CYB5B	protein_coding	ENSG00000103018	-0,153094808	0,00475763	0,011286026
TEPSIN	protein_coding	ENSG00000167302	0,345312275	0,004760279	0,011291006
TXLNA	protein_coding	ENSG00000084652	-0,15657562	0,004761396	0,011292349
CLDN4	protein_coding	ENSG00000189143	1,156880654	0,004778405	0,011331378
AL357055.3	lincRNA	ENSG00000238198	1,466054161	0,004794864	0,011369093
C8orf31	processed_transcript	ENSG00000177335	0,90054796	0,004802887	0,0113868
THUMPD1	protein_coding	ENSG00000066654	-0,179192408	0,004810972	0,011404651
TCP1	protein_coding	ENSG00000120438	-0,138880832	0,004814786	0,011412372
RTL8C	protein_coding	ENSG00000134590	-0,202076705	0,004821429	0,011425477
HIST2H3C	protein_coding	ENSG00000203811	-1,972832845	0,004821213	0,011425477
NOL12	protein_coding	ENSG00000273899	-0,507018039	0,004826461	0,011436082
MFAP2	protein_coding	ENSG00000117122	-0,176135567	0,00482941	0,011441747
NRG2	protein_coding	ENSG00000158458	1,618463065	0,004830945	0,01144406
GRSF1	protein_coding	ENSG00000132463	-0,118866005	0,004832318	0,011445991

PALD1	protein_coding	ENSG00000107719	2,029145533	0,00483976	0,011462295
SNPH	protein_coding	ENSG00000101298	0,223240653	0,004850326	0,011484668
RGS10	protein_coding	ENSG00000148908	-0,223232966	0,004850287	0,011484668
KIF5C	protein_coding	ENSG00000168280	-0,25125228	0,004852037	0,011487393
ESS2	protein_coding	ENSG00000100056	-0,174038208	0,004857705	0,011499483
ACTG1P1	processed_pseudogene	ENSG00000178631	0,844868492	0,004858762	0,011500659
KARS	protein_coding	ENSG00000065427	-0,128943589	0,004864996	0,011514087
AL132639.3	antisense	ENSG00000259083	1,123419606	0,004870251	0,011525194
GABPA	protein_coding	ENSG00000154727	-0,207669086	0,004873852	0,011532385
KIF7	protein_coding	ENSG00000166813	-0,264982287	0,004892124	0,011572949
ZNF599	protein_coding	ENSG00000153896	-0,335448778	0,004891946	0,011572949
ANP32E	protein_coding	ENSG00000143401	-0,20329066	0,004902962	0,01159725
AC063944.1	lincRNA	ENSG00000239828	1,965165773	0,004912706	0,011618957
CASK	protein_coding	ENSG00000147044	-0,127691055	0,004913883	0,011620402
NRBP1	protein_coding	ENSG00000115216	-0,134754573	0,00491841	0,011629765
FBXL22	protein_coding	ENSG00000197361	-0,891179091	0,004926613	0,01164782
ACOT7	protein_coding	ENSG00000097021	0,195197685	0,004934226	0,011664475
RPL21	protein_coding	ENSG00000122026	-0,19113657	0,004939924	0,011676599
AL355312.1	processed_pseudogene	ENSG00000216906	1,985181215	0,004947854	0,011693994
SOX4	protein_coding	ENSG00000124766	-0,389355029	0,004948948	0,011695233
IGSF5	protein_coding	ENSG00000183067	1,029910765	0,004955287	0,011708864
LINC00884	antisense	ENSG00000233058	0,900856783	0,004956305	0,01170992
LRP6	protein_coding	ENSG00000070018	0,169435429	0,00496102	0,01171971
AL117336.2	antisense	ENSG00000271335	0,607935023	0,004968913	0,011737003
ZNF783	protein_coding	ENSG00000204946	-0,304862593	0,004985569	0,01177499
SBK2	protein_coding	ENSG00000187550	-0,505750784	0,004986752	0,011776429
RPL9P9	transcribed_processed_	ENSG00000237550	-0,258519986	0,004993986	0,011792155
TBC1D20	protein_coding	ENSG00000125875	-0,148634035	0,004996301	0,011796263
TMEM141	protein_coding	ENSG00000244187	-0,3604534	0,005009825	0,011826831
SNORD117	snoRNA	ENSG00000201785	-1,380089882	0,005015769	0,011839501
EBLN2	protein_coding	ENSG00000255423	-0,574571965	0,005025385	0,011860835
ZNF131	protein_coding	ENSG00000172262	0,160290394	0,005029354	0,011868834
BTBD10	protein_coding	ENSG00000148925	0,155790669	0,005029975	0,011868936
CNEP1R1	protein_coding	ENSG00000205423	-0,248104656	0,005034983	0,011879386
RBM38	protein_coding	ENSG00000132819	0,170488658	0,005037543	0,011884059
AC007566.1	antisense	ENSG00000244055	1,028248448	0,005052606	0,011918223
VWA7	protein_coding	ENSG00000204396	1,004143799	0,005056624	0,011926329
ARHGAP5-AS1	antisense	ENSG00000258655	0,437942574	0,005063167	0,011939966
MRPL12	protein_coding	ENSG00000262814	-0,297214813	0,00506357	0,011939966
SGO1-AS1	processed_transcript	ENSG00000231304	1,367370867	0,005065407	0,011942924

IDI2-AS1	antisense	ENSG00000232656	1,082104588	0,005072975	0,011959393
B9D1	protein_coding	ENSG00000108641	-0,214854583	0,005075962	0,011963685
CDHR3	protein_coding	ENSG00000128536	-0,370452454	0,005075433	0,011963685
NSMCE1	protein_coding	ENSG00000169189	-0,184936509	0,005082837	0,011977624
WDSUB1	protein_coding	ENSG00000196151	-0,215663195	0,005083044	0,011977624
MST1L	transcribed_unprocesse	ENSG00000186715	0,860067059	0,005090522	0,011993867
RNF208	protein_coding	ENSG00000212864	0,922802625	0,005092629	0,011997452
DNAJB4	protein_coding	ENSG00000162616	-0,190353446	0,005103934	0,012022703
MTMR9LP	transcribed_unprocesse	ENSG00000220785	1,312428642	0,005107074	0,012028718
AC034236.2	antisense	ENSG00000271918	-0,706153991	0,005108986	0,01203184
SLC17A5	protein_coding	ENSG00000119899	0,223298779	0,005110799	0,012034729
SNORA79B	snoRNA	ENSG00000222489	-0,468377566	0,005113098	0,01203876
CCNI	protein_coding	ENSG00000118816	-0,111282829	0,00511565	0,012043387
IL37	protein_coding	ENSG00000125571	1,214950861	0,005118833	0,012049496
SLC25A11	protein_coding	ENSG00000108528	-0,184176477	0,005124499	0,012061449
PYCR2	protein_coding	ENSG00000143811	-0,164990993	0,005126371	0,01206447
AC020928.1	antisense	ENSG00000267254	0,626752457	0,005128826	0,012068863
UGGT2	protein_coding	ENSG00000102595	0,187025904	0,005132582	0,012076318
AL139289.1	antisense	ENSG00000229431	2,026464427	0,005137389	0,012086205
ACRBP	protein_coding	ENSG00000111644	1,12911611	0,005137963	0,012086205
PEX10	protein_coding	ENSG00000157911	-0,21514439	0,005142253	0,01209491
B3GAT3	protein_coding	ENSG00000149541	-0,240520285	0,005143213	0,012095365
AC096887.2	TEC	ENSG00000280417	-0,610598552	0,005143626	0,012095365
ATG4C	protein_coding	ENSG00000125703	-0,215416187	0,005157531	0,012126671
MRC1	protein_coding	ENSG00000260314	1,449472533	0,00516398	0,012140444
TAOK2	protein_coding	ENSG00000149930	-0,193408242	0,005168742	0,012150246
CDK5RAP1	protein_coding	ENSG00000101391	-0,144904732	0,005169485	0,0121506
STK32C	protein_coding	ENSG00000165752	-0,228845894	0,005180111	0,012174181
RPS18P12	processed_pseudogene	ENSG00000230897	-0,564062696	0,00518713	0,012189279
AL603832.1	antisense	ENSG00000225075	1,702453589	0,005192284	0,012199395
AC079753.1	TEC	ENSG00000280228	1,489805999	0,005192624	0,012199395
AF131215.7	lincRNA	ENSG00000270076	2,277003404	0,005201139	0,012216602
LEAP2	protein_coding	ENSG00000164406	1,044271685	0,00520067	0,012216602
GBE1	protein_coding	ENSG00000114480	0,094400604	0,005203286	0,012220245
GPBP1L1	protein_coding	ENSG00000159592	-0,095000741	0,005225355	0,012270671
SEL1L3	protein_coding	ENSG00000091490	0,470703046	0,005233842	0,012289193
NDUFS5	protein_coding	ENSG00000168653	-0,156057241	0,005237263	0,012295818
SREBF2-AS1	antisense	ENSG00000184068	0,784780698	0,005246269	0,012315506
MAPK8IP3	protein_coding	ENSG00000138834	0,300388183	0,00524745	0,012315506
SLC4A4	protein_coding	ENSG00000080493	-0,231387385	0,005247036	0,012315506

ZNF853	protein_coding	ENSG00000236609	1,712189854	0,005252652	0,012325877
SOX18	protein_coding	ENSG00000203883	-0,512009927	0,005253071	0,012325877
AC135048.4	TEC	ENSG00000279196	1,578132402	0,005254563	0,012327967
AC012213.1	antisense	ENSG00000253477	1,043270927	0,005262439	0,012342638
AL450124.1	unprocessed_pseudoge	ENSG00000204556	1,071058915	0,005261433	0,012342638
AP003486.1	lincRNA	ENSG00000255455	0,448006928	0,005266363	0,01235
AC024075.1	sense_intronic	ENSG00000267904	-0,577301408	0,005276666	0,012372748
CSNK1A1	protein_coding	ENSG00000113712	0,096666098	0,00528733	0,012396336
AC234775.3	sense_overlapping	ENSG00000261101	0,992019546	0,005294203	0,01241103
AP001062.1	antisense	ENSG00000184441	0,687694833	0,005296787	0,01241425
ARHGEF16	protein_coding	ENSG00000130762	0,374231074	0,005296707	0,01241425
VPS25	protein_coding	ENSG00000131475	-0,158959505	0,005306896	0,012436522
AC008040.1	antisense	ENSG00000239219	0,796081437	0,005308814	0,012439596
ZNF114	protein_coding	ENSG00000178150	-0,195234391	0,005310192	0,012441403
EIF4ENIF1	protein_coding	ENSG00000184708	-0,158072464	0,005319988	0,01246293
AC079880.2	processed_pseudogene	ENSG00000270292	0,888084028	0,005321533	0,012463703
GIT2	protein_coding	ENSG00000139436	0,11654251	0,005321326	0,012463703
TBCB	protein_coding	ENSG00000105254	-0,170320648	0,005338567	0,01250217
CASC15	lincRNA	ENSG00000272168	1,773964366	0,005351936	0,012532048
LINC01962	lincRNA	ENSG00000248473	-1,198576332	0,005353132	0,012533418
NPR1	protein_coding	ENSG00000169418	1,976411696	0,005355297	0,012535734
GAB1	protein_coding	ENSG00000109458	-0,267572571	0,005355344	0,012535734
DNM3	protein_coding	ENSG00000197959	0,476869264	0,005365178	0,012557321
ZDHHC6	protein_coding	ENSG00000023041	-0,156524861	0,005369086	0,012565034
LRMDA	protein_coding	ENSG00000148655	0,587712214	0,005370886	0,012567813
PGAM1P7	processed_pseudogene	ENSG00000213997	0,820755477	0,00537436	0,012573937
ZSCAN20	protein_coding	ENSG00000121903	0,251377605	0,00537473	0,012573937
CDR2L	protein_coding	ENSG00000109089	-0,298756247	0,005379684	0,012584093
GPR143	protein_coding	ENSG00000101850	1,424782818	0,00538296	0,01259032
RPS6KA1	protein_coding	ENSG00000117676	0,15387784	0,005386521	0,012597211
FGF7P6	transcribed_unprocesse	ENSG00000227449	1,347568767	0,005397737	0,012622004
AC026369.2	antisense	ENSG00000256694	2,026862322	0,005417892	0,012666246
PHOSPHO2	protein_coding	ENSG00000144362	-0,439175099	0,005417628	0,012666246
TCP11L1	protein_coding	ENSG00000176148	0,126132192	0,005420316	0,012670468
AC132872.3	antisense	ENSG00000275888	1,084470944	0,005423857	0,012677299
ENHO	protein_coding	ENSG00000168913	-0,773144565	0,005428631	0,012687013
AC074043.1	processed_pseudogene	ENSG00000239455	2,002709973	0,005433795	0,012697634
RBMXL1	protein_coding	ENSG00000213516	-0,150043334	0,005440672	0,012712256
MIR4697HG	TEC	ENSG00000280237	-0,674812917	0,005444503	0,012719757
PDE8B	protein_coding	ENSG00000113231	1,947568238	0,00544942	0,012729796

CORO2B	protein_coding	ENSG00000103647	0,786923578	0,005450189	0,012730143
AC016773.1	lincRNA	ENSG00000270195	-1,129770159	0,005453721	0,012736941
MRPL44	protein_coding	ENSG00000135900	-0,192762621	0,00546321	0,01275765
IFNAR1	protein_coding	ENSG00000142166	0,132297676	0,005475877	0,012785775
RNF20	protein_coding	ENSG00000155827	-0,16018733	0,005479688	0,012793217
ANXA4	protein_coding	ENSG00000196975	0,16984579	0,005489728	0,012815196
AC104109.2	lincRNA	ENSG00000270177	0,644764422	0,005497359	0,012831549
MCCC1	protein_coding	ENSG00000078070	0,164950758	0,005503842	0,01284522
RADX	protein_coding	ENSG00000147231	0,184340081	0,005504497	0,012845287
AP001148.1	TEC	ENSG00000278989	1,291019019	0,005510438	0,012857689
LUC7L3	protein_coding	ENSG00000108848	-0,233785819	0,005513915	0,012864226
DPM3	protein_coding	ENSG00000179085	-0,2915352	0,005514494	0,012864226
NBPF15	protein_coding	ENSG00000266338	-0,172700726	0,005515267	0,012864565
MORN3	protein_coding	ENSG00000139714	0,8948219	0,005521111	0,012876733
VEGFB	protein_coding	ENSG00000173511	-0,152772046	0,005523053	0,012878793
ZNF768	protein_coding	ENSG00000169957	-0,205543016	0,00552325	0,012878793
KIAA0895	protein_coding	ENSG00000164542	-0,20284813	0,005530191	0,01289351
RAB38	protein_coding	ENSG00000123892	-0,316584183	0,005532311	0,012896988
TIMM17A	protein_coding	ENSG00000134375	0,172398862	0,00553952	0,012912326
IFNGR1	protein_coding	ENSG00000027697	0,196925126	0,005545291	0,012924309
OGT	protein_coding	ENSG00000147162	-0,154701466	0,005568194	0,01297556
SH3PXD2A	protein_coding	ENSG00000107957	-0,259782263	0,005568546	0,01297556
HSD17B14	protein_coding	ENSG00000087076	0,385074142	0,005569895	0,01297723
ILK	protein_coding	ENSG00000166333	0,146132621	0,005579082	0,012997158
GCN1	protein_coding	ENSG00000089154	-0,207037659	0,005581802	0,013002016
BCL6B	protein_coding	ENSG00000161940	1,112711269	0,00558734	0,013013438
TMEM205	protein_coding	ENSG00000105518	0,238666846	0,005598497	0,013037944
PTPRE	protein_coding	ENSG00000132334	0,442236033	0,005604296	0,013049968
ABCB1	protein_coding	ENSG00000085563	0,350612616	0,00561295	0,013068635
CBLN3	protein_coding	ENSG00000139899	0,505033752	0,005617872	0,013077165
DDX21	protein_coding	ENSG00000165732	-0,147944043	0,005617889	0,013077165
AL359921.2	antisense	ENSG00000273058	0,832049925	0,005631017	0,013106236
ACSBG1	protein_coding	ENSG00000103740	2,117297217	0,005634823	0,013113607
AL137077.2	lincRNA	ENSG00000283078	-0,914438725	0,005635937	0,013114711
CCDC198	protein_coding	ENSG00000100557	0,380054876	0,0056396	0,013121746
PPT2	protein_coding	ENSG00000221988	-0,27044408	0,005641966	0,013124274
AC002059.1	sense_intronic	ENSG00000273216	1,60902529	0,005641542	0,013124274
MTMR3	protein_coding	ENSG00000100330	0,205445999	0,005645108	0,013130094
NXPH3	protein_coding	ENSG00000182575	1,945407651	0,005648795	0,013137179
SNCB	protein_coding	ENSG00000074317	-0,458422241	0,005660835	0,013163687

ENKUR	protein_coding	ENSG00000151023	1,084239339	0,005662606	0,013166312
AC007666.1	antisense	ENSG00000236754	1,278002097	0,005673254	0,013189575
GSDMB	protein_coding	ENSG00000073605	0,458696448	0,005682002	0,013208418
CLTB	protein_coding	ENSG00000175416	0,172297794	0,005684057	0,013211697
SSBP1	protein_coding	ENSG00000106028	-0,182629324	0,005691106	0,013226582
RHOQP1	processed_pseudogene	ENSG00000258568	-0,755829371	0,005702516	0,013250097
EIF1B-AS1	processed_transcript	ENSG00000280739	0,561911903	0,005702115	0,013250097
DMKN	protein_coding	ENSG00000161249	0,21978538	0,005709606	0,013265067
HSPA4	protein_coding	ENSG00000170606	0,106000582	0,005717468	0,01328183
LAMA4	protein_coding	ENSG00000112769	1,390072946	0,005719261	0,013284489
PLD5	protein_coding	ENSG00000180287	1,859115915	0,005725722	0,01329799
MS4A4A	protein_coding	ENSG00000110079	1,137572367	0,005729161	0,013304472
LINC00641	processed_transcript	ENSG00000258441	0,269911688	0,005730877	0,01330695
FASTKD5	protein_coding	ENSG00000215251	-0,183825144	0,005734962	0,013314927
ABI3BP	protein_coding	ENSG00000154175	0,514998529	0,00573684	0,013316273
SDHAF2	protein_coding	ENSG00000167985	-0,261792223	0,005736572	0,013316273
RILP	protein_coding	ENSG00000167705	-0,317046737	0,005743782	0,013330879
KIAA0355	protein_coding	ENSG00000166398	0,205605978	0,00574518	0,013332614
VTRNA1-1	misc_RNA	ENSG00000199990	-0,943800535	0,005747952	0,013336029
MRPL55	protein_coding	ENSG00000162910	-0,190932059	0,00574782	0,013336029
PMM1	protein_coding	ENSG00000100417	-0,191848801	0,005767294	0,013379392
PARD6G	protein_coding	ENSG00000178184	-0,245794051	0,005775407	0,013396697
OAT	protein_coding	ENSG00000065154	-0,155923176	0,005776101	0,013396792
ISYNA1	protein_coding	ENSG00000105655	-0,17926873	0,00578621	0,013418722
STAG3L5P	transcribed_unprocessed	ENSG00000242294	-0,461959704	0,005793006	0,013432963
AC006333.2	antisense	ENSG00000272686	-0,432418894	0,005798801	0,013444881
STUB1	protein_coding	ENSG00000103266	-0,168578446	0,00580623	0,013460583
FUZ	protein_coding	ENSG00000010361	-0,240318326	0,005809814	0,013467369
TOP1MT	protein_coding	ENSG00000184428	-0,169251504	0,005820015	0,013489491
IGDCC4	protein_coding	ENSG00000103742	0,975233941	0,005827295	0,013504838
AMZ2P1	transcribed_unprocessed	ENSG00000214174	0,232508289	0,005831906	0,013513998
RPL15	protein_coding	ENSG00000174748	-0,180011064	0,005833945	0,013517194
FAM129A	protein_coding	ENSG00000135842	0,135190024	0,005835998	0,013520424
ADGRG5	protein_coding	ENSG00000159618	0,460008505	0,005838305	0,013524242
AC026464.6	protein_coding	ENSG00000272617	-0,427282476	0,00584243	0,013532268
TOMM40L	protein_coding	ENSG00000158882	-0,175519391	0,005864655	0,013582212
GTF2IRD2	protein_coding	ENSG00000196275	1,027521219	0,005878687	0,013613172
TMEM87A	protein_coding	ENSG00000103978	-0,151459393	0,005880578	0,013616015
BRCA2	protein_coding	ENSG00000139618	-0,202162805	0,005889689	0,01363557
LINC01277	lincRNA	ENSG00000229017	1,79357577	0,005896323	0,013649389

PSMC6	protein_coding	ENSG00000100519	-0,184140477	0,005897725	0,013651094
TOE1	protein_coding	ENSG00000132773	-0,173656141	0,005911269	0,0136809
OXSR1	protein_coding	ENSG00000172939	-0,122210859	0,005928044	0,013718174
SOX15	protein_coding	ENSG00000129194	-0,407640172	0,005931367	0,013724316
USP46-AS1	lincRNA	ENSG00000248866	-0,504962972	0,005934607	0,013726039
SPTBN2	protein_coding	ENSG00000173898	1,144522186	0,005932958	0,013726039
EIF5A2	protein_coding	ENSG00000163577	0,200069984	0,005934789	0,013726039
SMPD2	protein_coding	ENSG00000135587	-0,268215171	0,005933566	0,013726039
KLRC1	protein_coding	ENSG00000134545	1,269430076	0,005945819	0,013749396
TPPP	protein_coding	ENSG00000171368	-0,515226347	0,005946228	0,013749396
AC007546.1	sense_intronic	ENSG00000277595	0,899199045	0,005950019	0,013756611
NCBP1	protein_coding	ENSG00000136937	-0,129401912	0,005958923	0,013775643
RPL13	protein_coding	ENSG00000167526	-0,192534898	0,005962342	0,013781994
QKI	protein_coding	ENSG00000112531	-0,172799318	0,005967922	0,013793338
ANKDD1A	protein_coding	ENSG00000166839	0,643757935	0,005970541	0,013797836
AC064836.3	lincRNA	ENSG00000273456	-0,754955897	0,005972156	0,013798458
PIGG	protein_coding	ENSG00000174227	0,141112781	0,005971798	0,013798458
LINC00536	lincRNA	ENSG00000249917	1,245896074	0,005975989	0,01380576
HDAC1	protein_coding	ENSG00000116478	-0,136162908	0,005980707	0,013815104
INTS12	protein_coding	ENSG00000138785	0,190609795	0,005986787	0,01382759
SLC38A7	protein_coding	ENSG00000103042	-0,242145514	0,005987536	0,013827764
AC025180.1	antisense	ENSG00000249899	1,181875077	0,0060094	0,013876694
RN7SKP97	misc_RNA	ENSG00000222898	1,332538784	0,006017714	0,013894328
BRPF1	protein_coding	ENSG00000156983	0,194367127	0,006029466	0,013919895
SMARCC2	protein_coding	ENSG00000139613	-0,144666048	0,006035719	0,013932763
NONOP2	processed_pseudogene	ENSG00000237522	1,233486189	0,00607395	0,014019436
NPLOC4	protein_coding	ENSG00000182446	0,139706924	0,006083549	0,014040012
ANKRD45	protein_coding	ENSG00000183831	1,559283882	0,00609081	0,014055187
AC114811.2	antisense	ENSG00000260641	0,744357983	0,00611894	0,014118514
PPP1CB	protein_coding	ENSG00000213639	0,204342914	0,006124142	0,014128925
ADRM1	protein_coding	ENSG00000130706	0,165838895	0,00613091	0,014142553
MAPK8	protein_coding	ENSG00000107643	-0,140770926	0,006131428	0,014142553
AL645941.3	antisense	ENSG00000263756	1,872958952	0,006136609	0,014152914
NEMP1	protein_coding	ENSG00000166881	-0,137578535	0,00614532	0,014169817
DACT1	protein_coding	ENSG00000165617	-0,398369293	0,006145301	0,014169817
CCDC200	protein_coding	ENSG00000236383	1,905025706	0,006166745	0,01421762
CIDEC	protein_coding	ENSG00000187288	1,899931333	0,006173662	0,014230366
NT5DC4	protein_coding	ENSG00000144130	1,274376973	0,006173365	0,014230366
S100A1	protein_coding	ENSG00000160678	0,886709674	0,006182839	0,014249918
RFESD	protein_coding	ENSG00000175449	-0,329903446	0,00618402	0,01425104

AL136295.5	protein_coding	ENSG00000259529	1,072576646	0,006185288	0,01425236
TMED10	protein_coding	ENSG00000170348	0,11148212	0,006188034	0,014257087
FCHSD2	protein_coding	ENSG00000137478	0,150954087	0,006195247	0,014272102
PRC1-AS1	antisense	ENSG00000258725	1,286094476	0,006199049	0,014279256
UBE2B	protein_coding	ENSG00000119048	0,245578622	0,006209627	0,014302015
DNAJC5	protein_coding	ENSG00000101152	0,178202266	0,006216759	0,014316835
SERPINA9	protein_coding	ENSG00000170054	0,847398407	0,006221058	0,014325126
RAB30-AS1	lincRNA	ENSG00000246067	-0,263042133	0,006229363	0,01434103
TNFSF18	protein_coding	ENSG00000120337	0,889248307	0,006228831	0,01434103
RNF182	protein_coding	ENSG00000180537	-0,133478163	0,006232517	0,014346681
AL731533.2	lincRNA	ENSG00000273001	1,533537466	0,006247999	0,014380704
ESCO2	protein_coding	ENSG00000171320	-0,203895512	0,006252981	0,014390557
MFSD4A	protein_coding	ENSG00000174514	1,075965737	0,006260699	0,014406703
FBP1	protein_coding	ENSG00000165140	0,944806975	0,006277483	0,014443705
ADAL	protein_coding	ENSG00000168803	0,168767473	0,006280915	0,01444998
SMU1	protein_coding	ENSG00000122692	-0,117844171	0,006285262	0,014456736
HEATR6	protein_coding	ENSG00000068097	-0,127532563	0,006286872	0,014458819
GLRX5	protein_coding	ENSG00000182512	-0,148310519	0,00629469	0,014475176
PRDM16	protein_coding	ENSG00000142611	-0,446336011	0,006301333	0,014488828
RPS3	protein_coding	ENSG00000149273	-0,1966597	0,006302331	0,014489497
STX17-AS1	antisense	ENSG00000255145	1,379376218	0,006309463	0,014504269
NOMO1	protein_coding	ENSG00000103512	0,17281759	0,006311159	0,014506542
CPD	protein_coding	ENSG00000108582	-0,15392478	0,006321027	0,014527595
JSRP1	protein_coding	ENSG00000167476	0,906178966	0,006332949	0,014553365
TRPC6	protein_coding	ENSG00000137672	0,935749173	0,006341662	0,014571756
SEMA6A	protein_coding	ENSG00000092421	0,450655708	0,006350196	0,014589166
KANK2	protein_coding	ENSG00000197256	-0,181644733	0,006350662	0,014589166
ARMH4	protein_coding	ENSG00000139971	0,854699781	0,006352408	0,014591542
NPL	protein_coding	ENSG00000135838	0,987658505	0,006370683	0,014631883
CENPP	protein_coding	ENSG00000188312	-0,205103354	0,006371764	0,014632727
TNIK	protein_coding	ENSG00000154310	0,205838915	0,006372897	0,014633691
NANOS3	protein_coding	ENSG00000187556	1,589371824	0,006390537	0,014671814
FMO4	protein_coding	ENSG00000076258	0,607321643	0,006390931	0,014671814
PPP3CB-AS1	processed_transcript	ENSG00000221817	0,294365255	0,006393289	0,014672302
IGF1R	protein_coding	ENSG00000140443	0,206343231	0,006392912	0,014672302
NAGLU	protein_coding	ENSG00000108784	-0,233090475	0,006393126	0,014672302
NAAA	protein_coding	ENSG00000138744	0,785741335	0,006396078	0,01467706
DDIAS	protein_coding	ENSG00000165490	-0,163444546	0,006400581	0,01468575
DDX3X	protein_coding	ENSG00000215301	0,128537933	0,006418192	0,01472451
FBXL18	protein_coding	ENSG00000155034	0,274263153	0,006426891	0,014742819

HPD	protein_coding	ENSG00000158104	1,85230189	0,0064307	0,014749906
ZNF112	protein_coding	ENSG00000062370	0,235884995	0,006436478	0,014761509
AC010336.1	antisense	ENSG00000214248	-0,973432048	0,006440602	0,014769314
IPCEF1	protein_coding	ENSG00000074706	0,993718189	0,00644143	0,014769562
IGSF8	protein_coding	ENSG00000162729	-0,305703941	0,006450479	0,014788656
MVB12A	protein_coding	ENSG00000141971	-0,189591512	0,006452927	0,014792615
AC018638.4	processed_pseudogene	ENSG00000243302	0,620933455	0,006455243	0,01479627
FGGY	protein_coding	ENSG00000172456	0,214109939	0,00646122	0,014808316
MGAT2	protein_coding	ENSG00000168282	-0,12675716	0,006464975	0,014815268
MRPS35	protein_coding	ENSG00000061794	-0,176892204	0,006472585	0,01483105
MSL3	protein_coding	ENSG00000005302	-0,193345829	0,006483083	0,014853443
TUB	protein_coding	ENSG00000166402	-0,23946393	0,006486209	0,014858947
PRKAR2B	protein_coding	ENSG00000005249	-0,232470367	0,0064968	0,014881547
ECE1	protein_coding	ENSG00000117298	0,166496539	0,006503087	0,014894285
CD151	protein_coding	ENSG00000177697	-0,17326363	0,006515067	0,014920056
LRCH4	protein_coding	ENSG00000077454	0,38257263	0,006520232	0,014930218
PJA1	protein_coding	ENSG00000181191	0,160938379	0,00652165	0,014931798
ZBTB18	protein_coding	ENSG00000179456	-0,186028065	0,006534638	0,014959864
MRFAP1	protein_coding	ENSG00000179010	-0,096484121	0,006548179	0,01498919
COA5	protein_coding	ENSG00000183513	-0,1732916	0,006551397	0,014994885
PEX14	protein_coding	ENSG00000142655	-0,180721722	0,006560592	0,015014253
LOXL4	protein_coding	ENSG00000138131	1,829939814	0,006573315	0,015041692
IQCH	protein_coding	ENSG00000103599	0,347425203	0,006586348	0,015069834
MBLAC1	protein_coding	ENSG00000214309	0,560675925	0,00660615	0,015113456
HNF1B	protein_coding	ENSG00000275410	-0,32723188	0,00660924	0,015118838
AC060766.6	lincRNA	ENSG00000267711	1,148412984	0,006643596	0,015195735
RPL23A	protein_coding	ENSG00000198242	-0,140268594	0,006650212	0,015209172
MFAP1	protein_coding	ENSG00000140259	-0,130925804	0,006655938	0,01522057
MYCBPAP	protein_coding	ENSG00000136449	1,644533065	0,006657704	0,015222912
UQCRBP2	processed_pseudogene	ENSG00000227941	0,903579619	0,006679563	0,01527119
GAPLINC	lincRNA	ENSG00000266835	0,376497975	0,00668068	0,015272041
SLC25A46	protein_coding	ENSG00000164209	0,181050008	0,006691889	0,015295959
BEND7	protein_coding	ENSG00000165626	0,541453016	0,006699496	0,015309934
ZCCHC7	protein_coding	ENSG00000147905	-0,187818549	0,006698775	0,015309934
C5AR2	protein_coding	ENSG00000134830	1,941509707	0,006708375	0,015328517
GATA3-AS1	lincRNA	ENSG00000197308	0,895827704	0,006710856	0,015332478
AC097376.2	antisense	ENSG00000273247	-0,58448099	0,006724154	0,015360279
SNORD59A	snoRNA	ENSG00000207031	-1,496619587	0,006724522	0,015360279
CEP83	protein_coding	ENSG00000173588	-0,211232619	0,006735144	0,015382828
RPSA	protein_coding	ENSG00000168028	-0,190779468	0,006741613	0,01539589

PSMF1	protein_coding	ENSG00000125818	-0,135413894	0,006744735	0,015401305
TATDN3	protein_coding	ENSG00000203705	-0,178965723	0,006767432	0,015451411
LRWD1	protein_coding	ENSG00000161036	-0,198472989	0,006787969	0,015496576
AC022149.1	processed_pseudogene	ENSG00000269378	-0,400268415	0,006798587	0,015519089
IL10RB-DT	antisense	ENSG00000223799	1,515005366	0,006809043	0,015541228
MTCH1	protein_coding	ENSG00000137409	0,155216442	0,006818738	0,015561623
CEP126	protein_coding	ENSG00000110318	0,356537204	0,006824744	0,015573597
ZCCHC24	protein_coding	ENSG00000165424	-0,292372096	0,006827148	0,015577351
PNPT1	protein_coding	ENSG00000138035	-0,190395304	0,006832233	0,01558722
IFT20	protein_coding	ENSG00000109083	0,149052878	0,00683551	0,015592962
ENOX1	protein_coding	ENSG00000120658	-0,315068026	0,006841559	0,015605024
BBX	protein_coding	ENSG00000114439	0,137955211	0,006848153	0,015618327
AIG1	protein_coding	ENSG00000146416	0,16536468	0,00686247	0,015649239
AC010504.1	antisense	ENSG00000267219	1,251724827	0,006866464	0,015654129
PIK3CD-AS2	antisense	ENSG00000231789	0,456656881	0,006866904	0,015654129
ICE2	protein_coding	ENSG00000128915	-0,148343558	0,006866181	0,015654129
RPL7P38	processed_pseudogene	ENSG00000239473	1,920790186	0,006882495	0,015687927
MMP9	protein_coding	ENSG00000100985	0,553551054	0,00688342	0,015688293
FAM86C2P	transcribed_unprocesse	ENSG00000160172	-0,373787935	0,006885996	0,01569242
AL157400.3	lincRNA	ENSG00000235100	0,894208332	0,006892168	0,015704601
MRPS6	protein_coding	ENSG00000243927	-0,180634628	0,006892873	0,015704601
ARHGAP25	protein_coding	ENSG00000163219	1,453366053	0,006904127	0,015728495
KIF26B	protein_coding	ENSG00000162849	0,848418953	0,006906397	0,01573192
AC019068.1	antisense	ENSG00000233611	-0,422772938	0,006910934	0,015740505
NEDD8	protein_coding	ENSG00000129559	-0,166505465	0,006928329	0,015778372
TRAPPC6A	protein_coding	ENSG00000007255	0,34785394	0,006931157	0,015783061
FAM92A	protein_coding	ENSG00000188343	-0,222164248	0,006932688	0,015784795
TRIM16L	protein_coding	ENSG00000108448	0,189486054	0,006937193	0,0157933
CU638689.4	lincRNA	ENSG00000280145	1,11940146	0,006946448	0,015811799
RNFT1	protein_coding	ENSG00000189050	0,303415646	0,006946861	0,015811799
MYOF	protein_coding	ENSG00000138119	-0,133158916	0,006950959	0,015819371
TMEM39B	protein_coding	ENSG00000121775	-0,206363489	0,006951976	0,015819931
ZNF263	protein_coding	ENSG00000006194	-0,155306444	0,006966613	0,01585148
AC123595.1	lincRNA	ENSG00000234292	1,485486623	0,006973575	0,015865561
CHRNB2	protein_coding	ENSG00000160716	1,994589093	0,006976896	0,015871355
RIMS1	protein_coding	ENSG00000079841	0,988842406	0,006996189	0,015911885
PCMTD1	protein_coding	ENSG00000168300	0,217564558	0,006996264	0,015911885
NEDD1	protein_coding	ENSG00000139350	-0,195420168	0,007001816	0,015922746
PITPNM3	protein_coding	ENSG00000091622	0,495811961	0,007005569	0,015929513
OR7E59P	unprocessed_pseudoge	ENSG00000250561	1,380527505	0,007009767	0,015937294

KIAA0040	protein_coding	ENSG00000235750	1,072370088	0,007015592	0,015948769
ABCF3	protein_coding	ENSG00000161204	-0,135442902	0,007017084	0,015950393
MTF2	protein_coding	ENSG00000143033	-0,139389067	0,007018013	0,015950737
RASSF2	protein_coding	ENSG00000101265	-0,304984222	0,007038579	0,015995707
SRPK1	protein_coding	ENSG00000096063	-0,114015405	0,007041139	0,015999753
MARCH9	protein_coding	ENSG00000139266	-0,199099992	0,007046248	0,016009588
CPTP	protein_coding	ENSG00000224051	-0,240202134	0,007049828	0,016015947
KCTD7	protein_coding	ENSG00000243335	-0,23457825	0,007052128	0,016019397
FA2H	protein_coding	ENSG00000103089	1,219626708	0,007055839	0,016026053
L2HGDH	protein_coding	ENSG00000087299	-0,192980446	0,007070241	0,016056986
PRKAR2A-AS1	antisense	ENSG00000224424	0,451329273	0,007089431	0,016097002
STT3A	protein_coding	ENSG00000134910	0,098790165	0,007089286	0,016097002
SCN4B	protein_coding	ENSG00000177098	1,371204161	0,007095459	0,016108907
RASGRP2	protein_coding	ENSG00000068831	1,026055775	0,007105106	0,016129023
AKR7A2	protein_coding	ENSG00000053371	-0,163503164	0,0071068	0,016131083
NDUFAF6	protein_coding	ENSG00000156170	0,182167659	0,007136116	0,016195833
RUFY3	protein_coding	ENSG00000018189	-0,206213471	0,007147614	0,016220133
PDCD5	protein_coding	ENSG00000105185	-0,129630711	0,007158414	0,016242846
COA6	protein_coding	ENSG00000168275	-0,248383389	0,007162238	0,016249724
PPA2	protein_coding	ENSG00000138777	-0,185700641	0,007170449	0,016266554
ZMYND11	protein_coding	ENSG00000015171	0,167931657	0,007173023	0,016266995
ZC3H18	protein_coding	ENSG00000158545	-0,205272807	0,007173001	0,016266995
SNORD69	snoRNA	ENSG00000212452	-1,102392305	0,00717173	0,016266995
CST6	protein_coding	ENSG00000175315	1,250075143	0,007174893	0,016269436
COP1	protein_coding	ENSG00000143207	-0,163153634	0,007176757	0,016271865
PTBP1	protein_coding	ENSG00000011304	-0,133093884	0,007193906	0,016308942
LINC01144	lincRNA	ENSG00000281912	-0,897072111	0,007209288	0,01634201
AL138762.1	antisense	ENSG00000272572	-0,758434514	0,007213207	0,016349085
HLA-DRB1	protein_coding	ENSG00000196126	-0,135774469	0,007221612	0,016366327
HNRNPAB	protein_coding	ENSG00000197451	-0,154128098	0,007237422	0,016400346
ALDH3B1	protein_coding	ENSG00000006534	0,222684129	0,007239395	0,016403004
KMT2A	protein_coding	ENSG00000118058	0,204847977	0,007244523	0,01641281
MINDY2	protein_coding	ENSG00000128923	-0,205157372	0,007245449	0,016413094
C6orf52	protein_coding	ENSG00000137434	0,571792617	0,00724667	0,016414048
STOML1	protein_coding	ENSG00000067221	0,265738668	0,007261028	0,016443526
SH2D5	protein_coding	ENSG00000189410	0,210013283	0,007261288	0,016443526
AC026254.2	lincRNA	ENSG00000266313	0,982492772	0,007263312	0,016444479
CHRDL1	protein_coding	ENSG00000101938	0,112790196	0,007263222	0,016444479
AC004241.1	antisense	ENSG00000257433	0,790507611	0,007282169	0,016485353
AC010969.2	lincRNA	ENSG00000269973	-0,256182179	0,007284915	0,016489747

FRA10AC1	protein_coding	ENSG00000148690	-0,185244163	0,007292531	0,016505166
AC091729.3	antisense	ENSG00000229043	-0,26657092	0,007294654	0,016508148
PLEKHB2	protein_coding	ENSG00000115762	-0,093646258	0,007303791	0,016527004
AC019077.1	antisense	ENSG00000214559	1,875542373	0,007307691	0,016534004
AC120024.1	lincRNA	ENSG00000260369	-0,862454017	0,00731437	0,016547289
RAB11FIP3	protein_coding	ENSG00000090565	-0,210812609	0,007324184	0,016567665
GPR162	protein_coding	ENSG00000250510	-0,348088376	0,007337238	0,016595362
PTPDC1	protein_coding	ENSG00000158079	0,214375121	0,007345596	0,016612436
AC006978.1	processed_pseudogene	ENSG00000235859	-0,519958986	0,007351218	0,016623316
LINC00640	lincRNA	ENSG00000258479	1,097316329	0,007355437	0,016631024
CPEB2-DT	lincRNA	ENSG00000247624	1,360687447	0,007361318	0,016642486
ANKRD13B	protein_coding	ENSG00000198720	-0,285303428	0,007368629	0,016657178
PCGF2	protein_coding	ENSG00000277258	0,26205891	0,00738096	0,016683214
FAM169A	protein_coding	ENSG00000198780	-0,155918631	0,007385227	0,01669102
CTC1	protein_coding	ENSG00000178971	-0,231965903	0,007427325	0,016784314
SHISAL1	protein_coding	ENSG00000138944	1,720089007	0,007430428	0,016789477
C17orf100	protein_coding	ENSG00000256806	-0,341989463	0,007442966	0,016815953
EVA1B	protein_coding	ENSG00000142694	-0,334026322	0,007444908	0,016818488
INTS9	protein_coding	ENSG00000104299	-0,178451245	0,00744908	0,016826061
CNIH1	protein_coding	ENSG00000100528	-0,209546331	0,007453009	0,016833081
HOXA5	protein_coding	ENSG00000106004	-0,605960194	0,007453984	0,016833431
LINC01503	lincRNA	ENSG00000233901	0,923077243	0,007458999	0,016842901
RSL24D1	protein_coding	ENSG00000137876	-0,231273938	0,007465787	0,016856372
CTNNBL1	protein_coding	ENSG00000132792	-0,145320578	0,007471751	0,016867981
EFNA3	protein_coding	ENSG00000143590	-0,577445826	0,0074733	0,016869621
BCL2L1	protein_coding	ENSG00000171552	-0,146438688	0,007479728	0,016882274
BTD	protein_coding	ENSG00000169814	-0,203534944	0,007484717	0,016891675
SH3RF3	protein_coding	ENSG00000172985	-0,212215318	0,007517849	0,016964581
TUBA1A	protein_coding	ENSG00000167552	-0,182376809	0,007518927	0,016965148
MPP6	protein_coding	ENSG00000105926	-0,177228655	0,00753534	0,01700031
NFYC-AS1	antisense	ENSG00000272145	-0,555297875	0,007538989	0,017002931
PRMT9	protein_coding	ENSG00000164169	0,19340101	0,007537753	0,017002931
AC005911.1	sense_intronic	ENSG00000278356	-0,810665566	0,007538235	0,017002931
RPS20P23	processed_pseudogene	ENSG00000242399	1,764508177	0,007545477	0,017015694
SYNGAP1	protein_coding	ENSG00000197283	0,45747746	0,007549364	0,017022587
USB1	protein_coding	ENSG00000103005	-0,280984856	0,007557191	0,017038362
BMI1	protein_coding	ENSG00000168283	-0,218529331	0,007567152	0,017058946
SLC35E3	protein_coding	ENSG00000175782	0,148664159	0,007577985	0,01708149
LINC00857	lincRNA	ENSG00000237523	-0,197083246	0,007583388	0,017089913
C17orf80	protein_coding	ENSG00000141219	0,125640403	0,00758258	0,017089913

TMEM254-AS1	antisense	ENSG00000230091	0,934962044	0,007589253	0,01710125
TRIM68	protein_coding	ENSG00000167333	-0,214575501	0,007595631	0,017113743
SECTM1	protein_coding	ENSG00000141574	-0,385514585	0,007603502	0,017129595
WFIKKN1	protein_coding	ENSG00000127578	1,863088336	0,007605851	0,017131122
UBE4B	protein_coding	ENSG00000130939	0,132771111	0,007605461	0,017131122
MNS1	protein_coding	ENSG00000138587	-0,27016492	0,007621332	0,017164107
LINC00326	lincRNA	ENSG00000231023	1,188234692	0,00763418	0,017191155
AC122718.2	processed_pseudogene	ENSG00000251682	1,036120423	0,007637116	0,017195879
ZBTB11-AS1	antisense	ENSG00000256628	0,314012466	0,007642545	0,017206213
NDUFAF1	protein_coding	ENSG00000137806	0,160145789	0,00764393	0,017207442
CCNYL2	transcribed_unprocesse	ENSG00000182632	0,189266512	0,007652841	0,017225612
CCDC18	protein_coding	ENSG00000122483	-0,180024842	0,007685642	0,017297543
AC012358.3	antisense	ENSG00000240401	1,709623294	0,007687807	0,017300518
NPB	protein_coding	ENSG00000183979	-0,875842691	0,00769658	0,017318361
RAPGEF4	protein_coding	ENSG00000091428	1,057747904	0,007704879	0,017335132
UBXN2B	protein_coding	ENSG00000215114	0,152797893	0,007719179	0,0173654
AC099518.4	antisense	ENSG00000261465	1,682608102	0,007723501	0,017373219
TENT5B	protein_coding	ENSG00000158246	0,227385141	0,007753964	0,01743983
CROCCP3	transcribed_unprocesse	ENSG00000080947	-0,526203404	0,007763594	0,017459573
ZNF343	protein_coding	ENSG00000088876	-0,225098938	0,0077729	0,017478585
FIG4	protein_coding	ENSG00000112367	0,152533795	0,007774493	0,017480251
GHITM	protein_coding	ENSG00000165678	0,160629463	0,00777787	0,017485928
TSPAN3	protein_coding	ENSG00000140391	0,092627363	0,007783079	0,017495719
EIF3E	protein_coding	ENSG00000104408	-0,199767389	0,007786004	0,017500378
C1GALT1P2	processed_pseudogene	ENSG00000251435	1,129350828	0,007792072	0,017512098
SAMD11	protein_coding	ENSG00000187634	0,513317765	0,007803117	0,017534997
ANKLE2	protein_coding	ENSG00000176915	0,127355774	0,007806441	0,017540547
AL592166.1	antisense	ENSG00000225721	0,79570467	0,007808071	0,017542286
KIF13B	protein_coding	ENSG00000197892	0,253115773	0,007816699	0,017559748
AASDH	protein_coding	ENSG00000157426	-0,195748249	0,00782193	0,017569574
CHD1L	protein_coding	ENSG00000131778	-0,12621195	0,007829443	0,017584525
IMMT	protein_coding	ENSG00000132305	-0,098564541	0,007834469	0,017593886
LINC00520	lincRNA	ENSG00000258791	0,932679796	0,007841997	0,017608864
AL357992.1	antisense	ENSG00000236591	0,930221308	0,00784711	0,017616487
TBR1	protein_coding	ENSG00000136535	1,302621474	0,007846557	0,017616487
NDUFB4	protein_coding	ENSG00000065518	-0,185636773	0,00785751	0,017637905
ZCCHC2	protein_coding	ENSG00000141664	-0,227425234	0,007859375	0,01764016
HNRNPA1P38	processed_pseudogene	ENSG00000253605	-1,7292866	0,00786147	0,017642932
EMD	protein_coding	ENSG00000102119	-0,144045681	0,007906789	0,017742695
ZNF184	protein_coding	ENSG00000096654	-0,166245909	0,007921821	0,017774484

EIF1AD	protein_coding	ENSG00000175376	-0,14735292	0,007930949	0,017793017
MANEA	protein_coding	ENSG00000172469	-0,251128267	0,007933585	0,017796984
TMCC1-AS1	antisense	ENSG00000271270	-0,282812312	0,007942787	0,017815679
AC093503.2	antisense	ENSG00000269292	1,724844026	0,007948447	0,017824475
AC087294.1	antisense	ENSG00000235530	-0,752620011	0,007948326	0,017824475
SLC25A16	protein_coding	ENSG00000122912	-0,172069845	0,007956526	0,017840643
AC010547.1	antisense	ENSG00000247324	1,529902056	0,007969093	0,017866866
CITED4	protein_coding	ENSG00000179862	0,311682568	0,007972609	0,017872796
RABL6	protein_coding	ENSG00000196642	-0,20405025	0,007975719	0,017877814
AKAP6	protein_coding	ENSG00000151320	0,647910643	0,007978891	0,017882337
MARVELD1	protein_coding	ENSG00000155254	-0,188862673	0,007979481	0,017882337
AC020594.1	antisense	ENSG00000237133	1,100591959	0,007995205	0,017915619
TMEM185A	protein_coding	ENSG00000269556	0,300065631	0,00801239	0,017951424
SPINK13	protein_coding	ENSG00000214510	-0,821145826	0,008012935	0,017951424
AC009404.1	lincRNA	ENSG00000236255	0,533719345	0,008015726	0,017955717
ASIC4	protein_coding	ENSG00000072182	1,818444577	0,008034652	0,017996147
AL359258.2	antisense	ENSG00000260879	1,224860897	0,008035697	0,017996522
PSMD11	protein_coding	ENSG00000108671	0,081152174	0,008043102	0,018011139
ZNF225	protein_coding	ENSG00000256294	0,218015081	0,008044532	0,018012375
APOOL	protein_coding	ENSG00000155008	0,183000414	0,008053657	0,018030838
AL353719.1	lincRNA	ENSG00000260475	-0,966754248	0,008059791	0,018040632
CARD11	protein_coding	ENSG00000198286	1,649778492	0,008059179	0,018040632
XKR5	protein_coding	ENSG00000275591	-0,598345372	0,008065977	0,018052507
AC010894.2	lincRNA	ENSG00000226853	0,755625949	0,008069305	0,018057986
ADSSL1	protein_coding	ENSG00000185100	0,50685821	0,008075819	0,018070592
B3GNT4	protein_coding	ENSG00000176383	-0,701732539	0,008085179	0,018089562
CAMK1D	protein_coding	ENSG00000183049	0,19076082	0,008108572	0,018139924
WASF1	protein_coding	ENSG00000112290	0,260056006	0,008110697	0,018142697
RSPH4A	protein_coding	ENSG00000111834	1,167716534	0,008120837	0,018163399
FAM3C2	processed_pseudogene	ENSG00000174028	0,448718707	0,008150922	0,018228701
CEP19	protein_coding	ENSG00000174007	0,257326794	0,008158349	0,01823937
RILPL2	protein_coding	ENSG00000150977	0,203946444	0,008157839	0,01823937
MADD	protein_coding	ENSG00000110514	-0,174698329	0,008158361	0,01823937
PHB	protein_coding	ENSG00000167085	-0,158650903	0,008169632	0,018262578
S1PR1	protein_coding	ENSG00000170989	-0,105312909	0,008202521	0,018334099
AC025031.2	antisense	ENSG00000258096	1,83455899	0,008208623	0,018343742
ZNF226	protein_coding	ENSG00000167380	-0,164914649	0,008207919	0,018343742
TRMT2B	protein_coding	ENSG00000188917	-0,138127483	0,008209796	0,018344364
AC006077.2	TEC	ENSG00000279799	-0,865327447	0,008232529	0,018393157
PROSER2	protein_coding	ENSG00000148426	-0,184615005	0,008233907	0,01839423

AMY2B	protein_coding	ENSG00000240038	0,566398015	0,008235829	0,018396521
FOXK2	protein_coding	ENSG00000141568	-0,123686319	0,008237112	0,018397383
S100A4	protein_coding	ENSG00000196154	0,582168969	0,008240187	0,018400243
BCL9L	protein_coding	ENSG00000186174	-0,261823534	0,008239594	0,018400243
AP003469.2	lincRNA	ENSG00000253395	1,187661803	0,008276685	0,01847973
LINC01436	lincRNA	ENSG00000231106	0,334951193	0,008291802	0,018511469
AC104806.2	lincRNA	ENSG00000250075	1,02590497	0,008295811	0,018518404
DCTN5	protein_coding	ENSG00000166847	0,112527996	0,008315306	0,0185599
TCF25	protein_coding	ENSG00000141002	-0,155694289	0,008318837	0,018565762
MBTD1	protein_coding	ENSG00000011258	0,177652268	0,008345089	0,018622323
COL6A5	protein_coding	ENSG00000172752	1,510914271	0,008347368	0,01862388
HIST1H2APS5	unprocessed_pseudoge	ENSG00000234816	-0,440706836	0,008347603	0,01862388
SNX5	protein_coding	ENSG00000089006	-0,112740351	0,008398653	0,018735737
SSSCA1	protein_coding	ENSG00000173465	-0,218175314	0,008407353	0,018753105
NKILA	antisense	ENSG00000278709	0,643383248	0,008433485	0,018807825
CMTM1	protein_coding	ENSG00000089505	-0,570998282	0,008433718	0,018807825
EIF3M	protein_coding	ENSG00000149100	-0,176793727	0,008436977	0,018813047
CCDC74B	protein_coding	ENSG00000152076	-0,624651619	0,008449608	0,018839163
HDAC6	protein_coding	ENSG00000094631	-0,185467768	0,008470155	0,018882921
ELP4	protein_coding	ENSG00000109911	0,187371638	0,008471897	0,018884753
AC020661.4	sense_intronic	ENSG00000280036	-1,228077712	0,008477093	0,018894281
TAF1	protein_coding	ENSG00000147133	-0,150745987	0,008479553	0,01889771
AC008755.1	protein_coding	ENSG00000130749	-0,218969368	0,008548773	0,019049905
SMCHD1	protein_coding	ENSG00000101596	-0,193992365	0,008554791	0,019061246
SUMF2	protein_coding	ENSG00000129103	-0,160478593	0,008559299	0,019069218
AL158151.3	antisense	ENSG00000268050	1,712006425	0,008563125	0,019073598
KLHL25	protein_coding	ENSG00000183655	-0,323587	0,008562597	0,019073598
ACP1	protein_coding	ENSG00000143727	-0,143490147	0,008583146	0,019116118
SLC46A1	protein_coding	ENSG00000076351	-0,187197146	0,008594157	0,019138563
BAG5	protein_coding	ENSG00000166170	-0,112907947	0,008597962	0,019144957
MANSC1	protein_coding	ENSG00000111261	0,733527834	0,008600045	0,019147516
GPI	protein_coding	ENSG00000105220	-0,099115229	0,008602381	0,019150638
AL021408.1	lincRNA	ENSG00000236166	1,10964585	0,008620411	0,019188695
MECOM	protein_coding	ENSG00000085276	0,175529938	0,008639637	0,019229403
ADSL	protein_coding	ENSG00000239900	-0,281032234	0,008642701	0,019234136
FAM241B	protein_coding	ENSG00000171224	-0,200604952	0,008652675	0,019254245
AP4S1	protein_coding	ENSG00000100478	0,208290751	0,00866636	0,019282604
HDDC3	protein_coding	ENSG00000184508	-0,229790318	0,008692745	0,019339212
PRF1	protein_coding	ENSG00000180644	1,655206048	0,008699904	0,019353041
AC093323.1	protein_coding	ENSG00000170846	-0,170499765	0,008724929	0,019404499

RPL29P30	transcribed_processed_	ENSG00000235420	0,780247806	0,008724097	0,019404499
MIR193BHG	lincRNA	ENSG00000262454	-0,802129731	0,008736819	0,019428835
SNORA80D	snoRNA	ENSG00000207217	-0,526993226	0,008749465	0,019454849
TDG	protein_coding	ENSG00000139372	-0,172477488	0,00875314	0,019460912
ARNTL2	protein_coding	ENSG00000029153	0,102473101	0,008765063	0,019485307
PHIP	protein_coding	ENSG00000146247	-0,200361981	0,008767339	0,019488255
AIMP1	protein_coding	ENSG00000164022	-0,217012022	0,008776085	0,019505581
TNFRSF1B	protein_coding	ENSG00000028137	1,316294681	0,008802968	0,01956321
RPGR	protein_coding	ENSG00000156313	0,202032147	0,008805933	0,019567679
PDCD10	protein_coding	ENSG00000114209	-0,233202017	0,008807816	0,019569744
MAMDC4	protein_coding	ENSG00000177943	0,488993163	0,008819035	0,019592548
AL139023.1	lincRNA	ENSG00000257869	-0,321950962	0,008826109	0,019604365
TXNDC9	protein_coding	ENSG00000115514	-0,242669977	0,008826266	0,019604365
MEGF11	protein_coding	ENSG00000157890	1,394878548	0,008835836	0,019623497
C12orf50	protein_coding	ENSG00000165805	1,114834895	0,008839222	0,019626771
USP12	protein_coding	ENSG00000152484	0,118146931	0,008839224	0,019626771
AC010680.2	antisense	ENSG00000270574	0,567766423	0,008846892	0,019641672
R3HDM2	protein_coding	ENSG00000179912	0,252776356	0,008848389	0,019642868
FSTL1	protein_coding	ENSG00000163430	-0,099249361	0,008852601	0,019650092
EID3	protein_coding	ENSG00000255150	-0,500331849	0,008866341	0,019678462
IGF2	protein_coding	ENSG00000167244	0,615897787	0,008873264	0,019691695
LINC02506	lincRNA	ENSG00000251129	0,243130832	0,008918334	0,019789574
FMR1	protein_coding	ENSG00000102081	0,160302779	0,00892296	0,019797697
MSMO1	protein_coding	ENSG00000052802	0,204149431	0,008928936	0,019808812
UNC93B1	protein_coding	ENSG00000110057	-0,192383536	0,008930861	0,019810942
KIF17	protein_coding	ENSG00000117245	0,272803232	0,008932571	0,019812591
AC003991.1	antisense	ENSG00000228113	1,64740726	0,008938182	0,019822893
PTPRO	protein_coding	ENSG00000151490	1,613108421	0,008949421	0,019845673
SCAPER	protein_coding	ENSG00000140386	0,149341353	0,008959873	0,019866702
OSBPL5	protein_coding	ENSG00000021762	0,191628594	0,008964409	0,019874612
SH3RF3-AS1	lincRNA	ENSG00000259863	1,007825912	0,009002194	0,019956226
RFK	protein_coding	ENSG00000135002	0,223747474	0,009006745	0,019964157
AC100830.1	antisense	ENSG00000259635	1,757430749	0,009017219	0,019985214
ZNF75D	protein_coding	ENSG00000186376	0,227173	0,00901897	0,019986935
STARD13-AS	processed_transcript	ENSG00000236581	0,925215962	0,009054908	0,020064408
SH2D4B	protein_coding	ENSG00000178217	1,054132162	0,009057021	0,020066923
CAPN1	protein_coding	ENSG00000014216	0,160449881	0,009063535	0,020079185
CCDC18-AS1	processed_transcript	ENSG00000223745	0,327357526	0,009071562	0,020094798
PDE4D	protein_coding	ENSG00000113448	1,749105707	0,009080197	0,020111754
ZNF286A	protein_coding	ENSG00000187607	-0,192882148	0,009084947	0,020120101

ASAH2	protein_coding	ENSG00000188611	0,680437431	0,009089409	0,020127811
MUC19	protein_coding	ENSG00000205592	1,181865595	0,009110742	0,020172872
LINC00211	lincRNA	ENSG00000237803	0,717557882	0,009112792	0,020175234
RAB4A	protein_coding	ENSG00000168118	-0,140233532	0,009117346	0,020183137
LINC02516	lincRNA	ENSG00000261083	1,336620778	0,009128729	0,020206156
AC139149.1	antisense	ENSG00000229848	1,179333582	0,009134976	0,020213438
TECTA	protein_coding	ENSG00000109927	0,948510475	0,009134213	0,020213438
AC092279.2	TEC	ENSG00000279425	1,143953437	0,00913361	0,020213438
ACTR8	protein_coding	ENSG00000113812	0,137023678	0,009139048	0,020220267
AC104590.1	antisense	ENSG00000259251	1,208929705	0,009150428	0,020243263
LINC00466	processed_transcript	ENSG00000224209	0,505621928	0,009157348	0,020254201
AC098934.2	transcribed_processed_	ENSG00000234996	1,161475003	0,009156966	0,020254201
CKLF	protein_coding	ENSG00000217555	-0,370443741	0,00917294	0,020286499
MYLK-AS2	antisense	ENSG00000250174	1,68883309	0,009178923	0,020297543
MIOX	protein_coding	ENSG00000100253	1,595816458	0,009187328	0,02031394
MMP25	protein_coding	ENSG00000008516	1,489593313	0,009190225	0,020318154
SUPT20H	protein_coding	ENSG00000102710	-0,13411586	0,009206258	0,020351407
COL19A1	protein_coding	ENSG00000082293	0,456068326	0,009230948	0,020403787
SBNO2	protein_coding	ENSG00000064932	0,230266116	0,009243358	0,020429017
AC093227.1	lincRNA	ENSG00000267152	-0,563703312	0,009276813	0,020500746
GZMM	protein_coding	ENSG00000197540	1,266136395	0,0092791	0,020503591
GOLGA8R	protein_coding	ENSG00000186399	1,356310107	0,009280222	0,020503863
AC011374.2	lincRNA	ENSG00000272112	0,974595781	0,009287018	0,020516667
MDN1	protein_coding	ENSG00000112159	-0,232023141	0,009292087	0,020525655
AC007405.1	lincRNA	ENSG00000234350	1,170478601	0,009299184	0,020539119
PIN4	protein_coding	ENSG00000102309	-0,198096507	0,009313109	0,020567661
ARPC1A	protein_coding	ENSG00000241685	-0,260718766	0,009316092	0,020572033
FGD4	protein_coding	ENSG00000139132	0,286083764	0,009334639	0,020610771
B4GALT3	protein_coding	ENSG00000158850	-0,139175609	0,009335835	0,020611192
MAP3K13	protein_coding	ENSG00000073803	0,17493526	0,009369168	0,020678105
ZNF185	protein_coding	ENSG00000147394	-0,113870966	0,009369066	0,020678105
SET	protein_coding	ENSG00000119335	-0,128604804	0,009367978	0,020678105
GAD1	protein_coding	ENSG00000128683	-0,216816338	0,009376026	0,020691015
EMC3-AS1	transcribed_unprocesse	ENSG00000180385	0,252848295	0,009377921	0,02069297
EDA	protein_coding	ENSG00000158813	-0,533032366	0,009397133	0,020733133
SDHC	protein_coding	ENSG00000143252	-0,117205107	0,009416949	0,02077375
NUP54	protein_coding	ENSG00000138750	-0,192906989	0,009417568	0,02077375
ALDH2	protein_coding	ENSG00000111275	1,737656145	0,00944055	0,020819966
SWSAP1	protein_coding	ENSG00000173928	-0,438589192	0,009447958	0,020834062
GSKIP	protein_coding	ENSG00000100744	0,209924263	0,009452875	0,020842664

TUT4	protein_coding	ENSG00000134744	-0,170171275	0,0094622	0,020860982
SYNE3	protein_coding	ENSG00000176438	-0,226425355	0,009496714	0,020934823
COPS8	protein_coding	ENSG00000198612	0,111963142	0,009499668	0,020939086
AL158152.1	lincRNA	ENSG00000269929	0,45494695	0,009541778	0,021029643
FAM180A	protein_coding	ENSG00000189320	1,593058089	0,009561596	0,021071057
PTCH1	protein_coding	ENSG00000185920	-0,426384922	0,009586026	0,021122626
JTB	protein_coding	ENSG00000143543	-0,181928889	0,009591089	0,021131511
AC016738.1	antisense	ENSG00000223947	0,987110105	0,009606265	0,021162674
AC022126.1	antisense	ENSG00000248898	1,597824489	0,009617245	0,021184587
CERS1	protein_coding	ENSG00000223802	-0,557018726	0,009621296	0,021191235
CERS1	protein_coding	ENSG00000223802	-0,557018726	0,009621296	0,021191235
OGFOD1P1	processed_pseudogene	ENSG00000231665	1,631619607	0,009633126	0,021212736
PFKL	protein_coding	ENSG00000141959	-0,182828732	0,009632237	0,021212736
ANGPTL6	protein_coding	ENSG00000130812	1,136307873	0,009648782	0,021244697
YWHAE	protein_coding	ENSG00000108953	-0,153531559	0,009649712	0,021244697
LINC01135	lincRNA	ENSG00000234807	0,954844084	0,009665857	0,021277957
DAPK2	protein_coding	ENSG00000035664	-0,338033806	0,009672537	0,021290377
COMMD2	protein_coding	ENSG00000114744	-0,18346704	0,009681062	0,021306855
GEMIN7	protein_coding	ENSG00000142252	-0,1951835	0,009691334	0,021327174
MYLK3	protein_coding	ENSG00000140795	1,410887706	0,009721974	0,021392307
C12orf73	protein_coding	ENSG00000204954	0,230258225	0,009728084	0,021403454
BAIAP2L2	protein_coding	ENSG00000128298	1,709775261	0,009737535	0,021421951
AP005329.3	lincRNA	ENSG00000272688	0,487036903	0,009743663	0,021433131
PRR7-AS1	antisense	ENSG00000246334	-0,5799245	0,009747402	0,021439058
ARHGAP44	protein_coding	ENSG00000006740	-0,476278752	0,009750651	0,021443904
TYMS	protein_coding	ENSG00000176890	-0,207620065	0,009765212	0,021473626
AC025031.5	TEC	ENSG00000278896	1,107010799	0,009776039	0,021495128
ARHGAP27P1-BPTFP1	processed_transcript	ENSG00000215769	0,685245631	0,009783556	0,021509352
SAA2	protein_coding	ENSG00000134339	1,562265738	0,009787493	0,0215157
SNORD53	snoRNA	ENSG00000265145	-1,096299862	0,009791847	0,021522964
HNRNPCP2	processed_pseudogene	ENSG00000204253	-0,28539294	0,009800534	0,02153975
Z84485.1	antisense	ENSG00000246982	0,626004616	0,009803627	0,021544239
LINC01123	lincRNA	ENSG00000204588	-0,50296266	0,009805326	0,021545664
KIF16B	protein_coding	ENSG00000089177	0,215525284	0,009811645	0,02155724
CPE	protein_coding	ENSG00000109472	0,361269883	0,009820123	0,021573557
BHMG1	protein_coding	ENSG00000237452	1,579521955	0,009836443	0,021607094
PHF10	protein_coding	ENSG00000130024	-0,186498433	0,009839117	0,021610655
ERVK13-1	processed_transcript	ENSG00000260565	0,35026237	0,009841955	0,021614574
EYS	protein_coding	ENSG00000188107	1,252181852	0,009853112	0,021636759
ELOVL1	protein_coding	ENSG00000066322	0,136494655	0,009868861	0,021669023

LINC01389	antisense	ENSG00000225762	0,874712687	0,009874403	0,02167887
AC004706.3	TEC	ENSG00000282936	0,652169929	0,009878241	0,021684976
CALCOCO1	protein_coding	ENSG00000012822	0,189042162	0,009881509	0,021689827
SMYD3	protein_coding	ENSG00000185420	-0,128548692	0,009888329	0,021702475
SUB1	protein_coding	ENSG00000113387	-0,218893572	0,009895006	0,021714805
AL158151.1	antisense	ENSG00000204055	0,885850135	0,00990136	0,021726425
ZRANB3	protein_coding	ENSG00000121988	-0,21055139	0,009908989	0,021740838
NR2F6	protein_coding	ENSG00000160113	-0,211897881	0,009920778	0,021764377
GALE	protein_coding	ENSG00000117308	-0,188842439	0,009923552	0,021768133
SH3BGRL2	protein_coding	ENSG00000198478	-0,282373436	0,009933059	0,021786658
AC073115.2	lincRNA	ENSG00000237471	1,104751874	0,009957596	0,02183814
SNRPEP4	processed_pseudogene	ENSG00000233270	-0,589802904	0,00996712	0,021856692
ZPLD1	protein_coding	ENSG00000170044	1,299034682	0,009968333	0,021857014
FAM71F2	protein_coding	ENSG00000205085	0,885132004	0,00997207	0,021862871
AC090515.2	lincRNA	ENSG00000245975	0,89535051	0,009988461	0,021896467
OSBP2	protein_coding	ENSG00000184792	0,276545055	0,009994068	0,021906417
AP006284.1	antisense	ENSG00000254815	1,552032052	0,010020179	0,021961303
AL157394.1	sense_overlapping	ENSG00000261438	-0,44933333	0,010040698	0,022003923
PREB	protein_coding	ENSG00000138073	-0,165779978	0,010051003	0,022022768
NOP10	protein_coding	ENSG00000182117	-0,182425924	0,010051445	0,022022768
RPP21	protein_coding	ENSG00000241370	-0,771597892	0,010079022	0,022080831
RPP21	protein_coding	ENSG00000241370	-0,771597892	0,010079022	0,022080831
AC027237.2	lincRNA	ENSG00000259215	1,552526716	0,010080512	0,022081737
MTPN	protein_coding	ENSG00000105887	0,107252552	0,010082877	0,022084559
SEPT11	protein_coding	ENSG00000138758	0,09740222	0,010089424	0,022096538
IPO9-AS1	antisense	ENSG00000231871	1,053426841	0,010094034	0,022104275
AC012615.1	lincRNA	ENSG00000261526	-0,606645004	0,01009999	0,022114957
PPP1R11	protein_coding	ENSG00000204619	-0,139615572	0,010128198	0,022171986
ANKRD26	protein_coding	ENSG00000107890	-0,20830259	0,010127829	0,022171986
SLX4IP	protein_coding	ENSG00000149346	0,189415706	0,010130946	0,022175636
AC021752.1	lincRNA	ENSG00000273674	1,361091092	0,010133535	0,022176569
AC067852.3	sense_intronic	ENSG00000267632	0,75847433	0,010133294	0,022176569
AL023806.1	lincRNA	ENSG00000270638	-0,940730739	0,010134937	0,022177272
CLSTN3	protein_coding	ENSG00000139182	0,540437706	0,010142052	0,022190473
FCF1	protein_coding	ENSG00000119616	-0,138221199	0,010146238	0,022197262
AC012506.4	lincRNA	ENSG00000235497	1,541469358	0,010147456	0,02219756
PPP6R3	protein_coding	ENSG00000110075	-0,133563565	0,010153391	0,022208174
SRPX	protein_coding	ENSG00000101955	-0,447469864	0,010174154	0,022251215
MRPL41	protein_coding	ENSG00000182154	-0,223398381	0,010177478	0,022256112
AC104024.1	lincRNA	ENSG00000230709	1,010061597	0,010191714	0,022284866

RNF113A	protein_coding	ENSG00000125352	0,179279898	0,010203731	0,022308766
FKBP7	protein_coding	ENSG00000079150	0,333500707	0,010212387	0,022325309
MMGT1	protein_coding	ENSG00000169446	0,156492178	0,010225936	0,022352548
METTL22	protein_coding	ENSG00000067365	-0,168157011	0,010236871	0,022374064
TBL1XR1	protein_coding	ENSG00000177565	-0,16816601	0,010240133	0,02237881
SPECC1	protein_coding	ENSG00000128487	1,143565564	0,010241287	0,022378947
AP000320.1	antisense	ENSG00000225555	1,154270685	0,010245356	0,022385453
AC099552.1	lincRNA	ENSG00000217825	0,984583946	0,010263447	0,022422593
NCOA6	protein_coding	ENSG00000198646	-0,201821793	0,010268458	0,02243115
KPNA2P1	processed_pseudogene	ENSG00000236530	1,720398043	0,010269593	0,022431242
DPP4	protein_coding	ENSG00000197635	0,493262585	0,010274846	0,022440324
NDUFS6	protein_coding	ENSG00000145494	-0,152238194	0,010313146	0,022521573
LINC01556	lincRNA	ENSG00000204709	-1,055860574	0,010323172	0,022541068
DUOX1	protein_coding	ENSG00000137857	1,601105144	0,010326512	0,022545962
AL138767.1	antisense	ENSG00000196566	0,963304238	0,010343875	0,022581465
NLGN3	protein_coding	ENSG00000196338	0,580454665	0,010346161	0,022584053
LINC01357	lincRNA	ENSG00000224167	0,348981513	0,010348129	0,022585945
IGFL2	protein_coding	ENSG00000204866	1,559477011	0,010357668	0,022604359
DXO	protein_coding	ENSG00000204348	0,225530149	0,010362668	0,022612866
ETFRF1	protein_coding	ENSG00000205707	0,298766283	0,010368242	0,022622621
AL022323.4	antisense	ENSG00000279110	1,683297258	0,010375051	0,02263507
OSCP1	protein_coding	ENSG00000116885	0,338429508	0,010380582	0,022644729
DCAF15	protein_coding	ENSG00000132017	-0,193591254	0,010394287	0,022672215
RN7SKP114	misc_RNA	ENSG00000222259	-1,578484102	0,010411445	0,022707224
RAB7A	protein_coding	ENSG00000075785	0,125248048	0,01041399	0,022710359
KLHL7-DT	lincRNA	ENSG00000230658	0,562025193	0,010458158	0,022804255
AC100860.1	lincRNA	ENSG00000253266	1,562248693	0,010506937	0,022908184
CCDC71L	protein_coding	ENSG00000253276	-0,172766786	0,01051608	0,022925681
SINHCAF	protein_coding	ENSG00000139146	-0,127705338	0,010518106	0,022927661
DRC1	protein_coding	ENSG00000157856	1,532694037	0,010536007	0,022961799
GOLGA3	protein_coding	ENSG00000090615	-0,222162484	0,010534995	0,022961799
NLRC4	protein_coding	ENSG00000091106	1,412625943	0,010545486	0,022980018
AL035446.1	lincRNA	ENSG00000234147	0,301997488	0,010560643	0,023010601
CD164	protein_coding	ENSG00000135535	-0,161402879	0,010566649	0,023021242
SNORC	protein_coding	ENSG00000182600	0,580784352	0,010589759	0,023069141
H2AFY	protein_coding	ENSG00000113648	-0,09455768	0,010606294	0,023102707
AC012313.1	lincRNA	ENSG00000232098	-0,348872812	0,010610054	0,023108443
RASEF	protein_coding	ENSG00000165105	1,525273054	0,010645495	0,023183171
ABHD14A	protein_coding	ENSG00000248487	0,385098369	0,010646663	0,023183253
AL162171.1	protein_coding	ENSG00000070778	-0,193727097	0,01065808	0,023205649

HSPBP1	protein_coding	ENSG00000133265	-0,199605896	0,010673713	0,023237221
AC091133.1	antisense	ENSG00000230532	0,749022934	0,010676441	0,023240692
AC008403.1	protein_coding	ENSG00000142235	0,662645911	0,010680746	0,023247596
AL133325.3	lincRNA	ENSG00000278041	-1,043885555	0,010708999	0,023306617
MSI2	protein_coding	ENSG00000153944	-0,125551163	0,01073707	0,023365231
AC099343.2	sense_intronic	ENSG00000270426	1,428814989	0,010775111	0,023445527
THNSL1	protein_coding	ENSG00000185875	-0,201255678	0,010779453	0,023452486
PRKCQ-AS1	processed_transcript	ENSG00000237943	-0,358656895	0,010790352	0,023473709
RNF43	protein_coding	ENSG00000108375	1,563704142	0,010826399	0,023549629
MTFR1	protein_coding	ENSG00000066855	-0,170247831	0,010846645	0,023591166
DTX3	protein_coding	ENSG00000178498	-0,20465657	0,010849428	0,023594716
SHLD1	protein_coding	ENSG00000171984	0,580882806	0,010857714	0,023610233
TENM1	protein_coding	ENSG00000009694	0,864372127	0,01086263	0,023618418
NSUN4	protein_coding	ENSG00000117481	0,139715036	0,010870459	0,023632936
AC079209.1	lincRNA	ENSG00000253214	0,950659968	0,010890826	0,023672249
NAT8L	protein_coding	ENSG00000185818	-0,259345654	0,01089085	0,023672249
DICER1	protein_coding	ENSG00000100697	-0,2127664	0,010898826	0,023687075
CYB5R2	protein_coding	ENSG00000166394	0,131114745	0,010917025	0,023724114
BLID	protein_coding	ENSG00000259571	0,981837374	0,010921788	0,023731949
CCDC114	protein_coding	ENSG00000105479	0,50511354	0,010926154	0,023738922
ADO	protein_coding	ENSG00000181915	-0,131447565	0,010970343	0,023832406
STAT5B	protein_coding	ENSG00000173757	-0,139319033	0,010987476	0,023867098
CHST7	protein_coding	ENSG00000147119	-0,205137281	0,010989775	0,023869563
AL731571.1	antisense	ENSG00000273599	0,957299579	0,010997569	0,023881434
SLC9A6	protein_coding	ENSG00000198689	-0,165941581	0,010996684	0,023881434
AC105052.2	processed_pseudogene	ENSG00000213385	-0,922971616	0,011009443	0,023904687
EIPR1	protein_coding	ENSG00000032389	-0,169229969	0,011022231	0,02392992
ZNF580	protein_coding	ENSG00000213015	0,277228917	0,011023439	0,02393001
SYT12	protein_coding	ENSG00000173227	0,933118472	0,011029817	0,023941321
REN	protein_coding	ENSG00000143839	0,706308412	0,011041881	0,023964971
AL356234.1	processed_pseudogene	ENSG00000220412	1,666614745	0,011049757	0,023979528
AL034417.2	lincRNA	ENSG00000238290	1,52538883	0,011078148	0,024038597
RYR3	protein_coding	ENSG00000198838	1,651824981	0,011087572	0,024056502
CCN3	protein_coding	ENSG00000136999	0,506276002	0,011091947	0,024061508
SNRPC	protein_coding	ENSG00000124562	-0,144979796	0,011092226	0,024061508
C12orf43	protein_coding	ENSG00000157895	-0,182859129	0,011101103	0,024078218
SFMBT2	protein_coding	ENSG00000198879	0,370113005	0,011115558	0,02410702
KLHL2	protein_coding	ENSG00000109466	0,184825438	0,011126246	0,024125099
RFXAP	protein_coding	ENSG00000133111	-0,438767841	0,011125385	0,024125099
IFT43	protein_coding	ENSG00000119650	-0,181711597	0,011175165	0,024228607

PPIP5K2	protein_coding	ENSG00000145725	-0,144751974	0,011188593	0,024255158
ZBED6	protein_coding	ENSG00000257315	-0,547611273	0,011191927	0,024259821
TNRC18P1	processed_pseudogene	ENSG00000249661	1,119455639	0,011198344	0,024271165
SMCO4	protein_coding	ENSG00000166002	-0,279977239	0,011215487	0,024305751
CFP	protein_coding	ENSG00000126759	0,918478071	0,01122678	0,024327656
RNF130	protein_coding	ENSG00000113269	-0,115668059	0,011248306	0,024371725
EEA1	protein_coding	ENSG00000102189	0,15521628	0,011250015	0,024372855
PAXX	protein_coding	ENSG00000148362	-0,185818333	0,011298638	0,024475609
AC023157.3	antisense	ENSG00000276900	-0,311953556	0,011303267	0,024483051
GK5	protein_coding	ENSG00000175066	-0,188800315	0,011315818	0,024507649
MTMR6	protein_coding	ENSG00000139505	0,177175219	0,011318259	0,024510346
SRRM2	protein_coding	ENSG00000167978	-0,199023902	0,011333627	0,024541036
AC120057.3	TEC	ENSG00000279641	1,615918811	0,011345971	0,024565171
CDH23	protein_coding	ENSG00000107736	1,470488748	0,011362575	0,024598523
YTHDC1	protein_coding	ENSG00000083896	0,137965232	0,011368546	0,024608852
LINC01152	lincRNA	ENSG00000256124	0,793270976	0,011376492	0,024623454
KRT8P45	processed_pseudogene	ENSG00000224520	-0,521620571	0,011379344	0,024627028
MED19	protein_coding	ENSG00000156603	-0,17998194	0,011392316	0,0246525
DDT	protein_coding	ENSG00000099977	-0,20857931	0,011410738	0,024689761
DDT	protein_coding	ENSG00000099977	-0,20857931	0,011410738	0,024689761
PLA2G2D	protein_coding	ENSG00000117215	1,455518446	0,011412928	0,024691893
MYO1G	protein_coding	ENSG00000136286	0,986435801	0,011425528	0,024716546
AL360270.3	antisense	ENSG00000273010	0,817226316	0,011445736	0,02475765
PDIK1L	protein_coding	ENSG00000175087	0,180777102	0,011448314	0,024760616
AC107896.1	antisense	ENSG00000267743	1,446780569	0,011466678	0,02479048
AC008035.1	lincRNA	ENSG00000272369	0,627857648	0,011466184	0,02479048
TEX261	protein_coding	ENSG00000144043	-0,161278751	0,011464359	0,02479048
ACSS1	protein_coding	ENSG00000154930	-0,411570684	0,011466957	0,02479048
DNAJB14	protein_coding	ENSG00000164031	-0,219727599	0,011471165	0,024796963
CAGE1	protein_coding	ENSG00000164304	1,102291651	0,011478913	0,024811099
PDLIM1	protein_coding	ENSG00000107438	-0,121662128	0,011480315	0,024811514
IQCD	protein_coding	ENSG00000166578	0,294057538	0,011485178	0,024819407
AC104964.3	sense_overlapping	ENSG00000261451	1,363825694	0,011508991	0,024868248
AXDND1	protein_coding	ENSG00000162779	0,697187622	0,011517418	0,024881933
VAMP2	protein_coding	ENSG00000220205	0,293308866	0,011517751	0,024881933
NFIX	protein_coding	ENSG00000008441	0,209487642	0,011520307	0,024884833
CACNA1S	protein_coding	ENSG00000081248	1,447506081	0,011526522	0,024895636
GAS2L2	protein_coding	ENSG00000270765	1,637048112	0,011539031	0,024920029
SNAPIN	protein_coding	ENSG00000143553	-0,193229322	0,011540602	0,024920798
ITGA7	protein_coding	ENSG00000135424	0,633895755	0,011546802	0,024931562

PRCD	protein_coding	ENSG00000214140	1,533026981	0,011555379	0,024947455
GCSH	protein_coding	ENSG00000140905	-0,641069792	0,011566052	0,02496787
SLA2	protein_coding	ENSG00000101082	1,616078802	0,011578029	0,024991094
PHGDH	protein_coding	ENSG00000092621	0,181495856	0,011588315	0,025010664
NLRP10	protein_coding	ENSG00000182261	-0,315469593	0,011596824	0,025026396
CDC42BPB	protein_coding	ENSG00000198752	-0,206466845	0,011605525	0,025042537
KLRA1P	transcribed_unprocesse	ENSG00000256667	0,441808581	0,01162482	0,025081534
AC005840.2	lincRNA	ENSG00000256433	0,472208738	0,011648169	0,025129267
TNFRSF12A	protein_coding	ENSG00000006327	-0,203056713	0,011650249	0,025131111
AC002350.1	TEC	ENSG00000278993	0,880419352	0,011652037	0,025132325
GMIP	protein_coding	ENSG00000089639	0,250469277	0,011684268	0,025199193
DBNDD2	protein_coding	ENSG00000244274	-0,47657948	0,011691597	0,02521235
AC006160.1	antisense	ENSG00000249502	1,048920136	0,0117005	0,025228896
PKIB	protein_coding	ENSG00000135549	1,174152401	0,011707675	0,025241714
CCDC124	protein_coding	ENSG00000007080	-0,159395683	0,011719762	0,025265117
IL10RB	protein_coding	ENSG00000243646	0,204967803	0,011723491	0,025270499
ALG8	protein_coding	ENSG00000159063	-0,142378935	0,011728843	0,025279379
POLR2M	protein_coding	ENSG00000255529	-0,208936518	0,011755489	0,025334148
HBG2	protein_coding	ENSG00000196565	1,452784896	0,011762063	0,025345652
DMXL1	protein_coding	ENSG00000172869	0,156480554	0,011782686	0,025387424
MTMR1	protein_coding	ENSG00000063601	-0,114866792	0,011801501	0,025425292
EFCAB8	protein_coding	ENSG00000215529	0,759908223	0,011805121	0,02543042
ARL3	protein_coding	ENSG00000138175	-0,180783311	0,011834918	0,025491931
MLC1	protein_coding	ENSG00000100427	1,018871952	0,011836836	0,025493385
LDHC	protein_coding	ENSG00000166796	1,520349611	0,011844194	0,025506553
RDX	protein_coding	ENSG00000137710	-0,174319707	0,011846879	0,025509658
GM2A	protein_coding	ENSG00000196743	-0,103197751	0,01185435	0,025523066
FAM120AOS	protein_coding	ENSG00000188938	0,130139098	0,01185757	0,025527318
TSR3	protein_coding	ENSG00000007520	-0,16351259	0,011859969	0,025529802
CERCAM	protein_coding	ENSG00000167123	-0,169961819	0,011868035	0,025544485
AKT3	protein_coding	ENSG00000117020	-0,152091568	0,011875747	0,025558402
PAK3	protein_coding	ENSG00000077264	0,35677337	0,011898703	0,02560512
ZFP2	protein_coding	ENSG00000198939	1,240993068	0,011900727	0,025606788
AC092168.2	sense_intronic	ENSG00000232034	-1,115722033	0,011902466	0,025607843
FDPS	protein_coding	ENSG00000160752	-0,126937831	0,011922309	0,025647846
PIGBOS1	protein_coding	ENSG00000225973	0,234721287	0,011929001	0,02565955
TM9SF4	protein_coding	ENSG00000101337	-0,134060734	0,011951268	0,025702256
MRPL19	protein_coding	ENSG00000115364	-0,140282269	0,011951361	0,025702256
ATR	protein_coding	ENSG00000175054	-0,116646266	0,011965506	0,025729977
LRRC69	protein_coding	ENSG00000214954	0,583040991	0,01197439	0,025746382

TMEM198B	transcribed_unitary_pse	ENSG00000182796	0,443542706	0,01197742	0,025750197
GTF2IRD1P1	transcribed_unprocesse	ENSG00000230583	1,512259967	0,012009806	0,025817118
AC133065.3	TEC	ENSG00000280153	-0,958172886	0,012034885	0,025868319
AL353625.1	transcribed_processed_	ENSG00000213073	0,879251204	0,012048153	0,025894123
LRRC75B	protein_coding	ENSG00000178026	-0,658935441	0,012062512	0,025922267
MAPK1IP1L	protein_coding	ENSG00000168175	-0,119807696	0,0120708	0,025937361
LDB3	protein_coding	ENSG00000122367	1,381003955	0,012079055	0,025952379
PSMB1	protein_coding	ENSG00000008018	-0,145691171	0,012091753	0,025976941
MT-ATP8	protein_coding	ENSG00000228253	0,586451048	0,012105853	0,026004509
AL121658.1	lincRNA	ENSG00000272716	-0,741332347	0,012131475	0,026056817
AC022001.2	antisense	ENSG00000271716	1,596492573	0,012133531	0,026058504
RASL10A	protein_coding	ENSG00000100276	1,211159648	0,0122068	0,026213115
AC242426.2	antisense	ENSG00000237188	0,412156163	0,012208208	0,026213394
HCN1	protein_coding	ENSG00000164588	1,394250122	0,012217988	0,026231649
SNAP47	protein_coding	ENSG00000143740	-0,128552632	0,012221082	0,026235545
NME1	protein_coding	ENSG00000239672	-0,156387659	0,012228442	0,026245851
CTDP1	protein_coding	ENSG00000060069	-0,222418417	0,012227776	0,026245851
COL6A2	protein_coding	ENSG00000142173	-0,178129059	0,012250593	0,026290642
CUX1	protein_coding	ENSG00000257923	0,149972576	0,012281321	0,026353829
AC074143.1	protein_coding	ENSG00000140939	-0,284735972	0,012293321	0,026376819
MMUT	protein_coding	ENSG00000146085	0,154527676	0,012305298	0,026399755
FAM27C	processed_transcript	ENSG00000231527	1,199439108	0,012315627	0,026418507
ROGDI	protein_coding	ENSG00000067836	-0,373762005	0,012316614	0,026418507
USP6NL	protein_coding	ENSG00000148429	-0,157873705	0,012335576	0,026456412
PHYHD1	protein_coding	ENSG00000175287	1,601179008	0,012339977	0,026463084
HACD2	protein_coding	ENSG00000206527	-0,127635474	0,012359471	0,026502117
RBM44	protein_coding	ENSG00000177483	0,52669141	0,012375361	0,026533416
RPRD1B	protein_coding	ENSG00000101413	0,133270199	0,012426656	0,02664061
C19orf38	protein_coding	ENSG00000214212	0,972280176	0,012434841	0,026653807
DBIL5P	transcribed_unitary_pse	ENSG00000231784	-0,456790038	0,012435411	0,026653807
TMEM158	protein_coding	ENSG00000249992	-0,190535021	0,012442512	0,02666624
AL121917.1	antisense	ENSG00000225806	0,692365282	0,012457245	0,026695026
ATP5F1B	protein_coding	ENSG00000110955	-0,107394327	0,012459207	0,026696442
COPS5	protein_coding	ENSG00000121022	-0,159424773	0,012461536	0,026697532
GHDC	protein_coding	ENSG00000167925	-0,237978683	0,01246232	0,026697532
AL135999.1	antisense	ENSG00000258727	0,590769314	0,012465355	0,026698458
SLC25A3	protein_coding	ENSG00000075415	-0,118542743	0,012464071	0,026698458
TGFB1I1	protein_coding	ENSG00000140682	-0,202858616	0,012473946	0,026714069
ST8SIA5	protein_coding	ENSG00000101638	1,458117111	0,012489713	0,026745043
MTMR2	protein_coding	ENSG00000087053	0,117097714	0,012505868	0,02677684

MICU3	protein_coding	ENSG00000155970	1,306352602	0,012508147	0,026778925
TRIM52-AS1	processed_transcript	ENSG00000248275	-0,24268489	0,012533497	0,026827596
MCOLN3	protein_coding	ENSG00000055732	0,183372013	0,012532495	0,026827596
MTMR14	protein_coding	ENSG00000163719	0,1499491	0,012544937	0,026849281
LZTS1	protein_coding	ENSG00000061337	-1,02636097	0,012550398	0,026858166
AC022098.1	antisense	ENSG00000267169	-0,451867689	0,012565067	0,026886752
RPS18	protein_coding	ENSG00000231500	-0,197961814	0,012583689	0,026923791
LINC00634	transcribed_unitary_pse	ENSG00000205704	1,020888968	0,012625378	0,02701017
RNPEPL1	protein_coding	ENSG00000142327	-0,240284158	0,012630298	0,027017877
TMEM189	protein_coding	ENSG00000240849	-0,219070804	0,012640515	0,027036913
ATG5	protein_coding	ENSG00000057663	-0,139582017	0,012642936	0,027039272
AGAP2	protein_coding	ENSG00000135439	0,469842907	0,012649882	0,027051307
ZNHIT3	protein_coding	ENSG00000273611	-0,196175055	0,012681579	0,027116263
PPP5D1	protein_coding	ENSG00000230510	0,723214859	0,012692222	0,027136191
PTMA	protein_coding	ENSG00000187514	-0,155848536	0,012696731	0,027143001
AC073585.1	transcribed_processed_	ENSG00000255624	0,184137879	0,012699392	0,027145859
ANGEL2	protein_coding	ENSG00000174606	-0,131347791	0,012709276	0,027164157
AC092535.2	processed_pseudogene	ENSG00000251639	-0,45847161	0,01271404	0,027171508
AIP	protein_coding	ENSG00000110711	-0,178208017	0,012718409	0,027178013
NCR3LG1	protein_coding	ENSG00000188211	0,228893476	0,012723521	0,027186103
HERC2P9	transcribed_unprocesse	ENSG00000206149	0,260656205	0,012728204	0,027193277
TSSC2	transcribed_unprocesse	ENSG00000223756	1,372053778	0,012735882	0,027206848
AC048382.5	antisense	ENSG00000275120	1,046869427	0,012758041	0,027251346
C11orf88	protein_coding	ENSG00000183644	1,347283462	0,012768521	0,02727089
RPS15AP36	processed_pseudogene	ENSG00000213013	1,457563792	0,01279298	0,027320284
ZNF154	protein_coding	ENSG00000179909	1,361338434	0,012798875	0,027327184
DOT1L	protein_coding	ENSG00000104885	-0,255782346	0,012798207	0,027327184
NT5DC1	protein_coding	ENSG00000178425	0,178839012	0,012803117	0,027333396
CAPN5	protein_coding	ENSG00000149260	-0,241476931	0,012835827	0,027397524
AC009238.2	processed_pseudogene	ENSG00000233275	0,96836628	0,012846405	0,027414397
AGO1	protein_coding	ENSG00000092847	-0,169641156	0,012864927	0,027451066
CDC5L	protein_coding	ENSG00000096401	-0,134367718	0,012879496	0,027479295
XPNPEP1	protein_coding	ENSG00000108039	-0,099464511	0,012883406	0,027484778
FAM57B	protein_coding	ENSG00000149926	0,778694787	0,012897355	0,027511674
SLC39A13	protein_coding	ENSG00000165915	-0,189283036	0,012899412	0,027513201
LINC01764	lincRNA	ENSG00000267308	1,385480005	0,012909569	0,027532001
AC090844.3	lincRNA	ENSG00000265799	1,321528474	0,012919932	0,027548373
AL157702.2	lincRNA	ENSG00000227482	0,999537861	0,012919459	0,027548373
AC016394.1	antisense	ENSG00000227540	-0,406574775	0,012922484	0,027550951
SLC15A3	protein_coding	ENSG00000110446	1,296926693	0,012925003	0,027553457

KDM8	protein_coding	ENSG00000155666	-0,376205277	0,012927712	0,027556368
AC096667.1	antisense	ENSG00000283839	0,668102834	0,012941617	0,027580274
AC005229.4	antisense	ENSG00000273314	-0,319924729	0,012940954	0,027580274
PLA2G3	protein_coding	ENSG00000100078	1,432193224	0,012945785	0,027586291
FYN	protein_coding	ENSG00000010810	0,130202244	0,012957529	0,027608449
FAM193B	protein_coding	ENSG00000146067	-0,263940941	0,012978667	0,027650614
RNF214	protein_coding	ENSG00000167257	-0,137771756	0,01299936	0,027691824
DDX56	protein_coding	ENSG00000136271	-0,124069523	0,013006555	0,027704274
CDIPTOSP	transcribed_unitary_pse	ENSG00000214725	-0,977219531	0,013036719	0,02776564
MIPEPP3	transcribed_unprocesse	ENSG00000233325	0,662333959	0,013051399	0,027794018
NHLH1	protein_coding	ENSG00000171786	1,366188627	0,013065256	0,027820639
WHAMMP2	transcribed_unprocesse	ENSG00000248334	0,31193731	0,013070191	0,027828258
RNF8	protein_coding	ENSG00000112130	-0,168512251	0,013078108	0,027842225
AGO2	protein_coding	ENSG00000123908	0,170755136	0,013081693	0,027846966
LINC02211	lincRNA	ENSG00000245662	1,545011599	0,013093072	0,027868296
TMEM206	protein_coding	ENSG00000065600	-0,169589383	0,013104025	0,027888715
AC084018.2	lincRNA	ENSG00000274292	0,780346863	0,013110199	0,02789896
ABCA4	protein_coding	ENSG00000198691	0,685548448	0,013121542	0,027920202
LRRCC1	protein_coding	ENSG00000133739	-0,257727614	0,013125873	0,027926519
RUNDC3A-AS1	antisense	ENSG00000267750	1,434672001	0,01313186	0,027936359
GDPD5	protein_coding	ENSG00000158555	0,265856398	0,013134251	0,02793855
IGFN1	protein_coding	ENSG00000163395	0,724238215	0,01315649	0,027982952
JMJD8	protein_coding	ENSG00000161999	-0,167977895	0,013161988	0,027991744
LUC7L2	protein_coding	ENSG00000146963	-0,166161301	0,013223593	0,028117447
AC103810.3	unprocessed_pseudoge	ENSG00000265982	1,091395038	0,013223837	0,028117447
RPP30	protein_coding	ENSG00000148688	-0,147516597	0,013242441	0,028154085
DUSP11	protein_coding	ENSG00000144048	0,210719968	0,013249674	0,028166543
AL391069.2	antisense	ENSG00000237976	0,68345387	0,013254034	0,028172892
ORAI1	protein_coding	ENSG00000276045	0,190958619	0,013264812	0,02819288
SPG11	protein_coding	ENSG00000104133	-0,136547941	0,013275901	0,028212782
LYSMD1	protein_coding	ENSG00000163155	-0,289977098	0,013276927	0,028212782
FOCAD	protein_coding	ENSG00000188352	-0,102179207	0,013306078	0,028271798
ELN-AS1	antisense	ENSG00000232415	0,544289603	0,013314918	0,028287649
TBC1D25	protein_coding	ENSG00000068354	-0,17318612	0,013327058	0,028310509
AC087481.3	sense_intronic	ENSG00000270015	-0,31449031	0,013343647	0,028342813
AC012181.1	sense_intronic	ENSG00000261114	1,161587393	0,013350837	0,028355147
AC016598.2	lincRNA	ENSG00000251205	1,359929286	0,013384697	0,028424118
EIF4G3	protein_coding	ENSG00000075151	0,168207784	0,013399953	0,028453571
VPS35	protein_coding	ENSG00000069329	-0,130630217	0,013407652	0,02846697
DIP2B	protein_coding	ENSG00000066084	-0,170119704	0,013415847	0,028481423

CCDC9	protein_coding	ENSG00000105321	0,219106688	0,013418132	0,028483325
RNASEH1-AS1	antisense	ENSG00000234171	-0,204412597	0,01342205	0,028488693
MED1	protein_coding	ENSG00000125686	0,122088312	0,013441682	0,028527412
AC131238.1	antisense	ENSG00000274859	1,158049881	0,013450568	0,028543316
PLCZ1	protein_coding	ENSG00000139151	0,944966967	0,013459722	0,028559787
BTG3-AS1	processed_transcript	ENSG00000280594	0,626999068	0,013467265	0,028572835
EIF4BP3	processed_pseudogene	ENSG00000224546	-0,297040234	0,01347242	0,028580816
AP000640.2	processed_transcript	ENSG00000255355	1,002258004	0,013481018	0,0285961
EIF2B3	protein_coding	ENSG00000070785	-0,162035537	0,013512755	0,028660456
RAP2C-AS1	antisense	ENSG00000232160	0,668556976	0,013545216	0,028726335
PRPF8	protein_coding	ENSG00000174231	-0,150994356	0,013551206	0,028736065
SPCS1	protein_coding	ENSG00000114902	-0,145616039	0,013553824	0,028738646
Z92544.2	processed_transcript	ENSG00000261659	0,849866235	0,013561377	0,02875169
LINC00475	transcribed_unprocesse	ENSG00000225511	0,557060584	0,013563586	0,028752296
RPL23AP82	transcribed_unprocesse	ENSG00000184319	-0,182298422	0,013564467	0,028752296
NTMT1	protein_coding	ENSG00000148335	-0,16477786	0,01357208	0,028762489
BCL7C	protein_coding	ENSG00000099385	-0,209193963	0,013571451	0,028762489
LY6G6C	protein_coding	ENSG00000204421	1,310456915	0,013577332	0,028770645
STAU1	protein_coding	ENSG00000124214	0,10480265	0,013594843	0,028804776
ZFX	protein_coding	ENSG00000005889	-0,132786079	0,013624512	0,028864657
GATA3	protein_coding	ENSG00000107485	-0,172241368	0,013627098	0,028867154
RAB39B	protein_coding	ENSG00000155961	0,904702464	0,013646423	0,028905105
AC025279.1	processed_transcript	ENSG00000198106	1,337458285	0,01365047	0,028910691
AC119674.1	lincRNA	ENSG00000260971	1,41461655	0,013658514	0,028924742
SH3TC2-DT	lincRNA	ENSG00000250072	-0,255857837	0,013665951	0,028937502
PNKD	protein_coding	ENSG00000127838	-0,191775765	0,013675197	0,028954092
AL033543.1	sense_intronic	ENSG00000279175	-1,245860059	0,013678854	0,028958845
NT5E	protein_coding	ENSG00000135318	0,118878996	0,013688742	0,028976787
AC006504.5	lincRNA	ENSG00000267575	-0,207681643	0,013694832	0,028986688
EXOC6B	protein_coding	ENSG00000144036	0,122213981	0,013696731	0,028987717
METTL26	protein_coding	ENSG00000130731	-0,198833815	0,013701259	0,028993635
AC061975.5	unprocessed_pseudoge	ENSG00000266306	0,453403247	0,013702355	0,028993635
AMZ2	protein_coding	ENSG00000196704	-0,119312602	0,013707191	0,029000876
WDPCP	protein_coding	ENSG00000143951	0,238928903	0,013725116	0,029035806
PIK3C2B	protein_coding	ENSG00000133056	-0,315650584	0,013728868	0,029040747
PPP1R21	protein_coding	ENSG00000162869	0,161261722	0,013733782	0,029048146
MEX3B	protein_coding	ENSG00000183496	-0,266743698	0,013738884	0,029055941
PFDN5	protein_coding	ENSG00000123349	-0,161669974	0,013741324	0,029058105
ESR2	protein_coding	ENSG00000140009	1,311512184	0,013743227	0,029059133
AC096720.2	TEC	ENSG00000279464	-0,747210968	0,013751352	0,029073315

ITSN1	protein_coding	ENSG00000205726	0,116571186	0,01377028	0,029110332
DNAJC12	protein_coding	ENSG00000108176	1,210969441	0,013781462	0,029130968
G6PD	protein_coding	ENSG00000160211	0,142692724	0,013787584	0,029140906
AC005831.1	TEC	ENSG00000280202	-0,475573653	0,013794444	0,029152401
ZNF574	protein_coding	ENSG00000105732	-0,219975797	0,013806557	0,029174994
AC010761.3	antisense	ENSG00000265073	1,168202407	0,013835063	0,029232217
ACO1	protein_coding	ENSG00000122729	-0,104598072	0,013854456	0,029267163
NUP58	protein_coding	ENSG00000139496	-0,13016405	0,013853495	0,029267163
AC004129.2	processed_pseudogene	ENSG00000234406	0,896058963	0,013875669	0,029308956
ECM1	protein_coding	ENSG00000143369	0,163702948	0,013879573	0,029314184
PLCG2	protein_coding	ENSG00000197943	0,210197132	0,013882582	0,02931752
ABLIM3	protein_coding	ENSG00000173210	0,208020429	0,013926881	0,029408044
L3HYPDH	protein_coding	ENSG00000126790	-0,173587544	0,013933499	0,02941899
MIR3142HG	lincRNA	ENSG00000253522	1,436086091	0,013940424	0,029430582
AC084757.2	lincRNA	ENSG00000259469	-0,889032399	0,013943914	0,02943189
FAM238C	transcribed_unprocesse	ENSG00000283709	1,513678966	0,013943126	0,02943189
HOXB3	protein_coding	ENSG00000120093	0,257222042	0,013975557	0,029495645
AC087239.1	lincRNA	ENSG00000278743	-0,848636498	0,013982223	0,029506677
RPSAP9	processed_pseudogene	ENSG00000234618	0,822909958	0,014025876	0,029595752
UBE2V2	protein_coding	ENSG00000169139	-0,185083607	0,014041176	0,029623752
AC022400.9	TEC	ENSG00000279689	-1,057469985	0,014042034	0,029623752
ITM2B	protein_coding	ENSG00000136156	0,135182897	0,014066714	0,029672766
PLIN1	protein_coding	ENSG00000166819	0,950536338	0,014070685	0,02967809
C17orf82	lincRNA	ENSG00000187013	0,637705901	0,014077616	0,029689656
DNAH11	protein_coding	ENSG00000105877	0,497267178	0,014108799	0,029750402
KHNYN	protein_coding	ENSG00000100441	-0,169381161	0,01410932	0,029750402
EPG5	protein_coding	ENSG00000152223	-0,135938574	0,014126599	0,029783774
RNF217	protein_coding	ENSG00000146373	0,17439389	0,014166384	0,029864584
CNPY2	protein_coding	ENSG00000257727	-0,245312301	0,014177965	0,029885926
DDX52	protein_coding	ENSG00000278053	-0,107341684	0,014182527	0,029892469
FABP5	protein_coding	ENSG00000164687	-0,189151559	0,014191209	0,029907695
AL591806.1	antisense	ENSG00000228917	1,304744969	0,014222758	0,029971105
FLG	protein_coding	ENSG00000143631	-0,414255594	0,014300276	0,030131359
AL845472.2	TEC	ENSG00000279561	-0,669442988	0,014316976	0,030163449
C15orf48	protein_coding	ENSG00000166920	0,736381915	0,014330942	0,030189771
CDC42BPG	protein_coding	ENSG00000171219	0,373000863	0,014340029	0,030205813
UMODL1	protein_coding	ENSG00000177398	1,266670796	0,014342952	0,030208867
LINC00519	lincRNA	ENSG00000258955	1,499280261	0,01434942	0,030219385
PLEKHA7	protein_coding	ENSG00000166689	0,443704355	0,014383684	0,030285326
UBE2F	protein_coding	ENSG00000184182	0,233782601	0,014382736	0,030285326

AC079322.1	lincRNA	ENSG00000261187	1,26060587	0,014433611	0,03038733
AP002986.1	lincRNA	ENSG00000175773	1,025392278	0,014533577	0,030594649
BORCS5	protein_coding	ENSG00000165714	-0,197409736	0,014546342	0,030618377
AC113410.3	TEC	ENSG00000279691	-0,972991662	0,014554414	0,030632225
NCLN	protein_coding	ENSG00000125912	-0,178429652	0,014557995	0,030636617
AC112907.3	antisense	ENSG00000263826	0,907843665	0,014616196	0,030755944
PLAGL2	protein_coding	ENSG00000126003	-0,188960151	0,014633167	0,030788496
ZNF182	protein_coding	ENSG00000147118	0,214521578	0,014643604	0,030807295
SNHG20	sense_overlapping	ENSG00000234912	-0,31230791	0,014682896	0,030886788
ELOVL3	protein_coding	ENSG00000119915	-0,67905912	0,01469645	0,030912131
AL078644.2	lincRNA	ENSG00000273004	0,873988472	0,014698322	0,030912898
ZNF17	protein_coding	ENSG00000186272	-0,267232048	0,014710556	0,030935456
ACY3	protein_coding	ENSG00000132744	1,285922805	0,014746798	0,031008491
AC067751.1	antisense	ENSG00000238280	1,277346434	0,014766959	0,031047701
AKR1A1	protein_coding	ENSG00000117448	-0,141997326	0,014775772	0,031063047
SMIM6	protein_coding	ENSG00000259120	0,530396697	0,014777599	0,031063704
AC000403.1	lincRNA	ENSG00000278727	1,184922485	0,014787305	0,031080921
AC112184.1	processed_pseudogene	ENSG00000251050	1,237494086	0,014797243	0,031096395
PRXL2C	protein_coding	ENSG00000158122	-0,160699306	0,014797699	0,031096395
VPS35L	protein_coding	ENSG00000103544	0,123047262	0,014811453	0,031122109
UBL7	protein_coding	ENSG00000138629	-0,142064061	0,014819303	0,031135414
VPS51	protein_coding	ENSG00000149823	-0,163368195	0,01484033	0,031176396
AC005042.1	processed_pseudogene	ENSG00000227331	-0,825683303	0,014849157	0,031191746
INKA1	protein_coding	ENSG00000185614	0,538126382	0,014861236	0,031213923
TANC1	protein_coding	ENSG00000115183	0,155548871	0,014872608	0,031234608
ALDH4A1	protein_coding	ENSG00000159423	-0,247257116	0,014893451	0,031275181
AC104248.1	antisense	ENSG00000253796	0,855280766	0,014898791	0,03128319
AC013652.1	lincRNA	ENSG00000259345	0,444491117	0,014907759	0,031298816
BTBD10P2	processed_pseudogene	ENSG00000219433	-1,364966745	0,014968809	0,031411309
FBXO47	protein_coding	ENSG00000204952	0,860236247	0,014969957	0,031411309
ZNF778	protein_coding	ENSG00000170100	0,160601221	0,014970529	0,031411309
TSPAN4	protein_coding	ENSG00000214063	-0,178383836	0,014966638	0,031411309
C3orf18	protein_coding	ENSG00000088543	-0,236752762	0,014969283	0,031411309
AC022400.8	TEC	ENSG00000279088	-0,297803145	0,014967095	0,031411309
CPQ	protein_coding	ENSG00000104324	0,441054344	0,015016451	0,031504441
FLOT1	protein_coding	ENSG00000137312	-0,106740396	0,015025774	0,031517552
UBE3D	protein_coding	ENSG00000118420	-0,223944712	0,015025445	0,031517552
FAM32A	protein_coding	ENSG00000105058	-0,109792791	0,01503693	0,031537726
TMPRSS11F	protein_coding	ENSG00000198092	1,312968176	0,015052182	0,031566489
PI4KA	protein_coding	ENSG00000241973	0,148701402	0,015062285	0,031584445

CSRP1	protein_coding	ENSG00000159176	-0,10407381	0,015063971	0,031584752
RRN3P1	transcribed_unprocesse	ENSG00000248124	0,388460199	0,015068304	0,031590608
AMIGO1	protein_coding	ENSG00000181754	-0,800243784	0,015101015	0,031655949
TMEM42	protein_coding	ENSG00000169964	-0,206958392	0,015110132	0,031671825
PRPF39	protein_coding	ENSG00000185246	-0,175161317	0,015122773	0,031695083
PAX9	protein_coding	ENSG00000198807	-0,428164164	0,01512721	0,031701142
ERO1A	protein_coding	ENSG00000197930	-0,159826599	0,015131591	0,031707084
NRBP2	protein_coding	ENSG00000185189	-0,274688066	0,015143309	0,031725155
BBS5	protein_coding	ENSG00000163093	-0,500496927	0,01514223	0,031725155
FDPSP8	processed_pseudogene	ENSG00000224763	-0,992437822	0,015163919	0,031763948
TUBBP1	transcribed_processed_	ENSG00000127589	-0,48213217	0,015164923	0,031763948
SLC25A53	protein_coding	ENSG00000269743	-0,3363426	0,015178914	0,031790005
AC069185.1	lincRNA	ENSG00000255046	0,712250762	0,015198194	0,031827135
RBBP5	protein_coding	ENSG00000117222	0,102902337	0,015228478	0,031887297
NKX2-5	protein_coding	ENSG00000183072	0,482058073	0,015242757	0,031913939
MOCOS	protein_coding	ENSG00000075643	0,397148894	0,015260743	0,031948334
ARL10	protein_coding	ENSG00000175414	-0,425147116	0,015275861	0,03197672
WDYHV1	protein_coding	ENSG00000156795	-0,209091958	0,015288987	0,032000931
TMEM8A	protein_coding	ENSG00000129925	0,209598512	0,015325634	0,032074363
LINC01934	lincRNA	ENSG00000234663	1,251907202	0,015335839	0,032092445
PITX3	protein_coding	ENSG00000107859	-0,569559376	0,0153522	0,032123406
MATK	protein_coding	ENSG00000007264	-0,466074183	0,015363803	0,032144404
AC005180.1	lincRNA	ENSG00000267405	0,620059798	0,01537059	0,032155325
RUNX3	protein_coding	ENSG00000020633	-0,251530027	0,01537445	0,03216012
CRAMP1	protein_coding	ENSG00000007545	0,281312112	0,015396463	0,032202881
RC3H2	protein_coding	ENSG00000056586	0,127500407	0,015418619	0,032242773
PI4KB	protein_coding	ENSG00000143393	0,123976508	0,01541868	0,032242773
HLA-DRA	protein_coding	ENSG00000204287	-0,19942504	0,015433794	0,03227109
TMC8	protein_coding	ENSG00000167895	0,670681752	0,015447928	0,032297351
SEMA3E	protein_coding	ENSG00000170381	0,375606605	0,015460443	0,032320222
RPL26L1	protein_coding	ENSG00000037241	-0,175628862	0,01547451	0,03234633
VTA1	protein_coding	ENSG00000009844	-0,156705194	0,015492253	0,032380119
TCEAL1	protein_coding	ENSG00000172465	-0,214559646	0,015495433	0,032383465
NR1D2	protein_coding	ENSG00000174738	0,14256607	0,01551386	0,032418672
AC026471.3	antisense	ENSG00000260740	1,352427764	0,015517133	0,032422207
ITPKA	protein_coding	ENSG00000137825	-0,374938557	0,015523064	0,032431298
TMEM50A	protein_coding	ENSG00000183726	-0,129332697	0,015545862	0,032475619
AC126474.2	TEC	ENSG00000280088	-0,300160757	0,015557832	0,032497315
AC113404.3	translated_processed_p	ENSG00000254893	0,583240058	0,015570997	0,032521503
LHX2	protein_coding	ENSG00000106689	-0,497851493	0,015596833	0,032572146

TLR10	protein_coding	ENSG00000174123	1,448202007	0,015635219	0,032648986
PGM5P2	transcribed_unprocesse	ENSG00000277778	0,616534593	0,015638603	0,032652727
LINC00189	sense_overlapping	ENSG00000215533	1,221375682	0,015652049	0,032677476
AC073332.1	antisense	ENSG00000237773	0,636620111	0,015691843	0,032757222
AC000068.1	antisense	ENSG00000185065	-0,923842659	0,015712862	0,03279776
TMEM236	protein_coding	ENSG00000148483	0,610966439	0,015717733	0,032802684
TTC14	protein_coding	ENSG00000163728	-0,206809772	0,015718419	0,032802684
SMAD5-AS1	antisense	ENSG00000164621	1,077223703	0,015724332	0,032811684
LINC02141	lincRNA	ENSG00000261807	1,264237608	0,015729438	0,032817894
IMMP2L	protein_coding	ENSG00000184903	0,240705147	0,015730508	0,032817894
RNF148	protein_coding	ENSG00000235631	1,158945234	0,015743081	0,032840784
AC092171.5	lincRNA	ENSG00000273084	0,553561724	0,015750529	0,03285298
WWOX	protein_coding	ENSG00000186153	-0,19300609	0,015759441	0,032868225
U91328.1	lincRNA	ENSG00000272462	-0,371160058	0,015773019	0,032893199
CHSY3	protein_coding	ENSG00000198108	-0,309319133	0,015810337	0,032967671
AIFM3	protein_coding	ENSG00000183773	0,565540691	0,015818217	0,03298075
POC5	protein_coding	ENSG00000152359	0,150986402	0,015822125	0,032985544
CHAT	protein_coding	ENSG00000070748	1,350271344	0,015836668	0,033012507
SEC61A2	protein_coding	ENSG00000065665	0,231404983	0,015847361	0,03303144
GAL3ST1	protein_coding	ENSG00000128242	0,977324926	0,01585668	0,033045897
TCF4	protein_coding	ENSG00000196628	0,250062502	0,015859131	0,033045897
TTC3	protein_coding	ENSG00000182670	0,118480139	0,015858085	0,033045897
ARHGAP40	protein_coding	ENSG00000124143	0,246877585	0,015872544	0,033067128
FBXO41	protein_coding	ENSG00000163013	-0,312367605	0,015871589	0,033067128
SMIM10L1	protein_coding	ENSG00000256537	-0,144419449	0,01588074	0,033080842
MPHOSPH6	protein_coding	ENSG00000135698	-0,19449293	0,015885859	0,033088146
AC008269.1	lincRNA	ENSG00000229321	0,927020479	0,015887643	0,033088502
AC091564.2	antisense	ENSG00000254400	1,428221341	0,015927236	0,033162991
AL031727.1	processed_pseudogene	ENSG00000226396	-0,412955578	0,015928261	0,033162991
SLC3A1	protein_coding	ENSG00000138079	0,331445518	0,015925955	0,033162991
AC005392.1	lincRNA	ENSG00000227606	0,745713928	0,015938418	0,03317811
MARCH8	protein_coding	ENSG00000165406	-0,157506472	0,015938757	0,03317811
AL022328.2	antisense	ENSG00000273137	1,267291697	0,015984117	0,033269153
EIF5A	protein_coding	ENSG00000132507	-0,131155861	0,015994787	0,033287983
ADAMTS7P4	transcribed_unprocesse	ENSG00000218052	-0,527294374	0,015998735	0,033292822
HR	protein_coding	ENSG00000168453	1,202756301	0,016002831	0,033297968
RPL8	protein_coding	ENSG00000161016	-0,177972869	0,016006867	0,033302988
ZNF300	protein_coding	ENSG00000145908	0,234391947	0,016030998	0,033349809
OCIAD1	protein_coding	ENSG00000109180	0,127519722	0,016051278	0,033388612
PSMC2	protein_coding	ENSG00000161057	-0,139210882	0,016100767	0,033488158

FGF12	protein_coding	ENSG00000114279	0,186794355	0,016118378	0,033521388
RNF217-AS1	antisense	ENSG00000236548	0,899918908	0,016121025	0,033523495
AL136126.1	processed_pseudogene	ENSG00000241255	0,791852502	0,016146731	0,033573545
LRP11	protein_coding	ENSG00000120256	0,132149881	0,016164998	0,03360812
TICAM2	protein_coding	ENSG00000243414	-0,69809461	0,016178794	0,033633395
POU2F1	protein_coding	ENSG00000143190	-0,216947047	0,016191235	0,033655845
DPCD	protein_coding	ENSG00000166171	-0,191512801	0,016229451	0,033731864
RNF166	protein_coding	ENSG00000158717	-0,15690998	0,016243984	0,03375865
KNDC1	protein_coding	ENSG00000171798	-0,427411763	0,016259748	0,033787988
AC007216.4	antisense	ENSG00000263307	1,17035318	0,01628484	0,0338367
SLC16A13	protein_coding	ENSG00000174327	0,473757894	0,016299473	0,033863674
PSMD4	protein_coding	ENSG00000159352	-0,106978732	0,016305176	0,033872092
KIAA1671	protein_coding	ENSG00000197077	-0,211631795	0,016381261	0,034026704
AL359265.1	unprocessed_pseudoge	ENSG00000227815	1,099017686	0,016390575	0,034042602
PPP1R3E	protein_coding	ENSG00000235194	-0,34498556	0,01639875	0,034056133
PHOSPHO1	protein_coding	ENSG00000173868	0,83217759	0,016403092	0,034061701
CCT6P3	transcribed_unprocesse	ENSG00000234585	0,499973221	0,016414607	0,034082162
PNPO	protein_coding	ENSG00000108439	0,127074539	0,016417617	0,03408496
MIRLET7BHG	lincRNA	ENSG00000197182	-0,384056956	0,01646383	0,034177446
PDXK	protein_coding	ENSG00000160209	0,141048697	0,016532747	0,034317037
PDXK	protein_coding	ENSG00000160209	0,141048697	0,016532747	0,034317037
AL136038.4	processed_pseudogene	ENSG00000270878	-0,866969359	0,0165615	0,034373241
PPP3CB	protein_coding	ENSG00000107758	-0,145070151	0,016625525	0,034502633
AL683807.1	lincRNA	ENSG00000223511	0,621922876	0,016665772	0,034582658
SNX24	protein_coding	ENSG00000064652	-0,143601532	0,016667738	0,034583239
AL360091.1	sense_intronic	ENSG00000229832	-1,160447191	0,016681738	0,034608785
PSMD3	protein_coding	ENSG00000108344	-0,101663919	0,016698307	0,034639656
DDRGK1	protein_coding	ENSG00000198171	0,178632392	0,016707791	0,034655824
IMPA1P1	transcribed_unprocesse	ENSG00000251521	1,316229901	0,016763229	0,034767299
BNIP3L	protein_coding	ENSG00000104765	-0,15668559	0,016774129	0,034786388
SAP130	protein_coding	ENSG00000136715	-0,160854646	0,016794047	0,034824175
MMP1	protein_coding	ENSG00000196611	0,562462817	0,016804442	0,034842178
ATP6V1G2	protein_coding	ENSG00000213760	0,556329841	0,016806127	0,034842178
AC092835.1	protein_coding	ENSG00000233757	-0,36148806	0,016819291	0,034865946
ALOX15P1	transcribed_unprocesse	ENSG00000274114	1,093063669	0,016876644	0,0349813
M1AP	protein_coding	ENSG00000159374	-0,149511574	0,016916436	0,035060236
LIAS	protein_coding	ENSG00000121897	-0,201810032	0,016931198	0,035087286
LINC01273	lincRNA	ENSG00000231742	0,596169288	0,01693392	0,035089381
LAMB2	protein_coding	ENSG00000172037	0,207951745	0,016936326	0,035090821
STAG3L3	transcribed_unprocesse	ENSG00000174353	-0,362210252	0,016955834	0,035127692

DMXL2	protein_coding	ENSG00000104093	0,144237056	0,016981119	0,035172969
PGM2	protein_coding	ENSG00000169299	0,131918205	0,016979612	0,035172969
SMTN	protein_coding	ENSG00000183963	0,181543211	0,01699012	0,035184507
CMTR1	protein_coding	ENSG00000137200	-0,123941833	0,016990111	0,035184507
AL499616.1	antisense	ENSG00000232892	1,020036737	0,017014614	0,035231675
AP000442.1	lincRNA	ENSG00000255008	0,631212891	0,017028424	0,035256711
LINC00322	lincRNA	ENSG00000237864	1,034022995	0,017033571	0,035263807
AL358852.1	antisense	ENSG00000278899	0,928567776	0,017067681	0,035327292
UBE2A	protein_coding	ENSG00000077721	0,116702918	0,017067449	0,035327292
DARS	protein_coding	ENSG00000115866	-0,127368088	0,017086118	0,035361886
KDELC1	protein_coding	ENSG00000134901	-0,172535806	0,017092321	0,035371153
USP10	protein_coding	ENSG00000103194	-0,091936126	0,017113782	0,035411993
TSPAN15	protein_coding	ENSG00000099282	-0,201011998	0,017123321	0,035428156
FAM86B1	protein_coding	ENSG00000186523	0,378900133	0,017137831	0,035454601
AC092757.2	sense_overlapping	ENSG00000259732	-1,210034204	0,017165617	0,035508502
AC124045.1	lincRNA	ENSG00000272077	0,382294014	0,017174563	0,035519844
TERC	lincRNA	ENSG00000270141	-1,156485452	0,017174557	0,035519844
AC008467.1	lincRNA	ENSG00000249476	0,899881237	0,017183827	0,03553542
SHB	protein_coding	ENSG00000107338	0,259000953	0,017194357	0,035553609
AP002992.1	antisense	ENSG00000255236	0,968303725	0,017242661	0,035649897
CALML6	protein_coding	ENSG00000169885	1,218262779	0,017265156	0,035692108
HMG20A	protein_coding	ENSG00000140382	-0,096026147	0,017268298	0,035692108
FMNL2	protein_coding	ENSG00000157827	-0,135211505	0,017267965	0,035692108
CYTH3	protein_coding	ENSG00000008256	0,129001595	0,017291899	0,035737287
SNORD99	snoRNA	ENSG00000221539	-0,689995219	0,01729928	0,035748941
TMEM147	protein_coding	ENSG00000105677	-0,158791219	0,017316979	0,035781911
CRKL	protein_coding	ENSG00000099942	-0,093386015	0,017354031	0,035854858
DAXX	protein_coding	ENSG00000204209	-0,090414385	0,017357924	0,035859289
AP002761.3	antisense	ENSG00000257038	0,503653825	0,01740816	0,035959449
KCNMB4	protein_coding	ENSG00000135643	-0,493798886	0,017412581	0,035964958
AL365184.2	lincRNA	ENSG00000230015	1,295581161	0,017418817	0,035974216
PIEZO1	protein_coding	ENSG00000103335	0,312556014	0,017426512	0,035986485
AMZ1	protein_coding	ENSG00000174945	1,211985139	0,017442171	0,036015194
PRSS53	protein_coding	ENSG00000151006	-0,720516975	0,017467372	0,0360636
DZANK1	protein_coding	ENSG00000089091	0,462464967	0,017493576	0,036114066
NFAM1	protein_coding	ENSG00000235568	1,339089789	0,017509109	0,036142494
TRMT6	protein_coding	ENSG00000089195	0,157146605	0,0175225	0,036166497
CAPN3	protein_coding	ENSG00000092529	1,207155891	0,017594193	0,036310817
ETS1-AS1	antisense	ENSG00000254588	1,381214494	0,01760998	0,036339742
MAP1B	protein_coding	ENSG00000131711	0,169164502	0,017615858	0,036348215

AC009303.4	TEC	ENSG00000279227	0,504196271	0,017630097	0,036373936
AL121768.1	protein_coding	ENSG00000165521	-0,281501404	0,017635471	0,036381364
SNORD116-6	snoRNA	ENSG00000207442	1,034608747	0,017641879	0,036390922
TMEM169	protein_coding	ENSG00000163449	0,718200511	0,017670633	0,036446569
MIR646HG	lincRNA	ENSG00000228340	0,631410639	0,017684699	0,036459116
ANKFN1	protein_coding	ENSG00000153930	0,694815096	0,017685605	0,036459116
TM4SF19	protein_coding	ENSG00000145107	0,476756205	0,017681213	0,036459116
PPP3CA	protein_coding	ENSG00000138814	0,13594199	0,017681946	0,036459116
FER1L4	transcribed_unitary_pse	ENSG00000088340	0,688055316	0,017682496	0,036459116
SNORA23	snoRNA	ENSG00000201998	-0,25292007	0,01769846	0,036481951
LBX2-AS1	antisense	ENSG00000257702	0,312398271	0,017704324	0,036490372
HGD	protein_coding	ENSG00000113924	0,970777586	0,01770958	0,036494354
PYGB	protein_coding	ENSG00000100994	0,144340792	0,017711594	0,036494354
PRPF6	protein_coding	ENSG00000101161	-0,107144019	0,017710837	0,036494354
SEPHS2	protein_coding	ENSG00000179918	0,150688272	0,017714887	0,036497471
NSDHL	protein_coding	ENSG00000147383	0,129309158	0,017719993	0,036504324
RASA2	protein_coding	ENSG00000155903	-0,158219338	0,017733501	0,036528482
KALRN	protein_coding	ENSG00000160145	0,588079543	0,017783504	0,036624125
MAST2	protein_coding	ENSG00000086015	-0,153012949	0,017792424	0,036638816
TRMT1L	protein_coding	ENSG00000121486	0,13015877	0,017796516	0,036643564
RBFOX2	protein_coding	ENSG00000100320	0,079083472	0,017807779	0,036663075
FCHO1	protein_coding	ENSG00000130475	-0,324531628	0,017816043	0,036676408
RGS14	protein_coding	ENSG00000169220	0,261671355	0,017836024	0,036713854
GOLGA8N	protein_coding	ENSG00000232653	1,26693067	0,017866218	0,036768627
SMPDL3B	protein_coding	ENSG00000130768	0,751703462	0,017899585	0,0368336
EPS15L1	protein_coding	ENSG00000127527	0,112275865	0,017902938	0,036836803
MIR4453HG	bidirectional_promoter_	ENSG00000268471	-0,504524004	0,01792951	0,036884078
ZNF236	protein_coding	ENSG00000130856	-0,189765981	0,017928507	0,036884078
AP002387.2	antisense	ENSG00000254682	-0,4390701	0,017952734	0,036928149
NBPF10	protein_coding	ENSG00000271425	0,335996418	0,017970296	0,036960566
OSBPL6	protein_coding	ENSG00000079156	0,139890758	0,017980285	0,036977404
HAS2	protein_coding	ENSG00000170961	1,065741583	0,017992149	0,036998093
GTF2I	protein_coding	ENSG00000263001	-0,118717865	0,018015	0,037041368
CSDE1	protein_coding	ENSG00000009307	0,094828825	0,018019726	0,037047373
RNASEK	protein_coding	ENSG00000219200	0,486429676	0,018027624	0,037059894
AC073052.1	processed_pseudogene	ENSG00000228446	-0,667357115	0,018054958	0,037112365
CEP131	protein_coding	ENSG00000141577	-0,232473574	0,018105842	0,037213229
DDX5	protein_coding	ENSG00000108654	0,09252126	0,018125295	0,037249478
AC007923.4	lincRNA	ENSG00000266877	1,36094505	0,018138179	0,037272222
GATA4	protein_coding	ENSG00000136574	-0,204702664	0,018166529	0,037326739

AC016571.1	sense_overlapping	ENSG00000251405	1,150317955	0,018178657	0,037347917
AP003068.2	antisense	ENSG00000254614	0,286805782	0,018202286	0,037392718
PDE8A	protein_coding	ENSG00000073417	-0,12360461	0,018223439	0,037432422
TSPOAP1-AS1	antisense	ENSG00000265148	0,897153206	0,018241434	0,037465633
APBA2	protein_coding	ENSG00000034053	-0,164252392	0,018261443	0,037502974
U2AF1L4	protein_coding	ENSG00000161265	-0,637705931	0,018275033	0,037527125
PPFIA3	protein_coding	ENSG00000177380	-0,224657625	0,018287507	0,037548981
VSTM2L	protein_coding	ENSG00000132821	0,25509054	0,018336476	0,037645758
GPC1	protein_coding	ENSG00000063660	0,219044342	0,018345227	0,037659955
TADA3	protein_coding	ENSG00000171148	0,164028709	0,018354169	0,03767454
KREMEN2	protein_coding	ENSG00000131650	0,304101427	0,018363077	0,037689054
KAT6A	protein_coding	ENSG00000083168	-0,158332109	0,018397551	0,037756031
AP006296.1	processed_pseudogene	ENSG00000257043	-1,235161907	0,018406455	0,037770525
EPB41L2	protein_coding	ENSG00000079819	-0,10593893	0,018411951	0,037778022
NCKAP5	protein_coding	ENSG00000176771	1,098273346	0,018417113	0,037784834
TGIF1	protein_coding	ENSG00000177426	0,17415686	0,018431193	0,037809939
TRAPPC1	protein_coding	ENSG00000170043	-0,13312591	0,018445455	0,037835413
AC138207.6	transcribed_unprocesse	ENSG00000265798	1,052443376	0,018461033	0,037863579
CD74	protein_coding	ENSG00000019582	0,114801567	0,018467434	0,03787292
IK	protein_coding	ENSG00000113141	-0,122733243	0,018471368	0,0378772
AC060780.1	lincRNA	ENSG00000267002	0,352878232	0,018487753	0,03790701
LPAR5	protein_coding	ENSG00000184574	1,108301151	0,018493678	0,037915367
IER3IP1	protein_coding	ENSG00000134049	-0,237797068	0,018500763	0,037926102
AC108010.1	processed_transcript	ENSG00000242588	0,688315459	0,018554513	0,038032488
FAM122C	protein_coding	ENSG00000156500	0,342804058	0,018565203	0,038050596
LRRC24	protein_coding	ENSG00000254402	0,579660562	0,018587734	0,038092969
ARPC4	protein_coding	ENSG00000241553	-0,180483073	0,018593245	0,038100455
SPRTN	protein_coding	ENSG00000010072	0,148421968	0,018632029	0,038172303
YRDC	protein_coding	ENSG00000196449	-0,137939883	0,018630471	0,038172303
AL139220.2	lincRNA	ENSG00000230615	-0,249810631	0,018636921	0,038178511
C12orf76	protein_coding	ENSG00000174456	-0,235479514	0,01864014	0,038181292
SUGT1P4-STRA6LP	processed_transcript	ENSG00000254876	0,467151236	0,018642342	0,038181988
TMEM70	protein_coding	ENSG00000175606	-0,178159594	0,018645584	0,038184815
AC005050.1	lincRNA	ENSG00000267052	1,0050472	0,018653092	0,038196378
DENND5B-AS1	antisense	ENSG00000255867	0,811780744	0,018718275	0,038326028
FBXW4P1	processed_pseudogene	ENSG00000230701	-0,848953538	0,018720345	0,03832644
LEPR	protein_coding	ENSG00000116678	0,203617461	0,018752642	0,03838873
AC009283.1	lincRNA	ENSG00000273576	0,667439981	0,018788741	0,038454951
S1PR2	protein_coding	ENSG00000267534	0,269298471	0,018787426	0,038454951
AP003721.1	antisense	ENSG00000256196	1,308114819	0,018800385	0,038474944

MTHFSD	protein_coding	ENSG00000103248	-0,169774433	0,018803109	0,03847668
NDUFC1	protein_coding	ENSG00000109390	-0,186616741	0,018862707	0,038594785
AP001107.6	antisense	ENSG00000254756	1,097380858	0,018891143	0,038649111
AC022413.1	TEC	ENSG00000280161	-0,594618337	0,018918024	0,038700246
AC131571.1	antisense	ENSG00000228061	1,090176999	0,018920347	0,038700295
AC093567.1	antisense	ENSG00000267764	0,746688741	0,018921821	0,038700295
FAM89B	protein_coding	ENSG00000176973	0,401793446	0,018936674	0,03872681
GALNS	protein_coding	ENSG00000141012	-0,154232075	0,018955669	0,038761792
GPR135	protein_coding	ENSG00000181619	0,342146484	0,018960165	0,038767121
SEPT5	protein_coding	ENSG00000184702	-0,242568864	0,018969598	0,03878254
SLC25A25-AS1	antisense	ENSG00000234771	0,440683323	0,018984353	0,038804972
GDAP2	protein_coding	ENSG00000196505	0,115455946	0,018982559	0,038804972
LINC00174	processed_transcript	ENSG00000179406	0,477418768	0,019024825	0,038883823
CLEC9A	protein_coding	ENSG00000197992	1,143727492	0,019074839	0,038982159
AL008721.2	sense_intronic	ENSG00000272977	0,551935672	0,019090709	0,039010704
AL358790.1	antisense	ENSG00000282772	-0,956691867	0,019130042	0,039087186
CTXND1	protein_coding	ENSG00000259417	0,846773591	0,019154398	0,039129154
ADARB1	protein_coding	ENSG00000197381	0,13987688	0,019153801	0,039129154
ABCD4	protein_coding	ENSG00000119688	0,184555005	0,019157751	0,039132105
SYS1	protein_coding	ENSG00000204070	-0,1468148	0,019162758	0,039138435
POMT2	protein_coding	ENSG00000009830	-0,158767103	0,019217785	0,039246915
FAM185A	protein_coding	ENSG00000222011	-0,377670559	0,01924645	0,039301543
LRRC1	protein_coding	ENSG00000137269	-0,151946366	0,019273327	0,039349071
NENF	protein_coding	ENSG00000117691	-0,168925348	0,019273562	0,039349071
AURKAIP1	protein_coding	ENSG00000175756	-0,21452129	0,019278673	0,039355588
KCNG3	protein_coding	ENSG00000171126	0,397198121	0,019288458	0,039371643
CNN3	protein_coding	ENSG00000117519	0,132161913	0,019299998	0,039391279
Z97989.1	antisense	ENSG00000255389	0,730356715	0,019332635	0,039453965
TBCK	protein_coding	ENSG00000145348	0,204171432	0,019334653	0,039454157
TMEM116	protein_coding	ENSG00000198270	0,213301601	0,019342773	0,039466801
TP53TG1	lincRNA	ENSG00000182165	0,307018157	0,019352913	0,039479634
VAMP8	protein_coding	ENSG00000118640	0,830516612	0,019352652	0,039479634
CEMIP2	protein_coding	ENSG00000135048	-0,110399929	0,019365398	0,039501174
EIF5B	protein_coding	ENSG00000158417	-0,190794368	0,019401582	0,039570788
CHST4	protein_coding	ENSG00000140835	-0,745997265	0,019403385	0,039570788
ZNF10	protein_coding	ENSG00000256223	0,251321331	0,019407281	0,039574799
HYAL3	protein_coding	ENSG00000186792	0,352496449	0,019416312	0,039587602
RALGPS1	protein_coding	ENSG00000136828	-0,262813858	0,01941742	0,039587602
AC012213.2	lincRNA	ENSG00000261670	0,953086403	0,019425253	0,039599635
ZNF585B	protein_coding	ENSG00000245680	0,22538244	0,019445666	0,03963731

YAE1	protein_coding	ENSG00000241127	0,214954117	0,019454774	0,039651934
ZNF7	protein_coding	ENSG00000147789	-0,12324572	0,019461474	0,039661649
MYT1	protein_coding	ENSG00000196132	-0,475182168	0,019533183	0,039803834
KRT79	protein_coding	ENSG00000185640	1,29076033	0,019554907	0,039844142
GPALPP1	protein_coding	ENSG00000133114	0,129347518	0,01956437	0,039855638
PRKAR1A	protein_coding	ENSG00000108946	-0,133458514	0,019564435	0,039855638
HEY1	protein_coding	ENSG00000164683	0,361050585	0,019571045	0,039865145
SEM1	protein_coding	ENSG00000127922	-0,164833616	0,019578325	0,039876013
CHTF18	protein_coding	ENSG00000127586	-0,203560726	0,01961146	0,039939535
RPL3P2	processed_pseudogene	ENSG00000227939	0,782915527	0,019624155	0,039957452
NCK2	protein_coding	ENSG00000071051	-0,158193485	0,019622545	0,039957452
ANAPC1P1	transcribed_unprocesse	ENSG00000233673	0,755764381	0,019631658	0,039968762
AC007906.2	protein_coding	ENSG00000277639	0,573616048	0,019670116	0,040043085
PRDM11	protein_coding	ENSG00000019485	-0,158785462	0,019779916	0,040262611
AC098617.1	antisense	ENSG00000233766	0,555910529	0,019796607	0,040292588
TBC1D5	protein_coding	ENSG00000131374	-0,119336186	0,019803029	0,040297662
CTDSPL2	protein_coding	ENSG00000137770	-0,161261399	0,01980209	0,040297662
ENTPD3-AS1	antisense	ENSG00000223797	-0,363367492	0,019807974	0,040303726
AICDA	protein_coding	ENSG00000111732	1,284580152	0,019825625	0,040335638
MMP24OS	protein_coding	ENSG00000126005	-0,225618944	0,019832253	0,04034512
ZBED5-AS1	antisense	ENSG00000247271	0,260064291	0,019850183	0,040373586
PTS	protein_coding	ENSG00000150787	-0,154487438	0,01990073	0,040472382
CSNK2A1	protein_coding	ENSG00000101266	-0,083136953	0,019960273	0,040589451
KCNMA1	protein_coding	ENSG00000156113	0,108243584	0,019983579	0,040632815
EME2	protein_coding	ENSG00000197774	0,296480015	0,019990808	0,040640055
RO60	protein_coding	ENSG00000116747	-0,150752916	0,019991103	0,040640055
VGF	protein_coding	ENSG00000128564	0,365685792	0,019995331	0,040643652
CHEK2	protein_coding	ENSG00000183765	-0,160663906	0,019996835	0,040643652
RN7SL473P	misc_RNA	ENSG00000277452	1,182842299	0,020026647	0,040700211
RDH11	protein_coding	ENSG00000072042	0,108086094	0,020031525	0,040706091
LINC01399	transcribed_processed_	ENSG00000233080	0,370245301	0,020065992	0,040772092
MRGPRX3	protein_coding	ENSG00000179826	1,218299521	0,020071976	0,04078021
RPS20P22	transcribed_processed_	ENSG00000239218	0,921567815	0,020107931	0,040849214
RMND1	protein_coding	ENSG00000155906	-0,148763433	0,020118727	0,040867098
ZHX1	protein_coding	ENSG00000165156	0,164052589	0,02012215	0,040870004
TMEM106A	protein_coding	ENSG00000184988	0,280750459	0,020139796	0,040901795
PEX1	protein_coding	ENSG00000127980	-0,146452111	0,020142144	0,040902512
NAGK	protein_coding	ENSG00000124357	-0,157422416	0,020151039	0,040916524
ANKZF1	protein_coding	ENSG00000163516	0,157595747	0,020168867	0,04094867
FBH1	protein_coding	ENSG00000134452	0,118634369	0,020201229	0,041010314

AC090004.1	protein_coding	ENSG00000268279	-1,067582371	0,020204051	0,041011983
TESK1	protein_coding	ENSG00000107140	0,211550402	0,020219895	0,041040083
AC007541.1	antisense	ENSG00000260329	-0,486689383	0,020225495	0,041047387
PSMB3	protein_coding	ENSG00000277791	-0,187048766	0,020228335	0,041049091
FAM161B	protein_coding	ENSG00000156050	0,227101563	0,020260467	0,041110227
SFXN5	protein_coding	ENSG00000144040	0,257812641	0,020290021	0,041166122
NOP53	protein_coding	ENSG00000105373	-0,133309725	0,020336247	0,041249524
TINF2	protein_coding	ENSG00000092330	-0,136306831	0,020335096	0,041249524
RBBP8	protein_coding	ENSG00000101773	-0,161921666	0,020337162	0,041249524
AL359643.3	lincRNA	ENSG00000272142	-0,696512734	0,020390538	0,041353697
GPATCH11	protein_coding	ENSG00000152133	-0,166974899	0,020418482	0,041406274
TUBAP2	processed_pseudogene	ENSG00000214391	-0,464211245	0,020430693	0,041422846
ABCC4	protein_coding	ENSG00000125257	-0,13379648	0,020429694	0,041422846
FAM86GP	unprocessed_pseudoge	ENSG00000166492	-1,053025587	0,020435597	0,041428693
CR381653.2	unprocessed_pseudoge	ENSG00000279208	-0,20647351	0,02045728	0,041468553
DUSP28	protein_coding	ENSG00000188542	-0,272592985	0,020464488	0,041479064
ZNF268	protein_coding	ENSG00000090612	0,138998088	0,020477169	0,041496566
MTA3	protein_coding	ENSG00000057935	-0,106425026	0,02047556	0,041496566
ZNF516	protein_coding	ENSG00000101493	-0,179397342	0,020486953	0,041512291
UBALD1	protein_coding	ENSG00000153443	0,326513964	0,020496212	0,041524248
ZDHHC2	protein_coding	ENSG00000104219	-0,132204093	0,020496903	0,041524248
XKR9	protein_coding	ENSG00000221947	0,999015597	0,020503244	0,041532992
NDEL1	protein_coding	ENSG00000166579	0,115175735	0,020513026	0,041548702
MDC1	protein_coding	ENSG00000137337	-0,160015237	0,020519597	0,041557909
AC099520.1	lincRNA	ENSG00000251574	0,569642991	0,020557041	0,041629632
E2F5	protein_coding	ENSG00000133740	-0,163359365	0,020596983	0,041706399
BX323046.1	lincRNA	ENSG00000273175	1,021081066	0,020621223	0,041742202
AL365357.1	processed_pseudogene	ENSG00000213058	-0,231726163	0,020624841	0,041742202
UBE3C	protein_coding	ENSG00000009335	-0,091542274	0,020616951	0,041742202
SLC39A1	protein_coding	ENSG00000143570	-0,1371083	0,020623766	0,041742202
ICAM3	protein_coding	ENSG00000076662	-0,235744096	0,020621713	0,041742202
SNX18P7	processed_pseudogene	ENSG00000234373	0,547939167	0,020669041	0,041827532
CR1L	protein_coding	ENSG00000197721	1,263636592	0,020714924	0,041916248
DYNC1LI2	protein_coding	ENSG00000135720	-0,133017144	0,020733995	0,0419507
DUOXA1	protein_coding	ENSG00000140254	0,812124031	0,020794832	0,042069639
SCAF11	protein_coding	ENSG00000139218	0,101055251	0,020802475	0,042080951
TRIM11	protein_coding	ENSG00000154370	0,221206588	0,020819574	0,042111387
KRT5	protein_coding	ENSG00000186081	0,846880964	0,020831247	0,042130813
MRPL9	protein_coding	ENSG00000143436	-0,122548032	0,020833286	0,042130813
CHCHD10	protein_coding	ENSG00000250479	-0,132942045	0,020842706	0,042145707

TRPM8	protein_coding	ENSG00000144481	1,245265133	0,020891245	0,042239692
GALC	protein_coding	ENSG00000054983	-0,172743042	0,020897723	0,042248625
GCNT2P1	processed_pseudogene	ENSG00000205318	0,793262592	0,020908586	0,042266421
NFIA	protein_coding	ENSG00000162599	-0,324625602	0,020914375	0,042273957
RPL10P13	processed_pseudogene	ENSG00000258245	1,05560345	0,020943409	0,042328471
LYNX1	protein_coding	ENSG00000180155	0,959966386	0,020951892	0,042341442
AC018553.2	antisense	ENSG00000283689	-1,061874342	0,020980378	0,042394833
LINC00265	lincRNA	ENSG00000188185	0,509648489	0,020988398	0,042406861
NOMO2	protein_coding	ENSG00000185164	0,179453591	0,021002218	0,042430604
NOMO2	protein_coding	ENSG00000185164	0,179453591	0,021002218	0,042430604
AC044781.1	antisense	ENSG00000229751	1,053693083	0,021010916	0,042443995
AC022034.1	lincRNA	ENSG00000237807	0,471985137	0,021013232	0,042444493
NSRP1	protein_coding	ENSG00000126653	-0,151245684	0,021029583	0,042473339
AC107294.2	lincRNA	ENSG00000272970	1,179997336	0,021053576	0,042517609
ACBD5	protein_coding	ENSG00000107897	0,112275121	0,021056362	0,042519049
FOLR3	protein_coding	ENSG00000110203	0,593076051	0,021059742	0,042521687
PPARGC1A	protein_coding	ENSG00000109819	0,814819043	0,021094258	0,042587186
SDHAF4	protein_coding	ENSG00000154079	0,217103992	0,021108962	0,042612678
AP000808.1	lincRNA	ENSG00000250508	1,184179439	0,021194463	0,042781068
STON2	protein_coding	ENSG00000140022	1,117573963	0,021225835	0,042836228
MOB3A	protein_coding	ENSG00000172081	-0,21851504	0,021225967	0,042836228
CHD1	protein_coding	ENSG00000153922	-0,161292819	0,02124593	0,042872296
RPL34	protein_coding	ENSG00000109475	-0,171499656	0,021294785	0,042966654
SCOC	protein_coding	ENSG00000153130	-0,193201983	0,021323376	0,04302011
SMC2	protein_coding	ENSG00000136824	-0,15161934	0,021348499	0,043066559
APOBEC3C	protein_coding	ENSG00000244509	-0,108007515	0,021356417	0,043078296
SEC11A	protein_coding	ENSG00000140612	-0,146455551	0,021380947	0,043123535
CFAP47	protein_coding	ENSG00000165164	0,288355497	0,021392766	0,04314313
DLX1	protein_coding	ENSG00000144355	-0,438823557	0,021401685	0,043156873
AL118516.1	antisense	ENSG00000260708	-0,373625811	0,021424821	0,04319928
JPT1	protein_coding	ENSG00000189159	0,114984741	0,02143163	0,043208761
RHOJ	protein_coding	ENSG00000126785	0,590344497	0,021443482	0,043228407
GLMN	protein_coding	ENSG00000174842	-0,166770137	0,021450906	0,043239124
PRRT3-AS1	antisense	ENSG00000230082	-0,363545762	0,021486082	0,043305773
ERCC8	protein_coding	ENSG00000049167	0,159987441	0,021499429	0,043328416
RPS25	protein_coding	ENSG00000118181	-0,174313491	0,021503101	0,043331559
ARHGEF9	protein_coding	ENSG00000131089	0,119192213	0,021511303	0,043343827
AC079145.1	antisense	ENSG00000227210	0,899632033	0,021545973	0,043409421
FAM172BP	processed_pseudogene	ENSG00000175841	1,083510298	0,02158455	0,043482467
MFAP3L	protein_coding	ENSG00000198948	0,198911807	0,021586469	0,043482467

BIN1	protein_coding	ENSG00000136717	0,127816267	0,02160799	0,043521544
ZNF444	protein_coding	ENSG00000167685	-0,23248856	0,021665689	0,043633471
AC005332.6	lincRNA	ENSG00000278730	0,137496115	0,021679874	0,043656543
POSTN	protein_coding	ENSG00000133110	1,20385544	0,02168353	0,043656543
PMVK	protein_coding	ENSG00000163344	-0,182102748	0,021682511	0,043656543
TEK	protein_coding	ENSG00000120156	0,717401011	0,02170457	0,043694613
HOXB-AS4	antisense	ENSG00000242207	0,939802588	0,021713659	0,043708508
EDNRA	protein_coding	ENSG00000151617	1,114293617	0,021715733	0,043708508
SLC38A10	protein_coding	ENSG00000157637	-0,183019851	0,021719433	0,043711665
DNAJC24	protein_coding	ENSG00000170946	0,15191169	0,021738399	0,043745541
ZFHX3	protein_coding	ENSG00000140836	0,189063153	0,021748903	0,043762386
ZFHX3	protein_coding	ENSG00000140836	0,189063153	0,021748903	0,043762386
TMEM230	protein_coding	ENSG00000089063	-0,147831933	0,021755842	0,043772054
PROSER1	protein_coding	ENSG00000120685	-0,238964852	0,021762491	0,043776842
HRH4	protein_coding	ENSG00000134489	1,073086998	0,021781259	0,0438103
RN7SKP30	misc_RNA	ENSG00000223305	-0,984651152	0,021805952	0,043847067
CCDC152	protein_coding	ENSG00000198865	1,034052976	0,021801686	0,043847067
TMEM184A	protein_coding	ENSG00000164855	1,012052385	0,021805309	0,043847067
TPM4	protein_coding	ENSG00000167460	-0,105763964	0,021831154	0,043893439
ZKSCAN1	protein_coding	ENSG00000106261	0,147548863	0,02185728	0,04394166
SNX32	protein_coding	ENSG00000172803	0,97080844	0,021866227	0,043955339
SLC25A21-AS1	antisense	ENSG00000258708	-0,44467532	0,021868849	0,043956302
PAK2	protein_coding	ENSG00000180370	0,118750075	0,021887578	0,043989636
DDHD1	protein_coding	ENSG00000100523	0,136643895	0,021899072	0,044006106
PAOX	protein_coding	ENSG00000148832	-0,493633216	0,021900065	0,044006106
CCNY	protein_coding	ENSG00000108100	-0,138848931	0,021942755	0,04408757
DCTN2	protein_coding	ENSG00000175203	-0,117883403	0,021947033	0,044091846
AC090844.2	lincRNA	ENSG00000264968	1,156386443	0,02200385	0,044201661
UBL3	protein_coding	ENSG00000122042	-0,125578837	0,022020252	0,044230279
ZNF280B	protein_coding	ENSG00000275004	0,230682327	0,022030901	0,044247334
NAALADL2	protein_coding	ENSG00000177694	0,530752156	0,022053502	0,044288389
PEAR1	protein_coding	ENSG00000187800	0,261312083	0,02209384	0,044365055
MAP3K20-AS1	antisense	ENSG00000238133	1,021521816	0,022114484	0,044402161
TMEM14A	protein_coding	ENSG00000096092	-0,181523159	0,022129227	0,044427412
PAX2	protein_coding	ENSG00000075891	-0,536022029	0,022146225	0,044457186
ITGA10	protein_coding	ENSG00000143127	0,545822773	0,022152011	0,044464449
ASGR1	protein_coding	ENSG00000141505	0,694705373	0,022154408	0,04446491
CHRM4	protein_coding	ENSG00000180720	0,526486736	0,022159521	0,044470819
COQ5	protein_coding	ENSG00000110871	0,15700023	0,022202134	0,044551978
GMFB	protein_coding	ENSG00000197045	-0,187909368	0,022210957	0,044565324

LCE1F	protein_coding	ENSG00000240386	-0,513754177	0,022215141	0,044569358
MMP3	protein_coding	ENSG00000149968	-0,349518094	0,02223661	0,044607398
AC103796.1	sense_overlapping	ENSG00000255496	0,992126003	0,022238452	0,044607398
AC107032.2	lincRNA	ENSG00000257526	0,704965737	0,022272525	0,044669711
PRKACA	protein_coding	ENSG00000072062	0,166340534	0,022273873	0,044669711
DEPTOR	protein_coding	ENSG00000155792	-0,478065196	0,02227683	0,044671274
ADGRE2	protein_coding	ENSG00000127507	0,948793607	0,022283029	0,044679335
OGFR	protein_coding	ENSG00000060491	-0,173837975	0,022291233	0,044691416
LAMTOR5	protein_coding	ENSG00000134248	-0,152050417	0,022294714	0,044694026
AL139174.1	processed_pseudogene	ENSG00000254708	1,02226157	0,022334022	0,044768452
CACNG8	protein_coding	ENSG00000142408	0,306540161	0,022358824	0,04480503
RAB6A	protein_coding	ENSG00000175582	0,113528417	0,022358761	0,04480503
MST1P2	unprocessed_pseudoge	ENSG00000186301	0,598153771	0,022357815	0,04480503
AC024337.2	lincRNA	ENSG00000273771	0,857179058	0,022402229	0,044887624
AC026202.3	antisense	ENSG00000268509	1,008411003	0,022422984	0,044920435
KLHL18	protein_coding	ENSG00000114648	-0,139850436	0,022422196	0,044920435
ZNF695	protein_coding	ENSG00000197472	0,809548977	0,022444671	0,044959489
AC022364.1	antisense	ENSG00000247934	0,620346513	0,022475446	0,045016738
SULF1	protein_coding	ENSG00000137573	1,020674114	0,022496462	0,045054432
LINS1	protein_coding	ENSG00000140471	0,169405701	0,022506304	0,045069742
AC073389.1	antisense	ENSG00000268584	-0,629215344	0,022562224	0,045177312
KIAA1586	protein_coding	ENSG00000168116	0,178009664	0,022587973	0,045224456
CPHL1P	transcribed_unitary_pse	ENSG00000240216	1,02310844	0,022590358	0,045224817
AC006017.1	antisense	ENSG00000229591	0,740555588	0,022620268	0,045276423
IGFBP6	protein_coding	ENSG00000167779	0,218557175	0,022620551	0,045276423
TNFRSF19	protein_coding	ENSG00000127863	-0,682087469	0,022649053	0,045329047
ENOSF1	protein_coding	ENSG00000132199	-0,234296905	0,022688872	0,045404309
MAML3	protein_coding	ENSG00000196782	0,440454985	0,022691542	0,045405222
CLDN11	protein_coding	ENSG00000013297	0,863463672	0,022731813	0,04547693
ABL2	protein_coding	ENSG00000143322	0,137175377	0,022730189	0,04547693
LINC00205	bidirectional_promoter_	ENSG00000223768	-0,354800087	0,022742671	0,045494215
INVS	protein_coding	ENSG00000119509	-0,151157933	0,022753917	0,045512271
NAP1L3	protein_coding	ENSG00000186310	-0,335750385	0,022770694	0,045541389
RPL39L	protein_coding	ENSG00000163923	-0,151340284	0,022777795	0,045551148
UVRAG-DT	antisense	ENSG00000255507	0,779540994	0,022793025	0,04557716
YKT6	protein_coding	ENSG00000106636	0,1096139	0,022814051	0,045614757
CYB5RL	protein_coding	ENSG00000215883	-0,197972895	0,022866372	0,045714913
AC004477.3	lincRNA	ENSG00000278765	-0,595168452	0,022874684	0,045727072
RUNX1T1	protein_coding	ENSG00000079102	0,679205115	0,02289235	0,045757927
ORC3	protein_coding	ENSG00000135336	-0,141782019	0,022904632	0,045778015

TUFM	protein_coding	ENSG00000178952	-0,107508974	0,022913937	0,045792151
CDK5RAP2	protein_coding	ENSG00000136861	-0,159250938	0,022928569	0,045816927
MIR9-3HG	lincRNA	ENSG00000255571	-0,608963262	0,022935671	0,045826655
MAP3K15	protein_coding	ENSG00000180815	-0,160268562	0,022972653	0,045896076
RHEB	protein_coding	ENSG00000106615	-0,138409767	0,022981852	0,045905786
THOC2	protein_coding	ENSG00000125676	-0,149028862	0,02298199	0,045905786
AC100827.4	lincRNA	ENSG00000260672	1,054181394	0,023015804	0,045968852
AL513164.1	antisense	ENSG00000224658	1,066749367	0,023061301	0,046055238
DNAJC9-AS1	antisense	ENSG00000236756	0,764549217	0,023068346	0,046064821
AC137630.3	antisense	ENSG00000272434	0,967043172	0,023072861	0,046069351
PITPNA-AS1	antisense	ENSG00000236618	-0,1876531	0,023078766	0,046076655
ABCD2	protein_coding	ENSG00000173208	0,596341245	0,023088172	0,046090948
SLC10A5	protein_coding	ENSG00000253598	0,669011245	0,023096267	0,046102621
PAK6	protein_coding	ENSG00000137843	1,06860546	0,023139395	0,046184215
PAK6	protein_coding	ENSG00000137843	1,06860546	0,023139395	0,046184215
C1orf122	protein_coding	ENSG00000197982	0,234512313	0,023183325	0,046267392
ONECUT1	protein_coding	ENSG00000169856	-0,536009437	0,023188007	0,046272235
TBCC	protein_coding	ENSG00000124659	0,158936256	0,023198662	0,046288993
MARCO	protein_coding	ENSG00000019169	1,146403196	0,023222116	0,046331285
ZNF335	protein_coding	ENSG00000198026	-0,172689187	0,023323117	0,046523744
CA12	protein_coding	ENSG00000074410	0,750262564	0,023355173	0,046583157
ZHX2	protein_coding	ENSG00000178764	0,345948978	0,023363127	0,046592415
C9orf78	protein_coding	ENSG00000136819	-0,114444277	0,023364358	0,046592415
AL365203.2	lincRNA	ENSG00000273038	0,40938246	0,023383059	0,046625173
AC012618.3	transcribed_unprocesse	ENSG00000234773	0,638916931	0,023398493	0,046651413
AC016526.2	sense_overlapping	ENSG00000258454	1,114409676	0,023416138	0,046682056
EYA4	protein_coding	ENSG00000112319	-0,171862971	0,023444102	0,046733262
PSMC4	protein_coding	ENSG00000013275	0,111424457	0,023447424	0,046735342
AL137802.2	lincRNA	ENSG00000261135	0,961443126	0,023450733	0,046737396
ARRDC1	protein_coding	ENSG00000197070	-0,208097111	0,023454644	0,046740648
CDC26	protein_coding	ENSG00000176386	-0,206521138	0,023460266	0,046747309
GNPDA1	protein_coding	ENSG00000113552	-0,104343649	0,023469131	0,046760431
KRT10	protein_coding	ENSG00000186395	0,178938679	0,02354128	0,046895621
AGL	protein_coding	ENSG00000162688	0,10411502	0,023541556	0,046895621
PCAT6	antisense	ENSG00000228288	0,715707158	0,023549076	0,046906046
B4GALNT3	protein_coding	ENSG00000139044	0,826282702	0,023552822	0,046908952
AC090826.1	antisense	ENSG00000261821	-0,976122989	0,023596953	0,04699228
COL1A1	protein_coding	ENSG00000108821	0,524466211	0,023618796	0,047031214
NOL7	protein_coding	ENSG00000225921	-0,09836788	0,023640883	0,047070626
CPNE4	protein_coding	ENSG00000196353	0,978954416	0,023659856	0,04710383

SMG1P1	transcribed_unprocesse	ENSG00000237296	0,545838229	0,023692401	0,047164043
HEXA-AS1	antisense	ENSG00000260339	0,86006567	0,023709164	0,047192833
WDR60	protein_coding	ENSG00000126870	-0,204035285	0,023720142	0,047210103
ELP3	protein_coding	ENSG00000134014	-0,108281395	0,023744462	0,047253922
STAM-AS1	antisense	ENSG00000260589	0,555511109	0,023750842	0,047262033
TFPT	protein_coding	ENSG00000105619	-0,159773702	0,023802485	0,047360203
AL035461.2	lincRNA	ENSG00000275632	-0,623555634	0,023830331	0,04740641
TIMM9	protein_coding	ENSG00000100575	-0,158536698	0,0238289	0,04740641
MT-ND4L	protein_coding	ENSG00000212907	0,325739239	0,023851582	0,047439482
REEP5	protein_coding	ENSG00000129625	-0,104084311	0,023850138	0,047439482
MRPS11	protein_coding	ENSG00000181991	-0,115839654	0,023864485	0,047460544
PXDNL	protein_coding	ENSG00000147485	-0,209106189	0,023902172	0,047530885
LYN	protein_coding	ENSG00000254087	-0,146228975	0,023947423	0,047616253
LRRC23	protein_coding	ENSG00000010626	-0,214123682	0,023951482	0,047619707
SNORA74D	snoRNA	ENSG00000252213	-0,290809764	0,02396191	0,047635821
ELF2	protein_coding	ENSG00000109381	-0,132524315	0,023967926	0,047643165
AL121753.2	TEC	ENSG00000279253	0,721761382	0,024015738	0,047733578
DERL1	protein_coding	ENSG00000136986	0,096846876	0,024065792	0,047823796
NDUFAB1	protein_coding	ENSG00000004779	-0,14531338	0,024064509	0,047823796
TRIM3	protein_coding	ENSG00000110171	0,172959736	0,024070009	0,047827541
ADD3	protein_coding	ENSG00000148700	0,132428226	0,024104681	0,047891797
TSNARE1	protein_coding	ENSG00000171045	-0,225750192	0,024129203	0,047935874
AC004232.3	TEC	ENSG00000279031	-0,831420192	0,024134179	0,047941115
COQ10A	protein_coding	ENSG00000135469	0,157599383	0,024158303	0,047984388
WDR31	protein_coding	ENSG00000148225	0,278977653	0,024221493	0,048105241
DCP2	protein_coding	ENSG00000172795	-0,135618982	0,024231362	0,048120182
FUT8	protein_coding	ENSG00000033170	-0,130971471	0,024244564	0,048141737
CORO1A	protein_coding	ENSG00000102879	0,434987676	0,024283411	0,048214206
MAP4K5	protein_coding	ENSG00000012983	0,130937801	0,02429069	0,048223991
NOL9	protein_coding	ENSG00000162408	-0,138514818	0,024318868	0,04827526
AC104073.4	lincRNA	ENSG00000282381	-0,761143031	0,024325578	0,048283906
MRVI1	protein_coding	ENSG00000072952	1,05849149	0,024330944	0,048287515
LGALSL	protein_coding	ENSG00000119862	0,152198833	0,024332105	0,048287515
NOTCH3	protein_coding	ENSG00000074181	0,811384094	0,024348995	0,048316358
EGLN2	protein_coding	ENSG00000269858	-0,249141127	0,024352728	0,048319092
AC006504.8	sense_intronic	ENSG00000281468	-0,414209208	0,024392629	0,048388898
KANSL2	protein_coding	ENSG00000139620	0,143999931	0,024397436	0,048393754
RF00432	snoRNA	ENSG00000206878	-0,786360364	0,024399985	0,048394129
B3GALT4	protein_coding	ENSG00000235863	-0,53945554	0,02440347	0,048396362
AC114956.2	antisense	ENSG00000248554	0,718798668	0,024436321	0,048456825

AL162258.2	antisense	ENSG00000272030	0,706812934	0,024441591	0,048462591
C1R	protein_coding	ENSG00000159403	0,982856193	0,024458661	0,048491748
AC097534.1	antisense	ENSG00000248774	-0,542469219	0,024465538	0,048496007
AC019294.2	processed_transcript	ENSG00000260288	0,996207315	0,02446464	0,048496007
AC009502.2	processed_pseudogene	ENSG00000231802	-0,59653326	0,024486434	0,048531017
ADGRA2	protein_coding	ENSG00000020181	0,283109596	0,024487932	0,048531017
TBC1D24	protein_coding	ENSG00000162065	-0,263678439	0,024570991	0,048690921
TKFC	protein_coding	ENSG00000149476	-0,150077497	0,024596561	0,048736882
HGSNAT	protein_coding	ENSG00000165102	-0,166959627	0,02461966	0,04877794
GLYATL2	protein_coding	ENSG00000156689	0,435704324	0,024633894	0,048801425
THNSL2	protein_coding	ENSG00000144115	0,476867028	0,024645006	0,048818724
RBMS1	protein_coding	ENSG00000153250	-0,11700871	0,024675548	0,048874505
BOLA1	protein_coding	ENSG00000178096	-0,21905953	0,024680333	0,048876011
TMEM150A	protein_coding	ENSG00000168890	-0,259695103	0,024681075	0,048876011
SH3TC2	protein_coding	ENSG00000169247	-0,133164147	0,024693753	0,048896396
AC005839.1	TEC	ENSG00000279089	-0,385485487	0,024699416	0,048902888
PHRF1	protein_coding	ENSG00000070047	-0,18080877	0,024716655	0,048930403
KANTR	protein_coding	ENSG00000232593	-0,229069282	0,024718084	0,048930403
ETAA1	protein_coding	ENSG00000143971	-0,172056136	0,02473754	0,04896419
AP1S3	protein_coding	ENSG00000152056	-0,184339313	0,024743106	0,048970482
PMS2P4	transcribed_unprocesse	ENSG00000067601	-0,292448174	0,024802285	0,04908287
AC068580.4	protein_coding	ENSG00000250644	1,086158443	0,024831659	0,049136259
PTCRA	protein_coding	ENSG00000171611	0,494003439	0,024898837	0,049264437
ORMDL3	protein_coding	ENSG00000172057	0,131659943	0,024916797	0,049295217
EIF4G1	protein_coding	ENSG00000114867	-0,184499872	0,024939776	0,049335918
USE1	protein_coding	ENSG00000053501	-0,161833496	0,024965528	0,049382099
AC040970.1	antisense	ENSG00000253210	0,345252727	0,024987328	0,049420453
LINC02643	lincRNA	ENSG00000230109	1,034220731	0,024997983	0,049435562
EIF3G	protein_coding	ENSG00000130811	-0,104939362	0,024999788	0,049435562
CBARP	protein_coding	ENSG00000099625	0,273125481	0,025029507	0,049489559
WBP1	protein_coding	ENSG00000239779	0,474766055	0,025036776	0,049499159
AL031775.1	antisense	ENSG00000272345	0,496178968	0,025049981	0,049520492
ATAD3B	protein_coding	ENSG00000160072	-0,187772956	0,02506433	0,049544082
AUH	protein_coding	ENSG00000148090	0,235451523	0,025125296	0,049659807
AL139811.1	processed_pseudogene	ENSG00000224072	1,038739359	0,025128515	0,049661383
AC027373.1	sense_overlapping	ENSG00000260368	0,739790414	0,025132529	0,04966453
PRKCG	protein_coding	ENSG00000126583	0,885053841	0,025141885	0,049678233
GREM2	protein_coding	ENSG00000180875	1,055214899	0,025145194	0,049679986
KLF17	protein_coding	ENSG00000171872	-0,492844182	0,025165156	0,049714635
HTR4	protein_coding	ENSG00000164270	1,047472738	0,02517059	0,049717434

CCNH	protein_coding	ENSG00000134480	0,132164472	0,025172405	0,049717434
PRR16	protein_coding	ENSG00000184838	-0,224826484	0,025173844	0,049717434
TJP3	protein_coding	ENSG00000105289	0,54566732	0,02522121	0,049806183
ZNF605	protein_coding	ENSG00000196458	-0,175430384	0,025248278	0,049854836
CHCHD2	protein_coding	ENSG00000106153	-0,165366303	0,025266377	0,049885772
BMP2K	protein_coding	ENSG00000138756	0,135647287	0,025272371	0,049892802
DPY19L1P1	unprocessed_pseudoge	ENSG00000229358	-0,336369831	0,025286957	0,049916793
KDM4A-AS1	antisense	ENSG00000236200	0,306495883	0,025303829	0,049945293
AC092944.1	processed_transcript	ENSG00000243176	0,838643185	0,025347518	0,050025979
MDFI	protein_coding	ENSG00000112559	0,481191419	0,025349586	0,050025979
NFYAP1	processed_pseudogene	ENSG00000237849	0,639095987	0,025443683	0,050206844
ANKS3	protein_coding	ENSG00000168096	-0,226290054	0,025462291	0,050238729
ZGLP1	protein_coding	ENSG00000220201	0,75462918	0,025491539	0,050291599
DOCK6	protein_coding	ENSG00000130158	0,201380289	0,025498274	0,050300048
RIBC1	protein_coding	ENSG00000158423	0,580719443	0,025503161	0,050304849
ZNF346-IT1	sense_intronic	ENSG00000251666	-0,623863538	0,025520075	0,050333372
ARHGEF10L	protein_coding	ENSG00000074964	-0,169307121	0,025536119	0,050360173
CDS2	protein_coding	ENSG00000101290	-0,103817034	0,025563904	0,050410122
RAB33B	protein_coding	ENSG00000172007	-0,183554743	0,025568011	0,050411441
THTPA	protein_coding	ENSG00000259431	-0,323632107	0,025569489	0,050411441
AFG3L2	protein_coding	ENSG00000141385	-0,130543351	0,025620898	0,050507941
AC138627.1	lincRNA	ENSG00000261404	0,911899692	0,025623446	0,050508109
CYP4Z1	protein_coding	ENSG00000186160	0,876111353	0,025629273	0,050514741
ABCG2	protein_coding	ENSG00000118777	0,366021539	0,025649811	0,050550362
DGUOK	protein_coding	ENSG00000114956	-0,123913411	0,025688526	0,050621798
AC012636.1	antisense	ENSG00000249825	1,064498579	0,025729099	0,05069688
AC096733.2	lincRNA	ENSG00000273472	0,840973298	0,025739082	0,050711678
AL078621.1	processed_pseudogene	ENSG00000144158	0,797394379	0,025760135	0,050743409
AC107027.3	sense_overlapping	ENSG00000261167	-0,261829566	0,025759071	0,050743409
KCNJ12	protein_coding	ENSG00000184185	1,062089652	0,025798515	0,050814131
AC134772.1	antisense	ENSG00000244380	0,802690596	0,025825228	0,050861862
CD79B	protein_coding	ENSG00000007312	0,818783608	0,025835744	0,050871847
FBXW2	protein_coding	ENSG00000119402	-0,114447499	0,025837739	0,050871847
LRRFIP1	protein_coding	ENSG00000124831	-0,120359518	0,025836709	0,050871847
AP001267.1	antisense	ENSG00000254873	1,062053381	0,025846797	0,050884795
LINC02256	lincRNA	ENSG00000261064	0,873041783	0,025851629	0,050886789
SOCS6	protein_coding	ENSG00000170677	0,115421319	0,025852771	0,050886789
BTBD16	protein_coding	ENSG00000138152	0,973480637	0,02585839	0,05088808
CLCN2	protein_coding	ENSG00000114859	0,2953277	0,025856669	0,05088808
OR4N2	protein_coding	ENSG00000176294	0,849001889	0,02588665	0,050933921

RCOR2	protein_coding	ENSG00000167771	0,744715231	0,025886269	0,050933921
RMDN1	protein_coding	ENSG00000176623	-0,112633173	0,025909295	0,050973587
DDX39B-AS1	antisense	ENSG00000234006	0,830132968	0,025912509	0,05097502
NBPF20	protein_coding	ENSG00000162825	0,341647066	0,025922643	0,050990066
IL12A-AS1	antisense	ENSG00000244040	0,929459986	0,025942575	0,051024378
UBAC2	protein_coding	ENSG00000134882	-0,107495658	0,025950679	0,05103053
ATP5ME	protein_coding	ENSG00000169020	-0,136788002	0,025950038	0,05103053
SLC16A1	protein_coding	ENSG00000155380	0,087706251	0,026005245	0,051132928
HLA-G	protein_coding	ENSG00000204632	0,783794672	0,026026632	0,051170076
RALGDS	protein_coding	ENSG00000160271	0,183416364	0,02603756	0,051185997
NBDY	protein_coding	ENSG00000204272	0,12371572	0,026042218	0,051185997
PAM	protein_coding	ENSG00000145730	-0,112486205	0,026040058	0,051185997
ROCK2	protein_coding	ENSG00000134318	-0,136586673	0,026177331	0,051446633
AC135352.1	unprocessed_pseudoge	ENSG00000251468	-0,696107202	0,026188615	0,051463877
NAPSA	protein_coding	ENSG00000131400	0,709416202	0,026230413	0,051541077
GRM5	protein_coding	ENSG00000168959	0,808220323	0,026269726	0,051613381
IGSF9	protein_coding	ENSG00000085552	0,937639098	0,026280412	0,051629431
GTF2E2	protein_coding	ENSG00000197265	-0,140331764	0,026316161	0,051694709
HCK	protein_coding	ENSG00000101336	0,923468086	0,026336003	0,051728731
PTPN11	protein_coding	ENSG00000179295	0,118463615	0,026343819	0,051739129
LINC02331	lincRNA	ENSG00000235269	0,470889142	0,026355939	0,051757976
HSF4	protein_coding	ENSG00000102878	-0,559748974	0,026365376	0,051771551
RTL5	protein_coding	ENSG00000242732	0,441672343	0,026373569	0,051780434
OXR1	protein_coding	ENSG00000164830	0,117530843	0,026374949	0,051780434
AC011511.2	antisense	ENSG00000266978	1,004972854	0,026416104	0,051852603
ZNF260	protein_coding	ENSG00000254004	-0,135817807	0,026416765	0,051852603
RP1L1	protein_coding	ENSG00000183638	0,953743328	0,02642197	0,051857857
UBE2Q2P1	transcribed_unprocesse	ENSG00000189136	0,357314112	0,026461577	0,051930624
AL031283.1	lincRNA	ENSG00000228140	0,887418775	0,026477051	0,05195602
LACTB2	protein_coding	ENSG00000147592	0,239504656	0,026501025	0,051998088
SNORD105B	snoRNA	ENSG00000238531	-1,071670845	0,026511625	0,052013911
TMEM108-AS1	antisense	ENSG00000251011	0,701211591	0,026544391	0,052073215
AL392172.1	lincRNA	ENSG00000228106	-0,238150488	0,026588461	0,05215468
MYEF2	protein_coding	ENSG00000104177	0,124959414	0,026596794	0,052166036
AC244669.1	transcribed_unprocesse	ENSG00000223804	0,415700064	0,02660647	0,052180025
PLCL1	protein_coding	ENSG00000115896	0,411101334	0,026627545	0,052211372
TCEA1	protein_coding	ENSG00000187735	0,129356195	0,026626294	0,052211372
DYNC2H1	protein_coding	ENSG00000187240	0,175879599	0,026650365	0,052251121
MECR	protein_coding	ENSG00000116353	-0,144191831	0,026661415	0,052267791
LAP3	protein_coding	ENSG00000002549	-0,119289643	0,026677704	0,052294725

AKIRIN2	protein_coding	ENSG00000135334	0,133495184	0,02668092	0,052296032
RPAP3	protein_coding	ENSG00000005175	0,138801244	0,026698574	0,052325634
AL022311.1	sense_overlapping	ENSG00000279738	-0,344486502	0,026713659	0,052350196
RPL37	protein_coding	ENSG00000145592	-0,131837296	0,026753825	0,052423899
CAVIN4	protein_coding	ENSG00000170681	0,561329767	0,026762769	0,052436415
PRPF38A	protein_coding	ENSG00000134748	-0,139357053	0,026774089	0,052453584
AL355607.2	lincRNA	ENSG00000260454	0,96272884	0,026783491	0,05246678
USP27X	protein_coding	ENSG00000273820	-0,274459848	0,026786484	0,05246678
CISH	protein_coding	ENSG00000114737	-0,844277361	0,026788499	0,05246678
INHBA-AS1	antisense	ENSG00000224116	1,043530594	0,026803825	0,052491784
AC007256.1	processed_pseudogene	ENSG00000213090	0,767407607	0,026818808	0,052516046
ALMS1-IT1	sense_intronic	ENSG00000230002	-0,475631387	0,026821335	0,052516046
SOCS5	protein_coding	ENSG00000171150	0,099691545	0,026835095	0,052532958
PPP1R3D	protein_coding	ENSG00000132825	-0,263262246	0,026833039	0,052532958
LTV1	protein_coding	ENSG00000135521	-0,184178819	0,026932587	0,052718779
EXT2	protein_coding	ENSG00000151348	-0,086709906	0,026957284	0,052762085
CCDC134	protein_coding	ENSG00000100147	-0,212843825	0,0269689	0,052779784
CERNA1	lincRNA	ENSG00000259577	0,639635382	0,026973869	0,052784472
NMRAL2P	transcribed_unprocessed	ENSG00000171658	0,67257853	0,026990717	0,052812403
AC091132.4	lincRNA	ENSG00000267198	0,754706824	0,027005625	0,052836533
ARL4C	protein_coding	ENSG00000188042	-0,110390512	0,027019334	0,052858312
FTL	protein_coding	ENSG00000087086	0,132610587	0,027026529	0,052867344
ELOA-AS1	antisense	ENSG00000236810	0,364763238	0,027043339	0,052895183
ADPRHL1	protein_coding	ENSG00000153531	0,179482523	0,02706567	0,052933813
FTSJ1	protein_coding	ENSG00000068438	0,090833161	0,027083412	0,052963461
ZNF252P	transcribed_unprocessed	ENSG00000196922	0,123669435	0,027147624	0,053083972
TBC1D2	protein_coding	ENSG00000095383	0,153730191	0,027199316	0,053179978
SULT1C2	protein_coding	ENSG00000198203	1,024512538	0,027207397	0,053190707
DYNLT3	protein_coding	ENSG00000165169	0,166434734	0,027211045	0,053192769
OTUD6B-AS1	antisense	ENSG00000253738	-0,162598689	0,027243008	0,053250175
STPG4	protein_coding	ENSG00000239605	-0,232885307	0,027305504	0,053363355
AC048344.4	sense_intronic	ENSG00000277342	-0,492860116	0,027306115	0,053363355
ZNF593	protein_coding	ENSG00000142684	-0,180416835	0,027337732	0,053420053
AC244154.1	transcribed_unprocessed	ENSG00000274487	0,494475291	0,027349207	0,053437385
AL133367.1	antisense	ENSG00000260285	0,625256272	0,027360859	0,05345506
DECR1	protein_coding	ENSG00000104325	0,136493198	0,02737282	0,053473335
ALK	protein_coding	ENSG00000171094	1,023102274	0,027488256	0,053688613
LGALS9	protein_coding	ENSG00000168961	1,007363665	0,027487046	0,053688613
KLHL8	protein_coding	ENSG00000145332	-0,16808696	0,027492404	0,053691601
TTC9B	protein_coding	ENSG00000174521	0,825428175	0,027512437	0,053721725

TATDN2	protein_coding	ENSG00000157014	-0,185178802	0,027513067	0,053721725
FAM118B	protein_coding	ENSG00000197798	0,156745117	0,027533315	0,053756144
AC069213.1	antisense	ENSG00000223711	0,955069133	0,027549964	0,053778411
FNTA	protein_coding	ENSG00000168522	0,129212097	0,027548788	0,053778411
SNHG26	processed_transcript	ENSG00000228649	-0,175333578	0,02759956	0,053870097
AC245297.3	lincRNA	ENSG00000274265	0,350452029	0,027643723	0,053951162
SMPD3	protein_coding	ENSG00000103056	0,885975539	0,027653706	0,0539641
P3H3	protein_coding	ENSG00000110811	-0,164053798	0,027655614	0,0539641
RPL31	protein_coding	ENSG00000071082	-0,140424016	0,027669645	0,053986342
DNAJC27	protein_coding	ENSG00000115137	0,264678075	0,027697084	0,054034737
RNF2	protein_coding	ENSG00000121481	0,151439459	0,027711833	0,05405837
SLC2A5	protein_coding	ENSG00000142583	0,904885775	0,027731503	0,054091595
AC079610.2	lincRNA	ENSG00000270659	-0,758591783	0,027759571	0,054141194
FCER1G	protein_coding	ENSG00000158869	0,775325214	0,027767591	0,054151686
AHNAK2	protein_coding	ENSG00000185567	-0,22182794	0,027771816	0,054154776
AKT1S1	protein_coding	ENSG00000204673	0,175237188	0,027831149	0,054265317
PIM3	protein_coding	ENSG00000198355	0,178064339	0,02784265	0,05428258
MTX2	protein_coding	ENSG00000128654	-0,097019411	0,027852304	0,054296241
AC025287.2	lincRNA	ENSG00000276408	0,817173438	0,027866952	0,054314472
ATOH8	protein_coding	ENSG00000168874	-0,615447517	0,027866172	0,054314472
PAAF1	protein_coding	ENSG00000175575	-0,165756326	0,027883172	0,054340922
GNAI3	protein_coding	ENSG00000065135	-0,088961092	0,027915494	0,054398743
AC093274.1	lincRNA	ENSG00000249359	0,85070418	0,027928997	0,054414717
NDUFB6	protein_coding	ENSG00000165264	-0,141744594	0,027927063	0,054414717
ZSCAN21	protein_coding	ENSG00000166529	0,188963503	0,027939512	0,054430033
SNAI3-AS1	antisense	ENSG00000260630	0,508440659	0,027973457	0,054490987
VPS29	protein_coding	ENSG00000111237	-0,146072779	0,027983483	0,05450534
EMILIN1	protein_coding	ENSG00000138080	0,563787132	0,027997212	0,054521726
SEC22B	protein_coding	ENSG00000265808	0,094337777	0,02799632	0,054521726
AC046136.1	lincRNA	ENSG00000242880	0,993332728	0,028014248	0,054549723
TDGF1P2	processed_pseudogene	ENSG00000183514	0,996889839	0,028025551	0,054561372
H2AFJ	protein_coding	ENSG00000246705	0,159463915	0,028025515	0,054561372
IL11RA	protein_coding	ENSG00000137070	-0,274560245	0,02804407	0,054592243
GPC2	protein_coding	ENSG00000213420	0,811465941	0,02805167	0,054601855
PSMA4	protein_coding	ENSG00000041357	-0,14776526	0,028075544	0,054643141
FSIP1	protein_coding	ENSG00000150667	0,37050394	0,028112166	0,054709224
PAPSS2	protein_coding	ENSG00000198682	-0,082602406	0,028125921	0,054730802
MPZ	protein_coding	ENSG00000158887	0,875391789	0,028144243	0,054756063
WNT7B	protein_coding	ENSG00000188064	-0,790746896	0,028141759	0,054756063
TSPAN9	protein_coding	ENSG00000011105	0,19892314	0,028159411	0,054777097

MORC3	protein_coding	ENSG00000159256	0,127665411	0,028160395	0,054777097
LINC00342	lincRNA	ENSG00000232931	-0,404556623	0,028173204	0,054796815
AL356020.1	antisense	ENSG00000258731	0,894637813	0,028185404	0,054815345
ATXN2-AS	antisense	ENSG00000258099	0,575941397	0,028197173	0,054833035
ATP6V1E2	protein_coding	ENSG00000250565	0,15149115	0,028259504	0,054949036
ZNF26	protein_coding	ENSG00000198393	0,119135946	0,028292612	0,055008197
ASB14	protein_coding	ENSG00000239388	0,936596638	0,028337326	0,055089911
SRGAP2C	protein_coding	ENSG00000171943	-0,130863297	0,028342259	0,055094279
CCDC168	protein_coding	ENSG00000175820	0,583358527	0,028365242	0,05513373
ACACB	protein_coding	ENSG00000076555	-0,18676168	0,028376367	0,055150126
ARSG	protein_coding	ENSG00000141337	-0,316576782	0,028439537	0,055267662
ZXDB	protein_coding	ENSG00000198455	-0,169641581	0,028467663	0,055317078
AP000695.3	TEC	ENSG00000279365	0,769682655	0,028474205	0,055324549
TMC1	protein_coding	ENSG00000165091	0,963092893	0,028516213	0,055400922
ATAD1	protein_coding	ENSG00000138138	0,127911726	0,028529069	0,055420649
AC037487.3	processed_pseudogene	ENSG00000272006	1,01941589	0,028541633	0,055432652
RPL27	protein_coding	ENSG00000131469	-0,181883053	0,028543356	0,055432652
ATXN7L2	protein_coding	ENSG00000162650	-0,237467046	0,028541284	0,055432652
Z99943.2	unprocessed_pseudoge	ENSG00000250762	0,943233985	0,02854734	0,05543514
P2RX6	protein_coding	ENSG00000099957	0,67697151	0,02857371	0,055480417
HSPA14	protein_coding	ENSG00000187522	-0,149617007	0,028576066	0,055480417
JADE3	protein_coding	ENSG00000102221	0,10546217	0,028583602	0,055489793
INTS13	protein_coding	ENSG00000064102	-0,14122602	0,028612136	0,05553993
AC012146.1	processed_transcript	ENSG00000234327	-0,181503162	0,028630675	0,055570659
FBXL19-AS1	antisense	ENSG00000260852	-0,45762376	0,028642467	0,05558602
RBPJ	protein_coding	ENSG00000168214	0,120024828	0,02864401	0,05558602
GTF2IP13	transcribed_unprocesse	ENSG00000272556	0,568687497	0,028692847	0,055675525
AC009948.2	antisense	ENSG00000270277	-0,514149789	0,028710728	0,05570495
GPN1	protein_coding	ENSG00000198522	-0,15460555	0,028720119	0,055717901
NUDT13	protein_coding	ENSG00000166321	-0,264173273	0,028725691	0,05572344
SHLD2P3	unprocessed_pseudoge	ENSG00000189014	0,707095743	0,028731282	0,055729014
CBWD5	protein_coding	ENSG00000147996	-0,213575533	0,028751458	0,055762874
CDK5	protein_coding	ENSG00000164885	-0,163653563	0,028762124	0,055778287
AC005696.1	antisense	ENSG00000262050	-0,767617565	0,028788108	0,055823399
EIF3J	protein_coding	ENSG00000104131	0,133965201	0,028792785	0,055827191
AC009996.1	sense_intronic	ENSG00000259659	-0,811445948	0,028826834	0,055887927
DNAJC19P5	processed_pseudogene	ENSG00000225808	-0,82758138	0,028832992	0,055894581
ARRB2	protein_coding	ENSG00000141480	-0,10209935	0,028968463	0,056151893
RNU6-26P	snRNA	ENSG00000206712	0,750923629	0,028990183	0,056188685
ZFP92	protein_coding	ENSG00000189420	-0,64359763	0,029043761	0,056287209

AC126696.3	lincRNA	ENSG00000260498	0,83160074	0,029079662	0,056351461
SCAT8	antisense	ENSG00000236345	0,956957389	0,029098533	0,056372051
AC079598.3	processed_pseudogene	ENSG00000258179	0,875404638	0,029095793	0,056372051
TFPI	protein_coding	ENSG00000003436	-0,117994875	0,029093948	0,056372051
PITHD1	protein_coding	ENSG00000057757	0,105307262	0,029118732	0,056400528
GBAP1	transcribed_unprocesse	ENSG00000160766	0,362577992	0,029116947	0,056400528
C7orf69	antisense	ENSG00000136275	0,28014684	0,029137882	0,056432291
AC245140.2	antisense	ENSG00000280195	0,55383109	0,029145506	0,056441727
LINC00964	lincRNA	ENSG00000249816	0,716793534	0,029167321	0,056478641
PKD1	protein_coding	ENSG00000008710	0,340051763	0,029192785	0,056522612
PCDH11X	protein_coding	ENSG00000102290	0,783176515	0,029205318	0,056540867
PCDHB6	protein_coding	ENSG00000113211	0,310765314	0,029207727	0,056540867
PSPN	protein_coding	ENSG00000125650	0,349511071	0,029222215	0,056563574
AC006059.1	antisense	ENSG00000230084	0,818911265	0,029234755	0,056582507
PARS2	protein_coding	ENSG00000162396	-0,245596206	0,02926481	0,056635333
LAMTOR2	protein_coding	ENSG00000116586	-0,181802081	0,029330119	0,056756368
KRT8P36	processed_pseudogene	ENSG00000240668	-0,31094762	0,029358823	0,056806552
MYO16-AS1	antisense	ENSG00000236242	0,586353118	0,029385152	0,056841989
AC091987.1	lincRNA	ENSG00000253584	0,502533592	0,029380404	0,056841989
UPB1	protein_coding	ENSG00000100024	0,843094788	0,029385451	0,056841989
ALG5	protein_coding	ENSG00000120697	0,178407867	0,02938883	0,056843164
PAQR5	protein_coding	ENSG00000137819	-0,202053643	0,029398417	0,056856345
EML3	protein_coding	ENSG00000149499	-0,190069304	0,029454943	0,056960295
AC107398.4	bidirectional_promoter_	ENSG00000282904	0,978630638	0,029463861	0,056961429
UBE2M	protein_coding	ENSG00000130725	-0,106927465	0,029462815	0,056961429
RNPC3	protein_coding	ENSG00000185946	-0,190225367	0,02946111	0,056961429
C15orf41	protein_coding	ENSG00000186073	-0,159215346	0,02946789	0,056963847
SEZ6	protein_coding	ENSG00000063015	0,97650989	0,029519849	0,057053534
KLHL5	protein_coding	ENSG00000109790	0,095904579	0,029518726	0,057053534
AL603756.1	antisense	ENSG00000271933	0,889912625	0,029525858	0,057059771
USP40	protein_coding	ENSG00000085982	-0,11710922	0,029535322	0,057072683
LINC02615	lincRNA	ENSG00000251432	0,171293149	0,029555489	0,057100894
PHF11	protein_coding	ENSG00000136147	0,201483446	0,0295536	0,057100894
HTRA4	protein_coding	ENSG00000169495	0,869903106	0,029569004	0,057121623
CMYA5	protein_coding	ENSG00000164309	0,641359215	0,029575345	0,057123112
MORN4	protein_coding	ENSG00000171160	0,173012408	0,029574036	0,057123112
AL365436.1	processed_pseudogene	ENSG00000223861	0,890824527	0,029600354	0,057156681
PLXNB1	protein_coding	ENSG00000164050	0,357097375	0,029601086	0,057156681
PCBP4	protein_coding	ENSG00000090097	0,16208484	0,029595798	0,057156681
GMPPA	protein_coding	ENSG00000144591	0,136649924	0,029625677	0,057198779

AC023983.2	lincRNA	ENSG00000273321	0,981125074	0,02966144	0,057262438
TSPAN31	protein_coding	ENSG00000135452	0,17520393	0,029680017	0,057292908
SCARNA7	scaRNA	ENSG00000238741	0,184162617	0,029684295	0,057295775
HAAO	protein_coding	ENSG00000162882	-0,284064062	0,029696442	0,057313826
SMPDL3A	protein_coding	ENSG00000172594	0,241690451	0,029708489	0,057331682
AC117382.2	antisense	ENSG00000261758	-0,814780846	0,029725403	0,057358924
RNF146	protein_coding	ENSG00000118518	0,148122206	0,029741256	0,057384116
GPSM1	protein_coding	ENSG00000160360	-0,193151545	0,029760758	0,057416343
MMAA	protein_coding	ENSG00000151611	-0,236438847	0,029776974	0,057442224
AC092718.6	antisense	ENSG00000261838	0,858312671	0,029794861	0,057465921
AC007743.1	antisense	ENSG00000233251	-0,469631205	0,029792776	0,057465921
H1FX-AS1	antisense	ENSG00000206417	-0,264972326	0,029893934	0,057651584
AL132780.1	antisense	ENSG00000257285	-0,491452715	0,029898287	0,057654557
IMPA2	protein_coding	ENSG00000141401	-0,426757875	0,029919913	0,057690835
AC010325.2	antisense	ENSG00000268375	-0,816175987	0,029929102	0,057703128
AC087257.1	lincRNA	ENSG00000248100	0,85739235	0,029958989	0,0577449
WSCD1	protein_coding	ENSG00000179314	0,958570338	0,029959214	0,0577449
DNAJC21	protein_coding	ENSG00000168724	0,103572893	0,02995428	0,0577449
ZXDC	protein_coding	ENSG00000070476	-0,135291495	0,030111752	0,058028003
DOCK10	protein_coding	ENSG00000135905	-0,143540734	0,030115844	0,058030437
CTSE	protein_coding	ENSG00000196188	0,840504288	0,030123673	0,05804007
C18orf32	protein_coding	ENSG00000177576	0,390507472	0,030145343	0,058076365
AK2	protein_coding	ENSG00000004455	-0,098427106	0,030169228	0,058116921
TOR1AIP2	protein_coding	ENSG00000169905	-0,0766	0,030180299	0,058132789
KRT8P46	processed_pseudogene	ENSG00000248971	0,477865288	0,030197292	0,058160059
WAC-AS1	antisense	ENSG00000254635	0,160669019	0,03021199	0,058182903
PPP3R1	protein_coding	ENSG00000221823	0,131605038	0,030238318	0,058228139
SNORA71C	snoRNA	ENSG00000201512	-0,335235216	0,030252669	0,058250305
RNF181	protein_coding	ENSG00000168894	0,148016498	0,030276296	0,058290325
AUP1	protein_coding	ENSG00000115307	0,090817115	0,030292366	0,058315791
AC012354.1	lincRNA	ENSG00000225156	0,529155845	0,030354047	0,058429048
AC112484.3	lincRNA	ENSG00000261159	0,714390667	0,030380251	0,058474001
SERPINA1	protein_coding	ENSG00000197249	0,228127894	0,03041084	0,058527384
AGGF1	protein_coding	ENSG00000164252	0,102688919	0,030422718	0,05854475
SCAMP2	protein_coding	ENSG00000140497	0,133136297	0,030437574	0,058567843
EXD1	protein_coding	ENSG00000178997	0,756374381	0,030460517	0,058606493
CR381653.1	lincRNA	ENSG00000278932	-0,26283526	0,030509061	0,058694385
ACAP1	protein_coding	ENSG00000072818	0,46801716	0,030516916	0,05870399
TRIM16	protein_coding	ENSG00000221926	0,153583458	0,030520825	0,058706005
SNX2	protein_coding	ENSG00000205302	0,106107553	0,030542941	0,058740877

YTHDF2	protein_coding	ENSG00000198492	-0,083037061	0,030544683	0,058740877
RLIM	protein_coding	ENSG00000131263	0,109919062	0,030684812	0,059004827
SNTA1	protein_coding	ENSG00000101400	-0,215822541	0,030689638	0,059008575
AC012020.1	processed_transcript	ENSG00000279277	0,940120381	0,030710936	0,059038457
AC016397.2	TEC	ENSG00000279822	-0,552365163	0,030710087	0,059038457
SMIM2-IT1	sense_intronic	ENSG00000235285	-0,805016009	0,030751174	0,05911027
URB1-AS1	lincRNA	ENSG00000256073	-0,219492639	0,030756505	0,059114976
CDYL2	protein_coding	ENSG00000166446	0,141510144	0,030767844	0,059131229
KRT83	protein_coding	ENSG00000170523	0,897966405	0,030780513	0,059150036
TMEM17	protein_coding	ENSG00000186889	-0,369254497	0,030795379	0,059173059
EIF5	protein_coding	ENSG00000100664	0,092089458	0,030814665	0,059204569
MAMSTR	protein_coding	ENSG00000176909	-0,491332829	0,030860081	0,059286275
SCAP	protein_coding	ENSG00000114650	-0,144795368	0,030890746	0,059339628
TOMM7	protein_coding	ENSG00000196683	-0,162134669	0,030920224	0,05939069
RBM19	protein_coding	ENSG00000122965	0,138974491	0,030934229	0,059412027
ITGA2	protein_coding	ENSG00000164171	-0,134471557	0,031046555	0,059622176
BBOF1	protein_coding	ENSG00000119636	0,308366928	0,031123177	0,059763726
BRMS1	protein_coding	ENSG00000174744	-0,120720512	0,031133576	0,059778098
ULK1	protein_coding	ENSG00000177169	0,218984362	0,031205444	0,059904872
S100A11	protein_coding	ENSG00000163191	-0,114922719	0,031202662	0,059904872
CPA6	protein_coding	ENSG00000165078	0,897644018	0,031218923	0,059923901
LMBR1	protein_coding	ENSG00000105983	-0,106767918	0,0312212	0,059923901
TPH1	protein_coding	ENSG00000129167	0,641764161	0,031260269	0,059993273
GIP	protein_coding	ENSG00000159224	0,919241109	0,031293825	0,060052055
ST3GAL4	protein_coding	ENSG00000110080	-0,134702147	0,031334922	0,060125292
SSBP2	protein_coding	ENSG00000145687	-0,252446486	0,031342217	0,060133664
KANSL3	protein_coding	ENSG00000114982	-0,11733106	0,031372116	0,060185398
SNORD1B	snoRNA	ENSG00000199961	-0,866239558	0,031380449	0,060195754
PPP4C	protein_coding	ENSG00000149923	-0,158050776	0,031385511	0,060199835
CBR3-AS1	processed_transcript	ENSG00000236830	-0,410794559	0,031431122	0,060281682
FCRLB	protein_coding	ENSG00000162746	0,315553595	0,031455238	0,060322294
PQBP1	protein_coding	ENSG00000102103	-0,144272191	0,031497103	0,060396932
SPDEF	protein_coding	ENSG00000124664	0,891290476	0,031576809	0,06054411
AC027097.1	antisense	ENSG00000267040	-0,290378147	0,031601094	0,060585011
CAMTA1-DT	antisense	ENSG00000237436	0,740273674	0,03161777	0,060611316
MGST3	protein_coding	ENSG00000143198	-0,144363949	0,031624667	0,060618871
NOMO3	protein_coding	ENSG00000103226	0,190840073	0,031631563	0,060626423
SLCO6A1	protein_coding	ENSG00000205359	0,915798148	0,031675751	0,060699771
AC019080.5	sense_intronic	ENSG00000280374	0,72332496	0,031673537	0,060699771
AL135744.1	antisense	ENSG00000260830	0,827163196	0,031687657	0,060716913

NTF4	protein_coding	ENSG00000225950	0,914047952	0,03169906	0,060722231
SLCO1B3	protein_coding	ENSG00000111700	0,536927192	0,031702211	0,060722231
ZNF16	protein_coding	ENSG00000170631	0,167440392	0,031697027	0,060722231
RPL35A	protein_coding	ENSG00000182899	-0,149503429	0,031702274	0,060722231
CLPX	protein_coding	ENSG00000166855	-0,104766591	0,031726987	0,060756403
ACTR1B	protein_coding	ENSG00000115073	-0,121742381	0,031726671	0,060756403
SIRT5	protein_coding	ENSG00000124523	-0,135937879	0,031729002	0,060756403
C15orf56	processed_transcript	ENSG00000176753	0,92259171	0,031746043	0,060783359
CS	protein_coding	ENSG00000062485	-0,100865647	0,031754531	0,060793935
PHKB	protein_coding	ENSG00000102893	0,12991324	0,031765174	0,060808635
IL18R1	protein_coding	ENSG00000115604	0,907867337	0,031779103	0,060829623
ZFP69B	protein_coding	ENSG00000187801	-0,249447235	0,03178273	0,060830888
FAM207A	protein_coding	ENSG00000160256	-0,157581763	0,031839683	0,060934208
GTPBP2	protein_coding	ENSG00000172432	-0,161897109	0,03184429	0,060937338
ST6GALNAC6	protein_coding	ENSG00000160408	-0,129985248	0,031861965	0,060965473
AL390728.6	lincRNA	ENSG00000259865	-0,488144241	0,031973453	0,06117309
MTATP6P1	unprocessed_pseudoge	ENSG00000248527	0,44662046	0,031996311	0,061211114
BCAR3	protein_coding	ENSG00000137936	0,091876819	0,032028578	0,061267127
AC018926.3	sense_intronic	ENSG00000277548	0,878783925	0,032041923	0,061286939
AC005845.1	lincRNA	ENSG00000255775	0,85615252	0,03204764	0,061292158
EIF4A2	protein_coding	ENSG00000156976	-0,109212871	0,032094168	0,061375422
STXBP1	protein_coding	ENSG00000136854	0,145906281	0,032120823	0,061420668
AC008982.2	sense_intronic	ENSG00000269688	-0,494104684	0,032193762	0,061554402
CCDC144A	protein_coding	ENSG00000170160	0,903434035	0,032199094	0,061558857
SUPT5H	protein_coding	ENSG00000196235	0,136788915	0,032224359	0,061601418
LRCH3	protein_coding	ENSG00000186001	0,095332942	0,032255769	0,061655715
AP001107.9	antisense	ENSG00000255468	-0,410646913	0,032263576	0,061664462
SMG1P7	transcribed_unprocesse	ENSG00000261556	0,20021357	0,032266358	0,061664462
TANC2	protein_coding	ENSG00000170921	-0,143322923	0,032331017	0,061782276
AL391422.1	processed_pseudogene	ENSG00000219992	0,871699028	0,032377153	0,061858911
MDH2	protein_coding	ENSG00000146701	-0,111363514	0,032397122	0,061891297
ABRAXAS2	protein_coding	ENSG00000165660	0,124885297	0,032407706	0,061905752
TMEM108	protein_coding	ENSG00000144868	-0,293801134	0,032441725	0,061964963
RPL18	protein_coding	ENSG00000063177	-0,151921008	0,032459353	0,06199286
SRP14P3	processed_pseudogene	ENSG00000235183	-0,823670835	0,032516536	0,06209629
BID	protein_coding	ENSG00000015475	-0,134534582	0,032523072	0,062102989
METTL23	protein_coding	ENSG00000181038	-0,171607102	0,032564058	0,062175463
OBSL1	protein_coding	ENSG00000124006	-0,167909165	0,032590879	0,062220882
CRK	protein_coding	ENSG00000167193	0,070366166	0,032608789	0,062249279
C7orf65	lincRNA	ENSG00000221845	0,841910192	0,032618017	0,062255306

AC025048.6	transcribed_processed_	ENSG00000280852	0,878863681	0,032616336	0,062255306
ANKRD13A	protein_coding	ENSG00000076513	0,085168861	0,032717601	0,062439564
PYURF	protein_coding	ENSG00000145337	-0,128677345	0,032784913	0,062562203
UBE2Z	protein_coding	ENSG00000159202	-0,087329442	0,032797816	0,062581003
AC138028.6	antisense	ENSG00000278341	0,760027767	0,032852371	0,062679268
CXCL16	protein_coding	ENSG00000161921	-0,474038563	0,032859133	0,062686337
PLCE1-AS1	antisense	ENSG00000268894	0,553021532	0,032905219	0,062768419
ARHGEF40	protein_coding	ENSG00000165801	-0,210074679	0,03295242	0,062852612
SLC7A5P1	unprocessed_pseudoge	ENSG00000260727	0,446879345	0,032956554	0,062854652
GLYR1	protein_coding	ENSG00000140632	-0,090342692	0,032964831	0,062864592
WNK1	protein_coding	ENSG00000060237	0,147444542	0,032982735	0,062892887
MTMR11	protein_coding	ENSG00000014914	-0,215001341	0,03301345	0,062945603
AL133243.1	sense_intronic	ENSG00000274159	-0,830819837	0,033045962	0,063001736
AC118553.2	protein_coding	ENSG00000283761	0,510819128	0,033056206	0,06301541
RUFY2	protein_coding	ENSG00000204130	0,14284594	0,033088983	0,063072029
SNORA2B	snoRNA	ENSG00000207313	-0,615293829	0,033094504	0,063076692
SMIM26	protein_coding	ENSG00000232388	-0,185447156	0,033105265	0,063091339
ANKRD24	protein_coding	ENSG00000089847	0,344152887	0,033113511	0,06310119
ERMP1	protein_coding	ENSG00000099219	-0,147053214	0,033116599	0,063101212
CCDC17	protein_coding	ENSG00000159588	0,576550934	0,033145823	0,06315103
AC087741.2	lincRNA	ENSG00000275479	0,775062158	0,033154179	0,063161082
U2AF1L5	protein_coding	ENSG00000275895	-0,374127877	0,0331647	0,063175257
CHML	protein_coding	ENSG00000203668	-0,190724765	0,033168305	0,063176257
AC004895.1	lincRNA	ENSG00000231704	0,853950878	0,033182128	0,063196716
TMEM59L	protein_coding	ENSG00000105696	0,66858879	0,033196566	0,063218345
ANXA6	protein_coding	ENSG00000197043	0,098027061	0,033211248	0,063240432
SELENOM	protein_coding	ENSG00000198832	0,195977843	0,033256883	0,063321452
STARD5	protein_coding	ENSG00000172345	-0,348377972	0,033260904	0,063323229
AC012531.1	lincRNA	ENSG00000260597	0,750046709	0,033291124	0,063374879
RUNDC3A	protein_coding	ENSG00000108309	0,735797743	0,033354139	0,063488945
PLA2G12AP1	processed_pseudogene	ENSG00000224680	0,892629051	0,033387649	0,063546834
TIMM10B	protein_coding	ENSG00000132286	-0,150469312	0,033434924	0,063630907
LINC01719	lincRNA	ENSG00000233396	0,436441281	0,03345377	0,063660867
ADAMTSL4	protein_coding	ENSG00000143382	-0,358373006	0,03346048	0,063667728
KIF9	protein_coding	ENSG00000088727	-0,226127099	0,033475152	0,063689738
FLVCR2	protein_coding	ENSG00000119686	-0,358990175	0,033548762	0,063823867
MST1R	protein_coding	ENSG00000164078	0,590767083	0,033564799	0,063846219
SPIRE2	protein_coding	ENSG00000204991	-0,188140767	0,033566737	0,063846219
PER3	protein_coding	ENSG00000049246	-0,238259527	0,033620833	0,063943184
FLT1	protein_coding	ENSG00000102755	0,800103378	0,033624949	0,063945082

AC092167.1	lincRNA	ENSG00000226965	0,904823754	0,033708423	0,064097882
ADRB2	protein_coding	ENSG00000169252	0,102167142	0,033713452	0,064101502
NF1	protein_coding	ENSG00000196712	0,116252751	0,033717428	0,064103118
NBN	protein_coding	ENSG00000104320	-0,127304252	0,033751622	0,06416218
PLEKHH2	protein_coding	ENSG00000152527	0,362284472	0,033755491	0,064163588
WIPF2	protein_coding	ENSG00000171475	-0,116593647	0,033802987	0,064247916
ZNF623	protein_coding	ENSG00000183309	-0,112208167	0,0338071	0,06424978
RPS6KA6	protein_coding	ENSG00000072133	0,165711993	0,033823811	0,064275582
ICAM2	protein_coding	ENSG00000108622	-0,37380079	0,033828949	0,064279391
MYBPC2	protein_coding	ENSG00000086967	0,90711539	0,033832104	0,06427943
RNF141	protein_coding	ENSG00000110315	-0,11916889	0,033850068	0,064307604
ITGB1BP2	protein_coding	ENSG00000147166	-0,53926552	0,033881303	0,064360982
MALL	protein_coding	ENSG00000144063	0,512812315	0,033887847	0,064367451
FBXW10	protein_coding	ENSG00000171931	0,844148044	0,033917785	0,064418351
AC108517.1	lincRNA	ENSG00000249111	0,777733991	0,033944303	0,064462622
TMEM248	protein_coding	ENSG00000106609	-0,099413953	0,033947381	0,064462622
IPO7	protein_coding	ENSG00000205339	0,114347093	0,033964453	0,064489069
ZNF774	protein_coding	ENSG00000196391	0,306941685	0,033968825	0,064491401
AC079174.2	antisense	ENSG00000277715	0,681015067	0,034015919	0,064574833
AMFR	protein_coding	ENSG00000159461	0,077555006	0,034022452	0,064581259
CDK8	protein_coding	ENSG00000132964	-0,117148609	0,03404044	0,064609423
KCNK3	protein_coding	ENSG00000171303	0,901685919	0,034058125	0,064637008
SELENOW	protein_coding	ENSG00000178980	-0,165243562	0,034097395	0,064705549
NGLY1	protein_coding	ENSG00000151092	0,118358473	0,034106482	0,064716806
POP4	protein_coding	ENSG00000105171	-0,141933051	0,0341101	0,064717683
HDAC11	protein_coding	ENSG00000163517	-0,210308381	0,034165077	0,064815996
AL158070.1	antisense	ENSG00000228317	0,831640462	0,034235722	0,064944013
ZBTB5	protein_coding	ENSG00000168795	0,190099056	0,034253368	0,064971476
AP002518.1	antisense	ENSG00000256195	0,817081808	0,034257323	0,064972969
ATRIP	protein_coding	ENSG00000164053	-0,705680932	0,034283247	0,065016125
ZNF805	protein_coding	ENSG00000204524	0,157277811	0,034291695	0,065020122
RECQL	protein_coding	ENSG00000004700	-0,113172162	0,034289555	0,065020122
MYOZ2	protein_coding	ENSG00000172399	0,874391372	0,034308999	0,065046918
SEPT6	protein_coding	ENSG00000125354	-0,139022328	0,034342586	0,065104578
AC083906.3	antisense	ENSG00000250643	-0,866339684	0,034388672	0,065179896
HDAC4	protein_coding	ENSG00000068024	-0,231449708	0,034385612	0,065179896
AC006001.3	transcribed_unprocesse	ENSG00000229180	0,172257028	0,034456139	0,065301738
FAM168A	protein_coding	ENSG00000054965	-0,118112137	0,034462643	0,06530803
AP003392.4	lincRNA	ENSG00000255121	-0,435606211	0,034512736	0,065396916
CGGBP1	protein_coding	ENSG00000163320	0,108484558	0,034559589	0,065479646

TSPAN10	protein_coding	ENSG00000182612	0,310854896	0,034609855	0,065568828
EPHB3	protein_coding	ENSG00000182580	0,716837769	0,034621524	0,065584876
TMEM260	protein_coding	ENSG00000070269	0,181226503	0,034624959	0,065585326
LRP2BP	protein_coding	ENSG00000109771	0,540976425	0,034668552	0,065661295
RPS29	protein_coding	ENSG00000213741	-0,17181874	0,034671469	0,065661295
ATP5MF	protein_coding	ENSG00000241468	-0,154427857	0,034686913	0,065678415
C17orf51	protein_coding	ENSG00000212719	-0,170867888	0,034686691	0,065678415
AL451050.2	lincRNA	ENSG00000272205	-0,845780504	0,034744895	0,065782128
AL512625.1	lincRNA	ENSG00000170161	0,546556668	0,034810838	0,065898106
PRRG1	protein_coding	ENSG00000130962	0,11908051	0,034812578	0,065898106
TIMMDC1	protein_coding	ENSG00000113845	-0,124925047	0,034823005	0,065911761
MDH1	protein_coding	ENSG00000014641	-0,141038538	0,034827334	0,065913871
AC147067.1	lincRNA	ENSG00000244459	0,642698621	0,034871506	0,06599138
LHPP	protein_coding	ENSG00000107902	-0,204864545	0,034877972	0,065997528
KRT32	protein_coding	ENSG00000108759	0,580182666	0,034910984	0,066053901
B4GALT7	protein_coding	ENSG00000027847	-0,147644025	0,034914437	0,06605434
ZNF575	protein_coding	ENSG00000176472	0,624950098	0,034957223	0,066129187
AL109809.4	processed_pseudogene	ENSG00000271461	0,726415985	0,034963785	0,066134668
AL596087.1	processed_pseudogene	ENSG00000215835	-0,536624925	0,034966569	0,066134668
ARHGDIB	protein_coding	ENSG00000111348	-0,120791059	0,034982985	0,066159615
RBM26	protein_coding	ENSG00000139746	-0,108669737	0,035006375	0,066197745
SLC35A3	protein_coding	ENSG00000117620	-0,159266173	0,035012363	0,066202966
SNRPD3	protein_coding	ENSG00000100028	-0,147087601	0,035027956	0,066226344
ULK3	protein_coding	ENSG00000140474	-0,154874925	0,035045878	0,066254121
ERVK9-11	lincRNA	ENSG00000269486	0,652313128	0,035053875	0,066263131
VNN2	protein_coding	ENSG00000112303	0,877242586	0,035124885	0,066391243
LINC01040	lincRNA	ENSG00000226037	0,877631156	0,035131393	0,066397427
AL138828.1	antisense	ENSG00000237596	0,222730264	0,035196415	0,066514186
RPL21P28	processed_pseudogene	ENSG00000220749	-0,409143322	0,035208657	0,066531192
ATXN10	protein_coding	ENSG00000130638	-0,127424825	0,035212273	0,066531896
YPEL4	protein_coding	ENSG00000166793	0,884985886	0,035234266	0,066567317
AC009802.1	antisense	ENSG00000283667	0,546352367	0,035247933	0,066587004
SLC7A1	protein_coding	ENSG00000139514	-0,146478971	0,035265994	0,066614987
TMEM201	protein_coding	ENSG00000188807	-0,25199038	0,035352753	0,06677272
AC026461.3	lincRNA	ENSG00000260823	0,665483318	0,03544323	0,066937445
AVL9	protein_coding	ENSG00000105778	-0,091292391	0,035458318	0,066959773
ADAM32	protein_coding	ENSG00000197140	0,420687519	0,035474386	0,066983949
MECP2	protein_coding	ENSG00000169057	-0,143190795	0,035489724	0,067006741
AC073611.2	antisense	ENSG00000257605	0,512987925	0,035518882	0,067049449
AC007405.2	antisense	ENSG00000235934	-0,85215014	0,035516269	0,067049449

RASD2	protein_coding	ENSG00000100302	-0,315208845	0,035558082	0,06711727
AC087163.3	TEC	ENSG00000280198	-0,756841861	0,035610824	0,067210638
HSPA8P3	processed_pseudogene	ENSG00000234788	-0,856038221	0,035642443	0,067264125
DDR1	protein_coding	ENSG00000204580	0,190105392	0,035697735	0,067362273
AL133243.3	sense_intronic	ENSG00000276517	0,358423469	0,035751395	0,067457324
TPRKBP2	processed_pseudogene	ENSG00000260452	-0,881351064	0,035785106	0,067508511
HCG4B	unprocessed_pseudoge	ENSG00000227262	0,847367561	0,035782058	0,067508511
ARG2	protein_coding	ENSG00000081181	0,239454548	0,035827253	0,067581804
ZNF582	protein_coding	ENSG00000018869	0,268146815	0,035863121	0,067643242
FOXO4	protein_coding	ENSG00000184481	0,324237005	0,035892316	0,067692083
ZCCHC8	protein_coding	ENSG00000033030	-0,104960651	0,035925689	0,067748794
ARMCX5-GPRASP2	processed_transcript	ENSG00000271147	0,338963111	0,035958512	0,067804456
AC023310.4	lincRNA	ENSG00000278626	0,80931655	0,035997868	0,067872429
DCAF6	protein_coding	ENSG00000143164	-0,109076209	0,036031987	0,067930514
INTS6-AS1	antisense	ENSG00000236778	0,460249137	0,036050932	0,067959983
FBXO25	protein_coding	ENSG00000147364	0,116984243	0,036062344	0,067975247
SNORD93	snoRNA	ENSG00000221740	-0,599585591	0,036068597	0,067980787
AC009237.9	processed_pseudogene	ENSG00000236750	0,668505475	0,036123508	0,068078025
TM7SF3	protein_coding	ENSG00000064115	-0,084512633	0,036146031	0,068114213
LINC01786	lincRNA	ENSG00000230415	0,740685192	0,036156653	0,068121712
RAB2A	protein_coding	ENSG00000104388	-0,142993698	0,036156434	0,068121712
AC024451.4	sense_intronic	ENSG00000269924	0,838707174	0,03617366	0,068147493
COPS6	protein_coding	ENSG00000168090	0,103851408	0,036187794	0,068167859
AC004988.1	antisense	ENSG00000203446	0,740228771	0,036192457	0,068170382
AL117336.1	sense_intronic	ENSG00000269952	0,806941216	0,036213306	0,06820339
QPCTL	protein_coding	ENSG00000011478	0,193049762	0,036230226	0,06822899
WIPF1	protein_coding	ENSG00000115935	0,317136684	0,036245524	0,068251532
AL645504.1	antisense	ENSG00000235121	0,80744301	0,036259468	0,068271522
GNG4	protein_coding	ENSG00000168243	-0,191848038	0,036301536	0,068344457
AL162171.4	sense_intronic	ENSG00000278576	-0,809705099	0,036359474	0,068447253
MRPS30	protein_coding	ENSG00000112996	-0,119192628	0,036366527	0,068454246
LENG8-AS1	antisense	ENSG00000226696	-0,422087605	0,036385879	0,068484389
RELN	protein_coding	ENSG00000189056	0,658455196	0,036421114	0,068544416
AMACR	protein_coding	ENSG00000242110	-0,384524537	0,0364304	0,068555602
FAM155A	protein_coding	ENSG00000204442	0,160958463	0,036465725	0,068615781
TPBG	protein_coding	ENSG00000146242	-0,189087335	0,036474433	0,068625871
CAND2	protein_coding	ENSG00000144712	-0,215083594	0,036546911	0,068755929
TM4SF4	protein_coding	ENSG00000169903	0,823542194	0,036587795	0,068826532
PIAS1	protein_coding	ENSG00000033800	-0,115659566	0,036611594	0,068864985
AL137918.1	TEC	ENSG00000279633	0,627008237	0,036694892	0,069015336

AC069277.1	lincRNA	ENSG00000189229	0,614934928	0,036712432	0,069035966
MMP10	protein_coding	ENSG00000166670	0,81788498	0,036712593	0,069035966
AREL1	protein_coding	ENSG00000119682	0,152616117	0,036747402	0,069095088
TALDO1	protein_coding	ENSG00000177156	-0,127484209	0,036769108	0,069129563
LRSAM1	protein_coding	ENSG00000148356	0,143371105	0,036788042	0,069158822
LINC01096	lincRNA	ENSG00000246095	0,344446565	0,036805845	0,069185947
SNORD116-13	snoRNA	ENSG00000207137	0,738631105	0,036825763	0,069217046
C13orf46	protein_coding	ENSG00000283199	0,515104108	0,036859364	0,069273852
H3F3C	protein_coding	ENSG00000188375	0,803939168	0,036880974	0,069301766
ZSCAN9	protein_coding	ENSG00000137185	-0,148795221	0,036879913	0,069301766
AL359764.1	lincRNA	ENSG00000225554	0,809439922	0,036925447	0,069378977
DYNLRB1	protein_coding	ENSG00000125971	-0,127826329	0,036945574	0,069410435
SCG2	protein_coding	ENSG00000171951	0,437567136	0,036978156	0,069465285
AL731563.3	lincRNA	ENSG00000272630	0,619764684	0,037003839	0,069507166
DOP1B	protein_coding	ENSG00000142197	-0,142023153	0,037018698	0,069528708
NEAT1	lincRNA	ENSG00000245532	0,28423128	0,037041456	0,069565082
ZRANB2-AS2	processed_transcript	ENSG00000229956	0,713105262	0,037077096	0,069612894
MAPK1	protein_coding	ENSG00000100030	-0,079014187	0,037074318	0,069612894
CROCC	protein_coding	ENSG00000058453	-0,229615735	0,037076832	0,069612894
CHI3L2	protein_coding	ENSG00000064886	0,383954928	0,037085835	0,069622926
TTC19	protein_coding	ENSG00000011295	0,087892756	0,037089267	0,069622996
SLC38A9	protein_coding	ENSG00000177058	0,13820024	0,037111203	0,069657799
ACAD11	protein_coding	ENSG00000240303	0,322115051	0,03712831	0,069683533
B3GALNT1	protein_coding	ENSG00000169255	-0,279533039	0,037147257	0,069712714
LINC01910	lincRNA	ENSG00000266278	-0,444110222	0,037193754	0,069793586
ST6GALNAC4	protein_coding	ENSG00000136840	-0,160321325	0,037215362	0,069827745
AC123768.3	antisense	ENSG00000262728	-0,763900973	0,037233387	0,069848785
MAP1LC3B2	protein_coding	ENSG00000258102	0,795719137	0,037230318	0,069848785
RAB27B	protein_coding	ENSG00000041353	-0,151730897	0,037240261	0,069855291
LINC01132	lincRNA	ENSG00000227630	0,725013246	0,037244813	0,069855696
TMEM38B	protein_coding	ENSG00000095209	0,156711044	0,037247289	0,069855696
TCEA1P4	processed_pseudogene	ENSG00000236184	0,847938493	0,037308218	0,069963569
IGBP1	protein_coding	ENSG00000089289	0,113270089	0,037315919	0,069971612
SH3D21	protein_coding	ENSG00000214193	-0,410088162	0,037344919	0,07001959
CNOT1	protein_coding	ENSG00000125107	-0,123868058	0,037435856	0,070183675
DANCR	processed_transcript	ENSG00000226950	-0,131308402	0,037554672	0,070380695
PREPL	protein_coding	ENSG00000138078	0,112270744	0,037551913	0,070380695
PREP	protein_coding	ENSG00000085377	-0,113977878	0,037550076	0,070380695
DQX1	protein_coding	ENSG00000144045	0,669929232	0,037610987	0,070474768
C7orf26	protein_coding	ENSG00000146576	-0,146739338	0,037611741	0,070474768

EMP3	protein_coding	ENSG00000142227	-0,09622382	0,037650515	0,070540976
AC091182.2	lincRNA	ENSG00000253746	-0,258715936	0,037659164	0,070550736
TMEM268	protein_coding	ENSG00000157693	-0,144211334	0,037679227	0,070581875
JMY	protein_coding	ENSG00000152409	0,148080783	0,037696607	0,070607982
SNX29	protein_coding	ENSG00000048471	0,151667133	0,037736979	0,070677147
SMIM27	protein_coding	ENSG00000235453	0,321855476	0,037778063	0,070747633
ZNF197	protein_coding	ENSG00000186448	0,110985956	0,037793459	0,070770003
ZNF197	protein_coding	ENSG00000186448	0,110985956	0,037793459	0,070770003
SH3BP5	protein_coding	ENSG00000131370	-0,126843966	0,037808179	0,070791104
ZNF717	protein_coding	ENSG00000227124	-0,178613609	0,037836927	0,070838465
NCKAP1L	protein_coding	ENSG00000123338	0,412590414	0,037866856	0,070888027
HOMER3-AS1	antisense	ENSG00000269019	0,390687237	0,037973677	0,071081511
STS	protein_coding	ENSG00000101846	0,165951729	0,038010657	0,071144239
ATP6V1B1-AS1	antisense	ENSG00000239322	-0,545172353	0,038021021	0,071157144
TMOD1	protein_coding	ENSG00000136842	0,666702332	0,038051883	0,071208406
CAPN10	protein_coding	ENSG00000142330	-0,203705914	0,038095402	0,07128334
PCGF7P	processed_pseudogene	ENSG00000268140	-0,700250332	0,03813959	0,071359514
ASAH2B	protein_coding	ENSG00000204147	0,225661448	0,038167243	0,071404739
AC006372.3	lincRNA	ENSG00000234210	0,725466022	0,038254415	0,071561297
TMEM53	protein_coding	ENSG00000126106	-0,232502645	0,038293007	0,071626956
TMEM132A	protein_coding	ENSG00000006118	0,16595459	0,038323763	0,071677948
HIST1H2BA	protein_coding	ENSG00000146047	0,801531497	0,038334199	0,071690929
CDYL	protein_coding	ENSG00000153046	0,103453426	0,038382873	0,071775412
AC026704.1	antisense	ENSG00000251307	0,82856645	0,03844283	0,071874514
PRPF18	protein_coding	ENSG00000165630	0,206630028	0,038442877	0,071874514
MRRF	protein_coding	ENSG00000148187	-0,109728425	0,038449318	0,071880004
NOC4L	protein_coding	ENSG00000184967	-0,136803105	0,038453891	0,071881999
IPO11	protein_coding	ENSG00000086200	-0,117675669	0,038548728	0,072052711
NOTCH2	protein_coding	ENSG00000134250	0,15649972	0,038560955	0,072068998
MSL3P1	transcribed_processed_	ENSG00000224287	-0,626735878	0,038639858	0,072209885
FOXL1	protein_coding	ENSG00000176678	0,390573923	0,038659718	0,072240417
SLC4A5	protein_coding	ENSG00000188687	0,676103392	0,03870619	0,072320667
GOT2	protein_coding	ENSG00000125166	-0,078004048	0,038719119	0,072338234
AL442663.4	processed_pseudogene	ENSG00000259015	-0,636070761	0,038758056	0,072404384
RPSAP58	processed_pseudogene	ENSG00000225178	-0,229858043	0,038802194	0,072480237
MPZL3	protein_coding	ENSG00000160588	0,599492414	0,038823114	0,072512709
PHLDB2	protein_coding	ENSG00000144824	-0,079326013	0,038854047	0,072563877
RTEL1P1	transcribed_processed_	ENSG00000251441	0,837409909	0,038889299	0,072623101
MARC2	protein_coding	ENSG00000117791	0,161384788	0,038897853	0,072632462
MLF2	protein_coding	ENSG00000089693	-0,115895184	0,038904035	0,072637393

IL18BP	protein_coding	ENSG00000137496	0,408199161	0,038907698	0,07263762
ZNF503	protein_coding	ENSG00000165655	-0,216375025	0,038940052	0,072691404
AL136038.3	antisense	ENSG00000261242	0,730377453	0,038955056	0,072712795
OGFRL1	protein_coding	ENSG00000119900	-0,143338187	0,038982368	0,072757154
EPS15	protein_coding	ENSG00000085832	0,09499727	0,038992121	0,072768736
NDUFB2	protein_coding	ENSG00000090266	-0,148765776	0,039061287	0,072891183
USP50	protein_coding	ENSG00000170236	0,820061789	0,039150618	0,073051236
AL355488.1	antisense	ENSG00000273373	-0,434673649	0,03924007	0,073211484
CCDC183	protein_coding	ENSG00000213213	0,713110688	0,039280159	0,073279612
AC008494.3	antisense	ENSG00000271797	0,772033623	0,039323904	0,073354549
ENOPH1	protein_coding	ENSG00000145293	-0,115102532	0,039339135	0,073376287
LONP1	protein_coding	ENSG00000196365	-0,116674997	0,039399166	0,073481577
AC245041.1	lincRNA	ENSG00000273760	0,59294048	0,039584169	0,073819904
TULP3	protein_coding	ENSG00000078246	-0,127551537	0,039633484	0,073902634
ZNF174	protein_coding	ENSG00000103343	-0,205080987	0,039635737	0,073902634
KRBA2	protein_coding	ENSG00000184619	-0,609325873	0,039707637	0,074029965
RHBDF2	protein_coding	ENSG00000129667	0,199715973	0,039724006	0,074051127
LEPROT	protein_coding	ENSG00000213625	-0,100980759	0,039726209	0,074051127
SIRT6	protein_coding	ENSG00000077463	0,198510041	0,039741914	0,074073669
SERPINB9	protein_coding	ENSG00000170542	0,641141819	0,039751442	0,074084695
CMSS1	protein_coding	ENSG00000184220	-0,146991137	0,039885822	0,074328385
AL772337.3	transcribed_unprocesse	ENSG00000234460	0,606155551	0,039913747	0,074373666
GCHFR	protein_coding	ENSG00000137880	0,737852482	0,039993944	0,074516333
PGBD1	protein_coding	ENSG00000137338	-0,132274608	0,040047248	0,074608871
AL355353.1	lincRNA	ENSG00000270761	0,70232524	0,040090863	0,074683343
GNL3L	protein_coding	ENSG00000130119	-0,1243768	0,04011615	0,074723661
ACOT11	protein_coding	ENSG00000162390	0,223689564	0,040142081	0,074765172
FKBP5	protein_coding	ENSG00000096060	-0,121834762	0,040204017	0,07487373
EEFSEC	protein_coding	ENSG00000132394	0,160106454	0,040343737	0,075127115
AL121761.1	lincRNA	ENSG00000268628	0,778248542	0,040375817	0,075180029
ETFA	protein_coding	ENSG00000140374	0,118579803	0,040434166	0,075279418
SMIM10L2B	protein_coding	ENSG00000196972	-0,805124085	0,040436536	0,075279418
POU3F2	protein_coding	ENSG00000184486	-0,275077468	0,040457476	0,075311566
ACAP2	protein_coding	ENSG00000114331	0,110657061	0,040527196	0,075434504
EIF4H	protein_coding	ENSG00000106682	-0,077848836	0,040538897	0,075449434
GSS	protein_coding	ENSG00000100983	-0,090929564	0,040548499	0,075460458
PCDHB2	protein_coding	ENSG00000112852	-0,154557324	0,040594858	0,075533026
PRICKLE1	protein_coding	ENSG00000139174	-0,458415299	0,040628764	0,075589255
PTPRR	protein_coding	ENSG00000153233	0,171547187	0,040636087	0,075596022
STEAP2	protein_coding	ENSG00000157214	-0,220552385	0,04064069	0,075597728

LINC02062	lincRNA	ENSG00000248489	0,751691774	0,040647145	0,075602879
ZMAT1	protein_coding	ENSG00000166432	0,309211545	0,040657019	0,075614387
CPSF1	protein_coding	ENSG00000071894	-0,150169812	0,040667342	0,075626728
CARD16	protein_coding	ENSG00000204397	0,759363332	0,040682822	0,075648656
ADAR	protein_coding	ENSG00000160710	-0,116387427	0,040733913	0,075736793
KRT33B	protein_coding	ENSG00000131738	0,398845459	0,04077067	0,075798263
ODC1-DT	lincRNA	ENSG00000257135	0,508198903	0,04083604	0,075912914
IER3	protein_coding	ENSG00000137331	0,093113951	0,04087438	0,075977301
NDUFAF4P1	processed_pseudogene	ENSG00000259467	0,806059584	0,040894542	0,076007888
UQCC3	protein_coding	ENSG00000204922	-0,20607385	0,040907098	0,076024337
IGFL3	protein_coding	ENSG00000188624	0,818938604	0,040966374	0,076127601
AP000350.6	lincRNA	ENSG00000273295	0,552536511	0,040983824	0,076153127
ABALON	antisense	ENSG00000281376	0,632483936	0,04103989	0,076243492
LGALS3BP	protein_coding	ENSG00000108679	-0,115599115	0,041036741	0,076243492
IL2RG	protein_coding	ENSG00000147168	0,790135522	0,041103346	0,076347549
ABCC10	protein_coding	ENSG00000124574	-0,176017024	0,041100418	0,076347549
UQCR10	protein_coding	ENSG00000184076	-0,157362705	0,041163798	0,076452912
KIAA0556	protein_coding	ENSG00000047578	0,149031476	0,041181582	0,076479016
AC023906.4	antisense	ENSG00000259709	0,758422441	0,041262368	0,076615169
SLC25A43	protein_coding	ENSG00000077713	0,110433449	0,041279016	0,076639144
RPS9	protein_coding	ENSG00000170889	-0,141450108	0,04131295	0,076695204
SF3B6	protein_coding	ENSG00000115128	-0,144625211	0,041323161	0,076707216
FAM66D	antisense	ENSG00000255052	0,550761595	0,041334646	0,076721593
PMPCB	protein_coding	ENSG00000105819	-0,140948613	0,0413875	0,076805793
SRXN1	protein_coding	ENSG00000271303	-0,473012283	0,041385844	0,076805793
RF02116	misc_RNA	ENSG00000275693	0,793465329	0,041425759	0,076869837
RPL7AP6	processed_pseudogene	ENSG00000242071	-0,283258169	0,041492706	0,076987099
AC008676.1	antisense	ENSG00000248544	0,601351651	0,0415321	0,077053222
UBE4A	protein_coding	ENSG00000110344	0,088481632	0,041589327	0,077152415
SLC25A36	protein_coding	ENSG00000114120	-0,121041553	0,04159523	0,077156387
AL049775.3	lincRNA	ENSG00000258945	0,617871157	0,041623125	0,077201148
MARC1	protein_coding	ENSG00000186205	0,712265933	0,041641607	0,077228445
GDI2	protein_coding	ENSG00000057608	-0,113419869	0,041652468	0,077241602
NUDT18	protein_coding	ENSG00000275074	-0,237768068	0,041667326	0,07726217
CHRNE	protein_coding	ENSG00000108556	0,777475397	0,041688338	0,077294143
LINC00526	lincRNA	ENSG00000264575	-0,612089127	0,041713309	0,07733345
SNHG25	lincRNA	ENSG00000266402	-0,413031533	0,041722923	0,077337294
COX6B1	protein_coding	ENSG00000126267	-0,132547444	0,041720271	0,077337294
CCNG2	protein_coding	ENSG00000138764	0,137697779	0,041734854	0,077352419
AC009133.4	antisense	ENSG00000275857	0,800936276	0,041755496	0,077383683

AC026471.6	TEC	ENSG00000280132	-0,499553797	0,041764149	0,077392727
KBTBD2	protein_coding	ENSG00000170852	0,114544011	0,041785411	0,077418138
SON	protein_coding	ENSG00000159140	-0,114536868	0,041782944	0,077418138
ARHGEF10	protein_coding	ENSG00000104728	-0,35740661	0,041818235	0,077471953
GATD3B	protein_coding	ENSG00000280071	-0,345997956	0,041832396	0,077491189
PCNX1	protein_coding	ENSG00000100731	0,140068795	0,041879735	0,077571874
EHBP1L1	protein_coding	ENSG00000173442	-0,188420304	0,041899296	0,077601098
PHC3	protein_coding	ENSG00000173889	0,146698266	0,041942166	0,077673483
NAP1L4P1	processed_pseudogene	ENSG00000177173	-0,447646402	0,041971323	0,077720462
GSN	protein_coding	ENSG00000148180	0,140533609	0,041983855	0,077736649
AC093904.3	lincRNA	ENSG00000244310	0,752205756	0,042008069	0,077774463
ATP2A1-AS1	antisense	ENSG00000260442	-0,297528122	0,042024087	0,077797095
FAM199X	protein_coding	ENSG00000123575	0,106524462	0,042028585	0,0777984
YAP1	protein_coding	ENSG00000137693	-0,093209316	0,042053072	0,077832781
DTX1	protein_coding	ENSG00000135144	-0,47255709	0,042054748	0,077832781
SLC37A4	protein_coding	ENSG00000137700	-0,146734807	0,042063449	0,07784186
AL356056.1	lincRNA	ENSG00000229227	0,733156089	0,04207998	0,077865427
AL358115.1	antisense	ENSG00000236364	0,658403988	0,042088577	0,077867283
SMAD5	protein_coding	ENSG00000113658	-0,109631284	0,042085721	0,077867283
NUTM2D	protein_coding	ENSG00000214562	0,581070296	0,042097253	0,07787403
FOXP4	protein_coding	ENSG00000137166	0,192741087	0,042099817	0,07787403
TBC1D17	protein_coding	ENSG00000104946	0,186856802	0,042107495	0,077881208
RPSAP19	processed_pseudogene	ENSG00000183298	-0,266194103	0,042225103	0,078084863
MYO15B	protein_coding	ENSG00000266714	0,661317195	0,042227879	0,078084863
RBM6	protein_coding	ENSG00000004534	-0,134085599	0,042229025	0,078084863
RB1-DT	lincRNA	ENSG00000231473	-0,716548032	0,04226458	0,078138234
REC8	protein_coding	ENSG00000100918	0,501896655	0,042265508	0,078138234
SEC31B	protein_coding	ENSG00000075826	0,371363958	0,042280383	0,078151646
GTF2IP5	unprocessed_pseudoge	ENSG00000224316	0,700347583	0,042277713	0,078151646
AC124319.1	protein_coding	ENSG00000173821	-0,178671931	0,042315764	0,078209995
LINC01998	lincRNA	ENSG00000243321	0,703004659	0,042335042	0,078233753
PCDHB13	protein_coding	ENSG00000187372	-0,585935251	0,042336247	0,078233753
AL031848.2	antisense	ENSG00000271746	-0,752666381	0,042347293	0,078247115
ZNF529	protein_coding	ENSG00000186020	-0,121223582	0,042367445	0,078277299
PDE3B	protein_coding	ENSG00000152270	-0,260432828	0,04237442	0,078283133
DDA1	protein_coding	ENSG00000130311	0,110245261	0,042395349	0,078314745
TTF1	protein_coding	ENSG00000125482	-0,138294001	0,042401702	0,078319427
LRRC36	protein_coding	ENSG00000159708	0,780927284	0,042457822	0,078416022
RAPSN	protein_coding	ENSG00000165917	0,744615197	0,042464184	0,07842071
GCLM	protein_coding	ENSG00000023909	-0,151560479	0,042480273	0,07844336

FAM21FP	transcribed_unprocesse	ENSG00000237840	0,638622746	0,042529807	0,078527759
COMMD1	protein_coding	ENSG00000173163	0,204903276	0,042544417	0,078547663
CARD19	protein_coding	ENSG00000165233	-0,206145428	0,0425628	0,078574529
CSAG1	protein_coding	ENSG00000198930	0,163498187	0,042623881	0,078680209
SYNGR2	protein_coding	ENSG00000108639	-0,119387217	0,042666085	0,078751027
KCNQ4	protein_coding	ENSG00000117013	0,749366634	0,042703076	0,07881221
AC016722.1	lincRNA	ENSG00000226548	0,787510365	0,042744959	0,078873072
MRPL51	protein_coding	ENSG00000111639	-0,149759572	0,04274759	0,078873072
ZNF367	protein_coding	ENSG00000165244	-0,193828134	0,042742764	0,078873072
RRP12	protein_coding	ENSG00000052749	0,148528858	0,042802427	0,078967147
SNF8	protein_coding	ENSG00000159210	0,111604328	0,042806873	0,078968247
AP001469.3	antisense	ENSG00000239415	-0,717574909	0,042822385	0,078989758
H19	processed_transcript	ENSG00000130600	-0,775071257	0,042859664	0,079044305
MMP17	protein_coding	ENSG00000198598	0,299582593	0,04285584	0,079044305
ACO2	protein_coding	ENSG00000100412	0,083801071	0,042907963	0,079126265
MIPEP	protein_coding	ENSG00000027001	-0,143781847	0,042922891	0,079146677
DHX57	protein_coding	ENSG00000163214	-0,110927196	0,042938548	0,07916843
RPS10P7	transcribed_processed_	ENSG00000223396	0,59388349	0,042984862	0,079246699
TMPRSS11D	protein_coding	ENSG00000153802	0,745726891	0,042995477	0,079259144
USP14	protein_coding	ENSG00000101557	0,139496211	0,043073617	0,079396053
NEXN	protein_coding	ENSG00000162614	0,773810678	0,043132092	0,079496694
AC097717.1	processed_transcript	ENSG00000232732	0,769984727	0,043172954	0,07955056
CDS1	protein_coding	ENSG00000163624	0,779287234	0,043169483	0,07955056
RPS7	protein_coding	ENSG00000171863	-0,167375312	0,043169353	0,07955056
SMIM15-AS1	antisense	ENSG00000251279	0,75351551	0,043251103	0,07968024
CFAP157	protein_coding	ENSG00000160401	0,711370178	0,043248896	0,07968024
AC003102.1	antisense	ENSG00000260793	0,648588476	0,043389455	0,079927944
AC005034.3	antisense	ENSG00000270696	-0,205917628	0,043478363	0,080084529
LINC02273	lincRNA	ENSG00000245954	0,77493945	0,04348481	0,080089211
AC092431.2	processed_pseudogene	ENSG00000232228	0,768555329	0,043546583	0,080189104
DIP2C	protein_coding	ENSG00000151240	-0,152433982	0,043546867	0,080189104
AC138409.2	transcribed_unprocesse	ENSG00000215158	-0,275365582	0,043570381	0,080225202
ANKRD7	protein_coding	ENSG00000106013	0,480602487	0,043584142	0,080243335
AL096870.2	processed_transcript	ENSG00000260669	0,729112085	0,043602343	0,08026964
AC027559.1	transcribed_unprocesse	ENSG00000259658	-0,418277402	0,043621242	0,080297225
ST20	protein_coding	ENSG00000180953	0,333815327	0,043647647	0,080338619
ELL2P1	processed_pseudogene	ENSG00000227295	0,748659964	0,043688802	0,080407154
POLR2J4	transcribed_unprocesse	ENSG00000272655	-0,387511948	0,043739569	0,080493365
ASMTL	protein_coding	ENSG00000169093	-0,171378908	0,043749731	0,080500681
MN1	protein_coding	ENSG00000169184	-0,432533065	0,043751394	0,080500681

CD63	protein_coding	ENSG00000135404	0,117351736	0,043756195	0,080502293
RBM18	protein_coding	ENSG00000119446	0,122413682	0,043778329	0,080535791
UBE2E1	protein_coding	ENSG00000170142	0,131194683	0,043789796	0,080549661
ZMPSTE24	protein_coding	ENSG00000084073	0,131744686	0,043800176	0,080561529
AC067956.1	lincRNA	ENSG00000236451	0,748940107	0,04388155	0,080703962
AC092295.2	antisense	ENSG00000267309	-0,485880174	0,04389382	0,080719291
CLINT1	protein_coding	ENSG00000113282	0,090793962	0,043995375	0,080894185
SERTAD3	protein_coding	ENSG00000167565	-0,142824839	0,043996813	0,080894185
EIF2B1	protein_coding	ENSG00000111361	-0,096257361	0,044022216	0,080933636
ACAT2	protein_coding	ENSG00000120437	-0,126427281	0,044045092	0,080968436
RANBP9	protein_coding	ENSG00000010017	0,112405194	0,044071928	0,081010508
AC083906.5	unprocessed_pseudoge	ENSG00000284731	-0,590633075	0,044163367	0,08117131
AL391832.3	lincRNA	ENSG00000258082	0,527832653	0,04418141	0,081197197
UEVLD	protein_coding	ENSG00000151116	0,120964378	0,04419513	0,081215135
MAP3K14-AS1	antisense	ENSG00000267278	0,70916043	0,044234106	0,081279476
AC073343.1	unprocessed_pseudoge	ENSG00000198580	0,737758014	0,044256873	0,081314026
AC010809.1	antisense	ENSG00000259287	0,643942259	0,044282172	0,081353219
PRUNE2	protein_coding	ENSG00000106772	0,764412882	0,044288845	0,081358191
KLC4	protein_coding	ENSG00000137171	0,16913357	0,044294528	0,081361345
VCPKMT	protein_coding	ENSG00000100483	0,200564019	0,04430359	0,081370702
RPS21	protein_coding	ENSG00000171858	-0,164267085	0,044378088	0,081499406
ACTG1P17	transcribed_processed_	ENSG00000259315	0,627153022	0,044381612	0,081499406
TMEM11	protein_coding	ENSG00000178307	-0,118010551	0,04439388	0,081514635
AC019211.1	lincRNA	ENSG00000239498	0,686626667	0,04440597	0,081529535
RN7SL600P	misc_RNA	ENSG00000274963	0,717863552	0,04445709	0,081608781
RNPEP	protein_coding	ENSG00000176393	-0,093110979	0,044490624	0,08166303
NUCB1	protein_coding	ENSG00000104805	0,117331686	0,044494791	0,08166337
AC005674.2	sense_overlapping	ENSG00000261490	0,408459369	0,044585601	0,081822716
HIST2H2BD	transcribed_unprocesse	ENSG00000220323	0,740295246	0,044600685	0,081843074
ST18	protein_coding	ENSG00000147488	0,570468879	0,044622467	0,081875719
AC091544.4	processed_transcript	ENSG00000260337	0,769285633	0,044686778	0,081986388
INAFM1	protein_coding	ENSG00000257704	0,34340555	0,044712607	0,082026438
AL157400.4	lincRNA	ENSG00000240996	0,588577896	0,04481466	0,082206303
STAMBP	protein_coding	ENSG00000124356	0,102307264	0,044849771	0,082263352
NR3C1	protein_coding	ENSG00000113580	-0,092208408	0,044912504	0,08237105
CDKN2D	protein_coding	ENSG00000129355	-0,175690004	0,044955471	0,082442481
LINC01521	lincRNA	ENSG00000213888	-0,377052516	0,045039861	0,082582474
AC134043.1	antisense	ENSG00000253256	0,739837651	0,045056839	0,082606218
LINC01778	lincRNA	ENSG00000223382	0,711440509	0,045084476	0,082649499
ZNF277	protein_coding	ENSG00000198839	0,150427368	0,045142545	0,082748557

AC079807.1	antisense	ENSG00000233230	0,590492232	0,045215485	0,082867447
SRP9	protein_coding	ENSG00000143742	-0,157330248	0,045215294	0,082867447
AC019193.2	lincRNA	ENSG00000272784	0,70603897	0,045254094	0,082930797
SLC25A6	protein_coding	ENSG00000169100	-0,136504821	0,045291299	0,082991561
AL031985.3	sense_overlapping	ENSG00000260920	0,29555639	0,045331356	0,083057542
MCEE	protein_coding	ENSG00000124370	0,242309622	0,045340838	0,083060085
LIMK1	protein_coding	ENSG00000106683	-0,124651624	0,045344894	0,083060085
SLC6A6	protein_coding	ENSG00000131389	-0,12713212	0,045341865	0,083060085
SNORA3B	snoRNA	ENSG00000212607	-0,511862502	0,045353819	0,083069014
AL117379.1	lincRNA	ENSG00000273759	-0,741105036	0,045369943	0,083091127
C8orf88	protein_coding	ENSG00000253250	-0,15200554	0,045413767	0,083163961
KCNMB2-AS1	antisense	ENSG00000237978	0,619451026	0,045421038	0,083169849
FAM122B	protein_coding	ENSG00000156504	-0,129410023	0,045450251	0,083215913
AC005479.1	lincRNA	ENSG00000259005	0,681687705	0,045498032	0,083295959
UBA3	protein_coding	ENSG00000144744	0,114354957	0,04553914	0,083363778
AKAP2	protein_coding	ENSG00000241978	-0,419586433	0,04562762	0,083518295
ZNF396	protein_coding	ENSG00000186496	0,524066405	0,045635353	0,083524994
BAZ2A	protein_coding	ENSG00000076108	0,150004584	0,045688422	0,083614664
ZNF133	protein_coding	ENSG00000125846	0,165540697	0,045711136	0,083648768
RF00019	misc_RNA	ENSG00000202272	0,766445857	0,045838739	0,083873154
AL109741.2	processed_pseudogene	ENSG00000228399	0,445361486	0,045841934	0,083873154
ATRNL1	protein_coding	ENSG00000107518	-0,193684903	0,04587052	0,08391797
RGS18	protein_coding	ENSG00000150681	0,755063171	0,04588685	0,083939474
COG8	protein_coding	ENSG00000213380	-0,284317779	0,045890459	0,083939474
PPP2CA	protein_coding	ENSG00000113575	0,08887819	0,045895992	0,083942108
ALDH1L1-AS2	antisense	ENSG00000246022	0,626411648	0,045975992	0,084080927
ZNF708	protein_coding	ENSG00000182141	0,326350242	0,045987507	0,084094487
PLCB3	protein_coding	ENSG00000149782	-0,126024866	0,046100264	0,084293164
HRK	protein_coding	ENSG00000135116	-0,526360052	0,046153633	0,084383224
AC020893.2	processed_pseudogene	ENSG00000251467	0,720848606	0,046182387	0,084428269
DOK1	protein_coding	ENSG00000115325	-0,23587385	0,046211644	0,084474226
AC011726.2	sense_intronic	ENSG00000253430	0,734516045	0,046250917	0,084538482
GPR61	protein_coding	ENSG00000156097	0,768233868	0,046425901	0,084850761
AC004982.2	lincRNA	ENSG00000272894	0,507420057	0,046499211	0,084977173
EEF1E1	protein_coding	ENSG00000124802	-0,219355671	0,046533083	0,085023923
LRRC37A9P	processed_pseudogene	ENSG00000271013	0,629043668	0,046589	0,085105131
GAN	protein_coding	ENSG00000261609	0,137479546	0,046584505	0,085105131
PPP2R1A	protein_coding	ENSG00000105568	-0,120639568	0,046589976	0,085105131
GRPEL2	protein_coding	ENSG00000164284	-0,116927354	0,046604824	0,085124671
SCAMP1-AS1	lincRNA	ENSG00000245556	0,189435693	0,046649333	0,085190795

AC108693.1	processed_pseudogene	ENSG00000239280	-0,61876155	0,046645352	0,085190795
STAT6	protein_coding	ENSG00000166888	0,107830539	0,046659204	0,085201235
HTR1D	protein_coding	ENSG00000179546	0,208880736	0,046688683	0,085247476
CARMIL1	protein_coding	ENSG00000079691	-0,097768422	0,046722315	0,08530129
C2orf16	protein_coding	ENSG00000221843	0,479366586	0,046837327	0,085496046
AC068888.1	antisense	ENSG00000257337	0,408357415	0,046844595	0,085501622
GSTZ1	protein_coding	ENSG00000100577	-0,179036459	0,046848719	0,085501622
PRKCI	protein_coding	ENSG00000163558	0,1094827	0,046870506	0,085533774
AL354710.2	antisense	ENSG00000239705	0,685463405	0,046908273	0,085595078
GGCX	protein_coding	ENSG00000115486	-0,0983495	0,046913343	0,085596714
RHOQP2	processed_pseudogene	ENSG00000232742	0,741074761	0,046921327	0,085603667
AC073575.2	antisense	ENSG00000274227	0,661576493	0,046966538	0,085664324
SCN3B	protein_coding	ENSG00000166257	0,733510836	0,046973737	0,085664324
TENT5C	protein_coding	ENSG00000183508	0,723880759	0,046973181	0,085664324
FUNDC2	protein_coding	ENSG00000165775	-0,149963465	0,046975458	0,085664324
NLRP2	protein_coding	ENSG00000022556	-0,256663199	0,04696481	0,085664324
EPC2	protein_coding	ENSG00000135999	0,117758642	0,046985193	0,085674458
LRRC4B	protein_coding	ENSG00000131409	0,645246404	0,04701922	0,085728883
AL354707.1	lincRNA	ENSG00000225489	0,691945556	0,047199813	0,086042856
FDX1	protein_coding	ENSG00000137714	0,115354584	0,047199707	0,086042856
HAUS2	protein_coding	ENSG00000137814	-0,109528003	0,047227286	0,086085286
PSMA3	protein_coding	ENSG00000100567	-0,146228612	0,04729094	0,086193654
NMNAT3	protein_coding	ENSG00000163864	-0,349340633	0,047306901	0,086215082
DMBX1	protein_coding	ENSG00000197587	-0,387642594	0,047327649	0,086245231
SNX11	protein_coding	ENSG00000002919	0,12678879	0,047346808	0,086272479
AP2M1	protein_coding	ENSG00000161203	0,090797047	0,04740471	0,086370311
TNFSF8	protein_coding	ENSG00000106952	0,756785191	0,047422804	0,086395604
SSR1P2	processed_pseudogene	ENSG00000226665	0,737457524	0,047440894	0,086420884
ERICH1	protein_coding	ENSG00000104714	-0,151892713	0,047470978	0,086468007
OR7E47P	transcribed_unprocessed	ENSG00000257542	0,73253293	0,047666371	0,086816204
YBX1P10	processed_pseudogene	ENSG00000213866	0,365459097	0,047690032	0,086847227
STMN2	protein_coding	ENSG00000104435	0,632631284	0,047691873	0,086847227
PDRG1	protein_coding	ENSG00000088356	0,160957195	0,047721431	0,086893337
POR	protein_coding	ENSG00000127948	0,165401967	0,047823277	0,087063325
ARHGEF11	protein_coding	ENSG00000132694	-0,135588693	0,047822344	0,087063325
MDFIC	protein_coding	ENSG00000135272	-0,148599963	0,047845051	0,087095233
SETBP1	protein_coding	ENSG00000152217	-0,235103932	0,047890839	0,087170847
ZNF354B	protein_coding	ENSG00000178338	-0,206997925	0,04794533	0,087262287
SGK3	protein_coding	ENSG00000104205	0,248448171	0,047959934	0,087281122
SGK3	protein_coding	ENSG00000104205	0,248448171	0,047959934	0,087281122

COPG2	protein_coding	ENSG00000158623	-0,099988877	0,047971981	0,0872953
SLC22A20P	transcribed_unitary_pse	ENSG00000197847	0,597362974	0,047977515	0,087297626
EIF3H	protein_coding	ENSG00000147677	0,109807909	0,048007273	0,087344023
AP005137.2	antisense	ENSG00000272703	0,654947909	0,048018348	0,087356424
PILRA	protein_coding	ENSG00000085514	0,578424392	0,048067879	0,087438775
AF131216.4	antisense	ENSG00000280273	0,668347479	0,048155019	0,087589522
DAP3	protein_coding	ENSG00000132676	0,087250129	0,048164177	0,08759841
LINC00680	transcribed_unprocesse	ENSG00000215190	0,200469672	0,04819831	0,087652717
AL109614.1	sense_intronic	ENSG00000276603	0,744227669	0,048286778	0,087805817
AL606760.1	antisense	ENSG00000226754	0,674844872	0,048304684	0,087830591
RNF157	protein_coding	ENSG00000141576	0,724486342	0,048321185	0,087852807
AC090377.1	lincRNA	ENSG00000267462	0,739214	0,048377403	0,087947221
LINC01693	lincRNA	ENSG00000227764	0,731953191	0,048429934	0,088034915
CHKB-DT	antisense	ENSG00000205559	-0,325739497	0,048502229	0,088158518
DDAH1	protein_coding	ENSG00000153904	0,102311532	0,04853095	0,088202904
AC074032.1	antisense	ENSG00000272368	0,627582003	0,048548898	0,088212074
KDELR3	protein_coding	ENSG00000100196	-0,126074658	0,048547518	0,088212074
ATP9A	protein_coding	ENSG00000054793	-0,132471534	0,048543839	0,088212074
HCFC2	protein_coding	ENSG00000111727	0,155278808	0,048714202	0,088496043
MXD4	protein_coding	ENSG00000123933	-0,169948431	0,048718129	0,088496043
RF00413	snoRNA	ENSG00000222588	-0,742346636	0,048717176	0,088496043
GPN2	protein_coding	ENSG00000142751	0,115418504	0,048755206	0,088555551
PTPN9	protein_coding	ENSG00000169410	-0,094128054	0,048784727	0,088601324
DTWD1	protein_coding	ENSG00000104047	-0,16181664	0,048832805	0,088680789
CNOT3	protein_coding	ENSG00000088038	0,131776862	0,048961284	0,088906236
TRMT9B	protein_coding	ENSG00000250305	0,255575072	0,04897035	0,088914826
CMAHP	transcribed_unitary_pse	ENSG00000168405	0,441761611	0,048988193	0,08893935
AL137002.2	antisense	ENSG00000283828	0,38174569	0,049005503	0,088962903
RAP2C	protein_coding	ENSG00000123728	0,138038457	0,049059158	0,089052423
MEGF10	protein_coding	ENSG00000145794	0,726809724	0,049093497	0,089106868
ACCS	protein_coding	ENSG00000110455	0,250177998	0,049131353	0,089162841
LAMTOR4	protein_coding	ENSG00000188186	-0,167947453	0,049133029	0,089162841
CEP57	protein_coding	ENSG00000166037	0,112675626	0,049139493	0,089166681
AP000919.4	antisense	ENSG00000272625	0,622091115	0,049159233	0,08918672
SUZ12	protein_coding	ENSG00000178691	-0,132055656	0,049156355	0,08918672
TBX1	protein_coding	ENSG00000184058	-0,720557963	0,049176062	0,08920936
DHRS4L1	transcribed_unprocesse	ENSG00000225766	0,627458678	0,04919026	0,089227225
MTIF3	protein_coding	ENSG00000122033	-0,147773203	0,049229771	0,089290998
AL451042.1	antisense	ENSG00000224621	0,672322816	0,049289039	0,089390591
ATP5F1A	protein_coding	ENSG00000152234	-0,103019728	0,049298669	0,08940015

AC005479.2	lincRNA	ENSG00000270000	0,737701369	0,049308827	0,089410666
GRK3	protein_coding	ENSG00000100077	0,143795186	0,049401045	0,089569963
NAA10	protein_coding	ENSG00000102030	-0,142094198	0,049480174	0,089705503
C21orf62-AS1	antisense	ENSG00000205930	0,614834426	0,049494028	0,08972269
AL034550.1	antisense	ENSG00000236772	0,727539212	0,049511725	0,089744628
LINC02028	processed_transcript	ENSG00000230102	0,728259561	0,049514881	0,089744628
VWA8-AS1	antisense	ENSG00000278338	0,643910147	0,049724622	0,090116815
DRAIC	lincRNA	ENSG00000245750	0,477369569	0,049790667	0,090228537
RER1	protein_coding	ENSG00000157916	-0,105295699	0,049797224	0,090232448
BCLAF1	protein_coding	ENSG00000029363	0,116040712	0,049832877	0,090285821
MAGEA12	protein_coding	ENSG00000213401	-0,12359384	0,049835484	0,090285821
WBP1L	protein_coding	ENSG00000166272	-0,094225489	0,049905683	0,090405012
CFAP69	protein_coding	ENSG00000105792	0,37701591	0,049975853	0,090524132
CMTM8	protein_coding	ENSG00000170293	-0,418497798	0,050036194	0,090625427
AC234582.1	antisense	ENSG00000231064	-0,57480663	0,050047637	0,090638149
HSPB1	protein_coding	ENSG00000106211	0,153157388	0,050081059	0,090690669
GRAMD4	protein_coding	ENSG00000075240	-0,145771762	0,050123687	0,09075985
RF00493	snoRNA	ENSG00000201882	-0,287672838	0,050148802	0,090797311
LINC00643	transcribed_unitary_pse	ENSG00000186369	0,585467208	0,050167776	0,090823646
IFNAR2	protein_coding	ENSG00000159110	0,147202214	0,050207939	0,090888334
MT-ND4	protein_coding	ENSG00000198886	0,416400885	0,050221672	0,09089229
LRRC8E	protein_coding	ENSG00000171017	0,139698108	0,050223419	0,09089229
STRN3	protein_coding	ENSG00000196792	0,08326373	0,050223123	0,09089229
C15orf65	protein_coding	ENSG00000261652	0,372690347	0,050258091	0,090947013
AL133383.1	lincRNA	ENSG00000261729	0,656125123	0,050352933	0,0911106
ZIK1	protein_coding	ENSG00000171649	-0,114009035	0,050449789	0,091277803
ANXA8L1	protein_coding	ENSG00000264230	-0,263751551	0,050480833	0,091325913
SPTSSA	protein_coding	ENSG00000165389	0,209941591	0,050557368	0,091456308
DLX2-DT	lincRNA	ENSG00000236651	0,633784919	0,050590735	0,091508596
ATP2A3	protein_coding	ENSG00000074370	0,209868075	0,050637096	0,091584378
COL8A2	protein_coding	ENSG00000171812	0,70567744	0,05068409	0,091645127
ATG12	protein_coding	ENSG00000145782	0,113925618	0,050677165	0,091645127
CCDC9B	protein_coding	ENSG00000188549	-0,165725316	0,05067966	0,091645127
AC087636.1	lincRNA	ENSG00000258773	-0,346406079	0,05070965	0,091683262
AP001330.1	lincRNA	ENSG00000253282	0,60319398	0,050738317	0,091727007
FAM135A	protein_coding	ENSG00000082269	0,157550434	0,050762069	0,091761857
TMEM198	protein_coding	ENSG00000188760	0,381381829	0,050770664	0,091766019
GTF2H1	protein_coding	ENSG00000110768	0,106520567	0,05077332	0,091766019
SIK2	protein_coding	ENSG00000170145	-0,115263366	0,050793044	0,091793579
AC073476.3	sense_overlapping	ENSG00000261096	-0,713956384	0,05080663	0,091810043

RAPGEFL1	protein_coding	ENSG00000108352	0,263126085	0,050928241	0,092021692
AC131009.1	lincRNA	ENSG00000255992	-0,620324638	0,050946281	0,09204618
LINC02447	lincRNA	ENSG00000245468	0,713903207	0,05095553	0,092054781
AC002116.2	antisense	ENSG00000267698	-0,36666923	0,050984063	0,092098215
RBPMS	protein_coding	ENSG00000157110	0,224113728	0,051006689	0,092120519
ARMC10	protein_coding	ENSG00000170632	-0,111439615	0,051009885	0,092120519
PLEKHG4B	protein_coding	ENSG00000153404	-0,236176638	0,051007854	0,092120519
TPO	protein_coding	ENSG00000115705	0,634368754	0,051121419	0,092313815
ANKLE1	protein_coding	ENSG00000160117	0,6047703	0,051169118	0,092391814
ARMCX1	protein_coding	ENSG00000126947	-0,094986086	0,051176771	0,092397498
AC068860.1	TEC	ENSG00000279266	0,719243395	0,051203822	0,092438201
LINC00402	lincRNA	ENSG00000235532	0,698277416	0,051216946	0,092453754
AL132656.4	TEC	ENSG00000280355	0,382534441	0,051227905	0,092465398
AC106782.1	transcribed_unprocesse	ENSG00000258130	0,710821902	0,051262968	0,092520544
PPP1R3G	protein_coding	ENSG00000219607	-0,547334229	0,051273422	0,09253127
MT-ATP6	protein_coding	ENSG00000198899	0,449592187	0,051298017	0,09256751
HOXC9	protein_coding	ENSG00000180806	0,22118685	0,051321769	0,092602224
SLC16A5	protein_coding	ENSG00000170190	0,183043096	0,051413166	0,092758975
GON7	protein_coding	ENSG00000170270	-0,214270113	0,051459493	0,092826227
AL353751.1	antisense	ENSG00000232110	0,711529593	0,051474933	0,092845913
RBM10	protein_coding	ENSG00000182872	-0,104219162	0,051627401	0,093112733
AC015921.1	antisense	ENSG00000262884	0,716031092	0,051686813	0,093203494
DNTTIP2	protein_coding	ENSG00000067334	-0,12419247	0,051696899	0,093213486
WASHC5	protein_coding	ENSG00000164961	-0,086303196	0,05173379	0,093271804
AC068722.2	lincRNA	ENSG00000273972	0,703449733	0,051749125	0,093284566
RPL9	protein_coding	ENSG00000163682	-0,163079435	0,051749966	0,093284566
SNX22	protein_coding	ENSG00000157734	-0,408791607	0,051757084	0,093289199
VPREB3	protein_coding	ENSG00000128218	0,700258796	0,051774188	0,093311827
SGMS1-AS1	antisense	ENSG00000226200	0,330956371	0,051781692	0,093317152
LYPLAL1-DT	antisense	ENSG00000228063	0,592197585	0,05180607	0,093352881
DHRS11	protein_coding	ENSG00000278535	-0,155823097	0,051811745	0,093354906
ANKRD53	protein_coding	ENSG00000144031	0,658122162	0,051862338	0,093437856
PPP1R7	protein_coding	ENSG00000115685	0,091783925	0,05189328	0,09348539
MHENCR	antisense	ENSG00000232442	-0,331550134	0,051932597	0,093548003
BRICD5	protein_coding	ENSG00000182685	0,560853414	0,051944449	0,09355292
HERPUD2	protein_coding	ENSG00000122557	0,103764342	0,051942364	0,09355292
INO80	protein_coding	ENSG00000128908	-0,123483621	0,051962004	0,093576319
UBAP1L	protein_coding	ENSG00000246922	0,472289788	0,051975871	0,093593075
CHCHD2P2	processed_pseudogene	ENSG00000215006	-0,50637098	0,052017707	0,093660186
IP6K1	protein_coding	ENSG00000176095	-0,112220872	0,052055191	0,093719449

SPACA6	protein_coding	ENSG00000182310	0,34634099	0,052160991	0,093901687
NAMPTP1	processed_pseudogene	ENSG00000229644	0,44205198	0,05217338	0,093911704
ASB3	protein_coding	ENSG00000115239	0,327239109	0,052175713	0,093911704
NECTIN1	protein_coding	ENSG00000110400	-0,277420892	0,052302124	0,094130973
CAPN12	protein_coding	ENSG00000182472	0,603914783	0,052357967	0,094223208
SLC39A7	protein_coding	ENSG00000112473	-0,126934594	0,052386006	0,094265397
AL136162.1	lincRNA	ENSG00000271888	-0,465860173	0,052423814	0,094325153
MT-ND5	protein_coding	ENSG00000198786	0,41261392	0,05250948	0,094471004
AC083949.1	antisense	ENSG00000224875	0,683233991	0,052547956	0,094531934
AC068025.1	antisense	ENSG00000264808	0,54877634	0,052588484	0,094595827
MRPS18A	protein_coding	ENSG00000096080	-0,124147476	0,052623166	0,09464233
OTUD5	protein_coding	ENSG00000068308	0,101534297	0,052658256	0,094697135
ZNF358	protein_coding	ENSG00000198816	0,251677199	0,052699874	0,094763669
AC099535.1	processed_pseudogene	ENSG00000230807	0,679968178	0,052708256	0,094770433
DPEP1	protein_coding	ENSG00000015413	0,700748751	0,05272883	0,094799115
PDLIM5	protein_coding	ENSG00000163110	0,068890389	0,052785981	0,094893547
NRROS	protein_coding	ENSG00000174004	0,700530929	0,05283609	0,094975302
GALM	protein_coding	ENSG00000143891	0,635024845	0,052855299	0,095001505
ABCF1	protein_coding	ENSG00000204574	-0,102642518	0,052891005	0,095057352
UBB	protein_coding	ENSG00000170315	-0,132100717	0,052982306	0,095213098
ZDHHC20P4	processed_pseudogene	ENSG00000232380	0,55651369	0,052999335	0,095233829
BTF3	protein_coding	ENSG00000145741	-0,124080476	0,053003128	0,095233829
ITK	protein_coding	ENSG00000113263	0,64290193	0,053072858	0,095350763
PSMB11	protein_coding	ENSG00000222028	0,705084035	0,053092683	0,095378026
ZNF250	protein_coding	ENSG00000196150	0,163920443	0,053110634	0,095401918
THSD1	protein_coding	ENSG00000136114	0,138647056	0,05311603	0,095403256
SNHG7	antisense	ENSG00000233016	0,13724401	0,053133526	0,095426324
PON2	protein_coding	ENSG00000105854	-0,13402302	0,053162905	0,095470728
AC006141.1	sense_intronic	ENSG00000264895	-0,595622968	0,053185022	0,095500585
AC135506.1	unprocessed_pseudogene	ENSG00000270441	0,601538763	0,053188844	0,095500585
NAT9	protein_coding	ENSG00000109065	0,157413977	0,053198116	0,095508873
DUS3L	protein_coding	ENSG00000141994	-0,122456168	0,053247698	0,095589522
EHD3	protein_coding	ENSG00000013016	-0,138815503	0,053269818	0,095620863
ZNF540	protein_coding	ENSG00000171817	0,5278819	0,053297705	0,095662547
NDUFA8	protein_coding	ENSG00000119421	-0,126176523	0,053313519	0,095682558
GATA6-AS1	antisense	ENSG00000266010	0,683023672	0,053338627	0,095719243
AC002454.1	antisense	ENSG00000237819	0,639563977	0,053349671	0,095730686
COQ8B	protein_coding	ENSG00000123815	0,180154365	0,053374957	0,095767682
AC087501.4	sense_intronic	ENSG00000271851	-0,339110414	0,053382528	0,095772887
AL645728.1	processed_transcript	ENSG00000215014	0,512374279	0,053416292	0,095825081

NR3C2	protein_coding	ENSG00000151623	0,196412355	0,053436525	0,095852994
VPS13D	protein_coding	ENSG00000048707	-0,117436466	0,053451541	0,095871544
RN7SL242P	misc_RNA	ENSG00000244389	-0,716450361	0,053468257	0,095876373
DNAI1	protein_coding	ENSG00000122735	0,683126406	0,053465862	0,095876373
EPHA5	protein_coding	ENSG00000145242	0,584063377	0,053459991	0,095876373
VWA5B2	protein_coding	ENSG00000145198	0,642086006	0,053482109	0,095892827
VEZT	protein_coding	ENSG00000028203	0,076247673	0,053613694	0,096120354
SCN9A	protein_coding	ENSG00000169432	0,12047788	0,053724988	0,096311467
HSPB1P2	processed_pseudogene	ENSG00000230216	0,705938514	0,05377078	0,096385133
AC090181.2	sense_intronic	ENSG00000269951	-0,476334467	0,053779799	0,096392875
ALDOC	protein_coding	ENSG00000109107	-0,384469045	0,053827146	0,096469308
CPNE9	protein_coding	ENSG00000144550	0,7030194	0,053846342	0,09648685
ARL2BP	protein_coding	ENSG00000102931	-0,084237094	0,053843366	0,09648685
AL121820.1	antisense	ENSG00000258623	0,658254025	0,053929579	0,096627558
KIRREL2	protein_coding	ENSG00000126259	-0,184840284	0,054033123	0,096804625
COX8A	protein_coding	ENSG00000176340	-0,124737601	0,054055443	0,096836155
ZNF844	protein_coding	ENSG00000223547	0,669236799	0,054147612	0,096984327
AC016251.1	TEC	ENSG00000279645	0,687478009	0,054145745	0,096984327
CC2D1A	protein_coding	ENSG00000132024	0,127331096	0,054229241	0,097122052
LCORL	protein_coding	ENSG00000178177	0,128060242	0,054332738	0,097298913
AC008972.1	lincRNA	ENSG00000271926	0,693063143	0,054349276	0,097320032
AL391280.1	processed_pseudogene	ENSG00000250995	0,700792283	0,05436873	0,097346369
ZBTB37	protein_coding	ENSG00000185278	-0,158067774	0,054408465	0,09740901
PRX	protein_coding	ENSG00000105227	-0,22755449	0,054424038	0,097428386
PIAS2	protein_coding	ENSG00000078043	0,104860597	0,05443583	0,097440991
POLK	protein_coding	ENSG00000122008	-0,130532839	0,054497627	0,097543095
TRIAP1	protein_coding	ENSG00000170855	-0,117056535	0,054512773	0,097561691
THRA	protein_coding	ENSG00000126351	-0,158971852	0,054562219	0,097641663
CLASRP	protein_coding	ENSG00000104859	-0,132892426	0,054598999	0,097698959
SNORD17	snoRNA	ENSG00000212232	-0,273720948	0,05463973	0,097763313
AL136452.1	processed_pseudogene	ENSG00000228997	0,30628947	0,054670912	0,097810572
KLHL3	protein_coding	ENSG00000146021	0,685496243	0,054727322	0,097902955
C1RL-AS1	antisense	ENSG00000205885	0,698498522	0,054747284	0,097930123
ZNF252P-AS1	antisense	ENSG00000255559	0,704288669	0,054755511	0,097936298
RPL5	protein_coding	ENSG00000122406	-0,128671141	0,054769596	0,097952949
NUDT14	protein_coding	ENSG00000183828	-0,173695608	0,054794374	0,097988718
MAGI2-AS3	processed_transcript	ENSG00000234456	0,153744389	0,054832045	0,098038989
KREMEN1	protein_coding	ENSG00000183762	-0,229220937	0,054830344	0,098038989
MATR3	protein_coding	ENSG00000015479	-0,287605795	0,05484022	0,098045058
SLC12A8	protein_coding	ENSG00000221955	0,338742803	0,054937655	0,098210695

CLDN15	protein_coding	ENSG00000106404	0,332794471	0,055019481	0,0983484
P2RY6	protein_coding	ENSG00000171631	0,653494875	0,055164404	0,098594238
NPM1	protein_coding	ENSG00000181163	-0,142852758	0,055166625	0,098594238
AC012447.1	antisense	ENSG00000265451	0,600215828	0,055179105	0,09860795
SPTBN4	protein_coding	ENSG00000160460	0,336582647	0,055231436	0,098692869
ESRRB	protein_coding	ENSG00000119715	0,683613327	0,055239787	0,098699192
AAR2	protein_coding	ENSG00000131043	-0,120640932	0,055250733	0,098710151
ZNF316	protein_coding	ENSG00000205903	0,206686724	0,055296577	0,09878345
ASL	protein_coding	ENSG00000126522	-0,175542883	0,055307243	0,0987939
MARK2	protein_coding	ENSG00000072518	0,155251246	0,055341499	0,098846481
SPHK2	protein_coding	ENSG00000063176	-0,170669333	0,055355442	0,098862776
TADA2B	protein_coding	ENSG00000173011	-0,131226268	0,055365888	0,098872823
ZNF782	protein_coding	ENSG00000196597	0,198935879	0,055379977	0,098889371
SNORA22B	snoRNA	ENSG00000206603	-0,575755713	0,055441402	0,098990437
ZNF106	protein_coding	ENSG00000103994	0,108104566	0,055453278	0,099003024
YIPF3	protein_coding	ENSG00000137207	0,113673096	0,055540376	0,099149892
NCBP2-AS1	antisense	ENSG00000225578	0,685517146	0,055579176	0,099210521
NPC1L1	protein_coding	ENSG00000015520	0,616015205	0,055641892	0,099304001
KIAA0319L	protein_coding	ENSG00000142687	0,111236256	0,055638848	0,099304001
TBC1D15	protein_coding	ENSG00000121749	-0,129500848	0,055650911	0,099304001
AL353743.1	transcribed_unprocesse	ENSG00000165121	0,256122777	0,055649712	0,099304001
PPP6C	protein_coding	ENSG00000119414	0,086043625	0,055684895	0,099355999
CECR2	protein_coding	ENSG00000099954	-0,355437785	0,055694084	0,09936375
SQLE	protein_coding	ENSG00000104549	0,090400144	0,055722423	0,099405662
AL022329.1	antisense	ENSG00000234884	0,640978346	0,05582258	0,099575675
TMCC1	protein_coding	ENSG00000172765	-0,111920241	0,055879261	0,099659447
SNORD37	snoRNA	ENSG00000206775	-0,645181709	0,055875754	0,099659447
DPP3	protein_coding	ENSG00000254986	0,106737384	0,05588803	0,09966642
HGFAC	protein_coding	ENSG00000109758	0,693591422	0,055917433	0,099697576
GTPBP10	protein_coding	ENSG00000105793	0,13265685	0,055924944	0,099697576
PRKAA1	protein_coding	ENSG00000132356	-0,129391046	0,05591551	0,099697576
OR4F13P	transcribed_unprocesse	ENSG00000214344	0,67083897	0,055924394	0,099697576
ADIRF	protein_coding	ENSG00000148671	0,616830743	0,055953529	0,099739865
DBP	protein_coding	ENSG00000105516	0,237296162	0,056010669	0,099833042
MYH7B	protein_coding	ENSG00000078814	0,663769834	0,056028022	0,099855294
MLX	protein_coding	ENSG00000108788	-0,113544826	0,056194209	0,100128918
YIF1A	protein_coding	ENSG00000174851	-0,1477996	0,056196196	0,100128918
FASTKD3	protein_coding	ENSG00000124279	-0,195231862	0,056194531	0,100128918
RPN2	protein_coding	ENSG00000118705	0,067276984	0,056210647	0,100145967
ADAMTSL3	protein_coding	ENSG00000156218	0,632856767	0,056277981	0,100257222

ASB16-AS1	antisense	ENSG00000267080	0,215620946	0,056305082	0,10029679
AP000229.1	lincRNA	ENSG00000273492	0,679972791	0,056325938	0,100325228
KLHDC4	protein_coding	ENSG00000104731	-0,129159326	0,056347794	0,10035544
MAD2L1BP	protein_coding	ENSG00000124688	0,119017308	0,056414144	0,100464885
MAGEA3	protein_coding	ENSG00000221867	-0,190900886	0,056435755	0,100494647
PRKG1	protein_coding	ENSG00000185532	0,682110764	0,056506288	0,100611509
SULT1A1	protein_coding	ENSG00000196502	0,632936556	0,056516259	0,100620526
SVOP	protein_coding	ENSG00000166111	0,664617415	0,056549304	0,100670621
DDX10P1	processed_pseudogene	ENSG00000237135	0,522112005	0,056589385	0,100733231
AC018638.8	transcribed_unprocesse	ENSG00000281896	0,508859277	0,056662416	0,100854479
XIRP2	protein_coding	ENSG00000163092	0,642902438	0,056712313	0,100934531
RHCG	protein_coding	ENSG00000140519	0,591501841	0,056740673	0,100976243
RABGEF1	protein_coding	ENSG00000154710	-0,264824447	0,056841179	0,101146328
AC005329.1	antisense	ENSG00000248015	0,469573198	0,05689083	0,101225898
TRAF3IP2-AS1	antisense	ENSG00000231889	0,288090209	0,056963269	0,101345996
PRKCD	protein_coding	ENSG00000163932	0,141934427	0,056969313	0,101347958
ZP3	protein_coding	ENSG00000188372	0,174245038	0,056996475	0,101387485
STOX2	protein_coding	ENSG00000173320	0,378288532	0,057040044	0,101456188
ESRP2	protein_coding	ENSG00000103067	0,682171451	0,057106269	0,101565174
AC103702.1	sense_intronic	ENSG00000257178	0,565940077	0,057114843	0,101571614
AL008718.3	sense_intronic	ENSG00000273353	-0,683099389	0,057135372	0,101599313
RCOR1	protein_coding	ENSG00000089902	0,138696911	0,057275002	0,101838778
MTX1	protein_coding	ENSG00000173171	-0,146005691	0,057291344	0,101859003
AC012462.3	antisense	ENSG00000230695	0,652926456	0,057362153	0,101976056
UQCRHL	protein_coding	ENSG00000233954	-0,161534709	0,057390489	0,102017587
HINT3	protein_coding	ENSG00000111911	0,160039632	0,057416363	0,102054736
ESYT3	protein_coding	ENSG00000158220	0,619943541	0,057453642	0,10211215
LMF1	protein_coding	ENSG00000103227	-0,180392706	0,057529615	0,102238316
CLDND1	protein_coding	ENSG00000080822	0,112069877	0,057600218	0,102354919
AL807752.1	processed_pseudogene	ENSG00000213590	-0,632600096	0,057606235	0,102356742
MAP2K2	protein_coding	ENSG00000126934	0,118114591	0,057654719	0,102427311
RAF1	protein_coding	ENSG00000132155	0,060871205	0,057655939	0,102427311
PPIAP31	processed_pseudogene	ENSG00000217094	-0,490937069	0,057706407	0,10250809
AGFG1	protein_coding	ENSG00000173744	0,087821985	0,057738623	0,102556435
SDHB	protein_coding	ENSG00000117118	-0,106799774	0,057830879	0,102711407
SNORA80B	snoRNA	ENSG00000206633	-0,352340997	0,0578608	0,102755651
HCCS	protein_coding	ENSG00000004961	0,112219733	0,057938352	0,102884467
UBE2I	protein_coding	ENSG00000103275	0,078254022	0,057955601	0,102906188
ADAM20	protein_coding	ENSG00000134007	0,601277942	0,057965579	0,102914995
LINC02246	lincRNA	ENSG00000281903	0,527708468	0,058022805	0,10300768

GORASP2	protein_coding	ENSG00000115806	0,075896805	0,058028766	0,103009346
ELK1	protein_coding	ENSG00000126767	-0,120042251	0,058058896	0,103053912
AC015849.5	lincRNA	ENSG00000270977	0,538396721	0,058090498	0,103101084
AC124068.2	antisense	ENSG00000261441	-0,517502565	0,058139941	0,103179907
UBQLNL	protein_coding	ENSG00000175518	0,664664839	0,058207103	0,10329016
CNOT8	protein_coding	ENSG00000155508	-0,1159584	0,058218662	0,103301735
CLUHP3	transcribed_unprocesse	ENSG00000131797	0,31360029	0,058362038	0,10354718
AC055720.2	3prime_overlapping_nc	ENSG00000256185	0,565415783	0,058442939	0,103681748
SPECC1L	protein_coding	ENSG00000100014	-0,120855497	0,058454463	0,103693222
MTG1	protein_coding	ENSG00000148824	-0,284775162	0,058550949	0,103855398
HSPD1P10	processed_pseudogene	ENSG00000216990	0,652708257	0,05857344	0,103886307
UCP3	protein_coding	ENSG00000175564	0,590082927	0,058658585	0,104028324
MAPK8IP1P2	processed_pseudogene	ENSG00000263503	0,669299163	0,058781105	0,104236595
FP565260.6	protein_coding	ENSG00000280433	0,374956063	0,058790266	0,104243827
MYNN	protein_coding	ENSG00000085274	0,10639111	0,058800906	0,104253681
OR7E99P	unprocessed_pseudoge	ENSG00000250710	0,597790371	0,05883925	0,104312648
POLB	protein_coding	ENSG00000070501	0,142270792	0,058919978	0,104446736
RPP25L	protein_coding	ENSG00000164967	-0,180757438	0,058933488	0,104461658
CNOT7	protein_coding	ENSG00000198791	-0,106680605	0,058965523	0,104509408
ADGRF3	protein_coding	ENSG00000173567	0,65666182	0,059046739	0,104644311
ROR1-AS1	antisense	ENSG00000223949	0,595489442	0,059128623	0,104771323
NUB1	protein_coding	ENSG00000013374	0,081451931	0,059126091	0,104771323
PARD6A	protein_coding	ENSG00000102981	-0,241157794	0,059192686	0,104875776
FLNA	protein_coding	ENSG00000196924	0,142694981	0,059240872	0,104952085
AC020912.1	lincRNA	ENSG00000284430	0,492903377	0,059275989	0,105005228
MADCAM1	protein_coding	ENSG00000099866	0,656169488	0,059317337	0,105060605
SPDYA	protein_coding	ENSG00000163806	0,547912704	0,059317494	0,105060605
AL356124.2	antisense	ENSG00000233351	0,659986354	0,059341781	0,105094546
ATP5F1C	protein_coding	ENSG00000165629	-0,11503493	0,059394318	0,105178506
CFDP1	protein_coding	ENSG00000153774	-0,082006921	0,059418226	0,10521176
AC116651.1	antisense	ENSG00000273133	0,579829956	0,059529697	0,105400042
AC008946.1	lincRNA	ENSG00000267939	0,378319819	0,059550904	0,105428489
AC022075.1	antisense	ENSG00000245648	0,633196268	0,059674056	0,105637397
LINC01842	lincRNA	ENSG00000267147	0,576176713	0,059699767	0,105673792
CNRIP1	protein_coding	ENSG00000119865	0,113839215	0,059736982	0,105730541
PRR34-AS1	antisense	ENSG00000241990	-0,243396929	0,059807218	0,105845719
EP400	protein_coding	ENSG00000183495	-0,145652179	0,059839991	0,105894583
FECH	protein_coding	ENSG00000066926	-0,091767024	0,059895176	0,105983096
DAD1	protein_coding	ENSG00000129562	0,120868846	0,059902573	0,10598704
AC036214.2	sense_intronic	ENSG00000272518	0,32745403	0,059963794	0,106086209

PGGT1B	protein_coding	ENSG00000164219	0,131743996	0,060332597	0,106720273
TMEM258	protein_coding	ENSG00000134825	-0,132008323	0,060331301	0,106720273
PDGFC	protein_coding	ENSG00000145431	0,106808162	0,060372223	0,106781157
AC087623.3	antisense	ENSG00000272159	0,598905447	0,060415455	0,106839195
KCND1	protein_coding	ENSG00000102057	0,437550872	0,060413204	0,106839195
AC139256.2	processed_transcript	ENSG00000270580	0,658441217	0,060429465	0,106854757
CDK5RAP3	protein_coding	ENSG00000108465	-0,321147128	0,060443659	0,106870642
TCAIM	protein_coding	ENSG00000179152	0,126057278	0,06049541	0,106943705
SNORD94	snoRNA	ENSG00000208772	-0,334503147	0,060491326	0,106943705
SMARCA2	protein_coding	ENSG00000080503	0,103589155	0,060511726	0,106963329
FANCM	protein_coding	ENSG00000187790	-0,11415583	0,060546153	0,10701496
GALK2	protein_coding	ENSG00000156958	0,093302904	0,060567176	0,107042894
RPL26	protein_coding	ENSG00000161970	-0,145516462	0,060646487	0,107173829
PCDHGB1	protein_coding	ENSG00000254221	-0,404815401	0,060658282	0,107185436
TIAL1	protein_coding	ENSG00000151923	0,09733931	0,060664817	0,10718775
FAM83B	protein_coding	ENSG00000168143	0,600367042	0,060805522	0,107427104
SERINC5	protein_coding	ENSG00000164300	-0,097341704	0,060818859	0,107441413
SLC35C1	protein_coding	ENSG00000181830	0,210954609	0,060884228	0,107547629
LCE2A	protein_coding	ENSG00000187173	-0,287659724	0,060958631	0,107669783
CAPRIN2	protein_coding	ENSG00000110888	0,13823811	0,060969291	0,107676363
SDHAF3	protein_coding	ENSG00000196636	-0,202254225	0,060972856	0,107676363
AC000123.2	TEC	ENSG00000279265	-0,642389296	0,060995428	0,10770695
AC139493.2	processed_transcript	ENSG00000283235	0,576346032	0,061111621	0,107902836
RNF115	protein_coding	ENSG00000265491	0,097449835	0,061118608	0,107905884
YAP1P1	processed_pseudogene	ENSG00000220494	0,56021042	0,061125847	0,107909375
TRUB1	protein_coding	ENSG00000165832	0,144170473	0,061148829	0,107940655
RANP1	processed_pseudogene	ENSG00000236603	-0,281253541	0,061184764	0,107990972
PFDN2	protein_coding	ENSG00000143256	-0,105884416	0,061187864	0,107990972
LINC02539	lincRNA	ENSG00000234956	0,535283726	0,061204231	0,108010564
EXTL1	protein_coding	ENSG00000158008	0,41900207	0,061217827	0,108017258
GLUD2	protein_coding	ENSG00000182890	-0,240917251	0,061218557	0,108017258
AL122035.2	antisense	ENSG00000272909	-0,649100574	0,061237303	0,10804104
SNORA72	snoRNA	ENSG00000207067	-0,412506232	0,061374508	0,108273797
CLIC3	protein_coding	ENSG00000169583	0,649765525	0,061426578	0,108356335
RPL5P24	processed_pseudogene	ENSG00000244052	0,662286747	0,061464154	0,108403973
RMDN3	protein_coding	ENSG00000137824	0,11256063	0,061459746	0,108403973
AC021078.1	processed_transcript	ENSG00000230551	0,221296682	0,061475753	0,108415107
AC005220.1	antisense	ENSG00000236352	0,60251774	0,061500254	0,108448991
ZNF771	protein_coding	ENSG00000179965	-0,204418111	0,061515397	0,108466368
SLC15A2	protein_coding	ENSG00000163406	0,520918817	0,061535222	0,108491997

AC103760.1	processed_transcript	ENSG00000254231	0,594215415	0,061542422	0,108495365
LINC01011	lincRNA	ENSG00000244041	-0,363256391	0,06160624	0,108589204
BLOC1S3	protein_coding	ENSG00000189114	-0,215603958	0,061678831	0,108707814
AC006449.5	antisense	ENSG00000277182	0,486340038	0,061699185	0,108734343
AP005264.7	TEC	ENSG00000280302	0,57444724	0,061775167	0,108858894
ASNSD1	protein_coding	ENSG00000138381	0,133673871	0,061956896	0,109163702
FKBP10	protein_coding	ENSG00000141756	-0,098153736	0,061958784	0,109163702
SCN4A	protein_coding	ENSG00000007314	0,621653055	0,061970129	0,109174312
AMMECR1	protein_coding	ENSG00000101935	0,122607541	0,061988614	0,109195745
KPNA6	protein_coding	ENSG00000025800	0,080554158	0,061992943	0,109195745
ZNF397	protein_coding	ENSG00000186812	-0,136471789	0,062031591	0,109249479
AC100827.2	unprocessed_pseudoge	ENSG00000260144	0,657285481	0,062041299	0,109252773
GSTA4	protein_coding	ENSG00000170899	-0,214373436	0,062107965	0,109360781
RALBP1	protein_coding	ENSG00000017797	-0,109404344	0,062126271	0,109383623
AL031282.2	processed_transcript	ENSG00000268575	0,227483443	0,062167365	0,10944658
SPATA5L1	protein_coding	ENSG00000171763	-0,120672465	0,062278728	0,109633225
CDK17	protein_coding	ENSG00000059758	0,091761154	0,062292007	0,109647191
AC007450.4	processed_pseudogene	ENSG00000278654	0,617431806	0,06231496	0,109678179
LCN12	protein_coding	ENSG00000184925	0,592061878	0,062404049	0,109825558
LINC01341	processed_transcript	ENSG00000227953	0,497781618	0,062511994	0,110006092
MFSD2B	protein_coding	ENSG00000205639	0,608062954	0,062523384	0,110016697
AC093627.5	lincRNA	ENSG00000242474	-0,440283357	0,062599386	0,110131533
AC069503.1	lincRNA	ENSG00000255856	-0,528907072	0,062595393	0,110131533
GTF3A	protein_coding	ENSG00000122034	-0,145854092	0,062685722	0,110264507
SPATA6	protein_coding	ENSG00000132122	-0,216567689	0,06268136	0,110264507
AC005261.3	lincRNA	ENSG00000268713	-0,344802227	0,062720476	0,110316178
SLC35A5	protein_coding	ENSG00000138459	-0,116220395	0,062797239	0,110441723
AC007823.1	lincRNA	ENSG00000260743	0,559623565	0,062828582	0,110487371
NIPAL1	protein_coding	ENSG00000163293	0,151750745	0,062871385	0,110543686
CCDC184	protein_coding	ENSG00000177875	-0,642866702	0,062869174	0,110543686
AC119674.2	protein_coding	ENSG00000284686	0,649151662	0,062886389	0,11056059
RPF1	protein_coding	ENSG00000117133	-0,108343654	0,062909037	0,110590927
SNORA71B	snoRNA	ENSG00000235408	-0,41218837	0,062920139	0,110600963
TBX2-AS1	antisense	ENSG00000267280	-0,414304406	0,062956766	0,110646381
PACS1	protein_coding	ENSG00000175115	-0,111215797	0,063068026	0,110832424
MIR155HG	lincRNA	ENSG00000234883	0,65106843	0,063148563	0,110954942
CRAT	protein_coding	ENSG00000095321	-0,136417824	0,063145181	0,110954942
FOXA3	protein_coding	ENSG00000170608	0,569467594	0,063169683	0,110982542
THBS3	protein_coding	ENSG00000169231	0,175352756	0,063206406	0,11103755
GLUD1	protein_coding	ENSG00000148672	-0,079537863	0,063220271	0,111052394

WAS	protein_coding	ENSG00000015285	0,639193365	0,063272045	0,111133821
HK1	protein_coding	ENSG00000156515	0,08958413	0,063319651	0,111207916
RF00019	misc_RNA	ENSG00000200090	0,608262897	0,063453358	0,111433202
AL034417.4	antisense	ENSG00000284747	0,529099063	0,063520689	0,111541894
TBC1D31	protein_coding	ENSG00000156787	-0,106402724	0,06356573	0,111606206
AC133552.2	transcribed_unprocesse	ENSG00000262587	-0,574282133	0,063568196	0,111606206
NR1H2	protein_coding	ENSG00000131408	-0,149480065	0,063639272	0,11172143
CCDC121	protein_coding	ENSG00000176714	0,217042963	0,063652681	0,111735406
CHRM5	protein_coding	ENSG00000184984	0,49734368	0,063783182	0,111954905
NTRK1	protein_coding	ENSG00000198400	0,62351684	0,063805554	0,111979303
ATP11A	protein_coding	ENSG00000068650	-0,157338908	0,063808002	0,111979303
PIN1	protein_coding	ENSG00000127445	-0,126572051	0,063883356	0,112101954
FGF18	protein_coding	ENSG00000156427	0,631544463	0,063926452	0,112167981
CCDC102A	protein_coding	ENSG00000135736	0,188396173	0,063988261	0,112266829
AL513217.1	antisense	ENSG00000223774	0,611395468	0,064002992	0,112279925
AC022154.1	antisense	ENSG00000268093	0,565200327	0,064006674	0,112279925
AC092139.3	TEC	ENSG00000279476	-0,372318907	0,064127594	0,112482421
BX255925.2	processed_pseudogene	ENSG00000270259	-0,576921704	0,064155062	0,112520978
AL157932.1	antisense	ENSG00000231530	0,532008457	0,064202315	0,112584599
SLC2A4	protein_coding	ENSG00000181856	-0,553632517	0,064292483	0,112733079
AC007570.1	antisense	ENSG00000255655	-0,649697502	0,064313608	0,112760481
COX11	protein_coding	ENSG00000166260	-0,131128943	0,064365573	0,112841944
AGRN	protein_coding	ENSG00000188157	0,18116882	0,064430564	0,112946228
AC004943.1	antisense	ENSG00000259209	0,452492982	0,064454674	0,112978836
ADAT1	protein_coding	ENSG00000065457	-0,099975121	0,064535188	0,113110297
SLC25A21	protein_coding	ENSG00000183032	0,478422655	0,064548418	0,113123819
SH2D6	protein_coding	ENSG00000152292	0,532369807	0,06456836	0,1131491
OGDHL	protein_coding	ENSG00000197444	-0,199430802	0,06460103	0,113196678
AL355922.2	processed_pseudogene	ENSG00000258599	0,597161952	0,064661266	0,113292547
PHACTR2	protein_coding	ENSG00000112419	-0,12054263	0,064669834	0,11329788
YWHAEP5	processed_pseudogene	ENSG00000234645	-0,637922097	0,064759162	0,113444689
FXYD5	protein_coding	ENSG00000089327	0,068321517	0,064782688	0,113466519
RRP36	protein_coding	ENSG00000124541	-0,107086736	0,06481119	0,113506746
TCAP	protein_coding	ENSG00000173991	-0,469518461	0,064873985	0,113607021
NDUFB3	protein_coding	ENSG00000119013	-0,131228124	0,06488435	0,113615471
AC020687.1	lincRNA	ENSG00000259560	0,637131665	0,064910971	0,113652384
ANKRD29	protein_coding	ENSG00000154065	0,186998366	0,064958626	0,113726113
AC108866.1	antisense	ENSG00000250938	0,590182706	0,06497513	0,113745298
ASNA1	protein_coding	ENSG00000198356	-0,114340642	0,065044754	0,113847747
TMEM145	protein_coding	ENSG00000167619	-0,636188377	0,065041318	0,113847747

COMMD3	protein_coding	ENSG00000148444	-0,157604786	0,065130056	0,113987324
LTK	protein_coding	ENSG00000062524	0,622986927	0,065138911	0,113993095
LRRC37A	protein_coding	ENSG00000176681	0,345670787	0,065155699	0,114012744
SC5D	protein_coding	ENSG00000109929	0,175781025	0,065240763	0,114151855
SYCE2	protein_coding	ENSG00000161860	0,370731162	0,065311478	0,114265837
HLA-DMB	protein_coding	ENSG00000242574	-0,319595187	0,065322643	0,114275623
CRLS1	protein_coding	ENSG00000088766	-0,110834345	0,065367105	0,114343653
AC090246.1	sense_intronic	ENSG00000277310	0,626561367	0,065374946	0,114347616
AC093297.2	lincRNA	ENSG00000272335	-0,275514004	0,06546712	0,114489311
PKP2	protein_coding	ENSG00000057294	-0,091097183	0,065463085	0,114489311
SCARNA18B	snoRNA	ENSG00000238754	-0,649313416	0,065524755	0,114580332
AL049776.1	TEC	ENSG00000279434	0,634879584	0,065594423	0,11469238
OGFOD2	protein_coding	ENSG00000111325	-0,318828275	0,065640842	0,114763759
AC079834.2	antisense	ENSG00000272944	0,456129497	0,065671729	0,114807975
AC244153.1	processed_transcript	ENSG00000276170	0,13818478	0,065703513	0,114853751
LYPD5	protein_coding	ENSG00000159871	0,612471536	0,065723324	0,11487859
HERC3	protein_coding	ENSG00000138641	-0,16170518	0,065854544	0,115088804
AC139100.2	sense_intronic	ENSG00000278000	0,643586104	0,065854812	0,115088804
MIR4458HG	lincRNA	ENSG00000247516	-0,233654594	0,06593212	0,115214091
AC013565.1	lincRNA	ENSG00000260123	0,535544928	0,065953274	0,115241238
AC004967.1	transcribed_unprocesse	ENSG00000243554	0,40691469	0,066002108	0,115316742
GIMAP2	protein_coding	ENSG00000106560	-0,638516704	0,06605226	0,115394536
DCAF7	protein_coding	ENSG00000136485	-0,084190477	0,066158362	0,115570055
WASHC3	protein_coding	ENSG00000120860	-0,128305588	0,066264526	0,11574565
PNPLA6	protein_coding	ENSG00000032444	-0,143390122	0,066290451	0,115781073
AL139246.5	lincRNA	ENSG00000272449	-0,414248111	0,066301526	0,115790557
AC027097.2	antisense	ENSG00000267787	0,450300514	0,066378812	0,11591566
ZNF777	protein_coding	ENSG00000196453	0,16679312	0,066391837	0,115928535
ART3	protein_coding	ENSG00000156219	0,543120993	0,066404223	0,115940293
PSTPIP1	protein_coding	ENSG00000140368	0,621607555	0,06657578	0,116229933
AC053503.5	antisense	ENSG00000268603	0,616054711	0,066601692	0,116265274
C8orf82	protein_coding	ENSG00000213563	-0,132970907	0,066648798	0,116327704
AC005578.1	TEC	ENSG00000280247	-0,597622023	0,066648105	0,116327704
LINC01807	lincRNA	ENSG00000232023	0,309733218	0,066683642	0,116378617
AC011498.1	lincRNA	ENSG00000267011	0,592962713	0,066711498	0,116417325
TMEM25	protein_coding	ENSG00000149582	-0,168487959	0,06675383	0,116481288
PPIAP72	processed_pseudogene	ENSG00000174715	-0,643491535	0,066775329	0,116508891
AL117332.1	antisense	ENSG00000275457	0,44862126	0,066796503	0,116535533
SLC25A32	protein_coding	ENSG00000164933	0,095520938	0,066801963	0,116535533
AC013394.1	protein_coding	ENSG00000279765	-0,393335304	0,066860285	0,116627355

INO80B	protein_coding	ENSG00000115274	0,637405677	0,066964749	0,116799644
AC091806.1	lincRNA	ENSG00000236393	-0,104374543	0,066994548	0,116841683
CLIP3	protein_coding	ENSG00000105270	0,624959476	0,067009607	0,116858008
MRPL2	protein_coding	ENSG00000112651	-0,106264389	0,067028611	0,116881211
KIAA1328	protein_coding	ENSG00000150477	-0,173096874	0,067035045	0,116882493
AP000880.1	antisense	ENSG00000255129	0,59367045	0,067047723	0,116894662
COMT	protein_coding	ENSG00000093010	0,124729309	0,067065104	0,116915026
ZCCHC4	protein_coding	ENSG00000168228	-0,149043507	0,067107032	0,116978176
AC087721.1	sense_intronic	ENSG00000259396	-0,637603301	0,067141573	0,11702844
JKAMP	protein_coding	ENSG00000050130	-0,117477155	0,067203302	0,117126082
TXNL4A	protein_coding	ENSG00000141759	-0,109132393	0,067222623	0,1171498
AC079684.1	sense_intronic	ENSG00000274943	-0,545283223	0,067312359	0,117296218
PTPN2	protein_coding	ENSG00000175354	-0,119185388	0,067322461	0,117303855
C9	protein_coding	ENSG00000113600	0,468020904	0,067368981	0,11737494
CYP2C18	protein_coding	ENSG00000108242	0,518565053	0,067375603	0,117376507
DEXI	protein_coding	ENSG00000182108	-0,165677578	0,067384083	0,11738131
SMG6	protein_coding	ENSG00000070366	0,145837972	0,067393022	0,117386911
NQO1	protein_coding	ENSG00000181019	0,587899101	0,067440792	0,117460143
MAK16	protein_coding	ENSG00000198042	-0,120792059	0,067504981	0,117551975
VASH2	protein_coding	ENSG00000143494	-0,347161167	0,067515232	0,117559845
PAXBP1	protein_coding	ENSG00000159086	-0,153566303	0,067614825	0,117723265
ALS2CL	protein_coding	ENSG00000178038	0,222819568	0,06763223	0,117743574
RF00019	misc_RNA	ENSG00000206914	-0,557726194	0,067663546	0,117788095
CITED1	protein_coding	ENSG00000125931	0,627292616	0,067759551	0,117945208
AC060765.1	lincRNA	ENSG00000253503	0,634545071	0,067841019	0,118076994
UBE2R2	protein_coding	ENSG00000107341	-0,096287543	0,067876698	0,118129069
RPL39P3	processed_pseudogene	ENSG00000235174	-0,235754496	0,067912842	0,118181944
FGFR1OP	protein_coding	ENSG00000213066	-0,216081849	0,067999731	0,118323108
UNC119B	protein_coding	ENSG00000175970	0,127048988	0,068022825	0,118343213
CYP21A1P	transcribed_unprocesse	ENSG00000204338	0,573573863	0,068022086	0,118343213
WNT9A	protein_coding	ENSG00000143816	0,575690737	0,068032446	0,118348108
RTN3	protein_coding	ENSG00000133318	-0,064448842	0,068037179	0,118348108
ANKK1	protein_coding	ENSG00000170209	0,470242094	0,068172268	0,118566739
ZNF121	protein_coding	ENSG00000197961	-0,099132181	0,06817443	0,118566739
SLC26A9	protein_coding	ENSG00000174502	0,495485394	0,068221948	0,118639321
SPATA4	protein_coding	ENSG00000150628	0,631153833	0,068246721	0,118672339
ZNF398	protein_coding	ENSG00000197024	0,129106832	0,068347507	0,118837519
SLC2A11	protein_coding	ENSG00000133460	0,309320068	0,068392278	0,118905283
AL359504.2	lincRNA	ENSG00000271576	-0,419752612	0,068568497	0,119201549
AL162431.2	antisense	ENSG00000243155	0,449860581	0,06861708	0,119275897

SSU72	protein_coding	ENSG00000160075	-0,097895283	0,068632994	0,11929345
CD226	protein_coding	ENSG00000150637	0,516939598	0,068649351	0,11931177
HSD17B13	protein_coding	ENSG00000170509	0,624452939	0,068729201	0,119430472
CEP170	protein_coding	ENSG00000143702	0,088507061	0,068729296	0,119430472
PLEC	protein_coding	ENSG00000178209	-0,281475162	0,068754384	0,119463946
DYDC2	protein_coding	ENSG00000133665	0,572456764	0,068805825	0,1195432
HIBADH	protein_coding	ENSG00000106049	0,100430091	0,068830915	0,119576662
PER2	protein_coding	ENSG00000132326	0,217973918	0,068859611	0,119606251
ABHD14B	protein_coding	ENSG00000114779	-0,10330509	0,068856673	0,119606251
AC005393.1	lincRNA	ENSG00000276445	-0,630176783	0,068954554	0,119761021
FUK	protein_coding	ENSG00000157353	-0,151091368	0,068967967	0,119774175
AC145285.2	lincRNA	ENSG00000251417	0,584962276	0,069049963	0,119906422
AC005342.1	antisense	ENSG00000203593	0,596035697	0,069113884	0,120007261
SMG5	protein_coding	ENSG00000198952	0,129190207	0,069159551	0,120066227
SNORD116-7	snoRNA	ENSG00000207133	0,580044073	0,069196859	0,120120828
AEN	protein_coding	ENSG00000181026	-0,092926015	0,069402078	0,120466878
AKR7L	polymorphic_pseudoger	ENSG00000211454	-0,582167142	0,0694473	0,120529611
PLXND1	protein_coding	ENSG00000004399	-0,15102136	0,069449973	0,120529611
AC008393.1	lincRNA	ENSG00000245317	-0,386079164	0,069664034	0,120890882
COPS3	protein_coding	ENSG00000141030	-0,123599471	0,069671346	0,120893343
GTPBP8	protein_coding	ENSG00000163607	-0,13526435	0,069717181	0,120962641
Z98884.2	sense_intronic	ENSG00000269925	-0,616817661	0,069726853	0,120969189
TEKT5	protein_coding	ENSG00000153060	0,572618733	0,06974676	0,120993492
NEK3	protein_coding	ENSG00000136098	-0,119311696	0,069821508	0,121112917
BCL2L12	protein_coding	ENSG00000126453	-0,149813623	0,069853728	0,12115856
CSMD2	protein_coding	ENSG00000121904	0,602590355	0,069860087	0,121159343
RFX2	protein_coding	ENSG00000087903	-0,126622068	0,069906084	0,121228864
SSR4	protein_coding	ENSG00000180879	0,139301259	0,070044234	0,121458171
AC120114.1	antisense	ENSG00000247735	0,378280193	0,070077302	0,121494968
MT-CO1	protein_coding	ENSG00000198804	0,42914105	0,070071551	0,121494968
SNORA54	snoRNA	ENSG00000207008	-0,372198408	0,07015633	0,121621701
C1orf220	lincRNA	ENSG00000213057	-0,597451335	0,070173105	0,121640499
AC123768.2	lincRNA	ENSG00000244952	0,624507946	0,070211273	0,121696375
ZNF148	protein_coding	ENSG00000163848	-0,115400209	0,070303327	0,121845635
RPL18A	protein_coding	ENSG00000105640	-0,14763163	0,070358725	0,121931344
ATP2B4	protein_coding	ENSG00000058668	0,116103898	0,070390821	0,121976659
MATN4	protein_coding	ENSG00000124159	0,598667236	0,070508185	0,122169712
AC010271.2	lincRNA	ENSG00000277453	0,515156264	0,07055018	0,12223215
AC114316.1	lincRNA	ENSG00000250049	0,536759265	0,070574767	0,122262239
ZNF70	protein_coding	ENSG00000187792	0,185038395	0,070579469	0,122262239

AC005495.1	TEC	ENSG00000278873	0,585156119	0,070674939	0,122417279
AC127522.1	antisense	ENSG00000259277	0,613259418	0,070756902	0,1225489
DCTN1	protein_coding	ENSG00000204843	-0,114769469	0,070799861	0,122612949
TMEM143	protein_coding	ENSG00000161558	-0,259288626	0,070812725	0,122624873
PDAP1	protein_coding	ENSG00000106244	-0,096003137	0,070846307	0,122672669
NR2C1	protein_coding	ENSG00000120798	-0,117936286	0,070909737	0,122772135
DSE	protein_coding	ENSG00000111817	0,0814244	0,070917535	0,122775271
LCMT1-AS1	bidirectional_promoter_	ENSG00000260448	0,415133057	0,070975557	0,122865351
AC090912.2	antisense	ENSG00000266850	0,619852042	0,071007526	0,122910317
ARMC2	protein_coding	ENSG00000118690	-0,278439191	0,071033242	0,122944455
AC110749.1	processed_pseudogene	ENSG00000244086	0,614155705	0,0710761	0,122997875
SLC6A9	protein_coding	ENSG00000196517	0,253826476	0,071073598	0,122997875
MAP7D1	protein_coding	ENSG00000116871	-0,113950686	0,071188358	0,123181745
PEX5L	protein_coding	ENSG00000114757	0,591216653	0,071206846	0,123203341
HSPA9P1	processed_pseudogene	ENSG00000226666	0,61525282	0,071325707	0,123398586
IGSF10	protein_coding	ENSG00000152580	0,614402544	0,071387038	0,123494277
LDHD	protein_coding	ENSG00000166816	0,348791194	0,071595789	0,123844955
FAM151B	protein_coding	ENSG00000152380	0,271100509	0,071715933	0,124042317
MAEA	protein_coding	ENSG00000090316	0,065026956	0,071741888	0,124076746
LINC02657	lincRNA	ENSG00000242147	-0,178961727	0,071754051	0,124087318
HMBOX1	protein_coding	ENSG00000147421	0,157295694	0,071762581	0,124091605
AL136040.1	antisense	ENSG00000273783	-0,372352558	0,071941102	0,124389816
LINC02656	lincRNA	ENSG00000212743	0,519503731	0,071968218	0,124415724
PLD2	protein_coding	ENSG00000129219	-0,138781082	0,071967635	0,124415724
RGS22	protein_coding	ENSG00000132554	0,590247789	0,07198687	0,124437481
AL049747.1	sense_intronic	ENSG00000268818	0,605289362	0,072016836	0,124478789
LYPD6B	protein_coding	ENSG00000150556	-0,302694934	0,072040912	0,12450991
AC098650.1	protein_coding	ENSG00000283563	0,491468653	0,072070702	0,124536863
TBC1D19	protein_coding	ENSG00000109680	0,185701444	0,072074723	0,124536863
CAMTA1	protein_coding	ENSG00000171735	0,135268128	0,072064411	0,124536863
SLC46A3	protein_coding	ENSG00000139508	-0,156629429	0,072085354	0,124544741
PTTG1IP	protein_coding	ENSG00000183255	0,064252565	0,072111556	0,124561276
LNPK	protein_coding	ENSG00000144320	-0,120131103	0,072113144	0,124561276
CYS1	protein_coding	ENSG00000205795	-0,207649072	0,072109851	0,124561276
AL645933.2	lincRNA	ENSG00000272221	0,38492547	0,072124305	0,124570063
LINC00869	transcribed_unprocesse	ENSG00000277147	0,575044808	0,072155398	0,124613271
NDUFA13	protein_coding	ENSG00000186010	-0,437573246	0,072167719	0,124624056
TSPO	protein_coding	ENSG00000100300	-0,192098038	0,0722103	0,124676593
CCDC85A	protein_coding	ENSG00000055813	-0,285728208	0,072208444	0,124676593
LINC02511	lincRNA	ENSG00000248869	0,61462669	0,072285696	0,124796265

C2orf76	protein_coding	ENSG00000186132	0,165268842	0,072313886	0,124834426
IRAK4	protein_coding	ENSG00000198001	0,133089213	0,072365755	0,124913453
LINC01602	lincRNA	ENSG00000205293	-0,296538474	0,072439276	0,125020032
MAFA	protein_coding	ENSG00000182759	0,514519574	0,072439691	0,125020032
SCAMP4	protein_coding	ENSG00000227500	-0,131084495	0,072487961	0,12508549
ICAM5	protein_coding	ENSG00000105376	-0,21249283	0,072489816	0,12508549
NDUFA4L2	protein_coding	ENSG00000185633	0,591630978	0,072523379	0,125132877
AC113194.1	antisense	ENSG00000272457	0,594817943	0,072551303	0,125170527
TPGS1	protein_coding	ENSG00000141933	-0,209840054	0,072620245	0,125278933
FAM122A	protein_coding	ENSG00000187866	-0,16078162	0,072672973	0,12535935
RRP8	protein_coding	ENSG00000132275	-0,121603099	0,07271447	0,125409836
CARMIL3	protein_coding	ENSG00000186648	-0,423396055	0,072709745	0,125409836
LRGUK	protein_coding	ENSG00000155530	0,558552682	0,072745365	0,125452571
AL139317.3	antisense	ENSG00000259049	-0,606150096	0,072768836	0,125482498
RPS13	protein_coding	ENSG00000110700	-0,136216518	0,072900711	0,125699335
SNORA74B	snoRNA	ENSG00000212402	-0,323229242	0,072932266	0,125743172
AC092656.1	transcribed_processed_	ENSG00000178636	0,604378571	0,072947502	0,12575887
TMC6	protein_coding	ENSG00000141524	0,173919838	0,072984965	0,125812879
KIF1C	protein_coding	ENSG00000129250	-0,104678447	0,073116716	0,126029401
SCARB2	protein_coding	ENSG00000138760	-0,090284442	0,073155573	0,126085781
ODAPH	protein_coding	ENSG00000174792	0,559992512	0,073229949	0,126182161
PLA2G4A	protein_coding	ENSG00000116711	0,304047501	0,073224932	0,126182161
TMCO5B	transcribed_unitary_pse	ENSG00000215296	0,602512819	0,073228778	0,126182161
AL662791.1	antisense	ENSG00000244349	0,594134594	0,0732773	0,126253144
KLHL2P1	unprocessed_pseudoge	ENSG00000250412	0,556416777	0,073307367	0,126294338
RF00019	misc_RNA	ENSG00000201217	0,607844342	0,073317103	0,126300503
CEP68	protein_coding	ENSG00000011523	-0,131328335	0,073334744	0,126320281
AC017091.1	lincRNA	ENSG00000251339	0,588229186	0,073354814	0,126344242
RNF34	protein_coding	ENSG00000170633	-0,095179078	0,073404374	0,126413622
MRPL46	protein_coding	ENSG00000259494	-0,156037242	0,073407422	0,126413622
PCBP2	protein_coding	ENSG00000197111	-0,088594868	0,073442714	0,126463779
AP000640.1	processed_transcript	ENSG00000254477	0,588751625	0,073508739	0,126556219
ACP2	protein_coding	ENSG00000134575	0,119200319	0,073507076	0,126556219
COMMD10	protein_coding	ENSG00000145781	0,161806719	0,073519972	0,126564934
COX5BP6	processed_pseudogene	ENSG00000237082	-0,612724145	0,073547658	0,12660197
RAP1GAP2	protein_coding	ENSG00000132359	-0,152563674	0,073621476	0,126718401
AP002770.1	antisense	ENSG00000256034	0,509959497	0,073640568	0,126740626
HSPA9	protein_coding	ENSG00000113013	-0,085672833	0,073678717	0,126795645
AC103923.1	antisense	ENSG00000235545	0,599161958	0,073686412	0,126798248
PLLP	protein_coding	ENSG00000102934	-0,223210473	0,073741385	0,126882198

AC025034.1	antisense	ENSG00000258302	0,524204854	0,07375146	0,126888889
AC005046.1	antisense	ENSG00000273055	0,56392906	0,07377553	0,126919655
SPAG1	protein_coding	ENSG00000104450	0,148834514	0,073997813	0,127291382
RAB43	protein_coding	ENSG00000172780	0,334328368	0,074019386	0,127317814
CLSTN2	protein_coding	ENSG00000158258	0,444437981	0,074031172	0,127327407
NFXL1	protein_coding	ENSG00000170448	0,112607393	0,074049303	0,127347913
ERN2	protein_coding	ENSG00000134398	0,54073755	0,074101962	0,127427789
SLC17A9	protein_coding	ENSG00000101194	-0,200807633	0,074204155	0,127592826
PHYH	protein_coding	ENSG00000107537	0,113213552	0,074248127	0,127633728
ZNF362	protein_coding	ENSG00000160094	-0,22253371	0,074248132	0,127633728
FOXF1	protein_coding	ENSG00000103241	-0,390971237	0,07424032	0,127633728
AL133355.1	sense_overlapping	ENSG00000260461	0,602719247	0,074252835	0,127633728
LINC01063	antisense	ENSG00000232065	0,537573632	0,074446327	0,1279556
DVL1	protein_coding	ENSG00000107404	-0,133177324	0,074457607	0,127964264
GEN1	protein_coding	ENSG00000178295	-0,141526813	0,074500497	0,128027248
IMPA1	protein_coding	ENSG00000133731	0,133737053	0,074542603	0,128088874
AC079298.3	antisense	ENSG00000280241	0,572329973	0,07455521	0,128090513
GARNL3	protein_coding	ENSG00000136895	0,257410488	0,074556048	0,128090513
NDUFV1	protein_coding	ENSG00000167792	-0,095808981	0,074577678	0,128116942
MIR548XHG	lincRNA	ENSG00000224141	0,582742527	0,074638787	0,128200446
AC145098.2	TEC	ENSG00000279821	0,398199676	0,074633782	0,128200446
IMPAD1	protein_coding	ENSG00000104331	-0,100775104	0,074653779	0,128215459
WWC2-AS2	lincRNA	ENSG00000251359	0,377993909	0,074691818	0,128270048
ZC2HC1A	protein_coding	ENSG00000104427	0,156955151	0,074729954	0,128324795
AC105219.1	sense_intronic	ENSG00000181097	0,536033779	0,074771259	0,128384974
INSIG2	protein_coding	ENSG00000125629	-0,122149344	0,074810829	0,128442164
RF00019	misc_RNA	ENSG00000207425	0,587008492	0,074829226	0,128462998
VCAN	protein_coding	ENSG00000038427	0,30199877	0,07485291	0,128492901
SNORD19C	snoRNA	ENSG00000222345	-0,514906443	0,074870512	0,128512362
RTEL1-TNFRSF6B	protein_coding	ENSG00000026036	-0,442446329	0,07490642	0,128563236
CWC22	protein_coding	ENSG00000163510	-0,123010374	0,074914468	0,128566291
TMEM123	protein_coding	ENSG00000152558	0,118387908	0,074961665	0,128636525
OAZ2	protein_coding	ENSG00000180304	-0,084411951	0,075027258	0,128738314
SNORD62B	snoRNA	ENSG00000231587	0,603192284	0,075038849	0,128747431
TRAF4	protein_coding	ENSG00000076604	0,102568966	0,075055102	0,128764546
GRPR	protein_coding	ENSG00000126010	0,596503785	0,075062553	0,128766557
OR2A1-AS1	antisense	ENSG00000244479	-0,492554177	0,075176281	0,128940084
FAM229A	protein_coding	ENSG00000225828	0,340211329	0,075170654	0,128940084
HMGB1P50	processed_pseudogene	ENSG00000271237	0,447497919	0,075231744	0,129024423
ZNF542P	transcribed_unprocesse	ENSG00000240225	0,287147772	0,075267826	0,129075511

TBKBP1	protein_coding	ENSG00000198933	-0,152166592	0,075441554	0,129362618
DBF4P1	processed_pseudogene	ENSG00000235489	-0,50883877	0,075532847	0,12950123
HOMER2	protein_coding	ENSG00000103942	-0,171174749	0,075535018	0,12950123
AL356235.1	transcribed_processed_	ENSG00000248503	0,438083509	0,075578553	0,129565039
FAM45BP	transcribed_processed_	ENSG00000221930	-0,586244383	0,07567532	0,129720085
COL4A3BP	protein_coding	ENSG00000113163	0,10241021	0,07570015	0,129751803
CYB5R4	protein_coding	ENSG00000065615	0,123862001	0,075833341	0,129969235
HSP90AB3P	processed_pseudogene	ENSG00000183199	0,320567804	0,07586042	0,130004781
ARHGEF34P	unprocessed_pseudoge	ENSG00000204959	-0,303061172	0,075904657	0,130069724
ETV2	protein_coding	ENSG00000105672	-0,549621332	0,075926832	0,130096852
FAM193A	protein_coding	ENSG00000125386	0,141513404	0,075935868	0,130099917
TMEM67	protein_coding	ENSG00000164953	0,139612207	0,075941307	0,130099917
ANKRD49	protein_coding	ENSG00000168876	-0,153806064	0,075990671	0,130173612
LRRC47	protein_coding	ENSG00000130764	-0,108025324	0,076196825	0,130515858
PHACTR1	protein_coding	ENSG00000112137	0,106092997	0,076224385	0,130552161
STIMATE	protein_coding	ENSG00000213533	-0,367489311	0,076275012	0,130627964
CDC6	protein_coding	ENSG00000094804	0,11251355	0,076283938	0,130632342
NFRKB	protein_coding	ENSG00000170322	-0,097568314	0,076463617	0,130929102
PCM1	protein_coding	ENSG00000078674	0,088128591	0,076527057	0,131026792
ORC5	protein_coding	ENSG00000164815	-0,152126531	0,076542133	0,131041665
AC005829.1	transcribed_unprocesse	ENSG00000261575	-0,439077906	0,076612977	0,131152005
BCHE	protein_coding	ENSG00000114200	0,293368397	0,076863155	0,131569297
SLC8A1-AS1	antisense	ENSG00000227028	0,408226701	0,076890578	0,131603024
RPS17	protein_coding	ENSG00000182774	-0,14370622	0,076895692	0,131603024
TFG	protein_coding	ENSG00000114354	0,059671714	0,076918692	0,131631404
FOXO1	protein_coding	ENSG00000150907	-0,185313951	0,076956094	0,131684423
LINC01121	processed_transcript	ENSG00000205054	0,552440369	0,077081248	0,131887579
NIPA2	protein_coding	ENSG00000140157	-0,084239592	0,077173205	0,132026709
CHURC1	protein_coding	ENSG00000258289	-0,131632665	0,077175437	0,132026709
AL157834.2	antisense	ENSG00000234026	0,592281103	0,077205139	0,132066505
AL357140.2	sense_intronic	ENSG00000228150	0,585332855	0,077231199	0,132100065
SRSF9	protein_coding	ENSG00000111786	-0,071911254	0,077306115	0,13221718
AC092919.1	lincRNA	ENSG00000240497	0,584631937	0,077332307	0,132250948
SELENOO	protein_coding	ENSG00000073169	-0,15575943	0,077385924	0,132331608
TUBGCP4	protein_coding	ENSG00000137822	-0,117308604	0,077393295	0,132333179
PINX1	protein_coding	ENSG00000254093	-0,162362796	0,077408424	0,132348014
LRRC29	protein_coding	ENSG00000125122	0,368217466	0,077434299	0,132381218
SMIM15	protein_coding	ENSG00000188725	0,107725121	0,077463117	0,132419447
AC010761.1	protein_coding	ENSG00000160602	0,250148789	0,07750766	0,13248455
TRPV4	protein_coding	ENSG00000111199	0,501149395	0,077561622	0,132565738

LINC01869	transcribed_unitary_pse	ENSG00000180279	0,540436037	0,07759348	0,132609139
NSUN5P1	transcribed_unprocesse	ENSG00000223705	-0,2602372	0,077608849	0,132624354
AC007639.1	lincRNA	ENSG00000263680	0,460867598	0,077659662	0,13270013
DLG2	protein_coding	ENSG00000150672	0,346050003	0,077930379	0,133151623
RP9P	transcribed_unprocesse	ENSG00000205763	-0,149446689	0,078050811	0,133346284
AC005562.1	processed_transcript	ENSG00000214719	-0,395112006	0,07821295	0,133612162
AC007773.1	antisense	ENSG00000267213	0,211136218	0,078258184	0,133678301
POLE4	protein_coding	ENSG00000115350	-0,129820708	0,078297761	0,133734766
TMEM80	protein_coding	ENSG00000177042	-0,265578217	0,078357719	0,13381489
RNU6-4P	snRNA	ENSG00000206932	0,574097767	0,078353676	0,13381489
TMEM99	protein_coding	ENSG00000167920	0,125891491	0,078377072	0,133836795
SPSB1	protein_coding	ENSG00000171621	0,15788636	0,078436428	0,133927001
PPP2R2D	protein_coding	ENSG00000175470	-0,096695403	0,078717502	0,134395734
DNAJC27-AS1	antisense	ENSG00000224165	0,438309837	0,078763447	0,134462984
TNS2	protein_coding	ENSG00000111077	-0,211055197	0,078774854	0,134471265
CMC2	protein_coding	ENSG00000103121	-0,110150742	0,078791546	0,134488566
AC044860.1	transcribed_unprocesse	ENSG00000229212	-0,449975494	0,078828588	0,134540596
TFEB	protein_coding	ENSG00000112561	0,21902222	0,078836456	0,134542829
AL034397.1	processed_pseudogene	ENSG00000217835	-0,539851164	0,078855344	0,13455267
REPS2	protein_coding	ENSG00000169891	0,47183874	0,078850707	0,13455267
AC107954.1	processed_pseudogene	ENSG00000261549	0,518544001	0,079187741	0,135108607
SREK1	protein_coding	ENSG00000153914	-0,103621793	0,079240699	0,135187718
DCAF13P3	processed_pseudogene	ENSG00000259378	0,571735947	0,079249412	0,135191337
MVK	protein_coding	ENSG00000110921	0,131941272	0,079266845	0,135203519
EGFEM1P	transcribed_unitary_pse	ENSG00000206120	0,496395605	0,079269738	0,135203519
CDC42SE2	protein_coding	ENSG00000158985	-0,099275285	0,079309669	0,135260378
NECTIN3-AS1	antisense	ENSG00000242242	0,439842568	0,079386107	0,135370518
KRR1	protein_coding	ENSG00000111615	-0,110909021	0,07939085	0,135370518
FREM2	protein_coding	ENSG00000150893	-0,152517204	0,07939405	0,135370518
RPS14P8	processed_pseudogene	ENSG00000239528	-0,332523349	0,079481215	0,135507873
RAP1GAP	protein_coding	ENSG00000076864	0,287257657	0,079488621	0,135509235
MAN1B1-DT	antisense	ENSG00000268996	0,318985144	0,079568583	0,135634279
OR2A20P	transcribed_unprocesse	ENSG00000170356	0,503590154	0,079656038	0,135772072
SRA1	protein_coding	ENSG00000213523	0,118411738	0,079685755	0,135811438
DND1	protein_coding	ENSG00000256453	0,428001535	0,079733577	0,135881651
SIK1B	protein_coding	ENSG00000275993	-0,202710759	0,079818218	0,136014593
ATP6V0E2	protein_coding	ENSG00000171130	-0,130140031	0,079913599	0,136165815
AC008747.1	antisense	ENSG00000267024	0,565033292	0,079965105	0,136230942
DDN	protein_coding	ENSG00000181418	-0,369561845	0,079960587	0,136230942
AC016712.1	processed_pseudogene	ENSG00000235225	0,548689402	0,079985383	0,136254169

SPTY2D1OS	protein_coding	ENSG00000247595	0,357769053	0,080019381	0,136300765
AC087457.1	antisense	ENSG00000250007	0,474387596	0,080245718	0,136663595
AC092159.3	antisense	ENSG00000233970	-0,514853441	0,080263086	0,136681825
SYCP2L	protein_coding	ENSG00000153157	0,580540226	0,080378333	0,136866718
WDR35	protein_coding	ENSG00000118965	-0,104995263	0,080397125	0,136887351
HIGD2B	protein_coding	ENSG00000175202	0,53104981	0,080410734	0,136899157
AC105446.1	antisense	ENSG00000227053	0,565306733	0,080449922	0,136947495
AC080112.1	antisense	ENSG00000266208	0,464115871	0,08045248	0,136947495
TMEM88	protein_coding	ENSG00000167874	0,575306971	0,080501292	0,137019212
DSG2	protein_coding	ENSG00000046604	0,082323415	0,080520177	0,137039983
RASSF1-AS1	antisense	ENSG00000281358	0,44361077	0,080552826	0,137080368
PLEKHF2	protein_coding	ENSG00000175895	0,129612863	0,080557273	0,137080368
ZNF25	protein_coding	ENSG00000175395	-0,132599153	0,080702659	0,137316371
IFT27	protein_coding	ENSG00000100360	0,131824432	0,080716845	0,137329117
DNAJC2	protein_coding	ENSG00000105821	0,105330558	0,080732863	0,137344974
IRX3	protein_coding	ENSG00000177508	-0,352619844	0,080797458	0,137443465
NLK	protein_coding	ENSG00000087095	0,106192286	0,080886715	0,137583887
COA6-AS1	antisense	ENSG00000231663	-0,357132019	0,080978331	0,137728301
CRB3	protein_coding	ENSG00000130545	0,565040072	0,081056012	0,13784899
AL356481.1	antisense	ENSG00000228395	0,531736662	0,081100203	0,137912707
ZNF490	protein_coding	ENSG00000188033	0,222081708	0,08111546	0,137927217
BAG6	protein_coding	ENSG00000204463	0,10002153	0,081125424	0,137932724
CRTC3	protein_coding	ENSG00000140577	-0,10891732	0,081187633	0,138015612
IRAK1BP1	protein_coding	ENSG00000146243	-0,157232323	0,081184755	0,138015612
MYO1B	protein_coding	ENSG00000128641	-0,074134128	0,081247556	0,138106032
SRGN	protein_coding	ENSG00000122862	-0,139377529	0,081257288	0,138111127
PPIAP22	processed_pseudogene	ENSG00000198618	-0,177893568	0,08134619	0,138250776
AC007950.3	lincRNA	ENSG00000276651	0,575007441	0,081359478	0,138261902
SNORA65	snoRNA	ENSG00000201302	-0,345289408	0,081375416	0,13827753
AC100849.1	lincRNA	ENSG00000253557	0,519832811	0,081401984	0,138311216
ACRV1	protein_coding	ENSG00000134940	0,539681878	0,081501091	0,13846814
MCPH1	protein_coding	ENSG00000147316	0,098533252	0,081573955	0,138580454
DPY19L2P2	transcribed_unprocesse	ENSG00000170629	0,438482267	0,081585032	0,138587793
AL132800.1	antisense	ENSG00000258744	0,500277993	0,081709904	0,138782575
KIF1B	protein_coding	ENSG00000054523	0,099402068	0,081713231	0,138782575
MARCH4	protein_coding	ENSG00000144583	-0,097709336	0,081732206	0,138803308
RBM42	protein_coding	ENSG00000126254	-0,120085605	0,081777451	0,138868646
ZC4H2	protein_coding	ENSG00000126970	-0,167954437	0,081884801	0,139039429
HTT	protein_coding	ENSG00000197386	0,117781592	0,081926759	0,139099158
AC016586.1	sense_intronic	ENSG00000268670	-0,557985676	0,081993595	0,139201111

ANKRD30B	protein_coding	ENSG00000180777	0,531972618	0,082070703	0,139320486
TMEM255B	protein_coding	ENSG00000184497	0,322220478	0,082094104	0,139348678
RIT1	protein_coding	ENSG00000143622	0,095634111	0,08212194	0,139372858
AC116407.4	TEC	ENSG00000280033	0,57018545	0,082118241	0,139372858
LINC00460	lincRNA	ENSG00000233532	0,452622954	0,082149336	0,139407817
SREBF1	protein_coding	ENSG00000072310	-0,17084153	0,08217909	0,139446772
MDH1B	protein_coding	ENSG00000138400	-0,397879877	0,082283779	0,139612865
AP000442.2	antisense	ENSG00000255139	0,558919399	0,082345996	0,139706873
RAD51C	protein_coding	ENSG00000108384	0,099785563	0,082370614	0,139737079
OTULINL	protein_coding	ENSG00000145569	0,197499675	0,082383936	0,13974812
MT-CO2	protein_coding	ENSG00000198712	0,37893856	0,082415627	0,139790317
RDH12	protein_coding	ENSG00000139988	0,55244298	0,082481529	0,139890528
VWA8	protein_coding	ENSG00000102763	-0,115485297	0,082506075	0,139920588
MAP4	protein_coding	ENSG00000047849	-0,094998588	0,082585046	0,140042935
MTLN	protein_coding	ENSG00000175701	-0,149562626	0,082709151	0,140241789
NDFIP1	protein_coding	ENSG00000131507	-0,082158445	0,082855295	0,140466367
RPS15	protein_coding	ENSG00000115268	-0,14078943	0,082854082	0,140466367
ERC2	protein_coding	ENSG00000187672	0,236541965	0,082933693	0,140587656
HS6ST1P1	processed_pseudogene	ENSG00000187952	-0,580095176	0,082962136	0,140612628
ZDHHC1	protein_coding	ENSG00000159714	0,221271785	0,082959264	0,140612628
AC108488.1	lincRNA	ENSG00000242282	0,27272865	0,083063586	0,140761311
MIR29B2CHG	lincRNA	ENSG00000203709	-0,352093888	0,083063169	0,140761311
COL4A6	protein_coding	ENSG00000197565	0,128538447	0,083143557	0,140885192
DDX42	protein_coding	ENSG00000198231	0,104778285	0,083177321	0,140930761
TRAPPC9	protein_coding	ENSG00000167632	0,142496178	0,083192931	0,140945565
CRYBG3	protein_coding	ENSG00000080200	0,117215351	0,083359809	0,141216626
AC073389.3	lincRNA	ENSG00000272791	-0,560610601	0,083403132	0,141278347
AC026979.3	lincRNA	ENSG00000272375	0,547334812	0,083422663	0,141299762
AC092651.2	unprocessed_pseudoge	ENSG00000266931	0,548099959	0,08354993	0,141503639
MKS1	protein_coding	ENSG00000011143	-0,111629172	0,083575845	0,141535842
FASTK	protein_coding	ENSG00000164896	-0,152621087	0,08360831	0,141579132
NDUFV3	protein_coding	ENSG00000160194	0,070483795	0,083751909	0,141798882
LTBP4	protein_coding	ENSG00000090006	-0,174532758	0,083812095	0,141889069
CHAMP1	protein_coding	ENSG00000198824	-0,112895993	0,083845525	0,141933949
AC104695.1	lincRNA	ENSG00000227938	0,498524908	0,083872828	0,141959306
ULK4P3	transcribed_unprocesse	ENSG00000178081	-0,387808479	0,083874348	0,141959306
STK24-AS1	antisense	ENSG00000224418	0,467142818	0,083907726	0,142004082
AGFG2	protein_coding	ENSG00000106351	-0,197880292	0,083933336	0,142035703
RRAS	protein_coding	ENSG00000126458	0,140192703	0,083976529	0,142097073
PID1	protein_coding	ENSG00000153823	0,100333577	0,084028504	0,14217329

SPATA24	protein_coding	ENSG00000170469	-0,246052008	0,084104669	0,142290421
ADAMTS10	protein_coding	ENSG00000142303	-0,190699371	0,084118666	0,142302362
UNC79	protein_coding	ENSG00000133958	0,576015068	0,08413467	0,142317697
AP000688.1	sense_intronic	ENSG00000230212	0,51948546	0,08418749	0,142395301
RASL10B	protein_coding	ENSG00000270885	0,57114592	0,084197927	0,14240121
AC055854.1	processed_transcript	ENSG00000253125	0,559891478	0,084225744	0,142436512
EIF2AK3-DT	antisense	ENSG00000234028	0,316673715	0,084240033	0,142448931
CHD8	protein_coding	ENSG00000100888	0,09356344	0,084290236	0,142522072
BIN3	protein_coding	ENSG00000147439	-0,118320333	0,084312732	0,142548357
AL354836.1	antisense	ENSG00000226332	0,571364502	0,08434256	0,142587034
DYRK1B	protein_coding	ENSG00000105204	0,345641441	0,084366225	0,142615287
MRPL38	protein_coding	ENSG00000204316	-0,361063289	0,084379812	0,14262559
AC092692.1	TEC	ENSG00000279349	0,479834462	0,084386228	0,14262559
KCNH3	protein_coding	ENSG00000135519	-0,323074561	0,084395467	0,142629452
SEC22B4P	transcribed_unprocesse	ENSG00000277406	0,502262307	0,08442336	0,142664836
RPL27A	protein_coding	ENSG00000166441	-0,135789687	0,084451687	0,142689192
RAB26	protein_coding	ENSG00000167964	-0,564155639	0,084450703	0,142689192
UPK1A-AS1	antisense	ENSG00000226510	0,515611041	0,084460203	0,142691826
C3orf62	protein_coding	ENSG00000188315	0,204679182	0,084509358	0,142761003
FAM219B	protein_coding	ENSG00000178761	0,118154046	0,084515071	0,142761003
MTURN	protein_coding	ENSG00000180354	-0,187629706	0,08453706	0,142786388
RSPH10B2	protein_coding	ENSG00000169402	0,56401283	0,084552101	0,142800033
RNU6-1016P	snRNA	ENSG00000252498	0,560141722	0,084783129	0,143166672
NEPNP	unitary_pseudogene	ENSG00000218233	0,537898249	0,08478315	0,143166672
HTR7P1	transcribed_processed_	ENSG00000183935	-0,212917932	0,084804366	0,14319071
LINC00337	lincRNA	ENSG00000225077	0,335906996	0,084894323	0,143324721
FRG1CP	transcribed_unprocesse	ENSG00000282826	0,144070357	0,08489771	0,143324721
IL22RA1	protein_coding	ENSG00000142677	-0,404739837	0,084915578	0,143343086
ADAMTSL4-AS1	processed_transcript	ENSG00000203804	0,374770815	0,084950223	0,143389768
SETP17	processed_pseudogene	ENSG00000255222	0,52424686	0,085125202	0,143673296
SLC10A3	protein_coding	ENSG00000126903	-0,135201933	0,085132678	0,143674091
NDUFV2	protein_coding	ENSG00000178127	-0,466712069	0,085147653	0,143687541
CAMK2D	protein_coding	ENSG00000145349	0,087127368	0,085220447	0,14379855
TET3	protein_coding	ENSG00000187605	0,197754473	0,085245949	0,143829747
SYNE1-AS1	antisense	ENSG00000234577	0,567415085	0,085358891	0,14400846
KPNA3	protein_coding	ENSG00000102753	-0,122301402	0,08541675	0,144094222
LINC02361	lincRNA	ENSG00000256576	-0,276166644	0,085454809	0,14414657
PIGS	protein_coding	ENSG00000087111	-0,083970037	0,085590552	0,14436367
AC025580.2	antisense	ENSG00000259354	0,532722109	0,085610968	0,144374359
PCBP2-OT1	non_coding	ENSG00000282977	-0,56523078	0,085605165	0,144374359

AC091133.2	antisense	ENSG00000250838	0,362977261	0,085692276	0,144499595
GPR137B	protein_coding	ENSG00000077585	-0,16904613	0,085756179	0,144595465
SLC23A3	protein_coding	ENSG00000213901	0,538053578	0,085802384	0,144651186
RHOF	protein_coding	ENSG00000139725	-0,132539928	0,085803332	0,144651186
AC018413.1	antisense	ENSG00000265778	0,517771477	0,08592478	0,144844023
AC006128.1	TEC	ENSG00000279716	0,545128555	0,085936029	0,14485108
NUDT16	protein_coding	ENSG00000198585	-0,109486968	0,085945716	0,144855505
AC132938.5	TEC	ENSG00000279744	0,564678811	0,086067082	0,145048138
BTAF1	protein_coding	ENSG00000095564	0,119672566	0,086123302	0,145130961
MYO3B	protein_coding	ENSG00000071909	0,419696304	0,086150789	0,145165354
LRRC57	protein_coding	ENSG00000180979	-0,112115747	0,086163505	0,145174853
ZMIZ1-AS1	antisense	ENSG00000224596	0,530121939	0,086246059	0,14530201
SERF1B	protein_coding	ENSG00000205572	0,418808626	0,086285126	0,145355888
SERF1B	protein_coding	ENSG00000205572	0,418808626	0,086285126	0,145355888
HINT2	protein_coding	ENSG00000137133	-0,166565527	0,086301588	0,14537168
AC231981.1	antisense	ENSG00000235078	0,486318665	0,086346399	0,145435218
AP000766.1	lincRNA	ENSG00000261098	0,476913894	0,086376845	0,145474552
PRCC	protein_coding	ENSG00000143294	-0,134339087	0,086428575	0,145549723
AC079848.1	antisense	ENSG00000250012	-0,56414488	0,086512249	0,145678672
AL158196.1	antisense	ENSG00000276968	0,521242683	0,086611654	0,145834088
RPL5P34	processed_pseudogene	ENSG00000234009	0,388331038	0,086649325	0,145885541
SMCP	protein_coding	ENSG00000163206	-0,111959136	0,08669835	0,145956099
KDELC1P1	processed_pseudogene	ENSG00000250329	0,550337593	0,086917643	0,146313269
VAT1L	protein_coding	ENSG00000171724	-0,352340488	0,086961237	0,146374639
SLC30A9	protein_coding	ENSG00000014824	0,10041706	0,087021073	0,146463336
MFSD8	protein_coding	ENSG00000164073	0,143994039	0,08704239	0,146487193
C9orf72	protein_coding	ENSG00000147894	0,159044273	0,087100933	0,146550259
MTA2	protein_coding	ENSG00000149480	0,079019064	0,08709555	0,146550259
ANKRD50	protein_coding	ENSG00000151458	-0,106858356	0,0871013	0,146550259
AC131254.1	lincRNA	ENSG00000248964	0,548504139	0,087152154	0,146623794
AL133523.1	antisense	ENSG00000258982	0,556354791	0,087199191	0,146690897
SLC7A9	protein_coding	ENSG00000021488	0,545189263	0,087213841	0,146703509
AC017071.1	lincRNA	ENSG00000259915	0,539778257	0,087249881	0,146740062
IKBKG	protein_coding	ENSG00000269335	-0,163966893	0,087245108	0,146740062
ERCC2	protein_coding	ENSG00000104884	-0,09245669	0,087321349	0,146848219
ZFR	protein_coding	ENSG00000056097	0,079486815	0,087463308	0,14707489
ALMS1P1	transcribed_unprocessed	ENSG00000163016	0,46298687	0,087507432	0,147137026
IFITM1	protein_coding	ENSG00000185885	0,561910009	0,087526963	0,147151776
FAM149A	protein_coding	ENSG00000109794	-0,427158783	0,087530554	0,147151776
AGBL5	protein_coding	ENSG00000084693	-0,09478469	0,08757422	0,147213118

CABP1	protein_coding	ENSG00000157782	0,558991694	0,087688571	0,147393262
AC128657.1	processed_pseudogene	ENSG00000258073	-0,565808228	0,087803643	0,14757459
AC128709.1	lincRNA	ENSG00000229912	0,557350418	0,087897638	0,147720465
RPS4XP13	processed_pseudogene	ENSG00000240371	0,548149272	0,087993673	0,147869744
MT-CYB	protein_coding	ENSG00000198727	0,322513546	0,088014849	0,147893212
RAB13	protein_coding	ENSG00000143545	-0,086440667	0,088023137	0,147895022
SHANK2	protein_coding	ENSG00000162105	0,437845647	0,088084971	0,147986791
DOCK3	protein_coding	ENSG00000088538	0,384675121	0,088142776	0,148071779
DNASE1L2	protein_coding	ENSG00000167968	-0,479621316	0,088208295	0,148169708
CYB561D2	protein_coding	ENSG00000114395	-0,156461407	0,088310936	0,148329972
POC1B	protein_coding	ENSG00000139323	0,142002718	0,088347813	0,148379762
NOL6	protein_coding	ENSG00000165271	0,131593193	0,088385805	0,148431415
EIF4A1P10	processed_pseudogene	ENSG00000229132	-0,292172131	0,088427466	0,14848922
GSTP1	protein_coding	ENSG00000084207	0,119402995	0,088482661	0,148569741
SLC25A4	protein_coding	ENSG00000151729	-0,143642797	0,088525792	0,148629993
ACTA1	protein_coding	ENSG00000143632	0,560401264	0,088626089	0,148786206
LMBR1L	protein_coding	ENSG00000139636	-0,173698303	0,08872946	0,148947555
LINC01513	antisense	ENSG00000246016	0,537576679	0,088739363	0,148951987
MAGEC2	protein_coding	ENSG00000046774	-0,155723166	0,088823228	0,149080557
AC005000.1	processed_pseudogene	ENSG00000228532	-0,506530964	0,088891656	0,149183197
METTL7B	protein_coding	ENSG00000170439	0,451620505	0,08905206	0,149440169
CATSPER2	protein_coding	ENSG00000166762	0,35733578	0,089063921	0,149447846
AC010735.1	sense_intronic	ENSG00000261379	0,533149705	0,089097619	0,14949216
MTHFS	protein_coding	ENSG00000136371	-0,469083485	0,089390707	0,149971649
DYNLL1	protein_coding	ENSG00000088986	-0,094152404	0,089407354	0,149987309
AC113382.1	antisense	ENSG00000246323	0,493390977	0,089461166	0,150065308
AL160314.2	processed_transcript	ENSG00000258458	0,479797774	0,089562461	0,150222937
GPD1	protein_coding	ENSG00000167588	0,498995368	0,089624126	0,150314075
AC104964.4	lincRNA	ENSG00000272505	0,553448695	0,089730539	0,150480242
CD40	protein_coding	ENSG00000101017	0,123073479	0,089760763	0,15051862
AC097493.4	unprocessed_pseudoge	ENSG00000284648	-0,430801644	0,089772692	0,150526317
AC011510.1	antisense	ENSG00000269652	0,440871542	0,089834212	0,150617157
LINC02621	lincRNA	ENSG00000234756	0,521822409	0,089865634	0,150657524
AC026271.3	sense_intronic	ENSG00000264885	-0,277266643	0,089887601	0,150682034
AC023669.1	lincRNA	ENSG00000229459	0,473744629	0,089983291	0,150830116
PRORSD1P	transcribed_unitary_pse	ENSG00000162997	-0,480374516	0,089998274	0,150842903
MCTS1	protein_coding	ENSG00000232119	-0,103157922	0,09002786	0,150880161
JPH1	protein_coding	ENSG00000104369	-0,479569652	0,090142642	0,151060183
FAT3	protein_coding	ENSG00000165323	0,51844357	0,090175733	0,151103291
TBC1D7	protein_coding	ENSG00000145979	-0,135716807	0,090214983	0,151156711

TBC1D7	protein_coding	ENSG00000145979	-0,135716807	0,090214983	0,151156711
EEF1B2	protein_coding	ENSG00000114942	-0,133630723	0,090228247	0,151166586
AC068234.2	antisense	ENSG00000263293	0,546021	0,090251295	0,151192851
TH	protein_coding	ENSG00000180176	0,545450388	0,09032328	0,151301083
TUBGCP2	protein_coding	ENSG00000130640	-0,081003827	0,090351224	0,151323176
AL645929.1	unprocessed_pseudoge	ENSG00000230521	0,548128171	0,090344292	0,151323176
AC008781.1	antisense	ENSG00000228737	0,541994361	0,090360094	0,151325674
AC020661.1	lincRNA	ENSG00000251161	0,496674347	0,09071747	0,151911767
BMP4	protein_coding	ENSG00000125378	0,246324956	0,090725011	0,151911991
FABP4	protein_coding	ENSG00000170323	0,308979508	0,090816467	0,152052713
AC092999.1	TEC	ENSG00000279311	0,552083943	0,09082971	0,152062473
RN7SKP185	misc_RNA	ENSG00000199683	0,517079135	0,090875215	0,152126238
AC120049.1	lincRNA	ENSG00000267414	-0,494511316	0,09089831	0,15215248
SIRT7	protein_coding	ENSG00000187531	0,150430083	0,090933725	0,152199339
KDM5C-IT1	sense_intronic	ENSG00000235262	0,510740583	0,090945994	0,152207453
AC005838.2	lincRNA	ENSG00000266538	0,537863763	0,091091407	0,152438377
DAPK1	protein_coding	ENSG00000196730	0,452919548	0,091117969	0,152465393
BOD1L1	protein_coding	ENSG00000038219	0,147064564	0,091129851	0,152465393
CSTB	protein_coding	ENSG00000160213	0,093903272	0,091124778	0,152465393
LINC01268	lincRNA	ENSG00000227502	0,451369409	0,091163082	0,152508549
GUSB	protein_coding	ENSG00000169919	-0,09101965	0,091171792	0,152510681
FKTN	protein_coding	ENSG00000106692	-0,107902277	0,091227754	0,152591848
PRRG2	protein_coding	ENSG00000126460	-0,527900227	0,091251522	0,152619157
AC103810.2	antisense	ENSG00000265218	0,52761491	0,091266578	0,152628302
NSMCE1-DT	antisense	ENSG00000245888	0,525296653	0,091271874	0,152628302
GRK2	protein_coding	ENSG00000173020	0,128623055	0,091311202	0,152681621
DIRC3	lincRNA	ENSG00000231672	0,520467305	0,091368032	0,152764191
MAP2K6	protein_coding	ENSG00000108984	-0,546511374	0,091398423	0,152802548
EDIL3	protein_coding	ENSG00000164176	0,224864825	0,091550697	0,153044648
EEF1A1P13	processed_pseudogene	ENSG00000250182	-0,32126106	0,091577392	0,153076798
HLA-DPA1	protein_coding	ENSG00000231389	-0,109979672	0,091628594	0,153149903
MCUR1	protein_coding	ENSG00000050393	-0,07844134	0,091644224	0,153163546
AC244517.1	lincRNA	ENSG00000272108	0,541127528	0,091668351	0,153191386
AC107204.1	lincRNA	ENSG00000281392	0,529980152	0,091690409	0,153215764
MGAT3	protein_coding	ENSG00000128268	0,537581771	0,091744969	0,153294444
GSPT1	protein_coding	ENSG00000103342	-0,075306029	0,091866958	0,15348577
NEK5	protein_coding	ENSG00000197168	-0,49001254	0,091888304	0,153508929
PAPPA-AS1	antisense	ENSG00000256040	0,546690291	0,091923502	0,153555223
RPS27A	protein_coding	ENSG00000143947	-0,12609824	0,091938034	0,15356699
BTBD9	protein_coding	ENSG00000183826	-0,106739598	0,091978729	0,153622455

COX14	protein_coding	ENSG00000178449	-0,129707202	0,092130627	0,153863624
AC120114.3	TEC	ENSG00000279789	0,488567692	0,092237981	0,154030369
THUMPD3-AS1	antisense	ENSG00000206573	0,13294323	0,09231794	0,154151343
AL118506.1	antisense	ENSG00000268858	0,176403847	0,09233975	0,15416266
NEUROD2	protein_coding	ENSG00000171532	0,490546634	0,092335745	0,15416266
ZNF264	protein_coding	ENSG00000083844	0,111807559	0,092373914	0,154207144
AP001437.1	antisense	ENSG00000273210	0,542530718	0,092409556	0,154235027
LINC01775	lincRNA	ENSG00000267201	-0,415662052	0,092413176	0,154235027
STARD13	protein_coding	ENSG00000133121	-0,092900301	0,092408378	0,154235027
MTF1	protein_coding	ENSG00000188786	-0,124022245	0,092459161	0,154299219
RPSAP16	processed_pseudogene	ENSG00000235145	0,484888106	0,092553668	0,154444369
DHX29	protein_coding	ENSG00000067248	0,089213702	0,092611641	0,154528535
AC087500.1	antisense	ENSG00000261879	0,313515094	0,09263237	0,15455055
EDEM3	protein_coding	ENSG00000116406	-0,103883987	0,092651487	0,154569872
NAA50	protein_coding	ENSG00000121579	-0,079298607	0,092750569	0,154722585
THAP7-AS1	antisense	ENSG00000230513	-0,227172193	0,092847028	0,154870897
PSMD1	protein_coding	ENSG00000173692	0,053120429	0,092873894	0,154903112
TNS1	protein_coding	ENSG00000079308	0,491417452	0,092885966	0,154905945
PRKD1	protein_coding	ENSG00000184304	0,134972403	0,092890698	0,154905945
RSPH10B	protein_coding	ENSG00000155026	0,518759385	0,092940799	0,154976894
LNP1	protein_coding	ENSG00000206535	-0,18244679	0,092974737	0,15502088
AC243829.1	antisense	ENSG00000274767	0,45167649	0,093050578	0,155130055
AC124798.1	antisense	ENSG00000260196	0,280854881	0,093055342	0,155130055
AC011603.2	antisense	ENSG00000258017	-0,38914998	0,093078418	0,155143304
USP2	protein_coding	ENSG00000036672	0,384105913	0,093074986	0,155143304
CAAP1	protein_coding	ENSG00000120159	0,128938924	0,093105375	0,155175626
SIGLEC15	protein_coding	ENSG00000197046	0,47546506	0,093133055	0,155209145
ERGIC2	protein_coding	ENSG00000087502	-0,137595666	0,093156568	0,155235717
AL732372.2	transcribed_unprocesse	ENSG00000237094	0,544100375	0,09324189	0,155365275
FRG1HP	transcribed_unprocesse	ENSG00000276291	0,132593403	0,093377153	0,155578019
AC048382.6	antisense	ENSG00000276278	0,543127803	0,09340521	0,155612125
RCN3	protein_coding	ENSG00000142552	-0,368766031	0,093444928	0,155665649
AC117505.1	antisense	ENSG00000257514	0,534688105	0,093475725	0,155704306
RND2	protein_coding	ENSG00000108830	0,506538803	0,09348838	0,155712739
THBS4	protein_coding	ENSG00000113296	0,541406757	0,093515927	0,155745973
AC011815.1	lincRNA	ENSG00000268573	-0,184474894	0,093618234	0,155903701
CR392039.4	processed_pseudogene	ENSG00000279720	-0,341657329	0,093636976	0,155922253
LINC01715	lincRNA	ENSG00000229388	-0,422752868	0,09367382	0,155969146
FBLN7	protein_coding	ENSG00000144152	0,537887286	0,093680347	0,155969146
STX1B	protein_coding	ENSG00000099365	0,378853288	0,093737825	0,156039508

RPS24	protein_coding	ENSG00000138326	-0,12307137	0,0937315	0,156039508
AC132192.2	antisense	ENSG00000268403	-0,266755491	0,09374679	0,156041767
TET1	protein_coding	ENSG00000138336	0,488897377	0,093795998	0,156111006
LTB4R	protein_coding	ENSG00000213903	0,302630573	0,093879223	0,156236845
AC137630.2	antisense	ENSG00000235236	0,546096993	0,093918988	0,15629034
CRYBB3	protein_coding	ENSG00000100053	0,539580182	0,093934391	0,156303291
CNTNAP3B	protein_coding	ENSG00000154529	0,369612377	0,093958258	0,156330321
TDRKH	protein_coding	ENSG00000182134	-0,130802566	0,093997668	0,156383207
AL596244.1	sense_overlapping	ENSG00000261534	0,402174242	0,094011227	0,156393079
ERP27	protein_coding	ENSG00000139055	0,537853503	0,094078933	0,156493019
AC073343.2	antisense	ENSG00000228010	0,535913584	0,094235636	0,15674097
ACOT8	protein_coding	ENSG00000101473	0,144954383	0,094306349	0,156845866
TAS1R3	protein_coding	ENSG00000169962	0,520681968	0,094364154	0,156929278
AC023908.3	antisense	ENSG00000261136	0,491941438	0,094408028	0,156989514
RPRD1A	protein_coding	ENSG00000141425	0,093887874	0,094444351	0,157037182
TMEM40	protein_coding	ENSG00000088726	0,107995234	0,09452279	0,157144012
RPS6	protein_coding	ENSG00000137154	-0,137638947	0,094523924	0,157144012
AC124944.3	transcribed_unprocesse	ENSG00000260261	0,383177034	0,094611367	0,157276637
DNASE1L1	protein_coding	ENSG00000013563	0,127227217	0,094637323	0,157307035
AC009237.14	lincRNA	ENSG00000272913	-0,142014701	0,094688881	0,157367227
CEP295NL	protein_coding	ENSG00000178404	0,469766889	0,094681412	0,157367227
AC007342.7	TEC	ENSG00000279722	-0,502119946	0,094696758	0,157367567
LINC00476	lincRNA	ENSG00000175611	0,342111475	0,094861694	0,157628887
BLMH	protein_coding	ENSG00000108578	-0,082727875	0,094908533	0,157693942
DCAF17	protein_coding	ENSG00000115827	0,086000461	0,094965944	0,157776551
INTU	protein_coding	ENSG00000164066	0,159053077	0,094980344	0,157787692
EXOC3L4	protein_coding	ENSG00000205436	0,356193946	0,095041137	0,157875899
CREG1	protein_coding	ENSG00000143162	0,173951074	0,095076294	0,157916726
ZNF205	protein_coding	ENSG00000122386	0,138178518	0,095088814	0,157916726
MMD	protein_coding	ENSG00000108960	-0,129080483	0,095081633	0,157916726
EIF2D	protein_coding	ENSG00000143486	-0,075376743	0,095121691	0,15794838
YWHAZ	protein_coding	ENSG00000164924	-0,096155365	0,095123276	0,15794838
GMDS	protein_coding	ENSG00000112699	-0,110753406	0,095148461	0,157977409
TMSB4XP8	processed_pseudogene	ENSG00000187653	0,324681812	0,095163935	0,157990311
PITPNA	protein_coding	ENSG00000174238	-0,067994959	0,095314705	0,158218885
TCP1P1	unprocessed_pseudoge	ENSG00000229161	-0,52559429	0,095317042	0,158218885
LINC01626	lincRNA	ENSG00000223765	0,380110125	0,095328244	0,158224672
HUS1	protein_coding	ENSG00000136273	-0,092808887	0,095406733	0,158342134
PSMG3-AS1	lincRNA	ENSG00000230487	0,363332021	0,095495532	0,158476686
MRPS24	protein_coding	ENSG00000062582	0,459536909	0,095531199	0,158523049

NDUFS8	protein_coding	ENSG00000110717	-0,115733125	0,095559114	0,158556543
TNRC6C-AS1	processed_transcript	ENSG00000204282	0,489083513	0,095573263	0,158567191
BCL7B	protein_coding	ENSG00000106635	-0,089409433	0,095622933	0,158636768
TEX22	protein_coding	ENSG00000226174	0,410652706	0,095755978	0,158818949
MED27	protein_coding	ENSG00000160563	0,102633054	0,095754884	0,158818949
SNAP23	protein_coding	ENSG00000092531	0,094831347	0,095776165	0,158839585
PINCR	lincRNA	ENSG00000224294	0,232175318	0,095971298	0,159150333
LINC02449	lincRNA	ENSG00000215241	-0,524251594	0,096051364	0,159270231
OLA1	protein_coding	ENSG00000138430	0,116411078	0,096103242	0,159343371
CHPF	protein_coding	ENSG00000123989	-0,156325128	0,096124034	0,159364961
STAG3	protein_coding	ENSG00000066923	0,520385698	0,096256427	0,159571557
CENPS	protein_coding	ENSG00000175279	-0,269894013	0,096397759	0,15979294
AC005076.1	antisense	ENSG00000224046	0,350784244	0,096427112	0,159816727
SGPP1	protein_coding	ENSG00000126821	0,103231239	0,096435486	0,159816727
AC068888.2	sense_intronic	ENSG00000257475	-0,538596172	0,096435376	0,159816727
EEF1DP3	transcribed_unprocesse	ENSG00000229715	0,532210482	0,096501297	0,159912871
ALPL	protein_coding	ENSG00000162551	0,265587551	0,096561782	0,160000174
SCGB2B2	protein_coding	ENSG00000205209	0,5358166	0,096581445	0,160019826
AC009088.1	antisense	ENSG00000260060	-0,536624823	0,096802197	0,160361748
FRS3	protein_coding	ENSG00000137218	0,224372355	0,096803452	0,160361748
MGLL	protein_coding	ENSG00000074416	0,104329504	0,096899736	0,160508286
ARHGAP32	protein_coding	ENSG00000134909	-0,137920995	0,09694071	0,160563189
CELSR2	protein_coding	ENSG00000143126	-0,260449169	0,096969311	0,160597591
AC130324.3	antisense	ENSG00000275185	-0,363151493	0,096983133	0,160604087
AKAP7	protein_coding	ENSG00000118507	0,203210296	0,096988894	0,160604087
AC087289.6	antisense	ENSG00000267801	0,526874994	0,097003002	0,16061448
RASSF10	protein_coding	ENSG00000189431	-0,468365559	0,097047533	0,160675242
PRR36	protein_coding	ENSG00000183248	-0,535370426	0,097060204	0,16068325
CASC9	lincRNA	ENSG00000249395	0,526653695	0,097079241	0,160701795
AL355472.1	processed_pseudogene	ENSG00000235605	-0,539561618	0,097117955	0,160752906
AC142381.3	transcribed_unprocesse	ENSG00000260628	0,461624648	0,097183351	0,160835191
AC015813.5	TEC	ENSG00000279069	-0,279695699	0,097287182	0,160994038
EOMES	protein_coding	ENSG00000163508	-0,348096178	0,097433811	0,161223676
GUF1	protein_coding	ENSG00000151806	0,101643835	0,097469956	0,161270474
KRT8P11	processed_pseudogene	ENSG00000255815	-0,519840499	0,097483856	0,161280462
BEX5	protein_coding	ENSG00000184515	0,373036135	0,097573458	0,161415681
RNU6-1	snRNA	ENSG00000206625	0,530697138	0,097601757	0,161449474
ETFBKMT	protein_coding	ENSG00000139160	-0,21585536	0,097658817	0,161530834
PTK2	protein_coding	ENSG00000169398	0,06278223	0,097733655	0,161641582
FETUB	protein_coding	ENSG00000090512	0,353801906	0,09783711	0,161799639

EBNA1BP2	protein_coding	ENSG00000117395	-0,091811805	0,097954777	0,161981172
WDR20	protein_coding	ENSG00000140153	-0,104575909	0,097970809	0,161994622
BRWD3	protein_coding	ENSG00000165288	0,110152754	0,097983086	0,162001862
METTL21AP1	processed_pseudogene	ENSG00000229623	-0,426292018	0,098119765	0,162214766
RPS4XP17	processed_pseudogene	ENSG00000244097	0,499411336	0,098168122	0,162281631
RNU6-126P	snRNA	ENSG00000252494	-0,414899999	0,098235701	0,162380257
HIBCH	protein_coding	ENSG00000198130	-0,090703309	0,098328498	0,162520549
AL929236.1	sense_overlapping	ENSG00000228470	0,522642573	0,098366758	0,162570686
AC091053.1	antisense	ENSG00000254665	-0,469311287	0,098478478	0,162742211
MZT2B	protein_coding	ENSG00000152082	-0,14136054	0,098507351	0,162776811
GXYLT2	protein_coding	ENSG00000172986	0,192960854	0,098563055	0,162839611
MOB2	protein_coding	ENSG00000182208	0,163361655	0,098560885	0,162839611
LTO1	protein_coding	ENSG00000149716	-0,150362587	0,098569175	0,162839611
METTL25	protein_coding	ENSG00000127720	-0,18658112	0,09857792	0,162840943
AC013731.1	lincRNA	ENSG00000270557	0,475612789	0,098735008	0,163087301
PLD3	protein_coding	ENSG00000105223	0,11707526	0,098784237	0,163155476
PC	protein_coding	ENSG00000173599	-0,130607729	0,098808291	0,163182064
AC009108.5	TEC	ENSG00000280278	0,49328431	0,098942285	0,163390199
GPR89A	protein_coding	ENSG00000117262	0,188538961	0,098968974	0,163421114
AL121753.1	protein_coding	ENSG00000261582	0,5163443	0,098989751	0,163442264
CSE1L-AS1	antisense	ENSG00000227431	0,530985043	0,099010104	0,163462709
PFKM	protein_coding	ENSG00000152556	-0,066815282	0,099035102	0,163490819
TUBB3	protein_coding	ENSG00000258947	-0,240761192	0,099056774	0,163513435
MAP7D2	protein_coding	ENSG00000184368	0,439975729	0,099077294	0,163520987
N4BP2	protein_coding	ENSG00000078177	0,118073653	0,0990736	0,163520987
IL17RE	protein_coding	ENSG00000163701	0,239126296	0,099164762	0,163652178
HMGB3P32	processed_pseudogene	ENSG00000260828	0,494486332	0,099179491	0,163663317
TMEM120A	protein_coding	ENSG00000189077	-0,139278451	0,099209956	0,163700418
RAP1A	protein_coding	ENSG00000116473	-0,124621761	0,099229261	0,163719101
AP001160.3	antisense	ENSG00000269176	0,451736061	0,099274721	0,163764728
PTGR2	protein_coding	ENSG00000140043	-0,234795137	0,099280869	0,163764728
NOTCH2NLA	protein_coding	ENSG00000264343	0,274592194	0,099322121	0,163819598
NOTCH2NLA	protein_coding	ENSG00000264343	0,274592194	0,099322121	0,163819598
SEMA4C	protein_coding	ENSG00000168758	-0,148586086	0,099357199	0,163864278
AC079305.1	antisense	ENSG00000222043	0,433498103	0,099448918	0,164002356
AP000894.4	lincRNA	ENSG00000273355	0,519383528	0,099464283	0,164014507
CFL1P5	processed_pseudogene	ENSG00000213830	-0,529077747	0,099506936	0,164071651
CPA5	protein_coding	ENSG00000158525	0,510753982	0,099613093	0,164220283
TPMT	protein_coding	ENSG00000137364	0,112431672	0,09961166	0,164220283
SNRPGP10	processed_pseudogene	ENSG00000235363	-0,216436335	0,099760253	0,164449669

TNC	protein_coding	ENSG00000041982	0,391533314	0,099855973	0,164588386
AC018926.2	sense_intronic	ENSG00000276533	0,454957337	0,099860452	0,164588386
AC027020.2	lincRNA	ENSG00000270127	0,518881928	0,099885636	0,164616665
ASB6	protein_coding	ENSG00000148331	0,106002821	0,09991239	0,164647526
LINC01088	processed_transcript	ENSG00000249307	0,528655272	0,099955905	0,164706002
AC134349.2	unprocessed_pseudoge	ENSG00000256981	-0,488887241	0,099970589	0,164716964
LRRC6	protein_coding	ENSG00000129295	0,331220772	0,099998597	0,164744054
PFN1	protein_coding	ENSG00000108518	-0,090883775	0,100003095	0,164744054
RPL22P2	processed_pseudogene	ENSG00000241081	0,49440079	0,100048412	0,164805472
ATP5PB	protein_coding	ENSG00000116459	-0,104388856	0,100057694	0,164807524
CLMAT3	antisense	ENSG00000249035	0,467705324	0,100096773	0,164858654
LINC00896	lincRNA	ENSG00000236499	-0,523382902	0,10010725	0,164862671
JMJD1C-AS1	antisense	ENSG00000272767	-0,332142036	0,100211217	0,16502064
RAB11B	protein_coding	ENSG00000185236	0,113864971	0,100248932	0,165069492
AC109322.1	antisense	ENSG00000255224	0,410203367	0,100277993	0,165104089
AC139530.1	protein_coding	ENSG00000185527	0,377147766	0,100381333	0,165260968
ANG	protein_coding	ENSG00000214274	-0,223843133	0,100468385	0,165391009
LYPD3	protein_coding	ENSG00000124466	-0,463225705	0,100497855	0,165426245
PITPNB	protein_coding	ENSG00000180957	0,110614355	0,100523431	0,165428705
LYPLA1	protein_coding	ENSG00000120992	-0,14442785	0,100523547	0,165428705
ZSCAN22	protein_coding	ENSG00000182318	-0,183400821	0,10052292	0,165428705
ZNF565	protein_coding	ENSG00000196357	-0,212600556	0,100659603	0,165639318
GTF3C1	protein_coding	ENSG00000077235	0,115849181	0,100721996	0,165728692
AC002056.1	processed_pseudogene	ENSG00000213683	0,517648796	0,100789196	0,165812658
CAMK1	protein_coding	ENSG00000134072	-0,134628154	0,100781882	0,165812658
PLXDC1	protein_coding	ENSG00000161381	0,451559419	0,1009045	0,165989036
CSNK1E	protein_coding	ENSG00000213923	-0,121472061	0,10092077	0,166002486
GNG10	protein_coding	ENSG00000242616	-0,465525584	0,100930328	0,166004895
AC073111.5	protein_coding	ENSG00000284691	-0,42693465	0,100970229	0,166057204
KLHDC10	protein_coding	ENSG00000128607	0,089088549	0,101060763	0,166192772
OR2A7	protein_coding	ENSG00000243896	0,521800347	0,101189117	0,166388971
AC006213.1	protein_coding	ENSG00000159905	-0,163099975	0,101196296	0,166388971
LINC00653	lincRNA	ENSG00000273148	-0,513175459	0,101283763	0,166519436
AL162724.1	sense_intronic	ENSG00000227200	-0,511219259	0,101300091	0,166532933
AC009269.3	antisense	ENSG00000259624	0,508493319	0,10132578	0,166548464
ZNF524	protein_coding	ENSG00000171443	0,182104876	0,101318575	0,166548464
EPOP	protein_coding	ENSG00000273604	-0,118297204	0,101337974	0,16655516
TMEM59	protein_coding	ENSG00000116209	0,095843514	0,101366751	0,166589107
PRDM12	protein_coding	ENSG00000130711	-0,529097826	0,101422749	0,166654429
FAM47E	protein_coding	ENSG00000189157	0,46192842	0,101472353	0,16672258

FUNDC1	protein_coding	ENSG00000069509	-0,115148652	0,10150236	0,166758524
AC009061.2	antisense	ENSG00000270049	-0,315848537	0,101567346	0,16684639
NOG	protein_coding	ENSG00000183691	0,136307046	0,101572113	0,16684639
HNRNPA1P4	processed_pseudogene	ENSG00000206228	-0,507931099	0,101631275	0,166916836
ACADS	protein_coding	ENSG00000122971	-0,193702768	0,101628989	0,166916836
CALU	protein_coding	ENSG00000128595	-0,082060748	0,101645379	0,166926634
RPS19P3	processed_pseudogene	ENSG00000240463	0,526918217	0,101670853	0,166940148
GPHN	protein_coding	ENSG00000171723	-0,115517247	0,101664347	0,166940148
AL356966.1	sense_intronic	ENSG00000224356	-0,523285727	0,101685386	0,166940148
AL078621.3	TEC	ENSG00000279267	0,444403526	0,101686166	0,166940148
TOGARAM2	protein_coding	ENSG00000189350	0,49288828	0,101807765	0,167126401
AC106886.5	protein_coding	ENSG00000282034	0,320265073	0,101832382	0,167153434
WASH9P	unprocessed_pseudoge	ENSG00000279457	0,219100087	0,101866352	0,167195813
EEF1A1P7	processed_pseudogene	ENSG00000268222	-0,498885976	0,101956342	0,167330126
UPF1	protein_coding	ENSG00000005007	-0,124207536	0,102022587	0,167412054
AC026124.2	sense_intronic	ENSG00000276853	-0,483757275	0,102021529	0,167412054
C12orf29	protein_coding	ENSG00000133641	-0,142919323	0,102043075	0,167432277
UBE3B	protein_coding	ENSG00000151148	0,089494992	0,102223111	0,167714263
PPIE	protein_coding	ENSG00000084072	-0,084039453	0,102304851	0,167834945
RPL35	protein_coding	ENSG00000136942	-0,134010291	0,102488418	0,168122647
SOBP	protein_coding	ENSG00000112320	-0,317553043	0,102589421	0,168274873
NPRL3	protein_coding	ENSG00000103148	-0,136872824	0,102670175	0,168393866
TMEM71	protein_coding	ENSG00000165071	-0,174276411	0,102688313	0,168410147
CROT	protein_coding	ENSG00000005469	-0,125977727	0,102749403	0,168496862
SLIT1	protein_coding	ENSG00000187122	0,494030818	0,102786831	0,168544686
STRADB	protein_coding	ENSG00000082146	-0,103104598	0,102795001	0,168544686
PDSS1	protein_coding	ENSG00000148459	-0,157005425	0,102852819	0,168626005
HOXA3	protein_coding	ENSG00000105997	-0,407508277	0,102902279	0,168693609
STARD6	protein_coding	ENSG00000174448	0,412526829	0,102919	0,168707535
ENTPD8	protein_coding	ENSG00000188833	-0,447387695	0,102931594	0,168714696
C6orf201	protein_coding	ENSG00000185689	0,517345485	0,10294653	0,168725694
APBB2	protein_coding	ENSG00000163697	0,074706028	0,102959177	0,168732938
AL162274.2	antisense	ENSG00000277959	-0,459778476	0,103082563	0,168921649
AC016831.5	lincRNA	ENSG00000271204	0,415849183	0,103127075	0,16898109
LINC00667	lincRNA	ENSG00000263753	0,123245662	0,103185298	0,169062986
SLC25A20	protein_coding	ENSG00000178537	0,130308814	0,103195261	0,169065804
AL137003.1	antisense	ENSG00000229931	0,360069245	0,103246558	0,169136333
ZNF555	protein_coding	ENSG00000186300	0,165804345	0,103277013	0,169172713
SNORA28	snoRNA	ENSG00000272533	-0,335764064	0,103299693	0,16919635
RPL11	protein_coding	ENSG00000142676	-0,116028017	0,103481279	0,169480239

SLC25A14	protein_coding	ENSG00000102078	-0,126557511	0,103545776	0,169560746
AC005785.2	TEC	ENSG00000279203	0,48414957	0,103546969	0,169560746
CHMP4BP1	processed_pseudogene	ENSG00000258469	-0,522319242	0,103686195	0,169775176
AL139161.1	lincRNA	ENSG00000229291	0,48919313	0,103701023	0,169781771
USP7	protein_coding	ENSG00000187555	-0,102676409	0,103706778	0,169781771
CALHM5	protein_coding	ENSG00000178033	0,110000528	0,103755352	0,169847734
RF00100	misc_RNA	ENSG00000271394	0,463508163	0,103792924	0,169895681
LINC00484	lincRNA	ENSG00000235641	0,519676881	0,103825514	0,169935464
CCDC40	protein_coding	ENSG00000141519	-0,277070315	0,103839445	0,169944703
AL133243.4	TEC	ENSG00000279544	0,487350496	0,103942051	0,170099056
AL079301.1	sense_intronic	ENSG00000230922	-0,45949597	0,104009487	0,170195834
PRR19	protein_coding	ENSG00000188368	-0,336937324	0,104027274	0,170211359
NNMT	protein_coding	ENSG00000166741	0,098554141	0,10403707	0,170213808
GABRR2	protein_coding	ENSG00000111886	0,493119358	0,104048772	0,170219375
PCBP3	protein_coding	ENSG00000183570	0,504179264	0,104101928	0,170292754
CFAP410	protein_coding	ENSG00000160226	-0,224041448	0,104133256	0,170330416
ACAP3	protein_coding	ENSG00000131584	0,172637384	0,104279528	0,17055607
TEX9	protein_coding	ENSG00000151575	0,195462371	0,10439178	0,17072605
KHDRBS1	protein_coding	ENSG00000121774	-0,0711273	0,104481277	0,170846474
NOC2L	protein_coding	ENSG00000188976	-0,094784622	0,104483953	0,170846474
TTPA	protein_coding	ENSG00000137561	-0,376227611	0,104490404	0,170846474
DIAPH2	protein_coding	ENSG00000147202	0,093872557	0,104521406	0,170883543
HOXB7	protein_coding	ENSG00000260027	-0,097687847	0,10465327	0,171085491
CUTA	protein_coding	ENSG00000112514	-0,101823733	0,104702952	0,171153068
ANXA10	protein_coding	ENSG00000109511	-0,516782243	0,104793441	0,171287335
STRN4	protein_coding	ENSG00000090372	-0,084657791	0,104821379	0,171319347
IDH3B	protein_coding	ENSG00000101365	-0,064020353	0,105043299	0,171668371
CNKSR3	protein_coding	ENSG00000153721	0,09677255	0,105184489	0,171885417
AC012254.1	antisense	ENSG00000266957	0,46073421	0,105223645	0,171935705
NPTX1	protein_coding	ENSG00000171246	0,504812235	0,105250007	0,17196508
ATP5PBP5	processed_pseudogene	ENSG00000254944	0,51240607	0,105369097	0,172132232
TMTC2	protein_coding	ENSG00000179104	-0,193507889	0,105361415	0,172132232
SS18L2	protein_coding	ENSG00000008324	-0,147109046	0,105392225	0,172156303
LINC01615	lincRNA	ENSG00000223485	0,145769064	0,105410056	0,172171716
PRKRA	protein_coding	ENSG00000180228	0,093925393	0,105434783	0,172198391
LINC00639	lincRNA	ENSG00000259070	0,417561655	0,105453162	0,172214695
DPAGT1	protein_coding	ENSG00000172269	-0,09635603	0,105558806	0,172373496
AC006330.1	TEC	ENSG00000280388	0,510027746	0,10559288	0,17241541
MRPL49	protein_coding	ENSG00000149792	0,094510309	0,105664256	0,172516062
NR1H3	protein_coding	ENSG00000025434	-0,205457915	0,105671345	0,172516062

C1orf53	protein_coding	ENSG00000203724	-0,205115376	0,105797754	0,172708686
AC005532.2	processed_pseudogene	ENSG00000244167	0,501135116	0,105886482	0,172839772
PDP1	protein_coding	ENSG00000164951	0,077403677	0,10594821	0,172926769
FAM227A	protein_coding	ENSG00000184949	0,310197381	0,105974822	0,172956441
PPP1R9B	protein_coding	ENSG00000108819	0,127366931	0,106047132	0,173060684
TMEM68	protein_coding	ENSG00000167904	-0,129035159	0,106131911	0,173185257
SYMPK	protein_coding	ENSG00000125755	-0,099162695	0,106206881	0,173293804
AC009126.1	antisense	ENSG00000247121	0,375657491	0,106276529	0,173393652
AC084824.3	sense_intronic	ENSG00000274105	0,489385348	0,106301134	0,173420002
GAS6-AS1	antisense	ENSG00000233695	-0,223117305	0,106357643	0,173457001
MKRN1	protein_coding	ENSG00000133606	0,077442596	0,106353922	0,173457001
KCNH1-IT1	sense_intronic	ENSG00000234233	-0,423334625	0,10634231	0,173457001
ZDHHC8P1	transcribed_unprocesse	ENSG00000133519	-0,200896886	0,106351742	0,173457001
AP000525.1	sense_intronic	ENSG00000272872	0,351420694	0,106495143	0,173667439
RPS4XP16	transcribed_processed_	ENSG00000224892	0,433922232	0,106522338	0,173697976
AC008608.2	lincRNA	ENSG00000271737	-0,443528564	0,106557956	0,173742243
TAS2R15P	unprocessed_pseudoge	ENSG00000212125	0,500904333	0,106578737	0,173762313
PPM1L	protein_coding	ENSG00000163590	0,23184701	0,106603933	0,173789576
CCDC151	protein_coding	ENSG00000198003	0,405001777	0,106615785	0,173795085
AC021016.2	sense_overlapping	ENSG00000261338	0,38277628	0,106743432	0,173989333
ATXN1L	protein_coding	ENSG00000224470	-0,12112476	0,106761156	0,174004396
TAF1D	protein_coding	ENSG00000166012	0,086905525	0,106842764	0,174109732
SNORD11B	snoRNA	ENSG00000271852	-0,510806992	0,106842494	0,174109732
PTPRG	protein_coding	ENSG00000144724	0,077984937	0,10686535	0,174132702
AC006504.3	processed_pseudogene	ENSG00000267264	-0,488356362	0,106903062	0,174180315
AL391121.1	lincRNA	ENSG00000272933	0,514694987	0,106918069	0,174190929
PKN1	protein_coding	ENSG00000123143	0,10347834	0,107022744	0,174347616
RPL39	protein_coding	ENSG00000198918	-0,132017128	0,107033894	0,174351931
MYO7A	protein_coding	ENSG00000137474	0,252955088	0,107073061	0,17440188
AC017074.1	lincRNA	ENSG00000227359	0,441433659	0,107382547	0,174892086
AC096582.3	protein_coding	ENSG00000283247	0,50427193	0,107393487	0,174896015
AC017048.3	lincRNA	ENSG00000272551	0,434650241	0,107402766	0,17489724
C3orf80	protein_coding	ENSG00000180044	-0,507025661	0,107431213	0,174929675
AC003101.2	lincRNA	ENSG00000278546	-0,49275429	0,107478072	0,174992082
RNU6-36P	snRNA	ENSG00000206899	0,479375198	0,107492618	0,175001873
TRIM56	protein_coding	ENSG00000169871	0,123475497	0,107533871	0,17504128
AL133243.2	sense_intronic	ENSG00000276334	0,284717942	0,107533892	0,17504128
SMIM19	protein_coding	ENSG00000176209	0,129151717	0,107633791	0,17518999
GP6	protein_coding	ENSG00000088053	0,468274412	0,10768791	0,175264167
EMC10	protein_coding	ENSG00000161671	-0,102653365	0,107755367	0,175360039

PYGO1	protein_coding	ENSG00000171016	-0,187223968	0,10791637	0,17560812
TSHZ3	protein_coding	ENSG00000121297	-0,157226118	0,107973886	0,175687775
GTF2F2	protein_coding	ENSG00000188342	-0,09017393	0,10800664	0,175727129
DNAH10OS	antisense	ENSG00000250091	0,508433615	0,108059225	0,175798739
FAM156A	protein_coding	ENSG00000268350	0,475508154	0,108106522	0,175861735
RN7SL665P	misc_RNA	ENSG00000264169	0,417259177	0,108132893	0,175890684
ZIC5	protein_coding	ENSG00000139800	-0,126543668	0,108403438	0,176316773
STK19	protein_coding	ENSG00000204344	0,158316745	0,108755925	0,176875041
AC012513.3	TEC	ENSG00000279348	-0,187863403	0,108763921	0,176875041
NQO2	protein_coding	ENSG00000124588	0,103498756	0,108773382	0,176876403
AL359711.2	antisense	ENSG00000260273	0,452468661	0,108819527	0,176937411
LARGE1	protein_coding	ENSG00000133424	0,454927702	0,108857413	0,176984981
RDH16	protein_coding	ENSG00000139547	0,507345873	0,108890863	0,176997274
MOCS3	protein_coding	ENSG00000124217	0,103288528	0,108888786	0,176997274
KATNB1	protein_coding	ENSG00000140854	-0,106124226	0,108882591	0,176997274
EXPH5	protein_coding	ENSG00000110723	0,184781936	0,109177688	0,177449432
PCYOX1	protein_coding	ENSG00000116005	-0,071734027	0,109272889	0,177585933
COX7C	protein_coding	ENSG00000127184	-0,088859703	0,109278989	0,177585933
RNU2-17P	snRNA	ENSG00000222222	-0,491191232	0,109406072	0,177778365
RPS3A	protein_coding	ENSG00000145425	-0,140093836	0,109421837	0,177789897
VBP1	protein_coding	ENSG00000155959	-0,117463707	0,109447763	0,177817935
RYR2	protein_coding	ENSG00000198626	0,485394489	0,109578501	0,178016242
AC106799.2	lincRNA	ENSG00000248973	0,454878555	0,109651763	0,178121151
SLC25A27	protein_coding	ENSG00000153291	-0,310491556	0,109673455	0,178142279
LMCD1-AS1	antisense	ENSG00000227110	0,426171328	0,109719209	0,17819125
LINC01068	lincRNA	ENSG00000227676	0,465606458	0,109728611	0,17819125
BANF1P2	processed_pseudogene	ENSG00000230306	0,44194591	0,109729668	0,17819125
MBD1	protein_coding	ENSG00000141644	0,071133552	0,109813405	0,178313114
TOX4P1	processed_pseudogene	ENSG00000248697	0,429730083	0,109873016	0,178395785
GABPB2	protein_coding	ENSG00000143458	0,126269624	0,109943435	0,178495991
LRP4-AS1	antisense	ENSG00000247675	0,388043437	0,110059514	0,178670304
PTP4A2P1	processed_pseudogene	ENSG00000267185	0,469289561	0,110091076	0,178707398
GAB3	protein_coding	ENSG00000160219	0,283649235	0,110166217	0,178815219
ASS1P2	processed_pseudogene	ENSG00000223922	-0,499889662	0,110294066	0,17900857
SLC9C1	protein_coding	ENSG00000172139	0,468495041	0,110485796	0,179291374
FAM98B	protein_coding	ENSG00000171262	-0,113802064	0,110478132	0,179291374
AC096992.2	antisense	ENSG00000273486	0,429476876	0,110506191	0,179310282
TMEM126A	protein_coding	ENSG00000171202	-0,111423333	0,110535711	0,179343994
TCEANC	protein_coding	ENSG00000176896	0,257097623	0,110593457	0,179423494
AC091925.1	TEC	ENSG00000279130	0,498028009	0,110602885	0,179424597

SUZ12P1	transcribed_unprocesse	ENSG00000264538	0,119874938	0,110736784	0,179627605
CRIP1	protein_coding	ENSG00000213145	-0,507791925	0,110786871	0,179694641
POLRMTP1	processed_pseudogene	ENSG00000266066	0,470428114	0,110834743	0,179758071
AC073569.2	lincRNA	ENSG00000258048	0,496720756	0,110939214	0,179913283
AP003071.4	sense_overlapping	ENSG00000261625	0,476989836	0,110975608	0,179958073
RINL	protein_coding	ENSG00000187994	0,287727521	0,111043219	0,180039244
ZDHHC21	protein_coding	ENSG00000175893	-0,137104425	0,111042775	0,180039244
PRDX6	protein_coding	ENSG00000117592	0,062088403	0,111128851	0,18016384
GNA14	protein_coding	ENSG00000156049	0,465695153	0,111356902	0,18051929
C17orf97	protein_coding	ENSG00000187624	-0,302135873	0,111417278	0,18060289
AC113189.1	antisense	ENSG00000262624	0,50252444	0,111455212	0,180635828
AC011491.2	sense_overlapping	ENSG00000269444	-0,379223151	0,111453414	0,180635828
DAPP1	protein_coding	ENSG00000070190	0,499248885	0,111535566	0,180751775
FAM71E1	protein_coding	ENSG00000142530	0,479718982	0,111568793	0,180782978
FBXL8	protein_coding	ENSG00000135722	-0,32094508	0,111572449	0,180782978
RN7SKP203	misc_RNA	ENSG00000200488	0,301039574	0,111594169	0,180789606
AL133297.1	sense_intronic	ENSG00000260954	-0,502139502	0,111589863	0,180789606
AC009244.1	processed_pseudogene	ENSG00000238072	0,353211976	0,111634176	0,180840136
LRRC32	protein_coding	ENSG00000137507	0,405990902	0,11172092	0,180952071
TNFSF12	protein_coding	ENSG00000239697	-0,302389581	0,111717281	0,180952071
AC010247.2	antisense	ENSG00000259436	0,420228852	0,111780421	0,1810225
ATG7	protein_coding	ENSG00000197548	-0,091741594	0,111782056	0,1810225
AL583722.1	lincRNA	ENSG00000256050	-0,487039752	0,111939183	0,181262643
LINC00167	lincRNA	ENSG00000233220	-0,315379232	0,112001891	0,181327712
CNTF	protein_coding	ENSG00000242689	0,3295328	0,112002746	0,181327712
PRKCSH	protein_coding	ENSG00000130175	-0,099819675	0,112005889	0,181327712
SLC38A1	protein_coding	ENSG00000111371	0,093875046	0,112017134	0,181331604
EXOC4	protein_coding	ENSG00000131558	0,066760472	0,112083607	0,181424889
GPRC5B	protein_coding	ENSG00000167191	-0,242847477	0,112115851	0,181462761
ST13P20	transcribed_processed_	ENSG00000215875	0,323480865	0,112126734	0,181466055
ZBTB7A	protein_coding	ENSG00000178951	-0,117407056	0,112142187	0,181476745
HNRNPM	protein_coding	ENSG00000099783	-0,100523514	0,11215596	0,181484712
SLC17A7	protein_coding	ENSG00000104888	0,412588488	0,112308713	0,181717553
ORAI2	protein_coding	ENSG00000160991	0,127007558	0,112318327	0,181718772
RPS3AP38	processed_pseudogene	ENSG00000226318	0,457425813	0,112334075	0,181729916
HMGN2P4	processed_pseudogene	ENSG00000249014	-0,293037176	0,112374683	0,181781271
AC131212.3	TEC	ENSG00000280287	-0,430102592	0,112796077	0,182448545
AC005339.1	antisense	ENSG00000268565	0,498542156	0,112813967	0,182463091
ATMIN	protein_coding	ENSG00000166454	0,075878559	0,112908937	0,182602294
APOC1	protein_coding	ENSG00000130208	0,400181951	0,112932405	0,182625848

VPS9D1-AS1	antisense	ENSG00000261373	-0,209106232	0,112960342	0,182656624
IL2RB	protein_coding	ENSG00000100385	0,497604545	0,112983532	0,18267972
SP100	protein_coding	ENSG00000067066	0,075982486	0,113261111	0,183114093
TIMM29	protein_coding	ENSG00000142444	0,1052672	0,113282989	0,183135028
U2SURP	protein_coding	ENSG00000163714	-0,09448432	0,113306456	0,183158529
AC018868.2	processed_pseudogene	ENSG00000259751	0,490060836	0,113357155	0,183211604
GGA1	protein_coding	ENSG00000100083	-0,125904225	0,11335095	0,183211604
ACTR2	protein_coding	ENSG00000138071	0,080462084	0,113376407	0,183228281
LAYN	protein_coding	ENSG00000204381	-0,096596326	0,113576547	0,183537266
VDAC2	protein_coding	ENSG00000165637	0,067695092	0,113606828	0,183571735
ARMT1	protein_coding	ENSG00000146476	0,111189393	0,113661142	0,183645032
RN7SL650P	misc_RNA	ENSG00000274475	0,484904105	0,113773444	0,183811999
AC093810.1	lincRNA	ENSG00000249052	0,464327508	0,113873985	0,183923829
IL1B	protein_coding	ENSG00000125538	0,142054154	0,113876975	0,183923829
TOM1L2	protein_coding	ENSG00000175662	0,127768737	0,113868688	0,183923829
ATP6V1C1	protein_coding	ENSG00000155097	0,07061328	0,113878533	0,183923829
AC092171.4	lincRNA	ENSG00000272953	-0,497568011	0,114164229	0,184370734
ARHGEF33	protein_coding	ENSG00000214694	0,44133176	0,114251559	0,184497243
FUS	protein_coding	ENSG00000089280	0,112290048	0,114508536	0,184883104
ANKRD37	protein_coding	ENSG00000186352	-0,195191055	0,11450626	0,184883104
HAR1A	lincRNA	ENSG00000225978	-0,486833338	0,114596781	0,185011019
SNRPCP19	processed_pseudogene	ENSG00000234999	0,482918801	0,114651429	0,185079121
DCAF10	protein_coding	ENSG00000122741	0,090093634	0,114657012	0,185079121
ARMC12	protein_coding	ENSG00000157343	0,49440406	0,114704475	0,185141165
AL008729.1	antisense	ENSG00000215022	0,484201544	0,114735303	0,185176351
AC010210.1	processed_transcript	ENSG00000250608	0,437775054	0,114793309	0,185255392
IRF4	protein_coding	ENSG00000137265	0,490188784	0,114933225	0,185466596
LINC01293	lincRNA	ENSG00000230836	-0,092190283	0,115042224	0,185627882
AC006058.2	lincRNA	ENSG00000271192	-0,454066481	0,115380726	0,186159431
SCML1	protein_coding	ENSG00000047634	0,110743056	0,11548917	0,186319742
SLC36A1	protein_coding	ENSG00000123643	0,147156742	0,115581576	0,186454155
AC009502.1	lincRNA	ENSG00000227308	0,275416985	0,11562863	0,186515392
CASP7	protein_coding	ENSG00000165806	0,097239509	0,11564628	0,186529192
SPN	protein_coding	ENSG00000197471	0,495451941	0,11569082	0,186586359
TMPRSS5	protein_coding	ENSG00000166682	-0,439760717	0,115717459	0,186614648
SNORA5A	snoRNA	ENSG00000206838	-0,41482215	0,115749275	0,18665128
USP20	protein_coding	ENSG00000136878	0,145907214	0,115763042	0,186658804
AC011476.3	antisense	ENSG00000267265	0,455137212	0,115795167	0,186695927
AC105036.3	processed_transcript	ENSG00000260269	-0,466230915	0,11581618	0,186715127
ZNF460-AS1	antisense	ENSG00000267871	0,454490118	0,115832738	0,186727144

MNX1-AS1	lincRNA	ENSG00000243479	-0,22276583	0,11592817	0,186866297
NOM1	protein_coding	ENSG00000146909	-0,105612667	0,115957829	0,186899416
DDX11-AS1	antisense	ENSG00000245614	-0,309664286	0,11596781	0,186900816
AP000759.1	antisense	ENSG00000255320	0,470223547	0,115981305	0,186907879
C10orf90	protein_coding	ENSG00000154493	0,49403775	0,11602091	0,186957014
AL162411.1	lincRNA	ENSG00000236924	0,295209581	0,116280389	0,187360419
SAR1A	protein_coding	ENSG00000079332	0,073781438	0,11632357	0,187415272
TTC9	protein_coding	ENSG00000133985	-0,265583503	0,116340875	0,18742843
HEPACAM2	protein_coding	ENSG00000188175	0,44310505	0,11635831	0,187441795
LRIG3	protein_coding	ENSG00000139263	0,105741401	0,116399033	0,18749267
SLC25A29	protein_coding	ENSG00000197119	-0,17370704	0,116569208	0,187737296
LIPE	protein_coding	ENSG00000079435	-0,250905303	0,116563381	0,187737296
TYW1	protein_coding	ENSG00000198874	0,088349099	0,116620006	0,187798441
ZNHIT1	protein_coding	ENSG00000106400	-0,13142509	0,116625487	0,187798441
RNASEH2C	protein_coding	ENSG00000172922	-0,117315811	0,116636743	0,187801822
TSGA10IP	protein_coding	ENSG00000175513	0,486099113	0,116751248	0,187971434
AC145207.3	antisense	ENSG00000262831	0,452626574	0,116763873	0,187977004
RPLP0	protein_coding	ENSG00000089157	-0,116738693	0,116783093	0,18799319
RAB4B	protein_coding	ENSG00000167578	-0,284386877	0,116863796	0,18810834
OPCML	protein_coding	ENSG00000183715	0,477455273	0,116878522	0,18811728
AC008894.3	TEC	ENSG00000279198	0,449250877	0,116961659	0,188236317
TAS2R14	protein_coding	ENSG00000212127	0,453327122	0,116974781	0,188242665
TAS2R14	protein_coding	ENSG00000212127	0,453327122	0,116974781	0,188242665
LGALS9C	protein_coding	ENSG00000171916	0,30162457	0,117068566	0,188378808
FBXL7	protein_coding	ENSG00000183580	0,224697381	0,117102508	0,188418643
ATP5MPL	protein_coding	ENSG00000156411	-0,099625619	0,11714765	0,18847649
AC118344.2	TEC	ENSG00000279759	0,484829965	0,117236147	0,188604076
TFE3	protein_coding	ENSG00000068323	0,093899663	0,117333185	0,188745382
KLHL17	protein_coding	ENSG00000187961	-0,221568424	0,117362321	0,188777445
MYL6	protein_coding	ENSG00000092841	-0,10358478	0,117444577	0,18889494
SNORA9B	snoRNA	ENSG00000206897	-0,484866888	0,117484242	0,188943919
C8G	protein_coding	ENSG00000176919	0,405295314	0,117522936	0,188991329
HNRNPA1P10	processed_pseudogene	ENSG00000214223	-0,467071623	0,117552255	0,189023658
C19orf73	protein_coding	ENSG00000221916	-0,387757682	0,117582415	0,189057332
MELTF-AS1	antisense	ENSG00000228109	-0,328564441	0,117603019	0,189075638
AC116366.2	antisense	ENSG00000238160	0,387594165	0,117784868	0,189339898
ZXDA	protein_coding	ENSG00000198205	-0,140824821	0,117785849	0,189339898
HECTD2	protein_coding	ENSG00000165338	0,101079782	0,117859275	0,189443083
XRCC6P1	processed_pseudogene	ENSG00000237417	0,483042344	0,117906366	0,189489074
C19orf12	protein_coding	ENSG00000131943	-0,087193959	0,117902927	0,189489074

SLC10A6	protein_coding	ENSG00000145283	0,46919585	0,117929577	0,189511528
CDKN2C	protein_coding	ENSG00000123080	-0,078501272	0,117940593	0,189514382
RNF7	protein_coding	ENSG00000114125	-0,101291973	0,117987823	0,189575422
AC010326.4	transcribed_processed_	ENSG00000270804	0,361721999	0,118048944	0,189658769
MRPL40	protein_coding	ENSG00000185608	-0,105784103	0,11813053	0,189774979
AC012313.9	antisense	ENSG00000273901	-0,472143993	0,118455392	0,190281961
ERAP2	protein_coding	ENSG00000164308	-0,069616594	0,11852762	0,190383074
PSMB10	protein_coding	ENSG00000205220	0,32361992	0,118682832	0,190617454
NPM1P21	processed_pseudogene	ENSG00000248578	0,488711619	0,118763368	0,190716933
PDZD4	protein_coding	ENSG00000067840	-0,31826852	0,118762299	0,190716933
AL133163.3	lincRNA	ENSG00000259017	0,440752902	0,118876696	0,190883977
TRAPPC11	protein_coding	ENSG00000168538	-0,094668236	0,118966722	0,191013579
KCNAB3	protein_coding	ENSG00000170049	-0,350132278	0,119040829	0,191117605
ABHD17A	protein_coding	ENSG00000129968	-0,117829516	0,119168835	0,191308141
AL139274.2	antisense	ENSG00000272008	0,454266407	0,119244868	0,191415217
PIGV	protein_coding	ENSG00000060642	-0,084925157	0,119511923	0,191828889
CABLES2	protein_coding	ENSG00000149679	-0,170642683	0,119640592	0,192020389
FAM27B	processed_pseudogene	ENSG00000278763	-0,485766539	0,119680328	0,19205638
ETV5	protein_coding	ENSG00000244405	0,060415964	0,119681745	0,19205638
APRT	protein_coding	ENSG00000198931	-0,138781105	0,11969355	0,192060296
AC102945.2	TEC	ENSG00000279041	0,48343527	0,119797356	0,192211826
AL133351.1	antisense	ENSG00000224846	0,445033253	0,119813298	0,192222368
SENP3-EIF4A1	protein_coding	ENSG00000277957	0,47288247	0,119849395	0,19225782
TRNT1	protein_coding	ENSG00000072756	-0,094554133	0,119854144	0,19225782
ZNF75A	protein_coding	ENSG00000162086	0,109832992	0,119934109	0,192371047
NOTCH4	protein_coding	ENSG00000204301	0,396634597	0,120011195	0,192479637
PAXBP1-AS1	antisense	ENSG00000238197	0,253350033	0,120076316	0,192569024
AL391244.2	lincRNA	ENSG00000272455	0,377120113	0,120111447	0,192610304
RUBCN	protein_coding	ENSG00000145016	-0,112641712	0,120146149	0,192650889
TPT1P4	processed_pseudogene	ENSG00000217027	-0,473277426	0,120201392	0,192724401
MROH6	protein_coding	ENSG00000204839	-0,267208002	0,12026148	0,192805671
EZR	protein_coding	ENSG00000092820	-0,053250945	0,120312956	0,192873122
CIART	protein_coding	ENSG00000159208	0,421271088	0,120386214	0,192975479
ADM5	protein_coding	ENSG00000224420	-0,2757128	0,120413839	0,193004677
C11orf1	protein_coding	ENSG00000137720	0,116963198	0,120449684	0,193047043
AC233723.1	protein_coding	ENSG00000262165	0,456185284	0,120473725	0,193070488
GIGYF2	protein_coding	ENSG00000204120	0,106092937	0,120508721	0,193111483
PAQR6	protein_coding	ENSG00000160781	0,322431983	0,120542442	0,193150429
AC017100.1	antisense	ENSG00000259985	0,251367983	0,12055753	0,193159513
MYOM2	protein_coding	ENSG00000036448	0,481904176	0,120605566	0,193206292

EBF4	protein_coding	ENSG00000088881	0,446188031	0,120599118	0,193206292
ZFAND6	protein_coding	ENSG00000086666	0,097251099	0,12062818	0,193227427
TNFSF10	protein_coding	ENSG00000121858	0,466399072	0,12065159	0,193249834
RPL5P17	processed_pseudogene	ENSG00000243859	-0,48297819	0,120668212	0,193261364
MARS	protein_coding	ENSG00000166986	-0,06599147	0,120906337	0,193627623
AL109976.1	lincRNA	ENSG00000277287	0,477609776	0,120995386	0,193755104
AC012640.4	antisense	ENSG00000271980	0,486292948	0,121049599	0,193826784
ARFGEF3	protein_coding	ENSG00000112379	-0,133436552	0,121201224	0,194054418
GTDC1	protein_coding	ENSG00000121964	0,092347211	0,12121858	0,194067058
AL117339.4	lincRNA	ENSG00000272983	0,411238389	0,121248521	0,194099842
AC008937.3	antisense	ENSG00000271828	0,455606339	0,121449599	0,194406562
PAN3-AS1	antisense	ENSG00000261485	0,44719166	0,121490533	0,194456909
ING4	protein_coding	ENSG00000111653	-0,13591998	0,121695177	0,194769261
DNAAF1	protein_coding	ENSG00000154099	-0,452545391	0,121715834	0,194787122
TRIM66	protein_coding	ENSG00000166436	0,146325757	0,122009272	0,195226257
RIOK2	protein_coding	ENSG00000058729	0,103862093	0,122003305	0,195226257
RFX5	protein_coding	ENSG00000143390	-0,06709152	0,122018891	0,195226418
HTR7	protein_coding	ENSG00000148680	0,119437944	0,122090872	0,195326349
PSMA3-AS1	antisense	ENSG00000257621	-0,099198947	0,122234679	0,195538134
VAT1	protein_coding	ENSG00000108828	-0,088093576	0,122242319	0,195538134
ARHGEF26-AS1	processed_transcript	ENSG00000243069	0,461102846	0,122268938	0,195565462
AC093788.1	lincRNA	ENSG00000273449	-0,463762994	0,122280536	0,19556876
AC008691.1	antisense	ENSG00000249738	0,342519502	0,122465904	0,195849956
AC231533.1	antisense	ENSG00000232828	0,454583955	0,122493804	0,195879301
PGM1	protein_coding	ENSG00000079739	-0,070699221	0,122539792	0,195937564
MAST4-AS1	antisense	ENSG00000229666	0,339289865	0,122578827	0,1959847
AC010974.1	antisense	ENSG00000230065	0,421652735	0,122598694	0,196001186
MICE	transcribed_unprocesse	ENSG00000273340	0,479739985	0,12265381	0,196074017
UXT	protein_coding	ENSG00000126756	-0,103876451	0,122723239	0,196169716
KIAA0895L	protein_coding	ENSG00000196123	-0,164442785	0,122772359	0,196232939
CES3	protein_coding	ENSG00000172828	0,206527397	0,122881627	0,196376981
AC122688.3	TEC	ENSG00000279233	0,445750338	0,122878455	0,196376981
SLC27A3	protein_coding	ENSG00000143554	-0,291952139	0,122894414	0,196382114
ZFPL1	protein_coding	ENSG00000162300	0,105827887	0,122968141	0,196484619
KBTBD3	protein_coding	ENSG00000182359	-0,224598409	0,123017662	0,196548434
FMNL1	protein_coding	ENSG00000184922	0,148978734	0,123152161	0,196747999
KLF15	protein_coding	ENSG00000163884	0,469681094	0,123175679	0,196770245
ALAD	protein_coding	ENSG00000148218	-0,10310266	0,123209462	0,196808883
AP1G2	protein_coding	ENSG00000213983	0,143687748	0,123230269	0,196826791
UMAD1	protein_coding	ENSG00000219545	0,138475165	0,12330753	0,196919523

ZNF890P	transcribed_unprocesse	ENSG00000159904	-0,474630792	0,123306806	0,196919523
ZNF649	protein_coding	ENSG00000198093	-0,270047769	0,123429097	0,197098316
PKN2	protein_coding	ENSG00000065243	-0,087328782	0,123453167	0,197121406
BMS1P4	transcribed_unprocesse	ENSG00000242338	0,402091969	0,123508843	0,197194953
SMAP1	protein_coding	ENSG00000112305	0,085719901	0,123606101	0,197334874
AP003084.1	processed_pseudogene	ENSG00000254783	0,368531078	0,123629628	0,197357072
HK3	protein_coding	ENSG00000160883	0,410371693	0,123695222	0,197446416
NDOR1	protein_coding	ENSG00000188566	0,155406512	0,123742936	0,197507207
SNORD92	snoRNA	ENSG00000264994	-0,457965745	0,123769659	0,197534489
GNRHR2	transcribed_unitary_pse	ENSG00000211451	-0,406404765	0,123842875	0,197635961
NOVA1	protein_coding	ENSG00000139910	0,33031178	0,123909571	0,197727013
ANKRD12	protein_coding	ENSG00000101745	0,144595298	0,124123797	0,198053452
FAM86C1	protein_coding	ENSG00000158483	-0,127554261	0,124295924	0,198312672
SLIRP	protein_coding	ENSG00000119705	-0,111188874	0,124509702	0,198638301
AC007969.1	processed_pseudogene	ENSG00000233762	-0,257666683	0,124533824	0,198645881
ARPC5L	protein_coding	ENSG00000136950	0,083102984	0,124527922	0,198645881
NDUFS7	protein_coding	ENSG00000115286	-0,118739991	0,12457579	0,198697367
RPL39P5	transcribed_processed_	ENSG00000214289	0,448878138	0,124605848	0,198729856
SGMS1	protein_coding	ENSG00000198964	-0,078997956	0,124621182	0,198738858
RAB11FIP5	protein_coding	ENSG00000135631	0,109160234	0,124706389	0,198859278
AP003068.4	TEC	ENSG00000278952	0,363681635	0,124728939	0,198879775
LRRC37BP1	transcribed_unprocesse	ENSG00000250462	-0,135065236	0,124778144	0,198942766
CBSL	protein_coding	ENSG00000274276	0,20499538	0,124800159	0,198962398
CTAGE1	protein_coding	ENSG00000212710	0,45692844	0,124812795	0,198967078
AC087752.4	antisense	ENSG00000272509	-0,428902098	0,124913515	0,199079723
AL360219.1	lincRNA	ENSG00000273264	0,46756607	0,124905218	0,199079723
SCRN3	protein_coding	ENSG00000144306	-0,090123801	0,124911362	0,199079723
MLLT6	protein_coding	ENSG00000275023	-0,124822008	0,124922284	0,199079723
SKP1	protein_coding	ENSG00000113558	0,088091162	0,124957074	0,199104226
ARL6	protein_coding	ENSG00000113966	-0,153066165	0,124951422	0,199104226
MAMDC2-AS1	antisense	ENSG00000204706	0,471717244	0,124979153	0,199108469
COPB1	protein_coding	ENSG00000129083	0,076156426	0,124974377	0,199108469
MED4	protein_coding	ENSG00000136146	-0,076405743	0,125045186	0,199198195
AL117329.1	lincRNA	ENSG00000224271	0,4695485	0,125226042	0,199470808
TMPRSS11E	protein_coding	ENSG00000087128	0,474520758	0,125244525	0,199484756
KCTD10	protein_coding	ENSG00000110906	0,05978797	0,125325929	0,199583415
MRPS10	protein_coding	ENSG00000048544	-0,096538948	0,125323523	0,199583415
TAL2	protein_coding	ENSG00000186051	0,434330072	0,125339078	0,199588857
AC090559.1	antisense	ENSG00000255197	0,457752046	0,125381353	0,199625179
RPL7	protein_coding	ENSG00000147604	-0,082297151	0,125372964	0,199625179

RABGAP1L	protein_coding	ENSG00000152061	-0,087780013	0,1253985	0,199636982
AC035140.1	antisense	ENSG00000249085	0,42173198	0,125410341	0,199640336
TTYH3	protein_coding	ENSG00000136295	0,152178549	0,125430206	0,199656463
GTF3C6	protein_coding	ENSG00000155115	0,086971091	0,125476476	0,199714614
RPS8	protein_coding	ENSG00000142937	-0,110840912	0,125559499	0,19983125
MYOCOS	protein_coding	ENSG00000283683	0,405564187	0,12559391	0,199870506
AC025178.1	antisense	ENSG00000250764	0,440909778	0,125680336	0,199992528
CHMP5	protein_coding	ENSG00000086065	0,107743561	0,125755162	0,200096073
SMYD3-IT1	sense_intronic	ENSG00000230184	-0,467648119	0,125852794	0,200235886
ZFPM2	protein_coding	ENSG00000169946	0,475709878	0,125907193	0,200291361
UQCRH	protein_coding	ENSG00000173660	-0,087223349	0,125903746	0,200291361
NXNL2	protein_coding	ENSG00000130045	0,386211364	0,125928984	0,20031049
NHLRC4	protein_coding	ENSG00000257108	0,451833488	0,126240844	0,200790982
AC104825.1	lincRNA	ENSG00000251615	0,443226164	0,126265014	0,200813852
PCCB	protein_coding	ENSG00000114054	-0,081660347	0,126300342	0,200854464
AL360181.2	antisense	ENSG00000235245	0,38394647	0,12647235	0,201112413
HLX-AS1	antisense	ENSG00000257551	0,471939536	0,126562257	0,201224178
AP2B1	protein_coding	ENSG00000006125	0,056493804	0,126552524	0,201224178
CLEC16A	protein_coding	ENSG00000038532	0,106695075	0,12659079	0,201238342
RDM1	protein_coding	ENSG00000278023	-0,218293583	0,126583232	0,201238342
AC022400.5	processed_transcript	ENSG00000271816	0,455913298	0,12666382	0,201338831
OSTC	protein_coding	ENSG00000198856	0,106942056	0,126760973	0,201477647
AP003419.3	lincRNA	ENSG00000275484	-0,45346121	0,126777118	0,201487694
AL589765.4	antisense	ENSG00000249602	0,458648816	0,126797687	0,201504769
SMAD4	protein_coding	ENSG00000141646	-0,070524099	0,126940202	0,201715623
ZNF554	protein_coding	ENSG00000172006	0,203001667	0,127027903	0,201839348
WDR44	protein_coding	ENSG00000131725	0,078492568	0,127058063	0,20187163
ZNF687	protein_coding	ENSG00000143373	0,124597736	0,127127489	0,201960986
RNASET2	protein_coding	ENSG00000026297	-0,193986452	0,127133998	0,201960986
SINHCAFP2	processed_pseudogene	ENSG00000267742	-0,457725884	0,127215494	0,202074797
HRC	protein_coding	ENSG00000130528	-0,465001285	0,127277483	0,202157607
AC243829.2	antisense	ENSG00000276241	0,372121557	0,127287885	0,202158473
TCTN1	protein_coding	ENSG00000204852	-0,113057575	0,12734055	0,202226457
RANBP10	protein_coding	ENSG00000141084	0,150340886	0,127374216	0,2022486
TRABD2B	protein_coding	ENSG00000269113	-0,401429964	0,127369108	0,2022486
ZNF799	protein_coding	ENSG00000196466	-0,175712939	0,127534207	0,202486963
ZMYND10	protein_coding	ENSG00000004838	-0,342094861	0,127566574	0,202522675
AKR7A3	protein_coding	ENSG00000162482	-0,470372942	0,127588989	0,202542582
SRGAP2	protein_coding	ENSG00000266028	-0,10369859	0,127753744	0,202788428
PPP1R13B	protein_coding	ENSG00000088808	0,146282481	0,127978104	0,203128843

ST7-AS1	antisense	ENSG00000227199	0,381029007	0,128012551	0,203167796
GRIP2	protein_coding	ENSG00000144596	0,417440496	0,128161819	0,20338896
AC117395.1	lincRNA	ENSG00000242791	0,381584113	0,128191996	0,20342111
TCEAL9	protein_coding	ENSG00000185222	-0,105323616	0,128226234	0,203459697
RPS28	protein_coding	ENSG00000233927	-0,082055711	0,128264327	0,203499644
GSTO1	protein_coding	ENSG00000148834	-0,126858753	0,128271253	0,203499644
RN7SL555P	misc_RNA	ENSG00000263905	-0,449597355	0,128297962	0,203526274
DGCR6L	protein_coding	ENSG00000128185	-0,123418878	0,128313052	0,203534469
RPS6P25	processed_pseudogene	ENSG00000240616	0,465037898	0,128369475	0,203593982
PGBD5	protein_coding	ENSG00000177614	0,388958611	0,128380244	0,203593982
HLA-J	transcribed_unprocesse	ENSG00000204622	0,466102133	0,128380349	0,203593982
SETP2	processed_pseudogene	ENSG00000258893	0,412948067	0,128612842	0,203946914
AC099667.1	TEC	ENSG00000279144	-0,466811644	0,128730111	0,204117091
PGAM1P8	transcribed_processed_	ENSG00000255200	0,38904273	0,128757452	0,204144662
AC010536.2	sense_intronic	ENSG00000260671	0,407772212	0,128816406	0,204222346
RPL4P5	processed_pseudogene	ENSG00000230207	-0,318599123	0,1288503	0,204257333
ZNF862	protein_coding	ENSG00000106479	0,304912288	0,128858393	0,204257333
SLC2A1-AS1	lincRNA	ENSG00000227533	0,360381782	0,128893601	0,204297353
SMARCAL1	protein_coding	ENSG00000138375	0,078313342	0,129089169	0,20459152
NSFL1C	protein_coding	ENSG00000088833	0,083824946	0,129217631	0,204779293
RASSF8	protein_coding	ENSG00000123094	0,108367539	0,129238906	0,204781362
PISD	protein_coding	ENSG00000241878	-0,080950621	0,129229409	0,204781362
POT1-AS1	processed_transcript	ENSG00000224897	0,353430347	0,129293165	0,204851511
CHGB	protein_coding	ENSG00000089199	-0,360474197	0,129422314	0,205040295
VPS33B	protein_coding	ENSG00000184056	-0,102962897	0,129703978	0,205454062
NCDN	protein_coding	ENSG00000020129	-0,124927196	0,129708299	0,205454062
AC009948.4	TEC	ENSG00000279598	0,45286942	0,129713538	0,205454062
GAMT	protein_coding	ENSG00000130005	-0,143835448	0,129730233	0,20546464
CA5B	protein_coding	ENSG00000169239	0,130796852	0,129763253	0,205501067
AL035530.2	antisense	ENSG00000271913	0,463761093	0,129857373	0,205633757
ZCCHC14	protein_coding	ENSG00000140948	-0,132049275	0,129867092	0,205633757
AC009509.4	antisense	ENSG00000276261	0,450215386	0,129888653	0,20565202
CNKSR1	protein_coding	ENSG00000142675	0,457430909	0,129912916	0,205674558
EDC4	protein_coding	ENSG00000038358	-0,108247098	0,1299911	0,205782452
GNB5	protein_coding	ENSG00000069966	-0,065510708	0,130066965	0,20588666
DTD2	protein_coding	ENSG00000129480	-0,131495794	0,130080522	0,205892229
GLB1	protein_coding	ENSG00000170266	-0,081129512	0,130127625	0,205950891
AL160408.2	antisense	ENSG00000228830	0,4657239	0,130165229	0,205973598
FAM184A	protein_coding	ENSG00000111879	0,210695371	0,1301721	0,205973598
DDB1	protein_coding	ENSG00000167986	-0,056330354	0,130171333	0,205973598

AC004241.3	sense_intronic	ENSG00000276390	-0,43479084	0,130249034	0,206079432
AGAP1-IT1	sense_intronic	ENSG00000235529	0,460603173	0,130380488	0,206271507
APLN	protein_coding	ENSG00000171388	-0,183781139	0,130408485	0,206299886
PMS2P3	transcribed_unprocesse	ENSG00000127957	0,189860278	0,130499003	0,20642716
AK5	protein_coding	ENSG00000154027	-0,234120445	0,130536533	0,206470601
ZNF114-AS1	lincRNA	ENSG00000268186	0,289698645	0,130602125	0,206558419
MRPL37	protein_coding	ENSG00000116221	-0,074028606	0,130647757	0,206614658
AC145422.1	lincRNA	ENSG00000256742	0,447215721	0,130758832	0,206774375
AL133216.2	transcribed_unprocesse	ENSG00000276805	0,350700796	0,130935291	0,207037455
CNBD2	protein_coding	ENSG00000149646	-0,29895382	0,131042855	0,207191563
CACNA2D3	protein_coding	ENSG00000157445	0,340688673	0,131128414	0,207306734
C8orf59	protein_coding	ENSG00000176731	-0,126190855	0,131135913	0,207306734
AP003108.2	protein_coding	ENSG00000256591	-0,434586809	0,131151512	0,207315415
TCEA1P2	processed_pseudogene	ENSG00000230409	0,27985753	0,131226617	0,207405552
RCOR3	protein_coding	ENSG00000117625	0,113722224	0,131228759	0,207405552
AC092821.1	transcribed_unprocesse	ENSG00000214776	0,281686429	0,131275714	0,207463776
LMNTD2	protein_coding	ENSG00000185522	0,26506838	0,131342816	0,20755383
REX1BD	protein_coding	ENSG00000006015	-0,142364867	0,131439577	0,207690733
AC005498.3	antisense	ENSG00000269696	0,462288219	0,131715466	0,208097562
NPR3	protein_coding	ENSG00000113389	0,38861253	0,131717335	0,208097562
AL355987.4	processed_transcript	ENSG00000273066	-0,331396459	0,131768907	0,208163004
AC012557.1	lincRNA	ENSG00000271843	-0,430044292	0,131779172	0,208163186
SETD5	protein_coding	ENSG00000168137	0,086099728	0,131804175	0,208186649
PIGQ	protein_coding	ENSG00000007541	-0,155663209	0,131846931	0,208238146
SLC25A5	protein_coding	ENSG00000005022	-0,091143131	0,13197603	0,208425993
ZNF382	protein_coding	ENSG00000161298	0,089339724	0,131990131	0,208432213
AC027575.2	lincRNA	ENSG00000278107	0,346773189	0,132063731	0,20851633
PPIG	protein_coding	ENSG00000138398	-0,10240341	0,132057297	0,20851633
AC098818.2	antisense	ENSG00000260278	0,45819655	0,132131274	0,208606915
ACADSB	protein_coding	ENSG00000196177	-0,101777893	0,132156111	0,208630067
KRT18P32	processed_pseudogene	ENSG00000215149	0,431779096	0,132168211	0,208633111
TXNL1	protein_coding	ENSG00000091164	0,108020873	0,132212675	0,208687237
EPCAM	protein_coding	ENSG00000119888	0,395631526	0,132239666	0,208713778
SFT2D1	protein_coding	ENSG00000198818	0,088127705	0,132273228	0,208750685
CPLANE1	protein_coding	ENSG00000197603	0,099454784	0,132323579	0,208811444
UPF2	protein_coding	ENSG00000151461	-0,107431595	0,132332089	0,208811444
AC009065.4	lincRNA	ENSG00000260778	0,427584664	0,132394116	0,208877178
TXNL4B	protein_coding	ENSG00000140830	0,088451598	0,132385719	0,208877178
AC016739.1	processed_pseudogene	ENSG00000218175	0,37326191	0,132451277	0,208951288
AC007684.1	sense_intronic	ENSG00000273035	-0,457511371	0,132516264	0,209037731

IGHMBP2	protein_coding	ENSG00000132740	-0,117247583	0,132695829	0,209304888
PIK3CG	protein_coding	ENSG00000105851	0,176237626	0,132777008	0,209416828
PATL1	protein_coding	ENSG00000166889	0,100187286	0,132964075	0,209695747
CSAD	protein_coding	ENSG00000139631	0,23182708	0,133036222	0,209778078
SNRNP200	protein_coding	ENSG00000144028	-0,099195532	0,133036736	0,209778078
AC008543.1	lincRNA	ENSG00000197332	0,439309539	0,133160476	0,209957054
AL049794.1	lincRNA	ENSG00000273998	0,456654147	0,13335308	0,210228415
LRIT3	protein_coding	ENSG00000183423	0,434341147	0,133351292	0,210228415
RF00019	misc_RNA	ENSG00000207009	-0,411172779	0,133512351	0,210448819
C19orf57	protein_coding	ENSG00000132016	-0,350940599	0,13351341	0,210448819
HSPA1L	protein_coding	ENSG00000204390	-0,158273467	0,133585393	0,210546101
AC106782.2	lincRNA	ENSG00000260219	0,389935862	0,133604249	0,210559639
HYI	protein_coding	ENSG00000178922	0,109582381	0,133705246	0,21070262
WNT5A	protein_coding	ENSG00000114251	-0,098420772	0,133764594	0,210779949
TBC1D1	protein_coding	ENSG00000065882	-0,06966482	0,133837256	0,210878246
ENGASE	protein_coding	ENSG00000167280	-0,160395559	0,134070014	0,211228759
AC012511.1	lincRNA	ENSG00000272564	0,366079067	0,134143899	0,211328932
DGAT1	protein_coding	ENSG00000185000	-0,120194079	0,134201734	0,211403806
ERI3-IT1	sense_intronic	ENSG00000233602	0,460652653	0,134236932	0,211443014
AC005702.2	processed_pseudogene	ENSG00000267526	-0,436197306	0,134310775	0,21154308
DLX4	protein_coding	ENSG00000108813	0,247649264	0,134343097	0,21157774
STIM2	protein_coding	ENSG00000109689	0,107652787	0,134362493	0,211592041
CACTIN	protein_coding	ENSG00000105298	0,104794315	0,13439298	0,211623802
CSPP1	protein_coding	ENSG00000104218	-0,089290601	0,134491791	0,211763138
MTHFD2P1	transcribed_processed_	ENSG00000244681	0,37744501	0,134553353	0,211843806
ACTG1P20	processed_pseudogene	ENSG00000241547	-0,432763139	0,134572175	0,211857177
AC027290.2	TEC	ENSG00000280138	0,246303592	0,134648062	0,211960377
CRADD	protein_coding	ENSG00000169372	-0,131745109	0,134719483	0,212056531
TMEM9B	protein_coding	ENSG00000175348	-0,101901269	0,134738603	0,212070351
ZCWPW1	protein_coding	ENSG00000078487	0,281569039	0,134830222	0,212171942
AC064799.2	sense_intronic	ENSG00000260586	0,440944933	0,134816992	0,212171942
AC006453.2	transcribed_unprocesse	ENSG00000283196	0,214166736	0,134868448	0,21220958
AL136981.2	unprocessed_pseudoge	ENSG00000275318	0,322662915	0,13488692	0,212222364
FUOM	protein_coding	ENSG00000148803	-0,131591724	0,134962568	0,212325096
AC108673.3	lincRNA	ENSG00000273437	-0,458371867	0,135036282	0,212424769
NUP210L	protein_coding	ENSG00000143552	0,456688784	0,13510556	0,21251745
PKMP1	processed_pseudogene	ENSG00000236480	0,394873448	0,135129493	0,212522498
SUCLG1	protein_coding	ENSG00000163541	-0,109730316	0,135127357	0,212522498
AC116049.1	processed_pseudogene	ENSG00000236562	0,45643175	0,13515997	0,212537835
TMX1	protein_coding	ENSG00000139921	-0,102431559	0,13515441	0,212537835

ATP1A2	protein_coding	ENSG00000018625	0,429181963	0,135176729	0,212547893
UBE2J2	protein_coding	ENSG00000160087	0,097155748	0,1353432	0,212793333
TPR	protein_coding	ENSG00000047410	-0,104468943	0,135388487	0,21284822
AC005091.1	antisense	ENSG00000229893	0,393779853	0,135402153	0,212853389
JPX	lincRNA	ENSG00000225470	0,086356736	0,135429584	0,212880194
AL031846.1	transcribed_unprocesse	ENSG00000225720	0,430312255	0,135552171	0,213056558
FBXL15	protein_coding	ENSG00000107872	-0,141176419	0,135657819	0,213206274
AC244090.1	lincRNA	ENSG00000197180	0,26053058	0,135860191	0,21350797
TMEM222	protein_coding	ENSG00000186501	-0,095545556	0,135969202	0,213662913
LAMA1	protein_coding	ENSG00000101680	0,108999778	0,135983312	0,213668714
DPH6-DT	lincRNA	ENSG00000248079	0,446392122	0,136041081	0,213698296
AL138785.1	processed_pseudogene	ENSG00000225616	-0,411221996	0,136036415	0,213698296
ZSWIM8	protein_coding	ENSG00000214655	0,155890583	0,136045531	0,213698296
CDV3	protein_coding	ENSG00000091527	0,074430447	0,136054234	0,213698296
CBY1	protein_coding	ENSG00000100211	-0,130105688	0,136023996	0,213698296
TESK2	protein_coding	ENSG00000070759	0,223191059	0,136089799	0,213737789
AC021188.1	antisense	ENSG00000230747	-0,374138282	0,136201834	0,213897368
IMPDH1P8	processed_pseudogene	ENSG00000224728	-0,425489889	0,136276486	0,213998219
EVPL	protein_coding	ENSG00000167880	-0,205941488	0,136369777	0,214128321
UFL1	protein_coding	ENSG00000014123	0,10108373	0,136504086	0,214322805
FAM114A2	protein_coding	ENSG00000055147	-0,096977116	0,136582842	0,214430044
ZSCAN5A	protein_coding	ENSG00000131848	0,144907017	0,13666788	0,214547127
C22orf23	protein_coding	ENSG00000128346	0,425550508	0,136724299	0,214619269
STEAP3-AS1	antisense	ENSG00000229867	0,385144675	0,13676656	0,214669178
AC116366.3	protein_coding	ENSG00000283782	0,388641027	0,136945341	0,214900454
ZNF230	protein_coding	ENSG00000159882	-0,139582591	0,136938586	0,214900454
FAM160A2	protein_coding	ENSG00000051009	-0,151128979	0,137001664	0,214972392
THBS2	protein_coding	ENSG00000186340	0,447094288	0,137014759	0,214976493
ZFAND4	protein_coding	ENSG00000172671	-0,119991675	0,137037587	0,214995863
KRT20	protein_coding	ENSG00000171431	0,368481344	0,13711055	0,21509388
AC090791.1	lincRNA	ENSG00000254526	-0,37844858	0,137141738	0,215126352
COX6B2	protein_coding	ENSG00000160471	-0,449213291	0,13718461	0,215177145
DHRSX	protein_coding	ENSG00000169084	0,098208893	0,137205575	0,215193572
RSBN1	protein_coding	ENSG00000081019	-0,087764682	0,13724897	0,215245174
RF00272	snoRNA	ENSG00000252473	0,446234443	0,137360649	0,215403847
NSUN5	protein_coding	ENSG00000130305	-0,135200195	0,137472316	0,215561639
SCARNA6	scaRNA	ENSG00000251791	-0,269962525	0,137482292	0,215561639
RAB32	protein_coding	ENSG00000118508	0,097027319	0,137586167	0,21569153
ERCC5	protein_coding	ENSG00000134899	-0,236153206	0,137583368	0,21569153
ERCC5	protein_coding	ENSG00000134899	-0,236153206	0,137583368	0,21569153

TM9SF1	protein_coding	ENSG00000100926	-0,225563904	0,137618051	0,215725024
NDST1-AS1	lincRNA	ENSG00000254333	0,43033815	0,137683608	0,215811295
HDHD2	protein_coding	ENSG00000167220	-0,167086157	0,137695882	0,21581404
BNIP2	protein_coding	ENSG00000140299	-0,091485371	0,137727431	0,215846994
CFAP100	protein_coding	ENSG00000163885	-0,368730663	0,137833768	0,21599714
SLC25A5-AS1	processed_transcript	ENSG00000224281	0,278951361	0,137865197	0,216023476
MYH9	protein_coding	ENSG00000100345	0,146203942	0,137871639	0,216023476
C1orf74	protein_coding	ENSG00000162757	-0,113293803	0,137898079	0,2160484
PTPRG-AS1	processed_transcript	ENSG00000241472	-0,153556356	0,138000954	0,216186402
RRN3P3	transcribed_unprocesse	ENSG00000257122	0,222604682	0,138007243	0,216186402
LINC01106	lincRNA	ENSG00000175772	-0,381079884	0,138031425	0,216207769
CXCL1	protein_coding	ENSG00000163739	0,082308553	0,138042114	0,216208
TMEM130	protein_coding	ENSG00000166448	0,376359357	0,13814585	0,216353954
TMEM129	protein_coding	ENSG00000168936	0,13177668	0,138185455	0,216399457
AL390066.1	lincRNA	ENSG00000224950	0,433189646	0,138285426	0,216539478
TSPAN12	protein_coding	ENSG00000106025	0,212833226	0,138329694	0,216575727
ALCAM	protein_coding	ENSG00000170017	-0,084009436	0,138320124	0,216575727
HACD1	protein_coding	ENSG00000165996	-0,098235695	0,138363303	0,216611812
AC023946.1	antisense	ENSG00000255351	-0,454385725	0,138424608	0,216691247
CTNS	protein_coding	ENSG00000040531	0,099618309	0,138456407	0,216724483
FOXC2-AS1	antisense	ENSG00000260944	-0,310574767	0,138497085	0,216771613
PAICSP4	processed_pseudogene	ENSG00000254244	-0,452251701	0,138744431	0,217142181
FNIP1	protein_coding	ENSG00000217128	0,097121281	0,138764615	0,2171572
TRIM34	protein_coding	ENSG00000258659	-0,452294969	0,138806733	0,217206538
NUDT16P1	transcribed_unitary_pse	ENSG00000246082	-0,166761734	0,138879297	0,217303507
FAM222B	protein_coding	ENSG00000173065	-0,116899122	0,139093001	0,217621286
AL358472.1	processed_pseudogene	ENSG00000231416	-0,411592887	0,139112971	0,21763593
NRDE2	protein_coding	ENSG00000119720	-0,112805433	0,139179415	0,217723271
FAM98C	protein_coding	ENSG00000130244	-0,114170361	0,139230648	0,217786805
TRAF3	protein_coding	ENSG00000131323	0,095068811	0,139275901	0,217840977
ITPR3	protein_coding	ENSG00000096433	0,117486507	0,139289563	0,217845732
SLC18A3	protein_coding	ENSG00000187714	-0,16213436	0,139494234	0,218149198
FADS3	protein_coding	ENSG00000221968	-0,097463256	0,139519363	0,218171861
AC010245.2	lincRNA	ENSG00000272040	-0,417988188	0,139564672	0,218226075
AC141424.1	antisense	ENSG00000241525	-0,445002541	0,139636718	0,218322083
CEP83-DT	lincRNA	ENSG00000278916	-0,356688314	0,1396585	0,218339495
AC090825.1	lincRNA	ENSG00000259363	-0,249975081	0,139725279	0,218410599
MRAP2	protein_coding	ENSG00000135324	-0,382546697	0,139720646	0,218410599
MGST1	protein_coding	ENSG00000008394	-0,1030753	0,139764534	0,218455311
QSER1	protein_coding	ENSG00000060749	0,093860241	0,139860981	0,218589402

PAN2	protein_coding	ENSG00000135473	0,13476952	0,140062847	0,218888219
RGS17	protein_coding	ENSG00000091844	-0,166730433	0,1401768	0,219049613
COX5A	protein_coding	ENSG00000178741	-0,095184684	0,140275493	0,219187138
SCAF1	protein_coding	ENSG00000126461	-0,144427493	0,140349914	0,219286717
ZNF784	protein_coding	ENSG00000179922	-0,178783062	0,140370931	0,219302849
PSME1	protein_coding	ENSG00000092010	-0,09806819	0,140394496	0,219322958
KIAA1257	protein_coding	ENSG00000114656	0,402466341	0,140437336	0,219373173
AC009948.3	antisense	ENSG00000270956	-0,378734836	0,140601888	0,219613489
AJUBA	protein_coding	ENSG00000129474	-0,076250456	0,140677605	0,219715023
ZNF625	protein_coding	ENSG00000257591	0,448890516	0,140773622	0,219848244
AL133415.1	antisense	ENSG00000234961	0,43958511	0,140847006	0,219929356
TMCO4	protein_coding	ENSG00000162542	0,110274775	0,140838597	0,219929356
CXorf40B	protein_coding	ENSG00000197021	0,102292577	0,140966819	0,220099684
AL138781.2	lincRNA	ENSG00000275329	0,446635086	0,141114043	0,220312782
ANAPC4	protein_coding	ENSG00000053900	-0,098972068	0,141135258	0,220329132
MAPK11	protein_coding	ENSG00000185386	-0,11241943	0,141155261	0,220343588
AL391058.1	processed_pseudogene	ENSG00000232499	-0,360928394	0,141168677	0,22034776
MUCL3	protein_coding	ENSG00000168631	0,447202568	0,141227047	0,220422093
FAM21EP	transcribed_unprocesse	ENSG00000235618	0,440176031	0,141259712	0,2204563
SLC25A38	protein_coding	ENSG00000144659	0,100930318	0,141299389	0,220501444
MTUS2-AS1	antisense	ENSG00000179141	0,358184033	0,1413305	0,220533213
RUSC2	protein_coding	ENSG00000198853	0,123756872	0,141364185	0,220552216
AC022558.1	sense_intronic	ENSG00000259767	-0,42594023	0,141363104	0,220552216
PDSS2	protein_coding	ENSG00000164494	0,098238479	0,14138962	0,220575119
GRIN1	protein_coding	ENSG00000176884	0,416682401	0,14148359	0,220692845
BEST2	protein_coding	ENSG00000039987	0,387799811	0,141497363	0,220692845
AC006001.2	sense_intronic	ENSG00000226824	0,349716013	0,141494231	0,220692845
AC005014.2	lincRNA	ENSG00000272361	-0,432885346	0,141513194	0,220700753
CLOCK	protein_coding	ENSG00000134852	0,114290497	0,141545518	0,220734381
LINC01843	lincRNA	ENSG00000251169	-0,429804899	0,141587969	0,220783795
CLBA1	protein_coding	ENSG00000140104	-0,121143585	0,141646261	0,220857899
GGH	protein_coding	ENSG00000137563	-0,109491317	0,141738362	0,220984705
INSIG1	protein_coding	ENSG00000186480	-0,09499245	0,141775719	0,221026147
CZIB	protein_coding	ENSG00000162384	-0,09287978	0,14181567	0,221054825
CSNK2A3	protein_coding	ENSG00000254598	-0,307981656	0,141808579	0,221054825
LINC00271	lincRNA	ENSG00000231028	0,352021883	0,141871301	0,221124735
LINC02035	lincRNA	ENSG00000273033	0,175595999	0,141888966	0,221135462
AC034198.2	antisense	ENSG00000272263	-0,435711702	0,141964404	0,22123017
TMEM240	protein_coding	ENSG00000205090	-0,439252083	0,141971306	0,22123017
LINC02367	lincRNA	ENSG00000260423	0,343813623	0,14198536	0,22123526

RPL7AP60	processed_pseudogene	ENSG00000213152	0,366984501	0,142013026	0,221244752
RPS2	protein_coding	ENSG00000140988	-0,090161526	0,142002909	0,221244752
AL162231.2	antisense	ENSG00000230074	0,177554936	0,142071233	0,221318623
LINC01630	lincRNA	ENSG00000227115	0,377661931	0,142112291	0,221365769
NTNG2	protein_coding	ENSG00000196358	-0,120950314	0,142132603	0,221380596
AC025263.1	antisense	ENSG00000247131	0,412317045	0,142361499	0,221720278
AC093512.1	antisense	ENSG00000274904	-0,385915156	0,142427636	0,221806441
ZDHHC5	protein_coding	ENSG00000156599	-0,072190938	0,142450385	0,221825025
AC006480.2	lincRNA	ENSG00000273448	0,429420111	0,14267801	0,222162616
COX7A2P1	processed_pseudogene	ENSG00000258626	-0,424068303	0,142825913	0,222376032
MPC2	protein_coding	ENSG00000143158	0,111707842	0,142933722	0,222510105
DNAAF4	protein_coding	ENSG00000256061	-0,437135591	0,142929253	0,222510105
AL441992.1	antisense	ENSG00000223478	-0,158784002	0,142959102	0,222532725
DGKG	protein_coding	ENSG00000058866	0,250512903	0,143141811	0,222800224
SLC35A1	protein_coding	ENSG00000164414	-0,178003495	0,143248555	0,22294945
MTERF4	protein_coding	ENSG00000122085	-0,088039292	0,14328901	0,222995493
OR4F15	protein_coding	ENSG00000182854	0,406493897	0,143443313	0,223218691
SLC16A4	protein_coding	ENSG00000168679	0,436370553	0,143475168	0,223251323
AC012676.1	sense_intronic	ENSG00000262712	0,340330742	0,143558125	0,22336346
RPL11P3	processed_pseudogene	ENSG00000213613	0,437370491	0,143665738	0,223513942
MICALL1	protein_coding	ENSG00000100139	0,114857683	0,143740771	0,223613714
GUCY1A2	protein_coding	ENSG00000152402	0,263959528	0,143753864	0,223617121
STK36	protein_coding	ENSG00000163482	0,140797728	0,143947577	0,223901471
PXK	protein_coding	ENSG00000168297	-0,09042533	0,144065289	0,224067572
CNNM3-DT	lincRNA	ENSG00000273265	-0,445303145	0,144089942	0,224088923
UNC5A	protein_coding	ENSG00000113763	0,419314902	0,14412802	0,224131146
PPIAP11	processed_pseudogene	ENSG00000251495	0,379879537	0,144184177	0,224201476
HDAC4-AS1	antisense	ENSG00000222020	-0,33872946	0,144225074	0,224241512
INTS4P1	transcribed_unprocessed	ENSG00000164669	0,35463857	0,144231791	0,224241512
AL121672.2	lincRNA	ENSG00000235159	-0,346130112	0,144321006	0,22436321
LCT	protein_coding	ENSG00000115850	0,400459237	0,144386663	0,224448268
SERPIND1	protein_coding	ENSG00000099937	0,43424909	0,144424509	0,224490086
OPA1	protein_coding	ENSG00000198836	0,068492399	0,144446121	0,224506664
PHLDA3	protein_coding	ENSG00000174307	0,123882959	0,144538967	0,224633948
MFN2	protein_coding	ENSG00000116688	-0,065594391	0,144637864	0,224770616
TARID	antisense	ENSG00000227954	0,442321404	0,144701892	0,224836046
ZNF562	protein_coding	ENSG00000171466	-0,067006401	0,144696616	0,224836046
MYH3	protein_coding	ENSG00000109063	-0,224875194	0,14471793	0,224843931
SETSIP	protein_coding	ENSG00000230667	-0,441513252	0,144746017	0,224870535
PRDX1	protein_coding	ENSG00000117450	-0,07545536	0,144824548	0,224975497

SRGAP2B	protein_coding	ENSG00000196369	0,143251028	0,144842837	0,224986867
CD109	protein_coding	ENSG00000156535	-0,077483651	0,144883124	0,225032402
FAM209A	protein_coding	ENSG00000124103	0,419600183	0,145008106	0,22520947
SSH1	protein_coding	ENSG00000084112	0,082302461	0,145098003	0,225332024
CDK2AP1	protein_coding	ENSG00000111328	-0,078826387	0,145124752	0,225352042
ZNF787	protein_coding	ENSG00000142409	-0,094678567	0,145132868	0,225352042
SNORD91A	snoRNA	ENSG00000212163	-0,433330257	0,145279314	0,225562357
C11orf96	protein_coding	ENSG00000187479	0,437855554	0,145409715	0,22574773
AP002495.2	protein_coding	ENSG00000254469	-0,250755153	0,14560869	0,226039527
SUMO4	protein_coding	ENSG00000177688	0,442817483	0,145712432	0,226183455
RNF225	protein_coding	ENSG00000269855	-0,4022455	0,145736405	0,226203547
OPHN1	protein_coding	ENSG00000079482	-0,072828071	0,145752238	0,226211003
RWDD4	protein_coding	ENSG00000182552	-0,131203672	0,145912695	0,226442901
EDF1	protein_coding	ENSG00000107223	0,09528563	0,145953979	0,226489832
LLPH	protein_coding	ENSG00000139233	-0,086986546	0,145970831	0,226498846
DPF2	protein_coding	ENSG00000133884	0,083756181	0,146113332	0,22670281
AL139424.2	lincRNA	ENSG00000284642	0,435395969	0,146198929	0,22678415
AL390955.2	lincRNA	ENSG00000271040	0,410041451	0,146184821	0,22678415
MUL1	protein_coding	ENSG00000090432	-0,09846837	0,146196481	0,22678415
SETD3	protein_coding	ENSG00000183576	0,072395154	0,146341129	0,226987564
AF121898.1	antisense	ENSG00000253500	0,131975401	0,146385688	0,227039509
DCST2	protein_coding	ENSG00000163354	0,403207804	0,146529434	0,227244121
PNO1	protein_coding	ENSG00000115946	-0,104153519	0,146539773	0,227244121
AC068792.1	sense_intronic	ENSG00000275769	-0,416140215	0,14684196	0,227695517
AC131392.1	unprocessed_pseudoge	ENSG00000198237	-0,438993111	0,146874731	0,227729114
FAM104A	protein_coding	ENSG00000133193	-0,060311116	0,146948691	0,227826567
FAM209B	protein_coding	ENSG00000213714	0,43234047	0,146979831	0,227857621
RPL14P1	processed_pseudogene	ENSG00000139239	-0,186859402	0,147009743	0,227886769
TMEM115	protein_coding	ENSG00000126062	-0,091281121	0,147058668	0,227945382
AC090505.1	processed_pseudogene	ENSG00000234805	0,400889828	0,147076848	0,227956333
TMEM9B-AS1	antisense	ENSG00000254860	0,281998877	0,147176988	0,228094304
LLGL2	protein_coding	ENSG00000073350	0,402786577	0,147211331	0,22813029
BCDIN3D-AS1	antisense	ENSG00000258057	-0,267814929	0,147256568	0,228183153
TRAPPC2L	protein_coding	ENSG00000167515	-0,114536882	0,147531988	0,228592662
AC012184.3	processed_transcript	ENSG00000261777	0,283844255	0,147640507	0,228732815
GPATCH4	protein_coding	ENSG00000160818	-0,095558348	0,147644746	0,228732815
SNRNP27	protein_coding	ENSG00000124380	0,129372709	0,147948546	0,229186154
MT-CO3	protein_coding	ENSG00000198938	0,264673439	0,148005011	0,229256308
AC126474.1	TEC	ENSG00000279148	-0,308420262	0,148031231	0,229279606
SNORA80E	snoRNA	ENSG00000207475	-0,430234383	0,148075526	0,229330895

AC026356.1	sense_intronic	ENSG00000274964	-0,41499371	0,148160262	0,229444803
AK9	protein_coding	ENSG00000155085	0,246306112	0,148224794	0,229519993
CNTNAP3	protein_coding	ENSG00000106714	0,229180096	0,148231196	0,229519993
SESN3	protein_coding	ENSG00000149212	0,404127959	0,148271755	0,229548135
ABHD8	protein_coding	ENSG00000127220	0,120596615	0,148261976	0,229548135
PRR22	protein_coding	ENSG00000212123	0,304175902	0,148375286	0,22969108
MFSD14A	protein_coding	ENSG00000156875	-0,176896539	0,148490332	0,229851827
AC027796.2	processed_pseudogene	ENSG00000262248	-0,409822489	0,148580594	0,22997419
RALA	protein_coding	ENSG00000006451	0,068875269	0,148777054	0,230260895
UBA7	protein_coding	ENSG00000182179	0,417782714	0,148895031	0,230426099
C7orf25	protein_coding	ENSG00000136197	-0,352383868	0,14909668	0,230720756
TAX1BP3	protein_coding	ENSG00000213977	-0,127881281	0,149176967	0,230827582
AL133268.1	processed_pseudogene	ENSG00000219682	-0,420808213	0,149217382	0,230872699
SAMSN1	protein_coding	ENSG00000155307	0,419249659	0,149255283	0,230901849
SAMSN1	protein_coding	ENSG00000155307	0,419249659	0,149255283	0,230901849
SLC44A2	protein_coding	ENSG00000129353	-0,088917698	0,149258738	0,230901849
RF00019	misc_RNA	ENSG00000200742	-0,396006196	0,149304587	0,230955357
FAM185BP	transcribed_unprocesse	ENSG00000214439	0,420013969	0,149327479	0,230973347
ZNF587	protein_coding	ENSG00000198466	-0,087047172	0,149401359	0,231070196
SELENOT	protein_coding	ENSG00000198843	0,094035982	0,149423903	0,231087637
ERCC3	protein_coding	ENSG00000163161	-0,063544322	0,149621993	0,231376542
AC145138.1	unprocessed_pseudoge	ENSG00000251634	0,426637953	0,149636958	0,231382239
SLC35F5	protein_coding	ENSG00000115084	-0,057779012	0,149649414	0,231382815
PCDHB12	protein_coding	ENSG00000120328	-0,436570138	0,149659894	0,231382815
AC073548.1	TEC	ENSG00000279861	-0,411254416	0,149684421	0,231403292
RPS11	protein_coding	ENSG00000142534	-0,108091801	0,149707787	0,231421971
TMEM241	protein_coding	ENSG00000134490	-0,104494716	0,149760674	0,231486278
FTCD	protein_coding	ENSG00000160282	0,359365279	0,149902875	0,231688618
TAS2R64P	transcribed_unprocesse	ENSG00000256274	0,412469799	0,149965521	0,231767978
AC109460.1	antisense	ENSG00000260367	0,416069778	0,149979459	0,231772054
DEFB122	transcribed_unprocesse	ENSG00000204547	0,392092585	0,149991202	0,231772739
CEP76	protein_coding	ENSG00000101624	0,130813886	0,150023761	0,231788123
GNA11	protein_coding	ENSG00000088256	0,100586662	0,150022115	0,231788123
GIHCG	lincRNA	ENSG00000257698	-0,114356129	0,150169436	0,231995717
EPS8	protein_coding	ENSG00000151491	0,069113691	0,150211396	0,232043062
AC068987.3	lincRNA	ENSG00000260473	0,431061351	0,150321943	0,232196344
NARS	protein_coding	ENSG00000134440	0,054518185	0,150367227	0,232248801
TTLL5	protein_coding	ENSG00000119685	0,078724413	0,150450566	0,232360024
ZSCAN2	protein_coding	ENSG00000176371	-0,146386728	0,150509165	0,232433023
AC090617.3	antisense	ENSG00000262533	0,396125741	0,150628912	0,232600435

CA2	protein_coding	ENSG00000104267	0,396048993	0,150686345	0,232671605
AC020917.4	TEC	ENSG00000280332	-0,430948041	0,150706443	0,232685121
MBD4	protein_coding	ENSG00000129071	-0,080556056	0,150779442	0,232780305
MTDH	protein_coding	ENSG00000147649	-0,083791415	0,150813029	0,232814633
RPS2P7	processed_pseudogene	ENSG00000235508	-0,19317433	0,150959526	0,233023247
CALM2P2	processed_pseudogene	ENSG00000229097	-0,365642104	0,15099536	0,23306102
AC007038.2	antisense	ENSG00000272807	-0,416008136	0,151158675	0,233295539
RRN3	protein_coding	ENSG00000085721	0,066484345	0,151270134	0,233449996
C1QTNF6	protein_coding	ENSG00000133466	-0,155967872	0,151309938	0,233482236
SIRT3	protein_coding	ENSG00000142082	-0,114283742	0,15135432	0,233527203
ZFP41	protein_coding	ENSG00000181638	0,213482359	0,151370539	0,233534659
SNORD111	snoRNA	ENSG00000221066	-0,423042092	0,151449924	0,23363956
AC108727.1	sense_intronic	ENSG00000248773	-0,418252386	0,151543414	0,233766201
RF00003	snRNA	ENSG00000277918	-0,306143506	0,151616015	0,233860606
C2orf88	protein_coding	ENSG00000187699	-0,306560394	0,151668453	0,233923896
LINC00638	lincRNA	ENSG00000258701	0,385086884	0,151703576	0,233960474
LANCL3	protein_coding	ENSG00000147036	-0,165318764	0,151757021	0,234025301
FTLP12	processed_pseudogene	ENSG00000265095	-0,423589334	0,15177593	0,234036864
MRPL13	protein_coding	ENSG00000172172	-0,103033044	0,151821293	0,234089214
SSTR2	protein_coding	ENSG00000180616	-0,196468233	0,151834408	0,234091837
PRR29-AS1	lincRNA	ENSG00000264954	-0,364110762	0,151967222	0,234278992
RAB6B	protein_coding	ENSG00000154917	-0,088286033	0,152045706	0,23438237
MFSD1	protein_coding	ENSG00000118855	0,077870224	0,152090587	0,234433935
ALDH3A1	protein_coding	ENSG00000108602	0,388618225	0,152193528	0,23457498
TECPR1	protein_coding	ENSG00000205356	-0,130676152	0,152267258	0,234670984
AC084125.2	processed_transcript	ENSG00000255182	0,429793863	0,152286726	0,234683354
AC067930.2	antisense	ENSG00000254812	0,428295604	0,152381409	0,234811623
RSPO3	protein_coding	ENSG00000146374	0,419374886	0,152411804	0,234840815
BCL11A	protein_coding	ENSG00000119866	-0,161043663	0,152542701	0,23502485
AC135721.1	transcribed_processed_	ENSG00000267681	0,430290336	0,152777488	0,235368909
ZNF284	protein_coding	ENSG00000186026	-0,160176882	0,152832784	0,235436412
RNU6-1263P	snRNA	ENSG00000207331	0,349481837	0,152864344	0,235467344
PIH1D1	protein_coding	ENSG00000104872	-0,063736771	0,15300412	0,235664952
ANO8	protein_coding	ENSG00000074855	0,184633052	0,153064807	0,235740722
LBH	protein_coding	ENSG00000213626	0,430205285	0,153311081	0,236049116
AQP1	protein_coding	ENSG00000240583	0,385258977	0,153291803	0,236049116
SLC48A1	protein_coding	ENSG00000211584	0,104247425	0,153305787	0,236049116
PLEKHA5	protein_coding	ENSG00000052126	0,09253159	0,153302191	0,236049116
FBRSL1	protein_coding	ENSG00000112787	-0,141990003	0,153341225	0,236077805
DTNB	protein_coding	ENSG00000138101	-0,105811545	0,153442373	0,236198069

HNRNPA1L2	protein_coding	ENSG00000139675	0,190631405	0,15355814	0,236358533
CLDN10	protein_coding	ENSG00000134873	-0,426160938	0,153570097	0,2363592
AP001258.1	processed_pseudogene	ENSG00000255381	-0,375327147	0,153585989	0,236365922
AC006213.5	sense_intronic	ENSG00000277806	-0,333623885	0,153672675	0,236481585
AL132639.2	antisense	ENSG00000258940	-0,349845847	0,153829229	0,236697596
PRPF38B	protein_coding	ENSG00000134186	-0,077657716	0,15384239	0,236697596
SPRYD7	protein_coding	ENSG00000123178	-0,109142616	0,153859208	0,236697596
C6orf226	protein_coding	ENSG00000221821	-0,219544065	0,153856747	0,236697596
RAB29	protein_coding	ENSG00000117280	-0,057694511	0,153935365	0,236796996
RPL4P6	processed_pseudogene	ENSG00000230071	0,421134693	0,1540249	0,236916957
MYPOP	protein_coding	ENSG00000176182	0,200344822	0,154150449	0,237092292
GMDS-DT	lincRNA	ENSG00000250903	0,187418048	0,154282431	0,237277493
GSR	protein_coding	ENSG00000104687	-0,067637383	0,154378831	0,23740795
TIMM23B	protein_coding	ENSG00000204152	0,115759868	0,154457021	0,237510384
EXOC1	protein_coding	ENSG00000090989	0,07163457	0,154534163	0,237611192
ETNK2	protein_coding	ENSG00000143845	-0,130459346	0,154680524	0,237818406
GCNA	protein_coding	ENSG00000147174	0,235149667	0,154875062	0,238099657
AC008771.1	antisense	ENSG00000249042	-0,218198808	0,154942848	0,238186015
APOPT1	protein_coding	ENSG00000256053	0,131533845	0,154969328	0,238208867
HSF1	protein_coding	ENSG00000185122	0,08875332	0,155144344	0,238460019
TRAPPC2	protein_coding	ENSG00000196459	0,087342606	0,155250093	0,238586798
USP27X-AS1	lincRNA	ENSG00000234390	0,268531662	0,155364739	0,238745095
GCC1	protein_coding	ENSG00000179562	0,090112346	0,155754859	0,239326652
CCDC154	protein_coding	ENSG00000197599	0,425949467	0,155848152	0,239452063
ZDHHC24	protein_coding	ENSG00000174165	0,107106856	0,155914298	0,239535749
ACSL4	protein_coding	ENSG00000068366	0,079072294	0,156151464	0,239882145
AC083880.1	antisense	ENSG00000273391	0,372312315	0,156176839	0,239903159
AF129075.1	sense_intronic	ENSG00000231125	-0,405518672	0,156244816	0,239989603
OPLAH	protein_coding	ENSG00000178814	-0,186583989	0,156256981	0,239990316
AP002360.1	lincRNA	ENSG00000255135	0,157191174	0,15637551	0,240154377
AC098828.1	processed_pseudogene	ENSG00000213729	0,367569096	0,156401244	0,240175915
LINC02043	lincRNA	ENSG00000232233	0,371129158	0,156458499	0,240245849
ZNF35	protein_coding	ENSG00000169981	0,102529821	0,156526695	0,240332573
AL662797.1	antisense	ENSG00000272540	0,419575106	0,156562695	0,240369853
TMEM110-MUSTN1	protein_coding	ENSG00000248592	-0,356179215	0,156632157	0,240458498
RF00017	misc_RNA	ENSG00000276645	-0,354876483	0,156688742	0,240527363
SPAAR	protein_coding	ENSG00000235387	0,356207534	0,156732758	0,240576924
AL157756.1	antisense	ENSG00000254718	0,423274743	0,156991691	0,24093831
AC008429.3	lincRNA	ENSG00000254295	0,398630577	0,156989368	0,24093831
AL358074.1	processed_transcript	ENSG00000229582	-0,403291243	0,157097566	0,24108276

SGF29	protein_coding	ENSG00000176476	-0,136717785	0,157151891	0,241148084
RPS23	protein_coding	ENSG00000186468	-0,102129928	0,157243476	0,241270568
EGFR	protein_coding	ENSG00000146648	-0,099704643	0,157288352	0,241285274
ZBED3	protein_coding	ENSG00000132846	-0,261731492	0,157284623	0,241285274
AL442125.1	sense_intronic	ENSG00000276248	-0,33506535	0,157274635	0,241285274
CDC42P6	processed_pseudogene	ENSG00000237350	-0,337091401	0,15732769	0,241317973
TMEM64	protein_coding	ENSG00000180694	-0,079219016	0,1573332	0,241317973
RPS3AP5	processed_pseudogene	ENSG00000178429	0,35184687	0,157358055	0,241327765
KIF12	protein_coding	ENSG00000136883	-0,423604405	0,157363117	0,241327765
TARSL2	protein_coding	ENSG00000185418	0,099110729	0,157443132	0,241432421
AC104113.1	lincRNA	ENSG00000272308	0,425314634	0,157558706	0,241591586
HOXC4	protein_coding	ENSG00000198353	0,296111097	0,157641191	0,241699995
COL15A1	protein_coding	ENSG00000204291	0,365818232	0,157736058	0,241827369
RACK1	protein_coding	ENSG00000204628	-0,073643621	0,157842991	0,241973223
C4BPB	protein_coding	ENSG00000123843	0,280574747	0,157981592	0,242167597
FBXO30	protein_coding	ENSG00000118496	0,095800038	0,158113468	0,242351636
AL353651.1	lincRNA	ENSG00000235011	0,383915141	0,158155211	0,242397502
AP001528.1	lincRNA	ENSG00000246523	0,39099156	0,158301486	0,242557271
LINC00960	lincRNA	ENSG00000242516	0,113979431	0,158309178	0,242557271
RTF2	protein_coding	ENSG00000022277	-0,066171067	0,158287983	0,242557271
ALKBH8	protein_coding	ENSG00000137760	-0,077910386	0,158325212	0,242557271
ARFGAP3	protein_coding	ENSG00000242247	-0,084897569	0,158272599	0,242557271
METTL27	protein_coding	ENSG00000165171	-0,165196454	0,158330412	0,242557271
CYP2D8P	unprocessed_pseudoge	ENSG00000226450	-0,40797322	0,158389728	0,242630018
AC005614.1	antisense	ENSG00000269296	-0,387137941	0,158538173	0,242839277
VAMP4	protein_coding	ENSG00000117533	0,094046456	0,158632249	0,242965232
DRC3	protein_coding	ENSG00000171962	0,295528359	0,158710019	0,243066195
AC112220.2	bidirectional_promoter_	ENSG00000271643	-0,210447973	0,158837865	0,243225669
ADD3-AS1	processed_transcript	ENSG00000203876	0,357554217	0,158828901	0,243225669
NUDT7	protein_coding	ENSG00000140876	-0,121615176	0,158888215	0,243284606
AC016065.1	antisense	ENSG00000246089	-0,183416869	0,158909313	0,243298747
AC100810.3	lincRNA	ENSG00000282021	-0,425061536	0,15895165	0,243334701
AC009948.1	processed_pseudogene	ENSG00000238082	0,420741508	0,158956525	0,243334701
AC096637.2	antisense	ENSG00000231057	0,419648825	0,159011725	0,243401036
IFI27	protein_coding	ENSG00000165949	0,416159868	0,159140221	0,243579547
AC026436.1	processed_pseudogene	ENSG00000248794	-0,405991437	0,159187192	0,243633259
FOXD3	protein_coding	ENSG00000187140	0,240031072	0,159491733	0,24408114
AC073655.1	antisense	ENSG00000258172	0,339807562	0,159554415	0,244140634
PIGP	protein_coding	ENSG00000185808	0,126815977	0,159545748	0,244140634
UBE2Q2P2	transcribed_unprocesse	ENSG00000259429	-0,22238765	0,159596784	0,244187246

TRUB2	protein_coding	ENSG00000167112	-0,08682685	0,159645711	0,244236894
ZNF219	protein_coding	ENSG00000165804	0,143576935	0,159742731	0,244355862
WBP1LP2	processed_pseudogene	ENSG00000250474	0,348254093	0,159983033	0,244705199
HMGN2P46	transcribed_processed_	ENSG00000179362	0,412338337	0,160028909	0,244757115
LENG1	protein_coding	ENSG00000105617	-0,116583361	0,16010684	0,244858049
PDCD4	protein_coding	ENSG00000150593	-0,08711049	0,160217111	0,245008421
AC243312.1	processed_pseudogene	ENSG00000261866	0,35358845	0,160275172	0,245078937
AC100858.3	lincRNA	ENSG00000255491	-0,412978997	0,160308524	0,245111662
RAET1G	protein_coding	ENSG00000203722	0,406183471	0,160329399	0,245113976
ATG16L1	protein_coding	ENSG00000085978	0,06634796	0,160333939	0,245113976
NAPA-AS1	antisense	ENSG00000268061	-0,358120451	0,160544087	0,24539866
PNPLA4	protein_coding	ENSG00000006757	0,149352882	0,160543433	0,24539866
SLC25A40	protein_coding	ENSG00000075303	-0,087288185	0,160588942	0,245448931
ZNF236-DT	lincRNA	ENSG00000264278	0,367894675	0,160753891	0,245682735
WDR37	protein_coding	ENSG00000047056	0,073493552	0,160813156	0,245754998
ZBTB8OS	protein_coding	ENSG00000176261	0,090450741	0,160844259	0,245784217
FOXC1	protein_coding	ENSG00000054598	-0,109125916	0,160863206	0,245794856
ANKRD6	protein_coding	ENSG00000135299	-0,166366387	0,160881337	0,245804247
SCCPDH	protein_coding	ENSG00000143653	-0,075921503	0,16093609	0,245869587
DENND6A-DT	lincRNA	ENSG00000241933	0,418912902	0,16100471	0,2459561
AC106795.2	antisense	ENSG00000249684	-0,327768536	0,161032304	0,245979932
MTHFD2L	protein_coding	ENSG00000163738	0,137696898	0,161062915	0,24600837
BICRAL	protein_coding	ENSG00000112624	-0,109039958	0,161075112	0,246008679
AP000350.5	antisense	ENSG00000272973	-0,379405401	0,161092645	0,246017136
PRKAA2	protein_coding	ENSG00000162409	-0,103599148	0,161235835	0,24621748
C2orf49	protein_coding	ENSG00000135974	0,110200073	0,161364333	0,246395359
AC098820.1	processed_transcript	ENSG00000232485	0,341873192	0,161401489	0,246433748
AL162231.1	protein_coding	ENSG00000187186	0,421235931	0,161420104	0,246443825
MSMP	protein_coding	ENSG00000215183	0,415941794	0,161439593	0,246455233
KIF27	protein_coding	ENSG00000165115	0,121831138	0,16152524	0,24654928
FLII	protein_coding	ENSG00000177731	-0,070662049	0,16151834	0,24654928
RN7SKP71	misc_RNA	ENSG00000201428	0,319544302	0,161612152	0,246663584
RHOG	protein_coding	ENSG00000177105	0,103935583	0,161736224	0,246834583
SH2D1B	protein_coding	ENSG00000198574	0,419839171	0,161791253	0,246900193
STK26	protein_coding	ENSG00000134602	-0,101432797	0,161865519	0,246995148
AP002026.1	antisense	ENSG00000246090	0,311201346	0,16198604	0,247160666
ZBTB48	protein_coding	ENSG00000204859	0,132724411	0,162006134	0,247172938
AL139035.1	lincRNA	ENSG00000280710	0,408151322	0,162072577	0,247255917
SLC9A5	protein_coding	ENSG00000135740	0,226213202	0,16209	0,247264106
ALPP	protein_coding	ENSG00000163283	0,344065428	0,162134211	0,247313154

KPNA5	protein_coding	ENSG00000196911	0,115360471	0,162176856	0,247359806
HECTD4	protein_coding	ENSG00000173064	0,112519032	0,162212051	0,247395089
CRCP	protein_coding	ENSG00000241258	0,071592449	0,162231955	0,247407049
ADAM15	protein_coding	ENSG00000143537	-0,10730865	0,162246625	0,247411024
FOXC2	protein_coding	ENSG00000176692	-0,171988905	0,162312184	0,247492594
CFTR	protein_coding	ENSG00000001626	0,340909371	0,162360271	0,247547514
CCDC80	protein_coding	ENSG00000091986	0,05899348	0,162400161	0,247589928
OSBPL11	protein_coding	ENSG00000144909	0,059925937	0,162414496	0,247593378
NCOA2	protein_coding	ENSG00000140396	-0,103557615	0,162512487	0,247724348
AC125611.2	processed_pseudogene	ENSG00000257954	0,412145603	0,162561518	0,24777393
ANKH	protein_coding	ENSG00000154122	-0,089349818	0,162569175	0,24777393
AL049539.1	sense_intronic	ENSG00000275576	0,36179963	0,162704675	0,247962022
GMEB2	protein_coding	ENSG00000101216	-0,105576927	0,162720476	0,247967677
FRZB	protein_coding	ENSG00000162998	0,41684203	0,162819946	0,248092826
PPP6R1	protein_coding	ENSG00000105063	0,103872239	0,162826793	0,248092826
AL589986.2	lincRNA	ENSG00000236427	-0,360801534	0,162934335	0,248238242
STX18-AS1	antisense	ENSG00000247708	0,246812334	0,163027375	0,248361544
LINC02166	lincRNA	ENSG00000260259	0,417368922	0,163053671	0,248383155
RASSF1	protein_coding	ENSG00000068028	-0,101663022	0,163083332	0,248409888
RALGAPA2	protein_coding	ENSG00000188559	-0,116935403	0,163144677	0,248484875
TBCA	protein_coding	ENSG00000171530	-0,099314006	0,163279019	0,248671025
FAM81B	protein_coding	ENSG00000153347	0,391573979	0,163361961	0,24877887
IRF3	protein_coding	ENSG00000126456	0,104360945	0,163388335	0,248800561
FOXO6	protein_coding	ENSG00000204060	0,413066557	0,163518009	0,248979537
ANKRD23	protein_coding	ENSG00000163126	0,41338952	0,163746289	0,249290112
AC026304.1	lincRNA	ENSG00000268129	-0,285130272	0,163781621	0,249325394
LINC02001	lincRNA	ENSG00000267321	-0,147096965	0,164020665	0,249670761
NAA16	protein_coding	ENSG00000172766	0,109087207	0,164077879	0,249739318
OSBPL3	protein_coding	ENSG00000070882	0,067016773	0,164195897	0,249900404
NAB2	protein_coding	ENSG00000166886	0,114019405	0,164235031	0,249941417
AC022167.1	antisense	ENSG00000259939	0,391184179	0,164259413	0,249959976
ZNF12	protein_coding	ENSG00000164631	-0,068073643	0,164329213	0,250047642
AC103810.1	transcribed_unprocesse	ENSG00000264057	0,347762665	0,164610945	0,250457751
AC103746.1	lincRNA	ENSG00000275322	0,409282684	0,164680532	0,250545042
AC100861.1	processed_transcript	ENSG00000246582	0,143864273	0,164812573	0,250727331
TPM3	protein_coding	ENSG00000143549	-0,061661502	0,16485245	0,250765763
CEP162	protein_coding	ENSG00000135315	-0,111366071	0,164862289	0,250765763
RNF222	protein_coding	ENSG00000189051	0,414781188	0,164934826	0,250857493
AC098828.2	lincRNA	ENSG00000223734	0,413078655	0,165043396	0,251004009
AC015982.1	antisense	ENSG00000272606	0,340834928	0,165144143	0,251138606

CXorf56	protein_coding	ENSG00000018610	-0,072500325	0,165204815	0,251212245
LRRD1	protein_coding	ENSG00000240720	0,38885576	0,165238005	0,251244087
IRX5	protein_coding	ENSG00000176842	-0,349223704	0,165389358	0,251455577
CTTN	protein_coding	ENSG00000085733	-0,06751393	0,165435314	0,251506803
ACBD4	protein_coding	ENSG00000181513	-0,152169579	0,165468781	0,251539037
MIR378D2HG	lincRNA	ENSG00000264448	0,377348994	0,165619913	0,251731467
ABCA17P	transcribed_unitary_pse	ENSG00000238098	-0,411159331	0,165720324	0,25186542
SCARNA20	scaRNA	ENSG00000252577	-0,40938993	0,165780267	0,251937854
KAT6B	protein_coding	ENSG00000156650	0,114260255	0,165806272	0,251958705
ZNF613	protein_coding	ENSG00000176024	0,212389128	0,165882581	0,252037317
ARV1	protein_coding	ENSG00000173409	-0,084965131	0,165872562	0,252037317
LRRC37A15P	processed_pseudogene	ENSG00000230069	0,283348875	0,16597463	0,252158493
AP002360.3	lincRNA	ENSG00000272301	-0,388115143	0,166050265	0,252254718
LINC01881	transcribed_unprocesse	ENSG00000220804	0,290193103	0,166124957	0,252349494
ZNF829	protein_coding	ENSG00000185869	0,16365883	0,166162719	0,252383923
STC1	protein_coding	ENSG00000159167	0,109633171	0,166172233	0,252383923
UBE2W	protein_coding	ENSG00000104343	0,108551914	0,166319165	0,25258788
C11orf54	protein_coding	ENSG00000182919	-0,156474217	0,166331151	0,25258788
PHKG2	protein_coding	ENSG00000156873	0,107649174	0,166389416	0,252657654
SLC52A3	protein_coding	ENSG00000101276	0,358869159	0,166415791	0,252660292
GNG7	protein_coding	ENSG00000176533	0,234235729	0,166411303	0,252660292
EMC2	protein_coding	ENSG00000104412	-0,085934348	0,166460386	0,25270929
PDE2A	protein_coding	ENSG00000186642	0,142704719	0,166487918	0,252732381
IL1R1	protein_coding	ENSG00000115594	0,277331493	0,166561263	0,252825007
FZD7	protein_coding	ENSG00000155760	-0,102619627	0,166686923	0,252978303
MOSPD3	protein_coding	ENSG00000106330	-0,168863146	0,166679504	0,252978303
FAM200B	protein_coding	ENSG00000237765	-0,088889379	0,166832539	0,253177298
PTOV1	protein_coding	ENSG00000104960	-0,097980159	0,166842729	0,253177298
AL451164.1	lincRNA	ENSG00000236968	0,35680836	0,166943022	0,253292009
RPH3AL	protein_coding	ENSG00000181031	-0,136696537	0,16693412	0,253292009
FLJ45513	antisense	ENSG00000204584	0,162565404	0,1671099	0,253526447
NECAP2	protein_coding	ENSG00000157191	0,089984413	0,16717663	0,253608926
PRPF40B	protein_coding	ENSG00000110844	-0,128664217	0,167208189	0,253638042
KCMF1	protein_coding	ENSG00000176407	0,068149389	0,167346471	0,253829028
VN1R1	protein_coding	ENSG00000178201	-0,326721526	0,167437072	0,25394767
RAB27A	protein_coding	ENSG00000069974	0,107372923	0,167450353	0,253949035
KIF5A	protein_coding	ENSG00000155980	0,391875407	0,167540595	0,254048322
SLC22A31	protein_coding	ENSG00000259803	-0,4007915	0,16753692	0,254048322
LINC01182	lincRNA	ENSG00000250634	0,410084216	0,16756329	0,254063763
BCAP31	protein_coding	ENSG00000185825	0,078480073	0,167575553	0,254063763

AC090579.1	antisense	ENSG00000253582	-0,327002665	0,167603592	0,254076579
DNAL4	protein_coding	ENSG00000100246	0,113245511	0,167608782	0,254076579
AL683807.2	lincRNA	ENSG00000234622	0,352948233	0,167723531	0,254231736
PITPNC1	protein_coding	ENSG00000154217	0,171077546	0,167811996	0,254347031
AC090948.2	antisense	ENSG00000272498	0,389020028	0,168048535	0,254667907
AC090833.1	lincRNA	ENSG00000249867	0,178050234	0,168036792	0,254667907
GOLGA8UP	unprocessed_pseudoge	ENSG00000103832	0,403517788	0,168105233	0,254735008
RBMS2P1	processed_pseudogene	ENSG00000213250	-0,3394066	0,168148568	0,254750498
SLC49A3	protein_coding	ENSG00000169026	0,393866742	0,168161972	0,254750498
MAGI2	protein_coding	ENSG00000187391	0,209033102	0,168165139	0,254750498
AP002364.1	TEC	ENSG00000280367	0,353334438	0,168150377	0,254750498
AC015909.5	TEC	ENSG00000279792	-0,294233589	0,168223052	0,254819409
RPL7P24	processed_pseudogene	ENSG00000240003	0,389709897	0,16852029	0,255250804
AC092490.1	lincRNA	ENSG00000249790	0,372345655	0,168560433	0,255292754
SORD2P	transcribed_unprocesse	ENSG00000259479	0,185245718	0,168573264	0,255293335
AP002784.2	processed_pseudogene	ENSG00000256745	0,371965207	0,168630779	0,255361582
MRPL47	protein_coding	ENSG00000136522	-0,083371721	0,168646481	0,255366506
KIF3A	protein_coding	ENSG00000131437	-0,096443867	0,168665741	0,255376816
FAM167B	protein_coding	ENSG00000183615	0,361964616	0,168714763	0,255432184
NPPB	protein_coding	ENSG00000120937	0,34476463	0,168741354	0,255453585
MRPL37P1	processed_pseudogene	ENSG00000266946	0,363297745	0,168820929	0,25555519
MB	protein_coding	ENSG00000198125	0,362157757	0,168848247	0,255577679
SELENON	protein_coding	ENSG00000162430	-0,103970058	0,168865512	0,25558495
AL445483.1	lincRNA	ENSG00000260088	0,393637196	0,169035867	0,25582391
CHUK	protein_coding	ENSG00000213341	0,073205456	0,169060635	0,255835863
AC135983.2	transcribed_processed_	ENSG00000223509	-0,142833114	0,169068712	0,255835863
KEAP1	protein_coding	ENSG00000079999	-0,083834351	0,169186418	0,25599509
AL138831.1	antisense	ENSG00000230648	0,353903879	0,169216863	0,256022267
RUBCNL	protein_coding	ENSG00000102445	0,336214974	0,169274834	0,256091085
NUTM2B	protein_coding	ENSG00000188199	0,388691981	0,169484139	0,256388824
EXOC3-AS1	antisense	ENSG00000221990	-0,204657123	0,16952175	0,256426809
SYCE3	protein_coding	ENSG00000217442	-0,397054443	0,169550875	0,256451951
RF00019	misc_RNA	ENSG00000206814	0,371734412	0,169648649	0,256580915
TMEM214	protein_coding	ENSG00000119777	0,08672596	0,169873296	0,256882792
AC025031.1	lincRNA	ENSG00000257496	0,339987274	0,169901723	0,256906837
ADIPOR2	protein_coding	ENSG00000006831	-0,050653292	0,169996862	0,257031747
PCTP	protein_coding	ENSG00000141179	-0,085850375	0,170171844	0,257277349
FBXL6	protein_coding	ENSG00000182325	0,122764305	0,170195797	0,257294596
KCNN1	protein_coding	ENSG00000105642	0,216257988	0,170350416	0,257509362
AC244197.3	protein_coding	ENSG00000241489	-0,337984573	0,17036922	0,257518808

RPL5P4	processed_pseudogene	ENSG00000229994	0,267974759	0,17057194	0,257787229
NDUFA12	protein_coding	ENSG00000184752	-0,075239703	0,170561556	0,257787229
MED14	protein_coding	ENSG00000180182	-0,078249365	0,170632448	0,257859674
BASP1-AS1	antisense	ENSG00000215196	0,387360042	0,170690496	0,257926599
TMSB15B-AS1	antisense	ENSG00000231728	-0,335270906	0,170701884	0,257926599
ZSCAN16-AS1	antisense	ENSG00000269293	-0,156798153	0,17082365	0,25809157
CWF19L2	protein_coding	ENSG00000152404	0,086753881	0,170854618	0,258119345
UBE2MP1	processed_pseudogene	ENSG00000261461	0,398823457	0,171247656	0,2586622
MEIOC	protein_coding	ENSG00000180336	0,256010514	0,171235937	0,2586622
RPL14	protein_coding	ENSG00000188846	-0,08014097	0,17125178	0,2586622
ANKRD20A2	protein_coding	ENSG00000183148	-0,372013014	0,171314424	0,258737764
RXYLT1	protein_coding	ENSG00000118600	0,113633341	0,171364085	0,258793711
LINC01783	lincRNA	ENSG00000233421	-0,315191946	0,171495651	0,258973332
AC092279.1	lincRNA	ENSG00000268362	-0,208344621	0,171538332	0,259018714
ANKRD20A11P	transcribed_unprocessed	ENSG00000215559	-0,337489336	0,171674132	0,259204685
CEBPD	protein_coding	ENSG00000221869	0,209404865	0,171961743	0,259619827
AL450998.2	antisense	ENSG00000179743	0,219458693	0,17213625	0,259864161
PLAG1	protein_coding	ENSG00000181690	0,148482757	0,172161203	0,259882702
AL358075.2	antisense	ENSG00000227857	0,38357701	0,172261531	0,259995879
IL16	protein_coding	ENSG00000172349	0,404363912	0,172253429	0,259995879
ANKRD18A	protein_coding	ENSG00000180071	-0,200444019	0,172375696	0,260149045
ARHGEF25	protein_coding	ENSG00000240771	-0,127749795	0,172410868	0,260182981
AC004453.1	processed_pseudogene	ENSG00000146677	0,231992733	0,172450279	0,260223309
RPS15AP11	processed_pseudogene	ENSG00000234093	0,369682751	0,172541005	0,260335872
NXN	protein_coding	ENSG00000167693	-0,078025218	0,172558122	0,260335872
MCOLN2	protein_coding	ENSG00000153898	-0,087057926	0,172562953	0,260335872
PUS3	protein_coding	ENSG00000110060	-0,113418432	0,1728416	0,260737071
AC008443.5	antisense	ENSG00000250222	-0,223108659	0,172874212	0,260767088
LINC01134	lincRNA	ENSG00000236423	0,387191272	0,172971234	0,260869951
CAP2	protein_coding	ENSG00000112186	0,06494439	0,172993282	0,260869951
B3GNT2	protein_coding	ENSG00000170340	-0,081364021	0,172983209	0,260869951
POLI	protein_coding	ENSG00000101751	-0,095615873	0,172990556	0,260869951
AL445649.1	sense_intronic	ENSG00000276740	0,340619727	0,173039347	0,260920233
ZNF37BP	transcribed_processed_	ENSG00000234420	0,11064884	0,173123557	0,26102802
AC022211.1	sense_intronic	ENSG00000263786	0,376101173	0,173179071	0,261092528
AC011498.6	antisense	ENSG00000267769	0,40289271	0,173205677	0,261113446
LINC01963	lincRNA	ENSG00000260804	-0,172724302	0,173245055	0,261153614
AC078860.1	antisense	ENSG00000257761	0,348790376	0,173302222	0,26122059
LHFPL4	protein_coding	ENSG00000156959	-0,132189684	0,173359223	0,261287306
DOC2A	protein_coding	ENSG00000149927	0,239263037	0,173373417	0,261289498

F10	protein_coding	ENSG00000126218	0,306616494	0,173445219	0,261378504
RPS20P33	processed_pseudogene	ENSG00000242085	0,395105839	0,173464242	0,261387966
TMEM14DP	processed_pseudogene	ENSG00000214881	-0,382928703	0,173516157	0,261446986
AC063952.1	transcribed_unitary_pse	ENSG00000240661	0,162688701	0,173577447	0,261520123
BAD	protein_coding	ENSG00000002330	-0,133861646	0,173611971	0,261552925
ZNF213	protein_coding	ENSG00000085644	0,21942095	0,173711605	0,261683805
AL359182.1	antisense	ENSG00000226334	0,388517994	0,173769872	0,261752355
AL445647.1	lincRNA	ENSG00000272046	0,192789735	0,173787959	0,261757876
WASF2	protein_coding	ENSG00000158195	-0,082361081	0,173799062	0,261757876
C6orf89	protein_coding	ENSG00000198663	0,059787541	0,173843811	0,261806047
DUSP7	protein_coding	ENSG00000164086	-0,101279808	0,173886847	0,261851632
RPL24P2	processed_pseudogene	ENSG00000235065	-0,328316046	0,173952857	0,261915946
FRG1GP	unprocessed_pseudoge	ENSG00000283023	0,370115886	0,173955096	0,261915946
CLASP1	protein_coding	ENSG00000074054	0,071247424	0,174177564	0,262231655
BORCS6	protein_coding	ENSG00000196544	0,142462124	0,174193746	0,262236768
NOL10	protein_coding	ENSG00000115761	-0,080682572	0,174231501	0,262274354
AC025682.1	antisense	ENSG00000263766	0,350267277	0,174327754	0,262364886
AC004381.1	antisense	ENSG00000260510	0,315072659	0,174310504	0,262364886
AP001432.1	lincRNA	ENSG00000242553	0,394123655	0,174330018	0,262364886
RASSF4	protein_coding	ENSG00000107551	-0,144601861	0,174359408	0,262373789
AC021491.2	processed_transcript	ENSG00000250286	0,345026059	0,174568849	0,262666503
SORT1	protein_coding	ENSG00000134243	-0,067191777	0,174644473	0,262761013
HNRNPCP1	processed_pseudogene	ENSG00000258900	-0,238739591	0,174704677	0,262824874
DYNC2LI1	protein_coding	ENSG00000138036	-0,11641162	0,174712547	0,262824874
AC091948.1	antisense	ENSG00000247199	0,400674505	0,174821439	0,262933024
CFAP20	protein_coding	ENSG00000070761	0,07574073	0,174822899	0,262933024
AATK	protein_coding	ENSG00000181409	-0,36651198	0,174814794	0,262933024
RPL7AP9	processed_pseudogene	ENSG00000213272	0,32271708	0,174836645	0,262934418
PSMD5	protein_coding	ENSG00000095261	-0,05478294	0,174882786	0,262984525
BICDL1	protein_coding	ENSG00000135127	0,238540971	0,174906376	0,263000717
AC017104.4	processed_pseudogene	ENSG00000283312	0,38810068	0,174976104	0,263084549
GABBR1	protein_coding	ENSG00000204681	-0,194418991	0,174987783	0,263084549
TGFBRAP1	protein_coding	ENSG00000135966	0,091631787	0,175051469	0,263161008
CTSD	protein_coding	ENSG00000117984	0,129581872	0,175087835	0,263196386
GAPDH	protein_coding	ENSG00000111640	-0,071526673	0,175305791	0,26350471
AC093249.6	antisense	ENSG00000261840	-0,400365159	0,175651046	0,26395954
NOXO1	protein_coding	ENSG00000196408	0,362003239	0,175656917	0,26395954
WDR41	protein_coding	ENSG00000164253	-0,069130411	0,175656823	0,26395954
SOD1	protein_coding	ENSG00000142168	-0,084280394	0,175659862	0,26395954
FLCN	protein_coding	ENSG00000154803	0,089890688	0,175826703	0,264190893

AL596220.1	antisense	ENSG00000229739	0,399405221	0,175840871	0,264192826
FAM66B	lincRNA	ENSG00000215374	0,237568156	0,17611831	0,264590283
TBC1D8	protein_coding	ENSG00000204634	0,11352314	0,176175856	0,26465735
AL161454.1	processed_pseudogene	ENSG00000237631	-0,308483001	0,17636661	0,264919803
AC099336.2	processed_pseudogene	ENSG00000236439	-0,39439389	0,176376397	0,264919803
PELO	protein_coding	ENSG00000152684	-0,068759877	0,176412059	0,264953963
NDUFC2	protein_coding	ENSG00000151366	0,131962179	0,176570547	0,265172579
AC099811.1	sense_intronic	ENSG00000236194	-0,400506066	0,176630154	0,265242675
AC005546.1	lincRNA	ENSG00000266897	-0,33171571	0,176686322	0,265268755
SNORA80A	snoRNA	ENSG00000200792	-0,262661144	0,17667572	0,265268755
AC005077.4	processed_pseudogene	ENSG00000230882	0,360056167	0,176829424	0,26546417
LSM14A	protein_coding	ENSG00000257103	-0,063005025	0,176989888	0,265685618
SH3GL3	protein_coding	ENSG00000140600	-0,236278444	0,177073006	0,265790936
LIN7C	protein_coding	ENSG00000148943	0,108356022	0,177172545	0,265920885
PPP2R3A	protein_coding	ENSG00000073711	0,080320487	0,177265651	0,266021695
FOXJ3	protein_coding	ENSG00000198815	0,063525421	0,177264679	0,266021695
ELAVL1	protein_coding	ENSG00000066044	-0,050967639	0,177324247	0,266090159
CNKSR2	protein_coding	ENSG00000149970	-0,129535083	0,177459376	0,26627345
SLC25A51	protein_coding	ENSG00000122696	-0,107075421	0,177505788	0,266323607
ANKIB1	protein_coding	ENSG00000001629	0,075368249	0,177589893	0,266430306
LLPH-DT	antisense	ENSG00000239335	0,394489461	0,177667182	0,266526763
GRB10	protein_coding	ENSG00000106070	0,074487381	0,177728492	0,266599237
CSNK1G2	protein_coding	ENSG00000133275	-0,077806852	0,177756025	0,266621038
RBM47	protein_coding	ENSG00000163694	-0,220306834	0,177827974	0,266709451
SGCE	protein_coding	ENSG00000127990	0,079199816	0,177893766	0,266788619
MUTYH	protein_coding	ENSG00000132781	-0,119130175	0,178136147	0,267132585
AL590705.1	lincRNA	ENSG00000203279	-0,318657779	0,178198417	0,267186894
FRG1	protein_coding	ENSG00000109536	-0,116615617	0,178187441	0,267186894
AL354892.3	sense_intronic	ENSG00000266896	-0,386704205	0,178317372	0,267345709
Z69733.1	antisense	ENSG00000234405	0,309419753	0,178351779	0,267377749
CERS4	protein_coding	ENSG00000090661	0,124004188	0,178432068	0,267478563
AL355802.2	antisense	ENSG00000271754	0,395386979	0,178545277	0,267608825
RN7SL181P	misc_RNA	ENSG00000243738	-0,322008204	0,17854079	0,267608825
AC011933.2	sense_intronic	ENSG00000264853	0,392342139	0,178558107	0,267608825
AC020915.3	antisense	ENSG00000268516	-0,165334215	0,178584077	0,267628191
RNF215	protein_coding	ENSG00000099999	-0,131706408	0,178662042	0,267725469
SUFU	protein_coding	ENSG00000107882	0,162638151	0,178687782	0,267744478
NUDT9	protein_coding	ENSG00000170502	-0,122289033	0,17905349	0,268272852
CCDC160	protein_coding	ENSG00000203952	0,39688039	0,179397384	0,268748838
GFAP	protein_coding	ENSG00000131095	0,338943222	0,179391906	0,268748838

LINC02595	lincRNA	ENSG00000231566	0,336857988	0,179528079	0,268924985
AC016876.2	processed_transcript	ENSG00000264772	-0,374507065	0,1795999	0,269012923
AC011498.2	antisense	ENSG00000267030	0,392896132	0,179680405	0,269113853
SYT7	protein_coding	ENSG00000011347	-0,358664311	0,17969804	0,269120613
GPR157	protein_coding	ENSG00000180758	0,144111053	0,17981003	0,269241724
AMPD2	protein_coding	ENSG00000116337	-0,091799116	0,179831418	0,269241724
NDUFA6	protein_coding	ENSG00000184983	-0,094855915	0,179823833	0,269241724
AC055811.4	TEC	ENSG00000278864	-0,248709057	0,179805086	0,269241724
CENPU	protein_coding	ENSG00000151725	-0,086180424	0,179890693	0,269310811
HIRA	protein_coding	ENSG00000100084	-0,14668582	0,179959034	0,269393459
FBF1	protein_coding	ENSG00000188878	-0,156022712	0,180007103	0,269445752
AC104964.1	lincRNA	ENSG00000253641	0,351611942	0,180036679	0,269470356
CCDC78	protein_coding	ENSG00000162004	-0,284293347	0,18006405	0,269489178
AC074141.1	transcribed_unprocesse	ENSG00000283236	0,393967655	0,180075533	0,269489178
FAM102A	protein_coding	ENSG00000167106	0,114162222	0,180142304	0,269550235
DISP2	protein_coding	ENSG00000140323	-0,222647576	0,180142617	0,269550235
SNHG17	processed_transcript	ENSG00000196756	0,114131069	0,1802078	0,269628099
HSD17B7	protein_coding	ENSG00000132196	0,104824791	0,180302386	0,26974994
ACTBP7	processed_pseudogene	ENSG00000185607	0,384565552	0,180432037	0,269924221
C8orf48	protein_coding	ENSG00000164743	-0,163866995	0,180488639	0,269989205
AC079921.2	lincRNA	ENSG00000249685	0,395277664	0,180554922	0,270068658
KCTD3	protein_coding	ENSG00000136636	-0,058565099	0,18065287	0,270195461
LNCSRLR	antisense	ENSG00000240032	0,240837239	0,180697319	0,270240971
MPV17	protein_coding	ENSG00000115204	0,072198634	0,180709649	0,270240971
NDUFA9	protein_coding	ENSG00000139180	-0,11058597	0,180795514	0,270349663
FAM92A1P1	unprocessed_pseudoge	ENSG00000157021	-0,245434409	0,180845856	0,270405228
COL25A1	protein_coding	ENSG00000188517	0,390258622	0,180966125	0,270565332
BPTF	protein_coding	ENSG00000171634	0,097714922	0,18103743	0,270651558
GNPNAT1	protein_coding	ENSG00000100522	-0,071155858	0,181050189	0,270651558
RAB34	protein_coding	ENSG00000109113	-0,077441362	0,181074518	0,270668199
SNCAIP	protein_coding	ENSG00000064692	0,203233814	0,181166054	0,270785291
AC064850.1	processed_pseudogene	ENSG00000235651	0,395019125	0,181185372	0,270794431
AC025594.2	TEC	ENSG00000275106	-0,388584414	0,181270862	0,270902461
BCAS4	protein_coding	ENSG00000124243	-0,107024174	0,18144461	0,271142364
RF00019	misc_RNA	ENSG00000206728	-0,331808937	0,18156974	0,271301218
TTC23	protein_coding	ENSG00000103852	0,09201749	0,181577368	0,271301218
AC023632.2	lincRNA	ENSG00000253704	0,38931563	0,181672548	0,271387824
RPS24P8	processed_pseudogene	ENSG00000224094	0,369862421	0,18167278	0,271387824
ASAP1	protein_coding	ENSG00000153317	-0,073988902	0,181675028	0,271387824
DHX30	protein_coding	ENSG00000132153	-0,080056177	0,181724662	0,271442198

DNAH7	protein_coding	ENSG00000118997	0,328571179	0,181766953	0,271485596
CNNM2	protein_coding	ENSG00000148842	0,106400822	0,181820804	0,271546253
PIPSL	transcribed_processed_	ENSG00000180764	0,300580731	0,181897412	0,271640886
XPO6	protein_coding	ENSG00000169180	-0,082242918	0,182149565	0,27199764
AP001350.1	antisense	ENSG00000269570	-0,379331854	0,182483236	0,272438445
ZFP91	protein_coding	ENSG00000186660	-0,081505112	0,182459778	0,272438445
ACTR10	protein_coding	ENSG00000131966	-0,087960632	0,18248461	0,272438445
RASAL2-AS1	lincRNA	ENSG00000224687	0,218332621	0,182573366	0,272551112
AL356273.3	TEC	ENSG00000279838	-0,281424328	0,183090529	0,273303257
CRBN	protein_coding	ENSG00000113851	-0,090656612	0,183110996	0,273313917
FUBP3	protein_coding	ENSG00000107164	-0,059849805	0,183247692	0,273498047
AC110015.1	antisense	ENSG00000227279	0,381827619	0,18335772	0,273642352
PPP1CA	protein_coding	ENSG00000172531	-0,088155878	0,183385593	0,273664037
AC112721.1	lincRNA	ENSG00000222022	0,349969929	0,183498398	0,273772616
CRMP1	protein_coding	ENSG00000072832	0,336248734	0,183489317	0,273772616
MTCO3P12	unprocessed_pseudoge	ENSG00000198744	0,390856896	0,183487762	0,273772616
GPR160	protein_coding	ENSG00000173890	-0,142554703	0,183541523	0,273797123
AC095057.3	sense_overlapping	ENSG00000260296	0,389717954	0,18354116	0,273797123
ARHGAP23	protein_coding	ENSG00000275832	0,10961192	0,183565645	0,273813191
AC022898.2	sense_intronic	ENSG00000273792	-0,31266426	0,183655964	0,273908073
AC023790.2	lincRNA	ENSG00000255621	0,369926922	0,183703719	0,273959375
MYO9B	protein_coding	ENSG00000099331	-0,108188441	0,183737569	0,273989933
SAMD10	protein_coding	ENSG00000130590	-0,21469777	0,18378853	0,274046001
WASHC2A	protein_coding	ENSG00000099290	-0,085936951	0,183868775	0,274125795
AL390728.4	transcribed_unprocesse	ENSG00000227671	-0,169672655	0,183857482	0,274125795
AC053527.1	antisense	ENSG00000250220	0,388959751	0,18393844	0,274209726
ZNF346	protein_coding	ENSG00000113761	-0,088628109	0,184003981	0,274280913
AC005225.4	TEC	ENSG00000279026	0,389276003	0,184012938	0,274280913
AC131009.3	sense_intronic	ENSG00000273568	-0,318056107	0,18408732	0,274371843
ZNF254	protein_coding	ENSG00000213096	0,134943259	0,184144679	0,27443739
RF00422	scaRNA	ENSG00000252274	-0,381679494	0,184267923	0,274601112
RN7SKP70	misc_RNA	ENSG00000252464	-0,355450649	0,184412961	0,274797285
RBBP6	protein_coding	ENSG00000122257	0,098759083	0,184455664	0,274840949
GAS5-AS1	antisense	ENSG00000270084	0,377898057	0,184947224	0,275553361
THRB-IT1	sense_intronic	ENSG00000224822	0,327908743	0,185079116	0,275729838
AC098679.2	transcribed_processed_	ENSG00000251429	0,348484972	0,185127648	0,275782108
FGFRL1	protein_coding	ENSG00000127418	0,146451099	0,185247496	0,275940601
SNORD116-14	snoRNA	ENSG00000206621	0,357599172	0,185368009	0,276100063
PIBF1	protein_coding	ENSG00000083535	0,083311693	0,185407817	0,276139301
AC006435.2	antisense	ENSG00000263345	0,359683557	0,185457717	0,276153461

AC112484.2	processed_pseudogene	ENSG00000250796	0,367605893	0,185449475	0,276153461
ZNF532	protein_coding	ENSG00000074657	0,100495504	0,185440993	0,276153461
SPAG6	protein_coding	ENSG00000077327	0,355376195	0,185583806	0,276304581
AC104564.5	sense_intronic	ENSG00000265625	-0,385261897	0,185586149	0,276304581
LSG1	protein_coding	ENSG00000041802	0,049207371	0,185618394	0,27633253
PLAUR	protein_coding	ENSG00000011422	0,060231549	0,185761794	0,27652594
RN7SKP192	misc_RNA	ENSG00000223223	0,364531546	0,185820476	0,276593219
AC021242.3	antisense	ENSG00000272267	0,353432421	0,186021384	0,276872176
RF00019	misc_RNA	ENSG00000252367	-0,33919579	0,186061098	0,276911191
TLK2P1	processed_pseudogene	ENSG00000226049	0,347232628	0,186076075	0,276913388
DEPDC4	protein_coding	ENSG00000166153	-0,216284171	0,186153607	0,277008668
SVIP	protein_coding	ENSG00000198168	-0,106043694	0,186228532	0,277100058
GPX1P1	processed_pseudogene	ENSG00000197582	0,386870213	0,186268343	0,277139189
DUS1L	protein_coding	ENSG00000169718	-0,091604957	0,186562896	0,277557304
AC002470.1	lincRNA	ENSG00000272829	-0,354593946	0,186684401	0,277717927
OTUB1	protein_coding	ENSG00000167770	-0,075706274	0,186815209	0,277892366
PHLDB1	protein_coding	ENSG00000019144	0,089734967	0,18685656	0,277933719
SIKE1	protein_coding	ENSG00000052723	0,063235959	0,186967928	0,278079202
SLC2A2	protein_coding	ENSG00000163581	0,378740346	0,187019047	0,278135063
RPL19	protein_coding	ENSG00000108298	-0,097831248	0,187057113	0,278171504
YTHDF3-AS1	lincRNA	ENSG00000270673	0,312727967	0,18709158	0,278202589
E2F3	protein_coding	ENSG00000112242	0,076533985	0,187326233	0,278531321
SP2	protein_coding	ENSG00000167182	0,182056542	0,187517321	0,278795235
AC060766.5	transcribed_processed_	ENSG00000267648	0,33678183	0,187542845	0,278812971
HUS1B	protein_coding	ENSG00000188996	0,33915705	0,187586415	0,278857532
FRMPD3	protein_coding	ENSG00000147234	0,221851144	0,187672377	0,278965101
AGK	protein_coding	ENSG00000006530	0,061352286	0,18781843	0,279161968
ELK3	protein_coding	ENSG00000111145	-0,060283199	0,18791462	0,2792847
EOGT	protein_coding	ENSG00000163378	-0,081263488	0,18818335	0,279663829
RPL37A	protein_coding	ENSG00000197756	-0,082162392	0,188320541	0,279847434
ZBTB39	protein_coding	ENSG00000166860	-0,121526497	0,188499173	0,280092591
FAM118A	protein_coding	ENSG00000100376	0,085986589	0,188549288	0,280146761
AL136985.2	lincRNA	ENSG00000272226	0,355530934	0,188610875	0,280217967
CCDC32	protein_coding	ENSG00000128891	0,108049072	0,188672265	0,280288871
C11orf49	protein_coding	ENSG00000149179	-0,096821522	0,188801471	0,280421872
FAM206A	protein_coding	ENSG00000119328	-0,105418414	0,18880281	0,280421872
ROMO1	protein_coding	ENSG00000125995	-0,111584939	0,188776666	0,280421872
POU5F1	protein_coding	ENSG00000204531	0,320478526	0,188909647	0,280560236
NDUFAF5	protein_coding	ENSG00000101247	-0,128097205	0,18901305	0,280693481
DOLK	protein_coding	ENSG00000175283	-0,098538879	0,189068688	0,280755777

HOXC-AS1	antisense	ENSG00000250451	-0,382452607	0,189133942	0,280832343
AC145207.9	TEC	ENSG00000278876	-0,3705665	0,189184243	0,280886697
GRID1	protein_coding	ENSG00000182771	0,348237021	0,189279998	0,280988184
ARIH2	protein_coding	ENSG00000177479	-0,043815525	0,18927362	0,280988184
DOK3	protein_coding	ENSG00000146094	0,355378912	0,189316698	0,281022326
AC090498.1	processed_pseudogene	ENSG00000279483	-0,222282609	0,189401406	0,281124142
TRMT11	protein_coding	ENSG00000066651	-0,094117012	0,189412702	0,281124142
AMER1	protein_coding	ENSG00000184675	-0,136387882	0,189639969	0,281441082
MRPL58	protein_coding	ENSG00000167862	-0,09805904	0,18965901	0,281448976
AL627402.1	processed_pseudogene	ENSG00000217624	0,380248354	0,189698273	0,281479407
DLGAP3	protein_coding	ENSG00000116544	-0,224461347	0,189706964	0,281479407
LINC02154	lincRNA	ENSG00000235385	0,092915935	0,189879389	0,281699333
ACTBL2	protein_coding	ENSG00000169067	0,384535606	0,18989212	0,281699333
TIPRL	protein_coding	ENSG00000143155	-0,102132036	0,189896391	0,281699333
AC092118.1	lincRNA	ENSG00000187185	0,374560806	0,189933404	0,281713488
AL139349.1	sense_intronic	ENSG00000254419	-0,304206411	0,189929823	0,281713488
SWI5	protein_coding	ENSG00000175854	0,107789153	0,190046211	0,281860423
SLC22A14	protein_coding	ENSG00000144671	0,358689545	0,190069854	0,281875105
SNAP25-AS1	antisense	ENSG00000227906	0,369817217	0,19023564	0,282100569
ZNF776	protein_coding	ENSG00000152443	0,082814448	0,190256656	0,282111337
AL158212.3	sense_overlapping	ENSG00000260917	0,177691257	0,190356529	0,282239023
TPT1P6	processed_pseudogene	ENSG00000214460	0,345080172	0,190438389	0,282339986
COG5	protein_coding	ENSG00000164597	0,079410809	0,190480322	0,282361333
TRAF7	protein_coding	ENSG00000131653	-0,084505187	0,190473335	0,282361333
LINC01862	lincRNA	ENSG00000268480	0,384636222	0,190584142	0,282491807
FOXH1	protein_coding	ENSG00000160973	0,326354109	0,190595886	0,282491807
SPAG4	protein_coding	ENSG00000061656	0,186365364	0,190663122	0,282571041
PWP2	protein_coding	ENSG00000241945	-0,336458775	0,190698667	0,2826033
TMEM161B	protein_coding	ENSG00000164180	0,093041334	0,1910112	0,283046002
AC003005.2	lincRNA	ENSG00000268266	0,371794861	0,191120983	0,283179981
RPL12P38	transcribed_processed_	ENSG00000213228	0,370214427	0,191129229	0,283179981
CDH26	protein_coding	ENSG00000124215	0,3754308	0,19117204	0,283215474
P2RY11	protein_coding	ENSG00000244165	0,254045667	0,191180802	0,283215474
AC004987.2	processed_pseudogene	ENSG00000233225	-0,379866094	0,191271651	0,283329594
MAP3K11	protein_coding	ENSG00000173327	0,115916859	0,191338303	0,283407858
PCNT	protein_coding	ENSG00000160299	-0,125854208	0,191354827	0,283411864
ZNF487	protein_coding	ENSG00000243660	0,170221562	0,191463815	0,283552809
TTC21B	protein_coding	ENSG00000123607	-0,062962817	0,1914876	0,283567557
AL161640.1	lincRNA	ENSG00000230404	0,311688058	0,191513515	0,28358546
ADORA2A	protein_coding	ENSG00000128271	0,353410112	0,19155927	0,28361226

EFEMP2	protein_coding	ENSG00000172638	0,261035772	0,191559228	0,28361226
HECTD3	protein_coding	ENSG00000126107	-0,090240516	0,191650653	0,283727075
DENND6B	protein_coding	ENSG00000205593	0,31650879	0,191715675	0,283802851
CHMP4A	protein_coding	ENSG00000254505	0,31769594	0,191996806	0,284198506
CEMIP	protein_coding	ENSG00000103888	-0,309787843	0,192095015	0,284323358
HPSE	protein_coding	ENSG00000173083	-0,161664173	0,19214042	0,28437004
AC015849.6	processed_pseudogene	ENSG00000271268	0,319778816	0,192154523	0,284370392
TMSB4XP4	processed_pseudogene	ENSG00000223551	-0,352279848	0,19236821	0,284666089
ESRRA	protein_coding	ENSG00000173153	0,105858081	0,192410112	0,284707553
SNORA37	snoRNA	ENSG00000207233	-0,299876181	0,192507326	0,284830852
AC006058.3	lincRNA	ENSG00000272121	0,23707752	0,192600385	0,284947983
MLXIP	protein_coding	ENSG00000175727	-0,093688787	0,19264411	0,284992115
GEMIN7-AS1	antisense	ENSG00000267348	0,312488895	0,192742306	0,285096257
RPL23AP97	processed_pseudogene	ENSG00000260615	0,379143685	0,192736267	0,285096257
MAP3K7	protein_coding	ENSG00000135341	0,066317582	0,19288797	0,285291141
SSSCA1-AS1	processed_transcript	ENSG00000260233	0,380861969	0,192947836	0,285359109
GTF2H2B	transcribed_unprocesse	ENSG00000226259	-0,250413391	0,193017468	0,285441507
CAV1	protein_coding	ENSG00000105974	-0,047770741	0,193049895	0,28546728
PPM1E	protein_coding	ENSG00000175175	-0,270545086	0,193062732	0,28546728
LMO7DN	lincRNA	ENSG00000178734	0,353211336	0,193169104	0,285603973
AL049548.1	antisense	ENSG00000226193	0,353243431	0,193255915	0,285703905
ZNF706	protein_coding	ENSG00000120963	-0,075820487	0,193264553	0,285703905
FARSA	protein_coding	ENSG00000179115	-0,064260476	0,193341782	0,285797473
SLC19A2	protein_coding	ENSG00000117479	0,09889694	0,193472349	0,285969867
EIF2S2	protein_coding	ENSG00000125977	-0,068156512	0,193569758	0,286072614
RNU2-22P	snRNA	ENSG00000223198	0,355952721	0,193560582	0,286072614
FARS2	protein_coding	ENSG00000145982	0,078359869	0,193696811	0,286239758
RCHY1	protein_coding	ENSG00000163743	0,089209287	0,193814006	0,286392309
SHISA4	protein_coding	ENSG00000198892	-0,168672048	0,193931115	0,286544713
CKS1BP3	processed_pseudogene	ENSG00000268942	-0,379551867	0,194112927	0,28679269
YAF2	protein_coding	ENSG00000015153	0,106130252	0,194228357	0,286914138
EHD2	protein_coding	ENSG00000024422	-0,118227026	0,194226106	0,286914138
TNNI3	protein_coding	ENSG00000129991	0,368715189	0,19433369	0,287036151
CCDC90B	protein_coding	ENSG00000137500	0,077574043	0,194408772	0,28712637
RN7SL851P	misc_RNA	ENSG00000240718	-0,321304767	0,194656855	0,287472069
AC011529.1	protein_coding	ENSG00000065989	-0,151315825	0,194727294	0,287555389
RGCC	protein_coding	ENSG00000102760	-0,369981417	0,194741946	0,287556322
AL133227.1	antisense	ENSG00000273828	0,336070224	0,194785964	0,287598848
RPS5	protein_coding	ENSG00000083845	-0,104376292	0,194798791	0,287598848
PTPN1	protein_coding	ENSG00000196396	0,053521652	0,194869191	0,28764067

RAB35	protein_coding	ENSG00000111737	-0,067793164	0,194866178	0,28764067
BNIP1	protein_coding	ENSG00000113734	-0,10461439	0,194841532	0,28764067
AL590714.1	antisense	ENSG00000224985	0,376868938	0,194990953	0,287799686
SOS1	protein_coding	ENSG00000115904	-0,084243816	0,195019118	0,287820545
NCK1-DT	antisense	ENSG00000239213	0,189328406	0,19503529	0,287823701
FAM24B	protein_coding	ENSG00000213185	-0,195703015	0,195134561	0,287949481
GPBP1	protein_coding	ENSG00000062194	0,057229074	0,195362347	0,288264873
C8orf49	lincRNA	ENSG00000255394	0,376595133	0,195413947	0,288320269
SIX3	protein_coding	ENSG00000138083	-0,197012545	0,195532692	0,288474717
AC073115.1	lincRNA	ENSG00000229628	0,377584	0,195774448	0,288810611
ZNF658B	transcribed_unprocesse	ENSG00000198416	0,215507748	0,195800845	0,288828778
RABL2A	protein_coding	ENSG00000144134	0,227619005	0,195867874	0,288906875
SLC16A1-AS1	antisense	ENSG00000226419	0,118061308	0,195921019	0,288943705
MAGIX	protein_coding	ENSG00000269313	0,248606795	0,195910349	0,288943705
ADAM21	protein_coding	ENSG00000139985	-0,257929674	0,195964907	0,28898765
AC096677.1	antisense	ENSG00000224536	0,349146855	0,196017746	0,289020629
PNPLA7	protein_coding	ENSG00000130653	0,261001982	0,196019988	0,289020629
SQOR	protein_coding	ENSG00000137767	0,090635295	0,196029545	0,289020629
AGAP9	protein_coding	ENSG00000204172	0,203895712	0,196171902	0,289188939
TMED9	protein_coding	ENSG00000184840	0,078024112	0,196159256	0,289188939
AC079416.1	transcribed_processed_	ENSG00000261056	-0,363719727	0,196267905	0,289309669
ZDHHC17	protein_coding	ENSG00000186908	0,085336988	0,196292291	0,289324822
AC110769.2	lincRNA	ENSG00000270019	0,309365016	0,196439865	0,289521531
MSANTD4	protein_coding	ENSG00000170903	0,067316899	0,196575585	0,289700744
AC105137.2	sense_intronic	ENSG00000276744	-0,352972162	0,196653342	0,289794513
TMEM238	protein_coding	ENSG00000233493	0,375672514	0,196790092	0,289975198
CHAC2	protein_coding	ENSG00000143942	-0,137829069	0,196849075	0,290041274
CAPN14	protein_coding	ENSG00000214711	0,338677987	0,196950884	0,290087083
ZC2HC1C	protein_coding	ENSG00000119703	0,332812316	0,196914681	0,290087083
IL6R	protein_coding	ENSG00000160712	0,170033866	0,19694816	0,290087083
HIST3H2A	protein_coding	ENSG00000181218	-0,107955663	0,196923956	0,290087083
NDST2	protein_coding	ENSG00000166507	-0,366222589	0,196908114	0,290087083
VAPB	protein_coding	ENSG00000124164	-0,069407295	0,197099616	0,290285303
UQCRC2	protein_coding	ENSG00000140740	-0,072978328	0,197147288	0,290334665
ZNF703	protein_coding	ENSG00000183779	0,175641493	0,197245502	0,290437595
AGPAT1	protein_coding	ENSG00000204310	0,093948058	0,197234047	0,290437595
AL391650.1	protein_coding	ENSG00000236782	0,323559411	0,197293289	0,290487104
PEG10	protein_coding	ENSG00000242265	0,08137851	0,197324382	0,290512028
PPP6R2	protein_coding	ENSG00000100239	-0,070810148	0,197436865	0,290646475
DICER1-AS1	antisense	ENSG00000235706	0,312967703	0,19746084	0,290650339

AL589684.1	lincRNA	ENSG00000223811	0,214247823	0,197581149	0,290799943
AC090114.1	processed_pseudogene	ENSG00000213280	0,352525003	0,197590834	0,290799943
SNORA71A	snoRNA	ENSG00000225091	-0,162953385	0,197608226	0,290804672
MLEC	protein_coding	ENSG00000110917	0,073123666	0,19764472	0,29083751
AC010491.1	antisense	ENSG00000261360	0,375598131	0,197679815	0,290868283
LIMS2	protein_coding	ENSG00000072163	0,370555714	0,197730065	0,290921351
AC020765.2	antisense	ENSG00000275441	-0,305279792	0,197877702	0,291117687
HIST1H1PS1	unprocessed_pseudoge	ENSG00000216331	0,373695909	0,197983324	0,291231297
AC004233.3	lincRNA	ENSG00000272079	0,36583293	0,198343007	0,291739463
CDKL4	protein_coding	ENSG00000205111	0,352503321	0,198447978	0,291855608
PDP2	protein_coding	ENSG00000172840	-0,112040154	0,19845043	0,291855608
UBE2CP2	processed_pseudogene	ENSG00000265939	0,373271506	0,19848732	0,291888931
AC005332.4	lincRNA	ENSG00000274712	-0,274440947	0,198649707	0,292106788
AL021578.1	sense_intronic	ENSG00000275894	0,293725554	0,198784616	0,292284212
ASB9	protein_coding	ENSG00000102048	-0,241420141	0,19882252	0,292318988
AC003070.2	lincRNA	ENSG00000267446	0,305874788	0,198853976	0,29233048
AC003072.1	unprocessed_pseudoge	ENSG00000250318	-0,347697743	0,198858842	0,29233048
ALOX12-AS1	antisense	ENSG00000215067	0,222230491	0,198967096	0,292468654
TTC29	protein_coding	ENSG00000137473	0,362939745	0,199043533	0,29253908
AL138689.1	sense_intronic	ENSG00000274204	0,348809282	0,199034505	0,29253908
TUBB8	protein_coding	ENSG00000261456	0,31897849	0,199092037	0,2925894
DDX25	protein_coding	ENSG00000109832	0,322151637	0,199127624	0,292620733
AC130650.2	lincRNA	ENSG00000276564	-0,366978061	0,199195282	0,29269649
RPS2P46	processed_pseudogene	ENSG00000189343	-0,176155568	0,199250531	0,29269649
USP34	protein_coding	ENSG00000115464	0,077665982	0,199238791	0,29269649
SPINDOC	protein_coding	ENSG00000168005	-0,079561051	0,199226064	0,29269649
ABCC1	protein_coding	ENSG00000103222	-0,087179735	0,199241367	0,29269649
VN1R42P	processed_pseudogene	ENSG00000223476	-0,308448774	0,199295299	0,292741287
HRASLS	protein_coding	ENSG00000127252	-0,132004733	0,19933799	0,292783027
AC087164.1	antisense	ENSG00000266677	0,371341854	0,199444412	0,292875737
AP000251.1	lincRNA	ENSG00000237594	-0,255871616	0,19944954	0,292875737
TAS2R5	protein_coding	ENSG00000127366	0,371975556	0,199475375	0,292875737
EMC1	protein_coding	ENSG00000127463	-0,052307245	0,199493884	0,292875737
MED16	protein_coding	ENSG00000175221	-0,091873066	0,199486386	0,292875737
IGFLR1	protein_coding	ENSG00000126246	-0,366887573	0,199501069	0,292875737
AC007431.3	TEC	ENSG00000280028	0,277548724	0,199420284	0,292875737
AL034370.1	processed_pseudogene	ENSG00000214019	0,334446482	0,199544986	0,292898279
ANKRD39	protein_coding	ENSG00000213337	-0,169567712	0,199532219	0,292898279
SNRPGP2	processed_pseudogene	ENSG00000264350	-0,204791193	0,19981263	0,293249162
PTGES3P3	processed_pseudogene	ENSG00000229204	-0,352265935	0,199807431	0,293249162

ASAH1	protein_coding	ENSG00000104763	0,080383494	0,199839597	0,293267755
COPE	protein_coding	ENSG00000105669	0,10403277	0,199868888	0,293289754
RF00019	misc_RNA	ENSG00000200120	-0,366579814	0,200044342	0,293521905
AC011497.2	processed_pseudogene	ENSG00000269374	0,222856634	0,200055715	0,293521905
ANKHD1	protein_coding	ENSG00000131503	-0,220619215	0,200159937	0,293653813
MRPL33	protein_coding	ENSG00000243147	-0,09806349	0,200332775	0,293886361
IRF2BPL	protein_coding	ENSG00000119669	-0,103951026	0,200418145	0,29399057
ILKAP	protein_coding	ENSG00000132323	-0,082498567	0,200606036	0,29424514
LINC01249	lincRNA	ENSG00000231532	0,289723482	0,200626213	0,294253692
SCARNA9	antisense	ENSG00000254911	0,274651523	0,200858637	0,294567274
HMGB1P1	processed_pseudogene	ENSG00000124097	-0,369557889	0,200871341	0,294567274
IQUB	protein_coding	ENSG00000164675	0,355341639	0,200883104	0,294567274
AC007383.2	lincRNA	ENSG00000227946	0,184855741	0,200988575	0,294700863
CYP51A1P3	processed_pseudogene	ENSG00000215223	0,34854944	0,201170892	0,294947101
FO393418.1	lincRNA	ENSG00000223695	0,364784578	0,201221704	0,295000511
AC122710.2	lincRNA	ENSG00000272071	-0,36215718	0,201261934	0,295038401
ODF3B	protein_coding	ENSG00000177989	0,258111197	0,201312079	0,295090818
AC234775.2	processed_pseudogene	ENSG00000237682	0,344044021	0,201391107	0,295185565
AP001350.2	TEC	ENSG00000280010	0,334747495	0,201424559	0,295213499
AC009097.4	antisense	ENSG00000261513	0,34452695	0,201516532	0,295327193
RAB28	protein_coding	ENSG00000157869	-0,085907543	0,201578246	0,295375425
PTRHD1	protein_coding	ENSG00000184924	-0,113573186	0,20156712	0,295375425
CTSB	protein_coding	ENSG00000164733	0,054628287	0,201624945	0,295422747
UHRF1BP1	protein_coding	ENSG00000065060	-0,080991023	0,201651918	0,295441163
AC110769.1	lincRNA	ENSG00000243179	0,316484505	0,201683804	0,295466773
SAT2	protein_coding	ENSG00000141504	0,076040121	0,201921961	0,295794544
MTA1	protein_coding	ENSG00000182979	-0,071701644	0,20195586	0,295823075
DYNLRB2	protein_coding	ENSG00000168589	0,361856383	0,202309462	0,296298705
RPS10P3	processed_pseudogene	ENSG00000217716	-0,351549063	0,202466246	0,296502907
NDUFAF4	protein_coding	ENSG00000123545	-0,099104479	0,202477802	0,296502907
RPL10P3	processed_pseudogene	ENSG00000230734	-0,304506022	0,202497496	0,2965037
PFN4	protein_coding	ENSG00000176732	0,230021545	0,20251746	0,2965037
RPL12	protein_coding	ENSG00000197958	-0,086165022	0,202521713	0,2965037
AC068025.2	lincRNA	ENSG00000266111	0,309665557	0,202569078	0,296551877
TMEM117	protein_coding	ENSG00000139173	0,168309785	0,202634873	0,296627024
ELAC1	protein_coding	ENSG00000141642	0,153526552	0,202681545	0,296674171
ZNF705E	protein_coding	ENSG00000214534	0,310869343	0,202894559	0,296964776
AC024940.3	processed_pseudogene	ENSG00000243517	-0,328835847	0,202991962	0,297086137
AL139094.1	processed_pseudogene	ENSG00000220685	-0,315103022	0,203105034	0,297230413
AC113385.3	unprocessed_pseudoge	ENSG00000275374	0,315923542	0,203131213	0,297247515

SIPA1L3	protein_coding	ENSG00000105738	-0,110520128	0,20322364	0,29736155
RNU6-343P	snRNA	ENSG00000201579	-0,326464295	0,203381121	0,297570751
RBMXP2	processed_pseudogene	ENSG00000215210	-0,277583275	0,203417823	0,297603018
AC092072.1	protein_coding	ENSG00000178386	0,285480091	0,203432195	0,297603018
UROD	protein_coding	ENSG00000126088	-0,085870728	0,203450949	0,297609226
VCP	protein_coding	ENSG00000165280	-0,048348633	0,203584717	0,297783665
VPS45	protein_coding	ENSG00000136631	0,069980758	0,203756117	0,298013118
SPHKAP	protein_coding	ENSG00000153820	0,370493555	0,203794336	0,298026512
TMEM104	protein_coding	ENSG00000109066	0,118722794	0,20378867	0,298026512
ASCC1	protein_coding	ENSG00000138303	0,090870971	0,203945204	0,298225875
PCDHGC3	protein_coding	ENSG00000240184	-0,237690597	0,204172337	0,298536724
MAGED2	protein_coding	ENSG00000102316	0,087909078	0,204278861	0,298671189
AL359091.5	sense_intronic	ENSG00000273186	-0,311602877	0,204325403	0,298717943
GLO1	protein_coding	ENSG00000124767	-0,071072106	0,204490963	0,298938678
UROS	protein_coding	ENSG00000188690	-0,081801929	0,20489252	0,299504353
RASD1	protein_coding	ENSG00000108551	0,361280574	0,204985952	0,299598223
AP006545.1	sense_overlapping	ENSG00000260949	-0,366226592	0,204984089	0,299598223
MARCH2	protein_coding	ENSG00000099785	0,088619565	0,205044348	0,299662218
MROH1	protein_coding	ENSG00000179832	0,128914898	0,205071364	0,299680348
STX8	protein_coding	ENSG00000170310	0,082079233	0,205107383	0,299711629
AC012370.1	sense_intronic	ENSG00000232693	0,302826988	0,205317304	0,299997001
RPL23P8	processed_pseudogene	ENSG00000219451	-0,36387596	0,205414131	0,300109895
KLRG1	protein_coding	ENSG00000139187	-0,279717415	0,205423833	0,300109895
KIN	protein_coding	ENSG00000151657	0,084136686	0,205509917	0,300198293
INTS6	protein_coding	ENSG00000102786	-0,054051671	0,205513614	0,300198293
ST3GAL6	protein_coding	ENSG00000064225	-0,130314293	0,205530071	0,300200951
C1QTNF12	protein_coding	ENSG00000184163	0,327486518	0,205686785	0,300408458
NTAN1	protein_coding	ENSG00000157045	-0,082829036	0,205721207	0,300437337
AC022007.1	lincRNA	ENSG00000206567	-0,327641352	0,205784413	0,300508246
AC009949.1	TEC	ENSG00000279964	0,308703537	0,205830084	0,300553541
PIGL	protein_coding	ENSG00000108474	-0,084483634	0,205971986	0,300739334
SCARNA21	scaRNA	ENSG00000252835	-0,21924803	0,206062579	0,300850192
RDH10	protein_coding	ENSG00000121039	-0,086068118	0,206208413	0,301041679
AC024940.2	transcribed_unprocesse	ENSG00000177359	0,351635275	0,206260242	0,301095912
CATIP-AS1	processed_transcript	ENSG00000225062	0,347386046	0,20637121	0,301236461
CIZ1	protein_coding	ENSG00000148337	0,071042089	0,206602401	0,301552466
RETREG1	protein_coding	ENSG00000154153	0,144537428	0,206617237	0,30155266
WDR81	protein_coding	ENSG00000167716	0,131888897	0,206678347	0,301620385
STIM1	protein_coding	ENSG00000167323	0,083348547	0,206735559	0,301682412
CSPG4P10	transcribed_unprocesse	ENSG00000276710	-0,344448565	0,206850046	0,301828005

PRRC2B	protein_coding	ENSG00000130723	-0,104137106	0,206873162	0,301840261
PDXP	protein_coding	ENSG00000241360	-0,311178774	0,206891305	0,30184526
Z94721.2	processed_transcript	ENSG00000272980	-0,328477702	0,206994004	0,301973613
PLBD2	protein_coding	ENSG00000151176	0,126212621	0,207267813	0,302330051
FUCA2	protein_coding	ENSG00000001036	-0,059797255	0,207327429	0,302395503
PPARD	protein_coding	ENSG00000112033	-0,097321193	0,207560573	0,302714026
AC003002.3	protein_coding	ENSG00000269533	-0,300867943	0,207771838	0,303000597
AC079781.5	processed_transcript	ENSG00000284707	0,329680375	0,207827353	0,303060007
RN7SKP79	misc_RNA	ENSG00000200243	-0,3040521	0,207960262	0,30323226
AC009090.1	sense_intronic	ENSG00000260038	-0,322486302	0,20825727	0,303643748
SULT2B1	protein_coding	ENSG00000088002	0,248250133	0,208334827	0,303735236
LMAN2L	protein_coding	ENSG00000114988	-0,087804389	0,208452908	0,30386419
AP001029.2	antisense	ENSG00000267199	-0,364220581	0,208655011	0,304136925
AC129492.5	lincRNA	ENSG00000266824	0,343143164	0,208669664	0,304136925
AL513329.1	lincRNA	ENSG00000227740	0,347462002	0,208686754	0,30414022
AC107375.1	antisense	ENSG00000259891	-0,362712833	0,208723364	0,304150352
AL359697.1	sense_intronic	ENSG00000279623	-0,328631979	0,208710691	0,304150352
UHRF2	protein_coding	ENSG00000147854	-0,060472747	0,209165412	0,304751197
AC083964.1	antisense	ENSG00000272293	0,348300404	0,209492213	0,30520566
HSPA8P4	processed_pseudogene	ENSG00000248610	-0,342740541	0,209643149	0,305403863
S100A14	protein_coding	ENSG00000189334	0,344491545	0,209784698	0,305588364
TBRG1	protein_coding	ENSG00000154144	0,072624319	0,209800751	0,305590043
BX284668.6	lincRNA	ENSG00000272426	0,340953697	0,209842057	0,305628503
ACADM	protein_coding	ENSG00000117054	0,080222838	0,209879138	0,305660805
AF064860.1	sense_overlapping	ENSG00000225330	0,363765542	0,209916798	0,305693945
CEP104	protein_coding	ENSG00000116198	0,092007901	0,210103917	0,305922997
HSD11B2	protein_coding	ENSG00000176387	-0,343892395	0,210098721	0,305922997
RTEL1	protein_coding	ENSG00000258366	-0,25574737	0,210159436	0,305982114
AC098935.2	processed_pseudogene	ENSG00000271680	0,350478411	0,210249728	0,306091846
AL078590.3	lincRNA	ENSG00000232310	-0,131707556	0,210282862	0,306096628
RN7SL546P	misc_RNA	ENSG00000239224	-0,346910167	0,21027353	0,306096628
LDHAP7	processed_pseudogene	ENSG00000235847	-0,3548605	0,210380834	0,306217508
PLGRKT	protein_coding	ENSG00000107020	-0,103929924	0,210417893	0,306249715
GPR4	protein_coding	ENSG00000177464	0,3538809	0,210681025	0,306609115
ATP8B2	protein_coding	ENSG00000143515	-0,091578342	0,210694728	0,306609115
HSPE1P2	processed_pseudogene	ENSG00000258645	-0,320493231	0,21086484	0,306834896
TRIM46	protein_coding	ENSG00000163462	-0,201325634	0,210885014	0,306842482
AC010226.1	antisense	ENSG00000249249	-0,36062116	0,210916153	0,306845899
TOPORS	protein_coding	ENSG00000197579	-0,074494876	0,210917284	0,306845899
AL031728.1	antisense	ENSG00000231105	0,325434376	0,210953333	0,306854813

ALG12	protein_coding	ENSG00000182858	0,078900445	0,210946884	0,306854813
AL358178.1	processed_pseudogene	ENSG00000216813	0,323974484	0,211020271	0,306908648
DIS3L2	protein_coding	ENSG00000144535	0,078438728	0,211005942	0,306908648
RF01241	snoRNA	ENSG00000238390	-0,355361986	0,211052096	0,306933169
S100P	protein_coding	ENSG00000163993	0,334305763	0,211145404	0,307047096
TESC	protein_coding	ENSG00000088992	-0,352894415	0,211163378	0,307051462
U73169.1	sense_intronic	ENSG00000213600	-0,320421309	0,211193684	0,30707376
AL354872.2	lincRNA	ENSG00000271992	0,312235731	0,211671418	0,307746565
WBP2NL	protein_coding	ENSG00000183066	0,317915585	0,211741888	0,307805382
FKRP	protein_coding	ENSG00000181027	0,105734724	0,211740109	0,307805382
F8A1	protein_coding	ENSG00000277203	-0,247646475	0,211784405	0,307823552
AC009053.1	transcribed_unprocesse	ENSG00000214331	0,148858178	0,211776342	0,307823552
CLYBL	protein_coding	ENSG00000125246	-0,193823951	0,211882346	0,307944085
SRP9P1	processed_pseudogene	ENSG00000180581	-0,238836698	0,211909032	0,307961046
INE1	sense_intronic	ENSG00000224975	-0,326639632	0,211998717	0,308069554
CC2D2B	protein_coding	ENSG00000188649	0,307145884	0,212133243	0,308221367
AC005593.1	TEC	ENSG00000279584	0,360215749	0,212120085	0,308221367
NPM1P24	processed_pseudogene	ENSG00000215086	-0,312309813	0,212527421	0,308772219
AL691403.2	lincRNA	ENSG00000273565	0,322962087	0,212565021	0,308783099
TNFRSF21	protein_coding	ENSG00000146072	0,0783801	0,212561189	0,308783099
IFITM3	protein_coding	ENSG00000142089	0,093278975	0,212614579	0,308833217
SNAP25	protein_coding	ENSG00000132639	-0,169221637	0,212633511	0,308838844
AL049795.1	antisense	ENSG00000224066	0,354700338	0,212649632	0,308840388
AC012213.4	lincRNA	ENSG00000271830	0,335826792	0,212710392	0,308885241
NET1	protein_coding	ENSG00000173848	0,054930067	0,212710636	0,308885241
AC092162.3	TEC	ENSG00000279205	0,359209021	0,212798901	0,308991538
AC108463.1	processed_pseudogene	ENSG00000227992	-0,194695177	0,21286796	0,309069932
MFGE8	protein_coding	ENSG00000140545	0,080424002	0,212890003	0,309080057
AC004477.1	processed_transcript	ENSG00000263412	0,231733001	0,212926351	0,309110947
SNAPC4	protein_coding	ENSG00000165684	0,124903298	0,213029337	0,309238567
G6PC3	protein_coding	ENSG00000141349	-0,101286867	0,213184867	0,309442436
AJM1	protein_coding	ENSG00000232434	-0,172196194	0,213243178	0,309505172
EDRF1-DT	lincRNA	ENSG00000224023	0,340845343	0,213261838	0,309510352
RPL28	protein_coding	ENSG00000108107	0,073421775	0,213322062	0,309575851
AL359921.1	antisense	ENSG00000230325	-0,346016203	0,213706329	0,310111561
AC090124.3	processed_pseudogene	ENSG00000255281	0,31822228	0,21372639	0,310118731
AL354714.2	processed_pseudogene	ENSG00000213080	-0,214526262	0,213789481	0,310122517
BET1	protein_coding	ENSG00000105829	0,11005104	0,213784772	0,310122517
BRD1	protein_coding	ENSG00000100425	-0,091339169	0,213747565	0,310122517
SEPT7P9	transcribed_unprocesse	ENSG00000120555	-0,353350552	0,213761153	0,310122517

DISP3	protein_coding	ENSG00000204624	0,318615327	0,213844627	0,310180572
AC004771.4	antisense	ENSG00000262429	0,342101146	0,213876567	0,310204964
AL359513.1	lincRNA	ENSG00000278238	0,269483702	0,213993413	0,310336909
MYL2	protein_coding	ENSG00000111245	0,342796551	0,213997801	0,310336909
AC055764.2	lincRNA	ENSG00000273375	-0,355083203	0,214169739	0,310564291
SNTB2	protein_coding	ENSG00000168807	-0,084003285	0,214211416	0,310602767
SCML2P2	processed_pseudogene	ENSG00000271105	-0,319388345	0,214255871	0,310645264
NKD1	protein_coding	ENSG00000140807	-0,357349061	0,214302188	0,310690455
RAB18	protein_coding	ENSG00000099246	0,080561748	0,214353231	0,310742491
TM9SF2	protein_coding	ENSG00000125304	-0,052748006	0,214528017	0,310973894
AL513327.1	antisense	ENSG00000225313	0,357775388	0,214569434	0,31101195
UGDH-AS1	antisense	ENSG00000249348	0,258691527	0,214612946	0,311053036
GMPPB	protein_coding	ENSG00000173540	0,084308019	0,214633033	0,311060168
PTPRA	protein_coding	ENSG00000132670	-0,066474565	0,214675964	0,311100404
AC010240.3	lincRNA	ENSG00000272023	0,320949363	0,214693706	0,311104134
MTERF1	protein_coding	ENSG00000127989	-0,118887689	0,215244924	0,311880847
AL606834.2	sense_intronic	ENSG00000270062	-0,31821466	0,215297213	0,311934574
THAP8	protein_coding	ENSG00000161277	0,179025623	0,215389353	0,312046029
TIMM44	protein_coding	ENSG00000104980	0,060982193	0,215631854	0,312375288
ARHGEF7	protein_coding	ENSG00000102606	-0,070058087	0,215853687	0,312674562
AGAP5	protein_coding	ENSG00000172650	-0,285770903	0,215904323	0,312725823
CEL	protein_coding	ENSG00000170835	0,305458119	0,215975398	0,312805878
HOXA4	protein_coding	ENSG00000197576	-0,338961744	0,215990095	0,312805878
SERPINB7	protein_coding	ENSG00000166396	0,07147151	0,216010077	0,312812728
LY6E-DT	lincRNA	ENSG00000247317	-0,243059997	0,216177385	0,313010813
RF00134	snoRNA	ENSG00000281780	-0,288348275	0,216173496	0,313010813
AC090772.3	lincRNA	ENSG00000265750	-0,358382838	0,216212436	0,313039464
PARG	protein_coding	ENSG00000227345	-0,054746571	0,216227787	0,313039592
AL132777.1	processed_pseudogene	ENSG00000258445	-0,347695758	0,216329618	0,313164911
ERV3-1	protein_coding	ENSG00000213462	0,245294649	0,216365107	0,313194181
AC005632.2	sense_intronic	ENSG00000261596	-0,335055955	0,216572147	0,313471753
TEKT2	protein_coding	ENSG00000092850	0,280529573	0,216715039	0,313656443
RNH1	protein_coding	ENSG00000023191	-0,079545076	0,216747855	0,313681803
HELLPAR	macro_lncRNA	ENSG00000281344	0,140379691	0,216847689	0,313804142
RPS26P21	processed_pseudogene	ENSG00000242829	-0,280573324	0,216910486	0,313828588
AP5B1	protein_coding	ENSG00000254470	-0,114101896	0,216907228	0,313828588
COL6A1	protein_coding	ENSG00000142156	-0,144200883	0,216893179	0,313828588
ARF1	protein_coding	ENSG00000143761	-0,05305752	0,21697268	0,313896429
LINC01004	antisense	ENSG00000228393	0,318409112	0,217057494	0,313952695
ZNF513	protein_coding	ENSG00000163795	-0,119796642	0,217044618	0,313952695

AL512408.1	antisense	ENSG00000260063	0,202576363	0,217075703	0,31395689
KCNAB2	protein_coding	ENSG00000069424	0,117508075	0,217106492	0,31397928
RBBP4P1	processed_pseudogene	ENSG00000249485	-0,355222578	0,217203381	0,314075109
DHFR	protein_coding	ENSG00000228716	-0,06878611	0,217190712	0,314075109
AC005786.3	antisense	ENSG00000267436	-0,335597611	0,217313406	0,314212053
AC002480.2	antisense	ENSG00000232949	0,351229586	0,217334908	0,31422099
RPS8P10	unprocessed_pseudoge	ENSG00000223877	-0,337036691	0,21738741	0,314274743
LINC00852	antisense	ENSG00000231177	-0,315474735	0,217468828	0,31437029
AC090286.2	transcribed_processed_	ENSG00000235672	0,340344074	0,21751958	0,314421494
NIPSNAP3B	protein_coding	ENSG00000165028	0,31043699	0,217799518	0,314803955
CHCHD2P6	processed_pseudogene	ENSG00000235084	-0,353161643	0,218022774	0,315104438
MORC2	protein_coding	ENSG00000133422	-0,070882158	0,21808055	0,315165732
TIMP2	protein_coding	ENSG00000035862	0,076339245	0,218155819	0,31519422
ZNF384	protein_coding	ENSG00000126746	-0,079577213	0,218161733	0,31519422
POLR2K	protein_coding	ENSG00000147669	-0,09938088	0,218124681	0,31519422
AL158835.2	sense_intronic	ENSG00000234134	-0,352298425	0,218149612	0,31519422
ANKRD17	protein_coding	ENSG00000132466	0,079386688	0,218308977	0,315384737
GLRX	protein_coding	ENSG00000173221	0,066012388	0,218427704	0,315534034
MRS2	protein_coding	ENSG00000124532	-0,082628688	0,21858223	0,31573502
AC092287.1	antisense	ENSG00000260352	0,351824478	0,218616663	0,315761416
HSP90B2P	processed_pseudogene	ENSG00000259706	-0,339620223	0,218631295	0,315761416
AL022324.2	processed_pseudogene	ENSG00000231466	0,347704734	0,2188437	0,31604593
MUC15	protein_coding	ENSG00000169550	0,333771777	0,218916534	0,316108484
AL139287.1	sense_intronic	ENSG00000240731	-0,336892161	0,21891784	0,316108484
EPGN	protein_coding	ENSG00000182585	-0,206096849	0,218972289	0,316164847
RAB22A	protein_coding	ENSG00000124209	0,070223306	0,219117547	0,316330041
HAGH	protein_coding	ENSG00000063854	0,077161685	0,21928165	0,316544667
COX18	protein_coding	ENSG00000163626	-0,099243068	0,219403303	0,31669799
AC136475.1	antisense	ENSG00000251661	0,351407404	0,219452702	0,316747004
ICOSLG	protein_coding	ENSG00000160223	0,331783709	0,219590842	0,316924085
CETN2	protein_coding	ENSG00000147400	-0,086943064	0,219630901	0,316959596
TECR	protein_coding	ENSG00000099797	0,078974232	0,219660517	0,316980033
AC027271.1	lincRNA	ENSG00000272576	-0,328380178	0,219680626	0,316986749
AC093732.1	antisense	ENSG00000233845	0,352574052	0,21974948	0,317063794
AC127521.1	antisense	ENSG00000262823	0,307095974	0,219783156	0,317090076
AC019117.2	lincRNA	ENSG00000236318	0,315953277	0,219868369	0,317190704
TASP1	protein_coding	ENSG00000089123	0,125469112	0,220038618	0,317391663
PET100	protein_coding	ENSG00000229833	-0,200120334	0,220026658	0,317391663
SLFNL1-AS1	antisense	ENSG00000281207	0,223307477	0,220054612	0,317392411
PNLDC1	protein_coding	ENSG00000146453	0,307694249	0,220144998	0,31750045

VPS4B	protein_coding	ENSG00000119541	-0,059076507	0,220278852	0,317671161
ACOT4	protein_coding	ENSG00000177465	0,305992953	0,220447335	0,317891784
AC068790.3	sense_intronic	ENSG00000269997	0,350133633	0,220467991	0,31789922
BNC2	protein_coding	ENSG00000173068	0,106583784	0,220685196	0,318190044
ECHDC1	protein_coding	ENSG00000093144	-0,076269779	0,221070704	0,318723474
AC069528.2	TEC	ENSG00000279320	0,33319041	0,221097885	0,318740256
SEMA6A-AS1	antisense	ENSG00000248445	0,351902056	0,221120548	0,318750523
IDH3G	protein_coding	ENSG00000067829	-0,068567963	0,221373279	0,319089768
TAF1A	protein_coding	ENSG00000143498	-0,096247135	0,221387001	0,319089768
AKAP13	protein_coding	ENSG00000170776	0,086892077	0,221414186	0,319106525
COX7B	protein_coding	ENSG00000131174	-0,088202624	0,22148718	0,319189297
IFT52	protein_coding	ENSG00000101052	-0,087851811	0,221579658	0,3192777
AL122020.1	processed_pseudogene	ENSG00000213315	0,348677059	0,221629372	0,3193269
AC092849.1	antisense	ENSG00000240211	0,349170934	0,221706133	0,31941506
RN7SKP180	misc_RNA	ENSG00000200783	0,335768668	0,221842639	0,319589277
ANKAR	protein_coding	ENSG00000151687	0,152454671	0,22190406	0,319655308
WASH6P	transcribed_unprocesse	ENSG00000182484	-0,149380475	0,221982215	0,319745434
RPL37P23	processed_pseudogene	ENSG00000243680	0,315498133	0,222068791	0,319847676
CRYZL1	protein_coding	ENSG00000205758	-0,066316664	0,222105061	0,31986067
AC108449.2	sense_intronic	ENSG00000259366	-0,311279327	0,222109004	0,31986067
HM13-AS1	antisense	ENSG00000230613	0,332724358	0,22238246	0,320231992
AP000769.2	lincRNA	ENSG00000270117	0,325875146	0,222508413	0,320368379
AC126323.1	transcribed_unprocesse	ENSG00000166104	0,289444995	0,222508414	0,320368379
NSD3	protein_coding	ENSG00000147548	-0,069960483	0,222557801	0,320394504
ZKSCAN8	protein_coding	ENSG00000198315	0,073585911	0,222585471	0,320411847
ZNF500	protein_coding	ENSG00000103199	-0,123617875	0,222629086	0,320452141
PTPRD-AS1	lincRNA	ENSG00000225706	0,294600366	0,222662652	0,320477964
AC098614.3	transcribed_processed_	ENSG00000271943	0,33893109	0,222718238	0,320535475
AF064860.2	lincRNA	ENSG00000231713	0,34980186	0,222831969	0,320676655
AL355297.4	antisense	ENSG00000271551	-0,336158831	0,223112332	0,321057597
PPIAP29	processed_pseudogene	ENSG00000214975	-0,253560873	0,223220151	0,321190213
NTN1	protein_coding	ENSG00000065320	-0,110754115	0,223310135	0,321297149
EFR3B	protein_coding	ENSG00000084710	-0,068575583	0,223359052	0,321344988
STXBP3	protein_coding	ENSG00000116266	0,075130822	0,223434506	0,321430997
RABEP1	protein_coding	ENSG00000029725	0,04661487	0,223467088	0,321455322
SRP72	protein_coding	ENSG00000174780	-0,070973711	0,223534502	0,321529745
NHS	protein_coding	ENSG00000188158	-0,178170067	0,223603939	0,321607069
LINC02018	lincRNA	ENSG00000272690	0,314779731	0,223784468	0,321844151
RN7SL834P	misc_RNA	ENSG00000243650	0,346099935	0,223834179	0,321893073
AP003900.1	lincRNA	ENSG00000277693	0,333642159	0,22407654	0,322219017

GPX1	polymorphic_pseudoge	ENSG00000233276	0,107148635	0,224130369	0,322273827
AC010200.1	processed_pseudogene	ENSG00000257803	-0,309774132	0,224255237	0,322430769
SETD9	protein_coding	ENSG00000155542	-0,111040144	0,224466539	0,322711953
TK2	protein_coding	ENSG00000166548	-0,0792837	0,224499752	0,32273708
SLC16A10	protein_coding	ENSG00000112394	0,28226192	0,224867956	0,323243747
RNF123	protein_coding	ENSG00000164068	0,103813574	0,225049804	0,323482478
B3GNTL1	protein_coding	ENSG00000175711	-0,07668594	0,225069525	0,323488153
COX6C	protein_coding	ENSG00000164919	-0,081973858	0,22515235	0,32358452
AL121832.2	antisense	ENSG00000273619	0,296673258	0,225211606	0,323647003
AL596202.1	antisense	ENSG00000235381	-0,268632909	0,225260658	0,323672138
CHD3	protein_coding	ENSG00000170004	0,114386688	0,225245962	0,323672138
LY6G5C	protein_coding	ENSG00000204428	0,308895362	0,225391434	0,3237744
ZSCAN23	protein_coding	ENSG00000187987	0,226739743	0,225426544	0,3237744
COX7A2	protein_coding	ENSG00000112695	-0,078124579	0,225400067	0,3237744
GLI4	protein_coding	ENSG00000250571	-0,156547578	0,225391493	0,3237744
SMARCD3	protein_coding	ENSG00000082014	-0,189505936	0,225414861	0,3237744
HAUS7	protein_coding	ENSG00000213397	-0,341073397	0,225355083	0,3237744
TRPT1	protein_coding	ENSG00000149743	0,111586082	0,225459773	0,323799449
MCPH1-AS1	antisense	ENSG00000249898	0,329965292	0,225513259	0,323853588
AP003419.2	lincRNA	ENSG00000274251	-0,33764509	0,225875037	0,324350417
LRRK2	protein_coding	ENSG00000188906	0,130123814	0,226040569	0,324519954
BBS9	protein_coding	ENSG00000122507	0,095546616	0,226033613	0,324519954
SCFD2	protein_coding	ENSG00000184178	-0,074758202	0,226037887	0,324519954
ZNF331	protein_coding	ENSG00000130844	-0,223485231	0,226106164	0,324568688
PGAM1P5	transcribed_processed_	ENSG00000257150	0,33557945	0,226090563	0,324568688
ALS2	protein_coding	ENSG00000003393	-0,061027533	0,22616942	0,324614143
GLG1	protein_coding	ENSG00000090863	-0,062977652	0,226169483	0,324614143
MISP3	protein_coding	ENSG00000141854	-0,317093236	0,226207301	0,324645704
DCDC1	protein_coding	ENSG00000170959	0,308618086	0,226379869	0,324870635
AC136469.2	TEC	ENSG00000279827	0,343640644	0,226455073	0,324955821
POPDC3	protein_coding	ENSG00000132429	0,099667784	0,226565349	0,325089584
AC006538.1	sense_intronic	ENSG00000261342	0,279626471	0,226579991	0,325089584
BANF1P3	processed_pseudogene	ENSG00000237758	0,324465179	0,226626902	0,325134146
RN7SL4P	misc_RNA	ENSG00000263740	-0,21537317	0,226931521	0,325508679
CCDC59	protein_coding	ENSG00000133773	0,085071074	0,226935573	0,325508679
EFCAB10	protein_coding	ENSG00000185055	0,166046532	0,226971386	0,32551605
AC011330.1	unprocessed_pseudoge	ENSG00000249839	0,328915974	0,226972454	0,32551605
UCN	protein_coding	ENSG00000163794	0,244335332	0,227093424	0,325666769
CAMK2N2	protein_coding	ENSG00000163888	-0,104286384	0,227242994	0,325858478
RTN3P1	processed_pseudogene	ENSG00000251333	0,315722306	0,22730726	0,325927845

RPS15A	protein_coding	ENSG00000134419	-0,098454594	0,227365389	0,325988404
AC135050.5	sense_intronic	ENSG00000262766	0,345837738	0,22739149	0,326003039
SF3A1	protein_coding	ENSG00000099995	-0,068870457	0,22743938	0,326048905
RN7SL751P	misc_RNA	ENSG00000240964	0,325749113	0,227543918	0,326175968
TATDN1P1	processed_pseudogene	ENSG00000234369	0,289495325	0,227645199	0,326298346
AL137856.1	antisense	ENSG00000231128	-0,343717991	0,227811603	0,326514045
MIR4713HG	lincRNA	ENSG00000259240	0,334044894	0,228018467	0,326761721
ZYG11B	protein_coding	ENSG00000162378	0,053579362	0,228032204	0,326761721
SCARNA11	scaRNA	ENSG00000251898	-0,324271783	0,228005287	0,326761721
AL139339.2	antisense	ENSG00000273485	-0,342326649	0,228098379	0,326833712
AL392046.1	antisense	ENSG00000230534	0,344628084	0,228281178	0,327072788
HIPK3	protein_coding	ENSG00000110422	0,064328913	0,228306467	0,327086171
AC104297.1	processed_pseudogene	ENSG00000213383	0,30134589	0,228351201	0,32712741
SCYL3	protein_coding	ENSG00000000457	0,087792305	0,228495842	0,327311756
DCUN1D1	protein_coding	ENSG00000043093	-0,081287146	0,228534215	0,327343862
LUM	protein_coding	ENSG00000139329	0,292483248	0,228700124	0,327558628
GLUD1P3	transcribed_unprocesse	ENSG00000250959	0,264618959	0,228739425	0,327592039
UBOX5	protein_coding	ENSG00000185019	0,12474814	0,228833154	0,327703393
REPIN1	protein_coding	ENSG00000214022	-0,08754328	0,228856415	0,327713822
C6orf132	protein_coding	ENSG00000188112	0,134252227	0,228952072	0,327827911
SGK2	protein_coding	ENSG00000101049	0,296028523	0,228999253	0,327872578
PLPP3	protein_coding	ENSG00000162407	-0,097511861	0,229037745	0,327904799
NPM1P46	processed_pseudogene	ENSG00000213104	0,342763623	0,229143265	0,328031656
BLACAT1	protein_coding	ENSG00000281406	-0,169506665	0,229158341	0,328031656
LINC00668	lincRNA	ENSG00000265933	-0,340582192	0,229349999	0,328283096
AL136295.2	lincRNA	ENSG00000259321	0,290888389	0,229493294	0,32846528
AC063943.1	lincRNA	ENSG00000278344	0,314148227	0,22963478	0,32864485
EBLN1	protein_coding	ENSG00000223601	0,336179526	0,229719727	0,328743483
YOD1	protein_coding	ENSG00000180667	0,084445457	0,22985482	0,32891386
CDC16	protein_coding	ENSG00000130177	0,055792341	0,230214501	0,329405569
AF196972.1	antisense	ENSG00000224292	-0,317845676	0,230294419	0,329496932
AC027796.4	antisense	ENSG00000262903	0,310377052	0,230340276	0,329539555
AC009120.1	processed_pseudogene	ENSG00000239763	0,336519156	0,230360198	0,329545069
GSDMA	protein_coding	ENSG00000167914	0,315722401	0,230422965	0,329611872
GPS1	protein_coding	ENSG00000169727	-0,070783544	0,230458454	0,329639647
AQP7P1	unprocessed_pseudoge	ENSG00000186466	0,28818108	0,23058996	0,329804748
PAIP1	protein_coding	ENSG00000172239	-0,070143784	0,230641004	0,329854752
AC097460.1	antisense	ENSG00000245322	0,320029623	0,230676022	0,329881831
AC027801.4	lincRNA	ENSG00000262837	0,321003158	0,230803389	0,330040962
EPN1	protein_coding	ENSG00000063245	-0,092172126	0,230937384	0,330209547

AC116036.2	lincRNA	ENSG00000272360	-0,328427913	0,231024337	0,33031085
AIFM2	protein_coding	ENSG00000042286	0,081143104	0,231094961	0,330382884
AC099548.2	transcribed_unprocesse	ENSG00000253882	0,336956061	0,231106935	0,330382884
AL445183.3	processed_pseudogene	ENSG00000236360	0,300835756	0,231183803	0,330469739
PSMC5	protein_coding	ENSG00000087191	-0,04622462	0,231331668	0,330658061
LINC01109	lincRNA	ENSG00000271167	0,29375048	0,231402625	0,330736435
COQ6	protein_coding	ENSG00000119723	0,070236343	0,231436669	0,330762043
AC022211.3	sense_intronic	ENSG00000265800	0,338293924	0,231564524	0,33092171
AC061999.1	TEC	ENSG00000280331	0,332017127	0,231639709	0,331006092
DBI	protein_coding	ENSG00000155368	0,077643059	0,231664959	0,331019111
TTC9C	protein_coding	ENSG00000162222	-0,088829166	0,231701435	0,331048167
PRSS16	protein_coding	ENSG00000112812	0,341213226	0,231807288	0,331176335
SEC22A	protein_coding	ENSG00000121542	0,090567329	0,231899887	0,331285553
AL009174.1	processed_pseudogene	ENSG00000227008	0,276719939	0,231930404	0,331306073
AC005520.3	sense_intronic	ENSG00000270140	-0,264955622	0,231987924	0,331365159
DUSP12	protein_coding	ENSG00000081721	-0,064968487	0,232010522	0,33137436
AL121890.4	lincRNA	ENSG00000277425	-0,268580598	0,232056672	0,331417195
COQ4	protein_coding	ENSG00000167113	-0,068312414	0,232120144	0,331484762
TMEM139	protein_coding	ENSG00000178826	0,340422053	0,232145428	0,331497788
RN7SL30P	misc_RNA	ENSG00000243845	0,301877356	0,232182372	0,331527461
NPHP1	protein_coding	ENSG00000144061	0,115043962	0,232268526	0,331608746
DNM1P38	unprocessed_pseudoge	ENSG00000259637	-0,307478262	0,232271636	0,331608746
RPL41P1	processed_pseudogene	ENSG00000227063	-0,199633594	0,232424209	0,331803475
TCEA2	protein_coding	ENSG00000171703	0,098606802	0,232466354	0,331840543
AC026803.2	lincRNA	ENSG00000267898	0,314813828	0,232551825	0,331939448
TRIM69	protein_coding	ENSG00000185880	-0,066838081	0,232588678	0,331968948
APOM	protein_coding	ENSG00000204444	0,204947265	0,23260904	0,331974908
ELOVL2-AS1	antisense	ENSG00000230314	0,290475224	0,232733929	0,332122237
SAP30BP	protein_coding	ENSG00000161526	0,047519527	0,232744657	0,332122237
RNF168	protein_coding	ENSG00000163961	-0,080780066	0,232820298	0,332207062
TTC6	protein_coding	ENSG00000139865	0,286446692	0,232924266	0,332332292
AC009237.15	lincRNA	ENSG00000273305	0,285705826	0,233005447	0,3324216
MSI1	protein_coding	ENSG00000135097	0,320042134	0,233019276	0,3324216
MKNK1	protein_coding	ENSG00000079277	0,07343611	0,2330546	0,33244887
AC026691.1	antisense	ENSG00000270021	0,296147692	0,233158376	0,332573774
ZZEF1	protein_coding	ENSG00000074755	-0,093204431	0,233183376	0,332586304
AAGAB	protein_coding	ENSG00000103591	0,056395937	0,233293334	0,332719997
H2AFZP3	processed_pseudogene	ENSG00000218502	-0,302348073	0,233428561	0,332889708
CHST15	protein_coding	ENSG00000182022	-0,134546883	0,233560957	0,333055359
AC004943.2	antisense	ENSG00000259768	0,111945015	0,23359682	0,333060187

ABCD3	protein_coding	ENSG00000117528	-0,069008265	0,233581996	0,333060187
OLMALINC	lincRNA	ENSG00000235823	-0,142166722	0,233753266	0,333260079
AL137782.1	processed_transcript	ENSG00000261553	0,230046795	0,233794537	0,333295751
LINC01970	lincRNA	ENSG00000265692	0,324863478	0,234496662	0,33427346
AC079630.1	antisense	ENSG00000225342	0,321296106	0,234536147	0,33430651
MBP	protein_coding	ENSG00000197971	-0,069266817	0,234595472	0,334367834
ZNF420	protein_coding	ENSG00000197050	0,07622268	0,234627099	0,334389674
BUD31	protein_coding	ENSG00000106245	0,073447814	0,234787842	0,334595515
BCR	protein_coding	ENSG00000186716	-0,086413584	0,234819195	0,334616945
ST3GAL1	protein_coding	ENSG00000008513	-0,12010881	0,234887138	0,334690511
ZRANB1	protein_coding	ENSG00000019995	-0,05722542	0,235021422	0,334858588
AC005759.1	antisense	ENSG00000268650	-0,336187643	0,23511669	0,334971056
ST5	protein_coding	ENSG00000166444	-0,116662735	0,235242011	0,335103045
AL606834.1	sense_intronic	ENSG00000269906	-0,210755273	0,235238061	0,335103045
DDX27	protein_coding	ENSG00000124228	-0,049427661	0,235311839	0,335157628
VGLL4	protein_coding	ENSG00000144560	-0,050988908	0,235313011	0,335157628
KRAS	protein_coding	ENSG00000133703	-0,075045186	0,235394176	0,335249951
AC112230.1	processed_pseudogene	ENSG00000271597	0,302652204	0,235659325	0,335604274
DLGAP1-AS1	antisense	ENSG00000177337	0,134167404	0,235758157	0,335693766
RN7SL396P	misc_RNA	ENSG00000244642	0,25286126	0,235771268	0,335693766
SMIM4	protein_coding	ENSG00000168273	0,108640565	0,235743445	0,335693766
NFIB	protein_coding	ENSG00000147862	-0,080100809	0,236038936	0,336051547
PTMS	protein_coding	ENSG00000159335	0,069117107	0,236255295	0,336336232
SCAND1	protein_coding	ENSG00000171222	-0,125628012	0,236366021	0,336470508
AC005005.4	sense_overlapping	ENSG00000278920	-0,304787118	0,236396212	0,336490132
AC087241.4	sense_intronic	ENSG00000274885	0,323570251	0,23661148	0,336773175
SENP1	protein_coding	ENSG00000079387	-0,060369672	0,236645607	0,336798377
AC091563.1	lincRNA	ENSG00000254343	-0,293573438	0,236679961	0,336823897
DBNL	protein_coding	ENSG00000136279	0,071303903	0,236701842	0,336831664
PARP12	protein_coding	ENSG00000059378	0,110273528	0,236942925	0,337151338
NFE2	protein_coding	ENSG00000123405	0,307574028	0,237029008	0,337250431
LINC02606	lincRNA	ENSG00000284693	-0,26275759	0,237086412	0,337308707
PHC1P1	processed_pseudogene	ENSG00000179899	0,2925901	0,23721929	0,337469569
POLM	protein_coding	ENSG00000122678	0,121736158	0,237232386	0,337469569
EEF1AKMT3	protein_coding	ENSG00000123427	-0,10399258	0,23729113	0,337529723
RNY1	misc_RNA	ENSG00000201098	-0,143837775	0,237337393	0,337572117
TWF2	protein_coding	ENSG00000247596	-0,113630729	0,23735414	0,337572526
LINC00452	lincRNA	ENSG00000229373	0,33587945	0,237591603	0,337886824
AC012456.2	antisense	ENSG00000230790	0,317618281	0,237613888	0,337895087
MKRN2	protein_coding	ENSG00000075975	-0,055634191	0,237660864	0,337938458

OLFML3	protein_coding	ENSG00000116774	-0,143277808	0,23771867	0,337997222
ABAT	protein_coding	ENSG00000183044	0,20984083	0,237862265	0,338177947
RNU6-574P	snRNA	ENSG00000206992	0,30419291	0,237933185	0,338255328
POLR2J3	protein_coding	ENSG00000168255	0,317647954	0,238023971	0,33836094
AC009075.1	lincRNA	ENSG00000261673	0,296693656	0,238089008	0,338429937
SLC38A6	protein_coding	ENSG00000139974	0,105462189	0,23827176	0,338666236
AC004803.1	antisense	ENSG00000250132	-0,171939438	0,238296743	0,338678276
FAM107A	protein_coding	ENSG00000168309	0,335849919	0,238342088	0,338716358
RPS19BP1	protein_coding	ENSG00000187051	0,081806107	0,238356567	0,338716358
LINC01116	lincRNA	ENSG00000163364	0,051873884	0,238373946	0,338717586
LINC01806	lincRNA	ENSG00000227403	-0,333501433	0,238391991	0,33871976
SIX3-AS1	lincRNA	ENSG00000236502	-0,245921647	0,238495301	0,338843074
AC002553.2	antisense	ENSG00000275413	0,331641782	0,238615934	0,338990983
ZNF614	protein_coding	ENSG00000142556	-0,085002007	0,238703745	0,339092243
CDC34	protein_coding	ENSG00000099804	0,084778533	0,238814128	0,339225554
AC027307.3	lincRNA	ENSG00000268798	-0,277270442	0,238849601	0,339252446
TRRAP	protein_coding	ENSG00000196367	-0,103564189	0,239231752	0,339771708
SAC3D1	protein_coding	ENSG00000168061	-0,089298874	0,239276117	0,339811187
AC046185.3	TEC	ENSG00000279369	-0,285743027	0,239399859	0,33996338
NPM1P27	processed_pseudogene	ENSG00000249353	-0,105350353	0,239525531	0,340118294
MRPS15	protein_coding	ENSG00000116898	-0,086962735	0,239706706	0,340351994
PARL	protein_coding	ENSG00000175193	-0,109286521	0,239810753	0,340476156
RGS16	protein_coding	ENSG00000143333	0,333203336	0,239853195	0,340512842
UFSP2	protein_coding	ENSG00000109775	0,072491186	0,239871648	0,34051547
PIK3R2	protein_coding	ENSG00000105647	-0,303959423	0,239962204	0,340620445
MIR22HG	lincRNA	ENSG00000186594	0,11520524	0,240090827	0,340779436
ATP5F1E	protein_coding	ENSG00000124172	0,06545468	0,240107514	0,340779538
LINC00921	lincRNA	ENSG00000281005	-0,328923467	0,24015912	0,340829196
AC099066.2	processed_transcript	ENSG00000227496	0,333756642	0,240328304	0,341045701
NUTM2A	protein_coding	ENSG00000184923	0,333934734	0,240534213	0,341314287
AC012464.2	sense_intronic	ENSG00000258303	0,332995132	0,240615973	0,341406683
RPS4XP3	processed_pseudogene	ENSG00000214541	-0,278873655	0,240670983	0,341437495
LGALS9B	protein_coding	ENSG00000170298	-0,3119454	0,24066175	0,341437495
MAD1L1	protein_coding	ENSG00000002822	0,076098557	0,240694018	0,341446557
ANXA7	protein_coding	ENSG00000138279	-0,05487405	0,240730809	0,341475129
WDR83	protein_coding	ENSG00000123154	-0,112781024	0,241020346	0,341862192
EMC4	protein_coding	ENSG00000128463	0,057343438	0,241140925	0,341996245
MAP2K4P1	transcribed_processed_	ENSG00000269904	0,306033392	0,241148205	0,341996245
PAN3	protein_coding	ENSG00000152520	-0,082130542	0,241171698	0,342005914
NVL	protein_coding	ENSG00000143748	0,055719324	0,241356892	0,342244874

AP1AR	protein_coding	ENSG00000138660	0,064184359	0,241414662	0,342303127
ATOX1	protein_coding	ENSG00000177556	0,077953898	0,241823301	0,342858835
DHDH	protein_coding	ENSG00000104808	0,282028592	0,242059238	0,343169625
HIF1AN	protein_coding	ENSG00000166135	-0,064288287	0,242214666	0,343366242
VTRNA1-2	misc_RNA	ENSG00000202111	-0,332171625	0,242411393	0,343615419
RAB40A	protein_coding	ENSG00000172476	0,319370879	0,242423945	0,343615419
SLC35A2	protein_coding	ENSG00000102100	-0,073629956	0,242651062	0,34391357
AC073415.1	processed_pseudogene	ENSG00000227470	-0,331255084	0,242777855	0,3440695
ZNF606	protein_coding	ENSG00000166704	-0,090602562	0,242962528	0,34430743
CNIH2	protein_coding	ENSG00000174871	0,33249269	0,243030989	0,344367818
FTCDNL1	protein_coding	ENSG00000226124	0,134466724	0,243038721	0,344367818
DCAF12	protein_coding	ENSG00000198876	0,071158819	0,243072633	0,344392077
AC110994.2	processed_pseudogene	ENSG00000282978	0,313775789	0,243182514	0,34452396
PRDM4	protein_coding	ENSG00000110851	-0,058111085	0,243356694	0,344746912
AL359851.1	lincRNA	ENSG00000260585	0,329736971	0,243542111	0,344961926
AC083967.1	lincRNA	ENSG00000254337	0,28540058	0,243538318	0,344961926
ZFHX2-AS1	processed_transcript	ENSG00000157306	0,328522086	0,243566812	0,34497309
LINC01356	lincRNA	ENSG00000215866	0,331562255	0,243639495	0,345052206
AC079598.1	lincRNA	ENSG00000257940	0,263908217	0,243734076	0,345162321
EIF3C	protein_coding	ENSG00000184110	-0,150471519	0,243779244	0,34520245
TMEM183B	processed_pseudogene	ENSG00000224831	-0,316436451	0,243921691	0,345380317
DNASE2	protein_coding	ENSG00000105612	0,073467576	0,24422112	0,34578042
PEX12	protein_coding	ENSG00000108733	-0,09199433	0,244252385	0,345800815
UIMC1	protein_coding	ENSG00000087206	0,059497832	0,244302388	0,345847735
AL136982.3	sense_intronic	ENSG00000237280	-0,24655974	0,244423802	0,345995733
SLC39A4	protein_coding	ENSG00000147804	-0,156530583	0,244543806	0,346141716
RPL6P27	transcribed_processed_	ENSG00000235552	-0,144144527	0,244595836	0,34619147
TXNP4	processed_pseudogene	ENSG00000262519	-0,29649662	0,244696594	0,346310181
KLHL41	protein_coding	ENSG00000239474	0,320878327	0,244845656	0,346497234
AC108751.4	lincRNA	ENSG00000243885	0,321089854	0,24490202	0,346553086
SAP30	protein_coding	ENSG00000164105	0,081961894	0,245005413	0,34665156
ITIH4	protein_coding	ENSG00000055955	-0,28369995	0,245003948	0,34665156
ANXA2P1	processed_pseudogene	ENSG00000213406	-0,293013417	0,245360668	0,347130255
PAGE1	protein_coding	ENSG00000068985	0,307835298	0,245445151	0,347225828
LRRC20	protein_coding	ENSG00000172731	0,091223467	0,245804126	0,347709679
CHPT1	protein_coding	ENSG00000111666	-0,057210044	0,245879849	0,347792807
EXD3	protein_coding	ENSG00000187609	0,171300786	0,246104333	0,348086329
RDH5	protein_coding	ENSG00000135437	0,32591843	0,246199065	0,348196305
AP001527.2	lincRNA	ENSG00000277459	0,324677041	0,246255597	0,348228233
AK8	protein_coding	ENSG00000165695	0,321611231	0,246251552	0,348228233

SIRPA	protein_coding	ENSG00000198053	-0,118140468	0,246488996	0,34853425
SHTN1	protein_coding	ENSG00000187164	-0,093800705	0,246704285	0,34881462
IL7	protein_coding	ENSG00000104432	0,182794759	0,246743769	0,348846398
LINC02243	lincRNA	ENSG00000236091	0,317981773	0,246881218	0,348968558
PPP1R2B	protein_coding	ENSG00000231989	0,266222106	0,246864582	0,348968558
NDFIP2	protein_coding	ENSG00000102471	0,080003201	0,2468559	0,348968558
TMPPE	protein_coding	ENSG00000188167	-0,157723335	0,247098253	0,34925127
PURPL	lincRNA	ENSG00000250337	-0,099104019	0,247199522	0,349370328
CLIP1-AS1	lincRNA	ENSG00000257097	0,311695846	0,247353707	0,349564152
CALM2P3	processed_pseudogene	ENSG00000215482	0,284956499	0,247412911	0,349623729
AC021483.2	lincRNA	ENSG00000261229	-0,295265514	0,247447454	0,349648452
LINC02228	lincRNA	ENSG00000251273	0,190262151	0,247499467	0,34969516
CRTC2	protein_coding	ENSG00000160741	0,097828488	0,247514609	0,34969516
THOC7	protein_coding	ENSG00000163634	-0,080745635	0,247873077	0,350153373
RPL34-AS1	lincRNA	ENSG00000234492	0,291005891	0,247903902	0,350172799
HOPX	protein_coding	ENSG00000171476	-0,311240831	0,248131049	0,350469514
FRMD8	protein_coding	ENSG00000126391	0,102835807	0,248272372	0,350644976
AC008147.1	antisense	ENSG00000257256	-0,297275113	0,248634395	0,351132096
FTH1P16	processed_pseudogene	ENSG00000227376	0,324416597	0,248793395	0,351302758
ZNF93	protein_coding	ENSG00000184635	0,285032092	0,248778755	0,351302758
IARS	protein_coding	ENSG00000196305	-0,044763993	0,248806624	0,351302758
RMND5B	protein_coding	ENSG00000145916	-0,080593157	0,248992178	0,35154055
AC007881.3	lincRNA	ENSG00000272735	0,324977648	0,249158787	0,351751565
CPPED1	protein_coding	ENSG00000103381	-0,066175791	0,249181372	0,351759236
AL596223.1	antisense	ENSG00000229261	0,121953125	0,249244768	0,351803481
LRP10	protein_coding	ENSG00000197324	0,080303128	0,24924702	0,351803481
EPRS	protein_coding	ENSG00000136628	-0,051220676	0,249470932	0,352071067
LEMD2	protein_coding	ENSG00000161904	-0,063128489	0,249470827	0,352071067
HINFP	protein_coding	ENSG00000172273	0,09104964	0,249644672	0,352270202
JAK2	protein_coding	ENSG00000096968	0,078385874	0,249646386	0,352270202
CNBD1	protein_coding	ENSG00000176571	0,310115501	0,249681929	0,352296118
IARS2P1	processed_pseudogene	ENSG00000251563	-0,28043502	0,24982572	0,352474755
AC128709.2	lincRNA	ENSG00000235126	0,30305167	0,250038493	0,352726424
TMX2	protein_coding	ENSG00000213593	-0,061254372	0,250028727	0,352726424
AL359834.1	lincRNA	ENSG00000282849	0,222221154	0,250149546	0,35283455
RPS6KB1	protein_coding	ENSG00000108443	0,063336252	0,250135105	0,35283455
EIF2S1	protein_coding	ENSG00000134001	-0,077296649	0,250200784	0,3528593
AC016251.2	TEC	ENSG00000280291	0,262925457	0,250201502	0,3528593
MAPK6-DT	lincRNA	ENSG00000259438	0,272574241	0,250336005	0,352967775
AL513523.1	processed_pseudogene	ENSG00000223599	0,307187714	0,250347256	0,352967775

SLC35F3	protein_coding	ENSG00000183780	-0,074555511	0,250333645	0,352967775
ZNF18	protein_coding	ENSG00000154957	-0,097963364	0,250301365	0,352967775
INPP5B	protein_coding	ENSG00000204084	-0,053134424	0,25039059	0,353004606
RN7SL749P	misc_RNA	ENSG00000242853	0,273590353	0,250432578	0,353015269
FAM155A-IT1	sense_intronic	ENSG00000227248	0,311267166	0,25042486	0,353015269
LINC01126	lincRNA	ENSG00000279873	-0,291563996	0,250483264	0,353038191
HLA-DRB6	transcribed_unprocesse	ENSG00000229391	0,243329061	0,25047319	0,353038191
AC025449.1	processed_pseudogene	ENSG00000270558	-0,262276579	0,250544046	0,353099593
TMEM231	protein_coding	ENSG00000205084	-0,091021521	0,250577265	0,353122145
TIE1	protein_coding	ENSG00000066056	0,221694435	0,250660621	0,353196244
AL079307.1	sense_overlapping	ENSG00000258535	0,277299405	0,250664287	0,353196244
AC024884.2	lincRNA	ENSG00000276272	0,274767749	0,250765488	0,353294885
AL356317.1	processed_pseudogene	ENSG00000213754	0,323714588	0,250768744	0,353294885
RPL10AP2	processed_pseudogene	ENSG00000188873	-0,318134869	0,250797301	0,353309369
LTA4H	protein_coding	ENSG00000111144	-0,043189694	0,250813477	0,353309369
AC010536.3	antisense	ENSG00000277504	0,3238486	0,250967671	0,353502296
LINC01203	lincRNA	ENSG00000226985	0,265657308	0,251126927	0,353702325
GALNT7	protein_coding	ENSG00000109586	0,06792609	0,251149552	0,353709903
AL080317.3	antisense	ENSG00000272356	0,288702071	0,251194908	0,353739172
RPL19P21	processed_pseudogene	ENSG00000230508	-0,275371978	0,251223622	0,353739172
ABHD2	protein_coding	ENSG00000140526	0,075116258	0,251239324	0,353739172
DENND4B	protein_coding	ENSG00000198837	-0,111498036	0,251213927	0,353739172
PCDHGA9	protein_coding	ENSG00000261934	-0,228140053	0,25132779	0,353839441
ERICH6-AS1	antisense	ENSG00000240137	-0,155500229	0,251523649	0,354066578
MID2	protein_coding	ENSG00000080561	0,09168673	0,251588376	0,354133388
AMH	protein_coding	ENSG00000104899	-0,152119638	0,252004068	0,35469417
RPL22	protein_coding	ENSG00000116251	-0,064442585	0,252030548	0,354707098
RPS3P2	processed_pseudogene	ENSG00000231307	0,323302619	0,252048136	0,354707512
AC007318.1	processed_pseudogene	ENSG00000172974	0,158719003	0,252103541	0,354761141
TEKT4P2	transcribed_unprocesse	ENSG00000188681	-0,12269016	0,252179997	0,354844384
ZNF789	protein_coding	ENSG00000198556	0,131937896	0,252232886	0,354894457
GNE	protein_coding	ENSG00000159921	0,06325006	0,252363913	0,355054455
AC068946.1	protein_coding	ENSG00000280537	0,310705365	0,252499762	0,355221216
AL136115.2	lincRNA	ENSG00000269967	0,316291767	0,252573648	0,355227698
RN7SL605P	misc_RNA	ENSG00000265566	-0,284276174	0,252539536	0,355227698
VSIG10L	protein_coding	ENSG00000186806	-0,218224754	0,252573609	0,355227698
FGF8	protein_coding	ENSG00000107831	-0,321284531	0,252525788	0,355227698
AC026124.1	sense_intronic	ENSG00000250280	-0,303328027	0,252722133	0,355412161
PBX1	protein_coding	ENSG00000185630	0,319278916	0,252820586	0,355466436
SIK1	protein_coding	ENSG00000142178	0,312391054	0,252830052	0,355466436

EPHA2	protein_coding	ENSG00000142627	0,071764951	0,252805345	0,355466436
TMEM221	protein_coding	ENSG00000188051	-0,290074538	0,252798237	0,355466436
NNT	protein_coding	ENSG00000112992	-0,085211484	0,252920948	0,355569856
AC005037.1	antisense	ENSG00000183308	0,299987255	0,253009437	0,35566988
AC009171.2	lincRNA	ENSG00000263105	0,30865233	0,253074187	0,355736521
AC008115.3	sense_intronic	ENSG00000275560	0,279356765	0,253617741	0,356476143
GPR89B	protein_coding	ENSG00000188092	0,182607641	0,253687146	0,356549261
VDAC1P8	transcribed_processed_	ENSG00000229036	0,322443795	0,254103907	0,357110534
TOLLIP	protein_coding	ENSG00000078902	0,080119015	0,254420728	0,357531286
MIR181A2HG	antisense	ENSG00000224020	-0,187223312	0,25459419	0,357726028
C2orf66	protein_coding	ENSG00000187944	0,256767312	0,254583895	0,357726028
TNRC18	protein_coding	ENSG00000182095	-0,111584279	0,254888168	0,358114558
AL031595.3	sense_overlapping	ENSG00000280434	-0,315387709	0,254959929	0,358190845
RNU6-33P	snRNA	ENSG00000207524	0,321052899	0,255066096	0,358315454
NAT2	protein_coding	ENSG00000156006	0,304428336	0,255132947	0,358384819
RELL1	protein_coding	ENSG00000181826	0,069886286	0,255169743	0,358411959
CEP164	protein_coding	ENSG00000110274	0,084697449	0,255211427	0,358445961
SV2A	protein_coding	ENSG00000159164	0,299097365	0,255321164	0,358575533
AC138932.1	transcribed_unprocesse	ENSG00000183458	0,230448716	0,25534364	0,358582544
AL022238.4	protein_coding	ENSG00000284431	-0,298558022	0,255363979	0,358586555
LARGE2	protein_coding	ENSG00000165905	-0,319269229	0,255469803	0,358710595
ATF2	protein_coding	ENSG00000115966	-0,061420174	0,255577567	0,358837343
ZFP30	protein_coding	ENSG00000120784	-0,239649903	0,25627739	0,359770654
AP2A2	protein_coding	ENSG00000183020	-0,057395476	0,256395157	0,359911346
AC129502.1	TEC	ENSG00000280321	-0,272020637	0,2564791	0,360004541
AC007681.1	antisense	ENSG00000270571	0,321119725	0,256571496	0,360097183
TMEM225B	protein_coding	ENSG00000244219	0,279784554	0,256580216	0,360097183
CD79A	protein_coding	ENSG00000105369	0,297383075	0,256626391	0,360137344
PCNPP1	processed_pseudogene	ENSG00000258359	-0,309559176	0,256724747	0,360250723
JDP2	protein_coding	ENSG00000140044	-0,091146445	0,25676358	0,360280566
LRRC37B	protein_coding	ENSG00000185158	0,099823195	0,256883689	0,36042444
ZFAND2B	protein_coding	ENSG00000158552	-0,086823841	0,256919887	0,360447208
SNORD11	snoRNA	ENSG00000238317	-0,279434487	0,256935065	0,360447208
AL590644.1	lincRNA	ENSG00000204362	0,287389539	0,257009249	0,36052662
TXNP6	processed_pseudogene	ENSG00000234036	0,315838046	0,257051143	0,360560726
CCM2L	protein_coding	ENSG00000101331	0,218970834	0,25708598	0,360584932
AC243562.1	unprocessed_pseudoge	ENSG00000259570	0,271006284	0,257185518	0,360699875
LRRN4CL	protein_coding	ENSG00000177363	0,310867622	0,25721865	0,360721677
RASGRF2	protein_coding	ENSG00000113319	0,312929263	0,257415414	0,360972935
AP000553.1	processed_transcript	ENSG00000207751	0,314066158	0,257459659	0,360994462

AL109806.1	TEC	ENSG00000280387	0,273211913	0,257465967	0,360994462
AL451085.2	antisense	ENSG00000271380	-0,317449622	0,257603951	0,361064515
MGST2	protein_coding	ENSG00000085871	0,271679706	0,257588035	0,361064515
GPRASP1	protein_coding	ENSG00000198932	0,248839386	0,257586497	0,361064515
PSMA1	protein_coding	ENSG00000129084	-0,098968022	0,257568447	0,361064515
AC093752.1	transcribed_unprocesse	ENSG00000245958	-0,103390363	0,257563603	0,361064515
FAM166A	protein_coding	ENSG00000188163	0,275011606	0,257633217	0,361080859
CD200R1	protein_coding	ENSG00000163606	0,314319597	0,257695989	0,361120432
MAP3K12	protein_coding	ENSG00000139625	0,101554978	0,257696667	0,361120432
AL354751.1	unprocessed_pseudoge	ENSG00000234537	-0,283092395	0,25772611	0,361137017
KCTD9P2	processed_pseudogene	ENSG00000181819	0,314521207	0,257819467	0,361218475
KCNS1	protein_coding	ENSG00000124134	-0,310275193	0,257813149	0,361218475
HNRNPA3P5	processed_pseudogene	ENSG00000236565	-0,307605236	0,257989839	0,361432487
RCL1	protein_coding	ENSG00000120158	-0,070687072	0,25804812	0,361489444
LINC01483	lincRNA	ENSG00000227517	0,317819467	0,258071189	0,361497069
ARHGEF5	protein_coding	ENSG00000050327	-0,110683741	0,258093422	0,361503522
RF01233	snoRNA	ENSG00000238832	-0,285991982	0,258179253	0,361599049
ZNF721	protein_coding	ENSG00000182903	0,088713472	0,258289959	0,361716996
MEIS2	protein_coding	ENSG00000134138	0,073159416	0,258298738	0,361716996
LINC00847	lincRNA	ENSG00000245060	-0,111900351	0,258363623	0,361783157
NDUFA10	protein_coding	ENSG00000130414	-0,055936435	0,258473813	0,361912746
KCTD17	protein_coding	ENSG00000100379	-0,08409595	0,258500789	0,361925809
CDC42EP4	protein_coding	ENSG00000179604	-0,069736684	0,25886004	0,362404056
CYP39A1	protein_coding	ENSG00000146233	0,307599482	0,2589361	0,362477937
CDON	protein_coding	ENSG00000064309	0,274960546	0,258948159	0,362477937
RN7SL471P	misc_RNA	ENSG00000263426	0,31195664	0,258990496	0,362512459
RPS13P2	processed_pseudogene	ENSG00000228929	-0,215394303	0,25984784	0,363687675
LRRC63	protein_coding	ENSG00000173988	0,269747685	0,259915565	0,363757642
AC100861.2	antisense	ENSG00000250714	0,314979927	0,260281413	0,364220665
QSOX1	protein_coding	ENSG00000116260	-0,074894279	0,260281925	0,364220665
AP001453.4	lincRNA	ENSG00000257086	0,194806713	0,260360097	0,364280346
HSP90AB2P	transcribed_processed_	ENSG00000205940	-0,315136157	0,260353354	0,364280346
PTBP3	protein_coding	ENSG00000119314	0,05777696	0,260381893	0,36428599
SWT1	protein_coding	ENSG00000116668	0,11676643	0,260469426	0,364383598
CTHRC1	protein_coding	ENSG00000164932	-0,07682923	0,260544908	0,364464334
ARSA	protein_coding	ENSG00000100299	0,127763525	0,26069212	0,364645392
TPST1	protein_coding	ENSG00000169902	-0,069615448	0,261200661	0,365331802
DIRC3-AS1	lincRNA	ENSG00000233143	0,273924474	0,261352978	0,365519916
PPFIA4	protein_coding	ENSG00000143847	-0,259836388	0,261445025	0,365623719
AC138035.1	lincRNA	ENSG00000238035	0,313455403	0,261467186	0,36562978

CAPZA1	protein_coding	ENSG00000116489	-0,072301529	0,261570642	0,365749514
AC009318.3	antisense	ENSG00000274315	-0,290095084	0,261595064	0,365758727
UBE2L3	protein_coding	ENSG00000185651	-0,072416541	0,26175733	0,365960657
AC003681.1	antisense	ENSG00000279159	0,296702247	0,261840017	0,366051308
PCF11-AS1	lincRNA	ENSG00000269939	0,316062724	0,261904478	0,36611647
LINC02482	lincRNA	ENSG00000251580	0,206172185	0,262010472	0,36621974
MYO1C	protein_coding	ENSG00000197879	-0,061455803	0,262014065	0,36621974
KCNE1	protein_coding	ENSG00000180509	0,281482831	0,262138854	0,366321751
AC073911.3	TEC	ENSG00000279817	0,285014411	0,262140631	0,366321751
AC104986.2	lincRNA	ENSG00000253948	0,26787985	0,262404473	0,366665468
MAP2K1	protein_coding	ENSG00000169032	-0,051733407	0,262512113	0,366790888
CTIF	protein_coding	ENSG00000134030	0,091974757	0,26258345	0,366865569
RAB3GAP1	protein_coding	ENSG00000115839	-0,050667482	0,26267826	0,366948039
SLC35C2	protein_coding	ENSG00000080189	-0,067223567	0,262665157	0,366948039
TFAP2E	protein_coding	ENSG00000116819	0,310753911	0,262783623	0,367070224
AC012313.8	lincRNA	ENSG00000269473	-0,255522837	0,262905522	0,367190484
PRPH	protein_coding	ENSG00000135406	0,314458773	0,262897626	0,367190484
POMP	protein_coding	ENSG00000132963	0,074821159	0,262926168	0,367193052
STARD3	protein_coding	ENSG00000131748	-0,073729576	0,262943167	0,367193052
AL596094.1	lincRNA	ENSG00000272764	-0,261391215	0,263098691	0,367385222
ZNF124	protein_coding	ENSG00000196418	0,112732053	0,263155654	0,367439748
DNAJC16	protein_coding	ENSG00000116138	0,062110391	0,263269614	0,367573845
AC104971.1	bidirectional_promoter_	ENSG00000267226	-0,244437686	0,263314985	0,367612167
AC012574.2	lincRNA	ENSG00000254002	0,313849569	0,263365764	0,367658032
LACTB	protein_coding	ENSG00000103642	-0,055445901	0,263533113	0,367866614
PSMG1	protein_coding	ENSG00000183527	-0,077717251	0,263622623	0,367966516
KCNQ2	protein_coding	ENSG00000075043	0,269154636	0,263657178	0,367989705
GOLGA7	protein_coding	ENSG00000147533	0,075046268	0,26369822	0,368021945
HIC2	protein_coding	ENSG00000169635	0,101078528	0,263830406	0,368181372
AC091271.1	lincRNA	ENSG00000273702	-0,161672949	0,264042626	0,36845246
PURG	protein_coding	ENSG00000172733	-0,30962657	0,264151122	0,368578781
AL050402.1	lincRNA	ENSG00000227880	0,294483915	0,264251309	0,368693494
AC005618.1	lincRNA	ENSG00000272070	0,237410139	0,264303352	0,36874102
OTUD3	protein_coding	ENSG00000169914	-0,089176077	0,264372373	0,368812228
GNAS-AS1	antisense	ENSG00000235590	-0,19787662	0,264478766	0,368910466
BCL2L15	protein_coding	ENSG00000188761	0,278502979	0,264471401	0,368910466
AC015799.1	TEC	ENSG00000279432	-0,300878022	0,264552839	0,368988693
SCLT1	protein_coding	ENSG00000151466	-0,066644704	0,264681309	0,369142774
EIF1B	protein_coding	ENSG00000114784	0,083673987	0,264930278	0,369464881
AC108463.3	lincRNA	ENSG00000271590	0,304919277	0,265027817	0,369550652

GATD1	protein_coding	ENSG00000177225	-0,08748414	0,265198012	0,369762829
NRAV	antisense	ENSG00000248008	0,078463945	0,265258022	0,369821361
PSMA5	protein_coding	ENSG00000143106	-0,074250821	0,26547405	0,370097388
RPS4XP14	processed_pseudogene	ENSG00000243663	0,288227746	0,265544613	0,370170598
ZNF418	protein_coding	ENSG00000196724	-0,13532626	0,265604393	0,370228768
PERM1	protein_coding	ENSG00000187642	0,27800223	0,265658727	0,370279341
MAX	protein_coding	ENSG00000125952	-0,050112956	0,265702626	0,370315361
CDH18	protein_coding	ENSG00000145526	-0,064357647	0,265739236	0,37034122
B3GNT10	protein_coding	ENSG00000214654	0,125180119	0,26577711	0,370368836
FDXR	protein_coding	ENSG00000161513	0,088436	0,265836006	0,370388457
S100A10	protein_coding	ENSG00000197747	0,054710151	0,265845367	0,370388457
FTH1P10	transcribed_processed_	ENSG00000223361	0,281679315	0,265810751	0,370388457
AC114728.1	processed_pseudogene	ENSG00000241612	0,288194342	0,265942866	0,370499129
USP11	protein_coding	ENSG00000102226	-0,056803138	0,266140745	0,370749622
PLOD1	protein_coding	ENSG00000083444	0,050085419	0,266164266	0,370757206
AES	protein_coding	ENSG00000104964	0,068763833	0,266190288	0,370768272
SNX29P1	unprocessed_pseudoge	ENSG00000158482	0,310799993	0,266227174	0,370794468
KCNJ14	protein_coding	ENSG00000182324	0,264857919	0,266353601	0,370945363
AL359399.1	antisense	ENSG00000258748	-0,271283892	0,266399957	0,370969951
BBS1	protein_coding	ENSG00000174483	-0,269284083	0,266407431	0,370969951
MYCBP2	protein_coding	ENSG00000005810	0,098324824	0,266525536	0,371109215
LINC01089	lincRNA	ENSG00000212694	0,184190744	0,266547561	0,371109857
FTH1P2	processed_pseudogene	ENSG00000234975	0,299899669	0,266562185	0,371109857
RF00019	misc_RNA	ENSG00000207370	0,311344615	0,266671564	0,371236937
AC131159.1	antisense	ENSG00000276292	0,264302592	0,266775794	0,37135683
GDPD2	protein_coding	ENSG00000130055	-0,304839925	0,266936675	0,371555561
SCARNA18	snoRNA	ENSG00000238835	0,307921607	0,267411272	0,372190906
AC114763.1	lincRNA	ENSG00000228043	-0,210215276	0,267519181	0,372315832
AC026979.2	lincRNA	ENSG00000271869	0,288706276	0,267859725	0,372764484
HNRNPH1	protein_coding	ENSG00000169045	-0,059805797	0,267971497	0,372894728
AC106900.1	lincRNA	ENSG00000270460	0,265659253	0,26808667	0,373008721
SNORA12	snoRNA	ENSG00000212464	-0,134370066	0,268089788	0,373008721
PSTK	protein_coding	ENSG00000179988	0,080106318	0,268109338	0,373010617
CCS	protein_coding	ENSG00000173992	0,097261105	0,268177563	0,373080229
NLGN2	protein_coding	ENSG00000169992	-0,105664579	0,268226724	0,373123311
ULK4P2	transcribed_unprocesse	ENSG00000260128	-0,288513139	0,268372779	0,373301166
INPP5F	protein_coding	ENSG00000198825	-0,063318995	0,268485363	0,373432442
CEP170P1	transcribed_unprocesse	ENSG00000154608	0,156559183	0,268511105	0,373442921
IL15RA	protein_coding	ENSG00000134470	0,306847219	0,268572033	0,373502332
DPY19L4	protein_coding	ENSG00000156162	-0,066399777	0,268627079	0,373553555

SNORD19	snoRNA	ENSG00000212493	-0,296382951	0,268667723	0,373584746
CIB1	protein_coding	ENSG00000185043	-0,06759868	0,268749727	0,37367344
TMEM248P1	processed_pseudogene	ENSG00000249555	0,280217694	0,26878819	0,373681616
PPM1J	protein_coding	ENSG00000155367	-0,225702346	0,268792047	0,373681616
SH3BGRL3	protein_coding	ENSG00000142669	0,062359725	0,268819304	0,37369418
AC241585.1	processed_pseudogene	ENSG00000223612	-0,218934438	0,268864027	0,373731019
RPL17P22	processed_pseudogene	ENSG00000243592	0,309359225	0,269072853	0,373970604
LNX1	protein_coding	ENSG00000072201	0,311512549	0,269154289	0,374058441
GUSBP5	transcribed_processed_	ENSG00000236296	-0,267530777	0,269250455	0,374166735
AP005329.1	antisense	ENSG00000264235	0,267963786	0,269396377	0,374344153
FOXD4L1	protein_coding	ENSG00000184492	0,307838496	0,269455415	0,374400824
EVI5	protein_coding	ENSG00000067208	-0,049219118	0,269714637	0,374735618
AL139089.1	lincRNA	ENSG00000273723	-0,307672438	0,269752277	0,374762528
LINC00630	lincRNA	ENSG00000223546	0,110298654	0,269843624	0,374864042
EHHADH	protein_coding	ENSG00000113790	-0,084030265	0,269957268	0,374996516
AC104695.3	lincRNA	ENSG00000270210	0,266978583	0,270208345	0,375319867
AC074351.1	antisense	ENSG00000234707	-0,310099056	0,270318185	0,375447009
THAP5	protein_coding	ENSG00000177683	-0,089527372	0,270382358	0,37551071
PI4K2B	protein_coding	ENSG00000038210	-0,085336987	0,270408621	0,375521756
HDAC2-AS2	antisense	ENSG00000228624	0,131881223	0,270566274	0,375715252
SHC1P2	processed_pseudogene	ENSG00000267691	0,250061933	0,270735707	0,37592508
SYP	protein_coding	ENSG00000102003	0,137149245	0,27081466	0,376009253
GAPDHP60	processed_pseudogene	ENSG00000248180	-0,308704552	0,270835454	0,376012668
AC097658.1	processed_pseudogene	ENSG00000228981	-0,145546146	0,270879623	0,376048536
AP003969.2	TEC	ENSG00000279684	0,254693749	0,270982428	0,376165793
BX322562.1	lincRNA	ENSG00000273796	-0,305597314	0,271076854	0,376271404
PABPC1P3	processed_pseudogene	ENSG00000230673	-0,300522586	0,271219231	0,376443557
RN7SL664P	misc_RNA	ENSG00000265078	-0,296643842	0,271390777	0,376656167
AC090241.1	processed_pseudogene	ENSG00000267587	0,24864363	0,271642951	0,376980643
ASRGL1	protein_coding	ENSG00000162174	0,063792707	0,271972267	0,377412125
AC087241.3	antisense	ENSG00000257023	0,291270833	0,272047854	0,377491474
NIM1K	protein_coding	ENSG00000177453	0,309233716	0,272082658	0,377514228
PI3	protein_coding	ENSG00000124102	0,304742829	0,272194437	0,377643773
ZNF44	protein_coding	ENSG00000197857	0,129293151	0,272269426	0,377722262
NEK4	protein_coding	ENSG00000114904	-0,054540298	0,272382267	0,37785325
MT-ND2	protein_coding	ENSG00000198763	0,214415885	0,272491768	0,377979586
TTC39C	protein_coding	ENSG00000168234	0,090229679	0,272546069	0,378029341
ZNF559-ZNF177	protein_coding	ENSG00000270011	-0,302525148	0,272568541	0,378034946
MAP1S	protein_coding	ENSG00000130479	-0,098651225	0,272605295	0,378060355
AC134878.2	transcribed_unprocesse	ENSG00000278212	-0,297315041	0,272638231	0,378080468

NXPE3	protein_coding	ENSG00000144815	-0,050626094	0,272847038	0,37834445
STK32A	protein_coding	ENSG00000169302	0,305163302	0,272969263	0,378488344
DUSP5	protein_coding	ENSG00000138166	0,061115562	0,273296974	0,378917119
UCHL5	protein_coding	ENSG00000116750	-0,0811111	0,273376666	0,379001989
PCDHB4	protein_coding	ENSG00000081818	-0,292053937	0,273399392	0,379007877
AC016394.2	antisense	ENSG00000272599	-0,146012948	0,273500176	0,379073179
RNMT	protein_coding	ENSG00000101654	0,070690011	0,273501946	0,379073179
BSCL2	protein_coding	ENSG00000168000	-0,240767151	0,273497089	0,379073179
COPS8P2	processed_pseudogene	ENSG00000214552	-0,284605629	0,273747604	0,379384321
PIGT	protein_coding	ENSG00000124155	0,047031946	0,27376343	0,379384321
DIDO1	protein_coding	ENSG00000101191	-0,069255141	0,274046196	0,379750522
PDCD6IPP1	transcribed_unprocesse	ENSG00000275325	-0,274166764	0,274844104	0,380830468
SNORA38B	snoRNA	ENSG00000200394	-0,277062479	0,27496577	0,380973313
RPL36	protein_coding	ENSG00000130255	-0,081247443	0,274996567	0,380990245
AC073342.1	antisense	ENSG00000231840	-0,2937966	0,275192527	0,381235983
STEAP1	protein_coding	ENSG00000164647	-0,100505063	0,275274668	0,38132402
AP002008.2	transcribed_unitary_pse	ENSG00000255093	0,290612675	0,275344007	0,381394312
AC022417.1	TEC	ENSG00000279923	-0,299535801	0,275405537	0,381453779
TMEM191A	transcribed_unprocesse	ENSG00000226287	-0,174082733	0,275591979	0,381686237
PRKCE	protein_coding	ENSG00000171132	0,042283588	0,275705145	0,381810652
HECTD1	protein_coding	ENSG00000092148	-0,064638688	0,275719042	0,381810652
ASB1	protein_coding	ENSG00000065802	-0,071822095	0,275755181	0,381834915
CUTALP	transcribed_unitary_pse	ENSG00000226752	-0,084693575	0,2758726	0,381971716
LCLAT1	protein_coding	ENSG00000172954	0,060148342	0,275911504	0,381999794
AP2A1	protein_coding	ENSG00000196961	0,080140841	0,27596751	0,382051545
AC009227.1	antisense	ENSG00000224675	0,287240129	0,276086124	0,382189957
MIER2	protein_coding	ENSG00000105556	0,090707841	0,276549864	0,382806082
DAZAP2P1	processed_pseudogene	ENSG00000223825	-0,282159988	0,276615356	0,382855752
ACADVL	protein_coding	ENSG00000072778	-0,054077061	0,276623081	0,382855752
AC019129.2	antisense	ENSG00000273106	0,266171445	0,27668129	0,382884641
RWDD1	protein_coding	ENSG00000111832	-0,084643466	0,276672406	0,382884641
AL161787.1	processed_pseudogene	ENSG00000219928	-0,263897863	0,276842828	0,383082337
ITGB3	protein_coding	ENSG00000259207	0,146799632	0,276910087	0,383149557
DOCK11	protein_coding	ENSG00000147251	-0,145943002	0,277115176	0,383407465
AC011313.1	antisense	ENSG00000257642	-0,14495358	0,27718524	0,383452669
CCDC159	protein_coding	ENSG00000183401	0,214692233	0,277181396	0,383452669
ARF3	protein_coding	ENSG00000134287	0,0657439	0,27739144	0,383686163
RNF169	protein_coding	ENSG00000166439	-0,060602859	0,277380087	0,383686163
AC002558.3	sense_intronic	ENSG00000262265	-0,304501068	0,277500795	0,383811539
SLC16A9	protein_coding	ENSG00000165449	0,302872434	0,277531493	0,383828113

AL445524.1	antisense	ENSG00000233461	-0,108173574	0,277600323	0,383871536
HCN2	protein_coding	ENSG00000099822	-0,094857453	0,277583536	0,383871536
FAM86KP	unprocessed_pseudoge	ENSG00000163612	-0,266737597	0,277763225	0,384070906
AC006064.4	antisense	ENSG00000269968	0,281249698	0,277925078	0,384242895
C7orf57	protein_coding	ENSG00000164746	0,298677935	0,277923853	0,384242895
TYK2	protein_coding	ENSG00000105397	0,083911184	0,278097891	0,384455901
AL031009.1	TEC	ENSG00000278987	-0,283368016	0,278614408	0,385118042
PRKRA-AS1	antisense	ENSG00000223960	0,097875676	0,278735638	0,385233689
PPP4R3A	protein_coding	ENSG00000100796	-0,051756721	0,278734907	0,385233689
SLC52A1	protein_coding	ENSG00000132517	0,284161454	0,278932385	0,385479632
RPL37AP1	processed_pseudogene	ENSG00000226243	-0,301240165	0,279240637	0,385864419
AL021878.2	sense_intronic	ENSG00000270083	-0,297698969	0,279248443	0,385864419
LRCH2	protein_coding	ENSG00000130224	0,25624954	0,279333487	0,385955929
AC026410.3	processed_pseudogene	ENSG00000248569	0,301474854	0,279405446	0,385977349
CAPNS1	protein_coding	ENSG00000126247	0,046417142	0,279392857	0,385977349
SERINC3	protein_coding	ENSG00000132824	0,041771359	0,279383736	0,385977349
AC091181.1	TEC	ENSG00000279555	-0,283502666	0,279650088	0,386289285
AC092117.1	lincRNA	ENSG00000276791	0,215208103	0,279801272	0,386472092
HOXA7	protein_coding	ENSG00000122592	-0,192764196	0,280193142	0,386987296
MAGI1-IT1	sense_intronic	ENSG00000272610	0,297567542	0,280242932	0,38703
DAB2IP	protein_coding	ENSG00000136848	0,094610909	0,280306787	0,387092123
RPS6KB2	protein_coding	ENSG00000175634	0,0860822	0,280424697	0,387228878
YTHDC2	protein_coding	ENSG00000047188	-0,059833561	0,280724623	0,38761694
PDPR	protein_coding	ENSG00000090857	0,081457007	0,280838224	0,387747693
FLG-AS1	antisense	ENSG00000237975	0,137140597	0,280891297	0,387753981
AC015712.6	processed_transcript	ENSG00000272808	0,259804312	0,280899495	0,387753981
CFHR1	protein_coding	ENSG00000244414	0,295066083	0,280899115	0,387753981
AC093525.8	TEC	ENSG00000279520	-0,300680359	0,280938785	0,387782118
CHST12	protein_coding	ENSG00000136213	-0,08777225	0,28104121	0,387897391
ZNF200	protein_coding	ENSG00000010539	0,074380399	0,281130526	0,387994555
AC092802.1	antisense	ENSG00000226026	0,292140485	0,281316845	0,388199455
AL139412.1	lincRNA	ENSG00000236947	0,294098621	0,281300959	0,388199455
LINC01909	lincRNA	ENSG00000266258	-0,300782998	0,281396779	0,388260886
AC245060.4	transcribed_unprocesse	ENSG00000272779	0,06853863	0,281399223	0,388260886
WDR75	protein_coding	ENSG00000115368	-0,047229224	0,281503207	0,388370162
CCM2	protein_coding	ENSG00000136280	-0,068409564	0,281516294	0,388370162
AL158201.1	processed_pseudogene	ENSG00000228328	-0,301104329	0,281602726	0,388463271
ENSAP2	processed_pseudogene	ENSG00000259032	0,301661534	0,28162614	0,388469441
AC025946.1	lincRNA	ENSG00000226990	-0,298791059	0,281668479	0,388501715
COX17	protein_coding	ENSG00000138495	-0,095705583	0,281692024	0,388508064

SYNGR4	protein_coding	ENSG00000105467	0,249282131	0,281738645	0,38852011
TWISTNB	protein_coding	ENSG00000105849	0,063842267	0,281728396	0,38852011
CD46	protein_coding	ENSG00000117335	0,059654204	0,281777491	0,388547556
RPL21P16	processed_pseudogene	ENSG00000220842	-0,243489598	0,281798155	0,388549927
RTN4RL2	protein_coding	ENSG00000186907	-0,177283604	0,281824923	0,388560714
ENDOV	protein_coding	ENSG00000173818	-0,089929287	0,281860529	0,388583682
HSP90AA2P	processed_pseudogene	ENSG00000224411	-0,231681421	0,281900812	0,388613097
ZNF576	protein_coding	ENSG00000124444	-0,088776536	0,281984115	0,388701808
FEZ2	protein_coding	ENSG00000171055	-0,052765144	0,282064607	0,388760506
PIGZ	protein_coding	ENSG00000119227	-0,182582459	0,282056877	0,388760506
MMP12	protein_coding	ENSG00000262406	0,264476467	0,282089118	0,388768164
CIAO2B	protein_coding	ENSG00000166595	0,085903646	0,282281276	0,388954584
CBWD2	protein_coding	ENSG00000136682	0,082185987	0,28228025	0,388954584
UBXN4	protein_coding	ENSG00000144224	0,059101553	0,282246188	0,388954584
AC005224.1	lincRNA	ENSG00000231595	-0,282318431	0,282427802	0,389130339
FKBP1C	protein_coding	ENSG00000198225	0,29193073	0,2824731	0,389166607
AC109466.1	antisense	ENSG00000241956	0,275803318	0,282539038	0,389231305
CORO7	protein_coding	ENSG00000262246	-0,153079472	0,282753517	0,389500613
S100Z	protein_coding	ENSG00000171643	0,226777062	0,282942833	0,389735225
EPB41L4A-AS1	lincRNA	ENSG00000224032	-0,062818953	0,283027681	0,389825917
FAM168B	protein_coding	ENSG00000152102	0,064164949	0,283118567	0,389898732
PAIP2B	protein_coding	ENSG00000124374	0,138752142	0,283174271	0,389923081
BCDIN3D	protein_coding	ENSG00000186666	-0,126636384	0,283156051	0,389923081
ZNF292	protein_coding	ENSG00000188994	0,062836029	0,283225855	0,38996793
MICOS10	protein_coding	ENSG00000173436	0,106929816	0,283263217	0,389993191
AC009120.2	processed_transcript	ENSG00000259972	-0,196247282	0,28330511	0,390024688
AP001783.1	antisense	ENSG00000254607	0,203685454	0,283447615	0,390142312
AL049828.1	lincRNA	ENSG00000258526	0,285169668	0,283442337	0,390142312
EEF1B2P3	processed_pseudogene	ENSG00000232472	0,170931422	0,283437391	0,390142312
JAG2	protein_coding	ENSG00000184916	-0,112872865	0,283518582	0,390213804
TRMO	protein_coding	ENSG00000136932	-0,090169933	0,283575745	0,39026629
AL022068.1	antisense	ENSG00000228412	-0,28834436	0,283890787	0,390621229
ITIH3	protein_coding	ENSG00000162267	0,296842992	0,283882839	0,390621229
IL17RA	protein_coding	ENSG00000177663	0,07711107	0,283870254	0,390621229
GNB1	protein_coding	ENSG00000078369	0,031749399	0,284038762	0,390798619
MRPL1	protein_coding	ENSG00000169288	-0,093187619	0,284144341	0,390891437
TCAF1P1	unprocessed_pseudoge	ENSG00000223459	-0,234289213	0,284128984	0,390891437
COLEC12	protein_coding	ENSG00000158270	0,277289433	0,284325657	0,391088407
TMEM14C	protein_coding	ENSG00000111843	-0,071049572	0,284310503	0,391088407
CABLES1	protein_coding	ENSG00000134508	-0,082730766	0,284380556	0,391137687

MPDZ	protein_coding	ENSG00000107186	-0,067976229	0,284490469	0,391262624
GNRH1	protein_coding	ENSG00000147437	-0,287076594	0,284596369	0,391382026
FOXJ2	protein_coding	ENSG00000065970	-0,093700582	0,285071231	0,391997655
AC087276.1	sense_overlapping	ENSG00000254577	-0,298538583	0,285082254	0,391997655
AC019163.1	antisense	ENSG00000248980	0,285860802	0,285134307	0,392042946
ASMTL-AS1	antisense	ENSG00000236017	-0,299249106	0,285314478	0,392264374
FRY	protein_coding	ENSG00000073910	-0,154109048	0,285353881	0,392292251
AL121906.2	lincRNA	ENSG00000275223	0,293673198	0,285387964	0,392312811
SDHAP3	transcribed_unprocesse	ENSG00000185986	0,219998304	0,285481335	0,392414864
PPM1H	protein_coding	ENSG00000111110	0,078720961	0,285799941	0,39280016
R3HDM1	protein_coding	ENSG00000048991	0,073451657	0,285796476	0,39280016
TOR1AIP1	protein_coding	ENSG00000143337	0,0530588	0,285985923	0,393029434
HOXA11	protein_coding	ENSG00000005073	-0,244476536	0,286013029	0,393040351
PKD1P6	transcribed_unprocesse	ENSG00000250251	0,196883725	0,286074264	0,393098162
RPL23P2	processed_pseudogene	ENSG00000176054	0,29804434	0,286110176	0,39312117
SLC9A3R2	protein_coding	ENSG00000065054	-0,112483154	0,286313455	0,393374126
AC009093.4	lincRNA	ENSG00000260953	0,282947056	0,286389637	0,393452439
LPAL2	transcribed_unprocesse	ENSG00000213071	0,265911963	0,286414259	0,393459909
AGTPBP1	protein_coding	ENSG00000135049	-0,068252203	0,286470347	0,393510603
INSYN1	protein_coding	ENSG00000205363	-0,262202786	0,286654853	0,39373768
ANKRD18B	protein_coding	ENSG00000230453	-0,102403973	0,286814626	0,393930756
RN7SL192P	misc_RNA	ENSG00000276757	-0,289326172	0,286850181	0,393940565
MORC4	protein_coding	ENSG00000133131	0,043521018	0,286860182	0,393940565
AL133477.1	processed_pseudogene	ENSG00000238251	-0,283003075	0,286960452	0,394051879
RRP15	protein_coding	ENSG00000067533	-0,074218053	0,287014786	0,394100104
AP001830.1	antisense	ENSG00000255337	0,297816969	0,287037817	0,394105344
AC122718.1	processed_pseudogene	ENSG00000250461	0,293895458	0,287157346	0,394243067
DNAJC4	protein_coding	ENSG00000110011	0,100489937	0,287250362	0,394344372
TRPM4	protein_coding	ENSG00000130529	0,09669566	0,287521919	0,394690755
UBE2FP1	processed_pseudogene	ENSG00000224080	0,21250456	0,287725461	0,39494373
NSUN2	protein_coding	ENSG00000037474	-0,045271249	0,287770756	0,394953038
GMPR	protein_coding	ENSG00000137198	-0,162319919	0,28775879	0,394953038
ULK2	protein_coding	ENSG00000083290	0,286949687	0,28785019	0,395035623
ARFRP1	protein_coding	ENSG00000101246	0,086424043	0,28803103	0,395257355
MOCS2	protein_coding	ENSG00000164172	-0,068510331	0,288174553	0,39542785
AC022210.1	processed_pseudogene	ENSG00000213399	-0,266615109	0,288195629	0,395430315
IFITM4P	processed_pseudogene	ENSG00000235821	0,252110729	0,288300478	0,395547716
XPOTP1	processed_pseudogene	ENSG00000214185	0,294792891	0,288368712	0,395588407
TMEM98	protein_coding	ENSG00000006042	-0,145927343	0,288353161	0,395588407
UBE2D3P1	processed_pseudogene	ENSG00000225022	0,293240508	0,288734347	0,39601053

TPT1P9	processed_pseudogene	ENSG00000234782	0,274925366	0,288720291	0,39601053
RAB11FIP2	protein_coding	ENSG00000107560	0,068029472	0,288724353	0,39601053
FAM45A	protein_coding	ENSG00000119979	-0,052159728	0,288903236	0,396215672
FGF20	protein_coding	ENSG00000078579	0,29000227	0,289267201	0,396688305
AC092338.1	protein_coding	ENSG00000140743	-0,069878312	0,28930176	0,396709173
LINC02009	lincRNA	ENSG00000283646	0,271607791	0,289346984	0,396744661
AKT3-IT1	sense_intronic	ENSG00000228939	0,295445987	0,289429794	0,396831678
MRPL40P1	processed_pseudogene	ENSG00000256037	0,256565746	0,289555548	0,39697756
CDC42EP3	protein_coding	ENSG00000163171	0,035636708	0,28969606	0,397143653
LINC01184	lincRNA	ENSG00000245937	0,067591223	0,289866872	0,39735126
Z97055.2	antisense	ENSG00000261251	-0,294427312	0,289929956	0,397411174
RPL5P9	processed_pseudogene	ENSG00000236330	-0,284391452	0,290002904	0,3974846
DNAJC28	protein_coding	ENSG00000177692	0,203636739	0,290089664	0,397576947
CYP2D7	polymorphic_pseudogene	ENSG00000205702	-0,284570427	0,290363379	0,397898904
GPR39	protein_coding	ENSG00000183840	0,072575312	0,290400429	0,397923088
DDI2	protein_coding	ENSG00000197312	-0,069091202	0,290432347	0,397926035
AL353763.2	TEC	ENSG00000280077	-0,203171366	0,290441382	0,397926035
AC133552.1	processed_pseudogene	ENSG00000260714	0,252667717	0,290610936	0,398131741
AC079779.2	processed_transcript	ENSG00000228643	0,26428686	0,290656465	0,398167519
TBP	protein_coding	ENSG00000112592	-0,070149061	0,290737464	0,398251879
AC009630.1	antisense	ENSG00000253133	0,282598937	0,29084838	0,398377206
BRK1	protein_coding	ENSG00000254999	0,057822757	0,290876888	0,398389648
DPM1	protein_coding	ENSG00000000419	-0,084546685	0,290976883	0,398499991
CDH13	protein_coding	ENSG00000140945	0,251279154	0,291168387	0,398735636
ADIRF-AS1	processed_transcript	ENSG00000272734	-0,142247018	0,291286379	0,398870585
TMEM105	processed_transcript	ENSG00000185332	0,292987262	0,29140257	0,399003052
RNU4-24P	snRNA	ENSG00000201435	0,242079831	0,291499223	0,399108749
AC004034.1	antisense	ENSG00000262482	0,251635517	0,291554712	0,399131434
AC015912.3	lincRNA	ENSG00000274213	0,216302251	0,291538818	0,399131434
PCDHB10	protein_coding	ENSG00000120324	0,292157504	0,291637448	0,399218051
NDUFAF7	protein_coding	ENSG00000003509	0,06242466	0,291710027	0,399290754
AC006372.1	lincRNA	ENSG00000223872	0,267375355	0,291853139	0,399459986
AC024475.4	lincRNA	ENSG00000255314	0,239127583	0,292042586	0,399692609
XYLT1	protein_coding	ENSG00000103489	0,249189587	0,292240631	0,399936968
MAP2K4	protein_coding	ENSG00000065559	0,05134786	0,29246297	0,40021454
LINC02230	lincRNA	ENSG00000251543	-0,289129906	0,292651454	0,400445748
PSMB8	protein_coding	ENSG00000204264	0,095585793	0,292718029	0,400510125
SNX8	protein_coding	ENSG00000106266	0,070466224	0,292784704	0,400530156
PPIC	protein_coding	ENSG00000168938	0,060717522	0,292791255	0,400530156
SPTLC1P1	transcribed_processed_	ENSG00000230397	-0,252819282	0,292790233	0,400530156

HTATIP2	protein_coding	ENSG00000109854	0,071461292	0,293003652	0,400793977
AC116533.1	processed_pseudogene	ENSG00000244398	-0,103963449	0,293155916	0,400975514
PCDHGA11	protein_coding	ENSG00000253873	0,286738149	0,293433833	0,401328882
HSD11B1L	protein_coding	ENSG00000167733	-0,176414193	0,293608158	0,40154053
ARFGEF1	protein_coding	ENSG00000066777	0,049583986	0,293651599	0,401573163
PAX8	protein_coding	ENSG00000125618	0,292278509	0,293728859	0,401652036
AC004923.4	antisense	ENSG00000255306	0,289470043	0,293748543	0,401652175
AL138966.2	antisense	ENSG00000277368	-0,280022511	0,294134309	0,402152836
PALM	protein_coding	ENSG00000099864	-0,076375578	0,294308893	0,402364711
GALNT2	protein_coding	ENSG00000143641	0,052159383	0,294768606	0,402861887
PCMT1	protein_coding	ENSG00000120265	-0,058105602	0,294744427	0,402861887
ZFP69	protein_coding	ENSG00000187815	-0,088723529	0,294738803	0,402861887
HDHD3	protein_coding	ENSG00000119431	-0,088821939	0,294770762	0,402861887
LINC00933	transcribed_unprocesse	ENSG00000259728	0,279693387	0,29475825	0,402861887
AC136604.3	antisense	ENSG00000250999	0,28993932	0,294884827	0,402990925
AL671277.2	unprocessed_pseudoge	ENSG00000237669	0,269253167	0,295239303	0,403421592
CBWD1	protein_coding	ENSG00000172785	0,075231413	0,295343475	0,403537051
C19orf66	protein_coding	ENSG00000130813	-0,08153643	0,295426842	0,40362407
AC007038.1	antisense	ENSG00000229127	0,265424712	0,29550852	0,403708769
HNRNPA1P33	processed_pseudogene	ENSG00000213412	-0,26778194	0,295555187	0,403745629
AC018529.2	antisense	ENSG00000278330	0,291264342	0,295614717	0,403773165
REXO1	protein_coding	ENSG00000079313	-0,084263729	0,29560203	0,403773165
ZNF763	protein_coding	ENSG00000197054	0,282381408	0,2956897	0,403848688
GABBR2	protein_coding	ENSG00000136928	-0,123759173	0,295805869	0,403980448
AC092119.3	antisense	ENSG00000275445	0,291021253	0,295841404	0,404002077
AL512422.1	antisense	ENSG00000231441	0,272244324	0,295953001	0,404100663
KMT2E-AS1	antisense	ENSG00000239569	0,245486335	0,29594436	0,404100663
TMEM127	protein_coding	ENSG00000135956	0,071497925	0,296015968	0,404159733
PLCH1	protein_coding	ENSG00000114805	0,258487562	0,296045342	0,404172933
PSME2P2	processed_pseudogene	ENSG00000225131	0,204063555	0,296091168	0,40420859
AC010536.1	lincRNA	ENSG00000226180	-0,239576784	0,29626583	0,404405245
NAIF1	protein_coding	ENSG00000171169	0,084266645	0,296274657	0,404405245
NDUFA1	protein_coding	ENSG00000125356	-0,083221659	0,29634405	0,404473046
MFSD10	protein_coding	ENSG00000109736	-0,099444163	0,296428524	0,404561421
RHBDL3	protein_coding	ENSG00000141314	0,262570368	0,296508576	0,404630709
MLYCD	protein_coding	ENSG00000103150	-0,141987131	0,29651875	0,404630709
ZNF445	protein_coding	ENSG00000185219	-0,082562727	0,296541552	0,404634903
HNRNPKP4	processed_pseudogene	ENSG00000243547	-0,222447053	0,296778224	0,404930906
LCA5L	protein_coding	ENSG00000157578	0,220523268	0,29688107	0,405044286
C3orf52	protein_coding	ENSG00000114529	-0,056230442	0,296927161	0,405080223

AL078590.2	lincRNA	ENSG00000231971	0,225243513	0,297062046	0,405223542
CEP135	protein_coding	ENSG00000174799	0,065986307	0,29707173	0,405223542
BET1L	protein_coding	ENSG00000177951	-0,071966724	0,297142046	0,405292503
SUCLA2	protein_coding	ENSG00000136143	-0,068663931	0,297263585	0,405431316
XK	protein_coding	ENSG00000047597	0,153761977	0,297387443	0,405573273
YBX1P5	processed_pseudogene	ENSG00000251108	-0,279759552	0,297504431	0,405705843
D2HGDH	protein_coding	ENSG00000180902	-0,111154726	0,297533941	0,40571911
KLK14	protein_coding	ENSG00000129437	-0,241196682	0,297573183	0,405745644
SEC23IP	protein_coding	ENSG00000107651	-0,051758348	0,297648033	0,405820724
VARS2	protein_coding	ENSG00000137411	-0,067236723	0,297701513	0,40586666
OST4	protein_coding	ENSG00000228474	0,071907024	0,297731938	0,40588116
ST3GAL2	protein_coding	ENSG00000157350	-0,07800238	0,297823962	0,405979627
ROPN1L	protein_coding	ENSG00000145491	0,247410418	0,297874782	0,406021917
AP001619.1	sense_intronic	ENSG00000227698	-0,279901316	0,298006442	0,406174384
AC103706.1	lincRNA	ENSG00000261220	-0,27035498	0,298304544	0,406448594
GRASP	protein_coding	ENSG00000161835	0,276772437	0,298259083	0,406448594
AQR	protein_coding	ENSG00000021776	0,045768539	0,298294007	0,406448594
RBM43	protein_coding	ENSG00000184898	-0,095343437	0,298306713	0,406448594
ANKRD20A1	protein_coding	ENSG00000260691	-0,287932433	0,298262158	0,406448594
NPTN-IT1	sense_intronic	ENSG00000281183	0,277531138	0,298328433	0,406451187
AC092747.4	lincRNA	ENSG00000275764	-0,158665261	0,298491043	0,406583991
FBXW11P1	processed_pseudogene	ENSG00000230870	0,287664395	0,298453404	0,406583991
QDPR	protein_coding	ENSG00000151552	-0,094784109	0,298499786	0,406583991
HOXB4	protein_coding	ENSG00000182742	-0,203425957	0,298505204	0,406583991
H2AFVP1	transcribed_processed_	ENSG00000258741	-0,277127652	0,298733286	0,406867634
RAD51-AS1	processed_transcript	ENSG00000245849	-0,249483399	0,298862986	0,407017254
PIGH	protein_coding	ENSG00000100564	-0,055892025	0,298934116	0,407087094
RSKR	protein_coding	ENSG00000167524	-0,189854223	0,299003427	0,407154447
SH3KBP1	protein_coding	ENSG00000147010	-0,046534684	0,299054901	0,407197505
C6orf163	protein_coding	ENSG00000203872	0,280982291	0,299087455	0,407214797
PRRT4	protein_coding	ENSG00000224940	0,248652382	0,299149489	0,407234418
HEBP1	protein_coding	ENSG00000013583	-0,071507466	0,299161432	0,407234418
AP003108.4	TEC	ENSG00000279632	-0,286585137	0,29913906	0,407234418
MIS12	protein_coding	ENSG00000167842	0,064443805	0,299184688	0,407239047
HSPG2	protein_coding	ENSG00000142798	0,137031732	0,299218649	0,407258245
AL357033.1	antisense	ENSG00000167046	-0,262134218	0,299447549	0,407542749
AL512625.2	lincRNA	ENSG00000229422	-0,27620724	0,299546035	0,407595646
TMEM270	protein_coding	ENSG00000175877	0,287014792	0,299535068	0,407595646
RNU1-27P	snRNA	ENSG00000206596	0,250281062	0,299536633	0,407595646
AC010327.5	processed_transcript	ENSG00000276570	-0,278195671	0,299859992	0,407995783

AC079336.2	sense_intronic	ENSG00000265222	0,28279086	0,2999194	0,408049546
MAN2B2	protein_coding	ENSG00000013288	0,058819145	0,299959738	0,408054484
COX16	protein_coding	ENSG00000133983	-0,183935871	0,299962821	0,408054484
CCT8	protein_coding	ENSG00000156261	-0,049665685	0,300171476	0,408311246
AC012360.2	lincRNA	ENSG00000272861	-0,259152097	0,300484498	0,408547368
AL359878.1	processed_transcript	ENSG00000205740	0,158590696	0,30047362	0,408547368
UXS1	protein_coding	ENSG00000115652	-0,070220483	0,300474065	0,408547368
GFER	protein_coding	ENSG00000127554	-0,0722113	0,30041473	0,408547368
GCAT	protein_coding	ENSG00000100116	-0,107181048	0,300456003	0,408547368
TMEM52	protein_coding	ENSG00000178821	-0,254751814	0,300424731	0,408547368
WWTR1-IT1	sense_intronic	ENSG00000241985	0,245186158	0,300394868	0,408547368
GPR68	protein_coding	ENSG00000119714	0,24284793	0,300578678	0,408578442
C11orf74	protein_coding	ENSG00000166352	0,078680632	0,300584644	0,408578442
TERF1	protein_coding	ENSG00000147601	-0,068581038	0,300568536	0,408578442
PCDHGB9P	unprocessed_pseudoge	ENSG00000276545	0,264256056	0,300587036	0,408578442
AL391994.1	processed_pseudogene	ENSG00000227962	0,253262262	0,300621791	0,408598604
AL450326.1	lincRNA	ENSG00000230555	-0,231855504	0,300822148	0,408843831
AC069307.1	lincRNA	ENSG00000269949	-0,287930134	0,300906079	0,408925235
SDAD1	protein_coding	ENSG00000198301	0,045742888	0,300937023	0,408925235
HEXA	protein_coding	ENSG00000213614	-0,059744074	0,300941858	0,408925235
AL450384.1	antisense	ENSG00000225527	0,241437325	0,301162747	0,409198273
TMEM259	protein_coding	ENSG00000182087	0,079074156	0,301211446	0,40921831
EIF2B5	protein_coding	ENSG00000145191	0,041330925	0,301217397	0,40921831
AC087741.3	TEC	ENSG00000279259	0,239982766	0,301262708	0,409252759
FOXO3B	processed_pseudogene	ENSG00000240445	-0,153812092	0,301452107	0,409482927
AC245052.3	processed_pseudogene	ENSG00000235081	0,278330073	0,30153594	0,409569677
AC025031.4	lincRNA	ENSG00000275481	0,208305735	0,301668379	0,409722432
ACTR6	protein_coding	ENSG00000075089	-0,086375863	0,301753513	0,409810923
PHAX	protein_coding	ENSG00000164902	0,05108915	0,301911435	0,409998248
RAB3A	protein_coding	ENSG00000105649	0,254397594	0,302039557	0,410145082
AC007040.2	protein_coding	ENSG00000258881	-0,197935935	0,302362596	0,41055656
UBXN11	protein_coding	ENSG00000158062	0,085476682	0,30239597	0,410574696
RBBP4P2	processed_pseudogene	ENSG00000242457	0,235506852	0,302531467	0,410731475
CYP51A1P2	processed_pseudogene	ENSG00000233588	0,261113161	0,302606423	0,410801017
LSM12P1	processed_pseudogene	ENSG00000232024	-0,173748679	0,302622748	0,410801017
SMC6	protein_coding	ENSG00000163029	-0,06364911	0,302669343	0,410837076
SMIM1	protein_coding	ENSG00000235169	-0,27942772	0,302720023	0,410878675
RGPD1	protein_coding	ENSG00000187627	0,245034532	0,302824111	0,410992755
DHX9	protein_coding	ENSG00000135829	0,043491608	0,302993861	0,41119593
ZNF573	protein_coding	ENSG00000189144	-0,121971052	0,303407087	0,411729479

ZNF426-DT	lincRNA	ENSG00000278611	0,265270929	0,303430631	0,411734188
ZNF570	protein_coding	ENSG00000171827	0,07650359	0,303514832	0,411821196
ZNF558	protein_coding	ENSG00000167785	-0,078376951	0,303699743	0,412017577
AC090607.2	transcribed_processed_	ENSG00000259562	0,271135446	0,30368455	0,412017577
TMEM191B	protein_coding	ENSG00000278558	-0,276827353	0,303803831	0,412131528
AC016588.2	lincRNA	ENSG00000282393	-0,260305521	0,303970663	0,412330575
MIER3	protein_coding	ENSG00000155545	-0,079861784	0,30403494	0,412390491
ZNF583	protein_coding	ENSG00000198440	0,102195253	0,304100946	0,412452745
SBF2-AS1	antisense	ENSG00000246273	-0,073546473	0,304193648	0,412551194
LINC02458	lincRNA	ENSG00000246363	0,247535107	0,304220219	0,412559951
AC016907.2	antisense	ENSG00000233862	0,278065958	0,304484472	0,412891009
SPATA20	protein_coding	ENSG00000006282	0,063942337	0,304707407	0,413166001
AL162386.2	sense_intronic	ENSG00000234740	0,245341942	0,304735387	0,413176623
AP003086.1	lincRNA	ENSG00000251323	0,284381257	0,304822376	0,413222514
ZNF615	protein_coding	ENSG00000197619	0,073556952	0,304822404	0,413222514
DRGX	protein_coding	ENSG00000165606	-0,237023897	0,304829675	0,413222514
ADPGK-AS1	antisense	ENSG00000260898	0,268512782	0,304892093	0,413224884
CTAGE3P	processed_pseudogene	ENSG00000232872	0,282390302	0,304891844	0,413224884
GABRB1	protein_coding	ENSG00000163288	0,277460445	0,304929947	0,413224884
UTP3	protein_coding	ENSG00000132467	0,059960417	0,304933827	0,413224884
MATN3	protein_coding	ENSG00000132031	-0,248237918	0,304952308	0,413224884
AC055811.2	protein_coding	ENSG00000264187	-0,26798593	0,30488974	0,413224884
AC008750.2	antisense	ENSG00000255441	0,256436981	0,305142639	0,413455475
DHRS4-AS1	antisense	ENSG00000215256	-0,082019872	0,305229013	0,413545189
IFT74	protein_coding	ENSG00000096872	-0,075053174	0,305360144	0,413695525
AC055855.2	antisense	ENSG00000261318	0,266111401	0,305563435	0,41386159
C5orf66	antisense	ENSG00000224186	0,188062854	0,305553962	0,41386159
AC010531.6	antisense	ENSG00000270006	-0,265907653	0,305547135	0,41386159
AP002884.1	lincRNA	ENSG00000250303	-0,124706419	0,305518359	0,41386159
AL021707.2	antisense	ENSG00000228274	-0,207107271	0,305920416	0,414317731
AC008610.1	antisense	ENSG00000248367	-0,251025597	0,305970697	0,414358468
AL355312.4	antisense	ENSG00000273132	0,280964003	0,306314065	0,414796021
SBK1	protein_coding	ENSG00000188322	-0,225251113	0,306334244	0,414796021
MRPL53	protein_coding	ENSG00000204822	0,234858899	0,306460005	0,414938917
AL592148.3	antisense	ENSG00000272750	0,219291129	0,306480913	0,414939832
AC124276.2	lincRNA	ENSG00000255400	0,221355582	0,306611866	0,415089727
AC137630.4	lincRNA	ENSG00000273211	0,281799421	0,30670155	0,415171577
DHX16	protein_coding	ENSG00000204560	-0,045316789	0,30671281	0,415171577
DHX40	protein_coding	ENSG00000108406	-0,061863562	0,306770157	0,415221799
PARD3-AS1	antisense	ENSG00000226386	0,261585104	0,306978202	0,415454968

ITGB2-AS1	antisense	ENSG00000227039	0,193235048	0,306982937	0,415454968
SLC25A23	protein_coding	ENSG00000125648	-0,073032133	0,307044591	0,41551099
CCDC15	protein_coding	ENSG00000149548	0,063554954	0,307119824	0,415557962
AC020915.1	protein_coding	ENSG00000267216	-0,207024606	0,307113756	0,415557962
MRPL21	protein_coding	ENSG00000197345	-0,06298517	0,307218084	0,415652663
AC073869.1	transcribed_processed_	ENSG00000152117	0,110520299	0,307230345	0,415652663
AL442067.1	sense_intronic	ENSG00000276573	-0,233158489	0,307278859	0,415690877
AC044787.1	processed_pseudogene	ENSG00000213862	-0,254905131	0,30739151	0,415815847
SLC15A1	protein_coding	ENSG00000088386	0,280922632	0,308007216	0,416621249
TRMT112P6	processed_pseudogene	ENSG00000268412	-0,275038636	0,308075839	0,41668659
RASA3	protein_coding	ENSG00000185989	0,063869697	0,30840066	0,417098419
AC034102.5	antisense	ENSG00000257809	0,212674969	0,308464066	0,417156665
FGFBP3	protein_coding	ENSG00000174721	0,228153565	0,308526161	0,417213131
AC104938.1	antisense	ENSG00000260037	-0,263992953	0,308724082	0,417453252
KCNE5	protein_coding	ENSG00000176076	0,274237377	0,308786839	0,417510585
AC002524.1	processed_pseudogene	ENSG00000233247	0,241384646	0,308848105	0,417565896
AC090578.1	antisense	ENSG00000253553	0,278529299	0,308981195	0,417690766
PWAR5	TEC	ENSG00000279192	0,146253941	0,308968915	0,417690766
DIS3	protein_coding	ENSG00000083520	0,055977328	0,309094399	0,417816261
LYPLA2	protein_coding	ENSG00000011009	-0,076517986	0,309185341	0,417911647
IGIP	protein_coding	ENSG00000182700	0,143547489	0,309227065	0,417940501
MORN1	protein_coding	ENSG00000116151	-0,13398803	0,309374974	0,418112857
SPATA32	protein_coding	ENSG00000184361	0,266420226	0,309431778	0,418162072
ARHGAP17	protein_coding	ENSG00000140750	0,063114081	0,309611458	0,4183222
AC007014.2	sense_intronic	ENSG00000274038	0,261296484	0,3095924	0,4183222
AL132656.1	transcribed_processed_	ENSG00000224886	0,220121931	0,309578622	0,4183222
AC015849.4	sense_overlapping	ENSG00000270894	-0,277591172	0,309650037	0,418346767
CHMP3	protein_coding	ENSG00000115561	-0,048897658	0,309685757	0,418367466
IDUA	protein_coding	ENSG00000127415	0,177371609	0,309761267	0,418441914
AC078864.1	lincRNA	ENSG00000257989	0,234496903	0,309807385	0,41847665
API5P1	processed_pseudogene	ENSG00000234558	-0,269702667	0,309980908	0,418655896
AL592435.1	antisense	ENSG00000232995	-0,158663578	0,310010984	0,418668945
DDN-AS1	antisense	ENSG00000257913	-0,264593182	0,310070391	0,418721603
LINC00997	lincRNA	ENSG00000281332	-0,25967235	0,310490495	0,419178518
RPL41P5	processed_pseudogene	ENSG00000256393	-0,269771217	0,31045084	0,419178518
TMEM144	protein_coding	ENSG00000164124	0,07920424	0,310483894	0,419178518
SLC16A7	protein_coding	ENSG00000118596	-0,08263556	0,310478159	0,419178518
MFSD9	protein_coding	ENSG00000135953	0,089845802	0,310763875	0,419519981
RGPD3	protein_coding	ENSG00000153165	0,280009432	0,310908739	0,419632682
TAF10	protein_coding	ENSG00000166337	0,07062109	0,31089195	0,419632682

A1BG	protein_coding	ENSG00000121410	-0,276935669	0,310881004	0,419632682
TSNAX	protein_coding	ENSG00000116918	-0,093241886	0,310950741	0,419661755
RPL13AP25	processed_pseudogene	ENSG00000136149	-0,232742584	0,311005804	0,419680837
TMEM91	protein_coding	ENSG00000142046	-0,214197043	0,310999799	0,419680837
AC009107.2	antisense	ENSG00000260927	0,263278676	0,311111892	0,419796376
CALHM2	protein_coding	ENSG00000138172	-0,073298267	0,311349316	0,420089104
RTN4RL1	protein_coding	ENSG00000185924	0,265634071	0,311434687	0,42017665
CCDC3	protein_coding	ENSG00000151468	0,273429912	0,31151565	0,420202958
RNFT2	protein_coding	ENSG00000135119	0,081678613	0,311502288	0,420202958
PDLIM2	protein_coding	ENSG00000120913	-0,084317608	0,311481058	0,420202958
AC104564.2	sense_intronic	ENSG00000264007	-0,277885916	0,311652895	0,420360442
TMEFF1	protein_coding	ENSG00000241697	-0,257830032	0,311716894	0,420419115
FAM174A	protein_coding	ENSG00000174132	0,100509113	0,311787791	0,420487085
AC018804.1	unprocessed_pseudoge	ENSG00000227632	-0,232700263	0,311880337	0,42058424
C16orf86	protein_coding	ENSG00000159761	-0,277778543	0,31193294	0,420627522
WDR38	protein_coding	ENSG00000136918	0,279449308	0,31200583	0,42069815
SNORA46	snoRNA	ENSG00000207493	-0,260062101	0,31204852	0,420728053
TCAF1	protein_coding	ENSG00000198420	-0,080940794	0,312159997	0,420850689
POU5F2	protein_coding	ENSG00000248483	-0,278635525	0,312311215	0,421026884
SLC11A1	protein_coding	ENSG00000018280	0,274872937	0,312360232	0,421065288
LINC00106	lincRNA	ENSG00000236871	0,245784394	0,31238564	0,421071508
PPDPF	protein_coding	ENSG00000125534	0,10045297	0,312405906	0,421071508
AC011461.1	sense_intronic	ENSG00000267197	-0,240795127	0,312429192	0,421075222
AL121760.1	lincRNA	ENSG00000230839	0,20414664	0,312554025	0,421215786
AC084036.1	antisense	ENSG00000272990	0,273878077	0,312590056	0,421236666
NPM1P39	processed_pseudogene	ENSG00000225159	-0,219761841	0,312673712	0,421266363
CPLX1	protein_coding	ENSG00000168993	-0,169785579	0,312666847	0,421266363
DPY19L2P3	transcribed_unprocesse	ENSG00000227855	-0,24755308	0,312636092	0,421266363
NR6A1	protein_coding	ENSG00000148200	0,145405555	0,312771843	0,421370895
AC234782.3	processed_pseudogene	ENSG00000234345	0,268502969	0,31281619	0,421379567
KRT18P1	processed_pseudogene	ENSG00000228666	0,238509627	0,312819371	0,421379567
ARL4AP5	processed_pseudogene	ENSG00000218996	0,279402623	0,312868221	0,421416773
ZFHX4	protein_coding	ENSG00000091656	0,176840617	0,312888085	0,421416773
DOK4	protein_coding	ENSG00000125170	-0,148376096	0,313406235	0,422086931
AC080013.5	antisense	ENSG00000272247	0,277519277	0,313495204	0,422147137
FAM133A	protein_coding	ENSG00000179083	-0,090075332	0,313520416	0,422147137
LMLN	protein_coding	ENSG00000185621	-0,113720006	0,313496348	0,422147137
AC010335.3	TEC	ENSG00000280121	0,2630253	0,313533269	0,422147137
LINC00663	lincRNA	ENSG00000266904	0,200786931	0,313749125	0,422410041
AL390067.1	antisense	ENSG00000226609	-0,2711467	0,31388459	0,422564682

RRBP1	protein_coding	ENSG00000125844	0,087575913	0,314039754	0,422726936
AC008669.1	sense_overlapping	ENSG00000260686	-0,21914304	0,314046334	0,422726936
GUSBP9	unprocessed_pseudoge	ENSG00000215630	-0,195433823	0,31435108	0,423109374
AP000446.1	antisense	ENSG00000255234	0,265883627	0,314406068	0,423155617
LINC00310	lincRNA	ENSG00000227456	0,277956453	0,314472135	0,423197816
NECAB3	protein_coding	ENSG00000125967	0,082797985	0,314504407	0,423197816
LRRC8C	protein_coding	ENSG00000171488	-0,063244666	0,314519957	0,423197816
MOB4	protein_coding	ENSG00000115540	-0,099339059	0,314500727	0,423197816
YJEFN3	protein_coding	ENSG00000250067	-0,209688922	0,314653592	0,423349853
AL592295.4	lincRNA	ENSG00000283696	0,277819418	0,314719875	0,423411258
LINC01763	lincRNA	ENSG00000225446	0,239616733	0,314800889	0,423492473
Z97353.1	processed_pseudogene	ENSG00000231369	0,235264029	0,314914956	0,423618138
NFU1P2	processed_pseudogene	ENSG00000233557	0,183997445	0,31495061	0,423638314
MYORG	protein_coding	ENSG00000164976	-0,142077783	0,314998089	0,423674392
CMAS	protein_coding	ENSG00000111726	-0,047018763	0,315132538	0,423827433
NLRP11	protein_coding	ENSG00000179873	-0,256028132	0,315302181	0,424027784
LFNG	protein_coding	ENSG00000106003	0,137264975	0,315585985	0,424325983
MFSD13A	protein_coding	ENSG00000138111	-0,093502144	0,315582477	0,424325983
AC107021.2	sense_overlapping	ENSG00000261051	-0,179062115	0,315562842	0,424325983
CREB3L4	protein_coding	ENSG00000143578	-0,08380183	0,315807284	0,424540041
SH2D3A	protein_coding	ENSG00000125731	0,233448776	0,31583678	0,424551866
KDM7A-DT	bidirectional_promoter_	ENSG00000260231	0,246489035	0,316015656	0,424764473
FAM160A1	protein_coding	ENSG00000164142	-0,17623621	0,316050455	0,42478341
SACM1L	protein_coding	ENSG00000211456	0,061251655	0,316160462	0,424875578
ARFGAP2	protein_coding	ENSG00000149182	-0,056306513	0,316142915	0,424875578
AC004156.1	lincRNA	ENSG00000267666	0,250345214	0,316383054	0,425146855
AC106038.1	antisense	ENSG00000246792	0,27507208	0,316495217	0,425269713
SMURF2P1	transcribed_unprocesse	ENSG00000248121	-0,187995658	0,316548437	0,425313361
CAP1	protein_coding	ENSG00000131236	-0,039463173	0,316580746	0,425328907
TACO1	protein_coding	ENSG00000136463	-0,060609268	0,316702842	0,425465074
AC023024.1	antisense	ENSG00000259172	0,275914299	0,31680406	0,425517437
MIF-AS1	antisense	ENSG00000218537	0,233440137	0,316769014	0,425517437
PEX19	protein_coding	ENSG00000162735	0,057916934	0,316798509	0,425517437
TSSC4	protein_coding	ENSG00000184281	-0,088135297	0,317041246	0,425808129
AC013470.2	protein_coding	ENSG00000226690	0,273269143	0,317075713	0,425826538
GET4	protein_coding	ENSG00000239857	0,182154877	0,317120471	0,425858761
AP000254.1	antisense	ENSG00000273271	0,27043813	0,317175952	0,42590538
NINJ2	protein_coding	ENSG00000171840	0,256319115	0,317196804	0,425905495
Z74021.1	processed_pseudogene	ENSG00000232346	-0,246537073	0,317401269	0,426152135
AC006482.1	lincRNA	ENSG00000228675	0,253414208	0,317425826	0,42615721

AIMP1P2	processed_pseudogene	ENSG00000227177	0,277081932	0,317449086	0,426160541
GPD2	protein_coding	ENSG00000115159	-0,04888753	0,317475767	0,426168465
AC017083.3	protein_coding	ENSG00000273398	-0,257114679	0,317545823	0,426234609
ABHD13	protein_coding	ENSG00000139826	-0,063480495	0,317769733	0,426507245
MIR3936HG	processed_transcript	ENSG00000233006	-0,205113173	0,318082891	0,426899627
NBPF8	transcribed_unprocesse	ENSG00000270231	-0,102955242	0,318223777	0,427060765
AC003965.1	lincRNA	ENSG00000263280	-0,256170953	0,318304808	0,42714156
CYP20A1	protein_coding	ENSG00000119004	-0,055384903	0,318376539	0,427209867
LINC01023	lincRNA	ENSG00000272523	0,270903555	0,318435478	0,427261001
XIST	lincRNA	ENSG00000229807	0,22221335	0,318507034	0,427329055
RN7SL381P	misc_RNA	ENSG00000263968	-0,263804708	0,318664091	0,427483844
C19orf25	protein_coding	ENSG00000119559	-0,095217402	0,31870217	0,427506966
DTX2	protein_coding	ENSG00000091073	0,115997979	0,318823845	0,427642212
MTCO1P12	unprocessed_pseudoge	ENSG00000237973	0,247659926	0,318963817	0,42780198
AC100821.2	lincRNA	ENSG00000260955	0,264273562	0,319139698	0,428009887
NTRK3	protein_coding	ENSG00000140538	0,208520992	0,319330514	0,428237796
PURA	protein_coding	ENSG00000185129	-0,061169575	0,319461726	0,428385747
CGNL1	protein_coding	ENSG00000128849	-0,126175381	0,319519036	0,428434586
AC010680.4	lincRNA	ENSG00000271141	0,275662796	0,319616825	0,428537692
AC012065.5	processed_pseudogene	ENSG00000279307	0,272215429	0,319902913	0,428893237
SEC24B	protein_coding	ENSG00000138802	0,065019172	0,319955901	0,42893624
DMGDH	protein_coding	ENSG00000132837	-0,253902329	0,320246846	0,429298222
PTDSS1	protein_coding	ENSG00000156471	-0,049404216	0,320410834	0,429489981
LINC02377	lincRNA	ENSG00000250102	0,200887582	0,320637409	0,429737519
RPL23P4	processed_pseudogene	ENSG00000223903	0,265268481	0,321003429	0,430192586
FITM1	protein_coding	ENSG00000139914	0,232412267	0,321018894	0,430192586
HSBP1	protein_coding	ENSG00000230989	-0,066919886	0,321144448	0,430332721
LINC01270	lincRNA	ENSG00000203999	0,124402873	0,321314995	0,430350745
FTH1P7	processed_pseudogene	ENSG00000232187	0,239579242	0,321232664	0,430350745
AC068733.3	processed_transcript	ENSG00000282556	-0,271783249	0,321291304	0,430350745
STN1	protein_coding	ENSG00000107960	0,066953128	0,321297922	0,430350745
CUL3	protein_coding	ENSG00000036257	0,058202947	0,321261108	0,430350745
CNOT2	protein_coding	ENSG00000111596	-0,051688974	0,321325758	0,430350745
CAPZA2	protein_coding	ENSG00000198898	-0,080535314	0,321249525	0,430350745
SPTAN1	protein_coding	ENSG00000197694	0,074778059	0,32150213	0,430558844
UBE2E2	protein_coding	ENSG00000182247	-0,071190783	0,321607708	0,430672113
HDAC7	protein_coding	ENSG00000061273	0,077918825	0,321683002	0,430744818
RPL38	protein_coding	ENSG00000172809	-0,063514084	0,321957951	0,43108484
DNM1P47	transcribed_unprocesse	ENSG00000259660	-0,203736739	0,322020652	0,431140647
TMEM216	protein_coding	ENSG00000187049	0,129663765	0,322113193	0,431236396

AP001781.1	lincRNA	ENSG00000254990	0,269477444	0,322269749	0,431389672
RGMB	protein_coding	ENSG00000174136	-0,05358259	0,322257956	0,431389672
CFL2	protein_coding	ENSG00000165410	0,068457967	0,32234571	0,431463193
DHX8	protein_coding	ENSG00000067596	0,041844137	0,322706079	0,431917364
ZNF276	protein_coding	ENSG00000158805	0,106420108	0,32273899	0,431933226
HMGB1P10	processed_pseudogene	ENSG00000213707	-0,199401672	0,322838881	0,432038722
BBOX1-AS1	antisense	ENSG00000254560	0,259676447	0,32291887	0,4321118
DNAJA2	protein_coding	ENSG00000069345	-0,061086791	0,322935625	0,4321118
ZNF653	protein_coding	ENSG00000161914	0,126746643	0,32298346	0,432147614
SMIM30	protein_coding	ENSG00000214194	-0,086641801	0,323012833	0,432158722
RPUSD1	protein_coding	ENSG00000007376	-0,095120933	0,323034432	0,432159429
LINC01535	lincRNA	ENSG00000226686	0,269838307	0,323081306	0,432193947
RPS4XP2	transcribed_processed_	ENSG00000226948	0,269374307	0,323123048	0,432221595
SYBU	protein_coding	ENSG00000147642	-0,132110087	0,323234792	0,432342871
MRPS30-DT	antisense	ENSG00000251141	0,240815532	0,323297653	0,432398753
ACP6	protein_coding	ENSG00000162836	-0,093335058	0,323442573	0,432564369
MED10	protein_coding	ENSG00000133398	0,060941042	0,323468659	0,43257105
AC104563.1	processed_pseudogene	ENSG00000240036	-0,261654115	0,323497255	0,432581087
DESI2	protein_coding	ENSG00000121644	0,042987916	0,323589556	0,432676302
LINC01968	lincRNA	ENSG00000237222	0,270650073	0,323723939	0,432799556
RPL5P1	processed_pseudogene	ENSG00000241061	0,254982003	0,323717622	0,432799556
RN7SL359P	misc_RNA	ENSG00000244384	-0,239031502	0,323801171	0,432874593
KRTAP4-9	protein_coding	ENSG00000212722	0,268537625	0,324262424	0,433462968
SNORD63B	snoRNA	ENSG00000222937	0,242546456	0,324337139	0,433534588
C17orf64	protein_coding	ENSG00000141371	0,229803653	0,324454176	0,433661639
NPW	protein_coding	ENSG00000183971	-0,27057006	0,324474476	0,433661639
OR51B5	protein_coding	ENSG00000167355	0,228903133	0,32476171	0,434017245
FOXD3-AS1	antisense	ENSG00000230798	0,121485964	0,32488176	0,434149394
LINC01516	lincRNA	ENSG00000231422	0,251483303	0,324922275	0,434175246
AC124319.5	TEC	ENSG00000280248	-0,266779001	0,325280543	0,434625663
SMAD9	protein_coding	ENSG00000120693	-0,249863422	0,325333253	0,434667775
BX649632.1	antisense	ENSG00000273249	0,237857902	0,325564197	0,434941352
AC022211.4	TEC	ENSG00000279035	-0,269298077	0,325580428	0,434941352
RPL7AP66	processed_pseudogene	ENSG00000175886	-0,270812359	0,32563357	0,434984012
AC006064.3	antisense	ENSG00000255966	0,263004783	0,325864837	0,435264592
AP003071.1	lincRNA	ENSG00000260895	0,245916968	0,326369967	0,435910916
POLR3A	protein_coding	ENSG00000148606	0,051911874	0,326456647	0,435998296
AC003080.1	processed_pseudogene	ENSG00000234358	0,239889055	0,326657368	0,436227865
POLR2J2	protein_coding	ENSG00000228049	0,250078755	0,326671077	0,436227865
STX7	protein_coding	ENSG00000079950	0,060424448	0,326721683	0,436258983

QRSL1	protein_coding	ENSG00000130348	-0,065912156	0,32673692	0,436258983
FRAS1	protein_coding	ENSG00000138759	0,258602943	0,326972242	0,436522756
DOHH	protein_coding	ENSG00000129932	0,092972304	0,326977041	0,436522756
TWSG1	protein_coding	ENSG00000128791	-0,075328534	0,327050871	0,436592904
TMSB10P1	processed_pseudogene	ENSG00000228499	-0,235370418	0,327076136	0,436598213
AC008764.10	TEC	ENSG00000279977	-0,262727902	0,327120754	0,436629355
AC008498.1	unprocessed_pseudoge	ENSG00000276945	0,252166959	0,327150768	0,436641
AC004923.1	transcribed_unprocesse	ENSG00000254610	-0,219480503	0,32718141	0,436653483
AC104135.1	lincRNA	ENSG00000236209	0,21407669	0,327350431	0,436822209
AC006111.2	sense_overlapping	ENSG00000263235	0,25680563	0,327331008	0,436822209
DNAL1	protein_coding	ENSG00000119661	0,062751573	0,327407602	0,436870075
SETD4	protein_coding	ENSG00000185917	-0,081774561	0,327451817	0,436900648
AC012306.2	lincRNA	ENSG00000272667	-0,17960961	0,327686662	0,437185548
GNAO1	protein_coding	ENSG00000087258	-0,262476375	0,32785952	0,437387717
AC090229.1	lincRNA	ENSG00000267397	0,269378781	0,328145204	0,437740367
AC087741.1	antisense	ENSG00000262580	0,200210046	0,328681444	0,438398675
EEF1A1P4	processed_pseudogene	ENSG00000245205	0,269660418	0,328661216	0,438398675
AC254562.3	lincRNA	ENSG00000281538	0,245316568	0,328981415	0,438770245
C1orf131	protein_coding	ENSG00000143633	0,06970485	0,329392266	0,43928964
RAD21-AS1	antisense	ENSG00000253327	0,252867516	0,329495813	0,439399163
PMS2P2	unprocessed_pseudoge	ENSG00000278416	0,258751078	0,329602433	0,439512769
AC004067.1	antisense	ENSG00000273447	0,257184439	0,3296756	0,439576367
ATP11B	protein_coding	ENSG00000058063	-0,05955882	0,329697208	0,439576367
BCL2L2	protein_coding	ENSG00000129473	-0,084224304	0,329714423	0,439576367
AC040977.1	lincRNA	ENSG00000262089	0,189177765	0,329768904	0,439620424
AL136985.3	lincRNA	ENSG00000283445	0,265907192	0,329790763	0,43962099
RNU6-90P	snRNA	ENSG00000272337	-0,245573758	0,32988312	0,439715525
NSUN5P2	transcribed_unprocesse	ENSG00000106133	-0,154335592	0,330055671	0,439916936
ST3GAL6-AS1	antisense	ENSG00000239445	-0,192223847	0,330146182	0,439951802
AC234783.1	processed_pseudogene	ENSG00000235946	0,21069969	0,330107339	0,439951802
RPL13AP5	processed_pseudogene	ENSG00000236552	-0,112875776	0,330138774	0,439951802
NACC1	protein_coding	ENSG00000160877	-0,076831117	0,330271152	0,440089743
TAS2R43	protein_coding	ENSG00000255374	-0,26411336	0,330568858	0,440457824
TAS2R43	protein_coding	ENSG00000255374	-0,26411336	0,330568858	0,440457824
AC004846.2	antisense	ENSG00000258944	-0,264469782	0,330728394	0,440641767
LSS	protein_coding	ENSG00000160285	0,078538735	0,330760163	0,440655469
COX4I1	protein_coding	ENSG00000131143	-0,069898891	0,331107947	0,441090153
PIGK	protein_coding	ENSG00000142892	-0,070498931	0,33113592	0,441098767
MMAB	protein_coding	ENSG00000139428	0,084115102	0,331293358	0,441279827
CYCSP6	processed_pseudogene	ENSG00000214429	0,244347418	0,331487077	0,441509185

LINC01583	lincRNA	ENSG00000259518	-0,104502924	0,331794988	0,441775851
SNHG19	lincRNA	ENSG00000260260	-0,136137241	0,331728903	0,441775851
SLC9B1	protein_coding	ENSG00000164037	0,252692828	0,331776345	0,441775851
KDM5C	protein_coding	ENSG00000126012	0,068232499	0,331757547	0,441775851
OR7E38P	unprocessed_pseudoge	ENSG00000183444	0,08124491	0,331740341	0,441775851
AC010463.3	sense_intronic	ENSG00000269815	-0,244052925	0,332053957	0,442091961
S1PR4	protein_coding	ENSG00000125910	0,263440653	0,332338927	0,442442645
AC025171.1	sense_overlapping	ENSG00000177738	-0,140814493	0,332528773	0,442637924
AC073050.1	lincRNA	ENSG00000228222	0,260578526	0,332601919	0,442679465
CASC19	processed_transcript	ENSG00000254166	0,267484607	0,332603147	0,442679465
WDR59	protein_coding	ENSG00000103091	-0,049891825	0,332697032	0,442775688
EIF2S3	protein_coding	ENSG00000130741	0,040726066	0,332949215	0,443082559
SLC4A7	protein_coding	ENSG00000033867	-0,060319339	0,333058611	0,443199384
IKZF4	protein_coding	ENSG00000123411	-0,199528665	0,333142499	0,443282254
AC245297.1	unprocessed_pseudoge	ENSG00000215861	0,254843878	0,333256928	0,443405747
BICRA	protein_coding	ENSG00000063169	-0,170707909	0,333387007	0,443550047
AC022415.1	transcribed_processed_	ENSG00000242615	0,22600884	0,333509762	0,443684583
MRPS31P4	transcribed_unprocesse	ENSG00000250299	-0,193342536	0,333604362	0,443781649
KCNK15-AS1	antisense	ENSG00000244558	0,246947398	0,333802253	0,444016098
ADK	protein_coding	ENSG00000156110	-0,065651507	0,333900792	0,444118369
AC040162.1	protein_coding	ENSG00000261884	-0,263919605	0,333946305	0,444150101
KMT2D	protein_coding	ENSG00000167548	0,068695654	0,334037137	0,444242101
AL022328.4	antisense	ENSG00000273253	0,263324406	0,334092384	0,444286767
LINC00926	lincRNA	ENSG00000247982	0,263912105	0,33430388	0,444539198
ACKR2	protein_coding	ENSG00000144648	0,241726612	0,334601209	0,44490409
C9orf3	protein_coding	ENSG00000148120	0,075370422	0,334621672	0,44490409
AC005332.5	lincRNA	ENSG00000277476	0,164407191	0,334720504	0,445006646
LINC01033	lincRNA	ENSG00000249069	0,207449505	0,334893253	0,445207455
ISOC2	protein_coding	ENSG00000063241	-0,082700315	0,334922704	0,445217749
AC093591.3	processed_pseudogene	ENSG00000282855	0,263166944	0,335032502	0,445334842
NUDT5	protein_coding	ENSG00000165609	-0,056173142	0,335310558	0,445675558
AC021192.1	lincRNA	ENSG00000249171	0,202055941	0,335453333	0,445836435
AC090124.2	lincRNA	ENSG00000255227	0,238883407	0,335505332	0,445876651
AC009238.3	unprocessed_pseudoge	ENSG00000248821	0,231046144	0,335529876	0,445880379
MID1	protein_coding	ENSG00000101871	-0,046449492	0,335563888	0,445896686
USPL1	protein_coding	ENSG00000132952	0,052511602	0,335697599	0,446045462
AL023803.2	lincRNA	ENSG00000274825	-0,251875074	0,335851283	0,446205095
EXOG	protein_coding	ENSG00000157036	-0,099004779	0,335861251	0,446205095
AL160313.1	lincRNA	ENSG00000247970	0,256363892	0,33588955	0,446213787
CAPN10-DT	antisense	ENSG00000260942	0,241223543	0,335997416	0,446328173

SEC62	protein_coding	ENSG00000008952	0,06136723	0,336038304	0,446353579
AC006042.4	antisense	ENSG00000234141	0,238771677	0,336143626	0,446464562
SLAMF7	protein_coding	ENSG00000026751	0,226936254	0,336179734	0,446483607
CD14	protein_coding	ENSG00000170458	0,243511002	0,336307921	0,446596016
AP001429.1	sense_intronic	ENSG00000270116	0,263705515	0,336292486	0,446596016
SAPCD1	protein_coding	ENSG00000228727	-0,263953189	0,336377649	0,44665969
SOX7	protein_coding	ENSG00000171056	-0,172198727	0,336442058	0,446716295
PLPP7	protein_coding	ENSG00000160539	0,264311976	0,336543165	0,44673486
KIAA1211	protein_coding	ENSG00000109265	0,144229448	0,336539519	0,44673486
SCNM1	protein_coding	ENSG00000163156	0,061924631	0,336540487	0,44673486
SCNM1	protein_coding	ENSG00000163156	0,061924631	0,336540487	0,44673486
AC026992.2	antisense	ENSG00000261634	0,213826507	0,336587865	0,44676528
AC006252.1	antisense	ENSG00000243224	-0,230071368	0,336722217	0,446914688
AC011416.3	lincRNA	ENSG00000283897	-0,229777069	0,336749173	0,446921544
CASC10	protein_coding	ENSG00000204682	-0,124247677	0,336822889	0,446990453
Z83847.1	antisense	ENSG00000261202	-0,261796574	0,33727282	0,447558588
VTN	protein_coding	ENSG00000109072	0,260885028	0,337481205	0,447787583
C15orf39	protein_coding	ENSG00000167173	-0,088803839	0,337489052	0,447787583
LINC02604	lincRNA	ENSG00000273142	0,204909299	0,337531864	0,447815417
NPIPB4	protein_coding	ENSG00000185864	0,257647237	0,337678299	0,447922772
HEATR5A	protein_coding	ENSG00000129493	0,065522651	0,337667267	0,447922772
THY1	protein_coding	ENSG00000154096	-0,06334828	0,337635225	0,447922772
CAHM	lincRNA	ENSG00000270419	-0,24465531	0,337896434	0,448183138
CUL4B	protein_coding	ENSG00000158290	0,040822871	0,337936145	0,448206825
GRIN3B	protein_coding	ENSG00000116032	0,196707934	0,337983009	0,448239995
CCDC25	protein_coding	ENSG00000147419	0,045079393	0,338044534	0,448292602
ATG16L2	protein_coding	ENSG00000168010	-0,113363581	0,338105755	0,448344801
AL157911.1	antisense	ENSG00000258553	0,259207745	0,338162993	0,448391711
AC006213.3	lincRNA	ENSG00000267058	-0,188770304	0,338394161	0,448669226
BEX2	protein_coding	ENSG00000133134	0,215326312	0,338456085	0,44872232
AC007216.3	sense_intronic	ENSG00000261560	-0,260183097	0,338565774	0,448838732
GGNBP1	transcribed_unitary_pse	ENSG00000204188	0,23569823	0,338691169	0,448975949
ZBTB41	protein_coding	ENSG00000177888	0,067936755	0,338751637	0,449027084
DLST	protein_coding	ENSG00000119689	-0,045163337	0,338788773	0,449047288
PPARA	protein_coding	ENSG00000186951	0,083213156	0,339567017	0,450049726
AC091152.2	antisense	ENSG00000267160	0,1743811	0,339621362	0,450092668
CAPN15	protein_coding	ENSG00000103326	-0,095153718	0,339784384	0,450279622
Z94721.1	bidirectional_promoter_	ENSG00000227598	0,262070555	0,339944776	0,450463067
AL513365.2	antisense	ENSG00000225891	0,230005326	0,34003644	0,450526317
DSCR9	lincRNA	ENSG00000230366	0,235017635	0,340030092	0,450526317

RPL21P44	processed_pseudogene	ENSG00000229585	0,193664274	0,340077709	0,450551891
SLC25A39	protein_coding	ENSG00000013306	-0,059579918	0,340106286	0,450560647
AP006222.1	transcribed_processed_	ENSG00000228463	-0,238748054	0,340260955	0,450736433
ZNF710	protein_coding	ENSG00000140548	0,087379828	0,340354267	0,450830924
PHTF2	protein_coding	ENSG00000006576	-0,05139177	0,340501229	0,450972818
TYMP	protein_coding	ENSG00000025708	-0,10599944	0,340505366	0,450972818
CD2AP	protein_coding	ENSG00000198087	0,056527886	0,340566549	0,451024725
CAPSL	protein_coding	ENSG00000152611	0,254874692	0,34063102	0,45108098
AC005696.3	lincRNA	ENSG00000272911	-0,20897007	0,340770809	0,45112046
ACOT1	protein_coding	ENSG00000184227	0,127556338	0,340764611	0,45112046
STAP2	protein_coding	ENSG00000178078	0,086926338	0,340766549	0,45112046
MBIP	protein_coding	ENSG00000151332	0,075360974	0,340746592	0,45112046
GNL1	protein_coding	ENSG00000204590	0,073919745	0,340751932	0,45112046
AL024498.1	lincRNA	ENSG00000272097	-0,231766764	0,340862006	0,451194006
RNF5P1	processed_pseudogene	ENSG00000253570	-0,204219367	0,340888967	0,451194006
ASXL2	protein_coding	ENSG00000143970	0,058419311	0,34091436	0,451194006
AL160291.1	lincRNA	ENSG00000262412	0,25721634	0,341090664	0,451373849
CCNO	protein_coding	ENSG00000152669	-0,159777203	0,341094261	0,451373849
MAGEA6	protein_coding	ENSG00000197172	-0,060720381	0,341118762	0,451377149
GNB3	protein_coding	ENSG00000111664	-0,223377097	0,341212894	0,451459832
AC012181.2	sense_intronic	ENSG00000261270	0,25354571	0,341225271	0,451459832
PLAC1	protein_coding	ENSG00000170965	0,140545884	0,341268333	0,451487681
SMC2-AS1	lincRNA	ENSG00000270332	0,256251611	0,341294927	0,451493741
MED14OS	antisense	ENSG00000234636	0,256551401	0,34167062	0,451961588
ZNF865	protein_coding	ENSG00000261221	0,132424862	0,341701872	0,451973777
NEMP2	protein_coding	ENSG00000189362	0,037614855	0,341756888	0,452017397
AC067838.1	lincRNA	ENSG00000272338	-0,239169219	0,341791318	0,452033784
TBXAS1	protein_coding	ENSG00000059377	0,242601894	0,341914466	0,452167496
AMT	protein_coding	ENSG00000145020	-0,220967712	0,342008374	0,452262524
TSTD2	protein_coding	ENSG00000136925	0,067498595	0,342060217	0,452301918
SLC43A1	protein_coding	ENSG00000149150	-0,126465694	0,342168542	0,452415987
ZNF333	protein_coding	ENSG00000160961	-0,082493942	0,342244997	0,452487907
SNHG6	processed_transcript	ENSG00000245910	-0,06516585	0,34227039	0,45249231
AC006213.2	antisense	ENSG00000266921	0,204489646	0,342411734	0,452649995
IQGAP1	protein_coding	ENSG00000140575	0,04062721	0,342629356	0,452908488
SELENOI	protein_coding	ENSG00000138018	-0,040556906	0,342905301	0,453244037
AC022182.1	lincRNA	ENSG00000254777	0,235319119	0,343014524	0,453359189
ZNF431	protein_coding	ENSG00000196705	0,077633002	0,343150681	0,453509921
SNRPN	protein_coding	ENSG00000128739	0,158815747	0,343443709	0,453849135
SAFB2	protein_coding	ENSG00000130254	0,075029081	0,343451606	0,453849135

AC015819.2	lincRNA	ENSG00000278607	0,256598442	0,343549793	0,453949636
AC239799.1	lincRNA	ENSG00000276509	0,229477034	0,343632492	0,454009103
RN7SL2	misc_RNA	ENSG00000274012	-0,176547132	0,343639069	0,454009103
ZDHHC4	protein_coding	ENSG00000136247	0,048415778	0,344080286	0,454504197
HSBP1L1	protein_coding	ENSG00000226742	-0,069441548	0,344058409	0,454504197
AC121320.1	sense_intronic	ENSG00000266237	-0,22388937	0,344049386	0,454504197
AP006621.2	lincRNA	ENSG00000255142	-0,246907935	0,344329658	0,454804307
RAD52	protein_coding	ENSG00000002016	0,073628207	0,344512464	0,455011289
INTS7	protein_coding	ENSG00000143493	-0,052729341	0,344530732	0,455011289
AC005070.3	antisense	ENSG00000272918	0,258133134	0,34458574	0,455054635
NCBP3	protein_coding	ENSG00000074356	0,052579134	0,344635153	0,455090587
AL109763.1	sense_overlapping	ENSG00000228708	-0,248340628	0,344800888	0,455280127
AC087392.4	antisense	ENSG00000262434	-0,197547826	0,344952562	0,455451078
COL24A1	protein_coding	ENSG00000171502	0,245172679	0,345017645	0,455478366
AC253536.3	sense_intronic	ENSG00000232545	-0,257347165	0,344997269	0,455478366
AC105020.6	antisense	ENSG00000275454	0,258169056	0,34506164	0,455507127
DGKZ	protein_coding	ENSG00000149091	0,069168917	0,345291951	0,45578182
AF250324.1	lincRNA	ENSG00000272566	0,255309453	0,345383843	0,455873776
AC127502.1	transcribed_unprocesse	ENSG00000215302	-0,156587853	0,345566284	0,456085231
PPP2R5E	protein_coding	ENSG00000154001	0,042980344	0,345603993	0,45610565
BLZF2P	unprocessed_pseudoge	ENSG00000258565	0,24904109	0,345855729	0,456408506
STX18	protein_coding	ENSG00000168818	0,060306264	0,346014882	0,456583427
AC073896.4	sense_overlapping	ENSG00000258199	-0,137857032	0,346032803	0,456583427
CELSR3	protein_coding	ENSG00000008300	0,089701063	0,346084329	0,456622038
AL136295.6	lincRNA	ENSG00000276698	0,220597378	0,346223289	0,456775997
LSP1P4	transcribed_unprocesse	ENSG00000143429	0,258396989	0,346433438	0,457023851
AL136987.1	lincRNA	ENSG00000236069	0,209832588	0,346519074	0,457107423
RCN1	protein_coding	ENSG00000049449	0,04112915	0,346583564	0,457163091
EIF3FP3	processed_pseudogene	ENSG00000233426	0,15457406	0,346759739	0,457307243
HLCS	protein_coding	ENSG00000159267	-0,063288651	0,346727504	0,457307243
ARNT2	protein_coding	ENSG00000172379	-0,127394267	0,346739916	0,457307243
GSTT2B	protein_coding	ENSG00000133433	0,255655011	0,346910009	0,457476003
EIF3K	protein_coding	ENSG00000178982	-0,061439154	0,347022682	0,457595165
RBM7	protein_coding	ENSG00000076053	0,077242838	0,347149411	0,457732847
CASP8	protein_coding	ENSG00000064012	0,039283465	0,347202184	0,45775764
GPATCH3	protein_coding	ENSG00000198746	-0,077983196	0,347212852	0,45775764
TSPYL1	protein_coding	ENSG00000189241	-0,055189126	0,347452286	0,458014422
PANX2	protein_coding	ENSG00000073150	-0,138199372	0,347437142	0,458014422
TMEM151A	protein_coding	ENSG00000179292	-0,193535518	0,347593686	0,458171369
LGMNP1	processed_pseudogene	ENSG00000214269	0,224697757	0,347672749	0,458187244

AC117382.1	processed_pseudogene	ENSG00000240695	-0,249548611	0,347668504	0,458187244
ZNF876P	transcribed_unprocesse	ENSG00000198155	-0,258350295	0,347641564	0,458187244
LINC01637	lincRNA	ENSG00000237476	-0,228008976	0,347838959	0,458349446
AC090970.2	sense_intronic	ENSG00000274528	0,181173508	0,347840523	0,458349446
AC008957.1	antisense	ENSG00000250155	-0,233258687	0,347885019	0,458378629
PRR13P5	processed_pseudogene	ENSG00000187534	0,252375142	0,348199509	0,458734065
AL353804.1	lincRNA	ENSG00000228906	-0,223941134	0,348449134	0,458990885
AHI1	protein_coding	ENSG00000135541	0,064928193	0,348431107	0,458990885
PYROXD1	protein_coding	ENSG00000121350	-0,088117227	0,348461583	0,458990885
AC098591.2	processed_pseudogene	ENSG00000250568	-0,211248505	0,348536529	0,459060122
TNFRSF17	protein_coding	ENSG00000048462	0,226642398	0,348739222	0,459268103
MVD	protein_coding	ENSG00000167508	0,070469464	0,348727399	0,459268103
AL049697.1	protein_coding	ENSG00000213204	-0,232315501	0,348786405	0,459300749
AC020658.1	processed_pseudogene	ENSG00000244705	-0,217352187	0,348910363	0,459434486
RNF139-AS1	lincRNA	ENSG00000245149	-0,242680539	0,349010836	0,459507784
GTF3C3	protein_coding	ENSG00000119041	-0,046633605	0,348991266	0,459507784
NXT2	protein_coding	ENSG00000101888	-0,091212591	0,349057171	0,459539289
AC015849.2	lincRNA	ENSG00000270829	0,208569766	0,349243919	0,459755634
TSPY26P	transcribed_processed_	ENSG00000235217	0,242360003	0,349295324	0,459793793
PRKCZ-AS1	antisense	ENSG00000182873	-0,250911564	0,349540086	0,460086457
TPMTP1	processed_pseudogene	ENSG00000267156	0,217306749	0,349841994	0,460454297
HES7	protein_coding	ENSG00000179111	-0,127230614	0,349956751	0,460575781
TM4SF19-AS1	antisense	ENSG00000235897	-0,247788994	0,350122392	0,460764213
CD28	protein_coding	ENSG00000178562	0,241027656	0,350234118	0,460881673
SNORD62A	snoRNA	ENSG00000235284	-0,251124541	0,350267033	0,460895416
FAM9B	protein_coding	ENSG00000177138	0,21576038	0,350306134	0,460917296
CYB5A	protein_coding	ENSG00000166347	-0,094306479	0,350334306	0,460924795
AL137779.2	antisense	ENSG00000259088	0,230846688	0,350393852	0,460944
FAM133CP	processed_pseudogene	ENSG00000183055	0,199898637	0,350393246	0,460944
ZNF664	protein_coding	ENSG00000179195	0,049391187	0,350510032	0,461067262
RPS7P1	processed_pseudogene	ENSG00000263266	-0,153154215	0,350699621	0,461287066
AC073263.2	lincRNA	ENSG00000279070	-0,251627272	0,350970303	0,461554303
FCF1P7	processed_pseudogene	ENSG00000224727	0,249890238	0,350927715	0,461554303
PFKP	protein_coding	ENSG00000067057	-0,033664709	0,350951346	0,461554303
STOX1	protein_coding	ENSG00000165730	-0,195277943	0,350993235	0,461554867
LINC02268	lincRNA	ENSG00000248174	0,248752094	0,351104684	0,461662911
SRF	protein_coding	ENSG00000112658	0,066332922	0,351120417	0,461662911
MRPL39	protein_coding	ENSG00000154719	-0,057280759	0,351156675	0,461680987
CCDC97	protein_coding	ENSG00000142039	-0,100971393	0,351216685	0,461730288
AL133375.1	antisense	ENSG00000245261	0,236543572	0,351285312	0,461790909

AC118754.1	protein_coding	ENSG00000183018	0,166625769	0,351314177	0,461799255
UBLCP1	protein_coding	ENSG00000164332	0,073021975	0,351483015	0,461991582
REEP6	protein_coding	ENSG00000115255	-0,102138826	0,351944532	0,462568558
HSF2BP	protein_coding	ENSG00000160207	-0,107308711	0,352067627	0,462671047
RNU6-3P	snRNA	ENSG00000207041	0,246893487	0,352052392	0,462671047
MRPL32	protein_coding	ENSG00000106591	-0,076637512	0,352094962	0,462677323
AC127537.1	antisense	ENSG00000260647	0,252148872	0,352468993	0,463079819
EIF4BP7	processed_pseudogene	ENSG00000225031	-0,230997064	0,352463449	0,463079819
TBX6	protein_coding	ENSG00000149922	-0,254004047	0,352457143	0,463079819
TIGD2	protein_coding	ENSG00000180346	-0,066932429	0,352553547	0,463161238
AC124319.3	antisense	ENSG00000263069	0,253160185	0,352601521	0,463178689
OSTCP8	processed_pseudogene	ENSG00000226801	0,194975831	0,352611996	0,463178689
AC092354.2	lincRNA	ENSG00000272370	-0,254283964	0,352641167	0,463187342
FAM177A1	protein_coding	ENSG00000151327	0,054612688	0,352765185	0,463320565
AC096649.1	antisense	ENSG00000229750	0,226634696	0,352834525	0,463381962
BX679664.3	processed_pseudogene	ENSG00000244716	-0,194578911	0,352959585	0,463516525
ZNF2	protein_coding	ENSG00000275111	-0,080823029	0,353021005	0,463567502
AL132780.5	TEC	ENSG00000280129	-0,250768373	0,353161904	0,463722832
OR10A6	protein_coding	ENSG00000279000	0,240490192	0,353480873	0,464111946
ANGPT1	protein_coding	ENSG00000154188	0,123354622	0,353587337	0,464222013
ZNF598	protein_coding	ENSG00000167962	0,078297246	0,353863478	0,464554818
PTCHD1	protein_coding	ENSG00000165186	-0,126113008	0,354539384	0,465412364
CCDC163	protein_coding	ENSG00000280670	0,122273732	0,354592559	0,465452377
CYP4F26P	lincRNA	ENSG00000226562	-0,222207772	0,354929068	0,465864276
C7orf43	protein_coding	ENSG00000146826	0,115416236	0,354959011	0,465873765
THAP6	protein_coding	ENSG00000174796	0,084179581	0,355057767	0,465936876
MCOLN1	protein_coding	ENSG00000090674	0,075341327	0,355077471	0,465936876
CUEDC2	protein_coding	ENSG00000107874	-0,069423483	0,355097967	0,465936876
GRAMD1A	protein_coding	ENSG00000089351	-0,072935466	0,35508249	0,465936876
SLC30A3	protein_coding	ENSG00000115194	-0,22587284	0,355168616	0,465999764
RASA4	protein_coding	ENSG00000105808	-0,213765192	0,355413469	0,466291195
GLA	protein_coding	ENSG00000102393	-0,057546223	0,355586043	0,466487766
SYPL1	protein_coding	ENSG00000008282	-0,051333477	0,35563003	0,466515632
MUS81	protein_coding	ENSG00000172732	-0,05765195	0,355665902	0,466532851
AC010247.1	processed_transcript	ENSG00000254887	0,219550731	0,355730949	0,466588332
RCBTB1	protein_coding	ENSG00000136144	-0,049751581	0,355801227	0,46665067
FAU	protein_coding	ENSG00000149806	-0,064977249	0,355892697	0,466740791
AC015987.1	antisense	ENSG00000224746	0,20939755	0,355944978	0,466779508
LINC02585	antisense	ENSG00000228350	0,251213352	0,35618511	0,467064549
CHRNA9	protein_coding	ENSG00000174343	0,234302394	0,356210547	0,467068043

AC068587.4	processed_transcript	ENSG00000283674	-0,232275964	0,356310414	0,467169123
RTBDN	protein_coding	ENSG00000132026	0,132737386	0,356506662	0,467306937
TPRA1	protein_coding	ENSG00000163870	0,075565336	0,35646794	0,467306937
TAF15	protein_coding	ENSG00000270647	-0,084506987	0,35649076	0,467306937
AC007406.5	sense_overlapping	ENSG00000261799	0,177314261	0,35645153	0,467306937
MPC1	protein_coding	ENSG00000060762	0,083176217	0,356616505	0,467421045
AP000437.1	TEC	ENSG00000279549	0,232552814	0,356648122	0,467432615
BRCC3	protein_coding	ENSG00000185515	-0,054709254	0,356673231	0,467435652
HOXA-AS2	antisense	ENSG00000253552	0,250619316	0,356709307	0,467453062
AL136531.1	antisense	ENSG00000229728	-0,203368017	0,356741975	0,467466004
AC005332.1	antisense	ENSG00000265100	-0,246033059	0,356774588	0,467478873
RAPGEF6	protein_coding	ENSG00000158987	-0,073740057	0,356850281	0,467548183
GCFC2	protein_coding	ENSG00000005436	0,057052443	0,356935037	0,467596744
KIFC3	protein_coding	ENSG00000140859	-0,067018087	0,35691661	0,467596744
BNIP3P1	transcribed_processed_	ENSG00000197358	0,251753075	0,356955739	0,467596744
RNF167	protein_coding	ENSG00000108523	0,062732729	0,357048357	0,467688198
AL365226.1	antisense	ENSG00000224349	-0,197264262	0,357118319	0,467720097
MFSD6	protein_coding	ENSG00000151690	0,211323713	0,357107475	0,467720097
BMPR1AP1	processed_pseudogene	ENSG00000219642	-0,210020786	0,357238192	0,46784722
AC008429.1	antisense	ENSG00000204758	-0,129895497	0,357578999	0,468263649
UTP15	protein_coding	ENSG00000164338	-0,051759225	0,357638149	0,468311206
TBC1D32	protein_coding	ENSG00000146350	-0,091391128	0,357739384	0,468413865
AP003392.5	lincRNA	ENSG00000271751	0,249560642	0,357896702	0,468578366
MYRF	protein_coding	ENSG00000124920	-0,160958478	0,357910711	0,468578366
AL109917.1	antisense	ENSG00000231050	0,22854795	0,358037914	0,468714982
SEMA6C	protein_coding	ENSG00000143434	0,194550049	0,358144133	0,46882411
AL353194.1	antisense	ENSG00000229539	-0,20345493	0,358322992	0,46900688
AC144652.1	lincRNA	ENSG00000273117	0,122814146	0,358329489	0,46900688
SMARCE1P5	processed_pseudogene	ENSG00000230793	0,251047987	0,358447011	0,469130763
HERC2P3	transcribed_unprocesse	ENSG00000180229	0,161012465	0,358516614	0,469191919
AL139353.1	protein_coding	ENSG00000203546	0,23118489	0,358642877	0,469327213
RIMKLA	protein_coding	ENSG00000177181	0,210120417	0,358748626	0,469435646
CHIC1	protein_coding	ENSG00000204116	0,073189925	0,359138877	0,469916323
KRT18P31	processed_pseudogene	ENSG00000249850	0,248742192	0,359241133	0,470020135
ZBTB17	protein_coding	ENSG00000116809	0,069019129	0,35929257	0,470057447
NSA2	protein_coding	ENSG00000164346	-0,063834969	0,359349804	0,47010234
PNKP	protein_coding	ENSG00000039650	-0,064216737	0,359388892	0,470123489
NUDT17	protein_coding	ENSG00000186364	-0,105720807	0,35981241	0,470647483
AP2S1	protein_coding	ENSG00000042753	-0,060684197	0,360033466	0,470906599
GUSBP1	transcribed_unprocesse	ENSG00000183666	0,075965492	0,360074918	0,470930784

AL356488.3	antisense	ENSG00000273382	0,183867916	0,360151934	0,471001477
YWHAB	protein_coding	ENSG00000166913	0,04777403	0,360429685	0,471334662
ZNF630	protein_coding	ENSG00000221994	0,223837027	0,360541316	0,471450583
RAB20	protein_coding	ENSG00000139832	-0,21232864	0,360598186	0,471494886
RPL3P7	processed_pseudogene	ENSG00000225093	-0,245948216	0,3608479	0,47179132
AC025048.4	lincRNA	ENSG00000267416	0,236339111	0,360943308	0,47188598
OGFRP1	lincRNA	ENSG00000182057	0,214246126	0,360982623	0,471898958
SNORA11F	snoRNA	ENSG00000221164	-0,20155179	0,360999252	0,471898958
EPHX3	protein_coding	ENSG00000105131	0,239776158	0,361418926	0,472345927
KRT13	protein_coding	ENSG00000171401	0,232000784	0,3614333	0,472345927
SRD5A1	protein_coding	ENSG00000145545	0,05262014	0,36141501	0,472345927
SELENOH	protein_coding	ENSG00000211450	-0,081076542	0,361386803	0,472345927
LHX9	protein_coding	ENSG00000143355	-0,199060364	0,361466702	0,472359482
OBSCN-AS1	antisense	ENSG00000162913	-0,241735702	0,361512065	0,472388663
RPSAP54	processed_pseudogene	ENSG00000213621	0,247909272	0,36157147	0,472406094
RPSAP47	processed_pseudogene	ENSG00000188856	-0,231596191	0,361560576	0,472406094
JUN	protein_coding	ENSG00000177606	-0,057992542	0,361714249	0,472562536
AC060780.2	processed_pseudogene	ENSG00000267340	0,226854287	0,361784933	0,472624775
SNORA22C	snoRNA	ENSG00000207344	0,245522577	0,36186436	0,472698428
AC232271.1	lincRNA	ENSG00000270012	-0,239144568	0,362162129	0,473050336
HNMT	protein_coding	ENSG00000150540	0,219978878	0,362179885	0,473050336
AC021086.1	lincRNA	ENSG00000247877	0,248766774	0,362218506	0,473070654
AC011912.1	lincRNA	ENSG00000279145	0,145588943	0,362371996	0,4731506
AL513175.2	processed_pseudogene	ENSG00000236792	-0,204387975	0,362322363	0,4731506
BMP10	protein_coding	ENSG00000163217	0,213239006	0,36236673	0,4731506
GSK3A	protein_coding	ENSG00000105723	0,073726522	0,362339732	0,4731506
HOGA1	protein_coding	ENSG00000241935	0,221635889	0,362444464	0,473215097
AC010478.1	antisense	ENSG00000259663	0,213952884	0,362581621	0,473364038
RNF125	protein_coding	ENSG00000101695	-0,207263614	0,362649699	0,473422782
COX10	protein_coding	ENSG00000006695	-0,052370055	0,362736148	0,473498485
AL445222.1	sense_intronic	ENSG00000271833	0,248164155	0,362753861	0,473498485
GSTK1	protein_coding	ENSG00000197448	0,055026475	0,362904597	0,473637103
LAGE3	protein_coding	ENSG00000196976	-0,081634533	0,362906244	0,473637103
RF00086	snoRNA	ENSG00000252128	-0,192718801	0,362939281	0,47365008
NMB	protein_coding	ENSG00000197696	0,217164414	0,36302403	0,473730537
SCO2	protein_coding	ENSG00000130489	-0,247262362	0,363217301	0,473922441
PTMAP5	transcribed_processed_	ENSG00000214182	0,15818691	0,36321566	0,473922441
HSPA8P8	processed_pseudogene	ENSG00000229091	-0,246596584	0,363305915	0,474007908
CLMN	protein_coding	ENSG00000165959	0,135415198	0,363393094	0,474091493
AC107952.2	lincRNA	ENSG00000272343	0,207979162	0,363424061	0,474101736

TMEM63C	protein_coding	ENSG00000165548	0,231481004	0,363531823	0,474212153
MAN2C1	protein_coding	ENSG00000140400	-0,081051433	0,363626266	0,474305184
VPS13A	protein_coding	ENSG00000197969	-0,05902721	0,363659654	0,474318569
MTMR4	protein_coding	ENSG00000108389	-0,046336201	0,363735603	0,474387461
AL512506.1	sense_intronic	ENSG00000274001	0,241509365	0,363934001	0,474616034
AC090159.1	lincRNA	ENSG00000255094	-0,233944506	0,363992745	0,474662462
SYNE2	protein_coding	ENSG00000054654	-0,125996048	0,364207541	0,47491237
EFR3A	protein_coding	ENSG00000132294	0,044421266	0,36424735	0,474934085
HAUS6P1	processed_pseudogene	ENSG00000227344	-0,24610315	0,364647298	0,475425344
CCT8P1	processed_pseudogene	ENSG00000226015	-0,227455817	0,364818228	0,475590892
TRPV3	protein_coding	ENSG00000167723	0,162801887	0,364843837	0,475590892
NDUFA5	protein_coding	ENSG00000128609	-0,07831268	0,364828071	0,475590892
TOM1L1	protein_coding	ENSG00000141198	0,046970516	0,364893377	0,47562524
TRMT10A	protein_coding	ENSG00000145331	-0,102142803	0,365033784	0,475778019
FKBP3	protein_coding	ENSG00000100442	-0,049944047	0,365134812	0,475879456
CTBP2	protein_coding	ENSG00000175029	0,03475293	0,365198615	0,475932367
AC090527.3	lincRNA	ENSG00000275709	0,237298001	0,365228332	0,475940853
QRICH2	protein_coding	ENSG00000129646	-0,131390208	0,365383063	0,476112238
ELL3	protein_coding	ENSG00000128886	-0,244568265	0,365707985	0,476505354
BMS1	protein_coding	ENSG00000165733	-0,059509031	0,365990993	0,476843811
ITFG2-AS1	antisense	ENSG00000258325	-0,202717641	0,366056182	0,47689845
ATP6V0C	protein_coding	ENSG00000185883	-0,216900191	0,366081218	0,476900774
CCDC6	protein_coding	ENSG00000108091	0,040634785	0,366160567	0,476973848
PDE7A	protein_coding	ENSG00000205268	-0,069286545	0,366325807	0,47715879
AP003119.2	antisense	ENSG00000255100	-0,244462047	0,366421918	0,477223364
POLR3GP1	transcribed_processed_	ENSG00000268295	-0,236793056	0,366413248	0,477223364
SNRK	protein_coding	ENSG00000163788	-0,054597635	0,366490414	0,477282266
MSANTD1	protein_coding	ENSG00000188981	0,244996663	0,36656501	0,477349102
GAREM1	protein_coding	ENSG00000141441	-0,118054655	0,366625197	0,477397167
GABARAP	protein_coding	ENSG00000170296	0,124661662	0,366851064	0,477660953
C20orf96	protein_coding	ENSG00000196476	-0,188652797	0,367198357	0,478082797
SCN1A	protein_coding	ENSG00000144285	0,205338318	0,367239558	0,47810609
RPL7P23	processed_pseudogene	ENSG00000244363	0,245853085	0,367431774	0,478313017
RMND5A	protein_coding	ENSG00000153561	0,051218983	0,367445143	0,478313017
BUD23	protein_coding	ENSG00000071462	0,058765005	0,367595611	0,478478516
FAM78B	protein_coding	ENSG00000188859	-0,159156794	0,367680822	0,478559059
AC068205.2	processed_transcript	ENSG00000283341	0,194804189	0,368168682	0,479163632
CCDC183-AS1	antisense	ENSG00000228544	0,199292095	0,368235063	0,479219615
ZNF646	protein_coding	ENSG00000167395	-0,090663898	0,36830882	0,479285191
AL031719.2	TEC	ENSG00000280231	-0,207109318	0,368367883	0,479331639

TNFRSF10C	protein_coding	ENSG00000173535	0,0948481	0,368596872	0,479599178
UBXN1	protein_coding	ENSG00000162191	0,051957814	0,368647422	0,479634523
CRTC1	protein_coding	ENSG00000105662	0,105230213	0,36885854	0,479878761
EXOSC3P1	processed_pseudogene	ENSG00000229007	-0,231382586	0,368923231	0,479932479
CAST	protein_coding	ENSG00000153113	0,040887213	0,369197558	0,48025889
CCDC113	protein_coding	ENSG00000103021	0,083167388	0,369232845	0,480274331
AL162741.1	lincRNA	ENSG00000260179	0,224511444	0,369311559	0,480322756
STK11	protein_coding	ENSG00000118046	0,0711162	0,369316912	0,480322756
KRT18P4	processed_pseudogene	ENSG00000229222	0,241912747	0,369579092	0,480633262
LINC01670	lincRNA	ENSG00000279094	-0,201448541	0,369624043	0,480657049
RPL17P34	processed_pseudogene	ENSG00000213432	-0,205441393	0,369644254	0,480657049
CRIPT	protein_coding	ENSG00000119878	-0,076129686	0,369747522	0,480760852
TRIM33	protein_coding	ENSG00000197323	-0,063590954	0,369776657	0,480768255
UNC5C	protein_coding	ENSG00000182168	0,210913279	0,369857971	0,480841225
RNF31	protein_coding	ENSG00000092098	0,076428751	0,369879669	0,480841225
GFI1	protein_coding	ENSG00000162676	-0,115409965	0,370058275	0,481012436
AC008147.3	transcribed_processed_	ENSG00000257531	0,225672441	0,370045124	0,481012436
EEF1A1P11	processed_pseudogene	ENSG00000228502	-0,188388016	0,370187739	0,481150223
ZNF628	protein_coding	ENSG00000197483	-0,114163068	0,370283221	0,481243829
ST13P6	processed_pseudogene	ENSG00000225259	0,220349212	0,370414687	0,48132319
AL117339.5	protein_coding	ENSG00000283930	0,217677175	0,370367806	0,48132319
NDUFAF8	protein_coding	ENSG00000224877	-0,073249226	0,370391589	0,48132319
DAAM2	protein_coding	ENSG00000146122	0,186692816	0,370444332	0,481331216
NALT1	antisense	ENSG00000237886	-0,244885316	0,37048773	0,481357111
SLC28A2	protein_coding	ENSG00000137860	0,189313752	0,370535227	0,481388326
GNA13	protein_coding	ENSG00000120063	-0,047900865	0,370675321	0,48153983
AC004982.1	lincRNA	ENSG00000272732	0,233343991	0,370776292	0,481541975
CYB561A3	protein_coding	ENSG00000162144	-0,060285854	0,370794364	0,481541975
AC079414.3	sense_intronic	ENSG00000276007	-0,193258017	0,370782037	0,481541975
AL451074.1	transcribed_processed_	ENSG00000215838	0,201724568	0,370754398	0,481541975
SRGAP2D	unprocessed_pseudoge	ENSG00000270872	-0,145056621	0,370719581	0,481541975
RPL23AP34	processed_pseudogene	ENSG00000225991	0,2225149	0,371161346	0,481988046
TMEM191C	protein_coding	ENSG00000206140	0,231493433	0,371237024	0,4820558
APLP1	protein_coding	ENSG00000105290	0,066697935	0,37162507	0,482529133
PABPC3	protein_coding	ENSG00000151846	0,242314488	0,371850146	0,482788313
MITF	protein_coding	ENSG00000187098	-0,072630574	0,371871759	0,482788313
SRCAP	protein_coding	ENSG00000080603	-0,178762467	0,371912908	0,482811174
AC008764.8	antisense	ENSG00000279529	0,228855676	0,37194568	0,482823158
RPL13AP7	processed_pseudogene	ENSG00000213885	-0,240257294	0,37210045	0,482966919
GHR	protein_coding	ENSG00000112964	-0,066576644	0,372103523	0,482966919

CYBC1	protein_coding	ENSG00000178927	-0,055366167	0,372158026	0,483007095
PLAC8	protein_coding	ENSG00000145287	-0,066979472	0,37230993	0,483173669
AC012066.1	processed_pseudogene	ENSG00000213197	0,236593667	0,372452425	0,483328013
TIRAP	protein_coding	ENSG00000150455	-0,137753139	0,37248564	0,483340534
NMI	protein_coding	ENSG00000123609	0,083018899	0,37251966	0,483354097
AC092535.3	antisense	ENSG00000251652	-0,241739734	0,3726059	0,483379902
ARTN	protein_coding	ENSG00000117407	0,24346638	0,372592482	0,483379902
COL1A2	protein_coding	ENSG00000164692	0,234380936	0,372610251	0,483379902
AC009032.1	sense_intronic	ENSG00000274093	0,213024699	0,372760831	0,483544661
ANKHD1-EIF4EBP3	protein_coding	ENSG00000254996	-0,228860053	0,372936124	0,483741456
AC096642.1	lincRNA	ENSG00000277007	-0,239960374	0,372972172	0,483757621
RPS19	protein_coding	ENSG00000105372	-0,066986876	0,373203492	0,48402704
NPM1P19	processed_pseudogene	ENSG00000223864	0,206296191	0,373236397	0,484039108
TXNDC16	protein_coding	ENSG00000087301	0,069390366	0,373524143	0,484381649
LATS1	protein_coding	ENSG00000131023	0,052096748	0,373678897	0,484551696
ERP29	protein_coding	ENSG00000089248	-0,048688002	0,37374149	0,484602223
AL023803.1	antisense	ENSG00000261431	0,241670297	0,373816824	0,484629928
AC211476.2	antisense	ENSG00000272843	-0,213264467	0,373883019	0,484629928
RPS2P55	processed_pseudogene	ENSG00000216866	0,227640671	0,373869176	0,484629928
AC010733.1	processed_pseudogene	ENSG00000232713	0,215780885	0,373788996	0,484629928
SURF1	protein_coding	ENSG00000148290	0,066340027	0,373911579	0,484629928
ZNF227	protein_coding	ENSG00000131115	0,051300272	0,37392826	0,484629928
DNAJB1	protein_coding	ENSG00000132002	-0,033625574	0,373894689	0,484629928
HSD17B10	protein_coding	ENSG00000072506	-0,057642648	0,374039918	0,484713383
POM121	protein_coding	ENSG00000196313	-0,071552124	0,374022248	0,484713383
CHST2	protein_coding	ENSG00000175040	-0,068463974	0,374091593	0,48474972
RNY3	misc_RNA	ENSG00000202354	-0,219347646	0,374129037	0,484767614
AL121944.1	antisense	ENSG00000272009	-0,209982749	0,37419908	0,484774884
AC026471.2	antisense	ENSG00000260625	-0,240776746	0,3741926	0,484774884
PSMB4	protein_coding	ENSG00000159377	-0,049692149	0,374205557	0,484774884
FAM225A	lincRNA	ENSG00000231528	-0,166448569	0,374266667	0,484792809
TTLL7-IT1	sense_intronic	ENSG00000233061	0,214630506	0,374257465	0,484792809
RAPGEF1	protein_coding	ENSG00000107263	-0,063201855	0,37496147	0,485662124
GRM2	protein_coding	ENSG00000164082	-0,233692047	0,375236398	0,48598753
RPS3AP2	processed_pseudogene	ENSG00000219027	-0,198500946	0,37532358	0,48606975
AL157700.1	lincRNA	ENSG00000260118	0,228842925	0,375443371	0,486192277
RFX1	protein_coding	ENSG00000132005	-0,09091175	0,375465601	0,486192277
AC006262.2	lincRNA	ENSG00000269729	-0,227176528	0,375558214	0,4862815
AC023043.4	sense_intronic	ENSG00000274849	-0,232292571	0,375957871	0,486768255
AP006621.3	lincRNA	ENSG00000255284	-0,122580431	0,376380874	0,487254417

AP002833.1	lincRNA	ENSG00000254938	-0,229473186	0,3763749	0,487254417
EDC3	protein_coding	ENSG00000179151	-0,054092481	0,376464553	0,48730123
C16orf45	protein_coding	ENSG00000166780	-0,063418766	0,376442155	0,48730123
CCN5	protein_coding	ENSG00000064205	0,224348359	0,376547279	0,487377553
LIPT1	protein_coding	ENSG00000144182	-0,079750941	0,37658672	0,487397844
CDC42EP1	protein_coding	ENSG00000128283	-0,074589616	0,376679812	0,487487566
DST	protein_coding	ENSG00000151914	0,079758722	0,37671338	0,487500248
AC092718.7	TEC	ENSG00000278985	-0,221694821	0,376830299	0,487620784
AC012313.4	TEC	ENSG00000268543	-0,169393471	0,377014787	0,487797961
AC009090.5	TEC	ENSG00000279803	-0,226350252	0,377002102	0,487797961
PATJ	protein_coding	ENSG00000132849	0,069527923	0,377149016	0,487940851
DSTYK	protein_coding	ENSG00000133059	-0,05939353	0,37726797	0,488063962
SLC35E2B	protein_coding	ENSG00000189339	0,094281403	0,377316825	0,488096378
AC078795.3	antisense	ENSG00000270135	-0,224037969	0,377368092	0,48813191
RAB11FIP1	protein_coding	ENSG00000156675	0,055428731	0,377717395	0,488552928
RN7SL16P	misc_RNA	ENSG00000243005	-0,204060304	0,377840112	0,488650022
AC067945.3	processed_transcript	ENSG00000231858	0,213204908	0,377828884	0,488650022
AC010442.3	processed_pseudogene	ENSG00000250645	-0,195184463	0,378054888	0,488896958
CAB39L	protein_coding	ENSG00000102547	-0,072438907	0,378222726	0,489083167
OSR1	protein_coding	ENSG00000143867	0,08958271	0,378269184	0,489112405
AL163540.1	unprocessed_pseudoge	ENSG00000204790	0,188205487	0,378402701	0,489254202
DUSP8P5	processed_pseudogene	ENSG00000235316	-0,216293989	0,378530269	0,489357442
IQCB1	protein_coding	ENSG00000173226	-0,047493583	0,378515626	0,489357442
AC027796.1	sense_intronic	ENSG00000261916	-0,198647803	0,378725006	0,489578335
CYB5R3	protein_coding	ENSG00000100243	-0,054334486	0,378929403	0,48978082
AC017104.1	processed_transcript	ENSG00000224376	0,222656326	0,378988713	0,489826612
RNU6-341P	snRNA	ENSG00000252782	-0,232018523	0,379154915	0,490010542
AC092653.2	TEC	ENSG00000280037	0,2316239	0,379248263	0,490100301
AC003665.1	protein_coding	ENSG00000006025	0,075450076	0,379333004	0,490148045
PPIEL	transcribed_unprocesse	ENSG00000243970	0,208362421	0,379330579	0,490148045
AC093462.1	antisense	ENSG00000277324	0,233949524	0,379368266	0,490162727
AC079949.1	lincRNA	ENSG00000256001	0,239109034	0,379479062	0,490274996
PLSCR1	protein_coding	ENSG00000188313	0,056529757	0,379511428	0,490285927
AC005288.1	antisense	ENSG00000266469	0,219884454	0,37968078	0,490473817
TBX15	protein_coding	ENSG00000092607	-0,236395383	0,379753096	0,490536339
RNU6-80P	snRNA	ENSG00000206922	-0,191656733	0,379837332	0,49061425
GSTM1	protein_coding	ENSG00000134184	-0,065245114	0,379892234	0,490654265
METTL6	protein_coding	ENSG00000206562	-0,053958283	0,380155541	0,490963425
ABRACL	protein_coding	ENSG00000146386	-0,091462765	0,380278636	0,491091476
KIRREL1	protein_coding	ENSG00000183853	0,060869195	0,380488071	0,491300072

TMSB4X	protein_coding	ENSG00000205542	0,055769605	0,380487558	0,491300072
AC093278.2	sense_overlapping	ENSG00000261269	0,238301935	0,380559171	0,491360943
RECK	protein_coding	ENSG00000122707	0,084245918	0,380677861	0,491468948
ZRANB2	protein_coding	ENSG00000132485	-0,063758601	0,380690745	0,491468948
AC005726.3	sense_intronic	ENSG00000264608	-0,236441374	0,380770711	0,491541244
FALEC	lincRNA	ENSG00000228126	0,236181014	0,380888891	0,491662858
LZTR1	protein_coding	ENSG00000099949	-0,067828863	0,381106134	0,491881368
CCNJL	protein_coding	ENSG00000135083	-0,076355902	0,381091885	0,491881368
ZNF503-AS2	antisense	ENSG00000237149	-0,143069324	0,381208353	0,491935195
STPG2	protein_coding	ENSG00000163116	0,229267601	0,381217645	0,491935195
ACAA2	protein_coding	ENSG00000167315	-0,058821949	0,381219795	0,491935195
COG6	protein_coding	ENSG00000133103	0,054186054	0,381293553	0,491982149
HSPA6	protein_coding	ENSG00000173110	-0,173628537	0,381304156	0,491982149
AC005822.1	processed_pseudogene	ENSG00000235554	0,22440579	0,38153594	0,492188322
SLCO1B7	protein_coding	ENSG00000205754	0,203683908	0,381532592	0,492188322
COX5B	protein_coding	ENSG00000135940	-0,062158783	0,38151287	0,492188322
PI4KAP2	transcribed_unitary_pse	ENSG00000183506	0,109894844	0,381666687	0,492326022
PEPD	protein_coding	ENSG00000124299	0,047571888	0,381867061	0,492553514
IPO7P1	processed_pseudogene	ENSG00000232951	0,215497661	0,38206301	0,492652386
LHFPL6	protein_coding	ENSG00000183722	0,048283051	0,382085737	0,492652386
B3GALNT2	protein_coding	ENSG00000162885	0,036962508	0,382087836	0,492652386
MARF1	protein_coding	ENSG00000166783	-0,063060443	0,382078939	0,492652386
ATL1	protein_coding	ENSG00000198513	-0,120271848	0,382021787	0,492652386
PMS2P10	unprocessed_pseudoge	ENSG00000276840	0,235583671	0,382082419	0,492652386
OCEL1	protein_coding	ENSG00000099330	-0,131276816	0,382425748	0,493057084
CT45A11P	unprocessed_pseudoge	ENSG00000230162	0,233111009	0,382533131	0,49316453
HSPD1P5	processed_pseudogene	ENSG00000249193	-0,206633949	0,382760923	0,493427186
AL441883.1	antisense	ENSG00000261071	-0,212869734	0,382915388	0,493595287
ZNF410	protein_coding	ENSG00000119725	0,206089697	0,383123228	0,493832165
EEF1DP2	processed_pseudogene	ENSG00000226721	0,228620598	0,383155216	0,493842361
AC105339.2	processed_transcript	ENSG00000252690	0,169405758	0,383376555	0,494073339
PCDHGB7	protein_coding	ENSG00000254122	0,199031415	0,383382603	0,494073339
AC092675.1	antisense	ENSG00000222000	-0,133964271	0,383429573	0,494102823
HEXD	protein_coding	ENSG00000169660	0,101298898	0,383508995	0,494174121
TBX2	protein_coding	ENSG00000121068	-0,084187415	0,384222771	0,495062762
ENO2	protein_coding	ENSG00000111674	0,070832133	0,38433727	0,495148079
SCRIB	protein_coding	ENSG00000180900	-0,08019693	0,384330192	0,495148079
AL162311.3	antisense	ENSG00000258938	0,236047627	0,384511309	0,495293184
FLOT2	protein_coding	ENSG00000132589	0,056668906	0,384522349	0,495293184
FLNB	protein_coding	ENSG00000136068	-0,071850403	0,384508519	0,495293184

PIH1D2	protein_coding	ENSG00000150773	0,14123995	0,384547514	0,495294494
SVEP1	protein_coding	ENSG00000165124	-0,110022192	0,384801947	0,495591079
PRRT3	protein_coding	ENSG00000163704	0,141757121	0,385230796	0,496081097
KLHL22	protein_coding	ENSG00000099910	-0,065788059	0,385210927	0,496081097
ATPAF2	protein_coding	ENSG00000171953	-0,065922559	0,385265009	0,496094006
PCOTH	processed_transcript	ENSG00000205861	0,216895807	0,385423453	0,496259222
C1GALT1	protein_coding	ENSG00000106392	0,063997588	0,385441707	0,496259222
SATB2-AS1	antisense	ENSG00000225953	-0,192375429	0,385598305	0,49642968
DLGAP4-AS1	antisense	ENSG00000232907	0,234631875	0,385733988	0,496573192
ACTC1	protein_coding	ENSG00000159251	-0,18911289	0,385847919	0,496688685
AL031432.2	unprocessed_pseudoge	ENSG00000261349	-0,223822712	0,385976539	0,496823071
TMEM220	protein_coding	ENSG00000187824	0,145951903	0,386062589	0,496902648
AC068790.2	sense_intronic	ENSG00000269938	-0,233475033	0,386214731	0,497067278
AP001469.2	antisense	ENSG00000228137	-0,223330701	0,386386772	0,497257495
AC012360.1	lincRNA	ENSG00000235319	0,217972708	0,38672156	0,497657121
KIAA1549	protein_coding	ENSG00000122778	0,086668972	0,386758301	0,497673176
GOLGA6L5P	transcribed_unprocesse	ENSG00000230373	0,228269056	0,386801373	0,497697375
C11orf94	protein_coding	ENSG00000234776	0,209010199	0,387045307	0,497980004
AL139407.1	sense_intronic	ENSG00000277687	0,219940167	0,387143954	0,498075681
ZNF235	protein_coding	ENSG00000159917	0,082631373	0,387233574	0,498159732
AL118511.1	antisense	ENSG00000223393	0,205039061	0,387274996	0,498181772
AC090510.1	lincRNA	ENSG00000246283	0,2341948	0,387394477	0,498304216
AFDN-DT	lincRNA	ENSG00000198221	-0,23390038	0,387512859	0,498425231
FKBP1B	protein_coding	ENSG00000119782	-0,227313234	0,387655125	0,498576949
PLEKHH3	protein_coding	ENSG00000068137	0,091197305	0,387701846	0,498603084
AC092437.1	TEC	ENSG00000280310	0,203775846	0,387724066	0,498603084
AL591848.3	lincRNA	ENSG00000260698	0,229771926	0,387883617	0,498731187
MT1XP1	processed_pseudogene	ENSG00000233929	-0,204847466	0,387908413	0,498731187
RAB5B	protein_coding	ENSG00000111540	0,043722869	0,387920947	0,498731187
AC126755.1	transcribed_unprocesse	ENSG00000205746	0,180470989	0,387877126	0,498731187
EEF1A1P5	processed_pseudogene	ENSG00000196205	-0,069641101	0,388002107	0,498773
PLOD2	protein_coding	ENSG00000152952	-0,041833562	0,387994263	0,498773
PTGES	protein_coding	ENSG00000148344	0,131123947	0,388074147	0,498834342
AC007032.1	antisense	ENSG00000273320	0,204743534	0,388319271	0,499118146
AC090198.1	antisense	ENSG00000253106	-0,189677678	0,388425456	0,499223342
AC073842.2	antisense	ENSG00000242798	0,207177374	0,388738078	0,499499936
AC005005.3	antisense	ENSG00000273387	0,191648845	0,388685645	0,499499936
KRT18P5	processed_pseudogene	ENSG00000236670	-0,23130089	0,388710315	0,499499936
CYP51A1	protein_coding	ENSG00000001630	0,140455282	0,388721931	0,499499936
AC018638.2	processed_pseudogene	ENSG00000230715	0,223531544	0,388844247	0,499605056

AAMDC	protein_coding	ENSG00000087884	-0,074116705	0,389140197	0,499953987
GARS-DT	processed_transcript	ENSG00000196295	-0,080462295	0,389269492	0,500081644
LINC02614	processed_transcript	ENSG00000241288	-0,135363912	0,389288324	0,500081644
CAMSAP3	protein_coding	ENSG00000076826	0,230013134	0,389530493	0,500361396
SNORA5C	snoRNA	ENSG00000201772	-0,140003183	0,389564293	0,500373475
FAM110D	protein_coding	ENSG00000197245	0,230334998	0,389593984	0,500380275
CHM	protein_coding	ENSG00000188419	-0,054786546	0,389822678	0,500642651
AC096536.2	antisense	ENSG00000242396	0,195980146	0,390150499	0,501032292
AP001267.3	antisense	ENSG00000255435	0,230796006	0,390378599	0,501293831
NOC2LP2	processed_pseudogene	ENSG00000217950	-0,21384154	0,390601969	0,501549263
ZNF793	protein_coding	ENSG00000188227	0,230094425	0,390752183	0,501710734
AC005144.1	lincRNA	ENSG00000283517	-0,21396346	0,390811854	0,501724533
MICU2	protein_coding	ENSG00000165487	-0,062028455	0,390845175	0,501735904
TRIM4	protein_coding	ENSG00000146833	-0,052543704	0,390947002	0,501835212
TSC22D1	protein_coding	ENSG00000102804	0,035868146	0,391220803	0,502155245
AC233699.1	unprocessed_pseudoge	ENSG00000261499	0,224939774	0,391449694	0,502417598
CNIH4	protein_coding	ENSG00000143771	-0,048348421	0,391506702	0,502445422
SLC27A2	protein_coding	ENSG00000140284	-0,145567795	0,391520367	0,502445422
TECRP1	processed_pseudogene	ENSG00000235043	0,229236448	0,391635724	0,502562016
SMAD3	protein_coding	ENSG00000166949	0,05052194	0,391820914	0,502768203
CYP27A1	protein_coding	ENSG00000135929	0,22430315	0,391855415	0,502781017
ADGRL1	protein_coding	ENSG00000072071	0,081819351	0,392239999	0,503242986
APCDD1L-DT	processed_transcript	ENSG00000231290	0,232083561	0,392443764	0,503472922
SLC25A34-AS1	antisense	ENSG00000224459	0,191660512	0,39248488	0,503494176
BACE2-IT1	antisense	ENSG00000224388	-0,20350015	0,392730605	0,503777891
SERTAD4-AS1	antisense	ENSG00000203706	0,098342797	0,392792043	0,503825191
AC015813.1	lincRNA	ENSG00000264112	-0,103463524	0,392998279	0,503995491
HSP90AB1	protein_coding	ENSG00000096384	-0,030989598	0,392998532	0,503995491
SVIL2P	transcribed_unprocesse	ENSG00000234814	0,214066266	0,392961461	0,503995491
AC011939.2	sense_intronic	ENSG00000261544	0,197775808	0,393153664	0,504162914
AC083798.2	antisense	ENSG00000272758	-0,158068711	0,393204054	0,504196008
HMGB1P19	processed_pseudogene	ENSG00000253463	0,209450013	0,393313662	0,504305027
PDCD6IPP2	transcribed_unprocesse	ENSG00000261377	-0,164650333	0,393470008	0,504473956
OR10V3P	unprocessed_pseudoge	ENSG00000254743	-0,195349268	0,393498062	0,504478388
AC017002.1	lincRNA	ENSG00000224959	-0,125460383	0,393554119	0,504518719
PIN4P1	processed_pseudogene	ENSG00000227973	-0,22585401	0,393642956	0,504569529
TRAM2	protein_coding	ENSG00000065308	-0,057075777	0,393638241	0,504569529
C4orf47	protein_coding	ENSG00000205129	0,226229761	0,393673256	0,504576834
TRAPPC2B	protein_coding	ENSG00000256060	-0,101147565	0,393698407	0,504577537
AC080023.1	antisense	ENSG00000254554	0,22152546	0,393972749	0,504897593

AC004057.1	transcribed_processed_	ENSG00000196656	-0,209007521	0,394288661	0,50527088
PDXDC2P	transcribed_unprocesse	ENSG00000255185	-0,196269204	0,39432744	0,505289003
CU634019.6	lincRNA	ENSG00000280018	-0,229181092	0,394460319	0,505396123
TAOK1	protein_coding	ENSG00000160551	-0,054322687	0,394445473	0,505396123
PCGF3	protein_coding	ENSG00000185619	-0,046046251	0,394751816	0,505738007
AC025569.1	antisense	ENSG00000258168	0,218902256	0,394980256	0,505999067
AP001033.2	sense_intronic	ENSG00000273284	-0,227109312	0,39511354	0,5061382
SMKR1	protein_coding	ENSG00000240204	-0,23009681	0,39528562	0,506327009
MGME1	protein_coding	ENSG00000125871	-0,041162967	0,395312302	0,506329564
VDAC1P6	processed_pseudogene	ENSG00000231341	-0,18444107	0,395338814	0,506331902
MEF2C-AS1	antisense	ENSG00000248309	0,22873866	0,395458202	0,506453183
AC092868.1	processed_pseudogene	ENSG00000259674	-0,228553536	0,39567599	0,506700459
CNN2P9	processed_pseudogene	ENSG00000213149	-0,185460812	0,395787843	0,506812053
AL121983.2	lincRNA	ENSG00000227373	0,213153724	0,395848286	0,5068407
C1orf56	protein_coding	ENSG00000143443	-0,140979071	0,39588435	0,5068407
AC114490.1	transcribed_unitary_pse	ENSG00000241014	0,203621892	0,395877186	0,5068407
NFU1	protein_coding	ENSG00000169599	0,058094545	0,395982912	0,506935242
GAS2	protein_coding	ENSG00000148935	-0,187550854	0,396079805	0,507027637
AC004889.1	antisense	ENSG00000244198	-0,176362495	0,396566762	0,507524291
PMFBP1	protein_coding	ENSG00000118557	0,183719024	0,39651552	0,507524291
SDR39U1	protein_coding	ENSG00000100445	0,073917548	0,396518064	0,507524291
ADAM21P1	transcribed_processed_	ENSG00000235812	-0,19793062	0,396604251	0,5075406
AC032011.1	lincRNA	ENSG00000274340	0,208686176	0,396653587	0,507572066
CUL9	protein_coding	ENSG00000112659	0,06880158	0,396922117	0,507853368
LSM1	protein_coding	ENSG00000175324	-0,054390203	0,396922939	0,507853368
AC092614.1	lincRNA	ENSG00000227542	-0,217183065	0,396954145	0,507861612
LINC00294	lincRNA	ENSG00000280798	-0,071003972	0,397065351	0,507912982
ST13P15	processed_pseudogene	ENSG00000243759	-0,220288748	0,397068589	0,507912982
LINC01359	processed_transcript	ENSG00000226891	0,181833331	0,397057305	0,507912982
MSH3	protein_coding	ENSG00000113318	0,037804057	0,397140275	0,507972999
GAPDHP40	processed_pseudogene	ENSG00000248626	0,195566141	0,397346806	0,508205475
MED12	protein_coding	ENSG00000184634	-0,076475725	0,397493663	0,508361604
RNGTT	protein_coding	ENSG00000111880	-0,054676968	0,397525186	0,508370219
AC010642.2	antisense	ENSG00000283103	0,089084161	0,397762801	0,508642375
MYCBP2-AS1	processed_transcript	ENSG00000236051	0,206153403	0,397840439	0,508709938
ATP23	protein_coding	ENSG00000166896	-0,064198752	0,397956742	0,50882693
RPL35P1	processed_pseudogene	ENSG00000237991	0,226278335	0,398114243	0,508996579
SUMO1P3	processed_pseudogene	ENSG00000235082	0,223801883	0,398162549	0,509026609
AL606760.3	antisense	ENSG00000259818	0,215744778	0,398336628	0,509217417
CCDC120	protein_coding	ENSG00000147144	0,148242669	0,398538372	0,509411816

AC131009.4	TEC	ENSG00000279283	-0,212219703	0,398525597	0,509411816
VARS	protein_coding	ENSG00000204394	-0,053869741	0,39862174	0,509486626
NBPF19	protein_coding	ENSG00000271383	0,107042475	0,398867545	0,509769027
CLIC4P1	processed_pseudogene	ENSG00000236739	0,226478205	0,399147273	0,510094745
CHST13	protein_coding	ENSG00000180767	0,194252915	0,399483993	0,510493252
MAP1LC3C	protein_coding	ENSG00000197769	-0,185892967	0,399519322	0,510506591
SHOX2	protein_coding	ENSG00000168779	0,049159666	0,399768035	0,510728939
AC092120.1	sense_intronic	ENSG00000261692	0,183929703	0,399749852	0,510728939
RDM1P5	transcribed_processed_	ENSG00000263818	0,207974557	0,399766187	0,510728939
AC091132.5	processed_transcript	ENSG00000267246	0,202872878	0,400065381	0,511076983
LTB4R2	protein_coding	ENSG00000213906	0,171062374	0,400150889	0,51115438
AC046176.1	processed_pseudogene	ENSG00000236814	-0,213916349	0,400376203	0,511410346
EFNA5	protein_coding	ENSG00000184349	-0,071049611	0,400442845	0,511463616
ANKFY1	protein_coding	ENSG00000185722	-0,052817106	0,400475745	0,511473786
PDGFRA	protein_coding	ENSG00000134853	0,227880041	0,400581778	0,511577352
SND1-IT1	lincRNA	ENSG00000279078	0,209737617	0,400651613	0,511634679
AC105129.1	processed_pseudogene	ENSG00000259232	-0,177890379	0,400740933	0,51171688
ECH1	protein_coding	ENSG00000104823	0,068093764	0,400770551	0,511722841
PTOV1-AS1	antisense	ENSG00000268006	0,224438788	0,40091312	0,511873013
ABHD11-AS1	transcribed_unitary_pse	ENSG00000225969	-0,18329568	0,400964126	0,511906269
SNX13	protein_coding	ENSG00000071189	0,049246053	0,401099867	0,512047695
SCN5A	protein_coding	ENSG00000183873	0,224400392	0,401350701	0,512312835
KDM4A	protein_coding	ENSG00000066135	0,036621641	0,401368352	0,512312835
FLVCR1	protein_coding	ENSG00000162769	-0,054253183	0,401382494	0,512312835
AC027612.3	processed_pseudogene	ENSG00000232531	-0,181815759	0,401495192	0,512424791
LIN37	protein_coding	ENSG00000267796	-0,14899778	0,401671487	0,512617896
KIZ	protein_coding	ENSG00000088970	-0,070917533	0,401848382	0,512811741
ARHGAP22	protein_coding	ENSG00000128805	-0,054769593	0,402195237	0,513222441
CNTD1	protein_coding	ENSG00000176563	0,201517704	0,40227879	0,513297125
ATP5MD	protein_coding	ENSG00000173915	-0,054754919	0,40247342	0,51351352
OGFOD1	protein_coding	ENSG00000087263	0,041980098	0,402578226	0,51361529
SORBS3	protein_coding	ENSG00000120896	-0,074653149	0,403089378	0,514235439
HIC1	protein_coding	ENSG00000177374	-0,187396849	0,403306523	0,514480457
KCNMB3	protein_coding	ENSG00000171121	0,117089635	0,403341423	0,514492977
ZNF764	protein_coding	ENSG00000169951	-0,109992418	0,403522392	0,514691807
NAA60	protein_coding	ENSG00000122390	0,07953366	0,403585094	0,514739773
AC119403.1	antisense	ENSG00000253392	-0,22501875	0,40370538	0,514861171
MIATNB	lincRNA	ENSG00000244625	-0,123547784	0,404160285	0,51540928
CMPK1	protein_coding	ENSG00000162368	0,05564562	0,404255225	0,5154983
AC055713.1	lincRNA	ENSG00000247373	0,200286334	0,404295531	0,515517647

AC016355.1	lincRNA	ENSG00000270964	-0,197210432	0,404690747	0,515989508
AC008514.2	processed_pseudogene	ENSG00000253966	-0,223599849	0,404871719	0,516123994
MITD1	protein_coding	ENSG00000158411	0,064961168	0,404825621	0,516123994
UNKL	protein_coding	ENSG00000059145	-0,064700137	0,404847177	0,516123994
C19orf54	protein_coding	ENSG00000188493	-0,117408151	0,404903679	0,516132658
SNORD73B	snoRNA	ENSG00000201264	-0,194237525	0,404990536	0,516211292
SLC12A5-AS1	antisense	ENSG00000204044	-0,208256857	0,405165034	0,516401619
TMPRSS11B	protein_coding	ENSG00000185873	0,199937516	0,405308799	0,516520657
MEMO1	protein_coding	ENSG00000162959	-0,11927227	0,405306523	0,516520657
AC087683.2	sense_intronic	ENSG00000276174	-0,197004417	0,405340345	0,516528765
GJB4	protein_coding	ENSG00000189433	0,192304743	0,405390685	0,51656082
PRKDC	protein_coding	ENSG00000253729	-0,057405168	0,405461209	0,516618587
AP001486.2	sense_overlapping	ENSG00000260966	-0,145625904	0,405530916	0,516675307
AC009303.1	processed_pseudogene	ENSG00000224967	-0,202985658	0,405633646	0,51677409
CCDC28A	protein_coding	ENSG00000024862	-0,066346232	0,405692945	0,516817534
PTPRS	protein_coding	ENSG00000105426	-0,060295253	0,405755858	0,516833478
AL161891.1	sense_intronic	ENSG00000276672	0,124238764	0,405748862	0,516833478
AC132872.1	lincRNA	ENSG00000260563	-0,224208098	0,406017923	0,517135168
AC006369.1	antisense	ENSG00000236213	-0,179706612	0,406514463	0,517632187
AC021016.3	antisense	ENSG00000273361	-0,223337973	0,406546178	0,517632187
EEF1A1P10	processed_pseudogene	ENSG00000243746	-0,223078625	0,406540699	0,517632187
TMC7	protein_coding	ENSG00000170537	0,128257599	0,406490158	0,517632187
PDZD11	protein_coding	ENSG00000120509	-0,051408402	0,406559576	0,517632187
FNDC4	protein_coding	ENSG00000115226	-0,14125937	0,406506888	0,517632187
AC006064.2	antisense	ENSG00000247853	0,224279223	0,406740036	0,517680978
AC093249.2	antisense	ENSG00000260167	-0,211658111	0,40674934	0,517680978
AL133260.1	processed_pseudogene	ENSG00000219747	0,206094941	0,406689334	0,517680978
DYNC1H1	protein_coding	ENSG00000197102	0,080790666	0,406730263	0,517680978
MBOAT7	protein_coding	ENSG00000125505	0,060557441	0,406698633	0,517680978
ABI1	protein_coding	ENSG00000136754	0,035368816	0,406651284	0,517680978
AC026403.1	processed_pseudogene	ENSG00000229119	0,175875751	0,407031117	0,518007458
AC010680.3	antisense	ENSG00000271011	0,214526875	0,407124908	0,518030391
AC009686.2	lincRNA	ENSG00000272264	-0,200156863	0,407111974	0,518030391
CPEB1	protein_coding	ENSG00000214575	0,116325967	0,407117539	0,518030391
AKTIP	protein_coding	ENSG00000166971	-0,076872473	0,407220618	0,518120029
B3GAT3P1	processed_pseudogene	ENSG00000244009	0,183672635	0,407277464	0,518160213
FLVCR1-DT	lincRNA	ENSG00000198468	-0,122823537	0,407498846	0,5184059
ARHGEF12	protein_coding	ENSG00000196914	0,06152373	0,407521128	0,5184059
CADM2	protein_coding	ENSG00000175161	0,210385007	0,407579643	0,518448181
CCDC173	protein_coding	ENSG00000154479	-0,196615069	0,407837153	0,518743566

GTF2IRD1	protein_coding	ENSG00000006704	-0,066935537	0,40792566	0,518823967
ASB12	protein_coding	ENSG00000198881	0,201189826	0,408013224	0,518903157
NRADDP	transcribed_unitary_pse	ENSG00000236409	-0,183611423	0,408048991	0,518916468
AL356535.1	processed_pseudogene	ENSG00000213087	0,195764995	0,408088078	0,518934
AC019226.1	TEC	ENSG00000281469	0,219153185	0,408151444	0,518982401
AC116347.1	processed_pseudogene	ENSG00000238000	0,221470317	0,408223051	0,519041273
TTC23L	protein_coding	ENSG00000205838	0,222665746	0,408281117	0,519050747
FBXW5	protein_coding	ENSG00000159069	-0,055174394	0,408270867	0,519050747
MCCC2	protein_coding	ENSG00000131844	-0,041041526	0,408410939	0,519183609
AC116021.1	lincRNA	ENSG00000254639	0,210747077	0,40850893	0,519211636
AC021087.3	processed_transcript	ENSG00000260774	0,21777165	0,408481836	0,519211636
RAE1	protein_coding	ENSG00000101146	0,042785034	0,408486048	0,519211636
AHSA2P	transcribed_unitary_pse	ENSG00000173209	0,132985238	0,40854741	0,519228367
MINAR1	protein_coding	ENSG00000169330	-0,17049697	0,408625534	0,519295477
KIF3C	protein_coding	ENSG00000084731	-0,055031078	0,408813518	0,519469999
C15orf40	protein_coding	ENSG00000169609	-0,061087444	0,408803711	0,519469999
RN7SKP48	misc_RNA	ENSG00000201901	-0,209889708	0,408942667	0,519601914
MACROD1	protein_coding	ENSG00000133315	-0,084854616	0,409040979	0,519694634
AC078899.1	processed_pseudogene	ENSG00000213985	0,223453926	0,409135108	0,519782028
SPART-AS1	antisense	ENSG00000120664	0,204795251	0,409207522	0,519841826
AC140479.2	processed_pseudogene	ENSG00000230650	-0,216247068	0,409261523	0,519878226
CGA	protein_coding	ENSG00000135346	0,214216185	0,409400654	0,520022755
AC079944.2	processed_pseudogene	ENSG00000241889	-0,217298131	0,409446068	0,520048232
PAQR3	protein_coding	ENSG00000163291	-0,053240801	0,409676624	0,520308846
JCHAIN	protein_coding	ENSG00000132465	0,183159243	0,409780203	0,520408171
TPRG1-AS1	lincRNA	ENSG00000234076	0,215022289	0,409925435	0,520528149
FUNDC2P1	processed_pseudogene	ENSG00000255883	-0,212798328	0,409902056	0,520528149
LRRC40	protein_coding	ENSG00000066557	0,058274649	0,409958358	0,520537728
DIS3L	protein_coding	ENSG00000166938	0,035648068	0,410096436	0,520680816
HMGN1P36	processed_pseudogene	ENSG00000235734	-0,217057909	0,410155881	0,520724056
RPL14P3	processed_pseudogene	ENSG00000241923	0,222553909	0,410459156	0,521076831
PROM2	protein_coding	ENSG00000155066	0,093779931	0,410517256	0,521118333
LIX1-AS1	lincRNA	ENSG00000251513	0,209789584	0,410616398	0,521211927
ZFP82	protein_coding	ENSG00000181007	-0,083351068	0,411269525	0,521976358
AC008443.6	antisense	ENSG00000250900	0,219427221	0,411356802	0,522054824
PRKAG2	protein_coding	ENSG00000106617	-0,053712757	0,411462003	0,522156026
AC133435.1	processed_pseudogene	ENSG00000233597	-0,199784175	0,411809888	0,522536891
AL137803.1	transcribed_processed_	ENSG00000230863	0,153749672	0,411813081	0,522536891
MIRLET7A1HG	lincRNA	ENSG00000269946	0,221404107	0,41205099	0,522806422
SERGEF	protein_coding	ENSG00000129158	0,073145708	0,412157499	0,522909211

TLR5	protein_coding	ENSG00000187554	-0,173567708	0,412240622	0,522982319
PFKFB4	protein_coding	ENSG00000114268	0,080281452	0,412476691	0,523249439
METTL14-DT	lincRNA	ENSG00000281731	0,211701168	0,412615427	0,523360693
AKNA	protein_coding	ENSG00000106948	-0,084040536	0,412606887	0,523360693
SPACA9	protein_coding	ENSG00000165698	0,077799486	0,412657454	0,523379168
SERF2	protein_coding	ENSG00000140264	-0,066188569	0,412681029	0,523379168
BX322234.2	lincRNA	ENSG00000233085	0,207974083	0,41298811	0,523736236
MT-ND6	protein_coding	ENSG00000198695	0,21721746	0,413023595	0,523748853
HSD17B6	protein_coding	ENSG00000025423	0,213494836	0,413092111	0,52380335
AC125611.3	antisense	ENSG00000258232	-0,207811442	0,413172163	0,52387247
MIR497HG	antisense	ENSG00000267532	0,203383916	0,413329346	0,52403937
TTC4	protein_coding	ENSG00000243725	-0,176123888	0,413376385	0,524066612
BZW1	protein_coding	ENSG00000082153	-0,046499405	0,413524849	0,524222427
AC019131.2	lincRNA	ENSG00000272777	0,209083395	0,413572207	0,524250059
AC024580.1	TEC	ENSG00000279794	-0,182465687	0,413617688	0,52427531
MAGI1	protein_coding	ENSG00000151276	0,05393998	0,413734569	0,524391052
AC115618.1	antisense	ENSG00000204620	0,204411345	0,413773444	0,524407919
AL583722.2	antisense	ENSG00000258430	-0,219117577	0,414063283	0,524742828
AL135791.1	antisense	ENSG00000236514	-0,215818897	0,414281972	0,524987534
AC009022.1	processed_transcript	ENSG00000196696	0,212177773	0,414578497	0,52533084
RPL24P8	processed_pseudogene	ENSG00000236801	-0,168185054	0,414782842	0,525557305
AL035252.3	lincRNA	ENSG00000277938	0,207969267	0,414956979	0,525745468
AL161911.1	processed_pseudogene	ENSG00000270917	0,197696288	0,415042793	0,525821712
ATP8A1	protein_coding	ENSG00000124406	0,157487748	0,415124248	0,525892423
ZNF619	protein_coding	ENSG00000177873	0,081300142	0,415281254	0,52605883
ANO7	protein_coding	ENSG00000146205	-0,200163853	0,415432996	0,526218549
AC087286.4	sense_intronic	ENSG00000259453	-0,203200974	0,415488556	0,526256425
TMEM231P1	transcribed_unprocesse	ENSG00000262583	0,215970653	0,415688864	0,526445113
EDRF1	protein_coding	ENSG00000107938	0,040649979	0,415798714	0,526551718
AC121761.2	sense_intronic	ENSG00000273987	0,154416409	0,415830289	0,526559191
PARP1P1	processed_pseudogene	ENSG00000227105	0,173286695	0,415935076	0,526659366
AC006547.2	antisense	ENSG00000243762	0,202197844	0,41597655	0,526679365
SNORA2A	snoRNA	ENSG00000206612	-0,217817326	0,416124049	0,526833596
AC010913.1	processed_transcript	ENSG00000272702	-0,178948877	0,416289585	0,52701064
GORAB-AS1	antisense	ENSG00000231407	0,210788806	0,416395009	0,527111569
AC114491.1	processed_pseudogene	ENSG00000224550	0,194870364	0,417158816	0,528045875
FAM50A	protein_coding	ENSG00000071859	-0,034239125	0,41733293	0,52822986
POFUT2	protein_coding	ENSG00000186866	-0,058432046	0,417355674	0,52822986
AC090241.3	lincRNA	ENSG00000274776	0,2046901	0,417462839	0,52833289
AC090616.6	TEC	ENSG00000278867	-0,215163429	0,417525195	0,528379204

AC116407.2	lincRNA	ENSG00000277511	-0,207469067	0,417602499	0,528444426
C7orf61	protein_coding	ENSG00000185955	0,21660131	0,417662124	0,528487271
TYRO3P	processed_pseudogene	ENSG00000259581	0,218769585	0,417726145	0,528535671
PPCS	protein_coding	ENSG00000127125	-0,055482016	0,41797918	0,528823205
MIAT	lincRNA	ENSG00000225783	0,189677317	0,418093751	0,528935531
CDC42-IT1	sense_intronic	ENSG00000230068	-0,202428749	0,41830531	0,529170536
TRIM24	protein_coding	ENSG00000122779	0,056141227	0,418380418	0,529232908
ADA	protein_coding	ENSG00000196839	0,06388169	0,418567174	0,529436493
RARG	protein_coding	ENSG00000172819	-0,067436508	0,418745811	0,529629784
TCTA	protein_coding	ENSG00000145022	0,066231078	0,418831896	0,529673337
ZP4	protein_coding	ENSG00000116996	-0,211582769	0,418813674	0,529673337
CDC37L1-DT	lincRNA	ENSG00000273061	0,212840943	0,418978547	0,52982613
LRRC8C-DT	antisense	ENSG00000231999	0,154129135	0,419295209	0,530176376
NFASC	protein_coding	ENSG00000163531	-0,060303773	0,419307215	0,530176376
AL133467.2	lincRNA	ENSG00000258927	0,205728373	0,419490235	0,530342398
HIST2H4B	protein_coding	ENSG00000270276	-0,201807651	0,419482941	0,530342398
CPA3	protein_coding	ENSG00000163751	-0,210684352	0,419554986	0,530391566
AC068870.2	lincRNA	ENSG00000277782	0,17475845	0,419586981	0,530399321
NRL	protein_coding	ENSG00000129535	0,172312738	0,41970894	0,530520791
RPL26P3	processed_pseudogene	ENSG00000240179	0,183879782	0,41997112	0,530819478
AL031665.2	antisense	ENSG00000276026	0,217423876	0,420023268	0,530852676
DGLUCY	protein_coding	ENSG00000133943	-0,055828802	0,420134067	0,530959992
AC021106.1	processed_pseudogene	ENSG00000249863	-0,126427406	0,420661386	0,531593655
AC092535.1	antisense	ENSG00000227189	-0,211890717	0,420798383	0,531707731
LINC00472	lincRNA	ENSG00000233237	-0,094584378	0,420803505	0,531707731
UBL5	protein_coding	ENSG00000198258	-0,053220635	0,420904101	0,531802077
RPTOR	protein_coding	ENSG00000141564	0,067597057	0,420965611	0,53184703
ZDHHC11B	protein_coding	ENSG00000206077	0,204601269	0,42107587	0,531953562
USF1	protein_coding	ENSG00000158773	-0,051362639	0,421340612	0,532255233
AC068580.1	sense_intronic	ENSG00000229512	0,200647403	0,421478302	0,532396378
AC034231.1	antisense	ENSG00000249572	0,195607531	0,421531644	0,532405692
HDAC11-AS1	antisense	ENSG00000244502	-0,192749023	0,421537593	0,532405692
AP000936.3	processed_pseudogene	ENSG00000234268	0,195889231	0,421632451	0,532492708
AC117498.2	lincRNA	ENSG00000257660	-0,19806747	0,421934525	0,532841395
PCNP	protein_coding	ENSG00000081154	-0,058440031	0,422106165	0,533025333
AC009163.6	lincRNA	ENSG00000274220	0,192642052	0,422171092	0,5330745
AC145207.2	antisense	ENSG00000262413	0,189332826	0,422219229	0,533102461
NDUFV2-AS1	antisense	ENSG00000266053	0,214946509	0,422322277	0,533199748
AC008038.1	processed_pseudogene	ENSG00000283041	0,084584659	0,422450793	0,533264177
AL354890.1	processed_pseudogene	ENSG00000223787	-0,2036297	0,422460195	0,533264177

AC092718.3	protein_coding	ENSG00000260643	0,213645932	0,422477309	0,533264177
SEC14L4	protein_coding	ENSG00000133488	-0,1313638	0,422431713	0,533264177
STRIP2	protein_coding	ENSG00000128578	-0,074318798	0,422507453	0,533269407
LASP1	protein_coding	ENSG00000002834	0,044078399	0,422682423	0,533457418
NMD3	protein_coding	ENSG00000169251	-0,063079236	0,422882735	0,533677388
LINC02236	lincRNA	ENSG00000251365	0,203122085	0,423256486	0,534083335
ZNF385B	protein_coding	ENSG00000144331	0,2077645	0,423324516	0,534136317
SNORA40B	snoRNA	ENSG00000208308	0,210743069	0,423371605	0,534162871
SNORA59A	snoRNA	ENSG00000239149	0,213472906	0,423414864	0,534184588
MIR181A1HG	lincRNA	ENSG00000229989	0,130029587	0,423604318	0,534380258
C17orf113	protein_coding	ENSG00000267221	-0,197504655	0,423622068	0,534380258
CNNM4	protein_coding	ENSG00000158158	0,057813789	0,423691621	0,534409044
HMGA1	protein_coding	ENSG00000137309	-0,05132714	0,423697	0,534409044
HMGN3-AS1	antisense	ENSG00000270362	-0,179561509	0,423912896	0,5346156
RPS27AP12	processed_pseudogene	ENSG00000225224	0,214508245	0,423901324	0,5346156
ZNF326	protein_coding	ENSG00000162664	-0,061302326	0,424010975	0,534706413
LINC02021	lincRNA	ENSG00000249846	0,214648543	0,424167802	0,534871296
ADI1	protein_coding	ENSG00000182551	0,047705911	0,424439607	0,535181135
POLL	protein_coding	ENSG00000166169	-0,071390472	0,424481314	0,535200821
AC040169.1	antisense	ENSG00000260018	-0,193236571	0,424540461	0,535242491
POLD4	protein_coding	ENSG00000175482	-0,102286858	0,424616664	0,535283482
AL596325.2	sense_intronic	ENSG00000272574	-0,187048238	0,42462517	0,535283482
MROH2B	protein_coding	ENSG00000171495	0,190990069	0,424860625	0,53554738
AC021851.1	lincRNA	ENSG00000272800	0,207494922	0,424896487	0,53555967
PXDN	protein_coding	ENSG00000130508	0,054535272	0,425024399	0,535687976
FABP5P7	processed_pseudogene	ENSG00000234964	-0,189705624	0,42518161	0,535787344
RPS15AP1	processed_pseudogene	ENSG00000214535	-0,207476564	0,425173949	0,535787344
MCF2	protein_coding	ENSG00000101977	0,204221239	0,425172348	0,535787344
GTF2IP12	transcribed_unprocesse	ENSG00000283050	0,154107434	0,425349152	0,535965541
NARF-IT1	sense_intronic	ENSG00000266236	0,209216109	0,425414218	0,536014598
FRAT1	protein_coding	ENSG00000165879	0,165240602	0,425472983	0,536041873
TOB2	protein_coding	ENSG00000183864	0,062421653	0,425488137	0,536041873
AC139887.2	antisense	ENSG00000249592	0,148388237	0,425576824	0,536087744
LINC01117	lincRNA	ENSG00000224577	0,103145889	0,425553772	0,536087744
PLOD3	protein_coding	ENSG00000106397	-0,046543611	0,425845358	0,536393066
NME2P1	processed_pseudogene	ENSG00000123009	0,211599634	0,426181486	0,536783485
AC069234.5	lincRNA	ENSG00000277423	0,187903588	0,426405427	0,536949713
FAM192BP	processed_pseudogene	ENSG00000231841	0,187159559	0,426390515	0,536949713
LINC02609	processed_transcript	ENSG00000233593	-0,105488713	0,426409673	0,536949713
RBM15-AS1	antisense	ENSG00000227963	0,169598339	0,426527795	0,537021797

SLCO1B1	protein_coding	ENSG00000134538	0,123180343	0,42651648	0,537021797
STAG3L1	transcribed_unprocesse	ENSG00000205583	-0,204777814	0,426750798	0,537269588
KRT18P29	processed_pseudogene	ENSG00000213958	-0,207019153	0,426839687	0,537315534
UBE2Q2P6	transcribed_unprocesse	ENSG00000275695	-0,207187808	0,426832798	0,537315534
CSF2RA	protein_coding	ENSG00000198223	-0,038613275	0,42695263	0,537424724
SLC2A10	protein_coding	ENSG00000197496	-0,212646745	0,42703567	0,537496262
AL513477.1	transcribed_processed_	ENSG00000269896	-0,194486778	0,427068556	0,537504669
TNK2	protein_coding	ENSG00000061938	0,077797415	0,427103963	0,537516248
RFX7	protein_coding	ENSG00000181827	0,056757507	0,427189728	0,537591197
AL137127.1	3prime_overlapping_nc	ENSG00000272084	0,198619659	0,427319992	0,537665231
COPS4	protein_coding	ENSG00000138663	-0,049435604	0,427302333	0,537665231
SLC6A16	protein_coding	ENSG00000063127	-0,209247035	0,427327203	0,537665231
CWC27	protein_coding	ENSG00000153015	0,043803111	0,427445401	0,537764131
PAWR	protein_coding	ENSG00000177425	-0,040997598	0,427458246	0,537764131
SHLD3	protein_coding	ENSG00000253251	-0,112196906	0,427538688	0,537832342
LOH12CR2	lincRNA	ENSG00000205791	0,170351728	0,42767082	0,537932573
NDUFB11	protein_coding	ENSG00000147123	0,060434117	0,427808589	0,538057297
SNX3	protein_coding	ENSG00000112335	-0,049626174	0,427822447	0,538057297
IPO7P2	processed_pseudogene	ENSG00000225674	0,213882829	0,427852429	0,53806201
AC131235.3	lincRNA	ENSG00000273181	-0,185626988	0,428030321	0,538173433
IL36RN	protein_coding	ENSG00000136695	0,178478729	0,428045988	0,538173433
MFSD4B	protein_coding	ENSG00000173214	-0,073610049	0,428022351	0,538173433
NUDT16L1	protein_coding	ENSG00000168101	-0,084231998	0,427994971	0,538173433
NHP2P1	processed_pseudogene	ENSG00000105988	0,195690715	0,428167074	0,538292674
AK7	protein_coding	ENSG00000140057	0,203381659	0,428246475	0,538328278
CREBBP	protein_coding	ENSG00000005339	0,066217121	0,428247888	0,538328278
AC074044.1	antisense	ENSG00000272844	-0,20247227	0,428307475	0,538370185
NDUFA11	protein_coding	ENSG00000174886	0,080361075	0,428377953	0,538392782
NDUFAF3	protein_coding	ENSG00000178057	-0,059978125	0,428352497	0,538392782
RPL23	protein_coding	ENSG00000125691	-0,046218295	0,428622977	0,538667725
PCDH15	protein_coding	ENSG00000150275	0,18738046	0,428684921	0,538679557
AC073569.3	sense_intronic	ENSG00000277130	-0,203943725	0,428660162	0,538679557
C1orf109	protein_coding	ENSG00000116922	-0,044904181	0,428820954	0,538817482
PELI3	protein_coding	ENSG00000174516	0,086211347	0,428898588	0,538882016
AURKAP1	transcribed_processed_	ENSG00000213033	0,188928477	0,429033557	0,539018576
RASL11A	protein_coding	ENSG00000122035	-0,142256882	0,429098557	0,539067218
VAMP3	protein_coding	ENSG00000049245	0,04031313	0,429400247	0,539413185
AC016590.3	antisense	ENSG00000276846	-0,190917057	0,429455241	0,539449228
RPL10P9	processed_pseudogene	ENSG00000233913	-0,194033767	0,429553759	0,539539935
IL5RA	protein_coding	ENSG00000091181	0,174623989	0,429703517	0,539694987

AP000568.1	processed_pseudogene	ENSG00000229336	-0,203620829	0,429989733	0,540021397
SLC25A37	protein_coding	ENSG00000147454	-0,05135142	0,430295357	0,540372139
AP002807.1	antisense	ENSG00000255031	-0,178833949	0,430392997	0,540461665
AC034111.1	antisense	ENSG00000260093	-0,210340018	0,430482737	0,540541259
PLEKHO1	protein_coding	ENSG00000023902	-0,144554997	0,430546753	0,540588547
AC036108.3	antisense	ENSG00000261616	0,18875426	0,430607524	0,540631753
AL590762.2	processed_pseudogene	ENSG00000213706	0,178215884	0,430798005	0,540837796
SLK	protein_coding	ENSG00000065613	0,053886889	0,431052208	0,541123809
SEMA4B	protein_coding	ENSG00000185033	-0,073237511	0,43108295	0,54112928
AC046143.2	lincRNA	ENSG00000272707	0,210769256	0,431403086	0,541497998
SNX18P12	processed_pseudogene	ENSG00000279718	-0,210766173	0,431459703	0,541502785
IRAK1	protein_coding	ENSG00000184216	0,053560093	0,431459489	0,541502785
ATP13A2	protein_coding	ENSG00000159363	-0,056382787	0,431527548	0,541554795
PLPP4	protein_coding	ENSG00000203805	0,192554782	0,431722327	0,541766088
AC115837.1	processed_pseudogene	ENSG00000253341	-0,148424347	0,431827947	0,541865475
LGR4	protein_coding	ENSG00000205213	0,053855514	0,431926343	0,541925607
GDAP1	protein_coding	ENSG00000104381	-0,044143059	0,431928713	0,541925607
AC080080.1	TEC	ENSG00000279048	0,210653071	0,432049342	0,542043798
TMX2P1	processed_pseudogene	ENSG00000213839	0,181791412	0,432309423	0,542303747
SIPA1L1	protein_coding	ENSG00000197555	-0,051548083	0,432293276	0,542303747
UBXN8	protein_coding	ENSG00000104691	-0,058744269	0,432619522	0,542659555
AP005230.1	lincRNA	ENSG00000263745	0,200828462	0,432712086	0,542672981
SUDS3P1	processed_pseudogene	ENSG00000249129	0,200363627	0,432682472	0,542672981
LGALS3	protein_coding	ENSG00000131981	0,066273643	0,432736061	0,542672981
CASP3	protein_coding	ENSG00000164305	0,03948585	0,432701044	0,542672981
CACUL1	protein_coding	ENSG00000151893	0,031516216	0,433025833	0,543003169
LINC01031	lincRNA	ENSG00000232077	0,209120203	0,433166751	0,543113465
AL356653.1	processed_pseudogene	ENSG00000233514	-0,207031088	0,433157058	0,543113465
TSC2	protein_coding	ENSG00000103197	-0,055261535	0,433339432	0,543296763
CNPY3	protein_coding	ENSG00000137161	-0,043936495	0,433400429	0,543340024
AL158163.1	antisense	ENSG00000270589	0,1753133	0,433548677	0,543492657
ITPK1	protein_coding	ENSG00000100605	-0,052670058	0,433604029	0,543528825
DUS4L	protein_coding	ENSG00000105865	0,071387545	0,434148511	0,544144828
NUDT22	protein_coding	ENSG00000149761	-0,056801613	0,434136757	0,544144828
RN7SL767P	misc_RNA	ENSG00000241529	0,189494268	0,43424066	0,544193813
SYNGR1	protein_coding	ENSG00000100321	-0,057533843	0,434219199	0,544193813
STAM2	protein_coding	ENSG00000115145	-0,052833551	0,434299815	0,544234692
GRIP1	protein_coding	ENSG00000155974	0,111374758	0,434337804	0,544249045
SLC19A3	protein_coding	ENSG00000135917	-0,16770582	0,434466218	0,544376696
HSPA8P7	processed_pseudogene	ENSG00000224773	-0,188454582	0,43455985	0,544460753

AC115618.3	TEC	ENSG00000279528	-0,134232179	0,434607805	0,544487575
SNORD42B	snoRNA	ENSG00000238423	-0,160159045	0,434654367	0,544512647
LINC01703	lincRNA	ENSG00000225518	-0,201969525	0,434819432	0,544686163
AC091614.1	lincRNA	ENSG00000272094	-0,199642542	0,434918508	0,544777
C8orf44	protein_coding	ENSG00000213865	-0,136618721	0,434966164	0,544803421
RPS27AP11	processed_pseudogene	ENSG00000218208	0,204951821	0,435239421	0,545112391
CASZ1	protein_coding	ENSG00000130940	0,107111093	0,435347547	0,54521452
NPC1	protein_coding	ENSG00000141458	0,042016429	0,435550692	0,545402325
DCBLD1	protein_coding	ENSG00000164465	-0,050466538	0,435528508	0,545402325
DNAJC19P9	processed_pseudogene	ENSG00000258608	0,190074075	0,436070501	0,546019901
KRT8P33	processed_pseudogene	ENSG00000250539	-0,118235651	0,436261919	0,546226235
AL139424.1	antisense	ENSG00000271989	-0,202593212	0,436303203	0,546244578
STBD1	protein_coding	ENSG00000118804	-0,162892761	0,436570629	0,546546029
SEPT7-AS1	lincRNA	ENSG00000228878	0,182970126	0,436672584	0,546573581
ERVK3-1	protein_coding	ENSG00000142396	0,092849142	0,436626351	0,546573581
FAM210B	protein_coding	ENSG00000124098	0,046600813	0,436655993	0,546573581
L3MBTL1	protein_coding	ENSG00000185513	-0,107661047	0,437211485	0,547214716
RN7SL230P	misc_RNA	ENSG00000264916	0,195588064	0,437248751	0,547219577
PCYOX1L	protein_coding	ENSG00000145882	-0,070972152	0,43726873	0,547219577
MT-ND3	protein_coding	ENSG00000198840	0,139684347	0,437404294	0,547355831
LARP4P	transcribed_processed_	ENSG00000261315	-0,194328997	0,437460041	0,547392193
AC137834.2	antisense	ENSG00000276727	0,18291187	0,437763657	0,54773869
TERT	protein_coding	ENSG00000164362	-0,206786474	0,437920574	0,547901602
AC002094.4	sense_intronic	ENSG00000277450	-0,188776039	0,438047004	0,548026354
AGPAT4-IT1	TEC	ENSG00000279355	0,19297899	0,438151917	0,548124173
OCLNP1	unprocessed_pseudoge	ENSG00000230847	-0,174216356	0,438240723	0,548201832
AP000892.3	TEC	ENSG00000280143	0,186029121	0,438468723	0,548453589
ARRDC2	protein_coding	ENSG00000105643	-0,063475214	0,438620631	0,548610144
SEPT10	protein_coding	ENSG00000186522	0,049488548	0,438676575	0,548646658
RETREG3	protein_coding	ENSG00000141699	-0,039894201	0,438717344	0,548664191
AL133520.1	sense_intronic	ENSG00000273893	0,200190443	0,438762512	0,548687222
AC012442.2	3prime_overlapping_nc	ENSG00000243389	0,188616764	0,438887393	0,548809928
GNGT2	protein_coding	ENSG00000167083	-0,18292062	0,438914213	0,548810005
CSPG4P12	transcribed_unprocesse	ENSG00000259295	0,204924526	0,438981516	0,548860699
RBM41	protein_coding	ENSG00000089682	-0,048841194	0,439082925	0,548954026
ST3GAL5-AS1	lincRNA	ENSG00000232504	0,185957652	0,439122208	0,548969675
AC139530.2	antisense	ENSG00000262049	-0,106396487	0,439228551	0,549069152
SRR	protein_coding	ENSG00000167720	-0,062647228	0,439322836	0,549153545
TTC28	protein_coding	ENSG00000100154	-0,064204265	0,439488569	0,549260285
AC009163.3	sense_intronic	ENSG00000261476	0,206431356	0,439483627	0,549260285

AP003119.3	sense_overlapping	ENSG00000261578	-0,204866385	0,439472499	0,549260285
AC006213.4	antisense	ENSG00000267191	0,127242862	0,439699293	0,549379883
TMEM120B	protein_coding	ENSG00000188735	0,051067781	0,439640809	0,549379883
UBAP2	protein_coding	ENSG00000137073	0,042711581	0,439662121	0,549379883
DYM	protein_coding	ENSG00000141627	0,034062431	0,439684965	0,549379883
AC091390.4	transcribed_unprocesse	ENSG00000239969	0,20550434	0,439718194	0,549379883
Z95331.1	sense_overlapping	ENSG00000280383	-0,168333902	0,439874568	0,54954178
TPD52	protein_coding	ENSG00000076554	0,066372751	0,440003631	0,549669538
ERICH6	protein_coding	ENSG00000163645	0,145284165	0,440033638	0,549673544
AL162586.1	antisense	ENSG00000225032	0,194590685	0,440369127	0,550059123
CDK11A	protein_coding	ENSG00000008128	-0,20464054	0,440472948	0,550155299
JMJD7	protein_coding	ENSG00000243789	0,201197957	0,440595294	0,550241093
AP3S1	protein_coding	ENSG00000177879	0,048150262	0,440582815	0,550241093
MYLPF	protein_coding	ENSG00000180209	0,166992318	0,440912958	0,550604284
CYB561	protein_coding	ENSG00000008283	0,132331833	0,441227146	0,550963091
FAM43B	protein_coding	ENSG00000183114	-0,204966455	0,441286293	0,551003401
FGD1	protein_coding	ENSG00000102302	-0,094878952	0,441623255	0,551390574
AC125232.1	unprocessed_pseudoge	ENSG00000231259	0,121253697	0,441739603	0,551502268
SLC5A4-AS1	antisense	ENSG00000242082	0,196647048	0,44194956	0,55163008
IQCG	protein_coding	ENSG00000114473	0,065457689	0,441881491	0,55163008
VHL	protein_coding	ENSG00000134086	-0,040287605	0,44194028	0,55163008
AL035458.2	sense_overlapping	ENSG00000250917	0,179934945	0,441920921	0,55163008
HOMER3	protein_coding	ENSG00000051128	0,047001348	0,441986571	0,551642705
GRB14	protein_coding	ENSG00000115290	-0,105236006	0,442068952	0,551711951
AC024575.1	antisense	ENSG00000267277	0,204627747	0,442197554	0,551774145
AC004554.2	lincRNA	ENSG00000282840	0,193704347	0,442199494	0,551774145
DNM1P46	transcribed_unprocesse	ENSG00000182397	0,197516981	0,442158189	0,551774145
ABHD11	protein_coding	ENSG00000106077	-0,055737723	0,442287658	0,551850582
LINC01515	lincRNA	ENSG00000228065	-0,071822038	0,442540665	0,552099091
AF279873.1	processed_pseudogene	ENSG00000188512	0,189742859	0,442516065	0,552099091
PAM16	protein_coding	ENSG00000217930	0,188663232	0,442628383	0,552174936
METTL15	protein_coding	ENSG00000169519	0,055123615	0,4428946	0,552473437
SHANK1	protein_coding	ENSG00000161681	0,138153586	0,443428252	0,553105482
NRP2	protein_coding	ENSG00000118257	0,048866089	0,443620582	0,553311733
SLC36A4	protein_coding	ENSG00000180773	0,049076748	0,443747871	0,55343684
LINC02352	lincRNA	ENSG00000259448	0,20182287	0,443857415	0,553472495
RN7SL141P	misc_RNA	ENSG00000243398	0,176640552	0,443838327	0,553472495
TRIOBP	protein_coding	ENSG00000100106	-0,041258334	0,443809204	0,553472495
FAM81A	protein_coding	ENSG00000157470	0,064768987	0,444167371	0,553825325
NPHP3	protein_coding	ENSG00000113971	-0,09657176	0,444243725	0,553886858

ACTL10	protein_coding	ENSG00000182584	-0,205043281	0,444345482	0,553980053
AC016734.1	processed_pseudogene	ENSG00000228305	0,198746707	0,444571493	0,554228138
BCORL1	protein_coding	ENSG00000085185	0,138094797	0,44476179	0,554431673
TUSC3	protein_coding	ENSG00000104723	-0,034035977	0,444813403	0,554462313
U62317.2	antisense	ENSG00000272821	-0,128143734	0,444865567	0,554493636
AC138696.2	antisense	ENSG00000272172	0,1816137	0,444922102	0,554530404
ARLNC1	lincRNA	ENSG00000260896	0,193659572	0,445175895	0,554813004
KRT8P10	processed_pseudogene	ENSG00000231203	0,182007876	0,445274732	0,554893178
CUL2	protein_coding	ENSG00000108094	0,041720405	0,445294335	0,554893178
SYT5	protein_coding	ENSG00000129990	0,204591521	0,445363848	0,554946082
UBA6	protein_coding	ENSG00000033178	-0,037897946	0,445442452	0,555010309
IMPDH1P5	processed_pseudogene	ENSG00000213513	0,202993067	0,445565051	0,555129341
HILS1	transcribed_unitary_pse	ENSG00000188662	0,172391763	0,445631101	0,555177907
AC010422.6	protein_coding	ENSG00000269693	0,164865017	0,445856687	0,55542521
GEMIN8P4	processed_pseudogene	ENSG00000228175	-0,148775519	0,445911842	0,555426447
S100A16	protein_coding	ENSG00000188643	0,047256772	0,445890551	0,555426447
AL589993.1	processed_pseudogene	ENSG00000216809	0,204179209	0,445958379	0,555450681
ATP6V0B	protein_coding	ENSG00000117410	0,066248863	0,446000254	0,555469104
PHYKPL	protein_coding	ENSG00000175309	-0,060780012	0,446059394	0,555509027
AC069222.1	lincRNA	ENSG00000273374	0,178018608	0,446199389	0,555649634
BZW1P2	processed_pseudogene	ENSG00000198406	-0,099067332	0,446238031	0,555664016
AC087203.3	lincRNA	ENSG00000270074	0,185963493	0,44668666	0,55618889
HERC2P2	transcribed_unprocesse	ENSG00000276550	-0,106704086	0,446748421	0,556232023
AC093106.1	processed_pseudogene	ENSG00000236238	-0,18071844	0,446923718	0,556416502
ROCK1P1	transcribed_unprocesse	ENSG00000263006	0,189724543	0,446998189	0,556475439
AC000123.3	TEC	ENSG00000280347	-0,13035929	0,447172986	0,556659258
ATP6AP2	protein_coding	ENSG00000182220	-0,036523771	0,447230481	0,556663258
AC131212.4	TEC	ENSG00000280311	-0,193486037	0,447210078	0,556663258
AC140134.1	unprocessed_pseudoge	ENSG00000179978	0,187063665	0,447364233	0,556795947
AL049830.3	antisense	ENSG00000258525	-0,197561447	0,447580881	0,557031788
AC016876.3	antisense	ENSG00000276384	-0,202234301	0,447623321	0,557050805
RPL23AP67	processed_pseudogene	ENSG00000240991	0,170036918	0,447774765	0,557205462
AC087222.1	lincRNA	ENSG00000262663	0,178240203	0,447833057	0,55723188
MED23	protein_coding	ENSG00000112282	0,037651508	0,447850332	0,55723188
PRR12	protein_coding	ENSG00000126464	0,088966481	0,447936339	0,557305084
NSMCE3	protein_coding	ENSG00000185115	-0,057288372	0,448117781	0,557497008
LINC00390	lincRNA	ENSG00000226519	0,164528218	0,448288085	0,557669712
AMN1	protein_coding	ENSG00000151743	-0,064279343	0,448310981	0,557669712
AL391834.2	antisense	ENSG00000273226	-0,201593431	0,448644473	0,558016867
RASGEF1A	protein_coding	ENSG00000198915	-0,20103098	0,448618528	0,558016867

ENTPD3	protein_coding	ENSG00000168032	0,176175424	0,448712299	0,558067386
RAB31	protein_coding	ENSG00000168461	0,046751399	0,448826871	0,558176032
TCHP	protein_coding	ENSG00000139437	-0,057189254	0,448953849	0,558300094
SNORA7B	snoRNA	ENSG00000207088	-0,159072887	0,44903174	0,558363101
EEF1A1P25	processed_pseudogene	ENSG00000241429	-0,173072455	0,44917101	0,55850242
WNT2B	protein_coding	ENSG00000134245	-0,156689862	0,449456988	0,558824129
AC009108.3	lincRNA	ENSG00000270020	-0,201888034	0,449501469	0,558845555
AC134407.2	TEC	ENSG00000279573	0,191913276	0,449556866	0,55888055
NPEPL1	protein_coding	ENSG00000215440	-0,109536749	0,449641691	0,558952123
AC011468.1	sense_overlapping	ENSG00000260160	0,201303026	0,44987707	0,559210831
AC091180.2	lincRNA	ENSG00000248714	-0,175611256	0,450221348	0,559570952
BSND	protein_coding	ENSG00000162399	0,197313782	0,450478007	0,559856021
LPAR2	protein_coding	ENSG00000064547	0,146840987	0,450657758	0,560045481
AC090948.1	antisense	ENSG00000271964	-0,182315447	0,450756878	0,560100786
AC005912.1	processed_pseudogene	ENSG00000227081	0,141818123	0,450731096	0,560100786
PSMA6P2	processed_pseudogene	ENSG00000229083	0,20109945	0,451027015	0,5604025
CRYGS	protein_coding	ENSG00000213139	0,190121657	0,451091249	0,560448359
PKM	protein_coding	ENSG00000067225	-0,03384516	0,451241145	0,560600634
TEX41	lincRNA	ENSG00000226674	0,080448093	0,451342749	0,5606929
AGPAT2	protein_coding	ENSG00000169692	-0,05766267	0,45145509	0,560794116
IGFBP2	protein_coding	ENSG00000115457	-0,197817363	0,45147891	0,560794116
CTNNAL1	protein_coding	ENSG00000119326	-0,045856916	0,451507884	0,560796143
PPID	protein_coding	ENSG00000171497	0,053831566	0,451587916	0,560861581
AC020763.4	TEC	ENSG00000279569	0,182823231	0,451760831	0,561042364
AP000223.1	antisense	ENSG00000260583	-0,18449699	0,451833562	0,561098713
U73166.1	lincRNA	ENSG00000230454	0,193962615	0,451925572	0,561178996
AC004817.3	lincRNA	ENSG00000269927	-0,186967818	0,452104594	0,561212628
AC068205.1	lincRNA	ENSG00000283217	-0,200347266	0,45203691	0,561212628
RPL35P5	processed_pseudogene	ENSG00000225573	0,173680916	0,452171559	0,561212628
AC002558.1	processed_pseudogene	ENSG00000239291	-0,198886172	0,452077874	0,561212628
PLEKHA3	protein_coding	ENSG00000116095	0,043161956	0,452121707	0,561212628
DNM2	protein_coding	ENSG00000079805	0,042612819	0,452057132	0,561212628
CUL7	protein_coding	ENSG00000044090	-0,05593305	0,452164743	0,561212628
C2orf15	protein_coding	ENSG00000273045	-0,200571966	0,45214069	0,561212628
PRRT1	protein_coding	ENSG00000204314	0,127806745	0,452290322	0,561292098
PHF21B	protein_coding	ENSG00000056487	0,079962794	0,452283194	0,561292098
LRRC37A5P	transcribed_unprocesse	ENSG00000204173	0,168786325	0,452425011	0,561425277
RPL22P1	processed_pseudogene	ENSG00000213178	0,165807898	0,452792438	0,561779257
LIN52	protein_coding	ENSG00000205659	-0,050102423	0,452747558	0,561779257
GTF2IP1	transcribed_unprocesse	ENSG00000277053	0,138301178	0,452791555	0,561779257

AC138466.1	lincRNA	ENSG00000256312	0,185179548	0,452830359	0,561792321
NDUFS4	protein_coding	ENSG00000164258	-0,05086478	0,452948783	0,561905252
AC114271.1	antisense	ENSG00000274425	0,104009829	0,453694551	0,562762337
MILR1	protein_coding	ENSG00000271605	0,20042718	0,453849134	0,562910857
AC004890.2	transcribed_unprocesse	ENSG00000244560	-0,151755596	0,453869178	0,562910857
AC053513.1	lincRNA	ENSG00000256973	-0,197496895	0,453978808	0,562944701
SMARCA4	protein_coding	ENSG00000127616	-0,073121644	0,45393031	0,562944701
ADGRB3	protein_coding	ENSG00000135298	-0,147827065	0,453962776	0,562944701
AC080013.6	lincRNA	ENSG00000272440	-0,198865292	0,454109402	0,563004523
PRDX5	protein_coding	ENSG00000126432	0,058315108	0,454061501	0,563004523
EIF2AK4	protein_coding	ENSG00000128829	0,029217549	0,454089138	0,563004523
AL645728.2	transcribed_unprocesse	ENSG00000284740	0,180546024	0,454180655	0,563058827
GFOD1	protein_coding	ENSG00000145990	-0,091046954	0,454336354	0,563217807
AL672207.1	processed_pseudogene	ENSG00000178146	0,182331866	0,45438819	0,56323387
APOBEC3D	protein_coding	ENSG00000243811	0,16064191	0,454404235	0,56323387
Z97634.1	transcribed_processed_	ENSG00000236829	0,188542795	0,454441878	0,563246489
KRT87P	transcribed_unprocesse	ENSG00000135477	0,183299104	0,454573948	0,563376136
RCC2P6	processed_pseudogene	ENSG00000254454	-0,170562133	0,454999423	0,563801243
CACNA1H	protein_coding	ENSG00000196557	0,156303615	0,454997352	0,563801243
KRBOX4	protein_coding	ENSG00000147121	0,045792397	0,454996494	0,563801243
GLB1L	protein_coding	ENSG00000163521	-0,073053176	0,455058569	0,563840467
SCPEP1	protein_coding	ENSG00000121064	-0,035885402	0,455140539	0,563907966
DPP7	protein_coding	ENSG00000176978	-0,05561237	0,455216849	0,563968442
EQTN	protein_coding	ENSG00000120160	0,162322023	0,455289788	0,564024737
EP400P1	transcribed_unprocesse	ENSG00000185684	0,070991381	0,455435836	0,564171589
AC067930.5	TEC	ENSG00000279605	0,198205022	0,455688205	0,56445012
TMEM56	protein_coding	ENSG00000152078	0,06849554	0,45577345	0,564521618
NAPB	protein_coding	ENSG00000125814	0,065587151	0,456090433	0,564880119
KLC2	protein_coding	ENSG00000174996	-0,058675497	0,456193385	0,564973512
FCGR2A	protein_coding	ENSG00000143226	0,16754514	0,456267318	0,565030956
FCGR2A	protein_coding	ENSG00000143226	0,16754514	0,456267318	0,565030956
QPRT	protein_coding	ENSG00000103485	-0,177926692	0,456308814	0,565048226
SNORD53B	snoRNA	ENSG00000265706	-0,196858345	0,45634667	0,565060987
CA5BP1	transcribed_unprocesse	ENSG00000186312	-0,075144477	0,456478997	0,565190717
RYK	protein_coding	ENSG00000163785	-0,025792919	0,456643748	0,565326448
CCNJ	protein_coding	ENSG00000107443	-0,057438648	0,456801149	0,565487178
B3GALT1	protein_coding	ENSG00000172318	-0,136832421	0,456992622	0,565690065
LY6G5B	protein_coding	ENSG00000240053	0,165115953	0,457118359	0,56581156
ANOS1	protein_coding	ENSG00000011201	0,052686198	0,457248581	0,565870294
UBA1	protein_coding	ENSG00000130985	0,041739428	0,457201079	0,565870294

GAREM2	protein_coding	ENSG00000157833	-0,152224844	0,457234514	0,565870294
SETD1B	protein_coding	ENSG00000139718	-0,088346819	0,457334537	0,565942521
USF3	protein_coding	ENSG00000176542	0,067754574	0,45746422	0,566068847
ITGA11	protein_coding	ENSG00000137809	-0,195361148	0,457506	0,566086393
FCF1P2	transcribed_processed_	ENSG00000228638	0,098235526	0,457548818	0,566105221
AC069544.1	lincRNA	ENSG00000272853	-0,185457102	0,45763034	0,566171931
AL138820.1	lincRNA	ENSG00000277151	-0,198374768	0,457697863	0,566187163
MIOS	protein_coding	ENSG00000164654	0,037736727	0,457691104	0,566187163
C10orf143	protein_coding	ENSG00000237489	0,198430716	0,457818053	0,566301687
GPT2	protein_coding	ENSG00000166123	0,07880876	0,458053968	0,566553019
AC100797.1	sense_intronic	ENSG00000254207	0,198337639	0,458076486	0,566553019
ACTR3B	protein_coding	ENSG00000133627	0,098100063	0,458107697	0,566557457
KCNK13	protein_coding	ENSG00000152315	0,198143214	0,45823635	0,566640794
GLI3	protein_coding	ENSG00000106571	-0,106444017	0,458257965	0,566640794
AC138951.2	TEC	ENSG00000279107	0,164093163	0,458225443	0,566640794
RN7SL144P	misc_RNA	ENSG00000242559	-0,154385718	0,45832767	0,56669282
RPL37P2	processed_pseudogene	ENSG00000239559	-0,182574144	0,458706804	0,567127407
SERBP1P5	processed_pseudogene	ENSG00000249565	-0,151479965	0,458758085	0,56715662
H3F3AP4	processed_pseudogene	ENSG00000235655	-0,080651477	0,458841996	0,567199785
MIGA2	protein_coding	ENSG00000148343	0,074022297	0,45884831	0,567199785
AC011472.1	antisense	ENSG00000267082	0,194409697	0,459485965	0,567953784
AL590560.2	antisense	ENSG00000272668	0,19638633	0,459818072	0,568330038
YIPF2	protein_coding	ENSG00000130733	-0,055906999	0,459886429	0,568380275
LPIN3	protein_coding	ENSG00000132793	-0,072351001	0,460031965	0,568525886
AC110079.1	transcribed_unprocesse	ENSG00000260404	0,196886002	0,460135137	0,568619128
RPL7P18	processed_pseudogene	ENSG00000243806	0,186778417	0,460206817	0,568673444
CCDC142	protein_coding	ENSG00000135637	-0,088903854	0,4603323	0,568759968
SNORA47	snoRNA	ENSG00000238961	-0,186099877	0,460312657	0,568759968
CFAP52	protein_coding	ENSG00000166596	-0,186563338	0,460461414	0,568885224
ENPP5	protein_coding	ENSG00000112796	0,150141845	0,460505312	0,568905189
AL669831.1	transcribed_unprocesse	ENSG00000228327	0,190333607	0,460894035	0,569351121
MDK	protein_coding	ENSG00000110492	-0,155955227	0,460956872	0,56939445
ITGA6-AS1	antisense	ENSG00000232788	0,188785406	0,461114583	0,569554959
PHF20L1	protein_coding	ENSG00000129292	0,040831463	0,461274418	0,569718072
WASH7P	unprocessed_pseudoge	ENSG00000227232	0,146422145	0,461381112	0,569815536
CHKA	protein_coding	ENSG00000110721	0,056318305	0,461790964	0,570287372
HSPB11	protein_coding	ENSG00000081870	-0,05001883	0,461852453	0,570328967
BCRP2	transcribed_unprocesse	ENSG00000169668	-0,153767691	0,462021561	0,570503445
TCP10L	protein_coding	ENSG00000242220	-0,195041022	0,462083859	0,57054602
RPS20P14	processed_pseudogene	ENSG00000223803	0,185539	0,462156518	0,570601384

CCDC180	protein_coding	ENSG00000197816	-0,173341264	0,462197849	0,570618063
AL161457.2	antisense	ENSG00000233178	-0,16519433	0,462297661	0,570706936
LINC01725	lincRNA	ENSG00000233008	0,163916264	0,462370465	0,570728107
PCSK7	protein_coding	ENSG00000160613	-0,062321142	0,462352231	0,570728107
AP000919.1	transcribed_processed_	ENSG00000263606	0,180549361	0,462419916	0,570754799
ZNF700	protein_coding	ENSG00000196757	0,061225433	0,462646083	0,570999588
EBAG9	protein_coding	ENSG00000147654	0,060263334	0,462945119	0,571334279
AL122008.2	processed_pseudogene	ENSG00000232166	0,164007393	0,463064655	0,571447417
DLG5-AS1	processed_transcript	ENSG00000233871	0,190964655	0,463135252	0,571500152
PDE9A	protein_coding	ENSG00000160191	-0,122277932	0,463254569	0,571612995
RAP1GDS1	protein_coding	ENSG00000138698	0,03060428	0,46337006	0,571721105
TIMM8B	protein_coding	ENSG00000150779	-0,05707069	0,463478825	0,571820904
LINC01589	antisense	ENSG00000238120	-0,105771306	0,46360879	0,571918096
PLP1	protein_coding	ENSG00000123560	0,176615632	0,463641257	0,571918096
USP16	protein_coding	ENSG00000156256	-0,049008687	0,463615923	0,571918096
EEF1GP1	processed_pseudogene	ENSG00000186676	0,180817228	0,463767389	0,572039281
AC020915.2	sense_intronic	ENSG00000268201	0,192001003	0,463919336	0,572192289
TSPAN6	protein_coding	ENSG00000000003	0,051399784	0,464178824	0,572477912
FAM19A3	protein_coding	ENSG00000184599	-0,190408434	0,464234972	0,572512734
RN7SL65P	misc_RNA	ENSG00000243770	0,165377315	0,464294062	0,57255118
PINK1	protein_coding	ENSG00000158828	0,035666928	0,464641761	0,572945501
SEC13	protein_coding	ENSG00000157020	0,040807509	0,464838833	0,57315405
AL732292.2	lincRNA	ENSG00000273416	0,189810789	0,46499899	0,573306175
ETV4	protein_coding	ENSG00000175832	-0,046640863	0,465046067	0,573306175
RASA2-IT1	sense_intronic	ENSG00000250170	0,182189217	0,465027136	0,573306175
SINHCAFP1	processed_pseudogene	ENSG00000263829	0,156690964	0,465214525	0,573404128
HLA-DQA1	protein_coding	ENSG00000196735	0,189374793	0,465225554	0,573404128
NOXA1	protein_coding	ENSG00000188747	0,095739883	0,465240311	0,573404128
CENPQ	protein_coding	ENSG00000031691	-0,060076286	0,465251573	0,573404128
MGAT5B	protein_coding	ENSG00000167889	-0,070793084	0,46526531	0,573404128
AL512288.2	processed_pseudogene	ENSG00000238231	-0,167559765	0,465320328	0,573437477
DOCK4	protein_coding	ENSG00000128512	-0,043548109	0,465566598	0,573706496
GNMT	protein_coding	ENSG00000124713	0,160855461	0,465674381	0,573804839
GNMT	protein_coding	ENSG00000124713	0,160855461	0,465674381	0,573804839
AP000347.1	transcribed_unprocesse	ENSG00000272578	-0,191629305	0,465903496	0,574052667
PSMD13	protein_coding	ENSG00000185627	-0,048092746	0,466018308	0,574125151
IGDCC3	protein_coding	ENSG00000174498	-0,171384333	0,46599185	0,574125151
CARD6	protein_coding	ENSG00000132357	0,195140129	0,466328045	0,574472233
RPS20	protein_coding	ENSG00000008988	-0,065019832	0,466388448	0,574477633
LYSMD4	protein_coding	ENSG00000183060	-0,101167145	0,466367241	0,574477633

KRT18P15	processed_pseudogene	ENSG00000234737	0,188253497	0,466659232	0,574776655
AC092119.2	lincRNA	ENSG00000274460	0,190428324	0,46683657	0,574960551
BX322234.1	antisense	ENSG00000226445	0,185087641	0,466956345	0,575073534
OTUD7A	protein_coding	ENSG00000169918	0,169487343	0,467050724	0,575155229
KRT18P17	processed_pseudogene	ENSG00000213943	-0,193970665	0,467381366	0,575458747
UPK1B	protein_coding	ENSG00000114638	0,176897032	0,467334889	0,575458747
NKAPP1	transcribed_processed_	ENSG00000233382	0,163334195	0,467364564	0,575458747
AL137784.1	processed_pseudogene	ENSG00000219755	0,178202718	0,467514789	0,57558847
DNAH17-AS1	processed_transcript	ENSG00000267432	-0,192551125	0,467560258	0,575609897
R3HDM4	protein_coding	ENSG00000198858	-0,050815749	0,46764809	0,575683472
SCP2	protein_coding	ENSG00000116171	-0,04092657	0,467717941	0,575734904
UQCRC1	protein_coding	ENSG00000010256	-0,049350278	0,467829174	0,575837266
DCDC2	protein_coding	ENSG00000146038	0,097413369	0,467908303	0,575877029
OR2A9P	transcribed_unprocesse	ENSG00000228960	0,185480201	0,467917635	0,575877029
AC021148.1	transcribed_processed_	ENSG00000224097	0,177622603	0,468046702	0,576001311
WASH5P	transcribed_processed_	ENSG00000282458	-0,172815156	0,468108976	0,576043385
ISM1	protein_coding	ENSG00000101230	0,184057508	0,468330889	0,57628189
AL122035.1	antisense	ENSG00000258824	0,190301749	0,469009673	0,577047894
AC018868.1	processed_pseudogene	ENSG00000231409	0,181957884	0,469002292	0,577047894
AC090587.2	sense_overlapping	ENSG00000229368	-0,191705828	0,469123601	0,577153444
AC073848.1	lincRNA	ENSG00000251679	0,191769121	0,469378448	0,577405415
AC003101.1	lincRNA	ENSG00000228768	0,178550304	0,469384714	0,577405415
LSM3	protein_coding	ENSG00000170860	-0,055200603	0,469547196	0,577570649
TCF7	protein_coding	ENSG00000081059	0,184994202	0,469586198	0,577583983
PPHLN1	protein_coding	ENSG00000134283	-0,045173188	0,469696623	0,577685161
FAAP20	protein_coding	ENSG00000162585	-0,06358838	0,469906291	0,577908378
AKR1B1	protein_coding	ENSG00000085662	-0,04186877	0,470182222	0,578213057
AP002840.2	sense_intronic	ENSG00000270179	-0,189468108	0,470211498	0,578214391
LIN7B	protein_coding	ENSG00000104863	-0,087701846	0,470715845	0,578765181
SPCS3	protein_coding	ENSG00000129128	0,051835967	0,470790788	0,578787931
GAK	protein_coding	ENSG00000178950	-0,045950671	0,470784968	0,578787931
WDR13	protein_coding	ENSG00000101940	-0,04609279	0,471295265	0,579373405
AC079953.1	processed_pseudogene	ENSG00000241744	0,169246434	0,47137146	0,579406845
DENND4C	protein_coding	ENSG00000137145	0,042771868	0,471378967	0,579406845
PPP4R1-AS1	lincRNA	ENSG00000263627	0,160564362	0,471693888	0,579752638
PSMA6P1	processed_pseudogene	ENSG00000215414	0,125672256	0,471716822	0,579752638
AC010983.1	lincRNA	ENSG00000235056	0,159438799	0,472024315	0,580061036
ACTG1P14	processed_pseudogene	ENSG00000230581	0,192054528	0,472154081	0,580185741
AL021368.2	lincRNA	ENSG00000272316	0,156948858	0,472336749	0,580352751
SNX25	protein_coding	ENSG00000109762	-0,040369826	0,472346586	0,580352751

HOXA2	protein_coding	ENSG00000105996	-0,163405103	0,472460691	0,580458175
MUC1	protein_coding	ENSG00000185499	0,170593201	0,47252779	0,580505838
AL096677.1	antisense	ENSG00000234832	0,168420573	0,472637265	0,580605553
SKAP1	protein_coding	ENSG00000141293	0,119235176	0,472715592	0,580666994
SPRY4-AS1	antisense	ENSG00000231185	-0,110250591	0,472806243	0,580708788
LINC01836	lincRNA	ENSG00000267530	0,108487131	0,472789487	0,580708788
ACTR3	protein_coding	ENSG00000115091	0,03780487	0,473111077	0,581013603
TOR1B	protein_coding	ENSG00000136816	-0,039922525	0,473085477	0,581013603
AC010422.2	lincRNA	ENSG00000268945	0,170264223	0,473240691	0,581137982
TNFRSF13C	protein_coding	ENSG00000159958	-0,162593179	0,473320973	0,58120177
AC137932.3	antisense	ENSG00000268218	0,187061382	0,473424271	0,581251611
AC048380.2	TEC	ENSG00000278983	0,191489972	0,473406977	0,581251611
AC016582.2	transcribed_processed_	ENSG00000267422	0,191863086	0,473446582	0,581251611
KIF21B	protein_coding	ENSG00000116852	-0,101300565	0,473543755	0,581336112
ZNF567	protein_coding	ENSG00000189042	-0,055970866	0,473673222	0,581460245
AC104024.2	lincRNA	ENSG00000260328	0,176324158	0,474049927	0,581887845
AL844908.2	sense_intronic	ENSG00000273027	-0,170862003	0,474272239	0,58212589
SNORA15	snoRNA	ENSG00000207168	0,18164552	0,474302004	0,582127587
AC006213.6	sense_intronic	ENSG00000278492	-0,189521928	0,474461757	0,582288813
HID1	protein_coding	ENSG00000167861	0,190011631	0,474556869	0,582370692
CHRFAM7A	protein_coding	ENSG00000166664	-0,159100181	0,474659741	0,582462085
PCDHGA7	protein_coding	ENSG00000253537	0,166261172	0,474747696	0,582465469
PCED1A	protein_coding	ENSG00000132635	0,061580962	0,474736689	0,582465469
NECAP1	protein_coding	ENSG00000089818	0,045830172	0,474744757	0,582465469
AC097721.1	processed_pseudogene	ENSG00000232915	-0,159623259	0,475019479	0,582729202
DHX37	protein_coding	ENSG00000150990	-0,048272727	0,475012648	0,582729202
AC100786.1	antisense	ENSG00000246731	0,189879542	0,475204441	0,582921237
CCPG1	protein_coding	ENSG00000260916	0,073950253	0,475363629	0,583081635
ZDHHC11	protein_coding	ENSG00000188818	0,108153293	0,475435127	0,583134461
PCDHGA3	protein_coding	ENSG00000254245	0,186812051	0,475519242	0,583167881
AC012676.5	TEC	ENSG00000280063	0,182885304	0,475502227	0,583167881
ZNF843	protein_coding	ENSG00000176723	0,183937905	0,475669086	0,583316768
AC107464.3	antisense	ENSG00000272927	-0,157639695	0,475732624	0,583359806
CPSF1P1	transcribed_processed_	ENSG00000214076	-0,154117364	0,47612181	0,583802135
SPAG8	protein_coding	ENSG00000137098	0,184943911	0,476238935	0,583875938
GDE1	protein_coding	ENSG00000006007	-0,033563798	0,476225819	0,583875938
AGGF1P1	processed_pseudogene	ENSG00000214273	0,158806894	0,476320839	0,583941447
RPS14P4	processed_pseudogene	ENSG00000226928	0,145083084	0,476615327	0,584267549
KCNJ4	protein_coding	ENSG00000168135	0,190021236	0,476653856	0,584279858
TPI1P1	processed_pseudogene	ENSG00000226415	0,128003074	0,477012372	0,584684381

TARS	protein_coding	ENSG00000113407	-0,039170155	0,477048333	0,584693377
SAMD15	protein_coding	ENSG00000100583	-0,108386854	0,477076727	0,584693377
GAPDHP71	processed_pseudogene	ENSG00000213376	0,153269947	0,477134407	0,584729129
AC017083.2	antisense	ENSG00000273275	0,172320131	0,477328877	0,584850488
AP001439.1	antisense	ENSG00000224541	0,157523062	0,477342692	0,584850488
ZNF713	protein_coding	ENSG00000178665	0,163381276	0,477347498	0,584850488
MACF1	protein_coding	ENSG00000127603	0,05783082	0,477331347	0,584850488
MACF1	protein_coding	ENSG00000127603	0,05783082	0,477331347	0,584850488
H6PD	protein_coding	ENSG00000049239	-0,054623669	0,47755135	0,5850653
ZBTB4	protein_coding	ENSG00000174282	0,057626939	0,477799931	0,585334881
AC091153.3	antisense	ENSG00000244184	0,187964922	0,477864877	0,58537948
AP1G1	protein_coding	ENSG00000166747	0,02592405	0,477910615	0,585400544
VPS33B-DT	antisense	ENSG00000214432	0,189389224	0,478003121	0,58547889
C10orf25	antisense	ENSG00000165511	0,068967124	0,478074447	0,585531286
KCTD21-AS1	antisense	ENSG00000246174	0,112414792	0,478220836	0,585675604
GNAQ	protein_coding	ENSG00000156052	-0,047010799	0,478287475	0,585722242
AC021205.3	antisense	ENSG00000273007	0,184688005	0,478519242	0,585971082
CXXC4	protein_coding	ENSG00000168772	0,175895787	0,478640352	0,586084396
PRKXP1	processed_pseudogene	ENSG00000259205	0,161037722	0,478776419	0,586216008
RN7SL608P	misc_RNA	ENSG00000239884	-0,171467978	0,478809956	0,586222075
CCER2	protein_coding	ENSG00000262484	-0,175585572	0,478887924	0,586282536
AC008438.1	antisense	ENSG00000249637	0,187803498	0,479341981	0,586795174
SHF	protein_coding	ENSG00000138606	-0,186927276	0,479363878	0,586795174
RBM27	protein_coding	ENSG00000091009	0,061746342	0,479413211	0,586820539
TUBG2	protein_coding	ENSG00000037042	0,057167273	0,479502676	0,586895022
AP001893.1	antisense	ENSG00000254694	0,181980979	0,479566389	0,586937978
E2F3-IT1	sense_intronic	ENSG00000224707	0,167049176	0,47988899	0,587297762
SPIB	protein_coding	ENSG00000269404	-0,187126219	0,479984389	0,587379464
ANO1	protein_coding	ENSG00000131620	0,151168497	0,480103617	0,587490316
BDNF	protein_coding	ENSG00000176697	-0,032857865	0,480483587	0,587920199
AC025176.1	lincRNA	ENSG00000250081	0,18317247	0,480614051	0,587954299
AP002414.4	processed_pseudogene	ENSG00000267533	-0,157233108	0,480571662	0,587954299
AC021087.2	processed_pseudogene	ENSG00000250848	-0,164426163	0,480562315	0,587954299
TKT	protein_coding	ENSG00000163931	-0,032356484	0,480626123	0,587954299
LINC02249	lincRNA	ENSG00000225930	-0,166565653	0,481059386	0,588449217
UBE2D3	protein_coding	ENSG00000109332	0,032917139	0,481480079	0,588928699
LINC01001	lincRNA	ENSG00000230724	0,16769738	0,481565328	0,588997846
RPA4	protein_coding	ENSG00000204086	0,176340543	0,481694043	0,589120145
LMF2	protein_coding	ENSG00000100258	0,058737711	0,481816549	0,589199703
EXOSC1	protein_coding	ENSG00000171311	-0,047863514	0,481792782	0,589199703

DEF6	protein_coding	ENSG00000023892	0,18703348	0,481874384	0,589235296
CASC4	protein_coding	ENSG00000166734	0,034815286	0,48194361	0,589284813
AD000864.1	protein_coding	ENSG00000167604	0,114471464	0,482204385	0,589568521
MXRA8	protein_coding	ENSG00000162576	0,18426267	0,482268876	0,589612222
SUPT4H1	protein_coding	ENSG00000213246	-0,041523167	0,482379745	0,589712617
OSBPL1A	protein_coding	ENSG00000141447	-0,041510836	0,48250382	0,589829143
GOLGA8B	protein_coding	ENSG00000215252	0,136350496	0,482690322	0,590021963
CTBP2P8	processed_pseudogene	ENSG00000234383	0,163906211	0,482729966	0,590035256
TCTE3	protein_coding	ENSG00000184786	-0,172910256	0,4828071	0,59009437
NACA3P	processed_pseudogene	ENSG00000121089	-0,178171365	0,482858078	0,59012151
CASC8	antisense	ENSG00000246228	-0,163060413	0,482906328	0,590145313
AC008895.1	TEC	ENSG00000279948	-0,179678312	0,482949845	0,59016333
TLN1	protein_coding	ENSG00000137076	-0,057962839	0,483030187	0,590226341
AL136116.3	processed_pseudogene	ENSG00000217648	-0,172775576	0,483432492	0,590673775
SCARNA5	scaRNA	ENSG00000252010	-0,137756528	0,483453957	0,590673775
DCTN6	protein_coding	ENSG00000104671	-0,048921945	0,483662731	0,590893651
CORO2A	protein_coding	ENSG00000106789	0,139770919	0,483907148	0,591157043
JMJD7-PLA2G4B	protein_coding	ENSG00000168970	0,177636748	0,48411564	0,591376521
AL158211.4	lincRNA	ENSG00000272516	0,177488145	0,484197997	0,591441898
TSSK6	protein_coding	ENSG00000178093	0,137166973	0,484233269	0,591449758
SZT2	protein_coding	ENSG00000198198	0,077474983	0,484481268	0,59171743
AL121894.2	lincRNA	ENSG00000270001	0,183482189	0,484633751	0,591756096
AC026462.1	processed_pseudogene	ENSG00000234337	0,18624821	0,484573893	0,591756096
FBXO15	protein_coding	ENSG00000141665	0,186088975	0,484657186	0,591756096
MAST3	protein_coding	ENSG00000099308	0,066484257	0,48463958	0,591756096
CCDC162P	transcribed_unitary_pse	ENSG00000203799	0,160667101	0,484552268	0,591756096
PARD6G-AS1	antisense	ENSG00000267270	0,176443148	0,48474625	0,591829608
TXNDC17	protein_coding	ENSG00000129235	-0,045481838	0,485099387	0,592190253
AL356512.1	antisense	ENSG00000272195	0,178858826	0,485270059	0,592328091
IRF2	protein_coding	ENSG00000168310	0,064319565	0,485251574	0,592328091
BX005266.2	lincRNA	ENSG00000226007	0,161316386	0,485562911	0,592615014
UBE3A	protein_coding	ENSG00000114062	-0,03932377	0,48555103	0,592615014
GNPTG	protein_coding	ENSG00000090581	-0,046565033	0,485729265	0,592782769
XKRX	protein_coding	ENSG00000182489	0,180041782	0,485930343	0,592992879
AC022893.1	antisense	ENSG00000253636	-0,182075439	0,486159769	0,593237557
ERICH5	protein_coding	ENSG00000177459	0,176652943	0,486424754	0,593525594
HCFC1R1	protein_coding	ENSG00000103145	0,062631148	0,486720806	0,593851501
PCAT7	antisense	ENSG00000231806	0,087264723	0,487054842	0,594188367
AL132640.1	processed_pseudogene	ENSG00000259502	0,180338293	0,487038087	0,594188367
TFIP11	protein_coding	ENSG00000100109	-0,031498424	0,487209004	0,594341086

AC025283.2	protein_coding	ENSG00000262621	0,180133435	0,487407041	0,594511948
DCTN3	protein_coding	ENSG00000137100	-0,05031435	0,487381382	0,594511948
MARCKSL1	protein_coding	ENSG00000175130	-0,044759122	0,487491145	0,594579173
AL513548.3	lincRNA	ENSG00000275846	-0,184158252	0,487646302	0,594714631
DHRS4L2	protein_coding	ENSG00000187630	-0,055699156	0,487660198	0,594714631
AC098484.2	lincRNA	ENSG00000234917	-0,181137887	0,487896135	0,594966985
RNU6-6P	snRNA	ENSG00000272055	0,183534188	0,487942239	0,594987831
VPS26C	protein_coding	ENSG00000157538	-0,030744104	0,488046123	0,595079126
PEX13	protein_coding	ENSG00000162928	0,039781819	0,488137648	0,595155342
AL161756.1	antisense	ENSG00000214770	0,181578894	0,488293298	0,595309727
AC005523.1	lincRNA	ENSG00000268536	-0,18426545	0,488592751	0,595568603
ARHGAP21	protein_coding	ENSG00000107863	0,040366582	0,48854884	0,595568603
AL049840.5	sense_intronic	ENSG00000270108	-0,173292971	0,488582834	0,595568603
ACSL1	protein_coding	ENSG00000151726	-0,043199839	0,48865747	0,595612094
HCG25	antisense	ENSG00000232940	0,163105742	0,488794229	0,595743382
ASPHD2	protein_coding	ENSG00000128203	-0,121477721	0,489034311	0,596000578
RPL10P16	processed_pseudogene	ENSG00000178464	0,146487437	0,48917275	0,596098455
FAM49B	protein_coding	ENSG00000153310	0,043212348	0,489161804	0,596098455
AL359397.2	lincRNA	ENSG00000259071	0,139096064	0,489337639	0,596229729
DTWD2	protein_coding	ENSG00000169570	-0,059157505	0,489338616	0,596229729
LINC01711	lincRNA	ENSG00000268941	0,184452441	0,489613549	0,596529279
PRELID3A	protein_coding	ENSG00000141391	0,099933188	0,489651334	0,596539879
AC091965.5	TEC	ENSG00000279075	-0,180831751	0,489762698	0,596640113
BAK1P1	processed_pseudogene	ENSG00000175730	0,155481643	0,489803747	0,596644776
RPS23P8	processed_pseudogene	ENSG00000230629	-0,099162434	0,489882888	0,596644776
AC139256.1	transcribed_processed_	ENSG00000260735	-0,178599527	0,489857905	0,596644776
AL645940.1	lincRNA	ENSG00000272217	0,179653158	0,490257603	0,597065699
RN7SKP166	misc_RNA	ENSG00000222744	0,150506597	0,490320916	0,597073568
AC008969.1	processed_transcript	ENSG00000176593	-0,094562182	0,490322287	0,597073568
COMMD7	protein_coding	ENSG00000149600	-0,047791429	0,490380766	0,597107318
C1orf226	protein_coding	ENSG00000239887	-0,10018648	0,490437341	0,597107318
RIMBP3	protein_coding	ENSG00000275793	-0,181346292	0,490431882	0,597107318
BTF3P10	processed_pseudogene	ENSG00000231120	-0,159504575	0,490549342	0,597208228
GPR173	protein_coding	ENSG00000184194	-0,179518645	0,490732532	0,597395787
OVCH1-AS1	antisense	ENSG00000257599	0,174342202	0,490879334	0,597498131
NOC2LP1	transcribed_processed_	ENSG00000213225	0,164611636	0,490903998	0,597498131
ARL6IP5	protein_coding	ENSG00000144746	0,047662737	0,491052963	0,597643975
ELOF1	protein_coding	ENSG00000130165	-0,043049669	0,491136019	0,597709592
ZNF234	protein_coding	ENSG00000263002	-0,043021634	0,491240158	0,597800857
EI24P2	processed_pseudogene	ENSG00000236257	-0,181609725	0,49136648	0,597919104

AL136379.1	lincRNA	ENSG00000276255	0,178299641	0,491492508	0,59796603
AC010422.1	lincRNA	ENSG00000230310	0,149727936	0,491488635	0,59796603
MARCH3	protein_coding	ENSG00000173926	-0,039975281	0,491472976	0,59796603
PAIP1P1	processed_pseudogene	ENSG00000233892	0,180862818	0,491890944	0,598415283
AC114980.1	sense_intronic	ENSG00000251023	-0,178125162	0,491979624	0,598487668
AP001462.1	lincRNA	ENSG00000269038	0,167890888	0,492013492	0,598490836
FRMD3	protein_coding	ENSG00000172159	0,183568694	0,492040589	0,598490836
PCDHGA8	protein_coding	ENSG00000253767	0,152405927	0,492081936	0,598505634
LINC02019	lincRNA	ENSG00000273356	-0,178941877	0,492152231	0,598555636
NAP1L1P1	processed_pseudogene	ENSG00000254759	0,182080716	0,492579277	0,599039489
AC009309.1	antisense	ENSG00000273080	-0,173580864	0,49299079	0,599504393
NPM1P6	processed_pseudogene	ENSG00000213881	0,127282312	0,49317553	0,599693491
AL590428.1	antisense	ENSG00000231652	-0,143647913	0,493302718	0,599812589
GULP1	protein_coding	ENSG00000144366	0,061607065	0,493496053	0,600012096
E2F8	protein_coding	ENSG00000129173	0,044667241	0,493806067	0,600353435
AC004951.2	transcribed_unprocesse	ENSG00000239556	0,178887959	0,493837517	0,600356083
PCDHGA4	protein_coding	ENSG00000262576	-0,17380116	0,493879078	0,600371022
C11orf58	protein_coding	ENSG00000110696	0,043045631	0,493934777	0,600391143
SKIV2L	protein_coding	ENSG00000204351	-0,039394482	0,493954176	0,600391143
RPS7P11	processed_pseudogene	ENSG00000213326	-0,126541376	0,494091702	0,600431679
TMA7	protein_coding	ENSG00000232112	0,038436374	0,494104626	0,600431679
PPFIBP2	protein_coding	ENSG00000166387	-0,072115268	0,494068452	0,600431679
AC073107.1	transcribed_unprocesse	ENSG00000272693	-0,156586445	0,494028356	0,600431679
PCDHGA5	protein_coding	ENSG00000253485	0,181482386	0,494188472	0,600497989
ZNF837	protein_coding	ENSG00000152475	0,139371058	0,494282783	0,600577007
SPEF2	protein_coding	ENSG00000152582	0,06611817	0,494389579	0,600671185
AL359183.1	TEC	ENSG00000279406	-0,149756597	0,494572159	0,600857421
AP001628.1	lincRNA	ENSG00000225218	-0,165209067	0,494635148	0,600862762
AC078817.1	processed_pseudogene	ENSG00000230291	0,179855264	0,494630896	0,600862762
ZNF527	protein_coding	ENSG00000189164	0,051522599	0,494743439	0,600958717
MYL6B	protein_coding	ENSG00000196465	-0,044054547	0,494786222	0,600975093
LRRC27	protein_coding	ENSG00000148814	-0,083930869	0,495000006	0,601199154
HERC2P10	unprocessed_pseudoge	ENSG00000259845	0,17361297	0,495289035	0,601479868
POLR2C	protein_coding	ENSG00000102978	-0,040263346	0,495809185	0,602074978
AC011462.1	protein_coding	ENSG00000105329	0,047890944	0,495982374	0,60224963
HCN3	protein_coding	ENSG00000143630	0,094197886	0,496030305	0,602272174
C9orf16	protein_coding	ENSG00000171159	0,076285907	0,49614736	0,602378639
SKI	protein_coding	ENSG00000157933	0,064637824	0,496235438	0,602449913
AC104695.4	sense_intronic	ENSG00000270640	-0,173890346	0,496423603	0,602642681
AL645608.2	lincRNA	ENSG00000230699	-0,177944988	0,496579884	0,602796723

TMEM134	protein_coding	ENSG00000172663	0,070693723	0,49671411	0,602923974
AL604028.2	processed_pseudogene	ENSG00000234329	-0,175548476	0,496915192	0,603096667
FPGS	protein_coding	ENSG00000136877	-0,049779377	0,496914709	0,603096667
DEF8	protein_coding	ENSG00000140995	-0,041674575	0,497046518	0,60322036
AL606760.2	antisense	ENSG00000236723	-0,176853408	0,49722309	0,603398945
TOM1	protein_coding	ENSG00000100284	-0,051324815	0,49737017	0,603541722
APBA1	protein_coding	ENSG00000107282	-0,144645633	0,497549708	0,603688151
AOX2P	transcribed_unprocesse	ENSG00000243478	0,168634469	0,49754007	0,603688151
TTC37	protein_coding	ENSG00000198677	-0,027857542	0,497605616	0,603720272
AC087588.2	sense_intronic	ENSG00000274976	-0,166554867	0,497774219	0,603889106
AC116913.1	antisense	ENSG00000261351	-0,156829708	0,498037326	0,603996892
EIF4A2P2	processed_pseudogene	ENSG00000227382	0,156698643	0,497897421	0,603996892
UBE2D1	protein_coding	ENSG00000072401	0,064141875	0,498013466	0,603996892
ZMIZ2	protein_coding	ENSG00000122515	0,058908864	0,497997174	0,603996892
TM2D1	protein_coding	ENSG00000162604	0,045319435	0,497952798	0,603996892
DYSF	protein_coding	ENSG00000135636	-0,051656938	0,498039758	0,603996892
DDX11L2	unprocessed_pseudoge	ENSG00000236397	-0,129995894	0,498244477	0,604209438
AC008894.2	antisense	ENSG00000269243	0,149358567	0,498568468	0,604566588
RABGGTA	protein_coding	ENSG00000100949	-0,035810073	0,498743088	0,60470683
LAT2	protein_coding	ENSG00000086730	0,033315592	0,498846585	0,604796563
SOCS2-AS1	processed_transcript	ENSG00000246985	0,087037506	0,498879467	0,604800678
ST13P21	processed_pseudogene	ENSG00000227832	0,173778457	0,499023976	0,604877663
ST7-OT4	sense_intronic	ENSG00000214188	0,154983275	0,499031445	0,604877663
BMS1P10	transcribed_unprocesse	ENSG00000237238	0,140346665	0,499029761	0,604877663
CDK14	protein_coding	ENSG00000058091	-0,044433438	0,499080874	0,604901827
NECAB2	protein_coding	ENSG00000103154	0,146769533	0,499174097	0,604943319
SLC40A1	protein_coding	ENSG00000138449	-0,178028948	0,499166116	0,604943319
AC093752.2	lincRNA	ENSG00000250950	0,155916825	0,499341621	0,605074834
UBE2L6	protein_coding	ENSG00000156587	0,05170299	0,499336818	0,605074834
TPD52L1	protein_coding	ENSG00000111907	0,178708113	0,499409303	0,605121097
CRYZL2P-SEC16B	processed_transcript	ENSG00000254154	-0,166369441	0,499449205	0,605133695
LINC01202	lincRNA	ENSG00000280776	0,177338309	0,499540089	0,605208059
AL391988.1	antisense	ENSG00000277879	-0,16184854	0,499817884	0,605508848
UBXN6	protein_coding	ENSG00000167671	0,042959369	0,499868566	0,60553448
AC147651.1	lincRNA	ENSG00000223855	0,177738883	0,49995962	0,605609013
THSD7B	protein_coding	ENSG00000144229	0,153246302	0,500156812	0,605812096
BEX3	protein_coding	ENSG00000166681	-0,029099206	0,500198214	0,605826466
BRI3	protein_coding	ENSG00000164713	-0,033399855	0,500270899	0,605842947
BTN3A3	protein_coding	ENSG00000111801	-0,07019076	0,500257006	0,605842947
HACE1	protein_coding	ENSG00000085382	-0,047112208	0,500524155	0,606113858

AC099778.1	antisense	ENSG00000260236	0,147000166	0,500743186	0,606271708
TMSB15B	protein_coding	ENSG00000158427	-0,116549742	0,50071352	0,606271708
C19orf33	protein_coding	ENSG00000167644	-0,145782218	0,500743098	0,606271708
VPS37C	protein_coding	ENSG00000167987	0,052941411	0,501031937	0,606549701
LONP2	protein_coding	ENSG00000102910	-0,034398005	0,501203762	0,6067219
AC087392.5	lincRNA	ENSG00000277491	0,178143714	0,501272151	0,606733066
AC010999.2	lincRNA	ENSG00000276524	-0,170780858	0,501255369	0,606733066
CST3	protein_coding	ENSG00000101439	0,050378618	0,501506351	0,606980719
AL080317.1	antisense	ENSG00000230177	0,172324533	0,501739772	0,60716067
RPS15AP10	processed_pseudogene	ENSG00000225447	-0,157314608	0,501699324	0,60716067
MFSD3	protein_coding	ENSG00000167700	0,082079543	0,501743841	0,60716067
AC091982.3	lincRNA	ENSG00000275765	0,093809067	0,501962316	0,607389209
AKR1C1	protein_coding	ENSG00000187134	0,174158204	0,502038075	0,607419972
AC009078.3	TEC	ENSG00000280152	0,140076591	0,50204697	0,607419972
CPLANE2	protein_coding	ENSG00000132881	0,066184894	0,502078349	0,607422104
AC012676.3	sense_intronic	ENSG00000277170	-0,159478728	0,502135689	0,607455644
AL512353.1	antisense	ENSG00000228192	0,166369368	0,502243065	0,607524992
LINC01186	lincRNA	ENSG00000236751	0,09185601	0,502252255	0,607524992
NABP1	protein_coding	ENSG00000173559	-0,052636868	0,502337585	0,607556544
FGF13	protein_coding	ENSG00000129682	-0,057758973	0,502310592	0,607556544
AL731569.1	antisense	ENSG00000227896	0,149380035	0,502496212	0,607607479
U47924.3	lincRNA	ENSG00000275703	0,143906859	0,502463682	0,607607479
RPL7P15	processed_pseudogene	ENSG00000240622	-0,148967239	0,502495626	0,607607479
AL023803.3	sense_intronic	ENSG00000277581	-0,174937783	0,502498199	0,607607479
AC087392.3	lincRNA	ENSG00000262228	-0,144909322	0,502545684	0,607629074
AC073912.2	lincRNA	ENSG00000256299	0,172899433	0,5027034	0,607783938
CARMN	lincRNA	ENSG00000249669	0,142180163	0,503218149	0,608298712
TMEM254	protein_coding	ENSG00000133678	-0,071287767	0,503206369	0,608298712
GPD1L	protein_coding	ENSG00000152642	-0,040465436	0,503507123	0,608612157
RF00019	misc_RNA	ENSG00000200502	-0,168118015	0,503544328	0,60862126
RPS3AP44	processed_pseudogene	ENSG00000234031	-0,14209466	0,503622201	0,608679514
EPM2A	protein_coding	ENSG00000112425	0,085048406	0,503704308	0,608742876
PDE6D	protein_coding	ENSG00000156973	-0,046340656	0,5037571	0,608770806
LINC002481	lincRNA	ENSG00000246526	0,147068355	0,503793589	0,608779033
AC064807.1	antisense	ENSG00000228801	0,134823839	0,503849112	0,608810256
RABEP2	protein_coding	ENSG00000177548	-0,054025381	0,50401786	0,60897828
UHMK1	protein_coding	ENSG00000152332	-0,032770286	0,504263306	0,60923895
JOSD2	protein_coding	ENSG00000161677	0,071537371	0,504382095	0,609346573
AC004771.1	antisense	ENSG00000227495	-0,175867558	0,504435581	0,609375295
AC055822.1	antisense	ENSG00000272155	-0,142681214	0,504490898	0,60939951

LYPLAL1	protein_coding	ENSG00000143353	-0,056265498	0,50451505	0,60939951
AC099343.3	bidirectional_promoter_	ENSG00000271646	0,129892729	0,504971761	0,609915248
NPR2	protein_coding	ENSG00000159899	0,109353011	0,505428455	0,610430904
ARSB	protein_coding	ENSG00000113273	0,036510309	0,505474739	0,610450857
ZFC3H1	protein_coding	ENSG00000133858	0,040681327	0,505825784	0,610838839
RPSAP61	processed_pseudogene	ENSG00000214016	0,176680737	0,505885094	0,610874494
AC021321.1	lincRNA	ENSG00000271966	-0,174018198	0,505929761	0,610892464
LINC01569	lincRNA	ENSG00000262468	0,146186403	0,506121364	0,611087842
ARRDC1-AS1	antisense	ENSG00000203993	-0,057979148	0,506233758	0,611187567
DGKZP1	processed_pseudogene	ENSG00000179611	0,170797617	0,506421444	0,611378175
LINC00637	lincRNA	ENSG00000258735	-0,176006315	0,506627077	0,611590427
DDO	protein_coding	ENSG00000203797	0,167296113	0,506909846	0,611895764
AC004951.4	processed_transcript	ENSG00000273432	0,171713548	0,506992172	0,611959125
Z93930.3	sense_intronic	ENSG00000272858	-0,156531066	0,507116603	0,612073297
NPM1P37	processed_pseudogene	ENSG00000219085	-0,172470537	0,507225757	0,612169017
F2RL2	protein_coding	ENSG00000164220	0,088497288	0,507464985	0,612421703
MAMLD1	protein_coding	ENSG00000013619	-0,093525331	0,507526189	0,612459528
AC010761.2	antisense	ENSG00000264577	0,174537293	0,507679728	0,612608768
CCL26	protein_coding	ENSG00000006606	-0,070972803	0,507780862	0,612694757
RBMS1P1	processed_pseudogene	ENSG00000225422	-0,174003133	0,507842301	0,612732843
MT2P1	processed_pseudogene	ENSG00000162840	0,167771158	0,507947651	0,612787855
ARFGAP1	protein_coding	ENSG00000101199	0,052474377	0,507944342	0,612787855
HSPA13	protein_coding	ENSG00000155304	0,048152717	0,508162618	0,613011133
AL136454.1	protein_coding	ENSG00000231767	0,172381796	0,508325972	0,613131857
NPDC1	protein_coding	ENSG00000107281	-0,068882477	0,508359234	0,613131857
AGMAT	protein_coding	ENSG00000116771	-0,119342257	0,508343812	0,613131857
PLEKHB1	protein_coding	ENSG00000021300	-0,159878344	0,50838227	0,613131857
FKBP11	protein_coding	ENSG00000134285	0,094543445	0,508446364	0,6131731
ACAA1	protein_coding	ENSG00000060971	-0,055784235	0,508514124	0,613218761
AC026801.2	antisense	ENSG00000272323	-0,173034584	0,508566462	0,613245819
SNORD8	snoRNA	ENSG00000200785	0,173995986	0,508645005	0,613304472
RPS7P10	processed_pseudogene	ENSG00000226525	0,146273021	0,508733968	0,61337568
AC025423.1	antisense	ENSG00000256325	-0,151795411	0,508876715	0,613508471
VTA1P2	processed_pseudogene	ENSG00000253401	0,142527687	0,50890393	0,613508471
NBPF9	protein_coding	ENSG00000269713	0,040152996	0,508995968	0,613583361
AP001816.1	lincRNA	ENSG00000254531	0,085700816	0,509150139	0,613733139
HSP90AB4P	processed_pseudogene	ENSG00000282100	0,17579515	0,509180081	0,61373316
ROCR	lincRNA	ENSG00000228639	0,145927323	0,509215907	0,613740274
LINC01505	lincRNA	ENSG00000234323	-0,167664656	0,509284295	0,613786631
GSTM3	protein_coding	ENSG00000134202	-0,053267198	0,510224146	0,614847076

TTC32	protein_coding	ENSG00000183891	-0,06727349	0,510207226	0,614847076
RPS28P7	processed_pseudogene	ENSG00000227097	-0,049268289	0,510278033	0,614875886
AL591848.4	lincRNA	ENSG00000260855	-0,13974558	0,510462721	0,615062296
IFI35	protein_coding	ENSG00000068079	-0,051552796	0,510738635	0,615358597
SUPT16HP1	processed_pseudogene	ENSG00000256238	-0,148989431	0,510824102	0,615359275
PCCA	protein_coding	ENSG00000175198	0,041980301	0,510772136	0,615359275
HSPD1P6	transcribed_processed_	ENSG00000230067	0,143573161	0,510829206	0,615359275
AC012640.3	lincRNA	ENSG00000271715	0,16142255	0,511533851	0,61617192
ALPK1	protein_coding	ENSG00000073331	0,038203793	0,511685275	0,616318122
GKAP1	protein_coding	ENSG00000165113	-0,081940163	0,512085825	0,616764359
ITM2C	protein_coding	ENSG00000135916	0,038952815	0,512146202	0,616800857
LINC01547	lincRNA	ENSG00000183250	0,08885742	0,512283805	0,616889889
LPCAT3	protein_coding	ENSG00000111684	0,053498432	0,51231036	0,616889889
GLUD1P2	transcribed_unprocesse	ENSG00000265366	-0,152409765	0,512273651	0,616889889
PWWP2B	protein_coding	ENSG00000171813	-0,058144833	0,512368708	0,616923929
SHROOM4	protein_coding	ENSG00000158352	-0,167649198	0,512488435	0,617031864
AL162615.1	processed_pseudogene	ENSG00000237788	-0,168753256	0,512556918	0,617078093
AL445228.2	lincRNA	ENSG00000271387	-0,172595551	0,5126717	0,617143829
GAPDHP1	processed_pseudogene	ENSG00000228232	-0,092349984	0,51265876	0,617143829
AL138963.3	antisense	ENSG00000273149	0,147842363	0,512973838	0,617471297
SLC25A26	protein_coding	ENSG00000144741	-0,040325422	0,513039039	0,617513538
ANKS1A	protein_coding	ENSG00000064999	0,050810009	0,51334946	0,617850914
AL035458.1	processed_pseudogene	ENSG00000236456	-0,168017563	0,513413562	0,617891806
MEMO1P1	processed_pseudogene	ENSG00000226054	0,133274875	0,51351012	0,617930277
GUCY2C	protein_coding	ENSG00000070019	0,166651735	0,513523529	0,617930277
DUSP15	protein_coding	ENSG00000149599	0,13605437	0,513535913	0,617930277
SF3B1	protein_coding	ENSG00000115524	-0,025929603	0,513640402	0,618019749
AC127070.1	antisense	ENSG00000236617	0,168083044	0,513946167	0,618351375
MED28	protein_coding	ENSG00000118579	0,033721136	0,51411418	0,618517236
AC131235.2	antisense	ENSG00000272721	-0,167418568	0,51415655	0,618531928
AC107982.3	lincRNA	ENSG00000265478	0,168521575	0,514332051	0,61867048
BCYRN1	scRNA	ENSG00000236824	0,15005563	0,514312906	0,61867048
AC105094.2	lincRNA	ENSG00000267175	-0,161654018	0,514403812	0,618720513
TBL2	protein_coding	ENSG00000106638	-0,03884884	0,514573098	0,618887834
CCDC149	protein_coding	ENSG00000181982	0,153694454	0,515111735	0,619468221
SLC52A2	protein_coding	ENSG00000185803	-0,05113908	0,515116066	0,619468221
LDOC1	protein_coding	ENSG00000182195	-0,047633313	0,515205778	0,619539781
PPP1R14BP2	processed_pseudogene	ENSG00000213082	0,132240234	0,515382905	0,619706589
TPM3P8	processed_pseudogene	ENSG00000183022	-0,158716203	0,515404924	0,619706589
DDX39BP2	unprocessed_pseudoge	ENSG00000238024	0,171493243	0,515486491	0,61976833

ALKBH3	protein_coding	ENSG00000166199	0,03854273	0,515606232	0,619875956
TPTE2P5	transcribed_unprocesse	ENSG00000168852	0,171991374	0,515643455	0,619884371
BMP5	protein_coding	ENSG00000112175	0,141527486	0,515794837	0,619921014
C6orf48	protein_coding	ENSG00000204387	0,049387971	0,515793659	0,619921014
RBM14-RBM4	protein_coding	ENSG00000248643	-0,169083162	0,515777465	0,619921014
AP005482.3	sense_intronic	ENSG00000267249	0,171682336	0,51574147	0,619921014
DET1	protein_coding	ENSG00000140543	-0,067590487	0,515890695	0,619999891
VRK2	protein_coding	ENSG00000028116	-0,040084559	0,516073039	0,620182694
KIAA1522	protein_coding	ENSG00000162522	-0,069459919	0,516283801	0,620399622
AC004466.1	antisense	ENSG00000268069	-0,165073145	0,516367787	0,620464193
AC090023.1	lincRNA	ENSG00000255866	0,140223493	0,516540983	0,620569445
DR1	protein_coding	ENSG00000117505	0,030484797	0,516546151	0,620569445
AC080188.2	TEC	ENSG00000279384	-0,171675098	0,516534154	0,620569445
MUC20-OT1	lincRNA	ENSG00000242086	0,06466145	0,51658252	0,620576788
AL353622.1	antisense	ENSG00000270605	0,170852113	0,516774373	0,620676839
TOB1-AS1	processed_transcript	ENSG00000229980	0,145411859	0,516786854	0,620676839
DTD1	protein_coding	ENSG00000125821	-0,036165063	0,516782158	0,620676839
AL139241.1	antisense	ENSG00000230928	-0,150590429	0,517043069	0,620832965
AC007878.1	lincRNA	ENSG00000281195	0,146972951	0,517005483	0,620832965
CEP70	protein_coding	ENSG00000114107	-0,04320757	0,517050594	0,620832965
SOS1-IT1	sense_intronic	ENSG00000229692	-0,161209708	0,517017269	0,620832965
SNORA30B	snoRNA	ENSG00000202189	0,14557698	0,517068196	0,620832965
AC092902.4	processed_transcript	ENSG00000284624	-0,164418001	0,517226099	0,620986202
TTC17	protein_coding	ENSG00000052841	0,034816321	0,517328941	0,621073319
AC011472.2	3prime_overlapping_nc	ENSG00000267174	0,144408276	0,517487002	0,621226714
TPK1	protein_coding	ENSG00000196511	0,070777546	0,51757931	0,621301162
RPS2P5	processed_pseudogene	ENSG00000240342	-0,035967369	0,517910506	0,621662345
HIPK1-AS1	antisense	ENSG00000235527	0,16572462	0,518072206	0,621783658
RF00019	misc_RNA	ENSG00000201573	0,17090179	0,518240553	0,621949312
PHPT1	protein_coding	ENSG00000054148	-0,047094014	0,518282841	0,621963669
AL121983.1	lincRNA	ENSG00000224977	0,149729839	0,518377164	0,622040465
FZD9	protein_coding	ENSG00000188763	-0,134128302	0,518644601	0,622324973
AC023355.1	antisense	ENSG00000259488	-0,155982889	0,518692138	0,622345604
SNHG18	lincRNA	ENSG00000250786	0,100558214	0,519238577	0,62294575
AC068134.3	processed_pseudogene	ENSG00000251485	0,14874622	0,519253073	0,62294575
FAM210A	protein_coding	ENSG00000177150	0,040396468	0,519435494	0,62312815
AC007382.1	TEC	ENSG00000279519	-0,135207564	0,519471011	0,62313431
IL17B	protein_coding	ENSG00000127743	0,166083419	0,51950153	0,623134475
RPL23AP42	processed_pseudogene	ENSG00000234851	-0,087867827	0,519812481	0,623434535
GSTCD	protein_coding	ENSG00000138780	0,039680372	0,519798173	0,623434535

AP001363.2	antisense	ENSG00000257058	0,1618005	0,520130206	0,623779121
GDI2P2	processed_pseudogene	ENSG00000233994	0,169425777	0,520715108	0,624407558
CDC40	protein_coding	ENSG00000168438	0,035355796	0,520778547	0,62444712
U47924.2	antisense	ENSG00000272173	0,160857544	0,520859625	0,624500594
TPTE2P1	transcribed_unprocesse	ENSG00000253771	0,169610732	0,52088404	0,624500594
SH3BP2	protein_coding	ENSG00000087266	0,063041407	0,521024949	0,62463302
YWHABP2	processed_pseudogene	ENSG00000256464	0,16744873	0,521135331	0,624728836
NPIPA1	protein_coding	ENSG00000183426	-0,149658032	0,521607539	0,625258365
NPIPA1	protein_coding	ENSG00000183426	-0,149658032	0,521607539	0,625258365
PRELID3B	protein_coding	ENSG00000101166	-0,048385386	0,521707452	0,625341584
NIPA1	protein_coding	ENSG00000170113	0,038902157	0,521855539	0,625482533
ETFB	protein_coding	ENSG00000105379	-0,046994119	0,521923457	0,625527383
TMC3-AS1	antisense	ENSG00000259343	0,169424295	0,521957582	0,625531729
AC083899.1	unprocessed_pseudoge	ENSG00000204745	0,0623393	0,522003633	0,625550366
DACH1	protein_coding	ENSG00000276644	0,167560464	0,522221174	0,625774496
AL513165.1	antisense	ENSG00000234160	-0,118960642	0,522513405	0,626031264
DCLRE1C	protein_coding	ENSG00000152457	0,04345117	0,522468202	0,626031264
MREG	protein_coding	ENSG00000118242	-0,05392495	0,522527021	0,626031264
BRWD1-AS2	antisense	ENSG00000255568	-0,168278325	0,522589442	0,626069478
CDRT1	protein_coding	ENSG00000241322	0,151862082	0,5227117	0,626179369
ACLY	protein_coding	ENSG00000131473	-0,02194373	0,522745748	0,626183581
AC135048.3	lincRNA	ENSG00000275263	-0,144871841	0,522860187	0,626284088
PITRM1-AS1	antisense	ENSG00000237399	-0,161692783	0,523049939	0,626328482
TACR2	protein_coding	ENSG00000075073	0,169772449	0,523003081	0,626328482
NPIPA5	protein_coding	ENSG00000183793	0,165328837	0,52304512	0,626328482
STK10	protein_coding	ENSG00000072786	0,047007454	0,522999871	0,626328482
TCF12	protein_coding	ENSG00000140262	-0,023122458	0,522952271	0,626328482
HABP4	protein_coding	ENSG00000130956	0,040408837	0,523126997	0,626384185
MIPOL1	protein_coding	ENSG00000151338	-0,056981132	0,523444067	0,626727251
PRKCQ	protein_coding	ENSG00000065675	0,163568169	0,523477427	0,626730607
AC114488.2	antisense	ENSG00000235790	0,143607216	0,523720327	0,626971533
AL512343.2	antisense	ENSG00000272562	-0,158331238	0,523757128	0,626971533
NRSN2	protein_coding	ENSG00000125841	-0,054317373	0,523954733	0,627155622
LMO2	protein_coding	ENSG00000135363	0,151968733	0,524036185	0,627216513
WDR27	protein_coding	ENSG00000184465	0,060894441	0,524108761	0,627266773
HAVCR2	protein_coding	ENSG00000135077	-0,130690281	0,524257191	0,627407807
AC017083.1	antisense	ENSG00000273064	-0,165306462	0,524294291	0,627415597
AC093510.1	processed_transcript	ENSG00000255647	-0,167149217	0,52453443	0,627666346
AL121832.3	sense_intronic	ENSG00000275437	-0,167543984	0,524629284	0,627743225
SLC25A35	protein_coding	ENSG00000125434	0,107858339	0,524840954	0,627959863

ASH2L	protein_coding	ENSG00000129691	0,036343849	0,524980393	0,628053422
COMMD5	protein_coding	ENSG00000170619	-0,053525672	0,524971249	0,628053422
AC131159.2	antisense	ENSG00000277873	0,148990316	0,525081456	0,628137689
MTCP1	protein_coding	ENSG00000214827	-0,155701946	0,525136306	0,628166664
AC114956.1	antisense	ENSG00000248240	0,145259232	0,525417398	0,628391329
AGTR1	protein_coding	ENSG00000144891	0,058131731	0,525406746	0,628391329
SPNS1	protein_coding	ENSG00000169682	-0,165574052	0,525446674	0,628391329
ZNF711	protein_coding	ENSG00000147180	-0,05027744	0,525555694	0,628485062
ACOT13	protein_coding	ENSG00000112304	-0,051290302	0,525647323	0,628557987
AC018638.5	processed_pseudogene	ENSG00000243679	0,142154742	0,525812262	0,628718562
PCNPP5	processed_pseudogene	ENSG00000177197	-0,153448799	0,52612457	0,629032008
ELOC	protein_coding	ENSG00000154582	0,044727886	0,526166413	0,629032008
DNM1L	protein_coding	ENSG00000087470	-0,030123249	0,526141763	0,629032008
FHIT	protein_coding	ENSG00000189283	0,166258433	0,526348176	0,629193641
SIAE	protein_coding	ENSG00000110013	0,049525609	0,526362969	0,629193641
AC132938.3	antisense	ENSG00000265458	-0,152844038	0,526494205	0,629313838
SUCLG2P2	processed_pseudogene	ENSG00000236349	-0,157695523	0,52655778	0,629353151
APBB3	protein_coding	ENSG00000113108	-0,082057472	0,526594304	0,629360128
AC087683.1	processed_pseudogene	ENSG00000267088	0,138297782	0,526672484	0,629398191
SSR4P1	transcribed_processed_	ENSG00000235374	-0,119208802	0,526687526	0,629398191
AC055839.1	processed_pseudogene	ENSG00000282980	0,151377189	0,526799341	0,629458461
C1GALT1C1L	protein_coding	ENSG00000223658	0,166495345	0,526769853	0,629458461
CBX3P9	processed_pseudogene	ENSG00000217241	0,167378125	0,52705463	0,629726813
RAB43P1	processed_pseudogene	ENSG00000259856	0,167284473	0,527408379	0,630079397
AADACP1	transcribed_unprocesse	ENSG00000240602	-0,06554318	0,527411169	0,630079397
NAP1L4P3	processed_pseudogene	ENSG00000234145	0,164263215	0,527476141	0,630120313
SMC5-AS1	antisense	ENSG00000268364	-0,142011448	0,527524856	0,630141805
AC114956.3	processed_transcript	ENSG00000249492	0,167476623	0,527622796	0,630222093
AGAP12P	transcribed_unprocesse	ENSG00000265018	0,140041817	0,527747708	0,630334585
EPN2	protein_coding	ENSG00000072134	-0,031697655	0,52804927	0,630598532
CARD10	protein_coding	ENSG00000100065	-0,053708962	0,528043256	0,630598532
AC023055.1	protein_coding	ENSG00000257390	-0,164610141	0,528060936	0,630598532
SNRNP70	protein_coding	ENSG00000104852	0,033767399	0,528096398	0,630604165
HAPLN1	protein_coding	ENSG00000145681	0,163425645	0,528233431	0,630731074
PPBP	protein_coding	ENSG00000163736	-0,163640009	0,528570282	0,631096547
YBX3P1	processed_pseudogene	ENSG00000261614	0,152393308	0,529121903	0,631644854
STKLD1	protein_coding	ENSG00000198870	0,158562332	0,529101377	0,631644854
EML1	protein_coding	ENSG00000066629	0,041215077	0,529198891	0,631699992
AC129492.6	processed_pseudogene	ENSG00000271002	0,16351714	0,529299996	0,631714438
TPT1	protein_coding	ENSG00000133112	-0,054128153	0,529282186	0,631714438

AP003733.4	TEC	ENSG00000279491	0,156097538	0,529303395	0,631714438
LMBRD1	protein_coding	ENSG00000168216	0,05322176	0,529475601	0,631846429
RARS2	protein_coding	ENSG00000146282	-0,044455013	0,529755866	0,632144101
FBXO27	protein_coding	ENSG00000161243	0,068105958	0,529811967	0,632174265
RRH	protein_coding	ENSG00000180245	0,165776862	0,530329094	0,632726936
FAM129C	protein_coding	ENSG00000167483	0,164335706	0,530347397	0,632726936
NCSTN	protein_coding	ENSG00000162736	0,036679962	0,530367698	0,632726936
AL031847.1	antisense	ENSG00000226944	0,161173177	0,530582522	0,632946405
HLA-V	transcribed_unprocesse	ENSG00000181126	0,137395264	0,530632396	0,632969084
AL021068.2	processed_pseudogene	ENSG00000234604	-0,135715423	0,530681759	0,632991153
MTUS1	protein_coding	ENSG00000129422	-0,125255896	0,530727167	0,633008502
RPS16	protein_coding	ENSG00000105193	-0,058250428	0,53081866	0,633044001
CNTN1	protein_coding	ENSG00000018236	-0,164589786	0,530815642	0,633044001
FAM114A1	protein_coding	ENSG00000197712	-0,030485285	0,530926913	0,633136286
ANKRD10-IT1	sense_intronic	ENSG00000229152	0,13614705	0,5310829	0,633285481
H3F3A	protein_coding	ENSG00000163041	0,039301452	0,531140134	0,633316908
PICK1	protein_coding	ENSG00000100151	-0,049168492	0,531230276	0,633387569
CCDC24	protein_coding	ENSG00000159214	-0,069668963	0,531323641	0,633462063
AC011337.1	antisense	ENSG00000271795	0,166513104	0,531399573	0,633515767
ZNF283	protein_coding	ENSG00000167637	0,045853162	0,531474423	0,633568173
AC015674.1	antisense	ENSG00000264558	-0,142505559	0,531655382	0,633677101
AL136172.1	antisense	ENSG00000269846	-0,152434597	0,531661784	0,633677101
TMC4	protein_coding	ENSG00000167608	0,165394883	0,531689382	0,633677101
TSNAXIP1	protein_coding	ENSG00000102904	-0,164789707	0,531597584	0,633677101
MYZAP	protein_coding	ENSG00000263155	0,131746426	0,531741829	0,633702783
AL049712.1	antisense	ENSG00000228293	0,156121935	0,53180615	0,633742614
PARD3	protein_coding	ENSG00000148498	0,022257267	0,53188127	0,633758487
MIEN1	protein_coding	ENSG00000141741	-0,048400319	0,531858049	0,633758487
SEC63P1	processed_pseudogene	ENSG00000228057	0,142348511	0,532272966	0,634004212
FBXO2	protein_coding	ENSG00000116661	0,102739326	0,532223324	0,634004212
TMEM79	protein_coding	ENSG00000163472	0,090152432	0,53216783	0,634004212
FAM172A	protein_coding	ENSG00000113391	-0,043148474	0,532143232	0,634004212
AC079174.1	sense_intronic	ENSG00000258355	-0,14634344	0,532255199	0,634004212
SNORA63D	snoRNA	ENSG00000201229	0,153747167	0,532236664	0,634004212
AC079385.3	antisense	ENSG00000257918	0,162856886	0,532580194	0,63433332
GGA3	protein_coding	ENSG00000125447	-0,044357957	0,53267324	0,634407302
AL512633.1	processed_pseudogene	ENSG00000215284	0,136320434	0,532930819	0,634603523
AC018644.1	processed_pseudogene	ENSG00000229677	0,131844798	0,5329202	0,634603523
PDE10A	protein_coding	ENSG00000112541	0,147060184	0,532869708	0,634603523
ADCY5	protein_coding	ENSG00000173175	0,164666199	0,533019212	0,634671932

BAG1	protein_coding	ENSG00000107262	-0,038890632	0,533122447	0,634758004
CR1	protein_coding	ENSG00000203710	0,165496394	0,533235452	0,634855699
BTF3L4P2	processed_pseudogene	ENSG00000213189	0,159568237	0,533292517	0,634886784
SYDE1	protein_coding	ENSG00000105137	-0,041710981	0,533588115	0,635201825
HSPD1P1	processed_pseudogene	ENSG00000213430	-0,135940714	0,533748182	0,635318624
RNF170	protein_coding	ENSG00000120925	-0,041631368	0,533829954	0,635379083
MRPL3P1	processed_pseudogene	ENSG00000215349	0,144262254	0,53390023	0,635388982
GGTA2P	processed_pseudogene	ENSG00000237766	-0,149280138	0,533896589	0,635388982
AF001548.2	antisense	ENSG00000263335	0,164484174	0,534458863	0,63601567
GANAB	protein_coding	ENSG00000089597	-0,035263443	0,534495843	0,63601567
RPS4X	protein_coding	ENSG00000198034	-0,036720756	0,534519849	0,63601567
AC069155.1	antisense	ENSG00000233581	0,160837302	0,534553726	0,636019082
AC096537.1	bidirectional_promoter_	ENSG00000233384	0,10245571	0,534694789	0,636150015
TFCP2	protein_coding	ENSG00000135457	0,037571145	0,534858867	0,636293312
AL031729.1	processed_pseudogene	ENSG00000235912	0,161872608	0,535067422	0,636445678
CAV3	protein_coding	ENSG00000182533	-0,16348501	0,535065607	0,636445678
AL096828.3	lincRNA	ENSG00000273821	0,164149428	0,535167941	0,636528326
AC012467.2	antisense	ENSG00000271976	0,097033758	0,535292429	0,636602558
AC138392.1	processed_pseudogene	ENSG00000219201	0,159201319	0,535276836	0,636602558
SLC30A6	protein_coding	ENSG00000152683	-0,038564964	0,535405475	0,63668161
AC008752.3	unprocessed_pseudoge	ENSG00000267370	-0,161639729	0,535420986	0,63668161
PTGER1	protein_coding	ENSG00000160951	-0,150306744	0,535710514	0,636988964
GALNT4	protein_coding	ENSG00000257594	0,150008422	0,535997972	0,637241803
TMX3	protein_coding	ENSG00000166479	0,035360681	0,536024352	0,637241803
USP33	protein_coding	ENSG00000077254	0,034406039	0,536022242	0,637241803
SULT4A1	protein_coding	ENSG00000130540	-0,082706754	0,536047432	0,637241803
AC246787.1	processed_pseudogene	ENSG00000225200	0,145811216	0,536210781	0,637399045
HOXC-AS3	processed_transcript	ENSG00000251151	-0,162228852	0,536249784	0,637408465
CHKB	protein_coding	ENSG00000100288	0,1620863	0,536574584	0,637757576
TAF7L	protein_coding	ENSG00000102387	-0,153929682	0,536610759	0,637763613
MYCT1	protein_coding	ENSG00000120279	0,161113521	0,536664356	0,637790355
PCDHB11	protein_coding	ENSG00000197479	0,162795569	0,536701799	0,637797897
AL390961.3	TEC	ENSG00000279212	-0,138930781	0,537215284	0,638371117
TGIF2	protein_coding	ENSG00000118707	0,042589405	0,537332064	0,638435907
P4HA2	protein_coding	ENSG00000072682	0,036005679	0,537310901	0,638435907
PPARG	protein_coding	ENSG00000132170	0,049768574	0,537697449	0,638833034
AL928654.4	TEC	ENSG00000279495	-0,080077828	0,537769038	0,63888108
SARM1	protein_coding	ENSG00000004139	-0,054081817	0,537983094	0,639098364
AL035071.1	lincRNA	ENSG00000260257	-0,080451385	0,538095027	0,639194313
EPHA5-AS1	lincRNA	ENSG00000250846	-0,155006276	0,538208971	0,639260483

GSK3B	protein_coding	ENSG00000082701	0,025994552	0,538213068	0,639260483
PHBP11	processed_pseudogene	ENSG00000227621	0,128686034	0,538469389	0,63949086
UBE2SP1	processed_pseudogene	ENSG00000233966	-0,106211171	0,538467832	0,63949086
BOLA2B	protein_coding	ENSG00000169627	-0,144830857	0,538564756	0,639567087
GSDMC	protein_coding	ENSG00000147697	0,134902338	0,53860365	0,639576242
AC110792.3	lincRNA	ENSG00000272650	-0,137728033	0,538729705	0,639688893
FIGN	protein_coding	ENSG00000182263	0,056354354	0,538977329	0,639902873
RHOC	protein_coding	ENSG00000155366	0,04579275	0,539003512	0,639902873
DDX39B	protein_coding	ENSG00000198563	-0,074341368	0,538975347	0,639902873
RBM8B	processed_pseudogene	ENSG00000258427	-0,155230245	0,539040226	0,639909419
NOXRED1	protein_coding	ENSG00000165555	0,16152924	0,539120168	0,639930242
ANKRD11	protein_coding	ENSG00000167522	0,055128088	0,539091007	0,639930242
AC024293.1	processed_pseudogene	ENSG00000244313	0,062082458	0,539504874	0,640241751
DNMT3A	protein_coding	ENSG00000119772	0,054940788	0,539520138	0,640241751
RAB5C	protein_coding	ENSG00000108774	0,041464735	0,539435496	0,640241751
SH3BP1	protein_coding	ENSG00000100092	-0,06653767	0,539538685	0,640241751
AL353898.3	TEC	ENSG00000280378	-0,158233581	0,539483536	0,640241751
AP000763.3	antisense	ENSG00000256448	0,145430672	0,539644807	0,640307167
RAMAC	protein_coding	ENSG00000169612	-0,040985063	0,53965625	0,640307167
AL603750.1	TEC	ENSG00000280099	0,105584407	0,539720995	0,640346943
MOCS1	protein_coding	ENSG00000124615	-0,161836402	0,539944768	0,640575381
AC008507.2	lincRNA	ENSG00000267006	0,144039132	0,540047374	0,640660052
MYO1F	protein_coding	ENSG00000142347	0,147370314	0,540192347	0,64079497
AL359853.2	lincRNA	ENSG00000231966	0,136039297	0,540611823	0,641255479
LSM12	protein_coding	ENSG00000161654	-0,046221973	0,540671859	0,641289604
AP001351.1	antisense	ENSG00000248027	0,145010444	0,540890975	0,641512398
RPS3AP47	processed_pseudogene	ENSG00000205871	0,146894937	0,541013362	0,641620449
JARID2	protein_coding	ENSG00000008083	-0,04114067	0,541046419	0,641622553
AC009054.2	sense_intronic	ENSG00000261783	-0,158538241	0,541097257	0,641645742
AL034346.1	antisense	ENSG00000261730	-0,150023216	0,541179845	0,641706574
ARFIP1	protein_coding	ENSG00000164144	0,040451198	0,541291382	0,641801725
ACTG1P3	processed_pseudogene	ENSG00000215388	0,161110082	0,541438437	0,641938975
FZD5	protein_coding	ENSG00000163251	0,052309261	0,541472486	0,641942237
ADH5P4	processed_pseudogene	ENSG00000233859	0,158697399	0,541695157	0,642169104
PCDHB3	protein_coding	ENSG00000113205	-0,144174309	0,541940735	0,6424231
AC073410.1	processed_pseudogene	ENSG00000236047	0,150540622	0,542032008	0,642494162
GLRX3	protein_coding	ENSG00000108010	-0,036240504	0,542179662	0,642632042
RF00573	snoRNA	ENSG00000252427	-0,141646358	0,542247256	0,64267502
EIF3CL	protein_coding	ENSG00000205609	0,150008149	0,542420425	0,642843113
ZNF518A	protein_coding	ENSG00000177853	0,039177863	0,542689101	0,643124369

AC026333.4	lincRNA	ENSG00000274191	-0,140611875	0,54307066	0,64353936
KPTN	protein_coding	ENSG00000118162	-0,056673637	0,543166997	0,643616333
C16orf95	protein_coding	ENSG00000260456	-0,113671299	0,543395815	0,64385027
AC015712.1	lincRNA	ENSG00000232386	-0,159438412	0,543694376	0,644092396
FHOD3	protein_coding	ENSG00000134775	0,048109438	0,543682649	0,644092396
AC093535.2	TEC	ENSG00000279118	0,143704303	0,543637664	0,644092396
PLSCR3	protein_coding	ENSG00000187838	-0,142877475	0,543844864	0,644233463
ADI1P1	processed_pseudogene	ENSG00000235102	0,142603312	0,543881591	0,644239759
SCAT2	antisense	ENSG00000257596	0,154664993	0,544083445	0,644441642
AC068987.5	protein_coding	ENSG00000284730	0,143693506	0,544484444	0,644879364
AC091057.1	processed_transcript	ENSG00000187951	0,049329143	0,544624084	0,645007504
ZNF579	protein_coding	ENSG00000218891	-0,065736535	0,544658888	0,645011478
USP24	protein_coding	ENSG00000162402	0,040660991	0,544886317	0,645243554
LINC01429	lincRNA	ENSG00000227964	0,159385964	0,545140903	0,6454442
TMCC2	protein_coding	ENSG00000133069	0,05640563	0,545150164	0,6454442
SEPT7P2	transcribed_unprocessed	ENSG00000214765	-0,052920417	0,545092237	0,6454442
ZKSCAN2-DT	lincRNA	ENSG00000274925	-0,148328099	0,545852105	0,646237975
ZNF652	protein_coding	ENSG00000198740	-0,038082982	0,546025218	0,646405612
AC005410.2	antisense	ENSG00000266368	-0,149940725	0,546344353	0,646653667
AC091516.1	processed_pseudogene	ENSG00000257752	-0,134294997	0,546347737	0,646653667
HADHA	protein_coding	ENSG00000084754	0,023704106	0,546362539	0,646653667
MBD2	protein_coding	ENSG00000134046	-0,035346879	0,546392396	0,646653667
AC011472.4	antisense	ENSG00000273733	0,154204987	0,546563088	0,646814183
UPK1A	protein_coding	ENSG00000105668	0,147389343	0,546622634	0,646814183
CDC42EP2	protein_coding	ENSG00000149798	0,045634806	0,546622244	0,646814183
AC096772.1	antisense	ENSG00000272851	0,135422667	0,546869981	0,647044177
RAMACL	protein_coding	ENSG00000235272	0,122799551	0,546911645	0,647044177
KRTCAP2	protein_coding	ENSG00000163463	-0,136002492	0,546897466	0,647044177
AC023347.2	processed_pseudogene	ENSG00000236655	0,148646124	0,547068376	0,6470733
ERMAP	protein_coding	ENSG00000164010	0,053203798	0,547066193	0,6470733
GOLIM4	protein_coding	ENSG00000173905	0,041065285	0,54698381	0,6470733
ANKRD61	protein_coding	ENSG00000157999	-0,150337002	0,547094008	0,6470733
AC092828.1	sense_intronic	ENSG00000253284	0,158697734	0,547133917	0,647083187
LRIG2	protein_coding	ENSG00000198799	0,042154963	0,547350686	0,647302229
CU633906.2	lincRNA	ENSG00000278903	0,145633924	0,547527988	0,64736413
AL031118.1	lincRNA	ENSG00000271755	0,122747386	0,547521558	0,64736413
CLN3	protein_coding	ENSG00000188603	0,158700038	0,547513578	0,64736413
MTFMT	protein_coding	ENSG00000103707	0,038885997	0,547529282	0,64736413
CR786580.1	lincRNA	ENSG00000225411	0,138903034	0,547568503	0,647373184
TMEM164	protein_coding	ENSG00000157600	-0,034847166	0,547647214	0,647393266

SEMA6B	protein_coding	ENSG00000167680	-0,04974883	0,547648618	0,647393266
AC092755.1	sense_overlapping	ENSG00000227161	-0,139882861	0,548162796	0,647963745
ZNF197-AS1	antisense	ENSG00000233509	0,157934724	0,548202154	0,647972924
AC068700.1	sense_overlapping	ENSG00000260398	-0,137981163	0,548311733	0,648065098
FRAT2	protein_coding	ENSG00000181274	-0,059694672	0,548367141	0,648093238
RF00019	misc_RNA	ENSG00000252874	-0,145165072	0,54887443	0,648580662
AC008026.1	processed_pseudogene	ENSG00000239246	-0,153547377	0,548851735	0,648580662
FMC1	protein_coding	ENSG00000164898	0,14315031	0,548836304	0,648580662
CPNE1	protein_coding	ENSG00000214078	0,039535506	0,548973643	0,648660526
CYP46A1	protein_coding	ENSG00000036530	0,154341555	0,549150865	0,64883255
BX088651.4	lincRNA	ENSG00000237357	0,14985714	0,54940716	0,649097976
SPATA3-AS1	lincRNA	ENSG00000238062	-0,151789311	0,549592425	0,649279456
HNRNPUP1	processed_pseudogene	ENSG00000259051	-0,154481409	0,549725182	0,649398887
AC011445.2	lincRNA	ENSG00000269246	-0,153260819	0,550101123	0,649795626
AC022336.3	TEC	ENSG00000280042	0,147386102	0,55012439	0,649795626
CD44-AS1	antisense	ENSG00000255443	0,132810046	0,55018692	0,649832061
C6orf62	protein_coding	ENSG00000112308	0,025175308	0,550328515	0,64996187
LINC00853	antisense	ENSG00000224805	-0,147440581	0,550411512	0,650022463
COG4	protein_coding	ENSG00000103051	-0,027473853	0,550570414	0,650172685
ASAP1-IT2	sense_intronic	ENSG00000280543	-0,154403241	0,550860364	0,650477636
RHCE	protein_coding	ENSG00000188672	0,157350614	0,550908146	0,650479202
ANKRD20A21P	transcribed_unprocesse	ENSG00000277112	0,150940709	0,55092512	0,650479202
AL356275.1	lincRNA	ENSG00000284237	0,122057044	0,551497381	0,651057414
ASIC3	protein_coding	ENSG00000213199	0,157401396	0,551530309	0,651057414
ACTN3	protein_coding	ENSG00000248746	0,141622835	0,551553043	0,651057414
SLC37A3	protein_coding	ENSG00000157800	-0,034263081	0,551573556	0,651057414
PSMD9	protein_coding	ENSG00000110801	-0,053892193	0,551497642	0,651057414
HOXC10	protein_coding	ENSG00000180818	-0,050216628	0,551713856	0,651185543
RASGRP1	protein_coding	ENSG00000172575	0,150611974	0,551785707	0,651232872
SEPT9	protein_coding	ENSG00000184640	-0,036242398	0,551881013	0,651307876
CXXC5	protein_coding	ENSG00000171604	0,082932147	0,552020013	0,651434435
AC048341.2	lincRNA	ENSG00000275180	0,145049952	0,552132701	0,651467904
TTI2	protein_coding	ENSG00000129696	0,033289473	0,552118847	0,651467904
AMOTL1	protein_coding	ENSG00000166025	-0,036143902	0,552161797	0,651467904
ST6GALNAC5	protein_coding	ENSG00000117069	-0,155394458	0,552175429	0,651467904
C2CD4C	protein_coding	ENSG00000183186	-0,109757779	0,552385095	0,651677785
HIPK1	protein_coding	ENSG00000163349	0,048672545	0,552643384	0,651936459
KCNRG	protein_coding	ENSG00000198553	-0,154370162	0,552667929	0,651936459
HLX	protein_coding	ENSG00000136630	-0,067396174	0,552700637	0,651937547
XRCC6P2	processed_pseudogene	ENSG00000234825	-0,131441484	0,55289084	0,652124397

AC093388.1	antisense	ENSG00000272979	0,081516949	0,553263759	0,652490513
EIF4A3	protein_coding	ENSG00000141543	-0,027936291	0,553264871	0,652490513
ARID1A	protein_coding	ENSG00000117713	-0,038571943	0,55340538	0,652618696
AL355802.1	processed_pseudogene	ENSG00000219470	-0,151564772	0,553562909	0,652766933
RPL15P3	processed_pseudogene	ENSG00000212802	-0,1024939	0,554142943	0,653413348
AC108704.2	TEC	ENSG00000279500	-0,155262408	0,554360007	0,653631719
PDZK1	protein_coding	ENSG00000174827	0,155511423	0,554399744	0,653640995
PYGM	protein_coding	ENSG00000068976	0,14819526	0,55452347	0,653749289
AC104619.3	processed_pseudogene	ENSG00000250144	0,139920722	0,554766684	0,65399843
AC025265.1	antisense	ENSG00000257681	0,142815801	0,554905234	0,654124166
LCE3D	protein_coding	ENSG00000163202	-0,155020101	0,555039883	0,654245287
RPL34P27	processed_pseudogene	ENSG00000232858	0,138458807	0,555119658	0,654301718
LRTOMT	protein_coding	ENSG00000184154	0,055155214	0,555194659	0,654323824
DHRSX-IT1	sense_intronic	ENSG00000223571	-0,155113939	0,555202219	0,654323824
RTP3	protein_coding	ENSG00000163825	0,144566471	0,555395428	0,654508326
TAS2R20	protein_coding	ENSG00000255837	-0,152309609	0,555422594	0,654508326
HIGD1AP1	processed_pseudogene	ENSG00000258016	-0,127433863	0,555558582	0,654614625
EFCAB13	protein_coding	ENSG00000178852	0,120137532	0,555576635	0,654614625
AL050331.1	processed_pseudogene	ENSG00000233558	0,155373233	0,556050028	0,655097137
RNF207	protein_coding	ENSG00000158286	-0,087037099	0,556026448	0,655097137
AC104581.4	TEC	ENSG00000280046	0,137864177	0,556201032	0,655237401
LINC01108	lincRNA	ENSG00000226673	0,154778213	0,556559478	0,655584361
FAUP1	processed_pseudogene	ENSG00000235297	-0,154316504	0,55654982	0,655584361
GAPDHP72	transcribed_processed_	ENSG00000216624	0,118812749	0,55671721	0,655732498
AC011731.1	antisense	ENSG00000266495	0,13860885	0,556973706	0,655996939
AC091185.1	lincRNA	ENSG00000253476	-0,146268244	0,557010493	0,656002596
AL139384.2	lincRNA	ENSG00000277159	0,12459864	0,557090167	0,656058758
TCTEX1D4	protein_coding	ENSG00000188396	0,111448075	0,557185264	0,656133075
NUDCD1	protein_coding	ENSG00000120526	0,03349339	0,557392386	0,656339295
HEYL	protein_coding	ENSG00000163909	-0,091992942	0,55797832	0,656991523
AC008619.1	antisense	ENSG00000253858	0,142414741	0,558159862	0,65712983
SETD7	protein_coding	ENSG00000145391	-0,030836283	0,558159809	0,65712983
INTS6P1	processed_pseudogene	ENSG00000250492	0,154726503	0,558330343	0,657276206
SLC39A9	protein_coding	ENSG00000029364	0,025935209	0,558348286	0,657276206
PCDHGA10	protein_coding	ENSG00000253846	0,141071462	0,55853223	0,657455006
SPPL2A	protein_coding	ENSG00000138600	0,030339189	0,558712762	0,65762977
ZBTB40-IT1	sense_intronic	ENSG00000237200	0,146462756	0,558756458	0,657643461
AC068491.3	lincRNA	ENSG00000270190	-0,14813165	0,558872546	0,65774235
ID2-AS1	antisense	ENSG00000235092	0,146853872	0,558971857	0,657821482
AL359541.1	antisense	ENSG00000226889	0,139202336	0,559105657	0,657941191

RPS4XP1	processed_pseudogene	ENSG00000214203	0,142683499	0,559167879	0,657976661
AC073072.1	antisense	ENSG00000179428	-0,064705628	0,559247683	0,657995065
HNRNPD	protein_coding	ENSG00000138668	0,030633062	0,559232526	0,657995065
SRGAP1	protein_coding	ENSG00000196935	0,037905432	0,559325891	0,658049333
PRAF2	protein_coding	ENSG00000243279	0,099400205	0,55956835	0,658296825
ELOVL5	protein_coding	ENSG00000012660	-0,026699549	0,559640958	0,658329909
GORASP1	protein_coding	ENSG00000114745	-0,034814003	0,559660668	0,658329909
AC005034.5	sense_intronic	ENSG00000271452	0,154075358	0,559720588	0,658362635
LINC01278	processed_transcript	ENSG00000235437	0,058324152	0,559821856	0,658443988
SPEG	protein_coding	ENSG00000072195	-0,073530059	0,559919938	0,658521586
AC005670.1	lincRNA	ENSG00000261886	0,152163503	0,560594669	0,659277332
MSLN	protein_coding	ENSG00000102854	-0,055572951	0,560708195	0,659373034
GDPGP1	protein_coding	ENSG00000183208	0,103073224	0,560775707	0,659414617
AL118505.1	lincRNA	ENSG00000278192	-0,137022746	0,560972804	0,659608565
PIGF	protein_coding	ENSG00000151665	-0,034296648	0,561083268	0,659700632
AC019186.1	lincRNA	ENSG00000234584	0,128786863	0,561304644	0,65988526
CHMP1B	protein_coding	ENSG00000255112	-0,035058048	0,561278703	0,65988526
CD44	protein_coding	ENSG00000026508	-0,024018446	0,56150361	0,660081334
AC068831.1	antisense	ENSG00000258384	-0,150424679	0,561607054	0,660127267
OR10Y1P	unprocessed_pseudogene	ENSG00000254403	-0,150813491	0,561575161	0,660127267
HSF2	protein_coding	ENSG00000025156	0,042999428	0,561745392	0,660252034
SEPSECS-AS1	antisense	ENSG00000281501	-0,120737474	0,561871698	0,660324807
FBXO24	protein_coding	ENSG00000106336	-0,144921771	0,561858798	0,660324807
AP006621.1	antisense	ENSG00000255108	0,14136929	0,56195553	0,660385488
AL627171.1	lincRNA	ENSG00000278002	0,131929119	0,562197016	0,660570764
AGTRAP	protein_coding	ENSG00000177674	-0,042680178	0,562209812	0,660570764
LRRC70	protein_coding	ENSG00000186105	-0,128565325	0,562194539	0,660570764
TRPM7	protein_coding	ENSG00000092439	-0,026772577	0,562299353	0,660638124
MC1R	protein_coding	ENSG00000258839	0,068212063	0,562539055	0,660844036
AC106795.3	lincRNA	ENSG00000250101	-0,132184103	0,562692302	0,660948348
NOTUM	protein_coding	ENSG00000185269	0,146971688	0,562675031	0,660948348
AC135507.1	antisense	ENSG00000272182	-0,140981453	0,562952831	0,661216502
CREG2	protein_coding	ENSG00000175874	0,152872851	0,563016398	0,661253296
C12orf42	protein_coding	ENSG00000179088	0,140748954	0,563138653	0,661323213
SECISBP2	protein_coding	ENSG00000187742	-0,034916443	0,563140415	0,661323213
RFTN1	protein_coding	ENSG00000131378	0,026172138	0,563179505	0,661331252
AC127502.2	sense_intronic	ENSG00000270055	-0,074783973	0,563273609	0,661403889
AC013565.3	antisense	ENSG00000261407	-0,151955844	0,563502854	0,661635192
AC104187.1	antisense	ENSG00000271937	-0,138185908	0,563828139	0,661953327
DHDDS	protein_coding	ENSG00000117682	0,026236105	0,563870628	0,661953327

SCAMP5	protein_coding	ENSG00000198794	-0,098075527	0,563864823	0,661953327
UFSP1	protein_coding	ENSG00000176125	-0,131049963	0,564080173	0,662161422
AC062015.1	lincRNA	ENSG00000235070	0,150734386	0,564144318	0,662198818
LRRC37A3	protein_coding	ENSG00000176809	-0,052250238	0,564629138	0,662729977
AC118282.1	processed_pseudogene	ENSG00000237961	-0,13985603	0,564810974	0,662867537
REST	protein_coding	ENSG00000084093	0,03388695	0,564800009	0,662867537
TLE2	protein_coding	ENSG00000065717	-0,082783138	0,565031569	0,663088486
ACOT2	protein_coding	ENSG00000119673	0,041978712	0,565092393	0,663121924
SLC12A9-AS1	antisense	ENSG00000236305	-0,148170714	0,565237169	0,663253866
CFAP73	protein_coding	ENSG00000186710	-0,149913068	0,565357363	0,663356951
RF00019	misc_RNA	ENSG00000201988	-0,109380158	0,565434814	0,663409876
AL359715.1	lincRNA	ENSG00000233967	0,131580662	0,565492132	0,663429022
GOLT1B	protein_coding	ENSG00000111711	-0,048601372	0,565515826	0,663429022
ABCB7	protein_coding	ENSG00000131269	-0,034310448	0,565654065	0,663553241
AC090912.1	antisense	ENSG00000265943	0,151410479	0,565970335	0,663810349
AGGF1P2	processed_pseudogene	ENSG00000233435	-0,150398565	0,565919234	0,663810349
NSMCE2	protein_coding	ENSG00000156831	-0,047492806	0,565951565	0,663810349
GUCY2D	protein_coding	ENSG00000132518	0,139499278	0,56628616	0,664142792
EIF5AP4	processed_pseudogene	ENSG00000234743	0,144026442	0,566600917	0,664473944
PCOLCE	protein_coding	ENSG00000106333	-0,089599404	0,566689179	0,664539454
BTF3P6	processed_pseudogene	ENSG00000233956	-0,139141181	0,56683989	0,664678186
SBNO1	protein_coding	ENSG00000139697	0,029499025	0,567265571	0,665119684
AC110285.7	TEC	ENSG00000279692	-0,145401083	0,56728126	0,665119684
RFX3-AS1	lincRNA	ENSG00000232104	0,139907557	0,567329761	0,665129416
CTTNBP2	protein_coding	ENSG00000077063	0,136124226	0,567354419	0,665129416
ZCCHC18	protein_coding	ENSG00000166707	0,129176034	0,567433622	0,665184247
SCARF2	protein_coding	ENSG00000244486	-0,058815928	0,567532888	0,66526259
SNORA58	snoRNA	ENSG00000249020	0,141734265	0,567673412	0,665389283
CCDC22	protein_coding	ENSG00000101997	0,044755813	0,567962458	0,665690041
AC018738.1	processed_pseudogene	ENSG00000237039	-0,092139315	0,568081402	0,665715321
CELF5	protein_coding	ENSG00000161082	0,141785954	0,568033709	0,665715321
AC018809.1	sense_intronic	ENSG00000269894	0,128223408	0,568078639	0,665715321
DHX38	protein_coding	ENSG00000140829	0,046621148	0,568205048	0,665822175
LRRC2-AS1	antisense	ENSG00000268324	0,149380325	0,568317718	0,665878115
TOLLIP-AS1	antisense	ENSG00000255153	-0,137131623	0,56831129	0,665878115
SCARNA3	scaRNA	ENSG00000252906	-0,149408256	0,568448985	0,66599387
SH3GL1P1	transcribed_processed_	ENSG00000266777	-0,147113959	0,568767239	0,666328672
AL161669.3	lincRNA	ENSG00000278071	0,147776989	0,568819823	0,666347555
RPS12	protein_coding	ENSG00000112306	-0,045154389	0,568848335	0,666347555
EIF4EP2	processed_pseudogene	ENSG00000229944	-0,128657542	0,568921193	0,666394841

ALG1	protein_coding	ENSG00000033011	-0,041148742	0,569006354	0,666456531
AL359265.3	antisense	ENSG00000236021	0,149972605	0,569048514	0,666467852
SARDH	protein_coding	ENSG00000123453	0,132747578	0,569265067	0,666607281
PCSK4	protein_coding	ENSG00000115257	0,105648097	0,569256469	0,666607281
RAP2A	protein_coding	ENSG00000125249	-0,03584182	0,569222321	0,666607281
CASC1	protein_coding	ENSG00000118307	0,147577973	0,569446034	0,666781124
METRN	protein_coding	ENSG00000103260	-0,053120895	0,569617735	0,666944097
GGN	protein_coding	ENSG00000179168	0,135955739	0,569683271	0,666982754
SNORD89	snoRNA	ENSG00000212283	0,148453415	0,56981824	0,667102695
ECHDC2	protein_coding	ENSG00000121310	-0,042306629	0,56985314	0,667105473
ST13P18	processed_pseudogene	ENSG00000234322	-0,144000206	0,56989247	0,667113438
CSNK1G3	protein_coding	ENSG00000151292	-0,033155787	0,569974754	0,667171681
AC097468.3	lincRNA	ENSG00000272644	-0,134186841	0,57002761	0,667195474
RN7SL81P	misc_RNA	ENSG00000244218	-0,145214748	0,570119051	0,667264423
AC068522.1	processed_pseudogene	ENSG00000242970	-0,136150052	0,570277429	0,667411702
FAM133DP	processed_pseudogene	ENSG00000230562	-0,139664713	0,57053838	0,667679002
TBC1D8-AS1	lincRNA	ENSG00000272902	0,115869335	0,570581442	0,667691298
AL356299.2	sense_intronic	ENSG00000272945	-0,141724252	0,570766765	0,667870056
PLEKHA1	protein_coding	ENSG00000107679	-0,037353152	0,570884747	0,667970002
AC006273.1	lincRNA	ENSG00000272473	0,149431729	0,571082855	0,668163682
AL355032.1	processed_pseudogene	ENSG00000241494	-0,146613265	0,57118815	0,66821064
TIGD3	protein_coding	ENSG00000173825	-0,13566869	0,571162107	0,66821064
AC106820.5	lincRNA	ENSG00000260874	-0,142182938	0,571522453	0,668525465
SDHAP1	transcribed_unprocessed	ENSG00000185485	0,055096301	0,571500632	0,668525465
HSPA8P5	processed_pseudogene	ENSG00000256356	-0,147681561	0,571738096	0,66873957
AD000813.1	TEC	ENSG00000280023	-0,147247087	0,571849291	0,668831487
ABHD16A	protein_coding	ENSG00000204427	0,127107003	0,571926484	0,668883628
AFAP1	protein_coding	ENSG00000196526	0,032843473	0,57214607	0,669102287
AC011595.2	lincRNA	ENSG00000257890	0,141732484	0,572277963	0,669218371
AL133342.1	lincRNA	ENSG00000278231	-0,142182928	0,572512709	0,669416547
HBS1L	protein_coding	ENSG00000112339	0,027917262	0,572492339	0,669416547
AP004609.3	lincRNA	ENSG00000278376	0,132307213	0,572623324	0,669507718
AC020978.7	sense_overlapping	ENSG00000263276	0,126595485	0,572812255	0,669690438
DKKL1	protein_coding	ENSG00000104901	-0,146951924	0,572926861	0,669786247
MSN	protein_coding	ENSG00000147065	0,020402366	0,57312513	0,669950858
HIKESHI	protein_coding	ENSG00000149196	-0,03516616	0,573132996	0,669950858
TMEM266	protein_coding	ENSG00000169758	0,147957594	0,573211893	0,669984166
TTC16	protein_coding	ENSG00000167094	-0,124271334	0,573226823	0,669984166
CCDC171	protein_coding	ENSG00000164989	0,064914184	0,573315258	0,670011165
PFKFB1	protein_coding	ENSG00000158571	-0,136645428	0,573287806	0,670011165

AC010616.1	protein_coding	ENSG00000268041	-0,135310375	0,573560348	0,670259401
LAMP3	protein_coding	ENSG00000078081	0,141152213	0,573881311	0,670558064
FAM13A	protein_coding	ENSG00000138640	0,088059424	0,573858992	0,670558064
AC044810.2	sense_overlapping	ENSG00000254951	0,121893261	0,574141576	0,670823955
AC055813.1	processed_pseudogene	ENSG00000267253	-0,145932007	0,574547272	0,671259729
NDUFB9	protein_coding	ENSG00000147684	-0,045321764	0,574755901	0,671450725
HNRNPLP2	unprocessed_pseudoge	ENSG00000259917	-0,130451114	0,574776225	0,671450725
PLS3-AS1	antisense	ENSG00000271826	0,097205294	0,574964912	0,671632894
AC006042.3	processed_pseudogene	ENSG00000233264	0,147907463	0,575097728	0,671749781
ARID3C	protein_coding	ENSG00000205143	0,138477128	0,575322613	0,671974191
OR7M1P	unprocessed_pseudoge	ENSG00000273336	-0,137241047	0,575361603	0,671981464
AC015813.3	lincRNA	ENSG00000266290	0,113497327	0,575439518	0,672031974
PTMAP2	processed_pseudogene	ENSG00000197744	-0,102684577	0,575470383	0,672031974
AC078881.1	antisense	ENSG00000250131	0,145717015	0,576012031	0,672626211
SRPRB	protein_coding	ENSG00000144867	0,031930251	0,576294789	0,672918083
ZNF766	protein_coding	ENSG00000196214	0,035765819	0,576381978	0,672981577
PSORS1C1	protein_coding	ENSG00000204540	0,047702564	0,576524629	0,673109816
STK25	protein_coding	ENSG00000115694	-0,03074169	0,576603355	0,673163411
AC115223.1	processed_pseudogene	ENSG00000243199	0,106926878	0,577099429	0,673653508
LRRC34	protein_coding	ENSG00000171757	-0,085337071	0,57710539	0,673653508
AC020763.2	TEC	ENSG00000279122	-0,136430009	0,577121686	0,673653508
TAS2R30	protein_coding	ENSG00000256188	-0,141499111	0,577189676	0,673694529
LAMA5	protein_coding	ENSG00000130702	-0,062805102	0,577244583	0,673720275
KMT5A	protein_coding	ENSG00000183955	-0,027155834	0,57752859	0,674013393
STXBP5	protein_coding	ENSG00000164506	0,032398933	0,577678386	0,674149854
PSMD8	protein_coding	ENSG00000099341	0,038684394	0,578206582	0,674727867
RBPMS-AS1	antisense	ENSG00000254109	-0,145877376	0,57848537	0,675014789
SAFB	protein_coding	ENSG00000160633	-0,03513626	0,578531259	0,675029931
AC023043.1	antisense	ENSG00000260552	0,145897609	0,578567985	0,67503438
OCLM	protein_coding	ENSG00000262180	-0,129604028	0,578630044	0,675068385
AC144522.1	TEC	ENSG00000279925	-0,133815108	0,579552412	0,676106022
AC091180.3	antisense	ENSG00000250186	0,126489073	0,5801035	0,676677574
PLA2G16	protein_coding	ENSG00000176485	0,038911952	0,580141319	0,676677574
HEY2	protein_coding	ENSG00000135547	-0,134228683	0,580121065	0,676677574
AC106886.4	TEC	ENSG00000280211	0,13524826	0,580859026	0,677437657
AC079140.2	processed_pseudogene	ENSG00000250321	-0,120674244	0,580984624	0,67754561
AC067931.1	TEC	ENSG00000279766	0,145197937	0,581361969	0,677947122
LINC00839	lincRNA	ENSG00000185904	0,03475727	0,581592292	0,678177152
ATG14	protein_coding	ENSG00000126775	-0,033050704	0,581839119	0,678426397
GLRX3P2	processed_pseudogene	ENSG00000216657	-0,14327358	0,581939094	0,678465826

PIGA	protein_coding	ENSG00000165195	0,041285572	0,581909441	0,678465826
HDHD5-AS1	antisense	ENSG00000185837	0,134752983	0,582311652	0,678848525
OSR2	protein_coding	ENSG00000164920	0,082458474	0,582333542	0,678848525
AC008507.1	processed_pseudogene	ENSG00000266910	-0,138896721	0,582382364	0,678866853
AC009118.3	antisense	ENSG00000276259	-0,144628014	0,582507786	0,678974465
SYCP3	protein_coding	ENSG00000139351	0,119241811	0,583276081	0,679831359
ZNF519	protein_coding	ENSG00000175322	-0,103187592	0,583390082	0,679925593
AL596325.1	antisense	ENSG00000259788	-0,136573625	0,583761978	0,680320369
C2orf81	protein_coding	ENSG00000284308	0,135471373	0,583911509	0,680417309
ATF7	protein_coding	ENSG00000170653	0,057730692	0,583878941	0,680417309
LINC02303	lincRNA	ENSG00000258616	0,127256078	0,584021159	0,680506419
AC233728.1	antisense	ENSG00000233250	0,143529469	0,584111524	0,680573047
SFR1	protein_coding	ENSG00000156384	-0,048621803	0,584343343	0,680804475
GNAZ	protein_coding	ENSG00000128266	-0,0666194	0,584489646	0,680936248
LINC01206	lincRNA	ENSG00000242512	0,126478976	0,584741031	0,68119042
KHDC1	protein_coding	ENSG00000135314	0,081522025	0,584910023	0,681321341
CCZ1B	protein_coding	ENSG00000146574	0,039847388	0,584919853	0,681321341
HS2ST1	protein_coding	ENSG00000153936	0,033500559	0,584959989	0,681329398
MAP6D1	protein_coding	ENSG00000180834	-0,142614618	0,585114955	0,681471194
AC008649.2	antisense	ENSG00000267375	0,142058061	0,585261728	0,681564731
TATDN1	protein_coding	ENSG00000147687	-0,036676497	0,585230854	0,681564731
NACA2	protein_coding	ENSG00000253506	0,142222808	0,58558587	0,681903491
MAD2L2	protein_coding	ENSG00000116670	-0,031640823	0,585934392	0,682270602
FBXW9	protein_coding	ENSG00000132004	0,071528106	0,586021534	0,682333333
DCLK2	protein_coding	ENSG00000170390	-0,048178516	0,586181243	0,682480546
VILL	protein_coding	ENSG00000136059	0,061300549	0,586219335	0,682486153
AL645608.7	lincRNA	ENSG00000272512	0,142034168	0,586282803	0,682521301
HDAC2	protein_coding	ENSG00000196591	-0,023819797	0,58649316	0,682727437
AC015689.1	lincRNA	ENSG00000254480	0,114893369	0,586621138	0,682837659
AL021068.1	lincRNA	ENSG00000213062	0,120853227	0,58668506	0,682873309
SPPL2B	protein_coding	ENSG00000005206	0,063848771	0,586806898	0,682937608
TMED4	protein_coding	ENSG00000158604	0,021039845	0,5867874	0,682937608
TAPT1-AS1	antisense	ENSG00000263327	-0,134228297	0,586846116	0,682944498
AL022393.1	TEC	ENSG00000280107	0,13747931	0,586980106	0,683061672
TACC2	protein_coding	ENSG00000138162	-0,033691277	0,587120179	0,683185911
AL133467.4	lincRNA	ENSG00000270038	0,141170102	0,58716116	0,683194837
AC133965.1	processed_pseudogene	ENSG00000225933	-0,117383698	0,587254371	0,683226414
HMGCR	protein_coding	ENSG00000113161	-0,02571115	0,587254922	0,683226414
ZNF229	protein_coding	ENSG00000278318	-0,034035043	0,587346399	0,683255326
UCP2	protein_coding	ENSG00000175567	-0,142696245	0,58733212	0,683255326

BNC2-AS1	antisense	ENSG00000234779	-0,140618296	0,587452207	0,683339653
CDC14B	protein_coding	ENSG00000081377	-0,03576514	0,587505232	0,683362576
ATP1A3	protein_coding	ENSG00000105409	-0,06042905	0,587629339	0,683468171
ATP6V1F	protein_coding	ENSG00000128524	-0,038403795	0,587708467	0,683521444
AC104564.1	antisense	ENSG00000263370	-0,141094622	0,5878037	0,683593439
PTCD1	protein_coding	ENSG00000106246	-0,068975095	0,587886926	0,683651463
PROCA1	protein_coding	ENSG00000167525	0,135223128	0,587972873	0,683712645
YY2	protein_coding	ENSG00000230797	-0,116292261	0,588325774	0,684084223
SCARNA13	scaRNA	ENSG00000252481	-0,039762618	0,588511111	0,684260934
PURB	protein_coding	ENSG00000146676	0,036382261	0,588627411	0,684357361
AC020663.3	sense_intronic	ENSG00000275056	0,142093213	0,589000826	0,684752689
AC244093.1	processed_pseudogene	ENSG00000274308	-0,126174698	0,589132153	0,684866545
C8orf76	protein_coding	ENSG00000189376	-0,069211337	0,589173403	0,68487568
RPL7AP30	processed_pseudogene	ENSG00000241741	-0,120567911	0,589264489	0,684912487
C17orf107	protein_coding	ENSG00000205710	0,132521268	0,589278459	0,684912487
AC107918.4	transcribed_unprocesse	ENSG00000284610	-0,133035955	0,589305249	0,684912487
AC009302.1	processed_pseudogene	ENSG00000233868	0,140494083	0,589439889	0,684991338
AKT1	protein_coding	ENSG00000142208	-0,028810879	0,589428444	0,684991338
LINC00607	lincRNA	ENSG00000235770	0,127830566	0,589743072	0,685286641
ATP6V1B1	protein_coding	ENSG00000116039	0,140694653	0,589760824	0,685286641
AC146507.2	processed_pseudogene	ENSG00000240729	-0,129412336	0,589913363	0,685425055
ADAM8	protein_coding	ENSG00000151651	-0,094919208	0,589998591	0,685485249
RF00404	snoRNA	ENSG00000212371	-0,126198651	0,590127549	0,685596241
SEPT10P1	processed_pseudogene	ENSG00000253541	0,132850074	0,590207902	0,685650755
AC005476.2	antisense	ENSG00000259146	-0,140455261	0,590318242	0,685678449
FAM13A-AS1	antisense	ENSG00000248019	-0,141427103	0,590332036	0,685678449
ASB16	protein_coding	ENSG00000161664	0,122232619	0,590265424	0,685678449
AP000873.4	sense_overlapping	ENSG00000254676	-0,13617881	0,590423868	0,685746279
AC022034.3	lincRNA	ENSG00000254204	-0,127135663	0,590580844	0,685850921
AC016957.2	lincRNA	ENSG00000276136	-0,135117376	0,590563867	0,685850921
FAHD2B	protein_coding	ENSG00000144199	0,05367736	0,590891663	0,686173027
AC107884.1	antisense	ENSG00000251364	0,141550936	0,591085838	0,686359652
AC092535.4	antisense	ENSG00000273179	0,126902048	0,591333977	0,686608915
AC012555.1	lincRNA	ENSG00000257327	0,1188681	0,591425287	0,686676062
LINC02175	lincRNA	ENSG00000262155	0,135187955	0,591612313	0,686854327
AC245884.8	lincRNA	ENSG00000267838	0,125563427	0,591699248	0,686916374
ATP5F1EP2	processed_pseudogene	ENSG00000180389	0,135488026	0,591945076	0,687162865
KCNIP2-AS1	antisense	ENSG00000226009	0,140768033	0,592135626	0,687345164
AC245033.2	lincRNA	ENSG00000278013	-0,12339264	0,592520442	0,687677402
THAP1	protein_coding	ENSG00000131931	-0,041159245	0,592522431	0,687677402

NDUFB5	protein_coding	ENSG00000136521	-0,042224942	0,592473312	0,687677402
MAMDC2	protein_coding	ENSG00000165072	0,046740781	0,592642041	0,687777302
ZHX1-C8orf76	protein_coding	ENSG00000259305	0,138698408	0,592738844	0,687850723
AL606763.1	lincRNA	ENSG00000281091	-0,101070382	0,59278278	0,68786279
BX005019.1	lincRNA	ENSG00000259946	0,130230319	0,592928892	0,687993413
AC004816.2	lincRNA	ENSG00000275630	-0,139670569	0,593085948	0,688136719
Z97832.2	lincRNA	ENSG00000272374	0,111228949	0,593430018	0,688496983
GUK1	protein_coding	ENSG00000143774	0,036822593	0,593828567	0,688920409
AL031432.4	bidirectional_promoter_	ENSG00000284602	-0,12765923	0,593909831	0,688973136
EEF1D	protein_coding	ENSG00000104529	-0,021643803	0,593941201	0,688973136
AL513174.1	unprocessed_pseudoge	ENSG00000234382	0,122439658	0,594129615	0,689152721
AF201337.1	processed_pseudogene	ENSG00000254332	-0,134039667	0,594255755	0,689260055
TBCAP1	processed_pseudogene	ENSG00000226781	0,140260173	0,594496557	0,689461373
PRICKLE2	protein_coding	ENSG00000163637	0,130237759	0,594490014	0,689461373
NHLRC1	protein_coding	ENSG00000187566	-0,068795055	0,594782687	0,689754207
RN7SL368P	misc_RNA	ENSG00000239948	-0,134462673	0,594924443	0,689879591
AC092115.1	processed_pseudogene	ENSG00000260290	-0,124843845	0,595273092	0,690244862
RN7SL674P	misc_RNA	ENSG00000239899	-0,114814243	0,595435708	0,690381196
AC144530.1	processed_pseudogene	ENSG00000234742	0,13320238	0,595789159	0,690700036
ALDH7A1P1	processed_pseudogene	ENSG00000251400	-0,135937034	0,595800343	0,690700036
AP000781.1	antisense	ENSG00000254662	0,137773023	0,59596096	0,690847188
NDUFB1	protein_coding	ENSG00000183648	-0,040382733	0,59601597	0,690868792
ZC3H12B	protein_coding	ENSG00000102053	-0,130709811	0,596046966	0,690868792
HBE1	protein_coding	ENSG00000213931	0,112455192	0,596628046	0,691503234
GPS2	protein_coding	ENSG00000132522	0,110345645	0,59670996	0,691559094
ALG9	protein_coding	ENSG00000086848	0,048034475	0,596757833	0,691575497
AC005586.1	antisense	ENSG00000240449	-0,122609713	0,596961771	0,691694589
AL592437.2	processed_pseudogene	ENSG00000232486	0,120021139	0,596944933	0,691694589
AC092821.3	processed_transcript	ENSG00000284634	0,11288632	0,596947441	0,691694589
GVQW3	protein_coding	ENSG00000179240	-0,091896676	0,59704637	0,691753534
ARL14EP	protein_coding	ENSG00000152219	0,035389479	0,597118488	0,691798011
TMEM199	protein_coding	ENSG00000244045	-0,0331286	0,597313253	0,69198457
RHBDD1	protein_coding	ENSG00000144468	-0,031017445	0,597387671	0,692031693
HAX1	protein_coding	ENSG00000143575	-0,038477991	0,597636973	0,692281392
FOXD1-AS1	lincRNA	ENSG00000247993	0,1268201	0,597851155	0,692490382
RPS3AP6	processed_pseudogene	ENSG00000234797	-0,071208619	0,598023447	0,692611717
AC008737.1	sense_intronic	ENSG00000268743	0,136589442	0,598022093	0,692611717
ZNF354A	protein_coding	ENSG00000169131	0,039556553	0,598062903	0,692618303
AC011939.1	processed_pseudogene	ENSG00000259924	0,137520649	0,598168164	0,692661983
PRELID1P1	transcribed_processed_	ENSG00000217325	-0,130082281	0,598164293	0,692661983

AC009245.1	processed_pseudogene	ENSG00000230383	-0,138079347	0,598405938	0,692898198
DOCK9-DT	antisense	ENSG00000260992	0,137333942	0,598706633	0,693162108
ALDH6A1	protein_coding	ENSG00000119711	0,042514739	0,598720674	0,693162108
BCAP29	protein_coding	ENSG00000075790	-0,041414082	0,598735246	0,693162108
AL662889.1	sense_intronic	ENSG00000232184	0,132068119	0,598990039	0,693417944
RSF1-IT2	sense_intronic	ENSG00000254985	0,133078567	0,599053261	0,693451991
ABHD17B	protein_coding	ENSG00000107362	-0,036099504	0,59939566	0,693809187
FEZF1-AS1	antisense	ENSG00000230316	-0,115181302	0,599840476	0,694245706
AL132989.1	sense_overlapping	ENSG00000258682	0,136452974	0,599822121	0,694245706
FBXW11	protein_coding	ENSG00000072803	-0,027041828	0,600069306	0,694471361
SERTAD4	protein_coding	ENSG00000082497	-0,042034502	0,600109165	0,694478303
EFHC1	protein_coding	ENSG00000096093	-0,029078196	0,600375956	0,694736299
ANAPC2	protein_coding	ENSG00000176248	-0,042293135	0,600399849	0,694736299
ZPR1	protein_coding	ENSG00000109917	0,021294012	0,600545896	0,694866091
RF00019	misc_RNA	ENSG00000202382	-0,109017393	0,600746971	0,695059535
CCR1	protein_coding	ENSG00000163823	0,133004309	0,601147763	0,695484015
TASOR2	protein_coding	ENSG00000108021	0,025833291	0,601326584	0,695533963
TMX4	protein_coding	ENSG00000125827	0,024595315	0,601310324	0,695533963
SNAP29	protein_coding	ENSG00000099940	-0,031708085	0,601229948	0,695533963
SNORA53	snoRNA	ENSG00000212443	-0,097062002	0,601309629	0,695533963
TRMT10B	protein_coding	ENSG00000165275	0,06760661	0,601618887	0,695832819
RNU6-123P	snRNA	ENSG00000251972	0,106307013	0,601723618	0,695914707
C9orf147	antisense	ENSG00000230185	0,119347204	0,601839856	0,696009893
KATNAL1	protein_coding	ENSG00000102781	0,030627211	0,601984823	0,696138291
AC004922.1	protein_coding	ENSG00000284292	-0,11671559	0,602133425	0,696270877
AC124057.1	antisense	ENSG00000230483	-0,11419133	0,602359506	0,696478515
MTND4P7	processed_pseudogene	ENSG00000253257	-0,134841829	0,602380906	0,696478515
ZNF337-AS1	antisense	ENSG00000213742	-0,057108307	0,602511846	0,696551376
SSR1	protein_coding	ENSG00000124783	0,025276039	0,602506936	0,696551376
PFN1P1	processed_pseudogene	ENSG00000233328	0,121790527	0,602834585	0,696885207
APTX	protein_coding	ENSG00000137074	-0,0297719	0,603129012	0,697172034
SART1	protein_coding	ENSG00000175467	-0,032609643	0,603150686	0,697172034
AC016205.1	lincRNA	ENSG00000267374	-0,065614304	0,603240019	0,697196708
RN7SKP11	misc_RNA	ENSG00000252827	0,091967846	0,603234298	0,697196708
SPCS2P4	processed_pseudogene	ENSG00000228589	-0,133671719	0,603400837	0,697337152
MICAL1	protein_coding	ENSG00000135596	-0,034250306	0,603429536	0,697337152
HOXB8	protein_coding	ENSG00000120068	0,118275414	0,603550904	0,697438111
BCRP7	unprocessed_pseudogene	ENSG00000215544	-0,127412371	0,603789747	0,6976748
CACTIN-AS1	antisense	ENSG00000226800	0,122378527	0,60388026	0,697737943
TMPOP2	processed_pseudogene	ENSG00000262904	-0,086088567	0,603912432	0,697737943

AC068896.1	protein_coding	ENSG00000258539	-0,109666209	0,604210208	0,698003344
AP005061.1	transcribed_processed_	ENSG00000266405	-0,12378959	0,604193628	0,698003344
AC116914.1	antisense	ENSG00000262358	-0,119425029	0,604293341	0,698060063
GLUL	protein_coding	ENSG00000135821	0,027012137	0,604474504	0,698230011
AC080038.2	antisense	ENSG00000274565	0,125553706	0,604677355	0,698398311
SCAMP3	protein_coding	ENSG00000116521	0,032558269	0,604688309	0,698398311
CD47	protein_coding	ENSG00000196776	0,025363197	0,604956843	0,698669117
EIF3I	protein_coding	ENSG00000084623	-0,033153904	0,605178999	0,698886333
CNTNAP3P2	unprocessed_pseudoge	ENSG00000276386	0,113628329	0,605238587	0,698915794
PCDHGB5	protein_coding	ENSG00000276547	-0,06170144	0,605609361	0,699304583
TMCO1-AS1	antisense	ENSG00000224358	0,110332038	0,605651056	0,699313357
ITPR1	protein_coding	ENSG00000150995	0,043032311	0,605912152	0,699575447
AC156455.1	processed_transcript	ENSG00000256546	-0,049903504	0,606017946	0,699616611
N4BP2L2-IT2	sense_intronic	ENSG00000281026	0,117767195	0,606050138	0,699616611
RNVU1-18	snRNA	ENSG00000206737	-0,130847549	0,606029788	0,699616611
KIAA0391	protein_coding	ENSG00000100890	0,050459466	0,606116285	0,699653591
TTLL11-IT1	lincRNA	ENSG00000237548	-0,107375749	0,606377098	0,699836491
EEF1A1P3	processed_pseudogene	ENSG00000232587	0,135036343	0,606372668	0,699836491
TTC36	protein_coding	ENSG00000172425	0,125888371	0,60631546	0,699836491
AL022322.1	lincRNA	ENSG00000272720	0,114650201	0,606730163	0,700204571
HSPA1A	protein_coding	ENSG00000204389	0,029262659	0,606792077	0,700236623
FAS	protein_coding	ENSG00000026103	0,02756194	0,607140554	0,700599345
CEBPA-DT	antisense	ENSG00000267296	-0,10920244	0,607291657	0,700734283
STARD13-IT1	sense_intronic	ENSG00000230300	0,11806195	0,607827139	0,701312703
IBA57-DT	lincRNA	ENSG00000203684	-0,124584372	0,607975306	0,701444198
USP48	protein_coding	ENSG00000090686	-0,028102251	0,608194821	0,701657991
MTMR8	protein_coding	ENSG00000102043	-0,057482467	0,608271791	0,70170732
SLC24A1	protein_coding	ENSG00000074621	-0,044159719	0,608341969	0,701748807
TTN-AS1	antisense	ENSG00000237298	0,03648159	0,608401233	0,701773446
EPB41L5	protein_coding	ENSG00000115109	0,035203883	0,608431761	0,701773446
GPAA1	protein_coding	ENSG00000197858	-0,045672518	0,60869325	0,702035571
FAM204A	protein_coding	ENSG00000165669	0,03294222	0,608877526	0,702208617
HLA-DPB1	protein_coding	ENSG00000223865	-0,128335719	0,609045287	0,7023626
AL139289.2	antisense	ENSG00000234694	-0,107698413	0,60920908	0,702511989
AC005730.3	sense_intronic	ENSG00000266126	-0,117205183	0,609510901	0,70282052
AC008770.3	sense_overlapping	ENSG00000267274	-0,127791218	0,609552441	0,702828905
ZNF582-AS1	lincRNA	ENSG00000267454	0,11077938	0,609678972	0,702935281
AC005785.1	processed_transcript	ENSG00000268189	-0,132861632	0,60974438	0,702940265
LAD1	protein_coding	ENSG00000159166	0,128656429	0,609751841	0,702940265
EFNA2	protein_coding	ENSG00000099617	-0,109758901	0,609884565	0,703053756

RAB3B	protein_coding	ENSG00000169213	0,028347998	0,610075455	0,703234283
AC097641.2	antisense	ENSG00000277728	0,132842794	0,610295308	0,703448172
ZC3H15	protein_coding	ENSG00000065548	0,028178503	0,610451273	0,703588402
AC025171.4	lincRNA	ENSG00000272144	-0,110159511	0,610685427	0,703818729
FAM53B	protein_coding	ENSG00000189319	-0,049107849	0,610739297	0,703841264
AC007688.1	processed_pseudogene	ENSG00000241352	0,131855177	0,611215691	0,704350704
TP73	protein_coding	ENSG00000078900	-0,069504933	0,611364895	0,704483061
CFAP77	protein_coding	ENSG00000188523	-0,130808769	0,611400097	0,704484044
NSRP1P1	processed_pseudogene	ENSG00000235613	0,11360414	0,611460968	0,704514603
WDTC1	protein_coding	ENSG00000142784	0,036493049	0,612045837	0,705137082
PMS2P5	unprocessed_pseudoge	ENSG00000123965	0,122904652	0,612069988	0,705137082
FOXG1-AS1	antisense	ENSG00000257126	0,075664683	0,612206223	0,705214807
LYPD8	protein_coding	ENSG00000259823	-0,062433729	0,612174811	0,705214807
AC022968.1	processed_pseudogene	ENSG00000260747	-0,131125716	0,612408549	0,705368638
ECPAS	protein_coding	ENSG00000136813	-0,023609978	0,612390106	0,705368638
AC011448.1	protein_coding	ENSG00000258674	0,133003858	0,612628873	0,705582782
LINC00397	lincRNA	ENSG00000223404	0,131949778	0,612923235	0,705882169
AL359232.1	lincRNA	ENSG00000258561	0,112957303	0,612960444	0,705885385
PLA2G12A	protein_coding	ENSG00000123739	0,03053774	0,613027636	0,705923127
TNFAIP8L3	protein_coding	ENSG00000183578	-0,117889052	0,613273307	0,706166379
AC090109.1	antisense	ENSG00000258115	-0,107660283	0,613592741	0,706494533
AL139384.1	lincRNA	ENSG00000274922	0,126801365	0,613750851	0,706498834
AC026367.2	lincRNA	ENSG00000275409	-0,124001178	0,613685714	0,706498834
GAPDHP65	processed_pseudogene	ENSG00000235587	0,117801054	0,613758209	0,706498834
AL137845.2	processed_pseudogene	ENSG00000232842	-0,126395512	0,61364154	0,706498834
AC092329.4	processed_transcript	ENSG00000284428	-0,128778558	0,613768709	0,706498834
RN7SL220P	misc_RNA	ENSG00000266467	-0,123296336	0,613842037	0,706543587
MYLK2	protein_coding	ENSG00000101306	0,111568855	0,613968855	0,7066499
VMAC	protein_coding	ENSG00000187650	-0,105140897	0,614128458	0,706793933
MNT	protein_coding	ENSG00000070444	-0,044344282	0,614387313	0,707052171
FAM76A	protein_coding	ENSG00000009780	-0,041433937	0,614541504	0,707150261
AC008870.2	sense_intronic	ENSG00000260751	-0,128278629	0,614534299	0,707150261
ST6GALNAC3	protein_coding	ENSG00000184005	0,113928403	0,614600022	0,707177921
AC136632.1	transcribed_processed_	ENSG00000218227	0,089046294	0,614781562	0,707347124
LINC01347	transcribed_unprocesse	ENSG00000214837	0,131694616	0,614980301	0,707536096
AC073130.2	antisense	ENSG00000243243	0,10131379	0,615180777	0,707671649
CIB2	protein_coding	ENSG00000136425	-0,105263774	0,615191096	0,707671649
AC090114.3	unprocessed_pseudoge	ENSG00000280828	0,131243862	0,615537712	0,708018534
ZNF672	protein_coding	ENSG00000171161	-0,031886302	0,61558568	0,708034001
AC104118.1	lincRNA	ENSG00000271862	-0,123257463	0,615896272	0,708313389

USF2	protein_coding	ENSG00000105698	0,02584348	0,615897659	0,708313389
TWF1P1	processed_pseudogene	ENSG00000178082	-0,094190711	0,616000131	0,708391516
MMP11	protein_coding	ENSG00000099953	0,128134619	0,616062574	0,708423604
MRPS14	protein_coding	ENSG00000120333	0,033871453	0,616246374	0,708595231
PRR14	protein_coding	ENSG00000156858	-0,05057755	0,616478574	0,708822488
AL133330.1	sense_intronic	ENSG00000251194	-0,113785444	0,616532932	0,708845251
AL163051.1	antisense	ENSG00000260806	-0,121372759	0,616580556	0,708860269
FAM220A	protein_coding	ENSG00000178397	-0,035641897	0,616754738	0,709020777
C20orf204	protein_coding	ENSG00000196421	0,113132702	0,616804141	0,709037829
CEP128	protein_coding	ENSG00000100629	-0,040052728	0,616839633	0,709038888
AL161908.1	antisense	ENSG00000224842	0,113991181	0,616974745	0,709114711
AC027702.1	lincRNA	ENSG00000260588	0,126541013	0,616970604	0,709114711
LINC00893	antisense	ENSG00000241769	0,130045046	0,617229305	0,709326976
KAZN-AS1	lincRNA	ENSG00000234593	-0,037882117	0,617436104	0,709326976
SYNGR3	protein_coding	ENSG00000127561	0,102709334	0,617410809	0,709326976
DDX49	protein_coding	ENSG00000105671	0,032557762	0,617399926	0,709326976
STK39	protein_coding	ENSG00000198648	0,025759087	0,617292415	0,709326976
UBA52	protein_coding	ENSG00000221983	0,025629721	0,617306215	0,709326976
HDLBP	protein_coding	ENSG00000115677	-0,028168899	0,617394224	0,709326976
AC006116.5	sense_intronic	ENSG00000267298	-0,100980348	0,617232944	0,709326976
HESX1	protein_coding	ENSG00000163666	-0,109601087	0,617479629	0,709337246
KRT18P28	processed_pseudogene	ENSG00000176855	-0,127989806	0,617567529	0,709398489
TUBA3FP	processed_transcript	ENSG00000161149	-0,118524629	0,617638375	0,709440136
AC007780.1	processed_transcript	ENSG00000267009	0,106044486	0,617709572	0,709482181
RPL29P11	processed_pseudogene	ENSG00000224858	0,128046246	0,617807397	0,709554805
ADCY2	protein_coding	ENSG00000078295	0,107608086	0,61845288	0,710256373
UFD1	protein_coding	ENSG00000070010	0,033002817	0,618527168	0,710301915
EVL	protein_coding	ENSG00000196405	0,038623179	0,618591404	0,71033591
AP000577.1	TEC	ENSG00000280269	-0,126852452	0,618744115	0,710471492
ITPR1-DT	antisense	ENSG00000231249	0,129672511	0,619178799	0,710930816
AC093635.1	lincRNA	ENSG00000272663	0,127959171	0,619363293	0,711081779
HOMER1	protein_coding	ENSG00000152413	-0,031444366	0,619379619	0,711081779
KIAA0319	protein_coding	ENSG00000137261	0,098461012	0,619708281	0,711419279
ELMOD2	protein_coding	ENSG00000179387	-0,037420413	0,619836015	0,711526091
AC018521.5	processed_transcript	ENSG00000264920	0,083281349	0,619948191	0,711615032
GTF2IP4	transcribed_processed_	ENSG00000233369	-0,059267432	0,620071197	0,711716394
CC2D2A	protein_coding	ENSG00000048342	-0,028455218	0,620467845	0,712131814
HSPD1P4	processed_pseudogene	ENSG00000257576	-0,126383159	0,620587302	0,712229062
RF00410	snoRNA	ENSG00000202343	0,125449253	0,620682501	0,712298461
AL138752.1	processed_pseudogene	ENSG00000232454	-0,106813078	0,62150242	0,713199499

IQCH-AS1	lincRNA	ENSG00000259673	0,057875777	0,621695523	0,713341269
TNFAIP1	protein_coding	ENSG00000109079	0,017563458	0,621809077	0,71343165
FRG1-DT	lincRNA	ENSG00000245685	0,12761365	0,621900392	0,713456597
AC022868.1	processed_pseudogene	ENSG00000253833	-0,102466087	0,621873974	0,713456597
HSPD1P11	processed_pseudogene	ENSG00000251348	-0,113091675	0,622028508	0,713545393
ZNF592	protein_coding	ENSG00000166716	0,036051084	0,622071215	0,713545393
KRT4	protein_coding	ENSG00000170477	-0,103382555	0,622082164	0,713545393
GCSHP5	processed_pseudogene	ENSG00000224837	-0,12086986	0,622309161	0,713765848
EIF1AXP1	processed_pseudogene	ENSG00000236698	-0,108559597	0,622473994	0,713914982
AL354696.1	sense_intronic	ENSG00000277662	0,124784137	0,622646468	0,714072861
AC107214.1	antisense	ENSG00000232648	-0,119125484	0,622699486	0,714093735
DRG2	protein_coding	ENSG00000108591	0,030006813	0,623323409	0,714769267
ANXA2R	protein_coding	ENSG00000177721	0,085776848	0,623518655	0,714873252
ATF1	protein_coding	ENSG00000123268	0,042496724	0,623511581	0,714873252
AC099811.5	sense_intronic	ENSG00000278829	-0,12341892	0,623491526	0,714873252
TMEM106B	protein_coding	ENSG00000106460	-0,040929724	0,623664828	0,715000873
AL022067.1	sense_intronic	ENSG00000269919	0,108812173	0,623730427	0,715036111
RBFADN	lincRNA	ENSG00000261126	0,128019031	0,623765499	0,71503635
FAIM	protein_coding	ENSG00000158234	0,038854591	0,624145168	0,715431587
AC005244.1	processed_pseudogene	ENSG00000263648	-0,11282916	0,624295528	0,715563947
RAD51B	protein_coding	ENSG00000182185	0,04123046	0,624418609	0,715665027
AC006299.1	antisense	ENSG00000246225	-0,116929998	0,624499891	0,715718192
ADCK1	protein_coding	ENSG00000063761	-0,049388991	0,624630302	0,715827653
ZNF572	protein_coding	ENSG00000180938	-0,070518202	0,624720191	0,715890664
YBX1	protein_coding	ENSG00000065978	0,024120269	0,624836275	0,715983686
AC093616.1	transcribed_unprocesse	ENSG00000234231	-0,072966622	0,625146934	0,716259629
AC004980.3	unprocessed_pseudoge	ENSG00000230305	-0,122248146	0,625507707	0,716632951
AP001372.2	bidirectional_promoter_	ENSG00000254837	0,073523813	0,625587444	0,71668427
MST1	protein_coding	ENSG00000173531	0,110244338	0,625629925	0,716692904
AP000553.5	transcribed_unitary_pse	ENSG00000284630	-0,113828972	0,625726206	0,716763166
MTND2P28	unprocessed_pseudoge	ENSG00000225630	0,120547795	0,625786809	0,716792553
AC026191.1	antisense	ENSG00000254485	0,124548869	0,625909578	0,71689314
PDCD5P1	processed_pseudogene	ENSG00000255909	-0,107920634	0,62633941	0,717265287
CILP2	protein_coding	ENSG00000160161	0,124359238	0,626329198	0,717265287
AC006270.2	transcribed_processed_	ENSG00000264529	-0,102911981	0,626289318	0,717265287
AL732314.6	antisense	ENSG00000281849	-0,109053536	0,62639969	0,717294268
SIRPB1	protein_coding	ENSG00000101307	0,127258192	0,626834848	0,717752497
AC068790.6	sense_intronic	ENSG00000270095	0,100234499	0,626943052	0,71783632
GALNT13	protein_coding	ENSG00000144278	0,121335245	0,627106936	0,717983882
ZNF483	protein_coding	ENSG00000173258	-0,050262599	0,62745625	0,718343716

CEP41	protein_coding	ENSG00000106477	-0,027975992	0,627622121	0,718493509
SIRPAP1	processed_pseudogene	ENSG00000225774	-0,103745982	0,627910083	0,718783044
RPL10AP6	processed_pseudogene	ENSG00000226360	-0,087413104	0,628075388	0,718932146
BABAM1	protein_coding	ENSG00000105393	-0,035259631	0,628242899	0,719083758
AC004707.1	antisense	ENSG00000253102	0,115127781	0,628299038	0,719106045
AC008592.3	processed_pseudogene	ENSG00000251000	0,125441867	0,628332493	0,719106045
AC023421.2	antisense	ENSG00000267193	0,106481539	0,628420676	0,719146252
HMGN2P41	processed_pseudogene	ENSG00000261620	-0,12624186	0,628437751	0,719146252
SHKBP1	protein_coding	ENSG00000160410	0,034882586	0,628608498	0,719301511
ZNF85	protein_coding	ENSG00000105750	0,057747729	0,628772037	0,719448506
HMGN5	protein_coding	ENSG00000198157	0,105985224	0,629094404	0,719777208
AC145285.6	antisense	ENSG00000275807	0,123686268	0,629588343	0,720283156
AC009041.4	sense_intronic	ENSG00000276931	-0,123505632	0,629606847	0,720283156
AC128709.3	lincRNA	ENSG00000237167	0,12665642	0,629817046	0,720483441
AC004918.3	sense_intronic	ENSG00000270157	-0,099377052	0,629904932	0,72054379
AC106712.1	lincRNA	ENSG00000241280	-0,10411083	0,630112047	0,720740509
AC105206.1	sense_overlapping	ENSG00000177725	-0,103227858	0,630365258	0,720989931
RPL5P12	processed_pseudogene	ENSG00000242697	-0,124306836	0,630411667	0,721002805
LINC01474	lincRNA	ENSG00000236849	-0,105160204	0,630518222	0,721051468
AC007663.3	lincRNA	ENSG00000273139	-0,112081672	0,630524528	0,721051468
AL357054.1	processed_pseudogene	ENSG00000219712	-0,101205331	0,63056698	0,72105981
SLFN11	protein_coding	ENSG00000172716	0,125266136	0,630667265	0,721134281
SRRM2-AS1	antisense	ENSG00000205913	0,112349791	0,630748488	0,721186949
AP001033.1	lincRNA	ENSG00000264964	0,109019442	0,630864302	0,721238953
ATP6V0D2	protein_coding	ENSG00000147614	-0,114205317	0,630841075	0,721238953
OVAAL	lincRNA	ENSG00000236719	0,125543194	0,630927327	0,721270803
TMPOP1	processed_pseudogene	ENSG00000229068	0,111503431	0,631131338	0,721423605
ARL6IP4	protein_coding	ENSG00000182196	0,102598505	0,631117685	0,721423605
AL358933.1	lincRNA	ENSG00000261839	-0,125039318	0,631288947	0,721439172
SAA1	protein_coding	ENSG00000173432	0,105311216	0,631320832	0,721439172
ECHDC3	protein_coding	ENSG00000134463	0,054658552	0,631238126	0,721439172
ERGIC3	protein_coding	ENSG00000125991	0,024793925	0,631317948	0,721439172
SCAMP1	protein_coding	ENSG00000085365	-0,034565749	0,631253897	0,721439172
SYPL2	protein_coding	ENSG00000143028	0,120204011	0,631694401	0,721825849
AC067750.1	antisense	ENSG00000272631	0,111784817	0,631933481	0,722058813
AC015909.2	antisense	ENSG00000253730	0,122618543	0,632065811	0,722169784
TMEM251	protein_coding	ENSG00000153485	0,053672162	0,632529588	0,722659417
RAB37	protein_coding	ENSG00000172794	-0,106273501	0,632940293	0,723088365
HUWE1	protein_coding	ENSG00000086758	0,040137653	0,633089623	0,723218679
SYNGAP1-AS1	antisense	ENSG00000274259	-0,123147712	0,633133572	0,723228603

ST13P5	processed_pseudogene	ENSG00000212789	-0,119314868	0,633335755	0,723302501
CDKL2	protein_coding	ENSG00000138769	0,12499002	0,63331709	0,723302501
RAB23	protein_coding	ENSG00000112210	0,032118659	0,633345841	0,723302501
CD9	protein_coding	ENSG00000010278	-0,024037541	0,633305502	0,723302501
AC007497.1	sense_intronic	ENSG00000275191	0,10005245	0,633374594	0,723302501
GRAMD1C	protein_coding	ENSG00000178075	-0,042985347	0,633529414	0,723439022
VIT	protein_coding	ENSG00000205221	0,111639683	0,633645667	0,723531491
SNORA26	snoRNA	ENSG00000212588	0,114863338	0,633789672	0,723655635
AC068633.1	lincRNA	ENSG00000243276	0,106046268	0,633897762	0,723698475
LAMTOR5-AS1	processed_transcript	ENSG00000224699	-0,061793787	0,633868405	0,723698475
SMG1	protein_coding	ENSG00000157106	0,037450903	0,633969493	0,723740081
KRT8P8	processed_pseudogene	ENSG00000223940	-0,123880298	0,634047169	0,723760538
REP15	protein_coding	ENSG00000174236	0,121540641	0,634066991	0,723760538
PCDHB14	protein_coding	ENSG00000120327	-0,118274441	0,634093277	0,723760538
AC090587.1	antisense	ENSG00000228661	0,109482485	0,634263363	0,723897564
ZNF616	protein_coding	ENSG00000204611	0,053313337	0,634283917	0,723897564
AL035587.1	antisense	ENSG00000231113	0,085304659	0,634384399	0,723901818
GSTM2	protein_coding	ENSG00000213366	0,123719859	0,634411265	0,723901818
MYADM	protein_coding	ENSG00000179820	0,032833255	0,634428824	0,723901818
FAM89A	protein_coding	ENSG00000182118	-0,055617459	0,63436258	0,723901818
IZUMO4	protein_coding	ENSG00000099840	0,123981371	0,634563386	0,723999658
WAPL	protein_coding	ENSG00000062650	0,028899908	0,634585171	0,723999658
UBE2V1	protein_coding	ENSG00000244687	-0,072424649	0,634737358	0,724114793
PXMP2	protein_coding	ENSG00000176894	-0,081576965	0,634756698	0,724114793
AL513327.2	antisense	ENSG00000233246	0,12448561	0,634831868	0,724117575
AL021707.3	antisense	ENSG00000230149	0,11160976	0,634864845	0,724117575
VPS26BP1	processed_pseudogene	ENSG00000224895	0,108289985	0,634865053	0,724117575
RPSAP15	processed_pseudogene	ENSG00000237506	-0,116438282	0,63499793	0,724214696
RAB15	protein_coding	ENSG00000139998	-0,031978908	0,635020823	0,724214696
MSS51	protein_coding	ENSG00000166343	-0,11223739	0,635422435	0,724632424
AC113935.1	processed_pseudogene	ENSG00000226084	-0,060196044	0,63573743	0,724939549
ATG3	protein_coding	ENSG00000144848	0,025150979	0,635797787	0,724939549
POLR2F	protein_coding	ENSG00000100142	-0,106132901	0,635779577	0,724939549
AC008592.1	processed_transcript	ENSG00000250362	0,122574131	0,636071005	0,725210757
AC009137.2	sense_intronic	ENSG00000266801	-0,109248267	0,636348169	0,725486434
LINC01446	lincRNA	ENSG00000205628	0,070839805	0,636578406	0,725708583
ST13P3	processed_pseudogene	ENSG00000257773	-0,107216612	0,636619737	0,725715362
TERB1	protein_coding	ENSG00000249961	0,105956638	0,636659055	0,725719848
LINC01481	lincRNA	ENSG00000257613	0,100511272	0,637004217	0,726072941
AC025459.1	processed_pseudogene	ENSG00000251062	0,119520349	0,637117728	0,726121616

AC079944.1	processed_pseudogene	ENSG00000240776	0,111367498	0,637094094	0,726121616
MICOS10P3	processed_pseudogene	ENSG00000224237	-0,099516157	0,637204008	0,726179597
TMEM33	protein_coding	ENSG00000109133	-0,027629039	0,637405906	0,726320988
C6orf203	protein_coding	ENSG00000130349	-0,039822204	0,637434314	0,726320988
RPLP2	protein_coding	ENSG00000177600	-0,033800536	0,63764184	0,72651709
NHEJ1	protein_coding	ENSG00000187736	0,108749123	0,637801415	0,726618176
RAD50	protein_coding	ENSG00000113522	-0,0488674	0,637775596	0,726618176
RRAGD	protein_coding	ENSG00000025039	0,029605014	0,637844053	0,726626391
EAPP	protein_coding	ENSG00000129518	0,037709269	0,637926127	0,726679526
RANP3	processed_pseudogene	ENSG00000254500	0,109332767	0,637974292	0,726694031
EEF1B2P6	processed_pseudogene	ENSG00000213261	-0,08615972	0,638064134	0,726756005
PBX2P1	processed_pseudogene	ENSG00000244171	-0,097843379	0,63834403	0,727034432
DENND2C	protein_coding	ENSG00000175984	0,055271421	0,638573414	0,72721492
AP000864.1	sense_intronic	ENSG00000272788	-0,107231669	0,638538146	0,72721492
AC011676.1	antisense	ENSG00000253307	-0,102714506	0,638726478	0,727227715
GCOM2	processed_pseudogene	ENSG00000227725	-0,107850476	0,638713852	0,727227715
NCOR2	protein_coding	ENSG00000196498	0,040421183	0,63867917	0,727227715
WIZ	protein_coding	ENSG00000011451	0,039976513	0,638699943	0,727227715
AC068446.1	processed_pseudogene	ENSG00000237161	0,112042324	0,639050006	0,727555682
AP3B1	protein_coding	ENSG00000132842	0,021833593	0,6391421	0,727620141
PIP4K2A	protein_coding	ENSG00000150867	-0,026891405	0,639255811	0,727668813
ZC3H13	protein_coding	ENSG00000123200	-0,040682049	0,639294158	0,727672079
PJVK	protein_coding	ENSG00000204311	0,101554356	0,639346557	0,727691337
CISD1	protein_coding	ENSG00000122873	-0,03044517	0,639394667	0,727705711
AC015971.1	antisense	ENSG00000228363	0,101100033	0,639501863	0,727787327
SUCLA2P1	processed_pseudogene	ENSG00000224936	0,103032301	0,639652123	0,727917941
DLG3	protein_coding	ENSG00000082458	0,054216648	0,639696033	0,727927521
HDDC2	protein_coding	ENSG00000111906	-0,025901976	0,639784166	0,72798742
DFFBP1	processed_pseudogene	ENSG00000232303	-0,118807196	0,640079909	0,728198744
PANK3	protein_coding	ENSG00000120137	0,032607951	0,640061946	0,728198744
AC002310.6	TEC	ENSG00000278922	0,103922435	0,640111904	0,728198744
AC004925.1	TEC	ENSG00000279419	-0,111639948	0,640084794	0,728198744
DNM1P35	antisense	ENSG00000246877	-0,116897905	0,640195439	0,728253381
NDRG2	protein_coding	ENSG00000165795	0,10381016	0,640250086	0,728275152
RPL5P30	processed_pseudogene	ENSG00000243431	0,106138762	0,640441884	0,72845292
THORLNC	lincRNA	ENSG00000226856	-0,121334473	0,640775544	0,728751603
HLA-DQB1	protein_coding	ENSG00000179344	0,117821249	0,640820093	0,728761858
AL356481.3	antisense	ENSG00000280758	-0,113799283	0,64092261	0,728838031
AKR1E2	protein_coding	ENSG00000165568	-0,034134765	0,641043835	0,728895058
ZBED2	protein_coding	ENSG00000177494	-0,107719227	0,64102162	0,728895058

TRIM52	protein_coding	ENSG00000183718	-0,046282764	0,641140549	0,728964613
AC039056.2	sense_intronic	ENSG00000278493	0,122417018	0,641207714	0,729000566
AC093424.1	lincRNA	ENSG00000270457	0,12139483	0,641335995	0,729068214
MAGED1	protein_coding	ENSG00000179222	-0,024207098	0,641338309	0,729068214
TPRG1L	protein_coding	ENSG00000158109	0,034626006	0,641481923	0,729191057
FEM1B	protein_coding	ENSG00000169018	-0,026629108	0,641725819	0,729427873
DEPP1	protein_coding	ENSG00000165507	0,060767476	0,642047809	0,729753426
NAGPA-AS1	lincRNA	ENSG00000267072	-0,107554131	0,642507079	0,730234966
AC083843.2	lincRNA	ENSG00000254028	0,108290661	0,642599149	0,730299138
AC104117.2	processed_pseudogene	ENSG00000253144	-0,11427856	0,642944585	0,730651233
AC109347.2	lincRNA	ENSG00000272567	0,111137812	0,643061794	0,730703457
AC100774.1	sense_intronic	ENSG00000261529	0,103013541	0,64306025	0,730703457
AC036176.2	processed_pseudogene	ENSG00000267430	-0,120220973	0,643329509	0,730967161
EGFL8	protein_coding	ENSG00000241404	-0,120315921	0,643500388	0,731120816
AP000253.1	lincRNA	ENSG00000234509	0,111214053	0,643714732	0,731314518
LINC00470	lincRNA	ENSG00000132204	-0,120386041	0,64374219	0,731314518
AP3S2	protein_coding	ENSG00000157823	-0,041741324	0,643934835	0,731492853
MAP4K2	protein_coding	ENSG00000168067	0,032632406	0,644029084	0,731559399
MEA1	protein_coding	ENSG00000124733	-0,029503595	0,644256486	0,73177718
ZNF451-AS1	antisense	ENSG00000226803	0,106499884	0,64442398	0,731926892
KANK3	protein_coding	ENSG00000186994	0,04221477	0,644530395	0,732007221
C6orf99	lincRNA	ENSG00000203711	0,045784902	0,644643017	0,732094589
ATM	protein_coding	ENSG00000149311	0,028970811	0,644760931	0,732187957
AC004771.3	antisense	ENSG00000262227	0,120322549	0,645014408	0,732273083
AC022098.3	antisense	ENSG00000267783	0,105396211	0,645013631	0,732273083
AL359397.1	lincRNA	ENSG00000258857	-0,115149865	0,644905326	0,732273083
AC020898.1	processed_pseudogene	ENSG00000213757	-0,119823054	0,64501027	0,732273083
DNAJC13	protein_coding	ENSG00000138246	0,030492729	0,644966957	0,732273083
LINC01411	lincRNA	ENSG00000249306	0,102378826	0,645206982	0,732410626
ICA1	protein_coding	ENSG00000003147	0,036230637	0,645175297	0,732410626
AKR1C3	protein_coding	ENSG00000196139	-0,10481622	0,645424678	0,732599001
SLC22A13	protein_coding	ENSG00000172940	-0,118476823	0,645444366	0,732599001
XPOT	protein_coding	ENSG00000184575	-0,019861905	0,64555725	0,73268658
POLR2E	protein_coding	ENSG00000099817	-0,033160127	0,645747985	0,732862504
RPL34P18	processed_pseudogene	ENSG00000240509	-0,119676838	0,646005617	0,733114325
SCOC-AS1	antisense	ENSG00000196951	-0,114083985	0,646058474	0,733133744
LINC02601	lincRNA	ENSG00000223714	0,116096412	0,646333236	0,733404961
ECHS1	protein_coding	ENSG00000127884	-0,03256195	0,646387245	0,733425669
BAIAP3	protein_coding	ENSG00000007516	0,071164255	0,646713488	0,733733501
PERP	protein_coding	ENSG00000112378	0,018411955	0,646730095	0,733733501

AC009951.1	TEC	ENSG00000279166	0,115206908	0,6468639	0,733844715
RPL23AP53	transcribed_processed_	ENSG00000223508	-0,056142749	0,646935433	0,733885273
UPRT	protein_coding	ENSG00000094841	-0,031197765	0,646976445	0,733891206
RTRAF	protein_coding	ENSG00000087302	0,024369982	0,647053073	0,733937537
AC241584.1	processed_pseudogene	ENSG00000230832	0,113858225	0,647247148	0,734076477
RN7SL732P	misc_RNA	ENSG00000264618	0,115363968	0,647740422	0,734522789
ANXA9	protein_coding	ENSG00000143412	0,068779322	0,647693226	0,734522789
PLEKHG5	protein_coding	ENSG00000171680	0,053426111	0,647748108	0,734522789
RPL7P21	processed_pseudogene	ENSG00000231579	-0,118387663	0,647806794	0,734548726
IDH1-AS1	antisense	ENSG00000231908	-0,115302439	0,647965274	0,734687808
AC002550.2	antisense	ENSG00000276571	-0,092104628	0,648009437	0,734697267
SERBP1P6	processed_pseudogene	ENSG00000248873	0,1186168	0,648131759	0,734795333
SENP7	protein_coding	ENSG00000138468	-0,032270272	0,648216025	0,734809632
AC022405.1	sense_intronic	ENSG00000259254	-0,111233108	0,648185043	0,734809632
CD38	protein_coding	ENSG00000004468	0,05269031	0,648313963	0,734880037
BACH1-IT1	sense_intronic	ENSG00000248476	-0,099178569	0,648369442	0,734902308
GMPSP1	processed_pseudogene	ENSG00000250471	-0,117634701	0,648630208	0,73515238
KIFC2	protein_coding	ENSG00000167702	0,052070058	0,6486976	0,73515238
FOXQ1	protein_coding	ENSG00000164379	-0,118626197	0,648688372	0,73515238
RN7SKP173	misc_RNA	ENSG00000199691	0,108153962	0,649328102	0,735826253
AC083873.1	processed_pseudogene	ENSG00000237821	-0,117223984	0,649476758	0,73595405
SLC39A6	protein_coding	ENSG00000141424	-0,020019816	0,6495321	0,735966012
HTD2	protein_coding	ENSG00000255154	-0,113478161	0,649559081	0,735966012
SUMO2P1	processed_pseudogene	ENSG00000235238	-0,116002602	0,6501263	0,736542134
DDX59	protein_coding	ENSG00000118197	-0,026630719	0,650139385	0,736542134
PRMT5-AS1	antisense	ENSG00000237054	0,10900461	0,650279146	0,736640227
BBIP1	protein_coding	ENSG00000214413	0,033783317	0,650297803	0,736640227
TUBBP8	processed_pseudogene	ENSG00000259300	0,112985464	0,650346987	0,736655255
FAM76B	protein_coding	ENSG00000077458	0,04275162	0,65038701	0,736659906
DCST1-AS1	antisense	ENSG00000232093	-0,092749593	0,650466617	0,736709389
KLF7-IT1	sense_intronic	ENSG00000237892	-0,103776809	0,650837358	0,737088581
H3F3BP1	processed_pseudogene	ENSG00000236534	-0,107507331	0,650913143	0,737133707
AC090519.1	processed_pseudogene	ENSG00000250130	0,111754513	0,651049491	0,737247409
AC004233.4	lincRNA	ENSG00000274367	-0,104434637	0,651194055	0,737370401
ZNF428	protein_coding	ENSG00000131116	-0,038770568	0,651240668	0,737382472
ZMAT5	protein_coding	ENSG00000100319	-0,041575814	0,651683008	0,737842589
AL033519.3	lincRNA	ENSG00000228559	0,102691266	0,651800132	0,737853003
EEF1DP1	processed_pseudogene	ENSG00000228887	0,113100249	0,651791354	0,737853003
EMC6	protein_coding	ENSG00000127774	-0,031360487	0,651785834	0,737853003
AC127496.7	TEC	ENSG00000280351	0,09634876	0,651844159	0,737862118

PCNX3	protein_coding	ENSG00000197136	-0,037379598	0,651941012	0,737931024
AC073046.1	lincRNA	ENSG00000235499	0,101590847	0,65209638	0,738025425
ZNF274	protein_coding	ENSG00000171606	0,017326055	0,652075059	0,738025425
SERBP1P1	processed_pseudogene	ENSG00000213740	-0,111577144	0,652195148	0,738096479
AC142086.6	unprocessed_pseudoge	ENSG00000277304	-0,097772402	0,652247329	0,738114805
INTS4	protein_coding	ENSG00000149262	-0,024403452	0,652398125	0,738244719
LARP4B	protein_coding	ENSG00000107929	0,026476087	0,652763055	0,738616918
AL591895.1	antisense	ENSG00000242861	-0,113942919	0,652860462	0,738686383
AC104763.1	processed_pseudogene	ENSG00000241157	0,116457019	0,652969262	0,738732058
AC087821.1	TEC	ENSG00000278886	-0,110987232	0,652972866	0,738732058
TYMSOS	bidirectional_promoter_	ENSG00000176912	-0,064908478	0,653066964	0,738797763
AL033528.2	antisense	ENSG00000236528	0,108240779	0,653190374	0,738833439
AC093151.3	lincRNA	ENSG00000235358	0,108534138	0,65320657	0,738833439
AC016596.2	processed_pseudogene	ENSG00000264281	-0,114927212	0,653169484	0,738833439
LHX3	protein_coding	ENSG00000107187	-0,099839499	0,65349385	0,739117617
SLC29A3	protein_coding	ENSG00000198246	-0,100823327	0,653548904	0,739139124
C18orf21	protein_coding	ENSG00000141428	0,035218865	0,653772549	0,739310524
TTLL9	protein_coding	ENSG00000131044	0,116893978	0,653812539	0,739314984
SLC35E2A	protein_coding	ENSG00000215790	-0,065023982	0,653913415	0,739388287
CCDC85C	protein_coding	ENSG00000205476	0,034851017	0,65428051	0,739745578
BRD4	protein_coding	ENSG00000141867	0,028917604	0,654301537	0,739745578
STAC3	protein_coding	ENSG00000185482	0,117129599	0,654475057	0,739900971
AC092720.1	lincRNA	ENSG00000260750	-0,116579333	0,6547427	0,740162751
AC015802.4	antisense	ENSG00000267546	0,116618496	0,654813609	0,740180327
AC025175.1	lincRNA	ENSG00000272416	0,107805145	0,654830426	0,740180327
LINC01852	lincRNA	ENSG00000236914	0,102529166	0,655020302	0,740195565
AC025165.5	lincRNA	ENSG00000270039	-0,111524764	0,655060444	0,740195565
PA2G4P2	processed_pseudogene	ENSG00000235698	0,096908703	0,654972073	0,740195565
ATG101	protein_coding	ENSG00000123395	0,027705247	0,654987796	0,740195565
CCP110	protein_coding	ENSG00000103540	-0,028536108	0,655041689	0,740195565
GVQW2	protein_coding	ENSG00000279968	-0,114915778	0,654930452	0,740195565
FTH1P8	processed_pseudogene	ENSG00000219507	-0,109438778	0,655141892	0,740246816
GPR75	protein_coding	ENSG00000119737	0,112687548	0,65519735	0,740268696
ARL17B	protein_coding	ENSG00000228696	0,100858788	0,655253469	0,740291321
ARL17B	protein_coding	ENSG00000228696	0,100858788	0,655253469	0,740291321
ARL17B	protein_coding	ENSG00000228696	0,100858788	0,655253469	0,740291321
AL390318.1	TEC	ENSG00000279819	-0,089641338	0,655292703	0,740294869
RHEBP1	processed_pseudogene	ENSG00000229927	0,097434995	0,655423	0,740401285
AL121583.1	lincRNA	ENSG00000277692	0,101813964	0,655845063	0,740670563
AL451085.1	lincRNA	ENSG00000270361	0,101146618	0,655705136	0,740670563

AL080243.2	processed_pseudogene	ENSG00000237214	-0,11200349	0,655845039	0,740670563
TMCO2	protein_coding	ENSG00000188800	0,101436541	0,655878048	0,740670563
LRP4	protein_coding	ENSG00000134569	-0,042357433	0,655807933	0,740670563
SLC26A1	protein_coding	ENSG00000145217	-0,096246627	0,655773763	0,740670563
ZNRF2P1	transcribed_processed_	ENSG00000237004	0,078346403	0,656116372	0,740898904
GK	protein_coding	ENSG00000198814	-0,038650542	0,656943559	0,741792138
YIF1B	protein_coding	ENSG00000167645	0,029440931	0,657030746	0,741849744
AC087521.3	sense_overlapping	ENSG00000254409	-0,110164612	0,657790163	0,742666313
MYL12B	protein_coding	ENSG00000118680	0,030598547	0,657912704	0,742763778
TGFB3	protein_coding	ENSG00000119699	-0,104293842	0,658209801	0,74305829
PTP4A3	protein_coding	ENSG00000184489	0,059650269	0,658356321	0,743078185
CCDC189	protein_coding	ENSG00000196118	-0,076953009	0,658338433	0,743078185
AC136475.2	antisense	ENSG00000254910	-0,113650637	0,658498963	0,743180197
WDR45BP1	processed_pseudogene	ENSG00000265574	-0,111468418	0,658804701	0,743484341
TEF	protein_coding	ENSG00000167074	0,049879914	0,658939845	0,74359594
NDUFS1	protein_coding	ENSG00000023228	-0,01753974	0,659370505	0,744040991
TCHH	protein_coding	ENSG00000159450	-0,084640387	0,659625423	0,744287695
SP4	protein_coding	ENSG00000105866	0,030470615	0,659666701	0,744293324
SEC23A-AS1	lincRNA	ENSG00000258651	0,10304419	0,659763737	0,744361859
RF00019	misc_RNA	ENSG00000252965	0,111347602	0,659883462	0,744455985
AP000866.6	sense_intronic	ENSG00000279342	0,095598416	0,66008291	0,744640035
TMEM126B	protein_coding	ENSG00000171204	0,03513888	0,660379977	0,744934184
AK3P3	processed_pseudogene	ENSG00000230042	0,115062593	0,660488173	0,745015257
UVSSA	protein_coding	ENSG00000163945	-0,038460358	0,660561894	0,745057438
UVSSA	protein_coding	ENSG00000163945	-0,038460358	0,660561894	0,745057438
RNU6-890P	snRNA	ENSG00000206848	-0,099222904	0,66062339	0,745085825
AC005776.1	processed_pseudogene	ENSG00000270802	0,109096784	0,660836484	0,745253179
ST3GAL5	protein_coding	ENSG00000115525	0,0362864	0,660844445	0,745253179
DPH3	protein_coding	ENSG00000154813	-0,034597064	0,66099564	0,745382701
IRF9	protein_coding	ENSG00000213928	0,115074023	0,661197245	0,745425636
PLEKHF1	protein_coding	ENSG00000166289	0,03657294	0,661148261	0,745425636
CASD1	protein_coding	ENSG00000127995	-0,033174441	0,661215436	0,745425636
TSG101	protein_coding	ENSG00000074319	-0,033779392	0,661200771	0,745425636
PABPC1L	protein_coding	ENSG00000101104	-0,044610186	0,66118421	0,745425636
PCDHB7	protein_coding	ENSG00000113212	0,101570658	0,66142218	0,745617726
AC063965.1	sense_intronic	ENSG00000224745	0,112536113	0,66147898	0,745640774
NT5C2	protein_coding	ENSG00000076685	0,019637112	0,661782292	0,74594168
AC092343.1	antisense	ENSG00000247345	0,097594617	0,661889014	0,746020975
CCDC57	protein_coding	ENSG00000176155	0,051161565	0,662058263	0,746170732
CCDC96	protein_coding	ENSG00000173013	0,100961156	0,662133331	0,74620229

USP30	protein_coding	ENSG00000135093	0,02973864	0,662159029	0,74620229
LINC00304	lincRNA	ENSG00000180422	0,095614921	0,662424455	0,746411616
KMT2B	protein_coding	ENSG00000272333	0,048618514	0,6623941	0,746411616
C9orf116	protein_coding	ENSG00000160345	-0,068171992	0,662453956	0,746411616
AC040160.1	processed_transcript	ENSG00000262691	-0,104192471	0,6625559	0,746485471
LINC00115	lincRNA	ENSG00000225880	-0,110958182	0,66274717	0,746659954
AF233439.1	lincRNA	ENSG00000245857	0,113385321	0,663061873	0,746973471
TRAPPC13	protein_coding	ENSG00000113597	-0,033362674	0,663250908	0,747145391
AC073910.1	processed_pseudogene	ENSG00000226774	-0,106025856	0,663307878	0,747168529
ZNF568	protein_coding	ENSG00000198453	0,042598486	0,663541429	0,74739056
ACVR2B-AS1	antisense	ENSG00000229589	0,112953223	0,663737643	0,747570514
FMR1-AS1	processed_transcript	ENSG00000268066	-0,091466269	0,663783451	0,747581054
SGSM3	protein_coding	ENSG00000100359	-0,104192416	0,664066576	0,747858855
AC020904.2	transcribed_processed_	ENSG00000273654	0,109513391	0,664230087	0,748001926
AL353653.1	antisense	ENSG00000234693	0,097701619	0,664370932	0,748119459
COL4A4	protein_coding	ENSG00000081052	-0,100034283	0,664586994	0,748321671
PBX4	protein_coding	ENSG00000105717	-0,082904566	0,664679819	0,748385105
AC073352.1	lincRNA	ENSG00000272662	-0,102486967	0,664838544	0,748522728
WDR97	protein_coding	ENSG00000179698	-0,110043807	0,664932903	0,748587871
AC004890.1	processed_pseudogene	ENSG00000239719	-0,093785481	0,665104765	0,748740256
CCT2	protein_coding	ENSG00000166226	-0,022980386	0,66528649	0,748903727
AC004918.1	antisense	ENSG00000244701	-0,112361875	0,665667498	0,749291498
METTL11B	protein_coding	ENSG00000203740	0,111899238	0,66605618	0,749687863
AP002847.1	TEC	ENSG00000279138	-0,111922782	0,666385258	0,750017102
LMCD1	protein_coding	ENSG00000071282	0,056135828	0,666587014	0,750203011
GON4L	protein_coding	ENSG00000116580	-0,022706705	0,666911524	0,750527043
AC243585.2	antisense	ENSG00000277688	0,10321991	0,667020875	0,750608918
AC087386.1	lincRNA	ENSG00000258410	0,108281174	0,667233563	0,750807065
AC092803.2	lincRNA	ENSG00000260805	0,08451397	0,667318083	0,750860977
ALDH7A1	protein_coding	ENSG00000164904	0,020382428	0,667587468	0,75109673
TFAP2C	protein_coding	ENSG00000087510	0,058968986	0,667806353	0,751286726
AC093330.1	antisense	ENSG00000266844	-0,10152866	0,667878305	0,751326461
ZNF688	protein_coding	ENSG00000229809	-0,066879779	0,667940054	0,751354715
EIF4EBP2	protein_coding	ENSG00000148730	-0,022031455	0,668415755	0,751805128
GOLGA6L4	protein_coding	ENSG00000184206	-0,10373866	0,66845043	0,751805128
AL158825.2	sense_intronic	ENSG00000229587	0,104733709	0,668408162	0,751805128
SOCS5P4	processed_pseudogene	ENSG00000227536	-0,111317439	0,668560538	0,751887736
PSMC3IP	protein_coding	ENSG00000131470	-0,026786368	0,668793404	0,752108385
CASKIN2	protein_coding	ENSG00000177303	-0,04278348	0,66897079	0,752266621
THAP5P1	processed_pseudogene	ENSG00000224324	-0,101174212	0,6691296	0,752403953

RNU6-299P	snRNA	ENSG00000253032	-0,102741948	0,669220126	0,752464491
AC020891.2	lincRNA	ENSG00000259306	0,095635422	0,669494509	0,752638225
GRB2	protein_coding	ENSG00000177885	-0,016683405	0,669521425	0,752638225
UBE2N	protein_coding	ENSG00000177889	-0,0233849	0,669464264	0,752638225
CDIPT	protein_coding	ENSG00000103502	-0,030641862	0,669458929	0,752638225
ATP9B	protein_coding	ENSG00000166377	-0,034907295	0,669593214	0,752677673
KLF14	protein_coding	ENSG00000266265	-0,106782749	0,669898298	0,752979344
RBM20	protein_coding	ENSG00000203867	0,052252683	0,670132258	0,753201042
TCIM	protein_coding	ENSG00000176907	-0,100622287	0,670323602	0,75337482
DNAH1	protein_coding	ENSG00000114841	0,055707877	0,670385555	0,753403164
AC027309.2	processed_pseudogene	ENSG00000253683	0,098308074	0,670543667	0,753539566
PSMB8-AS1	lincRNA	ENSG00000204261	0,102255286	0,670633883	0,753599657
ZNF480	protein_coding	ENSG00000198464	0,026254478	0,670856114	0,753808081
ANAPC16	protein_coding	ENSG00000166295	0,031373327	0,670903992	0,75382058
LINC00313	lincRNA	ENSG00000185186	0,100235362	0,671117104	0,753999883
FAM95C	protein_coding	ENSG00000283486	0,108708549	0,671137097	0,753999883
SLC9A3R1	protein_coding	ENSG00000109062	0,033696728	0,671393333	0,754246441
FBXO7	protein_coding	ENSG00000100225	-0,01656588	0,671653026	0,754496854
RPL17P36	processed_pseudogene	ENSG00000236058	-0,106687233	0,671859839	0,754687842
SCGB1B2P	lincRNA	ENSG00000268751	-0,106203335	0,671901864	0,754693714
AC022306.2	lincRNA	ENSG00000275580	0,110555226	0,672046649	0,754773671
ALG13	protein_coding	ENSG00000101901	0,024994422	0,67204449	0,754773671
WASH3P	transcribed_unprocessed	ENSG00000185596	-0,046755378	0,672209233	0,754914931
AC084035.1	lincRNA	ENSG00000282860	0,101704268	0,672566117	0,755233017
AC064799.1	processed_pseudogene	ENSG00000212664	0,096452934	0,672533465	0,755233017
AP001347.1	antisense	ENSG00000224905	-0,106322225	0,672844471	0,755504221
ERI2	protein_coding	ENSG00000196678	0,030692178	0,672971661	0,75560567
GAPDHP38	processed_pseudogene	ENSG00000249210	0,109645894	0,673429245	0,75607805
POTEE	protein_coding	ENSG00000188219	0,103267632	0,673509291	0,756126529
C17orf49	protein_coding	ENSG00000258315	-0,1093634	0,673571903	0,756155431
AL590560.3	TEC	ENSG00000279430	0,109182614	0,673650729	0,756202532
EIF4HP1	processed_pseudogene	ENSG00000233830	-0,096728693	0,673746392	0,756261639
MACROD2	protein_coding	ENSG00000172264	0,109544963	0,673814003	0,756261639
CRLF1	protein_coding	ENSG00000006016	0,066779704	0,673802322	0,756261639
AL365217.1	processed_pseudogene	ENSG00000214558	0,108361077	0,673945288	0,756367598
KMT5AP1	processed_pseudogene	ENSG00000279730	0,102891236	0,67416992	0,756578302
AC009403.1	antisense	ENSG00000216895	0,076415788	0,67434573	0,756734197
LRRC37A4P	transcribed_unprocessed	ENSG00000214425	0,109625975	0,674473306	0,756835951
FASN	protein_coding	ENSG00000169710	-0,040506811	0,674781748	0,757140634
AC025159.1	antisense	ENSG00000257815	-0,077564583	0,674858273	0,757185075

IFT57	protein_coding	ENSG00000114446	-0,020827377	0,67499154	0,757293172
ATP2C2-AS1	antisense	ENSG00000261286	-0,061241269	0,675443562	0,757717413
RF02271	misc_RNA	ENSG00000275227	0,096374071	0,675438257	0,757717413
AC097505.1	antisense	ENSG00000272995	0,099278048	0,675625753	0,757880343
SYF2	protein_coding	ENSG00000117614	0,025049819	0,675688831	0,757909649
Z84492.1	antisense	ENSG00000271858	-0,095107625	0,675755977	0,757943513
RUSC1-AS1	antisense	ENSG00000225855	-0,10828583	0,676130924	0,758311979
CTAGE4	protein_coding	ENSG00000225932	-0,092976761	0,676158433	0,758311979
GAPDHP63	processed_pseudogene	ENSG00000218582	0,109355201	0,67629661	0,758425473
PDPK1	protein_coding	ENSG00000140992	-0,030623278	0,676456848	0,758563694
PSENEN	protein_coding	ENSG00000205155	0,055974059	0,676554916	0,758632187
RPL23AP49	transcribed_processed_	ENSG00000243422	0,106810765	0,676653578	0,758701338
RMDN2	protein_coding	ENSG00000115841	0,059826366	0,676699367	0,758711202
VDAC1P2	processed_pseudogene	ENSG00000213856	0,102920016	0,677049136	0,759061866
LRRC71	protein_coding	ENSG00000160838	-0,072148057	0,677096486	0,759073459
PSKH1	protein_coding	ENSG00000159792	0,043319829	0,677204857	0,759153455
TRMT13	protein_coding	ENSG00000122435	-0,031332262	0,677676158	0,759640269
ADGRB2	protein_coding	ENSG00000121753	0,041430126	0,67819925	0,760185081
AC109583.2	processed_pseudogene	ENSG00000273682	-0,085676216	0,678403827	0,760303097
NAP1L1	protein_coding	ENSG00000187109	-0,026063467	0,678409667	0,760303097
CXorf40A	protein_coding	ENSG00000197620	-0,035620525	0,678415748	0,760303097
PFDN4	protein_coding	ENSG00000101132	-0,036465828	0,678504298	0,760360788
CYTH2	protein_coding	ENSG00000105443	-0,025549046	0,679254336	0,761159724
STX17	protein_coding	ENSG00000136874	0,031078146	0,67933917	0,761213198
CAMTA2	protein_coding	ENSG00000108509	0,0404067	0,679554115	0,76141245
STAM	protein_coding	ENSG00000136738	-0,020353478	0,679622821	0,761447834
AL663070.1	processed_pseudogene	ENSG00000228477	0,097359868	0,6797214	0,761475088
LINC00843	processed_transcript	ENSG00000178440	0,103965728	0,679708627	0,761475088
AC139768.2	sense_intronic	ENSG00000278126	-0,104832786	0,680188713	0,761956988
SMG1P6	unprocessed_pseudoge	ENSG00000254634	-0,097527825	0,680291459	0,762030465
LINC01816	lincRNA	ENSG00000231327	-0,095445225	0,680625884	0,762363436
PPP1R14BP3	processed_pseudogene	ENSG00000179967	-0,06211819	0,680709439	0,762415389
ZNF815P	transcribed_unprocesse	ENSG00000235944	0,089898234	0,680759213	0,762429501
AC104958.2	antisense	ENSG00000272502	-0,090624929	0,681127168	0,762591747
PRDX2P3	processed_pseudogene	ENSG00000249140	-0,084017299	0,680970345	0,762591747
CDC42EP5	protein_coding	ENSG00000167617	0,091611973	0,681022372	0,762591747
SMAD6	protein_coding	ENSG00000137834	0,057494288	0,681096591	0,762591747
EZH2	protein_coding	ENSG00000106462	-0,022240524	0,681000062	0,762591747
AC026356.2	sense_intronic	ENSG00000276115	0,098142535	0,681066512	0,762591747
AC096887.1	protein_coding	ENSG00000272305	-0,094876153	0,681203983	0,762636118

AC067942.2	processed_pseudogene	ENSG00000248113	0,084673035	0,681366321	0,762776226
KRT3	protein_coding	ENSG00000186442	-0,10143917	0,681405947	0,762778953
AC098934.3	processed_pseudogene	ENSG00000235449	-0,106332041	0,681509562	0,762828531
NACA4P	transcribed_processed_	ENSG00000228224	0,103416666	0,681524622	0,762828531
AC018628.1	TEC	ENSG00000279133	0,063822594	0,681579849	0,762848715
TSTA3	protein_coding	ENSG00000104522	-0,027701014	0,681635137	0,762868965
BORCS7	protein_coding	ENSG00000166275	-0,036265376	0,681767215	0,76297515
AC034102.4	antisense	ENSG00000257553	-0,102372695	0,681918873	0,763103235
MANEA-DT	antisense	ENSG00000261366	0,094333713	0,682052107	0,763210689
TMEM92-AS1	antisense	ENSG00000251179	0,101235064	0,682117668	0,763242409
AC134682.1	antisense	ENSG00000261693	-0,106339965	0,682184622	0,763248294
AL359918.1	processed_pseudogene	ENSG00000228818	0,106253616	0,682271781	0,763248294
KMT2E	protein_coding	ENSG00000005483	0,026190968	0,682249469	0,763248294
FAM83E	protein_coding	ENSG00000105523	-0,104232296	0,682223206	0,763248294
NTSR1	protein_coding	ENSG00000101188	0,099411221	0,682358256	0,763249851
SLC25A33	protein_coding	ENSG00000171612	0,026770317	0,682384813	0,763249851
SYT17	protein_coding	ENSG00000103528	-0,099072566	0,682375755	0,763249851
AC011495.1	processed_pseudogene	ENSG00000243829	-0,099024774	0,682539482	0,763381218
ZNF629	protein_coding	ENSG00000102870	0,034101247	0,682643138	0,763455519
AC005306.1	processed_transcript	ENSG00000267283	-0,106321759	0,682976752	0,763620431
ANKRD13C	protein_coding	ENSG00000118454	0,022995514	0,682960247	0,763620431
PGD	protein_coding	ENSG00000142657	-0,022608289	0,682863474	0,763620431
STK3	protein_coding	ENSG00000104375	-0,028465388	0,682947333	0,763620431
AC027514.1	sense_intronic	ENSG00000266900	-0,102333382	0,682934308	0,763620431
SPATA25	protein_coding	ENSG00000149634	0,10678169	0,683153606	0,7637349
AGA	protein_coding	ENSG00000038002	-0,026620139	0,683129133	0,7637349
RPL24P4	processed_pseudogene	ENSG00000181524	-0,068634843	0,683486319	0,764065209
LINC00894	antisense	ENSG00000235703	0,094668718	0,683666131	0,764190857
MAPK3	protein_coding	ENSG00000102882	-0,027793033	0,683673234	0,764190857
AC034236.1	processed_pseudogene	ENSG00000185641	-0,085678266	0,684006556	0,764521769
HNRNPA1P35	processed_pseudogene	ENSG00000225695	-0,092184644	0,684121223	0,764608266
AL163051.2	sense_intronic	ENSG00000276182	-0,102794883	0,684160846	0,764610884
PYROXD2	protein_coding	ENSG00000119943	0,063900832	0,684510448	0,764942264
CHRNA7	protein_coding	ENSG00000175344	-0,098394996	0,684531951	0,764942264
AL021877.2	lincRNA	ENSG00000280356	0,097991124	0,684629672	0,765009783
FTH1P23	processed_pseudogene	ENSG00000242960	0,091616161	0,684767485	0,765122091
AC104692.2	processed_pseudogene	ENSG00000270405	-0,10308983	0,684963641	0,765299574
AF129075.2	antisense	ENSG00000273254	0,105872179	0,685068411	0,765374938
KRT8P32	processed_pseudogene	ENSG00000250221	0,099059768	0,68529572	0,76558719
KATNA1	protein_coding	ENSG00000186625	-0,026478378	0,685568693	0,76580872

AC083964.2	TEC	ENSG00000279852	-0,104043617	0,685538105	0,76580872
RGL2	protein_coding	ENSG00000237441	-0,024781286	0,685638321	0,765844786
AL591135.1	processed_pseudogene	ENSG00000218418	-0,104823568	0,685870354	0,766062243
CADM4	protein_coding	ENSG00000105767	0,064889894	0,685922941	0,766079259
AC012640.2	antisense	ENSG00000259802	0,065287122	0,686377613	0,766545323
CGN	protein_coding	ENSG00000143375	-0,049816708	0,686530649	0,766674486
FKBP9P1	transcribed_unprocesse	ENSG00000176826	0,104440721	0,68675721	0,766885739
ALKBH6	protein_coding	ENSG00000239382	-0,088588844	0,686901138	0,767004701
AC102953.2	lincRNA	ENSG00000273230	-0,084672771	0,687085454	0,767168744
FIGNL2	protein_coding	ENSG00000261308	0,087728935	0,687532807	0,767584665
MGMT	protein_coding	ENSG00000170430	-0,074489486	0,687532287	0,767584665
AC104076.1	antisense	ENSG00000236153	-0,091934575	0,6876605	0,767658231
STARD3NL	protein_coding	ENSG00000010270	-0,026163744	0,687673558	0,767658231
PGBD4	protein_coding	ENSG00000182405	-0,042604433	0,687731681	0,767681331
RPL21P119	processed_pseudogene	ENSG00000220793	0,104341795	0,687877681	0,767802517
AC007041.1	processed_pseudogene	ENSG00000235411	-0,102253155	0,687916191	0,767803716
EIF5AL1	protein_coding	ENSG00000253626	-0,074324689	0,687973775	0,767826202
POU5F1P5	processed_pseudogene	ENSG00000236375	-0,10285585	0,688144292	0,767974721
FABP6	protein_coding	ENSG00000170231	0,096872563	0,688481601	0,768309355
UXT-AS1	antisense	ENSG00000267064	0,102248264	0,688586493	0,768384601
COX6A1P2	processed_pseudogene	ENSG00000226976	0,080785857	0,688684926	0,768452632
PSMB7	protein_coding	ENSG00000136930	-0,020755771	0,689042011	0,768767428
UBP1	protein_coding	ENSG00000153560	-0,021078272	0,689011184	0,768767428
NUTM2A-AS1	antisense	ENSG00000223482	0,025921356	0,689084185	0,768772663
AL590139.1	processed_pseudogene	ENSG00000179101	0,08764504	0,689202309	0,768814412
RPS3AP26	processed_pseudogene	ENSG00000214389	0,075721818	0,689265926	0,768814412
PPT2-EGFL8	protein_coding	ENSG00000258388	0,100062129	0,689309031	0,768814412
WDR83OS	protein_coding	ENSG00000105583	-0,029892457	0,689307734	0,768814412
AC025031.3	sense_intronic	ENSG00000274591	-0,100079656	0,689265107	0,768814412
MIR4527HG	lincRNA	ENSG00000267761	0,097039317	0,689560844	0,768969819
RUNDC3B	protein_coding	ENSG00000105784	0,083816634	0,689525704	0,768969819
RPS6KA5	protein_coding	ENSG00000100784	-0,036171614	0,689555289	0,768969819
ZDHHC14	protein_coding	ENSG00000175048	-0,056209817	0,689658436	0,769036836
ATAT1	protein_coding	ENSG00000137343	0,041201338	0,690060892	0,769443781
AC234772.2	lincRNA	ENSG00000269902	-0,081185684	0,690697566	0,770111828
AC073896.2	antisense	ENSG00000257303	0,086028464	0,690777985	0,770117763
FAM229B	protein_coding	ENSG00000203778	0,040208379	0,690770827	0,770117763
PPP1R16A	protein_coding	ENSG00000160972	-0,039005668	0,690827633	0,770131251
AL590133.2	antisense	ENSG00000259357	-0,085945086	0,691060869	0,770307523
AL450405.1	processed_pseudogene	ENSG00000230202	0,057344539	0,691060394	0,770307523

FAXDC2	protein_coding	ENSG00000170271	-0,100228482	0,69109919	0,770308373
ESF1	protein_coding	ENSG00000089048	-0,027467937	0,691282954	0,770471329
RPL12P12	processed_pseudogene	ENSG00000236992	0,091512383	0,691332881	0,770485105
HHIPL2	protein_coding	ENSG00000143512	0,100177604	0,691439029	0,770519668
AC011944.2	TEC	ENSG00000279235	0,09739629	0,69141952	0,770519668
AC090061.1	lincRNA	ENSG00000247363	0,083764966	0,691521191	0,770522903
FCHSD1	protein_coding	ENSG00000197948	-0,0322005	0,691526137	0,770522903
CDK3	protein_coding	ENSG00000250506	-0,096249595	0,691554637	0,770522903
PDXDC1	protein_coding	ENSG00000179889	-0,018755076	0,691703766	0,770647196
PDXDC1	protein_coding	ENSG00000179889	-0,018755076	0,691703766	0,770647196
ZNF710-AS1	antisense	ENSG00000259291	-0,099884445	0,691790913	0,770702424
AC004461.2	sense_intronic	ENSG00000281530	-0,099480332	0,692080339	0,770982986
TMEM184B	protein_coding	ENSG00000198792	0,029286658	0,692200246	0,771032805
CROCCP2	transcribed_unprocesse	ENSG00000215908	0,034103924	0,692182717	0,771032805
AC073073.2	antisense	ENSG00000272604	0,088327479	0,692476024	0,771139125
SNRPFP1	processed_pseudogene	ENSG00000231878	0,086992478	0,692411137	0,771139125
MPRIPP1	processed_pseudogene	ENSG00000214820	-0,102403213	0,692483686	0,771139125
ENPP3	protein_coding	ENSG00000154269	0,093222144	0,692357526	0,771139125
ALDH9A1	protein_coding	ENSG00000143149	0,019305777	0,692405362	0,771139125
HADHB	protein_coding	ENSG00000138029	-0,02014229	0,692631465	0,771261813
AC062037.2	antisense	ENSG00000273196	0,103108211	0,69284628	0,771291642
AC079922.1	processed_pseudogene	ENSG00000231747	0,101450399	0,692814143	0,771291642
MAT1A	protein_coding	ENSG00000151224	0,102680503	0,692799823	0,771291642
GDF11	protein_coding	ENSG00000135414	0,030870704	0,692827406	0,771291642
AC244093.4	sense_intronic	ENSG00000277589	0,101238638	0,69283667	0,771291642
GPR83	protein_coding	ENSG00000123901	0,097239187	0,692999016	0,7714198
AK4P1	processed_pseudogene	ENSG00000263535	0,096180941	0,693200179	0,771601849
AC092159.2	antisense	ENSG00000233296	-0,091299706	0,693436181	0,771822655
BDH2	protein_coding	ENSG00000164039	0,07128121	0,693712613	0,772088436
NCKAP1	protein_coding	ENSG00000061676	0,020173337	0,693827335	0,772174217
DHFRP1	processed_pseudogene	ENSG00000188985	-0,069948352	0,693891259	0,772203458
ARFIP2	protein_coding	ENSG00000132254	0,022867934	0,694105845	0,772400351
AC024940.6	3prime_overlapping_nc	ENSG00000275097	0,093451602	0,694316315	0,772592644
AC010343.1	processed_pseudogene	ENSG00000240376	-0,077970582	0,694493017	0,772747343
ZNF224	protein_coding	ENSG00000267680	0,030630805	0,694533658	0,772750641
AC107075.1	unprocessed_pseudoge	ENSG00000277998	-0,09927623	0,695096046	0,773334413
AL031282.1	transcribed_processed_	ENSG00000227775	-0,09408658	0,695176143	0,773381574
TMBIM6	protein_coding	ENSG00000139644	0,013051469	0,695304883	0,773482842
AL121601.1	lincRNA	ENSG00000232412	-0,101174219	0,695515841	0,773675556
RPL23AP2	processed_pseudogene	ENSG00000225067	0,08813665	0,695704973	0,773843972

AP005119.2	processed_pseudogene	ENSG00000265417	-0,093829942	0,695778244	0,773883503
CSPG5	protein_coding	ENSG00000114646	-0,100949647	0,695997727	0,774085645
AC004832.5	sense_intronic	ENSG00000273350	-0,084262795	0,696210813	0,774280652
TEKT4	protein_coding	ENSG00000163060	-0,093361457	0,696384719	0,774432066
AL121574.1	antisense	ENSG00000225945	0,100584352	0,696480702	0,774496812
ITM2A	protein_coding	ENSG00000078596	0,027056278	0,696718565	0,774719315
ARHGEF17	protein_coding	ENSG00000110237	-0,033129051	0,696844981	0,774817877
TENM3-AS1	antisense	ENSG00000177822	0,043500844	0,697359876	0,775306324
PPA1	protein_coding	ENSG00000180817	-0,02591942	0,697351416	0,775306324
GRK5-IT1	sense_intronic	ENSG00000228485	0,084780285	0,69740654	0,775316176
SETP8	processed_pseudogene	ENSG00000213526	-0,089092225	0,697452579	0,775325333
ZNF330	protein_coding	ENSG00000109445	-0,018203247	0,697567249	0,775410779
PDE4B	protein_coding	ENSG00000184588	-0,092277906	0,697625004	0,775432952
TTLL1	protein_coding	ENSG00000100271	0,048653762	0,697736382	0,775472699
AC023509.3	antisense	ENSG00000270175	-0,095204584	0,697880554	0,775590905
AP003059.1	lincRNA	ENSG00000254731	0,093311205	0,697997403	0,775636709
AIF1L	protein_coding	ENSG00000126878	0,095787955	0,697975908	0,775636709
HNRNPCP3	processed_pseudogene	ENSG00000259419	0,096316221	0,698046002	0,775648689
DYNC1LI1	protein_coding	ENSG00000144635	-0,02083561	0,698170644	0,77574516
CTAGE8	protein_coding	ENSG00000244693	-0,085936446	0,69834014	0,775891455
PSMD6-AS2	antisense	ENSG00000239653	0,083100378	0,698499984	0,776027013
LINC00562	lincRNA	ENSG00000260388	-0,085454127	0,698786357	0,776303119
CTSH	protein_coding	ENSG00000103811	0,090835113	0,699137842	0,776651527
AL080317.2	lincRNA	ENSG00000271789	0,099786043	0,69921495	0,776695116
AC012510.1	antisense	ENSG00000273466	-0,090325189	0,699503115	0,776973133
CHCHD2P9	processed_pseudogene	ENSG00000186940	-0,094669144	0,699656279	0,777101174
CHORDC1	protein_coding	ENSG00000110172	-0,029903564	0,699769915	0,777185301
ZNF548	protein_coding	ENSG00000188785	0,027619231	0,699916962	0,777306524
SRP14	protein_coding	ENSG00000140319	0,024368938	0,700286638	0,777674966
NIT1	protein_coding	ENSG00000158793	-0,02438565	0,700344202	0,777696783
CTF1	protein_coding	ENSG00000150281	-0,074260339	0,700616573	0,777957117
PWWP2A	protein_coding	ENSG00000170234	0,027550818	0,700742451	0,778054768
GMPR2	protein_coding	ENSG00000100938	0,019269945	0,701174372	0,778492198
AP000941.1	antisense	ENSG00000255176	-0,09515023	0,70130709	0,77851312
METTL15P1	processed_pseudogene	ENSG00000174912	-0,09260673	0,701274915	0,77851312
CEACAMP6	unprocessed_pseudoge	ENSG00000238092	0,080694803	0,701255164	0,77851312
SNHG10	antisense	ENSG00000247092	-0,049613616	0,701498834	0,778683828
HSPA8P15	processed_pseudogene	ENSG00000219395	-0,0880128	0,701543871	0,778691677
AC020910.4	lincRNA	ENSG00000274104	0,081207248	0,701666941	0,778786133
TAF6	protein_coding	ENSG00000106290	-0,025141854	0,70174294	0,778828339

ADGRF4	protein_coding	ENSG00000153294	-0,091211175	0,70185907	0,778915077
ZNF747	protein_coding	ENSG00000169955	0,042720677	0,701938482	0,778961058
UTP4	protein_coding	ENSG00000141076	-0,02939979	0,702218086	0,779229181
AP001767.4	TEC	ENSG00000279900	-0,092922447	0,70250776	0,779508449
PTPN23	protein_coding	ENSG00000076201	0,031766517	0,702585944	0,779553028
OPRL1	protein_coding	ENSG00000125510	0,055250058	0,702651219	0,77958328
AL138963.2	processed_pseudogene	ENSG00000270381	0,078917053	0,70273984	0,779639429
TAS2R19	protein_coding	ENSG00000212124	0,098587388	0,70302653	0,779915304
PROB1	protein_coding	ENSG00000228672	-0,083117048	0,703363343	0,78024675
RAB24	protein_coding	ENSG00000169228	0,066774135	0,703527566	0,780386716
AP003392.6	antisense	ENSG00000272186	-0,097790408	0,704854824	0,781774413
IFI27L1	protein_coding	ENSG00000165948	-0,035208966	0,70506256	0,781962533
GTF3C4	protein_coding	ENSG00000125484	0,020077063	0,70517782	0,782048074
WNT7A	protein_coding	ENSG00000154764	0,064181584	0,705461619	0,782320508
AC055811.3	lincRNA	ENSG00000266498	0,088281821	0,705665328	0,782504102
IFI27L2	protein_coding	ENSG00000119632	0,038148792	0,705789661	0,782599662
BCLAF1P2	processed_pseudogene	ENSG00000279800	0,085957471	0,705949501	0,782649961
RHOA	protein_coding	ENSG00000067560	-0,01966979	0,705944339	0,782649961
SLC22A23	protein_coding	ENSG00000137266	-0,076719035	0,705899175	0,782649961
AC018809.2	antisense	ENSG00000269982	0,097956696	0,706025342	0,782691734
RBM45	protein_coding	ENSG00000155636	-0,023308811	0,706066798	0,782695386
AC002553.1	antisense	ENSG00000227782	0,093750689	0,706249759	0,782855891
AC007620.2	antisense	ENSG00000242539	-0,097137105	0,706348646	0,782880883
RIPOR1	protein_coding	ENSG00000039523	0,037730696	0,706311021	0,782880883
ACOX2	protein_coding	ENSG00000168306	0,035027357	0,706543317	0,78305433
AC080129.2	antisense	ENSG00000232874	-0,096253479	0,706603464	0,783078676
NEURL4	protein_coding	ENSG00000215041	-0,038719072	0,70675431	0,783203528
AC025857.2	sense_intronic	ENSG00000269899	0,093231961	0,706845208	0,783261939
AC004076.2	antisense	ENSG00000276449	-0,097321255	0,707032341	0,783402295
CPT1C	protein_coding	ENSG00000169169	0,09824411	0,707048263	0,783402295
SNX10	protein_coding	ENSG00000086300	0,092238891	0,707138761	0,783460241
SNX18P3	transcribed_unprocesse	ENSG00000225345	0,084482099	0,707181716	0,783465511
AC138150.1	antisense	ENSG00000224505	0,096911136	0,707336054	0,78356475
RN7SKP230	misc_RNA	ENSG00000202512	-0,096834394	0,707347701	0,78356475
FAM228B	protein_coding	ENSG00000219626	0,062287207	0,707445192	0,783630422
AC114495.2	processed_pseudogene	ENSG00000229447	-0,093715086	0,707803198	0,783942305
AP001992.1	transcribed_processed_	ENSG00000241170	0,082046043	0,707766564	0,783942305
RNU6-9	snRNA	ENSG00000207507	0,097878573	0,707881565	0,783986765
GTSE1-DT	lincRNA	ENSG00000277232	-0,089093542	0,707966397	0,783996048
MAPK6	protein_coding	ENSG00000069956	-0,019185072	0,707940089	0,783996048

RPL12P1	processed_pseudogene	ENSG00000204194	0,078359312	0,708227649	0,784243012
MAVS	protein_coding	ENSG00000088888	0,031996803	0,708413992	0,784364661
GPN3	protein_coding	ENSG00000111231	-0,025920751	0,70838492	0,784364661
HMGA1P2	processed_pseudogene	ENSG00000248641	-0,093083382	0,708519169	0,784438767
AURKC	protein_coding	ENSG00000105146	-0,091996305	0,708806269	0,784714271
LINC00173	processed_transcript	ENSG00000196668	-0,095994834	0,709296058	0,785171748
KIRREL1-IT1	sense_intronic	ENSG00000226520	0,093925147	0,709261928	0,785171748
AL353807.2	antisense	ENSG00000232519	-0,096328909	0,709631249	0,7855004
AC138969.2	unprocessed_pseudoge	ENSG00000227827	-0,095895837	0,70969185	0,785525086
EML6	protein_coding	ENSG00000214595	0,051261297	0,709817788	0,785622083
R3HCC1L	protein_coding	ENSG00000166024	0,022477466	0,709956614	0,785733334
UST	protein_coding	ENSG00000111962	0,031540701	0,710029179	0,785771244
MAP1A	protein_coding	ENSG00000166963	-0,066671574	0,710154499	0,785867529
AC104109.4	processed_transcript	ENSG00000273345	0,088577739	0,710392491	0,786088481
ARAP1	protein_coding	ENSG00000186635	-0,034203359	0,710775627	0,786431734
CASTOR2	protein_coding	ENSG00000274070	-0,062102031	0,710779378	0,786431734
LINC02076	lincRNA	ENSG00000220161	0,088449245	0,710970475	0,786456129
ZNF852	protein_coding	ENSG00000178917	0,050983715	0,710872305	0,786456129
RAD54L2	protein_coding	ENSG00000164080	0,024884997	0,710993152	0,786456129
RALY	protein_coding	ENSG00000125970	0,022172235	0,710888675	0,786456129
ZBTB21	protein_coding	ENSG00000173276	0,021260517	0,710949147	0,786456129
SLC30A5	protein_coding	ENSG00000145740	0,017546869	0,711213775	0,786657742
TLE1P1	processed_pseudogene	ENSG00000228158	0,093627577	0,711674148	0,787124503
WSB2	protein_coding	ENSG00000176871	0,011669256	0,712067863	0,787517491
AC020658.3	sense_intronic	ENSG00000260648	0,096451484	0,712133341	0,78754744
AL606491.1	lincRNA	ENSG00000225643	-0,092048878	0,712283309	0,787630813
ADCY7	protein_coding	ENSG00000121281	0,086726857	0,712285535	0,787630813
AL121718.1	lincRNA	ENSG00000230309	0,094285335	0,712338539	0,787646958
PTMAP4	processed_pseudogene	ENSG00000231503	-0,053617581	0,712430826	0,7876864
ZDHHC3	protein_coding	ENSG00000163812	0,01469175	0,71245102	0,7876864
BEGAIN	protein_coding	ENSG00000183092	-0,092718786	0,712525684	0,787726487
AC145124.1	antisense	ENSG00000255495	-0,085774693	0,712590108	0,787755248
LINC00648	lincRNA	ENSG00000259129	0,076253265	0,713323633	0,788523645
RNF150	protein_coding	ENSG00000170153	-0,073690881	0,713386226	0,788550337
ATPAF1	protein_coding	ENSG00000123472	-0,016720896	0,713733434	0,788891611
AC105429.1	sense_intronic	ENSG00000276337	-0,088156312	0,71385134	0,788979414
AC092667.1	antisense	ENSG00000230393	-0,080790102	0,714173507	0,789292954
AC016026.1	processed_transcript	ENSG00000093100	-0,094915263	0,714323148	0,789415796
AC124242.1	bidirectional_promoter_	ENSG00000245281	0,095370406	0,714367021	0,789421746
ZNF658	protein_coding	ENSG00000274349	0,037734673	0,714735458	0,789757456

KRT18P59	transcribed_processed_	ENSG00000187686	-0,081941646	0,714747826	0,789757456
RPL41P2	processed_pseudogene	ENSG00000256338	0,084706269	0,71486877	0,789848541
AL390879.1	transcribed_unprocesse	ENSG00000234062	-0,073271348	0,71519535	0,790166808
NUTM2B-AS1	antisense	ENSG00000225484	0,029584234	0,715325482	0,790268011
BISPR	lincRNA	ENSG00000282851	-0,094475759	0,715380619	0,790277018
MED28P1	processed_pseudogene	ENSG00000214875	-0,08912816	0,715564822	0,790277018
ZFP3	protein_coding	ENSG00000180787	0,040571909	0,715512216	0,790277018
AIDA	protein_coding	ENSG00000186063	0,020418332	0,715543003	0,790277018
POLR1D	protein_coding	ENSG00000186184	-0,021034664	0,71547747	0,790277018
BNIPL	protein_coding	ENSG00000163141	-0,09362791	0,715421362	0,790277018
AL451165.2	antisense	ENSG00000272288	0,072565793	0,715741767	0,790429876
AC141557.1	unprocessed_pseudoge	ENSG00000256673	-0,09054136	0,715791023	0,79044171
PHC1	protein_coding	ENSG00000111752	-0,037659336	0,71639471	0,791065764
AC004908.2	sense_intronic	ENSG00000272812	-0,089839971	0,716553004	0,791197961
AC010900.1	processed_pseudogene	ENSG00000232479	-0,092899331	0,717073806	0,791687773
NBPF2P	unprocessed_pseudoge	ENSG00000227001	-0,092967916	0,717073234	0,791687773
YLPM1	protein_coding	ENSG00000119596	-0,025169443	0,717154779	0,791734553
PTGR1	protein_coding	ENSG00000106853	-0,025189834	0,717214989	0,791758406
ORMDL2	protein_coding	ENSG00000123353	-0,027203102	0,717577254	0,792115688
RBPMS2	protein_coding	ENSG00000166831	0,057858076	0,717617104	0,792117044
GYS2	protein_coding	ENSG00000111713	0,076005868	0,717700129	0,792166056
AL133338.1	antisense	ENSG00000260000	-0,070682599	0,718052261	0,792406148
AQP11	protein_coding	ENSG00000178301	0,05242715	0,718072193	0,792406148
CUL5	protein_coding	ENSG00000166266	0,021766966	0,718037235	0,792406148
EIF3F	protein_coding	ENSG00000175390	-0,017335444	0,718035445	0,792406148
WDR43	protein_coding	ENSG00000163811	-0,018630136	0,718195294	0,792499353
POM121C	protein_coding	ENSG00000272391	0,035473357	0,718514784	0,792809244
ZNF181	protein_coding	ENSG00000197841	0,027430234	0,718694057	0,79295646
CHMP2A	protein_coding	ENSG00000130724	-0,022507362	0,718725528	0,79295646
AC103724.3	antisense	ENSG00000261449	-0,086205928	0,718770301	0,792963201
AIFM1	protein_coding	ENSG00000156709	-0,016765911	0,718852776	0,793011534
SPSB3	protein_coding	ENSG00000162032	0,032117057	0,718949451	0,793075526
HYPK	protein_coding	ENSG00000242028	0,083313236	0,719443216	0,793577517
ATP5PBP1	processed_pseudogene	ENSG00000224451	0,082951306	0,719550353	0,793653011
FMR1-IT1	sense_intronic	ENSG00000236337	-0,085297253	0,719604174	0,793669693
AC010326.3	sense_intronic	ENSG00000269867	-0,087725502	0,719807949	0,793851752
AL356019.2	lincRNA	ENSG00000258768	0,073337705	0,720092878	0,794123288
TULP4	protein_coding	ENSG00000130338	0,019205908	0,720220403	0,794135824
PSMC1	protein_coding	ENSG00000100764	-0,01915129	0,720203263	0,794135824
AL031708.1	protein_coding	ENSG00000261732	-0,091566187	0,720147007	0,794135824

DMAP1	protein_coding	ENSG00000178028	0,028275902	0,720417562	0,794310514
TIGD4	protein_coding	ENSG00000169989	0,092494798	0,72054495	0,794408264
RTL3	protein_coding	ENSG00000179300	0,093216956	0,720861707	0,794714771
AP001324.1	processed_pseudogene	ENSG00000227615	-0,074833941	0,720981062	0,794803632
NSUN3	protein_coding	ENSG00000178694	-0,026316179	0,721271803	0,795081408
LINC02104	lincRNA	ENSG00000271334	-0,083404603	0,72136589	0,795142387
WWC3	protein_coding	ENSG00000047644	-0,027569782	0,721561954	0,795315761
BTBD1	protein_coding	ENSG00000064726	0,018217661	0,72162067	0,795337737
ELOCP19	processed_pseudogene	ENSG00000241975	0,092941039	0,721734585	0,795392102
AC008429.2	processed_pseudogene	ENSG00000253785	-0,092281347	0,721747558	0,795392102
EEF1A1P22	processed_pseudogene	ENSG00000259612	0,089786354	0,721800567	0,795407782
AL031667.3	sense_intronic	ENSG00000273951	-0,089192592	0,722105077	0,795700593
NOVA2	protein_coding	ENSG00000104967	-0,087774254	0,72220581	0,795768839
ZNF493	protein_coding	ENSG00000196268	0,092656364	0,72231068	0,795841636
AC142472.1	lincRNA	ENSG00000276728	0,084926364	0,722668085	0,796172504
DHX32	protein_coding	ENSG00000089876	-0,016648107	0,722688615	0,796172504
DCTN4	protein_coding	ENSG00000132912	-0,013967257	0,722814202	0,796229941
RFNG	protein_coding	ENSG00000169733	-0,030029501	0,722818395	0,796229941
AC117503.3	TEC	ENSG00000279953	-0,075860993	0,722987809	0,79637379
ITGB5	protein_coding	ENSG00000082781	0,020699226	0,723070401	0,796421992
PANO1	protein_coding	ENSG00000274897	-0,07728017	0,72327456	0,796604083
AP000845.1	lincRNA	ENSG00000263884	-0,081584494	0,723463696	0,796726924
CYP2D6	protein_coding	ENSG00000100197	-0,090039379	0,723463785	0,796726924
CYP2D6	protein_coding	ENSG00000100197	-0,090039379	0,723463785	0,796726924
CYP2D6	protein_coding	ENSG00000100197	-0,090039379	0,723463785	0,796726924
RARA-AS1	antisense	ENSG00000265666	0,083106909	0,723924705	0,797148916
FN3K	protein_coding	ENSG00000167363	-0,08757073	0,723919075	0,797148916
BTRC	protein_coding	ENSG00000166167	-0,019089133	0,724049942	0,797244017
LINC02434	lincRNA	ENSG00000248370	-0,074305861	0,724457359	0,797649798
AC010809.2	antisense	ENSG00000259408	0,091267743	0,724646083	0,79781476
NIPSNAP1	protein_coding	ENSG00000184117	-0,023579713	0,724788038	0,797928216
PCF11	protein_coding	ENSG00000165494	-0,025276571	0,724895543	0,798003735
RSPH6A	protein_coding	ENSG00000104941	0,078270832	0,725224067	0,798322542
EEF2	protein_coding	ENSG00000167658	-0,009322631	0,725278321	0,798339417
STIM2-AS1	antisense	ENSG00000240005	0,080129699	0,725391792	0,798421469
MAP9	protein_coding	ENSG00000164114	0,028606565	0,72575845	0,798739312
PDK4	protein_coding	ENSG00000004799	-0,088961973	0,725752687	0,798739312
TSBP1-AS1	antisense	ENSG00000225914	0,073801996	0,725896566	0,798848451
SUMO1	protein_coding	ENSG00000116030	0,022430384	0,726172466	0,7991092
CASC2	antisense	ENSG00000177640	0,078485018	0,726782542	0,799425551

SNHG28	lincRNA	ENSG00000188004	-0,071336663	0,726730768	0,799425551
AC012435.2	lincRNA	ENSG00000261775	-0,090479492	0,726810739	0,799425551
UQCRFS1P1	processed_pseudogene	ENSG00000226085	0,08349576	0,726771416	0,799425551
SETP14	processed_pseudogene	ENSG00000240489	0,062392801	0,726670839	0,799425551
SEZ6L2	protein_coding	ENSG00000174938	0,03330796	0,726542212	0,799425551
RSL1D1	protein_coding	ENSG00000171490	-0,020192527	0,726625128	0,799425551
SHROOM2	protein_coding	ENSG00000146950	-0,040865788	0,726711204	0,799425551
AC134407.3	TEC	ENSG00000279880	-0,089378362	0,726726085	0,799425551
PMS2P6	unprocessed_pseudoge	ENSG00000174384	0,078071436	0,726884438	0,79946374
AC243960.2	processed_pseudogene	ENSG00000268034	-0,090566666	0,72702682	0,799577462
TRAPPC5	protein_coding	ENSG00000181029	-0,080282155	0,727552563	0,800112765
AC110056.1	processed_pseudogene	ENSG00000255450	0,090608925	0,727867216	0,800415881
SEMA7A	protein_coding	ENSG00000138623	-0,028149233	0,728277	0,800823573
ATP13A1	protein_coding	ENSG00000105726	-0,02425815	0,729132593	0,801721411
AC021087.1	antisense	ENSG00000248925	0,074384985	0,729207029	0,801728116
INPP5K	protein_coding	ENSG00000132376	0,021223883	0,72921687	0,801728116
AC135279.2	processed_pseudogene	ENSG00000256293	0,089467096	0,730197483	0,802745955
STIP1P3	processed_pseudogene	ENSG00000225536	-0,076162619	0,730295008	0,802745955
AC026877.1	processed_pseudogene	ENSG00000240809	-0,083424182	0,730228434	0,802745955
ATXN2	protein_coding	ENSG00000204842	0,01496829	0,730421237	0,802837062
STAB2	protein_coding	ENSG00000136011	0,084368757	0,730888621	0,803307733
CHRD	protein_coding	ENSG00000090539	-0,088858237	0,730987343	0,803373186
STRADA	protein_coding	ENSG00000266173	0,072210918	0,731055269	0,803404788
LINC02593	lincRNA	ENSG00000223764	-0,087982919	0,731147998	0,803463644
ULK4	protein_coding	ENSG00000168038	0,028020176	0,731237844	0,803476277
MAP10	protein_coding	ENSG00000212916	-0,048305929	0,731224181	0,803476277
RFXANK	protein_coding	ENSG00000064490	-0,029149328	0,731334207	0,803539111
NBR1	protein_coding	ENSG00000188554	-0,01080494	0,731420566	0,803590948
HNRNPKP2	processed_pseudogene	ENSG00000227347	-0,08632268	0,731727409	0,803832236
WDR93	protein_coding	ENSG00000140527	0,088883125	0,731757761	0,803832236
POU4F1	protein_coding	ENSG00000152192	-0,067272142	0,73173969	0,803832236
RF00019	misc_RNA	ENSG00000252759	0,088648805	0,731827372	0,80385452
RPL26P6	processed_pseudogene	ENSG00000229659	0,089351713	0,73189037	0,80385452
PHKA1P1	processed_pseudogene	ENSG00000232882	0,085331236	0,731895627	0,80385452
AC006116.8	antisense	ENSG00000267549	0,077259769	0,731976421	0,803861046
ZNF780A	protein_coding	ENSG00000197782	-0,021668773	0,731979956	0,803861046
DYNC1I2P1	processed_pseudogene	ENSG00000225137	0,071207872	0,732184129	0,803999169
TMEM170A	protein_coding	ENSG00000166822	-0,024842993	0,732167326	0,803999169
RNF126	protein_coding	ENSG00000070423	-0,024215166	0,732532972	0,804339165
MAATS1	protein_coding	ENSG00000183833	0,079244186	0,732609481	0,80438011

NFX1	protein_coding	ENSG00000086102	-0,01835442	0,732667771	0,804401049
AC118553.1	antisense	ENSG00000228084	-0,077929184	0,732912559	0,804626731
RN7SL689P	misc_RNA	ENSG00000263432	0,081372461	0,733192489	0,804844712
AC025198.1	processed_pseudogene	ENSG00000266501	0,077041765	0,733228837	0,804844712
CTSK	protein_coding	ENSG00000143387	-0,071685185	0,733159002	0,804844712
AL132655.1	lincRNA	ENSG00000268333	-0,085890559	0,73331993	0,804901624
RNU6ATAC24P	snRNA	ENSG00000252620	0,075310525	0,733384734	0,804929678
MYD88	protein_coding	ENSG00000172936	0,05700857	0,733452801	0,80496131
JAGN1	protein_coding	ENSG00000171135	0,024089212	0,733741823	0,805206287
SNORA55	snoRNA	ENSG00000201457	-0,085723893	0,733754535	0,805206287
AC107032.1	processed_pseudogene	ENSG00000243071	-0,087552396	0,734063052	0,805501749
CPEB1-AS1	antisense	ENSG00000259462	0,088035201	0,734218885	0,805586547
SSBP3	protein_coding	ENSG00000157216	-0,026089177	0,734322097	0,805656693
AC078819.1	processed_pseudogene	ENSG00000216285	-0,087548443	0,734438435	0,80574123
TUBB4A	protein_coding	ENSG00000104833	-0,087584948	0,734606787	0,80588282
SCART1	protein_coding	ENSG00000214279	0,053443471	0,735021272	0,806294394
SAP30L-AS1	antisense	ENSG00000245275	0,087672556	0,735107076	0,806345392
LEF1-AS1	processed_transcript	ENSG00000232021	-0,077320417	0,735311135	0,806482961
OXNAD1	protein_coding	ENSG00000154814	0,021749261	0,735557668	0,806710217
AC074212.1	processed_transcript	ENSG00000259605	-0,077486564	0,735862695	0,806997374
SEPT7	protein_coding	ENSG00000122545	-0,019650394	0,73589819	0,806997374
AC008105.3	antisense	ENSG00000267121	-0,086765622	0,735938796	0,806998755
ENPP1	protein_coding	ENSG00000197594	0,025672163	0,736123669	0,807158324
AC100814.1	lincRNA	ENSG00000272010	-0,081289921	0,736333569	0,80730216
INHA	protein_coding	ENSG00000123999	-0,083547983	0,736331201	0,80730216
RALGAPB	protein_coding	ENSG00000170471	-0,017362671	0,736492177	0,807432892
TMEM243	protein_coding	ENSG00000135185	0,031522495	0,736881807	0,807816872
AP005205.3	TEC	ENSG00000279479	0,078417631	0,737149608	0,808067261
LDHAP3	processed_pseudogene	ENSG00000236090	-0,086179437	0,737290431	0,808178437
AC015726.1	lincRNA	ENSG00000270091	0,086958771	0,737448349	0,80821212
EIF2A	protein_coding	ENSG00000144895	-0,020515398	0,737470677	0,80821212
TRMT1	protein_coding	ENSG00000104907	-0,021648089	0,737478783	0,80821212
OR7E53P	unprocessed_pseudogene	ENSG00000239978	-0,072908465	0,737440646	0,80821212
DTNBP1	protein_coding	ENSG00000047579	0,027514621	0,737601311	0,808300351
HS1BP3	protein_coding	ENSG00000118960	0,025859158	0,73770575	0,808300351
ZNF684	protein_coding	ENSG00000117010	-0,039078146	0,737716932	0,808300351
EYA2	protein_coding	ENSG00000064655	-0,084688729	0,737706251	0,808300351
LAMB4	protein_coding	ENSG00000091128	-0,084851697	0,737806572	0,808355384
PSD	protein_coding	ENSG00000059915	0,086866034	0,737972482	0,80849397
RPL26P19	processed_pseudogene	ENSG00000226221	-0,054588049	0,738054946	0,808541125

KANK1	protein_coding	ENSG00000107104	0,055304954	0,738359101	0,808810768
TPD52L2	protein_coding	ENSG00000101150	0,013810841	0,738379952	0,808810768
ZNF800	protein_coding	ENSG00000048405	0,021173962	0,738471297	0,808867626
RBM15	protein_coding	ENSG00000162775	-0,016588507	0,738570333	0,808932902
FKBP8	protein_coding	ENSG00000105701	0,027255112	0,738636841	0,808962546
GNAI1	protein_coding	ENSG00000127955	0,020109434	0,738702375	0,808991121
AC078802.1	antisense	ENSG00000269889	-0,082866213	0,738987713	0,809217192
AC135178.2	protein_coding	ENSG00000263809	0,080928674	0,73897659	0,809217192
FOXD4	protein_coding	ENSG00000170122	-0,078396336	0,739035924	0,809226779
AC078883.1	antisense	ENSG00000225205	0,068512496	0,739284734	0,809344387
RN7SL832P	lincRNA	ENSG00000243819	0,086894879	0,739301174	0,809344387
KRT18P10	processed_pseudogene	ENSG00000214207	0,083513227	0,739203894	0,809344387
AC006511.3	TEC	ENSG00000279865	0,084424767	0,73923257	0,809344387
YBX1P2	processed_pseudogene	ENSG00000231167	0,084058194	0,73939872	0,809398041
ZNF608	protein_coding	ENSG00000168916	0,074079734	0,739429112	0,809398041
MAT2B	protein_coding	ENSG00000038274	0,017377174	0,739619861	0,809563632
IL27RA	protein_coding	ENSG00000104998	0,023197615	0,739765155	0,809679456
IGLV5-52	IG_V_gene	ENSG00000211643	0,056365324	0,739885705	0,809768187
AC245595.1	lincRNA	ENSG00000232527	-0,08143196	0,740031917	0,809884991
ZSWIM9	protein_coding	ENSG00000185453	0,054431959	0,740150514	0,809971564
EFCAB7	protein_coding	ENSG00000203965	0,032695509	0,740273912	0,81006338
AC091153.1	processed_pseudogene	ENSG00000213939	-0,083640193	0,740517291	0,810286473
CLIC1P1	processed_pseudogene	ENSG00000231313	-0,080314599	0,740594942	0,810328209
SNTB1	protein_coding	ENSG00000172164	-0,031325703	0,7407528	0,810457694
AC010501.1	antisense	ENSG00000271714	0,074818403	0,740814335	0,810481785
MPV17L2	protein_coding	ENSG00000254858	-0,026946861	0,740863433	0,810492267
AP002833.3	TEC	ENSG00000279358	0,073720453	0,740931269	0,810523246
AC004812.2	antisense	ENSG00000277283	0,056578335	0,740986219	0,810540125
ETHE1	protein_coding	ENSG00000105755	-0,023670271	0,741539773	0,811102381
TMEM219	protein_coding	ENSG00000149932	-0,022580128	0,741598769	0,811123653
ECSCR	protein_coding	ENSG00000249751	0,084991935	0,741849502	0,811354625
ACY1	protein_coding	ENSG00000243989	-0,075370557	0,741948837	0,811419998
RPL18AP3	processed_pseudogene	ENSG00000213442	0,049679156	0,742232669	0,811687125
AP003108.3	TEC	ENSG00000279246	-0,083350837	0,742637326	0,812086349
RPS27AP16	transcribed_processed_	ENSG00000224631	0,048174739	0,742821836	0,812244807
SLC43A3	protein_coding	ENSG00000134802	-0,016211183	0,74286799	0,812251972
TFDP2	protein_coding	ENSG00000114126	-0,013937214	0,743174837	0,812500848
AL133551.1	lincRNA	ENSG00000272892	0,085264192	0,74354491	0,812862113
SMC1B	protein_coding	ENSG00000077935	0,085082839	0,74373761	0,813029441
CYB561D1	protein_coding	ENSG00000174151	-0,033741347	0,744047229	0,813281212

AL138955.1	sense_intronic	ENSG00000276809	0,08266878	0,744008888	0,813281212
AC117503.2	TEC	ENSG00000278861	-0,072807002	0,744090034	0,813284658
AC105285.1	antisense	ENSG00000245213	0,047488432	0,744148244	0,813304939
CDKAL1	protein_coding	ENSG00000145996	0,017274409	0,744278104	0,813403523
ZBTB8OSP2	processed_pseudogene	ENSG00000172799	0,065162247	0,744512446	0,813616276
RANBP17	protein_coding	ENSG00000204764	-0,025744853	0,744760336	0,813843812
AP005018.2	unprocessed_pseudoge	ENSG00000254851	-0,080678835	0,744880171	0,813931396
AC024060.1	antisense	ENSG00000271870	0,060662985	0,744976061	0,813992809
AC090286.3	processed_pseudogene	ENSG00000263045	-0,074885875	0,745136979	0,814080594
THBD	protein_coding	ENSG00000178726	0,069232471	0,745179317	0,814080594
APPL2	protein_coding	ENSG00000136044	0,016829864	0,745215171	0,814080594
PRRT2	protein_coding	ENSG00000167371	-0,077558887	0,74509676	0,814080594
LPGAT1	protein_coding	ENSG00000123684	-0,014645909	0,74540232	0,814241669
CHST3	protein_coding	ENSG00000122863	-0,021524643	0,745498533	0,814303398
PAXIP1-AS2	antisense	ENSG00000214106	-0,046121237	0,745644495	0,814409393
AL391684.1	bidirectional_promoter_	ENSG00000224934	-0,080438806	0,745674987	0,814409393
AC018475.1	processed_pseudogene	ENSG00000241556	0,072847596	0,745768948	0,814468644
SEC16A	protein_coding	ENSG00000148396	0,02848447	0,745897082	0,814524963
TMEM63A	protein_coding	ENSG00000196187	-0,030256508	0,745939658	0,814524963
GUSBP2	transcribed_unprocesse	ENSG00000241549	-0,050420619	0,745939164	0,814524963
NEPRO	protein_coding	ENSG00000163608	-0,015961091	0,746484865	0,815015629
EPHB1	protein_coding	ENSG00000154928	-0,083677346	0,746499075	0,815015629
CA15P1	unitary_pseudogene	ENSG00000241527	-0,072861806	0,74650822	0,815015629
AC012414.3	lincRNA	ENSG00000259383	-0,071540777	0,746561877	0,815030824
AP001273.1	TEC	ENSG00000279696	0,073689839	0,746661707	0,815096424
LEMD3	protein_coding	ENSG00000174106	0,019600831	0,746895989	0,815308784
TLL2	protein_coding	ENSG00000095587	-0,064474159	0,746960147	0,815335424
AC007950.1	protein_coding	ENSG00000140455	0,01676597	0,747458267	0,815835722
AC009148.1	antisense	ENSG00000260495	-0,0700363	0,747620444	0,815925893
RF00411	snoRNA	ENSG00000252192	-0,073785273	0,747592586	0,815925893
AC006449.2	antisense	ENSG00000275532	0,080177568	0,747709315	0,815937239
SLC6A11	protein_coding	ENSG00000132164	0,073684448	0,747710405	0,815937239
NUFIP2	protein_coding	ENSG00000108256	-0,019990396	0,747825622	0,816019552
AL109618.1	processed_pseudogene	ENSG00000234241	0,079335558	0,747934523	0,816094965
LINC01118	sense_overlapping	ENSG00000222005	-0,071546201	0,747997737	0,816120523
OR7E7P	unprocessed_pseudoge	ENSG00000238228	0,079948707	0,748061046	0,816146181
AL157902.2	TEC	ENSG00000279513	0,064279006	0,7482486	0,816307383
LINC02616	processed_transcript	ENSG00000261761	-0,044158504	0,748381556	0,816365584
ABCA11P	transcribed_processed_	ENSG00000251595	-0,067144428	0,748377082	0,816365584
MFSD12	protein_coding	ENSG00000161091	0,023358641	0,748664346	0,81663063

C22orf46	protein_coding	ENSG00000184208	0,03095586	0,748758114	0,816689477
RPLP0P6	processed_pseudogene	ENSG00000213553	-0,034776642	0,748841704	0,816737216
TRIM62	protein_coding	ENSG00000116525	0,042641613	0,749185104	0,817068302
POLR2L	protein_coding	ENSG00000177700	0,025990529	0,749293647	0,817113389
CD99	protein_coding	ENSG00000002586	-0,014732716	0,749306125	0,817113389
GABPAP	processed_pseudogene	ENSG00000235720	0,077625013	0,74959916	0,817353171
AC005519.1	sense_overlapping	ENSG00000258559	-0,082360369	0,749605712	0,817353171
FRG1DP	unprocessed_pseudoge	ENSG00000282870	0,082477505	0,749873596	0,8176018
AC040169.3	sense_intronic	ENSG00000274677	0,083276859	0,75006072	0,817762352
AC007485.2	TEC	ENSG00000279059	0,058371658	0,750858504	0,818588631
AC129492.8	TEC	ENSG00000279152	0,067533228	0,751019937	0,818677593
WHAMMP3	transcribed_unprocesse	ENSG00000276141	-0,044687089	0,750993562	0,818677593
ERCC6L2	protein_coding	ENSG00000182150	0,017284214	0,751198024	0,818828203
LINC01355	lincRNA	ENSG00000261326	0,074120326	0,751316504	0,818900667
MAU2	protein_coding	ENSG00000129933	-0,020783802	0,751344356	0,818900667
RHOA-IT1	sense_intronic	ENSG00000235908	-0,077735432	0,75148099	0,819006064
UBA5	protein_coding	ENSG00000081307	-0,014380452	0,75189308	0,81941164
GAPVD1	protein_coding	ENSG00000165219	-0,014460611	0,752357099	0,819830204
PSMA7	protein_coding	ENSG00000101182	-0,018331994	0,752349933	0,819830204
LINP1	lincRNA	ENSG00000223784	-0,080850213	0,752576898	0,820026148
RN7SL752P	misc_RNA	ENSG00000239437	-0,058751616	0,752672052	0,820086262
AC090739.1	antisense	ENSG00000254165	-0,08072348	0,752864643	0,820252527
LMNB1-DT	lincRNA	ENSG00000251072	-0,079126728	0,753022776	0,820381236
SNAPC1	protein_coding	ENSG00000023608	-0,023508809	0,753106356	0,820428712
TAF8	protein_coding	ENSG00000137413	0,014483173	0,753472935	0,820784465
AL691432.2	antisense	ENSG00000272106	-0,063858274	0,753781112	0,820924339
AC234782.1	processed_pseudogene	ENSG00000230312	0,081471683	0,753712532	0,820924339
NLRC5	protein_coding	ENSG00000140853	-0,037538294	0,75365215	0,820924339
GCKR	protein_coding	ENSG00000084734	-0,043501044	0,753731155	0,820924339
RENBP	protein_coding	ENSG00000102032	-0,068616598	0,753801467	0,820924339
HMGB1P41	processed_pseudogene	ENSG00000253516	0,076008965	0,754348439	0,821476398
RASSF8-AS1	antisense	ENSG00000246695	-0,037032736	0,754626908	0,821736017
AL390195.1	sense_overlapping	ENSG00000243960	-0,071142049	0,754905598	0,821995849
RIC8B	protein_coding	ENSG00000111785	0,017119683	0,755505287	0,822561494
AC107871.1	protein_coding	ENSG00000260007	-0,073885734	0,75564319	0,822667967
DANT2	lincRNA	ENSG00000235244	-0,068556873	0,7559505	0,822958851
MFSD14C	protein_coding	ENSG00000196312	0,037420619	0,756047	0,822974853
SEC23A	protein_coding	ENSG00000100934	-0,01602014	0,756052666	0,822974853
NOS1AP	protein_coding	ENSG00000198929	-0,052122084	0,756085575	0,822974853
FAM207BP	processed_pseudogene	ENSG00000228797	-0,067202713	0,756178489	0,823032308

SNHG1	processed_transcript	ENSG00000255717	-0,019994926	0,756260013	0,82307736
FHAD1	protein_coding	ENSG00000142621	-0,013694697	0,756305413	0,823083096
AC004854.2	antisense	ENSG00000272768	-0,06993271	0,756459641	0,823163584
CCDC88A	protein_coding	ENSG00000115355	0,019445024	0,756436673	0,823163584
LINC01705	lincRNA	ENSG00000232679	-0,07985712	0,756569743	0,823239717
USP45	protein_coding	ENSG00000123552	0,020572472	0,756778496	0,823423181
GPX4	protein_coding	ENSG00000167468	-0,019597552	0,75695049	0,82356663
GYG1	protein_coding	ENSG00000163754	0,020407778	0,757134208	0,82372282
RASSF5	protein_coding	ENSG00000266094	-0,066909806	0,757259923	0,823815893
ACVRL1	protein_coding	ENSG00000139567	0,058786087	0,757455393	0,823941135
RBMS2	protein_coding	ENSG00000076067	-0,013230026	0,757426043	0,823941135
AC093791.1	lincRNA	ENSG00000250038	-0,067936325	0,757770203	0,824239864
PLCXD1	protein_coding	ENSG00000182378	-0,022167847	0,758222157	0,824687726
AC073934.1	antisense	ENSG00000240790	-0,07468332	0,758442597	0,824883745
ITGA8	protein_coding	ENSG00000077943	0,066063138	0,75872513	0,825147272
ZMAT2	protein_coding	ENSG00000146007	0,014456534	0,75881944	0,825161503
ZNF251	protein_coding	ENSG00000198169	-0,021023944	0,758858911	0,825161503
SNORA22	snoRNA	ENSG00000206634	0,073840874	0,758824856	0,825161503
ACER3	protein_coding	ENSG00000078124	0,019033119	0,759213924	0,825460008
CYCS	protein_coding	ENSG00000172115	-0,021284339	0,759213069	0,825460008
AC093484.3	antisense	ENSG00000265401	0,077464398	0,759582119	0,825729012
POLR3DP1	processed_pseudogene	ENSG00000214626	-0,068494355	0,75956545	0,825729012
FSBP	protein_coding	ENSG00000265817	0,079107672	0,759582057	0,825729012
FSBP	protein_coding	ENSG00000265817	0,079107672	0,759582057	0,825729012
AC011939.3	antisense	ENSG00000275638	0,075653749	0,759773586	0,825856611
SNORD9	snoRNA	ENSG00000199436	0,064443203	0,759780029	0,825856611
RND3	protein_coding	ENSG00000115963	0,015313475	0,759967793	0,826016928
RHPN1	protein_coding	ENSG00000158106	-0,045802338	0,760070912	0,826085231
AC008264.2	antisense	ENSG00000273489	0,077401043	0,76020422	0,826137376
RN7SL3	misc_RNA	ENSG00000278771	0,04985382	0,760195092	0,826137376
TMEM56-RWDD3	protein_coding	ENSG00000271092	-0,078691946	0,760267938	0,826137376
AC092135.1	TEC	ENSG00000279294	-0,075559754	0,760280008	0,826137376
CARF	protein_coding	ENSG00000138380	-0,030514066	0,760658559	0,826504929
PPP1R12A	protein_coding	ENSG00000058272	-0,016533243	0,760772222	0,826584641
AC092045.1	processed_pseudogene	ENSG00000239557	-0,073496166	0,760859924	0,826587392
MRPS18C	protein_coding	ENSG00000163319	-0,017912591	0,76082265	0,826587392
AC007663.4	sense_intronic	ENSG00000273343	-0,07816403	0,760895658	0,826587392
HIGD1AP16	processed_pseudogene	ENSG00000219608	-0,064105593	0,761142261	0,826811491
CASKIN1	protein_coding	ENSG00000167971	0,071617152	0,761311693	0,82690795
EIF2AK2	protein_coding	ENSG00000055332	0,015652126	0,761302404	0,82690795

ATP12A	protein_coding	ENSG00000075673	0,071248014	0,76158514	0,827161154
PCDHGB3	protein_coding	ENSG00000262209	-0,076606057	0,761671546	0,827211194
ZNF436-AS1	antisense	ENSG00000249087	0,067843595	0,761715519	0,827215148
MCRIP2P1	processed_pseudogene	ENSG00000241418	0,064206416	0,762042805	0,827505032
ACAP2-IT1	sense_intronic	ENSG00000229325	-0,078004508	0,762063142	0,827505032
ANKS1B	protein_coding	ENSG00000185046	-0,037383686	0,762316472	0,827736293
IL1R2	protein_coding	ENSG00000115590	0,063732464	0,762441707	0,827804219
TEAD1	protein_coding	ENSG00000187079	-0,022767926	0,762493988	0,827804219
CCNL2	protein_coding	ENSG00000221978	-0,044333156	0,762500113	0,827804219
YWHAZP2	processed_pseudogene	ENSG00000213236	0,077282409	0,762762869	0,828045649
AC022532.1	TEC	ENSG00000280401	0,071659959	0,762824405	0,828068622
CDK11B	protein_coding	ENSG00000248333	0,026719449	0,762869985	0,828074273
AC104162.1	transcribed_unitary_pse	ENSG00000224479	0,06865689	0,762953527	0,828121128
DCXR-DT	antisense	ENSG00000264569	0,063772455	0,763029471	0,828159732
AC097534.2	antisense	ENSG00000272870	-0,056253334	0,763174422	0,828273225
DHRS1	protein_coding	ENSG00000157379	-0,026393311	0,763263631	0,828282385
AL391825.1	transcribed_processed_	ENSG00000229808	0,077406612	0,763229571	0,828282385
NUS1P1	processed_pseudogene	ENSG00000235636	-0,073596569	0,763700433	0,828668706
MTX3	protein_coding	ENSG00000177034	0,020931263	0,763660063	0,828668706
MRPL35P3	processed_pseudogene	ENSG00000226253	0,066979006	0,764050587	0,828925769
CDH19	protein_coding	ENSG00000071991	0,067711736	0,764058589	0,828925769
FHL3	protein_coding	ENSG00000183386	-0,039787246	0,763983387	0,828925769
AC096921.2	sense_overlapping	ENSG00000261468	0,069079389	0,764176658	0,82901001
PMS2P7	unprocessed_pseudoge	ENSG00000229018	0,077873747	0,764607453	0,829433483
SLC2A8	protein_coding	ENSG00000136856	0,031590697	0,764695886	0,829441674
ZNF329	protein_coding	ENSG00000181894	0,017222006	0,764662964	0,829441674
AC099568.2	lincRNA	ENSG00000272931	0,076803758	0,765027645	0,829757641
AC090425.2	antisense	ENSG00000272910	-0,072582764	0,765133649	0,829784854
PARP16	protein_coding	ENSG00000138617	-0,023033588	0,765125757	0,829784854
KRT18P48	processed_pseudogene	ENSG00000217889	0,064457201	0,765423418	0,830055217
GRPEL1	protein_coding	ENSG00000109519	0,013758461	0,765489305	0,830082777
TDRKH-AS1	antisense	ENSG00000203288	0,06761489	0,765892638	0,830476237
DNAH14	protein_coding	ENSG00000185842	0,023989183	0,766090914	0,830647317
AC021097.1	antisense	ENSG00000272661	-0,06608795	0,766165742	0,830684536
AL662890.1	lincRNA	ENSG00000225173	0,06391905	0,766331882	0,830791539
MAN2A2	protein_coding	ENSG00000196547	0,02472966	0,766345447	0,830791539
PCDHGA1	protein_coding	ENSG00000204956	0,07169904	0,766395495	0,830801882
AL139353.2	antisense	ENSG00000250365	-0,07470823	0,766450036	0,830813803
C1GALT1P1	processed_pseudogene	ENSG00000257818	-0,066730744	0,766487507	0,830813803
AC006249.1	sense_intronic	ENSG00000274578	0,076192276	0,766539099	0,830825816

PDCL3P5	processed_pseudogene	ENSG00000213089	0,070756036	0,766705528	0,83096229
HKDC1	protein_coding	ENSG00000156510	-0,014843435	0,766809125	0,831030655
FTH1P20	processed_pseudogene	ENSG00000226564	0,069596235	0,766919783	0,831106665
UBBP4	transcribed_unprocesse	ENSG00000263563	0,063503421	0,767022336	0,831173885
AC139712.1	processed_pseudogene	ENSG00000204993	0,064141018	0,767228981	0,831353889
STXBP4	protein_coding	ENSG00000166263	-0,017415523	0,767402741	0,831498242
RPL7AP11	processed_pseudogene	ENSG00000242445	0,075255232	0,767595505	0,831619239
LDHBP2	processed_pseudogene	ENSG00000213684	0,069803695	0,767578215	0,831619239
GCDH	protein_coding	ENSG00000105607	-0,023591067	0,767916406	0,831922961
ABCA2	protein_coding	ENSG00000107331	-0,031059347	0,76798785	0,831956415
RCN1P2	processed_pseudogene	ENSG00000214455	0,026834553	0,768124792	0,832018523
NGRN	protein_coding	ENSG00000182768	-0,019198366	0,768126315	0,832018523
AC104211.1	processed_transcript	ENSG00000248858	-0,067269736	0,768337853	0,832196055
TCIRG1	protein_coding	ENSG00000110719	0,028234153	0,76841194	0,832196055
YARS	protein_coding	ENSG00000134684	-0,011388602	0,768399836	0,832196055
SLC12A5	protein_coding	ENSG00000124140	-0,053526456	0,768461382	0,832205658
CDK7P1	processed_pseudogene	ENSG00000213194	-0,067288182	0,768652691	0,832368885
AL020996.1	antisense	ENSG00000228172	0,076472168	0,768721377	0,832399316
AC011481.2	lincRNA	ENSG00000267114	0,061999943	0,768789202	0,832428811
LINC00618	sense_overlapping	ENSG00000225163	0,074445708	0,768907638	0,8325131
TRPC1	protein_coding	ENSG00000144935	-0,015778037	0,769189497	0,832774312
AL356801.1	processed_pseudogene	ENSG00000259318	0,073321723	0,769483559	0,833048709
AC009275.1	lincRNA	ENSG00000273297	0,061082412	0,769557639	0,833084935
AC068790.9	lincRNA	ENSG00000275389	0,07226419	0,769854717	0,833278553
SEMA4G	protein_coding	ENSG00000095539	0,060647458	0,769858376	0,833278553
SEPT8	protein_coding	ENSG00000164402	-0,014858589	0,769829146	0,833278553
HNRNPKP1	processed_pseudogene	ENSG00000250859	-0,072462471	0,769962569	0,833303376
ADAM23	protein_coding	ENSG00000114948	-0,039880347	0,769957873	0,833303376
STON1	protein_coding	ENSG00000243244	-0,070588666	0,770178245	0,833492814
AL513175.1	processed_pseudogene	ENSG00000226723	-0,07442961	0,770826625	0,834150482
RPL21P75	processed_pseudogene	ENSG00000213860	0,075360828	0,770951761	0,834241881
AC012442.1	lincRNA	ENSG00000228251	0,075985982	0,771199448	0,834418573
RN7SL587P	misc_RNA	ENSG00000240233	0,06903583	0,771164202	0,834418573
EIF1	protein_coding	ENSG00000173812	0,012834668	0,771237098	0,834418573
RPL35P2	processed_pseudogene	ENSG00000220583	0,072579952	0,771629015	0,83479856
IPO5P1	transcribed_processed_	ENSG00000269837	-0,058528992	0,771882452	0,835028698
LRRC41	protein_coding	ENSG00000132128	0,017035095	0,771936166	0,835042761
DIABLO	protein_coding	ENSG00000184047	0,028671367	0,772065025	0,835138108
AL162377.1	antisense	ENSG00000231856	-0,054490047	0,772196547	0,835143045
AC072061.1	antisense	ENSG00000259826	-0,07384353	0,772232465	0,835143045

AC008764.6	lincRNA	ENSG00000269399	0,071012526	0,77219845	0,835143045
UPF3AP2	transcribed_processed_	ENSG00000214832	0,062469681	0,772133443	0,835143045
CNTNAP3C	protein_coding	ENSG00000283378	-0,073607084	0,773039262	0,83597149
AL365295.1	processed_transcript	ENSG00000237356	-0,073724958	0,773133997	0,836029856
ZNF169	protein_coding	ENSG00000175787	0,05084684	0,773208531	0,836066373
SYCE1L	protein_coding	ENSG00000205078	-0,036387819	0,773707626	0,836517839
AL365205.1	protein_coding	ENSG00000124593	-0,069060246	0,773699451	0,836517839
C20orf194	protein_coding	ENSG00000088854	0,025473325	0,77377057	0,836541795
PCDHB9	protein_coding	ENSG00000177839	0,068879026	0,773907156	0,83664536
AL138963.1	lincRNA	ENSG00000228886	0,065020512	0,77417897	0,836895097
COASY	protein_coding	ENSG00000068120	0,017138622	0,774383582	0,837029071
PTENP1	transcribed_processed_	ENSG00000237984	0,067903765	0,774384526	0,837029071
COX6A1	protein_coding	ENSG00000111775	0,023051714	0,774535822	0,837148488
AC005736.1	lincRNA	ENSG00000262185	0,066968172	0,774995958	0,837513415
NAIP	protein_coding	ENSG00000249437	0,067768738	0,774943953	0,837513415
POMZP3	protein_coding	ENSG00000146707	0,022764496	0,77497488	0,837513415
ROBO3	protein_coding	ENSG00000154134	-0,026094201	0,775124001	0,837607653
AL591684.2	processed_transcript	ENSG00000254929	0,064063698	0,775402533	0,837787151
TLE6	protein_coding	ENSG00000104953	0,064155275	0,775412651	0,837787151
IAPP	protein_coding	ENSG00000121351	-0,07373712	0,775376968	0,837787151
SEC24B-AS1	antisense	ENSG00000247950	-0,073440997	0,775625409	0,837972879
AC008443.1	processed_transcript	ENSG00000233937	-0,051909193	0,775755059	0,838068805
AL353803.4	lincRNA	ENSG00000261334	0,069307437	0,775808073	0,838081933
AL121652.1	lincRNA	ENSG00000273165	0,063877546	0,77587911	0,838114528
AL035701.1	antisense	ENSG00000231769	-0,07331312	0,776004888	0,838162107
CMTM6	protein_coding	ENSG00000091317	0,015294099	0,775967453	0,838162107
PNRC2P1	processed_pseudogene	ENSG00000228217	0,063272099	0,776096623	0,838217048
RPL7P32	processed_pseudogene	ENSG00000231006	0,067038799	0,776314492	0,838408206
PA2G4P4	processed_pseudogene	ENSG00000230457	-0,057521968	0,776363012	0,838416458
SOAT2	protein_coding	ENSG00000167780	0,073573549	0,776460802	0,838477916
AP000974.1	TEC	ENSG00000279742	0,073727881	0,776547345	0,838527222
FSCN3	protein_coding	ENSG00000106328	0,064607554	0,776833973	0,838792566
BX842568.2	processed_pseudogene	ENSG00000223723	0,065117267	0,777125965	0,839019505
AL078604.1	processed_pseudogene	ENSG00000216480	-0,071708782	0,777113481	0,839019505
EEF1A1P12	processed_pseudogene	ENSG00000214199	-0,063800147	0,777230385	0,839088073
SHARPIN	protein_coding	ENSG00000179526	-0,022425525	0,777362407	0,839186429
SNORD111B	snoRNA	ENSG00000221514	-0,07005001	0,777417984	0,839202256
CYP2J2	protein_coding	ENSG00000134716	0,042914068	0,777524524	0,839244297
ECD	protein_coding	ENSG00000122882	-0,016515017	0,777538768	0,839244297
PCBP2P1	processed_pseudogene	ENSG00000235701	-0,05938928	0,777792003	0,839473451

RPL23AP25	processed_pseudogene	ENSG00000233084	0,071374956	0,777990338	0,83964333
NAPG	protein_coding	ENSG00000134265	0,02286261	0,778257602	0,839887578
LINC02026	lincRNA	ENSG00000214146	0,071135823	0,778403677	0,839956827
LINC02454	lincRNA	ENSG00000256268	0,03004508	0,778372428	0,839956827
AC106897.1	antisense	ENSG00000249679	-0,072109065	0,778659791	0,840170274
AC004816.1	lincRNA	ENSG00000259153	-0,038184163	0,77868341	0,840170274
HSPA8P9	processed_pseudogene	ENSG00000241478	-0,069767159	0,778770334	0,840219861
XPNPEP3	protein_coding	ENSG00000196236	0,013853032	0,778857286	0,840269472
AL008729.2	lincRNA	ENSG00000272379	0,059382419	0,77925737	0,840568458
GNB2	protein_coding	ENSG00000172354	0,018661423	0,779197091	0,840568458
FBXL3	protein_coding	ENSG00000005812	0,018551664	0,77922836	0,840568458
AC002310.2	antisense	ENSG00000239791	-0,062721119	0,779823125	0,841089988
SMARCE1P1	processed_pseudogene	ENSG00000225215	0,067019582	0,779827914	0,841089988
SNORA60	snoRNA	ENSG00000199266	-0,070606031	0,779863886	0,841089988
AL135786.1	processed_pseudogene	ENSG00000231967	-0,058815998	0,780219638	0,841429423
SEPT7P7	processed_pseudogene	ENSG00000229897	0,059985201	0,780409681	0,841545876
OFD1	protein_coding	ENSG00000046651	0,015056794	0,780377626	0,841545876
AC109992.2	sense_overlapping	ENSG00000244327	-0,06426582	0,780490475	0,841588751
AC092903.2	lincRNA	ENSG00000248787	-0,071628517	0,78055923	0,841618642
GOLGA6L9	protein_coding	ENSG00000197978	-0,042838017	0,780710545	0,841737543
KRT18P38	processed_pseudogene	ENSG00000214012	0,070172027	0,78076875	0,841756048
PRR4	protein_coding	ENSG00000111215	-0,054085541	0,780832488	0,841780517
PRR4	protein_coding	ENSG00000111215	-0,054085541	0,780832488	0,841780517
RPS11P5	processed_pseudogene	ENSG00000232888	-0,066580007	0,78091305	0,841823118
LINC01385	lincRNA	ENSG00000251604	0,070445103	0,781330804	0,842229189
AC012313.7	antisense	ENSG00000269106	0,07113726	0,781398729	0,842258142
AL357079.1	antisense	ENSG00000237950	-0,070794351	0,781517056	0,842328786
MPP5	protein_coding	ENSG00000072415	-0,011985275	0,781546406	0,842328786
AC110285.2	lincRNA	ENSG00000262877	0,070299977	0,781611551	0,842354733
AC012464.1	antisense	ENSG00000257283	-0,071510041	0,781661539	0,842364342
AL022157.1	antisense	ENSG00000226310	-0,064386168	0,782012546	0,842654057
BCKDHA	protein_coding	ENSG00000248098	0,069887303	0,782685609	0,843335006
MGAT4A	protein_coding	ENSG00000071073	-0,06003836	0,783039868	0,843653982
CCDC144B	transcribed_unprocesse	ENSG00000154874	0,070933824	0,783063913	0,843653982
AC104393.1	sense_intronic	ENSG00000253509	0,071268073	0,783302354	0,843866544
AC009262.1	lincRNA	ENSG00000236212	0,061355946	0,783368689	0,843893682
AC008464.1	antisense	ENSG00000253660	0,058812204	0,783471876	0,843960513
HMGN1P8	processed_pseudogene	ENSG00000241120	-0,060181327	0,783694807	0,844122522
WRN	protein_coding	ENSG00000165392	-0,01516498	0,783704587	0,844122522
RC3H1-IT1	sense_intronic	ENSG00000236535	-0,063824699	0,783823893	0,844162364

AC008147.2	sense_intronic	ENSG00000257298	-0,064764705	0,783799392	0,844162364
CBS	protein_coding	ENSG00000160200	0,070449142	0,783966417	0,844265652
SRP19	protein_coding	ENSG00000153037	0,032723354	0,784101172	0,844265652
SLC2A1	protein_coding	ENSG00000117394	0,016752447	0,784148945	0,844265652
CEP295	protein_coding	ENSG00000166004	-0,014555678	0,784017713	0,844265652
RTTN	protein_coding	ENSG00000176225	-0,017093502	0,78416678	0,844265652
USP35	protein_coding	ENSG00000118369	-0,027041208	0,78411353	0,844265652
ST13P4	processed_pseudogene	ENSG00000232150	0,071055787	0,784248906	0,844309751
SNX33	protein_coding	ENSG00000173548	-0,023735193	0,784635923	0,844682069
AC012085.1	processed_pseudogene	ENSG00000186076	-0,069209413	0,784760279	0,844771601
AC018521.3	lincRNA	ENSG00000264243	0,062619645	0,785088392	0,844996965
TMEM94	protein_coding	ENSG00000177728	-0,028859805	0,785093231	0,844996965
METTL21EP	transcribed_unprocesse	ENSG00000250878	0,057995699	0,785065829	0,844996965
FAM234A	protein_coding	ENSG00000167930	-0,031287111	0,785277024	0,845150431
GPR153	protein_coding	ENSG00000158292	-0,037096623	0,785342252	0,845176283
EIF4A1P7	processed_pseudogene	ENSG00000235472	0,065115982	0,785393186	0,845186749
SMN2	protein_coding	ENSG00000205571	0,061182868	0,785473891	0,84522925
SMN2	protein_coding	ENSG00000205571	0,061182868	0,785473891	0,84522925
BOLA3	protein_coding	ENSG00000163170	-0,020777572	0,78560956	0,845330889
CTSL	protein_coding	ENSG00000135047	-0,012069294	0,785662896	0,84534393
AL136304.1	antisense	ENSG00000272223	0,069445546	0,785965488	0,845580789
AL691426.1	lincRNA	ENSG00000228707	0,05922257	0,786009143	0,8455834
AC004801.2	transcribed_processed_	ENSG00000240399	0,07001099	0,786071861	0,845606518
VPS16	protein_coding	ENSG00000215305	-0,018796197	0,78612815	0,845622718
SBSN	protein_coding	ENSG00000189001	-0,030899099	0,786239638	0,845698289
RAB5IF	protein_coding	ENSG00000101084	-0,030045588	0,786362016	0,845785566
AC025048.2	processed_transcript	ENSG00000267248	0,050062834	0,786612386	0,84601049
LINC01220	lincRNA	ENSG00000259687	0,068846238	0,786827182	0,846165898
LYPLA1P3	processed_pseudogene	ENSG00000218350	0,068212262	0,786839396	0,846165898
GABPB1-AS1	antisense	ENSG00000244879	-0,026562837	0,787087256	0,846243657
AC005775.1	antisense	ENSG00000266933	-0,066627219	0,787203159	0,846243657
AC108134.2	lincRNA	ENSG00000261889	-0,057464353	0,787241783	0,846243657
RN7SKP269	misc_RNA	ENSG00000202415	-0,063136046	0,787113012	0,846243657
AC103988.1	processed_pseudogene	ENSG00000261635	0,058758592	0,787121006	0,846243657
CSTF3-DT	processed_transcript	ENSG00000247151	-0,065668317	0,78720864	0,846243657
LZTS3	protein_coding	ENSG00000088899	0,031061657	0,787150945	0,846243657
PYGL	protein_coding	ENSG00000100504	0,008381111	0,787118109	0,846243657
RPEP4	processed_pseudogene	ENSG00000233010	-0,063065499	0,787495932	0,846472489
PLA2G4B	protein_coding	ENSG00000243708	0,067106007	0,787547949	0,846484039
SNRPEP2	processed_pseudogene	ENSG00000256968	-0,066166795	0,787666881	0,846512497

MT-ND1	protein_coding	ENSG00000198888	0,055544254	0,787680491	0,846512497
AC008735.1	sense_intronic	ENSG00000267096	0,058852802	0,787804002	0,846581792
GBA	protein_coding	ENSG00000177628	0,014893269	0,787850159	0,846587036
SGSM1	protein_coding	ENSG00000167037	0,063386026	0,787892902	0,846588612
MICU1	protein_coding	ENSG00000107745	0,013090847	0,787964654	0,846621356
AC004466.2	sense_intronic	ENSG00000274737	0,069898599	0,788036927	0,846654656
CAMK2N1	protein_coding	ENSG00000162545	-0,030373344	0,788327853	0,846922857
AC092683.1	lincRNA	ENSG00000230606	0,069082074	0,788372956	0,84692695
AL359313.1	lincRNA	ENSG00000236366	0,065267116	0,78853564	0,846994692
AC008280.3	lincRNA	ENSG00000272156	0,064371891	0,788559905	0,846994692
ZNF20	protein_coding	ENSG00000132010	0,069114537	0,788499821	0,846994692
AC007998.3	lincRNA	ENSG00000267583	-0,057757168	0,788894904	0,847223022
AC079336.3	sense_intronic	ENSG00000265337	0,057057281	0,788896405	0,847223022
AL355994.2	unprocessed_pseudoge	ENSG00000232456	0,066751846	0,788875074	0,847223022
CHD5	protein_coding	ENSG00000116254	0,04506801	0,789163786	0,847465797
ZCWPW2	protein_coding	ENSG00000206559	0,064143483	0,78934013	0,847596382
AP001178.1	sense_intronic	ENSG00000263727	0,061983337	0,789368039	0,847596382
BX255923.1	lincRNA	ENSG00000225655	0,061344184	0,789805547	0,848011082
LINC01315	lincRNA	ENSG00000229891	-0,068758009	0,789878289	0,848011082
ZCRB1	protein_coding	ENSG00000139168	0,022552383	0,789874708	0,848011082
PTPRD	protein_coding	ENSG00000153707	-0,020835754	0,789925943	0,848017853
IREB2	protein_coding	ENSG00000136381	-0,01250514	0,789991495	0,848043838
AC104561.1	lincRNA	ENSG00000253390	-0,066949001	0,79023506	0,848260903
HERC2	protein_coding	ENSG00000128731	-0,024869533	0,790366793	0,848326864
LRRC46	protein_coding	ENSG00000141294	-0,058666996	0,790379232	0,848326864
HRAS	protein_coding	ENSG00000174775	0,018059122	0,790450994	0,848359492
LKAAEAR1	protein_coding	ENSG00000171695	-0,068059438	0,790583683	0,848457503
SPIRE1	protein_coding	ENSG00000134278	-0,015765125	0,790850562	0,848672926
AL353763.1	TEC	ENSG00000279456	-0,068296319	0,790867169	0,848672926
WNK4	protein_coding	ENSG00000126562	-0,026931763	0,791193171	0,848978337
PROCR	protein_coding	ENSG00000101000	-0,015519721	0,791331655	0,849082513
AC022325.1	processed_pseudogene	ENSG00000249411	-0,057994021	0,791419583	0,849103386
PCDHGB8P	transcribed_unitary_pse	ENSG00000248449	0,065437534	0,791433907	0,849103386
GXYLT1P4	transcribed_processed_	ENSG00000275026	0,06541389	0,791998344	0,849664507
AL353596.1	antisense	ENSG00000232876	0,060307205	0,792443033	0,850097108
AL445472.1	lincRNA	ENSG00000227486	-0,063122128	0,792570023	0,850180899
AC244023.1	unprocessed_pseudoge	ENSG00000229509	0,058797991	0,792604045	0,850180899
PALM2	protein_coding	ENSG00000243444	0,054962012	0,792820566	0,850368675
AC073655.2	antisense	ENSG00000258365	0,067387746	0,792991493	0,850418589
IPO13	protein_coding	ENSG00000117408	0,018056895	0,792916878	0,850418589

AC087289.1	protein_coding	ENSG00000132481	-0,021682251	0,792990519	0,850418589
NDUFA7	protein_coding	ENSG00000267855	0,064859351	0,793239102	0,850639652
AC068446.2	lincRNA	ENSG00000247765	0,065279022	0,793408621	0,850776955
CRTC3-AS1	antisense	ENSG00000259736	0,068082854	0,793503332	0,850834031
RIDA	protein_coding	ENSG00000132541	-0,021591871	0,794393734	0,851744236
MID1IP1-AS1	antisense	ENSG00000238123	-0,059543106	0,794550125	0,851867385
RPL31P49	processed_pseudogene	ENSG00000241680	0,059156956	0,794657305	0,851937762
AC245452.1	antisense	ENSG00000224086	0,045400785	0,794877922	0,85212974
LDHAP5	processed_pseudogene	ENSG00000213574	0,057342625	0,794986268	0,852156807
HNRNPA3P6	processed_pseudogene	ENSG00000213300	-0,061259769	0,794948567	0,852156807
LINC02105	lincRNA	ENSG00000250447	-0,064275443	0,795140968	0,852233552
PTPRN2	protein_coding	ENSG00000155093	0,032324517	0,795367725	0,852389644
KIF4B	protein_coding	ENSG00000226650	-0,063501088	0,795369723	0,852389644
RNY4	misc_RNA	ENSG00000252316	-0,060816602	0,7955937	0,852540584
RPL4P3	processed_pseudogene	ENSG00000230364	-0,058778047	0,795583892	0,852540584
LRRC59	protein_coding	ENSG00000108829	-0,007457774	0,795932686	0,852859276
AC012640.1	antisense	ENSG00000248968	0,056448511	0,796048246	0,85293854
AL359532.1	sense_intronic	ENSG00000272914	0,066789194	0,79619758	0,853053982
LZTFL1	protein_coding	ENSG00000163818	-0,017517185	0,796342631	0,853164822
AC000089.1	processed_pseudogene	ENSG00000235776	0,067039934	0,796494409	0,853282858
RPL12P4	processed_pseudogene	ENSG00000185834	-0,043586623	0,796626734	0,853335473
TPTEP2	transcribed_unprocesse	ENSG00000244627	-0,060150766	0,796605193	0,853335473
PLPPR2	protein_coding	ENSG00000105520	0,024263513	0,796764444	0,853438413
MMP25-AS1	antisense	ENSG00000261971	-0,059522107	0,796985534	0,853594495
AC068620.1	lincRNA	ENSG00000269921	-0,066268133	0,796993397	0,853594495
AL353796.1	sense_overlapping	ENSG00000259994	-0,057314701	0,797172628	0,853741873
AL139100.1	processed_pseudogene	ENSG00000217130	-0,066029806	0,797377259	0,853916436
AC078852.2	sense_intronic	ENSG00000254094	0,057777122	0,797545174	0,854051664
AC009053.2	lincRNA	ENSG00000261079	0,066260465	0,797729628	0,854204587
HIST1H2AM	protein_coding	ENSG00000278677	0,052992665	0,797886905	0,854328396
RPL35P6	processed_pseudogene	ENSG00000244018	-0,054978878	0,798057878	0,85443859
SNORD70	snoRNA	ENSG00000212534	-0,06588976	0,798073138	0,85443859
NOSIP	protein_coding	ENSG00000142546	0,018177044	0,798229327	0,854561202
AL158166.1	sense_intronic	ENSG00000227076	-0,064511989	0,798275568	0,854566099
AC090220.1	sense_intronic	ENSG00000266965	0,054434831	0,798426808	0,854683394
LINC01424	antisense	ENSG00000236519	-0,063784082	0,798633442	0,854859971
DRD4	protein_coding	ENSG00000069696	0,063160473	0,798855126	0,855052637
AL139339.1	antisense	ENSG00000234699	0,061947305	0,798967496	0,855128286
HSD17B4	protein_coding	ENSG00000133835	0,011860281	0,799215269	0,855348839
AVPR2	protein_coding	ENSG00000126895	0,053427021	0,799261882	0,855354093

ADAM20P1	transcribed_unprocesse	ENSG00000259158	0,060570762	0,799481567	0,855544555
CFAP46	protein_coding	ENSG00000171811	0,057675323	0,799933044	0,85594947
FAM160B2	protein_coding	ENSG00000158863	-0,023574578	0,799943415	0,85594947
AC145343.1	lincRNA	ENSG00000265055	0,065726517	0,800093224	0,85602045
GOLGA8A	protein_coding	ENSG00000175265	-0,055430031	0,800092882	0,85602045
USP44	protein_coding	ENSG00000136014	0,047472446	0,800165038	0,856052628
DNAJC22	protein_coding	ENSG00000178401	0,030099585	0,800250363	0,856099256
CERNA3	antisense	ENSG00000253603	-0,063897055	0,80031351	0,856122155
AC093677.2	antisense	ENSG00000269559	0,063404325	0,800445924	0,856174493
MAN1A1	protein_coding	ENSG00000111885	0,024235922	0,800442225	0,856174493
AC069281.2	processed_transcript	ENSG00000274272	0,061941022	0,800854563	0,856566911
THAP12P7	processed_pseudogene	ENSG00000253919	-0,064895184	0,80093693	0,856610337
NDUFB2-AS1	antisense	ENSG00000240889	-0,063897778	0,801429888	0,857069032
PDE6B	protein_coding	ENSG00000133256	0,031503073	0,801449388	0,857069032
AC023830.3	sense_intronic	ENSG00000278434	0,05413366	0,801587608	0,85717215
ARF4-AS1	antisense	ENSG00000272146	0,053973916	0,801858687	0,857417323
LINC01545	lincRNA	ENSG00000204904	0,060672241	0,802011428	0,85753594
AC002310.1	antisense	ENSG00000235560	-0,055593228	0,802114165	0,857556376
B4GALT6	protein_coding	ENSG00000118276	-0,018622768	0,802083473	0,857556376
KIFAP3	protein_coding	ENSG00000075945	0,015101077	0,802286098	0,857695484
AL031058.1	antisense	ENSG00000261189	-0,063211134	0,802511073	0,857881987
RPSAP4	processed_pseudogene	ENSG00000239269	-0,054813346	0,802544207	0,857881987
SF3A3P2	processed_pseudogene	ENSG00000254449	0,0533913	0,802611989	0,85790973
PTPMT1	protein_coding	ENSG00000110536	0,026068366	0,803022748	0,858304057
HOXA11-AS	antisense	ENSG00000240990	0,051764319	0,803168037	0,858334556
AC087742.1	lincRNA	ENSG00000274363	0,057984606	0,803280103	0,858334556
AC087343.1	processed_pseudogene	ENSG00000243181	-0,060841096	0,803178394	0,858334556
FERMT3	protein_coding	ENSG00000149781	0,057598839	0,803241878	0,858334556
ZNF92	protein_coding	ENSG00000146757	0,024337288	0,803240271	0,858334556
GABARAPL2	protein_coding	ENSG00000034713	-0,016846977	0,803302379	0,858334556
CFL1	protein_coding	ENSG00000172757	0,011586449	0,803525171	0,858527884
PHF8	protein_coding	ENSG00000172943	0,017220061	0,803907548	0,858891692
YWHAZP4	processed_pseudogene	ENSG00000213131	-0,057284085	0,804161126	0,858983631
ZNF212	protein_coding	ENSG00000170260	-0,021740263	0,804133253	0,858983631
CCDC146	protein_coding	ENSG00000135205	-0,058579448	0,804082859	0,858983631
BMS1P2	transcribed_unprocesse	ENSG00000251079	0,059675826	0,804138616	0,858983631
RPLP1	protein_coding	ENSG00000137818	-0,019826104	0,80449941	0,859300224
GATC	protein_coding	ENSG00000257218	-0,011688543	0,804553497	0,859310628
TMEM208	protein_coding	ENSG00000168701	-0,022024378	0,804592945	0,859310628
AP000526.1	sense_intronic	ENSG00000271127	-0,05052046	0,804896288	0,859589839

AC008915.2	lincRNA	ENSG00000260136	0,036752639	0,805537061	0,860184574
IQSEC2	protein_coding	ENSG00000124313	-0,026005104	0,806175196	0,860821182
C5orf22	protein_coding	ENSG00000082213	0,015531211	0,806297882	0,860907364
OR10J6P	unprocessed_pseudoge	ENSG00000158731	0,063420013	0,806424096	0,860997304
AC022784.6	lincRNA	ENSG00000279949	-0,056087069	0,806848469	0,86117011
RPL17P50	processed_pseudogene	ENSG00000213700	0,063190658	0,806847026	0,86117011
NTAN1P2	processed_pseudogene	ENSG00000250569	0,062271409	0,806879864	0,86117011
LONRF2	protein_coding	ENSG00000170500	0,055816273	0,80679581	0,86117011
MINDY4	protein_coding	ENSG00000106125	-0,062649029	0,806803432	0,86117011
AC063950.1	transcribed_processed_	ENSG00000258230	0,052041883	0,806679025	0,86117011
MRPS31P5	transcribed_unprocesse	ENSG00000243406	0,060163184	0,806715521	0,86117011
ZNF544	protein_coding	ENSG00000198131	-0,012185965	0,807172635	0,861437753
EMSY	protein_coding	ENSG00000158636	0,017769996	0,807709027	0,861920507
AC103703.1	sense_intronic	ENSG00000283045	0,059713777	0,807707667	0,861920507
KIAA2013	protein_coding	ENSG00000116685	-0,014449062	0,807766711	0,861937217
AL049780.3	TEC	ENSG00000279594	-0,06070001	0,807971791	0,862111198
WDR90	protein_coding	ENSG00000161996	-0,028895086	0,808962786	0,863123693
AC097263.1	processed_pseudogene	ENSG00000228519	0,053999414	0,809107759	0,863143667
LAMB3	protein_coding	ENSG00000196878	0,017149904	0,809071323	0,863143667
UBXN2A	protein_coding	ENSG00000173960	-0,014799185	0,809034301	0,863143667
KIT	protein_coding	ENSG00000157404	0,051450156	0,809343189	0,863322562
PLEKHN1	protein_coding	ENSG00000187583	0,040765441	0,80935964	0,863322562
REPS1	protein_coding	ENSG00000135597	-0,011848005	0,809464279	0,863389274
SNORD91B	snoRNA	ENSG00000275084	0,061229086	0,809746242	0,863645108
RF00019	misc_RNA	ENSG00000199459	0,054482827	0,80983994	0,863700129
ASPHD1	protein_coding	ENSG00000174939	0,040785431	0,809919648	0,863740223
LINC01140	lincRNA	ENSG00000267272	0,062440783	0,810071622	0,863767558
AL445433.1	processed_pseudogene	ENSG00000227034	-0,058755063	0,810070927	0,863767558
AL162274.3	TEC	ENSG00000279982	-0,05631687	0,809996872	0,863767558
RN7SKP80	misc_RNA	ENSG00000202058	0,050428552	0,810159018	0,863815838
TUBBP5	transcribed_unprocesse	ENSG00000159247	0,060538941	0,810477138	0,864110105
RN7SKP9	misc_RNA	ENSG00000201793	0,058117197	0,810567138	0,864161139
ZNF91	protein_coding	ENSG00000167232	0,0244207	0,810727192	0,86428685
SNX1	protein_coding	ENSG00000028528	-0,00908272	0,810888016	0,864413368
PROS1	protein_coding	ENSG00000184500	-0,011869305	0,810975029	0,864461195
COX7A2L	protein_coding	ENSG00000115944	0,017246094	0,811122025	0,864528023
CAPN7	protein_coding	ENSG00000131375	0,011193929	0,811091362	0,864528023
AC116407.1	antisense	ENSG00000214708	0,057371469	0,811424819	0,864613664
AL391839.2	antisense	ENSG00000233825	-0,06010092	0,811410714	0,864613664
AC135977.1	processed_pseudogene	ENSG00000226268	0,060006139	0,811406455	0,864613664

TPI1	protein_coding	ENSG00000111669	-0,013906557	0,81145531	0,864613664
ARHGAP1	protein_coding	ENSG00000175220	-0,018822797	0,811406714	0,864613664
DPY19L1P2	unprocessed_pseudoge	ENSG00000231952	-0,059211783	0,81133752	0,864613664
TAS2R46	protein_coding	ENSG00000226761	0,05693925	0,811576788	0,864698178
PCDHB18P	transcribed_unprocesse	ENSG00000146001	-0,052029658	0,811632704	0,864712834
CCDC169	protein_coding	ENSG00000242715	0,044731192	0,811860896	0,864911022
PCDHGA6	protein_coding	ENSG00000253731	0,061197152	0,812433831	0,865431488
AL136531.2	protein_coding	ENSG00000274322	-0,054229002	0,812492214	0,86544873
AL109811.1	antisense	ENSG00000226849	0,057606975	0,812684339	0,865473586
AC097532.2	lincRNA	ENSG00000272769	0,054474596	0,812653504	0,865473586
STRA6LP	processed_transcript	ENSG00000255036	0,058980288	0,812598719	0,865473586
NAP1L2	protein_coding	ENSG00000186462	-0,055481543	0,812648546	0,865473586
PYCARD-AS1	antisense	ENSG00000261359	0,056655896	0,812744737	0,865492968
C9orf163	lincRNA	ENSG00000196366	-0,060432055	0,812947692	0,865619206
EXTL2	protein_coding	ENSG00000162694	-0,013741547	0,812992558	0,865622041
DPYD-IT1	sense_intronic	ENSG00000232542	0,0526003	0,813102944	0,865649695
PSMD6	protein_coding	ENSG00000163636	-0,012953128	0,813145426	0,865649989
VN1R108P	unprocessed_pseudoge	ENSG00000230772	0,061487798	0,81319811	0,865661143
FOXP4-AS1	antisense	ENSG00000234753	0,044966848	0,81338531	0,865815482
AC024361.2	lincRNA	ENSG00000263063	-0,05392696	0,813606802	0,86598986
ZNF34	protein_coding	ENSG00000196378	0,030668163	0,813770365	0,866090508
AC068669.1	TEC	ENSG00000279199	0,054451839	0,813846824	0,866126938
CCDC47	protein_coding	ENSG00000108588	0,012605678	0,814006525	0,866251949
TMLHE-AS1	antisense	ENSG00000224533	0,054397229	0,814136838	0,866270298
RPS7P3	processed_pseudogene	ENSG00000231940	-0,0549745	0,814154147	0,866270298
AOAH	protein_coding	ENSG00000136250	-0,059226448	0,814192712	0,866270298
NKX2-8	protein_coding	ENSG00000136327	-0,060565161	0,814103874	0,866270298
RPP38	protein_coding	ENSG00000152464	-0,014084538	0,814254119	0,866290693
PKD1P5	transcribed_unprocesse	ENSG00000254681	-0,059899928	0,814922436	0,866956749
AC007496.3	TEC	ENSG00000280392	-0,052460573	0,815315808	0,86733025
AC138393.3	lincRNA	ENSG00000278467	0,060275985	0,81545443	0,867432725
AC090360.1	protein_coding	ENSG00000267127	0,052359178	0,815662558	0,867579777
AC099522.1	transcribed_processed_	ENSG00000249149	0,056230522	0,815677272	0,867579777
MAP2K5	protein_coding	ENSG00000137764	0,022102756	0,815745715	0,867607582
CCNT2-AS1	antisense	ENSG00000224043	-0,047150379	0,816184233	0,868028967
SIAH2-AS1	antisense	ENSG00000244265	-0,058597188	0,81735648	0,869174429
STOM	protein_coding	ENSG00000148175	0,012240603	0,817331206	0,869174429
GRTP1	protein_coding	ENSG00000139835	-0,030989077	0,817388414	0,869174429
AC093157.2	antisense	ENSG00000235795	0,059826287	0,817505892	0,869224444
AF131215.4	sense_intronic	ENSG00000254936	-0,046842144	0,817520211	0,869224444

AC090181.3	TEC	ENSG00000278991	0,058900541	0,817859359	0,869539965
CAMKMT	protein_coding	ENSG00000143919	0,025712368	0,817995271	0,869639385
ZNF286B	protein_coding	ENSG00000249459	-0,028292963	0,818054761	0,869657552
MAPK9	protein_coding	ENSG00000050748	-0,013025216	0,818259683	0,869830315
CCNC	protein_coding	ENSG00000112237	-0,018638829	0,818372682	0,869905349
INIP	protein_coding	ENSG00000148153	-0,012131269	0,81851414	0,870010624
RIMS2	protein_coding	ENSG00000176406	-0,01493717	0,818777296	0,870245238
AC097358.2	antisense	ENSG00000272202	-0,053646785	0,8190165	0,870339066
INPP4A	protein_coding	ENSG00000040933	-0,014932683	0,818914691	0,870339066
LBHD1	protein_coding	ENSG00000162194	-0,030277128	0,819017635	0,870339066
AC027644.1	unprocessed_pseudoge	ENSG00000232546	0,053806649	0,819035316	0,870339066
MRGPRF-AS1	antisense	ENSG00000256508	0,056594962	0,81929628	0,870571273
LCMT1	protein_coding	ENSG00000205629	-0,014143669	0,819343147	0,87057597
STAC	protein_coding	ENSG00000144681	0,019422099	0,819550275	0,870750939
TBC1D22A	protein_coding	ENSG00000054611	0,012693435	0,819706408	0,870871714
AC073283.1	antisense	ENSG00000225187	0,054009951	0,820040419	0,871110702
AC007683.1	processed_pseudogene	ENSG00000224415	-0,058757744	0,819992818	0,871110702
DSC2	protein_coding	ENSG00000134755	-0,022372938	0,820058773	0,871110702
AP000240.1	sense_intronic	ENSG00000273017	-0,057704012	0,820145299	0,871112381
AC092117.2	TEC	ENSG00000279901	-0,058436754	0,820121645	0,871112381
CNN2P4	processed_pseudogene	ENSG00000213816	0,057435136	0,820457967	0,871356877
APBB1IP	protein_coding	ENSG00000077420	-0,013384537	0,820460459	0,871356877
RTN4	protein_coding	ENSG00000115310	-0,006849373	0,820725948	0,871593703
ULBP3	protein_coding	ENSG00000131019	0,034178446	0,820959773	0,87179688
SLC25A42	protein_coding	ENSG00000181035	0,022446693	0,821139282	0,871942359
AC011330.2	sense_intronic	ENSG00000275601	-0,057839806	0,821220885	0,871983866
MIR762HG	antisense	ENSG00000260083	0,042528431	0,821432931	0,872163867
BLOC1S6	protein_coding	ENSG00000104164	-0,014973134	0,821509827	0,87220036
AC132872.2	antisense	ENSG00000264548	0,049718617	0,822171964	0,872858169
LINC02453	lincRNA	ENSG00000245017	0,058260446	0,822402413	0,873057634
RN7SL128P	misc_RNA	ENSG00000240869	-0,045826154	0,822692997	0,873291618
MXRA7	protein_coding	ENSG00000182534	-0,012244712	0,822707979	0,873291618
AC011825.4	antisense	ENSG00000265008	0,053290518	0,822805015	0,873349421
C9orf43	protein_coding	ENSG00000157653	0,037763528	0,822848045	0,873349897
FTH1P3	processed_pseudogene	ENSG00000213453	0,054892348	0,822957119	0,873420466
ZCCHC10	protein_coding	ENSG00000155329	0,016948849	0,823183194	0,873615197
FARP1-AS1	antisense	ENSG00000231194	0,051525512	0,823306462	0,873700809
LINC00324	lincRNA	ENSG00000178977	-0,040761834	0,823388756	0,873742932
SLCO3A1	protein_coding	ENSG00000176463	-0,026864372	0,823779462	0,874112306
AC091057.2	antisense	ENSG00000247728	-0,057242746	0,823829797	0,874120493

AC078850.1	lincRNA	ENSG00000248187	0,057667429	0,823953906	0,87419768
TXN	protein_coding	ENSG00000136810	-0,015226556	0,823987789	0,87419768
NRGN	protein_coding	ENSG00000154146	-0,025963128	0,824047496	0,874215805
CARMIL2	protein_coding	ENSG00000159753	-0,05222153	0,824106169	0,874232829
SKIDA1	protein_coding	ENSG00000180592	-0,050479803	0,824231288	0,874320337
AC048351.1	transcribed_processed_	ENSG00000236297	0,057425014	0,824301541	0,874349638
AC010203.2	antisense	ENSG00000257696	0,052086747	0,824702841	0,874684831
AC015961.2	antisense	ENSG00000267707	0,050126628	0,824761263	0,874701562
RNPS1P1	processed_pseudogene	ENSG00000250896	-0,055739173	0,82515445	0,875073307
LINC01730	lincRNA	ENSG00000275491	-0,055705236	0,825203196	0,875079755
SNHG22	antisense	ENSG00000267322	0,056651683	0,82542093	0,875253731
PRDM5	protein_coding	ENSG00000138738	-0,016438257	0,825452604	0,875253731
AC089999.2	sense_intronic	ENSG00000277566	0,04857565	0,825714851	0,875486538
RTCA-AS1	lincRNA	ENSG00000224616	0,048749336	0,826331138	0,875994662
AC068338.2	lincRNA	ENSG00000260274	-0,046616731	0,826317521	0,875994662
AL035456.1	processed_pseudogene	ENSG00000235036	-0,053182623	0,826247455	0,875994662
AC073367.1	sense_intronic	ENSG00000225213	-0,051007801	0,826364931	0,875994662
IDI1	protein_coding	ENSG00000067064	0,015401894	0,826452784	0,876042514
BDP1	protein_coding	ENSG00000145734	0,017128622	0,826537938	0,8760875
SNORD67	snoRNA	ENSG00000212135	0,055957724	0,826764249	0,876282091
AC022382.1	antisense	ENSG00000269886	0,045615025	0,826934702	0,876417463
AC006254.1	protein_coding	ENSG00000243696	0,048142233	0,827083521	0,876529893
AC099522.2	lincRNA	ENSG00000272525	0,052504932	0,827494655	0,876920294
RPL30	protein_coding	ENSG00000156482	0,011953123	0,827613112	0,877000513
LINC02129	lincRNA	ENSG00000261045	0,056275585	0,827847132	0,877203176
DUSP9	protein_coding	ENSG00000130829	-0,050022068	0,828053188	0,877376189
FAM86JP	transcribed_unprocesse	ENSG00000171084	-0,022907015	0,828224636	0,877512516
AC010331.1	antisense	ENSG00000277383	0,055104393	0,828307461	0,877554937
SETD2	protein_coding	ENSG00000181555	0,012924121	0,828421822	0,877556246
XRCC1	protein_coding	ENSG00000073050	0,012546158	0,828382077	0,877556246
CELF1	protein_coding	ENSG00000149187	-0,012966399	0,828437057	0,877556246
UQCRQ	protein_coding	ENSG00000164405	0,01518024	0,828634811	0,877720394
MTSS1	protein_coding	ENSG00000170873	-0,019467576	0,828724285	0,877769835
ST8SIA6	protein_coding	ENSG00000148488	0,056222962	0,828888013	0,877897916
CUL1	protein_coding	ENSG00000055130	-0,011194362	0,829107169	0,878084686
TCEAL4	protein_coding	ENSG00000133142	-0,012385245	0,82928618	0,878183579
AL162390.1	sense_intronic	ENSG00000274421	0,050184054	0,829264029	0,878183579
MORN2	protein_coding	ENSG00000188010	-0,019153415	0,829418512	0,878278367
SEMA3F	protein_coding	ENSG00000001617	-0,035402656	0,829630446	0,878457432
ITGAX	protein_coding	ENSG00000140678	-0,049115327	0,829761322	0,878550654

CTDNEP1	protein_coding	ENSG00000175826	0,01699654	0,829867776	0,87861801
LRRC28	protein_coding	ENSG00000168904	0,015051697	0,830011705	0,878725033
AC040963.1	lincRNA	ENSG00000267215	0,05346233	0,830197214	0,878785346
SLC29A4	protein_coding	ENSG00000164638	0,022239119	0,830120091	0,878785346
MSANTD3	protein_coding	ENSG00000066697	-0,010552426	0,830156373	0,878785346
FRYL	protein_coding	ENSG00000075539	-0,012567896	0,830316245	0,878865984
ENO1-AS1	antisense	ENSG00000230679	0,055059444	0,830836517	0,879371296
NKX3-2	protein_coding	ENSG00000109705	-0,028845852	0,830955922	0,879452292
SIM1	protein_coding	ENSG00000112246	0,055124316	0,831333574	0,879715799
TECPR2	protein_coding	ENSG00000196663	0,015032237	0,831252326	0,879715799
AGER	protein_coding	ENSG00000204305	-0,041225728	0,831302039	0,879715799
AC009779.3	protein_coding	ENSG00000258311	0,055266513	0,831792951	0,880156499
GPLD1	protein_coding	ENSG00000112293	0,038309954	0,831876107	0,880199079
ITGA9-AS1	processed_transcript	ENSG00000235257	0,047097832	0,832103872	0,880394656
DNM1	protein_coding	ENSG00000106976	0,051415569	0,832450778	0,880579992
TCF7L1	protein_coding	ENSG00000152284	0,046228061	0,832382574	0,880579992
FH	protein_coding	ENSG00000091483	0,008578958	0,83244497	0,880579992
AP006621.5	TEC	ENSG00000279672	-0,048694914	0,832377267	0,880579992
FMN2	protein_coding	ENSG00000155816	0,013771902	0,832516127	0,880603701
ATP1B3	protein_coding	ENSG00000069849	0,010450641	0,8333024	0,881389935
CYHR1	protein_coding	ENSG00000187954	0,012831724	0,833349016	0,881393787
AC004801.6	lincRNA	ENSG00000276814	0,054777883	0,833465386	0,881468951
CNOT10	protein_coding	ENSG00000182973	0,010943856	0,833549015	0,881468951
HARS	protein_coding	ENSG00000170445	-0,007881073	0,833537959	0,881468951
PSMB6	protein_coding	ENSG00000142507	-0,012760142	0,833727907	0,88156722
PCDHGB6	protein_coding	ENSG00000253305	-0,041141555	0,833706643	0,88156722
CTSA	protein_coding	ENSG00000064601	-0,011246112	0,833846543	0,881647211
SELENOTP1	processed_pseudogene	ENSG00000233846	0,054649258	0,834140535	0,881912592
C10orf111	bidirectional_promoter_	ENSG00000176236	0,051484437	0,834255558	0,881943276
ARPC1B	protein_coding	ENSG00000130429	-0,025200837	0,834244389	0,881943276
RN7SL775P	misc_RNA	ENSG00000243370	-0,045940735	0,834686795	0,882353684
ERAL1	protein_coding	ENSG00000132591	-0,011800815	0,834782246	0,882363629
AL355336.1	sense_intronic	ENSG00000225092	0,053688601	0,83477606	0,882363629
IKBIP	protein_coding	ENSG00000166130	-0,015062227	0,834942652	0,882422168
MMP28	protein_coding	ENSG00000271447	-0,053376211	0,8349667	0,882422168
SCARNA1	scaRNA	ENSG00000252947	0,045309965	0,834945215	0,882422168
LINC00674	transcribed_unprocesse	ENSG00000237854	0,019863428	0,835029165	0,882442713
C11orf65	protein_coding	ENSG00000166323	0,048573477	0,835299936	0,882683379
AL133346.1	antisense	ENSG00000227220	0,053898821	0,835473148	0,88282093
AC099560.2	processed_pseudogene	ENSG00000234287	-0,048866457	0,835686827	0,883001227

AL590705.3	lincRNA	ENSG00000242375	-0,049177229	0,835763299	0,883036537
GRAMD1B	protein_coding	ENSG00000023171	-0,013924895	0,835900069	0,883135549
AC087893.1	sense_intronic	ENSG00000274598	0,045784681	0,836007034	0,883203064
UNC13B	protein_coding	ENSG00000198722	-0,014100869	0,836190906	0,883351815
FRG1JP	unprocessed_pseudoge	ENSG00000215548	-0,051757861	0,836269864	0,883389725
CNTD2	protein_coding	ENSG00000105219	0,045311417	0,836605903	0,883699184
MLKL	protein_coding	ENSG00000168404	-0,01476634	0,836734262	0,883789253
AC010864.1	lincRNA	ENSG00000273008	0,052307426	0,837132955	0,88414629
RNU6-8	snRNA	ENSG00000202337	-0,042976809	0,837158506	0,88414629
ZC3H11A	protein_coding	ENSG00000058673	-0,052401484	0,837314404	0,884265405
CASTOR1	protein_coding	ENSG00000239282	0,05268977	0,837434171	0,884346352
ADAMTSL1	protein_coding	ENSG00000178031	0,036730857	0,837481976	0,884351301
EIF2S2P4	processed_pseudogene	ENSG00000128692	0,036718061	0,837620704	0,884452257
YES1P1	processed_pseudogene	ENSG00000224003	0,047224281	0,837686834	0,884476549
RPL23AP18	processed_pseudogene	ENSG00000225338	-0,051588561	0,837773236	0,884522242
UBAC2-AS1	lincRNA	ENSG00000228889	-0,028122543	0,837870052	0,884578925
AC007613.1	antisense	ENSG00000262420	-0,050942625	0,837955621	0,884623728
UBQLN4P1	processed_pseudogene	ENSG00000241627	0,051904549	0,838108951	0,884740058
ICA1L	protein_coding	ENSG00000163596	-0,020273599	0,838259045	0,88485296
UBE2E3	protein_coding	ENSG00000170035	-0,009341862	0,838689638	0,885261926
CCZ1	protein_coding	ENSG00000122674	-0,011731291	0,838919151	0,885458614
RNF40	protein_coding	ENSG00000103549	-0,011362319	0,839045764	0,885546679
PEX26	protein_coding	ENSG00000215193	-0,011727647	0,839218087	0,885676545
AP000692.1	sense_overlapping	ENSG00000228107	0,051262944	0,839255175	0,885676545
ADCY10P1	transcribed_unprocesse	ENSG00000161912	0,029168287	0,839339737	0,88572021
ZDHHC9	protein_coding	ENSG00000188706	-0,013299513	0,839798404	0,886158629
ZC3H3	protein_coding	ENSG00000014164	0,020149407	0,840016869	0,886343553
TFCP2L1	protein_coding	ENSG00000115112	0,050760264	0,840574001	0,886748933
LZTS2	protein_coding	ENSG00000107816	-0,013658594	0,840506211	0,886748933
DNAJC30	protein_coding	ENSG00000176410	-0,024567772	0,840558209	0,886748933
RSPH9	protein_coding	ENSG00000172426	-0,038863209	0,840510189	0,886748933
C1orf50	protein_coding	ENSG00000164008	0,051116324	0,841340443	0,887511828
TSPAN13	protein_coding	ENSG00000106537	-0,033079766	0,841472199	0,887590871
LRG1	protein_coding	ENSG00000171236	-0,046395383	0,841501925	0,887590871
RAC1P2	processed_pseudogene	ENSG00000249936	0,031019942	0,841547107	0,887592881
RPS3AP3	processed_pseudogene	ENSG00000231241	0,043480853	0,841643334	0,887648726
SUGT1P3	transcribed_unprocesse	ENSG00000239827	-0,047087086	0,84211377	0,88809921
PLD6	protein_coding	ENSG00000179598	-0,010968723	0,842346502	0,888298975
AC073529.1	lincRNA	ENSG00000234129	-0,025483934	0,842393017	0,888302354
DIP2A-IT1	sense_intronic	ENSG00000223692	0,049733134	0,842565731	0,888438802

FO704657.1	antisense	ENSG00000272004	-0,044352041	0,842663478	0,888450519
MAP3K5	protein_coding	ENSG00000197442	-0,01556091	0,842626037	0,888450519
TTC7B	protein_coding	ENSG00000165914	0,012296326	0,842767267	0,888476001
TNS4	protein_coding	ENSG00000131746	0,046138525	0,843008743	0,888677493
AC013403.2	antisense	ENSG00000272148	0,041518652	0,843296381	0,888889339
ZFYVE28	protein_coding	ENSG00000159733	0,020600583	0,843275387	0,888889339
AC015917.2	antisense	ENSG00000265254	0,047491816	0,843892734	0,889472222
AL592293.2	processed_pseudogene	ENSG00000234297	-0,043363074	0,843944937	0,889481534
AL512844.1	processed_pseudogene	ENSG00000232952	0,047415848	0,844399878	0,889874556
BMPR1B	protein_coding	ENSG00000138696	0,049483242	0,844427838	0,889874556
CNFN	protein_coding	ENSG00000105427	0,046588925	0,844448001	0,889874556
AC100830.3	antisense	ENSG00000265967	-0,040543596	0,844793663	0,890193075
AFP	protein_coding	ENSG00000081051	0,029700625	0,844909058	0,890268933
CBWD3	protein_coding	ENSG00000196873	0,038204178	0,844996063	0,89031487
CBWD3	protein_coding	ENSG00000196873	0,038204178	0,844996063	0,89031487
SPON1-AS1	antisense	ENSG00000254418	0,047368254	0,845158164	0,890394183
RSF1-IT1	sense_intronic	ENSG00000255409	0,047830359	0,845133381	0,890394183
RNF11	protein_coding	ENSG00000123091	0,010262417	0,84566072	0,890877876
AC007390.2	sense_intronic	ENSG00000272054	0,049831346	0,845720515	0,890895108
CSPG4P11	unprocessed_pseudoge	ENSG00000259726	-0,043023423	0,845838974	0,890974134
FAM49A	protein_coding	ENSG00000197872	-0,043992647	0,845924196	0,891018142
KAT7	protein_coding	ENSG00000136504	-0,011008722	0,846036092	0,891090241
AL136964.1	lincRNA	ENSG00000274718	-0,042989708	0,846706529	0,891750586
PQLC2	protein_coding	ENSG00000040487	-0,0131072	0,846878086	0,891839676
IL18	protein_coding	ENSG00000150782	-0,013500434	0,846855302	0,891839676
GALNT16	protein_coding	ENSG00000100626	-0,049477075	0,847464564	0,892411471
RN7SKP255	misc_RNA	ENSG00000200312	-0,039896661	0,84767998	0,892592483
RPS18P9	transcribed_processed_	ENSG00000220848	0,041313026	0,847776282	0,892648059
TBC1D13	protein_coding	ENSG00000107021	-0,014071174	0,848039235	0,892879092
LGALS8-AS1	antisense	ENSG00000223776	0,041901808	0,848291167	0,893098498
EEF1A1P14	processed_pseudogene	ENSG00000233057	0,044066844	0,84854072	0,893278213
EXOC5	protein_coding	ENSG00000070367	0,010945034	0,848548973	0,893278213
EEF1A1P9	processed_pseudogene	ENSG00000249264	0,049574657	0,84885449	0,893553972
AP003352.1	antisense	ENSG00000245970	0,040924772	0,849034779	0,893697886
SEC14L1P1	processed_pseudogene	ENSG00000213693	-0,039740623	0,849285747	0,89391618
ATP5MG	protein_coding	ENSG00000167283	-0,012970292	0,849539177	0,894137041
SLC25A41	protein_coding	ENSG00000181240	0,048418161	0,849663176	0,89415953
LDAH	protein_coding	ENSG00000118961	0,011399708	0,849668568	0,89415953
AL669831.4	transcribed_unprocesse	ENSG00000230092	-0,047340389	0,849691333	0,89415953
AL049873.1	processed_pseudogene	ENSG00000213598	-0,046640674	0,850329372	0,89478505

SH3GL1P2	processed_pseudogene	ENSG00000264943	-0,047121853	0,850374837	0,894786985
AL591845.1	antisense	ENSG00000116883	-0,046804102	0,850520351	0,894894188
EIF3EP1	processed_pseudogene	ENSG00000234882	0,04678921	0,850580362	0,89491142
AL031284.1	processed_pseudogene	ENSG00000238084	-0,046361135	0,850761414	0,895055994
FAM131C	protein_coding	ENSG00000185519	-0,030460867	0,851062208	0,895326523
MSR1	protein_coding	ENSG00000038945	0,047980904	0,851609895	0,895792332
NDUFA4	protein_coding	ENSG00000189043	-0,013498016	0,851590235	0,895792332
FAM166B	protein_coding	ENSG00000215187	-0,046900656	0,851636016	0,895792332
ERFE	protein_coding	ENSG00000178752	-0,019746581	0,851731801	0,895801202
MAP4K1	protein_coding	ENSG00000104814	-0,046148985	0,851688344	0,895801202
AC009269.4	sense_intronic	ENSG00000259744	0,047256192	0,851776376	0,895802147
ZGPAT	protein_coding	ENSG00000197114	-0,023942492	0,851950581	0,895939416
LINC01232	processed_transcript	ENSG00000280734	0,022415502	0,852163425	0,896117301
PLAC8L1	protein_coding	ENSG00000173261	0,048502431	0,852357994	0,896230004
RPS16P5	transcribed_processed_	ENSG00000217527	0,045560998	0,852337879	0,896230004
LINC01534	lincRNA	ENSG00000225975	0,036115559	0,852428977	0,896258692
AP002490.1	antisense	ENSG00000251143	0,04807689	0,852691403	0,896462332
TCTEX1D2	protein_coding	ENSG00000213123	-0,044394225	0,852710075	0,896462332
YBX1P1	processed_pseudogene	ENSG00000224861	-0,033698355	0,853136665	0,896823786
TMPRSS15	protein_coding	ENSG00000154646	0,032735477	0,853141341	0,896823786
PAPOLA	protein_coding	ENSG00000090060	-0,010543545	0,853459414	0,897112166
SZT2-AS1	antisense	ENSG00000229372	-0,046892343	0,854075717	0,897713983
UBBP2	processed_pseudogene	ENSG00000228247	-0,043616818	0,854496846	0,898110603
MPG	protein_coding	ENSG00000103152	-0,015252212	0,854556327	0,898127095
MAP7	protein_coding	ENSG00000135525	0,044140878	0,854681796	0,898166912
OXER1	protein_coding	ENSG00000162881	-0,045451293	0,854664253	0,898166912
AL391095.1	lincRNA	ENSG00000224635	-0,046047155	0,855313804	0,898785025
GPRC5D-AS1	antisense	ENSG00000247498	-0,017601265	0,855727059	0,899173215
AC020911.2	antisense	ENSG00000267275	-0,043033855	0,855781072	0,899183903
RN7SL296P	misc_RNA	ENSG00000275090	-0,046368361	0,855959889	0,899297059
RPS4XP11	processed_pseudogene	ENSG00000234335	-0,046616702	0,856008243	0,899297059
AC068790.5	sense_intronic	ENSG00000270061	0,044403536	0,856020307	0,899297059
LINC00957	lincRNA	ENSG00000235314	-0,043977285	0,856430538	0,899681947
TRIB2	protein_coding	ENSG00000071575	0,020167298	0,856787013	0,900010327
NDUFAF2	protein_coding	ENSG00000164182	-0,015676591	0,856835858	0,90001554
AP000786.1	antisense	ENSG00000255893	-0,045245546	0,857062804	0,90020782
AC026367.3	lincRNA	ENSG00000275759	-0,046230293	0,857219421	0,900326215
TRPV1	protein_coding	ENSG00000196689	0,02831672	0,857763494	0,900851517
RNU7-45P	snRNA	ENSG00000238829	-0,042055256	0,857916103	0,900965657
AC018690.1	antisense	ENSG00000273306	0,045821899	0,858289248	0,901311376

AC018755.3	transcribed_processed_	ENSG00000269388	-0,041797611	0,858402718	0,901384381
AL031123.2	lincRNA	ENSG00000261211	0,044655525	0,858587453	0,901486058
TIAF1	protein_coding	ENSG00000221995	-0,045837478	0,858580543	0,901486058
C12orf74	protein_coding	ENSG00000187510	0,034169545	0,858828942	0,901693453
C12orf74	protein_coding	ENSG00000187510	0,034169545	0,858828942	0,901693453
C12orf74	protein_coding	ENSG00000187510	0,034169545	0,858828942	0,901693453
C12orf74	protein_coding	ENSG00000187510	0,034169545	0,858828942	0,901693453
LINC01410	lincRNA	ENSG00000238113	-0,033961671	0,859007044	0,901834278
ZNF543	protein_coding	ENSG00000178229	0,012750478	0,859244139	0,902037022
TMLHE	protein_coding	ENSG00000185973	-0,013137003	0,8593133	0,902063455
AC099336.1	transcribed_processed_	ENSG00000232626	0,037021002	0,859364331	0,902066852
AC112482.1	unprocessed_pseudoge	ENSG00000243016	0,044172845	0,8594045	0,902066852
RPL7P9	processed_pseudogene	ENSG00000137970	-0,021314661	0,859577432	0,902202197
CENPL	protein_coding	ENSG00000120334	-0,008499852	0,859676601	0,902260111
MNAT1	protein_coding	ENSG00000020426	0,011871768	0,85975428	0,902295466
AL161909.1	processed_pseudogene	ENSG00000232176	0,044863361	0,860219041	0,902690843
MRM1	protein_coding	ENSG00000278619	-0,015802186	0,860194659	0,902690843
GLIPR1L2	protein_coding	ENSG00000180481	-0,041886878	0,860344904	0,902776731
SNORA49	snoRNA	ENSG00000208892	0,02823877	0,860510718	0,902904529
DM1-AS	antisense	ENSG00000267395	0,044604657	0,860594361	0,902946099
GPBAR1	protein_coding	ENSG00000179921	0,043029604	0,860678444	0,902988126
M6PR	protein_coding	ENSG00000003056	-0,007446615	0,861038162	0,90331932
TSC22D1-AS1	antisense	ENSG00000278156	0,045219718	0,861673364	0,903939476
GUSBP3	transcribed_unprocesse	ENSG00000253203	-0,042052843	0,861844999	0,904073288
TWIST2	protein_coding	ENSG00000233608	-0,036312535	0,861891299	0,904075617
STK4-AS1	lincRNA	ENSG00000227477	0,038072267	0,862007055	0,904104814
AC010542.4	lincRNA	ENSG00000261519	-0,044050386	0,862044282	0,904104814
GTSF1	protein_coding	ENSG00000170627	0,044434889	0,862051377	0,904104814
SUSD2	protein_coding	ENSG00000099994	0,043623183	0,862164519	0,90417724
MSRB1	protein_coding	ENSG00000198736	0,021222959	0,86231568	0,904289529
AC008906.1	antisense	ENSG00000248734	-0,04437208	0,862416266	0,904348771
RAB3D	protein_coding	ENSG00000105514	-0,015264172	0,86282204	0,904728019
PSG8-AS1	lincRNA	ENSG00000225877	-0,037342509	0,862934511	0,904775997
ZNF746	protein_coding	ENSG00000181220	-0,012296707	0,862956024	0,904775997
AL121936.1	lincRNA	ENSG00000284607	0,045003083	0,863081855	0,90486167
AL603839.3	antisense	ENSG00000238287	-0,041686213	0,863778399	0,905499359
TUBGCP6	protein_coding	ENSG00000128159	-0,017743289	0,863759204	0,905499359
AC025171.2	antisense	ENSG00000215068	0,031412855	0,864157509	0,90585048
NKD2	protein_coding	ENSG00000145506	0,025252065	0,864495914	0,9061589
AC108471.2	bidirectional_promoter_	ENSG00000255458	-0,04095015	0,864730548	0,906358521

INSM2	protein_coding	ENSG00000168348	-0,035191292	0,864893972	0,906483488
OSGEPL1-AS1	antisense	ENSG00000253559	0,04414814	0,865074314	0,906626172
AC024243.1	antisense	ENSG00000272969	-0,038112859	0,865126515	0,906634552
WASH4P	unprocessed_pseudoge	ENSG00000234769	0,044003469	0,865202867	0,906668241
FHOD1	protein_coding	ENSG00000135723	0,010978969	0,865278826	0,906701513
KRTAP4-7	protein_coding	ENSG00000240871	0,042604518	0,865402027	0,906784284
GCC2-AS1	antisense	ENSG00000214184	0,044126203	0,865457972	0,906796578
ASAP3	protein_coding	ENSG00000088280	0,017593212	0,86567584	0,906978519
TRIM9	protein_coding	ENSG00000100505	-0,043160687	0,865792653	0,90705457
BACH1-IT2	lincRNA	ENSG00000228817	0,042527618	0,866039086	0,907266403
CCDC153	protein_coding	ENSG00000248712	0,036263512	0,866197497	0,907339666
MAP1LC3A	protein_coding	ENSG00000101460	-0,015699724	0,866161985	0,907339666
SLC26A10	protein_coding	ENSG00000135502	0,039880932	0,866514914	0,907625804
LINC01431	lincRNA	ENSG00000232645	0,04275676	0,866601446	0,907670087
DGCR6	protein_coding	ENSG00000183628	0,027026038	0,866656481	0,907681378
KIF3B	protein_coding	ENSG00000101350	-0,008447996	0,866741474	0,907724042
ODF2L	protein_coding	ENSG00000122417	-0,013167652	0,866804976	0,907744195
NXF3	protein_coding	ENSG00000147206	0,04337835	0,867106006	0,90801308
PCDHB16	protein_coding	ENSG00000272674	-0,042730066	0,86738818	0,908262193
IQCJ-SCHIP1-AS1	antisense	ENSG00000241211	-0,039648016	0,867466629	0,908288747
PI4KAP1	transcribed_unprocesse	ENSG00000274602	0,019752762	0,867502109	0,908288747
ZBED5	protein_coding	ENSG00000236287	-0,01159248	0,867623327	0,908369293
AC026471.4	lincRNA	ENSG00000261474	-0,042114539	0,867749415	0,908454929
ABR	protein_coding	ENSG00000159842	-0,008627859	0,868019442	0,908691239
AL021707.8	sense_intronic	ENSG00000273096	-0,041993578	0,868105283	0,908734719
AC093724.1	processed_pseudogene	ENSG00000213222	-0,036611664	0,868266159	0,908810356
AC022400.7	protein_coding	ENSG00000272916	0,031112235	0,868222112	0,908810356
FAH	protein_coding	ENSG00000103876	-0,010811765	0,868321749	0,908822161
USP3-AS1	antisense	ENSG00000259248	-0,033288203	0,86838577	0,90884279
ST13	protein_coding	ENSG00000100380	0,007818561	0,868504573	0,908920747
API5P2	processed_pseudogene	ENSG00000213962	0,035005954	0,868643868	0,90902014
EEF1B2P2	processed_pseudogene	ENSG00000213864	-0,039006686	0,868889603	0,909230906
AC008403.3	antisense	ENSG00000268530	-0,039332463	0,869160246	0,909436125
XRCC4	protein_coding	ENSG00000152422	-0,012329778	0,869174399	0,909436125
CIRBP	protein_coding	ENSG00000099622	-0,007821126	0,869572582	0,909806339
RBP7	protein_coding	ENSG00000162444	0,023513454	0,86989338	0,910095554
TAGLN2P1	processed_pseudogene	ENSG00000253676	0,042414313	0,870081205	0,910245627
SNHG21	antisense	ENSG00000250988	0,035097809	0,870339083	0,910468968
CYTH1	protein_coding	ENSG00000108669	-0,011868931	0,870424299	0,910511673
SYPL1P2	processed_pseudogene	ENSG00000265683	-0,035993002	0,870826476	0,910852758

PQLC2L	protein_coding	ENSG00000174899	0,013658243	0,870839188	0,910852758
STAG3L2	transcribed_unprocesse	ENSG00000277072	-0,023047642	0,871150187	0,911131583
AC092143.2	antisense	ENSG00000259006	0,041053054	0,871384317	0,911329985
P2RX5-TAX1BP3	protein_coding	ENSG00000257950	-0,041501029	0,871554332	0,911461317
LOXL1	protein_coding	ENSG00000129038	0,011951323	0,872150177	0,911991441
AC114341.1	lincRNA	ENSG00000278075	0,035143512	0,872403392	0,912209716
GAPDHP35	processed_pseudogene	ENSG00000250982	-0,037069674	0,8725334	0,912299146
AC074138.1	lincRNA	ENSG00000276071	0,038789572	0,872635906	0,912313305
RF00019	misc_RNA	ENSG00000201208	-0,037448582	0,872635296	0,912313305
REXO2	protein_coding	ENSG00000076043	0,006755053	0,87275198	0,912388149
STK16	protein_coding	ENSG00000115661	0,009532973	0,87301659	0,91261826
PATE2	protein_coding	ENSG00000196844	-0,040914192	0,873309425	0,912877851
AC011978.2	lincRNA	ENSG00000261542	-0,031598265	0,873452745	0,912934607
SMN1	protein_coding	ENSG00000172062	-0,013727964	0,873420235	0,912934607
CACNA1B	protein_coding	ENSG00000148408	0,037449778	0,873504469	0,912942146
EPB41L1	protein_coding	ENSG00000088367	-0,011597903	0,873881737	0,913243372
KCNC3	protein_coding	ENSG00000131398	-0,040283147	0,874158538	0,913486096
GPR137C	protein_coding	ENSG00000180998	0,026809446	0,874300918	0,913588334
JPH3	protein_coding	ENSG00000154118	-0,039833217	0,874361018	0,913604589
DRAP1	protein_coding	ENSG00000175550	-0,009002624	0,874491457	0,913694334
EIF2S2P3	processed_pseudogene	ENSG00000236493	-0,036552867	0,874607947	0,913769497
AC093726.1	antisense	ENSG00000272760	0,037500928	0,874671651	0,913789505
C17orf67	protein_coding	ENSG00000214226	-0,021015005	0,874838397	0,913917156
ZNF786	protein_coding	ENSG00000197362	0,022400159	0,874884881	0,913919166
RAD17	protein_coding	ENSG00000152942	-0,008970486	0,874936483	0,913926523
AC019205.1	antisense	ENSG00000229852	-0,030939446	0,87537102	0,914307338
AC069224.1	antisense	ENSG00000260572	-0,039581183	0,875390209	0,914307338
AC015871.3	sense_intronic	ENSG00000278600	0,035180295	0,875672599	0,914555709
GOLGA2	protein_coding	ENSG00000167110	0,011428773	0,875882753	0,91468204
HEBP2	protein_coding	ENSG00000051620	-0,009666176	0,875881334	0,91468204
ATE1	protein_coding	ENSG00000107669	-0,007520554	0,876402911	0,915178642
AC145423.3	sense_intronic	ENSG00000278112	-0,037694963	0,87646451	0,91519637
CD68	protein_coding	ENSG00000129226	0,03726624	0,876520468	0,915208206
TRO	protein_coding	ENSG00000067445	-0,021183737	0,87658634	0,915230393
AL449106.1	antisense	ENSG00000273062	0,038150636	0,876710327	0,91531325
HOXB-AS1	antisense	ENSG00000230148	-0,030312549	0,877068819	0,915640918
LIME1	protein_coding	ENSG00000203896	0,031166659	0,877229722	0,915762284
GAS8	protein_coding	ENSG00000141013	0,009830365	0,877415953	0,915910078
ADAT3	protein_coding	ENSG00000213638	-0,025137813	0,877817243	0,916282337
IDH3A	protein_coding	ENSG00000166411	-0,009064245	0,877994387	0,916420605

COG1	protein_coding	ENSG00000166685	0,009726706	0,878103402	0,91648775
LINC02600	lincRNA	ENSG00000250986	-0,03172894	0,878313516	0,916660402
AL161729.1	antisense	ENSG00000271155	-0,038646308	0,878538527	0,916848582
AL731577.2	sense_overlapping	ENSG00000249456	0,038891275	0,878714838	0,916985923
CYB5D2	protein_coding	ENSG00000167740	0,0113474	0,878881311	0,917112984
PLCG1-AS1	antisense	ENSG00000226648	0,033610142	0,879251398	0,917312487
AC027804.1	lincRNA	ENSG00000255179	0,038930714	0,879200406	0,917312487
MSNP1	processed_pseudogene	ENSG00000251593	-0,03903677	0,879139044	0,917312487
ERLEC1	protein_coding	ENSG00000068912	0,00964454	0,879221676	0,917312487
UBE2SP2	processed_pseudogene	ENSG00000224126	-0,038754375	0,879320977	0,917338415
LINC01002	processed_transcript	ENSG00000282508	0,038621503	0,879391573	0,917365402
AC105345.1	lincRNA	ENSG00000205959	0,038935897	0,879549224	0,917448762
FIBCD1	protein_coding	ENSG00000130720	0,028907075	0,879560946	0,917448762
ATF4P4	transcribed_processed_	ENSG00000256167	0,038253878	0,879606738	0,917449868
SIDT2	protein_coding	ENSG00000149577	0,016102674	0,879696923	0,917497274
AF124730.1	antisense	ENSG00000224649	0,039355674	0,879928668	0,917608452
AC027644.3	antisense	ENSG00000272831	-0,018559452	0,879924713	0,917608452
ASPH	protein_coding	ENSG00000198363	-0,007369638	0,87993774	0,917608452
PPP1R12B	protein_coding	ENSG00000077157	0,016253799	0,880092951	0,917723647
HMGB1P8	processed_pseudogene	ENSG00000259699	-0,037795222	0,880510532	0,918112406
CASP9	protein_coding	ENSG00000132906	0,016877895	0,880792236	0,918359451
EDEM2	protein_coding	ENSG00000088298	0,009767391	0,881082429	0,918568628
EDEM2	protein_coding	ENSG00000088298	0,009767391	0,881082429	0,918568628
EPB41L4A	protein_coding	ENSG00000129595	-0,02360973	0,881056693	0,918568628
RNFT1P3	unprocessed_pseudoge	ENSG00000187870	-0,030985695	0,881145726	0,918587925
AL391822.1	processed_pseudogene	ENSG00000229657	0,037241082	0,881372351	0,91874031
SCN1B	protein_coding	ENSG00000105711	0,033376142	0,881381489	0,91874031
AL583856.1	processed_pseudogene	ENSG00000232389	0,038385623	0,881459996	0,91877545
MAP4K3	protein_coding	ENSG00000011566	-0,010410577	0,881655208	0,918932225
AL359643.2	lincRNA	ENSG00000271978	-0,034836	0,881715593	0,918948464
C1orf43	protein_coding	ENSG00000143612	-0,009129785	0,881832126	0,919015557
ATP5PF	protein_coding	ENSG00000154723	-0,010776784	0,881914393	0,919015557
SREBF2	protein_coding	ENSG00000198911	-0,01172488	0,881871503	0,919015557
RN7SL239P	misc_RNA	ENSG00000242999	0,036592891	0,882000173	0,91905825
AC092756.1	sense_intronic	ENSG00000259771	0,038049051	0,882164878	0,919183177
AC133134.1	processed_pseudogene	ENSG00000244245	0,035877129	0,882618112	0,919608711
AP001282.1	processed_pseudogene	ENSG00000213252	0,036461766	0,88277623	0,919650659
AL139095.2	processed_pseudogene	ENSG00000220472	-0,037225981	0,882882568	0,919650659
WT1	protein_coding	ENSG00000184937	0,036912257	0,882838161	0,919650659
IPO4	protein_coding	ENSG00000196497	0,030177743	0,882874756	0,919650659

NPIPB5	protein_coding	ENSG00000243716	-0,031756558	0,882847201	0,919650659
PCSK1N	protein_coding	ENSG00000102109	-0,016808736	0,883185158	0,919872415
EIF2AK1	protein_coding	ENSG00000086232	0,005078269	0,883536917	0,920192057
LINC01311	antisense	ENSG00000260924	0,03431364	0,883600499	0,920211548
PEX16	protein_coding	ENSG00000121680	-0,011672097	0,883976122	0,920555992
AP005233.2	lincRNA	ENSG00000260877	-0,030207245	0,88451322	0,921068549
AL512791.2	lincRNA	ENSG00000275198	0,032731601	0,884755571	0,921136242
RF00019	misc_RNA	ENSG00000202251	0,031679336	0,884692947	0,921136242
DLG4	protein_coding	ENSG00000132535	-0,01124716	0,884757873	0,921136242
AC084824.4	sense_intronic	ENSG00000275854	-0,036881455	0,884754823	0,921136242
C19orf47	protein_coding	ENSG00000160392	0,010936447	0,884992497	0,921333745
AC127024.8	TEC	ENSG00000280069	0,03439419	0,885039993	0,921336425
RN7SL130P	misc_RNA	ENSG00000244256	0,034727388	0,88525491	0,921513384
RNF121	protein_coding	ENSG00000137522	0,009639389	0,885358063	0,921573989
NR2E3	protein_coding	ENSG00000278570	-0,034500088	0,885433931	0,921606187
SPOPL	protein_coding	ENSG00000144228	0,009675645	0,885639586	0,921773466
RN7SL431P	misc_RNA	ENSG00000264384	0,032123035	0,88571222	0,921802285
AC096586.1	antisense	ENSG00000272936	0,03546589	0,885802168	0,921835069
ZBTB8A	protein_coding	ENSG00000160062	-0,012768554	0,885833611	0,921835069
AC012485.3	antisense	ENSG00000283635	0,032084758	0,885899716	0,921853618
ELOB	protein_coding	ENSG00000103363	0,010346382	0,88594133	0,921853618
OXCT2	protein_coding	ENSG00000198754	0,034012047	0,886040981	0,921910538
NOS3	protein_coding	ENSG00000164867	-0,036431948	0,886254059	0,922085464
VIRMA	protein_coding	ENSG00000164944	0,005802997	0,886423502	0,922209016
AC005921.3	TEC	ENSG00000278963	0,036295385	0,886462738	0,922209016
RABAC1	protein_coding	ENSG00000105404	-0,012888236	0,886827569	0,922541766
DYNLT1	protein_coding	ENSG00000146425	0,00841625	0,886973417	0,922646689
EXTL3-AS1	antisense	ENSG00000246339	-0,02754959	0,887440591	0,923085837
LINC01003	lincRNA	ENSG00000261455	-0,025733054	0,887538654	0,923141021
TTC39B	protein_coding	ENSG00000155158	0,014387318	0,887933374	0,923504742
AC243547.1	antisense	ENSG00000244619	0,034933362	0,888074145	0,923557484
ONECUT2	protein_coding	ENSG00000119547	0,015295103	0,888178283	0,923618952
STRAP	protein_coding	ENSG00000023734	-0,006800337	0,888240319	0,923636633
Z97633.1	TEC	ENSG00000280327	-0,035427902	0,888308625	0,923660831
AL023881.1	lincRNA	ENSG00000276984	-0,034874142	0,888472377	0,923756267
LMX1B	protein_coding	ENSG00000136944	-0,015390507	0,888490486	0,923756267
TGM1	protein_coding	ENSG00000092295	-0,026005941	0,888691969	0,923918912
EEF1E1P1	processed_pseudogene	ENSG00000236307	0,036347673	0,888915978	0,92405812
ATP5IF1	protein_coding	ENSG00000130770	0,009477833	0,888893501	0,92405812
KRBA1	protein_coding	ENSG00000133619	0,021807762	0,889118431	0,924221733

SNED1	protein_coding	ENSG00000162804	-0,033866785	0,889178698	0,924237538
AL603766.1	processed_pseudogene	ENSG00000219487	0,032575152	0,889625673	0,924655276
FAM27E3	lincRNA	ENSG00000274026	0,028906763	0,889716273	0,924702582
ERG28	protein_coding	ENSG00000133935	-0,00915024	0,889831126	0,92477509
FOXN3-AS1	antisense	ENSG00000258920	0,024979906	0,889904545	0,924804531
RPSAP6	processed_pseudogene	ENSG00000214629	0,035918157	0,890086053	0,92489943
DOP1A	protein_coding	ENSG00000083097	0,009070729	0,890049447	0,92489943
DCT	protein_coding	ENSG00000080166	0,035740832	0,890560062	0,925345097
TSPAN8	protein_coding	ENSG00000127324	-0,033583643	0,890668527	0,925410917
AP001318.2	antisense	ENSG00000255062	0,034882774	0,8911617	0,925829525
GDNF-AS1	antisense	ENSG00000248587	0,034717067	0,891139831	0,925829525
AC007182.2	processed_pseudogene	ENSG00000242951	0,035516614	0,89140229	0,92591705
SEPT1	protein_coding	ENSG00000180096	0,034060522	0,891384795	0,92591705
MAST1	protein_coding	ENSG00000105613	0,019400192	0,891356662	0,92591705
AL590326.1	TEC	ENSG00000279140	-0,034112502	0,891426527	0,92591705
MEX3C	protein_coding	ENSG00000176624	-0,008984969	0,891684466	0,926138067
PRR34	lincRNA	ENSG00000182257	-0,029829778	0,891773776	0,926165369
SCARNA23	scaRNA	ENSG00000251869	-0,034743327	0,891801067	0,926165369
AC233280.1	lincRNA	ENSG00000229178	0,034947125	0,891964593	0,926288294
AC073063.1	processed_pseudogene	ENSG00000228335	-0,033366806	0,89238071	0,926646419
FAM53A	protein_coding	ENSG00000174137	-0,017078059	0,892399808	0,926646419
IKZF2	protein_coding	ENSG00000030419	0,012114173	0,892539782	0,926744845
AL807752.5	sense_overlapping	ENSG00000260190	0,032152455	0,892603403	0,926763987
APEH	protein_coding	ENSG00000164062	0,006807341	0,892798174	0,926919289
ACTRT3	protein_coding	ENSG00000184378	-0,018135037	0,893051773	0,927135648
KIF25	protein_coding	ENSG00000125337	0,034290518	0,893209598	0,927252562
TENT4B	protein_coding	ENSG00000121274	-0,010378363	0,8933697	0,927371826
AC127502.3	unprocessed_pseudogene	ENSG00000283345	0,033969653	0,893505445	0,927465796
RPL7L1P8	processed_pseudogene	ENSG00000228766	0,034219979	0,893659792	0,927557532
LMO4	protein_coding	ENSG00000143013	0,005288609	0,89368427	0,927557532
AC092718.4	sense_intronic	ENSG00000261061	0,010330156	0,893911213	0,927746128
NOP56P1	processed_pseudogene	ENSG00000235559	-0,033435275	0,893995322	0,927786473
AC006207.1	antisense	ENSG00000283138	-0,033610026	0,894061218	0,927807912
RNU4-23P	snRNA	ENSG00000199709	0,030148101	0,894159964	0,927833043
AC138866.1	unprocessed_pseudogene	ENSG00000253816	0,031823815	0,894175911	0,927833043
ANO7L1	transcribed_unprocessed	ENSG00000237276	0,025526102	0,894438936	0,928059017
GDF5	protein_coding	ENSG00000125965	0,031893631	0,89451074	0,928086568
DNM1P51	transcribed_unprocessed	ENSG00000235370	0,029070867	0,894621903	0,928154951
AC009570.1	antisense	ENSG00000272986	-0,027382746	0,894842378	0,928289777
C5orf15	protein_coding	ENSG00000113583	0,008902516	0,8948068	0,928289777

TBXA2R	protein_coding	ENSG00000006638	0,014556834	0,89495762	0,928304318
ATP5PD	protein_coding	ENSG00000167863	-0,008969454	0,894992178	0,928304318
RPS6KA4	protein_coding	ENSG00000162302	-0,009982413	0,89496929	0,928304318
ZNF404	protein_coding	ENSG00000176222	-0,015572063	0,895101234	0,928370483
RPL4P4	processed_pseudogene	ENSG00000229638	-0,015514488	0,895537174	0,928765234
GSTM4	protein_coding	ENSG00000168765	-0,011544555	0,895617689	0,928765234
RNU6-583P	snRNA	ENSG00000251821	-0,02865323	0,895612444	0,928765234
PCDHGB2	protein_coding	ENSG00000253910	0,033656134	0,895842449	0,928941315
KIAA1614	protein_coding	ENSG00000135835	-0,016387755	0,895878069	0,928941315
LINC01600	lincRNA	ENSG00000164385	0,025050811	0,896571439	0,929540464
RF00554	snoRNA	ENSG00000212458	-0,027518987	0,896532253	0,929540464
HMGB1P24	transcribed_processed_	ENSG00000275029	-0,029598324	0,896591857	0,929540464
GOLGA6L10	protein_coding	ENSG00000278662	0,031772124	0,896762301	0,929576205
GOLGA6L10	protein_coding	ENSG00000278662	0,031772124	0,896762301	0,929576205
CCDC110	protein_coding	ENSG00000168491	0,027455379	0,896682953	0,929576205
RAC1	protein_coding	ENSG00000136238	-0,006689781	0,896729556	0,929576205
RNU7-80P	snRNA	ENSG00000238778	-0,027421885	0,896955135	0,929729105
KRT18P16	processed_pseudogene	ENSG00000235275	0,030737907	0,897401963	0,930145252
SLC26A6	protein_coding	ENSG00000225697	-0,015510079	0,897560202	0,930262254
CCDC116	protein_coding	ENSG00000161180	0,032093078	0,897936134	0,930604856
ADAMTSL5	protein_coding	ENSG00000185761	0,012624863	0,898048794	0,930674588
CLDN7	protein_coding	ENSG00000181885	0,031482136	0,898460315	0,931054015
SYNRG	protein_coding	ENSG00000275066	-0,005837654	0,898522136	0,931071036
ARL17A	protein_coding	ENSG00000185829	-0,020178491	0,89878159	0,931292837
AC107871.2	lincRNA	ENSG00000260657	0,023848317	0,89906537	0,931539821
RALGAPA1P1	processed_pseudogene	ENSG00000229419	0,02261079	0,899224285	0,931610349
NXPH4	protein_coding	ENSG00000182379	0,032618758	0,899209079	0,931610349
CDCA4P4	unprocessed_pseudoge	ENSG00000234219	0,03034458	0,899288635	0,931629958
WASHC1	protein_coding	ENSG00000181404	-0,015741219	0,899345088	0,931641384
PRR13	protein_coding	ENSG00000205352	0,006801077	0,899707942	0,931970197
LIFR-AS1	antisense	ENSG00000244968	-0,032231581	0,899783059	0,932000936
PSMB2	protein_coding	ENSG00000126067	-0,005709124	0,899833989	0,932006621
AC107214.2	lincRNA	ENSG00000272744	0,030526185	0,899979001	0,932073442
DDX50P1	processed_pseudogene	ENSG00000229816	-0,032120212	0,899989392	0,932073442
AF131215.3	lincRNA	ENSG00000254839	-0,031830409	0,900170755	0,932214198
LIMD1-AS1	bidirectional_promoter_	ENSG00000230530	-0,030945649	0,900400908	0,932405464
FAM189A1	protein_coding	ENSG00000104059	0,030645266	0,900537295	0,932452541
STX16-NPEPL1	protein_coding	ENSG00000254995	0,028796517	0,900497014	0,932452541
RPL10P6	processed_pseudogene	ENSG00000230076	-0,027346939	0,900669964	0,932542832
AC099508.1	antisense	ENSG00000247033	-0,025725802	0,900872778	0,932617506

SH3BGRL	protein_coding	ENSG00000131171	0,008274121	0,900808753	0,932617506
STK4	protein_coding	ENSG00000101109	-0,005163585	0,900878499	0,932617506
LINC02109	lincRNA	ENSG00000259786	0,030521276	0,901006254	0,932702684
AC017007.2	processed_pseudogene	ENSG00000234841	-0,03164137	0,901094062	0,932746503
RNF112	protein_coding	ENSG00000128482	0,032108593	0,901170896	0,932778959
PDLIM1P4	processed_pseudogene	ENSG00000249274	0,029569389	0,901334293	0,932901007
AC106786.2	antisense	ENSG00000249996	0,032027908	0,901448226	0,932971849
CCR5	protein_coding	ENSG00000160791	-0,031051562	0,901547162	0,933027162
CACYBPP2	processed_pseudogene	ENSG00000177855	-0,024095913	0,902042171	0,933492353
RPL7P22	processed_pseudogene	ENSG00000226544	-0,026985757	0,902414429	0,933830471
AL160006.1	antisense	ENSG00000258634	-0,011385042	0,902600065	0,933975446
FXR1	protein_coding	ENSG00000114416	-0,004038594	0,902666133	0,93399669
CD33	protein_coding	ENSG00000105383	0,016308781	0,903185197	0,934486014
AC010325.1	protein_coding	ENSG00000261341	-0,02848462	0,903260783	0,934486014
AC005740.4	antisense	ENSG00000271871	0,029524625	0,903323251	0,93448804
KAZN	protein_coding	ENSG00000189337	0,010007403	0,903489794	0,934602584
CCND3	protein_coding	ENSG00000112576	0,006888836	0,903616248	0,934602584
TOX2	protein_coding	ENSG00000124191	-0,020220779	0,903539312	0,934602584
CYP2B7P	transcribed_unprocesse	ENSG00000256612	-0,029773332	0,903604158	0,934602584
RPS29P16	processed_pseudogene	ENSG00000235354	0,023283922	0,903712569	0,934655075
AL109811.2	antisense	ENSG00000271895	-0,028595008	0,9042294	0,935131361
ZNF860	protein_coding	ENSG00000197385	0,021956463	0,904264275	0,935131361
RESF1	protein_coding	ENSG00000174718	-0,007541769	0,904907701	0,935749569
SMIM3	protein_coding	ENSG00000256235	-0,011114111	0,905041807	0,935841062
FTO	protein_coding	ENSG00000140718	-0,006871752	0,905150946	0,935906731
UQCRB	protein_coding	ENSG00000156467	-0,007825965	0,905346903	0,936062156
RPS19P1	processed_pseudogene	ENSG00000214612	0,027131636	0,905628491	0,936258902
AC090589.3	sense_intronic	ENSG00000270060	0,030685447	0,905604395	0,936258902
AL157713.1	processed_pseudogene	ENSG00000237842	0,028279039	0,905830738	0,936420788
FO393419.3	processed_pseudogene	ENSG00000243659	0,02514314	0,90606109	0,936564508
ZMYM6	protein_coding	ENSG00000163867	-0,01081821	0,906017349	0,936564508
FRMD4A	protein_coding	ENSG00000151474	0,005820794	0,906235488	0,936697568
ORMDL1	protein_coding	ENSG00000128699	-0,00758399	0,906292943	0,936709749
TWF1	protein_coding	ENSG00000151239	0,009679741	0,906367882	0,936737016
SSC5D	protein_coding	ENSG00000179954	-0,029770157	0,906410669	0,936737016
AC026368.1	sense_intronic	ENSG00000277840	-0,025416579	0,906596569	0,936881928
AL136038.5	lincRNA	ENSG00000274015	-0,029443511	0,90675637	0,936955336
PEBP1P2	processed_pseudogene	ENSG00000270532	0,02869859	0,906780722	0,936955336
AC106820.4	sense_intronic	ENSG00000260293	0,026126574	0,906804652	0,936955336
AC105105.3	sense_intronic	ENSG00000274354	0,026115203	0,906966052	0,937074894

SHCBP1	protein_coding	ENSG00000171241	-0,027019217	0,907272969	0,937280515
FNDC11	protein_coding	ENSG00000125531	-0,029324286	0,907244743	0,937280515
ZNF577	protein_coding	ENSG00000161551	0,021935192	0,907444316	0,937380153
AC007229.1	processed_pseudogene	ENSG00000267544	0,029880463	0,907606775	0,937500753
AC012360.3	lincRNA	ENSG00000272994	-0,018556282	0,908025717	0,937661811
EIF4A1P2	processed_pseudogene	ENSG00000235001	0,024306115	0,908124948	0,937661811
AC011005.1	protein_coding	ENSG00000230626	0,027192014	0,907975398	0,937661811
LRRIQ4	protein_coding	ENSG00000188306	0,025066695	0,907812704	0,937661811
SENP8	protein_coding	ENSG00000166192	0,016479129	0,908098678	0,937661811
CEP290	protein_coding	ENSG00000198707	0,010452361	0,9078761	0,937661811
DESI1	protein_coding	ENSG00000100418	-0,005509424	0,90792794	0,937661811
SMIM29	protein_coding	ENSG00000186577	-0,007777315	0,90813122	0,937661811
ARAP3	protein_coding	ENSG00000120318	-0,008685991	0,908174153	0,937661811
PSPHP1	unprocessed_pseudoge	ENSG00000226278	0,014372203	0,908226949	0,937669119
AC092849.2	antisense	ENSG00000241357	0,02850732	0,908806287	0,93822001
YIPF4	protein_coding	ENSG00000119820	-0,006067828	0,908885202	0,938254253
TOMM20P4	processed_pseudogene	ENSG00000230311	0,029091551	0,909355199	0,93869219
RPL7P1	processed_pseudogene	ENSG00000214485	-0,019699644	0,9095393	0,93883498
AC007238.1	processed_pseudogene	ENSG00000231043	-0,02828644	0,909593212	0,938835571
HERC1	protein_coding	ENSG00000103657	0,009246068	0,909631422	0,938835571
AC012358.1	antisense	ENSG00000203327	-0,022949596	0,909830162	0,93899344
AC092718.1	lincRNA	ENSG00000245059	0,02949162	0,909889672	0,939007607
AC009318.2	antisense	ENSG00000273680	0,024736391	0,909938676	0,939010931
WDR33	protein_coding	ENSG00000136709	0,005611272	0,910010885	0,939012618
DERA	protein_coding	ENSG00000023697	-0,007160648	0,910031877	0,939012618
GALT	protein_coding	ENSG00000213930	-0,013919977	0,910148856	0,939038837
SNORD71	snoRNA	ENSG00000223224	0,025867426	0,910144748	0,939038837
RPL29P14	processed_pseudogene	ENSG00000241112	0,028523632	0,910388893	0,939239245
SLC41A3	protein_coding	ENSG00000114544	0,007583809	0,910581592	0,939390797
AC005534.1	antisense	ENSG00000224903	-0,023757868	0,910652021	0,939416203
REEP3	protein_coding	ENSG00000165476	0,00782112	0,910816456	0,939525542
SNRPB2	protein_coding	ENSG00000125870	-0,008281692	0,911009667	0,939596111
AC004951.1	lincRNA	ENSG00000228434	-0,028044675	0,911115528	0,939610795
AL445305.1	processed_pseudogene	ENSG00000213036	-0,026822209	0,911098927	0,939610795
AL162151.2	processed_pseudogene	ENSG00000234648	-0,028204831	0,911257405	0,939699812
LEPROTL1	protein_coding	ENSG00000104660	0,007096035	0,911304917	0,939699812
HDAC10	protein_coding	ENSG00000100429	-0,027732429	0,911339296	0,939699812
DHRS4	protein_coding	ENSG00000157326	-0,008520726	0,911416033	0,939731693
SURF6	protein_coding	ENSG00000148296	0,006191787	0,911756118	0,939987834
TNFAIP8	protein_coding	ENSG00000145779	-0,009817735	0,911720109	0,939987834

LCE1E	protein_coding	ENSG00000186226	-0,024110845	0,911821193	0,940007674
RPS15AP12	processed_pseudogene	ENSG00000232134	-0,027189328	0,911896191	0,940037741
RN7SL648P	misc_RNA	ENSG00000265727	-0,025463496	0,911951135	0,940047132
SPEF1	protein_coding	ENSG00000101222	0,028721892	0,912043171	0,940094755
ATP6V0E2-AS1	antisense	ENSG00000204934	-0,020414572	0,912151633	0,940159304
PI16	protein_coding	ENSG00000164530	0,024442124	0,912399467	0,940367491
AC051619.5	antisense	ENSG00000259520	0,02779107	0,912573123	0,940451952
RPL32	protein_coding	ENSG00000144713	-0,012205116	0,912545384	0,940451952
TEN1-CDK3	protein_coding	ENSG00000261408	0,024930434	0,912803645	0,940547739
PPP1R37	protein_coding	ENSG00000104866	0,008912327	0,91277326	0,940547739
PPIP5K1	protein_coding	ENSG00000168781	0,008641343	0,912790563	0,940547739
RHBDL1	protein_coding	ENSG00000103269	-0,027321164	0,912870044	0,940568904
FAM120C	protein_coding	ENSG00000184083	0,009588573	0,912981882	0,940636882
SAMD4B	protein_coding	ENSG00000179134	-0,008486899	0,913139814	0,94075234
AL589765.5	antisense	ENSG00000268288	0,023514977	0,913279517	0,940812565
ADNP-AS1	antisense	ENSG00000259456	-0,027397512	0,913367291	0,940812565
Z98200.1	lincRNA	ENSG00000271734	-0,024193733	0,913411986	0,940812565
NMT2	protein_coding	ENSG00000152465	0,005690411	0,913427626	0,940812565
RBM34	protein_coding	ENSG00000188739	-0,022349883	0,913327431	0,940812565
FAM171B	protein_coding	ENSG00000144369	0,007755075	0,91352438	0,940864971
AC015819.1	lincRNA	ENSG00000273669	0,028274866	0,913737971	0,940990449
AL136295.7	sense_intronic	ENSG00000278784	-0,026705672	0,913718736	0,940990449
AC130689.1	processed_pseudogene	ENSG00000108958	0,02359277	0,913831627	0,941039648
AC009806.1	lincRNA	ENSG00000254847	0,027379314	0,913956474	0,941065825
PGK1P2	processed_pseudogene	ENSG00000213290	-0,023515848	0,913944327	0,941065825
CAPRIN1	protein_coding	ENSG00000135387	-0,004033946	0,913994697	0,941065825
AC079250.1	processed_pseudogene	ENSG00000230979	-0,027383191	0,914144971	0,941173302
NNT-AS1	antisense	ENSG00000248092	-0,016940027	0,914533821	0,941241342
AC245052.1	lincRNA	ENSG00000223660	-0,022322536	0,914269051	0,941241342
SETP3	processed_pseudogene	ENSG00000266648	0,026371466	0,914538615	0,941241342
COX7CP1	processed_pseudogene	ENSG00000235957	0,025942112	0,914616702	0,941241342
PALB2	protein_coding	ENSG00000083093	0,006313191	0,914590475	0,941241342
MOB3B	protein_coding	ENSG00000120162	-0,007531342	0,914559536	0,941241342
ZNF282	protein_coding	ENSG00000170265	-0,007685981	0,914624083	0,941241342
TRIQK	protein_coding	ENSG00000205133	-0,010201788	0,9144614	0,941241342
BORCS8	protein_coding	ENSG00000254901	-0,010469906	0,914459084	0,941241342
AL162632.3	lincRNA	ENSG00000260046	-0,026667993	0,914855629	0,94143239
FO680682.1	lincRNA	ENSG00000266579	0,024275411	0,914913846	0,941445062
AL136984.1	sense_intronic	ENSG00000272033	0,025956211	0,915219727	0,941712567
FYTTD1	protein_coding	ENSG00000122068	-0,005156806	0,915389008	0,941839498

AC124854.1	lincRNA	ENSG00000249776	-0,021417503	0,915544963	0,9419325
AF131215.2	sense_intronic	ENSG00000254556	0,022488043	0,915571249	0,9419325
YWHAZP5	processed_pseudogene	ENSG00000213260	0,023274495	0,915632932	0,941948711
NDUFV2P1	processed_pseudogene	ENSG00000267809	-0,025614022	0,915733716	0,942005142
AC106779.1	antisense	ENSG00000261302	-0,022798222	0,915797456	0,942023463
AL589843.1	lincRNA	ENSG00000224848	0,025247177	0,91596697	0,942150579
SGSH	protein_coding	ENSG00000181523	0,010710374	0,916084779	0,942224503
RPL23AP65	processed_pseudogene	ENSG00000243964	-0,026679676	0,916305928	0,942404703
AC051619.6	3prime_overlapping_nc	ENSG00000259539	0,027140411	0,916632217	0,942490513
GHRLOS	antisense	ENSG00000240288	-0,026085071	0,916518847	0,942490513
YTHDF2P1	processed_pseudogene	ENSG00000270503	0,026394205	0,916665078	0,942490513
PPIAP6	processed_pseudogene	ENSG00000258477	-0,02339454	0,916597236	0,942490513
FCGRT	protein_coding	ENSG00000104870	-0,009207151	0,916499419	0,942490513
TGFBR3L	protein_coding	ENSG00000260001	0,02661399	0,916793586	0,94257539
FIP1L1	protein_coding	ENSG00000145216	0,005848216	0,917035651	0,942729748
GHRL	protein_coding	ENSG00000157017	-0,022521857	0,91701048	0,942729748
VDAC1P1	processed_pseudogene	ENSG00000073905	0,024197484	0,917103626	0,942752374
TUBA8	protein_coding	ENSG00000183785	-0,021932474	0,917405331	0,943015253
RN7SL151P	misc_RNA	ENSG00000244230	0,022379596	0,917711778	0,943168335
AC010468.1	processed_pseudogene	ENSG00000214784	0,023716622	0,917687391	0,943168335
AL109766.1	processed_pseudogene	ENSG00000259137	-0,022955379	0,917738199	0,943168335
RNU1-28P	snRNA	ENSG00000206588	0,022674481	0,917651707	0,943168335
NFATC3	protein_coding	ENSG00000072736	0,008186399	0,917832019	0,943217492
AC007091.1	lincRNA	ENSG00000223838	0,026005855	0,91792336	0,943242774
YWHAZP3	processed_pseudogene	ENSG00000229932	0,025353622	0,918040578	0,943242774
PDZD8	protein_coding	ENSG00000165650	0,007860396	0,917959012	0,943242774
SAR1B	protein_coding	ENSG00000152700	-0,004914834	0,918035447	0,943242774
AL158801.5	processed_pseudogene	ENSG00000279392	0,026713747	0,918154422	0,943312488
COL4A2	protein_coding	ENSG00000134871	0,005248336	0,918318601	0,943433906
AC091060.1	TEC	ENSG00000278986	0,024406199	0,918670981	0,943748651
AC005332.3	lincRNA	ENSG00000274561	-0,020380623	0,919217688	0,944262986
AP003064.2	antisense	ENSG00000255446	0,02635477	0,919330145	0,94433121
C19orf71	protein_coding	ENSG00000183397	-0,025628211	0,919656683	0,944404444
DLEU2L	sense_intronic	ENSG00000116652	-0,025659459	0,919547789	0,944404444
AC092135.3	TEC	ENSG00000279841	0,026205475	0,919702662	0,944404444
AC079416.2	TEC	ENSG00000279589	0,019695068	0,919628284	0,944404444
AC012313.10	TEC	ENSG00000279611	-0,025681288	0,919499761	0,944404444
AC138393.1	transcribed_unprocesse	ENSG00000185495	0,016617495	0,919619086	0,944404444
P2RX6P	transcribed_unprocesse	ENSG00000206145	-0,025041554	0,919723762	0,944404444
CCDC112	protein_coding	ENSG00000164221	-0,007366671	0,919805508	0,9444411

AL589743.1	transcribed_processed_	ENSG00000244306	-0,014999556	0,919853891	0,944443497
CCDC14	protein_coding	ENSG00000175455	-0,008563874	0,920031035	0,944578091
AP4B1	protein_coding	ENSG00000134262	0,0056312	0,920134567	0,944637099
CRY2	protein_coding	ENSG00000121671	-0,009281792	0,92051005	0,944927984
AC011773.4	antisense	ENSG00000260493	-0,021300721	0,920562434	0,944934464
CSKMT	protein_coding	ENSG00000214756	-0,014644133	0,920642422	0,944969276
EAF2	protein_coding	ENSG00000145088	-0,010257175	0,9209364	0,945223718
CACFD1	protein_coding	ENSG00000160325	-0,011697995	0,921217439	0,945464856
AL391095.3	lincRNA	ENSG00000275401	-0,024598877	0,921312667	0,945515276
GDPD3	protein_coding	ENSG00000102886	0,022375385	0,92159253	0,94570785
GNG12	protein_coding	ENSG00000172380	-0,00536209	0,921551488	0,94570785
RNU6ATAC14P	snRNA	ENSG00000252027	-0,021720149	0,921754038	0,945826262
HIST2H2AA4	protein_coding	ENSG00000272196	0,023305344	0,921855523	0,945883075
SMG1P5	transcribed_unprocesse	ENSG00000183604	0,021235788	0,921956063	0,945938912
AL355578.1	lincRNA	ENSG00000272515	-0,024597614	0,922073325	0,9460119
AC005329.3	antisense	ENSG00000280486	0,021820536	0,922352871	0,946103127
Z82243.1	lincRNA	ENSG00000273243	0,024373854	0,922278304	0,946103127
AMOT	protein_coding	ENSG00000126016	0,007622053	0,922342818	0,946103127
BACH2	protein_coding	ENSG00000112182	-0,021637008	0,92232339	0,946103127
ZNRF2P2	transcribed_processed_	ENSG00000225264	-0,01993146	0,922392888	0,946103127
AC092757.3	lincRNA	ENSG00000277144	0,02108246	0,92264798	0,946317451
DTX4	protein_coding	ENSG00000110042	0,010934627	0,923239064	0,946876347
EIF4A2P4	processed_pseudogene	ENSG00000224781	-0,022099737	0,923309785	0,94690153
SLC8A1	protein_coding	ENSG00000183023	0,008028334	0,923620053	0,947125008
ZNF609	protein_coding	ENSG00000180357	0,007353452	0,923614988	0,947125008
GATA2-AS1	antisense	ENSG00000244300	-0,009975988	0,923947013	0,947412921
AC084125.4	antisense	ENSG00000265393	0,023814402	0,924103671	0,947501375
AC011933.3	antisense	ENSG00000265342	-0,022125276	0,924125671	0,947501375
AC117383.1	antisense	ENSG00000249417	-0,015769623	0,924284285	0,947595028
GNPDA2	protein_coding	ENSG00000163281	-0,007824313	0,924309417	0,947595028
AC104596.1	antisense	ENSG00000250326	0,021327583	0,924484369	0,947679649
LINC01138	processed_transcript	ENSG00000274020	0,024598101	0,924447812	0,947679649
TSACC	protein_coding	ENSG00000163467	0,023948479	0,924614578	0,947718391
AC068491.4	TEC	ENSG00000279191	-0,018942495	0,924600046	0,947718391
AL161431.1	lincRNA	ENSG00000275216	0,007891647	0,924906462	0,947970193
FAM85B	antisense	ENSG00000253893	0,023601874	0,925093092	0,948114098
MTOR	protein_coding	ENSG00000198793	0,005849551	0,925177573	0,948153302
ADD2	protein_coding	ENSG00000075340	0,009252675	0,925519726	0,94845656
RWDD4P2	processed_pseudogene	ENSG00000175741	-0,013403435	0,925660063	0,94850559
FMNL3	protein_coding	ENSG00000161791	0,005598024	0,925651466	0,94850559

FNDC10	protein_coding	ENSG00000228594	0,023217228	0,926025788	0,948832938
AC044802.1	lincRNA	ENSG00000246777	0,022583964	0,92645904	0,949182022
RAD9A	protein_coding	ENSG00000172613	0,010760997	0,926448699	0,949182022
PIKFYVE	protein_coding	ENSG00000115020	0,006220004	0,926739932	0,949398217
AC024568.1	transcribed_processed_	ENSG00000253558	0,022393538	0,926762638	0,949398217
CYCSP55	processed_pseudogene	ENSG00000214810	-0,020684827	0,926828619	0,949418389
AC138207.8	transcribed_unprocesse	ENSG00000266865	0,023854552	0,926937419	0,949482419
AC135178.5	lincRNA	ENSG00000269947	0,023504984	0,927133227	0,949635562
AC024941.2	antisense	ENSG00000281333	0,019439772	0,927647226	0,949782578
AC009961.1	lincRNA	ENSG00000226266	-0,015649895	0,927420417	0,949782578
RPL23AP74	processed_pseudogene	ENSG00000227694	0,023300324	0,927639294	0,949782578
HMGN1P38	processed_pseudogene	ENSG00000253954	0,021275213	0,927431251	0,949782578
PABPC1P4	processed_pseudogene	ENSG00000255642	-0,019694454	0,927531007	0,949782578
ZBTB46	protein_coding	ENSG00000130584	0,010163606	0,927477601	0,949782578
ELMOD1	protein_coding	ENSG00000110675	0,008689945	0,927587903	0,949782578
AKAP10	protein_coding	ENSG00000108599	-0,00386983	0,927558991	0,949782578
AL035411.1	processed_pseudogene	ENSG00000185839	0,021804904	0,928093177	0,95018235
SETP12	processed_pseudogene	ENSG00000251609	-0,02154621	0,928137168	0,95018235
PARP4P1	unprocessed_pseudoge	ENSG00000237917	0,023646814	0,928176664	0,95018235
AC023389.2	transcribed_processed_	ENSG00000265713	0,021862422	0,928366022	0,950328763
C4orf48	protein_coding	ENSG00000243449	0,015063387	0,928547955	0,950467563
EXOC5P1	processed_pseudogene	ENSG00000180673	-0,02018525	0,928599144	0,950472524
LDLR	protein_coding	ENSG00000130164	0,005211232	0,928755831	0,950585463
AL627171.2	lincRNA	ENSG00000282885	-0,017387933	0,92890971	0,950695517
PKIG	protein_coding	ENSG00000168734	0,006763362	0,929038112	0,950779486
AC022613.3	sense_intronic	ENSG00000259644	-0,022414672	0,929272952	0,95097237
C1QTNF3	protein_coding	ENSG00000082196	0,023130039	0,929335176	0,950988597
TMEM202-AS1	lincRNA	ENSG00000261423	0,01804017	0,929683194	0,951297261
EML4	protein_coding	ENSG00000143924	-0,004779687	0,929782597	0,951304054
DNLZ	protein_coding	ENSG00000213221	-0,019630252	0,929780482	0,951304054
AC104785.1	lincRNA	ENSG00000270720	-0,018856677	0,929961014	0,951439138
AL356968.2	processed_pseudogene	ENSG00000235105	-0,02079009	0,930011692	0,951443526
PHETA1	protein_coding	ENSG00000198324	-0,008915816	0,930218677	0,951607814
TMEM167B	protein_coding	ENSG00000215717	-0,004985633	0,930542196	0,951889146
SLC7A6OS	protein_coding	ENSG00000103061	-0,005570051	0,930586507	0,951889146
AC004069.1	lincRNA	ENSG00000251259	-0,022016384	0,930913676	0,952081355
RAB11FIP4	protein_coding	ENSG00000131242	0,019373292	0,930889372	0,952081355
AL138999.1	sense_intronic	ENSG00000277386	0,02024437	0,930857922	0,952081355
KCNIP2	protein_coding	ENSG00000120049	-0,020234051	0,931034578	0,95215223
AC006486.2	TEC	ENSG00000279539	0,022504166	0,931075823	0,95215223

GNL3LP1	processed_pseudogene	ENSG00000215032	-0,020577312	0,931437108	0,952426717
SH3BP5L	protein_coding	ENSG00000175137	-0,005836756	0,931402581	0,952426717
CLEC11A	protein_coding	ENSG00000105472	-0,014089741	0,93180438	0,952754765
AL954705.1	processed_pseudogene	ENSG00000231181	-0,020358509	0,932102093	0,953011661
SFTA1P	lincRNA	ENSG00000225383	-0,005366917	0,932404949	0,95327379
ERF	protein_coding	ENSG00000105722	-0,006573103	0,932586212	0,953411585
CNTLN	protein_coding	ENSG00000044459	-0,006089958	0,93273321	0,953497059
AC253576.2	sense_intronic	ENSG00000275426	-0,01735802	0,932762798	0,953497059
BX324167.2	antisense	ENSG00000279954	0,018831176	0,932888381	0,953532153
AC034102.1	processed_pseudogene	ENSG00000237493	-0,019927355	0,932890111	0,953532153
AC073136.1	processed_pseudogene	ENSG00000223559	0,021845444	0,932939828	0,953535449
DCAF8L2	protein_coding	ENSG00000189186	0,021417789	0,933089216	0,953640613
AC023886.2	processed_pseudogene	ENSG00000250966	0,020556331	0,933400189	0,953910901
AC109587.1	antisense	ENSG00000244513	0,019166436	0,933758309	0,954229343
AL590867.2	processed_pseudogene	ENSG00000218426	-0,010043627	0,93386094	0,954286676
HIGD1A	protein_coding	ENSG00000181061	0,00799278	0,934061512	0,954396533
AC134349.1	sense_intronic	ENSG00000256712	-0,020770664	0,934052485	0,954396533
RF00019	misc_RNA	ENSG00000201555	-0,019976236	0,934233108	0,954476764
RPL3P4	processed_pseudogene	ENSG00000232573	0,010151699	0,934228293	0,954476764
RPS15AP38	processed_pseudogene	ENSG00000237668	0,02012022	0,934344713	0,954543238
FAM86B2	protein_coding	ENSG00000145002	0,02146542	0,934417793	0,954551767
TMEM86B	protein_coding	ENSG00000180089	-0,019610308	0,934446143	0,954551767
AC090515.4	antisense	ENSG00000259353	-0,013373662	0,934580495	0,954593922
CYCSP24	processed_pseudogene	ENSG00000227666	0,01941969	0,934578575	0,954593922
RN7SL564P	misc_RNA	ENSG00000240606	0,020743207	0,934662568	0,954630211
HIPK4	protein_coding	ENSG00000160396	0,018786965	0,934977999	0,954904828
AC016542.1	sense_intronic	ENSG00000272627	-0,018379214	0,935224533	0,955109056
HMGN2P17	processed_pseudogene	ENSG00000238249	0,020806087	0,935302219	0,955140834
DHCR24-DT	antisense	ENSG00000233203	0,017589426	0,935383171	0,955175944
TLK2P2	processed_pseudogene	ENSG00000228828	-0,019451105	0,935459446	0,955206275
WWP2	protein_coding	ENSG00000198373	-0,00472593	0,935629653	0,955242153
GLCE	protein_coding	ENSG00000138604	-0,006198008	0,935634306	0,955242153
MIXL1	protein_coding	ENSG00000185155	-0,016664624	0,935622217	0,955242153
AL031666.2	antisense	ENSG00000267882	0,020776365	0,93575994	0,955322866
B3GNT7	protein_coding	ENSG00000156966	0,020603184	0,935826967	0,95534374
CEACAMP9	unprocessed_pseudoge	ENSG00000230529	0,01696012	0,936530973	0,956014844
ARL1	protein_coding	ENSG00000120805	0,005401131	0,936757085	0,95619807
AL118558.3	lincRNA	ENSG00000271780	0,017807199	0,936878034	0,956226349
SLC22A17	protein_coding	ENSG00000092096	0,018150344	0,936839158	0,956226349
TAPBP	protein_coding	ENSG00000231925	-0,005095285	0,936930507	0,95623232

ZNF595	protein_coding	ENSG00000272602	-0,008448942	0,937317934	0,956580128
ZNF32-AS2	antisense	ENSG00000230565	-0,017661495	0,937741123	0,956964396
UPK3B	protein_coding	ENSG00000243566	0,017736039	0,937919003	0,957014094
MANSC4	protein_coding	ENSG00000205693	0,01722733	0,937929806	0,957014094
AP1M2	protein_coding	ENSG00000129354	-0,006092573	0,937862306	0,957014094
AC092685.1	processed_pseudogene	ENSG00000232906	-0,019227332	0,938194098	0,957236143
AL662907.1	TEC	ENSG00000278997	0,017144622	0,938242256	0,95723766
AC087632.1	protein_coding	ENSG00000259316	0,016957844	0,939043791	0,958007767
KTN1-AS1	antisense	ENSG00000186615	0,009112829	0,939183664	0,958102807
OLFML2A	protein_coding	ENSG00000185585	-0,012513745	0,939818848	0,958703102
PLEKHG2	protein_coding	ENSG00000090924	-0,005993118	0,939996648	0,958836786
RPSAP3	transcribed_processed_	ENSG00000242952	0,017204404	0,940062799	0,958856575
AC004148.2	antisense	ENSG00000263272	-0,018672557	0,940223686	0,958972987
AKR1C2	protein_coding	ENSG00000151632	-0,016191609	0,940359647	0,959063966
VN1R81P	unprocessed_pseudoge	ENSG00000268357	0,017152949	0,940616654	0,959278384
PCDH18	protein_coding	ENSG00000189184	-0,018890812	0,940703163	0,959318907
AC007485.1	antisense	ENSG00000275710	0,017882601	0,940863426	0,959353508
AL590648.3	lincRNA	ENSG00000237980	-0,016997868	0,940896826	0,959353508
UCHL1	protein_coding	ENSG00000154277	0,003571551	0,940924191	0,959353508
LRP12	protein_coding	ENSG00000147650	-0,004920357	0,940794024	0,959353508
GHET1	lincRNA	ENSG00000281189	-0,018768396	0,940971207	0,959353753
FBXO46	protein_coding	ENSG00000177051	-0,0058665	0,941058387	0,959354367
ZNF887P	transcribed_unprocesse	ENSG00000267500	-0,018049234	0,941065359	0,959354367
SAMD13	protein_coding	ENSG00000203943	-0,013708519	0,941231219	0,959475761
AC022498.2	TEC	ENSG00000279891	0,018079917	0,941299896	0,95949808
BRD7P2	processed_pseudogene	ENSG00000184100	-0,017555967	0,94146502	0,959618704
AC004494.1	antisense	ENSG00000262312	-0,016135698	0,941646432	0,959708225
SIPA1L2	protein_coding	ENSG00000116991	0,016325532	0,941602681	0,959708225
LINC00687	lincRNA	ENSG00000228422	0,017671837	0,942110396	0,960125297
AC073861.1	processed_pseudogene	ENSG00000242299	0,010559666	0,942149279	0,960125297
AC119800.1	sense_intronic	ENSG00000237852	-0,015247441	0,942225409	0,960155172
AL359198.1	antisense	ENSG00000272817	-0,016783248	0,942421493	0,960225774
GALNT1	protein_coding	ENSG00000141429	0,003801645	0,942467262	0,960225774
FAT4	protein_coding	ENSG00000196159	-0,005085914	0,942423817	0,960225774
AC127070.4	TEC	ENSG00000278949	0,018529729	0,942481962	0,960225774
SNX18P9	processed_pseudogene	ENSG00000224185	-0,015680017	0,942934119	0,960543299
AC009403.2	processed_transcript	ENSG00000283128	-0,015687688	0,94291546	0,960543299
NEK9	protein_coding	ENSG00000119638	-0,003770894	0,942994207	0,960556801
ABCB6	protein_coding	ENSG00000115657	0,018629641	0,943331239	0,960852389
EPHA6	protein_coding	ENSG00000080224	-0,013712273	0,943578684	0,961056702

HMOX1	protein_coding	ENSG00000100292	-0,003062011	0,944323602	0,961767657
AC091429.1	processed_pseudogene	ENSG00000242477	-0,017234394	0,944497609	0,961897113
KLHL42	protein_coding	ENSG00000087448	-0,003596564	0,944648764	0,962003285
TNPO1	protein_coding	ENSG00000083312	0,004025302	0,944739265	0,96204768
ARHGEF35	protein_coding	ENSG00000213214	0,013169381	0,945015114	0,962280806
SYT3	protein_coding	ENSG00000213023	-0,015664037	0,94543505	0,96266062
FAM83C-AS1	antisense	ENSG00000235214	-0,014134643	0,945569432	0,962749655
AC007292.1	antisense	ENSG00000267980	-0,015622522	0,945704552	0,962782739
LINC01635	lincRNA	ENSG00000228397	-0,016945804	0,945803958	0,962782739
IDH1	protein_coding	ENSG00000138413	0,004304263	0,945808883	0,962782739
RPS27	protein_coding	ENSG00000177954	-0,007233979	0,945773152	0,962782739
SNORA24B	snoRNA	ENSG00000206903	-0,014636182	0,945836636	0,962782739
SKOR1	protein_coding	ENSG00000188779	-0,016778363	0,946204247	0,963109137
AL669831.5	lincRNA	ENSG00000237491	-0,016787221	0,946306422	0,963120955
AC093591.1	processed_pseudogene	ENSG00000244021	-0,0160461	0,946348741	0,963120955
PUS7L	protein_coding	ENSG00000129317	0,004305476	0,946356734	0,963120955
ANO4	protein_coding	ENSG00000151572	-0,004020458	0,946686138	0,96340839
AL031673.1	antisense	ENSG00000278383	-0,016823215	0,946807807	0,963484402
AL139288.1	TEC	ENSG00000279306	-0,013832076	0,947072504	0,963705946
MRPS33	protein_coding	ENSG00000090263	-0,004510959	0,947126386	0,963712962
NKAP	protein_coding	ENSG00000101882	0,003127944	0,94720345	0,963743563
TMEM8B	protein_coding	ENSG00000137103	-0,012347242	0,947352541	0,963847443
RF00019	misc_RNA	ENSG00000199366	-0,015782269	0,947583199	0,963986479
AARSD1	protein_coding	ENSG00000266967	0,015462452	0,947579579	0,963986479
LINC00910	lincRNA	ENSG00000188825	0,009781381	0,947694178	0,964041795
TMEM30A	protein_coding	ENSG00000112697	-0,003170085	0,94773158	0,964041795
CHRNB1	protein_coding	ENSG00000170175	0,003446511	0,948030656	0,964298192
PTP4A2	protein_coding	ENSG00000184007	0,003696258	0,948080823	0,964301397
MYO5A	protein_coding	ENSG00000197535	-0,003876573	0,948208621	0,964335737
AC241952.1	transcribed_unprocessed	ENSG00000275131	0,006046199	0,948194122	0,964335737
RNU6-759P	snRNA	ENSG00000252549	-0,014047048	0,948268494	0,964348811
AC093510.2	sense_overlapping	ENSG00000260871	0,013702531	0,948394179	0,964428807
LINC01771	lincRNA	ENSG00000225675	0,016670712	0,94869144	0,964635438
CCDC126	protein_coding	ENSG00000169193	0,00883299	0,948669367	0,964635438
FNBP4	protein_coding	ENSG00000109920	0,00328374	0,948948972	0,964849465
MYL12BP1	processed_pseudogene	ENSG00000260490	0,01675359	0,949003183	0,964856753
GATD3A	protein_coding	ENSG00000160221	-0,014550275	0,949303763	0,965102718
GATD3A	protein_coding	ENSG00000160221	-0,014550275	0,949303763	0,965102718
NT5C3AP1	transcribed_processed_	ENSG00000213492	0,015674353	0,949339216	0,965102718
PIP4P1	protein_coding	ENSG00000165782	-0,004171457	0,949711167	0,965432991

GLIPR1L1	protein_coding	ENSG00000173401	-0,013695718	0,949965651	0,965643828
TMSB15B	protein_coding	ENSG00000269226	-0,014934062	0,950165716	0,965799327
RNASEH1	protein_coding	ENSG00000171865	-0,003003364	0,950310761	0,96589889
TRADD	protein_coding	ENSG00000102871	-0,005972109	0,950358865	0,965899917
MARCH7	protein_coding	ENSG00000136536	0,004751599	0,950433561	0,965927969
AC020658.4	sense_intronic	ENSG00000273786	-0,014314553	0,950480809	0,965928124
LRRIQ1	protein_coding	ENSG00000133640	-0,010072291	0,950665861	0,966068315
ZNF45	protein_coding	ENSG00000124459	0,003145653	0,950803127	0,966112071
PRXL2A	protein_coding	ENSG00000122378	-0,002820437	0,950787093	0,966112071
ANKRD18DP	transcribed_unprocesse	ENSG00000226435	-0,014590186	0,950907102	0,966169853
ZNF430	protein_coding	ENSG00000118620	-0,006076843	0,95108027	0,966297931
AL033519.2	processed_pseudogene	ENSG00000219023	-0,015401579	0,951141596	0,96631237
AP000487.1	antisense	ENSG00000246889	0,014630014	0,951344535	0,966470673
AC004839.1	antisense	ENSG00000272854	0,015289522	0,951524903	0,966606031
AC007066.2	sense_overlapping	ENSG00000261094	-0,013166211	0,951574421	0,966608457
METAP1D	protein_coding	ENSG00000172878	0,005853904	0,951721811	0,966710298
LINC02384	lincRNA	ENSG00000251301	0,015806144	0,951857713	0,96671411
EEF1A1P19	processed_pseudogene	ENSG00000249855	0,013003769	0,951929581	0,96671411
ZFP28	protein_coding	ENSG00000196867	0,004586325	0,951961233	0,96671411
APBB1	protein_coding	ENSG00000166313	-0,004726517	0,951916711	0,96671411
AF274858.1	transcribed_processed_	ENSG00000260081	0,015549923	0,951884791	0,96671411
DMC1	protein_coding	ENSG00000100206	0,011526948	0,952054522	0,966760979
AL158050.1	processed_pseudogene	ENSG00000232702	0,013848426	0,952115714	0,966775251
CHCHD3P3	processed_pseudogene	ENSG00000214973	-0,014956371	0,952440129	0,967008913
GTF2IP7	transcribed_unprocesse	ENSG00000227038	0,012101031	0,952411472	0,967008913
LINC01833	lincRNA	ENSG00000259439	0,008542406	0,952823187	0,967349944
AL031963.3	antisense	ENSG00000272277	-0,014754809	0,952894788	0,967374751
AC093627.4	lincRNA	ENSG00000240859	-0,013412517	0,952957387	0,967390418
AC007000.1	processed_pseudogene	ENSG00000213542	-0,013725201	0,953037953	0,967411134
SPICE1	protein_coding	ENSG00000163611	0,00422637	0,953119297	0,967411134
IGSF9B	protein_coding	ENSG00000080854	-0,008874545	0,953094556	0,967411134
FTLP2	processed_pseudogene	ENSG00000232368	-0,012163878	0,953354094	0,967601567
OLA1P1	processed_pseudogene	ENSG00000213790	-0,014302695	0,953515267	0,967646115
TAS2R31	protein_coding	ENSG00000256436	0,014880417	0,95344903	0,967646115
HS3ST5	protein_coding	ENSG00000249853	-0,014207862	0,953539524	0,967646115
BX537318.1	lincRNA	ENSG00000273145	-0,012371999	0,953775223	0,967837415
CMC1	protein_coding	ENSG00000187118	0,004324431	0,954048343	0,968066666
AC078846.1	lincRNA	ENSG00000273329	-0,009881407	0,954315727	0,968264545
PIGN	protein_coding	ENSG00000197563	-0,002881041	0,954337776	0,968264545
COPZ2	protein_coding	ENSG00000005243	0,005494776	0,954426727	0,968306894

SLC4A3	protein_coding	ENSG00000114923	0,00587789	0,954480819	0,968313875
PLS3	protein_coding	ENSG00000102024	0,001121105	0,954655525	0,96844321
ACSF3	protein_coding	ENSG00000176715	0,003461438	0,954727771	0,968468598
SVIL-AS1	transcribed_unprocesse	ENSG00000224597	0,003007011	0,955000664	0,968697508
AL445250.1	lincRNA	ENSG00000225096	-0,014138421	0,955098074	0,968714953
AC084876.2	TEC	ENSG00000280426	-0,01416868	0,955112325	0,968714953
KLHDC2	protein_coding	ENSG00000165516	-0,00284489	0,955441821	0,969001224
TREX2	protein_coding	ENSG00000183479	-0,013697593	0,955549196	0,969062204
MPRIP	protein_coding	ENSG00000133030	-0,002728451	0,955613521	0,969079521
CYCSP34	processed_pseudogene	ENSG00000176268	-0,011991401	0,955732269	0,969152024
AC011445.1	sense_overlapping	ENSG00000268262	0,012674601	0,955991526	0,969366994
CERKL	protein_coding	ENSG00000188452	0,010615273	0,956048933	0,96937728
AC008555.7	TEC	ENSG00000280106	-0,013320823	0,956180958	0,969463218
POLR2J	protein_coding	ENSG00000005075	0,004026082	0,956323143	0,969559449
GOLGA8H	protein_coding	ENSG00000261794	-0,011248942	0,956383138	0,969572347
ABHD12	protein_coding	ENSG00000100997	-0,003155658	0,956557281	0,969605702
SNX15	protein_coding	ENSG00000110025	-0,013301663	0,956557863	0,969605702
AC145423.2	sense_intronic	ENSG00000274427	0,01246519	0,956498404	0,969605702
SNORA30	snoRNA	ENSG00000206755	-0,011516485	0,956701261	0,969703132
C1RL	protein_coding	ENSG00000139178	0,009413208	0,956946789	0,969808214
CCNDBP1	protein_coding	ENSG00000166946	0,004121622	0,956906085	0,969808214
ZNF807	unprocessed_pseudoge	ENSG00000248830	-0,011832832	0,956896611	0,969808214
AC020910.5	TEC	ENSG00000279329	0,008457537	0,957090658	0,969906092
SEC16B	protein_coding	ENSG00000120341	0,01272683	0,957258894	0,970028652
SEC16B	protein_coding	ENSG00000120341	0,01272683	0,957258894	0,970028652
Z97198.1	lincRNA	ENSG00000224000	0,01137557	0,957524716	0,97018062
FNBP1P1	processed_pseudogene	ENSG00000257800	-0,008522562	0,95755077	0,97018062
INTS1	protein_coding	ENSG00000164880	-0,004773516	0,957495511	0,97018062
GPR19	protein_coding	ENSG00000183150	-0,008684326	0,957690028	0,970273784
KRTAP1-5	protein_coding	ENSG00000221852	-0,012529115	0,957926382	0,970465304
AC079354.2	antisense	ENSG00000231903	-0,012284618	0,958028552	0,970520873
CCDC87	protein_coding	ENSG00000182791	-0,010310396	0,958114286	0,970559787
RHOV	protein_coding	ENSG00000104140	-0,009152431	0,958175462	0,970573821
GPSM3	protein_coding	ENSG00000213654	-0,007246592	0,958444831	0,97079873
AL359715.3	lincRNA	ENSG00000272129	0,013026941	0,958603702	0,9709117
SNORD63	snoRNA	ENSG00000206989	-0,012884273	0,958941874	0,971206253
AL022341.1	antisense	ENSG00000228201	0,013364354	0,95900674	0,97122399
LINC00449	antisense	ENSG00000203441	-0,012808622	0,95953277	0,97170874
LRRC73	protein_coding	ENSG00000204052	-0,012788778	0,959582178	0,971710796
CSAG4	transcribed_unprocesse	ENSG00000242599	-0,010785081	0,959689787	0,971771787

NDUFB8	protein_coding	ENSG00000166136	0,004752818	0,959758701	0,97179359
AC022613.1	antisense	ENSG00000256802	0,006722719	0,960039392	0,972029814
AC009902.2	antisense	ENSG00000254027	-0,011428109	0,960088643	0,972031695
ATP6AP1L	protein_coding	ENSG00000205464	0,005723842	0,960141745	0,972037474
LINC01754	lincRNA	ENSG00000224565	0,010466744	0,960440162	0,972291596
RINT1	protein_coding	ENSG00000135249	-0,003779812	0,960542368	0,972347069
PTPRQ	protein_coding	ENSG00000139304	0,012585225	0,960634042	0,972391877
AC073957.2	antisense	ENSG00000257607	-0,01150489	0,960682646	0,972393084
IMMP1L	protein_coding	ENSG00000148950	0,003940586	0,961070516	0,972737677
ARHGAP12	protein_coding	ENSG00000165322	0,002327966	0,961161522	0,972781782
ZNF839	protein_coding	ENSG00000022976	-0,004376971	0,961228974	0,972802046
AC124016.1	antisense	ENSG00000272677	0,012770625	0,961379072	0,972874679
U52112.1	processed_transcript	ENSG00000273769	0,010130255	0,961395612	0,972874679
GLTP	protein_coding	ENSG00000139433	-0,001928741	0,961599917	0,973014163
CLTCL1	protein_coding	ENSG00000070371	-0,003634888	0,961628331	0,973014163
DUXAP9	transcribed_processed_	ENSG00000225210	0,005193086	0,961784113	0,973123781
AC092653.1	lincRNA	ENSG00000273245	0,01089588	0,9620236	0,973270065
STK32B	protein_coding	ENSG00000152953	-0,00229164	0,962008063	0,973270065
HFM1	protein_coding	ENSG00000162669	0,012549745	0,962333965	0,973536038
NEMF	protein_coding	ENSG00000165525	0,002878491	0,962431518	0,973586705
TNPO1P1	processed_pseudogene	ENSG00000225423	0,010448627	0,962570587	0,973679362
API5	protein_coding	ENSG00000166181	0,002337425	0,962659246	0,973721021
ZFYVE16	protein_coding	ENSG00000039319	-0,002793022	0,962750335	0,973765133
PHF5CP	processed_pseudogene	ENSG00000267178	0,010447216	0,962809298	0,97377675
FO393411.1	processed_pseudogene	ENSG00000180211	-0,01073529	0,963041425	0,973948943
CHD4	protein_coding	ENSG00000111642	0,003397475	0,963687112	0,974520396
COMMD6	protein_coding	ENSG00000188243	0,003641788	0,963755434	0,974541437
ST13P19	processed_pseudogene	ENSG00000228110	-0,010768302	0,964167305	0,974908428
CBLL1	protein_coding	ENSG00000105879	-0,002805201	0,964245782	0,974908428
GRN	protein_coding	ENSG00000030582	-0,003344821	0,964260964	0,974908428
EIF4A1P4	processed_pseudogene	ENSG00000257790	0,011791637	0,964363921	0,974916405
AL023806.3	TEC	ENSG00000280184	0,011087934	0,964335446	0,974916405
AL353708.3	lincRNA	ENSG00000272906	-0,010840588	0,964511209	0,975017245
CRNKL1	protein_coding	ENSG00000101343	-0,00214171	0,964867891	0,975329741
CFAP161	protein_coding	ENSG00000156206	-0,01058361	0,965093676	0,975502724
AL022394.1	transcribed_processed_	ENSG00000272407	-0,010220925	0,965134144	0,975502724
MDGA1	protein_coding	ENSG00000112139	0,009510971	0,965475372	0,975770847
MRPS36	protein_coding	ENSG00000134056	0,002694407	0,965494567	0,975770847
HDGFL3	protein_coding	ENSG00000166503	0,002315644	0,965553134	0,975781955
CCDC150	protein_coding	ENSG00000144395	0,003997515	0,965678944	0,975861014

ASH1L-AS1	antisense	ENSG00000235919	-0,008744166	0,965889787	0,975977906
HSDL2	protein_coding	ENSG00000119471	-0,002427201	0,965857317	0,975977906
STRN	protein_coding	ENSG00000115808	0,002582157	0,966199913	0,976243176
FANK1	protein_coding	ENSG00000203780	-0,010097371	0,966248622	0,976244298
AL513550.1	antisense	ENSG00000228506	-0,006077703	0,966394739	0,976321136
BMPR1AP2	processed_pseudogene	ENSG00000232460	-0,009223287	0,966419878	0,976321136
FRG1EP	unprocessed_pseudoge	ENSG00000282995	-0,010281638	0,966481601	0,976335401
C1DP1	processed_pseudogene	ENSG00000231245	-0,008362246	0,966659222	0,976424113
AL589843.2	transcribed_processed_	ENSG00000233820	0,008510474	0,966664633	0,976424113
BX664727.3	processed_transcript	ENSG00000240240	-0,010001036	0,966719465	0,976431411
KCNJ2-AS1	antisense	ENSG00000267365	-0,007920677	0,966804098	0,976468806
DDX1	protein_coding	ENSG00000079785	0,002181562	0,966915415	0,976533148
CLP1	protein_coding	ENSG00000172409	-0,002338112	0,967292358	0,976865738
AC063947.2	unprocessed_pseudoge	ENSG00000258033	0,0099281	0,96735252	0,976878394
KCNQ5-IT1	sense_intronic	ENSG00000233844	-0,009442833	0,967459256	0,97693808
CAP1P2	processed_pseudogene	ENSG00000232004	0,010783816	0,967616382	0,977048641
PSMD14	protein_coding	ENSG00000115233	0,002508932	0,967980067	0,977367754
APH1B	protein_coding	ENSG00000138613	-0,002142256	0,968067584	0,977408002
ZC3H11B	protein_coding	ENSG00000215817	-0,008865897	0,968467699	0,977763844
AC091133.4	processed_pseudogene	ENSG00000251550	0,00869705	0,968698316	0,977900116
ADH5	protein_coding	ENSG00000197894	-0,002009298	0,968735391	0,977900116
SLC25A28	protein_coding	ENSG00000155287	-0,002714294	0,968840705	0,977900116
AC118344.1	sense_intronic	ENSG00000205041	-0,009620077	0,968771337	0,977900116
AC105235.1	TEC	ENSG00000279786	0,010221778	0,968841071	0,977900116
AC112503.2	lincRNA	ENSG00000273454	-0,007690966	0,96899564	0,978008001
SOX9	protein_coding	ENSG00000125398	-0,002036111	0,969158347	0,978124087
ADGRB1	protein_coding	ENSG00000181790	-0,007431872	0,969241268	0,978159642
PTGES3L	protein_coding	ENSG00000267060	0,009120379	0,969433852	0,978257727
MBNL1	protein_coding	ENSG00000152601	0,003978386	0,969411262	0,978257727
GOLGA7B	protein_coding	ENSG00000155265	0,006322326	0,969497862	0,978274188
DDX47	protein_coding	ENSG00000213782	-0,005028061	0,970104883	0,978838548
RPSAP13	processed_pseudogene	ENSG00000233924	-0,008713673	0,970218133	0,978904659
BACE1-AS	processed_transcript	ENSG00000278768	-0,006823752	0,970353068	0,978992643
DAP3P2	processed_pseudogene	ENSG00000228507	-0,009033961	0,97049316	0,979085819
SHLD2P1	transcribed_unprocesse	ENSG00000165874	0,009057999	0,970622158	0,979167796
WFS1	protein_coding	ENSG00000109501	-0,002438579	0,970696639	0,979194771
AP000704.1	lincRNA	ENSG00000224790	0,009291996	0,971244582	0,979602969
TRPC4AP	protein_coding	ENSG00000100991	-0,001370216	0,971204474	0,979602969
TRIT1	protein_coding	ENSG00000043514	-0,002321668	0,971186898	0,979602969
WASH8P	unprocessed_pseudoge	ENSG00000226210	0,005798511	0,97162515	0,979938622

KRT18P11	processed_pseudogene	ENSG00000215089	0,009251355	0,971846662	0,980017456
ENY2	protein_coding	ENSG00000120533	0,002370016	0,971836602	0,980017456
SDF4	protein_coding	ENSG00000078808	0,001896228	0,971814215	0,980017456
AP001453.2	antisense	ENSG00000256940	0,003802534	0,971970136	0,980045597
AC010973.2	antisense	ENSG00000244151	-0,008115312	0,971965103	0,980045597
VMO1	protein_coding	ENSG00000182853	0,009257178	0,972281096	0,980310947
TCEAL3	protein_coding	ENSG00000196507	-0,001918457	0,972870838	0,980857341
RPL5P23	processed_pseudogene	ENSG00000240395	-0,008083902	0,973196819	0,981124638
RPAP1	protein_coding	ENSG00000103932	-0,002176808	0,973231631	0,981124638
HCG4	unprocessed_pseudoge	ENSG00000176998	-0,008138758	0,973469731	0,981316435
ARMH3	protein_coding	ENSG00000120029	-0,00208948	0,973808686	0,981609876
DDX24	protein_coding	ENSG00000089737	0,001169335	0,973913521	0,981667305
ACTG1P24	processed_pseudogene	ENSG00000226359	-0,007775283	0,974075983	0,98178281
AL358942.1	processed_pseudogene	ENSG00000225580	-0,007893514	0,974494229	0,9821561
ZNF446	protein_coding	ENSG00000083838	0,003400293	0,974689174	0,982304308
RECQL5	protein_coding	ENSG00000108469	0,002617329	0,974856807	0,982424976
RPL21P5	processed_pseudogene	ENSG00000258640	-0,006787774	0,975130293	0,982652302
C22orf15	protein_coding	ENSG00000169314	-0,006335422	0,975223141	0,982697584
RN7SL146P	misc_RNA	ENSG00000240106	0,006218297	0,975376708	0,982804042
AC005498.2	lincRNA	ENSG00000267421	0,007891841	0,975904343	0,983287389
RPS26P47	processed_pseudogene	ENSG00000234354	-0,006641309	0,97613001	0,983441284
Z83843.1	sense_intronic	ENSG00000271533	0,006555706	0,976152981	0,983441284
HOXC8	protein_coding	ENSG00000037965	0,007653319	0,976450004	0,983692204
AC116158.1	antisense	ENSG00000259326	0,007567292	0,97688029	0,984077345
ARPC3	protein_coding	ENSG00000111229	-0,001861556	0,976944121	0,984093311
DAND5	protein_coding	ENSG00000179284	-0,006982903	0,977195203	0,984249551
AL132780.4	TEC	ENSG00000279656	-0,006920579	0,97715659	0,984249551
GNAI2	protein_coding	ENSG00000114353	-0,00128322	0,977336842	0,984343873
AC011450.1	lincRNA	ENSG00000197813	0,007456961	0,977394286	0,98435339
CCDC7	protein_coding	ENSG00000216937	-0,002640662	0,977489134	0,984400576
AL117350.1	antisense	ENSG00000237481	-0,00666248	0,977629184	0,984493276
ELFN2	protein_coding	ENSG00000166897	-0,002953992	0,977728813	0,984545265
AC138028.4	antisense	ENSG00000260121	-0,006629148	0,977961844	0,984633355
EEF1A1P6	processed_pseudogene	ENSG00000233476	-0,004243128	0,977885977	0,984633355
METTL8	protein_coding	ENSG00000123600	-0,001347655	0,97802514	0,984633355
ETV1	protein_coding	ENSG00000006468	-0,001855749	0,97805633	0,984633355
RREB1	protein_coding	ENSG00000124782	-0,002275878	0,978030298	0,984633355
AL359237.1	lincRNA	ENSG00000258807	0,007066501	0,978297542	0,984797646
LCTL	protein_coding	ENSG00000188501	-0,006055684	0,978315555	0,984797646
GIT1	protein_coding	ENSG00000108262	0,002112057	0,978638077	0,985025615

SH3GL2	protein_coding	ENSG00000107295	-0,006352775	0,978617447	0,985025615
AC007292.2	antisense	ENSG00000269318	-0,006549636	0,978840534	0,985084364
RN7SL5P	misc_RNA	ENSG00000265735	0,004731383	0,978838541	0,985084364
NFKBIL1	protein_coding	ENSG00000204498	-0,002018023	0,978766855	0,985084364
RN7SL559P	misc_RNA	ENSG00000240750	0,005745106	0,979313284	0,985511774
HIPK2	protein_coding	ENSG00000064393	0,00190402	0,979796306	0,985860057
NKAPD1	protein_coding	ENSG00000150776	-0,00111921	0,979803579	0,985860057
DNAJC8	protein_coding	ENSG00000126698	-0,00138962	0,979715959	0,985860057
RHPN1-AS1	antisense	ENSG00000254389	0,006467711	0,980293328	0,986256065
PTOV1-AS2	antisense	ENSG00000269352	0,005546306	0,980269634	0,986256065
C3orf35	processed_transcript	ENSG00000198590	-0,005169196	0,980549739	0,986417263
TRDMT1	protein_coding	ENSG00000107614	0,002267577	0,980513646	0,986417263
STXBP2	protein_coding	ENSG00000076944	-0,00217953	0,981076089	0,986898356
AC010327.4	lincRNA	ENSG00000267649	-0,005625334	0,981350987	0,987126471
RN7SL552P	misc_RNA	ENSG00000239466	0,005530548	0,981718368	0,987399163
ZNF280C	protein_coding	ENSG00000056277	-0,001367739	0,981686109	0,987399163
TXNDC12	protein_coding	ENSG00000117862	-0,00116521	0,982092701	0,987727225
SHISA5	protein_coding	ENSG00000164054	0,000100838	0,982665672	0,9882533
RPL7AP50	processed_pseudogene	ENSG00000213609	-0,005094614	0,982773035	0,988261145
DISC1	protein_coding	ENSG00000162946	-0,005158735	0,982816313	0,988261145
RPL13P12	processed_pseudogene	ENSG00000215030	0,002874326	0,983436438	0,988833247
AC015883.1	TEC	ENSG00000279281	-0,004797386	0,983481687	0,988833247
AC125437.1	sense_intronic	ENSG00000267655	-0,005020036	0,983572338	0,988875914
AL670729.1	antisense	ENSG00000231563	-0,004778679	0,983779061	0,989003879
SNORA19	snoRNA	ENSG00000207468	-0,0049058	0,983796057	0,989003879
AL359091.4	antisense	ENSG00000272696	0,004911775	0,983916322	0,989027826
CACNB4	protein_coding	ENSG00000182389	-0,004121665	0,9838886	0,989027826
PTMAP9	processed_pseudogene	ENSG00000198134	0,004533391	0,984009524	0,989073038
AC011479.3	sense_intronic	ENSG00000269653	-0,004408086	0,984136285	0,989151975
AC092017.1	processed_pseudogene	ENSG00000226986	0,005004856	0,984406167	0,989336214
VAX2	protein_coding	ENSG00000116035	-0,003916876	0,984416063	0,989336214
AC078795.2	antisense	ENSG00000270096	-0,004653282	0,984519476	0,989391664
ZNF101	protein_coding	ENSG00000181896	-0,001184371	0,984605325	0,989429456
AC004834.1	antisense	ENSG00000284523	-0,004533077	0,984841348	0,98961815
AC090809.1	lincRNA	ENSG00000253363	-0,003525403	0,985145621	0,989826909
AL353764.1	lincRNA	ENSG00000233081	-0,004212792	0,985102684	0,989826909
AL049597.1	processed_pseudogene	ENSG00000230721	-0,004211276	0,985357256	0,989991051
ZNF564	protein_coding	ENSG00000249709	0,005087521	0,985409852	0,989995398
ACTBP2	processed_pseudogene	ENSG00000213763	0,004919683	0,985481965	0,990019353
GPR137	protein_coding	ENSG00000173264	-0,001398771	0,985553056	0,990042277

AP000266.1	antisense	ENSG00000232623	-0,004102374	0,985661441	0,990054173
GATAD1	protein_coding	ENSG00000157259	0,001029658	0,985628752	0,990054173
FXYD7	protein_coding	ENSG00000221946	-0,00399967	0,985728929	0,990073473
ALDOA	protein_coding	ENSG00000149925	-0,000928389	0,98578006	0,990076345
TEX29	protein_coding	ENSG00000153495	-0,00414651	0,985832955	0,990080986
AC079336.6	TEC	ENSG00000279781	-0,003895518	0,986451946	0,990654135
FP671120.5	lincRNA	ENSG00000281383	0,00265319	0,986622511	0,990679903
AC010654.1	lincRNA	ENSG00000277369	-0,003941771	0,986567358	0,990679903
PPIL6	protein_coding	ENSG00000185250	-0,003973832	0,986620331	0,990679903
HMGA1P4	antisense	ENSG00000234705	0,004403075	0,986808071	0,990817719
DMBT1	protein_coding	ENSG00000187908	-0,003309684	0,986992852	0,990954738
EEF1A1P8	processed_pseudogene	ENSG00000223529	-0,003515771	0,98710719	0,990972514
BBS4	protein_coding	ENSG00000140463	-0,001042614	0,987106272	0,990972514
PHF1	protein_coding	ENSG00000112511	-0,001235113	0,98733675	0,991154458
FBXO10	protein_coding	ENSG00000147912	0,002415857	0,987536962	0,991306925
FAHD2A	protein_coding	ENSG00000115042	0,001155826	0,98800593	0,991704234
FRG1BP	unprocessed_pseudoge	ENSG00000149531	0,001372893	0,988029465	0,991704234
PRKRIP1	protein_coding	ENSG00000128563	0,001076804	0,988087661	0,991714114
AC144836.1	lincRNA	ENSG00000262213	-0,003430642	0,988296028	0,991874708
CDK5R2	protein_coding	ENSG00000171450	0,003944946	0,988504583	0,992035474
TOMM34	protein_coding	ENSG00000025772	0,000805978	0,988616928	0,992099677
AC138207.4	sense_intronic	ENSG00000264456	0,004052717	0,98869421	0,992128687
HIST2H2AA3	protein_coding	ENSG00000203812	-0,00298438	0,988807619	0,992168285
PACS2	protein_coding	ENSG00000179364	-0,000957721	0,988898242	0,992187797
ADAM11	protein_coding	ENSG00000073670	0,001613822	0,989147738	0,992328608
FAM110A	protein_coding	ENSG00000125898	0,001570317	0,989155512	0,992328608
AC021945.1	TEC	ENSG00000279347	-0,003075416	0,989183734	0,992328608
CUEDC1	protein_coding	ENSG00000180891	-0,000922712	0,989549956	0,992647442
AC091057.3	lincRNA	ENSG00000269930	-0,002983177	0,989788292	0,992692317
CREB3L2	protein_coding	ENSG00000182158	-0,000754924	0,989786643	0,992692317
TAF1C	protein_coding	ENSG00000103168	-0,001158	0,989762239	0,992692317
TRIM80P	unitary_pseudogene	ENSG00000232724	0,003349544	0,989714977	0,992692317
MORF4L2-AS1	antisense	ENSG00000231154	-0,002910671	0,990026014	0,992882184
LINC00632	antisense	ENSG00000203930	-0,002640719	0,990419941	0,993131563
EIF3J-DT	lincRNA	ENSG00000179523	0,001133106	0,990411519	0,993131563
AL442125.2	sense_intronic	ENSG00000276916	-0,002817738	0,990355996	0,993131563
TGFBR1	protein_coding	ENSG00000106799	0,000598555	0,99055251	0,993215936
AL157813.1	sense_intronic	ENSG00000274929	-0,002737417	0,990650599	0,993265731
HM13-IT1	sense_intronic	ENSG00000235313	-0,002633615	0,990833572	0,993400624
AL391834.1	antisense	ENSG00000272842	0,002850074	0,991038144	0,993460036

PSMC1P1	processed_pseudogene	ENSG00000241506	0,002094974	0,991033102	0,993460036
FOXP1-IT1	sense_intronic	ENSG00000242094	0,003186579	0,990944988	0,993460036
LINC02590	lincRNA	ENSG00000233978	0,002915075	0,991287529	0,993661465
AC124016.2	lincRNA	ENSG00000273156	0,003026238	0,99166001	0,993813232
AL512598.1	lincRNA	ENSG00000273107	0,002868425	0,991826574	0,993813232
JCAD	protein_coding	ENSG00000165757	0,001616195	0,991698065	0,993813232
MAIP1	protein_coding	ENSG00000162972	0,000728098	0,991621102	0,993813232
XAB2	protein_coding	ENSG00000076924	0,000692783	0,991631515	0,993813232
PTCD2	protein_coding	ENSG00000049883	0,000647797	0,991794163	0,993813232
AC007637.1	sense_overlapping	ENSG00000256139	-0,002330151	0,991801686	0,993813232
DGCR5	transcribed_unprocesse	ENSG00000237517	-0,001980773	0,991607735	0,993813232
CATSPER3	protein_coding	ENSG00000152705	0,002687192	0,99215396	0,994092707
CTSZ	protein_coding	ENSG00000101160	-0,000145463	0,992414933	0,994305616
MPP1	protein_coding	ENSG00000130830	-0,000418665	0,992751198	0,994563226
SCARNA8	scaRNA	ENSG00000251733	-0,001993297	0,992769036	0,994563226
LIPT2	protein_coding	ENSG00000175536	-0,00135607	0,993216791	0,994963191
AC138356.2	processed_pseudogene	ENSG00000233170	0,00219891	0,993467139	0,995019583
GATAD2A	protein_coding	ENSG00000167491	-0,000436202	0,993388172	0,995019583
INF2	protein_coding	ENSG00000203485	-0,000575083	0,99343827	0,995019583
AC008740.1	unprocessed_pseudoge	ENSG00000257403	-0,00182078	0,993404811	0,995019583
NDUFA3	protein_coding	ENSG00000170906	0,000598294	0,993926619	0,995431171
AC092164.1	antisense	ENSG00000226833	-0,001600358	0,994079842	0,995536014
MPPE1	protein_coding	ENSG00000154889	0,000899645	0,994265757	0,995673584
RF00019	misc_RNA	ENSG00000199331	-0,001318991	0,994747362	0,996107235
SYCP2	protein_coding	ENSG00000196074	-0,001283144	0,995041878	0,996353509
AC079336.1	sense_intronic	ENSG00000263717	0,00126943	0,995309806	0,996573136
HLA-F-AS1	processed_transcript	ENSG00000214922	-0,000928871	0,995690856	0,996906003
TMEM179B	protein_coding	ENSG00000185475	0,000407585	0,995753506	0,996920063
HRCT1	protein_coding	ENSG00000196196	-0,000978957	0,99592124	0,997039324
MKRN3	protein_coding	ENSG00000179455	-0,000958735	0,996045551	0,997115104
COPS9	protein_coding	ENSG00000172428	0,000487003	0,996233978	0,997221739
AC000120.1	sense_overlapping	ENSG00000243107	0,001311686	0,996249314	0,997221739
SNX27	protein_coding	ENSG00000143376	0,000289959	0,996504773	0,997428768
AC018645.2	lincRNA	ENSG00000273014	-0,000561723	0,996568884	0,997444262
CFAP36	protein_coding	ENSG00000163001	-0,000133322	0,99763436	0,998413229
CPT1B	protein_coding	ENSG00000205560	-0,000384893	0,997634231	0,998413229
KRTCAP3	protein_coding	ENSG00000157992	-0,000440411	0,997698296	0,998428497
AL133410.1	lincRNA	ENSG00000227388	0,000972133	0,997815579	0,998448433
RPL26P30	transcribed_processed_	ENSG00000236264	-0,000440967	0,997775473	0,998448433
AC013400.1	bidirectional_promoter_	ENSG00000271991	0,000837515	0,997998564	0,998582815

RN7SL525P	misc_RNA	ENSG00000264910	0,000738158	0,998099459	0,998635051
KRT8P39	processed_pseudogene	ENSG00000233560	0,000678556	0,998441464	0,998879783
EIF2S3B	protein_coding	ENSG00000180574	-0,000176457	0,998396139	0,998879783
HSPA8P1	processed_pseudogene	ENSG00000234176	0,000774719	0,998519637	0,998909265
ATP5MDP1	processed_pseudogene	ENSG00000231549	0,000566831	0,998680939	0,999021902
CDKL1	protein_coding	ENSG00000100490	0,000149315	0,999263163	0,999555574
AP005136.2	sense_intronic	ENSG00000266783	0,0000672	0,999415624	0,999659325
C2orf74	protein_coding	ENSG00000237651	0,000420469	0,999510408	0,999694112
PRTFDC1	protein_coding	ENSG00000099256	0,000115777	0,999547887	0,999694112
AC009269.5	sense_intronic	ENSG00000274297	0,000142493	0,999692513	0,999790006
AC010186.3	sense_intronic	ENSG00000257027	0,000223593	0,999805086	0,999843559
