# Supplementary material for: Zika virus targets the human thymic epithelium
Source: Sci Rep. 2020 Jan 28;10:1378. doi: 10.1038/s41598-020-58135-y (PMC6987159; doi:10.1038/s41598-020-58135-y)
Supplement: Supplementary file 3 — Supplementary Table 2. [file 41598_2020_58135_MOESM3_ESM.pdf]

Zika virus targets the human thymic epithelium

Carolina V. Messias, Guilherme Loss-Morais, Joseane Biso de Carvalho, Mariela N. González,
Daniela P. Cunha, Zilton Vasconcelos, Luis W. P. Arge, Désio A. Farias-de-Oliveira, Alexandra L. Gerber,
Elyzabeth A. Portari, Nilma Ferreira, Lidiane M. S. Raphael, Myrna C. Bonaldo, Ingo Riederer,
Maria E. Lopes Moreira, Vinicius Cotta-de-Almeida, Ana T. R. Vasconcelos, Daniella A. Mendes-da-Cruz and
Wilson Savino

Supplemental Table 2: Reactome enrichment analysis from up regulated genes sorted by significance

ID	Description	GeneRatio	BgRatio	pvalue	p.adjust	qvalue	geneID	Count
R-HSA-6783783	Interleukin-10 signaling	20/755	47/10554	1,49E-11	1,43E-08	1,30E-08	TNF/CXCL10/CC	20
R-HSA-1474244	Extracellular matrix organizat	53/755	301/10554	6,10E-10	2,93E-07	2,66E-07	ADAMTS4/ACAN	53
R-HSA-909733	Interferon alpha/beta signalir	22/755	69/10554	1,10E-09	3,52E-07	3,19E-07	IFNB1/MX2/OASI	22
R-HSA-913531	Interferon Signaling	38/755	197/10554	1,39E-08	3,33E-06	3,02E-06	IFNB1/GBP6/GBI	38
R-HSA-216083	Integrin cell surface interactic	22/755	85/10554	7,87E-08	1,51E-05	1,37E-05	ICAM4/ITGA9/IT(	22
R-HSA-449147	Signaling by Interleukins	63/755	462/10554	4,06E-07	6,51E-05	5,90E-05	IFNL1/TNF/IFNL3	63
R-HSA-419037	NCAM1 interactions	14/755	42/10554	6,53E-07	8,97E-05	8,14E-05	CACNA1G/NCAN	14
R-HSA-3000178	ECM proteoglycans	19/755	76/10554	1,08E-06	0,00012932	0,000117287	ACAN/ITGA9/IBS	19
R-HSA-877300	Interferon gamma signaling	21/755	92/10554	1,49E-06	0,000158796	0,00014402	GBP6/GBP5/OAS	21
R-HSA-375165	NCAM signaling for neurite o	16/755	63/10554	6,09E-06	0,000584834	0,000530415	CACNA1G/NCAN	16
R-HSA-8948216	Collagen chain trimerization	13/755	44/10554	7,52E-06	0,000657354	0,000596187	COL26A1/COL20	13
R-HSA-202733	Cell surface interactions at th	25/755	137/10554	1,17E-05	0,000934445	0,000847495	ANGPT2/FCAMR	25
R-HSA-8874081	MET activates PTK2 signalin	10/755	30/10554	2,66E-05	0,001967959	0,00178484	COL2A1/COL11A	10
R-HSA-425407	SLC-mediated transmembra	36/755	246/10554	2,90E-05	0,001991653	0,00180633	FGF21/SLC6A7/S	36
R-HSA-1650814	Collagen biosynthesis and m	15/755	67/10554	5,91E-05	0,003612393	0,00327626	COL26A1/COL20	15
R-HSA-397014	Muscle contraction	31/755	206/10554	6,01E-05	0,003612393	0,00327626	KCNQ1/SCN3A/F	31
R-HSA-380108	Chemokine receptors bind cl	12/755	48/10554	0,00010406	0,005661544	0,005134737	CXCL10/CXCL11	12
R-HSA-418594	G alpha (i) signalling events	49/755	396/10554	0,000106043	0,005661544	0,005134737	CXCL10/CXCL11	49
R-HSA-373076	Class A/1 (Rhodopsin-like re	42/755	324/10554	0,000117464	0,005941229	0,005388398	CXCL10/CXCL11	42
R-HSA-1474290	Collagen formation	17/755	90/10554	0,00018564	0,008919992	0,008089987	COL26A1/COL20	17
R-HSA-445095	Interaction between L1 and A	9/755	31/10554	0,000224822	0,010288295	0,00933097	SCN3A/SCN11A/	9

R-HSA-500792	GPCR ligand binding	53/755	457/10554	0,000296356	0,012945362	0,011740796	CXCL10/CXCL11	53
R-HSA-1250342	PI3K events in ERBB4 signa	5/755	10/10554	0,000343994	0,014372987	0,01303558	HBEGF/NRG3/EI	5
R-HSA-1474228	Degradation of the extracellu	22/755	140/10554	0,000371652	0,014881561	0,013496832	ADAMTS4/ACAN	22
R-HSA-375276	Peptide ligand-binding recep	27/755	190/10554	0,000454398	0,017467065	0,015841754	CXCL10/CXCL11	27
R-HSA-8875878	MET promotes cell motility	10/755	41/10554	0,000483675	0,017876205	0,016212824	COL2A1/COL11A	10
R-HSA-1442490	Collagen degradation	13/755	64/10554	0,000502245	0,017876205	0,016212824	MMP8/COL26A1/	13
R-HSA-2173782	Binding and Uptake of Ligan	10/755	42/10554	0,000594781	0,020066969	0,018199737	COLEC11/STAB1	10
R-HSA-6785807	Interleukin-4 and Interleukin-	18/755	108/10554	0,000605559	0,020066969	0,018199737	TNF/NOS2/ITGAI	18
R-HSA-3906995	Diseases associated with O-	13/755	66/10554	0,00068472	0,021933875	0,019892928	ADAMTS4/MUC1	13
R-HSA-5669034	TNFs bind their physiological	8/755	29/10554	0,000734195	0,022760033	0,020642211	LTA/TNFRSF9/CI	8
R-HSA-3000171	Non-integrin membrane-ECM	12/755	59/10554	0,000808202	0,024271302	0,022012858	COL2A1/COL11A	12
R-HSA-2022090	Assembly of collagen fibrils a	12/755	61/10554	0,001102458	0,032104916	0,029117554	COL2A1/CTSS/C	12
R-HSA-2219530	Constitutive Signaling by Abe	13/755	71/10554	0,001397531	0,039500804	0,035825254	ICOS/HBEGF/PIK	13
R-HSA-8847993	ERBB2 Activates PTK6 Sign	5/755	13/10554	0,001465654	0,040242662	0,036498082	HBEGF/NRG3/EI	5
R-HSA-416700	Other semaphorin interactior	6/755	19/10554	0,001588605	0,04240692	0,038460956	PLXNA4/SEMA4A	6
R-HSA-449836	Other interleukin signaling	7/755	26/10554	0,00184087	0,047812858	0,043363871	IFNL1/CSF1/PTP	7
R-HSA-438066	Unblocking of NMDA recepto	6/755	20/10554	0,00213377	0,052898715	0,047976489	GRIA4/CAMK2B/	6
R-HSA-1250347	SHC1 events in ERBB4 sign	5/755	14/10554	0,002146774	0,052898715	0,047976489	HBEGF/NRG3/EI	5
R-HSA-1251985	Nuclear signaling by ERBB4	7/755	27/10554	0,002334133	0,05607755	0,050859533	STAT5A/HBEGF/	7
R-HSA-5173105	O-linked glycosylation	17/755	112/10554	0,002435904	0,057095222	0,051782511	ADAMTS4/MUC1	17
R-HSA-112316	Neuronal System	41/755	368/10554	0,00296016	0,067731273	0,061428878	GLS2/KCNQ1/SL	41
R-HSA-6785631	ERBB2 Regulates Cell Motili	5/755	15/10554	0,003032489	0,067772605	0,061466364	HBEGF/NRG3/EI	5
R-HSA-390522	Striated Muscle Contraction	8/755	36/10554	0,003315782	0,072419693	0,06568104	ACTN2/NEB/MYL	8
R-HSA-451927	Interleukin-2 family signaling	9/755	44/10554	0,003421113	0,073059769	0,066261557	STAT5A/CSF2RE	9
R-HSA-5602498	MyD88 deficiency (TLR2/4)	4/755	10/10554	0,003848748	0,079846234	0,072416541	BTK/CD36/TLR4/	4
R-HSA-5083635	Defective B3GALTL causes	8/755	37/10554	0,003970789	0,079846234	0,072416541	ADAMTS4/ADAM	8
R-HSA-425393	Transport of inorganic cation	16/755	108/10554	0,004123824	0,079846234	0,072416541	SLC6A12/SLC7A	16
R-HSA-1963640	GRB2 events in ERBB2 sign	5/755	16/10554	0,004154331	0,079846234	0,072416541	HBEGF/NRG3/EI	5
R-HSA-1963642	PI3K events in ERBB2 signa	5/755	16/10554	0,004154331	0,079846234	0,072416541	HBEGF/NRG3/EI	5
R-HSA-3781865	Diseases of glycosylation	19/755	138/10554	0,004320421	0,081410282	0,073835055	ADAMTS4/ACAN	19
R-HSA-166663	Initial triggering of compleme	6/755	23/10554	0,0046217	0,084020799	0,076202664	CFB/COLEC11/C	6
R-HSA-114608	Platelet degranulation	18/755	129/10554	0,004678077	0,084020799	0,076202664	TUBA4A/CTSW/I	18
R-HSA-5173214	O-glycosylation of TSR doma	8/755	38/10554	0,004721252	0,084020799	0,076202664	ADAMTS4/ADAM	8
R-HSA-5083625	Defective GALNT3 causes fa	5/755	17/10554	0,005543662	0,096248693	0,087292751	MUC17/MUC5AC	5

R-HSA-5603041	IRAK4 deficiency (TLR2/4)	4/755	11/10554	0,005708819	0,096248693	0,087292751	BTK/CD36/TLR4/	4
R-HSA-71288	Creatine metabolism	4/755	11/10554	0,005708819	0,096248693	0,087292751	CKM/SLC6A7/SL	4
R-HSA-76005	Response to elevated platele	18/755	134/10554	0,00697144	0,115509548	0,104761384	TUBA4A/CTSW/F	18
R-HSA-1369062	ABC transporters in lipid hon	5/755	18/10554	0,007231164	0,11778218	0,106822548	ABCA9/ABCA6/A	5
R-HSA-352230	Amino acid transport across	7/755	33/10554	0,007721482	0,123672399	0,112164682	SLC6A12/SLC7A	7
R-HSA-383280	Nuclear Receptor transcriptic	9/755	50/10554	0,008241701	0,129840568	0,117758903	NR1H4/NR5A1/N	9
R-HSA-2142753	Arachidonic acid metabolism	10/755	59/10554	0,008454898	0,131050917	0,118856629	CYP4F2/CYP4F3	10
R-HSA-373760	L1CAM interactions	16/755	117/10554	0,008971147	0,136845592	0,12411211	SCN3A/TUBA4A/	16
R-HSA-6806834	Signaling by MET	12/755	79/10554	0,00997826	0,149829814	0,13588815	MUC20/COL2A1/	12
R-HSA-1296071	Potassium Channels	14/755	99/10554	0,01054307	0,154065443	0,139729653	KCNQ1/KCNJ15/	14
R-HSA-2672351	Stimuli-sensing channels	15/755	109/10554	0,010580977	0,154065443	0,139729653	CLCA4/RYR1/TR	15
R-HSA-112315	Transmission across Chemic	26/755	227/10554	0,011441199	0,164104362	0,14883445	GLS2/SLC6A12/C	26
R-HSA-381042	PERK regulates gene expres	6/755	28/10554	0,012711782	0,17964739	0,1629312	ATF3/CXCL8/DD	6
R-HSA-189200	Cellular hexose transport	5/755	21/10554	0,014369808	0,197276933	0,178920314	FGF21/SLC5A10/	5
R-HSA-2142691	Synthesis of Leukotrienes (L	5/755	21/10554	0,014369808	0,197276933	0,178920314	CYP4F2/CYP4F3	5
R-HSA-399955	SEMA3A-Plexin repulsion sig	4/755	14/10554	0,014577448	0,19730884	0,178949252	RND1/PLXNA4/F	4
R-HSA-421270	Cell-cell junction organizatior	10/755	64/10554	0,014795206	0,197474897	0,179099857	CLDN16/NECTIN	10
R-HSA-6788656	Histidine, lysine, phenylalanir	8/755	46/10554	0,015224634	0,200422924	0,181773571	PIPOX/HAL/IL4I1	8
R-HSA-373755	Semaphorin interactions	10/755	65/10554	0,016402748	0,21301407	0,193193111	RND1/PLXNA4/S	10
R-HSA-977606	Regulation of Complement c	8/755	47/10554	0,017234278	0,213169119	0,193333732	CFB/C4B/C7/C1S	8
R-HSA-166058	MyD88:MAL(TIRAP) cascade	13/755	95/10554	0,017376177	0,213169119	0,193333732	NOD2/IRAK3/BTK	13
R-HSA-168188	Toll Like Receptor TLR6:TLF	13/755	95/10554	0,017376177	0,213169119	0,193333732	NOD2/IRAK3/BTK	13
R-HSA-1250196	SHC1 events in ERBB2 signa	5/755	22/10554	0,017527939	0,213169119	0,193333732	HBEGF/NRG3/EF	5
R-HSA-5365859	RA biosynthesis pathway	5/755	22/10554	0,017527939	0,213169119	0,193333732	DHRS9/ALDH8A1	5
R-HSA-4090294	SUMOylation of intracellular	6/755	30/10554	0,017745608	0,213169119	0,193333732	NR1H4/NR5A1/N	6
R-HSA-2219528	PI3K/AKT Signaling in Cance	13/755	97/10554	0,020376037	0,238797219	0,216577137	ICOS/HBEGF/PIK	13
R-HSA-381119	Unfolded Protein Response (	13/755	97/10554	0,020376037	0,238797219	0,216577137	ATF3/ERN1/CXC	13
R-HSA-166658	Complement cascade	9/755	58/10554	0,02109275	0,241311106	0,218857107	CFB/COLEC11/C	9
R-HSA-186797	Signaling by PDGF	9/755	58/10554	0,02109275	0,241311106	0,218857107	STAT5A/COL2A1	9
R-HSA-5619102	SLC transporter disorders	10/755	68/10554	0,022001409	0,243165137	0,22053862	SLC1A3/SLC5A1/	10
R-HSA-168179	Toll Like Receptor TLR1:TLF	13/755	98/10554	0,022013909	0,243165137	0,22053862	NOD2/IRAK3/BTK	13
R-HSA-181438	Toll Like Receptor 2 (TLR2)	13/755	98/10554	0,022013909	0,243165137	0,22053862	NOD2/IRAK3/BTK	13
R-HSA-1169410	Antiviral mechanism by IFN-s	11/755	78/10554	0,022361961	0,244202778	0,221479709	MX2/OASL/OAS2	11
R-HSA-5576891	Cardiac conduction	17/755	141/10554	0,023351967	0,247984547	0,224909585	KCNQ1/SCN3A/F	17
R-HSA-159418	Recycling of bile acids and sa	4/755	16/10554	0,023649376	0,247984547	0,224909585	NR1H4/ALB/ABC	4
R-HSA-399956	CRMPs in Sema3A signaling	4/755	16/10554	0,023649376	0,247984547	0,224909585	PLXNA4/FES/DP	4
R-HSA-6811558	PI5P, PP2A and IER3 Regula	13/755	99/10554	0,023747873	0,247984547	0,224909585	ICOS/HBEGF/PIK	13
R-HSA-983695	Antigen activates B Cell Rec	6/755	32/10554	0,023998505	0,247984547	0,224909585	PTPN6/BTK/PIK3	6

R-HSA-5684996	MAPK1/MAPK3 signaling	27/755	254/10554	0,02471329	0,24910456	0,22592538	CAMK2B/ACTN2/	27
R-HSA-983712	Ion channel transport	21/755	186/10554	0,024829396	0,24910456	0,22592538	ATP4A/FXYD3/C/	21
R-HSA-5260271	Diseases of Immune System	5/755	24/10554	0,025143749	0,24910456	0,22592538	BTK/CD36/TLR4/	5
R-HSA-5602358	Diseases associated with the	5/755	24/10554	0,025143749	0,24910456	0,22592538	BTK/CD36/TLR4/	5
R-HSA-198933	Immunoregulatory interactior	16/755	132/10554	0,026079339	0,255737188	0,231940842	KLRD1/ICAM4/V(	16
R-HSA-5083632	Defective C1GALT1C1 caus(	4/755	17/10554	0,029220365	0,268804544	0,243792281	MUC17/MUC5AC	4
R-HSA-5083636	Defective GALNT12 causes	4/755	17/10554	0,029220365	0,268804544	0,243792281	MUC17/MUC5AC	4
R-HSA-380994	ATF4 activates genes	5/755	25/10554	0,029636132	0,268804544	0,243792281	ATF3/CXCL8/DD	5
R-HSA-8854691	Interleukin-20 family signalin	5/755	25/10554	0,029636132	0,268804544	0,243792281	IFNL1/IFNL3/IFNI	5
R-HSA-112311	Neurotransmitter clearance	3/755	10/10554	0,02992933	0,268804544	0,243792281	SLC6A4/ACHE/S	3
R-HSA-193697	p75NTR regulates axonogen	3/755	10/10554	0,02992933	0,268804544	0,243792281	LINGO1/OMG/NC	3
R-HSA-209968	Thyroxine biosynthesis	3/755	10/10554	0,02992933	0,268804544	0,243792281	DIO2/DUOX2/SL(	3
R-HSA-381183	ATF6 (ATF6-alpha) activates	3/755	10/10554	0,02992933	0,268804544	0,243792281	DDIT3/HSPA5/XE	3
R-HSA-9020958	Interleukin-21 signaling	3/755	10/10554	0,02992933	0,268804544	0,243792281	STAT5A/IL21R/S1	3
R-HSA-1433557	Signaling by SCF-KIT	7/755	43/10554	0,031424317	0,275711989	0,250056988	GRAP/STAT5A/P	7
R-HSA-194068	Bile acid and bile salt metab(	7/755	43/10554	0,031424317	0,275711989	0,250056988	CYP7A1/CH25H/I	7
R-HSA-5673001	RAF/MAP kinase cascade	26/755	248/10554	0,031559125	0,275711989	0,250056988	CAMK2B/ACTN2/	26
R-HSA-1187000	Fertilization	5/755	26/10554	0,034603254	0,298944094	0,271127345	OVGP1/CATSPE	5
R-HSA-442755	Activation of NMDA receptor	7/755	44/10554	0,035151595	0,298944094	0,271127345	GRIA4/CAMK2B/	7
R-HSA-5633008	TP53 Regulates Transcriptio	7/755	44/10554	0,035151595	0,298944094	0,271127345	BCL2L14/TP63/P	7
R-HSA-913709	O-linked glycosylation of mu(	9/755	64/10554	0,037399929	0,315274843	0,285938518	MUC17/MUC5AC	9
R-HSA-199418	Negative regulation of the PI	13/755	106/10554	0,038823428	0,319209359	0,289506927	ICOS/HBEGF/PIK	13
R-HSA-351906	Apoptotic cleavage of cell ad	3/755	11/10554	0,039012022	0,319209359	0,289506927	DSG3/PKP1/CDH	3
R-HSA-1236394	Signaling by ERBB4	7/755	45/10554	0,039160233	0,319209359	0,289506927	STAT5A/HBEGF/	7
R-HSA-425366	Transport of bile salts and or	11/755	85/10554	0,039195322	0,319209359	0,289506927	SLC6A7/SLC6A1	11
R-HSA-912526	Interleukin receptor SHC sig	5/755	27/10554	0,040055302	0,32347181	0,293372757	CSF2RB/PTPN6/	5
R-HSA-1266695	Interleukin-7 signaling	6/755	36/10554	0,040559007	0,324810044	0,294586469	STAT5A/HIST1H	6
R-HSA-442660	Na+/Cl- dependent neurotrar	4/755	19/10554	0,042504763	0,332090055	0,301189075	SLC6A7/SLC6A1	4
R-HSA-5686938	Regulation of TLR by endog(	4/755	19/10554	0,042504763	0,332090055	0,301189075	LBP/CD36/TLR4/	4
R-HSA-9018678	Biosynthesis of specialized p	4/755	19/10554	0,042504763	0,332090055	0,301189075	PTGS2/ALOX15/(	4
R-HSA-5576892	Phase 0 - rapid depolarisatio	7/755	46/10554	0,043456367	0,336604061	0,305283053	SCN3A/CAMK2B	7
R-HSA-8957275	Post-translational protein ph(	13/755	108/10554	0,044156868	0,336604061	0,305283053	SERPINA10/PCS	13
R-HSA-166016	Toll Like Receptor 4 (TLR4) (	15/755	130/10554	0,044473368	0,336604061	0,305283053	NOD2/ITGAM/IR/	15
R-HSA-5578775	Ion homeostasis	8/755	56/10554	0,044483575	0,336604061	0,305283053	FXYD3/CAMK2B/	8
R-HSA-445355	Smooth Muscle Contraction	6/755	37/10554	0,045598346	0,342343835	0,31048874	TRIM72/LMOD1/S	6
R-HSA-1855204	Synthesis of IP3 and IP4 in tl	5/755	28/10554	0,045999546	0,342678786	0,310792524	INPP5D/PLCH2/I	5
R-HSA-210990	PECAM1 interactions	3/755	12/10554	0,049321284	0,356374088	0,323213478	PTPN6/INPP5D/F	3
R-HSA-211958	Miscellaneous substrates	3/755	12/10554	0,049321284	0,356374088	0,323213478	CYP4F2/CYP4F3	3

R-HSA-381033	ATF6 (ATF6-alpha) activates	3/755	12/10554	0,049321284	0,356374088	0,323213478	DDIT3/HSPA5/XE	3
R-HSA-933543	NF-kB activation through FAI	3/755	12/10554	0,049321284	0,356374088	0,323213478	IFIH1/DDX58/CAS	3
R-HSA-442729	CREB phosphorylation throu	4/755	20/10554	0,050226646	0,360207514	0,326690204	CAMK2B/ACTN2/	4
R-HSA-2129379	Molecules associated with el	6/755	38/10554	0,051010114	0,363116444	0,329328458	FBLN2/ITGB8/ITC	6
R-HSA-9006934	Signaling by Receptor Tyrosi	42/755	455/10554	0,051843653	0,365182551	0,331202314	FLRT1/GRAP/ST	42
R-HSA-5601884	PIWI-interacting RNA (piRNA	5/755	29/10554	0,052440366	0,365182551	0,331202314	DDX4/MOV10L1/	5
R-HSA-8863795	Downregulation of ERBB2 si	5/755	29/10554	0,052440366	0,365182551	0,331202314	HBEGF/NRG3/EF	5
R-HSA-512988	Interleukin-3, Interleukin-5 ar	7/755	48/10554	0,052931147	0,365948436	0,331896933	STAT5A/CSF2RE	7
R-HSA-418346	Platelet homeostasis	11/755	90/10554	0,055613074	0,381744029	0,346222746	FGR/NOS2/ATP2	11
R-HSA-174824	Plasma lipoprotein assembly	9/755	69/10554	0,056307754	0,383771291	0,348061371	FGF21/PCSK9/CU	9
R-HSA-446728	Cell junction organization	11/755	91/10554	0,059373365	0,399045627	0,36191443	CLDN16/NECTIN	11
R-HSA-8963899	Plasma lipoprotein remodelir	5/755	30/10554	0,059379318	0,399045627	0,36191443	FGF21/ALB/LIPC	5
R-HSA-388841	Costimulation by the CD28 fa	9/755	70/10554	0,060715647	0,400262561	0,363018128	PTPN6/ICOS/CD2	9
R-HSA-112308	Presynaptic depolarization ar	3/755	13/10554	0,060809921	0,400262561	0,363018128	CACNA2D2/CAC	3
R-HSA-2168880	Scavenging of heme from pla	3/755	13/10554	0,060809921	0,400262561	0,363018128	APOL1/ALB/HPX	3
R-HSA-381426	Regulation of Insulin-like Gro	14/755	125/10554	0,06250661	0,408631645	0,37060847	SERPINA10/PCS	14
R-HSA-1483249	Inositol phosphate metabolis	7/755	50/10554	0,063606692	0,413013725	0,374582796	IP6K3/INPP5D/PL	7
R-HSA-1296065	Inwardly rectifying K+ channe	5/755	31/10554	0,066815213	0,430935704	0,390837136	KCNJ15/ABCC9/I	5
R-HSA-442982	Ras activation upon Ca2+ inf	4/755	22/10554	0,067804796	0,434402726	0,393981551	CAMK2B/ACTN2/	4
R-HSA-112310	Neurotransmitter release cyc	7/755	51/10554	0,069400226	0,439462195	0,398570236	GLS2/SLC6A12/S	7
R-HSA-3560782	Diseases associated with gly	6/755	41/10554	0,069509109	0,439462195	0,398570236	ACAN/CHST6/NC	6
R-HSA-5683057	MAPK family signaling casca	28/755	293/10554	0,070677319	0,443927472	0,40262002	CAMK2B/ACTN2/	28
R-HSA-2024101	CS/DS degradation	3/755	14/10554	0,073418777	0,446553447	0,405001648	NCAN/CSPG4/HY	3
R-HSA-418359	Reduction of cytosolic Ca++	3/755	14/10554	0,073418777	0,446553447	0,405001648	ATP2B2/SLC8A2/	3
R-HSA-6803205	TP53 regulates transcription	3/755	14/10554	0,073418777	0,446553447	0,405001648	BCL2L14/TP63/B	3
R-HSA-8941856	RUNX3 regulates NOTCH si	3/755	14/10554	0,073418777	0,446553447	0,405001648	JAG1/HES1/MAM	3
R-HSA-918233	TRAF3-dependent IRF activa	3/755	14/10554	0,073418777	0,446553447	0,405001648	IFNB1/IFIH1/DDX	3
R-HSA-1500931	Cell-Cell communication	14/755	129/10554	0,076932503	0,464981981	0,421715406	ACTN2/CLDN16/	14
R-HSA-112409	RAF-independent MAPK1/3 a	4/755	23/10554	0,077634646	0,466293095	0,422904521	DUSP8/DUSP16/	4
R-HSA-1980145	Signaling by NOTCH2	5/755	33/10554	0,083160057	0,490287207	0,444665981	DLL4/JAG1/NEUI	5
R-HSA-418990	Adherens junctions interactio	5/755	33/10554	0,083160057	0,490287207	0,444665981	NECTIN4/CDH1/C	5
R-HSA-8964043	Plasma lipoprotein clearance	5/755	33/10554	0,083160057	0,490287207	0,444665981	PCSK9/CUBN/LIF	5
R-HSA-5362517	Signaling by Retinoic Acid	6/755	43/10554	0,083730613	0,490640968	0,444986825	DHRS9/ALDH8A1	6
R-HSA-76002	Platelet activation, signaling a	25/755	262/10554	0,085066568	0,492416545	0,446597184	TUBA4A/CTSW/F	25
R-HSA-193807	Synthesis of bile acids and b	3/755	15/10554	0,087079567	0,492416545	0,446597184	CYP7A1/NR1H4/A	3
R-HSA-2691230	Signaling by NOTCH1 HD Do	3/755	15/10554	0,087079567	0,492416545	0,446597184	DLL4/JAG1/NEUI	3
R-HSA-2691232	Constitutive Signaling by NO	3/755	15/10554	0,087079567	0,492416545	0,446597184	DLL4/JAG1/NEUI	3
R-HSA-168898	Toll-like Receptor Cascades	16/755	155/10554	0,087830389	0,492416545	0,446597184	NOD2/ITGAM/IRA	16

R-HSA-193368	Synthesis of bile acids and b	4/755	24/10554	0,088132826	0,492416545	0,446597184	CYP7A1/NR1H4/,	4
R-HSA-210500	Glutamate Neurotransmitter	4/755	24/10554	0,088132826	0,492416545	0,446597184	GLS2/SLC1A3/SL	4
R-HSA-977068	Termination of O-glycan bios	4/755	24/10554	0,088132826	0,492416545	0,446597184	MUC17/MUC5AC	4
R-HSA-192105	Synthesis of bile acids and b	5/755	34/10554	0,092054005	0,511352014	0,463770708	CYP7A1/CH25H/I	5
R-HSA-1566948	Elastic fibre formation	6/755	45/10554	0,099438022	0,549195055	0,498092453	FBLN2/ITGB8/IT(	6
R-HSA-399954	Sema3A PAK dependent Axo	3/755	16/10554	0,101717304	0,558573308	0,50659806	PLXNA4/FES/PL)	3
R-HSA-425410	Metal ion SLC transporters	4/755	26/10554	0,111033562	0,603464566	0,547312186	SLC30A10/CP/SL	4
R-HSA-452723	Transcriptional regulation of	5/755	36/10554	0,111230708	0,603464566	0,547312186	NR5A1/KLF4/LIN	5
R-HSA-5668541	TNFR2 non-canonical NF-kB	11/755	102/10554	0,111775955	0,603464566	0,547312186	TNF/LTA/LTB/TN	11
R-HSA-432142	Platelet sensitization by LDL	3/755	17/10554	0,117252362	0,625997333	0,567748279	FGR/PTPN6/PEC	3
R-HSA-9018677	Biosynthesis of DHA-derived	3/755	17/10554	0,117252362	0,625997333	0,567748279	PTGS2/ALOX15/(	3
R-HSA-9006931	Signaling by Nuclear Recepto	19/755	199/10554	0,120427656	0,639397664	0,57990171	HIST1H4D/FOSB	19
R-HSA-3000170	Syndecan interactions	4/755	27/10554	0,123377608	0,647900989	0,5876138	COL5A3/COL5A1	4
R-HSA-5621480	Dectin-2 family	4/755	27/10554	0,123377608	0,647900989	0,5876138	MUC17/MUC5AC	4
R-HSA-381070	IRE1alpha activates chapero	7/755	59/10554	0,126572945	0,661068481	0,599556057	ERN1/DNAJB9/H	7
R-HSA-5689896	Ovarian tumor domain protea	5/755	38/10554	0,132163894	0,67932142	0,61611056	NOD2/IFIH1/TNIF	5
R-HSA-1169408	ISG15 antiviral mechanism	8/755	71/10554	0,133243294	0,67932142	0,61611056	MX2/MX1/ISG15/	8
R-HSA-209776	Amine-derived hormones	3/755	18/10554	0,133602236	0,67932142	0,61611056	DIO2/DUOX2/SL(	3
R-HSA-5676594	TNF receptor superfamily (TI	3/755	18/10554	0,133602236	0,67932142	0,61611056	LTA/LTB/TNFSF1	3
R-HSA-8963889	Assembly of active LPL and	3/755	18/10554	0,133602236	0,67932142	0,61611056	FGF21/LIPC/ANG	3
R-HSA-3295583	TRP channels	4/755	28/10554	0,136273951	0,689259301	0,625123721	TRPM1/TRPC4/T	4
R-HSA-425397	Transport of vitamins, nucleo	5/755	39/10554	0,143247115	0,713266721	0,646897251	LCN15/SLCO2B1	5
R-HSA-977444	GABA B receptor activation	5/755	39/10554	0,143247115	0,713266721	0,646897251	KCNJ15/GNAL/G	5
R-HSA-991365	Activation of GABAB recepto	5/755	39/10554	0,143247115	0,713266721	0,646897251	KCNJ15/GNAL/G	5
R-HSA-3000480	Scavenging by Class A Rece	3/755	19/10554	0,150683012	0,732931072	0,664731833	COLEC11/APOE/	3
R-HSA-888590	GABA synthesis, release, reu	3/755	19/10554	0,150683012	0,732931072	0,664731833	SLC6A12/SLC6A	3
R-HSA-8936459	RUNX1 regulates genes invo	10/755	97/10554	0,15465052	0,732931072	0,664731833	GP1BA/HIST1H4	10
R-HSA-438064	Post NMDA receptor activati	5/755	40/10554	0,154716114	0,732931072	0,664731833	CAMK2B/ACTN2/	5
R-HSA-3214842	HDMs demethylate histones	6/755	51/10554	0,154916089	0,732931072	0,664731833	HIST1H4D/HIST1	6
R-HSA-170660	Adenylate cyclase activating	2/755	10/10554	0,157111135	0,732931072	0,664731833	GNAL/ADCY8	2
R-HSA-419771	Opsins	2/755	10/10554	0,157111135	0,732931072	0,664731833	OPN5/OPN3	2
R-HSA-419812	Calcitonin-like ligand recepto	2/755	10/10554	0,157111135	0,732931072	0,664731833	CALCRL/ADM2	2
R-HSA-428643	Organic anion transporters	2/755	10/10554	0,157111135	0,732931072	0,664731833	SLC5A8/SLC5A5	2
R-HSA-450341	Activation of the AP-1 family	2/755	10/10554	0,157111135	0,732931072	0,664731833	MAPK10/FOS	2
R-HSA-549127	Organic cation transport	2/755	10/10554	0,157111135	0,732931072	0,664731833	SLC22A1/SLC22/	2
R-HSA-8877330	RUNX1 and FOXP3 control t	2/755	10/10554	0,157111135	0,732931072	0,664731833	NFATC2/FOXP3	2
R-HSA-8964058	HDL remodeling	2/755	10/10554	0,157111135	0,732931072	0,664731833	ALB/APOE	2
R-HSA-114452	Activation of BH3-only protei	4/755	30/10554	0,163580864	0,745818397	0,676419993	TP63/PMAIP1/BM	4

R-HSA-3000157	Laminin interactions	4/755	30/10554	0,163580864	0,745818397	0,676419993	LAMA2/LAMC3/C	4
R-HSA-418360	Platelet calcium homeostasis	4/755	30/10554	0,163580864	0,745818397	0,676419993	ATP2B2/P2RX7/S	4
R-HSA-5626467	RHO GTPases activate IQGA	4/755	30/10554	0,163580864	0,745818397	0,676419993	TUBA4A/CDH1/T	4
R-HSA-2022870	Chondroitin sulfate biosynthe	3/755	20/10554	0,168410606	0,745818397	0,676419993	CSGALNACT1/N	3
R-HSA-3560783	Defective B4GALT7 causes	3/755	20/10554	0,168410606	0,745818397	0,676419993	NCAN/GPC6/CSP	3
R-HSA-3560801	Defective B3GAT3 causes JI	3/755	20/10554	0,168410606	0,745818397	0,676419993	NCAN/GPC6/CSP	3
R-HSA-4420332	Defective B3GALT6 causes	3/755	20/10554	0,168410606	0,745818397	0,676419993	NCAN/GPC6/CSP	3
R-HSA-622312	Inflammasomes	3/755	20/10554	0,168410606	0,745818397	0,676419993	AIM2/P2RX7/NLF	3
R-HSA-6803204	TP53 Regulates Transcriptio	3/755	20/10554	0,168410606	0,745818397	0,676419993	TP63/PMAIP1/BE	3
R-HSA-9013695	NOTCH4 Intracellular Domai	3/755	20/10554	0,168410606	0,745818397	0,676419993	ACTA2/HES1/MA	3
R-HSA-1660499	Synthesis of PIPs at the plas	6/755	53/10554	0,175918309	0,775493098	0,703333463	PLEKHA4/PLEKH	6
R-HSA-1236977	Endosomal/Vacuolar pathwa	2/755	11/10554	0,183355382	0,793714066	0,71985897	CTSS/HLA-B	2
R-HSA-2022923	Dermatan sulfate biosynthes	2/755	11/10554	0,183355382	0,793714066	0,71985897	NCAN/CSPG4	2
R-HSA-75072	mRNA Editing	2/755	11/10554	0,183355382	0,793714066	0,71985897	APOBEC2/APOB	2
R-HSA-9013700	NOTCH4 Activation and Trar	2/755	11/10554	0,183355382	0,793714066	0,71985897	DLL4/JAG1	2
R-HSA-196741	Cobalamin (Cbl, vitamin B12	3/755	21/10554	0,186701782	0,794450116	0,72052653	CTRB2/CUBN/TC	3
R-HSA-8849932	Synaptic adhesion-like molec	3/755	21/10554	0,186701782	0,794450116	0,72052653	GRIA4/GRIA1/GF	3
R-HSA-8848021	Signaling by PTK6	6/755	54/10554	0,186832181	0,794450116	0,72052653	HBEGF/NRG3/EF	6
R-HSA-9006927	Signaling by Non-Receptor T	6/755	54/10554	0,186832181	0,794450116	0,72052653	HBEGF/NRG3/EF	6
R-HSA-211897	Cytochrome P450 - arranged	7/755	66/10554	0,191064391	0,80249085	0,727819074	CYP7A1/CYP4F2	7
R-HSA-1296072	Voltage gated Potassium cha	5/755	43/10554	0,191228309	0,80249085	0,727819074	KCNQ1/KCNH4/K	5
R-HSA-975634	Retinoid metabolism and trar	5/755	43/10554	0,191228309	0,80249085	0,727819074	AKR1B10/GPC6/	5
R-HSA-442742	CREB phosphorylation throu	4/755	32/10554	0,192655414	0,802773926	0,72807581	CAMK2B/ACTN2/	4
R-HSA-168928	DDX58/IFIH1-mediated indu	8/755	78/10554	0,192966469	0,802773926	0,72807581	IFNB1/IFIH1/DHX	8
R-HSA-168643	Nucleotide-binding domain, l	6/755	55/10554	0,198001749	0,82017104	0,743854122	NOD2/AIM2/P2R)	6
R-HSA-2979096	NOTCH2 Activation and Trar	3/755	22/10554	0,20547499	0,831135294	0,753798152	DLL4/JAG1/NEUI	3
R-HSA-5694530	Cargo concentration in the E	4/755	33/10554	0,207758222	0,831135294	0,753798152	GRIA1/COL7A1/T	4
R-HSA-211979	Eicosanoids	2/755	12/10554	0,210162202	0,831135294	0,753798152	CYP4F2/CYP4F3	2
R-HSA-2160916	Hyaluronan uptake and degr	2/755	12/10554	0,210162202	0,831135294	0,753798152	SLC9A1/HYAL1	2
R-HSA-2197563	NOTCH2 intracellular domai	2/755	12/10554	0,210162202	0,831135294	0,753798152	HES1/MAML2	2
R-HSA-430116	GP1b-IX-V activation signall	2/755	12/10554	0,210162202	0,831135294	0,753798152	GP1BA/VWF	2
R-HSA-6803207	TP53 Regulates Transcriptio	2/755	12/10554	0,210162202	0,831135294	0,753798152	TP63/CASP10	2
R-HSA-6803211	TP53 Regulates Transcriptio	2/755	12/10554	0,210162202	0,831135294	0,753798152	TP63/TNFRSF10	2
R-HSA-71064	Lysine catabolism	2/755	12/10554	0,210162202	0,831135294	0,753798152	PIPOX/CRYM	2
R-HSA-879518	Transport of organic anions	2/755	12/10554	0,210162202	0,831135294	0,753798152	SLCO2B1/ALB	2
R-HSA-8851680	Butyrophilin (BTN) family inte	2/755	12/10554	0,210162202	0,831135294	0,753798152	PPL/BTN2A2	2
R-HSA-6809371	Formation of the cornified en	12/755	129/10554	0,211672487	0,833677296	0,756103622	KRT17/PPL/DSG	12
R-HSA-975138	TRAF6 mediated induction o	9/755	93/10554	0,219821842	0,86223996	0,782008529	NOD2/IRAK2/MA	9

R-HSA-936837	Ion transport by P-type ATPa	6/755	57/10554	0,221046504	0,863519065	0,783168613	ATP4A/FXYD3/C/	6
R-HSA-936440	Negative regulators of DDX5	4/755	34/10554	0,223185451	0,863558604	0,783204473	IFIH1/ISG15/DDX	4
R-HSA-2453902	The canonical retinoid cycle i	3/755	23/10554	0,224651042	0,863558604	0,783204473	DHRS9/STRA6/H	3
R-HSA-389948	PD-1 signaling	3/755	23/10554	0,224651042	0,863558604	0,783204473	PTPN6/CD274/PI	3
R-HSA-450321	JNK (c-Jun kinases) phosph	3/755	23/10554	0,224651042	0,863558604	0,783204473	NOD2/IRAK2/MA	3
R-HSA-112314	Neurotransmitter receptors a	14/755	156/10554	0,225681716	0,864064257	0,783663076	GRIA4/HTR3E/K(	14
R-HSA-168181	Toll Like Receptor 7/8 (TLR7	9/755	94/10554	0,228934031	0,869587366	0,788672259	NOD2/IRAK2/MA	9
R-HSA-975155	MyD88 dependent cascade i	9/755	94/10554	0,228934031	0,869587366	0,788672259	NOD2/IRAK2/MA	9
R-HSA-8854214	TBC/RABGAPs	5/755	46/10554	0,230425266	0,87166735	0,7905587	RAB33A/TBC1D1	5
R-HSA-211945	Phase I - Functionalization of	10/755	107/10554	0,234623295	0,87166735	0,7905587	CYP7A1/CYP4F2	10
R-HSA-190239	FGFR3 ligand binding and ac	2/755	13/10554	0,237317162	0,87166735	0,7905587	GALNT3/FGFR3	2
R-HSA-190372	FGFR3c ligand binding and a	2/755	13/10554	0,237317162	0,87166735	0,7905587	GALNT3/FGFR3	2
R-HSA-2022857	Keratan sulfate degradation	2/755	13/10554	0,237317162	0,87166735	0,7905587	ACAN/PRELP	2
R-HSA-209560	NF-kB is activated and signa	2/755	13/10554	0,237317162	0,87166735	0,7905587	NGFR/NFKBIA	2
R-HSA-432047	Passive transport by Aquapo	2/755	13/10554	0,237317162	0,87166735	0,7905587	MIP/AQP3	2
R-HSA-75109	Triglyceride biosynthesis	2/755	13/10554	0,237317162	0,87166735	0,7905587	MOGAT1/AGMO	2
R-HSA-373080	Class B/2 (Secretin family re	9/755	95/10554	0,238183537	0,87166735	0,7905587	UCN2/GNG2/WN	9
R-HSA-114604	GPVI-mediated activation ca	4/755	35/10554	0,238897742	0,87166735	0,7905587	PTPN6/PIK3R5/V	4
R-HSA-6806667	Metabolism of fat-soluble vita	5/755	47/10554	0,243977674	0,87166735	0,7905587	AKR1B10/GPC6/I	5
R-HSA-3371571	HSF1-dependent transactiva	3/755	24/10554	0,244153644	0,87166735	0,7905587	CAMK2B/CRYAB	3
R-HSA-392154	Nitric oxide stimulates guany	3/755	24/10554	0,244153644	0,87166735	0,7905587	NOS2/PDE1A/PD	3
R-HSA-9006335	Signaling by Erythropoietin	3/755	24/10554	0,244153644	0,87166735	0,7905587	STAT5A/PIK3R5/	3
R-HSA-933542	TRAF6 mediated NF-kB activ	3/755	24/10554	0,244153644	0,87166735	0,7905587	IFIH1/DDX58/NFI	3
R-HSA-982772	Growth hormone receptor sig	3/755	24/10554	0,244153644	0,87166735	0,7905587	STAT5A/PTPN6/I	3
R-HSA-416476	G alpha (q) signalling events	18/755	210/10554	0,244901337	0,87166735	0,7905587	NPFFR1/FFAR2/(	18
R-HSA-168142	Toll Like Receptor 10 (TLR1(	8/755	85/10554	0,261705935	0,886107496	0,803655191	NOD2/IRAK2/MA	8
R-HSA-168176	Toll Like Receptor 5 (TLR5) (	8/755	85/10554	0,261705935	0,886107496	0,803655191	NOD2/IRAK2/MA	8
R-HSA-975871	MyD88 cascade initiated on p	8/755	85/10554	0,261705935	0,886107496	0,803655191	NOD2/IRAK2/MA	8
R-HSA-1296041	Activation of G protein gated	3/755	25/10554	0,263909815	0,886107496	0,803655191	KCNJ15/GNG2/K	3
R-HSA-1296059	G protein gated Potassium c	3/755	25/10554	0,263909815	0,886107496	0,803655191	KCNJ15/GNG2/K	3
R-HSA-210991	Basigin interactions	3/755	25/10554	0,263909815	0,886107496	0,803655191	SLC7A10/SLC7A	3
R-HSA-9013507	NOTCH3 Activation and Tran	3/755	25/10554	0,263909815	0,886107496	0,803655191	DLL4/JAG1/NEUI	3
R-HSA-983170	Antigen Presentation: Folding	3/755	25/10554	0,263909815	0,886107496	0,803655191	HSPA5/TAP1/HL/	3
R-HSA-997272	Inhibition of voltage gated C	3/755	25/10554	0,263909815	0,886107496	0,803655191	KCNJ15/GNG2/K	3
R-HSA-166786	Creation of C4 and C2 activa	2/755	14/10554	0,26463356	0,886107496	0,803655191	COLEC11/C1S	2
R-HSA-170670	Adenylate cyclase inhibitory p	2/755	14/10554	0,26463356	0,886107496	0,803655191	GNAL/ADCY8	2
R-HSA-1855183	Synthesis of IP2, IP, and Ins	2/755	14/10554	0,26463356	0,886107496	0,803655191	INPP5J/MTMR7	2
R-HSA-3296469	Defects in cobalamin (B12) r	2/755	14/10554	0,26463356	0,886107496	0,803655191	CUBN/TCN2	2

R-HSA-418885	DCC mediated attractive sigr	2/755	14/10554	0,26463356	0,886107496	0,803655191	ABLIM2/ABLIM1		2
R-HSA-71240	Tryptophan catabolism	2/755	14/10554	0,26463356	0,886107496	0,803655191	KMO/TDO2		2
R-HSA-8983432	Interleukin-15 signaling	2/755	14/10554	0,26463356	0,886107496	0,803655191	STAT5A/IL15		2
R-HSA-997269	Inhibition of adenylate cyclas	2/755	14/10554	0,26463356	0,886107496	0,803655191	GNAL/ADCY8		2
R-HSA-168138	Toll Like Receptor 9 (TLR9) (	9/755	98/10554	0,266683352	0,889870489	0,807068038	NOD2/IRAK2/MA		9
R-HSA-9012852	Signaling by NOTCH3	5/755	49/10554	0,271668381	0,902133525	0,818189998	DLL4/JAG1/NEUI		5
R-HSA-6798695	Neutrophil degranulation	38/755	479/10554	0,273523703	0,902133525	0,818189998	TNFAIP6/ARHGA		38
R-HSA-9018519	Estrogen-dependent gene ex	13/755	150/10554	0,276004381	0,902133525	0,818189998	HIST1H4D/FOSB		13
R-HSA-983705	Signaling by the B Cell Rece	10/755	112/10554	0,279458565	0,902133525	0,818189998	NFATC2/PTPN6/		10
R-HSA-6794362	Protein-protein interactions a	8/755	87/10554	0,282591421	0,902133525	0,818189998	GRIA4/DLGAP1/I		8
R-HSA-1971475	A tetrasaccharide linker sequ	3/755	26/10554	0,283850196	0,902133525	0,818189998	NCAN/GPC6/CSI		3
R-HSA-2173788	Downregulation of TGF-beta	3/755	26/10554	0,283850196	0,902133525	0,818189998	BAMBI/PPP1R15		3
R-HSA-5223345	Miscellaneous transport and	3/755	26/10554	0,283850196	0,902133525	0,818189998	DMTN/NIPAL4/NI		3
R-HSA-912694	Regulation of IFNA signaling	3/755	26/10554	0,283850196	0,902133525	0,818189998	IFNB1/PTPN6/US		3
R-HSA-1227986	Signaling by ERBB2	5/755	50/10554	0,285758713	0,902133525	0,818189998	HBEGF/NRG3/EI		5
R-HSA-1793185	Chondroitin sulfate/dermatar	5/755	50/10554	0,285758713	0,902133525	0,818189998	CSGALNACT1/N		5
R-HSA-373752	Netrin-1 signaling	5/755	50/10554	0,285758713	0,902133525	0,818189998	UNC5B/TRPC4/A		5
R-HSA-1170546	Prolactin receptor signaling	2/755	15/10554	0,291949559	0,902133525	0,818189998	STAT5A/PRLR		2
R-HSA-211935	Fatty acids	2/755	15/10554	0,291949559	0,902133525	0,818189998	CYP4F2/CYP4F3		2
R-HSA-2214320	Anchoring fibril formation	2/755	15/10554	0,291949559	0,902133525	0,818189998	COL7A1/LAMA3		2
R-HSA-354194	GRB2:SOS provides linkage	2/755	15/10554	0,291949559	0,902133525	0,818189998	VWF/ITGA2B		2
R-HSA-372708	p130Cas linkage to MAPK si	2/755	15/10554	0,291949559	0,902133525	0,818189998	VWF/ITGA2B		2
R-HSA-3772470	Negative regulation of TCF-c	2/755	15/10554	0,291949559	0,902133525	0,818189998	DKK2/WNT4		2
R-HSA-391903	Eicosanoid ligand-binding re	2/755	15/10554	0,291949559	0,902133525	0,818189998	PTGER3/PTGER		2
R-HSA-549132	Organic cation/anion/zwitteri	2/755	15/10554	0,291949559	0,902133525	0,818189998	SLC22A1/SLC22/		2
R-HSA-75892	Platelet Adhesion to exposec	2/755	15/10554	0,291949559	0,902133525	0,818189998	GP1BA/VWF		2
R-HSA-844456	The NLRP3 inflammasome	2/755	15/10554	0,291949559	0,902133525	0,818189998	P2RX7/NLRP3		2
R-HSA-8866910	TFAP2 (AP-2) family regulate	2/755	15/10554	0,291949559	0,902133525	0,818189998	TGFA/ESR1		2
R-HSA-73728	RNA Polymerase I Promoter	6/755	63/10554	0,294662431	0,907598064	0,823146061	HIST1H4D/HIST1		6
R-HSA-6802957	Oncogenic MAPK signaling	7/755	76/10554	0,300290529	0,921314852	0,835586502	FAM131B/RASAL		7
R-HSA-5663202	Diseases of signal transducti	30/755	378/10554	0,301569249	0,921314852	0,835586502	FAM131B/RASAL		30
R-HSA-1368108	BMAL1:CLOCK,NPAS2 activ	3/755	27/10554	0,30390927	0,921314852	0,835586502	BHLHE41/NOCT/		3
R-HSA-5576893	Phase 2 - plateau phase	3/755	27/10554	0,30390927	0,921314852	0,835586502	KCNQ1/CACNA2		3
R-HSA-8963743	Digestion and absorption	3/755	27/10554	0,30390927	0,921314852	0,835586502	SLC5A1/PNLIPRI		3
R-HSA-450294	MAP kinase activation	6/755	64/10554	0,307411669	0,929001929	0,842558297	NOD2/IRAK2/MA		6
R-HSA-171306	Packaging Of Telomere Ends	5/755	52/10554	0,314311146	0,94226761	0,854589606	HIST1H4D/HIST1		5
R-HSA-2187338	Visual phototransduction	9/755	103/10554	0,316233149	0,94226761	0,854589606	AKR1B10/DHRS9		9
R-HSA-1433559	Regulation of KIT signaling	2/755	16/10554	0,319125595	0,94226761	0,854589606	PTPN6/KITLG		2

R-HSA-190840	Microtubule-dependent traffic	2/755	16/10554	0,319125595	0,94226761	0,854589606	TUBA4A/TUBAL3	2
R-HSA-193639	p75NTR signals via NF-kB	2/755	16/10554	0,319125595	0,94226761	0,854589606	NGFR/NFKBIA	2
R-HSA-210744	Regulation of gene expressio	2/755	16/10554	0,319125595	0,94226761	0,854589606	HES1/MAML2	2
R-HSA-391160	Signal regulatory protein fam	2/755	16/10554	0,319125595	0,94226761	0,854589606	PTPN6/FYB1	2
R-HSA-5334118	DNA methylation	6/755	65/10554	0,320253893	0,94226761	0,854589606	HIST1H4D/HIST1	6
R-HSA-1483191	Synthesis of PC	3/755	28/10554	0,324025508	0,94226761	0,854589606	ACHE/SLC44A5/S	3
R-HSA-201451	Signaling by BMP	3/755	28/10554	0,324025508	0,94226761	0,854589606	AMHR2/BMP2/SM	3
R-HSA-2022854	Keratan sulfate biosynthesis	3/755	28/10554	0,324025508	0,94226761	0,854589606	ACAN/CHST6/PR	3
R-HSA-400042	Adrenaline,noradrenaline inh	3/755	28/10554	0,324025508	0,94226761	0,854589606	CACNA2D2/GNG	3
R-HSA-8939211	ESR-mediated signaling	13/755	156/10554	0,324547949	0,94226761	0,854589606	HIST1H4D/FOSB	13
R-HSA-1474165	Reproduction	12/755	144/10554	0,334179003	0,964402469	0,874664816	HIST1H4D/OVGF	12
R-HSA-5619115	Disorders of transmembrane	12/755	144/10554	0,334179003	0,964402469	0,874664816	ABCC9/SLC1A3/S	12
R-HSA-163125	Post-translational modificatio	8/755	92/10554	0,336452115	0,967766226	0,877715576	RAET1L/ART4/O	8
R-HSA-420029	Tight junction interactions	3/755	29/10554	0,344141452	0,967766226	0,877715576	CLDN16/CLDN6/	3
R-HSA-933541	TRAF6 mediated IRF7 activa	3/755	29/10554	0,344141452	0,967766226	0,877715576	IFNB1/IFIH1/DDX	3
R-HSA-190872	Transport of connexons to th	2/755	17/10554	0,346042027	0,967766226	0,877715576	TUBA4A/TUBAL3	2
R-HSA-2142845	Hyaluronan metabolism	2/755	17/10554	0,346042027	0,967766226	0,877715576	SLC9A1/HYAL1	2
R-HSA-416993	Trafficking of GluR2-containi	2/755	17/10554	0,346042027	0,967766226	0,877715576	GRIA4/GRIA1	2
R-HSA-9014325	TICAM1,TRAF6-dependent i	2/755	17/10554	0,346042027	0,967766226	0,877715576	IRAK2/TLR4	2
R-HSA-975163	IRAK2 mediated activation o	2/755	17/10554	0,346042027	0,967766226	0,877715576	IRAK2/TLR4	2
R-HSA-5625886	Activated PKN1 stimulates tr	6/755	67/10554	0,346152494	0,967766226	0,877715576	HIST1H4D/HIST1	6
R-HSA-977225	Amyloid fiber formation	9/755	106/10554	0,346850505	0,967766226	0,877715576	HIST1H4D/HIST1	9
R-HSA-422356	Regulation of insulin secretio	7/755	80/10554	0,3473246	0,967766226	0,877715576	CACNA2D2/GNG	7
R-HSA-1912408	Pre-NOTCH Transcription ar	8/755	93/10554	0,347429082	0,967766226	0,877715576	HIST1H4D/HIST1	8
R-HSA-186712	Regulation of beta-cell devel	4/755	42/10554	0,353691538	0,982362912	0,890954041	NR5A2/HNF4A/H	4
R-HSA-427359	SIRT1 negatively regulates rI	6/755	68/10554	0,3591771	0,994723901	0,902164839	HIST1H4D/HIST1	6
R-HSA-1482839	Acyl chain remodelling of PE	3/755	30/10554	0,364203739	0,997397541	0,904589697	HRASLS2/RARRI	3
R-HSA-2424491	DAP12 signaling	3/755	30/10554	0,364203739	0,997397541	0,904589697	KLRD1/KLRC2/B	3
R-HSA-5683826	Surfactant metabolism	3/755	30/10554	0,364203739	0,997397541	0,904589697	CSF2RB/GATA6/	3
R-HSA-211000	Gene Silencing by RNA	11/755	135/10554	0,372176397	0,997397541	0,904589697	DDX4/HIST1H4D	11
R-HSA-6794361	Neurexins and neuroligins	5/755	56/10554	0,372267244	0,997397541	0,904589697	DLGAP1/NRXN2/	5
R-HSA-977443	GABA receptor activation	5/755	56/10554	0,372267244	0,997397541	0,904589697	KCNJ15/GNAL/G	5
R-HSA-181430	Norepinephrine Neurotransm	2/755	18/10554	0,372597	0,997397541	0,904589697	SLC22A1/SYT1	2
R-HSA-210993	Tie2 Signaling	2/755	18/10554	0,372597	0,997397541	0,904589697	ANGPT2/ANGPT	2
R-HSA-418457	cGMP effects	2/755	18/10554	0,372597	0,997397541	0,904589697	PDE1A/PDE3A	2
R-HSA-881907	Gastrin-CREB signalling patl	2/755	18/10554	0,372597	0,997397541	0,904589697	GAST/HBEGF	2
R-HSA-8963898	Plasma lipoprotein assembly	2/755	18/10554	0,372597	0,997397541	0,904589697	MTTP/APOE	2
R-HSA-937072	TRAF6-mediated induction o	2/755	18/10554	0,372597	0,997397541	0,904589697	IRAK2/TLR4	2

R-HSA-196854	Metabolism of vitamins and c	15/755	189/10554	0,376376688	1	0,906949997	CTRB2/SLC5A8/A	15
R-HSA-1912422	Pre-NOTCH Expression and	9/755	109/10554	0,377874773	1	0,906949997	HIST1H4D/HIST1	9
R-HSA-111885	Opioid Signalling	7/755	83/10554	0,383126226	1	0,906949997	PPP1R1B/GNAL/	7
R-HSA-196807	Nicotinate metabolism	3/755	31/10554	0,384163085	1	0,906949997	SLC5A8/PTGS2/I	3
R-HSA-2122948	Activated NOTCH1 Transmit	3/755	31/10554	0,384163085	1	0,906949997	DLL4/JAG1/NEUI	3
R-HSA-399719	Trafficking of AMPA receptor	3/755	31/10554	0,384163085	1	0,906949997	GRIA4/CAMK2B/	3
R-HSA-399721	Glutamate binding, activation	3/755	31/10554	0,384163085	1	0,906949997	GRIA4/CAMK2B/	3
R-HSA-450282	MAPK targets/ Nuclear event	3/755	31/10554	0,384163085	1	0,906949997	MAPK10/MEF2C/	3
R-HSA-400253	Circadian Clock	6/755	70/10554	0,385299856	1	0,906949997	BHLHE41/NR1D1	6
R-HSA-381038	XBP1(S) activates chaperone	5/755	57/10554	0,3868077	1	0,906949997	DNAJB9/HYOU1/	5
R-HSA-109606	Intrinsic Pathway for Apoptos	4/755	44/10554	0,386987208	1	0,906949997	TP63/PMAIP1/BM	4
R-HSA-2559582	Senescence-Associated Sec	9/755	110/10554	0,388270556	1	0,906949997	HIST1H4D/HIST1	9
R-HSA-1483255	PI Metabolism	7/755	84/10554	0,395097433	1	0,906949997	PLEKHA4/PLEKH	7
R-HSA-197264	Nicotinamide salvaging	2/755	19/10554	0,398704528	1	0,906949997	SLC5A8/PTGS2	2
R-HSA-3928664	Ephrin signaling	2/755	19/10554	0,398704528	1	0,906949997	EFNB2/EPHB2	2
R-HSA-422085	Synthesis, secretion, and dea	2/755	19/10554	0,398704528	1	0,906949997	ACHE/KLF4	2
R-HSA-8964038	LDL clearance	2/755	19/10554	0,398704528	1	0,906949997	PCSK9/LSR	2
R-HSA-2644602	Signaling by NOTCH1 PEST	5/755	58/10554	0,40132824	1	0,906949997	DLL4/JAG1/NEUI	5
R-HSA-2644603	Signaling by NOTCH1 in Car	5/755	58/10554	0,40132824	1	0,906949997	DLL4/JAG1/NEUI	5
R-HSA-2644606	Constitutive Signaling by NO	5/755	58/10554	0,40132824	1	0,906949997	DLL4/JAG1/NEUI	5
R-HSA-2894858	Signaling by NOTCH1 HD+P	5/755	58/10554	0,40132824	1	0,906949997	DLL4/JAG1/NEUI	5
R-HSA-2894862	Constitutive Signaling by NO	5/755	58/10554	0,40132824	1	0,906949997	DLL4/JAG1/NEUI	5
R-HSA-2173789	TGF-beta receptor signaling	3/755	32/10554	0,403974223	1	0,906949997	BAMBI/PPP1R15	3
R-HSA-2871796	FCERI mediated MAPK activ	3/755	32/10554	0,403974223	1	0,906949997	MAPK10/VAV1/F	3
R-HSA-2559580	Oxidative Stress Induced Se	10/755	125/10554	0,404508204	1	0,906949997	IFNB1/HIST1H4D	10
R-HSA-448424	Interleukin-17 signaling	6/755	72/10554	0,411422271	1	0,906949997	NOD2/IRAK2/MA	6
R-HSA-1236975	Antigen processing-Cross pr	8/755	99/10554	0,413926394	1	0,906949997	CTSS/BTK/NCF2	8
R-HSA-168164	Toll Like Receptor 3 (TLR3)	8/755	99/10554	0,413926394	1	0,906949997	NOD2/IRAK2/MA	8
R-HSA-912446	Meiotic recombination	7/755	86/10554	0,41902841	1	0,906949997	HIST1H4D/HIST1	7
R-HSA-2871809	FCERI mediated Ca+2 mobil	3/755	33/10554	0,423595823	1	0,906949997	NFATC2/BTK/VA	3
R-HSA-389356	CD28 co-stimulation	3/755	33/10554	0,423595823	1	0,906949997	VAV1/MAP3K8/P	3
R-HSA-420092	Glucagon-type ligand recepto	3/755	33/10554	0,423595823	1	0,906949997	GNG2/GLP2R/GI	3
R-HSA-445989	TAK1 activates NFkB by pho	3/755	33/10554	0,423595823	1	0,906949997	NOD2/IRAK2/NFI	3
R-HSA-389977	Post-chaperonin tubulin foldi	2/755	20/10554	0,424292742	1	0,906949997	TUBA4A/TUBAL3	2
R-HSA-212300	PRC2 methylates histones a	6/755	73/10554	0,424448047	1	0,906949997	HIST1H4D/HIST1	6
R-HSA-166166	MyD88-independent TLR4 ca	8/755	100/10554	0,425030053	1	0,906949997	NOD2/IRAK2/MA	8
R-HSA-937061	TRIF(TICAM1)-mediated TLI	8/755	100/10554	0,425030053	1	0,906949997	NOD2/IRAK2/MA	8
R-HSA-73887	Death Receptor Signalling	11/755	141/10554	0,428170193	1	0,906949997	TNF/LINGO1/OM	11

R-HSA-6802952	Signaling by BRAF and RAF	5/755	60/10554	0,430234693	1	0,906949997	FAM131B/VWF/E	5
R-HSA-190236	Signaling by FGFR	7/755	87/10554	0,430966646	1	0,906949997	FLRT1/GALNT3/I	7
R-HSA-112040	G-protein mediated events	4/755	47/10554	0,4364296	1	0,906949997	GNAL/ADCY8/PD	4
R-HSA-2299718	Condensation of Prophase C	6/755	74/10554	0,437432299	1	0,906949997	HIST1H4D/HIST1	6
R-HSA-1638074	Keratan sulfate/keratin metal	3/755	34/10554	0,442990375	1	0,906949997	ACAN/CHST6/PR	3
R-HSA-190861	Gap junction assembly	3/755	34/10554	0,442990375	1	0,906949997	TUBA4A/TUBAL3	3
R-HSA-70263	Gluconeogenesis	3/755	34/10554	0,442990375	1	0,906949997	FBP2/ENO3/PCK	3
R-HSA-70614	Amino acid synthesis and int	3/755	34/10554	0,442990375	1	0,906949997	GLS2/PSAT1/SEI	3
R-HSA-445144	Signal transduction by L1	2/755	21/10554	0,449302317	1	0,906949997	ITGA9/ITGA2B	2
R-HSA-4641263	Regulation of FZD by ubiquit	2/755	21/10554	0,449302317	1	0,906949997	LGR6/RSPO2	2
R-HSA-6804115	TP53 regulates transcription	2/755	21/10554	0,449302317	1	0,906949997	PLK3/BTG2	2
R-HSA-427389	ERCC6 (CSB) and EHMT2 (	6/755	76/10554	0,463225504	1	0,906949997	HIST1H4D/HIST1	6
R-HSA-140837	Intrinsic Pathway of Fibrin Cl	2/755	22/10554	0,47368505	1	0,906949997	GP1BA/VWF	2
R-HSA-3296482	Defects in vitamin and cofact	2/755	22/10554	0,47368505	1	0,906949997	CUBN/TCN2	2
R-HSA-389357	CD28 dependent PI3K/Akt si	2/755	22/10554	0,47368505	1	0,906949997	MAP3K8/PIK3R3	2
R-HSA-8935690	Digestion	2/755	22/10554	0,47368505	1	0,906949997	PNLIPRP3/MGAM	2
R-HSA-912631	Regulation of signaling by CE	2/755	22/10554	0,47368505	1	0,906949997	VAV1/PIK3R3	2
R-HSA-168638	NOD1/2 Signaling Pathway	3/755	36/10554	0,4809666	1	0,906949997	NOD2/IRAK2/CAI	3
R-HSA-8955332	Carboxyterminal post-transla	3/755	36/10554	0,4809666	1	0,906949997	TUBA4A/TUBAL3	3
R-HSA-389960	Formation of tubulin folding i	2/755	23/10554	0,497402574	1	0,906949997	TUBA4A/TUBAL3	2
R-HSA-428930	Thromboxane signalling thro	2/755	23/10554	0,497402574	1	0,906949997	GNG2/GNA15	2
R-HSA-3214815	HDACs deacetylate histones	7/755	94/10554	0,513102667	1	0,906949997	HIST1H2AA/HIST	7
R-HSA-2559586	DNA Damage/Telomere Stre	6/755	80/10554	0,513785174	1	0,906949997	HIST1H1A/HIST1	6
R-HSA-445717	Aquaporin-mediated transpo	4/755	52/10554	0,515903685	1	0,906949997	MIP/GNG2/AQP3	4
R-HSA-75153	Apoptotic execution phase	4/755	52/10554	0,515903685	1	0,906949997	HIST1H1A/DSG3	4
R-HSA-111465	Apoptotic cleavage of cellula	3/755	38/10554	0,517673898	1	0,906949997	DSG3/PKP1/CDH	3
R-HSA-8864260	Transcriptional regulation by	3/755	38/10554	0,517673898	1	0,906949997	TGFA/ESR1/APC	3
R-HSA-211981	Xenobiotics	2/755	24/10554	0,520425204	1	0,906949997	CYP2S1/CYP1A1	2
R-HSA-450302	activated TAK1 mediates p38	2/755	24/10554	0,520425204	1	0,906949997	NOD2/IRAK2	2
R-HSA-6783589	Interleukin-6 family signaling	2/755	24/10554	0,520425204	1	0,906949997	IL11/IL6	2
R-HSA-196849	Metabolism of water-soluble	9/755	123/10554	0,522183245	1	0,906949997	CTRB2/SLC5A8/I	9
R-HSA-110056	MAPK3 (ERK1) activation	1/755	10/10554	0,524112919	1	0,906949997	IL6	1
R-HSA-1300644	Interaction With The Zona Pe	1/755	10/10554	0,524112919	1	0,906949997	OVGP1	1
R-HSA-1483115	Hydrolysis of LPC	1/755	10/10554	0,524112919	1	0,906949997	PLA2G4C	1
R-HSA-168799	Neurotoxicity of clostridium to	1/755	10/10554	0,524112919	1	0,906949997	SYT1	1
R-HSA-1855167	Synthesis of pyrophosphates	1/755	10/10554	0,524112919	1	0,906949997	IP6K3	1
R-HSA-2161522	Abacavir transport and metal	1/755	10/10554	0,524112919	1	0,906949997	SLC22A1	1
R-HSA-2562578	TRIF-mediated programmed	1/755	10/10554	0,524112919	1	0,906949997	TLR4	1

R-HSA-399997	Acetylcholine regulates insuli	1/755	10/10554	0,524112919	1	0,906949997	GNA15	1
R-HSA-418889	Caspase activation via Depe	1/755	10/10554	0,524112919	1	0,906949997	UNC5B	1
R-HSA-418890	Role of second messengers	1/755	10/10554	0,524112919	1	0,906949997	TRPC4	1
R-HSA-428542	Regulation of commissural a	1/755	10/10554	0,524112919	1	0,906949997	SLIT2	1
R-HSA-74182	Ketone body metabolism	1/755	10/10554	0,524112919	1	0,906949997	BDH1	1
R-HSA-8853334	Signaling by FGFR3 fusions	1/755	10/10554	0,524112919	1	0,906949997	FGFR3	1
R-HSA-937042	IRAK2 mediated activation o	1/755	10/10554	0,524112919	1	0,906949997	IRAK2	1
R-HSA-6802949	Signaling by RAS mutants	4/755	53/10554	0,531183409	1	0,906949997	RASAL1/VWF/SF	4
R-HSA-1630316	Glycosaminoglycan metaboli	9/755	124/10554	0,532162192	1	0,906949997	ACAN/CHST6/CS	9
R-HSA-140877	Formation of Fibrin Clot (Clot	3/755	39/10554	0,535494358	1	0,906949997	GP1BA/CD177/V	3
R-HSA-76009	Platelet Aggregation (Plug Fo	3/755	39/10554	0,535494358	1	0,906949997	GP1BA/VWF/ITG	3
R-HSA-392451	G beta:gamma signalling thro	2/755	25/10554	0,54273089	1	0,906949997	GNG2/PIK3R5	2
R-HSA-418592	ADP signalling through P2Y p	2/755	25/10554	0,54273089	1	0,906949997	GNG2/GNA15	2
R-HSA-9013508	NOTCH3 Intracellular Domai	2/755	25/10554	0,54273089	1	0,906949997	HES1/MAML2	2
R-HSA-193704	p75 NTR receptor-mediated	7/755	97/10554	0,547056047	1	0,906949997	LINGO1/OMG/NC	7
R-HSA-1236974	ER-Phagosome pathway	6/755	83/10554	0,550483391	1	0,906949997	BTK/CD36/TLR4/	6
R-HSA-5654741	Signaling by FGFR3	3/755	40/10554	0,552934733	1	0,906949997	GALNT3/FGFR3/	3
R-HSA-5675221	Negative regulation of MAPK	3/755	40/10554	0,552934733	1	0,906949997	DUSP8/DUSP16/	3
R-HSA-5693571	Nonhomologous End-Joining	5/755	69/10554	0,554930474	1	0,906949997	HIST1H4D/HIST1	5
R-HSA-1483257	Phospholipid metabolism	15/755	212/10554	0,556810711	1	0,906949997	PLEKHA4/PLEKH	15
R-HSA-1059683	Interleukin-6 signaling	1/755	11/10554	0,558188672	1	0,906949997	IL6	1
R-HSA-1234158	Regulation of gene expressio	1/755	11/10554	0,558188672	1	0,906949997	EPAS1	1
R-HSA-1433617	Regulation of signaling by NO	1/755	11/10554	0,558188672	1	0,906949997	NODAL	1
R-HSA-1660517	Synthesis of PIPs at the late	1/755	11/10554	0,558188672	1	0,906949997	MTMR7	1
R-HSA-189085	Digestion of dietary carbohyd	1/755	11/10554	0,558188672	1	0,906949997	MGAM	1
R-HSA-193692	Regulated proteolysis of p75	1/755	11/10554	0,558188672	1	0,906949997	NGFR	1
R-HSA-196791	Vitamin D (calciferol) metabo	1/755	11/10554	0,558188672	1	0,906949997	CUBN	1
R-HSA-2025928	Calcineurin activates NFAT	1/755	11/10554	0,558188672	1	0,906949997	NFATC2	1
R-HSA-211999	CYP2E1 reactions	1/755	11/10554	0,558188672	1	0,906949997	CYP2S1	1
R-HSA-2206281	Mucopolysaccharidoses	1/755	11/10554	0,558188672	1	0,906949997	HYAL1	1
R-HSA-2586552	Signaling by Leptin	1/755	11/10554	0,558188672	1	0,906949997	STAT5A	1
R-HSA-446107	Type I hemidesmosome asse	1/755	11/10554	0,558188672	1	0,906949997	LAMA3	1
R-HSA-4755510	SUMOylation of immune resp	1/755	11/10554	0,558188672	1	0,906949997	NFKBIA	1
R-HSA-71182	Phenylalanine and tyrosine c	1/755	11/10554	0,558188672	1	0,906949997	IL4I1	1
R-HSA-8851805	MET activates RAS signaling	1/755	11/10554	0,558188672	1	0,906949997	MUC20	1
R-HSA-8934903	Receptor Mediated Mitophag	1/755	11/10554	0,558188672	1	0,906949997	MAP1LC3B	1
R-HSA-9027276	Erythropoietin activates Phos	1/755	11/10554	0,558188672	1	0,906949997	PIK3R5	1
R-HSA-381340	Transcriptional regulation of	6/755	84/10554	0,562436157	1	0,906949997	TNF/EGR2/CD36	6

R-HSA-180024	DARPP-32 events	2/755	26/10554	0,564304281	1	0,906949997	PPP1R1B/PDE4C	2
R-HSA-6803529	FGFR2 alternative splicing	2/755	26/10554	0,564304281	1	0,906949997	ESRP1/POLR2A	2
R-HSA-8853659	RET signaling	3/755	41/10554	0,56997997	1	0,906949997	PIK3R3/GDNF/GI	3
R-HSA-211976	Endogenous sterols	2/755	27/10554	0,585135882	1	0,906949997	CYP7A1/NR1H4	2
R-HSA-264876	Insulin processing	2/755	27/10554	0,585135882	1	0,906949997	CLTRN/MYRIP	2
R-HSA-354192	Integrin alphaIIb beta3 signal	2/755	27/10554	0,585135882	1	0,906949997	VWF/ITGA2B	2
R-HSA-5357769	Caspase activation via extrin	2/755	27/10554	0,585135882	1	0,906949997	UNC5B/TLR4	2
R-HSA-9006921	Integrin signaling	2/755	27/10554	0,585135882	1	0,906949997	VWF/ITGA2B	2
R-HSA-375280	Amine ligand-binding recepto	3/755	42/10554	0,586617542	1	0,906949997	TAAR1/HRH1/AD	3
R-HSA-6814122	Cooperation of PDCL (PhLP1	3/755	42/10554	0,586617542	1	0,906949997	GNG2/RGS6/GNA	3
R-HSA-1296346	Tandem pore domain potass	1/755	12/10554	0,589827442	1	0,906949997	KCNK6	1
R-HSA-1474151	Tetrahydrobiopterin (BH4) sy	1/755	12/10554	0,589827442	1	0,906949997	GCH1	1
R-HSA-1475029	Reversible hydration of carbo	1/755	12/10554	0,589827442	1	0,906949997	CA3	1
R-HSA-174403	Glutathione synthesis and re	1/755	12/10554	0,589827442	1	0,906949997	GGT1	1
R-HSA-1839130	Signaling by activated point r	1/755	12/10554	0,589827442	1	0,906949997	FGFR3	1
R-HSA-196108	Pregnenolone biosynthesis	1/755	12/10554	0,589827442	1	0,906949997	STAR	1
R-HSA-2029481	FCGR activation	1/755	12/10554	0,589827442	1	0,906949997	FGR	1
R-HSA-2033514	FGFR3 mutant receptor activ	1/755	12/10554	0,589827442	1	0,906949997	FGFR3	1
R-HSA-209822	Glycoprotein hormones	1/755	12/10554	0,589827442	1	0,906949997	INHBE	1
R-HSA-389359	CD28 dependent Vav1 pathw	1/755	12/10554	0,589827442	1	0,906949997	VAV1	1
R-HSA-417957	P2Y receptors	1/755	12/10554	0,589827442	1	0,906949997	LPAR6	1
R-HSA-451306	Ionotropic activity of kainate r	1/755	12/10554	0,589827442	1	0,906949997	GRIK5	1
R-HSA-451308	Activation of Ca-permeable K	1/755	12/10554	0,589827442	1	0,906949997	GRIK5	1
R-HSA-5687613	Diseases associated with sur	1/755	12/10554	0,589827442	1	0,906949997	CSF2RB	1
R-HSA-622323	Presynaptic nicotinic acetylch	1/755	12/10554	0,589827442	1	0,906949997	CHRNA6	1
R-HSA-629594	Highly calcium permeable po	1/755	12/10554	0,589827442	1	0,906949997	CHRNA6	1
R-HSA-8866427	VLDLR internalisation and de	1/755	12/10554	0,589827442	1	0,906949997	PCSK9	1
R-HSA-8984722	Interleukin-35 Signalling	1/755	12/10554	0,589827442	1	0,906949997	STAT4	1
R-HSA-9020558	Interleukin-2 signaling	1/755	12/10554	0,589827442	1	0,906949997	STAT5A	1
R-HSA-2172127	DAP12 interactions	3/755	43/10554	0,602837275	1	0,906949997	KLRD1/KLRC2/BT	3
R-HSA-432040	Vasopressin regulates renal	3/755	43/10554	0,602837275	1	0,906949997	GNG2/AQP3/ADC	3
R-HSA-447115	Interleukin-12 family signaling	4/755	58/10554	0,603724965	1	0,906949997	IL12B/IL12RB1/IL	4
R-HSA-1989781	PPARA activates gene expre	8/755	117/10554	0,605122546	1	0,906949997	CYP7A1/NR1H4/(	8
R-HSA-111933	Calmodulin induced events	2/755	28/10554	0,605221296	1	0,906949997	ADCY8/PDE1A	2
R-HSA-111997	CaM pathway	2/755	28/10554	0,605221296	1	0,906949997	ADCY8/PDE1A	2
R-HSA-1482788	Acyl chain remodelling of PC	2/755	28/10554	0,605221296	1	0,906949997	PLB1/PLA2G4C	2
R-HSA-202040	G-protein activation	2/755	28/10554	0,605221296	1	0,906949997	GNG2/GNAT2	2
R-HSA-397795	G-protein beta:gamma signa	2/755	28/10554	0,605221296	1	0,906949997	GNG2/PIK3R5	2

R-HSA-1980143	Signaling by NOTCH1	5/755	73/10554	0,605898641	1	0,906949997	DLL4/JAG1/NEUI	5
R-HSA-382556	ABC-family proteins mediate	7/755	103/10554	0,611688941	1	0,906949997	ABCA9/ABCC9/A	7
R-HSA-193648	NRAGE signals death throug	4/755	59/10554	0,617391512	1	0,906949997	NGFR/ARHGEF3	4
R-HSA-606279	Deposition of new CENPA-co	5/755	74/10554	0,618106347	1	0,906949997	HIST1H4D/HIST1	5
R-HSA-774815	Nucleosome assembly	5/755	74/10554	0,618106347	1	0,906949997	HIST1H4D/HIST1	5
R-HSA-140342	Apoptosis induced DNA fragı	1/755	13/10554	0,619203299	1	0,906949997	HIST1H1A	1
R-HSA-1679131	Trafficking and processing of	1/755	13/10554	0,619203299	1	0,906949997	CTSS	1
R-HSA-203615	eNOS activation	1/755	13/10554	0,619203299	1	0,906949997	CYGB	1
R-HSA-209543	p75NTR recruits signalling co	1/755	13/10554	0,619203299	1	0,906949997	NGFR	1
R-HSA-211227	Activation of DNA fragmenta	1/755	13/10554	0,619203299	1	0,906949997	HIST1H1A	1
R-HSA-350054	Notch-HLH transcription path	1/755	13/10554	0,619203299	1	0,906949997	MAML2	1
R-HSA-425561	Sodium/Calcium exchangers	1/755	13/10554	0,619203299	1	0,906949997	SLC8A2	1
R-HSA-5357786	TNFR1-induced proapoptotic	1/755	13/10554	0,619203299	1	0,906949997	TNF	1
R-HSA-5607763	CLEC7A (Dectin-1) induces I	1/755	13/10554	0,619203299	1	0,906949997	NFATC2	1
R-HSA-5654227	Phospholipase C-mediated c	1/755	13/10554	0,619203299	1	0,906949997	FGFR3	1
R-HSA-5668599	RHO GTPases Activate NAD	1/755	13/10554	0,619203299	1	0,906949997	NCF2	1
R-HSA-75205	Dissolution of Fibrin Clot	1/755	13/10554	0,619203299	1	0,906949997	SERPINF2	1
R-HSA-9027284	Erythropoietin activates RAS	1/755	13/10554	0,619203299	1	0,906949997	VAV1	1
R-HSA-400206	Regulation of lipid metabolis	8/755	119/10554	0,624452937	1	0,906949997	CYP7A1/NR1H4/	8
R-HSA-186763	Downstream signal transduc	2/755	29/10554	0,624560543	1	0,906949997	STAT5A/PDGFB	2
R-HSA-5218920	VEGFR2 mediated vascular	2/755	29/10554	0,624560543	1	0,906949997	CDH5/VAV1	2
R-HSA-5654732	Negative regulation of FGFR	2/755	29/10554	0,624560543	1	0,906949997	FGFR3/SPRY2	2
R-HSA-73777	RNA Polymerase I Chain Elo	6/755	90/10554	0,63072671	1	0,906949997	HIST1H4D/HIST1	6
R-HSA-5578749	Transcriptional regulation by	7/755	105/10554	0,632104393	1	0,906949997	HIST1H4D/HIST1	7
R-HSA-190828	Gap junction trafficking	3/755	45/10554	0,6339933	1	0,906949997	TUBA4A/TUBAL3	3
R-HSA-8957322	Metabolism of steroids	10/755	150/10554	0,638287102	1	0,906949997	CYP7A1/CH25H/	10
R-HSA-201722	Formation of the beta-catenii	6/755	91/10554	0,641486157	1	0,906949997	HIST1H4D/HIST1	6
R-HSA-5250924	B-WICH complex positively r	6/755	91/10554	0,641486157	1	0,906949997	HIST1H4D/HIST1	6
R-HSA-5693565	Recruitment and ATM-media	5/755	76/10554	0,641838956	1	0,906949997	HIST1H4D/HIST1	5
R-HSA-111996	Ca-dependent events	2/755	30/10554	0,643157458	1	0,906949997	ADCY8/PDE1A	2
R-HSA-389958	Cooperation of Prefoldin and	2/755	30/10554	0,643157458	1	0,906949997	TUBA4A/TUBAL3	2
R-HSA-5357956	TNFR1-induced NFkappaB s	2/755	30/10554	0,643157458	1	0,906949997	TNF/TRAF1	2
R-HSA-2132295	MHC class II antigen present	8/755	121/10554	0,643262791	1	0,906949997	TUBA4A/CTSS/H	8
R-HSA-2559583	Cellular Senescence	13/755	195/10554	0,645591808	1	0,906949997	IFNB1/HIST1H1A	13
R-HSA-193775	Synthesis of bile acids and b	1/755	14/10554	0,646477894	1	0,906949997	AKR1D1	1
R-HSA-209952	Peptide hormone biosynthes	1/755	14/10554	0,646477894	1	0,906949997	INHBE	1
R-HSA-2453864	Retinoid cycle disease event	1/755	14/10554	0,646477894	1	0,906949997	STRA6	1
R-HSA-2474795	Diseases associated with vis	1/755	14/10554	0,646477894	1	0,906949997	STRA6	1

R-HSA-3656237	Defective EXT2 causes exos	1/755	14/10554	0,646477894	1	0,906949997	GPC6	1
R-HSA-3656253	Defective EXT1 causes exos	1/755	14/10554	0,646477894	1	0,906949997	GPC6	1
R-HSA-428540	Activation of RAC1	1/755	14/10554	0,646477894	1	0,906949997	SLIT2	1
R-HSA-5682910	LGI-ADAM interactions	1/755	14/10554	0,646477894	1	0,906949997	LGI4	1
R-HSA-6804759	Regulation of TP53 Activity tl	1/755	14/10554	0,646477894	1	0,906949997	TP63	1
R-HSA-877312	Regulation of IFNG signaling	1/755	14/10554	0,646477894	1	0,906949997	PTPN6	1
R-HSA-112043	PLC beta mediated events	3/755	46/10554	0,648919527	1	0,906949997	ADCY8/PDE1A/G	3
R-HSA-5617472	Activation of anterior HOX ge	8/755	122/10554	0,652464763	1	0,906949997	EGR2/HIST1H4D	8
R-HSA-5619507	Activation of HOX genes dur	8/755	122/10554	0,652464763	1	0,906949997	EGR2/HIST1H4D	8
R-HSA-5693606	DNA Double Strand Break R	5/755	77/10554	0,653354828	1	0,906949997	HIST1H4D/HIST1	5
R-HSA-199977	ER to Golgi Anterograde Tra	10/755	152/10554	0,654878243	1	0,906949997	TUBA4A/GRIA1/T	10
R-HSA-5339562	Uptake and actions of bacter	2/755	31/10554	0,661019139	1	0,906949997	HBEGF/SYT1	2
R-HSA-5654687	Downstream signaling of acti	2/755	31/10554	0,661019139	1	0,906949997	FLRT1/FLRT2	2
R-HSA-157858	Gap junction trafficking and r	3/755	47/10554	0,663407476	1	0,906949997	TUBA4A/TUBAL3	3
R-HSA-948021	Transport to the Golgi and su	12/755	183/10554	0,665667857	1	0,906949997	TUBA4A/MGAT4	12
R-HSA-446652	Interleukin-1 family signaling	9/755	139/10554	0,670509567	1	0,906949997	NOD2/IL18RAP/II	9
R-HSA-181431	Acetylcholine binding and do	1/755	15/10554	0,671801347	1	0,906949997	CHRNA6	1
R-HSA-2162123	Synthesis of Prostaglandins (	1/755	15/10554	0,671801347	1	0,906949997	PTGS2	1
R-HSA-622327	Postsynaptic nicotinic acetylc	1/755	15/10554	0,671801347	1	0,906949997	CHRNA6	1
R-HSA-629602	Activation of Nicotinic Acetylc	1/755	15/10554	0,671801347	1	0,906949997	CHRNA6	1
R-HSA-71336	Pentose phosphate pathway	1/755	15/10554	0,671801347	1	0,906949997	RBKS	1
R-HSA-937039	IRAK1 recruits IKK complex	1/755	15/10554	0,671801347	1	0,906949997	PELI1	1
R-HSA-975144	IRAK1 recruits IKK complex	1/755	15/10554	0,671801347	1	0,906949997	PELI1	1
R-HSA-975577	N-Glycan antennae elongatic	1/755	15/10554	0,671801347	1	0,906949997	MGAT4C	1
R-HSA-1221632	Meiotic synapsis	5/755	79/10554	0,675668082	1	0,906949997	HIST1H4D/HIST1	5
R-HSA-3214858	RMTs methylate histone argi	5/755	79/10554	0,675668082	1	0,906949997	HIST1H2AA/HIST	5
R-HSA-416482	G alpha (12/13) signalling ev	5/755	79/10554	0,675668082	1	0,906949997	GNG2/ARHGEF3	5
R-HSA-9020591	Interleukin-12 signaling	3/755	48/10554	0,677456327	1	0,906949997	IL12B/IL12RB1/S	3
R-HSA-1222556	ROS, RNS production in pha	2/755	32/10554	0,678155467	1	0,906949997	NOS2/NCF2	2
R-HSA-2173796	SMAD2/SMAD3:SMAD4 hete	2/755	32/10554	0,678155467	1	0,906949997	JUNB/SMAD7	2
R-HSA-392518	Signal amplification	2/755	32/10554	0,678155467	1	0,906949997	GNG2/GNA15	2
R-HSA-451326	Activation of kainate recepto	2/755	32/10554	0,678155467	1	0,906949997	GNG2/GRIK5	2
R-HSA-456926	Thrombin signalling through	2/755	32/10554	0,678155467	1	0,906949997	GNG2/GNA15	2
R-HSA-5656169	Termination of translesion DI	2/755	32/10554	0,678155467	1	0,906949997	ISG15/USP43	2
R-HSA-163685	Integration of energy metabo	7/755	110/10554	0,680374983	1	0,906949997	CACNA2D2/GNG	7
R-HSA-8873719	RAB geranylgeranylation	4/755	64/10554	0,681161392	1	0,906949997	RAB17/RAB33A/I	4
R-HSA-5625740	RHO GTPases activate PKN	6/755	95/10554	0,682598652	1	0,906949997	HIST1H4D/HIST1	6
R-HSA-69473	G2/M DNA damage checkpo	6/755	95/10554	0,682598652	1	0,906949997	HIST1H4D/HIST1	6

R-HSA-6811436	COPI-independent Golgi-to-E	3/755	49/10554	0,691066686	1	0,906949997	TUBA4A/TUBAL3	3
R-HSA-1592389	Activation of Matrix Metallopr	2/755	33/10554	0,694578669	1	0,906949997	CTRB2/MMP8	2
R-HSA-163359	Glucagon signaling in metab	2/755	33/10554	0,694578669	1	0,906949997	GNG2/ADCY8	2
R-HSA-5357905	Regulation of TNFR1 signalir	2/755	33/10554	0,694578669	1	0,906949997	TNF/TRAF1	2
R-HSA-1295596	Spry regulation of FGF signa	1/755	16/10554	0,695313064	1	0,906949997	SPRY2	1
R-HSA-139853	Elevation of cytosolic Ca2+ le	1/755	16/10554	0,695313064	1	0,906949997	P2RX7	1
R-HSA-205043	NRIF signals cell death from	1/755	16/10554	0,695313064	1	0,906949997	NGFR	1
R-HSA-2559584	Formation of Senescence-As	1/755	16/10554	0,695313064	1	0,906949997	HIST1H1A	1
R-HSA-418038	Nucleotide-like (purinergic) re	1/755	16/10554	0,695313064	1	0,906949997	LPAR6	1
R-HSA-5684264	MAP3K8 (TPL2)-dependent	1/755	16/10554	0,695313064	1	0,906949997	MAP3K8	1
R-HSA-5654736	Signaling by FGFR1	3/755	50/10554	0,704240452	1	0,906949997	FLRT1/FLRT2/SF	3
R-HSA-157579	Telomere Maintenance	5/755	82/10554	0,707307136	1	0,906949997	HIST1H4D/HIST1	5
R-HSA-9013694	Signaling by NOTCH4	5/755	82/10554	0,707307136	1	0,906949997	DLL4/JAG1/ACTA	5
R-HSA-1489509	DAG and IP3 signaling	2/755	34/10554	0,710302938	1	0,906949997	ADCY8/PDE1A	2
R-HSA-196071	Metabolism of steroid hormo	2/755	34/10554	0,710302938	1	0,906949997	STAR/HSD17B1	2
R-HSA-5693607	Processing of DNA double-st	6/755	98/10554	0,711343265	1	0,906949997	HIST1H4D/HIST1	6
R-HSA-1483206	Glycerophospholipid biosynth	8/755	129/10554	0,712872726	1	0,906949997	ACHE/HRASLS2/	8
R-HSA-3928665	EPH-ephrin mediated repulsi	3/755	51/10554	0,716980695	1	0,906949997	EFNA1/EFNB2/EI	3
R-HSA-140534	Caspase activation via Death	1/755	17/10554	0,717142504	1	0,906949997	TLR4	1
R-HSA-1482922	Acyl chain remodelling of PI	1/755	17/10554	0,717142504	1	0,906949997	PLA2G4C	1
R-HSA-164378	PKA activation in glucagon s	1/755	17/10554	0,717142504	1	0,906949997	ADCY8	1
R-HSA-1810476	RIP-mediated NFkB activatio	1/755	17/10554	0,717142504	1	0,906949997	NFKBIA	1
R-HSA-196757	Metabolism of folate and pter	1/755	17/10554	0,717142504	1	0,906949997	ALDH1L1	1
R-HSA-199220	Vitamin B5 (pantothenate) m	1/755	17/10554	0,717142504	1	0,906949997	VNN1	1
R-HSA-202131	Metabolism of nitric oxide	1/755	17/10554	0,717142504	1	0,906949997	CYGB	1
R-HSA-203765	eNOS activation and regulati	1/755	17/10554	0,717142504	1	0,906949997	CYGB	1
R-HSA-264642	Acetylcholine Neurotransmitt	1/755	17/10554	0,717142504	1	0,906949997	SYT1	1
R-HSA-435354	Zinc transporters	1/755	17/10554	0,717142504	1	0,906949997	SLC30A1	1
R-HSA-450513	Tristetraprolin (TTP, ZFP36)	1/755	17/10554	0,717142504	1	0,906949997	ZFP36	1
R-HSA-6788467	IL-6-type cytokine receptor liç	1/755	17/10554	0,717142504	1	0,906949997	IL11	1
R-HSA-6807878	COPI-mediated anterograde	6/755	99/10554	0,720518621	1	0,906949997	TUBA4A/TUBAL3	6
R-HSA-5579029	Metabolic disorders of biolog	2/755	35/10554	0,725344082	1	0,906949997	GGT1/CYP26B1	2
R-HSA-204005	COPII-mediated vesicle trans	4/755	68/10554	0,726544846	1	0,906949997	GRIA1/COL7A1/T	4
R-HSA-8978868	Fatty acid metabolism	11/755	177/10554	0,729414112	1	0,906949997	CYP4F2/CYP4F3	11
R-HSA-8856825	Cargo recognition for clathrin	6/755	100/10554	0,729489737	1	0,906949997	TF/SGIP1/SYT1/I	6
R-HSA-1368082	RORA activates gene expres	1/755	18/10554	0,737409886	1	0,906949997	RORA	1
R-HSA-181429	Serotonin Neurotransmitter F	1/755	18/10554	0,737409886	1	0,906949997	SYT1	1
R-HSA-1839117	Signaling by cytosolic FGFR1	1/755	18/10554	0,737409886	1	0,906949997	STAT5A	1

R-HSA-1912420	Pre-NOTCH Processing in G	1/755	18/10554	0,737409886	1	0,906949997	ATP2A1	1
R-HSA-5654704	SHC-mediated cascade:FGF	1/755	18/10554	0,737409886	1	0,906949997	FGFR3	1
R-HSA-5654710	PI-3K cascade:FGFR3	1/755	18/10554	0,737409886	1	0,906949997	FGFR3	1
R-HSA-70221	Glycogen breakdown (glycog	1/755	18/10554	0,737409886	1	0,906949997	PHKG1	1
R-HSA-936964	Activation of IRF3/IRF7 medi	1/755	18/10554	0,737409886	1	0,906949997	TLR4	1
R-HSA-8878171	Transcriptional regulation by	15/755	239/10554	0,738734435	1	0,906949997	NFATC2/GP1BA/	15
R-HSA-156590	Glutathione conjugation	2/755	36/10554	0,739719228	1	0,906949997	GGT1/GSTO2	2
R-HSA-6802948	Signaling by high-kinase acti	2/755	36/10554	0,739719228	1	0,906949997	VWF/ITGA2B	2
R-HSA-3371497	HSP90 chaperone cycle for s	3/755	53/10554	0,74117802	1	0,906949997	TUBA4A/TUBAL3	3
R-HSA-351202	Metabolism of polyamines	5/755	86/10554	0,746034817	1	0,906949997	CKM/SLC6A7/SL	5
R-HSA-9006936	Signaling by TGF-beta family	6/755	102/10554	0,74681884	1	0,906949997	AMHR2/BAMBI/P	6
R-HSA-1500620	Meiosis	7/755	118/10554	0,748814391	1	0,906949997	HIST1H4D/HIST1	7
R-HSA-3299685	Detoxification of Reactive Ox	2/755	37/10554	0,753446549	1	0,906949997	GPX7/NCF2	2
R-HSA-5689901	Metalloprotease DUBs	2/755	37/10554	0,753446549	1	0,906949997	HIST1H2AA/NLR	2
R-HSA-8979227	Triglyceride metabolism	2/755	37/10554	0,753446549	1	0,906949997	MOGAT1/AGMO	2
R-HSA-9020702	Interleukin-1 signaling	6/755	103/10554	0,75517739	1	0,906949997	NOD2/IRAK3/IRA	6
R-HSA-163615	PKA activation	1/755	19/10554	0,75622685	1	0,906949997	ADCY8	1
R-HSA-176407	Conversion from APC/C:Cdc	1/755	19/10554	0,75622685	1	0,906949997	CDC14A	1
R-HSA-392851	Prostacyclin signalling throug	1/755	19/10554	0,75622685	1	0,906949997	GNG2	1
R-HSA-5423646	Aflatoxin activation and deto	1/755	19/10554	0,75622685	1	0,906949997	GGT1	1
R-HSA-5576886	Phase 4 - resting membrane	1/755	19/10554	0,75622685	1	0,906949997	KCNK6	1
R-HSA-8851708	Signaling by FGFR2 IIIa TM	1/755	19/10554	0,75622685	1	0,906949997	POLR2A	1
R-HSA-3214841	PKMTs methylate histone lys	4/755	71/10554	0,75729778	1	0,906949997	HIST1H4D/HIST1	4
R-HSA-1638091	Heparan sulfate/heparin (HS	3/755	55/10554	0,76370223	1	0,906949997	NCAN/GPC6/CSF	3
R-HSA-420499	Class C/3 (Metabotropic glut	2/755	38/10554	0,76654503	1	0,906949997	GRM4/GRM8	2
R-HSA-6805567	Keratinization	13/755	214/10554	0,769360946	1	0,906949997	KRT17/PPL/DSG	13
R-HSA-1257604	PIP3 activates AKT signaling	16/755	260/10554	0,770658694	1	0,906949997	ICOS/HBEGF/PIK	16
R-HSA-5668914	Diseases of metabolism	6/755	105/10554	0,771286032	1	0,906949997	CSF2RB/CUBN/C	6
R-HSA-2980736	Peptide hormone metabolism	5/755	89/10554	0,772502747	1	0,906949997	INHBE/ACHE/CL	5
R-HSA-111931	PKA-mediated phosphorylati	1/755	20/10554	0,773697067	1	0,906949997	ADCY8	1
R-HSA-1181150	Signaling by NODAL	1/755	20/10554	0,773697067	1	0,906949997	NODAL	1
R-HSA-388844	Receptor-type tyrosine-prote	1/755	20/10554	0,773697067	1	0,906949997	SLITRK6	1
R-HSA-418217	G beta:gamma signalling thr	1/755	20/10554	0,773697067	1	0,906949997	GNG2	1
R-HSA-5654706	FRS-mediated FGFR3 signa	1/755	20/10554	0,773697067	1	0,906949997	FGFR3	1
R-HSA-8876384	Listeria monocytogenes entr	1/755	20/10554	0,773697067	1	0,906949997	CDH1	1
R-HSA-8978934	Metabolism of cofactors	1/755	20/10554	0,773697067	1	0,906949997	GCH1	1
R-HSA-170834	Signaling by TGF-beta Rece	4/755	73/10554	0,7762701	1	0,906949997	BAMBI/PPP1R15	4
R-HSA-109581	Apoptosis	10/755	169/10554	0,777258278	1	0,906949997	HIST1H1A/DSG3	10

R-HSA-110313	Translesion synthesis by Y fa	2/755	39/10554	0,779034256	1	0,906949997	ISG15/USP43	2
R-HSA-168276	NS1 Mediated Effects on Ho	2/755	39/10554	0,779034256	1	0,906949997	ISG15/KPNA7	2
R-HSA-427413	NoRC negatively regulates rI	6/755	106/10554	0,779038326	1	0,906949997	HIST1H4D/HIST1	6
R-HSA-5250913	Positive epigenetic regulatior	6/755	106/10554	0,779038326	1	0,906949997	HIST1H4D/HIST1	6
R-HSA-9006925	Intracellular signaling by sec	18/755	293/10554	0,78356474	1	0,906949997	ICOS/HBEGF/PIK	18
R-HSA-194138	Signaling by VEGF	6/755	107/10554	0,786591134	1	0,906949997	VEGFD/PGF/CDH	6
R-HSA-1606322	ZBP1(DAI) mediated inductic	1/755	21/10554	0,789916804	1	0,906949997	NFKBIA	1
R-HSA-210745	Regulation of gene expressic	1/755	21/10554	0,789916804	1	0,906949997	HNF4A	1
R-HSA-416572	Sema4D induced cell migrati	1/755	21/10554	0,789916804	1	0,906949997	RND1	1
R-HSA-500657	Presynaptic function of Kaina	1/755	21/10554	0,789916804	1	0,906949997	GNG2	1
R-HSA-5621575	CD209 (DC-SIGN) signaling	1/755	21/10554	0,789916804	1	0,906949997	RELB	1
R-HSA-9008059	Interleukin-37 signaling	1/755	21/10554	0,789916804	1	0,906949997	PTPN6	1
R-HSA-5674135	MAP2K and MAPK activatior	2/755	40/10554	0,79093423	1	0,906949997	VWF/ITGA2B	2
R-HSA-6802946	Signaling by moderate kinas	2/755	40/10554	0,79093423	1	0,906949997	VWF/ITGA2B	2
R-HSA-6802955	Paradoxical activation of RAI	2/755	40/10554	0,79093423	1	0,906949997	VWF/ITGA2B	2
R-HSA-9007101	Rab regulation of trafficking	7/755	124/10554	0,792853441	1	0,906949997	RAB33A/TBC1D1	7
R-HSA-5357801	Programmed Cell Death	10/755	172/10554	0,795232732	1	0,906949997	HIST1H1A/DSG3	10
R-HSA-157118	Signaling by NOTCH	14/755	235/10554	0,7990591	1	0,906949997	DLL4/HIST1H4D/	14
R-HSA-5250941	Negative epigenetic regulatic	6/755	109/10554	0,801105204	1	0,906949997	HIST1H4D/HIST1	6
R-HSA-73854	RNA Polymerase I Promoter	6/755	109/10554	0,801105204	1	0,906949997	HIST1H4D/HIST1	6
R-HSA-168253	Host Interactions with Influen	2/755	41/10554	0,802265218	1	0,906949997	ISG15/KPNA7	2
R-HSA-204998	Cell death signalling via NRA	4/755	76/10554	0,802509141	1	0,906949997	NGFR/ARHGEF3	4
R-HSA-8866654	E3 ubiquitin ligases ubiquitin	3/755	59/10554	0,803961688	1	0,906949997	HIST1H2BE/HIST	3
R-HSA-390466	Chaperonin-mediated proteir	5/755	93/10554	0,804446917	1	0,906949997	TUBA4A/GNG2/F	5
R-HSA-140875	Common Pathway of Fibrin C	1/755	22/10554	0,804975459	1	0,906949997	CD177	1
R-HSA-198753	ERK/MAPK targets	1/755	22/10554	0,804975459	1	0,906949997	MEF2C	1
R-HSA-2024096	HS-GAG degradation	1/755	22/10554	0,804975459	1	0,906949997	GPC6	1
R-HSA-202427	Phosphorylation of CD3 and	1/755	22/10554	0,804975459	1	0,906949997	PTPRC	1
R-HSA-392170	ADP signalling through P2Y	1/755	22/10554	0,804975459	1	0,906949997	GNG2	1
R-HSA-5205685	Pink/Parkin Mediated Mitoph	1/755	22/10554	0,804975459	1	0,906949997	MAP1LC3B	1
R-HSA-5655332	Signaling by FGFR3 in disea	1/755	22/10554	0,804975459	1	0,906949997	FGFR3	1
R-HSA-8853338	Signaling by FGFR3 point mu	1/755	22/10554	0,804975459	1	0,906949997	FGFR3	1
R-HSA-5621481	C-type lectin receptors (CLRs	8/755	142/10554	0,805523033	1	0,906949997	MUC17/NFATC2/	8
R-HSA-381676	Glucagon-like Peptide-1 (GL	2/755	42/10554	0,813047602	1	0,906949997	GNG2/ADCY8	2
R-HSA-3928662	EPHB-mediated forward sigr	2/755	42/10554	0,813047602	1	0,906949997	EFNB2/EPHB2	2
R-HSA-211859	Biological oxidations	13/755	223/10554	0,816306252	1	0,906949997	CYP7A1/UGT2B7	13
R-HSA-167242	Abortive elongation of HIV-1	1/755	23/10554	0,818956045	1	0,906949997	POLR2A	1
R-HSA-212676	Dopamine Neurotransmitter	1/755	23/10554	0,818956045	1	0,906949997	SYT1	1

R-HSA-373753	Nephrin family interactions	1/755	23/10554	0,818956045	1	0,906949997	ACTN2	1
R-HSA-5620922	BBSome-mediated cargo-tar	1/755	23/10554	0,818956045	1	0,906949997	MCHR1	1
R-HSA-8862803	Deregulated CDK5 triggers n	1/755	23/10554	0,818956045	1	0,906949997	STAT4	1
R-HSA-8863678	Neurodegenerative Diseases	1/755	23/10554	0,818956045	1	0,906949997	STAT4	1
R-HSA-73864	RNA Polymerase I Transcrip	6/755	112/10554	0,821428342	1	0,906949997	HIST1H4D/HIST1	6
R-HSA-2029482	Regulation of actin dynamics	3/755	61/10554	0,821832031	1	0,906949997	BTK/MYH2/VAV1	3
R-HSA-177929	Signaling by EGFR	2/755	43/10554	0,823301764	1	0,906949997	ADAM12/SPRY2	2
R-HSA-75893	TNF signaling	2/755	43/10554	0,823301764	1	0,906949997	TNF/TRAF1	2
R-HSA-203927	MicroRNA (miRNA) biogenes	1/755	24/10554	0,831935647	1	0,906949997	POLR2A	1
R-HSA-75876	Synthesis of very long-chain	1/755	24/10554	0,831935647	1	0,906949997	ACSL5	1
R-HSA-8940973	RUNX2 regulates osteoblast	1/755	24/10554	0,831935647	1	0,906949997	HES1	1
R-HSA-2173793	Transcriptional activity of SM	2/755	44/10554	0,833047982	1	0,906949997	JUNB/SMAD7	2
R-HSA-6811438	Intra-Golgi traffic	2/755	44/10554	0,833047982	1	0,906949997	CYTH4/MAN1C1	2
R-HSA-1226099	Signaling by FGFR in diseas	3/755	63/10554	0,838294892	1	0,906949997	STAT5A/FGFR3/I	3
R-HSA-9033241	Peroxisomal protein import	3/755	63/10554	0,838294892	1	0,906949997	NOS2/PIPOX/BA	3
R-HSA-437239	Recycling pathway of L1	2/755	45/10554	0,84230634	1	0,906949997	TUBA4A/TUBAL3	2
R-HSA-3108232	SUMO E3 ligases SUMOylat	10/755	181/10554	0,842741737	1	0,906949997	NR1H4/HIST1H4	10
R-HSA-156588	Glucuronidation	1/755	25/10554	0,843985845	1	0,906949997	UGT2B7	1
R-HSA-182971	EGFR downregulation	1/755	25/10554	0,843985845	1	0,906949997	SPRY2	1
R-HSA-198725	Nuclear Events (kinase and t	1/755	25/10554	0,843985845	1	0,906949997	MEF2C	1
R-HSA-400685	Sema4D in semaphorin signa	1/755	25/10554	0,843985845	1	0,906949997	RND1	1
R-HSA-5654708	Downstream signaling of acti	1/755	25/10554	0,843985845	1	0,906949997	FGFR3	1
R-HSA-937041	IKK complex recruitment me	1/755	25/10554	0,843985845	1	0,906949997	TLR4	1
R-HSA-391251	Protein folding	5/755	99/10554	0,845575981	1	0,906949997	TUBA4A/GNG2/F	5
R-HSA-1168372	Downstream signaling events	4/755	83/10554	0,854072757	1	0,906949997	NFATC2/RASGR	4
R-HSA-3238698	WNT ligand biogenesis and t	1/755	26/10554	0,855173107	1	0,906949997	WNT4	1
R-HSA-389957	Prefoldin mediated transfer c	1/755	26/10554	0,855173107	1	0,906949997	TUBA4A	1
R-HSA-975576	N-glycan antennae elongatio	1/755	26/10554	0,855173107	1	0,906949997	MGAT4C	1
R-HSA-2122947	NOTCH1 Intracellular Domai	2/755	47/10554	0,85943839	1	0,906949997	HES1/MAML2	2
R-HSA-418597	G alpha (z) signalling events	2/755	47/10554	0,85943839	1	0,906949997	GNG2/ADCY8	2
R-HSA-167160	RNA Pol II CTD phosphoryla	1/755	27/10554	0,865559154	1	0,906949997	POLR2A	1
R-HSA-77075	RNA Pol II CTD phosphoryla	1/755	27/10554	0,865559154	1	0,906949997	POLR2A	1
R-HSA-2990846	SUMOylation	10/755	187/10554	0,869332923	1	0,906949997	NR1H4/HIST1H4	10
R-HSA-2029480	Fcgamma receptor (FCGR) (	4/755	86/10554	0,872404163	1	0,906949997	FGR/BTK/MYH2/\	4
R-HSA-194840	Rho GTPase cycle	7/755	138/10554	0,872624551	1	0,906949997	ARHGAP9/ARHG	7
R-HSA-6791312	TP53 Regulates Transcriptio	2/755	49/10554	0,874852099	1	0,906949997	PLK3/BTG2	2
R-HSA-73893	DNA Damage Bypass	2/755	49/10554	0,874852099	1	0,906949997	ISG15/USP43	2
R-HSA-3371556	Cellular response to heat stre	4/755	88/10554	0,883492307	1	0,906949997	CAMK2B/CRYAB	4

R-HSA-5205647	Mitophagy	1/755	29/10554	0,884152753	1	0,906949997	MAP1LC3B	1
R-HSA-72086	mRNA Capping	1/755	29/10554	0,884152753	1	0,906949997	POLR2A	1
R-HSA-8982491	Glycogen metabolism	1/755	29/10554	0,884152753	1	0,906949997	PHKG1	1
R-HSA-4551638	SUMOylation of chromatin or	3/755	70/10554	0,885961339	1	0,906949997	HIST1H4D/HIST1	3
R-HSA-3214847	HATs acetylate histones	7/755	142/10554	0,890102086	1	0,906949997	HIST1H2AA/HIST	7
R-HSA-375170	CDO in myogenesis	1/755	30/10554	0,892462935	1	0,906949997	MEF2C	1
R-HSA-3928663	EPHA-mediated growth cone	1/755	30/10554	0,892462935	1	0,906949997	EFNA1	1
R-HSA-525793	Myogenesis	1/755	30/10554	0,892462935	1	0,906949997	MEF2C	1
R-HSA-9022692	Regulation of MECP2 expres	1/755	30/10554	0,892462935	1	0,906949997	CAMK2B	1
R-HSA-1839124	FGFR1 mutant receptor activ	1/755	31/10554	0,900177729	1	0,906949997	STAT5A	1
R-HSA-2022928	HS-GAG biosynthesis	1/755	31/10554	0,900177729	1	0,906949997	GPC6	1
R-HSA-5654733	Negative regulation of FGFR	1/755	31/10554	0,900177729	1	0,906949997	SPRY2	1
R-HSA-917977	Transferrin endocytosis and	1/755	31/10554	0,900177729	1	0,906949997	TF	1
R-HSA-5654738	Signaling by FGFR2	3/755	73/10554	0,902236232	1	0,906949997	ESRP1/POLR2A/	3
R-HSA-73886	Chromosome Maintenance	5/755	110/10554	0,902394764	1	0,906949997	HIST1H4D/HIST1	5
R-HSA-8941326	RUNX2 regulates bone deve	1/755	32/10554	0,907339737	1	0,906949997	HES1	1
R-HSA-8939236	RUNX1 regulates transcriptio	6/755	130/10554	0,910957598	1	0,906949997	HIST1H4D/HIST1	6
R-HSA-8986944	Transcriptional Regulation by	2/755	55/10554	0,912216466	1	0,906949997	CAMK2B/MEF2C	2
R-HSA-113418	Formation of the Early Elong	1/755	33/10554	0,91398852	1	0,906949997	POLR2A	1
R-HSA-167158	Formation of the HIV-1 Early	1/755	33/10554	0,91398852	1	0,906949997	POLR2A	1
R-HSA-1839126	FGFR2 mutant receptor activ	1/755	33/10554	0,91398852	1	0,906949997	POLR2A	1
R-HSA-202433	Generation of second messe	1/755	33/10554	0,91398852	1	0,906949997	FYB1	1
R-HSA-5654726	Negative regulation of FGFR	1/755	33/10554	0,91398852	1	0,906949997	SPRY2	1
R-HSA-983231	Factors involved in megakary	8/755	166/10554	0,914729805	1	0,906949997	IFNB1/TUBA4A/H	8
R-HSA-2151201	Transcriptional activation of r	2/755	56/10554	0,917322489	1	0,906949997	NR1D1/MEF2C	2
R-HSA-5693567	HDR through Homologous R	6/755	132/10554	0,917973806	1	0,906949997	HIST1H4D/HIST1	6
R-HSA-167238	Pausing and recovery of Tat-	1/755	34/10554	0,920160811	1	0,906949997	POLR2A	1
R-HSA-167243	Tat-mediated HIV elongation	1/755	34/10554	0,920160811	1	0,906949997	POLR2A	1
R-HSA-4791275	Signaling by WNT in cancer	1/755	34/10554	0,920160811	1	0,906949997	DKK2	1
R-HSA-5654727	Negative regulation of FGFR	1/755	34/10554	0,920160811	1	0,906949997	SPRY2	1
R-HSA-5663084	Diseases of carbohydrate me	1/755	34/10554	0,920160811	1	0,906949997	HYAL1	1
R-HSA-983189	Kinesins	2/755	57/10554	0,922148446	1	0,906949997	TUBA4A/TUBAL3	2
R-HSA-6803157	Antimicrobial peptides	4/755	97/10554	0,923607311	1	0,906949997	RNASE7/PGLYRI	4
R-HSA-2454202	Fc epsilon receptor (FCERI)	6/755	134/10554	0,924503105	1	0,906949997	NFATC2/BTK/MA	6
R-HSA-917937	Iron uptake and transport	2/755	58/10554	0,926708209	1	0,906949997	TF/CP	2
R-HSA-71291	Metabolism of amino acids a	20/755	370/10554	0,928561314	1	0,906949997	GLS2/CKM/SLC6	20
R-HSA-8852135	Protein ubiquitination	3/755	79/10554	0,928644901	1	0,906949997	HIST1H2BE/HIST	3
R-HSA-4420097	VEGFA-VEGFR2 Pathway	4/755	99/10554	0,930633216	1	0,906949997	CDH5/NCF2/SH2	4

R-HSA-167287	HIV elongation arrest and rec	1/755	36/10554	0,9312099	1	0,906949997	POLR2A	1
R-HSA-167290	Pausing and recovery of HIV	1/755	36/10554	0,9312099	1	0,906949997	POLR2A	1
R-HSA-5663213	RHO GTPases Activate WAS	1/755	36/10554	0,9312099	1	0,906949997	BTK	1
R-HSA-71387	Metabolism of carbohydrates	14/755	275/10554	0,934310214	1	0,906949997	ACAN/CHST6/CS	14
R-HSA-176187	Activation of ATR in respons	1/755	37/10554	0,936147771	1	0,906949997	RAD9B	1
R-HSA-5685938	HDR through Single Strand A	1/755	37/10554	0,936147771	1	0,906949997	RAD9B	1
R-HSA-75105	Fatty acyl-CoA biosynthesis	1/755	37/10554	0,936147771	1	0,906949997	ACSL5	1
R-HSA-5693538	Homology Directed Repair	6/755	138/10554	0,936206271	1	0,906949997	HIST1H4D/HIST1	6
R-HSA-5607764	CLEC7A (Dectin-1) signaling	4/755	101/10554	0,937070285	1	0,906949997	NFATC2/RELB/N	4
R-HSA-4086398	Ca2+ pathway	2/755	61/10554	0,938920895	1	0,906949997	GNG2/GNAT2	2
R-HSA-8943724	Regulation of PTEN gene tra	2/755	61/10554	0,938920895	1	0,906949997	EGR1/SNAI1	2
R-HSA-5655302	Signaling by FGFR1 in disea	1/755	38/10554	0,940731629	1	0,906949997	STAT5A	1
R-HSA-8856828	Clathrin-mediated endocytos	6/755	140/10554	0,941431192	1	0,906949997	TF/SGIP1/SYT1/I	6
R-HSA-2262752	Cellular responses to stress	23/755	426/10554	0,941829995	1	0,906949997	IFNB1/TUBA4A/C	23
R-HSA-68875	Mitotic Prophase	6/755	141/10554	0,943897792	1	0,906949997	HIST1H4D/HIST1	6
R-HSA-5693616	Presynaptic phase of homolo	1/755	39/10554	0,944986823	1	0,906949997	RAD9B	1
R-HSA-8950505	Gene and protein expression	1/755	39/10554	0,944986823	1	0,906949997	STAT4	1
R-HSA-1483166	Synthesis of PA	1/755	40/10554	0,948936889	1	0,906949997	LIPH	1
R-HSA-5628897	TP53 Regulates Metabolic G	3/755	86/10554	0,951097506	1	0,906949997	GLS2/TP63/SESN	3
R-HSA-6796648	TP53 Regulates Transcriptio	2/755	65/10554	0,952225529	1	0,906949997	POLR2A/FOS	2
R-HSA-5654743	Signaling by FGFR4	1/755	41/10554	0,952603681	1	0,906949997	SPRY2	1
R-HSA-1461957	Beta defensins	1/755	42/10554	0,956007488	1	0,906949997	TLR1	1
R-HSA-168325	Viral Messenger RNA Synthe	1/755	42/10554	0,956007488	1	0,906949997	POLR2A	1
R-HSA-5693579	Homologous DNA Pairing an	1/755	42/10554	0,956007488	1	0,906949997	RAD9B	1
R-HSA-1169091	Activation of NF-kappaB in B	2/755	67/10554	0,957792253	1	0,906949997	NFKBIE/NFKBIA	2
R-HSA-212165	Epigenetic regulation of gene	6/755	148/10554	0,958712155	1	0,906949997	HIST1H4D/HIST1	6
R-HSA-5655253	Signaling by FGFR2 in disea	1/755	43/10554	0,959167148	1	0,906949997	POLR2A	1
R-HSA-5658442	Regulation of RAS by GAPs	2/755	68/10554	0,960337249	1	0,906949997	RASAL1/SPRED3	2
R-HSA-70326	Glucose metabolism	3/755	90/10554	0,960772441	1	0,906949997	FBP2/ENO3/PCK	3
R-HSA-8876198	RAB GEFs exchange GTP fo	3/755	90/10554	0,960772441	1	0,906949997	RAB9B/DENND2I	3
R-HSA-5610787	Hedgehog 'off' state	4/755	111/10554	0,961818976	1	0,906949997	TUBA4A/ADCY10	4
R-HSA-109704	PI3K Cascade	1/755	44/10554	0,962100152	1	0,906949997	FGFR3	1
R-HSA-69231	Cyclin D associated events ir	1/755	44/10554	0,962100152	1	0,906949997	CDKN1C	1
R-HSA-69236	G1 Phase	1/755	44/10554	0,962100152	1	0,906949997	CDKN1C	1
R-HSA-2682334	EPH-Ephrin signaling	3/755	92/10554	0,96490663	1	0,906949997	EFNA1/EFNB2/EI	3
R-HSA-1445148	Translocation of SLC2A4 (GI	2/755	70/10554	0,964992787	1	0,906949997	TUBA4A/TUBAL3	2
R-HSA-167169	HIV Transcription Elongation	1/755	46/10554	0,967349985	1	0,906949997	POLR2A	1
R-HSA-167200	Formation of HIV-1 elongatio	1/755	46/10554	0,967349985	1	0,906949997	POLR2A	1

R-HSA-167246	Tat-mediated elongation of tl	1/755	46/10554	0,967349985	1	0,906949997	POLR2A	1
R-HSA-167161	HIV Transcription Initiation	1/755	47/10554	0,969695889	1	0,906949997	POLR2A	1
R-HSA-167162	RNA Polymerase II HIV Pron	1/755	47/10554	0,969695889	1	0,906949997	POLR2A	1
R-HSA-73776	RNA Polymerase II Promoter	1/755	47/10554	0,969695889	1	0,906949997	POLR2A	1
R-HSA-73779	RNA Polymerase II Transcrip	1/755	47/10554	0,969695889	1	0,906949997	POLR2A	1
R-HSA-75953	RNA Polymerase II Transcrip	1/755	47/10554	0,969695889	1	0,906949997	POLR2A	1
R-HSA-76042	RNA Polymerase II Transcrip	1/755	47/10554	0,969695889	1	0,906949997	POLR2A	1
R-HSA-8939902	Regulation of RUNX2 expres	2/755	73/10554	0,971004792	1	0,906949997	BMP2/ESR1	2
R-HSA-112399	IRS-mediated signalling	1/755	48/10554	0,971873447	1	0,906949997	FGFR3	1
R-HSA-167152	Formation of HIV elongation	1/755	48/10554	0,971873447	1	0,906949997	POLR2A	1
R-HSA-8878159	Transcriptional regulation by	3/755	96/10554	0,971974295	1	0,906949997	JAG1/HES1/MAM	3
R-HSA-6807505	RNA polymerase II transcribe	2/755	74/10554	0,97277779	1	0,906949997	POU2F2/POLR2A	2
R-HSA-6811440	Retrograde transport at the T	1/755	49/10554	0,973894725	1	0,906949997	RAB9B	1
R-HSA-8852276	The role of GTSE1 in G2/M p	2/755	75/10554	0,974445939	1	0,906949997	TUBA4A/TUBAL3	2
R-HSA-201681	TCF dependent signaling in r	10/755	233/10554	0,974502512	1	0,906949997	HIST1H4D/LGR6	10
R-HSA-202403	TCR signaling	4/755	119/10554	0,974752385	1	0,906949997	INPP5D/FYB1/PT	4
R-HSA-5619084	ABC transporter disorders	2/755	76/10554	0,97601514	1	0,906949997	ABCC9/ABCA12	2
R-HSA-8878166	Transcriptional regulation by	4/755	121/10554	0,977272424	1	0,906949997	BMP2/ESR1/HES	4
R-HSA-5620920	Cargo trafficking to the perici	1/755	51/10554	0,977512448	1	0,906949997	MCHR1	1
R-HSA-1461973	Defensins	1/755	52/10554	0,979128948	1	0,906949997	TLR1	1
R-HSA-2428928	IRS-related events triggered	1/755	52/10554	0,979128948	1	0,906949997	FGFR3	1
R-HSA-5620924	Intraflagellar transport	1/755	52/10554	0,979128948	1	0,906949997	TUBA4A	1
R-HSA-72165	mRNA Splicing - Minor Pathw	1/755	52/10554	0,979128948	1	0,906949997	POLR2A	1
R-HSA-2428924	IGF1R signaling cascade	1/755	53/10554	0,98062939	1	0,906949997	FGFR3	1
R-HSA-5693532	DNA Double-Strand Break R	6/755	166/10554	0,981964664	1	0,906949997	HIST1H4D/HIST1	6
R-HSA-2404192	Signaling by Type 1 Insulin-li	1/755	54/10554	0,982022097	1	0,906949997	FGFR3	1
R-HSA-6781823	Formation of TC-NER Pre-In	1/755	54/10554	0,982022097	1	0,906949997	POLR2A	1
R-HSA-74751	Insulin receptor signalling ca	1/755	54/10554	0,982022097	1	0,906949997	FGFR3	1
R-HSA-5358351	Signaling by Hedgehog	5/755	147/10554	0,982829775	1	0,906949997	TUBA4A/ADCY10	5
R-HSA-71406	Pyruvate metabolism and Cit	1/755	55/10554	0,983314793	1	0,906949997	ADHFE1	1
R-HSA-69481	G2/M Checkpoints	6/755	168/10554	0,983603356	1	0,906949997	HIST1H4D/HIST1	6
R-HSA-983169	Class I MHC mediated antige	17/755	371/10554	0,984940533	1	0,906949997	ASB2/CTSS/ASB	17
R-HSA-446203	Asparagine N-linked glycosyl	13/755	302/10554	0,98570785	1	0,906949997	TUBA4A/MGAT4	13
R-HSA-156580	Phase II - Conjugation of cor	3/755	109/10554	0,986745372	1	0,906949997	UGT2B7/GGT1/G	3
R-HSA-5387390	Hh mutants abrogate ligand s	1/755	59/10554	0,987621452	1	0,906949997	HHAT	1
R-HSA-5676590	NIK-->noncanonical NF-kB s	1/755	59/10554	0,987621452	1	0,906949997	RELB	1
R-HSA-168254	Influenza Infection	5/755	154/10554	0,987968765	1	0,906949997	RPL3L/ISG15/KP	5
R-HSA-5607761	Dectin-1 mediated noncanon	1/755	60/10554	0,988511952	1	0,906949997	RELB	1

R-HSA-5610780	Degradation of GLI1 by the p	1/755	60/10554	0,988511952	1	0,906949997	GLI1	1
R-HSA-112382	Formation of RNA Pol II elon	1/755	61/10554	0,98933847	1	0,906949997	POLR2A	1
R-HSA-75955	RNA Polymerase II Transcrip	1/755	61/10554	0,98933847	1	0,906949997	POLR2A	1
R-HSA-192823	Viral mRNA Translation	2/755	89/10554	0,989594491	1	0,906949997	RPL3L/DNAJC3	2
R-HSA-3247509	Chromatin modifying enzyme	11/755	275/10554	0,990145228	1	0,906949997	HIST1H2AA/HIST	11
R-HSA-4839726	Chromatin organization	11/755	275/10554	0,990145228	1	0,906949997	HIST1H2AA/HIST	11
R-HSA-1834949	Cytosolic sensors of pathoge	1/755	63/10554	0,990817594	1	0,906949997	NFKBIA	1
R-HSA-5689880	Ub-specific processing prote	8/755	220/10554	0,990940589	1	0,906949997	HIST1H2AA/HIST	8
R-HSA-380320	Recruitment of NuMA to mitc	2/755	92/10554	0,991441113	1	0,906949997	TUBA4A/TUBAL3	2
R-HSA-4086400	PCP/CE pathway	2/755	92/10554	0,991441113	1	0,906949997	VANGL2/WNT4	2
R-HSA-6804756	Regulation of TP53 Activity tl	2/755	92/10554	0,991441113	1	0,906949997	PLK3/RAD9B	2
R-HSA-5688426	Deubiquitination	12/755	297/10554	0,991451705	1	0,906949997	NOD2/HIST1H2A	12
R-HSA-4608870	Asymmetric localization of P(	1/755	64/10554	0,991478419	1	0,906949997	VANGL2	1
R-HSA-195258	RHO GTPase Effectors	13/755	316/10554	0,991667994	1	0,906949997	TUBA4A/HIST1H	13
R-HSA-5358346	Hedgehog ligand biogenesis	1/755	65/10554	0,992091745	1	0,906949997	HHAT	1
R-HSA-6782210	Gap-filling DNA repair synthe	1/755	65/10554	0,992091745	1	0,906949997	POLR2A	1
R-HSA-194315	Signaling by Rho GTPases	20/755	444/10554	0,992634459	1	0,906949997	TUBA4A/ARHGA	20
R-HSA-1234176	Oxygen-dependent proline hy	1/755	66/10554	0,992660983	1	0,906949997	EPAS1	1
R-HSA-6782135	Dual incision in TC-NER	1/755	66/10554	0,992660983	1	0,906949997	POLR2A	1
R-HSA-1592230	Mitochondrial biogenesis	2/755	95/10554	0,99296624	1	0,906949997	NR1D1/MEF2C	2
R-HSA-5685942	HDR through Homologous R	1/755	67/10554	0,993189297	1	0,906949997	RAD9B	1
R-HSA-1632852	Macroautophagy	1/755	68/10554	0,993679626	1	0,906949997	MAP1LC3B	1
R-HSA-3371453	Regulation of HSF1-mediate	1/755	68/10554	0,993679626	1	0,906949997	HSPA5	1
R-HSA-6811434	COPI-dependent Golgi-to-EF	2/755	97/10554	0,993831719	1	0,906949997	TUBA4A/TUBAL3	2
R-HSA-3858494	Beta-catenin independent W	4/755	145/10554	0,993861815	1	0,906949997	VANGL2/GNG2/W	4
R-HSA-8948751	Regulation of PTEN stability	1/755	69/10554	0,994134698	1	0,906949997	FRK	1
R-HSA-202424	Downstream TCR signaling	2/755	98/10554	0,994224448	1	0,906949997	INPP5D/NFKBIA	2
R-HSA-380259	Loss of Nlp from mitotic cent	1/755	70/10554	0,994557044	1	0,906949997	TUBA4A	1
R-HSA-380284	Loss of proteins required for	1/755	70/10554	0,994557044	1	0,906949997	TUBA4A	1
R-HSA-70171	Glycolysis	1/755	70/10554	0,994557044	1	0,906949997	ENO3	1
R-HSA-2500257	Resolution of Sister Chromat	3/755	124/10554	0,994572294	1	0,906949997	TUBA4A/TUBAL3	3
R-HSA-195721	Signaling by WNT	13/755	330/10554	0,995246976	1	0,906949997	VANGL2/HIST1H	13
R-HSA-9609507	Protein localization	3/755	127/10554	0,995474356	1	0,906949997	NOS2/PIPOX/BA	3
R-HSA-5689603	UCH proteinases	2/755	102/10554	0,995564607	1	0,906949997	HIST1H2AA/SMA	2
R-HSA-167172	Transcription of the HIV genc	1/755	73/10554	0,995650406	1	0,906949997	POLR2A	1
R-HSA-8854518	AURKA Activation by TPX2	1/755	73/10554	0,995650406	1	0,906949997	TUBA4A	1
R-HSA-1234174	Regulation of Hypoxia-induci	1/755	75/10554	0,99625451	1	0,906949997	EPAS1	1
R-HSA-2262749	Cellular response to hypoxia	1/755	75/10554	0,99625451	1	0,906949997	EPAS1	1

R-HSA-8856688	Golgi-to-ER retrograde trans	3/755	131/10554	0,996453646	1	0,906949997	TUBA4A/TUBAL3	3
R-HSA-6811442	Intra-Golgi and retrograde G	6/755	200/10554	0,99668561	1	0,906949997	TUBA4A/CYTH4/	6
R-HSA-168273	Influenza Viral RNA Transcri	3/755	133/10554	0,996862637	1	0,906949997	RPL3L/DNAJC3/I	3
R-HSA-187037	Signaling by NTRK1 (TRKA)	1/755	78/10554	0,997007223	1	0,906949997	MEF2C	1
R-HSA-74752	Signaling by Insulin receptor	1/755	78/10554	0,997007223	1	0,906949997	FGFR3	1
R-HSA-6781827	Transcription-Coupled Nucle	1/755	79/10554	0,997222911	1	0,906949997	POLR2A	1
R-HSA-3700989	Transcriptional Regulation by	14/755	365/10554	0,997569982	1	0,906949997	GLS2/BCL2L14/T	14
R-HSA-5663220	RHO GTPases Activate Forr	3/755	138/10554	0,997694453	1	0,906949997	TUBA4A/TUBAL3	3
R-HSA-2871837	FCERI mediated NF-kB activ	1/755	82/10554	0,997781207	1	0,906949997	NFKBIA	1
R-HSA-380270	Recruitment of mitotic centro	1/755	82/10554	0,997781207	1	0,906949997	TUBA4A	1
R-HSA-380287	Centrosome maturation	1/755	82/10554	0,997781207	1	0,906949997	TUBA4A	1
R-HSA-6807070	PTEN Regulation	3/755	140/10554	0,997963102	1	0,906949997	EGR1/SNAI1/FRK	3
R-HSA-674695	RNA Polymerase II Pre-trans	1/755	84/10554	0,998089625	1	0,906949997	POLR2A	1
R-HSA-5632684	Hedgehog 'on' state	1/755	85/10554	0,998227383	1	0,906949997	GLI1	1
R-HSA-168255	Influenza Life Cycle	3/755	143/10554	0,998309694	1	0,906949997	RPL3L/DNAJC3/I	3
R-HSA-174143	APC/C-mediated degradation	1/755	86/10554	0,99835522	1	0,906949997	CDC14A	1
R-HSA-453276	Regulation of mitotic cell cycl	1/755	86/10554	0,99835522	1	0,906949997	CDC14A	1
R-HSA-2565942	Regulation of PLK1 Activity a	1/755	88/10554	0,998583933	1	0,906949997	TUBA4A	1
R-HSA-450531	Regulation of mRNA stability	1/755	88/10554	0,998583933	1	0,906949997	ZFP36	1
R-HSA-156902	Peptide chain elongation	1/755	89/10554	0,998686086	1	0,906949997	RPL3L	1
R-HSA-5687128	MAPK6/MAPK4 signaling	1/755	89/10554	0,998686086	1	0,906949997	CDC14A	1
R-HSA-156842	Eukaryotic Translation Elong	1/755	93/10554	0,999026205	1	0,906949997	RPL3L	1
R-HSA-2408557	Selenocysteine synthesis	1/755	93/10554	0,999026205	1	0,906949997	RPL3L	1
R-HSA-72764	Eukaryotic Translation Termi	1/755	93/10554	0,999026205	1	0,906949997	RPL3L	1
R-HSA-975956	Nonsense Mediated Decay (I	1/755	95/10554	0,999161702	1	0,906949997	RPL3L	1
R-HSA-141424	Amplification of signal from t	1/755	96/10554	0,999222216	1	0,906949997	DYNC1I1	1
R-HSA-141444	Amplification of signal from t	1/755	96/10554	0,999222216	1	0,906949997	DYNC1I1	1
R-HSA-5620912	Anchoring of the basal body	1/755	98/10554	0,999330469	1	0,906949997	TUBA4A	1
R-HSA-166520	Signaling by NTRKs	1/755	99/10554	0,999378814	1	0,906949997	MEF2C	1
R-HSA-5633007	Regulation of TP53 Activity	3/755	160/10554	0,999420914	1	0,906949997	TP63/PLK3/RAD9	3
R-HSA-72689	Formation of a pool of free 4	1/755	101/10554	0,999465295	1	0,906949997	RPL3L	1
R-HSA-156827	L13a-mediated translational	1/755	111/10554	0,999747431	1	0,906949997	RPL3L	1
R-HSA-5696398	Nucleotide Excision Repair	1/755	111/10554	0,999747431	1	0,906949997	POLR2A	1
R-HSA-1799339	SRP-dependent cotranslation	1/755	112/10554	0,999765691	1	0,906949997	RPL3L	1
R-HSA-69618	Mitotic Spindle Checkpoint	1/755	112/10554	0,999765691	1	0,906949997	DYNC1I1	1
R-HSA-72706	GTP hydrolysis and joining o	1/755	112/10554	0,999765691	1	0,906949997	RPL3L	1
R-HSA-73894	DNA Repair	9/755	316/10554	0,999772215	1	0,906949997	HIST1H4D/HIST1	9
R-HSA-927802	Nonsense-Mediated Decay (	1/755	115/10554	0,999812933	1	0,906949997	RPL3L	1

R-HSA-975957	Nonsense Mediated Decay (I	1/755	115/10554	0,999812933	1	0,906949997	RPL3L	1
R-HSA-2408522	Selenoamino acid metabolisı	1/755	118/10554	0,999850661	1	0,906949997	RPL3L	1
R-HSA-72613	Eukaryotic Translation Initiati	1/755	119/10554	0,999861465	1	0,906949997	RPL3L	1
R-HSA-72737	Cap-dependent Translation I	1/755	119/10554	0,999861465	1	0,906949997	RPL3L	1
R-HSA-8951664	Neddylation	5/755	234/10554	0,999861846	1	0,906949997	ASB2/ASB5/FBX(	5
R-HSA-983168	Antigen processing: Ubiquitir	8/755	309/10554	0,999896393	1	0,906949997	ASB2/ASB5/FBX(	8
R-HSA-2467813	Separation of Sister Chroma	3/755	188/10554	0,999905164	1	0,906949997	TUBA4A/TUBAL3	3
R-HSA-69620	Cell Cycle Checkpoints	7/755	293/10554	0,999928651	1	0,906949997	HIST1H4D/HIST1	7
R-HSA-68877	Mitotic Prometaphase	3/755	198/10554	0,999950864	1	0,906949997	TUBA4A/TUBAL3	3
R-HSA-5617833	Cilium Assembly	3/755	199/10554	0,999954005	1	0,906949997	TUBA4A/MCHR1	3
R-HSA-68882	Mitotic Anaphase	3/755	199/10554	0,999954005	1	0,906949997	TUBA4A/TUBAL3	3
R-HSA-2555396	Mitotic Metaphase and Anap	3/755	200/10554	0,999956947	1	0,906949997	TUBA4A/TUBAL3	3
R-HSA-9010553	Regulation of expression of S	2/755	171/10554	0,999960212	1	0,906949997	RPL3L/SLIT2	2
R-HSA-162599	Late Phase of HIV Life Cycle	1/755	138/10554	0,999966789	1	0,906949997	POLR2A	1
R-HSA-453279	Mitotic G1-G1/S phases	1/755	149/10554	0,999985491	1	0,906949997	CDKN1C	1
R-HSA-376176	Signaling by ROBO receptors	3/755	218/10554	0,99998701	1	0,906949997	RPL3L/SRGAP3/S	3
R-HSA-162587	HIV Life Cycle	1/755	151/10554	0,99998752	1	0,906949997	POLR2A	1
R-HSA-69275	G2/M Transition	2/755	194/10554	0,999992111	1	0,906949997	TUBA4A/TUBAL3	2
R-HSA-453274	Mitotic G2-G2/M phases	2/755	196/10554	0,999993152	1	0,906949997	TUBA4A/TUBAL3	2
R-HSA-5663205	Infectious disease	9/755	382/10554	0,999993512	1	0,906949997	RPL3L/CDH1/HB	9
R-HSA-68886	M Phase	9/755	393/10554	0,999996509	1	0,906949997	TUBA4A/HIST1H	9
R-HSA-1852241	Organelle biogenesis and ma	5/755	294/10554	0,999996513	1	0,906949997	TUBA4A/MCHR1	5
R-HSA-1428517	The citric acid (TCA) cycle aı	1/755	174/10554	0,999997798	1	0,906949997	ADHFE1	1
R-HSA-72163	mRNA Splicing - Major Pathv	1/755	183/10554	0,999998884	1	0,906949997	POLR2A	1
R-HSA-6791226	Major pathway of rRNA proc	1/755	185/10554	0,999999041	1	0,906949997	RPL3L	1
R-HSA-72172	mRNA Splicing	1/755	191/10554	0,999999391	1	0,906949997	POLR2A	1
R-HSA-8868773	rRNA processing in the nucle	1/755	195/10554	0,99999955	1	0,906949997	RPL3L	1
R-HSA-72312	rRNA processing	1/755	205/10554	0,999999789	1	0,906949997	RPL3L	1
R-HSA-162906	HIV Infection	1/755	232/10554	0,999999973	1	0,906949997	POLR2A	1
R-HSA-72203	Processing of Capped Intron	1/755	243/10554	0,999999988	1	0,906949997	POLR2A	1
R-HSA-381753	Olfactory Signaling Pathway	4/755	393/10554	0,999999999	1	0,906949997	OR1F1/RTP4/GN	4
R-HSA-72766	Translation	1/755	291/10554	1	1	0,906949997	RPL3L	1
